# Diversity of transducer-like proteins (Tlps) in *Campylobacter*

**DOI:** 10.1371/journal.pone.0214228

**Published:** 2019-03-25

**Authors:** Clifford Clark, Chrystal Berry, Walter Demczuk

**Affiliations:** 1 Division of Enteric Diseases, National Microbiology Laboratory, Public Health Agency of Canada, Winnipeg, Manitoba, Canada; 2 Streptococci and STI Unit, National Microbiology Laboratory, Public Health Agency of Canada, Winnipeg, Manitoba, Canada; Illinois Institute of Technology, UNITED STATES

## Abstract

*Campylobacter* transducer-like proteins (Tlps), also known as methyl-accepting chemotaxis proteins (MCPs), are associated with virulence as well as niche and host adaptation. While functional attributes of these proteins are being elucidated, little has been published regarding their sequence diversity or chromosomal locations and context, although they appear to define invertible regions within *Campylobacter jejuni* genomes. Genome assemblies for several species of C*ampylobacter* were obtained from the publicly available NCBI repositories. Genomes from all isolates were obtained from GenBank and assessed for Tlp content, while data from isolates with complete, finished genomes were used to determine the identity of Tlps as well as the gene content of putative invertible elements (IEs) in *C*. *jejuni* (Cj) and *C*. *coli* (Cc). Tlps from several *Campylobacter* species were organized into a nomenclature system and novel Tlps were defined and named for Cj and Cc. The content of Tlps appears to be species-specific, though diverse within species. Cj and Cc carried overlapping, related Tlp content, as did the three *C*. *fetus* subspecies. Tlp1 was detected in 88% of Cj isolates and approximately 43% of Cc, and was found in a different conserved chromosomal location and genetic context in each species. Tlp1 and Tlp 3 predominated in genomes from Cj whereas other Tlps were detected less frequently. Tlp13 and Tlp20 predominated in genomes from Cc while some Cj/Cc Tlps were not detected at all. Tlps 2–4 and 11–20 were less frequently detected and many showed sequence heterogeneity that could affect substrate binding, signal transduction, or both. Tlps other than Tlp1, 7, and 10 had substantial sequence identity in the C-terminal half of the protein, creating chromosomal repeats potentially capable of mediating the inversion of large chromosomal DNA. Cj and Cc Tlps were both found in association with only 14 different genes, indicating a limited genomic context. In Cj these Tlps defined IEs that were for the most part found at a single chromosomal location and comprised of a conserved set of genes. Cc IEs were situated at very different chromosomal locations, had different structures than Cj IEs, and were occasionally incomplete, therefore not capable of inversion. Tlps may have a role in *Campylobacter* genome structure and dynamics as well as acting as chemoreceptors mediating chemotactic responses.

## Introduction

*Campylobacter spp*. are critically important pathogenic bacteria often introduced into human populations through consumption of contaminated food or water. *C*. *jejuni* (Cj) and *C*. *coli* (Cc) are among the most frequently isolated causes of food- and water-borne gastroenteritis in developed and developing nations [1–4]. They have been isolated from a wide range of animal sources, including cattle, pigs, birds, pets, and poultry, with poultry being the most commonly identified source of human infection, though this has been contested [3,5]. There is some evidence that *Campylobacter* may be carried asymptomatically by humans [5]. Cj infection of humans can cause severe symptoms, including bloody diarrhea, and can rarely be responsible for severe sequelae such as the Guillain-Barré syndrome (GBS) in children and adults, reactive arthritis, inflammatory bowel disease, other gastrointestinal manifestations, and malnutrition [2,5,6]. Other *Campylobacter* species are less frequently associated with human disease though some, like *C*. *concisus* (Ccon), appear to be emerging human pathogens [7–9]. *C*. *fetus* (Cf) have been associated with animal and human abortions and septicemia [7]. Campylobacteriosis has a very high population burden of illness and is a costly public health issue for which the benefit:cost ratio of intervention to reduce cases is extremely high [10].

Cj and Cc constitute discrete populations of bacteria when assessed by whole genome sequencing (WGS) with a time of divergence estimated at ~6500 years ago, concurrent with the spread of agriculture [11]. While Cj populations are composed of clusters of related isolates, Cc populations are structured into three clades. Frequent horizontal gene transfer occurs between Cj and clade I of Cc, which contains isolates associated with human clinical samples and agricultural animals [11]. The other genera of *Campylobacter* are distinct from each other and from Cj and Cc based on the analysis of 16S rRNA and DNA sequences from nine proteins [12]. The Cl group whole genome sequences are highly related and form a coherent clade in dendrograms of multiple *Campylobacter* species [13]. Based on SNP phylogeny mammal-associated Cf strains sort into five different clades, with one clade (clade 5) containing *C*.*fetus* subsp. *fetus* (Cff) and *C*. *fetus* subsp. *venerealis* (Cfv) isolates [14]. Comparative genomics analysis of Ccon revealed extensive genetic diversity grouped into two main clades [15], and multi-locus sequence typing (MLST) of *C*. *upsaliensis* isolates from humans and dogs also detected high levels of diversity [16]. Little information is currently available about the population structures of other *Campylobacter* species.

Motility and chemotaxis are crucial virulence attributes of *Campylobacter* [17]. Cj methyl-accepting chemotaxis proteins (also known as chemoeffectors, transducer-like proteins, or Tlps) associated with chemotactic responses mediate interaction of CheY with the flagellar motor to cause clockwise rotation of flagella [18]. Tlps are classified into several groups of structurally different proteins (groups A, B, and C), though all sense environmental signals and transmit signals to the interior of the bacterial cell [18]. Group A Tlps have a similar structure to methyl-accepting proteins of *E*. *coli*, and currently include Tlp1 (CcaA), Tlp2, Tlp3 (CcmL), Tlp4 (DocC), Tlp7, Tlp10, Tlp11 (CcrG), Tlp12, and Tlp13 [18–20]. The *N*-terminal half of Group A Tlps consists of a periplasmic ligand-sensing domain bounded by two transmembrane domains and a *C*-terminal half responsible for intracellular signalling [18,21,22]. Sequence identities of the *N*- terminal ligand-binding domains of different Tlps are low, but the *C*-terminal signalling domains have higher identity [23]. Unlike *Escherichia coli* and *Salmonella* strains, which express the 4HB_MCP ligand-binding domain in the *N*-terminal half of chemoreceptors, Cj is similar to *Bacillus subtilis* in the presence of dCache (**ca**lcium channels and **che**motaxis receptors) ligand binding domains situated between two membrane spanning domains and located in the periplasmic space [21,22,24]. The substrate specificities mediated by ligand binding domains of several Tlps are well known and have recently been reviewed [25]. Tlp1 binds L-aspartic acid, interacts directly with CheV, and is capable of interacting with CheW in the absence of CheV [26]. Its signalling domain is capable of dimerization. In contrast, Tlp3 binds to multiple ligands (isoleucine, succinic acid, arginine, purine, malic acid, and thiamine) and inactivation of this chemoreceptor has pleiotropic effects on the biology and virulence of the bacterium [27]. Bile and sodium deoxycholate chemoattractant activity for strain NCTC 11168 is mediated through both Tlps 3 and 4 [23]. Tlp11 senses galactose, is a recently evolved sugar-binding chemoreceptor, and interacts with both CheW and CheV [28]. Finally, Tlp12 was recently found to mediate chemoattractant activity in response to glutamate and pyruvate [29].

A subset of Group A Tlps that includes Tlps 2–4 and 11 constitutes a group with very high identity in the *C*-terminal half of the protein associated with signaling domains, creating large repeat units [18,20] that mediate inversion of large chromosomal segments [30]. In addition to their role in chemotaxis, Tlps are associated with niche and host adaptation, colonization of the chicken gastrointestinal tract, and invasion of human and chicken cell lines [25,26,28].

Tlps are found variably in Cj isolates, and expression of the genes encoding Tlps is variable depending on the strain, growth conditions, and isolation source [19,20]. Further data indicate some conservation of locations of genes encoding Tlps within the genome and further suggest that these genes substitute for each other at one site in the genome [20]. Mund and colleagues [20] have also previously characterized the distribution of different Tlps within the Cj population, investigated DNA sequence differences between different Tlp classes, and assessed the association of Tlps with different sources and MLST sequence types. Almost all information on *tlp* genes collected to date appears to be associated only with Cj, while published detection and analyses of the genes in Cc or other *Campylobacter* species is lacking. Furthermore, Cj Tlp types have been characterized in only a few Cj strains, most notably NCTC 11168, in which Tlps 1–4 were identified, and a small number of other strains.

Therefore we have further investigated the sequence variability of Tlps in *Campylobacter* spp. and have further determined the chromosomal locations, and genomic context of Tlps in both Cj and Cc isolates. In this work we focus on those containing the conserved C-terminal half long repeats capable of mediating the inversion of large chromosomal regions. A major goal of this work has been to provide new datasets for hypothesis generation about the evolution of Group A Tlps and their role(s) in the biology and virulence of the organism. In addition we have attempted to establish empiric criteria for grouping Tlps into classes and to assess variability within each Tlp class. We hope that this bioinformatics-based exercise and the information it has generated will spur functional studies and further characterization of Tlps and their role in the biology of *Campylobacter* spp.

## Methods

### Strain selection and Tlp identification

Assembled genomes from *Campylobacter* strains were obtained from the NCBI Genbank and RefSeq databases for initial detection and classification of Tlps, including both complete genomes and draft assemblies. Shotgun sequences were not used for this purpose as detection and classification were done manually. A provisional nomenclature was developed by constructing protein sequence dendrograms and renaming Tlps based on clustering and distances of the clusters from each other. Tlps as defined by this process generally exhibited between 94–100% sequence identity within each Tlp class.

Thirty-nine Cj and twenty-two Cc genbank (.gbk) formatted files for closed and finished genomes were obtained from the GenBank data repository. Searches for additional *Campylobacter* genera found assembled draft or closed genomes for *C*. *avium* (Ca, 1 strain), Ccon (2 strains), Cff (2 strains), *C*. *fetus* subsp. *testudinum* (Cft; 2 strains), Cfv (3 strains), *C*. *helveticus* (Ch; 1 strain), *C*. *lanienae* (Clan; 1 strain), Cl (7 strains), and *C*. *upsaliensis* (0 strains) (See S1 Table for strains and accession numbers). Only shotgun sequences were available for *C*. *upsaliensis* as of May 1, 2018. Thus, though a few *C*. *upsaliensis* Tlps have been mentioned in the scientific literature, analysis of this species was not included in this study. Annotated sequences in the downloaded .gbk formatted files were opened in MS Word format and queried for the presence of Tlps using the search terms ‘chemotaxis’ for methyl-accepting chemotaxis protein (MCP) and ‘chemoreceptor’ for the ribose and galactose chemoreceptor protein. Additional searches for *C*. *jejuni* and *C*. *coli* Tlps were performed using short C-terminal peptide sequences (eg. VNKKRF, VSKKRF, VKKKRF) of Tlps from isolates NCTC 11168 = ATCC 700819 (shortened throughout to NCTC 11168), 00–2425, a Canadian isolate sequenced by our laboratory and described in a previous study [30], and other isolates used in this study. These methods were valuable for detecting Tlps that were not annotated as chemotaxis proteins. Multifasta files were prepared from all protein or DNA sequences included in each analysis for sequence analysis as outlined below.

### Strategy used to develop the proposed extended Tlp nomenclature

A provisional naming scheme is proposed or *Campylobacter* Tlps to aid in organization and description of the data. Tlps for each species except Cj/Cc were named arbitrarily; each species was given a range for 100 names to accommodate the addition of new Tlps as further characterization is undertaken. Cj/Cc names continue the conventions already in place; Tlps have been allotted names in the range Tlp1 to Tlp99 to allow for new Tlp descriptions. Cl are allotted Tlp101-199, Clan Tlp200-299, Ch Tlp300-399, Cf Tlp400-499, Ccon Tlp500-599, and Ca Tlp600-699.

The existing Tlp designations were used as a guide for Tlp naming. Dendrograms generated using proteins sequences for Tlp proteins provided the initial means of discriminating clusters or clades of Tlps and distinguishing them from other clades. Protein sequence alignments comparing Tlps were used to validate and refine the assessments made using dendrograms. Newly described Tlps were considered different from closely related Tlps if they had a substantial number of aa differences in either the *N*-terminal (periplasmic substrate-binding or ligand-sensing) half of the protein OR had blocks of aa differences in either the *N*-terminal or C-terminal (signalling) halves of the protein, AND showed separate clade structure in dendrograms. A fixed % aa difference could not be used for the discrimination of different Tlps. For each pair of the most closely related Tlps there was a discontinuity in % protein identity and % nucleotide identity determined in blastn or blastp searches that correlated with classification of Tlps into different groups; this was used for validation. A fundamental assumption, which is not necessarily fulfilled in all cases, was that complete genome sequences (as opposed to draft or other sequence formats) had been closed and finished, so that the probability of major sequencing errors or poor sequence was minimal. However, no information on the degree to which these additional steps were done was available in the NCBI entries.

The classification of Tlp proteins and *tlp* genes presented here is directed toward allowing discussion of the data presented here, demonstrating Tlp diversity, and providing an initial framework for further studies aimed at understanding the function, evolution, and distribution of these virulence factors. This is not meant to be a final classification scheme; new Tlps are likely to be identified and a new or updated nomenclature may be necessary to describe these molecules as a whole.

### Identification of pseudogenes

Identification of *tlp* pseudogenes from the 39 Cj isolates and 22 Cc complete genomes was accomplished by scanning the downloaded Genbank flatfiles using the keywords “pseudogenes” and “methyl-accepting chemotaxis protein”. Pseudogenes were also found by searching the annotations of chromosomal locations and genes/proteins flanking the co-ordinates where Tlps are typically located. GenBank flatfiles were manually scanned to determine whether the Tlp/MCP was annotated as a pseudogene or if a sequence was present but had not been correctly annotated. Once a pseudogene was identified by any of the above methods the DNA sequence was obtained from the GenBank annotated file or a nucleotide fasta file and translated into all 6 reading frames using EditSeq within the DNASTAR Lasergene 10 or Lasergene 14 software packages (https://www.dnastar.com/t-products-dnastar-lasergene-genomics.aspx). This strategy allowed the identification of specific Tlp peptide sequences. Pseudogene *tlp* DNA sequences were entered into EditSeq or aligned with an intact DNA sequence to determine the number and location(s) of point mutations responsible for pseudogene formation. Within EditSeq full-length proteins were inferred by adding one or two nucleotide(s) where necessary to the pseudogene sequence and translating the amended *tlp* sequence.

### Sequence analysis methods

To produce protein sequence dendrograms, Tlp sequences were aligned using Muscle [31] in MEGA7 [32] with default parameters. A UPGMA dendrogram was constructed in MEGA7 for protein sequences using default parameters (Poisson model, assuming uniform evolutionary rates at all sites). The evolutionary history was inferred using the UPGMA method [33]. The evolutionary distances were computed using the Poisson correction method [34] and are in the units of the number of aa substitutions per site. In each analysis all positions containing gaps and missing data were eliminated. Evolutionary analyses were conducted in MEGA7 [32]. Neighbor Net DNA dendrograms were created with Splitstree 4 using the Kimura 2 parameter model [35].

### Automated detection and classification of *tlp* genes in large sequence databases

The manual methods described above for initial detection of *tlp* genes and Tlp proteins from annotated complete genome sequences were time-consuming and not conducive to high-throughput analysis. Therefore, using the Tlps already identified and characterized in these “reference” sequences, we employed a more efficient and robust bioinformatics method for detecting and assessing different group A Tlp classes in 1085 Cj and 812 Cc genomes downloaded from the NCBI refseq database. This method relied on an R-based BLAST script (MasterBlastR_RScript, S1 Archive) developed in-house for high-throughput detection of user-defined DNA sequences. This script was used to perform blastn queries of DNA sequences comprising the 5ʹ half of each *tlp* gene type (Tlp1-4, 11–20) encoding the Tlp sensory domain. These searches essentially queried the substrate specifity of the Tlp. To do a preliminary assessment of variability in the signalling domains of Tlps, the 3ʹ half of the genes encoding Tlp2 and Tlp3 were used for searches. Allelic variations within each Tlp type (1–4, 11–20) and the presence or absence of genes in the genomes were initially determined using the MasterBlastR_RScript with the e-value cutoff option set to 10e-50. When it became clear that these parameters detected more than one *tlp* type, a more stringent criterion of 95% nucleotide identity was used. The blastout fasta files of the initial blastn analysis were next filtered for sequence length >98% using SeqKit [36] and aligned and visualized using AliView [37] to ensure that only sequences with length similar to the query length were included in the results. This method also enabled the identification of sequence variants (alleles) for each tlp. For each *tlp* gene, sequence variants were assigned an allele number and BLAST analysis of each query *tlp* gene was repeated using the MasterBlastR_RScript script to determine the number of isolates with each allele.

### Identification of invertible elements (IEs) in genomes

The ~93 kb IE previously described and validated with PCR [30] was identified in other genomes by searching manually within GenBank flatfiles for: 1) strings of syntenous genes/proteins within the IE defined previously; 2) the conserved genes/proteins bracketing the IE; 3) two Tlps with conserved *C*-terminal halves in proximity to the features detected in the first two steps. This allowed characterization of the position of the IE in the genome, IE gene content, IE length, and comparison of Cj and Cc IEs. IEs were not sought or characterized in other *Campylobacter* spp.

## Results and discussion

### Group A Tlp proteins are heterogeneous and species-specific

**Fig 1:**
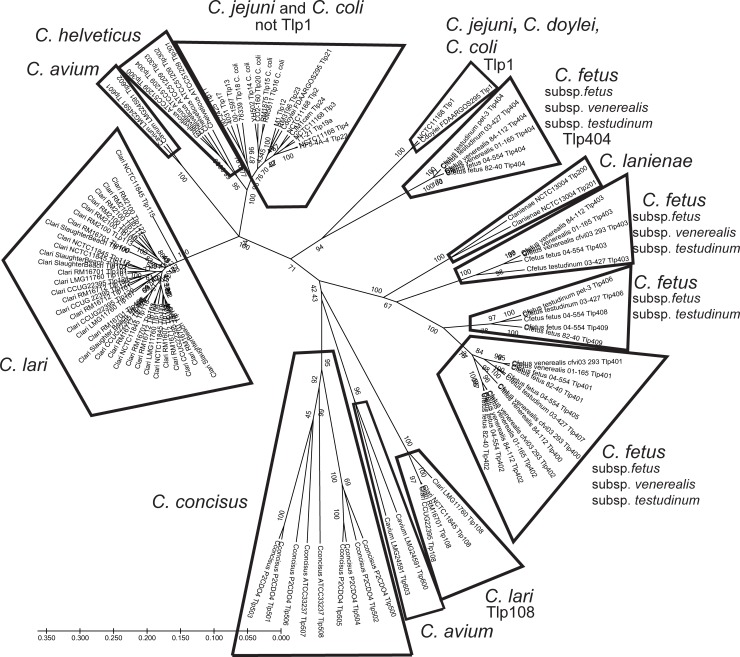
UPGMA dendrogram of *Campylobacter* Tlps. For the Cj/Cc group only one protein sequence was chosen to represent the Tlp type. Because fewer closed and high quality draft sequences were available for the other species, all Tlps associated with these species were included in the analysis.

**Fig 2:**
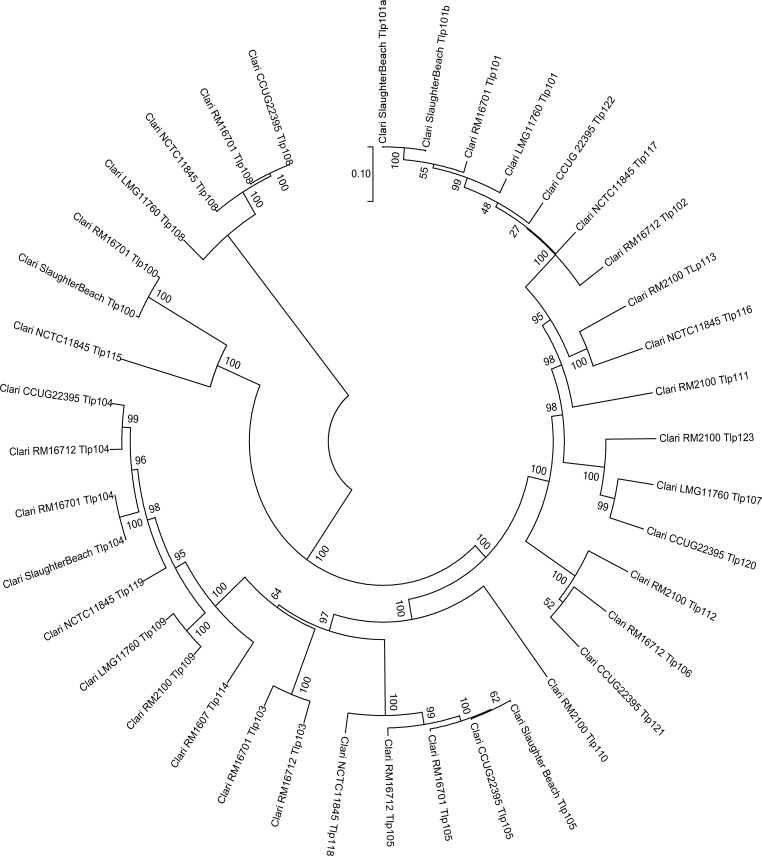
UPGMA dendrogram comparing all Tlp proteins detected in the seven Cl strains investigated.

Group A Tlps with different ligand sensing/small molecule binding domains are frequently classified according to their ligand-binding capability, allowing them to be distinguished from each other by amino acid (aa) sequence differences in the *N*-terminal half of the protein [10]. Protein FASTA files were obtained for Tlps (see Methods, S1 Table) from each of the 80 strains (11 *Campylobacter* species) included in this analysis and were used to generate a UPGMA dendrogram that shows the relationships of protein sequences without corrections for evolutionary models (Fig 1). We demonstrate here that each *Campylobacter* species has its own distinct set of Tlps which, for the most part, are found in coherent groups consistent with the population structure of the organism (see reference [13] for *Campylobacter* population structure). However, while most Cl Tlps form a coherent group, Tlp108 has a very different protein sequence. The differences between Cl Tlp108 and all other Cl proteins can be found in both the *N*-terminal and *C*-terminal halves (Alignment A in S2 Archive). There is much higher sequence identity in Cl Tlp protein sequences after aa 342 when Tlp108 proteins are removed from the alignment (Alignment B in S2 Archive). We speculate that differences in the *C*-terminal halves of Tlp proteins may also be associated with differences in Tlp function, and suggest this may be a fruitful avenue for further research. It was difficult to distinguish the different Cl Tlps in Fig 1, so we constructed a dendrogram from Cl Tlps only (Fig 2). Four protein sequence clusters were identified: 1) Tlp108; 2) Tlps100 & 115; 3) Tlps 103–105, 109, 110, 114, 118, 119; 4) Tlps 101, 102, 106, 107, 111–113, 116, 117, 120–123. The variety and number of Tlps identified in a species or species complex such as Cj/Cc appears to increase with the number of genome sequences examined.

Cf Tlps are distributed in four major groups, two of which contain a single Tlp type (Tlp403 and Tlp404) and two of which are composed of multiple Tlp types (Tlps 400–402, 405, 407 and Tlps 406, 408, 409; Fig 1). Overall, the structure of the Tlp dendrogram was similar to the *Campylobacter* population structure (see reference [13]). As noted previously [38], Cj/Cc/Cj subsp. *doylei* Tlp1 proteins and Tlp404 from all Cf subspecies are most closely related to each other. Substantial differences between these two Tlp types were detected, though there did appear to be an underlying scaffold of identical and conserved amino acids (Alignment C in S2 Archive). It is not clear whether these two Tlps may have arisen through convergent evolution or whether they may share a common ancestor. It would be interesting to characterize Tlp404 to determine ligand-binding specificity.

Our working hypothesis is that these groups, for example, the Cj and Cc Tlps other than Tlp1 and the Cl Tlps other than Tlp108, each represent evolution and differentiation from a common progenitor. A further hypothesis is that each species has evolved different sets of Tlps to help them exploit distinct ecological or host niches. Too few strains were characterized from other *Campylobacter* species to draw many conclusions. Ch Tlps grouped most closely with the non-Tlp1 Cj/Cc Tlps (Fig 1). Ca Tlps were distinct from either group and from Cl Tlps. Like Cf, Ca has different Tlp groups with very distinct protein sequences.

**Fig 3:**
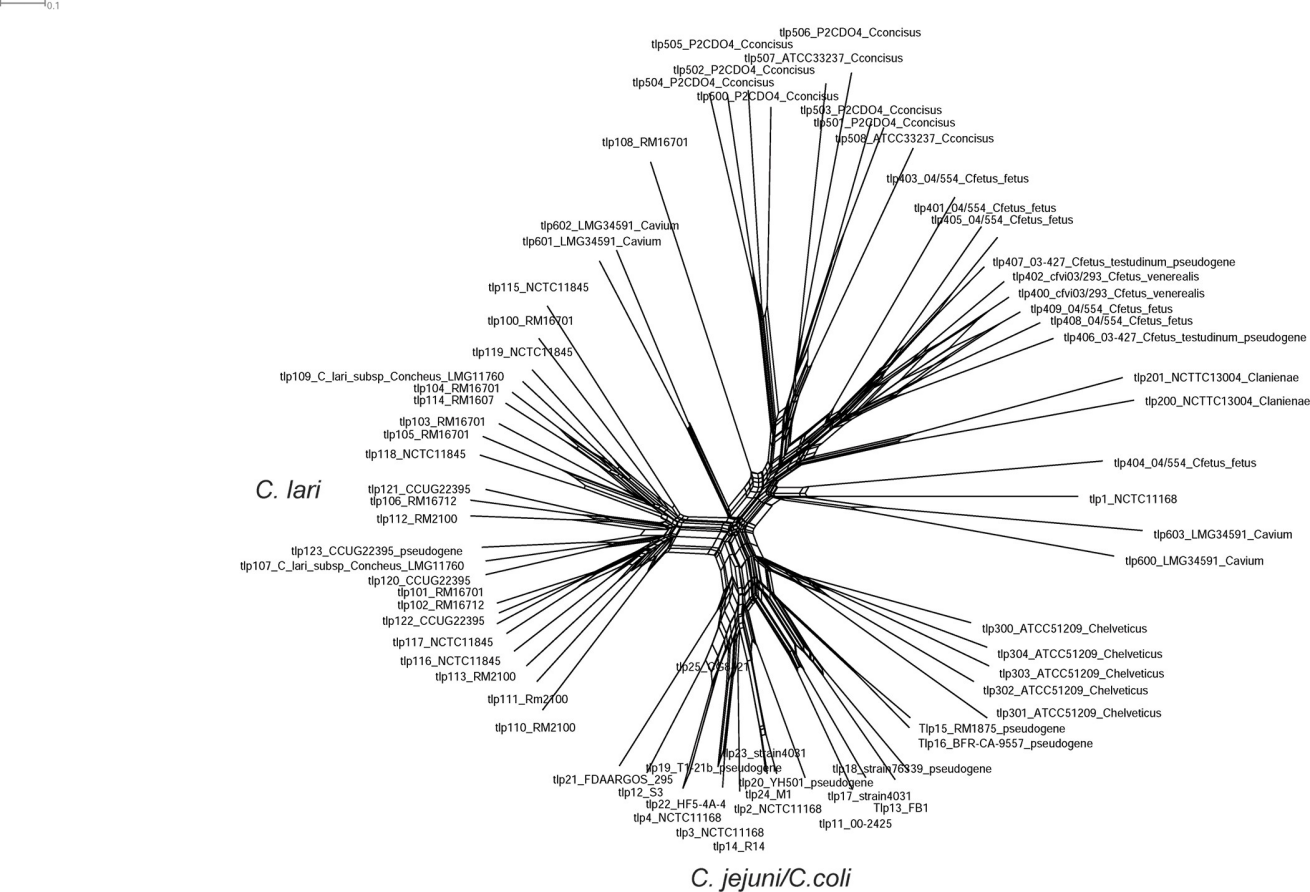
Neighbor Net dendrogram of *tlp* DNA sequences. This dendrogram was generated in Splitstree 4 using a single sequence from each Tlp type. The Kimura 2 parameter adjustment was used in the analysis.

Fig 3 shows the degree of recombination characterized in Tlp proteins using Splitstree analysis to generate a Neighbor-Net dendrogram. Extensive recombination was detected among Tlps from Cj/Cc, and Ch, among Cl Tlp types, and among Cfv and Cff. Less recombination was observed in the other *Campylobacter* species, but this may be partly a function of the fewer number of strains analyzed. For recombination to occur it is assumed that the four species would have to be capable of occupying overlapping environmental or host niches. A lack of recombination between Cft reptilian and human strains has been ascribed to the separate host reservoirs of the two groups, though considerable recombination was found between Cft human strains and Cff human strains [39]. Cl is found in close phylogenetic proximity to, but in a separate clade from, a clade containing Cj, Cc, Ch, Ca, and *C*. *upsaliensis* [13]. Cl shares with these species common animal and environmental sources, such as birds (gulls, shorebirds), water, and livestock [13]. Both of these properties might partially account for the observed recombination among Cl and Cj/Cc Tlps (Fig 3).

Selected domains identified in NCBI blastp searches [40] have been shown for representative protein sequences of each Tlp type (S1 Fig). The dCache1, cache2, single-cache2, and nitrate sensor (NIT) domains are chemoreceptor/Tlp protein sensor domains that interact with small molecules and occupy the entire extracellular region between two transmembrane helices bounding the *N*-terminal half of the protein [41,42]. Most of the 73 Tlps detected expressed dCache1 domains (54 Tlps), though others had a combined cache2 and single cache2 domain (Cc Tlps 15 and 16), a single cache2 domain (Ch Tlp302), or a NIT domain (Ccon Tlp502). No sensor domain was identified in: Cj Tlp25; Cj subsp. *doylei* Tlp21; Cc Tlp18; Cl Tlps 106, 110, 113, 115, 116, 121, & 123; Clan Tlp201; Cfv Tlps 400 & 402; Cff Tlps 409 & 500; Ccon Tlp500, and Ca Tlp602. It seems reasonable to speculate that these Tlps may have sensor domains that are not predicted by the most recently developed models for Cache domains [41]. Alternately they may be Tlps that have lost function by mutation and available to gain different functions through evolutionary processes. This hypothesis is supported by the finding that Cc Tlp16 was a pseudogene in all strains in which it was detected (S1 Spreadsheet), though this may not hold true when more instances of this Tlp are detected. Tlp401 and Tlp 403 were pseudogenes in Cfv strains but were intact in Cff and Cft strains (S1 Spreadsheet). Tlp pseudogenes were identified as outlined in the Methods section.

The *C*-terminal half of Tlp proteins is responsible for propagating a conformational change to the signalling domain of the protein, thus mediating the interaction of Tlps with other proteins of the chemotaxis signalling cascade [21,25]. Tlp domains present in the *C*-terminal portion of the protein include the variably present HAMP domain and the signalling domain containing the CheW interface, the dimer interface, and the MCP signal domain [18,21,25,42]. To characterize variability in Tlp *C*-termini, the dimer binding domains from Tlps in S1 Fig were aligned using S3 FASTA (Alignment D in S2 Archive). Where no dimer binding domains were identified by the algorithms used by NCBI blastp the overlapping MCP signal domain (pfam00015) was used instead. The resulting dendrogram in S3 Fig shows a very similar topology as Fig 1, indicating that the species association of each Tlp is determined by differences in the *C*-terminal half of all Tlps. There is a great deal of heterogeneity overall in the dimer binding (Alignment D in S2 Archive). Further analysis will be required to address the questions of whether this is due to separate evolution of the chemotaxis signalling apparatus in different species and whether there is any role for recombination in this evolution.

### Detection and characterization of Tlps in Cj and Cc

**Fig 4:**
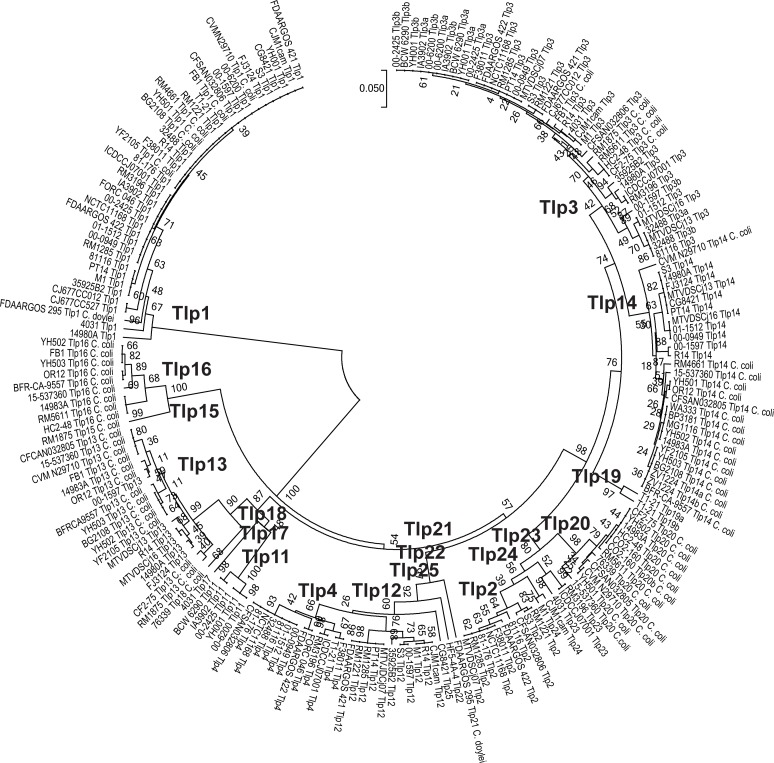
UPGMA dendrogram showing sequence relationships of Cj and Cc Tlps. The dendrogram was created using the data in S4 FASTA.

In addition to the previously described Tlps 1–4 and 11–13 [18,20], we have characterized an additional twelve Tlps (Tlps 14–25) based on differences in Tlp sensor and signalling domains as well as the phylogenetic relationships between of Cj and Cc Tlp protein sequences (Fig 4). As seen in Fig 1, Tlp1 diverges the most from all other Tlps. The next most divergent Tlp classes are Tlps 15 & 16. These Tlps are found only in Cc and are closely related. All other strains are part of a large clade that was subdivided into a large sub-clade that included Tlps 2, 3, 4, 14, 19, 20, 23, & 24 and a smaller sub-clade containing Tlps 11, 13, 17 & 18 (Fig 4). The dendrogram clusters many of the Tlps into related groups: 1) Tlps 13, 17, & 18; 2) Tlps 2, 20, 23, & 24; 3) Tlps 3 & 14; 4) Tlps 4 & 12. The similarity of Tlp 4 and Tlp 12 has been described previously [20,29]. We speculate that this grouping is evidence of continued evolution of the genes encoding Tlps under selective pressures encountered due to the different niche and host adaptations of the organisms. Though the criteria for assigning Tlps to different classes was initially somewhat arbitrary, the classification scheme is generally supported by a more objective strategy utilizing percentage identity of the peptide sequences (see below). However, this is a purely sequence-based schema, and functional differences in substrate binding may be found in Tlps when only a few amino acids are changed [41]; some members of a single Tlp class may therefore have different small molecule binding properties. Functional studies will be necessary to properly address this possibility. Twelve Tlp classes (Tlps 14–25) are newly described, though Tlp25 appears to have a large deletion in the sensor domain and may not be functional. Tlps 15, 16, 18, & 20 are present in Cc, while Tlps 4, 11, 12, 17, and 19 have not yet been detected in Cc. The remaining Tlps are found in both Cj and Cc.

Heterogeneity was found in the *C*-temini of Cj/Cc Tlps1-4, with Tlp1 diverging markedly from the other three Tlps (Alignment E in S2 Archive). When Tlp1 proteins are removed from the alignment along with Tlps with large *C*-terminal deletions, there is a very high level of protein sequence identity in the last ~211 amino acids of the proteins corresponding roughly to the signalling domain (Alignment F in S2 Archive). Notably, differences in the *C*-terminal half of Tlps contribute substantially to the differences between Tlp2 and Tlp23 (Alignment G in S2 Archive), Tlp3 and Tlp19 (Alignment H in S2 Archive), and among both Tlp4 and Tlp12 isolates (Alignment I in S2 Archive). The very large sequence differences in the *N*-terminal substrate-binding domains of Tlp4 and Tlp12 reflect the different substrates bound by these two chemoeffectors. At least three major *C*-terminal sequences exist among Cj/Cc Tlps, and different sequences are frequently found in a single Tlp type (Alignment J in S2 Archive). Tlps from several strains have large deletions in the *C*-terminal half of the protein (Alignment K in S2 Archive). In several Tlps (CJ677CC012, CF2-75, & HC2-48 Tlp3; WA333 Tlp14; HC2-48 Tlp16; HC2-48 Tlp20) the deletion is in the signalling domain, so these Tlps would be expected to be non-functional.

Two copies of Tlp3 are found in each of the Cj isolates 00–2425, 00–6200, 32488, BCW_6290, IA3901, and YH001. Two copies of Tlp14 are present in Cc strain ZV1224, two copies of Tlp19 are found in Cj strain T1-21, and two copies of Tlp20 are found in Cc isolate CO2-160 (S1 FASTA). The presence of two copies of the same gene would create a longer region of identity (genomic repeat) within the genome than Tlps with a high level of identity only in the C-terminal half of the protein; whether this has any functional significance, for example, in mediating inversions of intervening DNA sequences [30] at higher rates, is not known. Alignments of all different Tlps (except Tlps with deletions) demonstrate no identity between the different groups in N-termini but, with the exception of Tlp1, high levels of identity in the C-termini of the proteins (Alignment L in S2 Archive).

#### Tlp1 description and summary

Previous investigations indicated that Tlp1 was detected in all Cj sequences assessed [43] and the gene encoding Tlp1 is always found as an intact coding sequence. We found that the *tlp1* gene was detected in 88% of Cj but in only 43% of Cc genomes analyzed, consistent with the hypothesis that Tlp1 is essential for Cj growth and survival but may be essential for only a sub-population of Cc, such as clade 1. Tlp1 is involved in sensing aspartate, and the difference between Cj and Cc suggests a less important role of aspartate metabolism for some Cc. Perhaps aspartate is found more readily in hosts or environmental niches colonized by Cj, a commensal pathogen of the chicken gut microflora and mammalian hosts, than those associated with *tlp1*-negative Cc, which is more commonly associated with swine isolates (clade 1) or the environment (clades 2 and 3). Tlp1 was previously identified as a gene introduced into agricultural Cc clade 1 genomes through transfer from Cj that was not present in non-agricultural clade 2 and clade 3 isolates (see ref. [44], Table 1, Cj1506c). Tlp1 may enhance the fitness of the organism in specific hosts or agricultural niches of the Cc strains carrying it. Further work associating *tlp*1 gene content with specific hosts or environmental niches is required to begin to answer these questions. In contrast to previous work in which Tlp1 was among the least heterogeneous of all Tlps [19,45] we found that Tlp 1 had a substantial number of differences among strains, particularly when Tlp1 from Cj subsp. *doylei* strain FDAARGOS_295 was taken into account (Fig 4, Table 1, Alignment M in S2 Archive). The N-terminal 20 aa of Tlp1 are located in the cytoplasm, aa 20–30 span the membrane, aa 31–327 constitute the periplasmic sensory domain, aa 328–338 constitute the second membrane spanning helix, and the remaining C-terminal aa of the protein constitute the signalling domain [22]. Most aa substitutions are found in the N-terminal half of the protein, while the C-terminal half is more conserved (Table 1, Alignment M in S2 Archive). This is consistent with previous results [22] indicating that conservative aa substitutions present in 40 different Cj strains mapped to the membrane-proximal substrate-binding domain of Tlp1.

**Table 1:** Variability of amino acids in Cj and Cc Tlp proteins.

Tlp	Number of strains with Tlp	Number of aa in protein	Variant aa in N-terminal half	Variant aa in C-terminal half	Total variant aa
Tlp1	40	700	62/350	34/350	96
Tlp2	10	658–659	31/330	13/329	44
Tlp3	42	586–665	47/325^1,2^	43/340^1^^,^^2^	90
Tlp4	8	665	24/332	19/333	43
Tlp11	5	706	0/352	18/353	18
Tlp12	11	659–660	14/330^1^	33/331^1^	47
Tlp13	7	705	24/352	43/353	67
Tlp14	12	611–667	59/328	46/328^3^	105
Tlp15	1	579	NA	NA	NA
Tlp16	1	578	NA	NA	NA
Tlp17	1	706	NA	NA	NA
Tlp18	1	705	NA	NA	NA
Tlp19	1	606–611	107/305	0/306	107
Tlp20	12	600–661	21/330	37/331	58
Tlp21	1	653	NA	NA	NA
Tlp22	1	611	NA	NA	NA
Tlp23	3	658–659	27/329	22/330	49
Tlp24	2	655–656	0/328	2/328	2
Tlp25	1	447	NA	NA	NA

^1^This number does not include differences associated with large deletions in the proteins.

^2^This number does not include the 11 aa extension on Tlp3 in strains NCTC11168 & 35925B2.

^3^This number does not include any C-terminal extension past the consensus aa sequence.

#### Tlp2 description and summary

Tlp2 chemoreceptor proteins are most closely related to Tlp20, Tlp23, and Tlp24. While Tlps 2, 23, & 24 are present only in Cj, Tlp 20 was found in 649/812 (80%) of Cc genomes compared to only 6/1085 (1%) of Cj genomes. A Tlp2 protein from strain 35925B2 diverges from the full-length Tlp2 aa sequences after 52 aa and is truncated after 80 aa (S1 spreadsheet), indicating that it is not likely to be an essential chemoreceptor. With the exception of a small substitution of six amino acids after aa 362 in strain RM1285, the Tlp2 protein sequences from the isolates with closed, finished sequences are quite homogenous (Alignment N in S2 Archive).

#### Tlp3 description and summary

Tlp3 proteins bind multiple chemoattractant and chemorepellent ligands, including lysine, glucosamine, isoleucine, aspartate, succinic acid, arginine, purine, malic acid, and thiamine, and the binding site of the protein may be able to accommodate more than one chemoattractant at a time [33]. The periplasmic sensory domain is contained within aa 42–291, the HAMP 1 and HAMP 2 domains are found between aa 324–449, and the remaining *C*-terminal aa of the protein constitute the signalling domain (see S1 Fig) [21,45]. Using automated bioinformatics methods for all genomes, we found *tlp3* in 64% of Cj and 30% of Cc genomes, with the highest number of sequence variants of any *tlp* characterized in Cj (66 alleles) and Cc (14 alleles). Tlp3 may be undergoing rapid evolutionary change though, if so, it is unclear why. At least 7 strains carry the *tlp3* gene annotated as a pseudogene in isolates with complete genomes: 35925B2, 81–176, CJ677CC012, CO2-0160, FB1, HC2-48, and RM1875. It is not known how many of the alleles represented pseudogenes in the larger analysis using automated bioinformatics strategies. Tlp3 proteins have an almost equal number of positions with aa substitutions in the *N*- and *C*-terminal halves of the protein (Table 1). There is an additional *N*-terminal extension of 11 aa in six Cj strains and one Cc strain (Alignment O in S2 Archive), likely resulting from the annotated protein beginning from an alternate transcription start site. The aa known to be essential for isoleucine binding [21] are conserved in all strains. A region in the *C*-terminal half of Tlp3 from strain HC-48 extends from aa-471 to aa-579 and includes a large deletion and a 28 aa stretch of alternative peptide sequence in the signalling domain. The CF2-75 Tlp3 contains a *C*-terminal deletion that includes the methylation motif, while the Tlp3 from Cj677CC012 has a large deletion that includes the CheW interaction domain (Alignment O in S2 Archive). All three proteins are expected to be non-functional. Tlp3 proteins from strains 14980A, 35925B2, ICDCCJ07001, RM3196, and 00–1597 carry a divergent peptide motif that is a common alternative sequence in many Tlps from aa 420–455 in the *C*-terminal half of the protein. Because these amino acids are part of domain PRK 15041 (methyl-accepting chemotaxis protein I domain), this difference may affect protein function. We conclude that even within this closely related group of Tlp proteins there is the potential for considerable differences in function.

#### Tlp4 description and summary

Both Tlp4 and Tlp3 mediate chemotaxis toward bile and sodium deoxycholate, a function crucial for colonization of the mouse intestine [23]. Tlp4 was not highly prevalent in the genome sequences used for analysis and was identified in only 287 (27%) Cj and 197 (24%) Cc isolates. Eight complete genomes harbor *tlp4* genes encoding proteins with minimal sequence divergence (Table 1, S1 Spreadsheet, Alignment P in S2 Archive), none of which were annotated as pseudogenes. This group of Tlps shows some sequence divergence in both N- and C-terminal peptide sequences but no deletions (S1F Fig). The last 161 aa are identical in all strains.

#### Tlp11 description and summary

Tlp11, also designated Campylobacter ChemoReceptor for Galactose (CcrG), specifically binds and mediates chemotactic responses to galactose [28]. The *N*-terminal half of the protein containing the sensor/substrate binding domain was identical in all five strains with complete genome sequences carrying this Tlp, though two genomes carried one of the alternative *C*-terminal sequences differing by 18 amino acids (Alignment Q in S2 Archive). To date, Tlp11 has only been associated with invasive strains of Cj [28]. It was identified in 185 (17%) of Cj and in only 4 (<1%) of Cc genomes. Further analysis will be required to determine if all Cj and Cc isolates expressing Tlp11 have increased invasive capacity. None of the *tlp11* genes were annotated as pseudogenes.

#### Tlp12 description and summary

Genes encoding Tlp12 were found in 242 (22%) of Cj strains and 1 Cc genome from strain RC291, which was isolated from a retail chicken in Ireland. The *N*-terminal halves of Tlp12 protein sequences share a very high degree of identity (Alignment R in S2 Archive). The recently characterized Tlp12 protein from strain A17 [29] was included in this alignment and is unique, but differed from Tlp12 in isolatesFDAARGOS_421 and RM1221 by only two aa. *tlp12* from strain RM1285 was annotated as a pseudogene. An alternative peptide motif is found in the *C*-terminal half of Tlp12 from five strains (Alignment R in S2 Archive).

#### Tlp13 description and summary

Genes encoding Tlp13 were detected in 272 (25%) of Cj and in 669 (82%) of Cc (Table 1) and, except for strain 00–1597, the Cj Tlp13 proteins cluster separately from Cc (Fig 4). It would be interesting to know whether the Cc/Cj 00–1597 Tlp13 proteins had different substrate specificity or other functional differences from those of other Cj strains. *tlp13* was the Tlp gene most frequently identified in Cc strains. Few protein sequence differences are detected in the *N*-terminal half of the protein (Alignment S in S2 Archive). In addition to a deletion encompassing the CheW interaction domain in strain CF2-75 there are three different sequence variants in the *C*-terminal half of the protein. The *tlp13* gene in strains CG8421, HC2-48, RM4661, WA333, and YH501 are annotated as pseudogenes (S1 Spreadsheet).

#### Tlp14 description and summary

Although the *tlp14* gene was only detected in 17% of Cj genomes and 2% of Cc genomes there were 22 allelic variants in the Cj *tlp14* genes. Amino acid variants in Tlp14 alleles are distributed in both *N*- and *C*-terminal halves of the protein (Table 1, Alignment T in S2 Archive). Many appear in only one or a few strains and the remainder are not organized into discernable motifs that differentiate Cj and Cc Tlp14, though there do appear to be some single nucleotide polymorphisms (SNPs) that are characteristic of one or the other species. Cj strain 14980A has a 16 aa *N*-terminal extension, while Cc strains CFSAN032805, YH502, and YH503 have an 11 aa N-terminal extension compared to the consensus (Alignment T in S2 Archive). Additionally, strain RM4661 has an 11 aa *C*-terminal extension that appears to result from an upstream mutation in the gene causing read-through of the normal stop codon. Near the middle of Tlp14 there is a 14aa deletion in strain MG1116, a 6 aa deletion in strain CG8421, and an 11 aa deletion in strain WA333 (Alignment T in S2 Archive). Larger deletions in strains BG2108 and YF2105 start at the same amino acid as the MG116 smaller deletion, with substitution of some amino acids in the case of YF2105. Finally, there is a 7 aa insertion in strain MG1116 starting at aa 371. It is not clear whether any of these Tlp14 proteins are still functional. The variability of these sequence changes suggests that they may be associated with continuing Tlp sequence diversification, though whether there is any selection pressure resulting from a fitness disadvantage of strains carrying these altered Tlps remains a subject for future investigation. Strain WA333 Tlp14 has a large deletion that encompasses the CheW interface domain, which would make the Tlp non-functional; there has not been sufficient selective pressure to prevent this strain from surviving to be cultured and analyzed. Tlp14 was annotated as a pseudogene in Cc strains BG2108, MG1116, WA333 and WF2105 (S1 Spreadsheet). All of the features of this protein underscore the high degree of variability possible for some Tlps.

#### Tlp15 & Tlp16 description and summary

The genes encoding Tlp 15 (see S4 Fasta) and Tlp16 were detected in less than 10% Cc (all annotated as pseudogenes) and 2 Cj genomes. The pseudogenes were identified only by manually “reconstituting” the gene by adding or removing a nucleotide in the appropriate location in silico (see S1 Spreadsheet). Closely related Cc Tlp proteins were found in NCBI blastp searches, though none were full-length (for example, see accession nos. OOX98094.1, WP_072222046.1, WP_079754922.1, WP_023890395.1, WP_078383117.1). In contrast to all other Tlps, a single Cache 2 domain was identified in place of the dCache 1 domain in Tlps 15 & 16 (S1 Fig). Tlp16 differs in strains 14983A, HC2-48, and Rm5611 by having a coherent 13 aa motif replacing the consensus sequence within the dCache1 sensor/binding domain (Alignment U in S2 Archive). A large *C*-terminal amino acid deletion encompassing the CheW interface domain was detected in strain HC2-48. As with other non-functional Tlps, we speculate that these non-functional Tlps may be retained because they serve as raw material for further Tlp differentiation or because they are in the process of being lost altogether.

#### Tlp20 description and summary

The *tlp20* gene was the second most prevalent Tlp identified in Cc and was found in 649 (80%) of Cc and 9 (0.8%) of Cj genomes in large-scale searches; full-length protein sequence could not be reconstituted from the pseudogene DNA sequence for two strains. Similar to *tlp1* in Cj, Cc *tlp20* exhibits high sequence variability with a total of 43alleles. The *N*-terminal halves of Tlp20 proteins have very high sequence identity (Alignment V in S2 Archive). Deletions in the *C*-terminal half of the proteins are found in strains HC2-48 and CF2-75. Only strain 15–537360 has an alternate *C*-terminal peptide sequence. A majority of *tlp20* genes were pseudogenes.

#### Tlp23, Tlp24 & Tlp25 description and summary

All three Tlp23 amino acid sequences were very similar except for the presence of an alternative C-terminal sequence motif in strain 4031 (Alignment W in S2 Archive). All *tlp24* genes were pseudogenes and the ‘reconstituted’ Tlp24 proteins were nearly identical to each other and were similar to Tlp2 (Alignment X in S2 Archive). Two deletions–G1262 and A1303—were responsible for generating a pseudogene with disruption occurring in the *C*-terminal sequence of the protein (Alignment Y in S2 Archive). Though the *tlp25* gene 5ˊ sequence had high identity with both *tlp4* and *tlp12* genes there was a very large internal deletion encompassing most of the 5ˊ half of the *tlp25* gene (Alignment Z in S2 Archive) that would be expected to render the Tlp25 protein non-functional. To make a final determination of Tlp identity or to identify repeats that could be associated with rearrangement of large genomic segments it is therefore necessary to compare DNA sequences.

#### Description and summary of Tlps 17, 18, 19, 21, &22

A blastp search of NCBI protein databases returned 44 Cj strains with full-length proteins having nearly 100% sequence identity with Tlp17 from strain 4031. Tlp17 was not detected in Cc. There were 10 Cc with more than 95% aa sequence identity to strain 76339 Tlp18, and 88 alleles of the *tlp18* gene were detected in blastn searches. Neither Tlp19a nor Tlp19b returned hits with greater than 91% sequence identity, making these proteins unique. There is a possibility that these supposed *tlp* genes could be the result of poor sequence quality or mis-assembly. However, all sequences examined for this analysis were labeled as complete sequences in GenBank and were therefore presumably closed and finished. A blastp search of Tlp21 from Cj subsp. *doylei* detected an identical protein in Cj subsp. *doylei* strain 269.97 but did not have better than 69% identity in any other Cj strain in the database suggesting that this may be a subspecies-specific variant. We have observed that Tlp proteins with less than 94% sequence identity are not usually the same Tlp class and that there is commonly a noticeable gap in % DNA identity or % protein sequence identity differentiating different Tlp classes. Upon investigation of the HF5-4A-4 .gbk file we found that Tlp 22 is a truncated Tlp4 missing 54 aa and with a different 10 aa *N*-terminal sequence (Alignment ZZ in S2 Archive) created by an insertion of the CJIE1 prophage in which the two parts of the protein are separated by 32,057 nt. It is not clear what functionality the truncated Tlp may have, if any.

### Tlp classification and nomenclature

The existence of allelic variation in each of the *tlp* genes and the observation that some Tlps are quite closely related to others creates ambiguity in the classification of different Tlp classes, especially when it becomes evident that there are many more than the initial few detected in NCTC 11168, strain 81–176, and other reference strains that have become workhorses for elucidating Cj and Cc genetics, genomics, and virulence. An additional ambiguity arises from the changes evident in the 3´ relatively conserved half of group A *tlp* genes. Should classification and nomenclature be based on the entire gene (protein) sequence or just the 5´ half associated with substrate binding and initiation of the signal through the cytoplasmic membrane? To answer this question it may be necessary to acquire more data on whether and how changes in the *C*-terminal of Tlps affect downstream interactions to determine the net effects on motility; this is certainly an interesting and important research topic for the near future. Knowing the answer to whether the *C*-terminal half of the Tlp should be included in the development of a final nomenclature is essential because the amino acid/nucleotide changes in variants can reduce the total % identity to a point where the Tlp would not be classed with Tlps identical or very similar in their *N*-terminal substrate-binding halves.

If only the *N*-terminal half of each Group A Tlp is used for classification of Tlps, there is still a question of how much variability can be tolerated without changing the substrate specificity, or what constitutes alleles of a single Tlp class or type. This is a difficult question and one not likely to be answered quickly. Our subjective assessment of BLAST results indicates that, where two Tlps are related but distinct, there is a noticeable gap in the % identity values between the two groups of proteins. We have found this value to be variable depending on the *tlp* gene or Tlp protein used to initiate the search, and think it likely that classifications may change as more *Campylobacter* genome sequences are added to the database. A final classification and nomenclature system may need to await a consensus meeting of researchers most invested in Tlp genetics and biology, as well as a more complete understanding of structure-function relationships in Tlps.

### Genetic context of *tlp* genes in *Campylobacter*

Each *Campylobacter* species studied to date has a unique complement of Tlps. The genes/proteins and tRNAs adjacent to Tlps are also unique to each *Campylobacter* species (S2 Table). ABC transporter substrate-binding proteins detected adjacent to Tlps in both Cj/Cc and in Ch have only 56.2% identity and 71.4% similarity when the representative sequences of each (S5 FASTA) are compared. It is not clear whether there is any biological significance to the location of homologous proteins adjacent to Tlps in different species.

Only fifteen different flanking genes/proteins are found adjacent to genes encoding Tlp proteins in Cj and Cc (S2 Table; F5 FASTA). Eight are found in both species, four only in Cc, and two only in Cj. The gene encoding Tlp1 is always located between genes encoding a **t**wo-**c**omponent **r**esponse **r**egulator (TCRR) and a molybdenum-responsive transcriptional regulator (**M**od**E r**epressor **d**omain **p**rotein; MERDP, though protein annotations vary in different .gbk files) for both Cj and Cc strains (see S1 Spreadsheet). In contrast other *tlp* genes may each be flanked by different combinations of genes, suggesting that some or all of these *tlp* genes can be moved among a limited number of different locations, possibly by recombination (S2 Table). Insertion of a CJIE1 prophage adjacent to Tlp22 in strain HF5-4A-4 resulted in the phage repressor being located beside Tlp22. Within isolates 00–1597, CG8421, R14, RM1221, and S3, there are two Tlps in tandem separated by only one gene. The order of proteins is: SAM-dependent methyl transferase–Tlp–zinc transporter ZupT–Tlp–membrane protein. Similarly, Cc strain FB1 has a similar tandem arrangement of: ATP synthase–Tlp–transcriptional regulator–Tlp–hypothetical protein.

In the Cl strains assessed there are 24 different genes/proteins flanking Tlps, including five instances where two Tlps are adjacent to each other (S2 Table, S1 Spreadsheet). These involve Tlp104 & Tlp105 (3 strains), Tlp103 & Tlp105 (1 strain), Tlp107 & Tlp110 (1 strain), Tlp109 & Tlp113 (1 strain), and Tlp115 & 116 (1 strain). Of the17 Tlp-adjacent genes/proteins in Cf, four are in two pairs of Tlps: Tlp405-Tlp-408 and Tlp407-Tlp410. Tlp403 and Tlp404 are present in all three Cf subspecies (S1 Spreadsheet). There appear to be Tlps associated with only one Cf subspecies, but this may be a function of the small number of strains assessed. There are three Cl Tlps adjacent to tRNA-Gly, Cf Tlp410 is next to tRNA-Arg in Cf, and tRNA-Asn is 5ˊ of Tlp504 in Ccon (S2 Table).

These data lead to questions about how Tlps become distributed so differently in different *Campylobacter* species and what drives their distribution and evolution. Is the loss of function of specific Tlps correlated with differences in host range and niche specificity, or is there sufficient redundancy in the sensing system overall that pseudogene formation is something of an opportunity for further protein differentiation and acquisition of new sensing capabilities? How long are pseudogenes maintained in a strain before they become severely degraded? What selective pressures operate on maintenance of Tlps within the strain and what conclusions can be drawn about the host range, niche adaptation, and public health risk of specific species or strains based on their Tlp content? These will likely be fruitful areas for future research, especially now that the Tlp diversity in *Campylobacter* is becoming better understood.

### Invertible elements of Cj and Cc exhibit species-specific characteristics

Inversions of different sizes have been detected in Cj and may be associated with specific stimuli such as phage predation [46,47]. We previously defined the *Campylobacter* Invertible Element (IE) in Cj as a large (~92 kb), mostly syntenic region of the chromosome, bounded by Group A Tlps, that can exist in opposite orientations in different isolates [30]. The available evidence suggested the entire region may be capable of inversion, with a possible mechanism involving homologous recombination between the highly conserved C-terminal DNA sequences of the bounding Tlps. Approximate locations of the IEs in *Campylobacter* genomes have been mapped using data from closed, finished genome sequences of 38 Cj and 23 *Cc* strains (S3 Table). The locations of the IEs are very different in the two species, with most Cj IEs found in the region between 140,000–280,000 nt from the origin of replication while a majority of Cc IEs are located between 1,430,000–1,705,000 nt clockwise from the origin of replication (S4 Fig). Four Cc IEs span the origin of replication. It is unclear whether these differences in IE location contribute to fitness of the organism or are due to chance. In Cc strains FB1 and 15–537360 the IE is split into two and the parts are located at different places in the chromosome. The presence of outliers in Cj (32488, CFSAN032806) and Cc (BFR-CA-9557) (see S3 Table) suggests that there may not be a large fitness penalty for location of the IE at a different chromosomal location. IEs in the *Campylobacter* strains with closed, finished genome sequences have different lengths in Cj and Cc, with Cc IEs being consistently slightly larger than those in Cj (S3 Table, S4 Table).

Cj IEs are bounded by an ABC transporter substrate-binding protein homologous to Cj0143c in strain NCTC11168 and zinc transporter ZupT homologous to Cj0263 in NCTC11168 except for strains MTVDSCj13 and MTVDSCj16, in which the gene encoding ZupT and two adjacent genes have been lost. The difference in location of the element in Cj strains 32488, CFSAN32806, CJ677CC012 & _257, FDAARGOS_421 & _422, and FJ3124 is therefore the result of other chromosomal changes, indicating that the large-scale chromosomal structure of Cj is dynamic and that chromosomal rearrangement is not uncommon. The structure of the Cc IEs is less constrained. In addition to the terminal Tlps, several Cc strains have an intact gene or a pseudogene encoding a Tlp in the middle of the IE. These arrangements could theoretically produce inversions of either half of the IE.

Including the Tlps at each end, Cj and Cc IEs have 94 relatively conserved genes encoding homologous proteins and a tRNA-Glu (see Additional file 4:S2 Fig and Additional file5:S3 Table within reference [30] for general IE structure and content). A few strains may be missing a small number of the 94 core IE genes, but the correspondence between the two genera is quite good. This synteny suggests a common origin of the IEs, which have then been subjected to acquisition of additional loci due to horizontal gene transfer, including the insertion of one or more prophages (S3 Table). For example, prophage CJIE1 is inserted between IE genes encoding the TonB-dependent receptor and biopolymer transporter ExbB in Cc strains RM4661 and OR12 (IEs spanning YSS_RS08625—YSS_RS08905 and ATE51_RS09880—ATE51_RS10170, respectively), as well as between the IE genes encoding a pathogenicity protein and a serine protease of *Cj* RM1221 (IE consisting of CJE_RS01065—CJE_RS01345). This is consistent with previous findings demonstrating the prophage in different genomic locations in different strains [46,48] and the possibility that CJIE1, originally described as *Campylobacter* Mu-like prophage 1 (CMLP1) [49], behaves like a Mu phage and has somewhat limited selectivity in insertion sites [50,51]. Variants of the CJIE1 prophage are also located between genes encoding the initial chemotaxis protein and the SAM-dependent methyltransferase in the *Cc* OR12 IE (ATE51_RS09170—ATE5_RS09455) and between two hypothetical proteins in *Cj* strain R14 IE (H730_RS00845—H730_RS01105). Further examples of IE insertions include: 1) six genes encoding a transposase with a repeat region, a hypothetical protein, hydrogenase expression protein HypA, pyrrolidone carboxylate peptidase with repeat region, a second hypothetical protein, and a DNA methyltransferase (PJ17_RS01170—PJ17_RS01280) in strain 00–1597 (HS9,37, ST930); 2) a block of genes within the IE encoding a resolvase, a transposase, a hypothetical protein, tetracycline resistance ribosomal protection protein, and a second hypothetical protein (CJSA_RS01020—CJSA_RS01040) in hyperinvasive *Cj* strain IA3902; 3) GMC family oxidoreductase pseudogene and an adjacent hypothetical protein in several *Cj* strains. In addition, within *Cj* IEs there is a frequently detected region of degenerate sequence with very similar DNA sequence, but with different protein annotations in different strains. When present this degenerate region is flanked by restriction endonuclease subunit M/Cj0208 and N-acetyl-gamma-glutamyl-phosphate reductase (ArgC).

Proteins encoded within the Cc IE, but not the Cj IE, include alpha-2 macroglobulin, penicillin-binding protein 1C, CAAX aminoprotease, cytochrome C, a hypothetical protein, and trimethylamine N-oxide reductase I catalytic subunit; the last four genes/proteins are variably present. The IE heterogeneity is not limited to Cj; Cc strain HC2-48 has an IE indel containing two hypothetical proteins and a transcriptional regulator (R446_RS07315—R446_RS07325). Single gene insertions into Cj IEs include those encoding DNA methyltransferase (eg. Cj strainRM1221, Cj strain S3), hypothetical protein/serine protease (eg. CJM1cam, 4031, M1), and a second hypothetical protein (T1-21), while the Cc RM1875 IE includes a gene encoding a NAD(P)H-hydrate dehydratase (YSQ_RS08870).

## Conclusions

This work represents an attempt to develop a framework for understanding type A Tlps, which are one of the most important and interesting types of virulence- and niche-associated factors in *Campylobacter* spp. Tlps demonstrate considerable heterogeneity within each Tlp type in both the N-terminal sensing domain and in C-terminal signalling domains. This beautiful diversity is suggestive of continuing evolution of these type A Tlps within each species complex in the context of barriers that appear to make Tlps species-specific. At least some of the protein sequence variants in specific Tlp classes are sufficiently divergent that we hypothesize Tlp substrate specificity or function may be affected. However, this hypothesis requires further experimental evidence as the mechanisms of Tlp substrate recognition have not yet been thoroughly elucidated for all Tlps. The nomenclature for Tlp classification adapted and used in this work assists in communicating data pertaining to these proteins but should not be considered a final schema; it may be advantageous to completely overhaul the naming conventions as more is learned about the functional attributes of different Tlp classes. All findings for Tlps in species other than Cj and Cc reported here should be considered preliminary. Genomes from too few strains were examined to draw reasonable conclusions about the diversity and nature of Tlps from these organisms.

In addition to their role(s) in chemotaxis Tlps may provide functions related to genomic organization through rearrangement of IEs. The evidence for this includes: 1) the prior detection of IE inversions in some Cj isolates [30], 2) the large “repeat regions” created by DNA sequence identity in the C-terminal half of *tlp* genes that provide potential sites for homologous recombination, and 3) the conserved locations of *tlp* genes within the genome such that an identifiable chromosomal segment–the IE—is defined by terminal Tlp repeats. A multitude of differences between Cj and Cc are recognized [51]. Cc also have different chromosomal locations for Tlps and the IE. The different structure and location of Cc IEs, the lack of complete IEs in some strains, and the presence of a small number of IE genes/proteins characteristic of this species, all suggest that the evolution, and possibly the function, of the IE may be different in *Cc* than in Cj. Furthermore, the rapid adaptation strategies described for these organisms, eg. enhanced host-interaction phenotypes, are linked to several mechanisms, including large-scale genomic rearrangements [52]. Inversion of IEs may represent one class of the large-scale genomic rearrangements involved. Given the potential importance of Tlps for many aspects of *Campylobacter* biology and virulence, this should be a fruitful area for additional investigation.

## Supporting information

S1 FASTA (FASTA)

S2 FASTA (FASTA)

S3 FASTA (FASTA)

S4 FASTA (FASTA)

S5 FASTA (FASTA)

S1 Fig Selected Tlp protein domains. (DOCX)

S2 Fig Alignments of the methylation motif in *Campylobacter* Tlps. (DOCX)

S3 Fig Alignments of dimer interface domains in *Campylobacter* Tlps. (PDF)

S4 Fig Chromosomal locations of Cj and Cc IEs. (DOCX)

S1 Spreadsheet (XLSX)

S1 Table List of strains and accession numbers used in this analysis. (DOCX)

S2 Table Genetic context of Tlps: flanking genes/proteins and tRNAs. (DOCX)

S3 Table Invertible element size in Cj and Cc strains. (DOCX)

S4 Table Descriptive statistics for Cj and Cc Invertible Elements (IEs). (DOCX)

S1 Archive MasterBlastR_RScript. (ZIP)

S2 Archive Alignments of Tlp proteins and *tlp* genes. (ZIP)
