# Supplementary material for: Diversity of transducer-like proteins (Tlps) in Campylobacter
Source: PLoS One. 2019 Mar 25;14(3):e0214228. doi: 10.1371/journal.pone.0214228 (PMC6433261; doi:10.1371/journal.pone.0214228)
Supplement: S1 Fig — (DOCX) [file pone.0214228.s006.docx]

Domains were obtained from BLASTS of the protein sequences using blastp in NCBI. Not all domains present are shown, and not all domains shown are present in every protein.

dCache_1: small molecule recognition

single Cache2 domain small molecule recognition

NIT domain pfam08376 nitrate sensing domain

cache2 domain

CheW interface interaction of Tlp with CheW

Dimer interface

MCP signal domain pfam00015: transduces signal to CheA

Methylation motif A-A-X2-E-E-X2-S-S references [,]

>**NCTC11168_Tlp1 *C. jejuni***

MFKSLNIGLKLIFSVAAVVVIGLVILISLITKQVSQNITKNTEDILASITKEYATQTQGIFGEMIALNKSISGTLTEMFR

STSKEDLDIDNITNIITNTFDNSAYSNFTYLYLIDPPEYFKEESKFFNTQSGKFVMLYADEEKDNKGGIKAIQASDEIAN

LQVVQDILKKAKYGENKVYIGRPIKMNLEGQDFDAVNVAIPIFDRKNQVVGVIGMTLDFSDIATYLLDPKGQKYDGELRVLLNSDGFMAIHPNKNLVLKNLKDINPNKGAQETYKAISEGKNGVFNYIASDGDDSYAAINSFKVQDSSWAVLVTAPKYSVFKPLKKLQLIILGASFIFIFVVLGVVYYCVRKIVASRLPVILSSLESFFRFLNHEKIEPKAIEIRANDELGAMGRIINEN

IEKIQISLEQDQNAVDESVQTAREIEKGNLTARITKNPINPQLVELKDVLNRMLDVLQSKIGSNMNEINRVFDSYKALDF

STEVFNAKGEVEITTNILGKEIKKMLLASSNFAKDLANQSEELKNSMQKLADGSNAQASSLEQSAAAVEEINSSMQNVG

KTVEVASQADDIKNIVNVIKDIAEQTNLLALNAAIEAARAGEHGRGFAVVADEVRQLAERTGKSLSEIEANINILVQSVN

EVAESVKEQTAGITQINDAIAQLETVTKENVEVANVTNNITNEVNQIAAAILEDVNKKRF

>**NCTC11168_Tlp2 *C. jejuni***

MKSVKLKVSLIANLIAVVCLIILGVVTFIFVKQAIFHEVVNAEINYVKTAKNSIESFKARNSLALESLAKSILKHPIEQL

DSQDALMHYVGKDLKNFRDAGRFLAVYIAQPNGELVVSDPDSDAKNLDFGTYGKADNYDARTREYYIEAVKTNKLYITPSYIDVTTNLPCFTYSIPLYKDGKFIGVLAVDILAADLQAEFENLPGRTFVFDEENKVFVSTDKALLQKGYDISAIANLAKT

KEDLEPFEYTRPKDGNERFAVCTKVSGIYTACVGEPIEQIEAPVYKIAFIQTAIVIFTSIISVILLYFIVSKYLSPLAAI

QTGLTSFFDFINYKTKNVSTIEVKSNDEFGQISNAINENILATKRGLEQDNQAVKESVQTVSVVEGGNLTARITANPRNP

QLIELKNVLNKLLDVLQARVGSDMNAIHKIFEEYKSLDFRNKLENASGSVELTTNALGDEIVKMLKQSSDFANALANESG

KLQTAVQSLTTSSNSQAQSLEETAAALEEITSSMQNVSVKTSDVITQSEEIKNVTGIIGDIADQINLLALNAAIEAARAG

EHGRGFAVVADEVRKLAERTQKSLSEIEANTNLLVQSINDMAESIKEQTAGITQINDSVAQIDQTTKDNVEIANESAIIS

STVSDIANNILEDVKKKRF

>**NCTC11168_Tlp3 *C. jejuni***

MLKITKIKRKIMNSIKIKLSLIANLIAIFALIVLGIVSFYFTKTSLYESTLKNQTDLLKVTQSTVEDFRSTNQSFTRALE

KDIANLPYQSLITEENIINNVGPILKYYRHSINALNVYLGLNNGKVLLSQKSNDAKMPELRDDLDIKTKDWYQEALKTND

IFVTPAYLDTVLKQYVITYSKAIYKDGKIIGVLGVDIPSEDLQNLVAKTPGNTFLFDQKNKIFAATNKELLNPSIDHSPV

LNAYKLNGDNNFFSYKLNNEERLGACTKVFAYTACITESADIINKPIYKAAFIQAIVVIIVVVFSVILLYFIVSKYLSPL

AAIQTGLTSFFDFINYKTKNVSTIEVKSNDEFGQISNAINENILATKRGLEQDNQAVKESVQTVSVVEGGNLTARITANP

RNPQLIELKNVLNKLLDVLQARVGSDMNAIHKIFEEYKSLDFRNKLENASGSVELTTNALGDEIVKMLKQSSDFANALAN

ESGKLQTAVQSLTTSSNSQAQSLEETAAALEEITSSMQNVSVKTSDVITQSEEIKNVTGIIGDIADQINLLALNAAIEAA

RAGEHGRGFAVVADEVRKLAERTQKSLSEIEANTNLLVQSINDMAESIKEQTAGITQINDSVAQIDQTTKDNVEIANESA

IISSTVSDIANNILEDVKKKRF

>NCTC11168_Tlp4 ***C. jejuni***

MQSINSGKSVGISAKLTLWVGILVVLILAITSAISYFDSRNNTYELLKDTQLKTMQDVDAFFKSYAMSKRNGIQILANEL

TNRPDMSDEELINLIKVIKKVNDYDLVYVGFDNTGKNYQSDDQILDLSKGYDTKNRPWYKAAKEAKKLIVTEPYKSAASG

EVGLTYAAPFYDRNGNFRGVVGGDYDLANFSTNVLTVGKSDNTFTEVLDSEGTILFNDEVAKILTKTELSINIANAIKAN

PALIDPRNQDTLFTAKDHQGVDYAIMCNSAFNPLFRICTITENKVYTEAVNSILMKQVIVGIIAIIIALILIRFLISRSL

SPLAAIQTGLTSFFDFINYKTKNVSTIEVKSNDEFGQISNAINENILATKRGLEQDNQAVKESVQTVSVVEGGNLTARIT

ANPRNPQLIELKNVLNKLLDVLQARVGSDMNAIHKIFEEYKSLDFRNKLENASGSVELTTNALGDEIVKMLKQSSDFANA

LANESGKLQTAVQSLTTSSNSQAQSLEETAAALEEITSSMQNVSVKTSDVITQSEEIKNVTGIIGDIADQINLLALNAAI

EAARAGEHGRGFAVVADEVRKLAERTQKSLSEIEANTNLLVQSINDMAESIKEQTAGITQINDSVAQIDQTTKDNVEIAN

ESAIISSTVSDIANNILEDVKKKRF

**>00-2425_Tlp11 *C. jejuni***

MNFRSLNLSTKLILSVAIGIVLGIVVIVLTVSIYTSKSMEKEAKDSIFLSSKRYVNYMEGILNEEVVLTKAMATSLNEIF

SKNDQVNAGIIESLLRNTFDSSGYAAYAFLYLQDSSILTHVESLDKNFKNSDGKSVTMIFFDETTGKAGGIKSIHAPSNF

SQLPIIEKIKKNARYGDLDTIFLGSPSRLNYDGTEFLGINLGMPLFNKEGKFIGIVGFTFDFLEISETILDPKLDFYKDD

LRFLITDQGVIVIHKNKDAILKTLPEINQDASVQLIIDAVKNHKDLIIDNYVDLSGNLSYAGVASFSTLGDSSHWSMVVT

APKKSIFAPLYELNFILISIAIIVLIAILIILYFCVKNIVGSKLPIIVNSLQNFFDFINHKTKNVSTIEVKSNDELGQMG

KIINENILATKRGLEQDNQAVKESVQTVSVVEGGNLTARITANPRNPQLIELKNVLNKLLDVLQARVGSDMNAIHKIFEE

YKSLDFRNKLENASGSVELTTNALGDEIVKMLKQSSDFANALANESGKLQTAVQSLTTSSNSQAQSLEETAAALEEITSS

MQNVSVKTSDVITQSEEIKNVTGIIGDIADQINLLALNAAIEAARAGEHGRGFAVVADEVRKLAERTQKSLSEIEANTNL

LVQSINDMAESIKEQTAGITQINDSVAQIDQTTKDNVEIANESAIISSTVSDIANNILEDVKKKRF

**>RM1221_Tlp12 *C. jejuni***

MQKMDSGKSVGVSVKLTLWVGILVVLILAITSTVSYFDAKNHTYELLKENQLKTMDDVKVTFENYSKSKQKAIEVLAYES

AKKLEDENISLLLDSFKKAFDFDIVFIAFDKNNKMLLSNGTILDKKSNFDITKQIWYQEAKNNKGITITQPYKSPIDQEI

GITYVFPIYKNNQLIAFVGGDYNLDKFSKDVLSLGHSSTTYAAVYDSEGRIIFHEVLDRILTKNTLSVNIANAIKENPEY

IDPNKRDILFPVFDDKGIKYETMCDTSSNGLYRICAVTLDSNYTSAVNSILMKQVIVGIIAIIIALILIRFLISRSLSPL

AAIQTGLTSFFDFINYKTKNVSTIEVKSNDEFGQISNAINENILATKRGLEQDNQAVKESVQTVSVVEGGNLTARITANP

RNPQLIELKNVLNKLLDVLQARVGSDMNAIHKIFEEYKSLDFRNKLENASGSVELTTNALGDEIVKMLKQSSDFANALAN

ESGKLQTAVQSLTTSSNSQAQSLEETAAALEEITSSMQNVSVKTSDVITQSEEIKNVTGIIGDIADQINLLALNAAIEAA

RAGEHGRGFAVVADEVRKLAERTQKSLSEIEANTNLLVQSINDMAESIKEQTAGITQINDSVAQIDQTTKDNVEIANESA

IISSTVSDIANNILEDVKKKRF

**>00-1597_Tlp13 *C. jejuni***

MFRLSSVSSKLLLSVAISVILATALMIAIVSFQVASYSEKEAKDTIFLSSKRYVNYIQGMLNEEVTLTKGVATSLNEMFQ

NNDHIDIDLIESLIKNTFDSSHYAAYTFLYLKDTTVLSDMQNVDKKYISPDGKTFSMIFFDQIVEKSGGITTISTPNNFS

QLNLIQNIEQNAKYGDKDSVFVDSPRKLNYDNNEFLGINFGMPIFNNKGKFIGVIGYTIDLLEISETILDPKFDFFEGDL

RFLMNDQGIIAIHKNKNAILKTLFDINKDQSAQLIVEAVKNHKDEILDNYIASTGDLSYASISSFSTLGNSSHWSVIVTA

PKKSVLAPLYKLQYIIISVAIIALIAILAVVYFFIRKIIGSRIPLILKSLENFFRFLNHEKIEVQTIEIKANDELGKMGK

IINENILATKRGLEQDNQAVKESVQTVSVVEGGNLTARITANPRNPQLIELKNVLNRLLDALQARVGSDMNEIQRVFNSY

KSLDFTTEVKDANGAVEVTTNALGQEIIKMLKQSSDFANALANESGKLQTAVQSLTTSSNSQAQSLEETAAALEEITSSM

QNVSVKTSDVITQSEEIKNVTGIIGDIADQINLLALNAAIEAARAGEHGRGFAVVADEVRKLAERTQKSLSEIEANTNLL

VQSINDMAESIKEQTAGITQINDSVAQIDQTTKDNVEIANESAIISSTVSDIANNILEDVKKKRF

**>00-1597_Tlp14 *C. jejuni***

MNNIKIKLSVIANSIAIFALSILSIISFYFTKDSLYQSTLYTETELLKATQISIEDFRSRNISLLNTLEKDILKLPYEAL

NSQDNIVNNVGAILKYYRNSGNLLAVYIGLDNGENIMSSDLSEKKNTNITINGKANNYNATTREWYKEARNSNQIYITPA

YIDVVSNEYCITYSKALYKDGKFIGVLGFDVLLTSLQDRIARTPGNTFVFDHKDKVFAATNKALLDPSVDHSPVLNAYKA

HGDNNFFSYKLNNEERLGACTKVFAYTACITESTDVINKPIFKAAYIQVIALIIMISISIILLYFIVSKYLSPLAAIQTG

LTSFFDFINYKTKNVSTIEVKSNDEFGQISNAINENILATKRGLEQDNQAVKESVQTVSVVEGGNLTARITANPRNPQLI

ELKNVLNRLLDALQARVGSDMNEIQRVFNSYKSLDFTTEVKDANGAVEVTTNALGQEIIKMLKQSSDFANALANESGKLQTAVQSLTTSSNSQAQSLEETAAALEEITSSMQNVSVKTSDVITQSEEIKNVTGIIGDIADQINLLALNAAIEAARAGEHG

RGFAVVADEVRKLAERTQKSLSEIEANTNLLVQSINDMAESIKEQTAGITQINDSVAQIDQTTKDNVEIANESAIISSTV

SDIANNILEDVKKKRF

**>RM1875_Tlp15 *C. coli***

MKLSIRKKMLMLGGICLVSMLITFGIFYYNNLQGSEKIAQITKNLINKEINVKVELLTKSMAIALGDLIKNVHSEEEKVK

IIATAIENFRFEEDKSGYFFVYQKTTVKAHPVRKDLIGTDLYNAKDENGVFYVRELYQRALEKGGFVTFHFTKPQPNGEN

TIAEKTAYSYLIPNADDLWISTGVYKDTLEPYIDGNLKNCYHFFSKNFFKTIIFFTLFILIIIPFIFIFYRNLITGVQGI

KTNITSFFDFINHKTKNVSTIEVKSNDEFGQISKAINENILATKQGLEQDAKAVKESVETVGVVESGNLTARITANPRNP

QLIELKNVLNRLLDVLQTRVGSDMNAIHKIFEEYKSLDFRNKLDNANGSVEVTTNALGDEIVKMLKQSSDFANHLASESS

KLQSAVQNLTSSSNSQAASLEETAAALEEITSSMQNVSVKTSDVITQSEEIKNVTGIIGDIADQINLLALNAAIEAARAG

EHGRGFAVVADEVRKLAERTQKSLSEIEANTNLLVQSINDMAESIKEQTAGITQINESVAQIDQTTKDNVEIANESAIIS

STVSDIANNILEDVKKKRF

**>FB1_Tlp16 *C. coli***

MQLSIRKKMLMLGAICFISMLATFAIFYYNNLKGSQKIAQTTKNLINKEIDIKVELLTKSMAIALGDLIKDVDDEKEKIK

ISLPQLKILDLKRIKSGYFFVYQKTTVKAHPVRKDLIGTDLHNAKDENGIFYVRELYQRALDKGGFVTFHFTKPQPNGEN

TIAEKTAYSYLIPNADDLWISTGVYKDTLEPYIDRSLEELLSFFSKSFFKTVLFSIIFILIIIPFIFIFYRNLIVGVQGI

DANITSFFNFINHKTKNVSTIDVKTNDEFGLISKAINENILATKQGLEQDAKAVKESVETVGVVERGNLTARITANPRNP

QLIELKNVLNRLLDVLQTKVGSDMNAIHKIFEEYKSLDFRNKLDNANGSVEVTTNALGDEIVKMLKQSSDFANHLASESS

KLQSAVQNLTSSSNSQAASLEETAAALEEITSSMQNVSVKTSDVITQSEEIKNVTGIIGDIADQINLLALNAAIEAARAG

EHGRGFAVVADEVRKLAERTQKSLSEIEANTNLLVQSINDMAESIKEQTAGITQINESVAQIDQTTKDNVEIANESAIIS

STVSDIANNILEDVKKKRF

**>4031_Tlp17 *C. jejuni***

MNFRSLNISTKLILSVAIGVILGIIVLVSTVSIYISENMEKEAKDSIFLASKRYTNYMEGILNETVALTKGTATSLNDMF

EHNNQVDADLIESLMKNLFDSSLYSAYTFLYLKDTSVLGDAQGIDKRYTSSDGKTFAMIYFDQTTGKSGGIETIQTPNNF

GNLKIIEQVEKNAKYGDKDSLFVGPPTKLNYDGKDFLGINFGMPIFNNKGKLIGVAGYTLDFSEVSETILDPKLDFFEGD

LRFLMTDKGVITIHKNHNAILKTLGDINKDPSVELVNNAVKEHKTVIIDDYVASTGDLSYASVSSFSTANNSSHWSMVVT

APKNSVLAPLKKLEIIFIIISFFILLVILIIVYVCVKKIVGSRIPVILKSLENFFHFLNHKKHEVDLISIKADDELGKMG

KMINENILATKKGLEQDNQAVKESVQTVSVVESGNLTARITANPRNPQLIELKNVLNKLLDVLQARVGSDMNAIHKIFEE

YKSLDFRNKLENASGSVELTTNALGDEIVKMLKQSSDFANALANESGKLQTAVQSLTTSSNSQAQSLEETAAALEEITSS

MQNVSVKTSDVITQSEEIKNVTGIIGDIADQINLLALNAAIEAARAGEHGRGFAVVADEVRKLAERTQKSLSEIEANTNL

LVQSINDMAESIKEQTAGITQINESVAQIDQTTKDNVEIANESAIISSTVSDIANNILEDVKKKRF

**>76339_Tlp18 *C. coli***

MFKSLNIGSKLVLSVAVSVIAAIAILITILSFEVASYAEKEAKDTIFLSSKRYANYMEGVLNESVVLTKGISASINEMFS

KHDQVGADLIESLLKNTFDSSGYAAYAFLYLKDPSVLSDTYNMDKKYKSQNGNTFAMIFFDETTGKSGGIKAIQTPDNFS

QLRIIQDIEKNARYGSRDTLFIGSPTKLNYDGTEFLGINFGMPIFNSKGNFIGVVGYSLDFLEISQAMLDPKLDFFEGDL

RALTTDQGVITIHKDKNAILKTLTDINKDPSVKLITDLIKEHKDALIDNYVASTGDLSYASVVSFNTLGDSSRWSMIVTA

PKKSALEPLFRLQFAIITTAIIALIVILFIVYFCVRKIVGIRIPVILKSLEDFFRFLNHEKIEVHTIKISSNDELGKMAK

AINENILATKQGLEQDAKAVKESVETVEVVERGNLTARITANPRNPQLIELKNVLNKLLDVLQTKVGSDMNAIHKIFEEY

KSLDFRNKLDNANGSVEVTTNALGDEIVKMLKQSSDFANHLASESSKLQSAVQNLTSSSNSQAASLEETAAALEEITSSM

QNVSVKLSDVITQSEEIKNVTGIIGDIADQINLLALNAAIEAARAGEHGRGFAVVADEVRKLAERTQKSLSEIEANTNLL

VQSINDMAESIKEQTAGITQINESVAQIDQTTKDNVEIANESAIISNTVSDIANNILEDVRKKRF

**>T1-21_Tlp19a *C. jejuni***

MNSIKIKLSLIANLIAIFALIVLGIVSFYFTKTSLYESTLKNQTDLLKVTQSTVEDFRSTNQSFTRALEKDIANLPYQSL

ITEENIINNVGPILKYYRHSINALNVYLGLNNGKVLLSQKSNDAKMPELRDDLDIKTKDWYQEALKTNDIFVTPAYLDTI

LKQYVITYSKAIYKDGKIIGVLGVDIPLEDLQNSVANTPGNTFLFDQKNKIFAATNKELLNPSIDHSPVLNAYKTHGDYN

FFTYGLDGKERLGTCTKVFAPLPTCSYPNRFNFILYFINYKTKNVSTIEVKSNDEFGQISNAINENILATKRGLEQDNQA

VKESVQTVSVVEGGNLTARITANPRNPQLIELKNVLNKLLDVLQARVGSDMNAIHKIFEEYKSLDFRNKLENASGSVELT

TNALGDEIVKMLKQSSDFANALANESGKLQTAVQSLTTSSNSQAQSLEETAAALEEITSSMQNVSVKTSDVITQSEEIKN

VTGIIGDIADQINLLALNAAIEAARAGEHGRGFAVVADEVRKLAERTQKSLSEIEANTNLLVQSINDMAESIKEQTAGIT

QINDSVAQIDQTTKDNVEIANESAIISSTVSDIANNILEDVKKKRF

**>CO2-160_Tlp20 *C. coli***

MKSVKIKVSLIANLIAIVCLIFLGIITFIFVKDEVFNQVVKSESNYVRTAKNSMEAFKARNTAALESLAKNILKLPYEQI

SNQEALMRYVGKDLKVFRDAGGFLAVYIAQPDGELVVTNPDSDEKGLNFGIYGKADNYDARTRDYFKGAVKANGLYVTPSYLDLTTNLPCFTYATPLYKEGKFIGVLAIDILVKDLQREFENLPGRTFVFDSENSIFVSTDKELLKPGYDVSPVANIAKD

KKDYEPFRYVRPLDGTQRFGVCAKVLGEYTACVGESIDYIEEPVFKIAYIQIAIVIITSIISVLLLYFIVSRYLSPLASI

QVGLNSFFDFINHKTKNVSTIDVKTNDEFGQISKAINENILATKQGLEQDAKAVKESVETVGVVESGNLTARITANPRNP

QLIELKNVLNRLLDVLQTKVGSDMNAIHKIFEEYKSLDFRNKLDNANGSVEVTTNALGDEIVKMLKQSSDFANHLASESS

KLQSAVQNLTSSSNSQAASLEETAAALEEITSSMQNVSVKTSDVITQSEEIKNVTGIIGDIADQINLLALNAAIEAARAG

EHGRGFAVVADEVRKLAERTQKSLSEIEANTNLLVQSINDMAESIKEQTAGITQINESVAQIDQTTKDNVEIANESAIIS

STVSDIANNILEDVKKKRF

**>FDAARGOS_295_Tlp21 *C. doylei***

MKSSISTKLTILIGILIVLAFGISSMISYLSSLNNSRSLLQNNQMTVLKNTATAFENANANKELTMQALAKDLAKNLNNE

KDIYTILADFKNLTLFDSAFFGYDKMGKTYLSSGDYLDLSKNYDVTTRAWYKGAKENNGIVITPPYLSRSTGNIAIGYGI

PVVVEGKIVGVVGSEYNLANYAKDVLSVGRSQNTYTAIYDPQGTILFHEKTELMLQKNTLSTNITKIINQNTALLNAKTP

FVVDNGEGEQYEAFCRNVVSDFYRMCTLTQSKIYSDMANEILFKQILIGIIAISVILLFIQLIIKKYLSPLAAIQTGLTS

FFDFINHKTKNITAINIKSKDEFGQMANAINENILATKKGLEQDNQAVKESVQTVHVVESGNLTARITANPRNPQLIELK

NVLNKLLDVLQARVGSDMNEIQRVFNSYKSLDFTTEVKDANGAVELTTNALGDEIIKMLKQSSDFANALANESGKLQTAVQSLTTSSNSQAQSLEETAAALEEITSSMQNVSVKTSDVITQSEEIKNVTGIIGDIADQINLLALNAAIEAARAGEHGRGF

AVVADEVRKLAERTQKSLSEIEANTNLLVQSINDMAESIKEQTAGITQINDSVAQIDQTTKDNVEIANESAIISSTVSDI

ANNILEDVKKKRF

**>HF5-4A-4_Tlp22 *C. jejuni***

MTLICKAVLYYAMSKRNGIQILANELTNRPDMSDEELINLIKVIKKVNDYDLVYVGFDNTGKNYQSDDQILDLSKGYDTK

NRPWYKAAKEAKKLIVTEPYKSAASGEVGLTYAAPFYDRNGNFRGVVGGDYDLANFSTNVLTVGKSDNTFTEVLDSEGTILFNDEVAKILTKTELSINIANAIKANPALIDPRNQDTLFTAKDHQGVDYAIMCNSAFNPLFRICTITENKVYTEAVNSIL

MKQVIVGIIAIIIALILIRFLISRSLSPLAAIQTGLTSFFDFINYKTKNVSTIEVKSNDEFGQISNAINENILATKRGLE

QDNQAVKESVQTVSVVEGGNLTARITANPRNPQLIELKNVLNKLLDVLQARVGSDMNAIHKIFEEYKSLDFRNKLENASGSVELTTNALGDEIVKMLKQSSDFANALANESGKLQTAVQSLTTSSNSQAQSLEETAAALEEITSSMQNVSVKTSDVITQSEEIKNVTGIIGDIADQINLLALNAAIEAARAGEHGRGFAVVADEVRKLAERTQKSLSEIEANTNLLVQSINDMAESIKEQ

TAGITQINDSVAQIDQTTKDNVEIANESAIISSTVSDIANNILEDVKKKRF

**>ICDCCJ07001_Tlp23 *C. jejuni***

MKSVKLKVTLIANLITVVCLVILGVITFMFVKQAIFHEVVNAEINYVKTAKNSIESFKARNSLALESLAKSILKHPVEQL

DNQDALMHYVGKDLKNFRDAGRFLAVYIAQPNGELVVSDPDSDAKNLDFGTYGKADNYDARTREYYIEAVKTNKLYITPSYIDVTTNLPCFTYSIPLYKDGKFIGVLAVDILAADLQAEFENLPGRIFVFDEENKVFVSTDKTLLQQGYDISTIANLAKT

KKDFEPFEYTRPKDGSERFAVCVKVSGIYTACVAKPIEQIEAPVYKAAFIQAIVVIIVVVFSVILLYFIVIKYLSPLAAI

QTGLTSFFDFINHKTKNVSTIEVKSNDEFGQISNAINENILATKRGLEQDNQAVKESVETVHVVEGGNLTARITANPRNP

QLIELKNVLNRLLDALQARVGSDMNEIQRVFNSYKSLDFTTEVKDANGAVEVTTNALGQEIIKMLKQSSDFANALANESGKLQTAVQSLTTSSNSQAQSLEETAAALEEITSSMQNVSVKTSDVITQSEEIKNVTGIIGDIADQINLLALNAAIEAARAG

EHGRGFAVVADEVRKLAERTQKSLSEIEANTNLLVQSINDMAESIKEQTAGITQINDSVAQIDQTTKDNVEIANESAIIS

STVSDIANNILEDVKKKRF

**>M1_Tlp24 *C. jejuni***

MKSVKLKVALIANLIAVVCLVILGVITFMFVKQAIFHEVVKAETNYVKTAKNSMESFKARNSLALESLAKSILKHPVEQL

DSQDALMRYVGKDLKNFRDAGRFLAVYIAQPNGELVVSDPDSDAKKVDFGTYGKADNYDARTREYYIEAVKTNKLYVTPSYIDATTNLPCFTYSTPLYKDGKFIGVLAVDVLVTDLQAEFENLPGRTFVFDEENKVFASTDKTLLQQGYDISAIANLAKI

KENFEPFEYTRPKDGSERFAVCTKVSGVYTACVGEPIEQIEAPVYKIAFIQTAIVIFTSIISVILLYFIVSKYLSPLAAI

QTGLTSFFDFINHKTKNVSTIEVKSNDEFGQISSAINENILATKRGLEQDNQAVKESVETVSVVESGNLTARITANPRNP

QLIELKNVLNKLLDVLQARVVLI.CYS.NF.RIQSLDFRNKLENASGSVELTTNALGDEIVKMLKQSSDFANALANESGK

LQTAVQSLTTSSNSQAQSLEETAAALEEITSSMQNVSVKTSDVITQSEEIKNVTGIIGDIADQINLLALNAAIEAARAGE

HGRGFAVVADEVRKLAERTQKSLSEIEANTNLLVQSINDMAESIKEQTAGITQINESVAQIDQTTKDNVEIANESAIISS

TVSDIANNILEDVKKKRF

**>CG8421_Tlp25 *C. jejuni***

MQSINSGKSVGVSVKLTLWVGILVVLILAITSTVSYFDAKNHTYELLKENQLKTMDDVLMINIKRFRYKKSCYSILMKQV

IVGIIAIIIALILIRFLISRSLSPLAAIQTGLTSFFDFINYKTKNVSTIEVKSNDEFGQISNAINENILATKRGLEQDNQ

AVKESVQTVSVVEGGNLTARITANPRNPQLIELKNVLNKLLDVLQARVGSDMNAIHKIFEEYKSLDFRNKLENASGSVEL

TTNALGDEIVKMLKQSSDFANALANESGKLQTAVQSLTTSSNSQAQSLEETAAALEEITSSMQNVSVKTSDVITQSEEIK

NVTGIIGDIADQINLLALNAAIEAARAGEHGRGFAVVADEVRKLAERTQKSLSEIEANTNLLVQSINDMAESIKEQTAGI

TQINDSVAQIDQTTKDNVEIANESAIISSTVSDIANNILEDVKKKRF

**>*C. lari*_SlaughterBeach_Tlp100**

MILILSSVIFIGIGILSVTIISKSQEMLNDEAHKLLLSSANRYSNGIQAITQNAYSTLETAQGVIKNFANKDNNLDIEDL

KILISSMLDSNSWTYFAYIHLNQYHDNNPLNLTPSGKFLLLAKDENPQQKGSIKFIQAEEVILQQNSLIKALQTKQPAVG

RPRDYSINGEKLYLVNIVLPIFGKNNETIGAIGMLVRIDLLREELNDPNKSLFANDQRLLISSDGLIISSPKAEYIGKII

TEINPHPSAKTILDMQSTKTNGLFTFIPASTNEENLAQLVNFDLWEGSNDHWSVVTIAPKKSVEKPADSLAFIIFAISTI

VLFIIISVIYFYVKKSVVGAIHKLQTGLNSFFDFINHKTKDSAMIDVKTNDELGAMAKAINENITKTKNALEQDAKAVEQ

SVDTAKEIEGGNLTARITAIPANPQLVELKNVLNEMLNVLEQKVGSNMNEINRVFDSYKALDFTTEVKNAKGGVEVTTNVLGQEIVAMLRQSSEFASLLADESGKLQSAVKNLTDSSSSQASSLEETAAALEEITSSMQNVSHKTSEVIAQSEEIKNVTS

IIGDIADQINLLALNAAIEAARAGEHGRGFAVVADEVRNLAERTQKSLGEIEANTNILVQSINEMGESIKEQTTGITQIN

DAVAQIDHVTQENLKIAKDSAIVADNVNKIASDILEDARKKKF

**>*C. lari*_RM16712_Tlp101**

MKSIKIKISLISNIIAIICLVILGVISFIFTEKALNYEVVKAETNYVKAAEKSMRDFKNTHSHALKQLSQTITRLSYQEL

NTQEKLMNNTGELLKTVRDMNNYLAVYIAQPNGELIVSDPDSDSKGLNYGIYGKADNYDATTREFYIEAKKKNGLYITPS

YIDVTTGLPCFTYSMPLVKDGKFIGILAIDVLVKDLQTEFSELPGRTFVFDQAYTVFASTDKSLISAEQNPDIITVAKAY

EKAGDYNIFSYSTKNGQDRFGICVKIDNYTTCAGENIEVIKAPALKIAYIQATIVIFTSIASIVLLYFIISYFLSPLQSI

QTGLNSFFDFINYKTKDSAMIDVKTNDELGAMAKAINENITRTKNALEQDAKAVEQSVDTAKEIESGNLTARITAIPANP

QLVELKNVLNDMLNVLEQKVGSNMNEINRVFDSYKALDFTTEVKNAKGGVEVTTNVLGQEIVAMLRQSSEFASLLADESGKLQSAVKDLTDSSSSQASSLEETAAALEEITSSMQNVSHKTSEVIAQSEEIKNVTSIIGDIADQINLLALNAAIEAARAG

EHGRGFAVVADEVRNLAERTQKSLGEIEANTNILVQSINEMGESIKEQTTGITQINDAVAQIDHVTQENLKIAKDSAAIS

DNVNKIANDILEDARKKKF

**>*C. lari*_SlaughterBeach_Tlp102**

MKSIKLKISLIANIIAIICLIILGIISYIFTKKALNHEVVNAETNYVKVAEKSMRDFKSLHTHSLEQLSQAILRLPYNEL

NTQEKLMENTGDLLKTVRDINSYLAVYIAQSNGELIVSDPDSDSKGLDYGIYGKADNYDATTREFYIEARKKNGLYITAA

YIDATTGLPCFTYAMPLIKDGKFIGVLAIDVLVKDLQEKFNELPGRTFVFDHAYTVFASTDKSLVGGEQNPDIVTVAKAY

ENAGNYNIFNYTTQNGGDRFGICVKIDGYTTCAGEDVEVIETPALKIAYIQTTIVIFTSIASIILLYFIISYYLSPLQAI

QTGLNSFFDFINHKTKDSAMIDVKTNDELGAIAKAINENITKTKNALEQDAKAVEQSVDTVREVEGGNLTARITAIPAHP

QLLELKNYINEMLNVLEQKVGSNMNEINRVFDSYKALDFTTEVKNAKGGVEVTTNVLGQEIVAMLRQSSEFASLLADESGKLQSAVKNLTDSSSSQASSLEETAAALEEITSSMQNVSHKTSEVIAQSEEIKNVTSIIGDIADQINLLALNAAIEAARAG

EHGRGFAVVADEVRNLAERTQKSLGEIEANTNILVQSINEMGESIKEQTTGITQINDAVAQIDHVTQENLKIAKDSAAIS

DNVNKIANDILEDARKKKF

**>*C. lari*_RM16712_Tlp103**

MFQSITSRLTLVIAIISIIVLVGVNGLSYYNIKEDTYEYLEEIQKKTMLDTAEVFFIYSNAKRKAVSTLAEEIVKQDFSN

DENIYNFLEAFKKANNFDIIYFALEENGKHYKSDHTYLDKSKGFDVKTRAWYINAKKEGGLIVSDPYSSFADGKMKIAYA

MPVFKNDKFIGVVGGDYDLERFSKDVLSVGKSSQAYTAIYDIEGNAFFHPEVEKIGKKDELSENISVYMRANPHFLELNN

ENAIAYIPNEQGIMEAIMCTNSFNSKYKVCTVTKEKVYSEKVNEALIKQIIIGAISLVIALIVIRFMINYNLSPLKKIQT

GLNSFFDFINYKTKDSAMIDVKTNDELGAMAKAINENITRTKNALEQDAKAVEQSVDTAKEIESGNLTARITAIPANPQL

VELKNVLNDMLNVLEQKVGSNMNEINRVFDSYKALDFTTEVKNAKGGVEVTTNVLGQEIVAMLRQSSEFASLLADESGKLQSAVKDLTDSSSSQASSLEETAAALEEITSSMQNVSHKTSEVIAQSEEIKNVTSIIGDIADQINLLALNAAIEAARAGEH

GRGFAVVADEVRNLAERTQKSLGEIEANTNILVQSINEMGESIKEQTTGITQINDAVAQIDHVTQENLKIAKDSAAISDN

VNKIANDILEDARKKKF

**>*C. lari*_RM16712_Tlp104**

MFKFNSLSNKITMIACSLIAIILIVANIINYYQSKESTRYYLEEIQKKTMFDVNSAYNIYSGAKREAISSIVKFVEKNPK

VDTAEIFDMLETIKEAAGFNVVYIGFNDDGKLYQSNRIIRSPEETGFDARTRSWYQEAKAAGKLVVSDPYKSIEDGSITV

SYTAPILVNGKLLAVVGGDYDLERFSKDVLVMGHSQSSYAAVYDKNDGSIIFHEDKDRMLTKNDLSINIANAVKSDPDLI

DPNKEESLFYAKDGAGKTQVVTCNQALNPKYVVCSITDESVYTDAVNKVLFQQVIIALIAIIVALILVRFAIIKNLKPIA

VITTGLNSFFDFINYKTKDSAMIDVKTNDELGAMAKAINENITRTKNALEQDAKAVEQSVDTAKEIESGNLTARITAIPA

NPQLVELKNVLNDMLNVLEQKVGSNMNEINRVFDSYKALDFTTEVKNAKGGVEVTTNVLGQEIVAMLRQSSEFASLLADESGKLQSAVKDLTDSSSSQASSLEETAAALEEITSSMQNVSHKTSEVIAQSEEIKNVTSIIGDIADQINLLALNAAIEAAR

AGEHGRGFAVVADEVRNLAERTQKSLGEIEANTNILVQSINEMGESIKEQTTGITQINDAVAQIDHVTQENLKIAKDSAA

ISDNVNKIANDILEDARKKKF

**>*C. lari*_Slaughter_Beach_Tlp105**

MGKITKTLTGKLTFFAFLAIIAILFIANAFNYAEVKHDVQKLINDIQVKTMQDVLKSFDDYTASRSDAIKAVAAEIQKNP

NASLEEIYTMVKVAKESSRFDVLYVGLAKNGAMIRSNGNHQMPSDGYDPRTRTWYTSVSSGENKVVISKPYMAPSLKAPSLAFSYPIVVDGKFIGAVGGNYDLNTFSDNVLAMGRSQSGYTVVLDDEGTVLFHESSKALLTKDDLSQNIVKAYLSTPEGKAGQLSSEPMIIEDGSAPRKAVICQESSTGYNVCVIADEKIYKDPVNKALVKQIIIGAISLVIALIVIRFMINYNLSPLQK

IQTGLNSFFDFINHKTKDSAMIDVKTNDELGAMAKAINENITKTKNALEQDAKAVEQSVDTAKEIEGGNLTARITAIPAN

PQLVELKNVLNEMLNVLEQKVGSNMNEINRVFDSYKALDFTTEVKNAKGGVEVTTNVLGQEIVAMLRQSSEFASLLADESGKLQSAVKNLTDSSSSQASSLEETAAALEEITSSMQNVSHKTSEVIAQSEEIKNVTSIIGDIADQINLLALNAAIEAARA

GEHGRGFAVVADEVRNLAERTQKSLGEIEANTNILVQSINEMGESIKEQTTGITQINDAVAQIDHVTQENLKIAKDSAAI

SDNVNKIANDILEDARKKKF

**>*C. lari*_RM16712_Tlp106**

MFLSKNMSVKNKLSIIVAAIVALALFVITVMAFYSSREDLILNSKKSNEDYLLVTTTQVEGYVENYVDILLAIKKHIDQL

PEYQIKNFDTLSEYFAKDLKIFKDGSNTLAVYLGFPDGTMLVSDADSDKKEIPFRKRGGGIAHYDDPKYNATSRDWYKGALKNDGIYISDVYEDSVTKLPSFTYSVPIKKNGKLVAVLGIDLLLTSLQKTFDKLPGNVFVFDLASSIPFASNDKTLIMKD

YPSINEIKKYHKMVGDYKNFEYTGISSNEKRFGICANINNSKARINYVACAIQKQDSLDKLVIKNAFEQTLISIVILILS

CIFIHLFSAKLLSPLQSIQTGLNSFFDFINYKTKDSAMIDVKTNDELGAMAKAINENITRTKNALEQDAKAVEQSVDTAK

EIESGNLTARITAIPANPQLVELKNVLNDMLNVLEQKVGSNMNEINRVFDSYKALDFTTEVKNAKGGVEVTTNVLGQEIVAMLRQSSEFASLLADESGKLQSAVKDLTDSSSSQASSLEETAAALEEITSSMQNVSHKTSEVIAQSEEIKNVTSIIGDIA

DQINLLALNAAIEAARAGEHGRGFAVVADEVRNLAERTQKSLGEIEANTNILVQSINEMGESIKEQTTGITQINDAVAQI

DHVTQENLKIAKDSAAISDNVNKIANDILEDARKKKF

**>*C. lari*_LMG11760_Tlp107**

MLSSLKIKLSLVANLFAALCLIILGILSFYFTKNFLYSNELKRQNDILQVAKISLETFRDINTNLITNLEKSILEHPYEK

LNSEEALIENIGSTLKSYRKASGVLAVFIGLDNGENIVSDNNSDQKNRNVGIYGKAMNYDTRTRSWFIEAKKTNNVFITT

PYIDKATNQYVITYTKAIYKDNRFIGVIGIDIPIKDLQKDFESMPGNSFLFDHNGKVFVAKNKQLLDPSVDHTPVLNAYK

KNGDYTFFEYGLKNKERLGICAQISSYLVCSTESADIINEPIFKTASIQTIAVTVMVILSIILLYFIISYYISPLQKIQT

GLNSFFDFINHKTKDSAMIDVKTNDELGAMAKAINENITKTKNALEQDTKAVEQSVETAREIESGNLTARINAMPANPQLIELKNVLNEMLNVLEQKVGSNMNEINRVFDSYKALDFTTEVANAKGGVEVTTNVLGKEIVAMLRQSSEFANLLASESGKLQSAVKNLTDSSSSQASSLEETAAALEEITSSMQNVSHKTSEVIAQSEEIKNVTSIIGDIADQINLLALNAAIEAARAGEH

GRGFAVVADEVRNLAERTQKSLGEIEANTNILVQSINEMGESIKEQTTGITQINDAVAQIDHVTQENLKIANDSAIVADN

VNKIASDILEDARKKKF

**>*C. lari*_LMG11760_Tlp108**

MFSSTKKYLLGNFSNKIAFLVCIFVIVLLCVLGVFNYIKSKSNSHTLLVQFQQKVAFDVSKRFDLYASDRRNIINSLTKY

IKENKHNLNSKQYTSLLKSIGDSLGFDLTYVGFEDGSMFRSNGNNQTPESGYDPRTRGWYKEAKEKKELIVTEPYISSSM

KKPTISYANPIIENGEVIGVVAADYDLKKFSEEVLAIGKTPYSHAAVLAHDGTYLFHTDPSKILTSTNTSKDIVAYYLKT

PEGVNRSLSKDIFKIQTKENETRALICNGGINPKYVICSIADYDFYNDAAKQTLMEQIIISLIAIFITLIFIRMIISYNL

KPIAIISSGLHNFFNYLNHKDSHSYPIKLKTQDEFGKMAEEINENIEIIKEALNKDAKAIEESVNIAKKIEAGELDLHIS

SHANNPQIQELMKVLNNMLLTLQRKIGSNLNEILAVFDSYKHLDFTATINAPKGDIEKAINSLGDEIKNMLTQSLHQGEL

LKQKAEALKQSMQELTNDATHQTSSLQESARALEQMNSAMSEISIKTQDVVKQSNDIKNVTTVISDIADQINLLALNAAI

EAARAGEHGRGFAVVADEVRNLAERTQKSLGEIEANTNILVQSINDMGEAIKEEADDISQINESVATIEKLTQQNSQTAMQTNAIANEVDSLAQDMLSETKKRKF (no concensus methylation site?)

**>*C. lari*_RM2100_Tlp109**

MFNFRSLSSKLTFIVGLLIIAILITVNIISYYQSKNSTSQYLEEIQVKTMFDVNKAYEIYGTSKRTAIDSIVKFMEKNPH

PDINELFDILETIRYSAGYDVTYIGFEEDGKLYQSNKIIRSPEQTGFDARTRPWYQEAKTTGTLVVSDPYKSIEDGSITI

SYTAPIYVNGKLLAVVGGDYNLHTFAKDVLILGHSQSSYAAVYDKEGQIIFHENKDLMLTKNDLSINIANAAKANPDLID

PSKEDSLFYAKDGNNKIQVVTCVQALNPKYMVCSITDESVYSDAVNEVLFQQVIIAFIAIIIALILVRFAIIKNLKPIAV

ITAGLNSFFDFINHKTKDSAMINVNTNDELGAMAKAINENITKTKNALEQDAKAVEQSVETAKEIEAGNLTARITAIPAN

PQLIELKNVLNDMLSVLEEKVGSNMNEINRVFDSYKALDFTTEVANAKGGVEITTNVLGQEIVAMLRQSSEFANLLATQSGKLQSAVRELTDSSSSQASSLEETAAALEEITSSMQNVSHKTSEVIAQSEEIKNVTSIIGDIADQINLLALNAAIEAARA

GEHGRGFAVVADEVRNLAERTQKSLGEIEANTNILVQSINEMGESIKEQTTGITQINDAVAQIDHVTQENLKIANDSAAI

SENVNKIANDILEDAKKKRF

**>*C. lari*_RM2100_Tlp110**

MKINSIVSKVNILVGILFAATIVIIGSIAYFQTKQSSFEYLRENHNKVLFDVGYIFNTYEADNQSAIQNLANFAVENHIL

DNEQEIFNALKLTQEYVGFEIVFLATEDGITYDSTGVKKTLNNGFDGRSRSWYIGAKKNMGLYTSDPYKSVTSGIEGIAY

SAPLIINGKFKGVVAGVYSLEQYSADALEVGKTENSFVAVYSQDGTTMFHQDPKLILTKTVLGQNIAKAITEDPSLLDPE

NIDTLFYAKDDKGVTQAVLCDKTPNPNINICAMVENDTYTKASDLALKTQLIVGFIALIIVLVLIKFFASYLLNPIFIIQ

TGLNSFFDFINHKTKDSAMINVNTNDEFGVIAKAINENITKTKNALEQDAKAVEQSVETAKEIEAGNLTARITAIPANPQ

LIELKNVLNDMLSVLEEKVGSNMNEINRVFDSYKALDFTTEVANAKGGVEITTNVLGQEIVAMLRQSSEFANLLATQSGKLQSAVRELTDSSSSQASSLEETAAALEEITSSMQNVSHKTSEVIAQSEEIKNVTSIIGDIADQINLLALNAAIEAARAGE

HGRGFAVVADEVRNLAERTQKSLGEIEANTNILVQSINEMGESIKEQTTGITQINDAVAQIDHVTQENLKIANDSAAISE

NVNKIANDILEDAKKKRF

**>*C. lari*_RM2100_Tlp111**

MGIKLKISLIANIIAIVCLISLGITTFYFVKDALLKNTIEAQTNYLKSSKDLMNDFKTSTERSLQNLSRAILKHPLYKLK

DEESVLASLAVELKAFRDSGGFLGVYVGMPSGELITSDPRADEKQLNAFIFGRAQNYNATTRGWYRGAKEKNGMYVSDVYVDAATNLPCLTYALPLYKDGQFIGVAGIDVLVEELQKKIERIPGDVFIANDSNYAFVSSSKVYLGKVKNVETALGKYKEF

GDFKPFMFTGQNGNDRLGICSKLDKYSACIVTKMNLIEESSEKIAYTQAIIVIFTSIISVILLYFIISRYLSPLEKIQTG

LNSFFDFINHKTKDSAMIDVKTNDELGAMAKAINENITKTKNALEQDAKAVEQSVETAKEIEAGNLTARITAIPANPQLI

ELKNVLNDMLSVLEEKVGSNMNEINRVFDSYKALDFTTEVANAKGGVEITTNVLGQEIVAMLRQSSEFANLLATQSGKLQSAVRELTDSSSSQASSLEETAAALEEITSSMQNVSHKTSEVIAQSEEIKNVTSIIGDIADQINLLALNAAIEAARAGEHG

RGFAVVADEVRNLAERTQKSLGEIEANTNILVQSINEMGESIKEQTTGITQINDAVAQIDHVTQENLKIANDSAAISENV

NKIANDILEDAKKKRF

**>*C. lari*_RM2100_Tlp112**

MFFKNSFISVKNKLSYTTGIIVALALFIVAAMAFYSSRENLIINSKNANKDYLLVTTAQVETYIESYVEILLSIKKYIDS

LPKYQTENFDKLGEYFAKDLKVFKDGSNTLAVYLGFPDGTMLVSDAESDKKGIPFRKRGGGISHYDDPQYNATTRDWYKGALKNKGVFVSDVYEDSVTKFPSFTYSVPIEKNGKLVAVLGIDLLLTSLQKTFEKLPGSVFVFDNTSSIPFASNDKSLILK

SYPNIDEIKNHHKIVGDYKTFEYTGVDSGEKRFGVCANINNSNAHVSYVACAIQKQDDLDRLVIEDVFDQIITSIVILIL

SCFIVYFISSRLLSPLQVIQTGLNSFFDFINHKTKDSAMINVNTNDEFGAMAKAINENITKTKNALEQDAKAVEQSVETA

KEIEHGNLTARITAIPANPQLIELKNVLNNMLSVLEEKVGSNMNEINRVFDSYKALDFTTEVANAKGEVEITTNVLGQEI

VNMLRQSSEFANLLATQSGKLQSAVRELTDSSSSQASSLEETAAALEEITSSMQNVSSKTSEVIAQSEEIKNVTSIIGDI

ADQINLLALNAAIEAARAGEHGRGFAVVADEVRNLAERTQKSLGEIEANTNILVQSINEMGESIKEQTTGITQINDAVAQ

IDHVTQENLKIAKDSAAISENVNQIANDILEDAKKKRF

**>*C. lari*_RM2100_TLp113**

MKSIKLKVAMIANIMAVICLLILGIVTFIFVKQSLFDEIVNSEKNRLISTNNLVENFRESTSNSLLKLSETILRNPYSNL

NSQEALAQNVGVQLKAFRDAGNYLAVYIAQPDGELVVSDPDSDSKNIDYGFYGKADGYDARTREFYIEARKKNGLFITASYIDATTGLPCFTYAMPLNKDGKFVGILAIDVLVKDLIENLKQMPGDSFVYDKNRYAFASTHKNYTGNHPNISTIADAFSK

TKNNEPFFYTSAEGNERLALCNNSNDYTVCNVAYVDTINNSSEKIAYIQAIIVIFTSILSVVLLYFIVSRYLSPLEKIQT

GLNSFFDFINHKTKDSAMINVNTNDELGAMAKAINENITKTKNALEQDAKAVEQSVDTAKEIESGNLTARITAIPANPQL

IELKNVLNEMLSVLEEKVGSNMNEINRVLDSYKALDFTTEVTNAKGGVEITTNVLGQEIVAMLRQSSEFANLLATQSGKL

QSAVRELTDSSSSQASSLEETAAALEEITSSMQNVSHKTSEVIAQSEEIKNVTSIIGDIADQINLLALNAAIEAARAGEH

GRGFAVVADEVRNLAERTQKSLGEIEANTNILVQSINEMGESIKEQTTGITQINDAVAQIDHVTQENLKIANDSAIVADN

VNKIASDILEDAKKKKF

**>*C. lari*_RM1607_Tlp114**

MFKFNSLSNKLTSIVCFLIVIILAIVNILNYYDSKKSTSYYLEEIQKKTMFDVNYMYSSYSNSKRNIIESLAYSLSAIAY

NSSDREIFSVLDTAKRSGGFDTVHFGLEDSGKDYQIDTKFNLHSDPSKFDPRTRPWYKDAKTAGKLIVTDPYKSIVLNGQ

VVVTYSIPVFDNSKKFIGVVSGVYNLNTFSKDVLAIGHSESSYAGVYDKEGVIVFHEDKDRMLTKNDLSINIANAVKANP

DLIDPTKQETLFYAKDDQGKTQVVTCNQALNPKYMVCSITDESVYTDAVNKVLFQQIIIALIAIAVALLLVRFAIIKNLK

PIAVITTGLNSFFDFINHKTKDSAMIDVKTNDELGAMAKAINENITKTKNALEQDAKAVEQSVDTAKEIEGGNLTARITA

IPANPQLVELKNVLNEMLNVLEQKVGSNMNEINRVFDSYKALDFTTEVKNAKGGVEVTTNVLGQEIVAMLRQSSEFASLLADESGKLQSAVKNLTDSSSSQASSLEETAAALEEITSSMQNVSHKTSEVIAQSEEIKNVTSIIGDIADQINLLALNAAIE

AARAGEHGRGFAVVADEVRNLAERTQKSLGEIEANTNILVQSINEMGESIKEQTTGITQINDAVAQIDHVTQENLKIAKDSAIVADNVNKIANDILEDARKKKF

**>*C. lari*_NCTC11845_Tlp115**

LFSNLKIGTKIVTVVITIIVLGIGILASIITMQSSNILHTEADKLLQTSAFRYSNIIRGATESVHSTLLSTESSIDQILD

TQTSLEQNRIQDILEGAVDSNSWINYIYIHIIDISKFNNIDPTLLTDSGQFLMLINDTDLKNKGGIKLIQADDRILNQRS

VKAALEKQEEGVGRPQNFVINNEEILAYNIAIPITRNGKLLGVIGALGGLNTLQEELTNPERSVFKNDQRLLLGANGLIA

VSPATDFIGKNITEINPHASAKTLIELQKNQINTLFDFTPASTGNNNRAAIANFNLWDGANDYWSIVTMAPVESIQMPIT

KLAATIAMVSLFVIFAIALIVFFYINKAVSSRIVNLQNNLLYFFKFLNHEVKDTILSKDIKNNDELNTMAKAINENITKT

KNALEQDAKAVEQSVDTAKEIENGNLTARITAIPANPQLIELKNVLNDMLSVLEQKVGSNMNEINRVFDSYKALDFTTEVKNAKGGVEVTTNVLGQEIVAMLRQSSEFASLLADESGKLQSAVKDLTDSSSSQASSLEETAAALEEITSSMQNVSHKTSEVIAQSEEIKNVTSIIGDIADQINLLALNAAIEAARAGEHGRGFAVVADEVRNLAERTQKSLGEIEANTNILVQSINEMGESIKEQTTGITQINDAVAQIDHVTQENLKIAKDSATISDNVNKIANDILEDARKKKF

**>*C. lari*_NCTC11845_Tlp116**

MKSIKLKVSLIANTIAIFCLIILGVITFIFVKQALFDEIVKSEQNRLVSTKSLMEEFRENTTTTLKKLSETILRHPYSEL

NTQESLIQNVSSQLRAFRDAGGFLTVYLAQPDGEVILTNAESDKLNQDIIIFGKKQNFDARTREWYQEAKAKGIFVTPAY

IDTTTNLPCFTYAMSLSKDGKFIGVLAIDVLVKDLEDGLKQMPGASFVFDKNNFAFASTSKNYIANDPNVSIVAEAFSKT

KDGEPFYYTSKEGSERLAMCDRVNGYTICNMTYIDTIDQSSEKIAYIQAIIVIFTSIISVILLYFLVSHYLSPLQAIQTG

LNSFFDFINHKTKDSAMINVKTNDEFGAMAKAINENITKTKNALEQDAKAVEQSVETVREVESGNLTARITAIPANPQLL

ELKNYLNEMLSVLEQKVGSNMNEINRVFDSYKALDFTTEVKNAKGGVEVTTNVLGQEIVAMLRQSSEFASLLADESGKLQSAVKDLTDSSSSQASSLEETAAALEEITSSMQNVSHKTSEVIAQSEEIKNVTSIIGDIADQINLLALNAAIEAARAGEHG

RGFAVVADEVRNLAERTQKSLGEIEANTNILVQSINEMGESIKEQTTGITQINDAVAQIDHVTQENLKIAKDSATISDNV

NKIANDILEDARKKKF

**>*C. lari*_NCTC11845_Tlp117**

MKSIKLKLSLIANIMAIFCLIVLGVISFIFTKKALNYEVIKAETNYVRAAEKSMRDFKNTNINSLERLSQAIARFSYEEL

DTQEKLMHNTGKLLKSFRDAGNYLAVYIAQPNGELIVSDPDSDSKGLEYGTYGKADNYDATTREFYIEAKKKNGLYITAA

YIDATTGLPCFTYAMPLIKDGKFIGVLAIDVLVKDLQTEFSELPGRTFVFDHEFNVFAATDETLVSKEKNPDIITVAKAY

EQAGDYNIFNYTTQKGKDRFGICVKIDSYTTCAGEDIEVIETPALKIAYIQTSIVVFTSVASIILLYFIISYYLSPLQAI

QTGLNSFFDFINHKTKDSAMINVKTNDELGAMAKAINENITKTKNALEQDAKAVEQSVETVREVESGNLTARITAIPANPQLLELKNYLNEMLSVLEQKVGSNMNEINRVFDSYKALDFTTEVKNAKGGVEVTANVLGQEIVAMLRQSSEFASLLADESGKLQSAVKDLTDSSSSQASSLEETAAALEEITSSMQNVSHKTSEVIAQSEEIKNVTSIIGDIADQINLLALNAAIEAARAG

EHGRGFAVVADEVRNLAERTQKSLGEIEANTNILVQSINEMGESIKEQTTGITQINDAVAQIDHVTQENLKIAKDSATIS

DNVNKIANDILEDARKKKF

**>*C. lari*_NCTC11845_Tlp118**

MKSLANKLTFFVFLAIIAILFVANIFNYIEVKRDVQKLINDIQIKTMQDVLKSFDDYTASRSDAIKAVAAEIKKNPNTSL

EEIYTMVKVAKEASRFDVLYVGLAKNGAMIRSNGNHQMPSDGYDPRTRTWYTSVASGEDKVVISKPYMAPSLKAPSLAFSYPIIIDGKFMGAVGGNYDLNTFSDNVLAMGKSQSGYTVVLDDEGTILFHESSKDLLTKTNLSQNIVKTYLATPDGKDGKLSSEPMLIDDDNAPRKAVICQESSIGYNVCVIADEKIYNEPVNKALVNQIIIGIISLIIALIIVRFMISYNLSPLQAIQTG

LNSFFDFINHKTKDSAMINVKTNDELGAMAKAINENITKTKNALEQDAKAVEQSVETVREVESGNLTARITAIPANPQLL

ELKNYLNEMLSVLEQKVGSNMNEINRVFDSYKALDFTTEVKNAKGGVEVTTNVLGQEIVAMLRQSSEFASLLADESGKLQSAVKDLTDSSSSQASSLEETAAALEEITSSMQNVSHKTSEVIAQSEEIKNVTSIIGDIADQINLLALNAAIEAARAGEHG

RGFAVVADEVRNLAERTQKSLGEIEANTNILVQSINEMGESIKEQTTGITQINDAVAQIDHVTQENLKIAKDSATISDNV

NKIANDILEDARKKKF

**>*C. lari*_NCTC11845_Tlp119**

MFKFNSLSNKLTMIVCFLIAIILIVVNIINYYESKKTTAYYLEEIQKKTMFDVNEAYKIYSSSKRSAILSIVNFIEKNPN

PSTEELFDILETIRSSADFDVTYVGFEKDGKLYQSNKIIRSPEASGFDARTRPWYQESQQAKTLTVSDPYKSIEDDSITI

SYTAPIYNNGKLIAVVGGDYNLEKFAKDVLVIGHSNSSYAAVYDKEGVIIFHEEKDRMLTKNDLSINIANAVKANPDLIN

PNKQETLFYAKDQQGKTQVVTCNQSLNDKYIVCSITDESIYTDAVNKVLFQQIIIALIAIAIALILIRFTIMKNLKPIAV

ITTGLNSFFDFINHKTKDSAMINVKTNDELGAMAKAINENITKTKNALEQDAKAVEQSVDTAKEIENGNLTARITAIPAN

PQLIELKNVLNDMLSVLEQKVGSNMNEINRVFDSYKALDFTTEVKNAKGGVEVTTNVLGQEIVAMLRQSSEFASLLADESGKLQSAVKDLTDSSSSQASSLEETAAALEEITSSMQNVSHKTSEVIAQSEEIKNVTSIIGDIADQINLLALNAAIEAARA

GEHGRGFAVVADEVRNLAERTQKSLGEIEANTNILVQSINEMGESIKEQTTGITQINDAVAQIDHVTQENLKIAKDSATI

SDNVNKIANDILEDARKKKF

**>*C. lari*_CCUG22395_Tlp120**

MFNTLKVKLSLMANFFTALSLIILGVLSFYFTKTYLYDNELKRQNDILQVARTSLETFRKHNSDLILNLEKTILEFPYEK

LNSEQALIDNVGSILKSYRKASGVLSAFISLDNGENLVSNDTSDKNNKNIEIYGQNINYDARTRPWYIGAKENKDIFITS

PYIDKATNQYVITYTKSIYKNGNFVGIIGVDIPVKELQENFENMPGNSFLFDENGKIFVAKNKQLLDSSVDHGPVLNAHK

QNGDYSFFEYGLKGKERLGICAKISSYLVCSTESADVINEPIFKTATIQTIVVSIMVALSVIILYFIISYYLSPLQAIQK

GINSFFDFINHKTKDSAMIDVKTNDEFGVIAKAINENITKTKNALEQDAKAVEQSVDTAKEIESGNLTARITAIPANPQL

IELKNVLNEMLNVLEQKVGSNMNEINRVFDSYKALDFTTEVKNAKGGVEVTTNVLGQEIVAMLRQSSEFASLLADESLQSAVKNLTDSSSSQASSLEETAAALEEITSSMQNVSHKTSEVIAQSEEIKNVTSIIGDIADQINLLALNAAIEAARAGEHGK

GRGFAVVADEVRNLAERTQKSLGEIEANTNILVQSINEMGESIKEQTTGITQINDAVAQIDHVTQENLKIANDSAIVADN

VNKIASDILEDARKKKF

**>*C. lari*_CCUG22395_Tlp121**

MSVKNKLSIVVGVIVFLALSIITIMAFVSSRNNLISNSKQANEDYLLITETQVEGYVDGYIDILLAIKKYIDSLGKNQVL

NFDSMEQMLGDDLKIFKDGSNTLAVYVGFPDGTMLVSDTVSDKKGVNFRKRGGGISSYDDPSYDATSRDWYKGAIANDGVFISDVYEDSVTKLPSFTYSTPIKKNGKLIGVLGVDLLLTSLQKTFEKLPGNVFVFDTASSIPFASNNKSLILTQHPSIEE

IKKYHQQFGDYKTFEYISVDTQEERFGICANIDNSKAKVSYIACATQKQDELEMLVLKDAYKQIIFSIIILFISCFAIYF

FSSKLLSPLQAIQKGINSFFDFINHKTKDSAMIDVKTNDELGAMAKAINENIIKTKNALEQDAKAVEQSVDTAKEIESGN

LTARITAIPANPQLIELKNVLNEMLNVLEQKVGSNMNEINRVFDSYKALDFTTEVKNAKGEVEVTTNVLGQEIVAMLRQSSEFASLLADESGKLQSAVKNLTDSSSSQASSLEETAAALEEITSSMQNVSHKTSEVIAQSEEIKNVTSIIGDIADQINLL

ALNAAIEAARAGEHGRGFAVVADEVRNLAERTQKSLGEIEANTNILVQSINEMGESIKEQTTGITQINDAVAQIDHVTQE

NLKIAKDSAAISDNVNKIANDILEDARKKKF

**>*C. lari*_CCUG 22395_Tlp122**

MKSIKLKISLIANIIAIVCLIILSIVSFIFTKKALNYEVVKAETNYVRTAEKSMRDFKNLNTHSLEKLSQAILKLPYDAL

NTQDKLMQNTGNLLKAVRDMNSYLAVYIAQPNGELIVSDPDSDSKGLDYGIYGKADNYDATTREFYIEAKKKNGLYITPSYIDVTTGFPCFTYAMPLIKDGKFLGILAIDVLVKDLQNEFSELPGRTFVFDQAYTVFASTDKSLIGGEKNPDIVTVAKAY

EKAGNYNIFNYTTQNGQDRFGICVKIDDYTTCAGENIEVIEAPALKIAYIQTTIVIFTSIASIILLYFIISYYLSPLQAI

QTGLNSFFDFINHKTKDSAMIDVKTNDEFGVIAKAINENITKTKNALEQDAKAVEQSVDTAKEIESGNLTARITAIPANP

QLIELKNVLNEMLNVLEQKVGSNMNEINRVFDSYKALDFTTEVKNAKGGVEVTANVLGQEIVAMLRQSSEFASLLADESGKLQSAVKNLTDSSSSQASSLEETAAALEEITSSMQNVSHKTSEVIAQSEEIKNVTSIIGDIADQINLLALNAAIEAARAG

EHGRGFAVVADEVRNLAERTQKSLGEIEANTNILVQSINEMGESIKEQTTGITQINDAVAQIDHVTQENLKIANDSAIVA

DNVNQIANDILEDARKKKF

**>*C. lari*_RM2100_Tlp123**

MFSSLKIKLSLLANIFAALSLIVLGIISFIFTKNFLYENELKRQNDILQVSRISLETFRENNIKLINHLEESVLELPYEK

LNSQEDLIENLGQMLKSYRKASGVLATFIGLDNGENIVSDNSSDNKNTNVVIYGKAINYDTRTREWYIEARKTNKVFITF

PYIDKATNQYVITYTKSISKDGKFIGVIGVDIPIAIFKKTLKINQEIAFFNQNEKVFVAKNKQLLDPSVDHSPVINAHKQ

NGDYKFFEYGLKGQERLGICANIYDYRVCSTESAEIINKPIMQIALTQAIVVIIMIVLSIAVLYFIVSRYLSPLEKIQTG

LNSFFDFINHKTKDSAMINVNTNDELGAMAKAINENITKTKNALEQDAKAVEQSVETAKEIEAGNLTARITAIPANPQLI

ELKNVLNDMLSVLEEKVGSNMNEINRVFDSYKALDFTTEVANAKGGVEITTNVLGQEIVAMLRQSSEFANLLATQSGKLQSAVRELTDSSSSQASSLEETAAALEEITSSMQNVSHKTSEVIAQSEEIKNVTSIIGDIADQINLLALNAAIEAARAGEHG

RGFAVVADEVRNLAERTQKSLGEIEANTNILVQSINEMGESIKEQTTGITQINDAVAQIDHVTQENLKIANDSAAISENV

NKIANDILEDAKKKRF

**>*C. lanienae*_NCTC13004_Tlp200**

MTYFKFKFIFFFFYNNDRISQFLVYFPLPFKHLSEISLTLHKGKIMFNKLKLGTKTIASIVSIVVFCLAIMSWVIITITS

NLQSEEAKKLLINASDRDANLADSFLSQMYVALESNAASFERLIQDKRDEKELEHTLMGVLDSAGDAVYAYLYILDPFYT

QNSTNPNFKLNNSNLLILQTDDNTKAVGGVRTIKADDRFSALDGLNRAIRTGQSAVGNPSVINVGNSNLGILDINIPLKN

KNSQVIAVLGAVADLNTISKIMNHPDRQIFKDSQIFVINENGIVAVDKDNKYISKNLQTINKDVSVAQIIQAIKSKESGI

FEYYSVAGVKEMAALRTISIARDSATFGIIAAAPVSSIYEPVRKLIFYNIIGVLITAIIIALFIFYYINVGIVFRINNIS

NLLFGFFKFLNHESKTPPPYLQPKAEDEIGKMAIELNRNVQKIQEGLNQDNQAVKSAIQTANEVENGNLTARITLNPASP

ELLKLKNVLNKMLDVLEQKVGSDINAIQSVFDKFKQLDFTVRIDNPKGEVEIVTNLLGDEVTKMLKANLEQANNLQNKADELKGFVASLNEGAKSQSESLQESAAAVEEMSSSMNSINDRASEVIKQSEDIKNIITIIRDIADQTNLLALNAAIEAARAG

EHGRGFAVVADEVRKLAERTQKSLGEIEANVNILSQSINEMSQSISEQTTAINQINEAIVNVDGLTRQNRQIAQDSNIVA

NEVDSIAEVVVAEVKKKKF

**>*C. lanienae*_NCTC13004_Tlp201**

MFKNMKLSVKLALAGFVFTALIVATVAGIATYQATNRAESNSAAFMEAGSLSQANYIAAEFNKISDGLNAYKTAMESSLNSGIAMPVDIITNMLSEAIKDQKIAAGIFFYATDDSIYAKNPNSNDPRYTQAGDLAIYVNYHNRLERIEDNFKSKDYYANGLSKPRLTEPFSDHIEGKNITMASMTFPVKYRGKTIGIIGADLDLETYFIDRLKEIKLYKSDISFLISNKGIMVANPNPQN

RGLAIQQVNPNLTQLLPVLNNEEVDYATAAAMSQNLQEHAQFNLSKIDMHNVDENWGLATLVPTREIFAEVSSARNSLAVVGIIVAIIGGFFLFFATKMLITRIENIRALLSDFFDFLNYKRADARLAVITNNDEISQMAKAINDNIVIVKSNLDKDSKA

VEESLKRASEVENGNLTARITLNPASPELLKLKNVLNKMLDVLEQKVGSDINAIQSVFDKFKQLDFTVRIDNPKGEVEIV

TNLLGDEVTKMLKANLEQANNLQNKADELKGFVASLNEGAKSQSESLQESAAAVEEMSSSMNSINDRASEVIKQSEDIKNIITIIRDIADQTNLLALNAAIEAARAGEHGRGFAVVADEVRKLAERTQKSLGEIEANVNILSQSINEMSQSISE.TTAIN

QINEAIVNVDGLTRQNRQIAQDSNIVANEVDSIAEVVVAEVKKKKF

**>*C. helveticus*_ATCC51209_Tlp300**

MKSIKIKLALIANLMAVLCLVILGVVTFMFVKNEIYTEVVNAETNYVKTAKNSMESFRARNIAALKSLEKNILKHSLSRL

DNQEALMQFVGQDLKTFRDAGRFLAVYIAQPSGELLVSDLDSDSKNLDFGIYGKADNYDARTRDYYKEALKTNEIHITPS

YIDVTTGLPCFTYAVALHKDGKFIGVLAVDVLVTDLQKEFENLPGRTFVFDSENQVFASTDKEMLNSNNDISVIANVAKE

RADYEHFEYIKPEDKSERFGICARTMGNYTVCVGENISKIESPVYKIAFIQITIVIFTSIASVILLYFIISYFLSPLSFI

QSGLNSFFDFINHKTQDISTININTSDEFGQMAKAINENILATKQGLDQDKQAVKESVETVGIVENGNLTARITANPRNP

QLIELKNVLNDLLDVLQTRVGKDMNKIRSIFEEFKSLDFRNRIEDATGSVEVTTNALGEEIIKMLKQSSDFANSLANESS

KLQNAVQNLTTSSNSQAASLEETAAALEEITSSMQNVSQKTSDVITQSEEIKNVTSIIGDIADQINLLALNAAIEAARAG

EHGRGFAVVADEVRKLAERTQKSLSEIEANTNLLVQSINDMAESIKEQTAGITQINESVAQIDQTTKDNVEIANESAIIS

NNVSDIANNILEDVKKKKF

**>*C. helveticus*_ATCC51209_Tlp301**

MFKSLNIGLKLILSVAITVIIGVIAFISLTSVQVSSNMKEQMDKVLMQASKRYANYIEAKLNETIALVKSASRTINEDIR

KNGFDFNDVENVIKNTFDSSSEATFAFFLLEDSAILGDTNVKKQYRSEKGTFGMEFVDTAVEKSGGIETLQFSEKIKSFP

VVQRIRNEAKTADYDKVYVGVPAKLDMGRGEFIGVNIAMPVFDTNGKYIGCVGFVFDLQSFSTTLMDPILDLYDGNIRVLLSEDSTVAVHSTNLSLLLKNLAEISSSPQTKEIVNAVKENKDFLFENYTSSSGEESFVAIASFTTLDNSSKWSILTTAPK

ASVLAPLYKMQFIFVAIGIIFLIAVIAVVYYCVRVIIGARLPILVKSLETLFRFLNHEKVEPHIIKINADDELGAIGKML

NENILATKQGLDQDKQAVKESVETVGIVENGNLTARITANPRNPQLIELKNVLNNILDVLQARVGKDMNKIHSIFEEFKR

LDFRNRIEDATGSVEVTTNTLGEEIIKMLKQSSDFANSLANESSKLQNAVQNLTTSSNSQAASLEETAAALEEITSSMQN

VSQKTSDVITQSEEIKNVTSIIGDIADQINLLALNAAIEAARAGEHGRGFAVVADEVRKLAERTQKSLSEIEANTNLLVQ

SINDMAESIKEQTAGITQINESVAQIDQTTKDNVEIANESAIISNNVSDIANNILEDVKKKKF

**>*C. helveticus*_ATCC51209_Tlp302**

MNLTIRGKMLLLGSAVFAIILAILLNFYINQHNGLKYVEKVAGQIVEKGVNEKIKVLTLSMAESLGGLLEGVTDEKQQIA

IIAKAIENFRFEDDKSGYFFVYKKTVNVAHPVRKDLIGSDLKEAKDAKGVQYVVELYNQAKNGGGFVFFDFTKGGENSTV

AQKNAYAALIPNTEDIWISTGVYVDTLAKNVAEDTRGVKETFNSSFIRALIISAVLLILICPFVILFYKKLSASITTISQ

DLFHFFDFINHKTNEIKVVEVSGNDELAQMAKAINENILATKQGLDQDKQAVKESVETVGIVENGNLTARITANPRNPQLIELKNVLNDLLDVLQTRVGKDMNKIRSIFEEFKSLDFRNRIEDATGSVEVTTNALGEEIIKMLKQSSDFANSLANESSKL

QNAVQNLTTSSNSQAASLEETAAALEEITSSMQNVSQKTSDVITQSEEIKNVTSIIGDIADQINLLALNAAIEAARAGEH

GRGFAVVADEVRKLAERTQKSLSEIEANTNLLVQSINDMAESIKEQTAGITQINESVAQIDQTTKDNVEIANESAIISNN

VSDIANNILEDVKKKKF

**>*C. helveticus*_ATCC51209_Tlp303**

MTKTLGGKILACVVAIFVVVIGVIVTYNYISSSSQISTLFRSIQRGILDASYTTIDITMNVEAKQHLNAVAEQIVALDKN

DVIAQRRVLMTAEELIKYPSMYIVYENDGKVILQDYHPEVGIENLSSNFDNVGLDLRDRFWYKETKEKKQGIVSSTYISS

AGNYKGQRLVTATYPLIKNGEFIGVIGMDLFVGDFQKRFENFEREELPNLDVYITDTSGKIFSHKDPAIVESTTETEAEK

ALKEALKKAPEGEFNYNHNNEDRVGFYKQFPFGWTIVSVTTQSDYTNAINKQFFISTAIALVLLVVGAMFLVVFVKKLVA

PIHSIQSGLNSFFDFINHKTQDISTINVNTSDEFGQMAKAINENILATKQGLDQDKQAVKESVETVGIVENGNLTARITA

NPRNPQLIELKNVLNDLLDVLQARVGKDMNKIRSIFEEFKSLDFRNRIEDATGSVEVTTNALGEEIIKMLKQSSDFANSL

ANESSKLQNAVQNLTTSSNSQAASLEETAAALEEITSSMQNVSQKTSDVITQSEEIKNVTSIIGDIADQINLLALNAAIE

AARAGEHGRGFAVVADEVRKLAERTQKSLSEIEANTNLLVQSINDMAESIKEQTAGITQINESVAQIDQTTKDNVEIANE

SAIISNNVSDIANNILEDVKKKKF

**>*C. helveticus*_ATCC51209_Tlp304**

MVKSEKYGSVSSKLTLYVGILIVLILSTASAVAYFGSKENNFRLLKESQFKLMDDTLKTFNIYTGFKRNAMTVLSSQIGH

LDHLDEDEIYDLLEMTLKTAEFGEVFFASEQNAKTYLSNRTSLSLTQLDFKTRPWYEKTKQEGKLIATEPYKNATDGKTV

ITYTVPVIHNGTFVGIVGGDLNLAAISDQILMMGHTAESYSQVISPNGDILFHEEEEKILSKTTLSENIANAIKANPHLL

DDDNDDTLFYVEGNDGKAQAVMCDLTLNPYFRICTITAESSYSNASNKILLQQVIVGLVAIVVALILVRVLIARSLFPLG

SIQSGLNSFFDFINHKTQDISTININTSDEFGQMAKAINENILATKQGLDQDKQAVKESVETVGIVENGNLTARITANPR

NPQLIELKNVLNDLLDVLQARVGKDMNKIRSIFEEFKSLDFRNRIEDATGSVEVTTNALGEEIIKMLKQSSDFANSLANE

SSKLQNAVQNLTTSSNSQAASLEETAAALEEITSSMQNVSQKTSDVITQSEEIKNVTSIIGDIADQINLLALNAAIEAAR

AGEHGRGFAVVADEVRKLAERTQKSLSEIEANTNLLVQSINDMAESIKEQTAGITQINESVAQIDQTTKDNVEIANESAI

ISNNVSDIANNILEDVKKKKF

**>*C. fetus* subsp. *venerealis*_cfvi03/293_Tlp400**

MKSINTKVIAIIAILMAMLIVLFVTLEIFISKVNLSFKEINSISDRQELLYKNIINGERTGLTVRQLYIDINDKDALDIL

EATMKDFEVVRNKYKELSGGPANAANQSDKLLFIQNDILQGAKKGEKVTITDLEDLTPTWRSYRSVLEKRLEKLGEDNLK

ANNNLASDISVLTMGFTVFIITIIILSSLILLISKSYLLKAIKSIENGLKDFFDFLNHKNDNPKAISLKSNDEFGVMAKL

INDNTSNIKDSMEQDNKAVKESLEKANEVENGNLKARINTIPSSPGLEKLRQVLNKMLDTLERKIGSDINVIQQTFDSFK

ELDFTSRIPNAKGEVERVTNLLGDEITKMLKDNLAQANNLKEKANSLKEYVTTLNDSARSQANSLQESAAAVEEMSSSMSSINERAGEVIKQSEDIKNIITIIRDIADQTNLLALNAAIEAARAGEHGRGFAVVADEVRQLAERTGKSLAEIEANVNILS

QGINEMSQSINEQTEAINQINEAVANVDEQTKQNLAIASNTDRVTIEVETIANEVVSEVKRKKF

**>*C. fetus* subsp. *fetus*_04/554_Tlp401**

MLFGSKSIAGKVSFGVSILFIILLLVLAFINYSDSKDNSTKLLVSERQKVIQASENLMDSRFGSDINTINNLNDYISKND

YSQQEIEDILKIIDKTSNFELLYVGYQKDGMMIRSNGDSGLPSTENGMYDPRNRPWYINAVKENRVVITEPFISKTTGQF

TISISKPIYKNSELIGVISGQVALNEVNKKFAELGNAEGAYVLLMDKDAKIIMHPTIDYIGKTLSATKEILKNYQNKNFD

KYGRLTYTHEDGSLKIGQCATTKFDGWIICSNVDAKYFQSKVDAILYKQLLISFIFIIIAAFAVWILAKRLLQPTQSIEN

GLKDFFDFLNHKNDNPKAISLKSNDEFGVMAKLINDNTSNIKDSMEQDNKAVKESLEKANEVENGNLKARINTIPSSPGLEKLRQVLNKMLDTLERKIGSDINVIQQTFDSFKELDFTSRIPNAKGEVERVTNLLGDEIAKMLKDNLAQANNLKEKANSLKGYVENLNESASSQANSLQESAAAVEEMSSSMSSINERAGDVIKQSEDIKSIITIIRDIADQTNLLALNAAIEAARAGEH

GRGFAVVADEVRQLAERTGKSLAEIEANVNILSQGINEMSQSINEQTEAINQINEAVATVDEQTKQNVTIAQNSNKITNEVESIANIIVDEVKKKNGDVIKQ

**>*C. fetus* subsp. *venerealis*_cfvi03/293_Tlp402**

MKSINSKVMAIIAIIMAMLIACFVVVEIFISKVNASFNESEETKNEYVLIYKNIIDGERAGLNIRNLYIIPEDKNTLTIL

ENSVNDLVTNREEYKKLLGTTKLQTDEIFSKLTSFYRSSINKAKNNQNITIEDVQEITPIWREYRDLLEKQLASLVIRDK

STSDSFANDVNVLTMGFTVFIITIIILSSLILLISKSYLLKAIKSIENGLKDFFDFLNHKNDNPKAISLKSNDEFGVMAK

LINDNTSNIKDSMEQDNKAVKESLEKANEVENGNLKARINTIPSSPGLEKLRQVLNKMLDTLERKIGSDINVIQQTFDSF

KELDFTSRIPNAKGEVERVTNLLGDEITKMLKDNLAQANNLKEKANSLKEYVTTLNDSARSQANSLQESAAAVEEMSSSMSSINERAGEVIKQSEDIKNIITIIRDIADQTNLLALNAAIEAARAGEHGRGFAVVADEVRQLAERTGKSLAEIEANVNIL

SQGINEMSQSINEQTEAINQINEAVATVDEQTKQNVTIAQNSNKITNEVESIANEVFNEVNKKKFKNF

**>*C. fetus* subsp. *venerealis*_cfvi03/293_Tlp403**

MLFFNKSIASKISIGVSLLFAIALISFTYINYIDSRDNSIKLLSLERQKAVAGSENILDIELEFDANKAEQISKQISKPN

LTREEVQNILKTTNKITEFSSIHMVYESDGTIYKSDGTIKEANSIYDARKYPWYQEALKKNKLIITDPYQNLDNNTYITL

AAPIHVENKFIGVLGINLEAENLSKKIIKLGETKGGYIMILDSEGKIIMHQEKNQIGKSPNSTLSLVNNFQNKNIDKYGM

ISYTRSNGNQNIADCMNSKHSDWIICASMDKNIFEEGINNILKKQLWLSALYIIISSFIVLILAKRYLKPINNIVFGLKN

FFDFLNYKNDNPKDISLKSNDEFGVMATMINDNISIIKNAIKKDAQLVSESLQKVKEVEDGNLKARIVKCSGFTAAKQTK

RCFKLYVRSAEQKVGSDINVIQKTFDDFKNLDFTSNIPNARGEVEKIINTLGKNITKMLKDNLKQADILQAKANNLQDFV

IMLNESSKSQASSLGESAAAIEEMSNSMTSINERTMEVIKQSEDIKSIITIIRDIADQTNLLALNAAIEAARAGDHGRGF

AVVADEVRKLAERTGKSLGEIEANVNILSQSINDMSQSIKEQTETMNQINQSVANVDELTKQNVDIVNDTNKISIEVENI

ANSIVNDAKKNKF

**>*C. fetus* subsp. *venerealis*_84-112_Tlp404**

MKNLKLGTKLVLIVGIIITIGIAILSYIVARQTSSNMTKNAEYIITNDALKYAATIEGMMNEIIATTQSAHDVINDFFHR

VPMNEIKLENIESILSNVFDSSLHANYAMLYLTNPPEQFKGINKYTTESGKFLILFRDEDTSKKGGIESMQASDTAINDS

ILKKALIEGNPNGNRVFVGNVEKISFGSNSFIGINVALPIFNNDSKKPIGVIAFSLNFKEISNFLLDNKLDSFSGYTKAI

IAKDGTIAVHDNSNIILKKIQDINPHAKALADAVAKNEFKIFSNYTTSTGVSSYAVVAPFTTARDSSNWAIVTTAPIDSV

FAPLYSLQKTIFVASLIFLVVSLAFIYFYIKANLAMRLPILLNALDSFFKFINHESKEVQMIKIDANDELGAMGNMINAN

IAKTRDSLIKDQEAVQQSVETAKEIEGGNLTARIVKDPANPQLIELKNVLNKMLLVLQNKVGSNMNEINRVFNSYKSLDF

TTNIANAKGEVEVTTNVLGDEIKEMLRSSLSFAKDLAEQSKELRESMQKLTDGSRTQAHSLEQSAAAVEQISCSMQSISD

RTVETTKQAEDIKNIVGVIKDIADQTNLLALNAAIEAARAGEHGRGFAVVADEVRKLAERTNNSLGEIEVNVNILVQSVN

DMSESIKEQTIGLGQINESIAQLESVTQTNVGIANTTNDITQNVNTIADNILADVNKKKF

**>*C. fetus* subsp. *fetus*_04/554_Tlp405**

MQQRGLATKVSIGVSILFMILLIVLTYINYSNSKVNTTQLLSSERAKSIQAGKMLLDTQFKKAIVGIENLSKLFSSNNYD

YKEVENILKNINSSMDFEAMYIAYQDNGMIVASDGNSGLPTDEYDPRKQNWYQEAANLRKTLITEPYIDNVTKKQIITAATPFYKDNKLLYVVGADFTIDGIQKEFNELGNAEGAYMLLMDKNAKLIMHPNSDFIGKTLNATKEILKNYKANNVDEYGRLPYTHEDGSKKLGRCVSFGINDWIICSNVDIDFFSKKTDDILYKNIIISITFIILATLIIYILVKRFLKPIQIIQNGLKDF

FDFLNHKNDNPKAISLKSNDEFGVMAKLINDNTSNIKDSMEQDNKAVKESLEKANEVENGNLKARINTIPSSPGLEKLRQVLNKMLDTLERKIGSDINVIQQTFDSFKELDFTSRIPNAKGEVERVTNLLGDEIAKMLKDNLAQANNLKEKANSLKGYVENLNESASSQANSLQESAAAVEEMSSSMSSINERAGDVIKQSEDIKSIITIIRDIADQTNLLALNAAIEAARAGEHGRGFA

VVADEVRQLAERTGKSLAEIEANVNILSQGINEMSQSINEQTEAINQINEAVATVDEQTKQNVTIAQNSNKITNEVESIA

NEVFNEVNKKKF

**>*C. fetus* subsp. *testudinum*_pet-3_Tlp406**

MLENKINGLISSIEHISNIIESNNQNYVNENIEFLLSTIAKTSNLKLIYMALDNNGMMYSTGDIKGLPTSNFDARDREWY

KNAKNTNKLIITEPYISAANNLVSISAVKPIYINGKFVGVLGADMDFEDIENTFLQIGNSKYGYNFLIDKNGMVLIHPNE

QLIGKSFNFTKEIIQKIQNKDFDQYGRIAYTSQDGFNRFGRCSISESSGLFICNGLDAKFFADQTNSIIKKQSAFGIISI

IISSLIVWFVIKLNFKPLNNIVSGLKDFFDFLNHKNDNPKAINLKSNDEFGVMASLINSNISSIKESLDKDAKAVEEALI

RASEVEKGNLGARIMHEPDSPGLKKLKDVLNSMLNTLQGKIGSDINVIQKTFDDFKNLDFTSNIPNAKGEVEKVTNLLGNEITKMLKDNLNQANNLKEKANNLKEYVTTLNDSARSQANSLQESAAAVEEMSSSMSSINERAGEVIKQSEDIKNIITIIR

DIADQTNLLALNAAIEAARAGDHGRGFAVVADEVRQLAERTQKSLGEIEANVNILSQSINEMSQSISEQTEAINQINEAV

ANVDEQTKQNLAIASNTDRVTLEVETIANEVVSEVKRKKFN

**>*C. fetus* subsp. *testudinum*_03-427_Tlp407**

MGGGALKNLKLGTKLVLIVGIIITIGIAILSYIVARQTTSNMTKNAEHIITNDAFKYAAKIEGMMNEIIATTQSAHAVID

MHSLKKATVGIENLVKLFSSNNYNYQEVENILKNINNSMDFEAMYIAYQDNGMIVASDGNSGLPTAQYDPRKQNWYQEAANLRKTLITEPYIDNVTKKQIITAATPFYKDNKLLYVVGADFTIDVLQKEFNELGNAEGAYMLLMDKNAKLIIHPNKDFVGKTLNATKEILKNYKANNIDQYGRLPYTHEDGSKKLGRCVPFGINDWIICSNVDINFFSKKTDDILYKNIIISITFIILAT

LIIYLLVKRFLNPIQIIQNGLKDFFDFLNHKNDNPKAINLKSNDEFGVMAKLINENTSIIKDSMEQDNKAVTESLEKANE

VENGNLKARINTIPSSPGLEKLRQVLNKMMDTLERKIGSDINVIQQTFDSFKELDFTSRIPNAKGEVEKVTNLLGDEIAK

MLKDNLAQANNLKEKANSLKGYVENLNDSARSQANSLQESAAAVEEMSSSMSSINERAGDVIKQSEDIKSIITIIRDIAD

QTNLLALNAAIEAARAGEHGRGFAVVADEVRQLAERTGKSLAEIEANVNILSQGINEMSQSINEQTEAINQINEAVATVDEQTKQNVAIAQNSNKITNEVESIANEVFNEVNKKKF

**>*C. fetus* subsp. *fetus*_04-554_Tlp408**

MNTQLGDDIDAIENLAKLIGANNYSNQEVETILKAIENSSRFDLIFVGYQNDGMIIRSNGNSSLPTNEYDPRKRAWYERA

IKENKTIVSDPYMSKTVQKLCVTVAAPIYSNNKLIGVVGADKAIDVLSKEFIEIGNAEGAYILLMDKNAKVIVSPASEYI

GKTLNFTKEIIQKIQNKDFDAYGRVSYTHDGSQKLGKCINSSINDWIICSNIDVEFFKKKTDTIFYKQIILSIIFVIFAS

LTILLLAKKLLKPMDKIVSGLKDFFDFLNHKNDNPKAISLKSNDEFGVMASLINSNISSIKESLDKDAKAVEEALVRASE

VEKGNLGARIMHEPDSPGLKKLKDVLNSMLNTLQGKIGSDINVIQKTFDDFKNLDFTSNIPNAKGEVEKVTNLLGNEITK

MLKDNLNQANNLKEKANSLKEYVTTLNDSARSQANSLQESAAAVEEMSSSMSSINERAGEVIKQSEDIKNIITIIRDIAD

QTNLLALNAAIEAARAGDHGRGFAVVADEVRQLAERTQKSLGEIEANVNILSQSINEMSQSISEQTEAINQINEAVANVDEQTKQNLAIASNTDRVTIEVETIANEVVSEVKRKKF

**>*C. fetus* subsp. *fetus*_04-554_Tlp409**

MKSINTKVIAIIAILMAMLIALFVTVEIFISKVNLSFKEINSISDRQELLYKNIINGERAGLTVRQLYIDINDKDALDIL

EATMKDFEVVRNKYRELSGGPANAANQSDKLLFIQNDILQGAKKGEKVTITDLEDLTPTWRSYRSVLEKRLEKLGEENLK

ANNNFASDISVLTIGFTVFIITIIILSSLILLISKSYLLKAIKSIESGLKDFFDFLNHKNDNPKAISLKSNDEFGVMASL

INSNISSIKESLDKDAKAVEEALVRASEVEKGNLGARIMHEPDSPGLKKLKDVLNSMLNTLQGKIGSDINVIQKTFDDFK

NLDFTSNIPNAKGEVEKVTNLLGNEITKMLKDNLNQANNLKEKANSLKEYVTTLNDSARSQANSLQESAAAVEEMSSSMSSINERAGEVIKQSEDIKNIITIIRDIADQTNLLALNAAIEAARAGDHGRGFAVVADEVRQLAERTQKSLGEIEANVNILS

QSINEMSQSISEQTEAINQINEAVANVDEQTKQNLAIASNTDRVTIEVETIANEVVSEVKRKKF

**>*C. concisus*_P2CDO4_Tlp500**

MKKAANKIALIIVVLLVVSLGVFSAINYTNTKENIYDLAKDTKTSSSKILQFYMNAFFEDKIASVDSFAKYLESHPEILD

DKEILEKELMIGARSTEFSEFYFAFANDGAVYDAVLDGKDRKFVIFDKRKNYDARVKDWYKDTVAKDNVAFSAPYASSASGLNITITKKVVVNGKVVGVFGIDIGIDKLNEELGKIKPTPSSAIALFDLEHKKMIHFTHKDLVLSDSSEAMKIIDDYSNE

YKKNGDKTFTYNLRGTERLLACELYDQANWLVCSANSWSDYDGSLSKTLVSQVISSVIFIVVIVVLLIFIVSRSLKPLSK

ISDSLISFFKFLNYEIKEPVKSNIVSKDEFGVMSSLINENIQKIQTAKENENAFIQKANTFVNEIKDGNYEASLEADTNN

PALNQLKSTFKDLQLALKSAISSNGKDVLDLLNTYKNQDFTKRLDDDGKIASGINSLGIEISKMLNDNLNQAQVLEEKAK

LLASSVSKVASSANTQANSLQESAAAVEQMSSSMNAISQKTADVIRQSDEIKNIITIIRDIADQTNLLALNAAIEAARAG

EHGRGFAVVADEVRKLAERTQKSLGEIEANTNVLAQSINEMSESIKEQSEGINMINQSVAQIDNLTKENVVIANQANEVTSEVDEMAKAIVEEVRKKR

**>*C. concisus*_P2CDO4_Tlp501**

MKSITNKIALMLIVALCISFVAMSAASYYTAQNKTTELVTQAQRQILKDVKNAMDIFFNNNLHIVDSMSSILSKLDENRN

GIDAILAQGKSMSNKEVGLVYAGYNDGAMYRSNGKNETPKDGYDPRARDWYKLAKEKNGITFTDPYISSSLKQMVISFVAPIKDAGVAATNVSIEELSKEIMSISKTDYSYAFVADKDGKIIIHPDKNIINTAPELTKKLIERYKAKDFDENGLILYKNL

KGEDLYADFIELNDRGWLAISAMQKDVFTTNTLPLLKIQLILAVLFIVILSAFVYFLLKKSLNPIKTIQSKLDDLFKFVT

YEAKAPSKLEVRSNDEFGEMSKAINENIDKVVAGIKKDSTMIDELNKVANLMIKGSLGAKIGSTPNNPSLNELKELLNKF

FTSISANLKGITNVLSSYTKNDFTAKVEINEELEADLKAMILGVSNMGEVICTMLNSNLNDAKMLEEKATTLASAMKELT

QGASTQASSLQESAAAVEQMSSSMNAISQKTADVIRQSDEIKNIITIIRDIADQTNLLALNAAIEAARAGEHGRGFAVVA

DEVRKLAERTQKSLGEIEANTNVLAQSINEMSESIKEQSEGINMINQSVAQIDHLTKENVVIANRANEVTSDVDNMAKTIVSEVRKNKF

**>*C. concisus*_P2CDO4_Tlp502**

MNNLSIKIKILLIVILSLICLSTTSIYILNGVFKTRSQAVSSLNTAEDIIKQSEFIHELQKERGYSAGFIANGKDADKNL

KEQRAKVDGVLSKLSNKDELSSELNSIRAKVDSKESFALIAPKFHDMIENTLIFENSLASSSEPDMKDNLARIFSISKIK

EYFGITRAVLNAAFIKHNIDKNTYVNLVSFNANIKNLINDYVKFNAGTYADSLQKEILQSDEFKKIDDIIKSAIATPDET

AGKIEAASWFQSITNLIDSFRSYELYLLNDMKDKALQDMSEAGNLAVSMVILLACFILLLVLVSFFVGKNIISGIDSVKG

GLSEFFLYLNNKTNSAKLLSLKGKDEICIMSSLINENIQKIQTAKENENAFIQKANTFVNEIKDGNYEASLEADTNNPAL

NQLKSTFKDLQLALKSAISSNGKDVLDLLNTYKNQDFTKRLDDDGKIASGINSLGIEISKMLNDNLNQAQVLEEKAKLLA

SSVSKVASSANTQANSLQESAAAVEQMSSSMNAISQKTADVIRQSDEIKNIITIIRDIADQTNLLALNAAIEAARAGEHG

RGFAVVADEVRKLAERTQKSLGEIEANTNVLAQSINEMSESIKEQSEGINMINQSVAQIDHLTKENVVIANQANEVTSEV

DEMAKAIVEDVRKKRF

**>*C. concisus*_P2CDO4_Tlp503**

MRSISNKIALTLIVLLSVCFAVMSAVSYFNAKDEVVKLISQNQDQILSDIKSVTQSFIDDYMEDSQKLANKLAGSIGRED

EILARLKSTKENLKSIVIGAYFAAESNGYTYGSNGKTLTPEKDKYEPRGRGWYIAAKSSGKTIFTKPYIDMVAPEHDLCM

TFSTPITEGGKLLGATFTDVNIRLLSKKLLKMGKTEFGYVYFMDKDGVILLHDDESLINSSVEATKTLAAKFASKDFDEN

GLISYKNAKNENVLAKILPINDDGWLAVAAINADTFTSQTMPLLKIQLILAVLFIVILSAFVYFLLKKSLNPIKTIQSKL

DDLFKFVTYEAKAPSKLEVRSNDEFGEMSKAINENIDKVVAGIKKDSTMIDELNKVANLMIKGSLGAKIGSTPNNPSLNE

LKELLNKFFTSISANLKGITNVLSSYTKNDFTAKVEINEELEADLKAMILGVSNMGEVICTMLNSNLNDAKMLEEKATTL

ASAMKELTQGASTQASSLQESAAAVEQMSSSMNAISQKTADVIRQSDEIKNIITIIRDIADQTNLLALNAAIEAARAGEH

GRGFAVVADEVRKLAERTQKSLGEIEANTNVLAQSINEMSESIKEQSEGINMINQSVAQIDHLTKENVVIANRANEVTSDVDNMAKTIVSEVRKNKF

**>*C. concisus*_P2CDO4_Tlp504**

MKSLRIKISVILTAVIVLLLIGVSLISYNIARNLYTEKVVKDELPLAVSNVAGEIGYAIDKIINTSYQMTKNDYLLKWID

EGEPKDGLATLFNYNTDLMKAFNLSTAMFVSDKTLNYYTNDKILKQLSKDNPRDSWYFDVKNGKEVNSLNIQVSEATGSLTLYVNSKVEKDGKFYGVSAIGMNLDDIVNLVTSKTMGEGSKFLMVDSSGIVKIEKSDRVGKVNVKDVLGKEKFDVLMNKNGGVIRHFNGTRNLIIGSKYIPSLDWYLFGEMDEDVLLKDLHTLFYSAIGVILAAIIISVIVSLLMSSYLLKIILKLKTGL

LSFFDLLNHKTNKAQPIEITSKDEFGQMADLINKNTKAIEDGMKDQSIFIQKANTFVNEIKDGNYEASLEADTNNPALNQ

LKSTFKDLQLALKSAISSNGKDVLDLLNTYKNQDFTKRLDDDGKIASGINSLGIEISKMLNDNLNQAQVLEEKAKLLASS

VSKVASSANTQANSLQESAAAVEQMSSSMNAISQKTADVIRQSDEIKNIITIIRDIADQTNLLALNAAIEAARAGEHGRG

FAVVADEVRKLAERTQKSLGEIEANTNVLAQSINEMSESIKEQSEGINMINQSVAQIDNLTKENVVIANQANEVTSEVDE

MAKAIVEDVRKKR

**>*C. concisus*_P2CDO4_Tlp505**

MKSLRMRTALVLSVMLLVCVSVVGLVTYNMAKNLYTDRVESKEFPLAVNEVGYKIESYIDKIIDASYQITQNSFLIDWVK

NGEDEHGRKVLFDYFQTLAKNLKLDTIMFGSDKTLNYYVEDKILKVINPENDKWYYALKDGSSLSLIDINKAEDGSGKIM

MYINYKVQKDDKFYGIAATGVNLSEIVNFVQSQKIGRGGKFLLVDEKGDIKIGKDVSGNLKDLVGDANLKKLINENGSYI

SLDKDGRNLLIGSKYIKSMGWYLIGELDKDELLEDLNSLAYASVGVLVVVLILGLLLSLYLSSYLLKIILKLKTGLLSFF

DLLNHKTNKAQPIEITSKDEFGQMADLINKNTKAIEDGMKDQSIFIQKANTFVNEIKDGNYEASLEADTNNPALNQLKST

FKDLQLALKSAISSNGKDVLDLLNTYKNQDFTKRLDDDGKIASGINSLGIEISKMLNDNLNQAQVLEEKAKLLASSVSKV

ASSANTQANSLQESAAAVEQMSSSMNAISQKTADVIRQSDEIKNIITIIRDIADQTNLLALNAAIEAARAGEHGRGFAVV

ADEVRKLAERTQKSLGEIEANTNVLAQSINEMSESIKEQSEGINMINQSVAQIDHLTKENVVIANQANEVTSEVDEMAKAIVEDVRKKRF

**>*C. concisus*_P2CDO4_Tlp506**

MFRSITNKIALALMILLIISFCAISAVSYFTSKDKIVELVSGKEDQVLKDIKSVANTFFDENLEHIKKISSAVEGAQSGD

EIMNFVLLEKKVANSAVKYVYYGDEDGHFFQSDGKRTTVADNYDPRTRGWYKSTKEKNKEIYTEPRVTSTNDLVVSFTAPVTKNGKFVGVAGIDVDVKKVSDKIIEIGNTGDGGYAYVMRKDGTMLFSDVVSEVGTILPATKVVSEKYNNKEFDENGLISYKNSKNQDVTAKILPINDDGWLATVAIGADTFSNHTMPILKAQLMLAVLFIVILSAIVFVLLKRSLKPISVIQEKLSDTF

KFITYESSTAPSKLDIHTTDEFGVMSEEINKNIDKVIAGIKKDNTMLEELNVAANNMIRGNLGVKLNENPNNPSLIKLKD

LLNTFFSSISQNLGSVINILKAYSNNDYTAKIELRDDIEADLKAMIVGINNAGDAISAMLNSNLNQAQNLEEKATMLAES

MRNLTDGASKQADSIQESAAAVEQMSSSMNAISQKTGDVIRQSEEIKNIIVIIRDIADQTNLLALNAAIEAARAGEHGRG

FAVVADEVRKLAERTQKSLGEIEANTNVLTQSINEMSESIKEQAEGINMINRSVAQIDNVTKENRSVVSNTNDVTSEIDG

MAKVILTDVRKNKF

**>C. *concisus*_ATCC33237_Tlp507**

MKSITSKIAMIIISLLVISFAAISAVSYYTAESKVVELVSQTQDQILSDIKATTDSFFEDYLEVAKKSASDIAQTPNNDD

SYMERTKIQKENVSHLVSNIFYGRESDGHFFQSDGTRTTPADNYDPRTRGWYKAAKSANGAIYTEPYKAAMFNALVISFAAPVNKNGNFDGVLGLDLNIDALSKKILEMGKTKYGYVYLMNKDGLILMHNDPNNVGKTVPASKYLAEAFAAKKFDENGLIPYTNYKGENVTAKVMAINDQGWLAVAAIGADTFSSNTLPLLKAQIILAVAFIVILSAIVFVLLKKSLSPIRTIQTKLEDA

FKFITYETPNAPEKLAVTSEDEFGKMSESINENIEKVLNGVKRDSALIEEMNGIANLMIKGHMGAKINSVPNNPALVQLK

DLLNRFFTSISDNLKGIAVVLASYNKNDYTPRLELKPELESDLKDMIVGLQSAGDAVSNMLRENLKEAESLEAKAKILAE

SMKSLTDGAHKQADSIQESAAAIEEMSSSMNAISQKASDVTRQSEEIKNIIVIIRDIADQTNLLALNAAIEAARAGEHGR

GFAVVADEVRKLAERTQKSLGEIEANANVLAQSINEMSESIREQSEGINMINQSVSQIDSITKQNINIVGTTNEITDQID

DMAKTIVADVRKNKF

**>*C. concisus*_ATCC33237_Tlp508**

MRSIANKISLMLILALFISFSLISLASYNTAHDKAIELVIQTQKQILKDVKITLNSFFTNNQYAIEKMAETLSKISEKSD

GSMNADGINLALSQAKAVSGKEITLIYAGYEDGAMFRSNGQNKAGYDPRVRGWYKQAKAENKPIYTDPYMAASLNAMVITFAAPIKNIGVTGIDASIEELSNNISQISKTEYSYAFVTDQNGNVIMHPNKELVGKPHEIAKSLIKQYNEKKFDENGLIAYKNTKGEDVYAYMLEINDKGWLAITAMDQNIFSSHTLPILKVQIILAFIFIVVLSAFVYFLLKRSLNPIKTITDALVSFFR

FLNFEIKEPVVSKVATKDEFGVMSKLINDNIAKIFDNADQDSRVVSQSVETAKAIENGNLKARIVDVPANPKLIELKDVL

NKMLDVLEKRVGSDLNMIQKTFNDFRNSDFTSRILDAKGNVELVTNELGEEIANMLAFNLKQAQFLEEKAKNLDASMKQVTQGASTQANSLQESAAAIEQMSSSMSAISQKTVDVIKQSEEIKNIIVIIRDIADQTNLLALNAAIEAARAGEHGRGFAVVADEVRKLAERTQKSLGEIETNANILTQSINEMSESIREQSEGINMINQSVSQIDSITKQNVDIVSSTNEITAQIDEMAKTIVADVKKNKF

**>*C. avium*_LMG24591_Tlp600**

MKLSFKIITYIVAVFVVIMATSIYSSFRSLSSNVNKLHDLVQSTVLDASYTTINITMGIEAQQHLEYIASVMKNMPSSNI

GDIRRAISRISGAVKYPDMFIVYENGNYINESYTPGKVSFSDAYTSIDMDMRTRPWYVEAKAKNGFFVTNAYISQAGSTKGKMVATASLPFYDSNDKFAGVVAMDIIVDGFQERFKNFSTPIFPSLRVMIFDSNFDIISHPNSKLVMDNYDTPTEKYLKDNKGNVGENFVIVNNEGIASYVHYKKFPFGWSMAVSASIIDYERAVNNAVLQDIVLGIIMLIIGSLILVYIIRRFLAPLGN

ISTSLAQLFAFINHESKNAPSIKLLNSKDELAQMTRLMDDNIKRTKINMEDDTKLVNEVVSVVDEAKKGKFGTIISQNSK

NPQTNKIKDSLNEMTNALTTLLGFDLSKPASVFESFEKNDFTARIENPQGLEKGVNSLGDSIASMLKTSASYANKLSTRA

DELKESMQKLTDGSKAQANSLEQSAAAVEEISSSMQNISGKTEDVARQADDIKSIVEVIKDIADQTNLLALNAAIEAARA

GEHGRGFAVVADEVRQLAERTGKSLSEIEANINLLVQSVNEVSESIREQTAGVTQINESIAELESVTRENVSVANDTNSI

TEEVNTIASDILTDVNKKRF

**>*C. avium*_LMG24591_Tlp601**

MLSSIKIKVSAIANLISILALALLGFITFYFVEKNVANEVITAYSNYVKTADLQMREYDSKNLEILNTLAHNIQALGDEH

FASEEAIIDAVGPMLKNYKDSSKVLMIYLGFSADGEMLTSDPVSDERNLAYRIRGKADNYDARTRPWYKQALQDNKAVTSESYIDSLTGKPCITYSIPIYKGGKLIGILGIDVLTAEIQKMFDGLHARIFTFDREFNIFASKDQELLAKRTTANLPIIAQ

KHKEAGDFNYFIYTSTEGSERFVICASYIDYLTCVAEPLDIVKKPAIDIAYIQTIVVALVILISIFAMYFVMSRFLSPIN

HIQQGLNNFFDFLNHKKQTITPIHIKSNDEFGQIAKAINLNIENTRLGLEQDKQAVSQSVNTVHLVENGDLTARISANPK

NPQLIELKNVLNKMLDVLQEKIGSNMNEIRRVFDSYKALDFTTEVANAKGNVEVTANTLGKEIVKMLKQSNDFANSLVSDSTKLQEAVQELQNSSKSQASSLEESAAALEEITSSMQNVSSKTTDVIAQSEEIKSITNIIGDIAEQINLLALNAAIEAAR

AGEHGRGFAVVADEVRQLAEKTQKSLSEIEANINLLVQSINDMAESIKEQTTGITQINDAVAQIESVTRDNVRIANNSAS

ISDSVSSIANDILEDAKRKKF

**>*C. avium*_LMG24591_Tlp602**

MKTSVSSKLTLFIALLFLIILTAITLVMYQNAKATVYQHLSAVQNKTVEDVSRAYDIYADVKRNAMRKAAEILSQNIDSY

NEKDLIELAKMLKESAGYSIAYIALEHNGNNYYSNGNINDNKQGGYDTLNRPWYKAAKEKGELVVSEPYLSNTSGKFEMTYSAPLIKDGKFYGVVAGDFDLTVYSHGVLNFGKSASTDSFVVNVNDGSIMFHMDEKKMLQKDKLSQAIAEAVKVEPRILDPEHTDYIFYEVDDNGIEQAIMCHNTINPSYKACSVTQASVYENEVKKQLVSSAIVGIVSLLISLLIVRIIIARSIRPLQA

IQQGLNNFFDFLNHKKQTITPIHIKSNDEFGQIAKAINLNIENTRLGLEQDKQAVSQSVNTVHLVENGDLTARISANPKN

PQLIELKNVLNKMLDVLQEKIGSNMNEIRRVFDSYKALDFTTEIANAKGNVEVTANTLGKEIVKMLKQSNDFANSLVSDS

TKLQEAVQELQNSSKSQASSLEESAAALEEITSSMQNVSSKTTDVIAQSEEIKSITNIIGDIAEQINLLALNAAIEAARA

GEHGRGFAVVADEVRQLKLEKTQKSLSEIEANINLLVQSINDMAESIKEQTTGITQINDAVAQIESVTRDNVRIANNSAS

ISDSVSSIANDILEDAKRKKF

**>*C. avium*_LMG24591_Tlp603**

MFSRLSIGTKLMFSVGLTVVIGLCILVVVITTNLSSSMVKKAEMIIEEQTFAYANYAKGFFDELISVVNNSANVLESVFA

SSNINDISLQRLSNVISSVADSGYHISYSFFYILNPPSHFKQNPLFQTSNGKTIILFENQSINTKGGIAAVKSTDEISSL

DSVVKVIENASKANSSQISISKPMRMTMNNKTFVGSSVASAVYGENNELIGVIGAILDFAIIDEVLSDPSTFNFENEARA

FLYQDGTVGLHLNKDLQLSKLQDRIDKDKNKEALLAMAENETGVHDYTTSGGIDSYLSIHSFGLENNSSRWMMLVSAPKSAVLAELHSLQKIIIALSLVVLLAILLIVYFFVRKTVSGRLPTIVLALERLFKYINHEVNNIEPIKIRAKDELGKIGLMIN

ENAKNTQRALEKDSNLVKEALDVINHTRGGHATRRITLQGSNPQLNSLKDSVNQLLDLLSTAIGNDLPELNRVFDSFVKL

DFSTEVKEAKGRVEIVTNTLGEEIRKMLKTSASYANKLSTRADELKESMQKLTDGSKAQANSLEQSAAAVEEISSSMQNI

SGKTEDVARQADDIKSIVEVIKDIADQTNLLALNAAIEAARAGEHGRGFAVVADEVRQLAERTGKSLSEIEANINLLVQS

VNEVSESIREQTAGVTQINESIAELESVTRENVSVANDTNSITEEVNTIASDILTDVNKKRF

dCache_1: small molecule recognition

single Cache2 domain small molecule recognition

NIT domain pfam08376 nitrate sensing domain

cache2 domain

CheW interface interaction of Tlp with CheW

Dimer interface

MCP signal domain pfam00015: transduces signal to CheA

Methylation site

nitrate sensing domain
