# Supplementary material for: Diversity of transducer-like proteins (Tlps) in Campylobacter
Source: PLoS One. 2019 Mar 25;14(3):e0214228. doi: 10.1371/journal.pone.0214228 (PMC6433261; doi:10.1371/journal.pone.0214228)
Supplement: S2 Fig — (DOCX) [file pone.0214228.s007.docx]

CLUSTAL O(1.2.4) multiple sequence alignment 2018/05/16

Cfv Tlp404 AAAVEQISCS 10

Cfv Tlp403 AAAIEEMSNS 10

*C. concisus* Tlp507 AAAIEEMSSS 10

*C. jejuni*, *C. coli*, *C. lari*, and C. helveticus Tlps; C. avium Tlp601, Tlp602 AAALEEITSS 10

*C. jejuni*, *C. jejuni* supsp. *doylei*, *C. coli* Tlp1 AAAVEEINSS 10

*C. avium* Tlp600, Tlp603 AAAVEEISSS 10

Cfv Tlp400-402, Cff Tlp405, Cft Tlp406-409 AAAVEEMSSS 10

C. concisus Tlp500-506, Tlp508 AAAVEQMSSS 10

***:*::. *
