# Supplementary figures and images for: Diversity of transducer-like proteins (Tlps) in Campylobacter
Source: PLoS One. 2019 Mar 25;14(3):e0214228. doi: 10.1371/journal.pone.0214228 (PMC6433261; doi:10.1371/journal.pone.0214228)

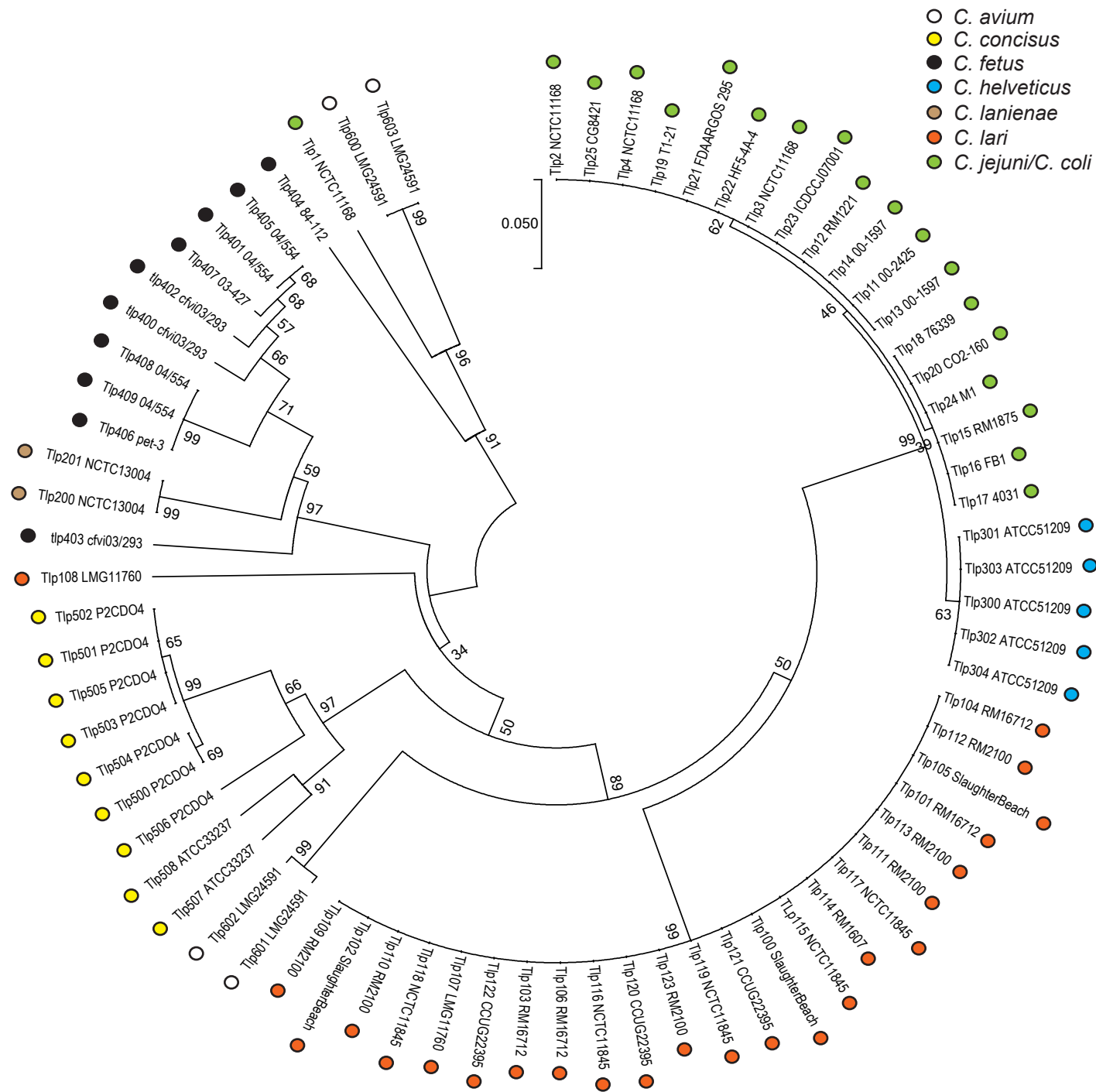

Supplement: S3 Fig — (PDF) [file pone.0214228.s008.pdf]

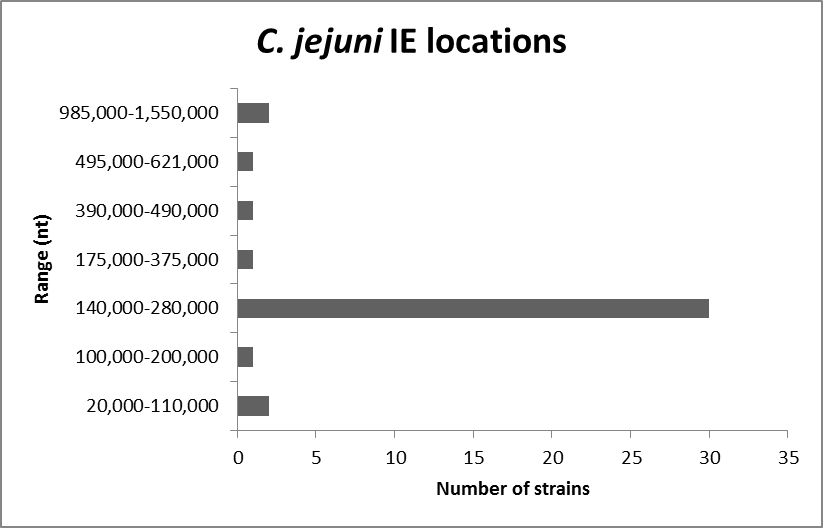

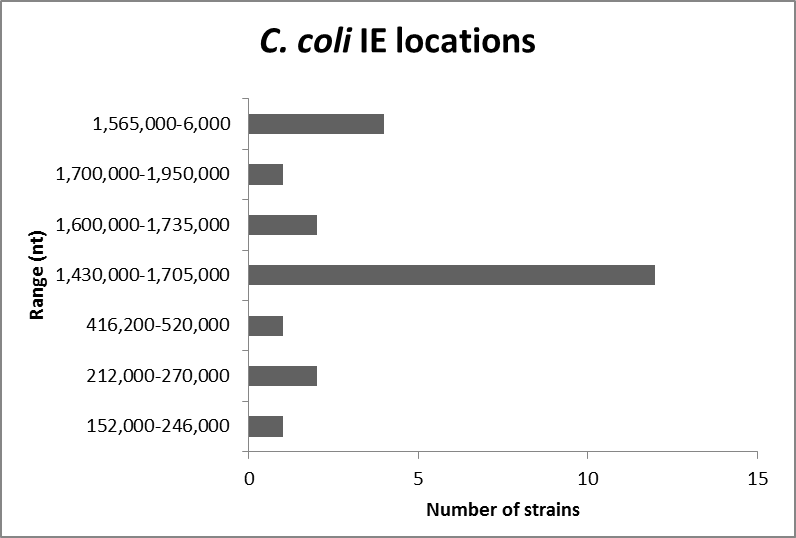

Supplement: S4 Fig — (DOCX) [file pone.0214228.s009.docx]
