# Supplementary material for: Diversity of transducer-like proteins (Tlps) in Campylobacter
Source: PLoS One. 2019 Mar 25;14(3):e0214228. doi: 10.1371/journal.pone.0214228 (PMC6433261; doi:10.1371/journal.pone.0214228)
Supplement: S1 Table — (DOCX) [file pone.0214228.s011.docx]

| **Strain** | **Accession Number** | **Reference/source** |
| --- | --- | --- |
| *C. jejuni* |  |  |
| NCTC11168 = ATCC 700819 | NC_002163.1 | 1,2 |
| RM1221 | NC_003912.7 | 3 |
| 81-176 | NC_008787.1 | 4 |
| 81116 | CP000814.1 | 5 |
| 00-0949 | NZ_CP010301.1 | 6 |
| 00-1597 | NZ_CP010306.1 | 6 |
| 00-2425 | NC_022362.2 | 7 |
| 01-1512 | NZ_CP010072.1 | 6 |
| 00-6200 | NZ_CP010307.1 | 6 |
| 14980A | NZ_CP017029.1 | 8 |
| 32488 | NC_021834.1 | 9 |
| 35925 | NZ_CP010906.1 | 10 |
| 4031 | NC_022529.1 | 11 |
| BCW_6290 | CP017673.1 | 12 |
| CFSAN032806 | CP023543.1 | 13 |
| CG8421 | NZ_CP005388.1 | 14 |
| CJ677CC012 | CP010487.1 | 15 |
| CJ677CC527 | NZ_CP010506.1 | 15 |
| CJM1cam | NZ_CP012149.1 | 16 |
| FJ3124 | CP017862.1 | 17 |
| F38011 | NZ_CP006851.1 | 18 |
| FDAARGOS_295 *C. jejuni* subsp. *doylei* | CP027403.1 | 19 |
| FDAARGOS_421 | CP023866.1 | 20 |
| FDAARGOS_422 | CP023867.1 | 21 |
| FORC_046 | NZ_CP017229.1 | 22 |
| HF5-4A-4 | CP007188.1 | 23 |
| IA3902 | NC_017279.1 | 24 |
| ICDCCJ07001 | CP002029.1 | 25 |
| M1 | NC_017280.1 | 26 |
| MTVDSCj07 | CP017031.1 | 27 |
| MTVDSCj13 | CP017032.1 | 28 |
| MTVDSCj16 | CP017033.1 | 29 |
| PT14 | CP003871.3 | 30 |
| R14 | NZ_CP005081.1 | 31 |
| RM1285 | NZ_CP012696.1 | 32 |
| RM3196 | NZ_CP012690.1 | 33 |
| S3 | NC_017281.1 | 34 |
| T1-21 | CP013116.1 | 35 |
| YH001 | NZ_CP010058.1 | 36 |
|  |  |  |
| *C. coli* |  |  |
| 14983A | NZ_CP017025.1 | 8 |
| 15-537360 | NC_022660.1 | 37 |
| 76339 | NC_022132.1 | 38 |
| BFR-CA-9557 | NZ_CP011777.1 | 39 |
| BG2108 | CP017878.1 | 40 |
| BP3183 | CP017871.1 | 40 |
| CF2-75 | NZ_CP013036.1 | 41 |
| CFSAN032805 | CP023545.1 | 42 |
| CO2-160 | NZ_CP013032.1 | 41 |
| CVM N29710 | NC_022347.1 | 43 |
| FB1 | NZ_CP011015.1 | 44 |
| HC2-48 | NZ_CP013034.1 | 41 |
| MG1116 | CP017868.1 | 40 |
| OR12 | NZ_CP013733.1 | 45 |
| RM1875 | NZ_CP007183.1 | 46 |
| RM4661 | NZ_CP007181.1 | 46 |
| RM5611 | NZ_CP007179.1 | 46 |
| WA333 | CP017873.1 | 40 |
| YF2105 | CP017865.1 | 40 |
| YH501 | CP015528.1 | 47 |
| YH502 | CP018900.1 | 48 |
| YH503 | CP025281.1 | 49 |
| ZV1224 | CP017875.1 | 50 |
|  |  |  |
| *C. avium* |  |  |
| LMG 24591 | NZ_CP022347.1 | 51 |
|  |  |  |
| *C. concisus* |  |  |
| ATCC 33237 | NZ_CP012541 | 52 |
| P2CDO4 | CP_021642 | 53 |
|  |  |  |
| *C. fetus* subsp. *fetus* |  |  |
| 82-40 | NC_008599 | 54, 55 |
| 04/554 | NZ_CP008808 | 56 |
|  |  |  |
| *C. fetus* subsp. *testudinum* |  |  |
| 03-427 | NC_022759.1 | 57 |
| pet-3 | NZ_CP009226.1 | 58 |
|  |  |  |
| *C. fetus* subsp. *venerealis* |  |  |
| 01/165 | NZ_CP014568.1 | 56 |
| 84-112 | NZ_HG004426.1 | 59 |
| cfvi03/293 | NZ_CP006999.2 | 60 |
|  |  |  |
| *C. helveticus* |  |  |
| ATCC 51209 | NZ_CP020478.1 | 61 |
|  |  |  |
| *C. lanienae* |  |  |
| NCTC 13004 | NZ_CP015578.1 | 62 |
|  |  |  |
| *C. lari* |  |  |
| CCUG 22395 | NZ_CP007776.2 | 63 |
| NCTC 11845 | NZ_CP007775.1 | 63 |
| RM 16701 | NZ_CP007777 | 63 |
| RM2100 | NC_012039.1 | 64 |
| RM16712 | NZ_CP007778.1 | 63 |
| Slaughter Beach | NZ_CP011372.1 | 65 |
| *C. lari* subsp. *concheus* LMG 11760 | NZ_CP007771 | 63 |

**References for Table 1.**

1. Parkhill J, Wren BW, Mungall K, Ketley JM, Churcher C, Basham D, et al. The genome sequence of the foodborne pathogen *Campylobacter jejuni* reveals hypervariable sequences. Nature. 2000; 403:665-8.
2. Gundogdu O, Bentley SD, Holden MT, Parkhill J, Dorrell N, Wren BW. Re-annotation and re-analysis of the Campylobacter jejuni NCTC11168 genome sequence. BMC Genomics. 2007; 8:162.
3. Fouts DE, Mongodin EF, Mandrell RE, Miller WG, Rasko DA, Ravel J, et al. Major structural differences and novel potential virulence mechanisms from the genomes of multiple *Campylobacte*r species. PLoS Biol. 2005; 3(1):E15.
4. Hofreuter D, Tsai J, Watson RO, Novik V, Altman B, Benitez M, et al. Unique features of a highly pathogenic *Campylobacter jejuni* strain. Infect Immun. 2006; 76(8):4694-4707.
5. Pearson BM, Caskin DJH, Segers RPAM, Wells JM, Muijten PJM, van Vliet AHM. The complete genome sequence of *Campylobacter jejuni* strain 81116 (NCTC11828). J Bacteriol 2007; 189(22):8402-3.
6. Clark CG, Chen C-y, Berry C, Walker M, McCorrister SJ, Chong PM, Westmacott GR. Comparison of genomes and proteomes of four whole genome-sequenced *Campylobacter jejuni* from different phylogenetic backgrounds. PLoS ONE. 2018; 13(1): e0190836. <https://doi.org/10.1371/journal.pone.0190836>
7. Clark CG, Berry C, Walker M, Petkau A, Barker DOR, Guan C, et al. Genomic insights from whole genome sequencing of four clonal outbreak *Campylobacter jejuni* assessed within the global *C. jejuni* population. BMC Genomics. 2016; 17:990. doi 10.1186/s12864-016-3340-8
8. Miller WG, Huynh S, Parker CT, Niedermeyer JA, Kathariou S. Complete genome sequences of multidrug-resistant *Campylobacter jejuni* strain 14980A (turkey feces) and *Campylobacter coli* strain 14983A (housefly from a turkey farm), harboring a novel gentamicin resistance element. Genome Announc. 2016; 4(5). pii: e01175-16. doi: 10.1128/genomeA.01175-16
9. Jones J, Zhao S, Zie Y, Thao K, Cao N, Clark TA, et al. *Campylobacter jejuni* 32488, complete sequence. 2013. Unpublished.
10. Ghaffar N, Connerton PL, Connerton IF. *Campylobacter jejuni* subsp. *jejuni* strain 35925. 2015. Unpublished.
11. Rossi M, Revez J, Schott T, Hanninen ML*. Campylobacter jejuni* 4031 genome sequence. 2013. Unpublished.
12. Weils AM, Weimer BC, Clothier KA. Kong NT. *Campylobacter jejuni* strain BCW_6920. 2016. Unpublished.
13. Hoffman M, Sanchez M, Gaugher J, Timme R. *Campylobacter jejuni* strain SFSAN032806 chromosome, complete. 2017. Unpublished.
14. Poly F, Read TD, Chen YH, Monteiro MA, Serichantalergs O, Pootong P, et al., Characterization of two *Campylobacter jejuni* strains for use in volunteer experimental-infection studies. Infect Immun. 2008; 76(12):5655-67.
15. Skarp CPA, Akinrinade O, Nilsson AJE, Ellström P, Myllykangas S, Rautelin H. 2015. Comparative genomics and genome biology of invasive *Campylobacter jejuni*. Sci Rep. 5:17300. doi: 10.1038/srep17300
16. Baig A. Second-site mutations responsible for motility defects in *Campylobacter jejuni* defined gene deletion mutants. 2015. Unpublished.
17. Marasini D, Fakhr MK. Complete genome sequences of *Campylobacter jejuni* strains isolated from retail chicken and chicken gizzards. Genome Announc. 2017; 5(47): pii e01351-17. doi: 10.1128/genomeA.01351-17.
18. Eucker TP, O’Loughlin JL, Samuelson DR, Chavez J, Bruce J, Konkel ME. *Campylobacter jejuni* subsp. *jejuni* F38011. 2013. Unpublished.
19. Kerrigan L, Tallon L, Sadzewicz L, Sengamalay N, Ott S, Gocinez A, et al. FDA dAtabase for Regulatory Grade microbial Sequences (FDA-ARGOS): Supporting development and validation of infectious diseases Dx tests. 2018. Unpublished.
20. Kerrigan L, Long C, Tallon L, Sadzewicz L, Ott S. Zhao X, et al. FDA dAtabase for Regulatory Grade microbial Sequences (FDA-ARGOS): Supporting development and validation of infectious diseases Dx tests. 2018. Unpublished.
21. Regulatory Grade microbial Sequences (FDA-ARGOS): Supporting development and validation of infectious diseases Dx tests. 2017. Unpublished.
22. Chun J, Ryu S. Genome analysis of the chromosome of *Campylobacter jejuni* isolates. 2016. Unpublished.
23. Timms AR. Campylobacter succession in poultry. 2014. Unpublished.
24. Luo Y, Sahin O, Dai L, Sippy R, Wu Z, Zhang Q. Development of a loop-mediated isothermal amplification assay for rapid, sensitive and specific detection of a *Campylobacter jejuni* clone. J Vet Med Sci. 2012; 74(5):591-6.
25. Zhang M, He L, Li Q, Sun H, Gu Y, You Y, et al. Genomic characterization of the Guillain-Barré syndrome associated *Campylobacter jejuni* ICDCCJ07001 isolate. PLoS ONE. 2010;5(11): e15060. doi: 10.1371/journal.pone.0015060
26. Friis C, Wassenaar TM, Javed MA, Snipen L, Lagesen K, Hallin PF, et al. Genomic characterization of *Campylobacter jejuni* strain M1. PLoS ONE 2010; 5(8): e12253. doi: 10.1371/journal.pone.0012253
27. Tavierne ME, Parker CT, Huynh S, DiRita VJ. Complete genome of *Campylobacter* *jejuni* subsp. *jejuni* str. MTVDSCj16, isolated from a naturally colonized farm-raised chicken. 2016. Unpublished.
28. Tavierne ME, Parker CT, Huynh S, DiRita VJ. Complete genome of *Campylobacter* *jejuni* subsp. *jejuni* str. MTVDSCj13, isolated from farm-raised chicken. 2016. Unpublished.
29. Tavierne ME, Parker CT, Huynh S, DiRita VJ. Complete genome of *Campylobacter* *jejuni* subsp. *jejuni* str. MTVDSCj16, isolated from a naturally colonized farm-raised chicken. 2016. Unpublished.
30. Brathwaite KJ, Siringan P, Moreton J, Wilson R, Connerton IF. Complete genome sequence of universal bacteriophage host strain *Campylobacter jejuni* subsp. jejuni PT14. Genome Announc. 2013; 1(6): pii: e00969-13. Doi: 10.1128genomeA.00969-13
31. Connerton IF, Cummings NJ, Siringan P. Phenotypic changes associated with genome rearrangements of *Campylobacter jejuni*. 2013. Unpublished.
32. Gunther MW IV, Bono JL, Needleman DS. Complete genome sequence of *Campylobacter jejuni* RM1285, a rod-shaped morphological variant. Genome Announc. 2015; 3(6): pii: e01361-15. doi: 10.1128/genomeA.e01361-15
33. Parker CT, Huynh S, Keikeme AP, Cooper KK, Miller WG. Complete genome sequences of *Campylobacter jejuni* strains RM3196 (233.94) and RM3198 (308.95) isolated from patients with Guillain-Barré Syndrome. Genome Announc. 2015; 3(6): pii: e01312-15. doi: 10.1128:genomeA. e01312-15
34. Cooper KK, Cooper MA, Zuccolo A, Law B, Joens LA. Complete genome sequence of *Campylobacter jejuni* strain S3. J Bacteriol 2011; 193:1491-2.
35. Marasini D and Fakhr MK. Whole-genome sequencing of a *Campylobacter jejuni* strain isolated from retail chicken meat reveals the presence of a megaplasmid with Mu-like prophage and multidrug resistance genes. Genome Announc. 2016; 4(3): pii: e00460-16. doi: 10.1128/genomeA.e00460-16
36. He Y,Yan X, Reed S, Xie Y, Chen CY, Irwin P. Complete genome sequence of *Campylobacter jejuni* YH001 from beef liver, which contains a novel plasmid. Genome Announc. 2015; 3(1): pii: e01492-14. doi 10.1128/genomeA.e01492-14
37. Pearson BM, Rokney A, Crossman LC, Miller WG, Wain J, van Vliet AH. Complete genome sequence of the *Campylobacter coli* clinical isolate 15-537360. Genome Announc. 2013; 1(6): pii: e01056-13. doi: 10.1128/genomeA.e01056-13
38. Skarp-de Haan CP, Culebro A, Schott T. Revez J, Schweda EK Hänninen ML, et al. Comparative genomics of unintrogressed *Campylobacter coli* clades 2 and 3. BMC Genomics. 2014; 15:129. doi: 10.1186/1471-2164-15—129
39. Zautner AE, Goldschmidt AM, Thurmer A, Schuldes J, Bader O, Lugert R, et al. SMRT sequencing of the *Campylobacter coli* BfR-CA-9557 genome sequence reveals unique methylation motifs. BMC Genomics. 2015; 16(1):1088. doi: 1186/s12862-015-2317-3.
40. Marasini D, Fahkr MK. Complete genome sequences of plasmid-bearing *Campylobacter* *coli* and *Campylobacter jejuni* strains isolated from retail chicken liver. Genome Announc.2017; 5(49): pii: e01350-17. doi: 10-1128/genomeA.01350-17
41. Marasini D, Fahkr MK. Complete genome sequences of plasmid-bearing *Campylobacter* *coli* strains HC2-48, CF2-75, and CO2-160 isolated from retail beef liver. Genome Announc.2016; 4(5): pii: e01004-16. doi: 10-1128/genomeA.e01004-16
42. Hoffman M, Sanchez M, Baugher J, Timme R. Whole genome sequencing of cultured foodborne pathogens. 2017. Unpublished.
43. Chen Y, Mukherjee S, Hoffman M, Kotewicz ML, Young S, Abbot J, et al. Whole-genome sequencing of gentamicin-resistant *Campylobacter coli* isolated from U.S.retail meats reveals novel plasmid-mediated aminoglycoside-resistance genes. Antimicrob Agents Chemother. 2013; 57(11):3298-5405.
44. Connerton IF. *Campylobacter coli* strain FB1, complete genome. 2015. Unpublished.
45. O’Kane PM, Connerton IF. Characterisation of aerotolerant forms of a robust chicken colonizing *Campylobacter coli*. Front Microbiol. 2017; 8(513). doi: 10.3389/fmicb.2017.00513. eCollection 2017
46. Wright MS, Miller WG. Genomic analysis of four host-associated *Campylobacter coli*. 2014. Unpublished.
47. He Y, Yan X, Reed S, Strobaugh TP Jr, Irwin PL. Complete genome sequence of *Campylobacter coli* YH501 isolated from retail chicken. 2016. Unpublished.
48. Ghatak S, He Y, Yan X, Reed S, Strobaugh TP Jr, Irwin PL. whole genome sequencing and analysis of *Campylobacter coli* YH502 from retail chicken reveals a plasmid-borne type VI secretion system. Genom Data. 2017; 11:128-131. doi: 10.1016/j.gdata.2017.02.005. eCollection 2017 Mar
49. He Y, Yan X, Reed S, Strobaugh TP Jr, Irwin PL. Complete genome sequence of *Campylobacter coli* YH503 isolated from retail chicken. 2016. Unpublished.
50. Marasini DM, Fakhr MK. Complete genome sequencing of multidrug resistant *Campylobacter* spp isolated from retail turkey and pork. 2016. Unpublished.
51. Miller WG, Chapman MH, Yee E, Revez J, Bono JL. Rossi M. Complete genome sequence of the hippuricase-positive *Campylobacter avium* Type strain LMG 24591. Genome Announc. 2017; 5(43): pii: e01221-17. doi: 10.1128/genomeA.01221-17.
52. Cornelius AJ, Miller WG, Lastovica AJ, On SLW, French NP, Vandenberg O, Biggs PJ. Complete genome sequence of *Campylobacter concisus* ATCC 33237 and draft genome sequences for an additional eight well-characterized *C. concisus* strains. Genome Announc. 2017; 5(29): pii: e00711-17. doi: 10.1128/genomeA.00711-17
53. Liu F, Ma R, Tay CYA, Octavia S, Lan R, Chung HKL, et al. Genomic analysis of oral *Campylobacter concisus* strains identified a potential bacterial molecular marker associated with active Crohn’s disease. Emerg Microbes Infect. 2018; 7(1):64.
54. Fouts D, Nelson K. Sequence of *Campylobacter fetus* subsp. *fetus* 82-40. 2006. Unpublished.
55. Fahmy D, Day CJ, Korolik V. Comparative in silico analysis of chemotaxis system of *Campylobacter fetus*. Arch Microbiol. 2012; 194:57-63.
56. van der Graaf-van Bloois L, Miller WG, Yee E, Rijnsburger M, Wagenaar JA, Duim B. Inconsistency of phenotypic and genomic characteristics of *Campylobacter fetus* subspecies requires reevaluation of current diagnostics. J Clin Microbiol. 2014; 52(12):4183-88.
57. Gilbert MJ, Miller WG, Yee E, Blaser MJ, Wagenaar JA, Duim B. Complete genome sequence of *Campylobacter fetus* subsp. *testudinum* strain 03-427T. Genome Announc. 2013; 1(6): pii: e1002-13. doi: 10-1128/genomeA_e1002-13
58. Wang CM, Wu ZY, Shia WY, Jhou Y-J, Tung K-C, Shyu C-L. Complete genome sequence of *Campylobacter fetus* subsp. *testudinum* strain pet-3, isolated from a lizard *(Hydrosaurus pustulatus*). Genome Announc. 2015; 3(1): pii: e01420-14. doi: 10-1128/genomeA.01420-14
59. Kienesberger S, Sprenger H, Wolfgruber S, Halwachs B, Thallinger GG, Perez-Perez GI, et al. Comparative genome analysis of *Campylobacter fetus* subspecies revealed horizontally acquired genetic elements important for virulence and niche specificity. PLoS ONE. 2014; 9(1): e85491. doi: 10.1371/journal.pone.0085491
60. van der Graaf-van Bloois L, Miller WG, Yee E, Bono JL, Rijnsburger M, et al. first closed genome sequence of *Campylobacter fetus* subsp. *venerealis* bv. intermedius. Genome Announc. 2014; 2(1): pii: e01246-13. doi: 10-1128/genomeA.01246-13
61. Miller WG, Yee E, Bono JL. Complete genome sequence of *the Campylobacter helveticus* type strain ATCC 51209. Genome Announc. 5(21): pii:e00398-17. doi: 10.1128/genomeA.00398-17
62. Miller WG, Yee E, Lopes BS, Chapman MH, Huynh S, Bono JL, et al. Comparative genomic analysis identifies a *Campylobacter* clade deficient in selenium metabolism. Genome Biol Evol. 2017; 9(7):1843-58.
63. Miller WG, Yee E, Chapman MH, Smith TP, Bono JL, Huynh S, et al. Comparative genomics of the *Campylobacter lari* group. Genome Biol Evol. 2014; 6(12):3252-66.
64. Miller WG, Wang G, Binnewies TT, Parker CT. the complete genome sequence and analysis of the human pathogen *Campylobacter lari*. Foodborne Pathog Dis. 2008; 5(4):371-386.
65. Meinersmann RJ, Bono JL, Lindsey RL, Genzlinger LL, Loparev VN, Oakley BB. Genome sequence of a urease-positive *Campylobacter lari* strain. Genome Announc. 2015; 3(5): pii: e01191-15. doi: 10.1128/genomeA.01191-15
