# Supplementary material for: Diversity of transducer-like proteins (Tlps) in Campylobacter
Source: PLoS One. 2019 Mar 25;14(3):e0214228. doi: 10.1371/journal.pone.0214228 (PMC6433261; doi:10.1371/journal.pone.0214228)
Supplement: S2 Table — (DOCX) [file pone.0214228.s012.docx]

| **Flanking genes/proteins^1^**  **(either orientation)** | ***C. jejuni*** | ***C. coli*** | ***C. lari*** | ***C. lanienae*** | ***C. helveticus*** | ***C. fetus*** | ***C. concisus*** | ***C. avium*** |
| --- | --- | --- | --- | --- | --- | --- | --- | --- |
| two-component response regulator (TCRR) – **Tlp1** – ModE repressor domain protein (MERDP) | 38/38 | 6/6 | 0 | 0 | 0 | 0 | 0 | 0 |
| ABC transporter substrate-binding protein – **Tlp2** - transcriptional initiation protein Tat | 14/18 | 1/2 | 0 | 0 | 0 | 0 | 0 | 0 |
| zinc transporter ZupT – **Tlp2** –transcriptional initiation protein Tat | 2/18 | 0/2 | 0 | 0 | 0 | 0 | 0 | 0 |
| zinc transporter ZupT – **Tlp2** – membrane protein (MP1) | 2/18 | 0/2 | 0 | 0 | 0 | 0 | 0 | 0 |
| ABC transporter substrate-binding protein – **Tlp2** – flagellar protein export ATPase FliI (FPEAF) | 0/18 | 1/2 | 0 | 0 | 0 | 0 | 0 | 0 |
| Transcriptional regulator (TR1) – **Tlp3** – paralyzed flagella protein PflA (PPPP) | 32/36 | 5/6 | 0 | 0 | 0 | 0 | 0 | 0 |
| ABC transporter substrate-binding protein – **Tlp3** – SAM-dependent methyltransferase | 2/36 | 0/6 | 0 | 0 | 0 | 0 | 0 | 0 |
| ABC transporter substrate-binding protein – **Tlp3** – transcriptional initiation protein Tat | 2/36 | 0/6 | 0 | 0 | 0 | 0 | 0 | 0 |
| Transcriptional regulator (TR1) – **Tlp3** – flagellar protein export ATPase FliI (FPEAF) | 0/36 | 1/6 | 0 | 0 | 0 | 0 | 0 | 0 |
| zinc transporter ZupT – **Tlp4** – SAM-dependent methyltransferase | 14/15 | 0 | 0 | 0 | 0 | 0 | 0 | 0 |
| Transcriptional regulator (TR1) – **Tlp4** – paralyzed flagella protein PflA (PPPP) | 1/15 | 0 | 0 | 0 | 0 | 0 | 0 | 0 |
| zinc transporter ZupT – **Tlp11** –transcriptional initiation protein Tat | 3/5 | 0 | 0 | 0 | 0 | 0 | 0 | 0 |
| zinc transporter ZupT – **Tlp11** – SAM-dependent methyltransferase | 2/5 | 0 | 0 | 0 | 0 | 0 | 0 | 0 |
| zinc transporter ZupT – **Tlp12** – SAM-dependent methyltransferase | 11/11 | 0 | 0 | 0 | 0 | 0 | 0 | 0 |
| zinc transporter ZupT – **Tlp13** – membrane protein (MP1) | 3/7 | 0/20 | 0 | 0 | 0 | 0 | 0 | 0 |
| SAM-dependent methyltransferase – **Tlp13** – membrane protein (MP1) | 4/7 | 16/20 | 0 | 0 | 0 | 0 | 0 | 0 |
| hypothetical protein (HP1) – **Tlp13** – membrane protein (MP1) | 0/7 | 1/20 | 0 | 0 | 0 | 0 | 0 | 0 |
| paralyzed flagella protein PflA (PPPP) – **Tlp13** – membrane protein (MP1) | 0/7 | 3/20 | 0 | 0 | 0 | 0 | 0 | 0 |
| ABC transporter substrate-binding protein – **Tlp14** - transcriptional initiation protein Tat | 13/13 | 0/14 | 0 | 0 | 0 | 0 | 0 | 0 |
| Transcriptional regulator (TR1) – **Tlp14** – paralyzed flagella protein PflA (PPPP) | 0/13 | 11/14 | 0 | 0 | 0 | 0 | 0 | 0 |
| Transcriptional regulator (TR1) – **Tlp14** – flagellar protein export ATPase FliI (FPEAF) | 0/13 | 1/14 | 0 | 0 | 0 | 0 | 0 | 0 |
| Transcriptional regulator (TR1) – **Tlp14** – SAM-dependent methyltransferase | 0/13 | 3/14 | 0 | 0 | 0 | 0 | 0 | 0 |
| ABC transporter substrate-binding protein – **Tlp15** – transcriptional initiation protein Tat | 0 | 1/2 | 0 | 0 | 0 | 0 | 0 | 0 |
| CAAX amino protease – **Tlp15** - transcriptional initiation protein Tat | 0 | 1/2 | 0 | 0 | 0 | 0 | 0 | 0 |
| trimethylamine N-oxide reductase (TNORIC) – **Tlp16** – flagellar protein export ATPase FliI (FPEAF) | 0 | 6/13 | 0 | 0 | 0 | 0 | 0 | 0 |
| trimethylamine N-oxide reductase (TNORIC) – **Tlp16** – paralyzed flagella protein PflA (PPPP) | 0 | 2/13 | 0 | 0 | 0 | 0 | 0 | 0 |
| trimethylamine N-oxide reductase (TNORIC) – **Tlp16** – transcriptional initiation protein Tat | 0 | 5/13 | 0 | 0 | 0 | 0 | 0 | 0 |
| zinc transporter ZupT – **Tlp17** – SAM-dependent methyltransferase | 2/2 | 0 | 0 | 0 | 0 | 0 | 0 | 0 |
| SAM-dependent methyltransferase – **Tlp18** – membrane protein (MP1) | 0 | 1/1 | 0 | 0 | 0 | 0 | 0 | 0 |
| ABC transporter substrate-binding protein – **Tlp19** - transcriptional initiation protein Tat | 1/1 | 0 | 0 | 0 | 0 | 0 | 0 | 0 |
| Transcriptional regulator (TR1) – **Tlp19** – paralyzed flagella protein PflA (PPPP) | 1/1 | 0 | 0 | 0 | 0 | 0 | 0 | 0 |
| ABC transporter substrate-binding protein – **Tlp20** – transcriptional initiation protein Tat | 0 | 9/15 | 0 | 0 | 0 | 0 | 0 | 0 |
| ABC transporter substrate-binding protein – **Tlp20** – SAM-dependent methyltransferase | 0 | 1/15 | 0 | 0 | 0 | 0 | 0 | 0 |
| ABC transporter substrate-binding protein – **Tlp20** – DNA-binding protein | 0 | 1/15 | 0 | 0 | 0 | 0 | 0 | 0 |
| ABC transporter substrate-binding protein – **Tlp20** – flagellar protein export ATPase FliI (FPEAF) | 0 | 4/15 | 0 | 0 | 0 | 0 | 0 | 0 |
| SAM-dependent DNA methyltransferase (inferred) – **Tlp21** – paralyzed flagella protein PflA (PPPP) | 1/1 | 0 | 0 | 0 | 0 | 0 | 0 | 0 |
| SAM-dependent methyltransferase – **Tlp22** – CJIE1 prophage repressor | 1/1 | 0 | 0 | 0 | 0 | 0 | 0 | 0 |
| ABC transporter substrate-binding protein – **Tlp23** - transcriptional initiation protein Tat | 3/3 | 0 | 0 | 0 | 0 | 0 | 0 | 0 |
| ABC transporter substrate-binding protein – **Tlp24** - transcriptional initiation protein Tat | 2/2 | 0 | 0 | 0 | 0 | 0 | 0 | 0 |
| zinc transporter ZupT – **Tlp25** – SAM-dependent methyltransferase | 1/1 | 0 | 0 | 0 | 0 | 0 | 0 | 0 |
| aspartate ammonia lyase – **Tlp100** – hypothetical protein (HP2) | 0 | 0 | 2/2 | 0 | 0 | 0 | 0 | 0 |
| low molecular weight protein-tyrosine-phosphatase – **Tlp101** – hypothetical protein (HP3) | 0 | 0 | 4/4 | 0 | 0 | 0 | 0 | 0 |
| low molecular weight protein-tyrosine-phosphatase – **Tlp102** – hypothetical protein (HP3) | 0 | 0 | 1/1 | 0 | 0 | 0 | 0 | 0 |
| flagellar biosynthesis protein FlhB – **Tlp103** – peroxiredoxin | 0 | 0 | 2/3 | 0 | 0 | 0 | 0 | 0 |
| Tlp105 – **Tlp103** – peroxiredoxin | 0 | 0 | 1/3 | 0 | 0 | 0 | 0 | 0 |
| tRNA-Gly –**Tlp104** – Tlp105 | 0 | 0 | 4/4 | 0 | 0 | 0 | 0 | 0 |
| Tlp104 – **Tlp105** – DUF2874 domain-containing protein | 0 | 0 | 3/4 | 0 | 0 | 0 | 0 | 0 |
| flagellar biosynthesis protein FlhB – **Tlp105** – Tlp103 | 0 | 0 | 1/4 | 0 | 0 | 0 | 0 | 0 |
| prepilin-type cleavage/methylation domain-containing protein – **Tlp106** – major outer membrane protein | 0 | 0 | 1/1 | 0 | 0 | 0 | 0 | 0 |
| aspartate ammonia lyase – **Tlp107** – DUF493 domain-containing protein | 0 | 0 | 1/1 | 0 | 0 | 0 | 0 | 0 |
| Flavodoxin FldA – **Tlp108** – pyridoxine 5ˊ-phosphate oxidase family protein | 0 | 0 | 4/4 | 0 | 0 | 0 | 0 | 0 |
| tRNA-Gly – **Tlp109** – Tlp113 | 0 | 0 | 1/2 | 0 | 0 | 0 | 0 | 0 |
| tRNA-Gly – **Tlp109** – DUF2874 domain-containing protein | 0 | 0 | 1/2 | 0 | 0 | 0 | 0 | 0 |
| aspartate ammonia lyase – **Tlp110** – Tlp107 | 0 | 0 | 1/1 | 0 | 0 | 0 | 0 | 0 |
| RecB-like helicase – **Tlp111** – 50S ribosomal protein | 0 | 0 | 1/1 | 0 | 0 | 0 | 0 | 0 |
| flagellar biosynthesis protein FlhB – **Tlp112** – peroxiredoxin | 0 | 0 | 1/1 | 0 | 0 | 0 | 0 | 0 |
| Tlp109 – **Tlp113** – hypothetical protein (HP4) | 0 | 0 | 1/1 | 0 | 0 | 0 | 0 | 0 |
| prepilin-type cleavage/methylation domain-containing protein – **Tlp114** – major outer membrane protein | 0 | 0 | 1/1 | 0 | 0 | 0 | 0 | 0 |
| Tlp116 – **Tlp115** – hypothetical protein (HP2) | 0 | 0 | 1/1 | 0 | 0 | 0 | 0 | 0 |
| aspartate ammonia lyase – **Tlp116** – Tlp115 | 0 | 0 | 1/1 | 0 | 0 | 0 | 0 | 0 |
| low molecular weight protein-tyrosine-phosphatase – **Tlp117** – hypothetical protein (HP3) | 0 | 0 | 1/1 | 0 | 0 | 0 | 0 | 0 |
| flagellar biosynthesis protein FlhB – **Tlp118** – peroxiredoxin | 0 | 0 | 1/1 | 0 | 0 | 0 | 0 | 0 |
| tRNA-Gly – **Tlp119** – DUF2874 domain-containing protein | 0 | 0 | 1/1 | 0 | 0 | 0 | 0 | 0 |
| aspartate ammonia lyase – **Tlp120** – hypothetical protein (HP2) | 0 | 0 | 1/1 | 0 | 0 | 0 | 0 | 0 |
| prepilin-type cleavage/methylation domain-containing protein – **Tlp121** – major outer membrane protein | 0 | 0 | 1/1 | 0 | 0 | 0 | 0 | 0 |
| low molecular weight protein-tyrosine-phosphatase – **Tlp122** – hypothetical protein (HP3) | 0 | 0 | 1/1 | 0 | 0 | 0 | 0 | 0 |
| Tlp110 – **Tlp123** – hypothetical protein (HP2) | 0 | 0 | 1/1 | 0 | 0 | 0 | 0 | 0 |
| DUF466 domain-containing protein – **Tlp200** – hypothetical protein (HP5) | 0 | 0 | 0 | 1/1 | 0 | 0 | 0 | 0 |
| DUF2393 domain-containing protein – **Tlp201** – hypothetical protein (HP6) | 0 | 0 | 0 | 1/1 | 0 | 0 | 0 | 0 |
| FkbM family methyltransferase – **Tlp300** - alpha-2-macroglobulin | 0 | 0 | 0 | 0 | 1/1 | 0 | 0 | 0 |
| phosphoribosylglycinamide formyltransferase – **Tlp301** – DUF2920 domain-containing protein | 0 | 0 | 0 | 0 | 1/1 | 0 | 0 | 0 |
| di-trans,poly-cis-decaprenylcistransferase – **Tlp302** – molybdopterin adenylyltransferase | 0 | 0 | 0 | 0 | 1/1 | 0 | 0 | 0 |
| hypothetical protein (HP7) – **Tlp303** – cation ABC transporter substrate-binding protein | 0 | 0 | 0 | 0 | 1/1 | 0 | 0 | 0 |
| DJ-1 family protein – **Tlp304** – hypothetical protein (HP8) | 0 | 0 | 0 | 0 | 1/1 | 0 | 0 | 0 |
| MFS transporter – **Tlp400** – YedE-related selenium metabolism membrane protein | 0 | 0 | 0 | 0 | 0 | 3/3 | 0 | 0 |
| molybdopterin molybdenumtransferase MoeA – **Tlp401** – hypothetical protein (HP9) | 0 | 0 | 0 | 0 | 0 | 4/4 | 0 | 0 |
| anaerobic C4-dicarboxylate transporter – **Tlp402** – disulfide bond formation protein B | 0 | 0 | 0 | 0 | 0 | 5/5 | 0 | 0 |
| iron-sulfur cluster assembly scaffold protein NifU – **Tlp403** – hemerythrin | 0 | 0 | 0 | 0 | 0 | 7/7 | 0 | 0 |
| NadC family protein – **Tlp404** – RluA family pseudouridine synthase | 0 | 0 | 0 | 0 | 0 | 4/6 | 0 | 0 |
| DUF2892 domain-containing protein – **Tlp404** – RluA family pseudouridine synthase | 0 | 0 | 0 | 0 | 0 | 2/6 | 0 | 0 |
| multicopper oxidase family protein – **Tlp405** – Tlp408 | 0 | 0 | 0 | 0 | 0 | 1/1 | 0 | 0 |
| group III hemoglobin – **Tlp406** – bifunctional aconitate hydratase 2/2-methylisocitrate dehydratase | 0 | 0 | 0 | 0 | 0 | 2/2 | 0 | 0 |
| hypothetical protein (HP10) – **Tlp407** – Tlp410 | 0 | 0 | 0 | 0 | 0 | 1/1 | 0 | 0 |
| Tlp405 – **Tlp408** – hypothetical protein (HP11) | 0 | 0 | 0 | 0 | 0 | 1/1 | 0 | 0 |
| MFS transporter – **Tlp409** – YedE-related selenium metabolism membrane protein | 0 | 0 | 0 | 0 | 0 | 2/2 | 0 | 0 |
| Tlp407 – **Tlp410** – tRNA-Arg | 0 | 0 | 0 | 0 | 0 | 1/1 | 0 | 0 |
| hypothetical protein(HP12) – **Tlp500** – periplasmic nitrate reductase precursor | 0 | 0 | 0 | 0 | 0 | 0 | 1/1 | 0 |
| periplasmic nitrate reductase component NapD – **Tlp501** - cytochrome oxidase biogenesis protein Sco1 | 0 | 0 | 0 | 0 | 0 | 0 | 1/1 | 0 |
| hypothetical protein (HP13) – **Tlp502** – anaerobic dimethyl sulfoxide reductase chain A | 0 | 0 | 0 | 0 | 0 | 0 | 1/1 | 0 |
| hypothetical protein (HP14) – **Tlp503** – GTP cyclohydrolase II | 0 | 0 | 0 | 0 | 0 | 0 | 1/1 | 0 |
| tRNA-Asn – **Tlp504** – pyridoxal kinase | 0 | 0 | 0 | 0 | 0 | 0 | 1/1 | 0 |
| MoeB/thiF family protein – **Tlp505** – histidine-binding protein precursor | 0 | 0 | 0 | 0 | 0 | 0 | 1/1 | 0 |
| Autoinducer-2 production protein LuxS – **Tlp506** – hypothetical protein (HP15) | 0 | 0 | 0 | 0 | 0 | 0 | 1/1 | 0 |
| EamA family transporter – **Tlp507** – Tlp508 | 0 | 0 | 0 | 0 | 0 | 0 | 1/1 | 0 |
| Tlp507 – **Tlp508** – nitrate reductase catalytic subunit NapA | 0 | 0 | 0 | 0 | 0 | 0 | 1/1 | 0 |
| ornithine carbamoyltransferase – **Tlp600** – amino acid ABC transporter ATP-binding protein | 0 | 0 | 0 | 0 | 0 | 0 | 0 | 1/1 |
| ABC transporter permease – **Tlp601** – tRNA (cytidine(34)-2'-O)-methyltransferase | 0 | 0 | 0 | 0 | 0 | 0 | 0 | 1/1 |
| hypothetical protein (HP16) – **Tlp602** – hypothetical protein (HP17) | 0 | 0 | 0 | 0 | 0 | 0 | 0 | 1/1 |
| isopenicillin N synthase family oxygenase – **Tlp603** – thiamine-phosphate kinase | 0 | 0 | 0 | 0 | 0 | 0 | 0 | 1/1 |

^1^ Different .gbk files frequently have different annotations with different protein descriptions for the same protein. One annotation has been selected to represent each protein and has been identified with a unique acronym here and in S1 Spreadsheet. Hypothetical proteins have been numbered to allow rapid visual discrimination. The data were manually curated.
