# Supplementary material for: Diversity of transducer-like proteins (Tlps) in Campylobacter
Source: PLoS One. 2019 Mar 25;14(3):e0214228. doi: 10.1371/journal.pone.0214228 (PMC6433261; doi:10.1371/journal.pone.0214228)
Supplement: S3 Table — (DOCX) [file pone.0214228.s013.docx]

|  | **Strain** | **Genome**  **Location (nt)** | **Invertible element (bp)** | **Comments** |
| --- | --- | --- | --- | --- |
|  | *C. jejuni* |  |  |  |
|  | FDAARGOS_421 | 497,377..620,365 | 133,039 | CJIE1 prophage inserted into region |
|  | RM1221 | 145,477..277,520 | 132,044 | CJIE1 prophage inserted into region |
|  | R14 | 146,973..276,823 | 129,851 | CJIE1 variant prophage inserted into region |
|  | 00-1597 | 146,919..247,202 | 100,284 | 5 gene indel |
|  | IA3902 | 146,724..245,536 | 98,813 | 5 gene indel |
|  | BCW_6290 | 146,737..245,529 | 98,793 | integrated TetO transposon |
|  | 35925B2 | 146,436..243,683 | 97,248 | initial Tlp is truncated; IE is not invertible |
|  | F38011 | 149,332..246,141 | 96,810 |  |
|  | FJ3124 | 275,260..371,834 | 96,575 |  |
|  | S3 | 144,760..240,401 | 95,642 |  |
|  | MTVDSCj13 | 145,874..241,443 | 95,570 |  |
|  | YH001 | 145,061..239,298 | 94,238 |  |
|  | 00-6200 | 146,917..241,153 | 94,237 |  |
|  | 00-2425 | 146,953..241,187 | 94,235 |  |
|  | FORC_046 | 156,287..250,429 | 94,143 |  |
|  | FDAARGOS_422 | 393,709..487,847 | 94,139 |  |
|  | CFSAN032806 | 1,447,273..1,541,407 | 94,134 |  |
|  | MTVDSCj07 | 147,422..241,553 | 94,132 |  |
|  | RM1285 | 147,424..241,555 | 94,132 |  |
|  | NCTC11168 | 146,705..240,829 | 94,125 |  |
|  | PT14 | 146,506..240,628 | 94,123 |  |
|  | 32488 | 986,075..1,080,184 | 94,110 |  |
|  | HF5-4A-4 | 180,249..274,225 | 93,977 |  |
|  | T1-21 | 103,474..197,341 | 93,868 |  |
|  | MTVDSCj16 | 180,116..273,756 | 93,641 |  |
|  | RM3196 | 151,965..245,539 | 93,575 |  |
|  | 01-1512 | 146,186..238,833 | 92,648 |  |
|  | 00-0949 | 146,185..238,831 | 92,647 |  |
|  | CG8421 | 148,115..240,669 | 92,554 | final Tlp is a pseudogene, measurement is approximate |
|  | ICDCCJ07001 | 153,740..245,903 | 92,164 |  |
|  | 81116 | 152,565..243,706 | 91,142 |  |
|  | CJM1cam | 151,369..242,500 | 91,132 |  |
|  | M1 | 151,368..242,499 | 91,132 |  |
|  | 14980A | 140,933..231,304 | 90,372 |  |
|  | 81-176 | 157,478..246,514 | 89,037 |  |
|  | 4031 | 143,496..232,076 | 88,581 |  |
|  | CJ677CC012 | 21,272..109,600 | 88,329 |  |
|  | CJ677CC527 | 21,272..108,??? | 88,329 | final Tlp is a pseudogene, measurement is approximate |
|  | *C. coli* |  |  |  |
|  | OR12 | 1,759,305..1,940,976 | 181,672 | two prophage insertions within the invertible element |
|  | RM4661 | 1,601,464..1,732,420 | 130,957 | only one Tlp, not invertible; CJIE1 prophage insertion |
|  | 15-537360 | 213,069..268,603  1,517,224..1.566,831 | 105,143 | two segments in different parts of the genome; 55,535 & 49,608 nt |
|  | YF2105 | 1,587,015..4456 | 105,139 | IE spans the origin of replication; only one Tlp, not invertible |
|  | FB1 | 212,803..268,339  1,516,224..1,565,785 | 105,099 | two segments in different parts of the genome; 55,537 & 49,562 nt |
|  | CF2-75 | 1,587,182..1,689,055 | 101,874 |  |
|  | MG1116 | 1,565,848..5,636 | 104,800 | IE spans the origin of replication; Tlp is degenerate but present |
|  | YH502 | 1,524,184..1,628,268 | 104,085 |  |
|  | CFSAN032805 | 1,474,178..1,578,196 | 104,018 |  |
|  | YH503 | 1,508,721..1,611,893 | 103,173 |  |
|  | BFR-CA-9557 | 416,210..519,378 | 103,169 |  |
|  | YH501 | 1,472,518..1,575686 | 103,169 |  |
|  | 14983A | 1,599,307..1,702,451 | 103,145 |  |
|  | CVM N29710 | 1,478,203..1,581,341 | 103,139 |  |
|  | RM5611 | 1,488,958..1,592,978 | 103,121 |  |
|  | HC2-48 | 1,337,011..1,441,851 | 104,841 |  |
|  | WAA333 | 1,433,280..1,535,786 | 102,507 |  |
|  | CO2-160 | 1,558,912..1,660,828 | 101,917 |  |
|  | BP3181 | 1,595,311..1,003 | 100,049 | IE spans the origin of replication; Tlp is degenerate but present |
|  | BG2108 | 1,595,464..4,456 | 104,497 | IE spans the origin of replication |
|  | RM1875 | 1,613,861..1,711,563 | 97,703 | no Tlp at end of IE, but Tlps at beginning and middle |
|  | 76339 | 152,787..245,244 | 92,458 |  |
|  | ZV1224 | 1,811,044..21,125 | 47,164 | only half of IE, other half not easily identifiable |
