# Supplementary material for: Diversity of transducer-like proteins (Tlps) in Campylobacter
Source: PLoS One. 2019 Mar 25;14(3):e0214228. doi: 10.1371/journal.pone.0214228 (PMC6433261; doi:10.1371/journal.pone.0214228)
Supplement: S4 Table — (DOCX) [file pone.0214228.s014.docx]

|  | with outliers | | no outliers | |
| --- | --- | --- | --- | --- |
|  | *C. jejuni* | *C. coli* | *C. jejuni* | *C. coli* |
| Mean | 96,672.24 | 104,906 | 93,674.6 | 102,652.3 |
| Median | 94,128.5 | 103,169 | 94,123 | 103,169 |
| Standard Deviation | 10,738.97 | 21,511.48 | 2,859.493 | 3,017.194 |
| Standard Error | 1,742.092 | 4,485.453 | 483.3426 | 674.6651 |
| 95% Confidence | 3,529.881 | 9,302.447 | 982.2894 | 1,412.119 |
| 99% Confidence | 4,730.833 | 12,644.37 | 1,318.845 | 1,930.325 |
| Size (no. of strains) | 38 | 23 | 35 | 20 |
| Total | 3,673,545 | 24,12,839 | 3,278,611 | 2,053,046 |
| Minimum | 88,329 | 47,164 | 88,329 | 92,458 |
| Maximum | 133,039 | 181,672 | 100,284 | 105,143 |

Calculated using the Descriptive Statistics tool in SigmaPlot 10.0, Systat Software, Inc.
