# Supplementary material for: Diversity of transducer-like proteins (Tlps) in Campylobacter
Source: PLoS One. 2019 Mar 25;14(3):e0214228. doi: 10.1371/journal.pone.0214228 (PMC6433261; doi:10.1371/journal.pone.0214228)
Supplement: S1 Archive — (ZIP) [file pone.0214228.s015.zip › Readme_MasterBlastR_documentation.docx]

Readme: MasterBlastR.RScript

MasterBlastR was written in R by the Streptococcus and STI Unit, National Microbiology Laboratory, Public Health Agency of Canada, Winnipeg, Manitoba. The script is run interactively through R-Studio. It is the base code that has been used for numerous molecular analysis of assembled whole genome sequencing data to determine antimicrobial resistance, virulence, toxin, MLST and other various molecular determinants. The basic principle is to use a reference gene sequence (referred in our laboratory to as a “wild type” gene) to query the whole genome sequencing assemble contig fasta file using BLAST and parse the BLAST output to get the analogous sequence from the genome of interest. The script may be run using a combination of single or multiple samples or loci, and output files include parsed multi-fasta sequences (for easy use in aligners), tabular csv files containing query results. Lookup files in the form of curated multi-fasta files for each locus is optional, and if present the script will attempt to locate and return information stored in the fasta headers.

To run the script edit the following lines:

SampleNo <- "list"

# for a single sample enter a sample number corresponding to the contig file name or for multiple samples, enter sample number in a list.csv and assign text to the SampleNo <- "list" . The file list.csv must have two columns "SampleNo" and "Variable". SampleNo must match the names of the contig assembly files (without the .fasta) Variable can contain any metadata associated with the a sample (MIC value for example) or may be blank.

LocusID <- "list"

# for a single gene enter the locus name as stored in reference gene fasta file or for multiple genes enter locus name in a loci.csv and assign text to the LocusID <- "list". The file loci.csv file must have one column "Locus_id"

SampList <- "list.csv" # name of sample list file

LocusList <- "loci.csv" # name of locus list file

setwd("C:\\Temp\\masterblastr_test\\")

# A place to run the code and where all the input and output files will be stored.

ContigsDir <- "W:\\Projects\\Streptococcus\\Pneumo\\contigs\\"

# location of assemblies

LkupDir <- "C:\\Temp\\masterblastr_test\\libraries\\"

# location of lookup libraries are multi-fasta files with fasta headers formated with underscores like: ">locus_locusNum_MutationList ">pbp1a_1_SSMK/WT/WT" or ">folP_25_Insert"

RefLocusDir <- "C:\\Temp\\masterblastr_test\\reference\\" # location of reference wild type genes

Blast_evalue <- "10e-50" #sets sensitivity of BLAST match 10e-50 to 10e-150; use 10e-5 for primers
