# Supplementary material for: Diversity of transducer-like proteins (Tlps) in Campylobacter
Source: PLoS One. 2019 Mar 25;14(3):e0214228. doi: 10.1371/journal.pone.0214228 (PMC6433261; doi:10.1371/journal.pone.0214228)
Supplement: S2 Archive — (ZIP) [file pone.0214228.s016.zip › Alignment A.docx]

Alignment A. Alignment of all *C. lari* Tlp protein sequences

CLUSTAL O(1.2.4) multiple sequence alignment 2018/05/11

Clari_LMG11760_Tlp108 MFSSTKKYLLGNFSNKIAFLVCIFVIVLLCVLGVFNYIKSKSNSHTLLVQFQQKVAFDVS 60

Clari_NCTC11845_Tlp108 MLSSLKKYLLGNFSNKIAFLVCIFVVVLLCVLGFFNYVKSKSNSHTLLVQFQQKVAFDVS 60

Clari_RM16701_Tlp108 MLSSLKKYLLGNFSNKIAFLVCIFVVVLLCVLGFFNYVKSKSNSHTLLVQFQQKVAFDVS 60

Clari_CCUG22395_Tlp108 MLSSLKKYLLGNFSNKIAFLVCIFVVVLLCVLGFFNYVKSKSNSHTLLVQFQQKVAFDVS 60

Clari_NCTC11845_Tlp115 ------LFSNLKIGTKIVTVVITII---VLGIGILASIITMQSSNILHTEAD-KLLQTSA 50

Clari_SlaughterBeach_Tlp100 ----------------MILILSSVI---FIGIGILSVTIISKSQEMLNDEAH-KLLLSSA 40

Clari_RM16701_Tlp100 ------MFKNFNLTTKMILILSSVI---FIGIGILSVTIISKSQEMLNDEAH-KLLLSSA 50

Clari_RM2100_Tlp110 -------MKINSIVSKVNILVGILFAATIVIIGSIAYFQTKQSSFEYLRENHNKVLFDVG 53

Clari_NCTC11845_Tlp118 ---------MKSLANKLTFFVFLAIIAILFVANIFNYIEVKRDVQKLINDIQIKTMQDVL 51

Clari_RM16712_Tlp105 -----MGKITKTLTSKLTFFVFLAIIAILFIANAFNYIEVKHDVQKLINDIQVKTMQDVL 55

Clari_RM16701_Tlp105 -----MGKITKTLTGKLTFFAFLAIIAILFIANAFNYAGVKHDVQKLINDIQVKTIQDVL 55

Clari_Slaughter_Beach_Tlp105 -----MGKITKTLTGKLTFFAFLAIIAILFIANAFNYAEVKHDVQKLINDIQVKTMQDVL 55

Clari_CCUG22395_Tlp105 -----MGKITKTLTGKLTFFAFLAIIAILFIANAFNYAEVKHDVQKLINDIQVKTMQDVL 55

Clari_RM16712_Tlp103 --------MFQSITSRLTLVIAIISIIVLVGVNGLSYYNIKEDTYEYLEEIQKKTMLDTA 52

Clari_RM16701_Tlp103 --------MFQNITGRLTLVIAIVSMIVLAVVNGLSYYNAKEDTYDYLEEIQRKTMLDTA 52

Clari_RM1607_Tlp114 ------MFKFNSLSNKLTSIVCFLIVIILAIVNILNYYDSKKSTSYYLEEIQKKTMFDVN 54

Clari_RM2100_Tlp109 ------MFNFRSLSSKLTFIVGLLIIAILITVNIISYYQSKNSTSQYLEEIQVKTMFDVN 54

Clari_LMG11760_Tlp109 ------MFNFRSLSSKLTFIVGLLIIAILITVNIISYYQSKNSTSQYLEEIQVKTMFDVN 54

Clari_NCTC11845_Tlp119 ------MFKFNSLSNKLTMIVCFLIAIILIVVNIINYYESKKTTAYYLEEIQKKTMFDVN 54

Clari_SlaughterBeach_Tlp104 ------MFKFNSLSNKLTMIACFLIAIILIVVNVINYFESKQNTSYYLEEIQKKTMIDVN 54

Clari_RM16701_Tlp104 ------MFKFNSLSNKLTMIACFLIAIILIVVNVINYFESKQNTSYYLEEIQKKTMIDVN 54

Clari_RM16712_Tlp104 ------MFKFNSLSNKITMIACSLIAIILIVANIINYYQSKESTRYYLEEIQKKTMFDVN 54

Clari_CCUG22395_Tlp104 ------MFKFNSLSNKLTMIACFLIAIILIVVNIINYYQSKESTSYYLEEIQKKTMFDVN 54

Clari_RM2100_Tlp112 ---MFFKNSFISVKNKLSYTTGIIVALALFIVAAMAFYSSRENLIINSKNANKDYLLVTT 57

Clari_RM16712_Tlp106 ----MFLSKNMSVKNKLSIIVAAIVALALFVITVMAFYSSREDLILNSKKSNEDYLLVTT 56

Clari_CCUG22395_Tlp121 ----------MSVKNKLSIVVGVIVFLALSIITIMAFVSSRNNLISNSKQANEDYLLITE 50

Clari_RM2100_Tlp123 --------MFSSLKIKLSLLANIFAALSLIVLGIISFIFTKNFLYENELKRQNDILQVSR 52

Clari_LMG11760_Tlp107 --------MLSSLKIKLSLVANLFAALCLIILGILSFYFTKNFLYSNELKRQNDILQVAK 52

Clari_CCUG22395_Tlp120 --------MFNTLKVKLSLMANFFTALSLIILGVLSFYFTKTYLYDNELKRQNDILQVAR 52

Clari_RM2100_Tlp111 ----------MGIKLKISLIANIIAIVCLISLGITTFYFVKDALLKNTIEAQTNYLKSSK 50

Clari_NCTC11845_Tlp117 ---------MKSIKLKLSLIANIMAIFCLIVLGVISFIFTKKALNYEVIKAETNYVRAAE 51

Clari_RM16712_Tlp101 ---------MKSIKIKISLISNIIAIICLVILGVISFIFTEKALNYEVVKAETNYVKAAE 51

Clari_CCUG22395_Tlp122 ---------MKSIKLKISLIANIIAIVCLIILSIVSFIFTKKALNYEVVKAETNYVRTAE 51

Clari_LMG11760_Tlp101 ---------MKSIKLKISLIANVIAIICLIVLGVISFIFTKKALNHEVVKAETNYVKVAE 51

Clari_SlaughterBeach_Tlp101 ---------MKSIKLKISLIANIIAIICLIILGIISYIFTKKALNHEVVNAETNYVKVAE 51

Clari_SlaughterBeach_Tlp102 ---------MKSIKLKISLIANIIAIICLIILGIISYIFTKKALNHEVVNAETNYVKVAE 51

Clari_RM16701_Tlp101 ---------MKSIKLKISLIANVIAIICLIVLGIISFIFTKKALNHEVVKAETNYVKVAE 51

Clari_RM2100_TLp113 ---------MKSIKLKVAMIANIMAVICLLILGIVTFIFVKQSLFDEIVNSEKNRLISTN 51

Clari_NCTC11845_Tlp116 ---------MKSIKLKVSLIANTIAIFCLIILGVITFIFVKQALFDEIVKSEQNRLVSTK 51

: . . . .

Clari_LMG11760_Tlp108 KRFDLYASDRRN-------IINSLTKYIK-------ENKHNLNSKQYTSLLKSIGDSLGF 106

Clari_NCTC11845_Tlp108 KRFDLYASDRRN-------VINSLTKYIK-------ENKNNLTPEQYTSLLKSIGDSLGF 106

Clari_RM16701_Tlp108 KRFDLYASDRRN-------VINSLTKYIK-------ENKNNLTPEQYTSLLKSIGDSLGF 106

Clari_CCUG22395_Tlp108 KRFDLYASDRRN-------VINSLTKYIK-------ENKNNLTPEQYTSLLKSIGDSLGF 106

Clari_NCTC11845_Tlp115 F---RYSNIIRGATESVHSTLLSTESSIDQIL----DTQTSLEQNRIQDILEGAVDSNSW 103

Clari_SlaughterBeach_Tlp100 N---RYSNGIQAITQNAYSTLETAQGVIKNFA----NKDNNLDIEDLKILISSMLDSNSW 93

Clari_RM16701_Tlp100 N---RYSNGIQAITQNAYSTLETAQGVIKNFA----NKDNNLDIEDLKILISSMLDSNSW 103

Clari_RM2100_Tlp110 YIFNTYEADNQS-------AIQNLANFAVE------NHILDN-EQEIFNALKLTQEYVGF 99

Clari_NCTC11845_Tlp118 KSFDDYTASRSD-------AIKAVAAEIK-------KNPNTS-LEEIYTMVKVAKEASRF 96

Clari_RM16712_Tlp105 KSFDDYTTSRSD-------AIKAVAAEIQ-------KNPNAS-LEEIYTMVKVAKESSRF 100

Clari_RM16701_Tlp105 KSFDDYTASRSD-------TIKAVAAEIQ-------KNPNAS-LEEIYTMVKVAKESSRF 100

Clari_Slaughter_Beach_Tlp105 KSFDDYTASRSD-------AIKAVAAEIQ-------KNPNAS-LEEIYTMVKVAKESSRF 100

Clari_CCUG22395_Tlp105 KSFDDYTASRSD-------AIKAVAAEIQ-------KNPNAS-LEEIYTMVKVAKESSRF 100

Clari_RM16712_Tlp103 EVFFIYSNAKRK-------AVSTLAEEIV-------KQDFSN-DENIYNFLEAFKKANNF 97

Clari_RM16701_Tlp103 EVFSIYSNAKRK-------AISTLAEEIV-------KQDFSD-DGNIYKILEAFKKANNF 97

Clari_RM1607_Tlp114 YMYSSYSNSKRN-------IIESLAYSLSA------IAYNSS-DREIFSVLDTAKRSGGF 100

Clari_RM2100_Tlp109 KAYEIYGTSKRT-------AIDSIVKFME-------KNPHPD-INELFDILETIRYSAGY 99

Clari_LMG11760_Tlp109 KAYEIYGTSKRT-------AIDSIVKFME-------KNPHPD-INELFDILETIRYSAGY 99

Clari_NCTC11845_Tlp119 EAYKIYSSSKRS-------AILSIVNFIE-------KNPNPS-TEELFDILETIRSSADF 99

Clari_SlaughterBeach_Tlp104 KAYEIYSNSKRT-------AISSIVSFME-------KNPNVE-TREIFDILATIKESAGF 99

Clari_RM16701_Tlp104 KAYEIYSNSKRT-------AISSIVSFME-------KNPNVE-TREIFDILATIKESAGF 99

Clari_RM16712_Tlp104 SAYNIYSGAKRE-------AISSIVKFVE-------KNPKVD-TAEIFDMLETIKEAAGF 99

Clari_CCUG22395_Tlp104 SAYNIYSGAKRD-------AIQSIVKFVE-------KNPRVD-SAELFDVLETVKEAAGF 99

Clari_RM2100_Tlp112 AQVETYIESYVE-------ILLSIKKYIDSLPKYQTENFDKL-GEYFAKDLKVFKDGSNT 109

Clari_RM16712_Tlp106 TQVEGYVENYVD-------ILLAIKKHIDQLPEYQIKNFDTL-SEYFAKDLKIFKDGSNT 108

Clari_CCUG22395_Tlp121 TQVEGYVDGYID-------ILLAIKKYIDSLGKNQVLNFDSM-EQMLGDDLKIFKDGSNT 102

Clari_RM2100_Tlp123 ISLETFRENNIK-------LINHLEESVLELPYEKLNSQEDL-IENLGQMLKSYRKASGV 104

Clari_LMG11760_Tlp107 ISLETFRDINTN-------LITNLEKSILEHPYEKLNSEEAL-IENIGSTLKSYRKASGV 104

Clari_CCUG22395_Tlp120 TSLETFRKHNSD-------LILNLEKTILEFPYEKLNSEQAL-IDNVGSILKSYRKASGV 104

Clari_RM2100_Tlp111 DLMNDFKTSTER-------SLQNLSRAILKHPLYKLKDEESV-LASLAVELKAFRDSGGF 102

Clari_NCTC11845_Tlp117 KSMRDFKNTNIN-------SLERLSQAIARFSYEELDTQEKL-MHNTGKLLKSFRDAGNY 103

Clari_RM16712_Tlp101 KSMRDFKNTHSH-------ALKQLSQTITRLSYQELNTQEKL-MNNTGELLKTVRDMNNY 103

Clari_CCUG22395_Tlp122 KSMRDFKNLNTH-------SLEKLSQAILKLPYDALNTQDKL-MQNTGNLLKAVRDMNSY 103

Clari_LMG11760_Tlp101 KSMRDFKNLHTH-------SLEQLSQAILKLPYNELNTQEKL-MENTGSLLKTVRDINSY 103

Clari_SlaughterBeach_Tlp101 KSMRDFKSLHTH-------SLEQLSQAILRLPYNELNTQEKL-MENTGDLLKTVRDINSY 103

Clari_SlaughterBeach_Tlp102 KSMRDFKSLHTH-------SLEQLSQAILRLPYNELNTQEKL-MENTGDLLKTVRDINSY 103

Clari_RM16701_Tlp101 KSMRDFKSLHTH-------SLEQLSQAILRLPYNDLNTQEKL-MENTGGLLKTVRDINSY 103

Clari_RM2100_TLp113 NLVENFRESTSN-------SLLKLSETILRNPYSNLNSQEAL-AQNVGVQLKAFRDAGNY 103

Clari_NCTC11845_Tlp116 SLMEEFRENTTT-------TLKKLSETILRHPYSELNTQESL-IQNVSSQLRAFRDAGGF 103

: : :

Clari_LMG11760_Tlp108 -DLTYVGFE---------------DGSMFRSNGN---------------NQTPESGYDPR 135

Clari_NCTC11845_Tlp108 -DLTYVGFE---------------DGKIFRSNGN---------------NQTPESGYDPR 135

Clari_RM16701_Tlp108 -DLTYVGFE---------------DGKIFRSNGN---------------NQTPESGYDPR 135

Clari_CCUG22395_Tlp108 -DLTYVGFE---------------DGKIFRSNGN---------------NQTPESGYDPR 135

Clari_NCTC11845_Tlp115 INYIYIHIIDISKFNNIDPTLLTDSGQFLMLINDTDLKNK-----GGI-KLIQADDRILN 157

Clari_SlaughterBeach_Tlp100 TYFAYIHLN---QYHDNNPLNLTPSGKFLLLAKDENPQQK-----GSI-KFIQAEEVILQ 144

Clari_RM16701_Tlp100 TYFAYIHLN---QYHSNNPLNLTPSGKFLLLAKDENPQQK-----GSI-NFIQAEEIIMQ 154

Clari_RM2100_Tlp110 -EIVFLAT---------------EDGITYDSTGV-------------K--KTLNNGFDGR 128

Clari_NCTC11845_Tlp118 -DVLYVGLA--------------KNGAMIRSNGN-------------H--QMPSDGYDPR 126

Clari_RM16712_Tlp105 -DVLYVGLA--------------KNGAMIRSNGN-------------H--QMPSDGYDPR 130

Clari_RM16701_Tlp105 -DVLYVGLA--------------KNGAMIRSNGN-------------H--QMPSDGYDPR 130

Clari_Slaughter_Beach_Tlp105 -DVLYVGLA--------------KNGAMIRSNGN-------------H--QMPSDGYDPR 130

Clari_CCUG22395_Tlp105 -DVLYVGLA--------------KNGAMIRSNGN-------------H--QMPSDGYDPR 130

Clari_RM16712_Tlp103 -DIIYFALE--------------ENGKHYKSDHT-------------Y--LDKSKGFDVK 127

Clari_RM16701_Tlp103 -DIIYFALE--------------ENGKHYKSDHT-------------Y--LDKSKGFDVK 127

Clari_RM1607_Tlp114 -DTVHFGLE--------------DSGKDYQIDTK-------------FNLHSDPSKFDPR 132

Clari_RM2100_Tlp109 -DVTYIGFE--------------EDGKLYQSNKI-------------I-RSPEQTGFDAR 130

Clari_LMG11760_Tlp109 -DVTYIGFE--------------EDGKLYQSNKI-------------I-RSPEQTGFDAR 130

Clari_NCTC11845_Tlp119 -DVTYVGFE--------------KDGKLYQSNKI-------------I-RSPEASGFDAR 130

Clari_SlaughterBeach_Tlp104 -DVVYLGFD--------------INGKLYQSNRI-------------V-RSPEETGFDAR 130

Clari_RM16701_Tlp104 -DVVYLGFD--------------INGKLYQSNKI-------------V-RSPEETGFDAR 130

Clari_RM16712_Tlp104 -NVVYIGFN--------------DDGKLYQSNRI-------------I-RSPEETGFDAR 130

Clari_CCUG22395_Tlp104 -NVVYLGFN--------------NDGKLYQSNRI-------------I-RSPEETGFDAR 130

Clari_RM2100_Tlp112 -LAVYLGFP--------------D-GTMLVSDAESDKKGIPFRKRGGGISHYDDPQYNAT 153

Clari_RM16712_Tlp106 -LAVYLGFP--------------D-GTMLVSDADSDKKEIPFRKRGGGIAHYDDPKYNAT 152

Clari_CCUG22395_Tlp121 -LAVYVGFP--------------D-GTMLVSDTVSDKKGVNFRKRGGGISSYDDPSYDAT 146

Clari_RM2100_Tlp123 -LATFIGLD--------------N-GENIVSDNSSDNKNTNVVIYGK------AINYDTR 142

Clari_LMG11760_Tlp107 -LAVFIGLD--------------N-GENIVSDNNSDQKNRNVGIYGK------AMNYDTR 142

Clari_CCUG22395_Tlp120 -LSAFISLD--------------N-GENLVSNDTSDKNNKNIEIYGQ------NINYDAR 142

Clari_RM2100_Tlp111 -LGVYVGMP--------------S-GELITSDPRADEKQLNAFIFGR------AQNYNAT 140

Clari_NCTC11845_Tlp117 -LAVYIAQP--------------N-GELIVSDPDSDSKGLEYGTYGK------ADNYDAT 141

Clari_RM16712_Tlp101 -LAVYIAQP--------------N-GELIVSDPDSDSKGLNYGIYGK------ADNYDAT 141

Clari_CCUG22395_Tlp122 -LAVYIAQP--------------N-GELIVSDPDSDSKGLDYGIYGK------ADNYDAT 141

Clari_LMG11760_Tlp101 -LAVYIAQS--------------N-GELIVSDPDSDSKGLDYGIYGK------ADNYDAT 141

Clari_SlaughterBeach_Tlp101 -LAVYIAQS--------------N-GELIVSDPDSDSKGLDYGIYGK------ADNYDAT 141

Clari_SlaughterBeach_Tlp102 -LAVYIAQS--------------N-GELIVSDPDSDSKGLDYGIYGK------ADNYDAT 141

Clari_RM16701_Tlp101 -LAVYIAQS--------------N-GELIVSDPDSDSKGLDYGIYGK------ADNYDAT 141

Clari_RM2100_TLp113 -LAVYIAQP--------------D-GELVVSDPDSDSKNIDYGFYGK------ADGYDAR 141

Clari_NCTC11845_Tlp116 -LTVYLAQP--------------D-GEVILTNAESDKLNQDIIIFGK------KQNFDAR 141

.. *

Clari_LMG11760_Tlp108 TRGWYKEAKE-KKELIVTEPYISSS-MKKPTISYANPIIENG-EVIGVVAADYDLKKFSE 192

Clari_NCTC11845_Tlp108 TRGWYKEAKE-KRELVVTEPYISSS-MKKPTISYASPIMEND-KVIGVVAADYDLKKFSE 192

Clari_RM16701_Tlp108 ARGWYKEAKE-KRELVVTEPYISSS-MKKPTISYASPIMENG-KVIGVVAADYDLKKFSE 192

Clari_CCUG22395_Tlp108 ARGWYKEAKE-KRELVVTEPYISSS-MKKPTISYASPIMENG-KVIGVVAADYDLKKFSE 192

Clari_NCTC11845_Tlp115 QRSVKAALEKQEEGVGRPQNFVINN-EEILAYNIAIPIT-RNGKLLGVIGALGGLNTLQE 215

Clari_SlaughterBeach_Tlp100 QNSLIKALQTKQPAVGRPRDYSING-EKLYLVNIVLPIFGKNNETIGAIGMLVRIDLLRE 203

Clari_RM16701_Tlp100 QNSLIKALHTKQPAVGRPRDYSING-EKLYLVNIVLPIFGKNNETIGAIGMLVRVDLLRE 213

Clari_RM2100_Tlp110 SRSWYIGAKK-NMGLYTSDPYKSVT-SGIEGIAYSAPLIING-KFKGVVAGVYSLEQYSA 185

Clari_NCTC11845_Tlp118 TRTWYTSVASGEDKVVISKPYMAPSLK-APSLAFSYPIIIDG-KFMGAVGGNYDLNTFSD 184

Clari_RM16712_Tlp105 TRTWYTSVTSGEDKVVISKPYMAPSLK-APSLAFSYPIVVNG-KFIGAVGGNYDLNTFSD 188

Clari_RM16701_Tlp105 TRTWYTSVSSGENKVVISKPYMAPSLK-APSLAFSYPIVVDG-KFIGAVGGNYDLNTFSD 188

Clari_Slaughter_Beach_Tlp105 TRTWYTSVSSGENKVVISKPYMAPSLK-APSLAFSYPIVVDG-KFIGAVGGNYDLNTFSD 188

Clari_CCUG22395_Tlp105 TRTWYTSVSSGENKVVISKPYMAPSLK-APSLAFSYPIVVDG-KFIGAVGGNYDLNTFSD 188

Clari_RM16712_Tlp103 TRAWYINAKK-EGGLIVSDPYSSFA-DGKMKIAYAMPVFKND-KFIGVVGGDYDLERFSK 184

Clari_RM16701_Tlp103 TRAWYINAKK-EGKLIVSDPYSSFA-DGKMKIAYAVPVFKNN-QFIGVVGGDYDLERFSK 184

Clari_RM1607_Tlp114 TRPWYKDAKT-AGKLIVTDPYKSIVLNGQVVVTYSIPVFDNSKKFIGVVSGVYNLNTFSK 191

Clari_RM2100_Tlp109 TRPWYQEAKT-TGTLVVSDPYKSIE-DGSITISYTAPIYVNG-KLLAVVGGDYNLHTFAK 187

Clari_LMG11760_Tlp109 TRPWYQEAKA-TGTLVVSDPYKSIE-DGSITISYTAPIYVNG-KLLAVIGGDYNLHTFAK 187

Clari_NCTC11845_Tlp119 TRPWYQESQQ-AKTLTVSDPYKSIE-DDSITISYTAPIYNNG-KLIAVVGGDYNLEKFAK 187

Clari_SlaughterBeach_Tlp104 TRSWYQEAKA-AGKLVVSDPYKSVE-DDAVTISYTAPIFVNG-KLLAVVGGDYNLHTFSK 187

Clari_RM16701_Tlp104 TRSWYQEAKA-AGKLVVSDPYKSVE-DDAVTISYTAPIFVNG-KLLAVVGGDYNLHTFSK 187

Clari_RM16712_Tlp104 TRSWYQEAKA-AGKLVVSDPYKSIE-DGSITVSYTAPILVNG-KLLAVVGGDYDLERFSK 187

Clari_CCUG22395_Tlp104 TRSWYQEAKT-AGKLVVSDPYKSIE-DDSITVSYTAPIFVNG-KLLAVVGGDYDLERFSK 187

Clari_RM2100_Tlp112 TRDWYKGALK-NKGVFVSDVYEDSV-TKFPSFTYSVPIEKNG-KLVAVLGIDLLLTSLQK 210

Clari_RM16712_Tlp106 SRDWYKGALK-NDGIYISDVYEDSV-TKLPSFTYSVPIKKNG-KLVAVLGIDLLLTSLQK 209

Clari_CCUG22395_Tlp121 SRDWYKGAIA-NDGVFISDVYEDSV-TKLPSFTYSTPIKKNG-KLIGVLGVDLLLTSLQK 203

Clari_RM2100_Tlp123 TREWYIEARK-TNKVFITFPYIDKA-TNQYVITYTKSISKDG-KFIGVIGVDIPIAIFKK 199

Clari_LMG11760_Tlp107 TRSWFIEAKK-TNNVFITTPYIDKA-TNQYVITYTKAIYKDN-RFIGVIGIDIPIKDLQK 199

Clari_CCUG22395_Tlp120 TRPWYIGAKE-NKDIFITSPYIDKA-TNQYVITYTKSIYKNG-NFVGIIGVDIPVKELQE 199

Clari_RM2100_Tlp111 TRGWYRGAKE-KNGMYVSDVYVDAA-TNLPCLTYALPLYKDG-QFIGVAGIDVLVEELQK 197

Clari_NCTC11845_Tlp117 TREFYIEAKK-KNGLYITAAYIDAT-TGLPCFTYAMPLIKDG-KFIGVLAIDVLVKDLQT 198

Clari_RM16712_Tlp101 TREFYIEAKK-KNGLYITPSYIDVT-TGLPCFTYSMPLVKDG-KFIGILAIDVLVKDLQT 198

Clari_CCUG22395_Tlp122 TREFYIEAKK-KNGLYITPSYIDVT-TGFPCFTYAMPLIKDG-KFLGILAIDVLVKDLQN 198

Clari_LMG11760_Tlp101 TREFYIETRK-KNGLYITAAYIDAT-TGLPCFTYAMPLIKDG-KFIGVLAIDVLVKDLQE 198

Clari_SlaughterBeach_Tlp101 TREFYIEARK-KNGLYITAAYIDAT-TGLPCFTYAMPLIKDG-KFIGVLAIDVLVKDLQE 198

Clari_SlaughterBeach_Tlp102 TREFYIEARK-KNGLYITAAYIDAT-TGLPCFTYAMPLIKDG-KFIGVLAIDVLVKDLQE 198

Clari_RM16701_Tlp101 TREFYIEARK-KNGLYITPSYIDAT-TGLPCFTYAMPLIKDG-KFIGVLAIDVLVKDLQE 198

Clari_RM2100_TLp113 TREFYIEARK-KNGLFITASYIDAT-TGLPCFTYAMPLNKDG-KFVGILAIDVLVKDLIE 198

Clari_NCTC11845_Tlp116 TREWYQEAK--AKGIFVTPAYIDTT-TNLPCFTYAMSLSKDG-KFIGVLAIDVLVKDLED 197

. : : : . . . . :

Clari_LMG11760_Tlp108 EVLAIGKTPYSHAAVLAH-DGTYLFHTDPSKILTSTN-TSKDIVA-----------YYLK 239

Clari_NCTC11845_Tlp108 EVLAIGKTPYSHAAVLAH-DGTYLFHTDSSRILTSTE-VSKDIIS-----------SYFK 239

Clari_RM16701_Tlp108 EVLAIGKTPYSHAAVLAH-DGTYLFHTDSSRILTSTD-ISKDIIS-----------SYFK 239

Clari_CCUG22395_Tlp108 EVLAIGKTPYSHAAVLAH-DGTYLFHTDSSRILTSTD-ISKDIIS-----------SYFK 239

Clari_NCTC11845_Tlp115 ELTNPERS----V-----------FKNDQRLLLGANGLIAVSPATDFIGKNITEINPHAS 260

Clari_SlaughterBeach_Tlp100 ELNDPNKS----L-----------FANDQRLLISSDGLIISSPKAEYIGKIITEINPHPS 248

Clari_RM16701_Tlp100 ELNDPNKS----L-----------FANDQRLLISSDGLIISSPKAEYIGKIITEINPHPS 258

Clari_RM2100_Tlp110 DALEVGKTENSFVAVYSQ-DGTTMFHQDPKLIL-TKTVLG-QNIA----------KAITE 232

Clari_NCTC11845_Tlp118 NVLAMGKSQSGYTVVLDD-EGTILFHESSKDLL-TKTNLS-QNIV----------KTYLA 231

Clari_RM16712_Tlp105 NVLAMGRSQSGYTVVLDD-EGTVLFHESSKALL-TKDDLS-QNIV----------KAYLS 235

Clari_RM16701_Tlp105 NVLAMGRSQSGYTVVLDD-EGTVLFHESSKALL-TKDDLS-QNIV----------KAYLS 235

Clari_Slaughter_Beach_Tlp105 NVLAMGRSQSGYTVVLDD-EGTVLFHESSKALL-TKDDLS-QNIV----------KAYLS 235

Clari_CCUG22395_Tlp105 NVLAMGRSQSGYTVVLDD-EGTVLFHESSKALL-TKDDLS-QNIV----------KAYLS 235

Clari_RM16712_Tlp103 DVLSVGKSSQAYTAIYDI-EGNAFFHPEVEKIG-KKDELS-ENIS----------VYMRA 231

Clari_RM16701_Tlp103 DVLSVGKSSQAYTAIYDI-EGNAFFHPEIEKIG-KKDKFS-ENIS----------AYMKA 231

Clari_RM1607_Tlp114 DVLAIGHSESSYAGVYDK-EGVIVFHEDKDRML-TKNDLS-INIA----------NAVKA 238

Clari_RM2100_Tlp109 DVLILGHSQSSYAAVYDK-EGQIIFHENKDLML-TKNDLS-INIA----------NAAKA 234

Clari_LMG11760_Tlp109 DVLILGHSQSSYAAVYDK-EGQIIFHENKDLML-TKNDLS-INIA----------NAAKA 234

Clari_NCTC11845_Tlp119 DVLVIGHSNSSYAAVYDK-EGVIIFHEEKDRML-TKNDLS-INIA----------NAVKA 234

Clari_SlaughterBeach_Tlp104 DVLAIGHSESSYAGVYDK-EGVIVFHEDKDRML-TKNDLS-INIA----------NAVKA 234

Clari_RM16701_Tlp104 DVLAIGHSESSYAGVYDK-EGVIVFHEDKDRML-TKNDLS-INIA----------NAVKA 234

Clari_RM16712_Tlp104 DVLVMGHSQSSYAAVYDKNDGSIIFHEDKDRML-TKNDLS-INIA----------NAVKS 235

Clari_CCUG22395_Tlp104 DVLVMGHSQSSYAAVYDKNDGSIIFHEDKDRML-TKNDLS-INIA----------NAVKA 235

Clari_RM2100_Tlp112 TFEKLP----GSVFVFDN-TSSIPFASNDKSLI-LKSYPNIDEIK----------NHHKI 254

Clari_RM16712_Tlp106 TFDKLP----GNVFVFDL-ASSIPFASNDKTLI-MKDYPSINEIK----------KYHKM 253

Clari_CCUG22395_Tlp121 TFEKLP----GNVFVFDT-ASSIPFASNNKSLI-LTQHPSIEEIK----------KYHQQ 247

Clari_RM2100_Tlp123 TLKI-N----QEIAFFNQ-NEKV-FVAKNKQLLDPS--VDHSPVI----------NAHKQ 240

Clari_LMG11760_Tlp107 DFESMP----GNSFLFDH-NGKV-FVAKNKQLLDPS--VDHTPVL----------NAYKK 241

Clari_CCUG22395_Tlp120 NFENMP----GNSFLFDE-NGKI-FVAKNKQLLDSS--VDHGPVL----------NAHKQ 241

Clari_RM2100_Tlp111 KIERIP----GDVFIAND-SNYA-FVSSSKVYLGKVK--NVETAL----------GKYKE 239

Clari_NCTC11845_Tlp117 EFSELP----GRTFVFDH-EFNV-FAATDETLVSKEKNPDIITVA----------KAYEQ 242

Clari_RM16712_Tlp101 EFSELP----GRTFVFDQ-AYTV-FASTDKSLISAEQNPDIITVA----------KAYEK 242

Clari_CCUG22395_Tlp122 EFSELP----GRTFVFDQ-AYTV-FASTDKSLIGGEKNPDIVTVA----------KAYEK 242

Clari_LMG11760_Tlp101 KFNELP----GRTFVFDH-AYTV-FASTDKSLVGQEQNPDIVTVA----------KAYEN 242

Clari_SlaughterBeach_Tlp101 KFNELP----GRTFVFDH-AYTV-FASTDKSLVGGEQNPDIVTVA----------KAYEN 242

Clari_SlaughterBeach_Tlp102 KFNELP----GRTFVFDH-AYTV-FASTDKSLVGGEQNPDIVTVA----------KAYEN 242

Clari_RM16701_Tlp101 KFNELP----GRTFVFDQ-AYTV-FASTDKSLVGGEQNPDIVTVA----------KAYEN 242

Clari_RM2100_TLp113 NLKQMP----GDSFVYDK-NRYA-FASTHKNYTGN--HPNISTIA----------DAFSK 240

Clari_NCTC11845_Tlp116 GLKQMP----GASFVFDK-NNFA-FASTSKNYIAN--DPNVSIVA----------EAFSK 239

*

Clari_LMG11760_Tlp108 ---TPEGVNRSLSKDIFKIQTKENETRALICN-----GGINPKYVICSIADYDFYNDAAK 291

Clari_NCTC11845_Tlp108 ---TPEGANKTLSKDIFKVQTKEEGTKALICN-----GSINPKYTICSIADYDFYSDAAK 291

Clari_RM16701_Tlp108 ---TPEGVNKTLSKDIFKVQTMEEGTKALICN-----GSINPKYTICSIADYDFYSDAAK 291

Clari_CCUG22395_Tlp108 ---TPEGANKTLSKDIFKVQTMEEGTKALICN-----GSINPKYTICSIADYDFYSDAAK 291

Clari_NCTC11845_Tlp115 AKTLIELQKNQINTLFDFTPASTGNNNRAAIANFNLWDGANDYWSIVTMAPVESIQMPIT 320

Clari_SlaughterBeach_Tlp100 AKTILDMQSTKTNGLFTFIPASTNEENLAQLVNFDLWEGSNDHWSVVTIAPKKSVEKPAD 308

Clari_RM16701_Tlp100 AKTILDMQSTKTNGLFTFIPASTNEENLAQLVNFDLWEGSNNHWSVVTIAPKKSVEKPAD 318

Clari_RM2100_Tlp110 ---DPSLLDPENIDTLFYAKDDKGVTQAVLCDKTP-N----PNINICAMVENDTYTKASD 284

Clari_NCTC11845_Tlp118 ---TPDGKDGKLSSEPMLIDDDNAPRKAVICQES--S----IGYNVCVIADEKIYNEPVN 282

Clari_RM16712_Tlp105 ---TPEGKAGQLSNKPMIIEDGSAPRKAVICQES--S----IGYNVCVIADEKIYKDPIN 286

Clari_RM16701_Tlp105 ---TPEGKAGQLSSEPMIIEDGSAPRKAVICQES--S----TGYNVCVIADEKIYKDPVN 286

Clari_Slaughter_Beach_Tlp105 ---TPEGKAGQLSSEPMIIEDGSAPRKAVICQES--S----TGYNVCVIADEKIYKDPVN 286

Clari_CCUG22395_Tlp105 ---TPEGKAGQLSSEPMIIEDGSAPRKAVICQES--S----TGYNVCVIADEKIYKDPVN 286

Clari_RM16712_Tlp103 ---NPHFLELNNENAIAYIPNEQGIMEAIMCTNSF-N----SKYKVCTVTKEKVYSEKVN 283

Clari_RM16701_Tlp103 ---NPQFLDPKNESAIAYIPNEQGVMEAIMCTNSF-S----SKYKVCTVTKEKVYSEKAN 283

Clari_RM1607_Tlp114 ---NPDLIDPTKQETLFYAKDDQGKTQVVTCNQAL-N----PKYMVCSITDESVYTDAVN 290

Clari_RM2100_Tlp109 ---NPDLIDPSKEDSLFYAKDGNNKIQVVTCVQAL-N----PKYMVCSITDESVYSDAVN 286

Clari_LMG11760_Tlp109 ---NPDLIDPSKEDSLFYAKDGNDKTQVVTCVQAL-N----PKYMVCSITDESVYSDAVN 286

Clari_NCTC11845_Tlp119 ---NPDLINPNKQETLFYAKDQQGKTQVVTCNQSL-N----DKYIVCSITDESIYTDAVN 286

Clari_SlaughterBeach_Tlp104 ---NPDLIDPTKQETLFYAKDDQGKTQVVTCNQAL-N----PKYMVCSITDESVYTDAVN 286

Clari_RM16701_Tlp104 ---NPDLIDPTKQETLFYAKDDQGKTQVVTCNQAL-N----PKYMVCSITDESVYTDAVN 286

Clari_RM16712_Tlp104 ---DPDLIDPNKEESLFYAKDGAGKTQVVTCNQAL-N----PKYVVCSITDESVYTDAVN 287

Clari_CCUG22395_Tlp104 ---DPDLIDPTKQETLFYAKDDQGKTQVATCNQTV-N----PKYMVCSITDESVYTDAVN 287

Clari_RM2100_Tlp112 ---V------GDYKTFEYTGVDSGEKRFGVCANIN-NSNAHVSYVACAIQKQDDLDRLVI 304

Clari_RM16712_Tlp106 ---V------GDYKNFEYTGISSNEKRFGICANIN-NSKARINYVACAIQKQDSLDKLVI 303

Clari_CCUG22395_Tlp121 ---F------GDYKTFEYISVDTQEERFGICANID-NSKAKVSYIACATQKQDELEMLVL 297

Clari_RM2100_Tlp123 ---N------GDYKFFEYG-LK-GQERLGICANIY-------DYRVCSTESAEIINKPIM 282

Clari_LMG11760_Tlp107 ---N------GDYTFFEYG-LK-NKERLGICAQIS-------SYLVCSTESADIINEPIF 283

Clari_CCUG22395_Tlp120 ---N------GDYSFFEYG-LK-GKERLGICAKIS-------SYLVCSTESADVINEPIF 283

Clari_RM2100_Tlp111 ---F------GDFKPFMFTGQN-GNDRLGICSKLD-------KYSACIVTKMNLIEESSE 282

Clari_NCTC11845_Tlp117 ---A------GDYNIFNYTTQK-GKDRFGICVKID-------SYTTCAGEDIEVIETPAL 285

Clari_RM16712_Tlp101 ---A------GDYNIFSYSTKN-GQDRFGICVKID-------NYTTCAGENIEVIKAPAL 285

Clari_CCUG22395_Tlp122 ---A------GNYNIFNYTTQN-GQDRFGICVKID-------DYTTCAGENIEVIEAPAL 285

Clari_LMG11760_Tlp101 ---T------GDYNIFNYTTQN-GGDRFGICVKID-------GYTTCAGENVEVIETPAL 285

Clari_SlaughterBeach_Tlp101 ---A------GNYNIFNYTTQN-GGDRFGICVKID-------GYTTCAGEDVEVIETPAL 285

Clari_SlaughterBeach_Tlp102 ---A------GNYNIFNYTTQN-GGDRFGICVKID-------GYTTCAGEDVEVIETPAL 285

Clari_RM16701_Tlp101 ---A------GDYNIFNYTTQN-GGDRFGICVKID-------GYTTCAGENVEVIETPAL 285

Clari_RM2100_TLp113 ---T------KNNEPFFYTSAE-GNERLALCNNSN-------DYTVCNVAYVDTINNSSE 283

Clari_NCTC11845_Tlp116 ---T------KDGEPFYYTSKE-GSERLAMCDRVN-------GYTICNMTYIDTIDQSSE 282

. .

Clari_LMG11760_Tlp108 QTLMEQIIISLIAIFITLIFIRMIISY-NLKPIAIISSGLHNFFNYLNHKDSHSYP-IKL 349

Clari_NCTC11845_Tlp108 QTLMEQIIISLIAIFITLIFIRMIISY-NLKPIAIISSGLHNFFNYLNHKDAHSHP-IKL 349

Clari_RM16701_Tlp108 QTLMEQIIISLIAIFITLIFIRMIISY-NLKPIAIISSGLHNFFNYLNHKDAHSHP-IKL 349

Clari_CCUG22395_Tlp108 QTLMEQIIISLIAIFITLIFIRMIISY-NLKPIAIISSGLHNFFNYLNHKDAHSHP-IKL 349

Clari_NCTC11845_Tlp115 KLAATIAMVSLFVIFAIALIVFFYINKAVSSRIVNLQNNLLYFFKFLNHEVKDTILSKDI 380

Clari_SlaughterBeach_Tlp100 SLAFIIFAISTIVLFIIISVIYFYVKKSVVGAIHKLQTGLNSFFDFINHKTKDSAM-IDV 367

Clari_RM16701_Tlp100 SLAFIIFAISVIVLFIIISVIYFYVKKSIVGTIHKLQTGLNSFFDFINHKTKDSAM-IDV 377

Clari_RM2100_Tlp110 LALKTQLIVGFIALIIVLVLIKFFASY-LLNPIFIIQTGLNSFFDFINHKTKDSAM-INV 342

Clari_NCTC11845_Tlp118 KALVNQIIIGIISLIIALIIVRFMISY-NLSPLQAIQTGLNSFFDFINHKTKDSAM-INV 340

Clari_RM16712_Tlp105 EALIKQIIIGAISLVIALIVIRFMINY-NLSPLKKIQTGLNSFFDFINYKTKDSAM-IDV 344

Clari_RM16701_Tlp105 KALVKQIIIGAISLVIALIVIRFMINY-NLSPLQKIQTGLNSFFDFINHKTKDSAM-IDV 344

Clari_Slaughter_Beach_Tlp105 KALVKQIIIGAISLVIALIVIRFMINY-NLSPLQKIQTGLNSFFDFINHKTKDSAM-IDV 344

Clari_CCUG22395_Tlp105 KALVKQIIIGVISLVIALIVIRFMINY-NLSPLQKIQTGLNSFFDFINHKTKDSAM-IDV 344

Clari_RM16712_Tlp103 EALIKQIIIGAISLVIALIVIRFMINY-NLSPLKKIQTGLNSFFDFINYKTKDSAM-IDV 341

Clari_RM16701_Tlp103 SALIKQVLAAFVAILIALVLIKIVISK-LLSPLQKIQTGLNSFFDFINHKTKDSAM-IDV 341

Clari_RM1607_Tlp114 KVLFQQIIIALIAIAVALLLVRFAIIK-NLKPIAVITTGLNSFFDFINHKTKDSAM-IDV 348

Clari_RM2100_Tlp109 EVLFQQVIIAFIAIIIALILVRFAIIK-NLKPIAVITAGLNSFFDFINHKTKDSAM-INV 344

Clari_LMG11760_Tlp109 EVLFQQVIIAFIAIIIALILVRFAIIK-NLKPIMIITAGLNSFFDFINHKTKDSAM-IDV 344

Clari_NCTC11845_Tlp119 KVLFQQIIIALIAIAIALILIRFTIMK-NLKPIAVITTGLNSFFDFINHKTKDSAM-INV 344

Clari_SlaughterBeach_Tlp104 KVLFQQIIIALIAIAVALLLVRFAIIK-NLKPIAVITTGLNSFFDFINHKTKDSAM-IDV 344

Clari_RM16701_Tlp104 KVLFQQIIIALIAIAVALLLVRFAIIK-NLKPIAVITTGLNSFFDFINHKTKDSAM-IDV 344

Clari_RM16712_Tlp104 KVLFQQVIIALIAIIVALILVRFAIIK-NLKPIAVITTGLNSFFDFINYKTKDSAM-IDV 345

Clari_CCUG22395_Tlp104 KVLFQQIIIALIAIAVALLLVRFAIIK-NLKPIAVITTGLNSFFDFINHKTKDSAM-IDV 345

Clari_RM2100_Tlp112 EDVFDQIITSIVILILSCFIVYFISSR-LLSPLQVIQTGLNSFFDFINHKTKDSAM-INV 362

Clari_RM16712_Tlp106 KNAFEQTLISIVILILSCIFIHLFSAK-LLSPLQSIQTGLNSFFDFINYKTKDSAM-IDV 361

Clari_CCUG22395_Tlp121 KDAYKQIIFSIIILFISCFAIYFFSSK-LLSPLQAIQKGINSFFDFINHKTKDSAM-IDV 355

Clari_RM2100_Tlp123 QIALTQAIVVIIMIVLSIAVLYFIVSR-YLSPLEKIQTGLNSFFDFINHKTKDSAM-INV 340

Clari_LMG11760_Tlp107 KTASIQTIAVTVMVILSIILLYFIISY-YISPLQKIQTGLNSFFDFINHKTKDSAM-IDV 341

Clari_CCUG22395_Tlp120 KTATIQTIVVSIMVALSVIILYFIISY-YLSPLQAIQKGINSFFDFINHKTKDSAM-IDV 341

Clari_RM2100_Tlp111 KIAYTQAIIVIFTSIISVILLYFIISR-YLSPLEKIQTGLNSFFDFINHKTKDSAM-IDV 340

Clari_NCTC11845_Tlp117 KIAYIQTSIVVFTSVASIILLYFIISY-YLSPLQAIQTGLNSFFDFINHKTKDSAM-INV 343

Clari_RM16712_Tlp101 KIAYIQATIVIFTSIASIVLLYFIISY-FLSPLQSIQTGLNSFFDFINYKTKDSAM-IDV 343

Clari_CCUG22395_Tlp122 KIAYIQTTIVIFTSIASIILLYFIISY-YLSPLQAIQTGLNSFFDFINHKTKDSAM-IDV 343

Clari_LMG11760_Tlp101 KIAYIQTIIVIFTSITSIVLLYFIISY-YLSPLQTIQNGLSSFFDFINHKTKDSAM-IDV 343

Clari_SlaughterBeach_Tlp101 KIAYIQTTIVIFTSIASIILLYFIISY-YLSPLQAIQTGLNSFFDFINHKTKDSAM-IDV 343

Clari_SlaughterBeach_Tlp102 KIAYIQTTIVIFTSIASIILLYFIISY-YLSPLQAIQTGLNSFFDFINHKTKDSAM-IDV 343

Clari_RM16701_Tlp101 KIAYIQTIIVIFTSIASIILLYFIISY-YLSPLQAIQKGLNSFFDFINHKTKDSAM-IDV 343

Clari_RM2100_TLp113 KIAYIQAIIVIFTSILSVVLLYFIVSR-YLSPLEKIQTGLNSFFDFINHKTKDSAM-INV 341

Clari_NCTC11845_Tlp116 KIAYIQAIIVIFTSIISVILLYFLVSH-YLSPLQAIQTGLNSFFDFINHKTKDSAM-INV 340

. : : : : .: **.::*:: .: .:

Clari_LMG11760_Tlp108 KTQDEFGKMAEEINENIEIIKEALNKDAKAIEESVNIAKKIEAGELDLHISSHANNPQIQ 409

Clari_NCTC11845_Tlp108 KTQDEFGKMADEINENINIIKEALSKDAKAIEESVNVARKIETGELDLHISSHANNPQIQ 409

Clari_RM16701_Tlp108 KTQDEFGKMADEINENIEIIKEALSKDAKAIEESVNVARKIETGELDLHISSHANNPQIQ 409

Clari_CCUG22395_Tlp108 KTQDEFGKMADEINENIEIIKEALSKDAKAIEESVNVARKIETGELDLHISSHANNPQIQ 409

Clari_NCTC11845_Tlp115 KNNDELNTMAKAINENITKTKNALEQDAKAVEQSVDTAKEIENGNLTARITAIPANPQLI 440

Clari_SlaughterBeach_Tlp100 KTNDELGAMAKAINENITKTKNALEQDAKAVEQSVDTAKEIEGGNLTARITAIPANPQLV 427

Clari_RM16701_Tlp100 KTNDELGAMAKAINENITKTKNALEQDAKAVEQSVDTAKEIEGGNLTARITAIPANPQLV 437

Clari_RM2100_Tlp110 NTNDEFGVIAKAINENITKTKNALEQDAKAVEQSVETAKEIEAGNLTARITAIPANPQLI 402

Clari_NCTC11845_Tlp118 KTNDELGAMAKAINENITKTKNALEQDAKAVEQSVETVREVESGNLTARITAIPANPQLL 400

Clari_RM16712_Tlp105 KTNDELGAMAKAINENITRTKNALEQDAKAVEQSVDTAKEIESGNLTARITAIPANPQLV 404

Clari_RM16701_Tlp105 KTNDELGAMAKAINENITKTKNALEQDAKAVEQSVDTAKEIEGGNLTARITAIPANPQLV 404

Clari_Slaughter_Beach_Tlp105 KTNDELGAMAKAINENITKTKNALEQDAKAVEQSVDTAKEIEGGNLTARITAIPANPQLV 404

Clari_CCUG22395_Tlp105 KTNDELGAMAKAINENITKTKNALEQDAKAVEQSVDTAKEIESGNLTARITAIPANPQLI 404

Clari_RM16712_Tlp103 KTNDELGAMAKAINENITRTKNALEQDAKAVEQSVDTAKEIESGNLTARITAIPANPQLV 401

Clari_RM16701_Tlp103 KTNDELGAMAKAINENITKTKNALEQDAKAVEQSVDTAKEIEGGNLTARITAIPANPQLV 401

Clari_RM1607_Tlp114 KTNDELGAMAKAINENITKTKNALEQDAKAVEQSVDTAKEIEGGNLTARITAIPANPQLV 408

Clari_RM2100_Tlp109 NTNDELGAMAKAINENITKTKNALEQDAKAVEQSVETAKEIEAGNLTARITAIPANPQLI 404

Clari_LMG11760_Tlp109 KTNDELGAMAKAINENITKTKNALEQDTKAVEQSVETAREIESGNLTARINAMPANPQLI 404

Clari_NCTC11845_Tlp119 KTNDELGAMAKAINENITKTKNALEQDAKAVEQSVDTAKEIENGNLTARITAIPANPQLI 404

Clari_SlaughterBeach_Tlp104 KTNDELGAMAKAINENITKTKNALEQDAKAVEQSVDTAKEIEGGNLTARITAIPANPQLV 404

Clari_RM16701_Tlp104 KTNDELGAMAKAINENITKTKNALEQDAKAVEQSVDTAKEIEGGNLTARITAIPANPQLV 404

Clari_RM16712_Tlp104 KTNDELGAMAKAINENITRTKNALEQDAKAVEQSVDTAKEIESGNLTARITAIPANPQLV 405

Clari_CCUG22395_Tlp104 KTNDELGAMAKAINENITKTKNALEQDAKAVEQSVDTAKEIESGNLTARITAIPANPQLI 405

Clari_RM2100_Tlp112 NTNDEFGAMAKAINENITKTKNALEQDAKAVEQSVETAKEIEHGNLTARITAIPANPQLI 422

Clari_RM16712_Tlp106 KTNDELGAMAKAINENITRTKNALEQDAKAVEQSVDTAKEIESGNLTARITAIPANPQLV 421

Clari_CCUG22395_Tlp121 KTNDELGAMAKAINENIIKTKNALEQDAKAVEQSVDTAKEIESGNLTARITAIPANPQLI 415

Clari_RM2100_Tlp123 NTNDELGAMAKAINENITKTKNALEQDAKAVEQSVETAKEIEAGNLTARITAIPANPQLI 400

Clari_LMG11760_Tlp107 KTNDELGAMAKAINENITKTKNALEQDTKAVEQSVETAREIESGNLTARINAMPANPQLI 401

Clari_CCUG22395_Tlp120 KTNDEFGVIAKAINENITKTKNALEQDAKAVEQSVDTAKEIESGNLTARITAIPANPQLI 401

Clari_RM2100_Tlp111 KTNDELGAMAKAINENITKTKNALEQDAKAVEQSVETAKEIEAGNLTARITAIPANPQLI 400

Clari_NCTC11845_Tlp117 KTNDELGAMAKAINENITKTKNALEQDAKAVEQSVETVREVESGNLTARITAIPANPQLL 403

Clari_RM16712_Tlp101 KTNDELGAMAKAINENITRTKNALEQDAKAVEQSVDTAKEIESGNLTARITAIPANPQLV 403

Clari_CCUG22395_Tlp122 KTNDEFGVIAKAINENITKTKNALEQDAKAVEQSVDTAKEIESGNLTARITAIPANPQLI 403

Clari_LMG11760_Tlp101 KSNDEFGAMAKAINENITKTKNALEQDTKAVEQSVETVREVEGGNLTARINAMPANPQLL 403

Clari_SlaughterBeach_Tlp101 KTNDELGAIAKAINENITKTKNALEQDAKAVEQSVDTVREVEGGNLTARITAIPAHPQLL 403

Clari_SlaughterBeach_Tlp102 KTNDELGAIAKAINENITKTKNALEQDAKAVEQSVDTVREVEGGNLTARITAIPAHPQLL 403

Clari_RM16701_Tlp101 KSNDEFGVIAKAINENITKTKNALEQDAKAVEQSVETVREVEGGNLAARITAMPAHPQLL 403

Clari_RM2100_TLp113 NTNDELGAMAKAINENITKTKNALEQDAKAVEQSVDTAKEIESGNLTARITAIPANPQLI 401

Clari_NCTC11845_Tlp116 KTNDEFGAMAKAINENITKTKNALEQDAKAVEQSVETVREVESGNLTARITAIPANPQLL 400

:.:**:. :*. ***** *:**.:*:**:*:**: .:::* *:* :*.: :**:

Clari_LMG11760_Tlp108 ELMKVLNNMLLTLQRKIGSNLNEILAVFDSYKHLDFTATINAPKGDIEKAINSLGDEIKN 469

Clari_NCTC11845_Tlp108 ELIEVLNKMLTTLQAKIGRDLNEIQAVFNSYKHLDFTAAINTPKGDVEKAINALGNEIKN 469

Clari_RM16701_Tlp108 ELIEVLNKMLTTLQTKIGRDLNEIQAVFDSYKHLDFTAAINTPKGDVEKAINALGNEIKN 469

Clari_CCUG22395_Tlp108 ELIEVLNKMLTTLQTKIGRDLNEIQAVFDSYKHLDFTAAINTPKGDVEKAINALGNEIKN 469

Clari_NCTC11845_Tlp115 ELKNVLNDMLSVLEQKVGSNMNEINRVFDSYKALDFTTEVKNAKGGVEVTTNVLGQEIVA 500

Clari_SlaughterBeach_Tlp100 ELKNVLNEMLNVLEQKVGSNMNEINRVFDSYKALDFTTEVKNAKGGVEVTTNVLGQEIVA 487

Clari_RM16701_Tlp100 ELKNVLNEMLNVLEQKVGSNMNEINRVFDSYKALDFTTEVKNAKGGVEVTTNVLGQEIVA 497

Clari_RM2100_Tlp110 ELKNVLNDMLSVLEEKVGSNMNEINRVFDSYKALDFTTEVANAKGGVEITTNVLGQEIVA 462

Clari_NCTC11845_Tlp118 ELKNYLNEMLSVLEQKVGSNMNEINRVFDSYKALDFTTEVKNAKGGVEVTTNVLGQEIVA 460

Clari_RM16712_Tlp105 ELKNVLNDMLNVLEQKVGSNMNEINRVFDSYKALDFTTEVKNAKGGVEVTTNVLGQEIVA 464

Clari_RM16701_Tlp105 ELKNVLNDMLNVLEQKVGSNMNEINRVFDSYKALDFTTEVKNAKGGVEVTTNVLGQEIVA 464

Clari_Slaughter_Beach_Tlp105 ELKNVLNEMLNVLEQKVGSNMNEINRVFDSYKALDFTTEVKNAKGGVEVTTNVLGQEIVA 464

Clari_CCUG22395_Tlp105 ELKNVLNEMLNVLEQKVGSNMNEINRVFDSYKALDFTTEVKNAKGGVEVTTNVLGQEIVA 464

Clari_RM16712_Tlp103 ELKNVLNDMLNVLEQKVGSNMNEINRVFDSYKALDFTTEVKNAKGGVEVTTNVLGQEIVA 461

Clari_RM16701_Tlp103 ELKNVLNEMLNVLEQKVGSNMNEINRVFDSYKALDFTTEVKNAKGGVEVTTNVLGQEIVA 461

Clari_RM1607_Tlp114 ELKNVLNEMLNVLEQKVGSNMNEINRVFDSYKALDFTTEVKNAKGGVEVTTNVLGQEIVA 468

Clari_RM2100_Tlp109 ELKNVLNDMLSVLEEKVGSNMNEINRVFDSYKALDFTTEVANAKGGVEITTNVLGQEIVA 464

Clari_LMG11760_Tlp109 ELKNVLNEMLNVLEQKVGSNMNEINRVFDSYKALDFTTEVANAKGGVEVTTNVLGKEIVA 464

Clari_NCTC11845_Tlp119 ELKNVLNDMLSVLEQKVGSNMNEINRVFDSYKALDFTTEVKNAKGGVEVTTNVLGQEIVA 464

Clari_SlaughterBeach_Tlp104 ELKNVLNEMLNVLEQKVGSNMNEINRVFDSYKALDFTTEVKNAKGGVEVTTNVLGQEIVA 464

Clari_RM16701_Tlp104 ELKNVLNDMLNVLEQKVGSNMNEINRVFDSYKALDFTTEVKNAKGGVEVTTNVLGQEIVA 464

Clari_RM16712_Tlp104 ELKNVLNDMLNVLEQKVGSNMNEINRVFDSYKALDFTTEVKNAKGGVEVTTNVLGQEIVA 465

Clari_CCUG22395_Tlp104 ELKNVLNEMLNVLEQKVGSNMNEINRVFDSYKALDFTTEVKNAKGGVEVTTNVLGQEIVA 465

Clari_RM2100_Tlp112 ELKNVLNNMLSVLEEKVGSNMNEINRVFDSYKALDFTTEVANAKGEVEITTNVLGQEIVN 482

Clari_RM16712_Tlp106 ELKNVLNDMLNVLEQKVGSNMNEINRVFDSYKALDFTTEVKNAKGGVEVTTNVLGQEIVA 481

Clari_CCUG22395_Tlp121 ELKNVLNEMLNVLEQKVGSNMNEINRVFDSYKALDFTTEVKNAKGEVEVTTNVLGQEIVA 475

Clari_RM2100_Tlp123 ELKNVLNDMLSVLEEKVGSNMNEINRVFDSYKALDFTTEVANAKGGVEITTNVLGQEIVA 460

Clari_LMG11760_Tlp107 ELKNVLNEMLNVLEQKVGSNMNEINRVFDSYKALDFTTEVANAKGGVEVTTNVLGKEIVA 461

Clari_CCUG22395_Tlp120 ELKNVLNEMLNVLEQKVGSNMNEINRVFDSYKALDFTTEVKNAKGGVEVTTNVLGQEIVA 461

Clari_RM2100_Tlp111 ELKNVLNDMLSVLEEKVGSNMNEINRVFDSYKALDFTTEVANAKGGVEITTNVLGQEIVA 460

Clari_NCTC11845_Tlp117 ELKNYLNEMLSVLEQKVGSNMNEINRVFDSYKALDFTTEVKNAKGGVEVTANVLGQEIVA 463

Clari_RM16712_Tlp101 ELKNVLNDMLNVLEQKVGSNMNEINRVFDSYKALDFTTEVKNAKGGVEVTTNVLGQEIVA 463

Clari_CCUG22395_Tlp122 ELKNVLNEMLNVLEQKVGSNMNEINRVFDSYKALDFTTEVKNAKGGVEVTANVLGQEIVA 463

Clari_LMG11760_Tlp101 KLKNYLNEMLNVLEQKVGSNMNEINRVFDSYKALDFTTEVANAKGGVEVTANALGKEIVA 463

Clari_SlaughterBeach_Tlp101 ELKNYINEMLNVLEQKVGSNMNEINRVFDSYKALDFTTEVKNAKGGVEVTTNVLGQEIVA 463

Clari_SlaughterBeach_Tlp102 ELKNYINEMLNVLEQKVGSNMNEINRVFDSYKALDFTTEVKNAKGGVEVTTNVLGQEIVA 463

Clari_RM16701_Tlp101 ELKNYLNEMLAVLEQKVGSNMNEINRVFDSYKALDFTTEVKNAKGGVEVTANALGQEIVA 463

Clari_RM2100_TLp113 ELKNVLNEMLSVLEEKVGSNMNEINRVLDSYKALDFTTEVTNAKGGVEITTNVLGQEIVA 461

Clari_NCTC11845_Tlp116 ELKNYLNEMLSVLEQKVGSNMNEINRVFDSYKALDFTTEVKNAKGGVEVTTNVLGQEIVA 460

:* : :*.** .*: *:* ::*** *::*** ****: : ** :* : * **.**

Clari_LMG11760_Tlp108 MLTQSLHQGELLKQKAEALKQSMQELTNDATHQTSSLQESARALEQMNSAMSEISIKTQD 529

Clari_NCTC11845_Tlp108 MLSQSLNQGELLNQKAEALKQSMQELTNDATHQTSSLQESARALEQMNSAMSEISIKAQD 529

Clari_RM16701_Tlp108 MLSQSLNQGELLNQKAEALKQSMQELTNDATHQTSSLQESARALEQMNSAMSEISIKAQD 529

Clari_CCUG22395_Tlp108 MLSQSLNQGELLNQKAEALKQSMQELTNDATHQTSSLQESARALEQMNSAMSEISIKAQD 529

Clari_NCTC11845_Tlp115 MLRQSSEFASLLADESGKLQSAVKDLTDSSSSQASSLEETAAALEEITSSMQNVSHKTSE 560

Clari_SlaughterBeach_Tlp100 MLRQSSEFASLLADESGKLQSAVKNLTDSSSSQASSLEETAAALEEITSSMQNVSHKTSE 547

Clari_RM16701_Tlp100 MLRQSSEFASLLADESGKLQSAVKNLTDSSSSQASSLEETAAALEEITSSMQNVSHKTSE 557

Clari_RM2100_Tlp110 MLRQSSEFANLLATQSGKLQSAVRELTDSSSSQASSLEETAAALEEITSSMQNVSHKTSE 522

Clari_NCTC11845_Tlp118 MLRQSSEFASLLADESGKLQSAVKDLTDSSSSQASSLEETAAALEEITSSMQNVSHKTSE 520

Clari_RM16712_Tlp105 MLRQSSEFASLLADESGKLQSAVKDLTDSSSSQASSLEETAAALEEITSSMQNVSHKTSE 524

Clari_RM16701_Tlp105 MLRQSSEFASLLADESGKLQSAVKNLTDSSSSQASSLEETAAALEEITSSMQNVSHKTSE 524

Clari_Slaughter_Beach_Tlp105 MLRQSSEFASLLADESGKLQSAVKNLTDSSSSQASSLEETAAALEEITSSMQNVSHKTSE 524

Clari_CCUG22395_Tlp105 MLRQSSEFASLLADESGKLQSAVKNLTDSSSSQASSLEETAAALEEITSSMQNVSHKTSE 524

Clari_RM16712_Tlp103 MLRQSSEFASLLADESGKLQSAVKDLTDSSSSQASSLEETAAALEEITSSMQNVSHKTSE 521

Clari_RM16701_Tlp103 MLRQSSEFASLLADESGKLQSAVKNLTDSSSSQASSLEETAAALEEITSSMQNVSHKTSE 521

Clari_RM1607_Tlp114 MLRQSSEFASLLADESGKLQSAVKNLTDSSSSQASSLEETAAALEEITSSMQNVSHKTSE 528

Clari_RM2100_Tlp109 MLRQSSEFANLLATQSGKLQSAVRELTDSSSSQASSLEETAAALEEITSSMQNVSHKTSE 524

Clari_LMG11760_Tlp109 MLRQSSEFANLLASESGKLQSAVKNLTDSSSSQASSLEETAAALEEITSSMQNVSHKTSE 524

Clari_NCTC11845_Tlp119 MLRQSSEFASLLADESGKLQSAVKDLTDSSSSQASSLEETAAALEEITSSMQNVSHKTSE 524

Clari_SlaughterBeach_Tlp104 MLRQSSEFASLLADESGKLQSAVKNLTDSSSSQASSLEETAAALEEITSSMQNVSHKTSE 524

Clari_RM16701_Tlp104 MLRQSSEFASLLADESGKLQSAVKNLTDSSSSQASSLEETAAALEEITSSMQNVSHKTSE 524

Clari_RM16712_Tlp104 MLRQSSEFASLLADESGKLQSAVKDLTDSSSSQASSLEETAAALEEITSSMQNVSHKTSE 525

Clari_CCUG22395_Tlp104 MLRQSSEFASLLADESGKLQSAVKNLTDSSSSQASSLEETAAALEEITSSMQNVSHKTSE 525

Clari_RM2100_Tlp112 MLRQSSEFANLLATQSGKLQSAVRELTDSSSSQASSLEETAAALEEITSSMQNVSSKTSE 542

Clari_RM16712_Tlp106 MLRQSSEFASLLADESGKLQSAVKDLTDSSSSQASSLEETAAALEEITSSMQNVSHKTSE 541

Clari_CCUG22395_Tlp121 MLRQSSEFASLLADESGKLQSAVKNLTDSSSSQASSLEETAAALEEITSSMQNVSHKTSE 535

Clari_RM2100_Tlp123 MLRQSSEFANLLATQSGKLQSAVRELTDSSSSQASSLEETAAALEEITSSMQNVSHKTSE 520

Clari_LMG11760_Tlp107 MLRQSSEFANLLASESGKLQSAVKNLTDSSSSQASSLEETAAALEEITSSMQNVSHKTSE 521

Clari_CCUG22395_Tlp120 MLRQSSEFASLLADESGKLQSAVKNLTDSSSSQASSLEETAAALEEITSSMQNVSHKTSE 521

Clari_RM2100_Tlp111 MLRQSSEFANLLATQSGKLQSAVRELTDSSSSQASSLEETAAALEEITSSMQNVSHKTSE 520

Clari_NCTC11845_Tlp117 MLRQSSEFASLLADESGKLQSAVKDLTDSSSSQASSLEETAAALEEITSSMQNVSHKTSE 523

Clari_RM16712_Tlp101 MLRQSSEFASLLADESGKLQSAVKDLTDSSSSQASSLEETAAALEEITSSMQNVSHKTSE 523

Clari_CCUG22395_Tlp122 MLRQSSEFASLLADESGKLQSAVKNLTDSSSSQASSLEETAAALEEITSSMQNVSHKTSE 523

Clari_LMG11760_Tlp101 MLRQSSEFANLLASESGKLQSAVKNLTDSSSSQASSLEETAAALEEITSSMQNVSHKTSE 523

Clari_SlaughterBeach_Tlp101 MLRQSSEFASLLADESGKLQSAVKNLTDSSSSQASSLEETAAALEEITSSMQNVSHKTSE 523

Clari_SlaughterBeach_Tlp102 MLRQSSEFASLLADESGKLQSAVKNLTDSSSSQASSLEETAAALEEITSSMQNVSHKTSE 523

Clari_RM16701_Tlp101 MLRQSSEFASLLADESGKLQSAVKNLTDSSSSQASSLEETAAALEEITSSMQNVSHKTSE 523

Clari_RM2100_TLp113 MLRQSSEFANLLATQSGKLQSAVRELTDSSSSQASSLEETAAALEEITSSMQNVSHKTSE 521

Clari_NCTC11845_Tlp116 MLRQSSEFASLLADESGKLQSAVKDLTDSSSSQASSLEETAAALEEITSSMQNVSHKTSE 520

** ** . ..** :: *:.::::**:.:: *:***:*:* ***::.*:*.::* *:.:

Clari_LMG11760_Tlp108 VVKQSNDIKNVTTVISDIADQINLLALNAAIEAARAGEHGRGFAVVADEVRNLAERTQKS 589

Clari_NCTC11845_Tlp108 VVKQSNDIKSVTTVISDIAEQINLLALNAAIEAARAGEHGRGFAVVAEEVRNLAERTQKS 589

Clari_RM16701_Tlp108 VVKQSNDIKSVTTVISDIAEQINLLALNAAIEAARAGEHGRGFAVVAEEVRNLAERTQKS 589

Clari_CCUG22395_Tlp108 VVKQSNDIKSVTTVISDIAEQINLLALNAAIEAARAGEHGRGFAVVAEEVRNLAERTQKS 589

Clari_NCTC11845_Tlp115 VIAQSEEIKNVTSIIGDIADQINLLALNAAIEAARAGEHGRGFAVVADEVRNLAERTQKS 620

Clari_SlaughterBeach_Tlp100 VIAQSEEIKNVTSIIGDIADQINLLALNAAIEAARAGEHGRGFAVVADEVRNLAERTQKS 607

Clari_RM16701_Tlp100 VIAQSEEIKNVTSIIGDIADQINLLALNAAIEAARAGEHGRGFAVVADEVRNLAERTQKS 617

Clari_RM2100_Tlp110 VIAQSEEIKNVTSIIGDIADQINLLALNAAIEAARAGEHGRGFAVVADEVRNLAERTQKS 582

Clari_NCTC11845_Tlp118 VIAQSEEIKNVTSIIGDIADQINLLALNAAIEAARAGEHGRGFAVVADEVRNLAERTQKS 580

Clari_RM16712_Tlp105 VIAQSEEIKNVTSIIGDIADQINLLALNAAIEAARAGEHGRGFAVVADEVRNLAERTQKS 584

Clari_RM16701_Tlp105 VIAQSEEIKNVTSIIGDIADQINLLALNAAIEAARAGEHGRGFAVVADEVRNLAERTQKS 584

Clari_Slaughter_Beach_Tlp105 VIAQSEEIKNVTSIIGDIADQINLLALNAAIEAARAGEHGRGFAVVADEVRNLAERTQKS 584

Clari_CCUG22395_Tlp105 VIAQSEEIKNVTSIIGDIADQINLLALNAAIEAARAGEHGRGFAVVADEVRNLAERTQKS 584

Clari_RM16712_Tlp103 VIAQSEEIKNVTSIIGDIADQINLLALNAAIEAARAGEHGRGFAVVADEVRNLAERTQKS 581

Clari_RM16701_Tlp103 VIAQSEEIKNVTSIIGDIADQINLLALNAAIEAARAGEHGRGFAVVADEVRNLAERTQKS 581

Clari_RM1607_Tlp114 VIAQSEEIKNVTSIIGDIADQINLLALNAAIEAARAGEHGRGFAVVADEVRNLAERTQKS 588

Clari_RM2100_Tlp109 VIAQSEEIKNVTSIIGDIADQINLLALNAAIEAARAGEHGRGFAVVADEVRNLAERTQKS 584

Clari_LMG11760_Tlp109 VIAQSEEIKNVTSIIGDIADQINLLALNAAIEAARAGEHGRGFAVVADEVRNLAERTQKS 584

Clari_NCTC11845_Tlp119 VIAQSEEIKNVTSIIGDIADQINLLALNAAIEAARAGEHGRGFAVVADEVRNLAERTQKS 584

Clari_SlaughterBeach_Tlp104 VIAQSEEIKNVTSIIGDIADQINLLALNAAIEAARAGEHGRGFAVVADEVRNLAERTQKS 584

Clari_RM16701_Tlp104 VIAQSEEIKNVTSIIGDIADQINLLALNAAIEAARAGEHGRGFAVVADEVRNLAERTQKS 584

Clari_RM16712_Tlp104 VIAQSEEIKNVTSIIGDIADQINLLALNAAIEAARAGEHGRGFAVVADEVRNLAERTQKS 585

Clari_CCUG22395_Tlp104 VIAQSEEIKNVTSIIGDIADQINLLALNAAIEAARAGEHGRGFAVVADEVRNLAERTQKS 585

Clari_RM2100_Tlp112 VIAQSEEIKNVTSIIGDIADQINLLALNAAIEAARAGEHGRGFAVVADEVRNLAERTQKS 602

Clari_RM16712_Tlp106 VIAQSEEIKNVTSIIGDIADQINLLALNAAIEAARAGEHGRGFAVVADEVRNLAERTQKS 601

Clari_CCUG22395_Tlp121 VIAQSEEIKNVTSIIGDIADQINLLALNAAIEAARAGEHGRGFAVVADEVRNLAERTQKS 595

Clari_RM2100_Tlp123 VIAQSEEIKNVTSIIGDIADQINLLALNAAIEAARAGEHGRGFAVVADEVRNLAERTQKS 580

Clari_LMG11760_Tlp107 VIAQSEEIKNVTSIIGDIADQINLLALNAAIEAARAGEHGRGFAVVADEVRNLAERTQKS 581

Clari_CCUG22395_Tlp120 VIAQSEEIKNVTSIIGDIADQINLLALNAAIEAARAGEHGRGFAVVADEVRNLAERTQKS 581

Clari_RM2100_Tlp111 VIAQSEEIKNVTSIIGDIADQINLLALNAAIEAARAGEHGRGFAVVADEVRNLAERTQKS 580

Clari_NCTC11845_Tlp117 VIAQSEEIKNVTSIIGDIADQINLLALNAAIEAARAGEHGRGFAVVADEVRNLAERTQKS 583

Clari_RM16712_Tlp101 VIAQSEEIKNVTSIIGDIADQINLLALNAAIEAARAGEHGRGFAVVADEVRNLAERTQKS 583

Clari_CCUG22395_Tlp122 VIAQSEEIKNVTSIIGDIADQINLLALNAAIEAARAGEHGRGFAVVADEVRNLAERTQKS 583

Clari_LMG11760_Tlp101 VIAQSEEIKNVTSIIGDIADQINLLALNAAIEAARAGEHGRGFAVVADEVRNLAERTQKS 583

Clari_SlaughterBeach_Tlp101 VIAQSEEIKNVTSIIGDIADQINLLALNAAIEAARAGEHGRGFAVVADEVRNLAERTQKS 583

Clari_SlaughterBeach_Tlp102 VIAQSEEIKNVTSIIGDIADQINLLALNAAIEAARAGEHGRGFAVVADEVRNLAERTQKS 583

Clari_RM16701_Tlp101 VIAQSEEIKNVTSIIGDIADQINLLALNAAIEAARAGEHGRGFAVVADEVRNLAERTQKS 583

Clari_RM2100_TLp113 VIAQSEEIKNVTSIIGDIADQINLLALNAAIEAARAGEHGRGFAVVADEVRNLAERTQKS 581

Clari_NCTC11845_Tlp116 VIAQSEEIKNVTSIIGDIADQINLLALNAAIEAARAGEHGRGFAVVADEVRNLAERTQKS 580

*: **::**.**::*.***:***************************:************

Clari_LMG11760_Tlp108 LGEIEANTNILVQSINDMGEAIKEEADDISQINESVATIEKLTQQNSQTAMQTNAIANEV 649

Clari_NCTC11845_Tlp108 LGEIEANTNILAQSISDMGDSIKEEANDISQINESVATIEKLTQQNSQTAIRTNAIANEV 649

Clari_RM16701_Tlp108 LGEIEANTNILAQSISDMGDSIKEEANDISQINESVATIEKLTQQNSQTAIRTNAIANEV 649

Clari_CCUG22395_Tlp108 LGEIEANTNILAQSISDMGDSIKEEANDISQINESVATIEKLTQQNSQTAIRTNAIANEV 649

Clari_NCTC11845_Tlp115 LGEIEANTNILVQSINEMGESIKEQTTGITQINDAVAQIDHVTQENLKIAKDSATISDNV 680

Clari_SlaughterBeach_Tlp100 LGEIEANTNILVQSINEMGESIKEQTTGITQINDAVAQIDHVTQENLKIAKDSAIVADNV 667

Clari_RM16701_Tlp100 LGEIEANTNILVQSINEMGESIKEQTTGITQINDAVAQIDHVTQENLKIAKDSAIVADNV 677

Clari_RM2100_Tlp110 LGEIEANTNILVQSINEMGESIKEQTTGITQINDAVAQIDHVTQENLKIANDSAAISENV 642

Clari_NCTC11845_Tlp118 LGEIEANTNILVQSINEMGESIKEQTTGITQINDAVAQIDHVTQENLKIAKDSATISDNV 640

Clari_RM16712_Tlp105 LGEIEANTNILVQSINEMGESIKEQTTGITQINDAVAQIDHVTQENLKIAKDSAAISDNV 644

Clari_RM16701_Tlp105 LGEIEANTNILVQSINEMGESIKEQTTGITQINDAVAQIDHVTQENLKIAKDSAAISDNV 644

Clari_Slaughter_Beach_Tlp105 LGEIEANTNILVQSINEMGESIKEQTTGITQINDAVAQIDHVTQENLKIAKDSAAISDNV 644

Clari_CCUG22395_Tlp105 LGEIEANTNILVQSINEMGESIKEQTTGITQINDAVAQIDHVTQENLKIAKDSAAISDNV 644

Clari_RM16712_Tlp103 LGEIEANTNILVQSINEMGESIKEQTTGITQINDAVAQIDHVTQENLKIAKDSAAISDNV 641

Clari_RM16701_Tlp103 LGEIEANTNILVQSINEMGESIKEQTTGITQINDAVAQIDHVTQENLKIAKDSAAISDNV 641

Clari_RM1607_Tlp114 LGEIEANTNILVQSINEMGESIKEQTTGITQINDAVAQIDHVTQENLKIAKDSAIVADNV 648

Clari_RM2100_Tlp109 LGEIEANTNILVQSINEMGESIKEQTTGITQINDAVAQIDHVTQENLKIANDSAAISENV 644

Clari_LMG11760_Tlp109 LGEIEANTNILVQSINEMGESIKEQTTGITQINDAVAQIDHVTQENLKIANDSAAISDNV 644

Clari_NCTC11845_Tlp119 LGEIEANTNILVQSINEMGESIKEQTTGITQINDAVAQIDHVTQENLKIAKDSATISDNV 644

Clari_SlaughterBeach_Tlp104 LGEIEANTNILVQSINEMGESIKEQTTGITQINDAVAQIDHVTQENLKIAKDSAAISDNV 644

Clari_RM16701_Tlp104 LGEIEANTNILVQSINEMGESIKEQTTGITQINDAVAQIDHVTQENLKIAKDSAAISDNV 644

Clari_RM16712_Tlp104 LGEIEANTNILVQSINEMGESIKEQTTGITQINDAVAQIDHVTQENLKIAKDSAAISDNV 645

Clari_CCUG22395_Tlp104 LGEIEANTNILVQSINEMGESIKEQTTGITQINDAVAQIDHVTQENLKIAKDSAAISDNV 645

Clari_RM2100_Tlp112 LGEIEANTNILVQSINEMGESIKEQTTGITQINDAVAQIDHVTQENLKIAKDSAAISENV 662

Clari_RM16712_Tlp106 LGEIEANTNILVQSINEMGESIKEQTTGITQINDAVAQIDHVTQENLKIAKDSAAISDNV 661

Clari_CCUG22395_Tlp121 LGEIEANTNILVQSINEMGESIKEQTTGITQINDAVAQIDHVTQENLKIAKDSAAISDNV 655

Clari_RM2100_Tlp123 LGEIEANTNILVQSINEMGESIKEQTTGITQINDAVAQIDHVTQENLKIANDSAAISENV 640

Clari_LMG11760_Tlp107 LGEIEANTNILVQSINEMGESIKEQTTGITQINDAVAQIDHVTQENLKIANDSAIVADNV 641

Clari_CCUG22395_Tlp120 LGEIEANTNILVQSINEMGESIKEQTTGITQINDAVAQIDHVTQENLKIANDSAIVADNV 641

Clari_RM2100_Tlp111 LGEIEANTNILVQSINEMGESIKEQTTGITQINDAVAQIDHVTQENLKIANDSAAISENV 640

Clari_NCTC11845_Tlp117 LGEIEANTNILVQSINEMGESIKEQTTGITQINDAVAQIDHVTQENLKIAKDSATISDNV 643

Clari_RM16712_Tlp101 LGEIEANTNILVQSINEMGESIKEQTTGITQINDAVAQIDHVTQENLKIAKDSAAISDNV 643

Clari_CCUG22395_Tlp122 LGEIEANTNILVQSINEMGESIKEQTTGITQINDAVAQIDHVTQENLKIANDSAIVADNV 643

Clari_LMG11760_Tlp101 LGEIEANTNILVQSINEMGESIKEQTTGITQINDAVAQIDHVTQENLKIANDSAIVADNV 643

Clari_SlaughterBeach_Tlp101 LGEIEANTNILVQSINEMGESIKEQTTGITQINDAVAQIDHVTQENLKIAKDSAAISDNV 643

Clari_SlaughterBeach_Tlp102 LGEIEANTNILVQSINEMGESIKEQTTGITQINDAVAQIDHVTQENLKIAKDSAAISDNV 643

Clari_RM16701_Tlp101 LGEIEANTNILVQSINEMGESIKEQTTGITQINDAVAQIDHVTQENLKIAKDSAIVADNV 643

Clari_RM2100_TLp113 LGEIEANTNILVQSINEMGESIKEQTTGITQINDAVAQIDHVTQENLKIANDSAIVADNV 641

Clari_NCTC11845_Tlp116 LGEIEANTNILVQSINEMGESIKEQTTGITQINDAVAQIDHVTQENLKIAKDSATISDNV 640

***********.***.:**::***:: .*:***::** *:::**:* : * : ::::*

Clari_LMG11760_Tlp108 DSLAQDMLSETKKRKF 665

Clari_NCTC11845_Tlp108 DSLAQDILSETKKRKF 665

Clari_RM16701_Tlp108 DSLAQDILSETKKRKF 665

Clari_CCUG22395_Tlp108 DSLAQDILSETKKRKF 665

Clari_NCTC11845_Tlp115 NKIANDILEDARKKKF 696

Clari_SlaughterBeach_Tlp100 NKIASDILEDARKKKF 683

Clari_RM16701_Tlp100 NKIANDILEDARKKKF 693

Clari_RM2100_Tlp110 NKIANDILEDAKKKRF 658

Clari_NCTC11845_Tlp118 NKIANDILEDARKKKF 656

Clari_RM16712_Tlp105 NKIANDILEDARKKKF 660

Clari_RM16701_Tlp105 NKIANDILEDARKKKF 660

Clari_Slaughter_Beach_Tlp105 NKIANDILEDARKKKF 660

Clari_CCUG22395_Tlp105 NKIANDILEDARKKKF 660

Clari_RM16712_Tlp103 NKIANDILEDARKKKF 657

Clari_RM16701_Tlp103 NKIANDILEDARKKKF 657

Clari_RM1607_Tlp114 NKIANDILEDARKKKF 664

Clari_RM2100_Tlp109 NKIANDILEDAKKKRF 660

Clari_LMG11760_Tlp109 NKIANDILEDARKKKF 660

Clari_NCTC11845_Tlp119 NKIANDILEDARKKKF 660

Clari_SlaughterBeach_Tlp104 NKIANDILEDARKKKF 660

Clari_RM16701_Tlp104 NKIANDILEDARKKKF 660

Clari_RM16712_Tlp104 NKIANDILEDARKKKF 661

Clari_CCUG22395_Tlp104 NKIANDILEDARKKKF 661

Clari_RM2100_Tlp112 NQIANDILEDAKKKRF 678

Clari_RM16712_Tlp106 NKIANDILEDARKKKF 677

Clari_CCUG22395_Tlp121 NKIANDILEDARKKKF 671

Clari_RM2100_Tlp123 NKIANDILEDAKKKRF 656

Clari_LMG11760_Tlp107 NKIASDILEDARKKKF 657

Clari_CCUG22395_Tlp120 NKIASDILEDARKKKF 657

Clari_RM2100_Tlp111 NKIANDILEDAKKKRF 656

Clari_NCTC11845_Tlp117 NKIANDILEDARKKKF 659

Clari_RM16712_Tlp101 NKIANDILEDARKKKF 659

Clari_CCUG22395_Tlp122 NQIANDILEDARKKKF 659

Clari_LMG11760_Tlp101 NKIASDILEDARKKKF 659

Clari_SlaughterBeach_Tlp101 NKIANDILEDARKKKF 659

Clari_SlaughterBeach_Tlp102 NKIANDILEDARKKKF 659

Clari_RM16701_Tlp101 NKIASDILEDARKKKF 659

Clari_RM2100_TLp113 NKIASDILEDAKKKKF 657

Clari_NCTC11845_Tlp116 NKIANDILEDARKKKF 656

:.:*.*:*.:::*::*
