# Supplementary material for: Diversity of transducer-like proteins (Tlps) in Campylobacter
Source: PLoS One. 2019 Mar 25;14(3):e0214228. doi: 10.1371/journal.pone.0214228 (PMC6433261; doi:10.1371/journal.pone.0214228)
Supplement: S2 Archive — (ZIP) [file pone.0214228.s016.zip › Alignment B.docx]

Alignment B. Alignment of all *C. lari* Tlp proteins except Tlp108

CLUSTAL O(1.2.4) multiple sequence alignment 2018/05/11

Clari_NCTC11845_Tlp115 ---LFSNLKIGTKIVTVVITII---VLGIGILASIITMQSSNILHTEAD-KLLQTSAF-- 51

Clari_SlaughterBeach_Tlp100 -------------MILILSSVI---FIGIGILSVTIISKSQEMLNDEAH-KLLLSSAN-- 41

Clari_RM16701_Tlp100 ---MFKNFNLTTKMILILSSVI---FIGIGILSVTIISKSQEMLNDEAH-KLLLSSAN-- 51

Clari_RM2100_Tlp110 ----MKINSIVSKVNILVGILFAATIVIIGSIAYFQTKQSSFEYLRENHNKVLFDVGYIF 56

Clari_NCTC11845_Tlp118 ------MKSLANKLTFFVFLAIIAILFVANIFNYIEVKRDVQKLINDIQIKTMQDVLKSF 54

Clari_RM16712_Tlp105 --MGKITKTLTSKLTFFVFLAIIAILFIANAFNYIEVKHDVQKLINDIQVKTMQDVLKSF 58

Clari_RM16701_Tlp105 --MGKITKTLTGKLTFFAFLAIIAILFIANAFNYAGVKHDVQKLINDIQVKTIQDVLKSF 58

Clari_Slaughter_Beach_Tlp105 --MGKITKTLTGKLTFFAFLAIIAILFIANAFNYAEVKHDVQKLINDIQVKTMQDVLKSF 58

Clari_CCUG22395_Tlp105 --MGKITKTLTGKLTFFAFLAIIAILFIANAFNYAEVKHDVQKLINDIQVKTMQDVLKSF 58

Clari_RM16712_Tlp103 -----MFQSITSRLTLVIAIISIIVLVGVNGLSYYNIKEDTYEYLEEIQKKTMLDTAEVF 55

Clari_RM16701_Tlp103 -----MFQNITGRLTLVIAIVSMIVLAVVNGLSYYNAKEDTYDYLEEIQRKTMLDTAEVF 55

Clari_RM1607_Tlp114 ---MFKFNSLSNKLTSIVCFLIVIILAIVNILNYYDSKKSTSYYLEEIQKKTMFDVNYMY 57

Clari_RM2100_Tlp109 ---MFNFRSLSSKLTFIVGLLIIAILITVNIISYYQSKNSTSQYLEEIQVKTMFDVNKAY 57

Clari_LMG11760_Tlp109 ---MFNFRSLSSKLTFIVGLLIIAILITVNIISYYQSKNSTSQYLEEIQVKTMFDVNKAY 57

Clari_NCTC11845_Tlp119 ---MFKFNSLSNKLTMIVCFLIAIILIVVNIINYYESKKTTAYYLEEIQKKTMFDVNEAY 57

Clari_SlaughterBeach_Tlp104 ---MFKFNSLSNKLTMIACFLIAIILIVVNVINYFESKQNTSYYLEEIQKKTMIDVNKAY 57

Clari_RM16701_Tlp104 ---MFKFNSLSNKLTMIACFLIAIILIVVNVINYFESKQNTSYYLEEIQKKTMIDVNKAY 57

Clari_RM16712_Tlp104 ---MFKFNSLSNKITMIACSLIAIILIVANIINYYQSKESTRYYLEEIQKKTMFDVNSAY 57

Clari_CCUG22395_Tlp104 ---MFKFNSLSNKLTMIACFLIAIILIVVNIINYYQSKESTSYYLEEIQKKTMFDVNSAY 57

Clari_RM2100_Tlp112 MFFKNSFISVKNKLSYTTGIIVALALFIVAAMAFYSSRENLIINSKNANKDYLLVTTAQV 60

Clari_RM16712_Tlp106 -MFLSKNMSVKNKLSIIVAAIVALALFVITVMAFYSSREDLILNSKKSNEDYLLVTTTQV 59

Clari_CCUG22395_Tlp121 -------MSVKNKLSIVVGVIVFLALSIITIMAFVSSRNNLISNSKQANEDYLLITETQV 53

Clari_RM2100_Tlp123 -----MFSSLKIKLSLLANIFAALSLIVLGIISFIFTKNFLYENELKRQNDILQVSRISL 55

Clari_LMG11760_Tlp107 -----MLSSLKIKLSLVANLFAALCLIILGILSFYFTKNFLYSNELKRQNDILQVAKISL 55

Clari_CCUG22395_Tlp120 -----MFNTLKVKLSLMANFFTALSLIILGVLSFYFTKTYLYDNELKRQNDILQVARTSL 55

Clari_RM2100_Tlp111 -------MGIKLKISLIANIIAIVCLISLGITTFYFVKDALLKNTIEAQTNYLKSSKDLM 53

Clari_NCTC11845_Tlp117 ------MKSIKLKLSLIANIMAIFCLIVLGVISFIFTKKALNYEVIKAETNYVRAAEKSM 54

Clari_RM16712_Tlp101 ------MKSIKIKISLISNIIAIICLVILGVISFIFTEKALNYEVVKAETNYVKAAEKSM 54

Clari_CCUG22395_Tlp122 ------MKSIKLKISLIANIIAIVCLIILSIVSFIFTKKALNYEVVKAETNYVRTAEKSM 54

Clari_LMG11760_Tlp101 ------MKSIKLKISLIANVIAIICLIVLGVISFIFTKKALNHEVVKAETNYVKVAEKSM 54

Clari_SlaughterBeach_Tlp101 ------MKSIKLKISLIANIIAIICLIILGIISYIFTKKALNHEVVNAETNYVKVAEKSM 54

Clari_SlaughterBeach_Tlp102 ------MKSIKLKISLIANIIAIICLIILGIISYIFTKKALNHEVVNAETNYVKVAEKSM 54

Clari_RM16701_Tlp101 ------MKSIKLKISLIANVIAIICLIVLGIISFIFTKKALNHEVVKAETNYVKVAEKSM 54

Clari_RM2100_TLp113 ------MKSIKLKVAMIANIMAVICLLILGIVTFIFVKQSLFDEIVNSEKNRLISTNNLV 54

Clari_NCTC11845_Tlp116 ------MKSIKLKVSLIANTIAIFCLIILGVITFIFVKQALFDEIVKSEQNRLVSTKSLM 54

: . . . . :

Clari_NCTC11845_Tlp115 -RYSNIIRGATESVHSTLLSTESSIDQIL----DTQTSLEQNRIQDILEGAVDSNSWINY 106

Clari_SlaughterBeach_Tlp100 -RYSNGIQAITQNAYSTLETAQGVIKNFA----NKDNNLDIEDLKILISSMLDSNSWTYF 96

Clari_RM16701_Tlp100 -RYSNGIQAITQNAYSTLETAQGVIKNFA----NKDNNLDIEDLKILISSMLDSNSWTYF 106

Clari_RM2100_Tlp110 NTYEADNQS-------AIQNLANFAVE------NHILDN-EQEIFNALKLTQEYVGF-EI 101

Clari_NCTC11845_Tlp118 DDYTASRSD-------AIKAVAAEIK-------KNPNTS-LEEIYTMVKVAKEASRF-DV 98

Clari_RM16712_Tlp105 DDYTTSRSD-------AIKAVAAEIQ-------KNPNAS-LEEIYTMVKVAKESSRF-DV 102

Clari_RM16701_Tlp105 DDYTASRSD-------TIKAVAAEIQ-------KNPNAS-LEEIYTMVKVAKESSRF-DV 102

Clari_Slaughter_Beach_Tlp105 DDYTASRSD-------AIKAVAAEIQ-------KNPNAS-LEEIYTMVKVAKESSRF-DV 102

Clari_CCUG22395_Tlp105 DDYTASRSD-------AIKAVAAEIQ-------KNPNAS-LEEIYTMVKVAKESSRF-DV 102

Clari_RM16712_Tlp103 FIYSNAKRK-------AVSTLAEEIV-------KQDFSN-DENIYNFLEAFKKANNF-DI 99

Clari_RM16701_Tlp103 SIYSNAKRK-------AISTLAEEIV-------KQDFSD-DGNIYKILEAFKKANNF-DI 99

Clari_RM1607_Tlp114 SSYSNSKRN-------IIESLAYSLSA------IAYNSS-DREIFSVLDTAKRSGGF-DT 102

Clari_RM2100_Tlp109 EIYGTSKRT-------AIDSIVKFME-------KNPHPD-INELFDILETIRYSAGY-DV 101

Clari_LMG11760_Tlp109 EIYGTSKRT-------AIDSIVKFME-------KNPHPD-INELFDILETIRYSAGY-DV 101

Clari_NCTC11845_Tlp119 KIYSSSKRS-------AILSIVNFIE-------KNPNPS-TEELFDILETIRSSADF-DV 101

Clari_SlaughterBeach_Tlp104 EIYSNSKRT-------AISSIVSFME-------KNPNVE-TREIFDILATIKESAGF-DV 101

Clari_RM16701_Tlp104 EIYSNSKRT-------AISSIVSFME-------KNPNVE-TREIFDILATIKESAGF-DV 101

Clari_RM16712_Tlp104 NIYSGAKRE-------AISSIVKFVE-------KNPKVD-TAEIFDMLETIKEAAGF-NV 101

Clari_CCUG22395_Tlp104 NIYSGAKRD-------AIQSIVKFVE-------KNPRVD-SAELFDVLETVKEAAGF-NV 101

Clari_RM2100_Tlp112 ETYIESYVE-------ILLSIKKYIDSLPKYQTENFDKL-GEYFAKDLKVFKDGSNT-LA 111

Clari_RM16712_Tlp106 EGYVENYVD-------ILLAIKKHIDQLPEYQIKNFDTL-SEYFAKDLKIFKDGSNT-LA 110

Clari_CCUG22395_Tlp121 EGYVDGYID-------ILLAIKKYIDSLGKNQVLNFDSM-EQMLGDDLKIFKDGSNT-LA 104

Clari_RM2100_Tlp123 ETFRENNIK-------LINHLEESVLELPYEKLNSQEDL-IENLGQMLKSYRKASGV-LA 106

Clari_LMG11760_Tlp107 ETFRDINTN-------LITNLEKSILEHPYEKLNSEEAL-IENIGSTLKSYRKASGV-LA 106

Clari_CCUG22395_Tlp120 ETFRKHNSD-------LILNLEKTILEFPYEKLNSEQAL-IDNVGSILKSYRKASGV-LS 106

Clari_RM2100_Tlp111 NDFKTSTER-------SLQNLSRAILKHPLYKLKDEESV-LASLAVELKAFRDSGGF-LG 104

Clari_NCTC11845_Tlp117 RDFKNTNIN-------SLERLSQAIARFSYEELDTQEKL-MHNTGKLLKSFRDAGNY-LA 105

Clari_RM16712_Tlp101 RDFKNTHSH-------ALKQLSQTITRLSYQELNTQEKL-MNNTGELLKTVRDMNNY-LA 105

Clari_CCUG22395_Tlp122 RDFKNLNTH-------SLEKLSQAILKLPYDALNTQDKL-MQNTGNLLKAVRDMNSY-LA 105

Clari_LMG11760_Tlp101 RDFKNLHTH-------SLEQLSQAILKLPYNELNTQEKL-MENTGSLLKTVRDINSY-LA 105

Clari_SlaughterBeach_Tlp101 RDFKSLHTH-------SLEQLSQAILRLPYNELNTQEKL-MENTGDLLKTVRDINSY-LA 105

Clari_SlaughterBeach_Tlp102 RDFKSLHTH-------SLEQLSQAILRLPYNELNTQEKL-MENTGDLLKTVRDINSY-LA 105

Clari_RM16701_Tlp101 RDFKSLHTH-------SLEQLSQAILRLPYNDLNTQEKL-MENTGGLLKTVRDINSY-LA 105

Clari_RM2100_TLp113 ENFRESTSN-------SLLKLSETILRNPYSNLNSQEAL-AQNVGVQLKAFRDAGNY-LA 105

Clari_NCTC11845_Tlp116 EEFRENTTT-------TLKKLSETILRHPYSELNTQESL-IQNVSSQLRAFRDAGGF-LT 105

: : :

Clari_NCTC11845_Tlp115 IYIHIIDISKFNNIDPTLLTDSGQFLMLINDTDLKNK-----GGI-KLIQADDRILNQRS 160

Clari_SlaughterBeach_Tlp100 AYIHLN---QYHDNNPLNLTPSGKFLLLAKDENPQQK-----GSI-KFIQAEEVILQQNS 147

Clari_RM16701_Tlp100 AYIHLN---QYHSNNPLNLTPSGKFLLLAKDENPQQK-----GSI-NFIQAEEIIMQQNS 157

Clari_RM2100_Tlp110 VFLAT---------------EDGITYDSTGV-------------K--KTLNNGFDGRSRS 131

Clari_NCTC11845_Tlp118 LYVGLA--------------KNGAMIRSNGN-------------H--QMPSDGYDPRTRT 129

Clari_RM16712_Tlp105 LYVGLA--------------KNGAMIRSNGN-------------H--QMPSDGYDPRTRT 133

Clari_RM16701_Tlp105 LYVGLA--------------KNGAMIRSNGN-------------H--QMPSDGYDPRTRT 133

Clari_Slaughter_Beach_Tlp105 LYVGLA--------------KNGAMIRSNGN-------------H--QMPSDGYDPRTRT 133

Clari_CCUG22395_Tlp105 LYVGLA--------------KNGAMIRSNGN-------------H--QMPSDGYDPRTRT 133

Clari_RM16712_Tlp103 IYFALE--------------ENGKHYKSDHT-------------Y--LDKSKGFDVKTRA 130

Clari_RM16701_Tlp103 IYFALE--------------ENGKHYKSDHT-------------Y--LDKSKGFDVKTRA 130

Clari_RM1607_Tlp114 VHFGLE--------------DSGKDYQIDTK-------------FNLHSDPSKFDPRTRP 135

Clari_RM2100_Tlp109 TYIGFE--------------EDGKLYQSNKI-------------I-RSPEQTGFDARTRP 133

Clari_LMG11760_Tlp109 TYIGFE--------------EDGKLYQSNKI-------------I-RSPEQTGFDARTRP 133

Clari_NCTC11845_Tlp119 TYVGFE--------------KDGKLYQSNKI-------------I-RSPEASGFDARTRP 133

Clari_SlaughterBeach_Tlp104 VYLGFD--------------INGKLYQSNRI-------------V-RSPEETGFDARTRS 133

Clari_RM16701_Tlp104 VYLGFD--------------INGKLYQSNKI-------------V-RSPEETGFDARTRS 133

Clari_RM16712_Tlp104 VYIGFN--------------DDGKLYQSNRI-------------I-RSPEETGFDARTRS 133

Clari_CCUG22395_Tlp104 VYLGFN--------------NDGKLYQSNRI-------------I-RSPEETGFDARTRS 133

Clari_RM2100_Tlp112 VYLGFP--------------D-GTMLVSDAESDKKGIPFRKRGGGISHYDDPQYNATTRD 156

Clari_RM16712_Tlp106 VYLGFP--------------D-GTMLVSDADSDKKEIPFRKRGGGIAHYDDPKYNATSRD 155

Clari_CCUG22395_Tlp121 VYVGFP--------------D-GTMLVSDTVSDKKGVNFRKRGGGISSYDDPSYDATSRD 149

Clari_RM2100_Tlp123 TFIGLD--------------N-GENIVSDNSSDNKNTNVVIYGK------AINYDTRTRE 145

Clari_LMG11760_Tlp107 VFIGLD--------------N-GENIVSDNNSDQKNRNVGIYGK------AMNYDTRTRS 145

Clari_CCUG22395_Tlp120 AFISLD--------------N-GENLVSNDTSDKNNKNIEIYGQ------NINYDARTRP 145

Clari_RM2100_Tlp111 VYVGMP--------------S-GELITSDPRADEKQLNAFIFGR------AQNYNATTRG 143

Clari_NCTC11845_Tlp117 VYIAQP--------------N-GELIVSDPDSDSKGLEYGTYGK------ADNYDATTRE 144

Clari_RM16712_Tlp101 VYIAQP--------------N-GELIVSDPDSDSKGLNYGIYGK------ADNYDATTRE 144

Clari_CCUG22395_Tlp122 VYIAQP--------------N-GELIVSDPDSDSKGLDYGIYGK------ADNYDATTRE 144

Clari_LMG11760_Tlp101 VYIAQS--------------N-GELIVSDPDSDSKGLDYGIYGK------ADNYDATTRE 144

Clari_SlaughterBeach_Tlp101 VYIAQS--------------N-GELIVSDPDSDSKGLDYGIYGK------ADNYDATTRE 144

Clari_SlaughterBeach_Tlp102 VYIAQS--------------N-GELIVSDPDSDSKGLDYGIYGK------ADNYDATTRE 144

Clari_RM16701_Tlp101 VYIAQS--------------N-GELIVSDPDSDSKGLDYGIYGK------ADNYDATTRE 144

Clari_RM2100_TLp113 VYIAQP--------------D-GELVVSDPDSDSKNIDYGFYGK------ADGYDARTRE 144

Clari_NCTC11845_Tlp116 VYLAQP--------------D-GEVILTNAESDKLNQDIIIFGK------KQNFDARTRE 144

.. * .

Clari_NCTC11845_Tlp115 VKAALEKQEEGVGRPQNFVINN-EEILAYNIAIPIT-RNGKLLGVIGALGGLNTLQEELT 218

Clari_SlaughterBeach_Tlp100 LIKALQTKQPAVGRPRDYSING-EKLYLVNIVLPIFGKNNETIGAIGMLVRIDLLREELN 206

Clari_RM16701_Tlp100 LIKALHTKQPAVGRPRDYSING-EKLYLVNIVLPIFGKNNETIGAIGMLVRVDLLREELN 216

Clari_RM2100_Tlp110 WYIGAKK-NMGLYTSDPYKSVT-SGIEGIAYSAPLIING-KFKGVVAGVYSLEQYSADAL 188

Clari_NCTC11845_Tlp118 WYTSVASGEDKVVISKPYMAPSLK-APSLAFSYPIIIDG-KFMGAVGGNYDLNTFSDNVL 187

Clari_RM16712_Tlp105 WYTSVTSGEDKVVISKPYMAPSLK-APSLAFSYPIVVNG-KFIGAVGGNYDLNTFSDNVL 191

Clari_RM16701_Tlp105 WYTSVSSGENKVVISKPYMAPSLK-APSLAFSYPIVVDG-KFIGAVGGNYDLNTFSDNVL 191

Clari_Slaughter_Beach_Tlp105 WYTSVSSGENKVVISKPYMAPSLK-APSLAFSYPIVVDG-KFIGAVGGNYDLNTFSDNVL 191

Clari_CCUG22395_Tlp105 WYTSVSSGENKVVISKPYMAPSLK-APSLAFSYPIVVDG-KFIGAVGGNYDLNTFSDNVL 191

Clari_RM16712_Tlp103 WYINAKK-EGGLIVSDPYSSFA-DGKMKIAYAMPVFKND-KFIGVVGGDYDLERFSKDVL 187

Clari_RM16701_Tlp103 WYINAKK-EGKLIVSDPYSSFA-DGKMKIAYAVPVFKNN-QFIGVVGGDYDLERFSKDVL 187

Clari_RM1607_Tlp114 WYKDAKT-AGKLIVTDPYKSIVLNGQVVVTYSIPVFDNSKKFIGVVSGVYNLNTFSKDVL 194

Clari_RM2100_Tlp109 WYQEAKT-TGTLVVSDPYKSIE-DGSITISYTAPIYVNG-KLLAVVGGDYNLHTFAKDVL 190

Clari_LMG11760_Tlp109 WYQEAKA-TGTLVVSDPYKSIE-DGSITISYTAPIYVNG-KLLAVIGGDYNLHTFAKDVL 190

Clari_NCTC11845_Tlp119 WYQESQQ-AKTLTVSDPYKSIE-DDSITISYTAPIYNNG-KLIAVVGGDYNLEKFAKDVL 190

Clari_SlaughterBeach_Tlp104 WYQEAKA-AGKLVVSDPYKSVE-DDAVTISYTAPIFVNG-KLLAVVGGDYNLHTFSKDVL 190

Clari_RM16701_Tlp104 WYQEAKA-AGKLVVSDPYKSVE-DDAVTISYTAPIFVNG-KLLAVVGGDYNLHTFSKDVL 190

Clari_RM16712_Tlp104 WYQEAKA-AGKLVVSDPYKSIE-DGSITVSYTAPILVNG-KLLAVVGGDYDLERFSKDVL 190

Clari_CCUG22395_Tlp104 WYQEAKT-AGKLVVSDPYKSIE-DDSITVSYTAPIFVNG-KLLAVVGGDYDLERFSKDVL 190

Clari_RM2100_Tlp112 WYKGALK-NKGVFVSDVYEDSV-TKFPSFTYSVPIEKNG-KLVAVLGIDLLLTSLQKTFE 213

Clari_RM16712_Tlp106 WYKGALK-NDGIYISDVYEDSV-TKLPSFTYSVPIKKNG-KLVAVLGIDLLLTSLQKTFD 212

Clari_CCUG22395_Tlp121 WYKGAIA-NDGVFISDVYEDSV-TKLPSFTYSTPIKKNG-KLIGVLGVDLLLTSLQKTFE 206

Clari_RM2100_Tlp123 WYIEARK-TNKVFITFPYIDKA-TNQYVITYTKSISKDG-KFIGVIGVDIPIAIFKKTLK 202

Clari_LMG11760_Tlp107 WFIEAKK-TNNVFITTPYIDKA-TNQYVITYTKAIYKDN-RFIGVIGIDIPIKDLQKDFE 202

Clari_CCUG22395_Tlp120 WYIGAKE-NKDIFITSPYIDKA-TNQYVITYTKSIYKNG-NFVGIIGVDIPVKELQENFE 202

Clari_RM2100_Tlp111 WYRGAKE-KNGMYVSDVYVDAA-TNLPCLTYALPLYKDG-QFIGVAGIDVLVEELQKKIE 200

Clari_NCTC11845_Tlp117 FYIEAKK-KNGLYITAAYIDAT-TGLPCFTYAMPLIKDG-KFIGVLAIDVLVKDLQTEFS 201

Clari_RM16712_Tlp101 FYIEAKK-KNGLYITPSYIDVT-TGLPCFTYSMPLVKDG-KFIGILAIDVLVKDLQTEFS 201

Clari_CCUG22395_Tlp122 FYIEAKK-KNGLYITPSYIDVT-TGFPCFTYAMPLIKDG-KFLGILAIDVLVKDLQNEFS 201

Clari_LMG11760_Tlp101 FYIETRK-KNGLYITAAYIDAT-TGLPCFTYAMPLIKDG-KFIGVLAIDVLVKDLQEKFN 201

Clari_SlaughterBeach_Tlp101 FYIEARK-KNGLYITAAYIDAT-TGLPCFTYAMPLIKDG-KFIGVLAIDVLVKDLQEKFN 201

Clari_SlaughterBeach_Tlp102 FYIEARK-KNGLYITAAYIDAT-TGLPCFTYAMPLIKDG-KFIGVLAIDVLVKDLQEKFN 201

Clari_RM16701_Tlp101 FYIEARK-KNGLYITPSYIDAT-TGLPCFTYAMPLIKDG-KFIGVLAIDVLVKDLQEKFN 201

Clari_RM2100_TLp113 FYIEARK-KNGLFITASYIDAT-TGLPCFTYAMPLNKDG-KFVGILAIDVLVKDLIENLK 201

Clari_NCTC11845_Tlp116 WYQEAK--AKGIFVTPAYIDTT-TNLPCFTYAMSLSKDG-KFIGVLAIDVLVKDLEDGLK 200

: : : . . . . :

Clari_NCTC11845_Tlp115 NPERS----V-----------FKNDQRLLLGANGLIAVSPATDFIGKNITEINPHASAKT 263

Clari_SlaughterBeach_Tlp100 DPNKS----L-----------FANDQRLLISSDGLIISSPKAEYIGKIITEINPHPSAKT 251

Clari_RM16701_Tlp100 DPNKS----L-----------FANDQRLLISSDGLIISSPKAEYIGKIITEINPHPSAKT 261

Clari_RM2100_Tlp110 EVGKTENSFVAVYSQ-DGTTMFHQDPKLIL-TKTVLG-QNIA----------KAITE--- 232

Clari_NCTC11845_Tlp118 AMGKSQSGYTVVLDD-EGTILFHESSKDLL-TKTNLS-QNIV----------KTYLA--- 231

Clari_RM16712_Tlp105 AMGRSQSGYTVVLDD-EGTVLFHESSKALL-TKDDLS-QNIV----------KAYLS--- 235

Clari_RM16701_Tlp105 AMGRSQSGYTVVLDD-EGTVLFHESSKALL-TKDDLS-QNIV----------KAYLS--- 235

Clari_Slaughter_Beach_Tlp105 AMGRSQSGYTVVLDD-EGTVLFHESSKALL-TKDDLS-QNIV----------KAYLS--- 235

Clari_CCUG22395_Tlp105 AMGRSQSGYTVVLDD-EGTVLFHESSKALL-TKDDLS-QNIV----------KAYLS--- 235

Clari_RM16712_Tlp103 SVGKSSQAYTAIYDI-EGNAFFHPEVEKIG-KKDELS-ENIS----------VYMRA--- 231

Clari_RM16701_Tlp103 SVGKSSQAYTAIYDI-EGNAFFHPEIEKIG-KKDKFS-ENIS----------AYMKA--- 231

Clari_RM1607_Tlp114 AIGHSESSYAGVYDK-EGVIVFHEDKDRML-TKNDLS-INIA----------NAVKA--- 238

Clari_RM2100_Tlp109 ILGHSQSSYAAVYDK-EGQIIFHENKDLML-TKNDLS-INIA----------NAAKA--- 234

Clari_LMG11760_Tlp109 ILGHSQSSYAAVYDK-EGQIIFHENKDLML-TKNDLS-INIA----------NAAKA--- 234

Clari_NCTC11845_Tlp119 VIGHSNSSYAAVYDK-EGVIIFHEEKDRML-TKNDLS-INIA----------NAVKA--- 234

Clari_SlaughterBeach_Tlp104 AIGHSESSYAGVYDK-EGVIVFHEDKDRML-TKNDLS-INIA----------NAVKA--- 234

Clari_RM16701_Tlp104 AIGHSESSYAGVYDK-EGVIVFHEDKDRML-TKNDLS-INIA----------NAVKA--- 234

Clari_RM16712_Tlp104 VMGHSQSSYAAVYDKNDGSIIFHEDKDRML-TKNDLS-INIA----------NAVKS--- 235

Clari_CCUG22395_Tlp104 VMGHSQSSYAAVYDKNDGSIIFHEDKDRML-TKNDLS-INIA----------NAVKA--- 235

Clari_RM2100_Tlp112 KLP----GSVFVFDN-TSSIPFASNDKSLI-LKSYPNIDEIK----------NHHKI--- 254

Clari_RM16712_Tlp106 KLP----GNVFVFDL-ASSIPFASNDKTLI-MKDYPSINEIK----------KYHKM--- 253

Clari_CCUG22395_Tlp121 KLP----GNVFVFDT-ASSIPFASNNKSLI-LTQHPSIEEIK----------KYHQQ--- 247

Clari_RM2100_Tlp123 I-N----QEIAFFNQ-NEKV-FVAKNKQLLDPS--VDHSPVI----------NAHKQ--- 240

Clari_LMG11760_Tlp107 SMP----GNSFLFDH-NGKV-FVAKNKQLLDPS--VDHTPVL----------NAYKK--- 241

Clari_CCUG22395_Tlp120 NMP----GNSFLFDE-NGKI-FVAKNKQLLDSS--VDHGPVL----------NAHKQ--- 241

Clari_RM2100_Tlp111 RIP----GDVFIAND-SNYA-FVSSSKVYLGKVK--NVETAL----------GKYKE--- 239

Clari_NCTC11845_Tlp117 ELP----GRTFVFDH-EFNV-FAATDETLVSKEKNPDIITVA----------KAYEQ--- 242

Clari_RM16712_Tlp101 ELP----GRTFVFDQ-AYTV-FASTDKSLISAEQNPDIITVA----------KAYEK--- 242

Clari_CCUG22395_Tlp122 ELP----GRTFVFDQ-AYTV-FASTDKSLIGGEKNPDIVTVA----------KAYEK--- 242

Clari_LMG11760_Tlp101 ELP----GRTFVFDH-AYTV-FASTDKSLVGQEQNPDIVTVA----------KAYEN--- 242

Clari_SlaughterBeach_Tlp101 ELP----GRTFVFDH-AYTV-FASTDKSLVGGEQNPDIVTVA----------KAYEN--- 242

Clari_SlaughterBeach_Tlp102 ELP----GRTFVFDH-AYTV-FASTDKSLVGGEQNPDIVTVA----------KAYEN--- 242

Clari_RM16701_Tlp101 ELP----GRTFVFDQ-AYTV-FASTDKSLVGGEQNPDIVTVA----------KAYEN--- 242

Clari_RM2100_TLp113 QMP----GDSFVYDK-NRYA-FASTHKNYTGN--HPNISTIA----------DAFSK--- 240

Clari_NCTC11845_Tlp116 QMP----GASFVFDK-NNFA-FASTSKNYIAN--DPNVSIVA----------EAFSK--- 239

*

Clari_NCTC11845_Tlp115 LIELQKNQINTLFDFTPASTGNNNRAAIANFNLWDGANDYWSIVTMAPVESIQMPITKLA 323

Clari_SlaughterBeach_Tlp100 ILDMQSTKTNGLFTFIPASTNEENLAQLVNFDLWEGSNDHWSVVTIAPKKSVEKPADSLA 311

Clari_RM16701_Tlp100 ILDMQSTKTNGLFTFIPASTNEENLAQLVNFDLWEGSNNHWSVVTIAPKKSVEKPADSLA 321

Clari_RM2100_Tlp110 DPSLLDPENIDTLFYAKDDKGVTQAVLCDKTP-N----PNINICAMVENDTYTKASDLAL 287

Clari_NCTC11845_Tlp118 TPDGKDGKLSSEPMLIDDDNAPRKAVICQES--S----IGYNVCVIADEKIYNEPVNKAL 285

Clari_RM16712_Tlp105 TPEGKAGQLSNKPMIIEDGSAPRKAVICQES--S----IGYNVCVIADEKIYKDPINEAL 289

Clari_RM16701_Tlp105 TPEGKAGQLSSEPMIIEDGSAPRKAVICQES--S----TGYNVCVIADEKIYKDPVNKAL 289

Clari_Slaughter_Beach_Tlp105 TPEGKAGQLSSEPMIIEDGSAPRKAVICQES--S----TGYNVCVIADEKIYKDPVNKAL 289

Clari_CCUG22395_Tlp105 TPEGKAGQLSSEPMIIEDGSAPRKAVICQES--S----TGYNVCVIADEKIYKDPVNKAL 289

Clari_RM16712_Tlp103 NPHFLELNNENAIAYIPNEQGIMEAIMCTNSF-N----SKYKVCTVTKEKVYSEKVNEAL 286

Clari_RM16701_Tlp103 NPQFLDPKNESAIAYIPNEQGVMEAIMCTNSF-S----SKYKVCTVTKEKVYSEKANSAL 286

Clari_RM1607_Tlp114 NPDLIDPTKQETLFYAKDDQGKTQVVTCNQAL-N----PKYMVCSITDESVYTDAVNKVL 293

Clari_RM2100_Tlp109 NPDLIDPSKEDSLFYAKDGNNKIQVVTCVQAL-N----PKYMVCSITDESVYSDAVNEVL 289

Clari_LMG11760_Tlp109 NPDLIDPSKEDSLFYAKDGNDKTQVVTCVQAL-N----PKYMVCSITDESVYSDAVNEVL 289

Clari_NCTC11845_Tlp119 NPDLINPNKQETLFYAKDQQGKTQVVTCNQSL-N----DKYIVCSITDESIYTDAVNKVL 289

Clari_SlaughterBeach_Tlp104 NPDLIDPTKQETLFYAKDDQGKTQVVTCNQAL-N----PKYMVCSITDESVYTDAVNKVL 289

Clari_RM16701_Tlp104 NPDLIDPTKQETLFYAKDDQGKTQVVTCNQAL-N----PKYMVCSITDESVYTDAVNKVL 289

Clari_RM16712_Tlp104 DPDLIDPNKEESLFYAKDGAGKTQVVTCNQAL-N----PKYVVCSITDESVYTDAVNKVL 290

Clari_CCUG22395_Tlp104 DPDLIDPTKQETLFYAKDDQGKTQVATCNQTV-N----PKYMVCSITDESVYTDAVNKVL 290

Clari_RM2100_Tlp112 V------GDYKTFEYTGVDSGEKRFGVCANIN-NSNAHVSYVACAIQKQDDLDRLVIEDV 307

Clari_RM16712_Tlp106 V------GDYKNFEYTGISSNEKRFGICANIN-NSKARINYVACAIQKQDSLDKLVIKNA 306

Clari_CCUG22395_Tlp121 F------GDYKTFEYISVDTQEERFGICANID-NSKAKVSYIACATQKQDELEMLVLKDA 300

Clari_RM2100_Tlp123 N------GDYKFFEYG-LK-GQERLGICANIY-------DYRVCSTESAEIINKPIMQIA 285

Clari_LMG11760_Tlp107 N------GDYTFFEYG-LK-NKERLGICAQIS-------SYLVCSTESADIINEPIFKTA 286

Clari_CCUG22395_Tlp120 N------GDYSFFEYG-LK-GKERLGICAKIS-------SYLVCSTESADVINEPIFKTA 286

Clari_RM2100_Tlp111 F------GDFKPFMFTGQN-GNDRLGICSKLD-------KYSACIVTKMNLIEESSEKIA 285

Clari_NCTC11845_Tlp117 A------GDYNIFNYTTQK-GKDRFGICVKID-------SYTTCAGEDIEVIETPALKIA 288

Clari_RM16712_Tlp101 A------GDYNIFSYSTKN-GQDRFGICVKID-------NYTTCAGENIEVIKAPALKIA 288

Clari_CCUG22395_Tlp122 A------GNYNIFNYTTQN-GQDRFGICVKID-------DYTTCAGENIEVIEAPALKIA 288

Clari_LMG11760_Tlp101 T------GDYNIFNYTTQN-GGDRFGICVKID-------GYTTCAGENVEVIETPALKIA 288

Clari_SlaughterBeach_Tlp101 A------GNYNIFNYTTQN-GGDRFGICVKID-------GYTTCAGEDVEVIETPALKIA 288

Clari_SlaughterBeach_Tlp102 A------GNYNIFNYTTQN-GGDRFGICVKID-------GYTTCAGEDVEVIETPALKIA 288

Clari_RM16701_Tlp101 A------GDYNIFNYTTQN-GGDRFGICVKID-------GYTTCAGENVEVIETPALKIA 288

Clari_RM2100_TLp113 T------KNNEPFFYTSAE-GNERLALCNNSN-------DYTVCNVAYVDTINNSSEKIA 286

Clari_NCTC11845_Tlp116 T------KDGEPFYYTSKE-GSERLAMCDRVN-------GYTICNMTYIDTIDQSSEKIA 285

. . .

Clari_NCTC11845_Tlp115 ATIAMVSLFVIFAIALIVFFYINKAVSSRIVNLQNNLLYFFKFLNHEVKDTILSKDIKNN 383

Clari_SlaughterBeach_Tlp100 FIIFAISTIVLFIIISVIYFYVKKSVVGAIHKLQTGLNSFFDFINHKTKDSAM-IDVKTN 370

Clari_RM16701_Tlp100 FIIFAISVIVLFIIISVIYFYVKKSIVGTIHKLQTGLNSFFDFINHKTKDSAM-IDVKTN 380

Clari_RM2100_Tlp110 KTQLIVGFIALIIVLVLIKFFASY-LLNPIFIIQTGLNSFFDFINHKTKDSAM-INVNTN 345

Clari_NCTC11845_Tlp118 VNQIIIGIISLIIALIIVRFMISY-NLSPLQAIQTGLNSFFDFINHKTKDSAM-INVKTN 343

Clari_RM16712_Tlp105 IKQIIIGAISLVIALIVIRFMINY-NLSPLKKIQTGLNSFFDFINYKTKDSAM-IDVKTN 347

Clari_RM16701_Tlp105 VKQIIIGAISLVIALIVIRFMINY-NLSPLQKIQTGLNSFFDFINHKTKDSAM-IDVKTN 347

Clari_Slaughter_Beach_Tlp105 VKQIIIGAISLVIALIVIRFMINY-NLSPLQKIQTGLNSFFDFINHKTKDSAM-IDVKTN 347

Clari_CCUG22395_Tlp105 VKQIIIGVISLVIALIVIRFMINY-NLSPLQKIQTGLNSFFDFINHKTKDSAM-IDVKTN 347

Clari_RM16712_Tlp103 IKQIIIGAISLVIALIVIRFMINY-NLSPLKKIQTGLNSFFDFINYKTKDSAM-IDVKTN 344

Clari_RM16701_Tlp103 IKQVLAAFVAILIALVLIKIVISK-LLSPLQKIQTGLNSFFDFINHKTKDSAM-IDVKTN 344

Clari_RM1607_Tlp114 FQQIIIALIAIAVALLLVRFAIIK-NLKPIAVITTGLNSFFDFINHKTKDSAM-IDVKTN 351

Clari_RM2100_Tlp109 FQQVIIAFIAIIIALILVRFAIIK-NLKPIAVITAGLNSFFDFINHKTKDSAM-INVNTN 347

Clari_LMG11760_Tlp109 FQQVIIAFIAIIIALILVRFAIIK-NLKPIMIITAGLNSFFDFINHKTKDSAM-IDVKTN 347

Clari_NCTC11845_Tlp119 FQQIIIALIAIAIALILIRFTIMK-NLKPIAVITTGLNSFFDFINHKTKDSAM-INVKTN 347

Clari_SlaughterBeach_Tlp104 FQQIIIALIAIAVALLLVRFAIIK-NLKPIAVITTGLNSFFDFINHKTKDSAM-IDVKTN 347

Clari_RM16701_Tlp104 FQQIIIALIAIAVALLLVRFAIIK-NLKPIAVITTGLNSFFDFINHKTKDSAM-IDVKTN 347

Clari_RM16712_Tlp104 FQQVIIALIAIIVALILVRFAIIK-NLKPIAVITTGLNSFFDFINYKTKDSAM-IDVKTN 348

Clari_CCUG22395_Tlp104 FQQIIIALIAIAVALLLVRFAIIK-NLKPIAVITTGLNSFFDFINHKTKDSAM-IDVKTN 348

Clari_RM2100_Tlp112 FDQIITSIVILILSCFIVYFISSR-LLSPLQVIQTGLNSFFDFINHKTKDSAM-INVNTN 365

Clari_RM16712_Tlp106 FEQTLISIVILILSCIFIHLFSAK-LLSPLQSIQTGLNSFFDFINYKTKDSAM-IDVKTN 364

Clari_CCUG22395_Tlp121 YKQIIFSIIILFISCFAIYFFSSK-LLSPLQAIQKGINSFFDFINHKTKDSAM-IDVKTN 358

Clari_RM2100_Tlp123 LTQAIVVIIMIVLSIAVLYFIVSR-YLSPLEKIQTGLNSFFDFINHKTKDSAM-INVNTN 343

Clari_LMG11760_Tlp107 SIQTIAVTVMVILSIILLYFIISY-YISPLQKIQTGLNSFFDFINHKTKDSAM-IDVKTN 344

Clari_CCUG22395_Tlp120 TIQTIVVSIMVALSVIILYFIISY-YLSPLQAIQKGINSFFDFINHKTKDSAM-IDVKTN 344

Clari_RM2100_Tlp111 YTQAIIVIFTSIISVILLYFIISR-YLSPLEKIQTGLNSFFDFINHKTKDSAM-IDVKTN 343

Clari_NCTC11845_Tlp117 YIQTSIVVFTSVASIILLYFIISY-YLSPLQAIQTGLNSFFDFINHKTKDSAM-INVKTN 346

Clari_RM16712_Tlp101 YIQATIVIFTSIASIVLLYFIISY-FLSPLQSIQTGLNSFFDFINYKTKDSAM-IDVKTN 346

Clari_CCUG22395_Tlp122 YIQTTIVIFTSIASIILLYFIISY-YLSPLQAIQTGLNSFFDFINHKTKDSAM-IDVKTN 346

Clari_LMG11760_Tlp101 YIQTIIVIFTSITSIVLLYFIISY-YLSPLQTIQNGLSSFFDFINHKTKDSAM-IDVKSN 346

Clari_SlaughterBeach_Tlp101 YIQTTIVIFTSIASIILLYFIISY-YLSPLQAIQTGLNSFFDFINHKTKDSAM-IDVKTN 346

Clari_SlaughterBeach_Tlp102 YIQTTIVIFTSIASIILLYFIISY-YLSPLQAIQTGLNSFFDFINHKTKDSAM-IDVKTN 346

Clari_RM16701_Tlp101 YIQTIIVIFTSIASIILLYFIISY-YLSPLQAIQKGLNSFFDFINHKTKDSAM-IDVKSN 346

Clari_RM2100_TLp113 YIQAIIVIFTSILSVVLLYFIVSR-YLSPLEKIQTGLNSFFDFINHKTKDSAM-INVNTN 344

Clari_NCTC11845_Tlp116 YIQAIIVIFTSIISVILLYFLVSH-YLSPLQAIQTGLNSFFDFINHKTKDSAM-INVKTN 343

. : : : : .: **.*:*::.**: : :::.*

Clari_NCTC11845_Tlp115 DELNTMAKAINENITKTKNALEQDAKAVEQSVDTAKEIENGNLTARITAIPANPQLIELK 443

Clari_SlaughterBeach_Tlp100 DELGAMAKAINENITKTKNALEQDAKAVEQSVDTAKEIEGGNLTARITAIPANPQLVELK 430

Clari_RM16701_Tlp100 DELGAMAKAINENITKTKNALEQDAKAVEQSVDTAKEIEGGNLTARITAIPANPQLVELK 440

Clari_RM2100_Tlp110 DEFGVIAKAINENITKTKNALEQDAKAVEQSVETAKEIEAGNLTARITAIPANPQLIELK 405

Clari_NCTC11845_Tlp118 DELGAMAKAINENITKTKNALEQDAKAVEQSVETVREVESGNLTARITAIPANPQLLELK 403

Clari_RM16712_Tlp105 DELGAMAKAINENITRTKNALEQDAKAVEQSVDTAKEIESGNLTARITAIPANPQLVELK 407

Clari_RM16701_Tlp105 DELGAMAKAINENITKTKNALEQDAKAVEQSVDTAKEIEGGNLTARITAIPANPQLVELK 407

Clari_Slaughter_Beach_Tlp105 DELGAMAKAINENITKTKNALEQDAKAVEQSVDTAKEIEGGNLTARITAIPANPQLVELK 407

Clari_CCUG22395_Tlp105 DELGAMAKAINENITKTKNALEQDAKAVEQSVDTAKEIESGNLTARITAIPANPQLIELK 407

Clari_RM16712_Tlp103 DELGAMAKAINENITRTKNALEQDAKAVEQSVDTAKEIESGNLTARITAIPANPQLVELK 404

Clari_RM16701_Tlp103 DELGAMAKAINENITKTKNALEQDAKAVEQSVDTAKEIEGGNLTARITAIPANPQLVELK 404

Clari_RM1607_Tlp114 DELGAMAKAINENITKTKNALEQDAKAVEQSVDTAKEIEGGNLTARITAIPANPQLVELK 411

Clari_RM2100_Tlp109 DELGAMAKAINENITKTKNALEQDAKAVEQSVETAKEIEAGNLTARITAIPANPQLIELK 407

Clari_LMG11760_Tlp109 DELGAMAKAINENITKTKNALEQDTKAVEQSVETAREIESGNLTARINAMPANPQLIELK 407

Clari_NCTC11845_Tlp119 DELGAMAKAINENITKTKNALEQDAKAVEQSVDTAKEIENGNLTARITAIPANPQLIELK 407

Clari_SlaughterBeach_Tlp104 DELGAMAKAINENITKTKNALEQDAKAVEQSVDTAKEIEGGNLTARITAIPANPQLVELK 407

Clari_RM16701_Tlp104 DELGAMAKAINENITKTKNALEQDAKAVEQSVDTAKEIEGGNLTARITAIPANPQLVELK 407

Clari_RM16712_Tlp104 DELGAMAKAINENITRTKNALEQDAKAVEQSVDTAKEIESGNLTARITAIPANPQLVELK 408

Clari_CCUG22395_Tlp104 DELGAMAKAINENITKTKNALEQDAKAVEQSVDTAKEIESGNLTARITAIPANPQLIELK 408

Clari_RM2100_Tlp112 DEFGAMAKAINENITKTKNALEQDAKAVEQSVETAKEIEHGNLTARITAIPANPQLIELK 425

Clari_RM16712_Tlp106 DELGAMAKAINENITRTKNALEQDAKAVEQSVDTAKEIESGNLTARITAIPANPQLVELK 424

Clari_CCUG22395_Tlp121 DELGAMAKAINENIIKTKNALEQDAKAVEQSVDTAKEIESGNLTARITAIPANPQLIELK 418

Clari_RM2100_Tlp123 DELGAMAKAINENITKTKNALEQDAKAVEQSVETAKEIEAGNLTARITAIPANPQLIELK 403

Clari_LMG11760_Tlp107 DELGAMAKAINENITKTKNALEQDTKAVEQSVETAREIESGNLTARINAMPANPQLIELK 404

Clari_CCUG22395_Tlp120 DEFGVIAKAINENITKTKNALEQDAKAVEQSVDTAKEIESGNLTARITAIPANPQLIELK 404

Clari_RM2100_Tlp111 DELGAMAKAINENITKTKNALEQDAKAVEQSVETAKEIEAGNLTARITAIPANPQLIELK 403

Clari_NCTC11845_Tlp117 DELGAMAKAINENITKTKNALEQDAKAVEQSVETVREVESGNLTARITAIPANPQLLELK 406

Clari_RM16712_Tlp101 DELGAMAKAINENITRTKNALEQDAKAVEQSVDTAKEIESGNLTARITAIPANPQLVELK 406

Clari_CCUG22395_Tlp122 DEFGVIAKAINENITKTKNALEQDAKAVEQSVDTAKEIESGNLTARITAIPANPQLIELK 406

Clari_LMG11760_Tlp101 DEFGAMAKAINENITKTKNALEQDTKAVEQSVETVREVEGGNLTARINAMPANPQLLKLK 406

Clari_SlaughterBeach_Tlp101 DELGAIAKAINENITKTKNALEQDAKAVEQSVDTVREVEGGNLTARITAIPAHPQLLELK 406

Clari_SlaughterBeach_Tlp102 DELGAIAKAINENITKTKNALEQDAKAVEQSVDTVREVEGGNLTARITAIPAHPQLLELK 406

Clari_RM16701_Tlp101 DEFGVIAKAINENITKTKNALEQDAKAVEQSVETVREVEGGNLAARITAMPAHPQLLELK 406

Clari_RM2100_TLp113 DELGAMAKAINENITKTKNALEQDAKAVEQSVDTAKEIESGNLTARITAIPANPQLIELK 404

Clari_NCTC11845_Tlp116 DEFGAMAKAINENITKTKNALEQDAKAVEQSVETVREVESGNLTARITAIPANPQLLELK 403

**:..:******** :********:*******:*.:*:* ***:***.*:**:***::**

Clari_NCTC11845_Tlp115 NVLNDMLSVLEQKVGSNMNEINRVFDSYKALDFTTEVKNAKGGVEVTTNVLGQEIVAMLR 503

Clari_SlaughterBeach_Tlp100 NVLNEMLNVLEQKVGSNMNEINRVFDSYKALDFTTEVKNAKGGVEVTTNVLGQEIVAMLR 490

Clari_RM16701_Tlp100 NVLNEMLNVLEQKVGSNMNEINRVFDSYKALDFTTEVKNAKGGVEVTTNVLGQEIVAMLR 500

Clari_RM2100_Tlp110 NVLNDMLSVLEEKVGSNMNEINRVFDSYKALDFTTEVANAKGGVEITTNVLGQEIVAMLR 465

Clari_NCTC11845_Tlp118 NYLNEMLSVLEQKVGSNMNEINRVFDSYKALDFTTEVKNAKGGVEVTTNVLGQEIVAMLR 463

Clari_RM16712_Tlp105 NVLNDMLNVLEQKVGSNMNEINRVFDSYKALDFTTEVKNAKGGVEVTTNVLGQEIVAMLR 467

Clari_RM16701_Tlp105 NVLNDMLNVLEQKVGSNMNEINRVFDSYKALDFTTEVKNAKGGVEVTTNVLGQEIVAMLR 467

Clari_Slaughter_Beach_Tlp105 NVLNEMLNVLEQKVGSNMNEINRVFDSYKALDFTTEVKNAKGGVEVTTNVLGQEIVAMLR 467

Clari_CCUG22395_Tlp105 NVLNEMLNVLEQKVGSNMNEINRVFDSYKALDFTTEVKNAKGGVEVTTNVLGQEIVAMLR 467

Clari_RM16712_Tlp103 NVLNDMLNVLEQKVGSNMNEINRVFDSYKALDFTTEVKNAKGGVEVTTNVLGQEIVAMLR 464

Clari_RM16701_Tlp103 NVLNEMLNVLEQKVGSNMNEINRVFDSYKALDFTTEVKNAKGGVEVTTNVLGQEIVAMLR 464

Clari_RM1607_Tlp114 NVLNEMLNVLEQKVGSNMNEINRVFDSYKALDFTTEVKNAKGGVEVTTNVLGQEIVAMLR 471

Clari_RM2100_Tlp109 NVLNDMLSVLEEKVGSNMNEINRVFDSYKALDFTTEVANAKGGVEITTNVLGQEIVAMLR 467

Clari_LMG11760_Tlp109 NVLNEMLNVLEQKVGSNMNEINRVFDSYKALDFTTEVANAKGGVEVTTNVLGKEIVAMLR 467

Clari_NCTC11845_Tlp119 NVLNDMLSVLEQKVGSNMNEINRVFDSYKALDFTTEVKNAKGGVEVTTNVLGQEIVAMLR 467

Clari_SlaughterBeach_Tlp104 NVLNEMLNVLEQKVGSNMNEINRVFDSYKALDFTTEVKNAKGGVEVTTNVLGQEIVAMLR 467

Clari_RM16701_Tlp104 NVLNDMLNVLEQKVGSNMNEINRVFDSYKALDFTTEVKNAKGGVEVTTNVLGQEIVAMLR 467

Clari_RM16712_Tlp104 NVLNDMLNVLEQKVGSNMNEINRVFDSYKALDFTTEVKNAKGGVEVTTNVLGQEIVAMLR 468

Clari_CCUG22395_Tlp104 NVLNEMLNVLEQKVGSNMNEINRVFDSYKALDFTTEVKNAKGGVEVTTNVLGQEIVAMLR 468

Clari_RM2100_Tlp112 NVLNNMLSVLEEKVGSNMNEINRVFDSYKALDFTTEVANAKGEVEITTNVLGQEIVNMLR 485

Clari_RM16712_Tlp106 NVLNDMLNVLEQKVGSNMNEINRVFDSYKALDFTTEVKNAKGGVEVTTNVLGQEIVAMLR 484

Clari_CCUG22395_Tlp121 NVLNEMLNVLEQKVGSNMNEINRVFDSYKALDFTTEVKNAKGEVEVTTNVLGQEIVAMLR 478

Clari_RM2100_Tlp123 NVLNDMLSVLEEKVGSNMNEINRVFDSYKALDFTTEVANAKGGVEITTNVLGQEIVAMLR 463

Clari_LMG11760_Tlp107 NVLNEMLNVLEQKVGSNMNEINRVFDSYKALDFTTEVANAKGGVEVTTNVLGKEIVAMLR 464

Clari_CCUG22395_Tlp120 NVLNEMLNVLEQKVGSNMNEINRVFDSYKALDFTTEVKNAKGGVEVTTNVLGQEIVAMLR 464

Clari_RM2100_Tlp111 NVLNDMLSVLEEKVGSNMNEINRVFDSYKALDFTTEVANAKGGVEITTNVLGQEIVAMLR 463

Clari_NCTC11845_Tlp117 NYLNEMLSVLEQKVGSNMNEINRVFDSYKALDFTTEVKNAKGGVEVTANVLGQEIVAMLR 466

Clari_RM16712_Tlp101 NVLNDMLNVLEQKVGSNMNEINRVFDSYKALDFTTEVKNAKGGVEVTTNVLGQEIVAMLR 466

Clari_CCUG22395_Tlp122 NVLNEMLNVLEQKVGSNMNEINRVFDSYKALDFTTEVKNAKGGVEVTANVLGQEIVAMLR 466

Clari_LMG11760_Tlp101 NYLNEMLNVLEQKVGSNMNEINRVFDSYKALDFTTEVANAKGGVEVTANALGKEIVAMLR 466

Clari_SlaughterBeach_Tlp101 NYINEMLNVLEQKVGSNMNEINRVFDSYKALDFTTEVKNAKGGVEVTTNVLGQEIVAMLR 466

Clari_SlaughterBeach_Tlp102 NYINEMLNVLEQKVGSNMNEINRVFDSYKALDFTTEVKNAKGGVEVTTNVLGQEIVAMLR 466

Clari_RM16701_Tlp101 NYLNEMLAVLEQKVGSNMNEINRVFDSYKALDFTTEVKNAKGGVEVTANALGQEIVAMLR 466

Clari_RM2100_TLp113 NVLNEMLSVLEEKVGSNMNEINRVLDSYKALDFTTEVTNAKGGVEITTNVLGQEIVAMLR 464

Clari_NCTC11845_Tlp116 NYLNEMLSVLEQKVGSNMNEINRVFDSYKALDFTTEVKNAKGGVEVTTNVLGQEIVAMLR 463

* :*:** ***:************:************ **** **:*:*.**:*** ***

Clari_NCTC11845_Tlp115 QSSEFASLLADESGKLQSAVKDLTDSSSSQASSLEETAAALEEITSSMQNVSHKTSEVIA 563

Clari_SlaughterBeach_Tlp100 QSSEFASLLADESGKLQSAVKNLTDSSSSQASSLEETAAALEEITSSMQNVSHKTSEVIA 550

Clari_RM16701_Tlp100 QSSEFASLLADESGKLQSAVKNLTDSSSSQASSLEETAAALEEITSSMQNVSHKTSEVIA 560

Clari_RM2100_Tlp110 QSSEFANLLATQSGKLQSAVRELTDSSSSQASSLEETAAALEEITSSMQNVSHKTSEVIA 525

Clari_NCTC11845_Tlp118 QSSEFASLLADESGKLQSAVKDLTDSSSSQASSLEETAAALEEITSSMQNVSHKTSEVIA 523

Clari_RM16712_Tlp105 QSSEFASLLADESGKLQSAVKDLTDSSSSQASSLEETAAALEEITSSMQNVSHKTSEVIA 527

Clari_RM16701_Tlp105 QSSEFASLLADESGKLQSAVKNLTDSSSSQASSLEETAAALEEITSSMQNVSHKTSEVIA 527

Clari_Slaughter_Beach_Tlp105 QSSEFASLLADESGKLQSAVKNLTDSSSSQASSLEETAAALEEITSSMQNVSHKTSEVIA 527

Clari_CCUG22395_Tlp105 QSSEFASLLADESGKLQSAVKNLTDSSSSQASSLEETAAALEEITSSMQNVSHKTSEVIA 527

Clari_RM16712_Tlp103 QSSEFASLLADESGKLQSAVKDLTDSSSSQASSLEETAAALEEITSSMQNVSHKTSEVIA 524

Clari_RM16701_Tlp103 QSSEFASLLADESGKLQSAVKNLTDSSSSQASSLEETAAALEEITSSMQNVSHKTSEVIA 524

Clari_RM1607_Tlp114 QSSEFASLLADESGKLQSAVKNLTDSSSSQASSLEETAAALEEITSSMQNVSHKTSEVIA 531

Clari_RM2100_Tlp109 QSSEFANLLATQSGKLQSAVRELTDSSSSQASSLEETAAALEEITSSMQNVSHKTSEVIA 527

Clari_LMG11760_Tlp109 QSSEFANLLASESGKLQSAVKNLTDSSSSQASSLEETAAALEEITSSMQNVSHKTSEVIA 527

Clari_NCTC11845_Tlp119 QSSEFASLLADESGKLQSAVKDLTDSSSSQASSLEETAAALEEITSSMQNVSHKTSEVIA 527

Clari_SlaughterBeach_Tlp104 QSSEFASLLADESGKLQSAVKNLTDSSSSQASSLEETAAALEEITSSMQNVSHKTSEVIA 527

Clari_RM16701_Tlp104 QSSEFASLLADESGKLQSAVKNLTDSSSSQASSLEETAAALEEITSSMQNVSHKTSEVIA 527

Clari_RM16712_Tlp104 QSSEFASLLADESGKLQSAVKDLTDSSSSQASSLEETAAALEEITSSMQNVSHKTSEVIA 528

Clari_CCUG22395_Tlp104 QSSEFASLLADESGKLQSAVKNLTDSSSSQASSLEETAAALEEITSSMQNVSHKTSEVIA 528

Clari_RM2100_Tlp112 QSSEFANLLATQSGKLQSAVRELTDSSSSQASSLEETAAALEEITSSMQNVSSKTSEVIA 545

Clari_RM16712_Tlp106 QSSEFASLLADESGKLQSAVKDLTDSSSSQASSLEETAAALEEITSSMQNVSHKTSEVIA 544

Clari_CCUG22395_Tlp121 QSSEFASLLADESGKLQSAVKNLTDSSSSQASSLEETAAALEEITSSMQNVSHKTSEVIA 538

Clari_RM2100_Tlp123 QSSEFANLLATQSGKLQSAVRELTDSSSSQASSLEETAAALEEITSSMQNVSHKTSEVIA 523

Clari_LMG11760_Tlp107 QSSEFANLLASESGKLQSAVKNLTDSSSSQASSLEETAAALEEITSSMQNVSHKTSEVIA 524

Clari_CCUG22395_Tlp120 QSSEFASLLADESGKLQSAVKNLTDSSSSQASSLEETAAALEEITSSMQNVSHKTSEVIA 524

Clari_RM2100_Tlp111 QSSEFANLLATQSGKLQSAVRELTDSSSSQASSLEETAAALEEITSSMQNVSHKTSEVIA 523

Clari_NCTC11845_Tlp117 QSSEFASLLADESGKLQSAVKDLTDSSSSQASSLEETAAALEEITSSMQNVSHKTSEVIA 526

Clari_RM16712_Tlp101 QSSEFASLLADESGKLQSAVKDLTDSSSSQASSLEETAAALEEITSSMQNVSHKTSEVIA 526

Clari_CCUG22395_Tlp122 QSSEFASLLADESGKLQSAVKNLTDSSSSQASSLEETAAALEEITSSMQNVSHKTSEVIA 526

Clari_LMG11760_Tlp101 QSSEFANLLASESGKLQSAVKNLTDSSSSQASSLEETAAALEEITSSMQNVSHKTSEVIA 526

Clari_SlaughterBeach_Tlp101 QSSEFASLLADESGKLQSAVKNLTDSSSSQASSLEETAAALEEITSSMQNVSHKTSEVIA 526

Clari_SlaughterBeach_Tlp102 QSSEFASLLADESGKLQSAVKNLTDSSSSQASSLEETAAALEEITSSMQNVSHKTSEVIA 526

Clari_RM16701_Tlp101 QSSEFASLLADESGKLQSAVKNLTDSSSSQASSLEETAAALEEITSSMQNVSHKTSEVIA 526

Clari_RM2100_TLp113 QSSEFANLLATQSGKLQSAVRELTDSSSSQASSLEETAAALEEITSSMQNVSHKTSEVIA 524

Clari_NCTC11845_Tlp116 QSSEFASLLADESGKLQSAVKDLTDSSSSQASSLEETAAALEEITSSMQNVSHKTSEVIA 523

******.*** :********::****************************** *******

Clari_NCTC11845_Tlp115 QSEEIKNVTSIIGDIADQINLLALNAAIEAARAGEHGRGFAVVADEVRNLAERTQKSLGE 623

Clari_SlaughterBeach_Tlp100 QSEEIKNVTSIIGDIADQINLLALNAAIEAARAGEHGRGFAVVADEVRNLAERTQKSLGE 610

Clari_RM16701_Tlp100 QSEEIKNVTSIIGDIADQINLLALNAAIEAARAGEHGRGFAVVADEVRNLAERTQKSLGE 620

Clari_RM2100_Tlp110 QSEEIKNVTSIIGDIADQINLLALNAAIEAARAGEHGRGFAVVADEVRNLAERTQKSLGE 585

Clari_NCTC11845_Tlp118 QSEEIKNVTSIIGDIADQINLLALNAAIEAARAGEHGRGFAVVADEVRNLAERTQKSLGE 583

Clari_RM16712_Tlp105 QSEEIKNVTSIIGDIADQINLLALNAAIEAARAGEHGRGFAVVADEVRNLAERTQKSLGE 587

Clari_RM16701_Tlp105 QSEEIKNVTSIIGDIADQINLLALNAAIEAARAGEHGRGFAVVADEVRNLAERTQKSLGE 587

Clari_Slaughter_Beach_Tlp105 QSEEIKNVTSIIGDIADQINLLALNAAIEAARAGEHGRGFAVVADEVRNLAERTQKSLGE 587

Clari_CCUG22395_Tlp105 QSEEIKNVTSIIGDIADQINLLALNAAIEAARAGEHGRGFAVVADEVRNLAERTQKSLGE 587

Clari_RM16712_Tlp103 QSEEIKNVTSIIGDIADQINLLALNAAIEAARAGEHGRGFAVVADEVRNLAERTQKSLGE 584

Clari_RM16701_Tlp103 QSEEIKNVTSIIGDIADQINLLALNAAIEAARAGEHGRGFAVVADEVRNLAERTQKSLGE 584

Clari_RM1607_Tlp114 QSEEIKNVTSIIGDIADQINLLALNAAIEAARAGEHGRGFAVVADEVRNLAERTQKSLGE 591

Clari_RM2100_Tlp109 QSEEIKNVTSIIGDIADQINLLALNAAIEAARAGEHGRGFAVVADEVRNLAERTQKSLGE 587

Clari_LMG11760_Tlp109 QSEEIKNVTSIIGDIADQINLLALNAAIEAARAGEHGRGFAVVADEVRNLAERTQKSLGE 587

Clari_NCTC11845_Tlp119 QSEEIKNVTSIIGDIADQINLLALNAAIEAARAGEHGRGFAVVADEVRNLAERTQKSLGE 587

Clari_SlaughterBeach_Tlp104 QSEEIKNVTSIIGDIADQINLLALNAAIEAARAGEHGRGFAVVADEVRNLAERTQKSLGE 587

Clari_RM16701_Tlp104 QSEEIKNVTSIIGDIADQINLLALNAAIEAARAGEHGRGFAVVADEVRNLAERTQKSLGE 587

Clari_RM16712_Tlp104 QSEEIKNVTSIIGDIADQINLLALNAAIEAARAGEHGRGFAVVADEVRNLAERTQKSLGE 588

Clari_CCUG22395_Tlp104 QSEEIKNVTSIIGDIADQINLLALNAAIEAARAGEHGRGFAVVADEVRNLAERTQKSLGE 588

Clari_RM2100_Tlp112 QSEEIKNVTSIIGDIADQINLLALNAAIEAARAGEHGRGFAVVADEVRNLAERTQKSLGE 605

Clari_RM16712_Tlp106 QSEEIKNVTSIIGDIADQINLLALNAAIEAARAGEHGRGFAVVADEVRNLAERTQKSLGE 604

Clari_CCUG22395_Tlp121 QSEEIKNVTSIIGDIADQINLLALNAAIEAARAGEHGRGFAVVADEVRNLAERTQKSLGE 598

Clari_RM2100_Tlp123 QSEEIKNVTSIIGDIADQINLLALNAAIEAARAGEHGRGFAVVADEVRNLAERTQKSLGE 583

Clari_LMG11760_Tlp107 QSEEIKNVTSIIGDIADQINLLALNAAIEAARAGEHGRGFAVVADEVRNLAERTQKSLGE 584

Clari_CCUG22395_Tlp120 QSEEIKNVTSIIGDIADQINLLALNAAIEAARAGEHGRGFAVVADEVRNLAERTQKSLGE 584

Clari_RM2100_Tlp111 QSEEIKNVTSIIGDIADQINLLALNAAIEAARAGEHGRGFAVVADEVRNLAERTQKSLGE 583

Clari_NCTC11845_Tlp117 QSEEIKNVTSIIGDIADQINLLALNAAIEAARAGEHGRGFAVVADEVRNLAERTQKSLGE 586

Clari_RM16712_Tlp101 QSEEIKNVTSIIGDIADQINLLALNAAIEAARAGEHGRGFAVVADEVRNLAERTQKSLGE 586

Clari_CCUG22395_Tlp122 QSEEIKNVTSIIGDIADQINLLALNAAIEAARAGEHGRGFAVVADEVRNLAERTQKSLGE 586

Clari_LMG11760_Tlp101 QSEEIKNVTSIIGDIADQINLLALNAAIEAARAGEHGRGFAVVADEVRNLAERTQKSLGE 586

Clari_SlaughterBeach_Tlp101 QSEEIKNVTSIIGDIADQINLLALNAAIEAARAGEHGRGFAVVADEVRNLAERTQKSLGE 586

Clari_SlaughterBeach_Tlp102 QSEEIKNVTSIIGDIADQINLLALNAAIEAARAGEHGRGFAVVADEVRNLAERTQKSLGE 586

Clari_RM16701_Tlp101 QSEEIKNVTSIIGDIADQINLLALNAAIEAARAGEHGRGFAVVADEVRNLAERTQKSLGE 586

Clari_RM2100_TLp113 QSEEIKNVTSIIGDIADQINLLALNAAIEAARAGEHGRGFAVVADEVRNLAERTQKSLGE 584

Clari_NCTC11845_Tlp116 QSEEIKNVTSIIGDIADQINLLALNAAIEAARAGEHGRGFAVVADEVRNLAERTQKSLGE 583

************************************************************

Clari_NCTC11845_Tlp115 IEANTNILVQSINEMGESIKEQTTGITQINDAVAQIDHVTQENLKIAKDSATISDNVNKI 683

Clari_SlaughterBeach_Tlp100 IEANTNILVQSINEMGESIKEQTTGITQINDAVAQIDHVTQENLKIAKDSAIVADNVNKI 670

Clari_RM16701_Tlp100 IEANTNILVQSINEMGESIKEQTTGITQINDAVAQIDHVTQENLKIAKDSAIVADNVNKI 680

Clari_RM2100_Tlp110 IEANTNILVQSINEMGESIKEQTTGITQINDAVAQIDHVTQENLKIANDSAAISENVNKI 645

Clari_NCTC11845_Tlp118 IEANTNILVQSINEMGESIKEQTTGITQINDAVAQIDHVTQENLKIAKDSATISDNVNKI 643

Clari_RM16712_Tlp105 IEANTNILVQSINEMGESIKEQTTGITQINDAVAQIDHVTQENLKIAKDSAAISDNVNKI 647

Clari_RM16701_Tlp105 IEANTNILVQSINEMGESIKEQTTGITQINDAVAQIDHVTQENLKIAKDSAAISDNVNKI 647

Clari_Slaughter_Beach_Tlp105 IEANTNILVQSINEMGESIKEQTTGITQINDAVAQIDHVTQENLKIAKDSAAISDNVNKI 647

Clari_CCUG22395_Tlp105 IEANTNILVQSINEMGESIKEQTTGITQINDAVAQIDHVTQENLKIAKDSAAISDNVNKI 647

Clari_RM16712_Tlp103 IEANTNILVQSINEMGESIKEQTTGITQINDAVAQIDHVTQENLKIAKDSAAISDNVNKI 644

Clari_RM16701_Tlp103 IEANTNILVQSINEMGESIKEQTTGITQINDAVAQIDHVTQENLKIAKDSAAISDNVNKI 644

Clari_RM1607_Tlp114 IEANTNILVQSINEMGESIKEQTTGITQINDAVAQIDHVTQENLKIAKDSAIVADNVNKI 651

Clari_RM2100_Tlp109 IEANTNILVQSINEMGESIKEQTTGITQINDAVAQIDHVTQENLKIANDSAAISENVNKI 647

Clari_LMG11760_Tlp109 IEANTNILVQSINEMGESIKEQTTGITQINDAVAQIDHVTQENLKIANDSAAISDNVNKI 647

Clari_NCTC11845_Tlp119 IEANTNILVQSINEMGESIKEQTTGITQINDAVAQIDHVTQENLKIAKDSATISDNVNKI 647

Clari_SlaughterBeach_Tlp104 IEANTNILVQSINEMGESIKEQTTGITQINDAVAQIDHVTQENLKIAKDSAAISDNVNKI 647

Clari_RM16701_Tlp104 IEANTNILVQSINEMGESIKEQTTGITQINDAVAQIDHVTQENLKIAKDSAAISDNVNKI 647

Clari_RM16712_Tlp104 IEANTNILVQSINEMGESIKEQTTGITQINDAVAQIDHVTQENLKIAKDSAAISDNVNKI 648

Clari_CCUG22395_Tlp104 IEANTNILVQSINEMGESIKEQTTGITQINDAVAQIDHVTQENLKIAKDSAAISDNVNKI 648

Clari_RM2100_Tlp112 IEANTNILVQSINEMGESIKEQTTGITQINDAVAQIDHVTQENLKIAKDSAAISENVNQI 665

Clari_RM16712_Tlp106 IEANTNILVQSINEMGESIKEQTTGITQINDAVAQIDHVTQENLKIAKDSAAISDNVNKI 664

Clari_CCUG22395_Tlp121 IEANTNILVQSINEMGESIKEQTTGITQINDAVAQIDHVTQENLKIAKDSAAISDNVNKI 658

Clari_RM2100_Tlp123 IEANTNILVQSINEMGESIKEQTTGITQINDAVAQIDHVTQENLKIANDSAAISENVNKI 643

Clari_LMG11760_Tlp107 IEANTNILVQSINEMGESIKEQTTGITQINDAVAQIDHVTQENLKIANDSAIVADNVNKI 644

Clari_CCUG22395_Tlp120 IEANTNILVQSINEMGESIKEQTTGITQINDAVAQIDHVTQENLKIANDSAIVADNVNKI 644

Clari_RM2100_Tlp111 IEANTNILVQSINEMGESIKEQTTGITQINDAVAQIDHVTQENLKIANDSAAISENVNKI 643

Clari_NCTC11845_Tlp117 IEANTNILVQSINEMGESIKEQTTGITQINDAVAQIDHVTQENLKIAKDSATISDNVNKI 646

Clari_RM16712_Tlp101 IEANTNILVQSINEMGESIKEQTTGITQINDAVAQIDHVTQENLKIAKDSAAISDNVNKI 646

Clari_CCUG22395_Tlp122 IEANTNILVQSINEMGESIKEQTTGITQINDAVAQIDHVTQENLKIANDSAIVADNVNQI 646

Clari_LMG11760_Tlp101 IEANTNILVQSINEMGESIKEQTTGITQINDAVAQIDHVTQENLKIANDSAIVADNVNKI 646

Clari_SlaughterBeach_Tlp101 IEANTNILVQSINEMGESIKEQTTGITQINDAVAQIDHVTQENLKIAKDSAAISDNVNKI 646

Clari_SlaughterBeach_Tlp102 IEANTNILVQSINEMGESIKEQTTGITQINDAVAQIDHVTQENLKIAKDSAAISDNVNKI 646

Clari_RM16701_Tlp101 IEANTNILVQSINEMGESIKEQTTGITQINDAVAQIDHVTQENLKIAKDSAIVADNVNKI 646

Clari_RM2100_TLp113 IEANTNILVQSINEMGESIKEQTTGITQINDAVAQIDHVTQENLKIANDSAIVADNVNKI 644

Clari_NCTC11845_Tlp116 IEANTNILVQSINEMGESIKEQTTGITQINDAVAQIDHVTQENLKIAKDSATISDNVNKI 643

***********************************************:*** :::***:*

Clari_NCTC11845_Tlp115 ANDILEDARKKKF 696

Clari_SlaughterBeach_Tlp100 ASDILEDARKKKF 683

Clari_RM16701_Tlp100 ANDILEDARKKKF 693

Clari_RM2100_Tlp110 ANDILEDAKKKRF 658

Clari_NCTC11845_Tlp118 ANDILEDARKKKF 656

Clari_RM16712_Tlp105 ANDILEDARKKKF 660

Clari_RM16701_Tlp105 ANDILEDARKKKF 660

Clari_Slaughter_Beach_Tlp105 ANDILEDARKKKF 660

Clari_CCUG22395_Tlp105 ANDILEDARKKKF 660

Clari_RM16712_Tlp103 ANDILEDARKKKF 657

Clari_RM16701_Tlp103 ANDILEDARKKKF 657

Clari_RM1607_Tlp114 ANDILEDARKKKF 664

Clari_RM2100_Tlp109 ANDILEDAKKKRF 660

Clari_LMG11760_Tlp109 ANDILEDARKKKF 660

Clari_NCTC11845_Tlp119 ANDILEDARKKKF 660

Clari_SlaughterBeach_Tlp104 ANDILEDARKKKF 660

Clari_RM16701_Tlp104 ANDILEDARKKKF 660

Clari_RM16712_Tlp104 ANDILEDARKKKF 661

Clari_CCUG22395_Tlp104 ANDILEDARKKKF 661

Clari_RM2100_Tlp112 ANDILEDAKKKRF 678

Clari_RM16712_Tlp106 ANDILEDARKKKF 677

Clari_CCUG22395_Tlp121 ANDILEDARKKKF 671

Clari_RM2100_Tlp123 ANDILEDAKKKRF 656

Clari_LMG11760_Tlp107 ASDILEDARKKKF 657

Clari_CCUG22395_Tlp120 ASDILEDARKKKF 657

Clari_RM2100_Tlp111 ANDILEDAKKKRF 656

Clari_NCTC11845_Tlp117 ANDILEDARKKKF 659

Clari_RM16712_Tlp101 ANDILEDARKKKF 659

Clari_CCUG22395_Tlp122 ANDILEDARKKKF 659

Clari_LMG11760_Tlp101 ASDILEDARKKKF 659

Clari_SlaughterBeach_Tlp101 ANDILEDARKKKF 659

Clari_SlaughterBeach_Tlp102 ANDILEDARKKKF 659

Clari_RM16701_Tlp101 ASDILEDARKKKF 659

Clari_RM2100_TLp113 ASDILEDAKKKKF 657

Clari_NCTC11845_Tlp116 ANDILEDARKKKF 656

*.******:**:*
