# Supplementary material for: Diversity of transducer-like proteins (Tlps) in Campylobacter
Source: PLoS One. 2019 Mar 25;14(3):e0214228. doi: 10.1371/journal.pone.0214228 (PMC6433261; doi:10.1371/journal.pone.0214228)
Supplement: S2 Archive — (ZIP) [file pone.0214228.s016.zip › Alignment C.docx]

Alignment C. Comparison of Tlp1 and Tlp404 protein sequences

CLUSTAL O(1.2.4) multiple sequence alignment 2018/05/11

FDAARGOS_295_Tlp1 ------------------MFKSLNVGLKLVFSVAIVVVIGLVILISLVTKQVSQSITENA 42

14980A_Tlp1 ------------------MFKSLNIGLKLIFSVAAVVVIGLVILISLITKQVSQNITKNT 42

CJ677CC527_Tlp1 ------------------MFKSLNIGLKLIFSVATVVVIGLIILISLITKQVSQNITKNT 42

CJ677CC012_Tlp1 ------------------MFKSLNIGLKLIFSVATVVVIGLIILISLITKQVSQNITKNT 42

4031_Tlp1 ------------------MFKSLNIGLKLIFSVATVVVIGLVILISLITKQVSQNITKNT 42

81116_Tlp1 ------------------MFKSLNIGLKLIFSVATVVVIGLVILISLITKQVSQNITKNT 42

35925B2_Tlp1 ------------------MFKSLNIGLKLIFSVATVVVIGLVILISLITKQVSQNITKNT 42

M1_Tlp1 ------------------MFKSLNIGLKLIFSVATVVVIGLVILISLITKQVSQNITKNT 42

PT14_Tlp1 ------------------MFKSLNIGLKLIFSVATVVVIGLVILISLITKQVSQNITKNT 42

81-176_Tlp1 ------------------MFKSLNIGLKLIFSVAAVVVIGLVILISLITKQVSQNITKNT 42

CVMN29710_Tlp1 ------------------MFKSLNIGLKLIFSVAAVVVIGLVILISLITKQVSQNITKNT 42

FB1_Tlp1 ------------------MFKSLNIGLKLIFSVAAVVVIGLVILISLITKQVSQNITKNT 42

BG2108_Tlp1 ------------------MFKSLNIGLKLIFSVAAVVVIGLVILISLITKQVSQNITKNT 42

YF2105_Tlp1 ------------------MFKSLNIGLKLIFSVAAVVVIGLVILISLITKQVSQNITKNT 42

YH501_Tlp1 ------------------MFKSLNIGLKLIFSVAAVVVIGLVILISLITKQVSQNITKNT 42

RM4661_Tlp1 ------------------MFKSLNIGLKLIFSVAAVVVIGLVILISLITKQVSQNITKNT 42

F38011_Tlp1 ------------------MFKSLNIGLKLIFSVAAVVVIGLVILISLITKQVSQNITKNT 42

T1-21_Tlp1 ------------------MFKSLNIGLKLIFSVAAVVVIGLVILISLITKQVSQNITKNT 42

ICDCCJ07001_Tlp1 ------------------MFKSLNIGLKLIFSVAAVVVIGLVILISLITKQVSQNITKNT 42

RM3196_Tlp1 ------------------MFKSLNIGLKLIFSVAAVVVIGLVILISLITKQVSQNITKNT 42

NCTC11168_Tlp1 ------------------MFKSLNIGLKLIFSVAAVVVIGLVILISLITKQVSQNITKNT 42

00-2425_Tlp1 ------------------MFKSLNIGLKLIFSVAAVVVIGLVILISLITKQVSQNITKNT 42

IA3902_Tlp1 ------------------MFKSLNIGLKLIFSVAAVVVIGLVILISLITKQVSQNITKNT 42

RM1285_Tlp1 ------------------MFKSLNIGLKLIFSVAAVVVIGLVILISLITKQVSQNITKNT 42

00-0949_Tlp1 ------------------MFKSLNIGLKLIFSVAAVVVIGLVILISLITKQVSQNITKNT 42

01-1512_Tlp1 ------------------MFKSLNIGLKLIFSVAAVVVIGLVILISLITKQVSQNITKNT 42

FDAARGOS_422_Tlp1 ------------------MFKSLNIGLKLIFSVAAVVVIGLVILISLITKQVSQNITKNT 42

FORC_056_Tlp1 ------------------MFKSLNIGLKLIFSVAAVVVIGLVILISLITKQVSQNITKNT 42

32488_Tlp1 ------------------MFKSLNIGLKLIFSVAAVVVIGLVILISLITKQVSQNITKNT 42

CFSAN032806_Tlp1 ------------------MFKSLNIGLKLIFSVAAVVVIGLVILISLITKQVSQNITKNT 42

YH001_Tlp1 ------------------MFKSLNIGLKLIFSVAAVVVIGLVILISLITKQVSQNITKNT 42

00-6200_Tlp1 ------------------MFKSLNIGLKLIFSVAAVVVIGLVILISLITKQVSQNITKNT 42

RM1221_Tlp1 ------------------MFKSLNIGLKLIFSVAAVVVIGLVILISLITKQVSQNITKNT 42

S3_Tlp1 ------------------MFKSLNIGLKLIFSVAAVVVIGLVILISLITKQVSQNITKNT 42

FDAARGOS_421_Tlp1 ------------------MFKSLNIGLKLIFSVAAVVVIGLVILISLITKQVSQNITKNT 42

FJ3124_Tlp1 ------------------MFKSLNIGLKLIFSVAAVVVIGLVILISLITKQVSQNITKNT 42

CG8421_Tlp1 ------------------MFKSLNIGLKLIFSVAAVVVIGLVILISLITKQVSQNITKNT 42

CJM1cam_Tlp1 ------------------MFKSLNIGLKLIFSVAAVVVIGLVILISLITKQVSQNITKNT 42

R14_Tlp1 ------------------MFKSLNIGLKLIFSVAAVVVIGLVILISLITKQVSQNITKNT 42

00-1597_Tlp1 ------------------MFKSLNIGLKLIFSVAAVVVIGLVILISLITKQVSQNITKNT 42

Cfetus_testudinum_pet-3_Tlp404 -------------------MKNLKLGTKLVLIVGIIITIGIAILSYIVARQTTSNMTKNA 41

Cfetus_testudinum_03-427_Tlp404 --------------MGGGALKNLKLGTKLVLIVGIIITIGIAILSYIVARQTTSNMTKNA 46

Cfetus_venerealis_84-112_Tlp404 -------------------MKNLKLGTKLVLIVGIIITIGIAILSYIVARQTSSNMTKNA 41

Cfetus_venerealis_01-165_Tlp404 MLNLISLNHKERKMMGGGALKNLKLGTKLVLIVGIIITIGIAILSYIVARQTSSNMTKNA 60

Cfetus_fetus_04-554_Tlp404 MLNLISLNHKERKMMGGGALKNLKLGTKLVLIVGIIITIGIAILSYIVARQTSSNMTKNA 60

Cfetus_fetus_82-40_Tlp404 MLNLISLNHKERKMMGGGALKNLKLGTKLVLIVGIIITIGIAILSYIVARQTSSNMTKNA 60

:*.*::* **:: *. ::.**: ** ::::*.:..:*:*:

FDAARGOS_295_Tlp1 EDIIASVSKEHAVQVQGIFNEIIALSKTVSNTLTEMFRVASKENLDMDSITNIVTNTFDN 102

14980A_Tlp1 EDILASITKEYATQTQGIFGEMIALNKSISGTLTEMFRSTSKEDLDIDNITNIITNTFDN 102

CJ677CC527_Tlp1 EDILASITKEYATQTQGIFGEMIALNKSISGTLTEMFRSSSKENLDIDSITNIITNTFDN 102

CJ677CC012_Tlp1 EDILASITKEYATQTQGIFGEMIALNKSISGTLTEMFRSSSKENLDIDSITNIITNTFDN 102

4031_Tlp1 EDILASITKEYATQTQGIFGEMIALNKSISGTLTEMFRSTSKEDLDIDNITNIITNTFDN 102

81116_Tlp1 EDILASITKEYATQTQGIFGEMIALNKSISGTLTEMFRSTSKEDLDIDNITNIITNTFDN 102

35925B2_Tlp1 EDILASITKEYATQTQGIFGEMIALNKSISGTLTEMFRSTSKEDLDIDNITNIITNTFDN 102

M1_Tlp1 EDILASITKEYATQTQGIFGEMIALNKSISGTLTEMFRSTSKEDLDIDNITNIITNTFDN 102

PT14_Tlp1 EDILASITKEYATQTQGIFGEMIALNKSISGTLTEMFRSTSKEDLDIDNITNIITNTFDN 102

81-176_Tlp1 EDILASITKEYATQTQGIFGEMIALNKSISGTLTEMFRSTSKEDLDIDNITNIITNTFDN 102

CVMN29710_Tlp1 EDILASITKEYATQTQGIFGEMIALNKSISGTLTEMFRSTSKEDLDIDNITNIITNTFDN 102

FB1_Tlp1 EDILASITKEYATQTQGIFGEMIALNKSISGTLTEMFRSTSKEDLDIDNITNIITNTFDN 102

BG2108_Tlp1 EDILASITKEYATQTQGIFGEMIALNKSISGTLTEMFRSTSKEDLDIDNITNIITNTFDN 102

YF2105_Tlp1 EDILASITKEYATQTQGIFGEMIALNKSISGTLTEMFRSTSKEDLDIDNITNIITNTFDN 102

YH501_Tlp1 EDILASITKEYATQTQGIFGEMIALNKSISGTLTEMFRSTSKEDLDIDNITNIITNTFDN 102

RM4661_Tlp1 EDILASITKEYATQTQGIFGEMIALNKSISGTLTEMFRSTSKEDLDIDNITNIITNTFDN 102

F38011_Tlp1 EDILASITKEYATQTQGIFGEMIALNKSISGTLTEMFRSTSKEDLDIDNITNIITNTFDN 102

T1-21_Tlp1 EDILASITKEYATQTQGIFGEMIALNKSISGTLTEMFRSTSKEDLDIDNITNIITNTFDN 102

ICDCCJ07001_Tlp1 EDILASITKEYATQTQGIFGEMIALNKSISGTLTEMFRSTSKEDLDIDNITNIITNTFDN 102

RM3196_Tlp1 EDILASITKEYATQTQGIFGEMIALNKSISGTLTEMFRSTSKEDLDIDNITNIITNTFDN 102

NCTC11168_Tlp1 EDILASITKEYATQTQGIFGEMIALNKSISGTLTEMFRSTSKEDLDIDNITNIITNTFDN 102

00-2425_Tlp1 EDILASITKEYATQTQGIFGEMIALNKSISGTLTEMFRSTSKEDLDIDNITNIITNTFDN 102

IA3902_Tlp1 EDILASITKEYATQTQGIFGEMIALNKSISGTLTEMFRSTSKEDLDIDNITNIITNTFDN 102

RM1285_Tlp1 EDILASITKEYATQTQGIFGEMIALNKSISGTLTEMFRSTSKEDLDIDNITNIITNTFDN 102

00-0949_Tlp1 EDILASITKEYATQTQGIFGEMIALNKSISGTLTEMFRSTSKEDLDIDNITNIITNTFDN 102

01-1512_Tlp1 EDILASITKEYATQTQGIFGEMIALNKSISGTLTEMFRSTSKEDLDIDNITNIITNTFDN 102

FDAARGOS_422_Tlp1 EDILASITKEYATQTQGIFGEMIALNKSISGTLTEMFRSTSKEDLDIDNITNIITNTFDN 102

FORC_056_Tlp1 EDILASITKEYATQTQGIFGEMIALNKSISGTLTEMFRSTSKEDLDIDNITNIITNTFDN 102

32488_Tlp1 EDILASITKEYATQTQGIFGEMIALNKSISGTLTEMFRSTSKEDLDIDNITNIITNTFDN 102

CFSAN032806_Tlp1 EDILASITKEYATQTQGIFGEMIALNKSISGTLTEMFRSTSKEDLDIDNITNIITNTFDN 102

YH001_Tlp1 EDILASITKEYATQTQGIFGEMIALNKSISGTLTEMFRSTSKEDLDIDNITNIITNTFDN 102

00-6200_Tlp1 EDILASITKEYATQTQGIFGEMIALNKSISGTLTEMFRSTSKEDLDIDNITNIITNTFDN 102

RM1221_Tlp1 EDILASITKEYATQTQGIFGEMIALNKSISGTLTEMFRSTSKEDLDIDNITNIITNTFDN 102

S3_Tlp1 EDILASITKEYATQTQGIFGEMIALNKSISGTLTEMFRSTSKEDLDIDNITNIITNTFDN 102

FDAARGOS_421_Tlp1 EDILASITKEYATQTQGIFGEMIALNKSISGTLTEMFRSTSKEDLDIDNITNIITNTFDN 102

FJ3124_Tlp1 EDILASITKEYATQTQGIFGEMIALNKSISGTLTEMFRSTSKEDLDIDNITNIITNTFDN 102

CG8421_Tlp1 EDILASITKEYATQTQGIFGEMIALNKSISGTLTEMFRSTSKEDLDIDNITNIITNTFDN 102

CJM1cam_Tlp1 EDILASITKEYATQTQGIFGEMIALNKSISGTLTEMFRSTSKEDLDIDNITNIITNTFDN 102

R14_Tlp1 EDILASITKEYATQTQGIFGEMIALNKSISGTLTEMFRSTSKEDLDIDNITNIITNTFDN 102

00-1597_Tlp1 EDILASITKEYATQTQGIFGEMIALNKSISGTLTEMFRSTSKEDLDIDNITNIITNTFDN 102

Cfetus_testudinum_pet-3_Tlp404 EHIITNDAFKYAAKIEGMMNEIIATTQSAHAVIDDFFHRIPSNEIKLENIESILSNVFDS 101

Cfetus_testudinum_03-427_Tlp404 EHIITNDAFKYAAKIEGMMNEIIATTQSAHAVIDDFFHRIPSNEIKLENIESILSNVFDS 106

Cfetus_venerealis_84-112_Tlp404 EYIITNDALKYAATIEGMMNEIIATTQSAHDVINDFFHRVPMNEIKLENIESILSNVFDS 101

Cfetus_venerealis_01-165_Tlp404 EYIITNDALKYAATIEGMMNEIIATTQSAHDVINDFFHRVPMNEIKLENIESILSNVFDS 120

Cfetus_fetus_04-554_Tlp404 EYIITNDAFKYAATIEGMMNEIIATTQSAHAVIDEFFHRVPMNEIKLENIESILSNVFDS 120

Cfetus_fetus_82-40_Tlp404 EYIITNDAFKYAATIEGMMNEIIATTQSAHAVIDDFFHRVPMNEIKLENIESILSNVFDS 120

* *::. : ::*. :*::.*:** .:: .: ::*: :::.::.* .*::*.**.

FDAARGOS_295_Tlp1 SVYSNFTYLYLIDPPEYFKEKSKFFNTQNGKFVMLYVDEETDNKGGIKAIQASDEIVNLQ 162

14980A_Tlp1 SAYSNFTYLYLIDPPEYFKEESKFFNTQSGKFVMLYADEEKDNKGGIKAIQASDEIANLQ 162

CJ677CC527_Tlp1 SAYSNFTYLYLIDPPEYFKEESKFFNTQSGKFVMLYVDEEKDSKGGIKAIQASDEIANLQ 162

CJ677CC012_Tlp1 SAYSNFTYLYLIDPPEYFKEESKFFNTQSGKFVMLYVDEEKDGKGGIKAIQASDEIANLQ 162

4031_Tlp1 SAYSNFTYLYLIDPPEYFKEESKFFNTQSGKFVMLYADEEKDNKGGIKAIQASDEIANLQ 162

81116_Tlp1 SAYSNFTYLYLIDPPEYFKEESKFFNTQSGKFVMLYADEEKDNKGGIKAIQASDEIANLQ 162

35925B2_Tlp1 SAYSNFTYLYLIDPPEYFKEESKFFNTQSGKFVMLYADEEKDNKGGIKAIQASDEIANLQ 162

M1_Tlp1 SAYSNFTYLYLIDPPEYFKEESKFFNTQSGKFVMLYADEEKDNKGGIKAIQASDEIANLQ 162

PT14_Tlp1 SAYSNFTYLYLIDPPEYFKEESKFFNTQSGKFVMLYADEEKDNKGGIKAIQASDEIANLQ 162

81-176_Tlp1 SAYSNFTYLYLIDPPEYFKEESKFFNTQSGKFVMLYADEEKDNKGGIKAIQASDEIANLQ 162

CVMN29710_Tlp1 SAYSNFTYLYLIDPPEYFKEESKFFNTQSGKFVMLYADEEKDNKGGIKAIQASDEIANLQ 162

FB1_Tlp1 SAYSNFTYLYLIDPPEYFKEESKFFNTQSGKFVMLYADEEKDNKGGIKAIQASDEIANLQ 162

BG2108_Tlp1 SAYSNFTYLYLIDPPEYFKEESKFFNTQSGKFVMLYADEEKDNKGGIKAIQASDEIANLQ 162

YF2105_Tlp1 SAYSNFTYLYLIDPPEYFKEESKFFNTQSGKFVMLYADEEKDNKGGIKAIQASDEIANLQ 162

YH501_Tlp1 SAYSNFTYLYLIDPPEYFKEESKFFNTQSGKFVMLYADEEKDNKGGIKAIQASDEIANLQ 162

RM4661_Tlp1 SAYSNFTYLYLIDPPEYFKEESKFFNTQSGKFVMLYADEEKDNKGGIKAIQASDEIANLQ 162

F38011_Tlp1 SVYSNFTYLYLIDPPEYFKEESKFFNTQSGKFVMLYADEEKDNKGGIKAIQASDEIANLQ 162

T1-21_Tlp1 SVYSNFTYLYLIDPPEYFKEESKFFNTQSGKFVMLYADEEKDNKGGIKAIQASDEIANLQ 162

ICDCCJ07001_Tlp1 SAYSNFTYLYLIDPPEYFKEESKFFNTQSGKFVMLYADEEKDNKGGIKAIQASDEIANLQ 162

RM3196_Tlp1 SAYSNFTYLYLIDPPEYFKEESKFFNTQSGKFVMLYADEEKDNKGGIKAIQASDEIANLQ 162

NCTC11168_Tlp1 SAYSNFTYLYLIDPPEYFKEESKFFNTQSGKFVMLYADEEKDNKGGIKAIQASDEIANLQ 162

00-2425_Tlp1 SAYSNFTYLYLIDPPEYFKEESKFFNTQSGKFVMLYADEEKDNKGGIKAIQASDEIANLQ 162

IA3902_Tlp1 SAYSNFTYLYLIDPPEYFKEESKFFNTQSGKFVMLYADEEKDNKGGIKAIQASDEIANLQ 162

RM1285_Tlp1 SAYSNFTYLYLIDPPEYFKEESKFFNTQSGKFVMLYADEEKDNKGGIKAIQASDEIANLQ 162

00-0949_Tlp1 SAYSNFTYLYLIDPPEYFKEESKFFNTQSGKFVMLYADEEKDNKGGIKAIQASDEIANLQ 162

01-1512_Tlp1 SAYSNFTYLYLIDPPEYFKEESKFFNTQSGKFVMLYADEEKDNKGGIKAIQASDEIANLQ 162

FDAARGOS_422_Tlp1 SAYSNFTYLYLIDPPEYFKEESKFFNTQSGKFVMLYADEEKDNKGGIKAIQASDEIANLQ 162

FORC_056_Tlp1 SAYSNFTYLYLIDPPEYFKEESKFFNTQSGKFVMLYADEEKDNKGGIKAIQASDEIANLQ 162

32488_Tlp1 SAYSNFTYLYLIDPPEYFKEESKFFNTQSGKFVMLYADEEKDNKGGIKAIQASDEIANLQ 162

CFSAN032806_Tlp1 SAYSNFTYLYLIDPPEYFKEESKFFNTQSGKFVMLYADEEKDNKGGIKAIQASDEIANLQ 162

YH001_Tlp1 SAYSNFTYLYLIDPPEYFKEESKFFNTQSGKFVMLYADEEKDNKGGIKAIQASDEIANLQ 162

00-6200_Tlp1 SAYSNFTYLYLIDPPEYFKEESKFFNTQSGKFVMLYADEEKDNKGGIKAIQASDEIANLQ 162

RM1221_Tlp1 SAYSNFTYLYLIDPPEYFKEESKFFNTQSGKFVMLYADEEKDNKGGIKAIQASDEIANLQ 162

S3_Tlp1 SAYSNFTYLYLIDPPEYFKEESKFFNTQSGKFVMLYADEEKDNKGGIKAIQASDEIANLQ 162

FDAARGOS_421_Tlp1 SAYSNFTYLYLIDPPEYFKEESKFFNTQSGKFVMLYADEEKDNKGGIKAIQASDEIANLQ 162

FJ3124_Tlp1 SAYSNFTYLYLIDPPEYFKEESKFFNTQSGKFVMLYADEEKDNKGGIKAIQASDEIANLQ 162

CG8421_Tlp1 SAYSNFTYLYLIDPPEYFKEESKFFNTQSGKFVMLYADEEKDNKGGIKAIQASDEIANLQ 162

CJM1cam_Tlp1 SAYSNFTYLYLIDPPEYFKEESKFFNTQSGKFVMLYADEEKDNKGGIKAIQASDEIANLQ 162

R14_Tlp1 SAYSNFTYLYLIDPPEYFKEESKFFNTQSGKFVMLYADEEKDNKGGIKAIQASDEIANLQ 162

00-1597_Tlp1 SAYSNFTYLYLIDPPEYFKEESKFFNTQSGKFVMLYADEEKDNKGGIKAIQASDEIANLQ 162

Cfetus_testudinum_pet-3_Tlp404 SLYADYAMLYLTNPPEQFKGISKY-TTESGKFLIVFHDEDTSKRGGIQAVQASDTTTNDS 160

Cfetus_testudinum_03-427_Tlp404 SLYADYAMLYLTNPPEQFKGISKY-TTESGKFLIVFHDEDTSKRGGIQAVQASDTTTNDS 165

Cfetus_venerealis_84-112_Tlp404 SLHANYAMLYLTNPPEQFKGINKY-TTESGKFLILFRDEDTSKKGGIESMQASDTAINDS 160

Cfetus_venerealis_01-165_Tlp404 SLHANYAMLYLTNPPEQFKGINKY-TTESGKFLILFRDEDTSKKGGIESMQASDTAINDS 179

Cfetus_fetus_04-554_Tlp404 SLYADYAMLYLTNPPEQFKGINKY-TTESGKFLILFRDEDTSKKGGIESMQASDTAINDS 179

Cfetus_fetus_82-40_Tlp404 SLYADYAMLYLTNPPEQFKGINKY-TTESGKFLILFHDEDTSKKGGIESMQASDTAINDS 179

* ::::: *** :*** ** .*: .*:.***:::: **:.. :***:::**** * .

FDAARGOS_295_Tlp1 VVQDILKKAKYGENKVYIGRPIRMNLEDQDFDAVNIAMPIFNRK-NQVVGVVGMTLDFSA 221

14980A_Tlp1 VVQDILKKAKYGENKVYIGRPIKMNLEGQDFDAVNLAMPIFDRK-NQVVGVIGMTLDFSD 221

CJ677CC527_Tlp1 VVQDILKKAKYGENKVYIGRPIRMNLEGQDFDAVNIAMPIFDRK-NQVVGVIGMTLDFSA 221

CJ677CC012_Tlp1 VVQDILKKAKYGENKVYIGRPIRMNLEGQDFDAVNIAMPIFDRK-NQVVGVIGMTLDFSA 221

4031_Tlp1 VVQDILKKAKYGENKVYIGRPIKMNLEGQDFDAVNVAMPIFDRK-NQVVGVIGMTLDFSA 221

81116_Tlp1 VVQDILKKAKYGENKVYIGRPIKMNLEGQDFDAVNVAMPIFDRK-NQVVGVIGMTLDFSA 221

35925B2_Tlp1 VVQDILKKAKYGENKVYIGRPIKMNLEGQDFDAVNVAMPIFDRK-NQVVGVIGMTLDFSA 221

M1_Tlp1 VVQDILKKAKYGENKVYIGRPIKMNLEGQDFDAVNVAMPIFDRK-NQVVGVIGMTLDFSA 221

PT14_Tlp1 VVQDILKKAKYGENKVYIGRPIKMNLEGQDFDAVNVAMPIFDRK-NQVVGVIGMTLDFSA 221

81-176_Tlp1 VVQDILKKAKYGENKVYIGRPIKMNLEGQDFDAVNVAMPIFDRK-NQVVGVIGMTLDFSD 221

CVMN29710_Tlp1 VVQDILKKAKYGENKVYIGRPIKMNLEGQDFNAVNVAMPIFDRK-NQVVGVIGMTLDFSD 221

FB1_Tlp1 VVQDILKKAKYGENKVYIGRPIKMNLEGQDFNAVNVAMPIFDRK-NQVVGVIGMTLDFSD 221

BG2108_Tlp1 VVQDILKKAKYGENKVYIGRPIKMNLEGQDFNAVNVAMPIFDRK-NQVVGVIGMTLDFSD 221

YF2105_Tlp1 VVQDILKKAKYGENKVYIGRPIKMNLEGQDFNAVNVAMPIFDRK-NQVVGVIGMTLDFSD 221

YH501_Tlp1 VVQDILKKAKYGENKVYIGRPIKMNLEGQDFNAVNVAMPIFDRK-NQVVGVIGMTLDFSD 221

RM4661_Tlp1 VVQDILKKAKYGENKVYIGRPIKMNLEGQDFNAVNVAMPIFDRK-NQVVGVIGMTLDFSD 221

F38011_Tlp1 VVQDILKKAKYGENKVYIGRPIKMNLEGQDFDAVNVAMPIFDRK-NQVVGVIGMTLDFSD 221

T1-21_Tlp1 VVQDILKKAKYGENKVYIGRPIKMNLEGQDFDAVNVAMPIFDRK-NQVVGVIGMTLDFSD 221

ICDCCJ07001_Tlp1 VVQDILKKAKYGENKVYIGRPIKMNLEGQDFDAVNVAMPIFDRK-NQVVGVIGMTLDFSD 221

RM3196_Tlp1 VVQDILKKAKYGENKVYIGRPIKMNLEGQDFDAVNVAMPIFDRK-NQVVGVIGMTLDFSD 221

NCTC11168_Tlp1 VVQDILKKAKYGENKVYIGRPIKMNLEGQDFDAVNVAIPIFDRK-NQVVGVIGMTLDFSD 221

00-2425_Tlp1 VVQDILKKAKYGENKVYIGRPIKMNLEGQDFDAVNVAIPIFDRK-NQVVGVIGMTLDFSD 221

IA3902_Tlp1 VVQDILKKAKYGENKVYIGRPIKMNLEGQDFDAVNVAIPIFDRK-NQVVGVIGMTLDFSD 221

RM1285_Tlp1 VVQDILKKAKYGENKVYIGRPIKMNLEGQDFDAVNVAIPIFDRK-NQVVGVIGMTLDFSD 221

00-0949_Tlp1 VVQDILKKAKYGENKVYIGRPIKMNLEGQDFDAVNVAIPIFDRK-NQVVGVIGMTLDFSD 221

01-1512_Tlp1 VVQDILKKAKYGENKVYIGRPIKMNLEGQDFDAVNVAIPIFDRK-NQVVGVIGMTLDFSD 221

FDAARGOS_422_Tlp1 VVQDILKKAKYGENKVYIGRPIKMNLEGQDFDAVNVAIPIFDRK-NQVVGVIGMTLDFSD 221

FORC_056_Tlp1 VVQDILKKAKYGENKVYIGRPIKMNLEGQDFDAVNVAMPIFDRK-NQVVGVIGMTLDFSD 221

32488_Tlp1 VVQDILKKAKYGENKVYIGHPIKMNLEGQDFDAVNVAMPIFDRK-NQVVGVIGMTLDFSD 221

CFSAN032806_Tlp1 VVQDILKKAKYGENKVYIGRPIKMNLEGQDFDAVNVAMPIFDRK-NQVVGVIGMTLDFSD 221

YH001_Tlp1 VVQDILKKAKYGENKVYIGRPIKMNLEGQDFDAVNVAMPIFDRK-NQVVGVIGMTLDFSD 221

00-6200_Tlp1 VVQDILKKAKYGENKVYIGRPIKMNLEGQDFDAVNVAMPIFDRK-NQVVGVIGMTLDFSD 221

RM1221_Tlp1 VVQDILKKAKYGENKVYIGRPIKMNLEGQDFDAVNVAMPIFDRK-NQVVGVIGMTLDFSD 221

S3_Tlp1 VVQDILKKAKYGENKVYIGRPIKMNLEGQDFDAVNVAMPIFDRK-NQVVGVIGMTLDFSD 221

FDAARGOS_421_Tlp1 VVQDILKKAKYGENKVYIGRPIKMNLEGQDFDAVNVAMPIFDRK-NQVVGVIGMTLDFSD 221

FJ3124_Tlp1 VVQDILKKAKYGENKVYIGRPIKMNLEGQDFDAVNVAMPIFDRK-NQVVGVIGMTLDFSD 221

CG8421_Tlp1 VVQDILKKAKYGENKVYIGRPIKMNLEGQDFDAVNVAMPIFDRK-NQVVGVIGMTLDFSD 221

CJM1cam_Tlp1 VVQDILKKAKYGENKVYIGRPIKMNLEGQDFDAVNVAMPIFDRK-NQVVGVIGMTLDFSD 221

R14_Tlp1 VVQDILKKAKYGENKVYIGRPIKMNLEGQDFDAVNVAMPIFDRK-NQVVGVIGMTLDFSD 221

00-1597_Tlp1 VVQDILKKAKYGENKVYIGRPIKMNLEGQDFDAVNVAMPIFDRK-NQVVGVIGMTLDFSD 221

Cfetus_testudinum_pet-3_Tlp404 IIKKALIEGKPNDNKVFVGSAEKISFGSDSFIGINVALPIFDNSSKKPIGVIAFSLDFKE 220

Cfetus_testudinum_03-427_Tlp404 IIKKALIEGKPNDNKVFVGSAEKISFGSDSFIGINVALPIFDNSSKKPIGVIAFSLDFKE 225

Cfetus_venerealis_84-112_Tlp404 ILKKALIEGNPNGNRVFVGNVEKISFGSNSFIGINVALPIFNNDSKKPIGVIAFSLNFKE 220

Cfetus_venerealis_01-165_Tlp404 ILKKALIEGNPNGNRVFVGNVEKISFGSNSFIGINVALPIFNNDSKKPIGVVAFSLNFKE 239

Cfetus_fetus_04-554_Tlp404 ILKKALIEGNPNGNRVFVGNVEKISFGSNSFIGINVALPIFNNDSKKPIGVIAFSLNFKE 239

Cfetus_fetus_82-40_Tlp404 ILKKALIEGNPNGNRVFVGNVEKISFGSNSFIGINVALPIFNNDSKKPIGVIAFSLNFKE 239

:::. * :.: . *:*::* ::.: .:.* .:*:*:***:.. :: :**:.::*:*.

FDAARGOS_295_Tlp1 IAAYLLDPKSQKYDGELRVLLNSDGFVAIHPNKNLVLKNLKDVNPNKGAQETYKAMSEGK 281

14980A_Tlp1 IATYLLDPKGQKYDGELRVLLNSDGLMAIHPNKNLVLKNLKDVNPNKGAQETYKAMSEGK 281

CJ677CC527_Tlp1 IATYLLDPKSQKYDGELRVLLNSDGFVAIHPNKNLVLKNLKDINPNKGARETYKAMSEGK 281

CJ677CC012_Tlp1 IATYLLDPKSQKYDGELRVLLNSDGFVAIHPNKNLVLKNLKDINPNKGARETYKAMSEGK 281

4031_Tlp1 IATYLLDPKSQKYNGELRILLNSDGLVAIHPNKNLVLKNLKDVNPNKGAQETYKAMSEGK 281

81116_Tlp1 IATYLLDPKSQKYNGELRILLNSDGLVAIHPNKNLVLKNLKDVNPNKGAQETYKAMSEGK 281

35925B2_Tlp1 IATYLLDPKSQKYNGELRILLNSDGLVAIHPNKNLVLKNLKDVNPNKGAQETYKAMSEGK 281

M1_Tlp1 IATYLLDPKSQKYNGELRILLNSDGLVAIHPNKNLVLKNLKDVNPNKGAQETYKAMSEGK 281

PT14_Tlp1 IATYLLDPKSQKYNGELRILLNSDGLVAIHPNKNLVLKNLKDVNPNKGAQETYKAMSEGK 281

81-176_Tlp1 IATYLLDPKGQKYDGELRVLLNSDGLMAIHPNKNLVLKNLKDVNPNKGAQETYKAISEGK 281

CVMN29710_Tlp1 IATYLLDPKGQKYDGELRVLLNSDGFMAIHPNKNLVLKNLKDVNPNKGAQETYKAISEGK 281

FB1_Tlp1 IATYLLDPKGQKYDGELRVLLNSDGFMAIHPNKNLVLKNLKDVNPNKGAQETYKAISEGK 281

BG2108_Tlp1 IATYLLDPKGQKYDGELRVLLNSDGFMAIHPNKNLVLKNLKDVNPNKGAQETYKAISEGK 281

YF2105_Tlp1 IATYLLDPKGQKYDGELRVLLNSDGFMAIHPNKNLVLKNLKDVNPNKGAQETYKAISEGK 281

YH501_Tlp1 IATYLLDPKGQKYDGELRVLLNSDGFMAIHPNKNLVLKNLKDVNPNKGAQETYKAISEGK 281

RM4661_Tlp1 IATYLLDPKGQKYDGELRVLLNSDGFMAIHPNKNLVLKNLKDVNPNKGAQETYKAISEGK 281

F38011_Tlp1 IATYLLDPKGQKYDGELRVLLNSDGFMAIHPNKNLVLKNLKDINPNKGAQETYKAISEGK 281

T1-21_Tlp1 IATYLLDPKGQKYDGELRVLLNSDGFMAIHPNKNLVLKNLKDINPNKGAQETYKAISEGK 281

ICDCCJ07001_Tlp1 IATYLLDPKGQKYDGELRVLLNSDGFMAIHPNKNLVLKNLKDINPNKGAQETYKAISEGK 281

RM3196_Tlp1 IATYLLDPKGQKYDGELRVLLNSDGFMAIHPNKNLVLKNLKDINPNKGAQETYKAISEGK 281

NCTC11168_Tlp1 IATYLLDPKGQKYDGELRVLLNSDGFMAIHPNKNLVLKNLKDINPNKGAQETYKAISEGK 281

00-2425_Tlp1 IATYLLDPKGQKYDGELRVLLNSDGFMAIHPNKNLVLKNLKDINPNKGAQETYKAISEGK 281

IA3902_Tlp1 IATYLLDPKGQKYDGELRVLLNSDGFMAIHPNKNLVLKNLKDINPNKGAQETYKAISEGK 281

RM1285_Tlp1 IATYLLDPKGQKYDGELRVLLNSDGFMAIHPNKNLVLKNLKDINPNKGAQETYKAISEGK 281

00-0949_Tlp1 IATYLLDPKGQKYDGELRVLLNSDGFMAIHPNKNLVLKNLKDINPNKGAQETYKAISEGK 281

01-1512_Tlp1 IATYLLDPKGQKYDGELRVLLNSDGFMAIHPNKNLVLKNLKDINPNKGAQETYKAISEGK 281

FDAARGOS_422_Tlp1 IATYLLDPKGQKYDGELRVLLNSDGFMAIHPNKNLVLKNLKDINPNKGAQETYKAISEGK 281

FORC_056_Tlp1 IATYLLDPKGQKYDGELRVLLNSDGFMAIHPNKNLVLKNLKDINPNKGAQETYKAISEGK 281

32488_Tlp1 IATYLLDPKGQKYDGELRVLLNSDGFMAIHPNKNLVLKNLKDINPNKGAQETYKAISEGK 281

CFSAN032806_Tlp1 IATYLLDPKGQKYDGELRVLLNSDGFMAIHPNKNLVLKNLKDINPNKGAQETYKAISEGK 281

YH001_Tlp1 IATYLLDPKGQKYDGELRVLLNSDGFMAIHPNKNLVLKNLKDINPNKGAQETYKAISEGK 281

00-6200_Tlp1 IATYLLDPKGQKYDGELRVLLNSDGFMAIHPNKNLVLKNLKDINPNKGAQETYKAISEGK 281

RM1221_Tlp1 IATYLLDPKGQKYDGELRVLLNSDGFMAIHPNKNLVLKNLKDINPNKGAQETYKAISEGK 281

S3_Tlp1 IATYLLDPKGQKYDGELRVLLNSDGFMAIHPNKNLVLKNLKDINPNKGAQETYKAISEGK 281

FDAARGOS_421_Tlp1 IATYLLDPKGQKYDGELRVLLNSDGFMAIHPNKNLVLKNLKDINPNKGAQETYKAISEGK 281

FJ3124_Tlp1 IATYLLDPKGQKYDGELRVLLNSDGFMAIHPNKNLVLKNLKDINPNKGAQETYKAISEGK 281

CG8421_Tlp1 IATYLLDPKGQKYDGELRVLLNSDGFMAIHPNKNLVLKNLKDINPNKGAQETYKAISEGK 281

CJM1cam_Tlp1 IATYLLDPKGQKYDGELRVLLNSDGFMAIHPNKNLVLKNLKDINPNKGAQETYKAISEGK 281

R14_Tlp1 IATYLLDPKGQKYDGELRVLLNSDGFMAIHPNKNLVLKNLKDINPNKGAQETYKAISEGK 281

00-1597_Tlp1 IATYLLDPKGQKYDGELRVLLNSDGFMAIHPNKNLVLKNLKDINPNKGAQETYKAISEGK 281

Cfetus_testudinum_pet-3_Tlp404 ISNYLLDNKLDSFSGSTKAIISKNGTIAVHDNSNILLKKIQDINPHAKVLSDAVANN-EF 279

Cfetus_testudinum_03-427_Tlp404 ISNYLLDNKLDSFSGSTKAIISKNGTIAVHDNSNILLKKIQDINPHAKVLSDAVANN-EF 284

Cfetus_venerealis_84-112_Tlp404 ISNFLLDNKLDSFSGYTKAIIAKDGTIAVHDNSNIILKKIQDINPHAKALADAVAKN-EF 279

Cfetus_venerealis_01-165_Tlp404 ISNFLLDNKLDSFSGYTKAIIAKDGTIAVHDNSNIILKKIQDINPHAKALADAVAKN-EF 298

Cfetus_fetus_04-554_Tlp404 ISNFLLDNKLDSFSGYTKAIIAKDGTIAVHDNSNIILKKIQDINPHAKALADAVAKN-EF 298

Cfetus_fetus_82-40_Tlp404 ISNFLLDNKLDSFSGYTKAIIAKDGTIAVHDNSNIILKKIQDINPHAKALADAVAKN-EF 298

*: :*** * :.:.* : :: .:* :*:* *.*::**:::*:**: . * .

FDAARGOS_295_Tlp1 NGVFNYIASDGDDSYAAINSFKV--QDSSWTVLVTAPKYSVFEPLKKLQLIIIGASFIFI 339

14980A_Tlp1 NGVFDYIASDGDDSYAAINTFKV--QDSSWTVLVTAPKYSVFEPLKKLQMIIISASLIFI 339

CJ677CC527_Tlp1 NGVFNYIAFDGDDSYAAINSFKV--QDSSWTVLVTAPKYSVFEPLKKLQLIIISASLIFI 339

CJ677CC012_Tlp1 NGVFNYIAFDGDDSYAAINSFKV--QDSSWTVLVTAPKYSVFEPLKKLQLIIISASLIFI 339

4031_Tlp1 NGVFNYIAFDGDDSYAAINSFKV--QDSSWTVLVTAPKYSVFEPLKKLQLIIIGASFIFI 339

81116_Tlp1 NGVFNYIAFDGDDSYAAINSFKV--QDSSWTVLVTAPKYSVFEPLKKLQLIIIGASFIFI 339

35925B2_Tlp1 NGVFNYIAFDGDDSYAAINSFKV--QDSSWTVLVTAPKYSVFEPLKKLQLIIIGASFIFI 339

M1_Tlp1 NGVFNYIAFDGDDSYAAINSFKV--QDSSWTVLVTAPKYSVFEPLKKLQLIIIGASFIFI 339

PT14_Tlp1 NGVFNYIAFDGDDSYAAINSFKV--QDSSWTVLVTAPKYSVFEPLKKLQLIIIGASFIFI 339

81-176_Tlp1 NGVFDYIASDGDDSYAAINSFKV--QDSSWAVLVTAPKYSVFKPLKKLQLIILGASFIFI 339

CVMN29710_Tlp1 NGVFDYIASDGDDSYAAINSFKV--QDSSWAVLVTAPKYSVFKPLKKLQLIILGASFIFI 339

FB1_Tlp1 NGVFDYIASDGDDSYAAINSFKV--QDSSWAVLVTAPKYSVFKPLKKLQLIILGASFIFI 339

BG2108_Tlp1 NGVFDYIASDGDDSYAAINSFKV--QDSSWAVLVTAPKYSVFKPLKKLQLIILGASFIFI 339

YF2105_Tlp1 NGVFDYIASDGDDSYAAINSFKV--QDSSWAVLVTAPKYSVFKPLKKLQLIILGASFIFI 339

YH501_Tlp1 NGVFDYIASDGDDSYAAINSFKV--QDSSWAVLVTAPKYSVFKPLKKLQLIILGASFIFI 339

RM4661_Tlp1 NGVFDYIASDGDDSYAAINSFKV--QDSSWAVLVTAPKYSVFKPLKKLQLIILGASFIFI 339

F38011_Tlp1 NGVFNYIASDGDDSYAAINSFKV--QDSSWAVLVTAPKYSVFKPLKKLQLIILGASFIFI 339

T1-21_Tlp1 NGVFNYIASDGDDSYAAINSFKV--QDSSWAVLVTAPKYSVFKPLKKLQLIILGASFIFI 339

ICDCCJ07001_Tlp1 NGVFNYIASDGDDSYAAINSFKV--QDSSWAVLVTTPKYSVFKPLKKLQLIILGASFIFI 339

RM3196_Tlp1 NGVFNYIASDGDDSYAAINSFKV--QDSSWAVLVTTPKYSVFKPLKKLQLIILGASFIFI 339

NCTC11168_Tlp1 NGVFNYIASDGDDSYAAINSFKV--QDSSWAVLVTAPKYSVFKPLKKLQLIILGASFIFI 339

00-2425_Tlp1 NGVFNYIASDGDDSYAAINSFKV--QDSSWAVLVTAPKYSVFKPLKKLQLIILGASFIFI 339

IA3902_Tlp1 NGVFNYIASDGDDSYAAINSFKV--QDSSWAVLVTAPKYSVFKPLKKLQLIILGASFIFI 339

RM1285_Tlp1 NGVFNYIASDGDDSYAAINSFKV--QDSSWAVLVTAPKYSVFKPLKKLQLIILGASFIFI 339

00-0949_Tlp1 NGVFNYIASDGDDSYAAINSFKV--QDSSWAVLVTAPKYSVFKPLKKLQLIILGASFIFI 339

01-1512_Tlp1 NGVFNYIASDGDDSYAAINSFKV--QDSSWAVLVTAPKYSVFKPLKKLQLIILGASFIFI 339

FDAARGOS_422_Tlp1 NGVFNYIASDGDDSYAAINSFKV--QDSSWAVLVTAPKYSVFKPLKKLQLIILGASFIFI 339

FORC_056_Tlp1 NGVFNYIASDGDDSYAAINSFKV--QDSSWAVLVTAPKYSVFKPLKKLQLIILGASFIFI 339

32488_Tlp1 NGVFNYIASDGDDSYAAINSFKV--QDSSWAVLVTAPKYSVFKPLKKLQLIILGASFIFI 339

CFSAN032806_Tlp1 NGVFNYIASDGDDSYAAINSFKV--QDSSWAVLVTAPKYSVFKPLKKLQLIILGASFIFI 339

YH001_Tlp1 NGVFNYIASDGDDSYAAINSFKV--QDSSWAVLVTAPKYSVFKPLKKLQLIILGASFIFI 339

00-6200_Tlp1 NGVFNYIASDGDDSYAAINSFKV--QDSSWAVLVTAPKYSVFKPLKKLQLIILGASFIFI 339

RM1221_Tlp1 NGVFNYIASDGDDSYAAINSFKV--QDSSWAVLVTAPKYSVFKPLKKLQLIILGASFIFI 339

S3_Tlp1 NGVFNYIASDGDDSYAAINSFKV--QDSSWAVLVTAPKYSVFKPLKKLQLIILGASFIFI 339

FDAARGOS_421_Tlp1 NGVFNYIASDGDDSYAAINSFKV--QDSSWAVLVTAPKYSVFKPLKKLQLIILGASFIFI 339

FJ3124_Tlp1 NGVFNYIASDGDDSYAAINSFKV--QDSSWAVLVTAPKYSVFKPLKKLQLIILGASFIFI 339

CG8421_Tlp1 NGVFNYIASDGDDSYAAINSFKV--QDSSWAVLVTAPKYSVFKPLKKLQLIILGASFIFI 339

CJM1cam_Tlp1 NGVFNYIASDGDDSYAAINSFKV--QDSSWAVLVTAPKYSVFKPLKKLQLIILGASFIFI 339

R14_Tlp1 NGVFNYIASDGDDSYAAINSFKV--QDSSWAVLVTAPKYSVFKPLKKLQLIILGASFIFI 339

00-1597_Tlp1 NGVFNYIASDGDDSYAAINSFKV--QDSSWAVLVTAPKYSVFKPLKKLQLIILGASFIFI 339

Cfetus_testudinum_pet-3_Tlp404 KIFPDYITSTGVNSYAVVAPFTTARDSSNWAVITTAPIESVLAPLYSLQKTIFVASLIFL 339

Cfetus_testudinum_03-427_Tlp404 KIFPDYITSTGVNSYAVVAPFTTARDSSNWAVITTAPIESVLAPLYSLQKTIFVASLIFL 344

Cfetus_venerealis_84-112_Tlp404 KIFSNYTTSTGVSSYAVVAPFTTARDSSNWAIVTTAPIDSVFAPLYSLQKTIFVASLIFL 339

Cfetus_venerealis_01-165_Tlp404 KIFSNYTTSTGVSSYAVVAPFTTARDSSNWAIVTTAPIDSVFAPLYSLQKTIFVASLIFL 358

Cfetus_fetus_04-554_Tlp404 KIFSNYTTSTGVSSYAVVAPFTTARDSSNWAIVTTAPIDSVFAPLYSLQKTIFVASLIFL 358

Cfetus_fetus_82-40_Tlp404 KIFSNYTTSTGVSSYAVVAPFTTARDSSNWAIVTKAPIDSVFAPLYSLQKTIFVASLIFL 358

: . :* : * .***.: *.. :.*.*:::..:* **: ** .** *: **:**:

FDAARGOS_295_Tlp1 FVVLGVVYYCVRKIVATRLPIILNSLESFFRFLNHEKIELKLIKIRANDELGAMGRIINE 399

14980A_Tlp1 IVVLGVVYYCVRKIVAARLPIILNSLESFFRFLNHEKIELKPIKIRANDELGAMGNIINE 399

CJ677CC527_Tlp1 FVVLGVVYYCVRKIVATRLPIILNSLESFFRFLNHEKIELKLIKIRANDELGAMGRIINE 399

CJ677CC012_Tlp1 FVVLGVVYYCVRKIVATRLPIILNSLESFFRFLNHEKIELKLIKIRANDELGAMGRIINE 399

4031_Tlp1 FVVLGVVYYCVRKIVASRLPVILSSLESFFRFLNHEKIEPKAIEIRANDELGAMGRIINE 399

81116_Tlp1 FVVLGVVYYCVRKIVASRLPVILSSLESFFRFLNHEKIEPKAIEIRANDELGAMGRIINE 399

35925B2_Tlp1 FVVLGVVYYCVRKIVASRLPVILSSLESFFRFLNHEKIEPKAIEIRANDELGAMGRIINE 399

M1_Tlp1 FVVLGVVYYCVRKIVASRLPVILSSLESFFRFLNHEKIEPKAIEIRANDELGAMGRIINE 399

PT14_Tlp1 FVVLGVVYYCVRKIVASRLPVILSSLESFFRFLNHEKIEPKAIEIRANDELGAMGRIINE 399

81-176_Tlp1 FVVLGVVYYCVRKIVASRLPVILSSLESFFRFLNHEKIEPKAIEIRANDELGAMGRIINE 399

CVMN29710_Tlp1 FVVLGVVYYCVRKIVASRLPVILSSLESFFRFLNHEKIEPKAIEIRANDELGAMGRIINE 399

FB1_Tlp1 FVVLGVVYYCVRKIVASRLPVILSSLESFFRFLNHEKIEPKAIEIRANDELGAMGRIINE 399

BG2108_Tlp1 FVVLGVVYYCVRKIVASRLPVILSSLESFFRFLNHEKIEPKAIEIRANDELGAMGRIINE 399

YF2105_Tlp1 FVVLGVVYYCVRKIVASRLPVILSSLESFFRFLNHEKIEPKAIEIRANDELGAMGRIINE 399

YH501_Tlp1 FVVLGVVYYCVRKIVASRLPVILSSLESFFRFLNHEKIEPKAIEIRANDELGAMGRIINE 399

RM4661_Tlp1 FVVLGVVYYCVRKIVASRLPVILSSLESFFRFLNHEKIEPKAIEIRANDELGAMGRIINE 399

F38011_Tlp1 FVVLGVVYYCVRKIVASRLPVILSSLESFFRFLNHEKIEPKAIEIRANDELGAMGRIINE 399

T1-21_Tlp1 FVVLGVVYYCVRKIVASRLPVILSSLESFFRFLNHEKIEPKAIEIRANDELGAMGRIINE 399

ICDCCJ07001_Tlp1 FVVLGVVYYCVRKIVASRLPVILSSLESFFRFLNHEKIEPKAIEIRANDELGAMGRIINE 399

RM3196_Tlp1 FVVLGVVYYCVRKIVASRLPVILSSLESFFRFLNHEKIEPKAIEIRANDELGAMGRIINE 399

NCTC11168_Tlp1 FVVLGVVYYCVRKIVASRLPVILSSLESFFRFLNHEKIEPKAIEIRANDELGAMGRIINE 399

00-2425_Tlp1 FVVLGVVYYCVRKIVASRLPVILSSLESFFRFLNHEKIEPKAIEIRANDELGAMGRIINE 399

IA3902_Tlp1 FVVLGVVYYCVRKIVASRLPVILSSLESFFRFLNHEKIEPKAIEIRANDELGAMGRIINE 399

RM1285_Tlp1 FVVLGVVYYCVRKIVASRLPVILSSLESFFRFLNHEKIEPKAIEIRANDELGAMGRIINE 399

00-0949_Tlp1 FVVLGVVYYCVRKIVASRLPVILSSLESFFRFLNHEKIEPKAIEIRANDELGAMGRIINE 399

01-1512_Tlp1 FVVLGVVYYCVRKIVASRLPVILSSLESFFRFLNHEKIEPKAIEIRANDELGAMGRIINE 399

FDAARGOS_422_Tlp1 FVVLGVVYYCVRKIVASRLPVILSSLESFFRFLNHEKIEPKAIEIRANDELGAMGRIINE 399

FORC_056_Tlp1 FVVLGVVYYCVRKIVASRLPVILSSLESFFRFLNHEKIEPKAIEIRANDELGAMGRIINE 399

32488_Tlp1 FVVLGVVYYCVRKIVASRLPVILSSLESFFRFLNHEKIEPKAIEIRANDELGAMGRIINE 399

CFSAN032806_Tlp1 FVVLGVVYYCVRKIVASRLPVILSSLESFFRFLNHEKIEPKAIEIRANDELGAMGRIINE 399

YH001_Tlp1 FVVLGVVYYCVRKIVASRLPVILSSLESFFRFLNHEKIEPKAIEIRANDELGAMGRIINE 399

00-6200_Tlp1 FVVLGVVYYCVRKIVASRLPVILSSLESFFRFLNHEKIEPKAIEIRANDELGAMGRIINE 399

RM1221_Tlp1 FVVLGVVYYCVRKIVASRLPVILSSLESFFRFLNHEKIEPKAIEIRANDELGAMGRIINE 399

S3_Tlp1 FVVLGVVYYCVRKIVASRLPVILSSLESFFRFLNHEKIEPKAIEIRANDELGAMGRIINE 399

FDAARGOS_421_Tlp1 FVVLGVVYYCVRKIVASRLPVILSSLESFFRFLNHEKIEPKAIEIRANDELGAMGRIINE 399

FJ3124_Tlp1 FVVLGVVYYCVRKIVASRLPVILSSLESFFRFLNHEKIEPKAIEIRANDELGAMGRIINE 399

CG8421_Tlp1 FVVLGVVYYCVRKIVASRLPVILSSLESFFRFLNHEKIEPKAIEIRANDELGAMGRIINE 399

CJM1cam_Tlp1 FVVLGVVYYCVRKIVASRLPVILSSLESFFRFLNHEKIEPKAIEIRANDELGAMGRIINE 399

R14_Tlp1 FVVLGVVYYCVRKIVASRLPVILSSLESFFRFLNHEKIEPKAIEIRANDELGAMGRIINE 399

00-1597_Tlp1 FVVLGVVYYCVRKIVASRLPVILSSLESFFRFLNHEKIEPKAIEIRANDELGAMGRIINE 399

Cfetus_testudinum_pet-3_Tlp404 VVSLAFIYFYIKANLAMRLPILLNALDSFFKFINHESKEVHMIKIHANDELGAMGNMINA 399

Cfetus_testudinum_03-427_Tlp404 VVSLAFIYFYIKANLAMRLPILLNALDSFFKFINHESKEVHMIKIHANDELGAMGNMINA 404

Cfetus_venerealis_84-112_Tlp404 VVSLAFIYFYIKANLAMRLPILLNALDSFFKFINHESKEVQMIKIDANDELGAMGNMINA 399

Cfetus_venerealis_01-165_Tlp404 VVSLAFIYFYIKANLAMRLPILLNALDSFFKFINHESKEVQMIKIDANDELGAMGNMINA 418

Cfetus_fetus_04-554_Tlp404 VVSLAFIYFYIKANLAMRLPILLNALDSFFKFINHESKEVQMIKIHANDELGAMGNMINA 418

Cfetus_fetus_82-40_Tlp404 VVSLAFIYFYIKANLAMRLPILLNALDSFFKFINHESKEVQMIKIHANDELGAMGNMINA 418

.* *..:*: :: :* ***::*.:*:***:*:***. * : *:* *********.:**

FDAARGOS_295_Tlp1 NIEKIQMSLEQDQNAVDESVQTAREIEKGNLTARITKNPINPQLVELKNVLNRMLDALQS 459

14980A_Tlp1 NIKKIQLSLEQDQSAVDESVQTAKEIEKGNLTARITKNPINPQLVELKNVLNKMLDVLQN 459

CJ677CC527_Tlp1 NIEKIQISLEQDQNAVDESVQTAREIEKGNLTARITKNPINPQLVELKNVLNRMLDVLQS 459

CJ677CC012_Tlp1 NIEKIQISLEQDQNAVDESVQTAREIEKGNLTARITKNPINPQLVELKNVLNRMLDVLQS 459

4031_Tlp1 NIEKIQISLEQDQNAVDESVQTAREIEKGNLTARITKNPINPQLVELKNVLNRMLDVLQS 459

81116_Tlp1 NIEKIQISLEQDQNAVDESVQTAREIEKGNLTARITKNPINPQLVELKNVLNRMLDVLQS 459

35925B2_Tlp1 NIEKIQISLEQDQNAVDESVQTAREIEKGNLTARITKNPINPQLVELKNVLNRMLDVLQS 459

M1_Tlp1 NIEKIQISLEQDQNAVDESVQTAREIEKGNLTARITKNPINPQLVELKNVLNRMLDVLQS 459

PT14_Tlp1 NIEKIQISLEQDQNAVDESVQTAREIEKGNLTARITKNPINPQLVELKNVLNRMLDVLQS 459

81-176_Tlp1 NIEKIQISLEQDQNAVDESVQTAREIEKGNLTARITKNPINPQLVELKNVLNRMLDVLQS 459

CVMN29710_Tlp1 NIEKIQISLEQDQNAVDESVQTAREIEKGNLTARITKNPINPQLVELKNVLNRMLDVLQS 459

FB1_Tlp1 NIEKIQISLEQDQNAVDESVQTAREIEKGNLTARITKNPINPQLVELKNVLNRMLDVLQS 459

BG2108_Tlp1 NIEKIQISLEQDQNAVDESVQTAREIEKGNLTARITKNPINPQLVELKNVLNRMLDVLQS 459

YF2105_Tlp1 NIEKIQISLEQDQNAVDESVQTAREIEKGNLTARITKNPINPQLVELKNVLNRMLDVLQS 459

YH501_Tlp1 NIEKIQISLEQDQNAVDESVQTAREIEKGNLTARITKNPINPQLVELKNVLNRMLDVLQS 459

RM4661_Tlp1 NIEKIQISLEQDQNAVDESVQTAREIEKGNLTARITKNPINPQLVELKNVLNRMLDVLQS 459

F38011_Tlp1 NIEKIQISLEQDQNAVDESVQTAREIEKGNLTARITKNPINPQLVELKNVLNRMLDVLQS 459

T1-21_Tlp1 NIEKIQISLEQDQNAVDESVQTAREIEKGNLTARITKNPINPQLVELKNVLNRMLDVLQS 459

ICDCCJ07001_Tlp1 NIEKIQISLEQDQNAVDESVQTAREIEKGNLTARITKNPINPQLVELKDVLNRMLDVLQS 459

RM3196_Tlp1 NIEKIQISLEQDQNAVDESVQTAREIEKGNLTARITKNPINPQLVELKDVLNRMLDVLQS 459

NCTC11168_Tlp1 NIEKIQISLEQDQNAVDESVQTAREIEKGNLTARITKNPINPQLVELKDVLNRMLDVLQS 459

00-2425_Tlp1 NIEKIQISLEQDQNAVDESVQTAREIEKGNLTARITKNPINPQLVELKDVLNRMLDVLQS 459

IA3902_Tlp1 NIEKIQISLEQDQNAVDESVQTAREIEKGNLTARITKNPINPQLVELKDVLNRMLDVLQS 459

RM1285_Tlp1 NIEKIQISLEQDQNAVDESVQTAREIEKGNLTARITKNPINPQLVELKDVLNRMLDVLQS 459

00-0949_Tlp1 NIEKIQISLEQDQNAVDESVQTAREIEKGNLTARITKNPINPQLVELKDVLNRMLDVLQS 459

01-1512_Tlp1 NIEKIQISLEQDQNAVDESVQTAREIEKGNLTARITKNPINPQLVELKDVLNRMLDVLQS 459

FDAARGOS_422_Tlp1 NIEKIQISLEQDQNAVDESVQTAREIEKGNLTARITKNPINPQLVELKDVLNRMLDVLQS 459

FORC_056_Tlp1 NIEKIQISLEQDQNAVDESVQTAREIEKGNLTARITKNPINPQLVELKDVLNRMLDVLQS 459

32488_Tlp1 NIEKIQISLEQDQNAVDESVQTAREIEKGDLTARITKNPINPQLVELKNVLNRMLDVLQS 459

CFSAN032806_Tlp1 NIEKIQISLEQDQNAVDESVQTAREIEKGDLTARITKNPINPQLVELKNVLNRMLDVLQS 459

YH001_Tlp1 NIEKIQISLEQDQNAVDESVQTVREIEKGNLTARITKNPINPQLVELKNVLNRMLDVLQS 459

00-6200_Tlp1 NIEKIQISLEQDQNAVDESVQTVREIEKGNLTARITKNPINPQLVELKNVLNRMLDVLQS 459

RM1221_Tlp1 NIEKIQISLEQDQNAVDESVQTAREIEKGNLTARITKNPINPQLVELKNVLNRMLDVLQS 459

S3_Tlp1 NIEKIQISLEQDQNAVDESVQTAREIEKGNLTARITKNPINPQLVELKNVLNRMLDVLQS 459

FDAARGOS_421_Tlp1 NIEKIQISLEQDQNAVDESVQTAREIEKGNLTARITKNPINPQLVELKNVLNRMLDVLQS 459

FJ3124_Tlp1 NIEKIQISLEQDQNAVDESVQTAREIEKGNLTARITKNPINPQLVELKNVLNRMLDVLQS 459

CG8421_Tlp1 NIEKIQISLEQDQNAVDESVQTAREIEKGNLTARITKNPINPQLVELKNVLNRMLDVLQS 459

CJM1cam_Tlp1 NIEKIQISLEQDQNAVDESVQTAREIEKGNLTARITKNPINPQLVELKNVLNRMLDVLQS 459

R14_Tlp1 NIEKIQISLEQDQNAVDESVQTAREIEKGNLTARITKNPINPQLVELKNVLNRMLDVLQS 459

00-1597_Tlp1 NIEKIQISLEQDQNAVDESVQTAREIEKGNLTARITKNPINPQLVELKNVLNRMLDVLQS 459

Cfetus_testudinum_pet-3_Tlp404 NIAKTRDSLIKDQEAVQQSVETAKEIESGNLTARIVKDPANPQLIELKNVLNKMLLVLQN 459

Cfetus_testudinum_03-427_Tlp404 NIAKTRDSLIKDQEAVQQSVETAKEIESGNLTARIVKDPANPQLIELKNVLNKMLLVLQN 464

Cfetus_venerealis_84-112_Tlp404 NIAKTRDSLIKDQEAVQQSVETAKEIEGGNLTARIVKDPANPQLIELKNVLNKMLLVLQN 459

Cfetus_venerealis_01-165_Tlp404 NIAKTRDSLIKDQEAVQQSVETAKEIEGGNLTARIVKDPANPQLIELKNVLNKMLLVLQN 478

Cfetus_fetus_04-554_Tlp404 NIAKTRDSLIKDQEAVQQSVETAKEIEGGNLTARIVKDPANPQLIELKNVLNKMLLVLQN 478

Cfetus_fetus_82-40_Tlp404 NIAKTRDSLIKDQEAVQQSVETAKEIEGGNLTARIVKDPANPQLIELKNVLNKMLLVLQN 478

** * : ** :**.**::**:*.:*** *:*****.*:* ****:***:***:** .**.

FDAARGOS_295_Tlp1 KIGSNMNEINRVFDSYKALDFSTEVFDAKGEVEITTNILGKEIKKMLVASSNFAKDLANQ 519

14980A_Tlp1 KIGSNMNEINRVFDSYKALDFSTEVFDAKGEVEITTNILGKEIKKMLVASSNFAKDLANQ 519

CJ677CC527_Tlp1 KIGSNMNEINRVFDSYKALDFSTEVFDAKGEVEITTNILGKEIKKMLVASSNFAKDLANQ 519

CJ677CC012_Tlp1 KIGSNMNEINRVFDSYKALDFSTEVFDAKGEVEITTNILGKEIKKMLVASSNFAKDLANQ 519

4031_Tlp1 KIGSNMNEINRVFDSYKALDFSTEVLDAKGEVEITTNILGKEIKKMLVASSNFAKDLANQ 519

81116_Tlp1 KIGSNMNEINRVFDSYKALDFSTEVLDAKGEVEITTNILGKEIKKMLVASSNFAKDLANQ 519

35925B2_Tlp1 KIGSNMNEINRVFDSYKALDFSTEVLDAKGEVEITTNILGKEIKKMLVASSNFAKDLANQ 519

M1_Tlp1 KIGSNMNEINRVFDSYKALDFSTEVLDAKGEVEITTNILGKEIKKMLVASSNFAKDLANQ 519

PT14_Tlp1 KIGSNMNEINRVFDSYKALDFSTEVLDAKGEVEITTNILGKEIKKMLVASSNFAKDLANQ 519

81-176_Tlp1 KIGSNMNEINRVFDSYKALDFSTEVFNAKGEVEITTNILGKEIKKMLLASSNFAKDLANQ 519

CVMN29710_Tlp1 KIGSNMNEINRVFDSYKALDFSTEVFNAKGEVEITTNILGKEIKKMLVASSNFAKDLANQ 519

FB1_Tlp1 KIGSNMNEINRVFDSYKALDFSTEVFNAKGEVEITTNILGKEIKKMLVASSNFAKDLANQ 519

BG2108_Tlp1 KIGSNMNEINRVFDSYKALDFSTEVFNAKGEVEITTNILGKEIKKMLVASSNFAKDLANQ 519

YF2105_Tlp1 KIGSNMNEINRVFDSYKALDFSTEVFNAKGEVEITTNILGKEIKKMLVASSNFAKDLANQ 519

YH501_Tlp1 KIGSNMNEINRVFDSYKALDFSTEVFNAKGEVEITTNILGKEIKKMLVASSNFAKDLANQ 519

RM4661_Tlp1 KIGSNMNEINRVFDSYKALDFSTEVFNAKGEVEITTNILGKEIKKMLLASSNFAKDLANQ 519

F38011_Tlp1 KIGSNMNEINRVFDSYKALDFSTEVFNAKGEVEITTNILGKEIKKMLLASSNFAKDLANQ 519

T1-21_Tlp1 KIGSNMNEINRVFDSYKALDFSTEVFNAKGEVEITTNILGKEIKKMLLASSNFAKDLANQ 519

ICDCCJ07001_Tlp1 KIGSNMNEINRVFDSYKALDFSTEVFNAKGEVEITTNILGKEIKKMLLASSNFAKDLANQ 519

RM3196_Tlp1 KIGSNMNEINRVFDSYKALDFSTEVFNAKGEVEITTNILGKEIKKMLLASSNFAKDLANQ 519

NCTC11168_Tlp1 KIGSNMNEINRVFDSYKALDFSTEVFNAKGEVEITTNILGKEIKKMLLASSNFAKDLANQ 519

00-2425_Tlp1 KIGSNMNEINRVFDSYKALDFSTEVFNAKGEVEITTNILGKEIKKMLLASSNFAKDLANQ 519

IA3902_Tlp1 KIGSNMNEINRVFDSYKALDFSTEVFNAKGEVEITTNILGKEIKKMLLASSNFAKDLANQ 519

RM1285_Tlp1 KIGSNMNEINRVFDSYKALDFSTEVFNAKGEVEITTNILGKEIKKMLLASSNFAKDLANQ 519

00-0949_Tlp1 KIGSNMNEINRVFDSYKALDFSTEVFNAKGEVEITTNILGKEIKKMLLASSNFAKDLANQ 519

01-1512_Tlp1 KIGSNMNEINRVFDSYKALDFSTEVFNAKGEVEITTNILGKEIKKMLLASSNFAKDLANQ 519

FDAARGOS_422_Tlp1 KIGSNMNEINRVFDSYKALDFSTEVFNAKGEVEITTNILGKEIKKMLLASSNFAKDLANQ 519

FORC_056_Tlp1 KIGSNMNEINRVFDSYKALDFSTEVFNAKGEVEITTNILGKEIKKMLLASSNFAKDLANQ 519

32488_Tlp1 KIGSNMNEINRVFDSYKALDFSTEVFNAKGEVEITTNILGKEIKKMLLASSNFAKDLANQ 519

CFSAN032806_Tlp1 KIGSNMNEINRVFDSYKALDFSTEVFNAKGEVEITTNILGKEIKKMLLASSNFAKDLANQ 519

YH001_Tlp1 KIGSNMNEINRVFDSYKALDFSTEVFNAKGEVEITTNILGKEIKKMLLASSNFAKDLANQ 519

00-6200_Tlp1 KIGSNMNEINRVFDSYKALDFSTEVFNAKGEVEITTNILGKEIKKMLLASSNFAKDLANQ 519

RM1221_Tlp1 KIGSNMNEINRVFDSYKALDFSTEVFNAKGEVEITTNILGKEIKKMLVASSNFAKDLANQ 519

S3_Tlp1 KIGSNMNEINRVFDSYKALDFSTEVFNAKGEVEITTNILGKEIKKMLVASSNFAKDLANQ 519

FDAARGOS_421_Tlp1 KIGSNMNEINRVFDSYKALDFSTEVFNAKGEVEITTNILGKEIKKMLVASSNFAKDLANQ 519

FJ3124_Tlp1 KIGSNMNEINRVFDSYKALDFSTEVFNAKGEVEITTNILGKEIKKMLVASSNFAKDLANQ 519

CG8421_Tlp1 KIGSNMNEINRVFDSYKALDFSTEVFNAKGEVEITTNILGKEIKKMLLASSNFAKDLANQ 519

CJM1cam_Tlp1 KIGSNMNEINRVFDSYKALDFSTEVFNAKGEVEITTNILGKEIKKMLLASSNFAKDLANQ 519

R14_Tlp1 KIGSNMNEINRVFDSYKALDFSTEVFNAKGEVEITTNILGKEIKKMLLASSNFAKDLANQ 519

00-1597_Tlp1 KIGSNMNEINRVFDSYKALDFSTEVFNAKGEVEITTNILGKEIKKMLLASSNFAKDLANQ 519

Cfetus_testudinum_pet-3_Tlp404 KVGSNMNEINRVFNSYKSLDFTTNIANAKGEVEVTTNVLGDEIKEMLRSSLSFAKDLAEQ 519

Cfetus_testudinum_03-427_Tlp404 KVGSNMNEINRVFNSYKSLDFTTNIANAKGEVEVTTNVLGDEIKEMLRSSLSFAKDLAEQ 524

Cfetus_venerealis_84-112_Tlp404 KVGSNMNEINRVFNSYKSLDFTTNIANAKGEVEVTTNVLGDEIKEMLRSSLSFAKDLAEQ 519

Cfetus_venerealis_01-165_Tlp404 KVGSNMNEINRVFNSYKSLDFTTNIANAKGEVEVTTNVLGDEIKEMLRSSLSFAKDLAEQ 538

Cfetus_fetus_04-554_Tlp404 KVGSNMNEINRVFNSYKSLDFTTNIANAKGEVEVTTNVLGDEIKEMLRSSLSFAKDLAEQ 538

Cfetus_fetus_82-40_Tlp404 KVGSNMNEINRVFNSYKSLDFTTNIANAKGEVEVTTNVLGDEIKEMLRSSLSFAKDLAEQ 538

*:***********:***:***:*:: :******:***:**.***:** :* .******:*

FDAARGOS_295_Tlp1 SEELKNSMQKLADGSNAQASSLEQSAAAVEEINSSMQNVSGKTVEVASQADDIKNIVNVI 579

14980A_Tlp1 SEELKNSMRKLADGSNAQASSLEQSAAAVEEINSSMQNVSGKTVEVASQADDIKNIVNVI 579

CJ677CC527_Tlp1 SEELKNSMQKLADGSNAQASSLEQSAAAVEEINSSMQNVSGKTVEVASQADDIKNIVNVI 579

CJ677CC012_Tlp1 SEELKNSMQKLADGSNAQASSLEQSAAAVEEINSSMQNVSGKTVEVASQADDIKNIVNVI 579

4031_Tlp1 SEELKNSMQKLADGSNAQASSLEQSAAAVEEINSSMQNVSGKTVEVASQADDIKNIVNVI 579

81116_Tlp1 SEELKNSMQKLADGSNAQASSLEQSAAAVEEINSSMQNVSGKTVEVASQADDIKNIVNVI 579

35925B2_Tlp1 SEELKNSMQKLADGSNAQASSLEQSAAAVEEINSSMQNVSGKTVEVASQADDIKNIVNVI 579

M1_Tlp1 SEELKNSMQKLADGSNAQASSLEQSAAAVEEINSSMQNVSGKTVEVASQADDIKNIVNVI 579

PT14_Tlp1 SEELKNSMQKLADGSNAQASSLEQSAAAVEEINSSMQNVSGKTVEVASQADDIKNIVNVI 579

81-176_Tlp1 SEELKNSMQKLADGSNAQASSLEQSAAAVEEINSSMQNVSGKTVEVASQADDIKNIVNVI 579

CVMN29710_Tlp1 SEELKNSMQKLADGSNAQASSLEQSAAAVEEINSSMQNVSGKTVEVASQADDIKNIVNVI 579

FB1_Tlp1 SEELKNSMQKLADGSNAQASSLEQSAAAVEEINSSMQNVSGKTVEVASQADDIKNIVNVI 579

BG2108_Tlp1 SEELKNSMQKLADGSNAQASSLEQSAAAVEEINSSMQNVSGKTVEVASQADDIKNIVNVI 579

YF2105_Tlp1 SEELKNSMQKLADGSNAQASSLEQSAAAVEEINSSMQNVSGKTVEVASQADDIKNIVNVI 579

YH501_Tlp1 SEELKNSMQKLADGSNAQASSLEQSAAAVEEINSSMQNVSGKTVEVASQADDIKNIVNVI 579

RM4661_Tlp1 SEELKNSMQKLADGSNAQASSLEQSAAAVEEINSSMQNVSGKTVEVASQADDIKNIVNVI 579

F38011_Tlp1 SEELKNSMQKLADGSNAQASSLEQSAAAVEEINSSMQNVSGKTVEVASQADDIKNIVNVI 579

T1-21_Tlp1 SEELKNSMQKLADGSNAQASSLEQSAAAVEEINSSMQNVSGKTVEVASQADDIKNIVNVI 579

ICDCCJ07001_Tlp1 SEELKNSMQKLADGSNAQASSLEQSAAAVEEINSSMQNVSGKTVEVASQADDIKNIVNVI 579

RM3196_Tlp1 SEELKNSMQKLADGSNAQASSLEQSAAAVEEINSSMQNVSGKTVEVASQADDIKNIVNVI 579

NCTC11168_Tlp1 SEELKNSMQKLADGSNAQASSLEQSAAAVEEINSSMQNVSGKTVEVASQADDIKNIVNVI 579

00-2425_Tlp1 SEELKNSMQKLADGSNAQASSLEQSAAAVEEINSSMQNVSGKTVEVASQADDIKNIVNVI 579

IA3902_Tlp1 SEELKNSMQKLADGSNAQASSLEQSAAAVEEINSSMQNVSGKTVEVASQADDIKNIVNVI 579

RM1285_Tlp1 SEELKNSMQKLADGSNAQASSLEQSAAAVEEINSSMQNVSGKTVEVASQADDIKNIVNVI 579

00-0949_Tlp1 SEELKNSMQKLADGSNAQASSLEQSAAAVEEINSSMQNVSGKTVEVASQADDIKNIVNVI 579

01-1512_Tlp1 SEELKNSMQKLADGSNAQASSLEQSAAAVEEINSSMQNVSGKTVEVASQADDIKNIVNVI 579

FDAARGOS_422_Tlp1 SEELKNSMQKLADGSNAQASSLEQSAAAVEEINSSMQNVSGKTVEVASQADDIKNIVNVI 579

FORC_056_Tlp1 SEELKNSMQKLADGSNAQASSLEQSAAAVEEINSSMQNVSGKTVEVASQADDIKNIVNVI 579

32488_Tlp1 SEELKNSMQKLADGSNAQASSLEQSAAAVEEINSSMQNVSGKTVEVASQADDIKNIVNVI 579

CFSAN032806_Tlp1 SEELKNSMQKLADGSNAQASSLEQSAAAVEEINSSMQNVSGKTVEVASQADDIKNIVNVI 579

YH001_Tlp1 SEELKNSMQKLADGSNAQASSLEQSAAAVEEINSSMQNVSGKTVEVASQADDIKNIVNVI 579

00-6200_Tlp1 SEELKNSMQKLADGSNAQASSLEQSAAAVEEINSSMQNVSGKTVEVASQADDIKNIVNVI 579

RM1221_Tlp1 SEELKNSMQKLADGSNAQASSLEQSAAAVEEINSSMQNVSGKTVEVASQADDIKNIVNVI 579

S3_Tlp1 SEELKNSMQKLADGSNAQASSLEQSAAAVEEINSSMQNVSGKTVEVASQADDIKNIVNVI 579

FDAARGOS_421_Tlp1 SEELKNSMQKLADGSNAQASSLEQSAAAVEEINSSMQNVSGKTVEVASQADDIKNIVNVI 579

FJ3124_Tlp1 SEELKNSMQKLADGSNAQASSLEQSAAAVEEINSSMQNVSGKTVEVASQADDIKNIVNVI 579

CG8421_Tlp1 SKELKNSMQKLADGSNAQASSLEQSAAAVEEINSSMQNVSGKTVEVASQADDIKNIVNVI 579

CJM1cam_Tlp1 SKELKNSMQKLADGSNAQASSLEQSAAAVEEINSSMQNVSGKTVEVASQADDIKNIVNVI 579

R14_Tlp1 SKELKNSMQKLADGSNAQASSLEQSAAAVEEINSSMQNVSGKTVEVASQADDIKNIVNVI 579

00-1597_Tlp1 SEELKNSMQKLADGSNAQASSLEQSAAAVEEINSSMQNVSGKTVEVASQADDIKNIVNVI 579

Cfetus_testudinum_pet-3_Tlp404 SKDLRESMQKLTDGSRTQAHSLEQSAAAVEQISCSMQSISDRTIETTKQAEDIKNIVGVI 579

Cfetus_testudinum_03-427_Tlp404 SKDLRESMQKLTDGSRTQAHSLEQSAAAVEQISCSMQSISDRTIETTKQAEDIKNIVGVI 584

Cfetus_venerealis_84-112_Tlp404 SKELRESMQKLTDGSRTQAHSLEQSAAAVEQISCSMQSISDRTVETTKQAEDIKNIVGVI 579

Cfetus_venerealis_01-165_Tlp404 SKELRESMQKLTDGSRTQAHSLEQSAAAVEQISCSMQSISDRTVETTKQAEDIKNIVGVI 598

Cfetus_fetus_04-554_Tlp404 SKELRESMQKLTDGSRTQAHSLEQSAAAVEQISCSMQSISDRTVETTKQAEDIKNIVGVI 598

Cfetus_fetus_82-40_Tlp404 SKELRESMQKLTDGSRTQAHSLEQSAAAVEQISCSMQSISDRTVETTKQAEDIKNIVGVI 598

*::*::**:**:***.:** **********:*..***.:*.:*:*.:.**:******.**

FDAARGOS_295_Tlp1 KDIAEQTNLLALNAAIEAARAGEHGRGFAVVADEVRQLAERTGKSLSEIEANINILVQSV 639

14980A_Tlp1 KDIAEQTNLLALNAAIEAARAGEHGRGFAVVADEVRQLAERTGKSLSEIEANINILVQSV 639

CJ677CC527_Tlp1 KDIAEQTNLLALNAAIEAARAGEHGRGFAVVADEVRQLAERTGKSLSEIEANINILVQSV 639

CJ677CC012_Tlp1 KDIAEQTNLLALNAAIEAARAGEHGRGFAVVADEVRQLAERTGKSLSEIEANINILVQSV 639

4031_Tlp1 KDIAEQTNLLALNAAIEAARAGEHGRGFAVVADEVRQLAERTGKSLSEIEANINILVQSV 639

81116_Tlp1 KDIAEQTNLLALNAAIEAARAGEHGRGFAVVADEVRQLAERTGKSLSEIEANINILVQSV 639

35925B2_Tlp1 KDIAEQTNLLALNAAIEAARAGEHGRGFAVVADEVRQLAERTGKSLSEIEANINILVQSV 639

M1_Tlp1 KDIAEQTNLLALNAAIEAARAGEHGRGFAVVADEVRQLAERTGKSLSEIEANINILVQSV 639

PT14_Tlp1 KDIAEQTNLLALNAAIEAARAGEHGRGFAVVADEVRQLAERTGKSLSEIEANINILVQSV 639

81-176_Tlp1 KDIAEQTNLLALNAAIEAARAGEHGRGFAVVADEVRQLAERTGKSLSEIEANINILVQSV 639

CVMN29710_Tlp1 KDIAEQTNLLALNAAIEAARAGEHGRGFAVVADEVRQLAERTGKSLSEIEANINILVQSV 639

FB1_Tlp1 KDIAEQTNLLALNAAIEAARAGEHGRGFAVVADEVRQLAERTGKSLSEIEANINILVQSV 639

BG2108_Tlp1 KDIAEQTNLLALNAAIEAARAGEHGRGFAVVADEVRQLAERTGKSLSEIEANINILVQSV 639

YF2105_Tlp1 KDIAEQTNLLALNAAIEAARAGEHGRGFAVVADEVRQLAERTGKSLSEIEANINILVQSV 639

YH501_Tlp1 KDIAEQTNLLALNAAIEAARAGEHGRGFAVVADEVRQLAERTGKSLSEIEANINILVQSV 639

RM4661_Tlp1 KDIAEQTNLLALNAAIEAARAGEHGRGFAVVADEVRQLAERTGKSLSEIEANINILVQSV 639

F38011_Tlp1 KDIAEQTNLLALNAAIEAARAGEHGRGFVVVADEVRQLAERTGKSLSEIEANINILVQSV 639

T1-21_Tlp1 KDIAEQTNLLALNAAIEAARAGEHGRGFAVVADEVRQLAERTGKSLSEIEANINILVQSV 639

ICDCCJ07001_Tlp1 KDIAEQTNLLALNAAIEAARAGEHGRGFAVVADEVRQLAERTGKSLSEIEANINILVQSV 639

RM3196_Tlp1 KDIAEQTNLLALNAAIEAARAGEHGRGFAVVADEVRQLAERTGKSLSEIEANINILVQSV 639

NCTC11168_Tlp1 KDIAEQTNLLALNAAIEAARAGEHGRGFAVVADEVRQLAERTGKSLSEIEANINILVQSV 639

00-2425_Tlp1 KDIAEQTNLLALNAAIEAARAGEHGRGFAVVADEVRQLAERTGKSLSEIEANINILVQSV 639

IA3902_Tlp1 KDIAEQTNLLALNAAIEAARAGEHGRGFAVVADEVRQLAERTGKSLSEIEANINILVQSV 639

RM1285_Tlp1 KDIAEQTNLLALNAAIEAARAGEHGRGFAVVADEVRQLAERTGKSLSEIEANINILVQSV 639

00-0949_Tlp1 KDIAEQTNLLALNAAIEAARAGEHGRGFAVVADEVRQLAERTGKSLSEIEANINILVQSV 639

01-1512_Tlp1 KDIAEQTNLLALNAAIEAARAGEHGRGFAVVADEVRQLAERTGKSLSEIEANINILVQSV 639

FDAARGOS_422_Tlp1 KDIAEQTNLLALNAAIEAARAGEHGRGFAVVADEVRQLAERTGKSLSEIEANINILVQSV 639

FORC_056_Tlp1 KDIAEQTNLLALNAAIEAARAGEHGRGFAVVADEVRQLAERTGKSLSEIEANINILVQSV 639

32488_Tlp1 KDIAEQTNLLALNAAIEAARAGEHGRGFAVVADEVRQLAERTGKSLSEIEANINILVQSV 639

CFSAN032806_Tlp1 KDIAEQTNLLALNAAIEAARAGEHGRGFAVVADEVRQLAERTGKSLSEIEANINILVQSV 639

YH001_Tlp1 KDIAEQTNLLALNAAIEAARAGEHGRGFAVVADEVRQLAERTGKSLSEIEANINILVQSV 639

00-6200_Tlp1 KDIAEQTNLLALNAAIEAARAGEHGRGFAVVADEVRQLAERTGKSLSEIEANINILVQSV 639

RM1221_Tlp1 KDIAEQTNLLALNAAIEAARAGEHGRGFAVVADEVRQLAERTGKSLSEIEANINILVQSV 639

S3_Tlp1 KDIAEQTNLLALNAAIEAARAGEHGRGFAVVADEVRQLAERTGKSLSEIEANINILVQSV 639

FDAARGOS_421_Tlp1 KDIAEQTNLLALNAAIEAARAGEHGRGFAVVADEVRQLAERTGKSLSEIEANINILVQSV 639

FJ3124_Tlp1 KDIAEQTNLLALNAAIEAARAGEHGRGFAVVADEVRQLAERTGKSLSEIEANINILVQSV 639

CG8421_Tlp1 KDIAEQTNLLALNAAIEAARAGEHGRGFAVVADEVRQLAERTGKSLSEIEANINILVQSV 639

CJM1cam_Tlp1 KDIAEQTNLLALNAAIEAARAGEHGRGFAVVADEVRQLAERTGKSLSEIEANINILVQSV 639

R14_Tlp1 KDIAEQTNLLALNAAIEAARAGEHGRGFAVVADEVRQLAERTGKSLSEIEANINILVQSV 639

00-1597_Tlp1 KDIAEQTNLLALNAAIEAARAGEHGRGFAVVADEVRQLAERTGKSLSEIEANINILVQSV 639

Cfetus_testudinum_pet-3_Tlp404 KDIADQTNLLALNAAIEAARAGEHGRGFAVVADEVRKLAERTNSSLGEIEVNVNILVQSV 639

Cfetus_testudinum_03-427_Tlp404 KDIADQTNLLALNAAIEAARAGEHGRGFAVVADEVRKLAERTNSSLGEIEVNVNILVQSV 644

Cfetus_venerealis_84-112_Tlp404 KDIADQTNLLALNAAIEAARAGEHGRGFAVVADEVRKLAERTNNSLGEIEVNVNILVQSV 639

Cfetus_venerealis_01-165_Tlp404 KDIADQTNLLALNAAIEAARAGEHGRGFAVVADEVRKLAERTNNSLGEIEVNVNILVQSV 658

Cfetus_fetus_04-554_Tlp404 KDIADQTNLLALNAAIEAARAGEHGRGFAVVADEVRKLAERTNNSLGEIEVNVNILVQSV 658

Cfetus_fetus_82-40_Tlp404 KDIADQTNLLALNAAIEAARAGEHGRGFAVVADEVRKLAERTNNSLGEIEVNVNILVQSV 658

****:***********************.*******:*****..**.***.*:*******

FDAARGOS_295_Tlp1 NEVAESVKEQTAGITQINDAIAQLEMVTKENVEVANVTNNITNEVNQIAVAILEDVNKKR 699

14980A_Tlp1 NEVAESVKEQTTGITQINDAIAQLESVTKENVEVANATNSITNEVNQIAAAILEDVNKKR 699

CJ677CC527_Tlp1 NEVAESVKEQTAGITQINDAIAQLETVTKENVEVANVTNNITNEVNQIAAAILEDVNKKR 699

CJ677CC012_Tlp1 NEVAESVKEQTAGITQINDAIAQLETVTKENVEVANVTNNITNEVNQIAAAILEDVNKKR 699

4031_Tlp1 NEVAESVKEQTAGITQINDAIAQLETVTKENVEVANVTNNITNEVNQIAAAILEDVDKKR 699

81116_Tlp1 NEVAESVKEQTAGITQINDAIAQLETVTKENVEVANVTNNITNEVNQIAAAILEDVDKKR 699

35925B2_Tlp1 NEVAESVKEQTAGITQINDAIAQLETVTKENVEVANVTNNITNEVNQIAAAILEDVDKKR 699

M1_Tlp1 NEVAESVKEQTAGITQINDAIAQLETVTKENVEVANVTNNITNEVNQIAAAILEDVDKKR 699

PT14_Tlp1 NEVAESVKEQTAGITQINDAIAQLETVTKENVEVANVTNNITNEVNQIAAAILEDVDKKR 699

81-176_Tlp1 NEVAESVKEQTAGITQINDAIAQLETVTKENVEVANVTNNITNEVNQIAAAILEDVSKKR 699

CVMN29710_Tlp1 NEVAESVKEQTAGITQINDAIAQLETVTKENVEVANVTNNITNEVNQIAAAILEDVNKKR 699

FB1_Tlp1 NEVAESVKEQTAGITQINDAIAQLETVTKENVEVANVTNNITNEVNQIAAAILEDVNKKR 699

BG2108_Tlp1 NEVAESVKEQTAGITQINDAIAQLETVTKENVEVANVTNNITNEVNQIAAAILEDVNKKR 699

YF2105_Tlp1 NEVAESVKEQTAGITQINDAIAQLETVTKENVEVANVTNNITNEVNQIAAAILEDVNKKR 699

YH501_Tlp1 NEVAESVKEQTAGITQINDAIAQLETVTKENVEVANVTNNITNEVNQIAAAILEDVNKKR 699

RM4661_Tlp1 NEVAESVKEQTAGITQINDAIAQLETVTKENVEVANVTNNITNEVNQIAAAILEDVNKKR 699

F38011_Tlp1 NEVAESVKEQTAGITQINDAIAQLETVTKENVEVANVTNNITNEVNQIAAAILEDVDKKR 699

T1-21_Tlp1 NEVAESVKEQTAGITQINDAIAQLETVTKENVEVANVTNNITNEVNQIAAAILEDVDKKR 699

ICDCCJ07001_Tlp1 NEVAESVKEQTAGITQINDAIAQLETVTKENVEVANVTNNITNEVNQIAAAILEDVNKKR 699

RM3196_Tlp1 NEVAESVKEQTAGITQINDAIAQLETVTKENVEVANVTNNITNEVNQIAAAILEDVNKKR 699

NCTC11168_Tlp1 NEVAESVKEQTAGITQINDAIAQLETVTKENVEVANVTNNITNEVNQIAAAILEDVNKKR 699

00-2425_Tlp1 NEVAESVKEQTAGITQINDAIAQLETVTKENVEVANVTNNITNEVNQIAAAILEDVNKKR 699

IA3902_Tlp1 NEVAESVKEQTAGITQINDAIAQLETVTKENVEVANVTNNITNEVNQIAAAILEDVNKKR 699

RM1285_Tlp1 NEVAESVKEQTAGITQINDAIAQLETVTKENVEVANVTNNITNEVNQIAAAILEDVNKKR 699

00-0949_Tlp1 NEVAESVKEQTAGITQINDAIAQLETVTKENVEVANVTNNITNEVNQIAAAILEDVNKKR 699

01-1512_Tlp1 NEVAESVKEQTAGITQINDAIAQLETVTKENVEVANVTNNITNEVNQIAAAILEDVNKKR 699

FDAARGOS_422_Tlp1 NEVAESVKEQTAGITQINDAIAQLETVTKENVEVANVTNNITNEVNQIAAAILEDVNKKR 699

FORC_056_Tlp1 NEVAESVKEQTAGITQINDAIAQLETVTKENVEVANVTNNITNEVNQIAAAILEDVNKKR 699

32488_Tlp1 NEVAESVKEQTAGITQINDAIAQLETVTKENVEVANVTNNITNEVNQIAAAILEDVNKKR 699

CFSAN032806_Tlp1 NEVAESVKEQTAGITQINDAIAQLETVTKENVEVANVTNNITNEVNQIAAAILEDVNKKR 699

YH001_Tlp1 NEVAESVKEQTAGITQINDAIAQLETVTKENVEVANVTNNITNEVNQIAAAILEDVNKKR 699

00-6200_Tlp1 NEVAESVKEQTAGITQINDAIAQLETVTKENVEVANVTNNITNEVNQIAAAILEDVNKKR 699

RM1221_Tlp1 NEVAESVKEQTAGITQINDAIAQLETVTKENVEVANVTNNITNEVNQIAAAILEDVNKKR 699

S3_Tlp1 NEVAESVKEQTAGITQINDAIAQLETVTKENVEVANVTNNITNEVNQIAAAILEDVNKKR 699

FDAARGOS_421_Tlp1 NEVAESVKEQTAGITQINDAIAQLETVTKENVEVANVTNNITNEVNQIAAAILEDVNKKR 699

FJ3124_Tlp1 NEVAESVKEQTAGITQINDAIAQLETVTKENVEVANVTNNITNEVNQIAAAILEDVNKKR 699

CG8421_Tlp1 NEVAESVKEQTAGITQINDAIAQLETVTKENVEVANVTNNITNEVNQIAAAILEDVNKKR 699

CJM1cam_Tlp1 NEVAESVKEQTAGITQINDAIAQLETVTKENVEVANVTNNITNEVNQIAAAILEDVNKKR 699

R14_Tlp1 NEVAESVKEQTAGITQINDAIAQLETVTKENVEVANVTNNITNEVNQIAAAILEDVNKKR 699

00-1597_Tlp1 NEVAESVKEQTAGITQINDAIAQLETVTKENVEVANVTNNITNEVNQIAAAILEDVNKKR 699

Cfetus_testudinum_pet-3_Tlp404 NDMSESIKEQTIGLGQINESIAQLESVTQTNAGIANTTNDITQNVNTIADNILADVNKKK 699

Cfetus_testudinum_03-427_Tlp404 NDMSESIKEQTIGLGQINESIAQLESVTQTNAGIANTTNDITQNVNTIADNILADVNKKK 704

Cfetus_venerealis_84-112_Tlp404 NDMSESIKEQTIGLGQINESIAQLESVTQTNVGIANTTNDITQNVNTIADNILADVNKKK 699

Cfetus_venerealis_01-165_Tlp404 NDMSESIKEQTIGLGQINESIAQLESVTQTNVGIANTTNDITQNVNTIADNILADVNKKK 718

Cfetus_fetus_04-554_Tlp404 NDMSESIKEQTIGLGQINESIAQLESVTQTNVGIANTTNDITQNVNTIADNILADVNKKK 718

Cfetus_fetus_82-40_Tlp404 NDMSESIKEQTIGLGQINESIAQLESVTQTNVGIANTTNDITQNVNTIADNILADVNKKK 718

*:::**:**** *: ***::***** **: *. :**.**.**::** ** ** **.**:

FDAARGOS_295_Tlp1 F 700

14980A_Tlp1 F 700

CJ677CC527_Tlp1 F 700

CJ677CC012_Tlp1 F 700

4031_Tlp1 F 700

81116_Tlp1 F 700

35925B2_Tlp1 F 700

M1_Tlp1 F 700

PT14_Tlp1 F 700

81-176_Tlp1 F 700

CVMN29710_Tlp1 F 700

FB1_Tlp1 F 700

BG2108_Tlp1 F 700

YF2105_Tlp1 F 700

YH501_Tlp1 F 700

RM4661_Tlp1 F 700

F38011_Tlp1 F 700

T1-21_Tlp1 F 700

ICDCCJ07001_Tlp1 F 700

RM3196_Tlp1 F 700

NCTC11168_Tlp1 F 700

00-2425_Tlp1 F 700

IA3902_Tlp1 F 700

RM1285_Tlp1 F 700

00-0949_Tlp1 F 700

01-1512_Tlp1 F 700

FDAARGOS_422_Tlp1 F 700

FORC_056_Tlp1 F 700

32488_Tlp1 F 700

CFSAN032806_Tlp1 F 700

YH001_Tlp1 F 700

00-6200_Tlp1 F 700

RM1221_Tlp1 F 700

S3_Tlp1 F 700

FDAARGOS_421_Tlp1 F 700

FJ3124_Tlp1 F 700

CG8421_Tlp1 F 700

CJM1cam_Tlp1 F 700

R14_Tlp1 F 700

00-1597_Tlp1 F 700

Cfetus_testudinum_pet-3_Tlp404 F 700

Cfetus_testudinum_03-427_Tlp404 F 705

Cfetus_venerealis_84-112_Tlp404 F 700

Cfetus_venerealis_01-165_Tlp404 F 719

Cfetus_fetus_04-554_Tlp404 F 719

Cfetus_fetus_82-40_Tlp404 F 719

*
