# Supplementary material for: Diversity of transducer-like proteins (Tlps) in Campylobacter
Source: PLoS One. 2019 Mar 25;14(3):e0214228. doi: 10.1371/journal.pone.0214228 (PMC6433261; doi:10.1371/journal.pone.0214228)
Supplement: S2 Archive — (ZIP) [file pone.0214228.s016.zip › Alignment D.docx]

Alignment D. Alignment of dimer interface domains in *Campylobacter* Tlps

CLUSTAL O(1.2.4) multiple sequence alignment

Tlp200_NCTC13004 ------------------------------------------------------------ 0  *C.lanienae*

Tlp201_NCTC13004 -----------------------------------------------------TNLLGDE 7  *C.lanienae*

tlp403_cfvi03/293 ------------------------------------------------------------ 0 *Cfv*

tlp402_cfvi03/293 ------------------------------------------------------------ 0 *Cfv*

Tlp401_04/554 ------------------------------------------------------------ 0 *Cff*

Tlp405_04/554 ------------------------------------------------------------ 0 *Cff*

Tlp407_03-427 ------------------------------------------------------------ 0 *Cft*

tlp400_cfvi03/293 ------------------------------------------------------------ 0 *Cfv*

Tlp409_04/554 ------------------------------------------------------------ 0 *Cff*

Tlp406_pet-3 ------------------------------------------------------------ 0 *Cft*

Tlp408_04/554 ------------------------------------------------------------ 0 *Cff*

Tlp507_ATCC33237 ------------------------------------------------------------ 0 *C. concisus*

Tlp508_ATCC33237 ------------------------------------------------------------ 0 *C. concisus*

Tlp506_P2CDO4 ------------------------------------------------------------ 0 *C. concisus*

Tlp501_P2CDO4 ------------------------------------------------------------ 0 *C. concisus*

Tlp503_P2CDO4 ------------------------------------------------------------ 0 *C. concisus*

Tlp502_P2CDO4 ------------------------------------------------------------ 0 *C. concisus*

Tlp505_P2CDO4 ------------------------------------------------------------ 0 *C. concisus*

Tlp500_P2CDO4 ------------------------------------------------------------ 0 *C. concisus*

Tlp504_P2CDO4 ------------------------------------------------------------ 0 *C. concisus*

Tlp404_84-112 ------------------------------------------------------------ 0 *Cfv*

Tlp1_NCTC11168 ------------------------------------------------------------ 0 *C. jejuni*

Tlp600_LMG24591 ------------------------------------------------------------ 0 *C. avium*

Tlp603_LMG24591 ------------------------------------------------------------ 0 *C. avium*

Tlp108_LMG11760 ------------------------------------------------------------ 0 *C. lari*

Tlp112_RM2100 ------------------------------------------------------------ 0 *C. lari*

Tlp109_RM2100 ------------------------------------------------------------ 0 *C. lari*

Tlp110_RM2100 ------------------------------------------------------------ 0 *C. lari*

Tlp111_RM2100 ------------------------------------------------------------ 0 *C. lari*

Tlp123_RM2100 ------------------------------------------------------------ 0 *C. lari*

TLp115_NCTC11845 ------------------------------------------------------------ 0 *C. lari*

Tlp116_NCTC11845 ------------------------------------------------------------ 0 *C. lari*

Tlp117_NCTC11845 ------------------------------------------------------------ 0 *C. lari*

Tlp118_NCTC11845 ------------------------------------------------------------ 0 *C. lari*

Tlp119_NCTC11845 ------------------------------------------------------------ 0 *C. lari*

Tlp101_RM16712 ------------------------------------------------------------ 0 *C. lari*

Tlp103_RM16712 ------------------------------------------------------------ 0 *C. lari*

Tlp104_RM16712 ------------------------------------------------------------ 0 *C. lari*

Tlp106_RM16712 ------------------------------------------------------------ 0 *C. lari*

Tlp102_SlaughterBeach ------------------------------------------------------------ 0 *C. lari*

Tlp105_SlaughterBeach ------------------------------------------------------------ 0 *C. lari*

Tlp121_CCUG22395 ------------------------------------------------------------ 0 *C. lari*

Tlp120_CCUG22395 ------------------------------------------------------------ 0 *C. lari*

Tlp107_LMG11760 ------------------------------------------------------------ 0 *C. lari*

Tlp122_CCUG22395 QLIELKNVLNEMLNVLEQKVGSNMNEINRVFDSYKALDFTTEVKNAKGGVEVTANVLGQE 60 *C. lari*

Tlp113_RM2100 ------------------------------------------------------------ 0 *C. lari*

Tlp100_SlaughterBeach ------------------------------------------------------------ 0 *C. lari*

Tlp114_RM1607 ------------------------------------------------------------ 0 *C. lari*

Tlp601_LMG24591 ------------------------------------------------------------ 0 *C. avium*

Tlp602_LMG24591 ------------------------------------------------------------ 0 *C. avium*

Tlp300_ATCC51209 ------------------------------------------------------------ 0 *C. helveticus*

Tlp301_ATCC51209 ------------------------------------------------------------ 0 *C. helveticus*

Tlp302_ATCC51209 ------------------------------------------------------------ 0 *C. helveticus*

Tlp303_ATCC51209 ------------------------------------------------------------ 0 *C. helveticus*

Tlp304_ATCC51209 ------------------------------------------------------------ 0 *C. helveticus*

Tlp2_NCTC11168 ------------------------------------------------------------ 0 *C. jejuni*

Tlp3_NCTC11168 ------------------------------------------------------------ 0 *C. jejuni*

Tlp4_NCTC11168 ------------------------------------------------------------ 0 *C. jejuni*

Tlp11_00-2425 ------------------------------------------------------------ 0 *C. jejuni*

Tlp12_RM1221 ------------------------------------------------------------ 0 *C. jejuni*

Tlp13_00-1597 ------------------------------------------------------------ 0 *C. jejuni*

Tlp14_00-1597 ------------------------------------------------------------ 0 *C. jejuni*

Tlp19_T1-21 ------------------------------------------------------------ 0 *C. jejuni*

Tlp21_FDAARGOS_295 ------------------------------------------------------------ 0 *C. jejuni* subsp. *doylei*

Tlp22_HF5-4A-4 ------------------------------------------------------------ 0 *C. jejuni*

Tlp23_ICDCCJ07001 ------------------------------------------------------------ 0 *C. jejuni*

Tlp25_CG8421 ------------------------------------------------------------ 0 *C. jejuni*

Tlp17_4031 ------------------------------------------------------------ 0 *C. jejuni*

Tlp24_M1 ------------------------------------------------------------ 0 *C. coli*

Tlp15_RM1875 ------------------------------------------------------------ 0 *C. coli*

Tlp16_FB1 ------------------------------------------------------------ 0 *C. coli*

Tlp20_CO2-160 ------------------------------------------------------------ 0 *C. coli*

Tlp18_76339 ------------------------------------------------------------ 0 *C. coli*

Tlp200_NCTC13004 ------------------ADELKGFVASLNEGAKSQSESLQESAAAVEEMSSSMNSINDR 42

Tlp201_NCTC13004 VTKMLKANLEQANNLQNKADELKGFVASLNEGAKSQSESLQESAAAVEEMSSSMNSINDR 67

tlp403_cfvi03/293 ----------------------------------SQASSLGESAAAIEEMSNSMTSINER 26

tlp402_cfvi03/293 ------------------------------------------------------------ 0

Tlp401_04/554 ------------------------------------------------------------ 0

Tlp405_04/554 ------------------------------------------------------------ 0

Tlp407_03-427 ------------------------------------------------------------ 0

tlp400_cfvi03/293 ----------------------------LNDSARSQANSLQESAAAVEEMSSSMSSINER 32

Tlp409_04/554 ------------------------------------------------------------ 0

Tlp406_pet-3 ----------QANNLKEKANNLKEYVTTLNDSARSQANSLQESAAAVEEMSSSMSSINER 50

Tlp408_04/554 ----------------------------LNDSARSQANSLQESAAAVEEMSSSMSSINER 32

Tlp507_ATCC33237 ------------ESLEAKAKILAESMKSLTDGAHKQADSIQESAAAIEEMSSSMNAISQK 48

Tlp508_ATCC33237 --------------LEEKAKNLDASMKQVTQGASTQANSLQESAAAIEQMSSSMSAISQK 46

Tlp506_P2CDO4 ------------QNLEEKATMLAESMRNLTDGASKQADSIQESAAAVEQMSSSMNAISQK 48

Tlp501_P2CDO4 --------------LEEKATTLASAMKELTQGASTQASSLQESAAAVEQMSSSMNAISQK 46

Tlp503_P2CDO4 --------------LEEKATTLASAMKELTQGASTQASSLQESAAAVEQMSSSMNAISQK 46

Tlp502_P2CDO4 -------------------------------------------AAAVEQMSSSMNAISQK 17

Tlp505_P2CDO4 ---------------------------------------------AVEQMSSSMNAISQK 15

Tlp500_P2CDO4 ---------------------------------------------AVEQMSSSMNAISQK 15

Tlp504_P2CDO4 ---------------------------------------------AVEQMSSSMNAISQK 15

Tlp404_84-112 -------------DLAEQSKELRESMQKLTDGSRTQAHSLEQSAAAVEQISCSMQSISDR 47

Tlp1_NCTC11168 -----------------------------------------------------------K 1

Tlp600_LMG24591 ------------------ADELKESMQKLTDGSKAQANSLEQSAAAVEEISSSMQNISGK 42

Tlp603_LMG24591 ------------------ADELKESMQKLTDGSKAQANSLEQSAAAVEEISSSMQNISGK 42

Tlp108_LMG11760 ------------ELLKQKAEALKQSMQELTNDATHQTSSLQESARALEQMNSAMSEISIK 48

Tlp112_RM2100 ----------------------------------------EETAAALEEITSSMQNVSSK 20

Tlp109_RM2100 ----------------------------------------EETAAALEEITSSMQNVSHK 20

Tlp110_RM2100 ----------------------------------------EETAAALEEITSSMQNVSHK 20

Tlp111_RM2100 ----------------------------------------EETAAALEEITSSMQNVSHK 20

Tlp123_RM2100 ----------------------------------------EETAAALEEITSSMQNVSHK 20

TLp115_NCTC11845 ---------------ADESGKLQSAVKDLTDSSSSQASSLEETAAALEEITSSMQNVSHK 45

Tlp116_NCTC11845 ---------------ADESGKLQSAVKDLTDSSSSQASSLEETAAALEEITSSMQNVSHK 45

Tlp117_NCTC11845 ---------------ADESGKLQSAVKDLTDSSSSQASSLEETAAALEEITSSMQNVSHK 45

Tlp118_NCTC11845 ---------------ADESGKLQSAVKDLTDSSSSQASSLEETAAALEEITSSMQNVSHK 45

Tlp119_NCTC11845 ---------------ADESGKLQSAVKDLTDSSSSQASSLEETAAALEEITSSMQNVSHK 45

Tlp101_RM16712 ---------------ADESGKLQSAVKDLTDSSSSQASSLEETAAALEEITSSMQNVSHK 45

Tlp103_RM16712 ---------------ADESGKLQSAVKDLTDSSSSQASSLEETAAALEEITSSMQNVSHK 45

Tlp104_RM16712 ---------------ADESGKLQSAVKDLTDSSSSQASSLEETAAALEEITSSMQNVSHK 45

Tlp106_RM16712 ---------------ADESGKLQSAVKDLTDSSSSQASSLEETAAALEEITSSMQNVSHK 45

Tlp102_SlaughterBeach ---------------ADESGKLQSAVKNLTDSSSSQASSLEETAAALEEITSSMQNVSHK 45

Tlp105_SlaughterBeach ---------------ADESGKLQSAVKNLTDSSSSQASSLEETAAALEEITSSMQNVSHK 45

Tlp121_CCUG22395 ---------------ADESGKLQSAVKNLTDSSSSQASSLEETAAALEEITSSMQNVSHK 45

Tlp120_CCUG22395 -----------------ADESLQSAVKNLTDSSSSQASSLEETAAALEEITSSMQNVSHK 43

Tlp107_LMG11760 ---------------ASESGKLQSAVKNLTDSSSSQASSLEETAAALEEITSSMQNVSHK 45

Tlp122_CCUG22395 IVAMLRQSSEFASLLADESGKLQSAVKNLTDSSSSQASSLEETAAALEEITSSMQNVSHK 120

Tlp113_RM2100 ----------------------------------------EETAAALEEITSSMQNVSHK 20

Tlp100_SlaughterBeach ---------------ADESGKLQSAVKNLTDSSSSQASSLEETAAALEEITSSMQNVSHK 45

Tlp114_RM1607 ---------------ADESGKLQSAVKNLTDSSSSQASSLEETAAALEEITSSMQNVSHK 45

Tlp601_LMG24591 --------------------------------SKSQASSLEESAAALEEITSSMQNVSSK 28

Tlp602_LMG24591 --------------------------------SKSQASSLEESAAALEEITSSMQNVSSK 28

Tlp300_ATCC51209 ---------------ANESSKLQNAVQNLTTSSNSQAASLEETAAALEEITSSMQNVSQK 45

Tlp301_ATCC51209 ---------------ANESSKLQNAVQNLTTSSNSQAASLEETAAALEEITSSMQNVSQK 45

Tlp302_ATCC51209 ---------------ANESSKLQNAVQNLTTSSNSQAASLEETAAALEEITSSMQNVSQK 45

Tlp303_ATCC51209 ---------------ANESSKLQNAVQNLTTSSNSQAASLEETAAALEEITSSMQNVSQK 45

Tlp304_ATCC51209 ---------------ANESSKLQNAVQNLTTSSNSQAASLEETAAALEEITSSMQNVSQK 45

Tlp2_NCTC11168 --------------------KLQTAVQSLTTSSNSQAQSLEETAAALEEITSSMQNVSVK 40

Tlp3_NCTC11168 ---------------ANESGKLQTAVQSLTTSSNSQAQSLEETAAALEEITSSMQNVSVK 45

Tlp4_NCTC11168 ---------------ANESGKLQTAVQSLTTSSNSQAQSLEETAAALEEITSSMQNVSVK 45

Tlp11_00-2425 ---------------ANESGKLQTAVQSLTTSSNSQAQSLEETAAALEEITSSMQNVSVK 45

Tlp12_RM1221 ---------------ANESGKLQTAVQSLTTSSNSQAQSLEETAAALEEITSSMQNVSVK 45

Tlp13_00-1597 ---------------ANESGKLQTAVQSLTTSSNSQAQSLEETAAALEEITSSMQNVSVK 45

Tlp14_00-1597 ---------------ANESGKLQTAVQSLTTSSNSQAQSLEETAAALEEITSSMQNVSVK 45

Tlp19_T1-21 ---------------ANESGKLQTAVQSLTTSSNSQAQSLEETAAALEEITSSMQNVSVK 45

Tlp21_FDAARGOS_295 ---------------ANESGKLQTAVQSLTTSSNSQAQSLEETAAALEEITSSMQNVSVK 45

Tlp22_HF5-4A-4 ---------------ANESGKLQTAVQSLTTSSNSQAQSLEETAAALEEITSSMQNVSVK 45

Tlp23_ICDCCJ07001 ---------------ANESGKLQTAVQSLTTSSNSQAQSLEETAAALEEITSSMQNVSVK 45

Tlp25_CG8421 ---------------ANESGKLQTAVQSLTTSSNSQAQSLEETAAALEEITSSMQNVSVK 45

Tlp17_4031 ---------------ANESGKLQTAVQSLTTSSNSQAQSLEETAAALEEITSSMQNVSVK 45

Tlp24_M1 ---------------ANESGKLQTAVQSLTTSSNSQAQSLEETAAALEEITSSMQNVSVK 45

Tlp15_RM1875 ---------------ASESSKLQSAVQNLTSSSNSQAASLEETAAALEEITSSMQNVSVK 45

Tlp16_FB1 ---------------ASESSKLQSAVQNLTSSSNSQAASLEETAAALEEITSSMQNVSVK 45

Tlp20_CO2-160 ---------------ASESSKLQSAVQNLTSSSNSQAASLEETAAALEEITSSMQNVSVK 45

Tlp18_76339 ---------------ASESSKLQSAVQNLTSSSNSQAASLEETAAALEEITSSMQNVSVK 45

Tlp200_NCTC13004 ASEVIKQSEDIKNIITIIRDIADQTNLLALNAAIEAARAGEHG--RGFAVVADEVRKLA- 99

Tlp201_NCTC13004 ASEVIKQSEDIKNIITIIRDIADQTNLLALNAAIEAARAGEHG--RGFAVVADEVRKLA- 124

tlp403_cfvi03/293 TMEVIKQSEDIKSIITIIRDIADQTNLLALNAAIEAARAGDHG--RGFAVVADEVRKLA- 83

tlp402_cfvi03/293 -GEVIKQSEDIKNIITIIRDIADQTNLLALNAAIEAARAGEHG--RGFAVVADEVRQLA- 56

Tlp401_04/554 -GDVIKQSEDIKSIITIIRDIADQTNLLALNAAIEAARAGEHG--RGFAVVADEVRQLA- 56

Tlp405_04/554 -GDVIKQSEDIKSIITIIRDIADQTNLLALNAAIEAARAGEHG--RGFAVVADEVRQLA- 56

Tlp407_03-427 -GDVIKQSEDIKSIITIIRDIADQTNLLALNAAIEAARAGEHG--RGFAVVADEVRQLA- 56

tlp400_cfvi03/293 AGEVIKQSEDIKNIITIIRDIADQTNLLALNAAIEAARAGEHG--RGFAVVADEVRQLA- 89

Tlp409_04/554 -GEVIKQSEDIKNIITIIRDIADQTNLLALNAAIEAARAGDHG--RGFAVVADEVRQLA- 56

Tlp406_pet-3 AGEVIKQSEDIKNIITIIRDIADQTNLLALNAAIEAARAGDHG--RGFAVVADEVRQLA- 107

Tlp408_04/554 AGEVIKQSEDIKNIITIIRDIADQTNLLALNAAIEAARAGDHG--RGFAVVADEVRQLA- 89

Tlp507_ATCC33237 ASDVTRQSEEIKNIIVIIRDIADQTNLLALNAAIEAARAGEHG--RGFAVVADEVRKLA- 105

Tlp508_ATCC33237 TVDVIKQSEEIKNIIVIIRDIADQTNLLALNAAIEAARAGEHG--RGFAVVADEVRKLA- 103

Tlp506_P2CDO4 TGDVIRQSEEIKNIIVIIRDIADQTNLLALNAAIEAARAGEHG--RGFAVVADEVRKLA- 105

Tlp501_P2CDO4 TADVIRQSDEIKNIITIIRDIADQTNLLALNAAIEAARAGEHG--RGFAVVADEVRKLA- 103

Tlp503_P2CDO4 TADVIRQSDEIKNIITIIRDIADQTNLLALNAAIEAARAGEHG--RGFAVVADEVRKLA- 103

Tlp502_P2CDO4 TADVIRQSDEIKNIITIIRDIADQTNLLALNAAIEAARAGEHG--RGFAVVADEVRKLA- 74

Tlp505_P2CDO4 TADVIRQSDEIKNIITIIRDIADQTNLLALNAAIEAARAGEHG--RGFAVVADEVRKLA- 72

Tlp500_P2CDO4 TADVIRQSDEIKNIITIIRDIADQTNLLALNAAIEAARAGEHG--RGFAVVADEVRKLA- 72

Tlp504_P2CDO4 TADVIRQSDEIKNIITIIRDIADQTNLLALNAAIEAARAGEHG--RGFAVVADEVRKLA- 72

Tlp404_84-112 TVETTKQAEDIKNIVGVIKDIADQTNLLALNAAIEAARAGEHG--RGFAVVADEVRKLA- 104

Tlp1_NCTC11168 TVEVASQADDIKNIVNVIKDIAEQTNLLALNAAIEAARAGEHG--RGFAVVADEVRQLA- 58

Tlp600_LMG24591 TEDVARQADDIKSIVEVIKDIADQTNLLALNAAIEAARAGEHG--RGFAVVADEVRQLA- 99

Tlp603_LMG24591 TEDVARQADDIKSIVEVIKDIADQTNLLALNAAIEAARAGEHG--RGFAVVADEVRQLA- 99

Tlp108_LMG11760 TQDVVKQSNDIKNVTTVISDIADQINLLALNAAIEAARAGEHG--RGFAVVADEVRNLA- 105

Tlp112_RM2100 TSEVIAQSEEIKNVTSIIGDIADQINLLALNAAIEAARAGEHG--RGFAVVADEVRNLA- 77

Tlp109_RM2100 TSEVIAQSEEIKNVTSIIGDIADQINLLALNAAIEAARAGEHG--RGFAVVADEVRNLA- 77

Tlp110_RM2100 TSEVIAQSEEIKNVTSIIGDIADQINLLALNAAIEAARAGEHG--RGFAVVADEVRNLA- 77

Tlp111_RM2100 TSEVIAQSEEIKNVTSIIGDIADQINLLALNAAIEAARAGEHG--RGFAVVADEVRNLA- 77

Tlp123_RM2100 TSEVIAQSEEIKNVTSIIGDIADQINLLALNAAIEAARAGEHG--RGFAVVADEVRNLA- 77

TLp115_NCTC11845 TSEVIAQSEEIKNVTSIIGDIADQINLLALNAAIEAARAGEHG--RGFAVVADEVRNLA- 102

Tlp116_NCTC11845 TSEVIAQSEEIKNVTSIIGDIADQINLLALNAAIEAARAGEHG--RGFAVVADEVRNLA- 102

Tlp117_NCTC11845 TSEVIAQSEEIKNVTSIIGDIADQINLLALNAAIEAARAGEHG--RGFAVVADEVRNLA- 102

Tlp118_NCTC11845 TSEVIAQSEEIKNVTSIIGDIADQINLLALNAAIEAARAGEHG--RGFAVVADEVRNLA- 102

Tlp119_NCTC11845 TSEVIAQSEEIKNVTSIIGDIADQINLLALNAAIEAARAGEHG--RGFAVVADEVRNLA- 102

Tlp101_RM16712 TSEVIAQSEEIKNVTSIIGDIADQINLLALNAAIEAARAGEHG--RGFAVVADEVRNLA- 102

Tlp103_RM16712 TSEVIAQSEEIKNVTSIIGDIADQINLLALNAAIEAARAGEHG--RGFAVVADEVRNLA- 102

Tlp104_RM16712 TSEVIAQSEEIKNVTSIIGDIADQINLLALNAAIEAARAGEHG--RGFAVVADEVRNLA- 102

Tlp106_RM16712 TSEVIAQSEEIKNVTSIIGDIADQINLLALNAAIEAARAGEHG--RGFAVVADEVRNLA- 102

Tlp102_SlaughterBeach TSEVIAQSEEIKNVTSIIGDIADQINLLALNAAIEAARAGEHG--RGFAVVADEVRNLA- 102

Tlp105_SlaughterBeach TSEVIAQSEEIKNVTSIIGDIADQINLLALNAAIEAARAGEHG--RGFAVVADEVRNLA- 102

Tlp121_CCUG22395 TSEVIAQSEEIKNVTSIIGDIADQINLLALNAAIEAARAGEHG--RGFAVVADEVRNLA- 102

Tlp120_CCUG22395 TSEVIAQSEEIKNVTSIIGDIADQINLLALNAAIEAARAGEHGKGRGFAVVADEVRNLA- 102

Tlp107_LMG11760 TSEVIAQSEEIKNVTSIIGDIADQINLLALNAAIEAARAGEHG--RGFAVVADEVRNLA- 102

Tlp122_CCUG22395 TSEVIAQSEEIKNVTSIIGDIADQINLLALNAAIEAARAGEHG--RGFAVVADEVRNLA- 177

Tlp113_RM2100 TSEVIAQSEEIKNVTSIIGDIADQINLLALNAAIEAARAGEHG--RGFAVVADEVRNLA- 77

Tlp100_SlaughterBeach TSEVIAQSEEIKNVTSIIGDIADQINLLALNAAIEAARAGEHG--RGFAVVADEVRNLA- 102

Tlp114_RM1607 TSEVIAQSEEIKNVTSIIGDIADQINLLALNAAIEAARAGEHG--RGFAVVADEVRNLA- 102

Tlp601_LMG24591 TTDVIAQSEEIKSITNIIGDIAEQINLLALNAAIEAARAGEHG--RGFAVVADEVRQLA- 85

Tlp602_LMG24591 TTDVIAQSEEIKSITNIIGDIAEQINLLALNAAIEAARAGEHG--RGFAVVADEVRQLKL 86

Tlp300_ATCC51209 TSDVITQSEEIKNVTSIIGDIADQINLLALNAAIEAARAGEHG--RGFAVVADEVRKLA- 102

Tlp301_ATCC51209 TSDVITQSEEIKNVTSIIGDIADQINLLALNAAIEAARAGEHG--RGFAVVADEVRKLA- 102

Tlp302_ATCC51209 TSDVITQSEEIKNVTSIIGDIADQINLLALNAAIEAARAGEHG--RGFAVVADEVRKLA- 102

Tlp303_ATCC51209 TSDVITQSEEIKNVTSIIGDIADQINLLALNAAIEAARAGEHG--RGFAVVADEVRKLA- 102

Tlp304_ATCC51209 TSDVITQSEEIKNVTSIIGDIADQINLLALNAAIEAARAGEHG--RGFAVVADEVRKLA- 102

Tlp2_NCTC11168 TSDVITQSEEIKNVTGIIGDIADQINLLALNAAIEAARAGEHG--RGFAVVADEVRKLA- 97

Tlp3_NCTC11168 TSDVITQSEEIKNVTGIIGDIADQINLLALNAAIEAARAGEHG--RGFAVVADEVRKLA- 102

Tlp4_NCTC11168 TSDVITQSEEIKNVTGIIGDIADQINLLALNAAIEAARAGEHG--RGFAVVADEVRKLA- 102

Tlp11_00-2425 TSDVITQSEEIKNVTGIIGDIADQINLLALNAAIEAARAGEHG--RGFAVVADEVRKLA- 102

Tlp12_RM1221 TSDVITQSEEIKNVTGIIGDIADQINLLALNAAIEAARAGEHG--RGFAVVADEVRKLA- 102

Tlp13_00-1597 TSDVITQSEEIKNVTGIIGDIADQINLLALNAAIEAARAGEHG--RGFAVVADEVRKLA- 102

Tlp14_00-1597 TSDVITQSEEIKNVTGIIGDIADQINLLALNAAIEAARAGEHG--RGFAVVADEVRKLA- 102

Tlp19_T1-21 TSDVITQSEEIKNVTGIIGDIADQINLLALNAAIEAARAGEHG--RGFAVVADEVRKLA- 102

Tlp21_FDAARGOS_295 TSDVITQSEEIKNVTGIIGDIADQINLLALNAAIEAARAGEHG--RGFAVVADEVRKLA- 102

Tlp22_HF5-4A-4 TSDVITQSEEIKNVTGIIGDIADQINLLALNAAIEAARAGEHG--RGFAVVADEVRKLA- 102

Tlp23_ICDCCJ07001 TSDVITQSEEIKNVTGIIGDIADQINLLALNAAIEAARAGEHG--RGFAVVADEVRKLA- 102

Tlp25_CG8421 TSDVITQSEEIKNVTGIIGDIADQINLLALNAAIEAARAGEHG--RGFAVVADEVRKLA- 102

Tlp17_4031 TSDVITQSEEIKNVTGIIGDIADQINLLALNAAIEAARAGEHG--RGFAVVADEVRKLA- 102

Tlp24_M1 TSDVITQSEEIKNVTGIIGDIADQINLLALNAAIEAARAGEHG--RGFAVVADEVRKLA- 102

Tlp15_RM1875 TSDVITQSEEIKNVTGIIGDIADQINLLALNAAIEAARAGEHG--RGFAVVADEVRKLA- 102

Tlp16_FB1 TSDVITQSEEIKNVTGIIGDIADQINLLALNAAIEAARAGEHG--RGFAVVADEVRKLA- 102

Tlp20_CO2-160 TSDVITQSEEIKNVTGIIGDIADQINLLALNAAIEAARAGEHG--RGFAVVADEVRKLA- 102

Tlp18_76339 LSDVITQSEEIKNVTGIIGDIADQINLLALNAAIEAARAGEHG--RGFAVVADEVRKLA- 102

:. *:::**.: :* ***:* ***************:** ***********:*

Tlp200_NCTC13004 ERTQKSLGEIEANVNILSQSINEMSQSISEQTTAINQINEAIVNVDGLTRQNRQIAQDSN 159 *C.lanienae*

Tlp201_NCTC13004 ERTQKSLGEIEANVNILSQSINEMSQSISE-TTAINQINEAIVNVDGLTRQNRQIAQDSN 183 *C.lanienae*

tlp403_cfvi03/293 ERTGKSLGEIEANVNILSQSINDMSQSIKEQTETMNQINQSVANVDELTKQNVDIVNDTN 143

tlp402_cfvi03/293 ERTGKSLAEIEANVNILSQGINEMSQSINEQTEAINQINEAVATVDEQTKQNVTIAQNSN 116

Tlp401_04/554 ERTGKSLAEIEANVNILSQGINEMSQSINEQTEAINQINEAVATVDEQTKQNVTIAQNSN 116

Tlp405_04/554 ERTGKSLAEIEANVNILSQGINEMSQSINEQTEAINQINEAVATVDEQTKQNVTIAQNSN 116

Tlp407_03-427 ERTGKSLAEIEANVNILSQGINEMSQSINEQTEAINQINEAVATVDEQTKQNVAIAQNSN 116

tlp400_cfvi03/293 ERTGKSLAEIEANVNILSQGINEMSQSINEQTEAINQINEAVANVDEQTKQNLAIASNTD 149

Tlp409_04/554 ERTQKSLGEIEANVNILSQSINEMSQSISEQTEAINQINEAVANVDEQTKQNLAIASNTD 116

Tlp406_pet-3 ERTQKSLGEIEANVNILSQSINEMSQSISEQTEAINQINEAVANVDEQTKQNLAIASNTD 167

Tlp408_04/554 ERTQKSLGEIEANVNILSQSINEMSQSISEQTEAINQINEAVANVDEQTKQNLAIASNTD 149

Tlp507_ATCC33237 ERTQKSLGEIEANANVLAQSINEMSESIREQSEGINMINQSVSQIDSITKQNINIVGTTN 165

Tlp508_ATCC33237 ERTQKSLGEIETNANILTQSINEMSESIREQSEGINMINQSVSQIDSITKQNVDIVSSTN 163

Tlp506_P2CDO4 ERTQKSLGEIEANTNVLTQSINEMSESIKEQAEGINMINRSVAQIDNVTKENRSVVSNTN 165

Tlp501_P2CDO4 ERTQKSLGEIEANTNVLAQSINEMSESIKEQSEGINMINQSVAQIDHLTKENVVIANRAN 163

Tlp503_P2CDO4 ERTQKSLGEIEANTNVLAQSINEMSESIKEQSEGINMINQSVAQIDHLTKENVVIANRAN 163

Tlp502_P2CDO4 ERTQKSLGEIEANTNVLAQSINEMSESIKEQSEGINMINQSVAQIDHLTKENVVIANQAN 134

Tlp505_P2CDO4 ERTQKSLGEIEANTNVLAQSINEMSESIKEQSEGINMINQSVAQIDHLTKENVVIANQAN 132

Tlp500_P2CDO4 ERTQKSLGEIEANTNVLAQSINEMSESIKEQSEGINMINQSVAQIDNLTKENVVIANQAN 132

Tlp504_P2CDO4 ERTQKSLGEIEANTNVLAQSINEMSESIKEQSEGINMINQSVAQIDNLTKENVVIANQAN 132

Tlp404_84-112 ERTNNSLGEIEVNVNILVQSVNDMSESIKEQTIGLGQINESIAQLESVTQTNVGIANTTN 164

Tlp1_NCTC11168 ERTGKSLSEIEANINILVQSVNEVAESVKEQTAGITQINDAIAQLETVTKENVEVANVTN 118

Tlp600_LMG24591 ERTGKSLSEIEANINLLVQSVNEVSESIREQTAGVTQINESIAELESVTRENVSVANDTN 159

Tlp603_LMG24591 ERTGKSLSEIEANINLLVQSVNEVSESIREQTAGVTQINESIAELESVTRENVSVANDTN 159

Tlp108_LMG11760 ERTQKSLGEIEANTNILVQSINDMGEAIKEEADDISQINESVATIEKLTQQNSQTAMQTN 165

Tlp112_RM2100 ERTQKSLGEIEANTNILVQSINEMGESIKEQTTGITQINDAVAQIDHVTQENLKIAKDSA 137

Tlp109_RM2100 ERTQKSLGEIEANTNILVQSINEMGESIKEQTTGITQINDAVAQIDHVTQENLKIANDSA 137

Tlp110_RM2100 ERTQKSLGEIEANTNILVQSINEMGESIKEQTTGITQINDAVAQIDHVTQENLKIANDSA 137

Tlp111_RM2100 ERTQKSLGEIEANTNILVQSINEMGESIKEQTTGITQINDAVAQIDHVTQENLKIANDSA 137

Tlp123_RM2100 ERTQKSLGEIEANTNILVQSINEMGESIKEQTTGITQINDAVAQIDHVTQENLKIANDSA 137

TLp115_NCTC11845 ERTQKSLGEIEANTNILVQSINEMGESIKEQTTGITQINDAVAQIDHVTQENLKIAKDSA 162

Tlp116_NCTC11845 ERTQKSLGEIEANTNILVQSINEMGESIKEQTTGITQINDAVAQIDHVTQENLKIAKDSA 162

Tlp117_NCTC11845 ERTQKSLGEIEANTNILVQSINEMGESIKEQTTGITQINDAVAQIDHVTQENLKIAKDSA 162

Tlp118_NCTC11845 ERTQKSLGEIEANTNILVQSINEMGESIKEQTTGITQINDAVAQIDHVTQENLKIAKDSA 162

Tlp119_NCTC11845 ERTQKSLGEIEANTNILVQSINEMGESIKEQTTGITQINDAVAQIDHVTQENLKIAKDSA 162

Tlp101_RM16712 ERTQKSLGEIEANTNILVQSINEMGESIKEQTTGITQINDAVAQIDHVTQENLKIAKDSA 162

Tlp103_RM16712 ERTQKSLGEIEANTNILVQSINEMGESIKEQTTGITQINDAVAQIDHVTQENLKIAKDSA 162

Tlp104_RM16712 ERTQKSLGEIEANTNILVQSINEMGESIKEQTTGITQINDAVAQIDHVTQENLKIAKDSA 162

Tlp106_RM16712 ERTQKSLGEIEANTNILVQSINEMGESIKEQTTGITQINDAVAQIDHVTQENLKIAKDSA 162

Tlp102_SlaughterBeach ERTQKSLGEIEANTNILVQSINEMGESIKEQTTGITQINDAVAQIDHVTQENLKIAKDSA 162

Tlp105_SlaughterBeach ERTQKSLGEIEANTNILVQSINEMGESIKEQTTGITQINDAVAQIDHVTQENLKIAKDSA 162

Tlp121_CCUG22395 ERTQKSLGEIEANTNILVQSINEMGESIKEQTTGITQINDAVAQIDHVTQENLKIAKDSA 162

Tlp120_CCUG22395 ERTQKSLGEIEANTNILVQSINEMGESIKEQTTGITQINDAVAQIDHVTQENLKIANDSA 162

Tlp107_LMG11760 ERTQKSLGEIEANTNILVQSINEMGESIKEQTTGITQINDAVAQIDHVTQENLKIANDSA 162

Tlp122_CCUG22395 ERTQKSLGEIEANTNILVQSINEMGESIKEQTTGITQINDAVAQIDHVTQENLKIANDSA 237

Tlp113_RM2100 ERTQKSLGEIEANTNILVQSINEMGESIKEQTTGITQINDAVAQIDHVTQENLKIANDSA 137

Tlp100_SlaughterBeach ERTQKSLGEIEANTNILVQSINEMGESIKEQTTGITQINDAVAQIDHVTQENLKIAKDSA 162

Tlp114_RM1607 ERTQKSLGEIEANTNILVQSINEMGESIKEQTTGITQINDAVAQIDHVTQENLKIAKDSA 162

Tlp601_LMG24591 EKTQKSLSEIEANINLLVQSINDMAESIKEQTTGITQINDAVAQIESVTRDNVRIA---- 141

Tlp602_LMG24591 EKTQKSLSEIEANINLLVQSINDMAESIKEQTTGITQINDAVAQIESVTRDNVRIA---- 142

Tlp300_ATCC51209 ERTQKSLSEIEANTNLLVQSINDMAESIKEQTAGITQINESVAQIDQTTKDNVEIANESA 162

Tlp301_ATCC51209 ERTQKSLSEIEANTNLLVQSINDMAESIKEQTAGITQINESVAQIDQTTKDNVEIANESA 162

Tlp302_ATCC51209 ERTQKSLSEIEANTNLLVQSINDMAESIKEQTAGITQINESVAQIDQTTKDNVEIANESA 162

Tlp303_ATCC51209 ERTQKSLSEIEANTNLLVQSINDMAESIKEQTAGITQINESVAQIDQTTKDNVEIANESA 162

Tlp304_ATCC51209 ERTQKSLSEIEANTNLLVQSINDMAESIKEQTAGITQINESVAQIDQTTKDNVEIANESA 162

Tlp2_NCTC11168 ERTQKSLSEIEANTNLLVQSINDMAESIKEQTAGITQINDSVAQIDQTTKDNVEIANESA 157

Tlp3_NCTC11168 ERTQKSLSEIEANTNLLVQSINDMAESIKEQTAGITQINDSVAQIDQTTKDNVEIANESA 162

Tlp4_NCTC11168 ERTQKSLSEIEANTNLLVQSINDMAESIKEQTAGITQINDSVAQIDQTTKDNVEIANESA 162

Tlp11_00-2425 ERTQKSLSEIEANTNLLVQSINDMAESIKEQTAGITQINDSVAQIDQTTKDNVEIANESA 162

Tlp12_RM1221 ERTQKSLSEIEANTNLLVQSINDMAESIKEQTAGITQINDSVAQIDQTTKDNVEIANESA 162

Tlp13_00-1597 ERTQKSLSEIEANTNLLVQSINDMAESIKEQTAGITQINDSVAQIDQTTKDNVEIANESA 162

Tlp14_00-1597 ERTQKSLSEIEANTNLLVQSINDMAESIKEQTAGITQINDSVAQIDQTTKDNVEIANESA 162

Tlp19_T1-21 ERTQKSLSEIEANTNLLVQSINDMAESIKEQTAGITQINDSVAQIDQTTKDNVEIANESA 162

Tlp21_FDAARGOS_295 ERTQKSLSEIEANTNLLVQSINDMAESIKEQTAGITQINDSVAQIDQTTKDNVEIANESA 162

Tlp22_HF5-4A-4 ERTQKSLSEIEANTNLLVQSINDMAESIKEQTAGITQINDSVAQIDQTTKDNVEIANESA 162

Tlp23_ICDCCJ07001 ERTQKSLSEIEANTNLLVQSINDMAESIKEQTAGITQINDSVAQIDQTTKDNVEIANESA 162

Tlp25_CG8421 ERTQKSLSEIEANTNLLVQSINDMAESIKEQTAGITQINDSVAQIDQTTKDNVEIANESA 162

Tlp17_4031 ERTQKSLSEIEANTNLLVQSINDMAESIKEQTAGITQINESVAQIDQTTKDNVEIANESA 162

Tlp24_M1 ERTQKSLSEIEANTNLLVQSINDMAESIKEQTAGITQINESVAQIDQTTKDNVEIANESA 162

Tlp15_RM1875 ERTQKSLSEIEANTNLLVQSINDMAESIKEQTAGITQINESVAQIDQTTKDNVEIANESA 162

Tlp16_FB1 ERTQKSLSEIEANTNLLVQSINDMAESIKEQTAGITQINESVAQIDQTTKDNVEIANESA 162

Tlp20_CO2-160 ERTQKSLSEIEANTNLLVQSINDMAESIKEQTAGITQINESVAQIDQTTKDNVEIANESA 162

Tlp18_76339 ERTQKSLSEIEANTNLLVQSINDMAESIKEQTAGITQINESVAQIDQTTKDNVEIANESA 162

*:* :**.***.* *:* *.:*::.::: * : : ** :: :: *: * .

Tlp200_NCTC13004 IVANEVDSI---------- 168

Tlp201_NCTC13004 IVANEVDSI---------- 192

tlp403_cfvi03/293 KISIEVENIANSI------ 156

tlp402_cfvi03/293 KITNEVESIANE------- 128

Tlp401_04/554 KITNEVESIANIIVDEVKK 135

Tlp405_04/554 KITNEVESIAN-------- 127

Tlp407_03-427 KITNEVESIAN-------- 127

tlp400_cfvi03/293 RVTIEVETIANEV------ 162

Tlp409_04/554 RVTIEVETIANEVVSEVKR 135

Tlp406_pet-3 RVTLEVETIANEVVSEV-- 184

Tlp408_04/554 RVTIEVETIA--------- 159

Tlp507_ATCC33237 EITDQIDDMAKTI------ 178

Tlp508_ATCC33237 EITAQIDEMAKT------- 175

Tlp506_P2CDO4 DVTSEID------------ 172

Tlp501_P2CDO4 EVTSDVDNMA--------- 173

Tlp503_P2CDO4 EVTSDVDNMA--------- 173

Tlp502_P2CDO4 EVTSEVDEMAKAIVEDV-- 151

Tlp505_P2CDO4 EVTSEVDEMAKAIVEDV-- 149

Tlp500_P2CDO4 EVTSEVDEMAKAIVEEV-- 149

Tlp504_P2CDO4 EVTSEVDEMAKAIVED--- 148

Tlp404_84-112 DI----------------- 166

Tlp1_NCTC11168 NITNEVNQIA--------- 128

Tlp600_LMG24591 SITEEVNTIASDILTDV-- 176

Tlp603_LMG24591 SITEEVNTIASDILTDV-- 176

Tlp108_LMG11760 AIANEVDSLAQDM------ 178

Tlp112_RM2100 AISENVNQIANDI------ 150

Tlp109_RM2100 AISENVNKIAND------- 149

Tlp110_RM2100 AISENVNKIAND------- 149

Tlp111_RM2100 AISENVNKIANDI------ 150

Tlp123_RM2100 AISENVNKIAND------- 149

TLp115_NCTC11845 TISDNVNKIANDILEDAR- 180

Tlp116_NCTC11845 TISDNVNKIANDILEDAR- 180

Tlp117_NCTC11845 TISDNVNKIANDILEDAR- 180

Tlp118_NCTC11845 TISDNVNKIANDILEDAR- 180

Tlp119_NCTC11845 TISDNVNKIANDILEDAR- 180

Tlp101_RM16712 AISDNVNKIANDILEDAR- 180

Tlp103_RM16712 AISDNVNKIANDILEDAR- 180

Tlp104_RM16712 AISDNVNKIANDILEDAR- 180

Tlp106_RM16712 AISDNVNKIANDILEDAR- 180

Tlp102_SlaughterBeach AISDNVNKIANDILEDAR- 180

Tlp105_SlaughterBeach AISDNVNKIANDILEDAR- 180

Tlp121_CCUG22395 AISDNVNKIANDILEDAR- 180

Tlp120_CCUG22395 IVADNVNKIASDI------ 175

Tlp107_LMG11760 ------------------- 162

Tlp122_CCUG22395 IVADNVNQIANDILEDAR- 255

Tlp113_RM2100 IVADNVNKIASDI------ 150

Tlp100_SlaughterBeach IVADNVNKIASDILEDAR- 180

Tlp114_RM1607 IVADNVNKIANDILEDAR- 180

Tlp601_LMG24591 ------------------- 141

Tlp602_LMG24591 ------------------- 142

Tlp300_ATCC51209 IISNNVSDIANNILE---- 177

Tlp301_ATCC51209 IISNNVSDIANNILE---- 177

Tlp302_ATCC51209 IISNNVSDIANNILE---- 177

Tlp303_ATCC51209 IISNNVSDIANNILE---- 177

Tlp304_ATCC51209 IISNNVSDIANNILE---- 177

Tlp2_NCTC11168 IISSTVSDIANNILE---- 172

Tlp3_NCTC11168 IISSTVSDIANNILE---- 177

Tlp4_NCTC11168 IISSTVSDIANNILE---- 177

Tlp11_00-2425 IISSTVSDIANNILE---- 177

Tlp12_RM1221 IISSTVSDIANNILE---- 177

Tlp13_00-1597 IISSTVSDIANNILE---- 177

Tlp14_00-1597 IISSTVSDIANNILE---- 177

Tlp19_T1-21 IISSTVSDIANNILE---- 177

Tlp21_FDAARGOS_295 IISSTVSDIANNILE---- 177

Tlp22_HF5-4A-4 IISSTVSDIANNILE---- 177

Tlp23_ICDCCJ07001 IISSTVSDIANNILE---- 177

Tlp25_CG8421 IISSTVSDIANNILE---- 177

Tlp17_4031 IISSTVSDIANNILE---- 177

Tlp24_M1 IISSTVSDIANNILE---- 177

Tlp15_RM1875 IISSTVSDIANNILE---- 177

Tlp16_FB1 IISSTVSDIANNILE---- 177

Tlp20_CO2-160 IISSTVSDIANNILE---- 177

Tlp18_76339 IISNTVSDIANNILE---- 17
