# Supplementary material for: Diversity of transducer-like proteins (Tlps) in Campylobacter
Source: PLoS One. 2019 Mar 25;14(3):e0214228. doi: 10.1371/journal.pone.0214228 (PMC6433261; doi:10.1371/journal.pone.0214228)
Supplement: S2 Archive — (ZIP) [file pone.0214228.s016.zip › Alignment E.docx]

Alignment E. Tlp1 vs Tlps 2, 3, and 4

CLUSTAL O(1.2.4) multiple sequence alignment 2018/04/27

NCTC11168_Tlp1 ----------MFKSLNIGLKLIFSVAAVVVIGLVILISLIT-KQVSQNITKNTEDILASI 49

NCTC11168_Tlp4 ---MQSINSGKSVGISAKLTLWVGILVVLILAITSAISYFDSRNNTYELLKDT------- 50

NCTC11168_Tlp2 -----------MKSVKLKVSLIANLIAVVCLIILGVVTFIFVKQAIFHEVVNA------- 42

NCTC11168_Tlp3 MLKITKIKRKIMNSIKIKLSLIANLIAIFALIVLGIVSFYFTKTSLYESTLKN------- 53

.:. :.* .: .:. : : :: : . .

NCTC11168_Tlp1 TKEYATQTQGIFGEMIALNKSISGTLTEMFRSTSKEDLDIDNITNIITNTFDNSAYSNFT 109

NCTC11168_Tlp4 QLKTMQDVDAFFKSYAMSKRNGIQIL-----------------ANELTNRPDM---SDEE 90

NCTC11168_Tlp2 EINYVKTAKNSIESFKARNSLALESL-----------------AKSILKHPIEQLDSQDA 85

NCTC11168_Tlp3 QTDLLKVTQSTVEDFRSTNQSFTRAL-----------------EKDIANLPYQSLITEEN 96

. .. . . : * : : : ::

NCTC11168_Tlp1 YLYLID-PPEYFKEESKFFNTQSGKFVMLYADEEKDNKGGIKAI-------QASDEIANL 161

NCTC11168_Tlp4 LINL----IKVIKKV--------NDYDLVYVGFDN---TGKNY--------QSDDQILDL 127

NCTC11168_Tlp2 LMHYVGKDLKNFRDA--------GRFLAVYIAQPN---GELVVSDPDSDAKNLDFGTYGK 134

NCTC11168_Tlp3 IINNVGPILKYYRHS--------INALNVYLGLNN---GKVLLSQKSNDAKMPE-----L 140

: : :. :* : .

NCTC11168_Tlp1 QVVQDIL----KKAKYGENKVYIGRPIKMNLEGQDFDAVNVAIPIFDRKNQVVGVIGMTL 217

NCTC11168_Tlp4 SKGYDTKNRPWYKAAKEAKKLIVTEPYKSAAS--GEVGLTYAAPFYDRNGNFRGVVGGDY 185

NCTC11168_Tlp2 ADNYDARTREYYIEAVKTNKLYITPSYIDVTT--NLPCFTYSIPLYK-DGKFIGVLAVDI 191

NCTC11168_Tlp3 RDDLDIKTKDWYQEALKTNDIFVTPAYLDTVL--KQYVITYSKAIYK-DGKIIGVLGVDI 197

* :.: : .. : ::. ..:. **:.

NCTC11168_Tlp1 DFSDIATYLLDPKGQKYDGELRVLLNSDGFMAIHPNKNLVL--KNLKDINPNKGAQETY- 274

NCTC11168_Tlp4 DLANFSTNVLTVG--KSDNTFTEVLDSEGTILFNDEVAKILTKTELSI-NIANAIKANPA 242

NCTC11168_Tlp2 LAADLQAEFENL---------------PGRTFVFDEENKVFVSTDKALLQKGYDISAIAN 236

NCTC11168_Tlp3 PSEDLQNLVAKT---------------PGNTFLFDQKNKIFAATNKELLNPSIDHSPVLN 242

:: . * . : :: .: : .

NCTC11168_Tlp1 KAISEGKNGVFNYIASD-GDDSYAAINSFKVQDSSWAVLVTAPKYSVFKPLKKLQLIILG 333

NCTC11168_Tlp4 LIDPRNQDTLFTAK-DHQGVDYAIMC--NSAFNPLFRICTITENKVYTEAVNSILMKQVI 299

NCTC11168_Tlp2 LAKTKEDLEPFEYTRPKDGNERFAVC--TKVS-GIYTACVGEPIEQIEAPVYKIAFIQTA 293

NCTC11168_Tlp3 AYKLNGDNNFFSYK--LNNEERLGAC--TKVF--AYTACITESADIINKPIYKAAFIQAI 296

. . * . : .. : : . :

NCTC11168_Tlp1 ASFIFIFVVLGVVYYCVRKIVASRLPVILSSLESFFRFLNHEKIEPKAIEIRANDELGAM 393

NCTC11168_Tlp4 VGIIAIIIALILIRFLI-SRSLSPLAAIQTGLTSFFDFINYKTKNVSTIEVKSNDEFGQI 358

NCTC11168_Tlp2 IVIFTSIISVILLYFIV-SKYLSPLAAIQTGLTSFFDFINYKTKNVSTIEVKSNDEFGQI 352

NCTC11168_Tlp3 VVIIVVVFSVILLYFIV-SKYLSPLAAIQTGLTSFFDFINYKTKNVSTIEVKSNDEFGQI 355

:: .. : :: : : . * * .* :.* *** *:*::. : .:**:::***:* :

NCTC11168_Tlp1 GRIINENIEKIQISLEQDQNAVDESVQTAREIEKGNLTARITKNPINPQLVELKDVLNRM 453

NCTC11168_Tlp4 SNAINENILATKRGLEQDNQAVKESVQTVSVVEGGNLTARITANPRNPQLIELKNVLNKL 418

NCTC11168_Tlp2 SNAINENILATKRGLEQDNQAVKESVQTVSVVEGGNLTARITANPRNPQLIELKNVLNKL 412

NCTC11168_Tlp3 SNAINENILATKRGLEQDNQAVKESVQTVSVVEGGNLTARITANPRNPQLIELKNVLNKL 415

.. ***** : .****::**.*****. :* ******** ** ****:***:***::

NCTC11168_Tlp1 LDVLQSKIGSNMNEINRVFDSYKALDFSTEVFNAKGEVEITTNILGKEIKKMLLASSNFA 513

NCTC11168_Tlp4 LDVLQARVGSDMNAIHKIFEEYKSLDFRNKLENASGSVELTTNALGDEIVKMLKQSSDFA 478

NCTC11168_Tlp2 LDVLQARVGSDMNAIHKIFEEYKSLDFRNKLENASGSVELTTNALGDEIVKMLKQSSDFA 472

NCTC11168_Tlp3 LDVLQARVGSDMNAIHKIFEEYKSLDFRNKLENASGSVELTTNALGDEIVKMLKQSSDFA 475

*****:::**:** *:::*:.**:*** .:: **.*.**:*** **.** *** **:**

NCTC11168_Tlp1 KDLANQSEELKNSMQKLADGSNAQASSLEQSAAAVEEINSSMQNVSGKTVEVASQADDIK 573

NCTC11168_Tlp4 NALANESGKLQTAVQSLTTSSNSQAQSLEETAAALEEITSSMQNVSVKTSDVITQSEEIK 538

NCTC11168_Tlp2 NALANESGKLQTAVQSLTTSSNSQAQSLEETAAALEEITSSMQNVSVKTSDVITQSEEIK 532

NCTC11168_Tlp3 NALANESGKLQTAVQSLTTSSNSQAQSLEETAAALEEITSSMQNVSVKTSDVITQSEEIK 535

: ***:* :*:.::*.*: .**:**.***::***:***.******* ** :* :*:::**

NCTC11168_Tlp1 NIVNVIKDIAEQTNLLALNAAIEAARAGEHGRGFAVVADEVRQLAERTGKSLSEIEANIN 633

NCTC11168_Tlp4 NVTGIIGDIADQINLLALNAAIEAARAGEHGRGFAVVADEVRKLAERTQKSLSEIEANTN 598

NCTC11168_Tlp2 NVTGIIGDIADQINLLALNAAIEAARAGEHGRGFAVVADEVRKLAERTQKSLSEIEANTN 592

NCTC11168_Tlp3 NVTGIIGDIADQINLLALNAAIEAARAGEHGRGFAVVADEVRKLAERTQKSLSEIEANTN 595

*:..:* ***:* *****************************:***** ********* *

NCTC11168_Tlp1 ILVQSVNEVAESVKEQTAGITQINDAIAQLETVTKENVEVANVTNNITNEVNQIAAAILE 693

NCTC11168_Tlp4 LLVQSINDMAESIKEQTAGITQINDSVAQIDQTTKDNVEIANESAIISSTVSDIANNILE 658

NCTC11168_Tlp2 LLVQSINDMAESIKEQTAGITQINDSVAQIDQTTKDNVEIANESAIISSTVSDIANNILE 652

NCTC11168_Tlp3 LLVQSINDMAESIKEQTAGITQINDSVAQIDQTTKDNVEIANESAIISSTVSDIANNILE 655

:****:*::***:************::**:: .**:***:** : *:. *.:** ***

NCTC11168_Tlp1 DVNKKRF 700

NCTC11168_Tlp4 DVKKKRF 665

NCTC11168_Tlp2 DVKKKRF 659

NCTC11168_Tlp3 DVKKKRF 662

**:****
