# Supplementary material for: Diversity of transducer-like proteins (Tlps) in Campylobacter
Source: PLoS One. 2019 Mar 25;14(3):e0214228. doi: 10.1371/journal.pone.0214228 (PMC6433261; doi:10.1371/journal.pone.0214228)
Supplement: S2 Archive — (ZIP) [file pone.0214228.s016.zip › Alignment F.docx]

Alignment F. Tlp proteins comparisons (except Tlp1)

Isolates with deletions in the C-terminal part of the protein were removed

CLUSTAL O(1.2.4) multiple sequence alignment

15-537360_Tlp20 ----------------MKSVKIKVSLIANLIAIVCLIF--LGIITFIFVKDEVFNQVVKS 42

76339_Tlp20 ----------------MKSVKIKVSLIANLIAIVCLIF--LGIITFIFVKDEVFNQVVKA 42

CFSAN032805_Tlp20 ----------------VKSVKIKVSLIANLIAIVCLIF--LGIITFIFVKDEVFNQVVKS 42

CVM_N29710_Tlp20 ----------------VKSVKIKVSLIANLIAIVCLI---LGIITFIFVKDEVFNQVVKS 41

YH501_Tlp20 -------------VKSVKSVKIKVSLIANLIAIVCLIL--LGIITFIFVKDEVFNQVVKS 45

CO2-160_Tlp20 ----------------MKSVKIKVSLIANLIAIVCLIF--LGIITFIFVKDEVFNQVVKS 42

CO2-160_Tlp20b ----------------MKSVKIKVSLIANLIAIVCLIF--LGIITFIFVKDEVFNQVVKS 42

RM5611_Tlp20 ----------------MKSVKIKVSLIANLIAIVCLIF--LGIITFIFVKDEVFNQVVKS 42

14903A_Tlp20 -------------VKSVKSVKIKVSLIANLIAIVCLIF--LGIITFIFVKDEVFNQVVKS 45

YH502_Tlp20 ----------------VKSVKIKVSLIANLIAIVCLIF--LGIITFIFVKDEVFNQVVKS 42

RM3196_Tlp23 ----------------MKSVKLKVTLIANLITVVCLVI--LGVITFMFVKQAIFHEVVNA 42

ICDCCJ07001_Tlp23 ----------------MKSVKLKVTLIANLITVVCLVI--LGVITFMFVKQAIFHEVVNA 42

RM1285_Tlp2 ----------------MKSVKLKVSLIANLIAVVCLII--LGVVTFIFVKQAIFHEVVNA 42

CFSAN032806_Tlp2 ----------------MKSVKLKVSLIANLIAVVCLII--LGVVTFIFVKQAIFHEVVNA 42

RM1221_Tlp2 ----------------MKSVKLKVSLIANLIAVVCLII--LGVVTFIFVKQAIFHEVVNA 42

S3_Tlp2 ----------------MKSVKLKVSLIANLIAVVCLII--LGVVTFIFVKQAIFHEVVNA 42

FDAARGOS_422_Tlp2 ----------------MKSVKLKVSLIANLIAVVCLII--LGVVTFMFVKQAIFHEVVKA 42

81-176_Tlp2 ----------------MKSVKLKVSLIANLIAVVCLII--LGVVTFIFVKQAIFHEVVNA 42

F38011_Tlp2 ----------------MKSVKLKVSLIANLIAVVCLII--LGVVTFIFVKQAIFHEVVNA 42

NCTC11168_Tlp2 ----------------MKSVKLKVSLIANLIAVVCLII--LGVVTFIFVKQAIFHEVVNA 42

MTVDSCj07_Tlp2 ----------------MKSVKLKVSLIANLIAVVCLII--LGVVTFIFVKQAIFHEVVNA 42

CJM1cam_Tlp24 ----------------MKSVKLKVALIANLIAVVCLVI--LGVITFMFVKQAIFHEVVKA 42

M1_Tlp24 ----------------MKSVKLKVALIANLIAVVCLVI--LGVITFMFVKQAIFHEVVKA 42

81116_Tlp2 ----------------MKSVKLKVALIANLIAVVCLVI--LGVITFMFVKQAIFHEVVKA 42

4031_Tlp23 ----------------MKSVKLKVALIANLIAVVCLVI--LGVITFMFVKQAIFHEVVKA 42

MG1116_Tlp14 ----------------MNNIKIKLSVIANSIAIFALSI--LSIISFYFTKDSLYQSTLHA 42

YH502_Tlp14 -----MLKITKIKRKNMNNIKIKLSVIANSIAIFALSI--LSIISFYFTKDSLYQSTLHA 53

BP3181_Tlp14 ----------------MNNIKIKLSVIANSIAIFALSI--LSIISFYFTKDSLYQSTLYT 42

ZV1224_Tlp14a ----------------MNNIKIKLSVIANSIAIFALSI--LSIISFYFTKDSLYQSTLHT 42

ZV1224_Tlp14b ----------------MNNIKIKLSVIANSIAIFALSI--LSIISFYFTKDSLYQSTLHT 42

YH503_Tlp14 -----MLKITKIKRKNMNNIKIKLSVIANSIAIFALSI--LSIISFYFTKDSLYQSTLHA 53

14903A_Tlp14 ----------------MNNIKIKLSVIANSIAIFALSI--LSIISFYFTKDSLYQSTLHA 42

OR12_Tlp14 ----------------MNNIKIKLSVIANSIAIFALSI--LSIISFYFTKDSLYQSTLHA 42

CFSAN032805_Tlp14 -----MLKITKIKRKNMNNIKIKLSVIANSIAIFALSI--LSIISFYFTKDSLYQSTLHA 53

BFR-CA-9557_Tlp14 ----------------MNNIKIKLSVIANSIAIFALSI--LSIISFYFTKDSLYQSTLHA 42

15-537360_Tlp14 ----------------MNNIKIKLSVIANSIAIFALSI--LSIISFYFTKDSLYQSTLHA 42

YH501_Tlp14 ----------------MNNIKIKLSVIANSIAIFALSI--LSIISFYFTKDSLYQSTLHA 42

CG8421_Tlp14 ----------------MNNIKIKLSVIANSIAIFALSI--LSIISFYFTKDSLYQSTLYT 42

MTVDSCj16_Tlp14 ----------------MNNIKIKLSVIANSIAIFALSI--LSIISFYFTKDSLYQSTLYT 42

01-1512_Tlp14 ----------------MNNIKIKLSVIANSIAIFALSI--LSIISFYFTKDSLYQSTLYT 42

00-0949_Tlp14 ----------------MNNIKIKLSVIANSIAIFALSI--LSIISFYFTKDSLYQSTLYT 42

MTVDSCj13_Tlp14 ----------------MNNIKIKLSVIANSIAIFALSI--LSIISFYFTKDSLYQSTLYT 42

S3_Tlp14 ----------------MNNIKIKLSVIANSIAIFALSI--LSIISFYFTKDSLYQSTLYT 42

PT14_Tlp14 ----------------MNNIKIKLSVIANSIAIFALSI--LSIISFYFTKDSLYQSTLYT 42

14980A_Tlp14 MLKVLLQKLIKFKRKNMNNIKIKLSVIANSIAIFALSI--LSIISFYFTKDSLYQSTLYT 58

FJ3124_Tlp14 ----------------MNNIKIKLSVIANSIAIFALSI--LSIISFYFTKDSLYQSTLYT 42

00-1597_Tlp14 ----------------MNNIKIKLSVIANSIAIFALSI--LSIISFYFTKDSLYQSTLYT 42

R14_Tlp14 ----------------MNNIKIKLSVIANSIAIFALSI--LSIISFYFTKDSLYQSTLYT 42

CG8421_Tlp25 ------------------------------------------------------------ 0

RM1875_Tlp3 ----------------MNSIKIKLSLIANLIAIFALIV--LGIVSFYFTKTSLYESTLKN 42

CJM1cam_Tlp3 ----------------MNSIKIKLSLIANLIAIFALIV--LGIVSFYFTKTSLHESALKN 42

M1_Tlp3 ----------------MNSIKIKLSLIANLIAIFALIV--LGIVSFYFTKTSLHESALKN 42

4031_Tlp3 ----------------MNSIKIKLSLIANLIAIFALIV--LGIVSFYFTKTSLHESALKN 42

R14_Tlp3 ----------------MNSIKIKLSLIANLIAIFALIV--LGIVSFYFTKTSLYESTLKN 42

RM5611_Tlp3 ----------------MNSIKIKLSLIANLIAIFALIV--LGIVSFYFTKTSLYESTLKN 42

MTVDSCj16_Tlp3 ----------------MNSIKIKLSLIANLIAIFALIV--LGIVSFYFTKTSLYESTLKN 42

01-1512_Tlp3 ----------------MNSIKIKLSLIANLIAIFALIV--LGIVSFYFTKTSLYESTLKN 42

MTVDSCj13_Tlp3 ----------------MNSIKIKLSLIANLIAIFALIV--LGIVSFYFTKTSLYESTLKN 42

32488_Tlp3a ----------------MNSIKIKLSLIANLIAIFALIV--LGIVSFYFTKTSLYESTLKN 42

81116_Tlp3 ----------------MNSIKIKLSLIANLIAIFALIV--LGIVSFYFTKTSLYESTLKN 42

32488_Tlp3b ----------------MNNIKIKLSVIANSIAIFALIV--LGIVSFYFTKTSLYESTLKN 42

FB1_Tlp3 ----------------MNSIKIKLSLIANLIAIFALIV--LGIVSFYFTKTSLYESTLKN 42

PT14_Tlp3 ----------------MNSIKIKLSLIANLIAIFALIV--LGIVSFYFTKTSLYESTLKN 42

00-6200_Tlp3a ----------------MNSIKIKLSLIANLIAIFALIV--LGIVSFYFTKTSLYESTLKN 42

RM1221_Tlp3 ----------------MNSIKIKLSLIANLIAIFALIV--LGIVSFYFTKTSLYESTLKN 42

S3_Tlp3 ----------------MNSIKIKLSLIANLIAIFALIV--LGIVSFYFTKTSLYESTLKN 42

FDAARGOS_421_Tlp3 -----MLKITKIKRKIMNSIKIKLSLIANLIAIFALIV--LGIVSFYFTKTSLYESTLKN 53

CFSAN032806_Tlp3 -----MLKITKIKRKIMNSIKIKLSLIANLIAIFALIV--LGIVSFYFTKTSLYESTLKN 53

IA3901_Tlp3b ----------------MNSIKIKLSLIANLIAIFALIV--LGIVSFYFTKTSLYESTLKN 42

00-6200_Tlp3b ----------------MNSIKIKLSLIANLIAIFALIV--LGIVSFYFTKTSLYESTLKN 42

BCW_6290_Tlp3b ----------------MNSIKIKLSLIANLIAIFALIV--LGIVSFYFTKTSLYESTLKN 42

00-2425_Tlp3a ----------------MNSIKIKLSLIANLIAIFALIV--LGIVSFYFTKTSLYESTLKN 42

00-2425_Tlp3b ----------------MNSIKIKLSLIANLIAIFALIV--LGIVSFYFTKTSLYESTLKN 42

YH001_Tlp3a ----------------MNSIKIKLSLIANLIAIFALIV--LGIVSFYFTKTSLYESTLKN 42

YH001_Tlp3b ----------------MNSIKIKLSLIANLIAIFALIV--LGIVSFYFTKTSLYESTLKN 42

00-0949_Tlp3 ----------------MNSIKIKLSLIANLIAIFALIV--LGIVSFYFTKTSLYESTLKN 42

NCTC11168_Tlp3 -----MLKITKIKRKIMNSIKIKLSLIANLIAIFALIV--LGIVSFYFTKTSLYESTLKN 53

F38011_Tlp3 ----------------MNSIKIKLSLIANLIAIFALIV--LGIVSFYFTKTSLYESTLKN 42

RM1285_Tlp3 ----------------MNSIKIKLSLIANLIAIFALIV--LGIVSFYFTKTSLYESTLKN 42

FDAARGOS_422_Tlp3 -----MLKITKIKRKIMNSIKIKLSLIANLIAIFALIV--LGIVSFYFTKTSLYESTLKN 53

MTVDSCj07_Tlp3 ----------------MNSIKIKLSLIANLIAIFALIV--LGIVSFYFTKTSLYESTLKN 42

IA3901_Tlp3a ----------------MNSIKIKLSLIANLIAIFALIV--LGIVSFYFTKTSLYESTLKN 42

BCW_6290_Tlp3a ----------------MNSIKIKLSLIANLIAIFALIV--LGIVSFYFTKTSLYESTLKN 42

35925B2_Tlp3 -----MLKITKIKRKIMNSIKIKLSLIANLIAIFALIV--LGIVSFYFTKTSLHESALKN 53

14980A_Tlp3 -----MLKITKIKRKIMNSIKIKLSLIANLIAIFALIV--LGIVSFYFTKTSLYESTLKN 53

00-1597_Tlp3b ----------------MNSIKIKLSLIANLIAIFALIV--LGIVSFYFTKTSLYESTLKN 42

RM3196_Tlp3 ----------------MNSIKIKLSLIANLIAIFALIV--LGIVSFYFTKTSLYESTLKN 42

FDAARGOS_295_Tlp21 ------------MKSS-ISTKLTILIGILIVLAFGISSM-----ISYLSSLNNSRSLLQN 42

FORC_046_Tlp4 -----MQSINSGKSVG-ISAKLTLWVGILVVLILAITSA-----ISYFDSRNNTYELLKD 49

FDAARGOS_422_Tlp4 -----MQSINSGKSVG-ISAKLTLWVGILVVLILAITSA-----ISYFDSRNNTYELLKD 49

ICDCCJ07001_Tlp4 -----MQSINSGKSVG-ISAKLTLWVGILVVLILAITSA-----ISYFDSRNNTYELLKD 49

RM3196_Tlp4 -----MQSINSGKSVG-ISAKLTLWVGILVVLILAITSA-----ISYFDSRNNTYELLKD 49

T1-21_Tlp4 -----MQSINSGKSVG-ISAKLTLWVGILVVLILAITSA-----ISYFDSRNNTYELLKD 49

F38011_Tlp4 -----MQSINSGKSAG-ISAKLTLWVGILVVLILAITSA-----VSYFDSRNNTYELLKD 49

HF5-4A-4_Tlp22 ------------------------------------------------------------ 0

00-0949_Tlp4 -----MQSINSGKSVG-ISAKLTLWVGILVVLILAITSA-----ISYFDSRNNTYELLKD 49

01-1512_Tlp4 -----MQSINSGKSVG-ISAKLTLWVGILVVLILAITSA-----ISYFDSRNNTYELLKD 49

81-176_Tlp4 -----MQSINSGKSVG-ISAKLTLWVGILVVLILAITSA-----ISYFDSRNNTYELLKD 49

32488_Tlp4 -----MQSINSGKSVG-ISAKLTLWVGILVVLILAITSA-----ISYFDSRNNTYELLKD 49

NCTC11168_Tlp4 -----MQSINSGKSVG-ISAKLTLWVGILVVLILAITSA-----ISYFDSRNNTYELLKD 49

CFSAN032806_Tlp4 -----MQSINSGKSVG-ISAKLTLWVGILVVLILAITSA-----ISYFDSRNNTYELLKD 49

81116_Tlp4 -----MQSINSGKSVG-ISAKLTLWVGILVVLILAITSA-----ISYFDSRNNTYELLKD 49

RM1285_Tlp12 -----MQSINSGKSVG-ISAKLTLWVGILVVLILAITST-----VSYFDAKNHTYELLKE 49

PT14_Tlp12 -----MQSINSGKSVG-ISAKLTLWVGILVVLILAITST-----VSYFDAKNHTYELLKE 49

MTVJDCj07_Tlp12 -----MQSINSGKSVG-ISAKLTLWVGILVVLILAITST-----VSYFDAKNHTYELLKE 49

RM1221_Tlp12 -----MQKMDSGKSVG-VSVKLTLWVGILVVLILAITST-----VSYFDAKNHTYELLKE 49

FDAARGOS_421_Tlp12 -----MQKMDSGKSVG-VSVKLTLWVGILVVLILAITST-----VSYFDAKNHTYELLKE 49

35925B2_Tlp12 -----MQKMNSGKSVG-ISAKLTLWVGILVVLILAITSA-----VSYFDAKNHTYELLKE 49

CJM1cam_Tlp12 -----MQKMNSGKSVG-ISAKLTLWVGILVVLILAITSA-----VSYFDAKNHTYELLKE 49

M1_Tlp12 -----MQKMNSGKSVG-ISAKLTLWVGILVVLILAITSA-----VSYFDAKNHTYELLKE 49

S3_Tlp12 -----MQKMDSGKSVG-VSVKLTLWVGILVVLILAITST-----VSYFDAKNHTYELLKE 49

00-1597_Tlp12 -----MQSINSGKSVG-VSVKLTLWVGILVVLILAITST-----VSYFDAKNHTYELLKE 49

R14_Tlp12 -----MQSINSGKSVG-ISAKLTLWVGILVVLILAITST-----VSYFDAKNHTYELLKE 49

RM1875_Tlp15 ------------MKLS-IRKKMLMLG------GICLVSMLITFGIFYYNNLQGSEKIAQI 41

YH503_Tlp16 ------------MQLS-IRKKMLMLG------AICFISMLATFAIFYYNNLKGSQKIAQT 41

FB1_Tlp16 ------------MQLS-IRKKMLMLG------AICFISMLATFAIFYYNNLKGSQKIAQT 41

BFR-CA-9557_Tlp16 ------------MQLS-IRKKMLMLG------AICFISMLATFAIFYYNNLKGSQKIAQT 41

15-537360_Tlp16 ------------MQLS-IRKKMLMLG------AICFISMLATFAIFYYNNLKGSQKIAQT 41

OR12_Tlp16 ------------MQLS-IRKKMLMLG------AICFISMLATFAIFYYNNLKGSQKIAQT 41

YH502_Tlp16 ------------MQLS-IRKKMLMLG------AICFISMLATFAIFYYNNLKGSQKIAQT 41

14903A_Tlp16 ------------MQLS-IRKKMLMLG------AICFISMLATFAIFYYNNLQGSQKIAQT 41

RM5611_Tlp16 ------------MQLS-IRKKMLMLG------AICFISMLATFAIFYYNNLQGSQKIAQT 41

00-2425_Tlp11 ---------MNFRSLN-LSTKLILSVAIGIVLGIVVIVLTVSIYTSKSMEKEAKDSIFLS 50

00-6200_Tlp11 ---------MNFRSLN-LSTKLILSVAIGIVLGIVVIVLTVSIYTSKSMEKEAKDSIFLS 50

YH001_Tlp11 ---------MNFRSLN-LSTKLILSVAIGIVLGIVVIVLTVSIYTSKSMEKEAKDSIFLS 50

IA3902_Tlp11 ---------MNFRSLN-LSTKLILSVAIGIVLGIVVIVLTVSIYTSKSMEKEAKDSIFLS 50

BCW_6290_Tlp11 ---------MNFRSLN-LSTKLILSVAIGIVLGIVVIVLTVSIYTSKSMEKEAKDSIFLS 50

76339_Tlp18 ----------MFKSLN-IGSKLVLSVAVSVIAAIAILITILSFEVASYAEKEAKDTIFLS 49

4031_Tlp17 ---------MNFRSLN-ISTKLILSVAIGVILGIIVLVSTVSIYISENMEKEAKDSIFLA 50

15-537360_Tlp13 ----------MFRLSS-VSSKLLLSVAISVILATALMIAIVSFQVASYSEKEARNTILLS 49

CVM_N29710_Tlp13 ----------MFRLSS-VSSKLLLSVAISVILATALMIAIVSFQVASYSEKEARNTILLS 49

FB1_Tlp13 ----------MFRLSS-VSSKLLLSVAISVILATALMIAIVSFQVASYSEKEARNTILLS 49

CFCAN032805_Tlp13 ----------MFRLSS-VSSKLLLSVAISVILATALMIAIVSFQVASYSEKEARNTILLS 49

BG2108_Tlp13 ----------MFRLSS-VSSKLLLSVAISVILATALMIAIVSFQVASYSEKEARNTILLS 49

YF2105_Tlp13 ----------MFRLSS-VSSKLLLSVAISVILATALMIAIVSFQVASYSEKEARNTILLS 49

YH503_Tlp13 ----------MFRLSS-VSSKLLLSVAISVILATALMIAIVSFQVASYSEKEARNTILLS 49

BFRCA9557_Tlp13 ----------MFRLSS-VSSKLLLSVAISVILATALMIAIVSFQVASYSEKEARNTILLS 49

YH502_Tlp13 ----------MFRLSS-VSSKLLLSVAISVILATALMIAIVSFQVASYSEKEARNTILLS 49

MTVDSCj13_Tlp13 ----------MFRLSS-VSSKLLLSVAISIIVAIALIIAIVSFQVASYSEKEARNTILLS 49

OR12_Tlp13 ----------MFRLSS-VSSKLLLSVAISVILAIALMIAIVSFQVASYSEKEAKDTILLS 49

00-1597_Tlp13 ----------MFRLSS-VSSKLLLSVAISVILATALMIAIVSFQVASYSEKEAKDTIFLS 49

14903A_Tlp13 ----------MFRLSS-VSSKLLLSVAISVILATALMIAIVSFQVASYSEKEAKDTIFLS 49

FJ3124_Tlp13 ----------MFRLSS-VSSKLLLSVAISVILATALMIAIVSFQVASYSEKEAKDTIFLS 49

R14_Tlp13 ----------MFRLSS-VSSKLLLSVAISVIVAIALMIAIVSFQVASYSEKEAKDTIFLS 49

MTVDSCj16_Tlp13 ----------MFRLSS-VSSKLLLSVAISVIVAIALMIAIVSFQVASYSEKEAKDTIFLS 49

14980A_Tlp13 ----------MFRLSS-VSSKLLLSVAISVIVATALMIAIVSFQVASYSEKEAKDTIFLS 49

15-537360_Tlp20 ESNYVRTAKNSMEAFKARNTAALESLAKNILKLPYEQISNQEALMRYVGKDLKVFRDAGG 102

76339_Tlp20 ESNYVRTAKNSMEAFKARNTAALESLAKNILKLPYEQISNQEALMRYVGKDLKVFRDAGG 102

CFSAN032805_Tlp20 ESNYVRTTKNSMEAFKARNTAALESLAKNILKLPYEQISNQEALMRYVGKDLKVFRDAGG 102

CVM_N29710_Tlp20 ESNYVRTTKNSMEAFKARNTAALESLAKNILKLPYEQISNQEALMRYVGKDLKVFRDAGG 101

YH501_Tlp20 ESNYVRTTKNSMEAFKARNTAALESLAKNILKLPYEQISNQEALMRYVGKDLKVFRDAGG 105

CO2-160_Tlp20 ESNYVRTAKNSMEAFKARNTAALESLAKNILKLPYEQISNQEALMRYVGKDLKVFRDAGG 102

CO2-160_Tlp20b ESNYVRTAKNSMEAFKARNTAALESLAKNILKLPYEQISNQEALMRYVGKDLKVFRDAGG 102

RM5611_Tlp20 ESNYVRTAKNSMEAFKARNTAALESLAKNILKLPYEQISNQEALMRYVGKDLKVFRDAGG 102

14903A_Tlp20 ESNYVRTAKNSMEAFKARNTAALESLAKNILKLPYEQISNQEALMRYVGKDLKVFRDAGG 105

YH502_Tlp20 ESNYVRTAKNSMEAFKARNTAALESLAKNILKLPYEQISNQEALMRYVGKDLKVFRDAGG 102

RM3196_Tlp23 EINYVKTAKNSIESFKARNSLALESLAKSILKHPVEQLDNQDALMHYVGKDLKNFRDAGR 102

ICDCCJ07001_Tlp23 EINYVKTAKNSIESFKARNSLALESLAKSILKHPVEQLDNQDALMHYVGKDLKNFRDAGR 102

RM1285_Tlp2 EINYVKTAKNSIESFKARNSLALESLAKSILKHPIEQLDSQDALMHYVGKDLKNFRDAGR 102

CFSAN032806_Tlp2 EINYVKTAKNSIESFKARNSLALESLAKSILKHPIEQLDSQDALMHYVGKDLKNFRDAGR 102

RM1221_Tlp2 EINYVKTAKNSIESFKARNSLALESLAKSILKHPIEQLDSQDALMHYVGKDLKNFRDAGR 102

S3_Tlp2 EINYVKTAKNSIESFKARNSLALESLAKSILKHPIEQLDSQDALMHYVGKDLKNFRDAGR 102

FDAARGOS_422_Tlp2 ETNYVKTAKNSMESFKARNSLALESLAKSILKHPVEQLDNQDALMHYVGKDLKNFRDAGR 102

81-176_Tlp2 EINYVKTAKNSIESFKARNSLALESLAKSILKHPIEQLDSQDALMHYVGKDLKNFRDAGR 102

F38011_Tlp2 EINYVKTAKNSIESFKARNSLALESLAKSILKHPIEQLDSQDALMHYVGKDLKNFRDAGR 102

NCTC11168_Tlp2 EINYVKTAKNSIESFKARNSLALESLAKSILKHPIEQLDSQDALMHYVGKDLKNFRDAGR 102

MTVDSCj07_Tlp2 EINYVKTAKNSIESFKARNSLALESLAKSILKHPIEQLDSQDALMHYVGKDLKNFRDAGR 102

CJM1cam_Tlp24 ETNYVKTAKNSMESFKARNSLALESLAKSILKHPVEQLDSQDALMRYVGKDLKNFRDAGR 102

M1_Tlp24 ETNYVKTAKNSMESFKARNSLALESLAKSILKHPVEQLDSQDALMRYVGKDLKNFRDAGR 102

81116_Tlp2 ETNYVKTAKNSMESFKARNSLALESLAKSILKHPVEQLDSQDALMRYVGKDLKNFRDAGR 102

4031_Tlp23 ETNYVKTAKNSMESFKARNSLALESLAKSILKHPVEQLDSQDALMRYVGKDLKNFRDAGR 102

MG1116_Tlp14 ETDLLKATQISIEDFRSRNISLLNTLEKDILNLPYEALNSQDNIINNAGAILKYYRNSGN 102

YH502_Tlp14 KTDLLKATQISIENFRSRNISLLNALEKDILNLPYEALNSQDNIVNNVGAILKYYRNSGN 113

BP3181_Tlp14 ETELLKATQISIEDFRSRNISLLNTLEKDILNLPYEALNSQDNIINNAGAILKYYRNSGN 102

ZV1224_Tlp14a ETELLKAAQISIEDFRSRNISLLNALEKDILNLPYEALNSQDNIINNVGAILKYYRNSGN 102

ZV1224_Tlp14b ETELLKAAQISIEDFRSRNISLLNALEKDILNLPYEALNSQDNIINNVGAILKYYRNSGN 102

YH503_Tlp14 KTELLKATQISIEDFRSRNISLLNTLEKDILNLPYEALNSQDNIINNAGAILKYYRNSGN 113

14903A_Tlp14 ETDLLKATQISIEDFRSRNISLLNTLEKDILNLPYEALNSQDNIVNNVGAILKYYRNSGN 102

OR12_Tlp14 ETDLLKATQISIEDFRSRNISLLNTLEKDILNLPYEALNSQDNIVNNVGAILKYYRNSGN 102

CFSAN032805_Tlp14 ETDLLKATQISIENFRSRNISLLNALEKDILNLPYEALNSQDNIVNNVGAILKYYRNSGN 113

BFR-CA-9557_Tlp14 ETDLLKATQISIENFRSRNISLLNALEKDILNLPYEALNSQDNIVNNVGAILKYYRNSGN 102

15-537360_Tlp14 ETDLLKATQISIENFRSRNISLLNALEKDILNLPYEALNSQDNIVNNVGAILKYYRNSGN 102

YH501_Tlp14 ETDLLKATQISIENFRSRNISLLNALEKDILNLPYEALNSQDNIVNNVGAILKYYRNSGN 102

CG8421_Tlp14 ETELLKATQISIEDFRSRNISLLNTLEKDILKLPYEALNSQDNIVNNVGAILKYYRNSGN 102

MTVDSCj16_Tlp14 ETELLKATQISIEDFRSRNISLLNTLEKDILKLPYEALNSQDNIVNNVGAILKYYRNSGN 102

01-1512_Tlp14 ETELLKATQISIEDFRSRNISLLNTLEKDILKLPYEALNSQDNIVNNVGAILKYYRNSGN 102

00-0949_Tlp14 ETELLKATQISIEDFRSRNISLLNTLEKDILKLPYEALNSQDNIVNNVGAILKYYRNSGN 102

MTVDSCj13_Tlp14 ETELLKATQISIEDFRSRNISLLNTLEKDILKLPYEALNSQDNIVNNAGAILKYYRNSGN 102

S3_Tlp14 ETELLKATQISIEDFRSRNISLLNTLEKDILKLPYEALNSQDNIVNNVGAILKYYRNSGN 102

PT14_Tlp14 ETELLKATQISIEDFRSRNISLLNTLEKDILKLPYEALNSQDNIVNNVGAILKYYRNSGN 102

14980A_Tlp14 ETELLKATQISIEDFRSRNISLLNTLEKDILKLPYEALNSQDNIVNNVGAILKYYRNSGN 118

FJ3124_Tlp14 ETELLKATQISIEDFRSRNISLLNTLEKDILKLPYEALNSQDNIVNNVGAILKYYRNSGN 102

00-1597_Tlp14 ETELLKATQISIEDFRSRNISLLNTLEKDILKLPYEALNSQDNIVNNVGAILKYYRNSGN 102

R14_Tlp14 ETELLKATQISIEDFRSRNISLLNTLEKDILKLPYEALNSQDNIVNNVGAILKYYRNSGN 102

CG8421_Tlp25 ------------------------------------------------------------ 0

RM1875_Tlp3 QTDLLKVTQSTVEDFRSTNQSFIRALEKDIANLPYQSLITEENIINNVGPILKYYRHSIN 102

CJM1cam_Tlp3 QTDLLKVTQSTVEDFRSTNQSFTRALEKDITNLPYQSLITEENIINNVGPILKYYRHSIN 102

M1_Tlp3 QTDLLKVTQSTVEDFRSTNQSFTRALEKDITNLPYQSLITEENIINNVGPILKYYRHSIN 102

4031_Tlp3 QTDLLKVTQSTVEDFRSTNQSFTRALEKDITNLPYQSLITEENIINNVGPILKYYRHSIN 102

R14_Tlp3 QTDLLKVTQSTVEDFRSTNQSFTRALEKDIANLPYQSLITEENIINNVGPILKYYRHSIN 102

RM5611_Tlp3 QTDLLKVTQSTVEDFRSTNQSFTRALEKDIANLPYQSLITEENIINNVGPILKYYHHSIN 102

MTVDSCj16_Tlp3 QTDLLKVTQSTVEDFRSTNQSFTRALEKDIANLPYQSLITEENIINNVGPILKYYRHSIN 102

01-1512_Tlp3 QTDLLKVTQSTVEDFRSTNQSFTRALEKDIANLPYQSLITEENIINNVGPILKYYRHSIN 102

MTVDSCj13_Tlp3 QTDLLKVTQSTVEDFRSTNQSFTRALEKDIANLPYQSLITEENIINNVGPILKYYRHSIN 102

32488_Tlp3a QTDLLKVTQSTVEDFRSTNQSFTRALEKDIANLPYQSLITEENIINNVGPILKYYRHSIN 102

81116_Tlp3 QTDLLKVTQSTVEDFRSTNQSFTRALEKDIANLPYQSLITEENIINNVGPILKYYRHSIN 102

32488_Tlp3b QTDLLKVTQSTVEDFRSTNQSFTRALEKDIANLPYQSLITEENIINNVGPILKYYRHSIN 102

FB1_Tlp3 QTDLLKVTQSTVEDFRSTNQSFTRALEKDIANLPYQSLITEENIINNVGPILKYYRHSIN 102

PT14_Tlp3 QTDLLKVTQSTVEDFRSTNQSFTRALEKDIANLPYQSLITEENIINNVGPILKYYRHSIN 102

00-6200_Tlp3a QTDLLKVTQSTVEDFRSTNQSFTRALEKDIANLPYQSLITEENIINNVGPILKYYHHSIN 102

RM1221_Tlp3 QTDLLKVTQSTVEDFRSTNQSFTRALEKDIANLPYQSLITEENIINNVGPILKYYRHSIN 102

S3_Tlp3 QTDLLKVTQSTVEDFRSTNQSFTRALEKDIANLPYQSLITEENIINNVGPILKYYRHSIN 102

FDAARGOS_421_Tlp3 QTDLLKVTQSTVEDFRSTNQSFTRALEKDIANLPYQSLITEENIINNVGPILKYYRHSIN 113

CFSAN032806_Tlp3 QTDLLKVTQSTVEDFRSTNQSFTRALEKDIANLPYQSLITEENIINNVGPILKYYRHSIN 113

IA3901_Tlp3b QTDLLKVTQSTVEDFRSTNQSFTRALEKDIANLPYQSLITEENIINNVGPILKYYHHSIN 102

00-6200_Tlp3b QTDLLKVTQSTVEDFRSTNQSFTRALEKDIANLPYQSLITEENIINNVGPILKYYHHSIN 102

BCW_6290_Tlp3b QTDLLKVTQSTVEDFRSTNQSFTRALEKDIANLPYQSLITEENIINNVGPILKYYHHSIN 102

00-2425_Tlp3a QTDLLKVTQSTVEDFRSTNQSFTRALEKDIANLPYQSLITEENIINNVGPILKYYHHSIN 102

00-2425_Tlp3b QTDLLKVTQSTVEDFRSTNQSFTRALEKDIANLPYQSLITEENIINNVGPILKYYHHSIN 102

YH001_Tlp3a QTDLLKVTQSTVEDFRSTNQSFTRALEKDIANLPYQSLITEENIINNVGPILKYYHHSIN 102

YH001_Tlp3b QTDLLKVTQSTVEDFRSTNQSFTRALEKDIANLPYQSLITEENIINNVGPILKYYHHSIN 102

00-0949_Tlp3 QTDLLKVTQSTVEDFRSTNQSFTRALEKDIANLPYQSLITEENIINNVGPILKYYRHSIN 102

NCTC11168_Tlp3 QTDLLKVTQSTVEDFRSTNQSFTRALEKDIANLPYQSLITEENIINNVGPILKYYRHSIN 113

F38011_Tlp3 QTDLLKVTQSTVEDFRSTNQSFTRALEKDIANLPYQSLITEENIINNVGPILKYYRHSIN 102

RM1285_Tlp3 QTDLLKVTQSTVEDFRSTNQSFTRALEKDIANLPYQSLITEENIINNVGPILKYYRHSIN 102

FDAARGOS_422_Tlp3 QTDLLKVTQSTVEDFRSTNQSFTRALEKDIANLPYQSLITEENIINNVGPILKYYRHSIN 113

MTVDSCj07_Tlp3 QTDLLKVTQSTVEDFRSTNQSFTRALEKDIANLPYQSLITEENIINNVGPILKYYRHSIN 102

IA3901_Tlp3a QTDLLKVTQSTVEDFRSTNQSFTRALEKDIANLPYQSLITEENIINNVGPILKYYHHSIN 102

BCW_6290_Tlp3a QTDLLKVTQSTVEDFRSTNQSFTRALEKDIANLPYQSLITEENIINNVGPILKYYHHSIN 102

35925B2_Tlp3 QTDLLKVTQSTVEDFRSTNQSFTRALEKDITNLPYQSLITEENIINNVGPILKYYRHSIN 113

14980A_Tlp3 QTDLLKVTQSTVEDFRSTNQSFTRALEKDIANLPYQSLITEENIINNVGPILEYYRHSIN 113

00-1597_Tlp3b QTDLLKVTQSTVEDFRSTNQSFTRALEKDIANLPYQSLITEENIINNVGPILKYYRHSIN 102

RM3196_Tlp3 QTDLLKVTQSTVEDFRSTNQSFTRALEKDIANLPYQSLITEENIINNVGPILKYYRHSIN 102

FDAARGOS_295_Tlp21 N--QMTVLKNTATAFENANANKELTMQALAKD-LAKNLN-NEKDIYTILADFKNLT---L 95

FORC_046_Tlp4 T--QLKTMQDVDAFFKSYAMSKRNGIQILANE-LTNRPDMSDEELINLIKVIKKVN---D 103

FDAARGOS_422_Tlp4 T--QLKTMQDVDAFFKSYAMSKRNGIQILANE-LTNRPDMSDEELINLIKVIKKVN---D 103

ICDCCJ07001_Tlp4 T--QLKTMQDVGAFFESYGMSKRNGIQILANE-LNKRPDMSDEELINLIKAFKEVN---G 103

RM3196_Tlp4 T--QLKTMQDVGAFFESYGMSKRNGIQILANE-LNKRPDMSDEELINLIKAFKEVN---G 103

T1-21_Tlp4 T--QLKTMQDVGAFFESYGMSKRNGIQILANE-LNKRPDMSDEELINLIKAFKEVN---G 103

F38011_Tlp4 T--QLKTMQDVGAFFESYGMSKRHGIQILANE-LNKRPDMSDEELINLIKAFKEVN---D 103

HF5-4A-4_Tlp22 -------MTLICKAVLYYAMSKRNGIQILANE-LTNRPDMSDEELINLIKVIKKVN---D 49

00-0949_Tlp4 T--QLKTMQDVDAFFKSYAMSKRNGIQILANE-LTNRPDMSDEELINLIKVIKKVN---D 103

01-1512_Tlp4 T--QLKTMQDVDAFFKSYAMSKRNGIQILANE-LTNRPDMSDEELINLIKVIKKVN---D 103

81-176_Tlp4 T--QLKTMQDVDAFFKSYAMSKRNGIQILANE-LTNRPDMSDEELINLIKVIKKVN---D 103

32488_Tlp4 T--QLKTMQDVDAFFKSYAMSKRNGIQILANE-LTNRPDMSDEELINLIKVIKKVN---D 103

NCTC11168_Tlp4 T--QLKTMQDVDAFFKSYAMSKRNGIQILANE-LTNRPDMSDEELINLIKVIKKVN---D 103

CFSAN032806_Tlp4 T--QLKTMQDVDAFFKSYAMSKRNGIQILANE-LTNRPDMSDEELINLIKVIKKVN---D 103

81116_Tlp4 T--QLKTMQDVDAFFKSYAMSKRNGIQILANE-LTNRPDMSDEELINLIKVIKKVN---D 103

RM1285_Tlp12 N--QLKTMDDVKVTFENYSKSKQKAIEVLAYE-SAK--KLEDENISLLLDSFKKAF---D 101

PT14_Tlp12 N--QLKTMDDVKVTFENYSKSKQKAIEVLAYE-SAK--KLEDENISLLLDSFKKAF---D 101

MTVJDCj07_Tlp12 N--QLKTMDDVKVTFENYSKSKQKAIEVLAYE-SAK--KLEDENISLLLDSFKKAF---D 101

RM1221_Tlp12 N--QLKTMDDVKVTFENYSKSKQKAIEVLAYE-SAK--KLEDENISLLLDSFKKAF---D 101

FDAARGOS_421_Tlp12 N--QLKTMDDVKVTFENYSKSKQKAIEVLAYE-SAK--KLEDENISLLLDSFKKAF---D 101

35925B2_Tlp12 N--QLKTMNDVKVTFENYSKSKQKAIEVLAYE-SAK--KLEDENISLLLDSFKKAF---D 101

CJM1cam_Tlp12 N--QLKTMDDVKVTFENYSKSKQKAIEVLAYE-SAK--KLEDENISLLLDSFKKAF---D 101

M1_Tlp12 N--QLKTMDDVKVTFENYSKSKQKAIEVLAYE-SAK--KLEDENISLLLDSFKKAF---D 101

S3_Tlp12 N--QLKTMDDVKVTFENYSKSKQKAIEVLAYE-SAK--KLEDENISLLLDSFKKAF---D 101

00-1597_Tlp12 N--QLKTMDDVKVTFENYSKSKQKAIEVLAYE-SAK--KLEDENISLLLDSFKKAF---D 101

R14_Tlp12 N--QLKTMDDVKVTFENYSKSKQKAIEVLAYE-SAK--KLEDENISLLLDSFKKAF---D 101

RM1875_Tlp15 TKNLINKE--IN----VKVELLTKSMAIALGD-LIKNVHSEEEKVKIIATAIENFRFEED 94

YH503_Tlp16 TKNLINKE--ID----IKVELLTKSMAIALGD-LIKDVDDEKEKIKISLPQLKILDLKRI 94

FB1_Tlp16 TKNLINKE--ID----IKVELLTKSMAIALGD-LIKDVDDEKEKIKISLPQLKILDLKRI 94

BFR-CA-9557_Tlp16 TKNLINKE--ID----IKVELLTKSMAIALGD-LIKDVDDEKEKIKISLPQLKILDLKR- 93

15-537360_Tlp16 TKNLINKE--ID----IKVELLTKSMAIALGD-LIKDVDDEKEKIKISLPQLKILDLKRI 94

OR12_Tlp16 TKNLINKE--ID----IKVELLTKSMAIALGD-LIKDVDDEKEKIKISLPQLKILDLKRI 94

YH502_Tlp16 TKNLINKE--ID----IKVELLTKSMAIALGD-LIKDVDDEKEKIKISLPQLKILDLKRI 94

14903A_Tlp16 TKNLINKE--ID----IKVELLTKSMAIALGD-LIKDVDDEKEKIKIIATAIENFRFEED 94

RM5611_Tlp16 TKNLINKE--IN----IKVELLTKSMAIALGD-LIKNVHSEEEKVKIIATAIENFRFEED 94

00-2425_Tlp11 SKRYVNYMEGIL----NEEVVLTKAMATSLNE-IFSKNDQVN--AGIIESLLRNTFDSSG 103

00-6200_Tlp11 SKRYVNYMEGIL----NEEVVLTKAMATSLNE-IFSKNDQVN--AGIIESLLRNTFDSSG 103

YH001_Tlp11 SKRYVNYMEGIL----NEEVVLTKAMATSLNE-IFSKNDQVN--AGIIESLLRNTFDSSG 103

IA3902_Tlp11 SKRYVNYMEGIL----NEEVVLTKAMATSLNE-IFSKNDQVN--AGIIESLLRNTFDSSG 103

BCW_6290_Tlp11 SKRYVNYMEGIL----NEEVVLTKAMATSLNE-IFSKNDQVN--AGIIESLLRNTFDSSG 103

76339_Tlp18 SKRYANYMEGVL----NESVVLTKGISASINE-MFSKHDQVG--ADLIESLLKNTFDSSG 102

4031_Tlp17 SKRYTNYMEGIL----NETVALTKGTATSLND-MFEHNNQVD--ADLIESLMKNLFDSSL 103

15-537360_Tlp13 SKRYVNYIQGML----NEEVTLTKGVATSLNE-MFQNNDHID--IDLIESLIKNTFDSSH 102

CVM_N29710_Tlp13 SKRYVNYIQGML----NEEVTLTKGVATSLNE-MFQNNDHID--IDLIESLIKNTFDSSH 102

FB1_Tlp13 SKRYVNYIQGML----NEEVTLTKGVATSLNE-MFQNNDHID--IDLIESLIKNTFDSSH 102

CFCAN032805_Tlp13 SKRYVNYIQGML----NEEVTLTKGVATSLNE-MFQNNDHID--IDLIESLIKNTFDSSH 102

BG2108_Tlp13 SKRYVNYIQGML----NEEVTLTKGVATSLNE-MFQNNDHID--IDLIESLIKNTFDSSH 102

YF2105_Tlp13 SKRYVNYIQGML----NEEVTLTKGVATSLNE-MFQNNDHID--IDLIESLIKNTFDSSH 102

YH503_Tlp13 SKRYVNYIQGML----NEEVTLTKGVATSLNE-MFQNNDHID--IDLIESLIKNTFDSSH 102

BFRCA9557_Tlp13 SKRYVNYIQGML----NEEVTLTKGVATSLNE-MFQNNDHID--IDLIESLIKNTFDSSH 102

YH502_Tlp13 SKRYVNYIQGML----NEEVTLTKGVATSLNE-MFQNNDHID--IDLIESLIKNTFDSSH 102

MTVDSCj13_Tlp13 SKRYVNYIQGIL----NEEVTLTKVVATSLNE-MFQNNDHVD--INLIESLIKNAFDSSH 102

OR12_Tlp13 SKRYVNYIQGML----NEEVTLTKGVATSLNE-MFQNNDHID--IDLIESLIKNTFDSSH 102

00-1597_Tlp13 SKRYVNYIQGML----NEEVTLTKGVATSLNE-MFQNNDHID--IDLIESLIKNTFDSSH 102

14903A_Tlp13 SKRYVNYIQGML----NEEVTLTKGVATSLNE-MFQNNDHID--IDLIESLIKNTFDSSH 102

FJ3124_Tlp13 SKRYVNYIQGML----NEEVTLTKGVATSLNE-MFQNNDHID--IDLIESLIKNTFDSSH 102

R14_Tlp13 SKRYVNYIQGIL----NEEVTLTKGVATSLNE-MFQNNDHVD--IDLIESLIKNTFDSSH 102

MTVDSCj16_Tlp13 SKRYVNYIQGIL----NEEVTLTKGVATSLNE-MFQNNDHVD--IDLIESLIKNTFDSSH 102

14980A_Tlp13 SKRYVNYIQGIL----NEEVTLTKGVATSLNE-MFQNNDHVD--IDLIESLIKNTFDSSH 102

15-537360_Tlp20 FLAVYIAQPDGELVVTDPDSDEKG----------------------------LNFGIYGK 134

76339_Tlp20 FLAVYIAQPDGELVVTDPDSDEKG----------------------------LNFGIYGK 134

CFSAN032805_Tlp20 FLAVYIAQPDGELVVTDPDSDEKG----------------------------LNF-IYGK 133

CVM_N29710_Tlp20 FLAVYIAQPDGELVVTDPDSDEKG----------------------------LNF-IYGK 132

YH501_Tlp20 FLAVYIAQPDGELVVTDPDSDEKG----------------------------LNF-IYGK 136

CO2-160_Tlp20 FLAVYIAQPDGELVVTNPDSDEKG----------------------------LNFGIYGK 134

CO2-160_Tlp20b FLAVYIAQPDGELVVTNPDSDEKG----------------------------LNFGIYGK 134

RM5611_Tlp20 FLAVYIAQPDGELVVTNPDSDEKG----------------------------LNFGIYGK 134

14903A_Tlp20 FLAVYIAQSDGELVVTDPDSDEKG----------------------------LNF-IYGK 136

YH502_Tlp20 FLAVYIAQSDGELVVTDPDSDEKG----------------------------LNF-IYGK 133

RM3196_Tlp23 FLAVYIAQPNGELVVSDPDSDAKN----------------------------LDFGTYGK 134

ICDCCJ07001_Tlp23 FLAVYIAQPNGELVVSDPDSDAKN----------------------------LDFGTYGK 134

RM1285_Tlp2 FLAVYIAQPNGELVVSDPDSDAKN----------------------------LDFGTYGK 134

CFSAN032806_Tlp2 FLAVYIAQPNGELVVSDPDSDAKN----------------------------LDFGTYGK 134

RM1221_Tlp2 FLAVYIAQPNGELVVSDPDSDAKN----------------------------LDFGTYGK 134

S3_Tlp2 FLAVYIAQPNGELVVSDPDSDAKN----------------------------LDFGTYGK 134

FDAARGOS_422_Tlp2 FLAVYIAQPNGELVVSDPDSDAKN----------------------------LDFGTYGK 134

81-176_Tlp2 FLAVYIAQPNGELVVSDPDSDAKN----------------------------LDFGTYGK 134

F38011_Tlp2 FLAVYIAQPNGELVVSDPDSDAKN----------------------------LDFGTYGK 134

NCTC11168_Tlp2 FLAVYIAQPNGELVVSDPDSDAKN----------------------------LDFGTYGK 134

MTVDSCj07_Tlp2 FLAVYIAQPNGELVVSDPDSDAKI----------------------------LDFGTYGK 134

CJM1cam_Tlp24 FLAVYIAQPNGELVVSDPDSDAKK----------------------------VDFGTYGK 134

M1_Tlp24 FLAVYIAQPNGELVVSDPDSDAKK----------------------------VDFGTYGK 134

81116_Tlp2 FLAVYIAQPNGELVVSDPDSDAKK----------------------------VDFGTYGK 134

4031_Tlp23 FLAVYIA-PNGELVVSDPDSDAKK----------------------------VDFGTYGK 133

MG1116_Tlp14 LLAVYIGLDNGENIVSDDLSEKKN----------------------------TNITINGK 134

YH502_Tlp14 LLAVYIGLDNGENIVSDDLSEKKN----------------------------TNITINGK 145

BP3181_Tlp14 LLAVYIGLDNGENIVSDDLSEKKN----------------------------TNITINGK 134

ZV1224_Tlp14a LLAVYIGLDNGENIVSDDLSEKKN----------------------------TNITINGK 134

ZV1224_Tlp14b LLAVYIGLDNGENIVSDDLSEKKN----------------------------TNITINGK 134

YH503_Tlp14 LLAVYIGLDNGENIVSDDLSEKKN----------------------------TNITINGK 145

14903A_Tlp14 LLAVYIGLDNGENIVSDDLSEKKN----------------------------TNITINGK 134

OR12_Tlp14 LLAVYIGLDNGENIVSDDLSEKKN----------------------------TNITINGK 134

CFSAN032805_Tlp14 LLAVYIGLDNGENIVSDDLSEKKN----------------------------TNITINGK 145

BFR-CA-9557_Tlp14 VLAVYIGLDNGENIVSDDLSEKKN----------------------------TNITINEK 134

15-537360_Tlp14 VLAVYIGLDNGENIVSDDLSEKKN----------------------------TNITINGK 134

YH501_Tlp14 LLAVYIGLDNGENIVSDDLSEKKN----------------------------TNITINGK 134

CG8421_Tlp14 LLAVYIGLDNGENIMSSDLSEKKN----------------------------TNITINGK 134

MTVDSCj16_Tlp14 LLAVYIGLDNGENIMSSDLSEKKN----------------------------TNITINGK 134

01-1512_Tlp14 LLAVYIGLDNGENIMSSDLSEKKN----------------------------TNITINGK 134

00-0949_Tlp14 LLAVYIGLDNGENIMSSDLSEKKN----------------------------TNITINGK 134

MTVDSCj13_Tlp14 LLAVYIGLDNGENIMSSDLSEKKN----------------------------TNITINGK 134

S3_Tlp14 LLAVYIGLDNGENIMSSDLSEKKN----------------------------TNITINGK 134

PT14_Tlp14 LLAVYIGLDNGENIMSSDLSEKKN----------------------------TNITINGK 134

14980A_Tlp14 LLAVYIGLDNGENIMSSDLSEKKN----------------------------TNITINGK 150

FJ3124_Tlp14 LLAVYIGLDNGENIMSSDLSEKKN----------------------------TNITINGK 134

00-1597_Tlp14 LLAVYIGLDNGENIMSSDLSEKKN----------------------------TNITINGK 134

R14_Tlp14 LLAVYIGLDNGENIMSSDLSEKKN----------------------------TNITINGK 134

CG8421_Tlp25 ------------------------------------------------------------ 0

RM1875_Tlp3 ALNVYLGLNNGKVLLSQKSNDAKM---------------------------------PEL 129

CJM1cam_Tlp3 ALNVYLGLNNGKVLLSQKSNDAKM---------------------------------PEL 129

M1_Tlp3 ALNVYLGLNNGKVLLSQKSNDAKM---------------------------------PEL 129

4031_Tlp3 ALNVYLGLNNGKVLLSQKSNDAKM---------------------------------PEL 129

R14_Tlp3 ALNVYLGLNNGKVLLSQKSNDAKM---------------------------------PEL 129

RM5611_Tlp3 ALNVYLGLNNGKVLLSQKSNDAKM---------------------------------PEL 129

MTVDSCj16_Tlp3 ALNVYLGLNNGKVLLSQKSNDAKM---------------------------------PEL 129

01-1512_Tlp3 ALNVYLGLNNGKVLLSQKSNDAKM---------------------------------PEL 129

MTVDSCj13_Tlp3 ALNVYLGLNNGKVLLSQKSNDAKM---------------------------------PEL 129

32488_Tlp3a ALNVYLGLNNGKVLLSQKSNDAKM---------------------------------PEL 129

81116_Tlp3 ALNVYLGLNNGKVLLSQKSNDAKM---------------------------------PEL 129

32488_Tlp3b ALNVYLGLNNGKVLLSQKSNDAKM---------------------------------PEL 129

FB1_Tlp3 ALNVYLGLNNGKVLLSQKSNDAKM---------------------------------PEL 129

PT14_Tlp3 ALNVYLGLNNGKVLLSQKSNDAKM---------------------------------PEL 129

00-6200_Tlp3a ALNVYLGLNNGKVLLSQKSNDAKM---------------------------------PEL 129

RM1221_Tlp3 ALNVYLGLNNGKVLLSQKSNDAKM---------------------------------PEL 129

S3_Tlp3 ALNVYLGLNNGKVLLSQKSNDAKM---------------------------------PEL 129

FDAARGOS_421_Tlp3 ALNVYLGLNNGKVLLSQKSNDAKM---------------------------------PEL 140

CFSAN032806_Tlp3 ALNVYLGLNNGKVLLSQKSNDAKM---------------------------------PEL 140

IA3901_Tlp3b ALNVYLGLNNGKVLLSQKSNDAKM---------------------------------PEL 129

00-6200_Tlp3b ALNVYLGLNNGKVLLSQKSNDAKM---------------------------------PEL 129

BCW_6290_Tlp3b ALNVYLGLNNGKVLLSQKSNDAKM---------------------------------PEL 129

00-2425_Tlp3a ALNVYLGLNNGKVLLSQKSNDAKM---------------------------------PEL 129

00-2425_Tlp3b ALNVYLGLNNGKVLLSQKSNDAKM---------------------------------PEL 129

YH001_Tlp3a ALNVYLGLNNGKVLLSQKSNDAKM---------------------------------PEL 129

YH001_Tlp3b ALNVYLGLNNGKVLLSQKSNDAKM---------------------------------PEL 129

00-0949_Tlp3 ALNVYLGLNNGKVLLSQKSNDAKM---------------------------------PEL 129

NCTC11168_Tlp3 ALNVYLGLNNGKVLLSQKSNDAKM---------------------------------PEL 140

F38011_Tlp3 ALNVYLGLNNGKVLLSQKSNDAKM---------------------------------PEL 129

RM1285_Tlp3 ALNVYLGLNNGKVLLSQKSNDAKM---------------------------------PEL 129

FDAARGOS_422_Tlp3 ALNVYLGLNNGKVLLSQKSNDAKM---------------------------------PEL 140

MTVDSCj07_Tlp3 ALNVYLGLNNGKVLLSQKSNDAKM---------------------------------PEL 129

IA3901_Tlp3a ALNVYLGLNNGKVLLSQKSNDAKM---------------------------------PEL 129

BCW_6290_Tlp3a ALNVYLGLNNGKVLLSQKSNDAKM---------------------------------PEL 129

35925B2_Tlp3 ALNVYLGLNNGKVLLSQESNDAKM---------------------------------PEL 140

14980A_Tlp3 ALNVYLGLNNGKVLLSQKSNDAKM---------------------------------PEL 140

00-1597_Tlp3b ALNVYLGLNNGKVLLSQKSNDAKM---------------------------------PEL 129

RM3196_Tlp3 ALNVYLGLNNGKVLLSQKSNDAKM---------------------------------PEL 129

FDAARGOS_295_Tlp21 FDSAFFGYDK----------MGKTYLSSG--------------------------DYLDL 119

FORC_046_Tlp4 YDLVYVGFDN----------TGKNYQSDD--------------------------QILDL 127

FDAARGOS_422_Tlp4 YDLVYVGFDN----------TGKNYQSDD--------------------------QILDL 127

ICDCCJ07001_Tlp4 YDLVYVGFDN----------TGKNYQSDD--------------------------QILDL 127

RM3196_Tlp4 YDLVYVGFDN----------TGKNYQSDD--------------------------QILDL 127

T1-21_Tlp4 YDLVYVGFDN----------TGKNYQSDD--------------------------QILDL 127

F38011_Tlp4 YDLVYVGFDN----------TGKNYQSDD--------------------------QILDL 127

HF5-4A-4_Tlp22 YDLVYVGFDN----------TGKNYQSDD--------------------------QILDL 73

00-0949_Tlp4 YDLVYVGFDN----------TGKNYQSDD--------------------------QILDL 127

01-1512_Tlp4 YDLVYVGFDN----------TGKNYQSDD--------------------------QILDL 127

81-176_Tlp4 YDLVYVGFDN----------TGKNYQSDD--------------------------QILDL 127

32488_Tlp4 YDLVYVGFDN----------TGKNYQSDD--------------------------QILDL 127

NCTC11168_Tlp4 YDLVYVGFDN----------TGKNYQSDD--------------------------QILDL 127

CFSAN032806_Tlp4 YDLVYVGFDN----------TGKNYQSDD--------------------------QILDL 127

81116_Tlp4 YDLVYVGFDN----------TGKNYQSDD--------------------------QILDL 127

RM1285_Tlp12 FDIVFIAFDK----------NNKMLLSNG--------------------------TILDK 125

PT14_Tlp12 FDIVFIAFDK----------NNKMLLSNG--------------------------TILDK 125

MTVJDCj07_Tlp12 FDIVFIAFDK----------NNKMLLSNG--------------------------TILDK 125

RM1221_Tlp12 FDIVFIAFDK----------NNKMLLSNG--------------------------TILDK 125

FDAARGOS_421_Tlp12 FDIVFIAFDK----------NNKMLLSNG--------------------------TILDK 125

35925B2_Tlp12 FDIVFIAFEK----------NNKMLLSNG--------------------------TILDK 125

CJM1cam_Tlp12 FDIVFIAFDK----------NNKMLLSNG--------------------------TILDK 125

M1_Tlp12 FDIVFIAFDK----------NNKMLLSNG--------------------------TILDK 125

S3_Tlp12 FDIVFIAFDK----------NNKMLLSNG--------------------------TILDK 125

00-1597_Tlp12 FDIVFIAFDK----------NNKMLLSNG--------------------------TILDK 125

R14_Tlp12 FDIVFIAFDK----------NNKMLLSNG--------------------------TILDK 125

RM1875_Tlp15 KSGYFFVYQKTTVKAHP---VRKD------------------------------------ 115

YH503_Tlp16 KSGYFFVYQKTTVKAHP---VRKD------------------------------------ 115

FB1_Tlp16 KSGYFFVYQKTTVKAHP---VRKD------------------------------------ 115

BFR-CA-9557_Tlp16 KSGYFFVYQKTTVKAHP---VRKD------------------------------------ 114

15-537360_Tlp16 KSGYFFVYQKTTVKAHP---VRKD------------------------------------ 115

OR12_Tlp16 KSGYFFVYQKTTVKAHP---VRKD------------------------------------ 115

YH502_Tlp16 KSGYFFVYQKTTVKAHP---VRKD------------------------------------ 115

14903A_Tlp16 QSGYFFVYQKTTVKAHP---VRKD------------------------------------ 115

RM5611_Tlp16 KSGYFFVYQKTTVKAHP---VRKD------------------------------------ 115

00-2425_Tlp11 YAAYAFLYLQDSSILTHVESLDKNFKNSDGKSVTMIFFDETTGKAGGIKSIHAPSNFSQL 163

00-6200_Tlp11 YAAYAFLYLQDSSILTHVESLDKNFKNSDGKSVTMIFFDETTGKAGGIKSIHAPSNFSQL 163

YH001_Tlp11 YAAYAFLYLQDSSILTHVESLDKNFKNSDGKSVTMIFFDETTGKAGGIKSIHAPSNFSQL 163

IA3902_Tlp11 YAAYAFLYLQDSSILTHVESLDKNFKNSDGKSVTMIFFDETTGKAGGIKSIHAPSNFSQL 163

BCW_6290_Tlp11 YAAYAFLYLQDSSILTHVESLDKNFKNSDGKSVTMIFFDETTGKAGGIKSIHAPSNFSQL 163

76339_Tlp18 YAAYAFLYLKDPSVLSDTYNMDKKYKSQNGNTFAMIFFDETTGKSGGIKAIQTPDNFSQL 162

4031_Tlp17 YSAYTFLYLKDTSVLGDAQGIDKRYTSSDGKTFAMIYFDQTTGKSGGIETIQTPNNFGNL 163

15-537360_Tlp13 YAAYTFLYLKDTTVLSDMQNVDKKYISPDGKTFSMIFFDQIVEKSGGITTISTPNNFSQL 162

CVM_N29710_Tlp13 YAAYTFLYLKDTTVLSDMQNVDKKYISPDGKTFSMIFFDQIVEKSGGITTISTPNNFSQL 162

FB1_Tlp13 YAAYTFLYLKDTTVLSDMQNVDKKYISPDGKTFSMIFFDQIVEKSGGITTISTPNNFSQL 162

CFCAN032805_Tlp13 YAAYTFLYLKDTTVLSDMQNVDKKYISPDGKTFSMIFFDQIVEKSGGITTISTPNNFSQL 162

BG2108_Tlp13 YAAYTFLYLKDTTVLSDMQNVDKKYISPDGKTFSMIFFDQIVEKSGGITTISTPNNFSQL 162

YF2105_Tlp13 YAAYTFLYLKDTTVLSDMQNVDKKYISPDGKTFSMIFFDQIVEKSGGITTISTPNNFSQL 162

YH503_Tlp13 YAAYTFLYLKDTTVLSDMQNVDKKYISPDGKTFSMIFFDQIVEKSGGITTISTPNNFSQL 162

BFRCA9557_Tlp13 YAAYTFLYLKDTTVLSDMQNVDKKYISPDGKTFSMIFFDQIVEKSGGITTISTPNNFSQL 162

YH502_Tlp13 YAAYTFLYLKDTTVLSDMQNVDKKYISPDGKTFSMIFFDQIVEKSGGITTISTPNNFSQL 162

MTVDSCj13_Tlp13 YAAYTFLYLKDTTVLSDMQNVDKKYISPDGKTFSMIFFDQIAEKSGGITTISTPNNFSQL 162

OR12_Tlp13 YAAYTFLYLKDTTVLSDMQNVDKKYISPDGKTFSMIFFDQIAEKSGGITTISTPNNFSQL 162

00-1597_Tlp13 YAAYTFLYLKDTTVLSDMQNVDKKYISPDGKTFSMIFFDQIVEKSGGITTISTPNNFSQL 162

14903A_Tlp13 YAAYTFLYLKDTTVLSDMQNVDKKYISPDGKTFSMIFFDQIVEKSGGITTISTPNNFSQL 162

FJ3124_Tlp13 YAAYTFLYLKDTTVLSDMQNVDKKYISPDGKTFSMIFFDQIVEKSGGITTISTPNNFSQL 162

R14_Tlp13 YAAYTFLYLKDTTVLSDMQNVDKKYISPDGKTFSMIFFDQIAEKSGGITTISTPNNFSQL 162

MTVDSCj16_Tlp13 YAAYTFLYLKDTTVLSDMQNVDKKYISPDGKTFSMIFFDQIAEKSGGITTISTPNNFSQL 162

14980A_Tlp13 YAAYTFLYLKDTTVLSDMQNVDKKYISPDGKTFSMIFFDQIAEKSGGITTISTPNNFSQL 162

15-537360_Tlp20 ADNYDARTRDYFKGAVKANGLYVTPSYLDLTT--NLPCFTYATPLYK-EGKFIGVLAI-D 190

76339_Tlp20 ADNYDARTRDYFKGAVKANGLYVTPSYLDLTT--NLPCFTYAIPLYK-EGKFIGVLAI-D 190

CFSAN032805_Tlp20 ADNYDARTRDYFKGAVKANGLYVTPSYLDLTT--NLPCFTYATPLYK-EGKFIGVLAI-D 189

CVM_N29710_Tlp20 ADNYDARTRDYFKGAVKANGLYVTPSYLDLTT--NLPCFTYATPLYK-EGKFIGVLAI-D 188

YH501_Tlp20 ADNYDARTRDYFKGAVKANGLYVTPSYLDLTT--NLPCFTYATPLYK-EGKFIGVLAI-D 192

CO2-160_Tlp20 ADNYDARTRDYFKGAVKANGLYVTPSYLDLTT--NLPCFTYATPLYK-EGKFIGVLAI-D 190

CO2-160_Tlp20b ADNYDARTRDYFKGAVKANGLYVTPSYLDLTT--NLPCFTYATPLYK-EGKFIGVLAI-D 190

RM5611_Tlp20 ADNYDARTRDYFKGAVKANGLYVTPSYLDLTT--NLPCFTYATPLYK-EGKFIGVLAI-D 190

14903A_Tlp20 ADNYDARTRDYFKGAVKANGLYVTPSYLDLTT--NLPCFTYATPLYK-EGKFIGVLAI-D 192

YH502_Tlp20 ADNYDARTRDYFKGAVKANGLYVTPSYLDLTT--NLPCFTYATPLYK-EGKFIGVLAI-D 189

RM3196_Tlp23 ADNYDARTREYYIEAVKTNKLYITPSYIDVTT--NLPCFTYSIPLYK-DGKFIGVLAV-D 190

ICDCCJ07001_Tlp23 ADNYDARTREYYIEAVKTNKLYITPSYIDVTT--NLPCFTYSIPLYK-DGKFIGVLAV-D 190

RM1285_Tlp2 ADNYDARTREYYIEAVKTNKLYITPSYIDVTT--NLPCFTYSIPLYK-DGKFIGVLAV-D 190

CFSAN032806_Tlp2 ADNYDARTREYYIEAVKTNKLYITPSYIDVTT--NLPCFTYSIPLYK-DGKFIGVLAV-D 190

RM1221_Tlp2 ADNYNARTREYYIEAVKTNKLYITPSYIDVTT--NLPCFTYSIPLYK-DGKFIGVLAV-D 190

S3_Tlp2 ADNYDARTREYYIEAVKTNKLYITPSYIDVTT--NLPCFTYSIPLYK-DGKFIGVLAV-D 190

FDAARGOS_422_Tlp2 ADNYDARTREYYIEAVKTNKLYITPSYIDVTT--NLPCFTYSIPLYK-DGKFIGVLAV-D 190

81-176_Tlp2 ADNYDARTREYYIEAVKTNKLYITPSYIDVTT--NLPCFTYSIPLYK-DGKFIGVLAV-D 190

F38011_Tlp2 ADNYDARTREYYIEAVKTNKLYITPSYIDVTT--NLPCFTYSIPLYK-DGKFIGVLAV-D 190

NCTC11168_Tlp2 ADNYDARTREYYIEAVKTNKLYITPSYIDVTT--NLPCFTYSIPLYK-DGKFIGVLAV-D 190

MTVDSCj07_Tlp2 ADNYDARTREYYIEAVKTNKLYITPSYIDVTT--NLPCFTYSIPLYK-DGKFIGVLAV-D 190

CJM1cam_Tlp24 ADNYDARTREYYIEAVKTNKLYVTPSYIDATT--NLPCFTYSTPLYK-DGKFIGVLAV-D 190

M1_Tlp24 ADNYDARTREYYIEAVKTNKLYVTPSYIDATT--NLPCFTYSTPLYK-DGKFIGVLAV-D 190

81116_Tlp2 ADNYDARTREYYIEAVKTNKLYVTPSYIDATT--NLPCFTYSTPLYK-DGKFIGVLAV-D 190

4031_Tlp23 ADNYDARTREYYIEAVKTNKLYVTPSYIDATT--NLPCFTYSTPLFK-DGKFIGVLAV-D 189

MG1116_Tlp14 ANNYNATTREWYKEARNSNQIYITPAYIDVVS--NEYAITYSKALYK-DGKFIGVLGF-D 190

YH502_Tlp14 ANNYNATTREWYKEARNSNQIYITPAYIDVVS--NEYAITYSKALYK-DGKFIGVLGI-D 201

BP3181_Tlp14 ANNYNATTREWYKEARNSNQMYITPAYIDVVS--NEYAITYSKALYK-DGKFIGVLGF-D 190

ZV1224_Tlp14a ANNYNATTREWYKEARNSNQTYITPAYIDVVS--NEYAITYSKALYK-DGKFIGVLGF-D 190

ZV1224_Tlp14b ANNYNATTREWYKEARNSNQTYITPAYIDVVS--NEYAITYSKALYK-DGKFIGVLGF-D 190

YH503_Tlp14 ANNYNATTREWYKEARNSNQIYITPAYIDVVS--NEYAITYSKALYK-DGKFIGVLGI-D 201

14903A_Tlp14 ANNYNATTREWYKEARNSNQTYITPAYIDVVS--NEYAITYSKALYK-DGKFIGVLGF-D 190

OR12_Tlp14 ANNYNATTREWYKEARNSNQTYITPAYIDVVS--NEYAITYSKALYK-DGKFIGVLGI-D 190

CFSAN032805_Tlp14 ANNYNATTREWYKEARNSNQTYITPAYIDVVS--NEYAITYSKALYK-DGKFIGVLGF-D 201

BFR-CA-9557_Tlp14 ANNYNATTREWYKEARNSNQTYITPAYIDVVS--NEYAITYSKALYK-DGKFIGVLGF-D 190

15-537360_Tlp14 ANNYNATTREWYKEARNSNQTYITPAYIDVVS--NEYAITYSKALYK-DGKFIGVLGL-D 190

YH501_Tlp14 ANNYNATTREWYKEARNSNQTYITPAYIDVVS--NEYAITYSKALYK-DGKFIGVLGF-D 190

CG8421_Tlp14 ANNYNATTREWYKEARNSNQIYITPAYIDAIS--NEYCITYSKALYK-DGKFIGVLGI-D 190

MTVDSCj16_Tlp14 ANNYNATTREWYKEARNSNQINITPAYIDAIS--NEYCITYSKALYK-DGKFIGVLGI-D 190

01-1512_Tlp14 ANNYNATTREWYKEARNSNQIYITPAYIDAIS--NEYCITYSKALYK-DGKFIGVLGI-D 190

00-0949_Tlp14 ANNYNATTREWYKEARNSNQIYITPAYIDAIS--NEYCITYSKALYK-DGKFIGVLGI-D 190

MTVDSCj13_Tlp14 ANNYNATTREWYKEARNSNQIYITPAYIDAIS--NEYCITYSKALYK-DGKFIGVLGI-D 190

S3_Tlp14 ANNYNATTREWYKEARNSNQIYITPAYIDAIS--NEYCITYSKALYK-DGKFIGVLGI-D 190

PT14_Tlp14 ANNYNATTREWYKEARNSNQIYITPAYIDAVS--NEYCITYSKALYK-DGKFIGVLGI-D 190

14980A_Tlp14 ANNYNATTREWYKEARNSNQIYITPAYIDAIS--NEYCITYSKALYK-DGKFIGVLGI-D 206

FJ3124_Tlp14 ANNYNATTREWYKEARNSNQIYITPAYIDAIS--NEYCITYSKALYK-DGKFIGVLGI-D 190

00-1597_Tlp14 ANNYNATTREWYKEARNSNQIYITPAYIDVVS--NEYCITYSKALYK-DGKFIGVLGF-D 190

R14_Tlp14 ANNYNATTREWYKEARNSNQIYITPAYIDAIS--NEYCITYSKALYK-DGKFIGVLGI-D 190

CG8421_Tlp25 ------------------------------------------MQSIN-SGKSVGVSVKLT 17

RM1875_Tlp3 RDDLDIKTKDWYQEALKTNDIFDTPAYLDTNL--KQYVITYSKAIYK-DGKIIGVLGV-D 185

CJM1cam_Tlp3 RDDLDIKTKDWYQEALKTNDIFVTPAYLDTNL--KQYVITYSKAIYK-DGKIIGVLGV-D 185

M1_Tlp3 RDDLDIKTKDWYQEALKTNDIFVTPAYLDTNL--KQYVITYSKAIYK-DGKIIGVLGV-D 185

4031_Tlp3 RDDLDIKTKDWYQEALKTNDIFVTPAYLDTNL--KQYVITYSKAIYK-DGKIIGVLGV-D 185

R14_Tlp3 RDDLDIKTKDWYQEALKTNDIFVTPAYLDTIL--KQYVITYSKAIYK-DGKIIGVLGV-D 185

RM5611_Tlp3 RDDLDIKTKDWYQEALKTNDIFVTPAYLDTVL--KQYVITYSKAIYK-DGKIIGVLGV-D 185

MTVDSCj16_Tlp3 RDDLDIKTKDWYQEALKTNDIFVTPAYLDTIL--KQYVITYSKAIYK-DGKIIGVLGV-D 185

01-1512_Tlp3 RDDLDIKTKDWYQEALKTNDIFVTPAYLDTVL--KQYVITYSKAIYK-DGKIIGVLGV-D 185

MTVDSCj13_Tlp3 RDDLDIKTKDWYQEALKTNDIFVTPAYLDTIL--KQYVITYSKAIYK-DGKIIGVLGV-D 185

32488_Tlp3a RDDLDIKTKDWYQEALKTNDIFVTPAYLDTIL--KQYVITYSKAIYK-DGKIIGVLGV-D 185

81116_Tlp3 RDDLDIKTKDWYQEALKTNDIFVTPAYLDTIL--KQYVITYSKAIYK-DGKIIGVLGV-D 185

32488_Tlp3b RDDLDIKTKDWYQEALKTNDIFVTPAYLDTIL--KQYVITYSKAIYK-DGKIIGVLGV-D 185

FB1_Tlp3 RDDLDIKTKDWYQEALKTNDIFVTPAYLDTVL--KQYVITYSKAIYK-DGKIIGVLGV-D 185

PT14_Tlp3 RDDLDIKTKDWYQEALKTNDIFVTPAYLDTVL--KQYVITYSKAIYK-DGKIIGVLGV-D 185

00-6200_Tlp3a RDDLDIKTKDWYQEALKTNDIFVTPAYLDTVL--KQYVITYSKAIYK-DGKIIGVLGV-D 185

RM1221_Tlp3 RDDLDIKTKDWYQEALKTNDIFVTPAYLDTIL--KQYVITYSKAIYK-DGKIIGVLGV-D 185

S3_Tlp3 RDDLDIKTKDWYQEALKTNDIFVTPAYLDTIL--KQYVITYSKAIYK-DGKIIGVLGV-D 185

FDAARGOS_421_Tlp3 RDDLDIKTKDWYQEALKTNDIFVTPAYLDTIL--KQYVITYSKAIYK-DGKIIGVLGV-D 196

CFSAN032806_Tlp3 RDDLDIKTKDWYQEALKTNDIFVTPAYLDTVL--KQYVITYSKAIYK-DGKIIGVLGV-D 196

IA3901_Tlp3b RDDLDIKTKDWYQEALKTNDIFVTPAYLDTVL--KQYVITYSKAIYK-DGKIIGVLGV-D 185

00-6200_Tlp3b RDDLDIKTKDWYQEALKTNDIFVTPAYLDTVL--KQYVITYSKAIYK-DGKIIGVLGV-D 185

BCW_6290_Tlp3b RDDLDIKTKDWYQEALKTNDIFVTPAYLDTVL--KQYVITYSKAIYK-DGKIIGVLGV-D 185

00-2425_Tlp3a RDDLDIKTKDWYQEALKTNDIFVTPAYLDTVL--KQYVITYSKAIYK-DGKIIGVLGV-D 185

00-2425_Tlp3b RDDLDIKTKDWYQEALKTNDIFVTPAYLDTVL--KQYVITYSKAIYK-DGKIIGVLGV-D 185

YH001_Tlp3a RDDLDIKTKDWYQEALKTNDIFVTPAYLDTVL--KQYVITYSKAIYK-DGKIIGVLGV-D 185

YH001_Tlp3b RDDLDIKTKDWYQEALKTNDIFVTPAYLDTVL--KQYVITYSKAIYK-DGKIIGVLGV-D 185

00-0949_Tlp3 RDDLDIKTKDWYQEALKTNDIFVTPAYLDTVL--KQYVITYSKAIYK-DGKIIGVLGV-D 185

NCTC11168_Tlp3 RDDLDIKTKDWYQEALKTNDIFVTPAYLDTVL--KQYVITYSKAIYK-DGKIIGVLGV-D 196

F38011_Tlp3 RDDLDIKTKDWYQEALKTNDIFVTPAYLDTVL--KQYVITYSKAIYK-DGKIIGVLGV-D 185

RM1285_Tlp3 RDDLDIKTKDWYQEALKTNDIFVTPAYLDTVL--KQYVITYSKAIYK-DGKIIGVLGV-D 185

FDAARGOS_422_Tlp3 RDDLDIKTKDWYQEALKTNDIFVTPAYLDTVL--KQYVITYSKAIYK-DGKIIGVLGV-D 196

MTVDSCj07_Tlp3 RDDLDIKTKDWYQEALKTNDIFVTPAYLDTVL--KQYVITYSKAIYK-DGKIIGVLGV-D 185

IA3901_Tlp3a RDDLDIKTKDWYQEALKTNDIFVTPAYLDTVL--KQYVITYSKAIYK-DGKIIGVLGV-D 185

BCW_6290_Tlp3a RDDLDIKTKDWYQEALKTNDIFVTPAYLDTVL--KQYVITYSKAIYK-DGKIIGVLGV-D 185

35925B2_Tlp3 RDDLDIKTKDWYQEALKTNDIFVTPAYLDTIL--KQYVITYSKAIYK-DGKIIGVLGI-D 196

14980A_Tlp3 RDDLDIKTKDWYQEALKTNDIFVTPAYLDTIL--KQYVITYSKAIYK-DGKIIGVLGV-D 196

00-1597_Tlp3b RDDLDIKTKDWYQEALKTNDIFVTPAYLDTVL--KQYVITYSKAIYK-DGKIIGVLGV-D 185

RM3196_Tlp3 RDDLDIKTKDWYQEALKTNDIFVTPAYLDTVL--KQYVITYSKAIYK-DGKIIGVLGV-D 185

FDAARGOS_295_Tlp21 SKNYDVTTRAWYKGAKENNGIVITPPYLSRST--GNIAIGYGIPVVV-EGKIVGVVGSEY 176

FORC_046_Tlp4 SKGYDTKNRPWYKAAKEAKKLIVTEPYKSAAS--GEVGLTYAAPFYDRNGNFRGVVGGDY 185

FDAARGOS_422_Tlp4 SKGYDTKNRPWYKAAKEAKKLIVTEPYKSAAS--GEVGLTYAAPFYDRNGNFRGVVGGDY 185

ICDCCJ07001_Tlp4 SKGYDTKNRPWYKAAKEAKKLIVTEPYKSANS--GEVGLTYAAPFYDRNGNFRGVVGGDY 185

RM3196_Tlp4 SKGYDTKNRPWYKAAKEAKKLIVTEPYKSANS--GEVGLTYAAPFYDRNGNFRGVVGGDY 185

T1-21_Tlp4 SKGYDTKNRPWYKAAKEAKKLIVTEPYKSANS--GEVGLTYAAPFYDRNGNFRGVVGGDY 185

F38011_Tlp4 SKGYDTKNRPWYKAAKEAKKLIVTEPYKSAAS--GEVGLTYAAPFYDRNGNFRGVVGGDY 185

HF5-4A-4_Tlp22 SKGYDTKNRPWYKAAKEAKKLIVTEPYKSAAS--GEVGLTYAAPFYDRNGNFRGVVGGDY 131

00-0949_Tlp4 SKGYDTKNRPWYKAAKEAKKLIVTEPYKSAAS--GEVGLTYAAPFYDRNGNFRGVVGGDY 185

01-1512_Tlp4 SKGYDTKNRPWYKAAKEAKKLIVTEPYKSAAS--GEVGLTYAAPFYDRNGNFRGVVGGDY 185

81-176_Tlp4 SKGYDTKNRPWYKAAKEAKKLIVTEPYKSAAS--GEVGLTYAAPFYDRNGNFRGVVGGDY 185

32488_Tlp4 SKGYDTKNRPWYKAAKEAKKLIVTEPYKSAAS--GEVGLTYAAPFYDRNGNFRGVVGGDY 185

NCTC11168_Tlp4 SKGYDTKNRPWYKAAKEAKKLIVTEPYKSAAS--GEVGLTYAAPFYDRNGNFRGVVGGDY 185

CFSAN032806_Tlp4 SKGYDTKNRPWYKAAKEAKKLIVTEPYKSAAS--GEVGLTYAAPFYDRNGNFRGVVGGDY 185

81116_Tlp4 SKGYDTKNRPWYKAAKEAKKLIVTEPYKSAAS--GEVGLTYAAPFYDRNGNFRGVVGGDY 185

RM1285_Tlp12 KSNFDITKQIWYQEAKNNKGITITQPYKSPID--QEIGITYVFPIYKNN-QLIAFVGGDY 182

PT14_Tlp12 KSNFDITKQIWYQEAKNNKGITITQPYKSPID--QEIGITYVFPIYKNN-QLIAFVGGDY 182

MTVJDCj07_Tlp12 KSNFDITKQIWYQEAKNNKGITITQPYKSPID--QEIGITYVFPIYKNN-QLIAFVGGDY 182

RM1221_Tlp12 KSNFDITKQIWYQEAKNNKGITITQPYKSPID--QEIGITYVFPIYKNN-QLIAFVGGDY 182

FDAARGOS_421_Tlp12 KSNFDITKQIWYQEAKNNKGITITQPYKSPID--QEIGITYVFPIYKNN-QLIAFVGGDY 182

35925B2_Tlp12 KSNFDITKQIWYQEAKNNKGITITQPYKSPID--QEIGITYVFPIYKNN-QLIAFVGGDY 182

CJM1cam_Tlp12 KSNFDITKQIWYQEAKNNKGITITQPYKSPID--QEIGITYVFPIYKNN-QLIAFVGGDY 182

M1_Tlp12 KSNFDITKQIWYQEAKNNKGITITQPYKSPID--QEIGITYVFPIYKNN-QLIAFVGGDY 182

S3_Tlp12 KSNFDITKQIWYQEAKNNKGITITQPYKSPID--QEIGITYVFPIYKNN-QLIAFVGGDY 182

00-1597_Tlp12 KSNFDITKQIWYQEAKNNKGITITQPYKSPID--QEIGITYVFPIYKNN-QLIAFVGGDY 182

R14_Tlp12 KSNFDITKQIWYQEAKNNKGITITQPYKSPID--QEIGITYVFPIYKNN-QLIAFVGGDY 182

RM1875_Tlp15 -----LIGTDLYNAKDENGVFYVRELYQRALEKGGFVTFHFTKPQPNGENTIAEKTAY-- 168

YH503_Tlp16 -----LIGTDLHNAKDENGIFYVRELYQRALDKGGFVTFHFTKPQPNGENTIAEKTAY-- 168

FB1_Tlp16 -----LIGTDLHNAKDENGIFYVRELYQRALDKGGFVTFHFTKPQPNGENTIAEKTAY-- 168

BFR-CA-9557_Tlp16 -----LIGTDLHNAKDENGIFYVRELYQRALDKGGFVTFHFTKPQPNGENTIAEKTAY-- 167

15-537360_Tlp16 -----LIGTDLHNAKDENGIFYVRELYQRALDKGGFVTFHFTKPQPNGENTIAEKTAY-- 168

OR12_Tlp16 -----LIGTDLHNAKDENGIFYVRELYQRALDKGGFVTFHFTKPQPNGENTIAEKTAY-- 168

YH502_Tlp16 -----LIGTDLHNAKDENGIFYVRELYQRALDKGGFVTFHFTKPQPNGENTIAEKTAY-- 168

14903A_Tlp16 -----LIGTDLYNAKDENGIFYVRELYQRALDKGGFVTFHFTKPQPNGENTIAEKTAY-- 168

RM5611_Tlp16 -----LIGSDLYNAKDENGIFYVRELYQRALDKGGFVTFHFTKPQPNGENTIAEKTAY-- 168

00-2425_Tlp11 PIIEKIKKNARYGD---LDTIFLGSPSRLNYDGTEFLGINLGMPLFNKEGKFIGIVGFTF 220

00-6200_Tlp11 PIIEKIKKNARYGD---LDTIFLGSPSRLNYDGTEFLGINLGMPLFNKEGKFIGIVGFTF 220

YH001_Tlp11 PIIEKIKKNARYGD---LDTIFLGSPSRLNYDGTEFLGINLGMPLFNKEGKFIGIVGFTF 220

IA3902_Tlp11 PIIEKIKKNARYGD---LDTIFLGSPSRLNYDGTEFLGINLGMPLFNKEGKFIGIVGFTF 220

BCW_6290_Tlp11 PIIEKIKKNARYGD---LDTIFLGSPSRLNYDGTEFLGINLGMPLFNKEGKFIGIVGFTF 220

76339_Tlp18 RIIQDIEKNARYGS---RDTLFIGSPTKLNYDGTEFLGINFGMPIFNSKGNFIGVVGYSL 219

4031_Tlp17 KIIEQVEKNAKYGD---KDSLFVGPPTKLNYDGKDFLGINFGMPIFNNKGKLIGVAGYTL 220

15-537360_Tlp13 NLIQNIEQNAKYGD---KDSVFVDSPRKLNYDNNEFLGINFGMPIFNNKGKFIGVIGYTI 219

CVM_N29710_Tlp13 NLIQNIEQNAKYGD---KDSVFVDSPRKLNYDNNEFLGINFGMPIFNNKGKFIGVIGYTI 219

FB1_Tlp13 NLIQNIEQNAKYGD---KDSVFVDSPRKLNYDNNEFLGINFGMPIFNNKGKFIGVIGYTI 219

CFCAN032805_Tlp13 NLIQNIEQNAKYGD---KDSVFVDSPRKLNYDNNEFLGINFGMPIFNNKGKFIGVIGYTI 219

BG2108_Tlp13 NLIQNIEQNAKYGD---KDSVFVDSPRKLNYDNNEFLGINFGMPIFNNKGKFIGVIGYTI 219

YF2105_Tlp13 NLIQNIEQNAKYGD---KDSVFVDSPRKLNYDNNEFLGINFGMPIFNNKGKFIGVIGYTI 219

YH503_Tlp13 NLIQNIEQNAKYGD---KDSVFVDSPRKLNYDNNEFLGINFGMPIFNNKGKFIGVIGYTI 219

BFRCA9557_Tlp13 NLIQNIEQNAKYGD---KDSVFVDSPRKLNYDNNEFLGINFGMPIFNNKGKFIGVIGYTI 219

YH502_Tlp13 NLIQNIEQNAKYGD---KDSVFVDSPRKLNYDNNEFLGINFGMPIFNNKGKFIGVIGYTI 219

MTVDSCj13_Tlp13 NLIQNIEQNAKYGD---KDSVFVGSPRKLNYDNNEFLGINFGMPIFNNKGKFIGVIGYTL 219

OR12_Tlp13 NLIQNIEQNAKYGD---KDSVFVDSPRKLNYDNNEFLGINFGMPIFNNKGKFIGVIGYTI 219

00-1597_Tlp13 NLIQNIEQNAKYGD---KDSVFVDSPRKLNYDNNEFLGINFGMPIFNNKGKFIGVIGYTI 219

14903A_Tlp13 NLIQNIEQNAKYGD---KDSVFVDSPRKLNYDNNEFLGINFGMPIFNNKGKFIGVIGYTI 219

FJ3124_Tlp13 NLIQNIEQNAKYGD---KDSVFVDSPRKLNYDNNEFLGINFGMPIFNNKGKFIGVIGYTI 219

R14_Tlp13 NLIQNIEQNAKYGD---KDSVFVGSPRKLNYDNNEFLGINFGMPIFNNKGKFIGVIGYTI 219

MTVDSCj16_Tlp13 NLIQNIEQNAKYGD---KDSVFVGSPRKLNYDNNEFLGINFGMPIFNNKGKFIGVIGYTI 219

14980A_Tlp13 NLIQNIEQNAKYGD---KDSVFVGSPRKLNYDNNEFLGINFGMPIFNNKGKFIGVIGYTI 219

.

15-537360_Tlp20 ILVKDLQREFENLPGR-------TFVFDSENSVFVSTDK--------ELLKPGYDVSPVA 235

76339_Tlp20 ILVKDLQREFENLPGR-------TFVFDSKNSIFASTDK--------ELLKPGYDVSPVA 235

CFSAN032805_Tlp20 ILVKDLQREFENLPGR-------TFVFDSENSIFVSTNK--------ELLKPGYDVSPVA 234

CVM_N29710_Tlp20 ILVKDLQREFENLPGR-------TFVFDSENSIFVSTDK--------ELLKPGYDVSPVA 233

YH501_Tlp20 ILVKDLQREFENLPGR-------TFVFDSENSIFVSTDK--------ELLKPGYDVSPVA 237

CO2-160_Tlp20 ILVKDLQREFENLPGR-------TFVFDSENSIFVSTDK--------ELLKPGYDVSPVA 235

CO2-160_Tlp20b ILVKDLQREFENLPGR-------TFVFDSENSIFVSTDK--------ELLKPGYDVSPVA 235

RM5611_Tlp20 ILVKDLQREFENLPGR-------TFVFDSENSIFVSTDK--------ELLKPGYDVSPVA 235

14903A_Tlp20 ILVKDLQREFENLPGR-------TFVFDSENSIFVSTNK--------ELLKPGYDVSLVA 237

YH502_Tlp20 ILVKDLQREFENLPGR-------TFVFDSENSIFVSTNK--------ELLKPGYDVSLVA 234

RM3196_Tlp23 VLAADLQAEFENLPGR-------IFVFDEENKVFVSTDK--------TLLQQGYDISTIA 235

ICDCCJ07001_Tlp23 ILAADLQAEFENLPGR-------IFVFDEENKVFVSTDK--------TLLQQGYDISTIA 235

RM1285_Tlp2 ILAADLQAEFENLPGR-------TFVFDEENKVFVSTDK--------ALLQKGYDISAIA 235

CFSAN032806_Tlp2 ILAADLQAEFENLPGR-------TFVFDEENKVFVSTDK--------TLLQQGYDISTIA 235

RM1221_Tlp2 ILAADLQAEFENLPGR-------TFVFDEENKVFVSTDK--------TLLQQGYDISTIA 235

S3_Tlp2 ILAADLQAEFENLPGR-------TFVFDEENKVFVSTDK--------TLLQQGYDISTIA 235

FDAARGOS_422_Tlp2 ILAADLQAEFENLPGR-------TFVFDEENKVFVSTDK--------ALLQKGYDISAIA 235

81-176_Tlp2 ILAADLQAEFENLPGR-------TFVFDEENKVFVSTDK--------ALLQKGYDISAIA 235

F38011_Tlp2 ILAADLQAEFENLPGR-------TFVFDEENKVFVSTDK--------ALLQKGYDISAIA 235

NCTC11168_Tlp2 ILAADLQAEFENLPGR-------TFVFDEENKVFVSTDK--------ALLQKGYDISAIA 235

MTVDSCj07_Tlp2 ILAADLQAEFENLPGR-------TFVFDEENKVFVSTDK--------ALLQKGYDISAIA 235

CJM1cam_Tlp24 VLVTDLQAEFENLPGR-------TFVFDEENKVFASTDK--------TLLQQGYDISAIA 235

M1_Tlp24 VLVTDLQAEFENLPGR-------TFVFDEENKVFASTDK--------TLLQQGYDISAIA 235

81116_Tlp2 VLVTDLQAEFENLPGR-------TFVFDEENKVFASTDK--------TLLQQGYDISAIA 235

4031_Tlp23 VLVTDLQAEFENLPGR-------TFVFDEENKVFASTDK--------TLLQQGYDISAIA 234

MG1116_Tlp14 VLLISLQDEIARTPGN-------TFVFDHQDRIFAATNK--------ALLDPSVDHSPVL 235

YH502_Tlp14 VLLTSLQDRIARTPGN-------TFVFDHKDRVFAATNK--------ALLDPSVDHSPVL 246

BP3181_Tlp14 VLLTSLQDRIARTPGN-------SFVFDHKDRIFAATNK--------ALLDPSVDHSPVL 235

ZV1224_Tlp14a VLLIDLQDKIARTPGN-------TFVFDHQDRIFAATNK--------ALLDPSVDHSPVL 235

ZV1224_Tlp14b VLLIDLQDKIARTPGN-------TFVFDHQDRIFAATNK--------ALLDPSVDHSPVL 235

YH503_Tlp14 VLLTSLQDRIARTPGN-------TFVFDHKDRVFAATNE--------ALLDPSVDHSPVL 246

14903A_Tlp14 VLLINLQDEIARTPGN-------TFVFDHQDRIFAATNK--------ALLDPSVDHSPVL 235

OR12_Tlp14 VLLTNLQDEIARTPGN-------TFVFDHKDRVFAATNK--------ALLDPSVDHSPVL 235

CFSAN032805_Tlp14 VLLINLQDEIARTPGN-------TFVFDHKDRVFAAANK--------ALLDPSVDHSPVL 246

BFR-CA-9557_Tlp14 VLLISLQDEIARTPGN-------TFVFDHKDRVFAATNK--------ALLDPSVDHSPVL 235

15-537360_Tlp14 VLLISLQDEIARTPGN-------TFVFDHKDRVFAATNK--------ALLDPSVDHSPVL 235

YH501_Tlp14 VLLISLQDEIARTPGN-------TFVFDHKDRVFAATNK--------ALLDPSVDHSPVL 235

CG8421_Tlp14 ILLTSLQDQIARTPGN-------TFVFDNKDKIFAATNE--------ALLDPSVDHSPVL 235

MTVDSCj16_Tlp14 ILLTSLQDQIARTPGN-------TFVFDNKDKIFAATNE--------ALLDPSVDHSPVL 235

01-1512_Tlp14 ILLTSLQDQIARTPGN-------TFVFDNKDKIFAATNE--------ALLDPSVDHSPVL 235

00-0949_Tlp14 ILLTSLQDQIARTPGN-------TFVFDNKDKIFAATNE--------ALLDPSVDHSPVL 235

MTVDSCj13_Tlp14 ILLTSLQDQIARTPGN-------TFVFDNKDKIFAATNE--------ALLDPSVDHSPVL 235

S3_Tlp14 ILLTSLQDQIARTPGN-------TFVFDNKDKIFAATNE--------ALLDPSVDHSPVL 235

PT14_Tlp14 ILLTSLQDQIARTPGN-------TFVFDNKDKIFAATNE--------ALLDPSVDHSPVL 235

14980A_Tlp14 ILLTSLQDQIARTPGN-------TFVFDNKDKIFAATNE--------ALLDPSVDHSPVL 251

FJ3124_Tlp14 ILLTSLQDQIARTPGN-------TFVFDNKDKIFAATNE--------ALLDPSVDHSPVL 235

00-1597_Tlp14 VLLTSLQDRIARTPGN-------TFVFDHKDKVFAATNK--------ALLDPSVDHSPVL 235

R14_Tlp14 ILLTSLQDQIARTPGN-------TFVFDNKDKIFAATNE--------ALLDPSVDHSPVL 235

CG8421_Tlp25 LWVGILVVLILAITST-------VSYFDAKNHTY-------------------------- 44

RM1875_Tlp3 IPSEDLQNLVANTPGN-------TFLFDQKNKIFAATNK--------ELLNPSIDHSPVL 230

CJM1cam_Tlp3 IPSEDLQNLVAKTPGN-------TFLFDQKNKIFAATNK--------ELLNPSIDHSPVL 230

M1_Tlp3 IPSEDLQNLVAKTPGN-------TFLFDQKNKIFAATNK--------ELLNPSIDHSPVL 230

4031_Tlp3 IPSEHLQNLVAKTPGN-------TFLFDQKNKIFAATNK--------ELLNPSIDHSPVL 230

R14_Tlp3 IPLEDLQNSVANTPGN-------TFLFDQKNKIFAATNK--------ELLNPSIDHSPVL 230

RM5611_Tlp3 IPSEDLQNLVAKTPGN-------TFLFDQKNKIFAATNK--------ELLNPSIDHSPVL 230

MTVDSCj16_Tlp3 IPSEDLQNLVAKTPGN-------TFLFDQKNKIFAATNE--------ALLDPSVDHSPVL 230

01-1512_Tlp3 IPSEDLQNLVAKTPGN-------TFLFDQKNKIFAATNK--------ELLNPSIDHSPVL 230

MTVDSCj13_Tlp3 IPSEDLQNLVANTPGN-------TFLFDQKNKIFAATNK--------ELLNPSIDHSPVL 230

32488_Tlp3a IPLEDLQNSVAKTPGN-------TFLFDQKNKIFAATNK--------ELLNPSIDHSPVL 230

81116_Tlp3 IPLEDLQNSVAKTPGN-------TFLFDQKNKIFAATNK--------ELLNPSIDHSPVL 230

32488_Tlp3b IPLEDLQNSVAKTPGN-------TFLFDQKNKIFAATNK--------ELLNPSIDHSPVL 230

FB1_Tlp3 IPSEDLQNLVAKTPGN-------TFLFDQKNKIFAATNK--------ELLNPSIDHSPVL 230

PT14_Tlp3 IPSEDLQNLVAKTPGN-------TFLFDQKNKIFAATNE--------ALLDPSVDHSPVL 230

00-6200_Tlp3a IPSEDLQNLVAKTPGN-------TFLFDQKNKIFAATNK--------ELLNPSIDHSPVL 230

RM1221_Tlp3 IPSEDLQNLVANTPGN-------TFLFDQKNKIFAATNK--------ELLNPSIDHSPVL 230

S3_Tlp3 IPSEDLQNLVANTPGN-------TFLFDQKNKIFAATNK--------ELLNPSIDHSPVL 230

FDAARGOS_421_Tlp3 IPSEDLQNLVANTPGN-------TFLFDQKNKIFAATNK--------ELLNPSIDHSPVL 241

CFSAN032806_Tlp3 IPSEDLQNLVAKTPGN-------TFLFDQKNKIFAATNK--------ELLNPSIDHSPVL 241

IA3901_Tlp3b IPSEDLQNLVAKTPGN-------TFLFDQKNKIFAATNK--------ELLNPSIDHSPVL 230

00-6200_Tlp3b IPSEDLQNLVAKTPGN-------TFLFDQKNKIFAATNK--------ELLNPSIDHSPVL 230

BCW_6290_Tlp3b IPSEDLQNLVAKTPGN-------TFLFDQKNKIFAATNK--------ELLNPSIDHSPVL 230

00-2425_Tlp3a IPSEDLQNLVAKTPGN-------TFLFDQKNKIFAATNK--------ELLNPSIDHSPVL 230

00-2425_Tlp3b IPSEDLQNLVAKTPGN-------TFLFDQKNKIFAATNK--------ELLNPSIDHSPVL 230

YH001_Tlp3a IPSEDLQNLVAKTPGN-------TFLFDQKNKIFAATNK--------ELLNPSIDHSPVL 230

YH001_Tlp3b IPSEDLQNLVAKTPGN-------TFLFDQKNKIFAATNK--------ELLNPSIDHSPVL 230

00-0949_Tlp3 IPSEDLQNLVAKTPGN-------TFLFDQKNKIFAATNK--------ELLNPSIDHSPVL 230

NCTC11168_Tlp3 IPSEDLQNLVAKTPGN-------TFLFDQKNKIFAATNK--------ELLNPSIDHSPVL 241

F38011_Tlp3 IPSEDLQNLVAKTPGN-------TFLFDQKNKIFAATNK--------ELLNPSIDHSPVL 230

RM1285_Tlp3 IPSEDLQNLVAKTPGN-------TFLFDQKNKIFAATNK--------ELLNPSIDHSPVL 230

FDAARGOS_422_Tlp3 IPSEDLQNLVAKTPGN-------TFLFDQKNKIFAATNK--------ELLNPSIDHSPVL 241

MTVDSCj07_Tlp3 IPSEDLQNLVAKTPGN-------TFLFDQKNKIFAATNK--------ELLNPSIDHSPVL 230

IA3901_Tlp3a IPSEDLQNLVAKTPGN-------TFLFDQKNKIFAATNK--------ELLNPSIDHSPVL 230

BCW_6290_Tlp3a IPSEDLQNLVAKTPGN-------TFLFDQKNKIFAATNK--------ELLNPSIDHSPVL 230

35925B2_Tlp3 IPSEDLQNLVAKTPGN-------TFLFDQKNKIFAATNK--------ELLNPSIDHSPVL 241

14980A_Tlp3 IPLEDLQNSVANTPGN-------IFLFDQKNKIFAATNK--------ELLNPSIDHSPVL 241

00-1597_Tlp3b IPSGDLQNLVAKTPGN-------TFLFDQKNKIFAATNK--------ELLNPSIDHSPVL 230

RM3196_Tlp3 IPSEDLQNLVAKTPGN-------TFLFDQKNKIFAATNK--------ELLNPSIDHSPVS 230

FDAARGOS_295_Tlp21 NLANYAKD-VL-SVGR--SQNTYTAIYDPQGTILFHEKTELMLQKNTLSTN-------IT 225

FORC_046_Tlp4 DLANFSTN-VL-TVGK--SDNTFTEVLDSEGTILFNDEVAKILTKTELSIN-------IA 234

FDAARGOS_422_Tlp4 DLANFSTN-VL-TVGK--SDNTFTEVLDSEGTILFNDEVAKILTKTELSIN-------IA 234

ICDCCJ07001_Tlp4 DLAKFSTD-VL-AVGK--SQNTYTVVLDPEGTILFRDDITKILTKTELSIN-------IA 234

RM3196_Tlp4 DLAKFSTD-VL-AVGK--SQNTYTVVLDPEGTILFRDDITKILTKTELSIN-------IA 234

T1-21_Tlp4 DLAKFSTD-VL-AVGK--SQNTYTVVLDPEGTILFRDDITKILTKTELSIN-------IA 234

F38011_Tlp4 DLANFSTN-VL-TVGK--SDNTFTEVLDSEGTILFNDEVAKILTKTELSIN-------IA 234

HF5-4A-4_Tlp22 DLANFSTN-VL-TVGK--SDNTFTEVLDSEGTILFNDEVAKILTKTELSIN-------IA 180

00-0949_Tlp4 DLANFSTN-VL-TVGK--SDNTFTEVLDSEGTILFNDEVAKILTKTELSIN-------IA 234

01-1512_Tlp4 DLANFSTN-VL-TVGK--SDNTFTEVLDSEGTILFNDEVAKILTKTELSIN-------IA 234

81-176_Tlp4 DLANFSTN-VL-TVGK--SDNTFTEVLDSEGTILFNDEVAKILTKTELSIN-------IA 234

32488_Tlp4 DLANFSTN-VL-TVGK--SDNTFTEVLDSEGTILFNDEVAKILTKTELSIN-------IA 234

NCTC11168_Tlp4 DLANFSTN-VL-TVGK--SDNTFTEVLDSEGTILFNDEVAKILTKTELSIN-------IA 234

CFSAN032806_Tlp4 DLANFSTN-VL-TVGK--SDNTFTEVLDSEGTILFNDEVAKILTKTELSIN-------IA 234

81116_Tlp4 DLANFSTN-VL-TVGK--SDNTFTEVLDSEGTILFNDEVAKILTKTELSIN-------IA 234

RM1285_Tlp12 NLDKFSKD-VL-SLGH--SSTTYAAVYDSEGRIIFHEVLDRILTKNTLSVN-------IA 231

PT14_Tlp12 NLDKFSKD-VL-SLGH--SSTTYAAVYDSEGRIIFHEVLDRILTKNTLSVN-------IA 231

MTVJDCj07_Tlp12 NLDKFSKD-VL-SLGH--SSTTYAAVYDSEGRIIFHEVLDRILTKNTLSVN-------IA 231

RM1221_Tlp12 NLDKFSKD-VL-SLGH--SSTTYAAVYDSEGRIIFHEVLDRILTKNTLSVN-------IA 231

FDAARGOS_421_Tlp12 NLDKFSKD-VL-SLGH--SSTTYAAVYDSEGRIIFHEVLDRILTKNTLSVN-------IA 231

35925B2_Tlp12 NLDKFSKD-VL-SLGH--SSTTYAAVYDSEGRIIFHEVLDRILTKNTLSIN-------IA 231

CJM1cam_Tlp12 NLDKFSKD-VL-SLGH--SSTTYAAVYDSEGRIIFHEVLDRILTKNTLSVN-------IA 231

M1_Tlp12 NLDKFSKD-VL-SLGH--SSTTYAAVYDSEGRIIFHEVLDRILTKNTLSVN-------IA 231

S3_Tlp12 NLDKFSKD-VL-SLGH--SSTTYAAVYDSEGRIIFHEVLDRILTKNTLSVN-------IA 231

00-1597_Tlp12 NLDKFSKD-VL-SLGH--SSTTYAAVYDSEGRIIFHEVLDRILTKNTLSVN-------IA 231

R14_Tlp12 NLDKFSKD-VL-SLGH--SSTTYAAVYDSEGRIIFHEVLDRILTKNTLSVN-------IA 231

RM1875_Tlp15 ------------------------------------------------------------ 168

YH503_Tlp16 ------------------------------------------------------------ 168

FB1_Tlp16 ------------------------------------------------------------ 168

BFR-CA-9557_Tlp16 ------------------------------------------------------------ 167

15-537360_Tlp16 ------------------------------------------------------------ 168

OR12_Tlp16 ------------------------------------------------------------ 168

YH502_Tlp16 ------------------------------------------------------------ 168

14903A_Tlp16 ------------------------------------------------------------ 168

RM5611_Tlp16 ------------------------------------------------------------ 168

00-2425_Tlp11 DFLEISET-IL-DPKLDFYKDDLRFLITDQGVIVIHKNKDAILKTL-PEINQDASVQLII 277

00-6200_Tlp11 DFLEISET-IL-DPKLDFYKDDLRFLITDQGVIVIHKNKDAILKTL-PEINQDASVQLII 277

YH001_Tlp11 DFLEISET-IL-DPKLDFYKDDLRFLITDQGVIVIHKNKDAILKTL-PEINQDASVQLII 277

IA3902_Tlp11 DFLEISET-IL-DPKLDFYKDDLRFLITDQGVIVIHKNKDAILKTL-PEINQDASVQLII 277

BCW_6290_Tlp11 DFLEISET-IL-DPKLDFYKDDLRFLITDQGVIVIHKNKDAILKTL-PEINQDASVQLII 277

76339_Tlp18 DFLEISQA-ML-DPKLDFFEGDLRALTTDQGVITIHKDKNAILKTL-TDINKDPSVKLIT 276

4031_Tlp17 DFSEVSET-IL-DPKLDFFEGDLRFLMTDKGVITIHKNHNAILKTL-GDINKDPSVELVN 277

15-537360_Tlp13 DLLEISET-IL-DPKFDFFEGDLRFLMNDQGIIAIHKNKNAILKTL-FDINKDQSAQLIV 276

CVM_N29710_Tlp13 DLLEISET-IL-DPKFDFFEGDLRFLMNDQGIIAIHKNKNAILKTL-FDINKDQSAQLIV 276

FB1_Tlp13 DLLEISET-IL-DPKFDFFEGDLRFLMNDQGIIAIHKNKNAILKTL-FDINKDQSAQLIV 276

CFCAN032805_Tlp13 DLLEISET-IL-DPKFDFFEGDLRFLMNDQGIIAIHKNKNAILKTL-FDINKDQSAQLIV 276

BG2108_Tlp13 DLLEISET-IL-DPKFDFFEGDLRFLMNDQGIIAIHKNKNAILKTL-FDINKDQSAQLIV 276

YF2105_Tlp13 DLLEISET-IL-DPKFDFFEGDLRFLMNDQGIIAIHKNKNAILKTL-FDINKDQSAQLIV 276

YH503_Tlp13 DLLEISET-IL-DPKFDFFEGDLRFLMNDQGIIAIHKNKNAILKTL-FDINKDQSAQLIV 276

BFRCA9557_Tlp13 DLLEISET-IL-DPKFDFFEGDLRFLMNDQGIIAIHKNKNAILKTL-FDINKDQSAQLIV 276

YH502_Tlp13 DLLEISET-IL-DPKFDFFEGDLRFLMNDQGIIAIHKNKNAILKTL-FDINKDQSAQLIV 276

MTVDSCj13_Tlp13 DLLEISEI-IL-DPKFDFFEGDLRILMNDQGIIAVHKNKNGILKTL-FDINKDQSAQLIV 276

OR12_Tlp13 DLLEISET-IL-DPKFDFFEGDLRFLMNDQGIIAIHKNKNAILKTL-FDINKDQSAQLIV 276

00-1597_Tlp13 DLLEISET-IL-DPKFDFFEGDLRFLMNDQGIIAIHKNKNAILKTL-FDINKDQSAQLIV 276

14903A_Tlp13 DLLEISET-IL-DPKFDFFEGDLRFLMNDQGIIAIHKNKNAILKTL-FDINKDQSAQLIV 276

FJ3124_Tlp13 DLLEISET-IL-DPKFDFFEGDLRFLMNDQGIIAIHKNKNAILKTL-FDINKDQSAQLIV 276

R14_Tlp13 DLLEISET-IL-DPKFDFFEGDLRFLMNDQGIIAIHKNKNAILKTL-FDINKDQSAQLIV 276

MTVDSCj16_Tlp13 DLLEISET-IL-DPKFDFFEGDLRFLMNDQGIIAIHKNKNAILKTL-FDINKDQSAQLIV 276

14980A_Tlp13 DLLEISET-IL-DPKFDFFEGDLRFLMNDQGIIAIHKNKNAILKTL-FDINKDQSAQLIV 276

15-537360_Tlp20 NIAKDKKDYEPFRYARP--------------------LDGTQR-----FGVCAKVL-GEY 269

76339_Tlp20 DIAKDKKDYEPFHYIRP--------------------LDGTER-----FGVCAKVL-GEY 269

CFSAN032805_Tlp20 NIAKDKKDYEPFRYVRP--------------------LDGTQR-----FGVCAKVL-GEY 268

CVM_N29710_Tlp20 NIAKDKKDYEPFRYVRP--------------------LDGTQR-----FGVCAKVL-GEY 267

YH501_Tlp20 NIAKDKKDYEPFRYVRP--------------------LDGTQR-----FGVCAKVL-GEY 271

CO2-160_Tlp20 NIAKDKKDYEPFRYVRP--------------------LDGTQR-----FGVCAKVL-GEY 269

CO2-160_Tlp20b NIAKDKKDYEPFRYVRP--------------------LDGTQR-----FGVCAKVL-GEY 269

RM5611_Tlp20 NIAKDKKDYEPFRYVRP--------------------LDGTQR-----FGVCAKVL-GEY 269

14903A_Tlp20 NIAKDKKDYEPFRYVRP--------------------LDGTQR-----FGVCAKVL-GEY 271

YH502_Tlp20 NIAKDKKDYEPFRYVRP--------------------LDGTQR-----FGVCAKVL-GEY 268

RM3196_Tlp23 NLAKTKKDFEPFEYTRP--------------------KDGSER-----FAVCVKVS-GIY 269

ICDCCJ07001_Tlp23 NLAKTKKDFEPFEYTRP--------------------KDGSER-----FAVCVKVS-GIY 269

RM1285_Tlp2 NLAKTKEDLEPFEYTRP--------------------KDGNER-----FAVCTKVS-GIY 269

CFSAN032806_Tlp2 NLAKTKEDLEPFEYTRP--------------------KDGNER-----FAVCTKVS-GIY 269

RM1221_Tlp2 NLAKTKEDLEPFEYTRP--------------------KDGNER-----FAVCTKVS-GIY 269

S3_Tlp2 NLAKTKEDLEPFEYTRP--------------------KDGNER-----FAVCTKVS-GIY 269

FDAARGOS_422_Tlp2 NLAKTKEDLEPFEYTRP--------------------KDGNER-----FAVCTKVS-GIY 269

81-176_Tlp2 NLAKTKEDLEPFEYTRP--------------------KDGNER-----FAVCTKVS-GIY 269

F38011_Tlp2 NLAKTKEDLEPFEYTRP--------------------KDGNER-----FAVCTKVS-GIY 269

NCTC11168_Tlp2 NLAKTKEDLEPFEYTRP--------------------KDGNER-----FAVCTKVS-GIY 269

MTVDSCj07_Tlp2 NLAKTKEDLEPFEYTRP--------------------KDGNER-----FAVCTKVS-GIY 269

CJM1cam_Tlp24 NLAKIKENFEPFEYTRP--------------------KDGSER-----FAVCTKVS-GVY 269

M1_Tlp24 NLAKIKENFEPFEYTRP--------------------KDGSER-----FAVCTKVS-GVY 269

81116_Tlp2 NLAKIKENFEPFEYTRP--------------------KDGSER-----FAVCTKVS-GVY 269

4031_Tlp23 NLAKIKENFEPFEYTRP--------------------KDGSER-----FAVCTKVS-GAY 268

MG1116_Tlp14 NAYKAHGDNNFFSYK----------------------LNNEER-----LGVCTKVF--AY 266

YH502_Tlp14 NAYKTHGDYNFFTYG----------------------LDGKER-----LGACTKVF--AY 277

BP3181_Tlp14 NAYKAHGDNNFFSYK----------------------LNNEER-----LGVCTKVF--AY 266

ZV1224_Tlp14a NAYKAHGDNNFFSYK----------------------LNNEER-----LGVCTKVF--AY 266

ZV1224_Tlp14b NAYKAHGDNNFFSYK----------------------LNNEER-----LGVCTKVF--AY 266

YH503_Tlp14 NAYKAHGDNNFFSYK----------------------LNNEER-----LGACTKAF--AY 277

14903A_Tlp14 NAYKAHGDNNFFSYK----------------------LNNEER-----LGVCTKVF--AY 266

OR12_Tlp14 NAYKAHGDNNFFSYK----------------------LNNEER-----LGTCTKVF--AY 266

CFSAN032805_Tlp14 NAYKAHGDNNFFSYK----------------------LNNEER-----LGTCTKVF--AY 277

BFR-CA-9557_Tlp14 NAYKAHGDNNFFSYK----------------------LNNEER-----LGTCTKVF--AY 266

15-537360_Tlp14 NAYKAHGDNNFFSYK----------------------LNNEER-----LGTCTKVF--AY 266

YH501_Tlp14 NAYKAHGDNNFFSYK----------------------LNNEER-----LGTCTKVF--AY 266

CG8421_Tlp14 NAYKAHGDNNFFSYK----------------------LNNEER-----LGACTKVF--AY 266

MTVDSCj16_Tlp14 NAYKAHGDNNFFSYK----------------------LNNEER-----LGACTKVF--AY 266

01-1512_Tlp14 NAYKAHGDNNFFSYK----------------------LNNEER-----LGACTKVF--AY 266

00-0949_Tlp14 NAYKAHGDNNFFSYK----------------------LNNEER-----LGACTKVF--AY 266

MTVDSCj13_Tlp14 NAYKAHGDNNFFSYK----------------------LNNEER-----LGACTKVF--AY 266

S3_Tlp14 NAYKAHGDNNFFSYK----------------------LNNEER-----LGACTKVF--AY 266

PT14_Tlp14 NAYKAHGDNNFFSYK----------------------LNNEER-----LGACTKVF--AY 266

14980A_Tlp14 NAYKAHGDNNFFSYK----------------------LNNEER-----LGACTKVF--AY 282

FJ3124_Tlp14 NAYKAHGDNNFFSYK----------------------LNNEER-----LGACTKVF--AY 266

00-1597_Tlp14 NAYKAHGDNNFFSYK----------------------LNNEER-----LGACTKVF--AY 266

R14_Tlp14 NAYKTHGDYNFFTYG----------------------LDGKER-----LGACTKVF--AY 266

CG8421_Tlp25 --------------E----------------------LLKENQ-----LKTMDDVL--MI 61

RM1875_Tlp3 NAYKTHGDHNFFNYG----------------------LDGKER-----LGACTKVF--AY 261

CJM1cam_Tlp3 NAYKTHGDYNFFTYG----------------------LDGKER-----LGTCTKVF--AY 261

M1_Tlp3 NAYKTHGDYNFFTYG----------------------LDGKER-----LGTCTKVF--AY 261

4031_Tlp3 NAYKTHGDYNFFTYG----------------------LDGKER-----LGTCTKVF--AY 261

R14_Tlp3 NAYKTHGDYNFFTYG----------------------LDGKER-----LGTCTKVF--AY 261

RM5611_Tlp3 NAYKLNGDNNFFSYK----------------------LNNEER-----LGACTKVF--AY 261

MTVDSCj16_Tlp3 NAYKAHGDNNFFSYK----------------------LNNEER-----LGACTKVF--AY 261

01-1512_Tlp3 NAYKLNGDNNFFSYK----------------------LNNEER-----LGACTKVF--AY 261

MTVDSCj13_Tlp3 NAYKLNGDNNFFSYK----------------------LNNEER-----LGACTKVF--AY 261

32488_Tlp3a NAYKLNGDNNFFSYK----------------------LNNEER-----LGACTKVF--AY 261

81116_Tlp3 NAYKLNGDNNFFSYK----------------------LNNEER-----LGACTKVF--AY 261

32488_Tlp3b NAYKLNGDNNFFSYK----------------------LNNEER-----LGACTKVF--AY 261

FB1_Tlp3 NAYKTHGDNNFFSYK----------------------LNNEER-----LGACTKVF--AY 261

PT14_Tlp3 NAYKAHGDNNFFSYK----------------------LNNEER-----LGACTKVF--AY 261

00-6200_Tlp3a NAYKLNGDNNFFSYK----------------------LNNEER-----LGACTKVF--AY 261

RM1221_Tlp3 NAYKLNGDNNFFSYK----------------------LNNEER-----LGACTKVF--AY 261

S3_Tlp3 NAYKLNGDNNFFSYK----------------------LNNEER-----LGACTKVF--AY 261

FDAARGOS_421_Tlp3 NAYKLNGDNNFFSYK----------------------LNNEER-----LGACTKVF--AY 272

CFSAN032806_Tlp3 NAYKLNGDNNFFSYK----------------------LNNEER-----LGACTKVF--AY 272

IA3901_Tlp3b NAYKLNGDNNFFSYK----------------------LNNEER-----LGACTKVF--AY 261

00-6200_Tlp3b NAYKLNGDNNFFSYK----------------------LNNEER-----LGACTKVF--AY 261

BCW_6290_Tlp3b NAYKLNGDNNFFSYK----------------------LNNEER-----LGACTKVF--AY 261

00-2425_Tlp3a NAYKLNGDNNFFSYK----------------------LNNEER-----LGACTKVF--AY 261

00-2425_Tlp3b NAYKLNGDNNFFSYK----------------------LNNEER-----LGACTKVF--AY 261

YH001_Tlp3a NAYKLNGDNNFFSYK----------------------LNNEER-----LGACTKVF--AY 261

YH001_Tlp3b NAYKLNGDNNFFSYK----------------------LNNEER-----LGACTKVF--AY 261

00-0949_Tlp3 NAYKLNGDNNFFSYK----------------------LNNEER-----LGACTKVF--AY 261

NCTC11168_Tlp3 NAYKLNGDNNFFSYK----------------------LNNEER-----LGACTKVF--AY 272

F38011_Tlp3 NAYKLNGDNNFFSYK----------------------LNNEER-----LGACTKVF--AY 261

RM1285_Tlp3 NAYKLNGDNNFFSYK----------------------LNNEER-----LGACTKVF--AY 261

FDAARGOS_422_Tlp3 NAYKLNGDNNFFSYK----------------------LNNEER-----LGACTKVF--AY 272

MTVDSCj07_Tlp3 NAYKLNGDNNFFSYK----------------------LNNEER-----LGACTKVF--AY 261

IA3901_Tlp3a NAYKLNGDNNFFSYK----------------------LNNEER-----LGACTKVF--AY 261

BCW_6290_Tlp3a NAYKLNGDNNFFSYK----------------------LNNEER-----LGACTKVF--AY 261

35925B2_Tlp3 NAYKTHGDYNFFTYG----------------------LDGKER-----LGTCAKVF--AY 272

14980A_Tlp3 NAYKTHGDYNFFTYG----------------------LDGKER-----LGTCTKVF--AY 272

00-1597_Tlp3b NAYKAHGDNNFFSYK----------------------LNNEER-----LGACTKVF--AY 261

RM3196_Tlp3 NAYKAHGDNNFFSYK----------------------LNNEER-----LGACTKVF--AY 261

FDAARGOS_295_Tlp21 KIINQNTALLNAK---TPFVVDNGEGEQYEAF-------------------CRNVVSDFY 263

FORC_046_Tlp4 NAIKANPALIDPRNQDTLFTAKDHQGVDYAIM-------------------CNSAFNPLF 275

FDAARGOS_422_Tlp4 NAIKANPALIDPRNQDTLFTAKDHQGVDYAIM-------------------CNSAFNPLF 275

ICDCCJ07001_Tlp4 NAIKANPALIDPRNQDTLFTAKDHQGVDYAIM-------------------CNSAFNPLF 275

RM3196_Tlp4 NAIKANPALIDPRNQDTLFTAKDHQGVDYAIM-------------------CNSAFNPLF 275

T1-21_Tlp4 NAIKANPALIDPRNQDTLFTAKDHQGVDYAIM-------------------CNSAFNPLF 275

F38011_Tlp4 NAIKANPALIDPRNQDTLFTAKDHQGVDYAIM-------------------CNSAFNPLF 275

HF5-4A-4_Tlp22 NAIKANPALIDPRNQDTLFTAKDHQGVDYAIM-------------------CNSAFNPLF 221

00-0949_Tlp4 NAIKANPALIDPRNQDTLFTAKDHQGVDYAIM-------------------CNSAFNPLF 275

01-1512_Tlp4 NAIKANPALIDPRNQDTLFTAKDHQGVDYAIM-------------------CNSAFNPLF 275

81-176_Tlp4 NAIKANPALIDPRNQDTLFTAKDHQGVDYAIM-------------------CNSAFNPLF 275

32488_Tlp4 NAIKANPALIDPRNQDTLFTAKDHQGVDYAIM-------------------CNSAFNPLF 275

NCTC11168_Tlp4 NAIKANPALIDPRNQDTLFTAKDHQGVDYAIM-------------------CNSAFNPLF 275

CFSAN032806_Tlp4 NAIKANPALIDPRNQDTLFTAKDHQGVDYAIM-------------------CNSAFNPLF 275

81116_Tlp4 NAIKANPALIDPRNQDTLFTAKDHQGVDYAIM-------------------CNSAFNPLF 275

RM1285_Tlp12 NAIKENPEYIDLNKRDILFPVFDDKGIKYEAM-------------------CDTSSNGLY 272

PT14_Tlp12 NAIKENPEYIDLNKRDILFPVFDDKGIKYEAM-------------------CDTSSNGLY 272

MTVJDCj07_Tlp12 NAIKENPEYIDLNKRDILFPVFDDKGIKYEAM-------------------CDTSSNGLY 272

RM1221_Tlp12 NAIKENPEYIDPNKRDILFPVFDDKGIKYETM-------------------CDTSSNGLY 272

FDAARGOS_421_Tlp12 NAIKENPEYIDPNKRDILFPVFDDKGIKYETM-------------------CDTSSNGLY 272

35925B2_Tlp12 NAIKENPKYIDLNKRDILFPVFDDKGIKYETM-------------------CDTSSNGLY 272

CJM1cam_Tlp12 NTIKENPEYIDLNKRDILFPVFDDKGIKYEAM-------------------CDTSSNGLY 272

M1_Tlp12 NTIKENPEYIDLNKRDILFPVFDDKGIKYEAM-------------------CDTSSNGLY 272

S3_Tlp12 NAIKENPEYIDPNKRDILFPVFDDKGIKYETM-------------------CDTSSNGLY 272

00-1597_Tlp12 NAIKENPEYIDPNKRDILFPVFDDKGIKYETM-------------------CDTSSNGLY 272

R14_Tlp12 NAIKENPEYIDPNKRDILFPVFDDKGIKYETM-------------------CDTSSNGLY 272

RM1875_Tlp15 ---------------------------------SYLIPNADDLWIS--TGVYKDTLEPYI 193

YH503_Tlp16 ---------------------------------SYLIPNADDLWIS--TGVYKDTLEPYI 193

FB1_Tlp16 ---------------------------------SYLIPNADDLWIS--TGVYKDTLEPYI 193

BFR-CA-9557_Tlp16 ---------------------------------SYLIPNADDLWIS--TGVYKDTLEPYI 192

15-537360_Tlp16 ---------------------------------SYLIPNADDLWIS--TGVYKDTLEPYI 193

OR12_Tlp16 ---------------------------------SYLIPNADDLWIS--TGVYKDTLEPYI 193

YH502_Tlp16 ---------------------------------SYLIPNADDLWIS--TGVYKDTLEPYI 193

14903A_Tlp16 ---------------------------------SYLIPNTDDLWIS--TGVYKDTLEPYI 193

RM5611_Tlp16 ---------------------------------SYLIPNADDLWIS--TGVYKDTLEPYI 193

00-2425_Tlp11 DAVKNHKDLIIDNY------VDLSGNLSYAGVASFSTLGDSSHWSMVVTAPKKSIFAPL- 330

00-6200_Tlp11 DAVKNHKDLIIDNY------VDLSGNLSYAGVASFSTLGDSSHWSMVVTAPKKSIFAPL- 330

YH001_Tlp11 DAVKNHKDLIIDNY------VDLSGNLSYAGVASFSTLGDSSHWSMVVTAPKKSIFAPL- 330

IA3902_Tlp11 DAVKNHKDLIIDNY------VDLSGNLSYAGVASFSTLGDSSHWSMVVTAPKKSIFAPL- 330

BCW_6290_Tlp11 DAVKNHKDLIIDNY------VDLSGNLSYAGVASFSTLGDSSHWSMVVTAPKKSIFAPL- 330

76339_Tlp18 DLIKEHKDALIDNY------VASTGDLSYASVVSFNTLGDSSRWSMIVTAPKKSALEPL- 329

4031_Tlp17 NAVKEHKTVIIDDY------VASTGDLSYASVSSFSTANNSSHWSMVVTAPKNSVLAPL- 330

15-537360_Tlp13 EAVKNHKDEILDNY------IASTGDLSYASISSFSTLGNSSHWSVIVTAPKKSVLAPL- 329

CVM_N29710_Tlp13 EAVKNHKDEILDNY------IASTGDLSYASISSFSTLGNSSHWSVIVTAPKKSVLAPL- 329

FB1_Tlp13 EAVKNHKDEILDNY------IASTGDLSYASISSFSTLGNSSHWSVIVTAPKKSVLAPL- 329

CFCAN032805_Tlp13 EAVKNHKDEILDNY------IASTGDLSYASISSFSTLGNSSHWSVIVTAPKKSVLAPL- 329

BG2108_Tlp13 EAVKNHKDEILDNY------IASTGDLSYASISSFSTLGNSSHWSVIVTAPKKSVLAPL- 329

YF2105_Tlp13 EAVKNHKDEILDNY------IASTGDLSYASISSFSTLGNSSHWSVIVTAPKKSVLAPL- 329

YH503_Tlp13 EAVKNHKDEILDNY------IASTGDLSYASISSFSTLGNSSHWSVIVTAPKKSVLAPL- 329

BFRCA9557_Tlp13 EAVKNHKDEILDNY------IASTGDLSYASISSFSTLGNSSHWSVIVTAPKKSVLAPL- 329

YH502_Tlp13 EAVKNHKDEILDNY------IASTGDLSYASISSFSTLGNSSHWSVIVTAPKKSVLAPL- 329

MTVDSCj13_Tlp13 EAVKNHKDEILDNY------IASTGDLSYASISSFSTLGNSSHWSVIVTAPKKSVLAPL- 329

OR12_Tlp13 EAVKNHKDEILDNY------IASTGDLSYASISSFSTLGNSSHWSVIVTAPKKSVLAPL- 329

00-1597_Tlp13 EAVKNHKDEILDNY------IASTGDLSYASISSFSTLGNSSHWSVIVTAPKKSVLAPL- 329

14903A_Tlp13 EAVKNHKDEILDNY------IASTGDLSYASISSFSTLGNSSHWSVIVTAPKKSVLAPL- 329

FJ3124_Tlp13 EAVKNHKDEILDNY------IASTGDLSYASISSFSTLGNSSHWSVIVTAPKKSVLAPL- 329

R14_Tlp13 EAVKNHKDEILDNY------IASTGDLSYASISSFSTLGNSSHWSVIVTAPKKSVLAPL- 329

MTVDSCj16_Tlp13 EAVKNHKDEILDNY------IASTGDLSYASISSFSTLGNSSHWSVIVTAPKKSVLAPL- 329

14980A_Tlp13 EAVKNHKDEILDNY------IASTGDLSYASISSFSTLGNSSHWSVIVTAPKKSVLAPL- 329

15-537360_Tlp20 TACVGEPIDYIEEPVFKIAYIQIAIVIITSIISVLLLYFIVSR-YLSPLAAIQTGLTSFF 328

76339_Tlp20 TACVGEPIDYIEEPVFKIAYIQIAIVIITSIISVLLLYFIVSR-YLSPLASIQAGLNSFF 328

CFSAN032805_Tlp20 TACVGEPIDYIEEPVFKIAYIQIAIVIITSIISVLLLYFIVSR-YLSPLAAIQTGLTSFF 327

CVM_N29710_Tlp20 TACVGEPIDYIEEPVFKIAYIQIAIVIITSIISVLLLYFIVSR-YLSPLAAIQTGLTSFF 326

YH501_Tlp20 TACVGEPIDYIEEPVFKIAYIQIAIVIITSIISVLLLYFIVSR-YLSPLAAIQTGLTSFF 330

CO2-160_Tlp20 TACVGESIDYIEEPVFKIAYIQIAIVIITSIISVLLLYFIVSR-YLSPLASIQVGLNSFF 328

CO2-160_Tlp20b TACVGESIDYIEEPVFKIAYIQIAIVIITSIISVLLLYFIVSR-YLSPLASIQVGLNSFF 328

RM5611_Tlp20 TACVGESIDYIEEPVFKIAYIQIAIVIITSIISVLLLYFIVSR-YLSPLASIQVGLNSFF 328

14903A_Tlp20 TACVGEPIDYIEEPVFKIAYIQIAIVIITSIISVLLLYFIVSR-YLSPLASIQVGLNSFF 330

YH502_Tlp20 TACVGEPIDYIEEPVFKIAYIQIAIVIITSIISVLLLYFIVSR-YLSPLASIQVGLNSFF 327

RM3196_Tlp23 TACVAKPIEQIEAPVYKAAFIQAIVVIIVVVFSVILLYFIVSK-YLSPLAAIQTGLTSFF 328

ICDCCJ07001_Tlp23 TACVAKPIEQIEAPVYKAAFIQAIVVIIVVVFSVILLYFIVIK-YLSPLAAIQTGLTSFF 328

RM1285_Tlp2 TACVGEPIEQIEAPVYKIAFIQTAIVIFTSIISVILLYFIVSK-YLSPLAAIQTGLTSFF 328

CFSAN032806_Tlp2 TACVGEPIEQIEAPVYKIAFIQTVIVVFASILSVILLYFIVSK-YLSPLAAIQTGLTSFF 328

RM1221_Tlp2 TACVGEPIEQIEAPVYKIAFIQTVIVVFASILSVILLYFIVSK-YLSPLAAIQTGLTSFF 328

S3_Tlp2 TACVGEPIEQIEAPVYKIAFIQTVIVVFASILSVILLYFIVSK-YLSPLAAIQTGLTSFF 328

FDAARGOS_422_Tlp2 TACVGEPIEQIEAPVYKIAFIQTAIVIFTSIISVILLYFIVSK-YLSPLAAIQTGLTSFF 328

81-176_Tlp2 TACVGEPIEQIEAPVYKIAFIQTAIVIFTSIISVILLYFIVSK-YLSPLAAIQTGLTSFF 328

F38011_Tlp2 TACVGEPIEQIEAPVYKIAFIQTAIVIFTSIISVILLYFIVSK-YLSPLAAIQTGLTSFF 328

NCTC11168_Tlp2 TACVGEPIEQIEAPVYKIAFIQTAIVIFTSIISVILLYFIVSK-YLSPLAAIQTGLTSFF 328

MTVDSCj07_Tlp2 TACVGEPIEQIEAPVYKIAFIQTAIVIFTSIISVILLYFIVSK-YLSPLAAIQTGLTSFF 328

CJM1cam_Tlp24 TACVGEPIEQIEAPVYKIAFIQTAIVIFTSIISVILLYFIVSK-YLSPLAAIQTGLTSFF 328

M1_Tlp24 TACVGEPIEQIEAPVYKIAFIQTAIVIFTSIISVILLYFIVSK-YLSPLAAIQTGLTSFF 328

81116_Tlp2 TACVGEPIEQIEAPVYKIAFIQTAIVIFTSIISVILLYFIVSK-YLSPLAAIQTGLTSFF 328

4031_Tlp23 TACVGEPIEQIEAPVYKIAFIQAIVVIIVVVFSVILLYFIVSK-YLSPLAAIQTGLTSFF 327

MG1116_Tlp14 TACITESTDVINKPIFKAAYIQVIALIVMISISIILLYFIVSK-YLSPLATI-------- 317

YH502_Tlp14 TACITESADIINKPIFKAAYIQVIALIVMISISIILLYFIVSK-YLSPLAAIQTGLTSFF 336

BP3181_Tlp14 TACITESADIINKPIFKAAYIQVIALIVMISISIILLYFIVSK-YLSPLAAIQTGLTSFF 325

ZV1224_Tlp14a TACITESTDVINKPIFKAAYIQVIALIIMISISIILLYFIVSK-YLSPLAAIQTGLTSFF 325

ZV1224_Tlp14b TACITESTDVINKPIFKAAYIQVIALIIMISISIILLYFIVSK-YLSPLAAIQTGLTSFF 325

YH503_Tlp14 TACITESTDVINKPIFKAAYIQVIALIIMISISIILLYFIVSK-YLSPLAAIQTGLTSFF 336

14903A_Tlp14 TACITESTDVINKPIFKAAYIQVIALIVMISISIILLYFIVSK-YLSPLAAIQTGLTSFF 325

OR12_Tlp14 TACITESTDVINKPIFKAAYIQVIALIIMISISIILLYFIVSK-YLSPLAAIQTGLTSFF 325

CFSAN032805_Tlp14 TACITESTDVINKPIFKAAYIQVIALIVMISISIILLYFIVSK-YLSPLAAIQTGLTSFF 336

BFR-CA-9557_Tlp14 TACITESTDVINKPIFKAAYIQVIALIIMISISIILLYFIVSK-YLSPLAAIQTGLTSFF 325

15-537360_Tlp14 TACITESTDVINKPIFKAAYIQVIALIVMISISIILLYFIVSK-YLSPLAAIQTGLTSFF 325

YH501_Tlp14 TACITESTDVINKPIFKAAYIQVIALIIMISISIILLYFIVSK-YLSPLAAIQTGLTSFF 325

CG8421_Tlp14 TACITESADIINKPIFKAAYIQVIALIVMISISI------VSK-YLSPLAAIQTGLTSFF 319

MTVDSCj16_Tlp14 TACITESADIINKPIYKAAFIQVIALIVMISISIILLYFIVSK-YLSPLAAIQTGLTSFF 325

01-1512_Tlp14 TACITESADIINKPIYKAAFIQVIALIVMISISIILLYFIVSK-YLSPLAAIQTGLTSFF 325

00-0949_Tlp14 TACITESADIINKPIYKAAFIQVIALIVMISISIILLYFIVSK-YLSPLAAIQTGLTSFF 325

MTVDSCj13_Tlp14 TACITESADIINKPIFKAAYIQVIALIVMISISIILLYFIVSK-YLSPLAAIQTGLTSFF 325

S3_Tlp14 TACITESADIINKPIFKAAYIQVIALIVMISISIILLYFIVSK-YLSPLAAIQTGLTSFF 325

PT14_Tlp14 TACITESADIINKPIFKAAYIQVIALIVMISISIILLYFIVSK-YLSPLAAIQTGLTSFF 325

14980A_Tlp14 TACITESADIINKPIFKAAYIQVIALIVMISISIILLYFIVSK-YLSPLAAIQTGLTSFF 341

FJ3124_Tlp14 TACITESADIINKPIFKAAYIQVIALIVMISISIILLYFIVSK-YLSPLAAIQTGLTSFF 325

00-1597_Tlp14 TACITESTDVINKPIFKAAYIQVIALIIMISISIILLYFIVSK-YLSPLAAIQTGLTSFF 325

R14_Tlp14 TACITESADIINKPIFKAAYIQVIALIVMISISIILLYFIVSK-YLSPLAAIQTGLTSFF 325

CG8421_Tlp25 ----NIKRFRYKKSCYSILMKQVIVGIIAIIIALILIRFLISR-SLSPLAAIQTGLTSFF 116

RM1875_Tlp3 TACITESADIINKPIYKAAFIQAIVVIIVVVFSVILLYFIVSK-YLSPLAAIQTGLTSFF 320

CJM1cam_Tlp3 TACITESADIINKPIHKAAFIQAIVVIIVVVFSVILLYFIVSK-YLSPLAAIQTGLTSFF 320

M1_Tlp3 TACITESADIINKPIHKAAFIQAIVVIIVVVFSVILLYFIVSK-YLSPLAAIQTGLTSFF 320

4031_Tlp3 TACITESADIINKPIHKAAFIQAIVVIIVVVFSVILLYFIVSK-YLSPLAAIQTGLTSFF 320

R14_Tlp3 TACITESADIINKPIHKAAFIQAIVVIIVVVFSVILLYFIVSK-YLSPLAAIQTGLTSFF 320

RM5611_Tlp3 TACITESADIINKPIYKAAFIQAIVVIIVVVFSVILLYFIVSK-YLSPLAAIQTGLTSFF 320

MTVDSCj16_Tlp3 TACITESADIINKPIYKAAFIQVIALIVMISISIILLYFIVSK-YLSPLAAIQTGLTSFF 320

01-1512_Tlp3 TACITESADIINKPIYKAAFIQVIALIVMISISIILLYFIVSK-YLSPLAAIQTGLTSFF 320

MTVDSCj13_Tlp3 TACITESADIINKPIFKAAYIQVIALIVMISISIILLYFIVSK-YLSPLAAIQTGLTSFF 320

32488_Tlp3a TACITESADIINKPIFKAAYIQVIALIVMISISIILLYFIVSK-YLSPLAAIQTGLTSFF 320

81116_Tlp3 TACITESADIINKPIFKAAYIQVIALIVMISISIILLYFIVSK-YLSPLAAIQTGLTSFF 320

32488_Tlp3b TACITESADIINKPIFKAAYIQVIALIVMISISIILLYFIVSK-YLSPLAAIQTGLTSFF 320

FB1_Tlp3 TACITESADIINKPIYKAAFIQAIVVIIVVVFSVILLYFIVSK-YLSPLAAIQTGLTSFF 320

PT14_Tlp3 TACITESADIINKPIYKAAFIQAIVVIIVVVFSVILLYFIVSK-YLSPLAAIQTGLTSFF 320

00-6200_Tlp3a TACITESADIINKPIYKAAFIQAIVVIIVVVFSVILLYFIVSK-YLSPLAAIQTGLTSFF 320

RM1221_Tlp3 TACITESADIINKPIFKAAFIQAIVVIIVVVFSVILLYFIVSK-YLSPLAAIQTGLTSFF 320

S3_Tlp3 TACITESADIINKPIFKAAFIQAIVVIIVVVFSVILLYFIVSK-YLSPLAAIQTGLTSFF 320

FDAARGOS_421_Tlp3 TACITESADIINKPIFKAAFIQAIVVIIVVVFSVILLYFIVSK-YLSPLAAIQTGLTSFF 331

CFSAN032806_Tlp3 TACITESADIINKPIYKAAFIQAIVVIIVVVFSVILLYFIVSK-YLSPLAAIQTGLTSFF 331

IA3901_Tlp3b TACITESADIINKPIYKAAFIQAIVVIIVVVFSVILLYFIVSK-YLSPLAAIQTGLTSFF 320

00-6200_Tlp3b TACITESADIINKPIYKAAFIQAIVVIIVVVFSVILLYFIVSK-YLSPLAAIQTGLTSFF 320

BCW_6290_Tlp3b TACITESADIINKPIYKAAFIQAIVVIIVVVFSVILLYFIVSK-YLSPLAAIQTGLTSFF 320

00-2425_Tlp3a TACITESADIINKPIYKAAFIQAIVVIIVVVFSVILLYFIVSK-YLSPLAAIQTGLTSFF 320

00-2425_Tlp3b TACITESADIINKPIYKAAFIQAIVVIIVVVFSVILLYFIVSK-YLSPLAAIQTGLTSFF 320

YH001_Tlp3a TACITESADIINKPIYKAAFIQAIVVIIVVVFSVILLYFIVSK-YLSPLAAIQTGLTSFF 320

YH001_Tlp3b TACITESADIINKPIYKAAFIQAIVVIIVVVFSVILLYFIVSK-YLSPLAAIQTGLTSFF 320

00-0949_Tlp3 TACITESADIINKPIYKAAFIQAIVVIIVVVFSIILLYFIVSK-YLSPLAAIQTGLTSFF 320

NCTC11168_Tlp3 TACITESADIINKPIYKAAFIQAIVVIIVVVFSVILLYFIVSK-YLSPLAAIQTGLTSFF 331

F38011_Tlp3 TACITESADIINKPIYKAAFIQAIVVIIVVVFSVILLYFIVSK-YLSPLAAIQTGLTSFF 320

RM1285_Tlp3 TACITESADIINKPIYKAAFIQAIVVIIVVVFSVILLYFIVSK-YLSPLAAIQTGLTSFF 320

FDAARGOS_422_Tlp3 TACITESADIINKPIYKAAFIQAIVVIIVVVFSVILLYFIVSK-YLSPLAAIQTGLTSFF 331

MTVDSCj07_Tlp3 TACITESADIINKPIYKAAFIQAIVVIIVVVFSVILLYFIVSK-YLSPLAAIQTGLTSFF 320

IA3901_Tlp3a TACITESADIINKPIYKAAFIQAIVVIIVVVFSVILLYFIVSK-YLSPLAAIQTGLTSFF 320

BCW_6290_Tlp3a TACITESADIINKPIYKAAFIQAIVVIIVVVFSVILLYFIVSK-YLSPLAAIQTGLTSFF 320

35925B2_Tlp3 TACITESADIINKPIHKAAFIQAIVVIIVVVFSVILLYFIISK-YLSPLAAIQTGLTSFF 331

14980A_Tlp3 TACITESADIINKPIHKAAFIQAIVVIIVVVFSVILLYFIVSK-YLSPLAAIQTGLTSFF 331

00-1597_Tlp3b TACITESADIINKPIFKAAYIQVIALIVMISISIILLYFIVSK-YLSPLAAIQTGLTSFF 320

RM3196_Tlp3 TACITESADIINKPIYKAAFIQAIVVIIVVVFSVILLYFIVSK-YLSPLAAIQTGLTSFF 320

FDAARGOS_295_Tlp21 RMCTLTQSKIYSDMANEILFKQILIGIIAISVILLFIQLIIKK-YLSPLAAIQTGLTSFF 322

FORC_046_Tlp4 RICTITENKVYTEAVNSILMKQVIVGIIAIIIALILIRFLISR-SLSPLAAIQTGLTSFF 334

FDAARGOS_422_Tlp4 RICTITENKVYTEAVNSILMKQVIVGIIAIIIALILIRFLISR-SLSPLAAIQTGLTSFF 334

ICDCCJ07001_Tlp4 RICTITENKVYTEAVNSILMKQVIVGIIAIIIALILIRFLISR-SLSPLAAIQTGLTSFF 334

RM3196_Tlp4 RICTITENKVYTEAVNSILMKQVIVGIIAIIIALILIRFLISR-SLSPLAAIQTGLTSFF 334

T1-21_Tlp4 RICTITENKVYTEAVNSILMKQVIVGIIAIIIALILIRFLISR-SLSPLAAIQTGLTSFF 334

F38011_Tlp4 RICTITENKVYTEAVNSILMKQVIVGIIAIIIALILIRFLISR-SLSPLAAIQTGLTSFF 334

HF5-4A-4_Tlp22 RICTITENKVYTEAVNSILMKQVIVGIIAIIIALILIRFLISR-SLSPLAAIQTGLTSFF 280

00-0949_Tlp4 RICTITENKVYTEAVNSILMKQVIVGIIAIIIALILI-ILISR-SLSPLAAIQTGLTSFF 333

01-1512_Tlp4 RICTITENKVYTEAVNSILMKQVIVGIIAIIIALILIRFLISR-SLSPLAAIQTGLTSFF 334

81-176_Tlp4 RICTITENKVYTEAVNSILMKQVIVGIIAIIIALILIRFLISR-SLSPLAAIQTGLTSFF 334

32488_Tlp4 RICTITENKVYTEAVNSILMKQVIVGIIAIIIALILIRFLISR-SLSPLAAIQTGLTSFF 334

NCTC11168_Tlp4 RICTITENKVYTEAVNSILMKQVIVGIIAIIIALILIRFLISR-SLSPLAAIQTGLTSFF 334

CFSAN032806_Tlp4 RICTITENKVYTEAVNSILMKQVIVGIIAIIIALILIRFLISR-SLSPLAAIQTGLTSFF 334

81116_Tlp4 RICTITENKVYTEAVNSILMKQVIVGIIAIIIALILIRFLISR-SLSPLAAIQTGLTSFF 334

RM1285_Tlp12 RICAVTLDSNYTSAVNSILMKQVIVGIIAIIIALILIRFLISR-SLSPLAAIQTGLTSFF 331

PT14_Tlp12 RICAVTLDSNYTSAVNSILMKQVIVGIIAIIIALILIRFLISR-SLSPLAAIQTGLTSFF 331

MTVJDCj07_Tlp12 RICAVTLDSNYTSAVNSILMKQVIVGIIAIIIALILIRFLISR-SLSPLAAIQTGLTSFF 331

RM1221_Tlp12 RICAVTLDSNYTSAVNSILMKQVIVGIIAIIIALILIRFLISR-SLSPLAAIQTGLTSFF 331

FDAARGOS_421_Tlp12 RICAVTLDSNYTSAVNSILMKQVIVGIIAIIIALILIRFLISR-SLSPLAAIQTGLTSFF 331

35925B2_Tlp12 RICAVTLDSNYTSAVNSILMKQVIVGIIAIIIALILIRFLISR-SLSPLAAIQTGLTSFF 331

CJM1cam_Tlp12 RICAVTLDSNYTSAVNSILMKQVIVGIIAIIIALILIRFLISR-SLSPLAAIQTGLTSFF 331

M1_Tlp12 RICAVTLDSNYTSAVNSILMKQVIVGIIAIIIALILIRFLISR-SLSPLAAIQTGLTSFF 331

S3_Tlp12 RICAVTLDSNYTSAVNSILMKQAIVGIIAIIIALILIRFLISR-SLSPLAAIQTGLTSFF 331

00-1597_Tlp12 RICAVTLDSNYTSAVNSILMKQAIVGIIAIIIALILIRFLISR-SLSPLAAIQTGLTSFF 331

R14_Tlp12 RICAVTLDSNYTSAVNSILMKQVIVGIIAIIIALILIRFLISR-SLSPLAAIQTGLTSFF 331

RM1875_Tlp15 DGNL---KNCYHFFSKNFFKTIIFFTLFILIIIPFIFIFYRNLITG--VQGIKTNITSFF 248

YH503_Tlp16 DRSL---EELLSFFSKSFFKTVLFSIIFILIIIPFIFIFYRNLIVG--VQGIDANITSFF 248

FB1_Tlp16 DRSL---EELLSFFSKSFFKTVLFSIIFILIIIPFIFIFYRNLIVG--VQGIDANITSFF 248

BFR-CA-9557_Tlp16 DRSL---EELLSFFSKSFFKTVLFSIIFILIIIPFIFIFYRNLIVG--VQGIDANITSFF 247

15-537360_Tlp16 DRSL---EELLSFFSKSFFKTVLFSIIFILIIIPFIFIFYRNLIVG--VQGIDANITSFF 248

OR12_Tlp16 DRSL---EELLSFFSKSFFKTVLFSIIFILIIIPFIFIFYRNLIVG--VQGIDANITSFF 248

YH502_Tlp16 DRSL---EELLSFFSKSFFKTVLFSIIFILIIIPFIFIFYRNLIVG--VQGIDANITSFF 248

14903A_Tlp16 DRSL---EELLSFFSKSFFKTVLFSIIFILIIIPFIFIFYRNLIVG--VQGIDANITSFF 248

RM5611_Tlp16 DRSL---EELLSFFSKSFFKTVLFSIIFILIIIPFIFIFYRNLIVG--VQGIDANITSFF 248

00-2425_Tlp11 -------------YELNFI--LISIAIIVLIAILIILYFCVKNIVGSKLPIIVNSLQNFF 375

00-6200_Tlp11 -------------YELNFI--LISIAIIVLIAILIILYFCVKNIVGSKLPIIVNSLQNFF 375

YH001_Tlp11 -------------YELNFI--LISIAIIVLIAILIILYFCVKNIVGSKLPIIVNSLQNFF 375

IA3902_Tlp11 -------------YELNFI--LISIAIIVLIAILIILYFCVKNIVGSKLPIIVNSLQNFF 375

BCW_6290_Tlp11 -------------YELNFI--LISIAIIVLIAILIILYFCVKNIVGSKLPIIVNSLQNFF 375

76339_Tlp18 -------------FRLQFA--IITTAIIALIVILFIVYFCVRKIVGIRIPVILKSLEDFF 374

4031_Tlp17 -------------KKLEII--FIIISFFILLVILIIVYVCVKKIVGSRIPVILKSLENFF 375

15-537360_Tlp13 -------------YKLQYI--IISVAIIALIAILAVVYFFIRKIIGSRIPLILKSLENFF 374

CVM_N29710_Tlp13 -------------YKLQYI--IISVAIIALIAILAVVYFFIRKIIGSRIPLILKSLENFF 374

FB1_Tlp13 -------------YKLQYI--IISVAIIALIAILAVVYFFIRKIIGSRIPLILKSLENFF 374

CFCAN032805_Tlp13 -------------YKLQYI--IISVAIIALIAILAVVYFFIRKIIGSRIPLILKSLENFF 374

BG2108_Tlp13 -------------YKLQYI--IISVAIIALIAILAVVYFFIRKIIGSRIPLILKSLENFF 374

YF2105_Tlp13 -------------YKLQYI--IISVAIIALIAILAVVYFFIRKIIGSRIPLILKSLENFF 374

YH503_Tlp13 -------------YKLQYI--IISVAIIALIAILAVVYFFIRKIIGSRIPLILKSLENFF 374

BFRCA9557_Tlp13 -------------YKLQYI--IISVAIIALIAILAVVYFFIRKIIGSRIPLILKSLENFF 374

YH502_Tlp13 -------------YKLQYI--IISVAIIALIAILAVVYFFIRKIIGSRIPLILKSLENFF 374

MTVDSCj13_Tlp13 -------------YKLQYT--IISVAIIALIAILTVVYFFIRKIIGSRIPLILKSLENFF 374

OR12_Tlp13 -------------YKLQYI--IISVAIIALIAILAVVYFFIRKIIGSRIPLILKSLENFF 374

00-1597_Tlp13 -------------YKLQYI--IISVAIIALIAILAVVYFFIRKIIGSRIPLILKSLENFF 374

14903A_Tlp13 -------------YKLQYI--IISVAIIALIAILAVVYFFIRKIIGSRIPLILKSLENFF 374

FJ3124_Tlp13 -------------YKLQYI--IISVAIIALIAILAVVYFFIRKIIGSRIPLILKSLENFF 374

R14_Tlp13 -------------YKLQYI--IISVAIIALIAILAVVYFFIRKIIGSRIPLILKSLENFF 374

MTVDSCj16_Tlp13 -------------YKLQYI--IISVAIIALIAILAVVYFFIRKIIGSRIPLILKSLENFF 374

14980A_Tlp13 -------------YKLQYI--IISVAIIALIAILAVVYFFIRKIIGSRIPLILKSLENFF 374

. .. : *

15-537360_Tlp20 DFINHKTKNVSTIDVKTNDEFGQISKAINENILATKQGLEQDAKAVKESVETVGVVESGN 388

76339_Tlp20 DFINHKTKDVSTIDVKTNDEFGQISKAINENILATKQGLEQDAKAVKESVETVGVVERGN 388

CFSAN032805_Tlp20 DFINHKTKNVSTIEIKSNDEFGQISKAINENILATKQGLEQDAKAVKESVETVGVVERGN 387

CVM_N29710_Tlp20 DFINHKTKNVSTIEIKTNDEFGQISKTINENILATKQGLEQDAKAVKESVETVGVVERGN 386

YH501_Tlp20 DFINHKTKNVSTIEIKTNDEFGQISKTINENILATKQGLEQDAKAVKESVETVGVVERGN 390

CO2-160_Tlp20 DFINHKTKNVSTIDVKTNDEFGQISKAINENILATKQGLEQDAKAVKESVETVGVVESGN 388

CO2-160_Tlp20b DFINHKTKNVSTIDVKTNDEFGQISKAINENILATKQGLEQDAKAVKESVETVGVVESGN 388

RM5611_Tlp20 DFINHKTKNVSTIDVKTNDEFGQISKAINENILATKQGLEQDAKAVKESVETVGVVESGN 388

14903A_Tlp20 DFINHKTKNVSTIEIKSNDEFGQISKAINENILATKQGLEQDAKAVKESVETVGVVESGN 390

YH502_Tlp20 DFINHKTKNVSTIDVKTNDEFGQISKAINENILATKQGLEQDAKAVKESVETVGVVESGN 387

RM3196_Tlp23 DFINHKTKNVSTIEVKSNDEFGQISNAINENILATKRGLEQDNQAVKESVETVHVVEGGN 388

ICDCCJ07001_Tlp23 DFINHKTKNVSTIEVKSNDEFGQISNAINENILATKRGLEQDNQAVKESVETVHVVEGGN 388

RM1285_Tlp2 DFINYKTKNVSTIEVKSNDEFGQISNAINKTFLLLK-EAEQDNQAVKESVQTVSVVEGGN 387

CFSAN032806_Tlp2 DFINYKTKNVSTIEVKSNDEFGQISNAINENILATKRGLEQDNQAVKESVQTVSVVEGGN 388

RM1221_Tlp2 DFINYKTKNVSTIEVKSNDEFGQISNAINENILATKRGLEQDNQAVKESVQTVSVVEGGN 388

S3_Tlp2 DFINYKTKNVSTIEVKSNDEFGQISNAINENILATKRGLEQDNQAVKESVQTVSVVEGGN 388

FDAARGOS_422_Tlp2 DFINYKTKNVSTIEVKSNDEFGQISNAINENILATKRGLEQDNQAVKESVQTVSVVEGGN 388

81-176_Tlp2 DFINYKTKNVSTIEVKSNDEFGQISNAINENILATKRGLEQDNQAVKESVQTVSVVEGGN 388

F38011_Tlp2 DFINYKTKNVSTIEVKSNDEFGQISNAINENILATKRGLEQDNQAVKESVQTVSVVEGGN 388

NCTC11168_Tlp2 DFINYKTKNVSTIEVKSNDEFGQISNAINENILATKRGLEQDNQAVKESVQTVSVVEGGN 388

MTVDSCj07_Tlp2 DFINYKTKNVSTIEVKSNDEFGQISNAINENILATKRGLEQDNQAVKESVQTVSVVEGGN 388

CJM1cam_Tlp24 DFINHKTKNVSTIEVKSNDEFGQISSAINENILATKRGLEQDNQAVKESVETVSVVESGN 388

M1_Tlp24 DFINHKTKNVSTIEVKSNDEFGQISSAINENILATKRGLEQDNQAVKESVETVSVVESGN 388

81116_Tlp2 DFINHKTKNVSTIEVKSNDEFGQISNAINENILATKRGLEQDNQAVKESVETVSVVESGN 388

4031_Tlp23 DFINHKTKNVSTIEVKSNDEFGQISNAINENILATKRGLEQDNQAVKESVETVSVVESGN 387

MG1116_Tlp14 ------TKNVSTIDIKTNDEFGQISKAINENILATKQGLEQDAKAVKESVETVGVVESGN 371

YH502_Tlp14 DFINYKTKNVSTIEIKSNDEFGQISKTINENILATKQGLEQDAKAVKESVETVGVVESGN 396

BP3181_Tlp14 DFINHKTKNVSTIEIKSNDEFGQISKTINENILATKQGLEQDAKAVKESVETVGVVESGN 385

ZV1224_Tlp14a DFINHKTKNVSTIDVKTNDEFGQISKAINENILATKQGLEQDAKAVKESVETVGVVESGN 385

ZV1224_Tlp14b DFINHKTKNVSTIDVKTNDEFGQISKAINENILATKQGLEQDAKAVKESVETVGVVESGN 385

YH503_Tlp14 DFINHKTKNVSTIEIKTNDEFGQISKTINENILATKQGLEQDAKAVKESVETVGVVERGN 396

14903A_Tlp14 DFINHKTKNVSTIEIKSNDEFGQISKTINENILATKQGLEQDAKAVKESVETVGVVESGN 385

OR12_Tlp14 DFINHKTKNVSTIEIKTNDEFGQISKTINENILATKQGLEQDAKAVKESVETVGVVERGN 385

CFSAN032805_Tlp14 DFINHKTKNVSTIDVKTNDEFGQISKTINENILATKQGLEQDAKAVKESVETVGVVERGN 396

BFR-CA-9557_Tlp14 DFINHKTKNVSTIDVKTNDEFGQISKAINENILATKQGLEQDAKAVKESVETVGVVERGN 385

15-537360_Tlp14 DFINHKTKNVSTIEIKSNDEFGQISKAINENILATKQGLEQDAKAVKESVETVGVVESGN 385

YH501_Tlp14 DFINHKTKNVSTIEIKSNDEFGQISKAINENILATKQGLEQDAKAVKESVETVGVVERGN 385

CG8421_Tlp14 DFINYKTKNVSTIEVKSNDEFGQISNAINENILATKRGLEQDNQAVKESVQTVSVVEGGN 379

MTVDSCj16_Tlp14 DFINYKTKNVSTIEVKSNDEFGQISNAINENILATKRGLEQDNQAVKESVQTVSVVEGGN 385

01-1512_Tlp14 DFINYKTKNVSTIEVKSNDEFGQISNAINENILATKRGLEQDNQAVKESVQTVSVVEGGN 385

00-0949_Tlp14 DFINYKTKNVSTIEVKSNDEFGQISNAINENILATKRGLEQDNQAVKESVQTVSVVEGGN 385

MTVDSCj13_Tlp14 DFINYKTKNVSTIEVKSNDEFGQISNAINENILATKRGLEQDNQAVKESVQTVSVVESGN 385

S3_Tlp14 DFINYKTKNVSTIEVKSNDEFGQISNAINENILATKRGLEQDNQAVKESVQTVSVVEGGN 385

PT14_Tlp14 DFINYKTKNVSTIEVKSNDEFGQISNAINENILATKRGLEQDNQAVKESVQTVSVVEGGN 385

14980A_Tlp14 DFINYKTKNVSTIEVKSNDEFGQISNAINENILATKRGLEQDNQAVKESVQTVSVVEGGN 401

FJ3124_Tlp14 DFINYKTKNVSTIEVKSNDEFGQISNAINENILATKRGLEQDNQAVKESVQTVSVVEGGN 385

00-1597_Tlp14 DFINYKTKNVSTIEVKSNDEFGQISNAINENILATKRGLEQDNQAVKESVQTVSVVEGGN 385

R14_Tlp14 DFINYKTKNVSTIEVKSNDEFGQISNAINENILATKRGLEQDNQAVKESVQTVSVVEGGN 385

CG8421_Tlp25 DFINYKTKNVSTIEVKSNDEFGQISNAINENILATKRGLEQDNQAVKESVQTVSVVEGGN 176

RM1875_Tlp3 DFINHKTKNVSTIDVKSNDEFGQISKAINENILATKQGLEQDAKAVKESVETVGVVESGN 380

CJM1cam_Tlp3 DFINHKTKNVSTIEVKSNDEFGQISSAINENILATKRGLEQDNQAVKESVETVSVVESGN 380

M1_Tlp3 DFINHKTKNVSTIEVKSNDEFGQISSAINENILATKRGLEQDNQAVKESVETVSVVESGN 380

4031_Tlp3 DFINHKTKNVSTIEVKSNDEFGQISNAINENILATKRGLEQDNQAVKESVETVSVVESGN 380

R14_Tlp3 DFINYKTKNVSTIEVKSNDEFGQISNAINENILATKRGLEQDNQAVKESVQTVSVVEGGN 380

RM5611_Tlp3 DFINYKTKNVSTIEVKSNDEFGQISNAINENILATKQGLEQDAKAVKESVETVGVVESGN 380

MTVDSCj16_Tlp3 DFINYKTKNVSTIEVKSNDEFGQISNAINENILATKRGLEQDNQAVKESVQTVSVVEGGN 380

01-1512_Tlp3 DFINYKTKNVSTIEVKSNDEFGQISNAINENILATKRGLEQDNQAVKESVQTVSVVEGGN 380

MTVDSCj13_Tlp3 DFINYKTKNVSTIEVKSNDEFGQISNAINENILATKRGLEQDNQAVKESVQTVSVVESGN 380

32488_Tlp3a DFINYKTKNVSTIEVKSNDEFGQISNAINENILATKRGLEQDNQAVKESVQTVSVVEGGN 380

81116_Tlp3 DFINYKTKNVSTIEVKSNDEFGQISNAINENILATKRGLEQDNQAVKESVQTVSVVEGGN 380

32488_Tlp3b DFINYKTKNVSTIEVKSNDEFGQISNAINENILATKRGLEQDNQAVKESVQTVSVVEGGN 380

FB1_Tlp3 DFINYKTKNVSTIEVKSNDEFGQISNAINENILATKQGLEQDAKAVKESVETVGVVERGN 380

PT14_Tlp3 DFINYKTKNVSTIEVKSNDEFGQISNAINENILATKRGLEQDNQAVKESVQTVSVVEGGN 380

00-6200_Tlp3a DFINHKTKNVSTIEVKSNDELGQMGKIINENILATKRGLEQDNQAVKESVQTVSVVEGGN 380

RM1221_Tlp3 DFINYKTKNVSTIEVKSNDEFGQISNAINENILATKRGLEQDNQAVKESVQTVSVVEGGN 380

S3_Tlp3 DFINYKTKNVSTIEVKSNDEFGQISNAINENILATKRGLEQDNQAVKESVQTVSVVEGGN 380

FDAARGOS_421_Tlp3 DFINYKTKNVSTIEVKSNDEFGQISNAINENILATKRGLEQDNQAVKESVQTVSVVEGGN 391

CFSAN032806_Tlp3 DFINYKTKNVSTIEVKSNDEFGQISNAINENILATKRGLEQDNQAVKESVQTVSVVEGGN 391

IA3901_Tlp3b DFINYKTKNVSTIEVKSNDEFGQISNAINENILATKRGLEQDNQAVKESVQTVSVVEGGN 380

00-6200_Tlp3b DFINYKTKNVSTIEVKSNDEFGQISNAINENILATKRGLEQDNQAVKESVQTVSVVEGGN 380

BCW_6290_Tlp3b DFINYKTKNVSTIEVKSNDEFGQISNAINENILATKRGLEQDNQAVKESVQTVSVVEGGN 380

00-2425_Tlp3a DFINHKTKNVSTIEVKSNDEFGQISNAINENILATKRGLEQDNQAVKESVQTVSVVEGGN 380

00-2425_Tlp3b DFINHKTKNVSTIEVKSNDEFGQISNAINENILATKRGLEQDNQAVKESVQTVSVVEGGN 380

YH001_Tlp3a DFINHKTKNVSTIEVKSNDEFGQISNAINENILATKRGLEQDNQAVKESVQTVSVVEGGN 380

YH001_Tlp3b DFINHKTKNVSTIEVKSNDEFGQISNAINENILATKRGLEQDNQAVKESVQTVSVVEGGN 380

00-0949_Tlp3 DFINYKTKNVSTIEVKSNDEFGQISNAINENILATKRGLEQDNQAVKESVQTVSVVEGGN 380

NCTC11168_Tlp3 DFINYKTKNVSTIEVKSNDEFGQISNAINENILATKRGLEQDNQAVKESVQTVSVVEGGN 391

F38011_Tlp3 DFINYKTKNVSTIEVKSNDEFGQISNAINENILATKRGLEQDNQAVKESVQTVSVVEGGN 380

RM1285_Tlp3 DFINYKTKNVSTIEVKSNDEFGQISNAINENILATKRGLEQDNQAVKESVQTVSVVEGGN 380

FDAARGOS_422_Tlp3 DFINYKTKNVSTIEVKSNDEFGQISNAINENILATKRGLEQDNQAVKESVQTVSVVEGGN 391

MTVDSCj07_Tlp3 DFINYKTKNVSTIEVKSNDEFGQISNAINENILATKRGLEQDNQAVKESVQTVSVVEGGN 380

IA3901_Tlp3a DFINYKTKNVSTIEVKSNDEFGQISNAINENILATKRGLEQDNQAVKESVQTVSVVEGGN 380

BCW_6290_Tlp3a DFINYKTKNVSTIEVKSNDEFGQISNAINENILATKRGLEQDNQAVKESVQTVSVVEGGN 380

35925B2_Tlp3 DFINHKTKNVSTIEVKSNDEFGQISSAINENILQTKKGLEQDNQAVKESVETVSVVESGN 391

14980A_Tlp3 DFINYKTKNVSTIEVKSNDEFGQISNAINENILATKRGLEQDNQAVKESVQTVSVVEGGN 391

00-1597_Tlp3b DFINYKTKNVSTIEVKSNDEFGQISNAINENILATKRGLEQDNQAVKESVQTVSVVEGGN 380

RM3196_Tlp3 DFINHKTKNVSTIEVKSNDEFGQISNAINENILATKRGLEQDNQAVKESVETVHVVEGGN 380

FDAARGOS_295_Tlp21 DFINHKTKNITAINIKSKDEFGQMANAINENILATKKGLEQDNQAVKESVQTVHVVESGN 382

FORC_046_Tlp4 DFINYKTKNVSTIEVKSNDEFGQISNAINENILATKRGLEQDNQAVKESVQTVSVVEGGN 394

FDAARGOS_422_Tlp4 DFINYKTKNVSTIEVKSNDEFGQISNAINENILATKRGLEQDNQAVKESVQTVSVVEGGN 394

ICDCCJ07001_Tlp4 DFINHKTKNVSTIEVKSNDEFGQISNAINENILATKQGLEQDAKAVKESVETVGVVESGN 394

RM3196_Tlp4 DFINHKTKNVSTIEVKSNDEFGQISNAINENILATKQGLEQDAKAVKESVETVGVVESGN 394

T1-21_Tlp4 DFINYKTKNVSTIEVKSNDEFGQISNAINENILATKRGLEQDNQAVKESVQTVSVVEGGN 394

F38011_Tlp4 DFINYKTKNVSTIEVKSNDEFGQISNAINENILATKRGLEQDNQAVKESVQTVSVVEGGN 394

HF5-4A-4_Tlp22 DFINYKTKNVSTIEVKSNDEFGQISNAINENILATKRGLEQDNQAVKESVQTVSVVEGGN 340

00-0949_Tlp4 DFINYKTKNVSTIEVKSNDEFGQISNAINENILATKRGLEQDNQAVKESVQTVSVVEGGN 393

01-1512_Tlp4 DFINYKTKNVSTIEVKSNDEFGQISNAINENILATKRGLEQDNQAVKESVQTVSVVEGGN 394

81-176_Tlp4 DFINYKTKNVSTIEVKSNDEFGQISNAINENILATKRGLEQDNQAVKESVQTVSVVEGGN 394

32488_Tlp4 DFINYKTKNVSTIEVKSNDEFGQISNAINENILATKRGLEQDNQAVKESVQTVSVVEGGN 394

NCTC11168_Tlp4 DFINYKTKNVSTIEVKSNDEFGQISNAINENILATKRGLEQDNQAVKESVQTVSVVEGGN 394

CFSAN032806_Tlp4 DFINYKTKNVSTIEVKSNDEFGQISNAINENILATKRGLEQDNQAVKESVQTVSVVEGGN 394

81116_Tlp4 DFINYKTKNVSTIEVKSNDEFGQISNAINENILATKRGLEQDNQAVKESVQTVSVVEGGN 394

RM1285_Tlp12 DFINYKTKNVSTIEVKSNDEFGQISNAINKTFLLLK-EAEQDNQAVKESVQTVSVVEGGN 390

PT14_Tlp12 DFINYKTKNVSTIEVKSNDEFGQISNAINENILATKRGLEQDNQAVKESVQTVSVVEGGN 391

MTVJDCj07_Tlp12 DFINYKTKNVSTIEVKSNDEFGQISNAINENILATKRGLEQDNQAVKESVQTVSVVEGGN 391

RM1221_Tlp12 DFINYKTKNVSTIEVKSNDEFGQISNAINENILATKRGLEQDNQAVKESVQTVSVVEGGN 391

FDAARGOS_421_Tlp12 DFINYKTKNVSTIEVKSNDEFGQISNAINENILATKRGLEQDNQAVKESVQTVSVVEGGN 391

35925B2_Tlp12 DFINHKTKNVSTIEVKSNDEFGQISSAINENILQTKKGLEQDNQAVKESVETVSVVESGN 391

CJM1cam_Tlp12 DFINYKTKNVSTIEVKSNDEFGQISNAINENILATKRGLEQDNQAVKESVQTVSVVEGGN 391

M1_Tlp12 DFINYKTKNVSTIEVKSNDEFGQISNAINENILATKRGLEQDNQAVKESVQTVSVVEGGN 391

S3_Tlp12 DFINYKTKNVSTIEVKSNDEFGQISNAINENILATKRGLEQDNQAVKESVQTVSVVEGGN 391

00-1597_Tlp12 DFINYKTKNVSTIEVKSNDEFGQISNAINENILATKRGLEQDNQAVKESVQTVSVVEGGN 391

R14_Tlp12 DFINYKTKNVSTIEVKSNDEFGQISNAINENILATKRGLEQDNQAVKESVQTVSVVEGGN 391

RM1875_Tlp15 DFINHKTKNVSTIEVKSNDEFGQISKAINENILATKQGLEQDAKAVKESVETVGVVESGN 308

YH503_Tlp16 NFINHKTKNVSTIEIKSNDEFGQISKTINENILATKRGLEQDNQAVKESVQTVSVVEGGN 308

FB1_Tlp16 NFINHKTKNVSTIDVKTNDEFGLISKAINENILATKQGLEQDAKAVKESVETVGVVERGN 308

BFR-CA-9557_Tlp16 NFINHKTKNVSTIEIKSNDEFGQISKTINENILATKQGLEQDAKAVKESVETVGVVERGN 307

15-537360_Tlp16 NFINHKTKNVSTIEIKSNDEFGQISKAINENILATKQGLEQDAKAVKESVETVGVVESGN 308

OR12_Tlp16 NFINHKTKNVSTIEIKSNDEFGQISKTINENILATKQGLEQDAKAVKESVETVGVVKRGN 308

YH502_Tlp16 NFINHKTKNVSTIEIKSNDEFGQISKTINENILATKQGLEQDAKAVKESVETVGVVESGN 308

14903A_Tlp16 NFINHKTKNVSTIEIKSNDEFGQISKAINENILATKQGLEQDAKAVKESVETVGVVESGN 308

RM5611_Tlp16 DFINHKTKNVSTIDVKTNDEFGQISKAINENILATKQGLEQDAKAVKESVETVGVVESGN 308

00-2425_Tlp11 DFINHKTKNVSTIEVKSNDELGQMGKIINENILATKRGLEQDNQAVKESVQTVSVVEGGN 435

00-6200_Tlp11 DFINHKTKNVSTIEVKSNDELGQMGKIINENILATKRGLEQDNQAVKESVQTVSVVEGGN 435

YH001_Tlp11 DFINHKTKNVSTIEVKSNDELGQMGKIINENILATKRGLEQDNQAVKESVQTVSVVEGGN 435

IA3902_Tlp11 DFINHKTKNVSTIEVKSNDELGQMGKIINENILATKRGLEQDNQAVKESVETVHVVEGGN 435

BCW_6290_Tlp11 DFINHKTKNVSTIEVKSNDELGQMGKIINENILATKRGLEQDNQAVKESVETVHVVEGGN 435

76339_Tlp18 RFLNHEKIEVHTIKISSNDELGKMAKAINENILATKQGLEQDAKAVKESVETVEVVERGN 434

4031_Tlp17 HFLNHKKHEVDLISIKADDELGKMGKMINENILATKKGLEQDNQAVKESVQTVSVVESGN 435

15-537360_Tlp13 RFLNHEKIEVQTIEIKANDELGKMGKIINENILATKQGLEQDAKAVKESVETVSVVEGGN 434

CVM_N29710_Tlp13 RFLNHEKIEVQTIEIKANDELGKMGKIINENILATKRGLEQDNQAVKESVQTVSVVEGGN 434

FB1_Tlp13 RFLNHEKIEVQTIEIKANDELGKMGKIINENILATKRGLEQDNQAVKESVQTVSVVEGGN 434

CFCAN032805_Tlp13 RFLNHEKIEVQTIEIKANDELGKMGKIINENILATKRGLEQDNQAVKESVQTVSVVEGGN 434

BG2108_Tlp13 RFLNHEKIEVQTIEIKANDELGKMGKIINENILATKRGLEQDNQAVKESVQTVSVVEGGN 434

YF2105_Tlp13 RFLNHEKIEVQTIEIKANDELGKMGKIINENILATKRGLEQDNQAVKESVQTVSVVEGGN 434

YH503_Tlp13 RFLNHEKIEVQTIEIKANDELGKMGKTINENILATKRGLEQDNQAVKESVQTVSVVEGGN 434

BFRCA9557_Tlp13 RFLNHEKIEIQTIEIKANDELGKMGKIINENILATKQGLEQDAKAVKESVETVGVVERGN 434

YH502_Tlp13 RFLNHEKIEVQTIEIKANDELGKMGKTINENILATKQGLEQDAKAVKESVETVGVVESGN 434

MTVDSCj13_Tlp13 RFLNHEKIEIQTIEIKANDELGKMGKIINENILATKQGLEQDAKAVKESVETVGVVESGN 434

OR12_Tlp13 RFLNHEKIEVQTIEIKANDELGKMGKIINENILATKRGLEQDNQAVKESVQTVSVVEGGN 434

00-1597_Tlp13 RFLNHEKIEVQTIEIKANDELGKMGKIINENILATKRGLEQDNQAVKESVQTVSVVEGGN 434

14903A_Tlp13 RFLNHEKIEVQTIEIKANDELGKMGKIINENILATKRGLEQDNQAVKESVQTVSVVEGGN 434

FJ3124_Tlp13 RFLNHEKIEVQTIEIKANDELGKMGKIINENILATKRGLEQDNQAVKESVQTVSVVEGGN 434

R14_Tlp13 RFLNHEKIEVQTIEIKANDELGKMGKIINENILATKRGLEQDNQAVKESVQTVSVVEGGN 434

MTVDSCj16_Tlp13 RFLNHEKIEVQTIEIKANDELGKMGKIINENILATKRGLEQDNQAVKESVQTVSVVEGGN 434

14980A_Tlp13 RFLNHEKIEVQTIEIKANDELGKMGKIINENILATKRGLEQDNQAVKESVQTVSVVEGGN 434

. :: *.:.:.**:* :.. **:.:* * *** :******:** **: **

15-537360_Tlp20 LTARI-------TANPRNPQLIELKNVLNRLLDALQARVGS---DMNEIQRVFNSYKSLD 438

76339_Tlp20 LTARI-------TANPRNPQLIELKNVLNKLLDVLQTKVGS---DMNAIHKIFEEYKSLD 438

CFSAN032805_Tlp20 LTARI-------TANPRNPQLIELKNVLNKLLDVLQTKVGS---DMNAIHKIFEEYKSLD 437

CVM_N29710_Tlp20 LTARI-------TANPRNPQLIELKNVLNKLLDVLQTKVGS---DMNAIHKIFEEYKSLD 436

YH501_Tlp20 LTARI-------TANPRNPQLIELKNVLNKLLDVLQTKVGS---DMNAIHKIFEEYKSLD 440

CO2-160_Tlp20 LTARI-------TANPRNPQLIELKNVLNRLLDVLQTKVGS---DMNAIHKIFEEYKSLD 438

CO2-160_Tlp20b LTARI-------TANPRNPQLIELKNVLNRLLDVLQTKVGS---DMNAIHKIFEEYKSLD 438

RM5611_Tlp20 LTARI-------TANPRNPQLIELKNVLNRLLDVLQTRVGS---DMNAIHKIFEEYKSLD 438

14903A_Tlp20 LTARI-------TANPRNPQLIELKNVLNRLLDVLQTKVGS---DMNAIHKIFEEYKSLD 440

YH502_Tlp20 LTARI-------TANPRNPQLIELKNVLNRLLDVLQTKVGS---DMNAIHKIFEEYKSLD 437

RM3196_Tlp23 LTARI-------TANPRNPQLIELKNVLNRLLDALQARVGS---DMNEIQRVFNSYKSLD 438

ICDCCJ07001_Tlp23 LTARI-------TANPRNPQLIELKNVLNRLLDALQARVGS---DMNEIQRVFNSYKSLD 438

RM1285_Tlp2 LTARI-------TANPRNPQLIELKNVLNKLLDVLQARVGS---DMNAIHKIFEEYKSLD 437

CFSAN032806_Tlp2 LTARI-------TANPRNPQLIELKNVLNKLLDVLQARVGS---DMNAIHKIFEEYKSLD 438

RM1221_Tlp2 LTARI-------TANPRNPQLIELKNVLNKLLDVLQARVGS---DMNAIHKIFEEYKSLD 438

S3_Tlp2 LTARI-------TANPRNPQLIELKNVLNKLLDVLQARVGS---DMNAIHKIFEEYKSLD 438

FDAARGOS_422_Tlp2 LTARI-------TANPRNPQLIELKNVLNKLLDVLQARVGS---DMNAIHKIFEEYKSLD 438

81-176_Tlp2 LTARI-------TANPRNPQLIELKNVLNKLLDVLQARVGS---DMNAIHKIFEEYKSLD 438

F38011_Tlp2 LTARI-------TANPRNPQLIELKNVLNKLLDVLQARVGS---DMNAIHKIFEEYKSLD 438

NCTC11168_Tlp2 LTARI-------TANPRNPQLIELKNVLNKLLDVLQARVGS---DMNAIHKIFEEYKSLD 438

MTVDSCj07_Tlp2 LTARI-------TANPRNPQLIELKNVLNKLLDVLQARVGS---DMNAIHKIFEEYKSLD 438

CJM1cam_Tlp24 LTARI-------TANPRNPQLIELKNVLNKLLDVLQARVVL---Y-ECYS-NF-RIQSLD 435

M1_Tlp24 LTARI-------TANPRNPQLIELKNVLNKLLDVLQARVVL---I--CYS-NF-RIQSLD 434

81116_Tlp2 LTARI-------TANPRNPQLIELKNVLNKLLDVLQARVGS---DMNAIHKIFEEYKSLD 438

4031_Tlp23 LTARI-------TANPRNPQLIELKNVLNKLLDVLQARVGS---DMNAIHKIFEEYKSLD 437

MG1116_Tlp14 LTARITANHARITANPRNPQLIELKNVLNRLLDVLQTKVGS---DMNAIHKIFEEYKSLD 428

YH502_Tlp14 LTARI-------TANPRNPQLIELKNVLNRLLDVLQTKVGS---DMNAIHKIFEEYKSLD 446

BP3181_Tlp14 LTARI-------TANPRNPQLIELKNVLNRLLDVLQTKVGS---DMNAIHKIFEEYKSLD 435

ZV1224_Tlp14a LTARI-------TANPRNPQLIELKNVLNRLLDVLQTKVGS---DMNAIHKIFEEYKSLD 435

ZV1224_Tlp14b LTARI-------TANPRNPQLIELKNVLNRLLDVLQTKVGS---DMNAIHKIFEEYKSLD 435

YH503_Tlp14 LTARI-------TANPRNPQLIELKNVLNRLLDVLQTKVGS---DMNAIHKIFEEYKSLD 446

14903A_Tlp14 LTARI-------TANPRNPQLIELKNVLNRLLDVLQTKVGS---DMNAIHKIFEEYKSLD 435

OR12_Tlp14 LTARI-------TANPRNPQLIELKNVLNKLLDVLQTKVGS---DMNAIHKIFEEYKSLD 435

CFSAN032805_Tlp14 LTARI-------TANPRNPQLIELKNVLNKLLDVLQTKVGS---DMNAIHKIFEEYKSLD 446

BFR-CA-9557_Tlp14 LTARI-------TANPRNPQLIELKNVLNRLLDVLQTKVGS---DMNAIHKIFEEYKSLD 435

15-537360_Tlp14 LTARI-------TANPRNPQLIELKNVLNRLLDVLQTKVGS---DMNAIHKIFEEYKSLD 435

YH501_Tlp14 LTARI-------TANPRNPQLIELKNVLNKLLDVLQTKVGS---DMNAIHKIFEEYKSLD 435

CG8421_Tlp14 LTARI-------TANPRNPQLIELKNVLNKLLDVLQARVGS---DMNAIHKIFEEYKSLD 429

MTVDSCj16_Tlp14 LTARI-------TANPRNPQLIELKNVLNKLLDVLQARVGS---DMNAIHKIFEEYKSLD 435

01-1512_Tlp14 LTARI-------TANPRNPQLIELKNVLNKLLDVLQARVGS---DMNAIHKIFEEYKSLD 435

00-0949_Tlp14 LTARI-------TANPRNPQLIELKNVLNKLLDVLQARVGS---DMNAIHKIFEEYKSLD 435

MTVDSCj13_Tlp14 LTARI-------TANPRNPQLIELKNVLNKLLDVLQARVGS---DMNAIHKIFEEYKSLD 435

S3_Tlp14 LTARI-------TANPRNPQLIELKNVLNKLLDVLQARVGS---DMNAIHKIFEEYKSLD 435

PT14_Tlp14 LTARI-------TANPRNPQLIELKNVLNKLLDVLQARVGS---DMNAIHKIFEEYKSLD 435

14980A_Tlp14 LTARI-------TANPRNPQLIELKNVLNKLLDVLQARVGS---DMNAIHKIFEEYKSLD 451

FJ3124_Tlp14 LTARI-------TANPRNPQLIELKNVLNKLLDVLQARVGS---DMNAIHKIFEEYKSLD 435

00-1597_Tlp14 LTARI-------TANPRNPQLIELKNVLNRLLDALQARVGS---DMNEIQRVFNSYKSLD 435

R14_Tlp14 LTARI-------TANPRNPQLIELKNVLNRLLDALQARVGS---DMNEIQRVFNSYKSLD 435

CG8421_Tlp25 LTARI-------TANPRNPQLIELKNVLNKLLDVLQARVGS---DMNAIHKIFEEYKSLD 226

RM1875_Tlp3 LTARI-------TANPRNPQLIELKNVLNRLLDVLQTRVGS---DMNAIHKIFEEYKSLD 430

CJM1cam_Tlp3 LTARI-------TANPRNPQLIELKNVLNKLLDVLQARVGS---DMNAIHKIFEEYKSLD 430

M1_Tlp3 LTARI-------TANPRNPQLIELKNVLNKLLDVLQARVGS---DMNAIHKIFEEYKSLD 430

4031_Tlp3 LTARI-------TANPRNPQLIELKNVLNKLLDVLQARVGS---DMNAIHKIFEEYKSLD 430

R14_Tlp3 LTARI-------TANPRNPQLIELKNVLNKLLDVLQARVGS---DMNAIHKIFEEYKSLD 430

RM5611_Tlp3 LTARI-------TANPRNPQLIELKNVLNRLLDVLQTKVGS---DMNAIHKIFEEYKSLD 430

MTVDSCj16_Tlp3 LTARI-------TANPRNPQLIELKNVLNKLLDVLQARVGS---DMNAIHKIFEEYKSLD 430

01-1512_Tlp3 LTARI-------TANPRNPQLIELKNVLNKLLDVLQARVGS---DMNAIHKIFEEYKSLD 430

MTVDSCj13_Tlp3 LTARI-------TANPRNPQLIELKNVLNKLLDVLQARVGS---DMNAIHKIFEEYKSLD 430

32488_Tlp3a LTARI-------TANPRNPQLIELKNVLNKLLDVLQARVGS---DMNAIHKIFEEYKSLD 430

81116_Tlp3 LTARI-------TANPRNPQLIELKNVLNKLLDVLQARVGS---DMNAIHKIFEEYKSLD 430

32488_Tlp3b LTARI-------TANPRNPQLIELKNVLNKLLDVLQARVGS---DMNAIHKIFEEYKSLD 430

FB1_Tlp3 LTARI-------TANPRNPQLIELKNVLNKLLDVLQARVGS---DMNAIHKIFEEYKSLD 430

PT14_Tlp3 LTARI-------TANPRNPQLIELKNVLNKLLDVLQARVGS---DMNAIHKIFEEYKSLD 430

00-6200_Tlp3a LTARI-------TANPRNPQLIELKNVLNKLLDVLQARVGS---DMNAIHKIFEEYKSLD 430

RM1221_Tlp3 LTARI-------TANPRNPQLIELKNVLNKLLDVLQARVGS---DMNAIHKIFEEYKSLD 430

S3_Tlp3 LTARI-------TANPRNPQLIELKNVLNKLLDVLQARVGS---DMNAIHKIFEEYKSLD 430

FDAARGOS_421_Tlp3 LTARI-------TANPRNPQLIELKNVLNKLLDVLQARVGS---DMNAIHKIFEEYKSLD 441

CFSAN032806_Tlp3 LTARI-------TANPRNPQLIELKNVLNKLLDVLQARVGS---DMNAIHKIFEEYKSLD 441

IA3901_Tlp3b LTARI-------TANPRNPQLIELKNVLNKLLDVLQARVGS---DMNAIHKIFEEYKSLD 430

00-6200_Tlp3b LTARI-------TANPRNPQLIELKNVLNKLLDVLQARVGS---DMNAIHKIFEEYKSLD 430

BCW_6290_Tlp3b LTARI-------TANPRNPQLIELKNVLNKLLDVLQARVGS---DMNAIHKIFEEYKSLD 430

00-2425_Tlp3a LTARI-------TANPRNPQLIELKNVLNKLLDVLQARVGS---DMNAIHKIFEEYKSLD 430

00-2425_Tlp3b LTARI-------TANPRNPQLIELKNVLNKLLDVLQARVGS---DMNAIHKIFEEYKSLD 430

YH001_Tlp3a LTARI-------TANPRNPQLIELKNVLNKLLDVLQARVGS---DMNAIHKIFEEYKSLD 430

YH001_Tlp3b LTARI-------TANPRNPQLIELKNVLNKLLDVLQARVGS---DMNAIHKIFEEYKSLD 430

00-0949_Tlp3 LTARI-------TANPRNPQLIELKNVLNKLLDVLQARVGS---DMNAIHKIFEEYKSLD 430

NCTC11168_Tlp3 LTARI-------TANPRNPQLIELKNVLNKLLDVLQARVGS---DMNAIHKIFEEYKSLD 441

F38011_Tlp3 LTARI-------TANPRNPQLIELKNVLNKLLDVLQARVGS---DMNAIHKIFEEYKSLD 430

RM1285_Tlp3 LTARI-------TANPRNPQLIELKNVLNKLLDVLQARVGS---DMNAIHKIFEEYKSLD 430

FDAARGOS_422_Tlp3 LTARI-------TANPRNPQLIELKNVLNKLLDVLQARVGS---DMNAIHKIFEEYKSLD 441

MTVDSCj07_Tlp3 LTARI-------TANPRNPQLIELKNVLNKLLDVLQARVGS---DMNAIHKIFEEYKSLD 430

IA3901_Tlp3a LTARI-------TANPRNPQLIELKNVLNKLLDVLQARVGS---DMNAIHKIFEEYKSLD 430

BCW_6290_Tlp3a LTARI-------TANPRNPQLIELKNVLNKLLDVLQARVGS---DMNAIHKIFEEYKSLD 430

35925B2_Tlp3 LTARI-------TANPRNPQLIELKNVLNRLLDALQTRVGSDGSDMNEIQRVFNSYKSLD 444

14980A_Tlp3 LTARI-------TANPRNPQLIELKNVLNRLLDALQARVGS---DMNEIQRVFNSYKSLD 441

00-1597_Tlp3b LTARI-------TANPRNPQLIELKNVLNRLLDALQARVGS---DMNEIQRVFNSYKSLD 430

RM3196_Tlp3 LTARI-------TANPRNPQLIELKNVLNRLLDALQARVGS---DMNEIQRVFNSYKSLD 430

FDAARGOS_295_Tlp21 LTARI-------TANPRNPQLIELKNVLNKLLDVLQARVGS---DMNEIQRVFNSYKSLD 432

FORC_046_Tlp4 LTARI-------TANPRNPQLIELKNVLNRLLDALQARVGS---DMNEIQRVFNSYKSLD 444

FDAARGOS_422_Tlp4 LTARI-------TANPRNPQLIELKNVLNRLLDALQARVGS---DMNEIQRVFNSYKSLD 444

ICDCCJ07001_Tlp4 LTARI-------TANPRNPQLIELKNVLNRLLDVLQTRVGS---DMNAIHKIFEEYKSLD 444

RM3196_Tlp4 LTARI-------TANPRNPQLIELKNVLNRLLDVLQTRVGS---DMNAIHKIFEEYKSLD 444

T1-21_Tlp4 LTARI-------TANPRNPQLIELKNVLNKLLDVLQARVGS---DMNAIHKIFEEYKSLD 444

F38011_Tlp4 LTARI-------TANPRNPQLIELKNVLNKLLDVLQARVGS---DMNAIHKIFEEYKSLD 444

HF5-4A-4_Tlp22 LTARI-------TANPRNPQLIELKNVLNKLLDVLQARVGS---DMNAIHKIFEEYKSLD 390

00-0949_Tlp4 LTARI-------TANPRNPQLIELKNVLNKLLDVLQARVGS---DMNAIHKIFEEYKSLD 443

01-1512_Tlp4 LTARI-------TANPRNPQLIELKNVLNKLLDVLQARVGS---DMNAIHKIFEEYKSLD 444

81-176_Tlp4 LTARI-------TANPRNPQLIELKNVLNKLLDVLQARVGS---DMNAIHKIFEEYKSLD 444

32488_Tlp4 LTARI-------TANPRNPQLIELKNVLNKLLDVLQARVGS---DMNAIHKIFEEYKSLD 444

NCTC11168_Tlp4 LTARI-------TANPRNPQLIELKNVLNKLLDVLQARVGS---DMNAIHKIFEEYKSLD 444

CFSAN032806_Tlp4 LTARI-------TANPRNPQLIELKNVLNKLLDVLQARVGS---DMNAIHKIFEEYKSLD 444

81116_Tlp4 LTARI-------TANPRNPQLIELKNVLNKLLDVLQARVGS---DMNAIHKIFEEYKSLD 444

RM1285_Tlp12 LTARI-------TANPRNPQLIELKNVLNKLLDVLQARVGS---DMNAIHKIFEEYKSLD 440

PT14_Tlp12 LTARI-------TANPRNPQLIELKNVLNKLLDVLQARVGS---DMNAIHKIFEEYKSLD 441

MTVJDCj07_Tlp12 LTARI-------TANPRNPQLIELKNVLNKLLDVLQARVGS---DMNAIHKIFEEYKSLD 441

RM1221_Tlp12 LTARI-------TANPRNPQLIELKNVLNKLLDVLQARVGS---DMNAIHKIFEEYKSLD 441

FDAARGOS_421_Tlp12 LTARI-------TANPRNPQLIELKNVLNKLLDVLQARVGS---DMNAIHKIFEEYKSLD 441

35925B2_Tlp12 LTARI-------TANPRNPQLIELKNVLNRLLDALQTRVGS---DMNEIQRVFNSYKSLD 441

CJM1cam_Tlp12 LTARI-------TANPRNPQLIELKNVLNRLLDALQARVGS---DMNEIQRVFNSYKSLD 441

M1_Tlp12 LTARI-------TANPRNPQLIELKNVLNRLLDALQARVGS---DMNEIQRVFNSYKSLD 441

S3_Tlp12 LTARI-------TANPRNPQLIELKNVLNRLLDALQARVGS---DMNEIQRVFNSYKSLD 441

00-1597_Tlp12 LTARI-------TANPRNPQLIELKNVLNRLLDALQARVGS---DMNEIQRVFNSYKSLD 441

R14_Tlp12 LTARI-------TANPRNPQLIELKNVLNRLLDALQARVGS---DMNEIQRVFNSYKSLD 441

RM1875_Tlp15 LTARI-------TANPRNPQLIELKNVLNRLLDVLQTRVGS---DMNAIHKIFEEYKSLD 358

YH503_Tlp16 LTARI-------TANPRNPQLIELKNVLNRLLDVLQTKVGS---DMNAIHKIFEEYKSLD 358

FB1_Tlp16 LTARI-------TANPRNPQLIELKNVLNRLLDVLQTKVGS---DMNAIHKIFEEYKSLD 358

BFR-CA-9557_Tlp16 LTARI-------TANPRNPQLIELKNVLNKLLDVLQTKVGS---DMNAIHKIFEEYKSLD 357

15-537360_Tlp16 LTARI-------TANPRNPQLIELKNVLNRLLDALQARVGS---DMNAIHKIFEEYKSLD 358

OR12_Tlp16 LTARI-------TANPRNPQLIELKNVLNKLLDVLQTKVGS---DMNAIHKIFEEYKSLD 358

YH502_Tlp16 LTARI-------TANPRNPQLIELKNVLNRLLDVLQTKVGS---DMNAIHKIFEEYKSLD 358

14903A_Tlp16 LTARI-------TANPRNPQLIELKNVLNRLLDVLQTKVGS---DMNAIHKIFEEYKSLD 358

RM5611_Tlp16 LTARI-------TANPRNPQLIELKNVLNRLLDVLQTKVGS---DMNAIHKIFEEYKSLD 358

00-2425_Tlp11 LTARI-------TANPRNPQLIELKNVLNKLLDVLQARVGS---DMNAIHKIFEEYKSLD 485

00-6200_Tlp11 LTARI-------TANPRNPQLIELKNVLNKLLDVLQARVGS---DMNAIHKIFEEYKSLD 485

YH001_Tlp11 LTARI-------TANPRNPQLIELKNVLNKLLDVLQARVGS---DMNAIHKIFEEYKSLD 485

IA3902_Tlp11 LTARI-------TANPRNPQLIELKNVLNRLLDALQARVGS---DMNEIQRVFNSYKSLD 485

BCW_6290_Tlp11 LTARI-------TANPRNPQLIELKNVLNRLLDALQARVGS---DMNEIQRVFNSYKSLD 485

76339_Tlp18 LTARI-------TANPRNPQLIELKNVLNKLLDVLQTKVGS---DMNAIHKIFEEYKSLD 484

4031_Tlp17 LTARI-------TANPRNPQLIELKNVLNKLLDVLQARVGS---DMNAIHKIFEEYKSLD 485

15-537360_Tlp13 LTARI-------TANPRNPQLIELKNVLNRLLDALQARVGS---DMNAIHKIFEEYKSLD 484

CVM_N29710_Tlp13 LTARI-------TANPRNPQLIELKNVLNRLLDALQARVGS---DMNAIHKIFEEYKSLD 484

FB1_Tlp13 LTARI-------TANPRNPQLIELKNVLNRLLDALQARVGS---DMNAIHKIFEEYKSLD 484

CFCAN032805_Tlp13 LTARI-------TANPRNPQLIELKNVLNRLLDALQARVGS---DMNAIHKIFEEYKSLD 484

BG2108_Tlp13 LTARI-------TANPRNPQLIELKNVLNRLLDVLQTKVGS---DMNAIHKIFEEYKSLD 484

YF2105_Tlp13 LTARI-------TANPRNPQLIELKNVLNRLLDVLQTKVGS---DMNAIHKIFEEYKSLD 484

YH503_Tlp13 LTARI-------TANPRNPQLIELKNVLNRLLDVLQTKVGS---DMNAIHKIFEEYKSLD 484

BFRCA9557_Tlp13 LTARI-------TANPRNPQLIELKNVLNKLLDVLQTKVGS---DMNAIHKIFEEYKSLD 484

YH502_Tlp13 LTARI-------TANPRNPQLIELKNVLNRLLDVLQTKVGS---DMNAIHKIFEEYKSLD 484

MTVDSCj13_Tlp13 LTARI-------TANPRNPQLIELKNVLNKLLDVLQARVGS---DMNAIHKIFEEYKSLD 484

OR12_Tlp13 LTARI-------TANPRNPQLIELKNVLNRLLDALQARVGS---DMNEIQRVFNSYKSLD 484

00-1597_Tlp13 LTARI-------TANPRNPQLIELKNVLNRLLDALQARVGS---DMNEIQRVFNSYKSLD 484

14903A_Tlp13 LTARI-------TANPRNPQLIELKNVLNRLLDALQARVGS---DMNEIQRVFNSYKSLD 484

FJ3124_Tlp13 LTARI-------TANPRNPQLIELKNVLNKLLDVLQARVGS---DMNAIHKIFEEYKSLD 484

R14_Tlp13 LTARI-------TANPRNPQLIELKNVLNKLLDVLQARVGS---DMNAIHKIFEEYKSLD 484

MTVDSCj16_Tlp13 LTARI-------TANPRNPQLIELKNVLNKLLDVLQARVGS---DMNAIHKIFEEYKSLD 484

14980A_Tlp13 LTARI-------TANPRNPQLIELKNVLNKLLDVLQARVGS---DMNAIHKIFEEYKSLD 484

***** *****************:***.**::*** ** * :***

15-537360_Tlp20 FTTEVKDANGAVEVTTNALGQEIIKMLKQSSDFANALANESGKLQTAVQSLTTSSNSQAA 498

76339_Tlp20 FRNKLDNANGSVEVTTNALGDEIVKMLKQSSDFANHLASESSKLQSAVQNLTSSSNSQAA 498

CFSAN032805_Tlp20 FRNKLDNANGSVEVTTNALGDEIVKMLKQSSDFANHLASESSKLQSAVQNLTSSSNSQAA 497

CVM_N29710_Tlp20 FRNKLDNANGSVEVTTNALGDEIVKMLKQSSDFANHLASESSKLQSAVQNLTSSSNSQAA 496

YH501_Tlp20 FRNKLDNANGSVEVTTNALGDEIVKMLKQSSDFANHLASESSKLQSAVQNLTSSSNSQAA 500

CO2-160_Tlp20 FRNKLDNANGSVEVTTNALGDEIVKMLKQSSDFANHLASESSKLQSAVQNLTSSSNSQAA 498

CO2-160_Tlp20b FRNKLDNANGSVEVTTNALGDEIVKMLKQSSDFANHLASESSKLQSAVQNLTSSSNSQAA 498

RM5611_Tlp20 FRNKLDNANGSVEVTTNALGDEIVKMLKQSSDFANHLASESSKLQSAVQNLTSSSNSQAA 498

14903A_Tlp20 FRNKLDNANGSVEVTTNALGDEIVKMLKQSSDFANHLASESSKLQSAVQNLTSSSNSQAA 500

YH502_Tlp20 FRNKLDNANGSVEVTTNALGDEIVKMLKQSSDFANHLASESSKLQSAVQNLTSSSNSQAA 497

RM3196_Tlp23 FTTEVKDANGAVEVTTNALGQEIIKMLKQSSDFANALANESGKLQTAVQSLTTSSNSQAQ 498

ICDCCJ07001_Tlp23 FTTEVKDANGAVEVTTNALGQEIIKMLKQSSDFANALANESGKLQTAVQSLTTSSNSQAQ 498

RM1285_Tlp2 FRNKLENASGSVELTTNALGDEIVKMLKQSSDFANALANESGKLQTAVQSLTTSSNSQAQ 497

CFSAN032806_Tlp2 FRNKLENASGSVELTTNALGDEIVKMLKQSSDFANALANESGKLQTAVQSLTTSSNSQAQ 498

RM1221_Tlp2 FRNKLENASGSVELTTNALGDEIVKMLKQSSDFANALANESGKLQTAVQSLTTSSNSQAQ 498

S3_Tlp2 FRNKLENASGSVELTTNALGDEIVKMLKQSSDFANALANESGKLQTAVQSLTTSSNSQAQ 498

FDAARGOS_422_Tlp2 FRNKLENASGSVELTTNALGDEIVKMLKQSSDFANALANESGKLQTAVQSLTTSSNSQAQ 498

81-176_Tlp2 FRNKLENASGSVELTTNALGDEIVKMLKQSSDFANALANESGKLQTAVQSLTTSSNSQAQ 498

F38011_Tlp2 FRNKLENASGSVELTTNALGDEIVKMLKQSSDFANALANESGKLQTAVQSLTTSSNSQAQ 498

NCTC11168_Tlp2 FRNKLENASGSVELTTNALGDEIVKMLKQSSDFANALANESGKLQTAVQSLTTSSNSQAQ 498

MTVDSCj07_Tlp2 FRNKLENASGSVELTTNALGDEIVKMLKQSSDFANALANESGKLQTAVQSLTTSSNSQAQ 498

CJM1cam_Tlp24 FRNKLENASGSVELTTNALGDEIVKMLKQSSDFANALANESGKLQTAVQSLTTSSNSQAQ 495

M1_Tlp24 FRNKLENASGSVELTTNALGDEIVKMLKQSSDFANALANESGKLQTAVQSLTTSSNSQAQ 494

81116_Tlp2 FRNKLENASGSVELTTNALGDEIVKMLKQSSDFANALANESGKLQTAVQSLTTSSNSQAQ 498

4031_Tlp23 FRNKLENASGSVELTTNALGDEIVKMLKQSSDFANALANESGKLQTAVQSLTTSSNSQAQ 497

MG1116_Tlp14 FRNKLDNANGSVEVTTNALGDEIVKMLKQSSDFANHLASESSKLQSAVQNLTSSSNSQAA 488

YH502_Tlp14 FRNKLDNANGSVEVTTNALGDEIVKMLKQSSDFANHLASESSKLQSAVQNLTSSSNSQAA 506

BP3181_Tlp14 FRNKLDNANGSVEVTTNALGDEIVKMLKQSSDFANHLASESSKLQSAVQNLTSSSNSQAA 495

ZV1224_Tlp14a FRNKLDNANGSVEVTTNALGDEIVKMLKQSSDFANHLASESSKLQSAVQNLTSSSNSQAA 495

ZV1224_Tlp14b FRNKLDNANGSVEVTTNALGDEIVKMLKQSSDFANHLASESSKLQSAVQNLTSSSNSQAA 495

YH503_Tlp14 FRNKLDNANGSVEVTTNALGDEIVKMLKQSSDFANHLASESSKLQSAVQNLTSSSNSQAA 506

14903A_Tlp14 FRNKLDNANGSVEVTTNALGDEIVKMLKQSSDFANHLASESSKLQSAVQNLTSSSNSQAA 495

OR12_Tlp14 FRNKLDNANGSVEVTTNALGDEIVKMLKQSSDFANHLASESSKLQSAVQNLTSSSNSQAA 495

CFSAN032805_Tlp14 FRNKLDNANGSVEVTTNALGDEIVKMLKQSSDFANHLASESSKLQSAVQNLTSSSNSQAA 506

BFR-CA-9557_Tlp14 FRNKLDNANGSVEVTTNALGDEIVKMLKQSSDFANHLASESSKLQSAVQNLTSSSNSQAA 495

15-537360_Tlp14 FRNKLDNANGSVEVTTNALGDEIVKMLKQSSDFANHLASESSKLQSAVQNLTSSSNSQAA 495

YH501_Tlp14 FRNKLDNANGSVEVTTNALGDEIVKMLKQSSDFANHLASESSKLQSAVQNLTSSSNSQAA 495

CG8421_Tlp14 FRNKLENASGSVELTTNALGDEIVKMLKQSSDFANALANESGKLQTAVQSLTTSSNSQAQ 489

MTVDSCj16_Tlp14 FRNKLENASGSVELTTNALGDEIVKMLKQSSDFANALANESGKLQTAVQSLTTSSNSQAQ 495

01-1512_Tlp14 FRNKLENASGSVELTTNALGDEIVKMLKQSSDFANALANESGKLQTAVQSLTTSSNSQAQ 495

00-0949_Tlp14 FRNKLENASGSVELTTNALGDEIVKMLKQSSDFANALANESGKLQTAVQSLTTSSNSQAQ 495

MTVDSCj13_Tlp14 FRNKLENASGSVELTTNALGDEIVKMLKQSSDFANALANESGKLQTAVQSLTTSSNSQAQ 495

S3_Tlp14 FRNKLENASGSVELTTNALGDEIVKMLKQSSDFANALANESGKLQTAVQSLTTSSNSQAQ 495

PT14_Tlp14 FRNKLENASGSVELTTNALGDEIVKMLKQSSDFANALANESGKLQTAVQSLTTSSNSQAQ 495

14980A_Tlp14 FRNKLENASGSVELTTNALGDEIVKMLKQSSDFANALANESGKLQTAVQSLTTSSNSQAQ 511

FJ3124_Tlp14 FRNKLENASGSVELTTNALGDEIVKMLKQSSDFANALANESGKLQTAVQSLTTSSNSQAQ 495

00-1597_Tlp14 FTTEVKDANGAVEVTTNALGQEIIKMLKQSSDFANALANESGKLQTAVQSLTTSSNSQAQ 495

R14_Tlp14 FTTEVKDANGAVEVTTNALGQEIIKMLKQSSDFANALANESGKLQTAVQSLTTSSNSQAQ 495

CG8421_Tlp25 FRNKLENASGSVELTTNALGDEIVKMLKQSSDFANALANESGKLQTAVQSLTTSSNSQAQ 286

RM1875_Tlp3 FRNKLDNANGSVEVTTNALGMKLVKMLKQSSDFANHLASESSKLQSAVQNLTSSSNSQAA 490

CJM1cam_Tlp3 FRNKLENASGSVELTTNALGDEIVKMLKQSSDFANALANESGKLQTAVQSLTTSSNSQAQ 490

M1_Tlp3 FRNKLENASGSVELTTNALGDEIVKMLKQSSDFANALANESGKLQTAVQSLTTSSNSQAQ 490

4031_Tlp3 FRNKLENASGSVELTTNALGDEIVKMLKQSSDFANALANESGKLQTAVQSLTTSSNSQAQ 490

R14_Tlp3 FRNKLENASGSVELTTNALGNEIVKMLKQSSDFANALANESGKLQTAVQSLTTSSNSQAQ 490

RM5611_Tlp3 FRNKLDNANGSVEVTTNALGDEIVKMLKQSSDFANHLASESSKLQSAVQNLTSSSNSQAA 490

MTVDSCj16_Tlp3 FRNKLENASGSVELTTNALGDEIVKMLKQSSDFANALANESGKLQTAVQSLTTSSNSQAQ 490

01-1512_Tlp3 FRNKLENASGSVELTTNALGDEIVKMLKQSSDFANALANESGKLQTAVQSLTTSSNSQAQ 490

MTVDSCj13_Tlp3 FRNKLENASGSVELTTNALGDEIVKMLKQSSDFANALANESGKLQTAVQSLTTSSNSQAQ 490

32488_Tlp3a FRNKLENASGSVELTTNALGDEIVKMLKQSSDFANALANESGKLQTAVQSLTTSSNSQAQ 490

81116_Tlp3 FRNKLENASGSVELTTNALGDEIVKMLKQSSDFANALANESGKLQTAVQSLTTSSNSQAQ 490

32488_Tlp3b FRNKLENASGSVELTTNALGDEIVKMLKQSSDFANALANESGKLQTAVQSLTTSSNSQAQ 490

FB1_Tlp3 FRNKLENASGSVELTTNALGDEIVKMLKQSSDFANALANESGKLQTAVQSLTTSSNSQAQ 490

PT14_Tlp3 FRNKLENASGSVELTTNALGDEIVKMLKQSSDFANALANESGKLQTAVQSLTTSSNSQAQ 490

00-6200_Tlp3a FRNKLENASGSVELTTNALGDEIVKMLKQSSDFANALANESGKLQTAVQSLTTSSNSQAQ 490

RM1221_Tlp3 FRNKLENASGSVELTTNALGDEIVKMLKQSSDFANALANESGKLQTAVQSLTTSSNSQAQ 490

S3_Tlp3 FRNKLENASGSVELTTNALGDEIVKMLKQSSDFANALANESGKLQTAVQSLTTSSNSQAQ 490

FDAARGOS_421_Tlp3 FRNKLENASGSVELTTNALGDEIVKMLKQSSDFANALANESGKLQTAVQSLTTSSNSQAQ 501

CFSAN032806_Tlp3 FRNKLENASGSVELTTNALGDEIVKMLKQSSDFANALANESGKLQTAVQSLTTSSNSQAQ 501

IA3901_Tlp3b FRNKLENASGSVELTTNALGDEIVKMLKQSSDFANALANESGKLQTAVQSLTTSSNSQAQ 490

00-6200_Tlp3b FRNKLENASGSVELTTNALGDEIVKMLKQSSDFANALANESGKLQTAVQSLTTSSNSQAQ 490

BCW_6290_Tlp3b FRNKLENASGSVELTTNALGDEIVKMLKQSSDFANALANESGKLQTAVQSLTTSSNSQAQ 490

00-2425_Tlp3a FRNKLENASGSVELTTNALGDEIVKMLKQSSDFANALANESGKLQTAVQSLTTSSNSQAQ 490

00-2425_Tlp3b FRNKLENASGSVELTTNALGDEIVKMLKQSSDFANALANESGKLQTAVQSLTTSSNSQAQ 490

YH001_Tlp3a FRNKLENASGSVELTTNALGDEIVKMLKQSSDFANALANESGKLQTAVQSLTTSSNSQAQ 490

YH001_Tlp3b FRNKLENASGSVELTTNALGDEIVKMLKQSSDFANALANESGKLQTAVQSLTTSSNSQAQ 490

00-0949_Tlp3 FRNKLENASGSVELTTNALGDEIVKMLKQSSDFANALANESGKLQTAVQSLTTSSNSQAQ 490

NCTC11168_Tlp3 FRNKLENASGSVELTTNALGDEIVKMLKQSSDFANALANESGKLQTAVQSLTTSSNSQAQ 501

F38011_Tlp3 FRNKLENASGSVELTTNALGDEIVKMLKQSSDFANALANESGKLQTAVQSLTTSSNSQAQ 490

RM1285_Tlp3 FRNKLENASGSVELTTNALGDEIVKMLKQSSDFANALANESGKLQTAVQSLTTSSNSQAQ 490

FDAARGOS_422_Tlp3 FRNKLENASGSVELTTNALGDEIVKMLKQSSDFANALANESGKLQTAVQSLTTSSNSQAQ 501

MTVDSCj07_Tlp3 FRNKLENASGSVELTTNALGDEIVKMLKQSSDFANALANESGKLQTAVQSLTTSSNSQAQ 490

IA3901_Tlp3a FRNKLENASGSVELTTNALGDEIVKMLKQSSDFANALANESGKLQTAVQSLTTSSNSQAQ 490

BCW_6290_Tlp3a FRNKLENASGSVELTTNALGDEIVKMLKQSSDFANALANESGKLQTAVQSLTTSSNSQAQ 490

35925B2_Tlp3 FTTEVKDANGAVEVTTNALGQEIIKMLKQSSDFANALANESGKLQTAVQSLTTSSNSQAQ 504

14980A_Tlp3 FTTEVKDANGAVEVTTNALGQEIIKMLKQSSDFANALANESGKLQTAVQSLTTSSNSQAQ 501

00-1597_Tlp3b FTTEVKDANGAVEVTTNALGQEIIKMLKQSSDFANALANESGKLQTAVQSLTTSSNSQAQ 490

RM3196_Tlp3 FTTEVKDANGAVEVTTNALGQEIIKMLKQSSDFANALANESGKLQTAVQSLTTSSNSQAQ 490

FDAARGOS_295_Tlp21 FTTEVKDANGAVELTTNALGDEIIKMLKQSSDFANALANESGKLQTAVQSLTTSSNSQAQ 492

FORC_046_Tlp4 FTTEVKDANGAVEVTTNALGQEIIKMLKQSSDFANALANESGKLQTAVQSLTTSSNSQAQ 504

FDAARGOS_422_Tlp4 FTTEVKDANGAVEVTTNALGQEIIKMLKQSSDFANALANESGKLQTAVQSLTTSSNSQAQ 504

ICDCCJ07001_Tlp4 FRNKLDNANGSVEVTTNALGDEIVKMLKQSSDFANHLASESSKLQSAVQNLTSSSNSQAA 504

RM3196_Tlp4 FRNKLDNANGSVEVTTNALGDEIVKMLKQSSDFANHLASESSKLQSAVQNLTSSSNSQAA 504

T1-21_Tlp4 FRNKLENASGSVELTTNALGDEIVKMLKQSSDFANALANESGKLQTAVQSLTTSSNSQAQ 504

F38011_Tlp4 FRNKLENASGSVELTTNALGDEIVKMLKQSSDFANALANESGKLQTAVQSLTTSSNSQAQ 504

HF5-4A-4_Tlp22 FRNKLENASGSVELTTNALGDEIVKMLKQSSDFANALANESGKLQTAVQSLTTSSNSQAQ 450

00-0949_Tlp4 FRNKLENASGSVELTTNALGDEIVKMLKQSSDFANALANESGKLQTAVQSLTTSSNSQAQ 503

01-1512_Tlp4 FRNKLENASGSVELTTNALGDEIVKMLKQSSDFANALANESGKLQTAVQSLTTSSNSQAQ 504

81-176_Tlp4 FRNKLENASGSVELTTNALGDEIVKMLKQSSDFANALANESGKLQTAVQSLTTSSNSQAQ 504

32488_Tlp4 FRNKLENASGSVELTTNALGDEIVKMLKQSSDFANALANESGKLQTAVQSLTTSSNSQAQ 504

NCTC11168_Tlp4 FRNKLENASGSVELTTNALGDEIVKMLKQSSDFANALANESGKLQTAVQSLTTSSNSQAQ 504

CFSAN032806_Tlp4 FRNKLENASGSVELTTNALGDEIVKMLKQSSDFANALANESGKLQTAVQSLTTSSNSQAQ 504

81116_Tlp4 FRNKLENAGGSVELTTNALGDEIVKMLKQSSDFANALANESGKLQTAVQSLTTSSNSQAQ 504

RM1285_Tlp12 FRNKLENASGSVELTTNALGDEIVKMLKQSSDFANALANESGKLQTAVQSLTTSSNSQAQ 500

PT14_Tlp12 FRNKLENASGSVELTTNALGDEIVKMLKQSSDFANALANESGKLQTAVQSLTTSSNSQAQ 501

MTVJDCj07_Tlp12 FRNKLENASGSVELTTNALGDEIVKMLKQSSDFANALANESGKLQTAVQSLTTSSNSQAQ 501

RM1221_Tlp12 FRNKLENASGSVELTTNALGDEIVKMLKQSSDFANALANESGKLQTAVQSLTTSSNSQAQ 501

FDAARGOS_421_Tlp12 FRNKLENASGSVELTTNALGDEIVKMLKQSSDFANALANESGKLQTAVQSLTTSSNSQAQ 501

35925B2_Tlp12 FTTEVKDANGAVEVTTNALGQEIIKMLKQSSDFANALANESGKLQTAVQSLTTSSNSQAQ 501

CJM1cam_Tlp12 FTTEVKDANGAVEVTTNALGQEIIKMLKQSSDFANALANESGKLQTAVQSLTTSSNSQAQ 501

M1_Tlp12 FTTEVKDANGAVEVTTNALGQEIIKMLKQSSDFANALANESGKLQTAVQSLTTSSNSQAQ 501

S3_Tlp12 FTTEVKDANGAVEVTTNALGQEIIKMLKQSSDFANALANESGKLQTAVQSLTTSSNSQAQ 501

00-1597_Tlp12 FTTEVKDANGAVEVTTNALGQEIIKMLKQSSDFANALANESGKLQTAVQSLTTSSNSQAQ 501

R14_Tlp12 FTTEVKDANGAVEVTTNALGQEIIKMLKQSSDFANALANESGKLQTAVQSLTTSSNSQAQ 501

RM1875_Tlp15 FRNKLDNANGSVEVTTNALGDEIVKMLKQSSDFANHLASESSKLQSAVQNLTSSSNSQAA 418

YH503_Tlp16 FRNKLDNANGSVEVTTNALGDEIVKMLKQSSDFANHLASESSKLQSAVQNLTSSSNSQAA 418

FB1_Tlp16 FRNKLDNANGSVEVTTNALGDEIVKMLKQSSDFANHLASESSKLQSAVQNLTSSSNSQAA 418

BFR-CA-9557_Tlp16 FRNKLDNANGSVEVTTNALGDEIVKMLKQSSDFANHLASESSKLQSAVQNLTSSSNSQAA 417

15-537360_Tlp16 FRNKLDNANGSVEVTTNALGDEIVKMLKQSSDFANHLASESSKLQSAVQNLTSSSNSQAA 418

OR12_Tlp16 FRNKLDNANGSVEVTTNALGDEIVKMLKQSSDFANHLASESSKLQSAVQNLTSSSNSQAA 418

YH502_Tlp16 FRNKLDNANGSVEVTTNALGDEIVKMLKQSSDFANHLASESSKLQSAVQNLTSSSNSQAA 418

14903A_Tlp16 FRNKLDNANGSVEVTTNALGDEIVKMLKQSSDFANHLASESSKLQSAVQNLTSSSNSQAA 418

RM5611_Tlp16 FRNKLDNANGSVEVTTNALGDEIVKMLKQSSDFANHLASESSKLQSAVQNLTSSSNSQAA 418

00-2425_Tlp11 FRNKLENASGSVELTTNALGDEIVKMLKQSSDFANALANESGKLQTAVQSLTTSSNSQAQ 545

00-6200_Tlp11 FRNKLENASGSVELTTNALGDEIVKMLKQSSDFANALANESGKLQTAVQSLTTSSNSQAQ 545

YH001_Tlp11 FRNKLENASGSVELTTNALGDEIVKMLKQSSDFANALANESGKLQTAVQSLTTSSNSQAQ 545

IA3902_Tlp11 FTTEVKDANGAVELTTNALGDEIVKMLKQSSDFANALANESGKLQTAVQSLTTSSNSQAQ 545

BCW_6290_Tlp11 FTTEVKDANGAVELTTNALGDEIVKMLKQSSDFANALANESGKLQTAVQSLTTSSNSQAQ 545

76339_Tlp18 FRNKLDNANGSVEVTTNALGDEIVKMLKQSSDFANHLASESSKLQSAVQNLTSSSNSQAA 544

4031_Tlp17 FRNKLENASGSVELTTNALGDEIVKMLKQSSDFANALANESGKLQTAVQSLTTSSNSQAQ 545

15-537360_Tlp13 FRNKLDNANGSVEVTTNALGDEIVKMLKQSSDFANHLASESSKLQSAVQNLTSSSNSQAA 544

CVM_N29710_Tlp13 FRNKLDNANGSVEVTTNALGDEIVKMLKQSSDFANHLASESSKLQSAVQNLTSSSNSQAA 544

FB1_Tlp13 FRNKLDNANGSVEVTTNALGDEIVKMLKQSSDFANHLASESSKLQSAVQNLTSSSNSQAA 544

CFCAN032805_Tlp13 FRNKLDNANGSVEVTTNALGDEIVKMLKQSSDFANHLASESSKLQSAVQNLTSSSNSQAA 544

BG2108_Tlp13 FRNKLDNANGSVEVTTNALGDEIVKMLKQSSDFANHLASESSKLQSAVQNLTSSSNSQAA 544

YF2105_Tlp13 FRNKLDNANGSVEVTTNALGDEIVKMLKQSSDFANHLASESSKLQSAVQNLTSSSNSQAA 544

YH503_Tlp13 FRNKLDNANGSVEVTTNALGDEIVKMLKQSSDFANHLASESSKLQSAVQNLTSSSNSQAA 544

BFRCA9557_Tlp13 FRNKLDNANGSVEVTTNALGDEIVKMLKQSSDFANHLASESSKLQSAVQNLTSSSNSQAA 544

YH502_Tlp13 FRNKLDNANGSVEVTTNALGDEIVKMLKQSSDFANHLASESSKLQSAVQNLTSSSNSQAA 544

MTVDSCj13_Tlp13 FRNKLENASGSVELTTNALGDEIVKMLKQSSDFANALANESGKLQTAVQSLTTSSNSQAQ 544

OR12_Tlp13 FTTEVKDANGAVEVTTNALGQEIIKMLKQSSDFANALANESSKLQTAVQSLTTSSNSQAQ 544

00-1597_Tlp13 FTTEVKDANGAVEVTTNALGQEIIKMLKQSSDFANALANESGKLQTAVQSLTTSSNSQAQ 544

14903A_Tlp13 FTTEVKDANGAVEVTTNALGQEIIKMLKQSSDFANALANESGKLQTAVQSLTTSSNSQAQ 544

FJ3124_Tlp13 FRNKLENASGSVELTTNALGDEIVKMLKQSSDFANALANESGKLQTAVQSLTTSSNSQAQ 544

R14_Tlp13 FRNKLENASGSVELTTNALGNEIVKMLKQSSDFANALANESGKLQTAVQSLTTSSNSQAQ 544

MTVDSCj16_Tlp13 FRNKLENASGSVELTTNALGDEIVKMLKQSSDFANALANESGKLQTAVQSLTTSSNSQAQ 544

14980A_Tlp13 FRNKLENASGSVELTTNALGDEIVKMLKQSSDFANALANESGKLQTAVQSLTTSSNSQAQ 544

* .::.:*.*:**:****** :::*********** **.**.***:***.**:******

15-537360_Tlp20 SLEETAAALEEITSSMQNVSVKTSDVITQSEEIKNVTGIIGDIADQINLLALNAAIEAAR 558

76339_Tlp20 SLEETAAALEEITSSMQNVSVKTSDVITQSEEIKNVTGIIGDIADQINLLALNAAIEAAR 558

CFSAN032805_Tlp20 SLEETAAALEEITSSMQNVSVKTSDVITQSEEIKNVTGIIGDIADQINLLALNAAIEAAR 557

CVM_N29710_Tlp20 SLEETAAALEEITSSMQNVSVKTSDVITQSEEIKNVTGIIGDIADQINLLALNAAIEAAR 556

YH501_Tlp20 SLEETAAALEEITSSMQNVSVKTSDVITQSEEIKNVTGIIGDIADQINLLALNAAIEAAR 560

CO2-160_Tlp20 SLEETAAALEEITSSMQNVSVKTSDVITQSEEIKNVTGIIGDIADQINLLALNAAIEAAR 558

CO2-160_Tlp20b SLEETAAALEEITSSMQNVSVKTSDVITQSEEIKNVTGIIGDIADQINLLALNAAIEAAR 558

RM5611_Tlp20 SLEETAAALEEITSSMQNVSVKTSDVITQSEEIKNVTGIIGDIADQINLLALNAAIEAAR 558

14903A_Tlp20 SLEETAAALEEITSSMQNVSVKTSDVITQSEEIKNVTGIIGDIADQINLLALNAAIEAAR 560

YH502_Tlp20 SLEETAAALEEITSSMQNVSVKTSDVITQSEEIKNVTGIIGDIADQINLLALNAAIEAAR 557

RM3196_Tlp23 SLEETAAALEEITSSMQNVSVKTSDVITQSEEIKNVTGIIGDIADQINLLALNAAIEAAR 558

ICDCCJ07001_Tlp23 SLEETAAALEEITSSMQNVSVKTSDVITQSEEIKNVTGIIGDIADQINLLALNAAIEAAR 558

RM1285_Tlp2 SLEETAAALEEITSSMQNVSVKTSDVITQSEEIKNVTGIIGDIADQINLLALNAAIEAAR 557

CFSAN032806_Tlp2 SLEETAAALEEITSSMQNVSVKTSDVITQSEEIKNVTGIIGDIADQINLLALNAAIEAAR 558

RM1221_Tlp2 SLEETAAALEEITSSMQNVSVKTSDVITQSEEIKNVTGIIGDIADQINLLALNAAIEAAR 558

S3_Tlp2 SLEETAAALEEITSSMQNVSVKTSDVITQSEEIKNVTGIIGDIADQINLLALNAAIEAAR 558

FDAARGOS_422_Tlp2 SLEETAAALEEITSSMQNVSVKTSDVITQSEEIKNVTGIIGDIADQINLLALNAAIEAAR 558

81-176_Tlp2 SLEETAAALEEITSSMQNVSVKTSDVITQSEEIKNVTGIIGDIADQINLLALNAAIEAAR 558

F38011_Tlp2 SLEETAAALEEITSSMQNVSVKTSDVITQSEEIKNVTGIIGDIADQINLLALNAAIEAAR 558

NCTC11168_Tlp2 SLEETAAALEEITSSMQNVSVKTSDVITQSEEIKNVTGIIGDIADQINLLALNAAIEAAR 558

MTVDSCj07_Tlp2 SLEETAAALEEITSSMQNVSVKTSDVITQSEEIKNVTGIIGDIADQINLLALNAAIEAAR 558

CJM1cam_Tlp24 SLEETAAALEEITSSMQNVSVKTSDVITQSEEIKNVTGIIGDIADQINLLALNAAIEAAR 555

M1_Tlp24 SLEETAAALEEITSSMQNVSVKTSDVITQSEEIKNVTGIIGDIADQINLLALNAAIEAAR 554

81116_Tlp2 SLEETAAALEEITSSMQNVSVKTSDVITQSEEIKNVTGIIGDIADQINLLALNAAIEAAR 558

4031_Tlp23 SLEETAAALEEITSSMQNVSVKTSDVITQSEEIKNVTGIIGDIADQINLLALNAAIEAAR 557

MG1116_Tlp14 SLEETAAALEEITSSMQNVSVKTSDVITQSEEIKNVTGIIGDIADQINLLALNAAIEAAR 548

YH502_Tlp14 SLEETAAALEEITSSMQNVSVKTSDVITQSEEIKNVTGIIGDIADQINLLALNAAIEAAR 566

BP3181_Tlp14 SLEETAAALEEITSSMQNVSVKTSDVITQSEEIKNVTGIIGDIADQINLLALNAAIEAAR 555

ZV1224_Tlp14a SLEETAAALEEITSSMQNVSVKTSDVITQSEEIKNVTGIIGDIADQINLLALNAAIEAAR 555

ZV1224_Tlp14b SLEETAAALEEITSSMQNVSVKTSDVITQSEEIKNVTGIIGDIADQINLLALNAAIEAAR 555

YH503_Tlp14 SLEETAAALEEITSSMQNVSVKTSDVITQSEEIKNVTGIIGDIADQINLLALNAAIEAAR 566

14903A_Tlp14 SLEETAAALEEITSSMQNVSVKTSDVITQSEEIKNVTGIIGDIADQINLLALNAAIEAAR 555

OR12_Tlp14 SLEETAAALEEITSSMQNVSVKTSDVITQSEEIKNVTGIIGDIADQINLLALNAAIEAAR 555

CFSAN032805_Tlp14 SLEETAAALEEITSSMQNVSVKTSDVITQSEEIKNVTGIIGDIADQINLLALNAAIEAAR 566

BFR-CA-9557_Tlp14 SLEETAAALEEITSSMQNVSVKTSDVITQSEEIKNVTGIIGDIADQINLLALNAAIEAAR 555

15-537360_Tlp14 SLEETAAALEEITSSMQNVSVKTSDVITQSEEIKNVTGIIGDIADQINLLALNAAIEAAR 555

YH501_Tlp14 SLEETAAALEEITSSMQNVSVKTSDVITQSEEIKNVTGIIGDIADQINLLALNAAIEAAR 555

CG8421_Tlp14 SLEETAAALEEITSSMQNVSVKTSDVITQSEEIKNVTGIIGDIADQINLLALNAAIEAAR 549

MTVDSCj16_Tlp14 SLEETAAALEEITSSMQNVSVKTSDVITQSEEIKNVTGIIGDIADQINLLALNAAIEAAR 555

01-1512_Tlp14 SLEETAAALEEITSSMQNVSVKTSDVITQSEEIKNVTGIIGDIADQINLLALNAAIEAAR 555

00-0949_Tlp14 SLEETAAALEEITSSMQNVSVKTSDVITQSEEIKNVTGIIGDIADQINLLALNAAIEAAR 555

MTVDSCj13_Tlp14 SLEETAAALEEITSSMQNVSVKTSDVITQSEEIKNVTGIIGDIADQINLLALNAAIEAAR 555

S3_Tlp14 SLEETAAALEEITSSMQNVSVKTSDVITQSEEIKNVTGIIGDIADQINLLALNAAIEAAR 555

PT14_Tlp14 SLEETAAALEEITSSMQNVSVKTSDVITQSEEIKNVTGIIGDIADQINLLALNAAIEAAR 555

14980A_Tlp14 SLEETAAALEEITSSMQNVSVKTSDVITQSEEIKNVTGIIGDIADQINLLALNAAIEAAR 571

FJ3124_Tlp14 SLEETAAALEEITSSMQNVSVKTSDVITQSEEIKNVTGIIGDIADQINLLALNAAIEAAR 555

00-1597_Tlp14 SLEETAAALEEITSSMQNVSVKTSDVITQSEEIKNVTGIIGDIADQINLLALNAAIEAAR 555

R14_Tlp14 SLEETAAALEEITSSMQNVSVKTSDVITQSEEIKNVTGIIGDIADQINLLALNAAIEAAR 555

CG8421_Tlp25 SLEETAAALEEITSSMQNVSVKTSDVITQSEEIKNVTGIIGDIADQINLLALNAAIEAAR 346

RM1875_Tlp3 SLEETAAALEEITSSMQNVSVKTSDVITQSEEIKNVTGIIGDIADQINLLALNAAIEAAR 550

CJM1cam_Tlp3 SLEETAAALEEITSSMQNVSVKTSDVITQSEEIKNVTGIIGDIADQINLLALNAAIEAAR 550

M1_Tlp3 SLEETAAALEEITSSMQNVSVKTSDVITQSEEIKNVTGIIGDIADQINLLALNAAIEAAR 550

4031_Tlp3 SLEETAAALEEITSSMQNVSVKTSDVITQSEEIKNVTGIIGDIADQINLLALNAAIEAAR 550

R14_Tlp3 SLEETAAALEEITSSMQNVSVKTSDVITQSEEIKNVTGIIGDIADQINLLALNAAIEAAR 550

RM5611_Tlp3 SLEETAAALEEITSSMQNVSVKTSDVITQSEEIKNVTGIIGDIADQINLLALNAAIEAAR 550

MTVDSCj16_Tlp3 SLEETAAALEEITSSMQNVSVKTSDVITQSEEIKNVTGIIGDIADQINLLALNAAIEAAR 550

01-1512_Tlp3 SLEETAAALEEITSSMQNVSVKTSDVITQSEEIKNVTGIIGDIADQINLLALNAAIEAAR 550

MTVDSCj13_Tlp3 SLEETAAALEEITSSMQNVSVKTSDVITQSEEIKNVTGIIGDIADQINLLALNAAIEAAR 550

32488_Tlp3a SLEETAAALEEITSSMQNVSVKTSDVITQSEEIKNVTGIIGDIADQINLLALNAAIEAAR 550

81116_Tlp3 SLEETAAALEEITSSMQNVSVKTSDVITQSEEIKNVTGIIGDIADQINLLALNAAIEAAR 550

32488_Tlp3b SLEETAAALEEITSSMQNVSVKTSDVITQSEEIKNVTGIIGDIADQINLLALNAAIEAAR 550

FB1_Tlp3 SLEETAAALEEITSSMQNVSVKTSDVITQSEEIKNVTGIIGDIADQINLLALNAAIEAAR 550

PT14_Tlp3 SLEETAAALEEITSSMQNVSVKTSDVITQSEEIKNVTGIIGDIADQINLLALNAAIEAAR 550

00-6200_Tlp3a SLEETAAALEEITSSMQNVSVKTSDVITQSEEIKNVTGIIGDIADQINLLALNAAIEAAR 550

RM1221_Tlp3 SLEETAAALEEITSSMQNVSVKTSDVITQSEEIKNVTGIIGDIADQINLLALNAAIEAAR 550

S3_Tlp3 SLEETAAALEEITSSMQNVSVKTSDVITQSEEIKNVTGIIGDIADQINLLALNAAIEAAR 550

FDAARGOS_421_Tlp3 SLEETAAALEEITSSMQNVSVKTSDVITQSEEIKNVTGIIGDIADQINLLALNAAIEAAR 561

CFSAN032806_Tlp3 SLEETAAALEEITSSMQNVSVKTSDVITQSEEIKNVTGIIGDIADQINLLALNAAIEAAR 561

IA3901_Tlp3b SLEETAAALEEITSSMQNVSVKTSDVITQSEEIKNVTGIIGDIADQINLLALNAAIEAAR 550

00-6200_Tlp3b SLEETAAALEEITSSMQNVSVKTSDVITQSEEIKNVTGIIGDIADQINLLALNAAIEAAR 550

BCW_6290_Tlp3b SLEETAAALEEITSSMQNVSVKTSDVITQSEEIKNVTGIIGDIADQINLLALNAAIEAAR 550

00-2425_Tlp3a SLEETAAALEEITSSMQNVSVKTSDVITQSEEIKNVTGIIGDIADQINLLALNAAIEAAR 550

00-2425_Tlp3b SLEETAAALEEITSSMQNVSVKTSDVITQSEEIKNVTGIIGDIADQINLLALNAAIEAAR 550

YH001_Tlp3a SLEETAAALEEITSSMQNVSVKTSDVITQSEEIKNVTGIIGDIADQINLLALNAAIEAAR 550

YH001_Tlp3b SLEETAAALEEITSSMQNVSVKTSDVITQSEEIKNVTGIIGDIADQINLLALNAAIEAAR 550

00-0949_Tlp3 SLEETAAALEEITSSMQNVSVKTSDVITQSEEIKNVTGIIGDIADQINLLALNAAIEAAR 550

NCTC11168_Tlp3 SLEETAAALEEITSSMQNVSVKTSDVITQSEEIKNVTGIIGDIADQINLLALNAAIEAAR 561

F38011_Tlp3 SLEETAAALEEITSSMQNVSVKTSDVITQSEEIKNVTGIIGDIADQINLLALNAAIEAAR 550

RM1285_Tlp3 SLEETAAALEEITSSMQNVSVKTSDVITQSEEIKNVTGIIGDIADQINLLALNAAIEAAR 550

FDAARGOS_422_Tlp3 SLEETAAALEEITSSMQNVSVKTSDVITQSEEIKNVTGIIGDIADQINLLALNAAIEAAR 561

MTVDSCj07_Tlp3 SLEETAAALEEITSSMQNVSVKTSDVITQSEEIKNVTGIIGDIADQINLLALNAAIEAAR 550

IA3901_Tlp3a SLEETAAALEEITSSMQNVSVKTSDVITQSEEIKNVTGIIGDIADQINLLALNAAIEAAR 550

BCW_6290_Tlp3a SLEETAAALEEITSSMQNVSVKTSDVITQSEEIKNVTGIIGDIADQINLLALNAAIEAAR 550

35925B2_Tlp3 SLEETAAALEEITSSMQNVSVKTSDVITQSEEIKNVTGIIGDIADQINLLALNAAIEAAR 564

14980A_Tlp3 SLEETAAALEEITSSMQNVSVKTSDVITQSEEIKNVTGIIGDIADQINLLALNAAIEAAR 561

00-1597_Tlp3b SLEETAAALEEITSSMQNVSVKTSDVITQSEEIKNVTGIIGDIADQINLLALNAAIEAAR 550

RM3196_Tlp3 SLEETAAALEEITSSMQNVSVKTSDVITQSEEIKNVTGIIGDIADQINLLALNAAIEAAR 550

FDAARGOS_295_Tlp21 SLEETAAALEEITSSMQNVSVKTSDVITQSEEIKNVTGIIGDIADQINLLALNAAIEAAR 552

FORC_046_Tlp4 SLEETAAALEEITSSMQNVSVKTSDVITQSEEIKNVTGIIGDIADQINLLALNAAIEAAR 564

FDAARGOS_422_Tlp4 SLEETAAALEEITSSMQNVSVKTSDVITQSEEIKNVTGIIGDIADQINLLALNAAIEAAR 564

ICDCCJ07001_Tlp4 SLEETAAALEEITSSMQNVSVKTSDVITQSEEIKNVTGIIGDIADQINLLALNAAIEAAR 564

RM3196_Tlp4 SLEETAAALEEITSSMQNVSVKTSDVITQSEEIKNVTGIIGDIADQINLLALNAAIEAAR 564

T1-21_Tlp4 SLEETAAALEEITSSMQNVSVKTSDVITQSEEIKNVTGIIGDIADQINLLALNAAIEAAR 564

F38011_Tlp4 SLEETAAALEEITSSMQNVSVKTSDVITQSEEIKNVTGIIGDIADQINLLALNAAIEAAR 564

HF5-4A-4_Tlp22 SLEETAAALEEITSSMQNVSVKTSDVITQSEEIKNVTGIIGDIADQINLLALNAAIEAAR 510

00-0949_Tlp4 SLEETAAALEEITSSMQNVSVKTSDVITQSEEIKNVTGIIGDIADQINLLALNAAIEAAR 563

01-1512_Tlp4 SLEETAAALEEITSSMQNVSVKTSDVITQSEEIKNVTGIIGDIADQINLLALNAAIEAAR 564

81-176_Tlp4 SLEETAAALEEITSSMQNVSVKTSDVITQSEEIKNVTGIIGDIADQINLLALNAAIEAAR 564

32488_Tlp4 SLEETAAALEEITSSMQNVSVKTSDVITQSEEIKNVTGIIGDIADQINLLALNAAIEAAR 564

NCTC11168_Tlp4 SLEETAAALEEITSSMQNVSVKTSDVITQSEEIKNVTGIIGDIADQINLLALNAAIEAAR 564

CFSAN032806_Tlp4 SLEETAAALEEITSSMQNVSVKTSDVITQSEEIKNVTGIIGDIADQINLLALNAAIEAAR 564

81116_Tlp4 SLEETAAALEEITSSMQNVSVKTSDVITQSEEIKNVTGIIGDIADQINLLALNAAIEAAR 564

RM1285_Tlp12 SLEETAAALEEITSSMQNVSVKTSDVITQSEEIKNVTGIIGDIADQINLLALNAAIEAAR 560

PT14_Tlp12 SLEETAAALEEITSSMQNVSVKTSDVITQSEEIKNVTGIIGDIADQINLLALNAAIEAAR 561

MTVJDCj07_Tlp12 SLEETAAALEEITSSMQNVSVKTSDVITQSEEIKNVTGIIGDIADQINLLALNAAIEAAR 561

RM1221_Tlp12 SLEETAAALEEITSSMQNVSVKTSDVITQSEEIKNVTGIIGDIADQINLLALNAAIEAAR 561

FDAARGOS_421_Tlp12 SLEETAAALEEITSSMQNVSVKTSDVITQSEEIKNVTGIIGDIADQINLLALNAAIEAAR 561

35925B2_Tlp12 SLEETAAALEEITSSMQNVSVKTSDVITQSEEIKNVTGIIGDIADQINLLALNAAIEAAR 561

CJM1cam_Tlp12 SLEETAAALEEITSSMQNVSVKTSDVITQSEEIKNVTGIIGDIADQINLLALNAAIEAAR 561

M1_Tlp12 SLEETAAALEEITSSMQNVSVKTSDVITQSEEIKNVTGIIGDIADQINLLALNAAIEAAR 561

S3_Tlp12 SLEETAAALEEITSSMQNVSVKTSDVITQSEEIKNVTGIIGDIADQINLLALNAAIEAAR 561

00-1597_Tlp12 SLEETAAALEEITSSMQNVSVKTSDVITQSEEIKNVTGIIGDIADQINLLALNAAIEAAR 561

R14_Tlp12 SLEETAAALEEITSSMQNVSVKTSDVITQSEEIKNVTGIIGDIADQINLLALNAAIEAAR 561

RM1875_Tlp15 SLEETAAALEEITSSMQNVSVKTSDVITQSEEIKNVTGIIGDIADQINLLALNAAIEAAR 478

YH503_Tlp16 SLEETAAALEEITSSMQNVSVKTSDVITQSEEIKNVTGIIGDIADQINLLALNAAIEAAR 478

FB1_Tlp16 SLEETAAALEEITSSMQNVSVKTSDVITQSEEIKNVTGIIGDIADQINLLALNAAIEAAR 478

BFR-CA-9557_Tlp16 SLEETAAALEEITSSMQNVSVKTSDVITQSEEIKNVTGIIGDIADQINLLALNAAIEAAR 477

15-537360_Tlp16 SLEETAAALEEITSSMQNVSVKTSDVITQSEEIKNVTGIIGDIADQINLLALNAAIEAAR 478

OR12_Tlp16 SLEETAAALEEITSSMQNVSVKTSDVITQSEEIKNVTGIIGDIADQINLLALNAAIEAAR 478

YH502_Tlp16 SLEETAAALEEITSSMQNVSVKTSDVITQSEEIKNVTGIIGDIADQINLLALNAAIEAAR 478

14903A_Tlp16 SLEETAAALEEITSSMQNVSVKTSDVITQSEEIKNVTGIIGDIADQINLLALNAAIEAAR 478

RM5611_Tlp16 SLEETAAALEEITSSMQNVSVKTSDVITQSEEIKNVTGIIGDIADQINLLALNAAIEAAR 478

00-2425_Tlp11 SLEETAAALEEITSSMQNVSVKTSDVITQSEEIKNVTGIIGDIADQINLLALNAAIEAAR 605

00-6200_Tlp11 SLEETAAALEEITSSMQNVSVKTSDVITQSEEIKNVTGIIGDIADQINLLALNAAIEAAR 605

YH001_Tlp11 SLEETAAALEEITSSMQNVSVKTSDVITQSEEIKNVTGIIGDIADQINLLALNAAIEAAR 605

IA3902_Tlp11 SLEETAAALEEITSSMQNVSVKTSDVITQSEEIKNVTGIIGDIADQINLLALNAAIEAAR 605

BCW_6290_Tlp11 SLEETAAALEEITSSMQNVSVKTSDVITQSEEIKNVTGIIGDIADQINLLALNAAIEAAR 605

76339_Tlp18 SLEETAAALEEITSSMQNVSVKLSDVITQSEEIKNVTGIIGDIADQINLLALNAAIEAAR 604

4031_Tlp17 SLEETAAALEEITSSMQNVSVKTSDVITQSEEIKNVTGIIGDIADQINLLALNAAIEAAR 605

15-537360_Tlp13 SLEETAAALEEITSSMQNVSVKTSDVITQSEEIKNVTGIIGDIADQINLLALNAAIEAAR 604

CVM_N29710_Tlp13 SLEETAAALEEITSSMQNVSVKTSDVITQSEEIKNVTGIIGDIADQINLLALNAAIEAAR 604

FB1_Tlp13 SLEETAAALEEITSSMQNVSVKTSDVITQSEEIKNVTGIIGDIADQINLLALNAAIEAAR 604

CFCAN032805_Tlp13 SLEETAAALEEITSSMQNVSVKTSDVITQSEEIKNVTGIIGDIADQINLLALNAAIEAAR 604

BG2108_Tlp13 SLEETAAALEEITSSMQNVSVKTSDVITQSEEIKNVTGIIGDIADQINLLALNAAIEAAR 604

YF2105_Tlp13 SLEETAAALEEITSSMQNVSVKTSDVITQSEEIKNVTGIIGDIADQINLLALNAAIEAAR 604

YH503_Tlp13 SLEETAAALEEITSSMQNVSVKTSDVITQSEEIKNVTGIIGDIADQINLLALNAAIEAAR 604

BFRCA9557_Tlp13 SLEETAAALEEITSSMQNVSVKTSDVITQSEEIKNVTGIIGDIADQINLLALNAAIEAAR 604

YH502_Tlp13 SLEETAAALEEITSSMQNVSVKTSDVITQSEEIKNVTGIIGDIADQINLLALNAAIEAAR 604

MTVDSCj13_Tlp13 SLEETAAALEEITSSMQNVSVKTSDVITQSEEIKNVTGIIGDIADQINLLALNAAIEAAR 604

OR12_Tlp13 SLEETAAALEEITSSMQNVSVKTSDVITQSEEIKNVTGIIGDIADQINLLALNAAIEAAR 604

00-1597_Tlp13 SLEETAAALEEITSSMQNVSVKTSDVITQSEEIKNVTGIIGDIADQINLLALNAAIEAAR 604

14903A_Tlp13 SLEETAAALEEITSSMQNVSVKTSDVITQSEEIKNVTGIIGDIADQINLLALNAAIEAAR 604

FJ3124_Tlp13 SLEETAAALEEITSSMQNVSVKTSDVITQSEEIKNVTGIIGDIADQINLLALNAAIEAAR 604

R14_Tlp13 SLEETAAALEEITSSMQNVSVKTSDVITQSEEIKNVTGIIGDIADQINLLALNAAIEAAR 604

MTVDSCj16_Tlp13 SLEETAAALEEITSSMQNVSVKTSDVITQSEEIKNVTGIIGDIADQINLLALNAAIEAAR 604

14980A_Tlp13 SLEETAAALEEITSSMQNVSVKTSDVITQSEEIKNVTGIIGDIADQINLLALNAAIEAAR 604

********************** *************************************

15-537360_Tlp20 AGEHGRGFAVVADEVRKLAERTQKSLSEIEANTNLLVQSINDMAESIKEQTAGITQINES 618

76339_Tlp20 AGEHGRGFAVVADEVRKLAERTQKSLSEIEANTNLLVQSINDMAESIKEQTAGITQINES 618

CFSAN032805_Tlp20 AGEHGRGFAVVADEVRKLAERTQKSLSEIEANTNLLVQSINDMAESIKEQTAGITQINES 617

CVM_N29710_Tlp20 AGEHGRGFAVVADEVRKLAERTQKSLSEIEANTNLLVQSINDMAESIKEQTAGITQINES 616

YH501_Tlp20 AGEHGRGFAVVADEVRKLAERTQKSLSEIEANTNLLVQSINDMAESIKEQTAGITQINES 620

CO2-160_Tlp20 AGEHGRGFAVVADEVRKLAERTQKSLSEIEANTNLLVQSINDMAESIKEQTAGITQINES 618

CO2-160_Tlp20b AGEHGRGFAVVADEVRKLAERTQKSLSEIEANTNLLVQSINDMAESIKEQTAGITQINES 618

RM5611_Tlp20 AGEHGRGFAVVADEVRKLAERTQKSLSEIEANTNLLVQSINDMAESIKEQTAGITQINES 618

14903A_Tlp20 AGEHGRGFAVVADEVRKLAERTQKSLSEIEANTNLLVQSINDMAESIKEQTAGITQINES 620

YH502_Tlp20 AGEHGRGFAVVADEVRKLAERTQKSLSEIEANTNLLVQSINDMAESIKEQTAGITQINES 617

RM3196_Tlp23 AGEHGRGFAVVADEVRKLAERTQKSLSEIEANTNLLVQSINDMAESIKEQTAGITQINDS 618

ICDCCJ07001_Tlp23 AGEHGRGFAVVADEVRKLAERTQKSLSEIEANTNLLVQSINDMAESIKEQTAGITQINDS 618

RM1285_Tlp2 AGEHGRGFAVVADEVRKLAERTQKSLSEIEANTNLLVQSINDMAESIKEQTAGITQINDS 617

CFSAN032806_Tlp2 AGEHGRGFAVVADEVRKLAERTQKSLSEIEANTNLLVQSINDMAESIKEQTAGITQINDS 618

RM1221_Tlp2 AGEHGRGFAVVADEVRKLAERTQKSLSEIEANTNLLVQSINDMAESIKEQTAGITQINDS 618

S3_Tlp2 AGEHGRGFAVVADEVRKLAERTQKSLSEIEANTNLLVQSINDMAESIKEQTAGITQINDS 618

FDAARGOS_422_Tlp2 AGEHGRGFAVVADEVRKLAERTQKSLSEIEANTNLLVQSINDMAESIKEQTAGITQINDS 618

81-176_Tlp2 AGEHGRGFAVVADEVRKLAERTQKSLSEIEANTNLLVQSINDMAESIKEQTAGITQINDS 618

F38011_Tlp2 AGEHGRGFAVVADEVRKLAERTQKSLSEIEANTNLLVQSINDMAESIKEQTAGITQINDS 618

NCTC11168_Tlp2 AGEHGRGFAVVADEVRKLAERTQKSLSEIEANTNLLVQSINDMAESIKEQTAGITQINDS 618

MTVDSCj07_Tlp2 AGEHGRGFAVVADEVRKLAERTQKSLSEIEANTNLLVQSINDMAESIKEQTAGITQINDS 618

CJM1cam_Tlp24 AGEHGRGFAVVADEVRKLAERTQKSLSEIEANTNLLVQSINDMAESIKEQTAGITQINES 615

M1_Tlp24 AGEHGRGFAVVADEVRKLAERTQKSLSEIEANTNLLVQSINDMAESIKEQTAGITQINES 614

81116_Tlp2 AGEHGRGFAVVADEVRKLAERTQKSLSEIEANTNLLVQSINDMAESIKEQTAGITQINES 618

4031_Tlp23 AGEHGRGFAVVADEVRKLAERTQKSLSEIEANTNLLVQSINDMAESIKEQTAGITQINES 617

MG1116_Tlp14 AGEHGRGFAVVADEVRKLAERTQKSLSEIEANTNLLVQSINDMAESIKEQTAGITQINES 608

YH502_Tlp14 AGEHGRGFAVVADEVRKLAERTQKSLSEIEANTNLLVQSINDMAESIKEQTAGITQINES 626

BP3181_Tlp14 AGEHGRGFAVVADEVRKLAERTQKSLSEIEANTNLLVQSINDMAESIKEQTAGITQINES 615

ZV1224_Tlp14a AGEHGRGFAVVADEVRKLAERTQKSLSEIEANTNLLVQSINDMAESIKEQTAGITQINES 615

ZV1224_Tlp14b AGEHGRGFAVVADEVRKLAERTQKSLSEIEANTNLLVQSINDMAESIKEQTAGITQINES 615

YH503_Tlp14 AGEHGRGFAVVADEVRKLAERTQKSLSEIEANTNLLVQSINDMAESIKEQTAGITQINES 626

14903A_Tlp14 AGEHGRGFAVVADEVRKLAERTQKSLSEIEANTNLLVQSINDMAESIKEQTAGITQINES 615

OR12_Tlp14 AGEHGRGFAVVADEVRKLAERTQKSLSEIEANTNLLVQSINDMAESIKEQTAGITQINES 615

CFSAN032805_Tlp14 AGEHGRGFAVVADEVRKLAERTQKSLSEIEANTNLLVQSINDMAESIKEQTAGITQINES 626

BFR-CA-9557_Tlp14 AGEHGRGFAVVADEVRKLAERTQKSLSEIEANTNLLVQSINDMAESIKEQTAGITQINES 615

15-537360_Tlp14 AGEHGRGFAVVADEVRKLAERTQKSLSEIEANTNLLVQSINDMAESIKEQTAGITQINES 615

YH501_Tlp14 AGEHGRGFAVVADEVRKLAERTQKSLSEIEANTNLLVQSINDMAESIKEQTAGITQINES 615

CG8421_Tlp14 AGEHGRGFAVVADEVRKLAERTQKSLSEIEANTNLLVQSINDMAESIKEQTAGITQINDS 609

MTVDSCj16_Tlp14 AGEHGRGFAVVADEVRKLAERTQKSLSEIEANTNLLVQSINDMAESIKEQTAGITQINDS 615

01-1512_Tlp14 AGEHGRGFAVVADEVRKLAERTQKSLSEIEANTNLLVQSINDMAESIKEQTAGITQINDS 615

00-0949_Tlp14 AGEHGRGFAVVADEVRKLAERTQKSLSEIEANTNLLVQSINDMAESIKEQTAGITQINDS 615

MTVDSCj13_Tlp14 AGEHGRGFAVVADEVRKLAERTQKSLSEIEANTNLLVQSINDMAESIKEQTAGITQINDS 615

S3_Tlp14 AGEHGRGFAVVADEVRKLAERTQKSLSEIEANTNLLVQSINDMAESIKEQTAGITQINDS 615

PT14_Tlp14 AGEHGRGFAVVADEVRKLAERTQKSLSEIEANTNLLVQSINDMAESIKEQTAGITQINDS 615

14980A_Tlp14 AGEHGRGFAVVADEVRKLAERTQKSLSEIEANTNLLVQSINDMAESIKEQTAGITQINDS 631

FJ3124_Tlp14 AGEHGRGFAVVADEVRKLAERTQKSLSEIEANTNLLVQSINDMAESIKEQTAGITQINDS 615

00-1597_Tlp14 AGEHGRGFAVVADEVRKLAERTQKSLSEIEANTNLLVQSINDMAESIKEQTAGITQINDS 615

R14_Tlp14 AGEHGRGFAVVADEVRKLAERTQKSLSEIEANTNLLVQSINDMAESIKEQTAGITQINDS 615

CG8421_Tlp25 AGEHGRGFAVVADEVRKLAERTQKSLSEIEANTNLLVQSINDMAESIKEQTAGITQINDS 406

RM1875_Tlp3 AGEHGRGFAVVADEVRKLAERTQKSLSEIEANTNLLVQSINDMAESIKEQTAGITQINES 610

CJM1cam_Tlp3 AGEHGRGFAVVADEVRKLAERTQKSLSEIEANTNLLVQSINDMAESIKEQTAGITQINES 610

M1_Tlp3 AGEHGRGFAVVADEVRKLAERTQKSLSEIEANTNLLVQSINDMAESIKEQTAGITQINES 610

4031_Tlp3 AGEHGRGFAVVADEVRKLAERTQKSLSEIEANTNLLVQSINDMAESIKEQTAGITQINES 610

R14_Tlp3 AGEHGRGFAVVADEVRKLAERTQKSLSEIEANTNLLVQSINDMAESIKEQTAGITQINDS 610

RM5611_Tlp3 AGEHGRGFAVVADEVRKLAERTQKSLSEIEANTNLLVQSINDMAESIKEQTAGITQINES 610

MTVDSCj16_Tlp3 AGEHGRGFAVVADEVRKLAERTQKSLSEIEANTNLLVQSINDMAESIKEQTAGITQINDS 610

01-1512_Tlp3 AGEHGRGFAVVADEVRKLAERTQKSLSEIEANTNLLVQSINDMAESIKEQTAGITQINDS 610

MTVDSCj13_Tlp3 AGEHGRGFAVVADEVRKLAERTQKSLSEIEANTNLLVQSINDMAESIKEQTAGITQINDS 610

32488_Tlp3a AGEHGRGFAVVADEVRKLAERTQKSLSEIEANTNLLVQSINDMAESIKEQTAGITQINDS 610

81116_Tlp3 AGEHGRGFAVVADEVRKLAERTQKSLSEIEANTNLLVQSINDMAESIKEQTAGITQINDS 610

32488_Tlp3b AGEHGRGFAVVADEVRKLAERTQKSLSEIEANTNLLVQSINDMAESIKEQTAGITQINDS 610

FB1_Tlp3 AGEHGRGFAVVADEVRKLAERTQKSLSEIEANTNLLVQSINDMAESIKEQTAGITQINES 610

PT14_Tlp3 AGEHGRGFAVVADEVRKLAERTQKSLSEIEANTNLLVQSINDMAESIKEQTAGITQINDS 610

00-6200_Tlp3a AGEHGRGFAVVADEVRKLAERTQKSLSEIEANTNLLVQSINDMAESIKEQTAGITQINDS 610

RM1221_Tlp3 AGEHGRGFAVVADEVRKLAERTQKSLSEIEANTNLLVQSINDMAESIKEQTAGITQINDS 610

S3_Tlp3 AGEHGRGFAVVADEVRKLAERTQKSLSEIEANTNLLVQSINDMAESIKEQTAGITQINDS 610

FDAARGOS_421_Tlp3 AGEHGRGFAVVADEVRKLAERTQKSLSEIEANTNLLVQSINDMAESIKEQTAGITQINDS 621

CFSAN032806_Tlp3 AGEHGRGFAVVADEVRKLAERTQKSLSEIEANTNLLVQSINDMAESIKEQTAGITQINDS 621

IA3901_Tlp3b AGEHGRGFAVVADEVRKLAERTQKSLSEIEANTNLLVQSINDMAESIKEQTAGITQINDS 610

00-6200_Tlp3b AGEHGRGFAVVADEVRKLAERTQKSLSEIEANTNLLVQSINDMAESIKEQTAGITQINDS 610

BCW_6290_Tlp3b AGEHGRGFAVVADEVRKLAERTQKSLSEIEANTNLLVQSINDMAESIKEQTAGITQINDS 610

00-2425_Tlp3a AGEHGRGFAVVADEVRKLAERTQKSLSEIEANTNLLVQSINDMAESIKEQTAGITQINDS 610

00-2425_Tlp3b AGEHGRGFAVVADEVRKLAERTQKSLSEIEANTNLLVQSINDMAESIKEQTAGITQINDS 610

YH001_Tlp3a AGEHGRGFAVVADEVRKLAERTQKSLSEIEANTNLLVQSINDMAESIKEQTAGITQINDS 610

YH001_Tlp3b AGEHGRGFAVVADEVRKLAERTQKSLSEIEANTNLLVQSINDMAESIKEQTAGITQINDS 610

00-0949_Tlp3 AGEHGRGFAVVADEVRKLAERTQKSLSEIEANTNLLVQSINDMAESIKEQTAGITQINDS 610

NCTC11168_Tlp3 AGEHGRGFAVVADEVRKLAERTQKSLSEIEANTNLLVQSINDMAESIKEQTAGITQINDS 621

F38011_Tlp3 AGEHGRGFAVVADEVRKLAERTQKSLSEIEANTNLLVQSINDMAESIKEQTAGITQINDS 610

RM1285_Tlp3 AGEHGRGFAVVADEVRKLAERTQKSLSEIEANTNLLVQSINDMAESIKEQTAGITQINDS 610

FDAARGOS_422_Tlp3 AGEHGRGFAVVADEVRKLAERTQKSLSEIEANTNLLVQSINDMAESIKEQTAGITQINDS 621

MTVDSCj07_Tlp3 AGEHGRGFAVVADEVRKLAERTQKSLSEIEANTNLLVQSINDMAESIKEQTAGITQINDS 610

IA3901_Tlp3a AGEHGRGFAVVADEVRKLAERTQKSLSEIEANTNLLVQSINDMAESIKEQTAGITQINDS 610

BCW_6290_Tlp3a AGEHGRGFAVVADEVRKLAERTQKSLSEIEANTNLLVQSINDMAESIKEQTAGITQINDS 610

35925B2_Tlp3 AGEHGRGFAVVADEVRKLAERTQKSLSEIEANTNLLVQSINDMAESIKEQTAGITQINES 624

14980A_Tlp3 AGEHGRGFAVVADEVRKLAERTQKSLSEIEANTNLLVQSINDMAESIKEQTAGITQINDS 621

00-1597_Tlp3b AGEHGRGFAVVADEVRKLAERTQKSLSEIEANTNLLVQSINDMAESIKEQTAGITQINDS 610

RM3196_Tlp3 AGEHGRGFAVVADEVRKLAERTQKSLSEIEANTNLLVQSINDMAESIKEQTAGITQINDS 610

FDAARGOS_295_Tlp21 AGEHGRGFAVVADEVRKLAERTQKSLSEIEANTNLLVQSINDMAESIKEQTAGITQINDS 612

FORC_046_Tlp4 AGEHGRGFAVVADEVRKLAERTQKSLSEIEANTNLLVQSINDMAESIKEQTAGITQINDS 624

FDAARGOS_422_Tlp4 AGEHGRGFAVVADEVRKLAERTQKSLSEIEANTNLLVQSINDMAESIKEQTAGITQINDS 624

ICDCCJ07001_Tlp4 AGEHGRGFAVVADEVRKLAERTQKSLSEIEANTNLLVQSINDMAESIKEQTAGITQINDS 624

RM3196_Tlp4 AGEHGRGFAVVADEVRKLAERTQKSLSEIEANTNLLVQSINDMAESIKEQTAGITQINDS 624

T1-21_Tlp4 AGEHGRGFAVVADEVRKLAERTQKSLSEIEANTNLLVQSINDMAESIKEQTAGITQINDS 624

F38011_Tlp4 AGEHGRGFAVVADEVRKLAERTQKSLSEIEANTNLLVQSINDMAESIKEQTAGITQINDS 624

HF5-4A-4_Tlp22 AGEHGRGFAVVADEVRKLAERTQKSLSEIEANTNLLVQSINDMAESIKEQTAGITQINDS 570

00-0949_Tlp4 AGEHGRGFAVVADEVRKLAERTQKSLSEIEANTNLLVQSINDMAESIKEQTAGITQINDS 623

01-1512_Tlp4 AGEHGRGFAVVADEVRKLAERTQKSLSEIEANTNLLVQSINDMAESIKEQTAGITQINDS 624

81-176_Tlp4 AGEHGRGFAVVADEVRKLAERTQKSLSEIEANTNLLVQSINDMAESIKEQTAGITQINDS 624

32488_Tlp4 AGEHGRGFAVVADEVRKLAERTQKSLSEIEANTNLLVQSINDMAESIKEQTAGITQINDS 624

NCTC11168_Tlp4 AGEHGRGFAVVADEVRKLAERTQKSLSEIEANTNLLVQSINDMAESIKEQTAGITQINDS 624

CFSAN032806_Tlp4 AGEHGRGFAVVADEVRKLAERTQKSLSEIEANTNLLVQSINDMAESIKEQTAGITQINDS 624

81116_Tlp4 AGEHGRGFAVVADEVRKLAERTQKSLSEIEANTNLLVQSINDMAESIKEQTAGITQINDS 624

RM1285_Tlp12 AGEHGRGFAVVADEVRKLAERTQKSLSEIEANTNLLVQSINDMAESIKEQTAGITQINDS 620

PT14_Tlp12 AGEHGRGFAVVADEVRKLAERTQKSLSEIEANTNLLVQSINDMAESIKEQTAGITQINDS 621

MTVJDCj07_Tlp12 AGEHGRGFAVVADEVRKLAERTQKSLSEIEANTNLLVQSINDMAESIKEQTAGITQINDS 621

RM1221_Tlp12 AGEHGRGFAVVADEVRKLAERTQKSLSEIEANTNLLVQSINDMAESIKEQTAGITQINDS 621

FDAARGOS_421_Tlp12 AGEHGRGFAVVADEVRKLAERTQKSLSEIEANTNLLVQSINDMAESIKEQTAGITQINDS 621

35925B2_Tlp12 AGEHGRGFAVVADEVRKLAERTQKSLSEIEANTNLLVQSINDMAESIKEQTAGITQINES 621

CJM1cam_Tlp12 AGEHGRGFAVVADEVRKLAERTQKSLSEIEANTNLLVQSINDMAESIKEQTAGITQINDS 621

M1_Tlp12 AGEHGRGFAVVADEVRKLAERTQKSLSEIEANTNLLVQSINDMAESIKEQTAGITQINDS 621

S3_Tlp12 AGEHGRGFAVVADEVRKLAERTQKSLSEIEANTNLLVQSINDMAESIKEQTAGITQINDS 621

00-1597_Tlp12 AGEHGRGFAVVADEVRKLAERTQKSLSEIEANTNLLVQSINDMAESIKEQTAGITQINDS 621

R14_Tlp12 AGEHGRGFAVVADEVRKLAERTQKSLSEIEANTNLLVQSINDMAESIKEQTAGITQINDS 621

RM1875_Tlp15 AGEHGRGFAVVADEVRKLAERTQKSLSEIEANTNLLVQSINDMAESIKEQTAGITQINES 538

YH503_Tlp16 AGEHGRGFAVVADEVRKLAERTQKSLSEIEANTNLLVQSINDMAESIKEQTAGITQINES 538

FB1_Tlp16 AGEHGRGFAVVADEVRKLAERTQKSLSEIEANTNLLVQSINDMAESIKEQTAGITQINES 538

BFR-CA-9557_Tlp16 AGEHGRGFAVVADEVRKLAERTQKSLSEIEANTNLLVQSINDMAESIKEQTAGITQINES 537

15-537360_Tlp16 AGEHGRGFAVVADEVRKLAERTQKSLSEIEANTNLLVQSINDMAESIKEQTAGITQINES 538

OR12_Tlp16 AGEHGRGFAVVADEVRKLAERTQKSLSEIEANTNLLVQSINDMAESIKEQTAGITQINES 538

YH502_Tlp16 AGEHGRGFAVVADEVRKLAERTQKSLSEIEANTNLLVQSINDMAESIKEQTAGITQINES 538

14903A_Tlp16 AGEHGRGFAVVADEVRKLAERTQKSLSEIEANTNLLVQSINDMAESIKEQTAGITQINES 538

RM5611_Tlp16 AGEHGRGFAVVADEVRKLAERTQKSLSEIEANTNLLVQSINDMAESIKEQTAGITQINES 538

00-2425_Tlp11 AGEHGRGFAVVADEVRKLAERTQKSLSEIEANTNLLVQSINDMAESIKEQTAGITQINDS 665

00-6200_Tlp11 AGEHGRGFAVVADEVRKLAERTQKSLSEIEANTNLLVQSINDMAESIKEQTAGITQINDS 665

YH001_Tlp11 AGEHGRGFAVVADEVRKLAERTQKSLSEIEANTNLLVQSINDMAESIKEQTAGITQINDS 665

IA3902_Tlp11 AGEHGRGFAVVADEVRKLAERTQKSLSEIEANTNLLVQSINDMAESIKEQTAGITQINDS 665

BCW_6290_Tlp11 AGEHGRGFAVVADEVRKLAERTQKSLSEIEANTNLLVQSINDMAESIKEQTAGITQINDS 665

76339_Tlp18 AGEHGRGFAVVADEVRKLAERTQKSLSEIEANTNLLVQSINDMAESIKEQTAGITQINES 664

4031_Tlp17 AGEHGRGFAVVADEVRKLAERTQKSLSEIEANTNLLVQSINDMAESIKEQTAGITQINES 665

15-537360_Tlp13 AGEHGRGFAVVADEVRKLAERTQKSLSEIEANTNLLVQSINDMAESIKEQTAGITQINES 664

CVM_N29710_Tlp13 AGEHGRGFAVVADEVRKLAERTQKSLSEIEANTNLLVQSINDMAESIKEQTAGITQINES 664

FB1_Tlp13 AGEHGRGFAVVADEVRKLAERTQKSLSEIEANTNLLVQSINDMAESIKEQTAGITQINES 664

CFCAN032805_Tlp13 AGEHGRGFAVVADEVRKLAERTQKSLSEIEANTNLLVQSINDMAESIKEQTAGITQINES 664

BG2108_Tlp13 AGEHGRGFAVVADEVRKLAERTQKSLSEIEANTNLLVQSINDMAESIKEQTAGITQINES 664

YF2105_Tlp13 AGEHGRGFAVVADEVRKLAERTQKSLSEIEANTNLLVQSINDMAESIKEQTAGITQINES 664

YH503_Tlp13 AGEHGRGFAVVADEVRKLAERTQKSLSEIEANTNLLVQSINDMAESIKEQTAGITQINES 664

BFRCA9557_Tlp13 AGEHGRGFAVVADEVRKLAERTQKSLSEIEANTNLLVQSINDMAESIKEQTAGITQINES 664

YH502_Tlp13 AGEHGRGFAVVADEVRKLAERTQKSLSEIEANTNLLVQSINDMAESIKEQTAGITQINES 664

MTVDSCj13_Tlp13 AGEHGRGFAVVADEVRKLAERTQKSLSEIEANTNLLVQSINDMAESIKEQTAGITQINDS 664

OR12_Tlp13 AGEHGRGFAVVADEVRKLAERTQKSLSEIEANTNLLVQSINDMAESIKEQTAGITQINDS 664

00-1597_Tlp13 AGEHGRGFAVVADEVRKLAERTQKSLSEIEANTNLLVQSINDMAESIKEQTAGITQINDS 664

14903A_Tlp13 AGEHGRGFAVVADEVRKLAERTQKSLSEIEANTNLLVQSINDMAESIKEQTAGITQINES 664

FJ3124_Tlp13 AGEHGRGFAVVADEVRKLAERTQKSLSEIEANTNLLVQSINDMAESIKEQTAGITQINDS 664

R14_Tlp13 AGEHGRGFAVVADEVRKLAERTQKSLSEIEANTNLLVQSINDMAESIKEQTAGITQINDS 664

MTVDSCj16_Tlp13 AGEHGRGFAVVADEVRKLAERTQKSLSEIEANTNLLVQSINDMAESIKEQTAGITQINDS 664

14980A_Tlp13 AGEHGRGFAVVADEVRKLAERTQKSLSEIEANTNLLVQSINDMAESIKEQTAGITQINDS 664

**********************************************************:*

15-537360_Tlp20 VAQIDQTTKDNVEIANESAIISSTVSDIANNILEDVKKKRF- 659

76339_Tlp20 VAQIDQTTKDNVEIANESAIISNTVSDIANNILEDVRKKRF- 659

CFSAN032805_Tlp20 VAQIDQTTKDNVEIANESAIISSTVSDIANNILEDVKKKRF- 658

CVM_N29710_Tlp20 VAQIDQTTKDNVEIANESAIISSTVSDIANNILEDVKKKRF- 657

YH501_Tlp20 VAQIDQTTKDNVEIANESAIISSTVSDIANNILEDVKKKRF- 661

CO2-160_Tlp20 VAQIDQTTKDNVEIANESAIISSTVSDIANNILEDVKKKRF- 659

CO2-160_Tlp20b VAQIDQTTKDNVEIANESAIISSTVSDIANNILEDVKKKRF- 659

RM5611_Tlp20 VAQIDQTTKDNVEIANESAIISSTVSDIANNILEDVKKKRF- 659

14903A_Tlp20 VAQIDQTTKDNVEIANESAIISSTVSDIANNILEDVKKKRF- 661

YH502_Tlp20 VAQIDQTTKDNVEIANESAIISSTVSDIANNILEDVKKKRF- 658

RM3196_Tlp23 VAQIDQTTKDNVEIANESAIISSTVSDIANNILEDVKKKRF- 659

ICDCCJ07001_Tlp23 VAQIDQTTKDNVEIANESAIISSTVSDIANNILEDVKKKRF- 659

RM1285_Tlp2 VAQIDQTTKDNVEIANESAIISSTVSDIANNILEDVKKKRF- 658

CFSAN032806_Tlp2 VAQIDQTTKDNVEIANESAIISNTVSDIANNILEDVKKKRF- 659

RM1221_Tlp2 VAQIDQTTKDNVEIANESAIISSTVSDIANNILEDVKKKRF- 659

S3_Tlp2 VAQIDQTTKDNVEIANESAIISSTVSDIANNILEDVKKKRF- 659

FDAARGOS_422_Tlp2 VAQIDQTTKDNVEIANESAIISSTVSDIANNILEDVKKKRF- 659

81-176_Tlp2 VAQIDQTTKDNVEIANESAIISSTVSDIANNILEDVKKKRF- 659

F38011_Tlp2 VAQIDQTTKDNVEIANESAIISSTVSDIANNILEDVKKKRF- 659

NCTC11168_Tlp2 VAQIDQTTKDNVEIANESAIISSTVSDIANNILEDVKKKRF- 659

MTVDSCj07_Tlp2 VAQIDQTTKDNVEIANESAIISSTVSDIANNILEDVKKKRF- 659

CJM1cam_Tlp24 VAQIDQTTKDNVEIANESAIISSTVSDIANNILEDVKKKRF- 656

M1_Tlp24 VAQIDQTTKDNVEIANESAIISSTVSDIANNILEDVKKKRF- 655

81116_Tlp2 VAQIDQTTKDNVEIANESAIISSTVSDIANNILEDVKKKRF- 659

4031_Tlp23 VAQIDQTTKDNVEIANESAIISSTVSDIANNILEDVKKKRF- 658

MG1116_Tlp14 VAQIDQTTKDNVEIANESAIISSTVSDIANNILEDVKKKRF- 649

YH502_Tlp14 VAQIDQTTKDNVEIANESAIISSTVSDIANNILEDVKKKRF- 667

BP3181_Tlp14 VAQIDQTTKDNVEIANESAIISSTVSDIANNILEDVKKKRF- 656

ZV1224_Tlp14a VAQIDQTTKDNVEIANESAIISSTVSDIANNILEDVKKKRF- 656

ZV1224_Tlp14b VAQIDQTTKDNVEIANESAIISSTVSDIANNILEDVKKKRF- 656

YH503_Tlp14 VAQIDQTTKDNVEIANESAIISSTVSDIANNILEDVKKKRF- 667

14903A_Tlp14 VAQIDQTTKDNVEIANESAIISSTVSDIANNILEDVKKKRF- 656

OR12_Tlp14 VAQIDQTTKDNVEIANESAIISSTVSDIANNILEDVKKKRF- 656

CFSAN032805_Tlp14 VAQIDQTTKDNVEIANESAIISSTVSDIANNILEDVKKKRF- 667

BFR-CA-9557_Tlp14 VAQIDQTTKDNVEIANESAIISSTVSDIANNILEDVKKKRF- 656

15-537360_Tlp14 VAQIDQTTKDNVEIANESAIISNTVSDIANNILEDVKKKRF- 656

YH501_Tlp14 VAQIDQTTKDNVEIANESAIISSTVSDIANNILEDVKKKRF- 656

CG8421_Tlp14 VAQIDQTTKDNVEIANESAIISSTVSDIANNILEDVKKKRF- 650

MTVDSCj16_Tlp14 VAQIDQTTKDNVEIANESAIISSTVSDIANNILEDVKKKRF- 656

01-1512_Tlp14 VAQIDQTTKDNVEIANESAIISSTVSDIANNILEDVKKKRF- 656

00-0949_Tlp14 VAQIDQTTKDNVEIANESAIISSTVSDIANNILEDVKKKRF- 656

MTVDSCj13_Tlp14 VAQIDQTTKDNVEIANESAIISSTVSDIANNILEDVKKKRF- 656

S3_Tlp14 VAQIDQTTKDNVEIANESAIISSTVSDIANNILEDVKKRGFN 657

PT14_Tlp14 VAQIDQTTKDNVEIANESAIISSTVSDIANNILEDVKKKRF- 656

14980A_Tlp14 VAQIDQTTKDNVEIANESAIISSTVSDIANNILEDVKKKRF- 672

FJ3124_Tlp14 VAQIDQTTKDNVEIANESAIISSTVSDIANNILEDVKKKRF- 656

00-1597_Tlp14 VAQIDQTTKDNVEIANESAIISSTVSDIANNILEDVKKKR-- 655

R14_Tlp14 VAQIDQTTKDNVEIANESAIISSTVSDIANNILEDVKKKRFF 657

CG8421_Tlp25 VAQIDQTTKDNVEIANESAIISSTVSDIANNILEDVKKKRF- 447

RM1875_Tlp3 VAQIDQTTKDNVEIANESAIISNTVSDIANNILEDVKKKRF- 651

CJM1cam_Tlp3 VAQIDQTTKDNVEIANESAIISSTVSDIANNILEDVKKKRF- 651

M1_Tlp3 VAQIDQTTKDNVEIANESAIISSTVSDIANNILEDVKKKRF- 651

4031_Tlp3 VAQIDQTTKDNVEIANESAIISSTVSDIANNILEDVKKKRF- 651

R14_Tlp3 VAQIDQTTKDNVEIANESAIISSTVSDIANNILEDVKKKRF- 651

RM5611_Tlp3 VAQIDQTTKDNVEIANESAIISSTVSDIANNILEDVKKKRF- 651

MTVDSCj16_Tlp3 VAQIDQTTKDNVEIANESAIISSTVSDIANNILEDVKKKRF- 651

01-1512_Tlp3 VAQIDQTTKDNVEIANESAIISSTVSDIANNILEDVKKKRF- 651

MTVDSCj13_Tlp3 VAQIDQTTKDNVEIANESAIISSTVSDIANNILEDVKKKRF- 651

32488_Tlp3a VAQIDQTTKDNVEIANESAIISSTVSDIANNILEDVKKKRF- 651

81116_Tlp3 VAQIDQTTKDNVEIANESAIISSTVSDIANNILEDVKKKRF- 651

32488_Tlp3b VAQIDQTTKDNVEIANESAIISSTVSDIANNILEDVKKKRF- 651

FB1_Tlp3 VAQIDQTTKDNVEIANESAIISSTVSDIANNILEDVKKKRF- 651

PT14_Tlp3 VAQIDQTTKDNVEIANESAIISSTVSDIANNILEDVKKKRF- 651

00-6200_Tlp3a VAQIDQTTKDNVEIANESAIISSTVSDIANNILEDIKKKRF- 651

RM1221_Tlp3 VAQIDQTTKDNVEIANESAIISSTVSDIANNILEDVKKKRF- 651

S3_Tlp3 VAQIDQTTKDNVEIANESAIISSTVSDIANNILEDVKKKRF- 651

FDAARGOS_421_Tlp3 VAQIDQTTKDNVEIANESAIISSTVSDIANNILEDVKKKRF- 662

CFSAN032806_Tlp3 VAQIDQTAKDNVEIANESAIISNTVSDIANNILEDVKKKRF- 662

IA3901_Tlp3b VAQIDQTTKDNVEIANESAIISSTVSDIANNILEDIKKKRF- 651

00-6200_Tlp3b VAQIDQTTKDNVEIANESAIISSTVSDIANNILEDIKKKRF- 651

BCW_6290_Tlp3b VAQIDQTTKDNVEIANESAIISSTVSDIANNILEDIKKKRF- 651

00-2425_Tlp3a VAQIDQTTKDNVEIANESAIISSTVSDIANNILEDVKKKRF- 651

00-2425_Tlp3b VAQIDQTTKDNVEIANESAIISSTVSDIANNILEDIKKKRF- 651

YH001_Tlp3a VAQIDQTTKDNVEIANESAIISSTVSDIANNILEDIKKKRF- 651

YH001_Tlp3b VAQIDQTTKDNVEIANESAIISSTVSDIANNILEDIKKKRF- 651

00-0949_Tlp3 VAQIDQTTKDNVEIANESAIISSTVSDIANNILEDVKKKRF- 651

NCTC11168_Tlp3 VAQIDQTTKDNVEIANESAIISSTVSDIANNILEDVKKKRF- 662

F38011_Tlp3 VAQIDQTTKDNVEIANESAIISSTVSDIANNILEDVKKKRF- 651

RM1285_Tlp3 VAQIDQTTKDNVEIANESAIISSTVSDIANNILEDVKKKRF- 651

FDAARGOS_422_Tlp3 VAQIDQTTKDNVEIANESAIISSTVSDIANNILEDVKKKRF- 662

MTVDSCj07_Tlp3 VAQIDQTTKDNVEIANESAIISSTVSDIANNILEDVKKKRF- 651

IA3901_Tlp3a VAQIDQTTKDNVEIANESAIISSTVSDIANNILEDVKKKRF- 651

BCW_6290_Tlp3a VAQIDQTTKDNVEIANESAIISSTVSDIANNILEDVKKKRF- 651

35925B2_Tlp3 VAQIDQTTKDNVEIANESAIISSTVSDIANNILEDVKKKRF- 665

14980A_Tlp3 VAQIDQTTKDNVEIANESAIISSTVSDIANNILEDVKKKRF- 662

00-1597_Tlp3b VAQIDQTTKDNVEIANESAIISSTVSDIANNILEDVKKKRF- 651

RM3196_Tlp3 VAQIDQTTKDNVEIANESAIISSTVSDIAN------------ 640

FDAARGOS_295_Tlp21 VAQIDQTTKDNVEIANESAIISSTVSDIANNILEDVKKKRF- 653

FORC_046_Tlp4 VAQIDQTTKDNVEIANESAIISSTVSDIANNILEDVKKKRF- 665

FDAARGOS_422_Tlp4 VAQIDQTTKDNVEIANESAIISSTVSDIANNILEDVKKKRF- 665

ICDCCJ07001_Tlp4 VAQIDQTTKDNVEIANESAIISSTVSDIANNILEDVKKKRF- 665

RM3196_Tlp4 VAQIDQTTKDNVEIANESAIISSTVSDIANNILEDVKKKRF- 665

T1-21_Tlp4 VAQIDQTTKDNVEIANESAIISSTVSDIANNILEDVKKKRF- 665

F38011_Tlp4 VAQIDQTTKDNVEIANESAIISSTVSDIANNILEDVKKKRF- 665

HF5-4A-4_Tlp22 VAQIDQTTKDNVEIANESAIISSTVSDIANNILEDVKKKRF- 611

00-0949_Tlp4 VAQIDQTTKDNVEIANESAIISSTVSDIANNILEDVKKKRF- 664

01-1512_Tlp4 VAQIDQTTKDNVEIANESAIISSTVSDIANNILEDVKKKRF- 665

81-176_Tlp4 VAQIDQTTKDNVEIANESAIISSTVSDIANNILEDVKKKRF- 665

32488_Tlp4 VAQIDQTTKDNVEIANESAIISSTVSDIANNILEDVKKKRF- 665

NCTC11168_Tlp4 VAQIDQTTKDNVEIANESAIISSTVSDIANNILEDVKKKRF- 665

CFSAN032806_Tlp4 VAQIDQTTKDNVEIANESAIISSTVSDIANNILEDVKKKRF- 665

81116_Tlp4 VAQIDQTTKDNVEIANESAIISSTVSDIANNILEDVKKKRF- 665

RM1285_Tlp12 VAQIDQTTKDNVEIANESAIISSTVSDIANNILEDVKKKRF- 661

PT14_Tlp12 VAQIDQTTKDNVEIANESAIISSTVSDIANNILEDVKKKRF- 662

MTVJDCj07_Tlp12 VAQIDQTTKDNVEIANESAIISSTVSDIANNILEDVKKKRF- 662

RM1221_Tlp12 VAQIDQTTKDNVEIANESAIISSTVSDIANNILEDVKKKRF- 662

FDAARGOS_421_Tlp12 VAQIDQTTKDNVEIANESAIISSTVSDIANNILEDVKKKRF- 662

35925B2_Tlp12 VAQIDQTTKDNVEIANESAIISSTVSDIANNILEDVKKKRF- 662

CJM1cam_Tlp12 VAQIDQTTKDNVEIANESAIISSTVSDIANNILEDVKKKRF- 662

M1_Tlp12 VAQIDQTTKDNVEIANESAIISSTVSDIANNILEDVKKKRF- 662

S3_Tlp12 VAQIDQTTKDNVEIANESAIISSTVSDIANNILEDVKKKRF- 662

00-1597_Tlp12 VAQIDQTTKDNVEIANESAIISSTVSDIANNILEDVKKKRF- 662

R14_Tlp12 VAQIDQTTKDNVEIANESAIISSTVSDIANNILEDVKKKRF- 662

RM1875_Tlp15 VAQIDQTTKDNVEIANESAIISSTVSDIANNILEDVKKKRF- 579

YH503_Tlp16 VAQIDQTTKDNVEIANESAIISSTVSDIANNILEDVKKKRF- 579

FB1_Tlp16 VAQIDQTTKDNVEIANESAIISSTVSDIANNILEDVKKKRF- 579

BFR-CA-9557_Tlp16 VAQIDQTTKDNVEIANESAIISSTVSDIANSILEDVKKKRF- 578

15-537360_Tlp16 VAQIDQTTKDNVEIANESAIISSTVSDIANNILEDVKKKRF- 579

OR12_Tlp16 VAQIDQTTKDNVEIANESAIISSTVSDIANNILEDVKKKRF- 579

YH502_Tlp16 VAQIDQTTKDNVEIANESAIISSTVSDIANNILEDVKKKRF- 579

14903A_Tlp16 VAQIDQTTKDNVEIANESAIISSTVSDIANNILEDVKKKRF- 579

RM5611_Tlp16 VAQIDQTTKDNVEIANESAIISSTVSDIANNILEDVKKKRF- 579

00-2425_Tlp11 VAQIDQTTKDNVEIANESAIISSTVSDIANNILEDVKKKRF- 706

00-6200_Tlp11 VAQIDQTTKDNVEIANESAIISSTVSDIANNILEDVKKKRF- 706

YH001_Tlp11 VAQIDQTTKDNVEIANESAIISSTVSDIANNILEDVKKKRF- 706

IA3902_Tlp11 VAQIDQTTKDNVEIANESAIISSTVSDIANNILEDVKKKRF- 706

BCW_6290_Tlp11 VAQIDQTTKDNVEIANESAIISSTVSDIANNILEDVKKKRF- 706

76339_Tlp18 VAQIDQTTKDNVEIANESAIISNTVSDIANNILEDVRKKRF- 705

4031_Tlp17 VAQIDQTTKDNVEIANESAIISSTVSDIANNILEDVKKKRF- 706

15-537360_Tlp13 VAQIDQTTKDNVEIANESAIISSTVSDIANNILEDVKKKRF- 705

CVM_N29710_Tlp13 VAQIDQTTKDNVEIANESAIISSTVSDIANNILEDVKKKRF- 705

FB1_Tlp13 VAQIDQTTKDNVEIANESAIISSTVSDIANNILEDVKKKRF- 705

CFCAN032805_Tlp13 VAQIDQTTKDNVEIANESAIISSTVSDIANNILEDVKKKRF- 705

BG2108_Tlp13 VAQIDQTTKDNVEIANESAIISSTVSDIANNILEDVKKKRF- 705

YF2105_Tlp13 VAQIDQTTKDNVEIANESAIISSTVSDIANNILEDVKKKRF- 705

YH503_Tlp13 VAQIDQTTKDNVEIANESAIISSTVSDIANNILEDVKKKRF- 705

BFRCA9557_Tlp13 VAQIDQTTKDNVEIANESAIISSTVSDIANSILEDVKKKRF- 705

YH502_Tlp13 VAQIDQTTKDNVEIANESAIISSTVSDIANNILEDVKKKRF- 705

MTVDSCj13_Tlp13 VAQIDQTTKDNVEIANESAIISSTVSDIANNILEDVKKKRF- 705

OR12_Tlp13 VAQIDQTTKDNVEIANESAIISSTVSDIANNILEDVKKKRF- 705

00-1597_Tlp13 VAQIDQTTKDNVEIANESAIISSTVSDIANNILEDVKKKRF- 705

14903A_Tlp13 VAQIDQTTKDNVEIANESAIISSTVSDIANNILEDVKKKRF- 705

FJ3124_Tlp13 VAQIDQTTKDNVEIANESAIISSTVSDIANNILEDVKKKRF- 705

R14_Tlp13 VAQIDQTTKDNVEIANESAIISSTVSDIANNILEDVKKKRF- 705

MTVDSCj16_Tlp13 VAQIDQTTKDNVEIANESAIISSTVSDIANNILEDVKKKRF- 705

14980A_Tlp13 VAQIDQTTKDNVEIANESAIISSTVSDIANNILEDVKKKRF- 705

*******:**************.****************
