# Supplementary material for: Diversity of transducer-like proteins (Tlps) in Campylobacter
Source: PLoS One. 2019 Mar 25;14(3):e0214228. doi: 10.1371/journal.pone.0214228 (PMC6433261; doi:10.1371/journal.pone.0214228)
Supplement: S2 Archive — (ZIP) [file pone.0214228.s016.zip › Alignment G.docx]

Alignment G. Comparison of Tlp2 and Tlp23 major protein variants

CLUSTAL O(1.2.4) multiple sequence alignment 2018/04/13

NCTC11168 MKSVKLKVSLIANLIAVVCLIILGVVTFIFVKQAIFHEVVNAEINYVKTAKNSIESFKAR 60

RM3196 MKSVKLKVTLIANLITVVCLVILGVITFMFVKQAIFHEVVNAEINYVKTAKNSIESFKAR 60

********:******:****:****:**:*******************************

NCTC11168 NSLALESLAKSILKHPIEQLDSQDALMHYVGKDLKNFRDAGRFLAVYIAQPNGELVVSDP 120

RM3196 NSLALESLAKSILKHPVEQLDNQDALMHYVGKDLKNFRDAGRFLAVYIAQPNGELVVSDP 120

****************:****.**************************************

NCTC11168 DSDAKNLDFGTYGKADNYDARTREYYIEAVKTNKLYITPSYIDVTTNLPCFTYSIPLYKD 180

RM3196 DSDAKNLDFGTYGKADNYDARTREYYIEAVKTNKLYITPSYIDVTTNLPCFTYSIPLYKD 180

************************************************************

NCTC11168 GKFIGVLAVDILAADLQAEFENLPGRTFVFDEENKVFVSTDKALLQKGYDISAIANLAKT 240

RM3196 GKFIGVLAVDVLAADLQAEFENLPGRIFVFDEENKVFVSTDKTLLQQGYDISTIANLAKT 240

**********:*************** ***************:***:*****:*******

NCTC11168 KEDLEPFEYTRPKDGNERFAVCTKVSGIYTACVGEPIEQIEAPVYKIAFIQTAIVIFTSI 300

RM3196 KKDFEPFEYTRPKDGSERFAVCVKVSGIYTACVAKPIEQIEAPVYKAAFIQAIVVIIVVV 300

*:*:***********.******.**********.:*********** ****: :**:. :

NCTC11168 ISVILLYFIVSKYLSPLAAIQTGLTSFFDFINYKTKNVSTIEVKSNDEFGQISNAINENI 360

RM3196 FSVILLYFIVSKYLSPLAAIQTGLTSFFDFINHKTKNVSTIEVKSNDEFGQISNAINENI 360

:*******************************:***************************

NCTC11168 LATKRGLEQDNQAVKESVQTVSVVEGGNLTARITANPRNPQLIELKNVLNKLLDVLQARV 420

RM3196 LATKRGLEQDNQAVKESVETVHVVEGGNLTARITANPRNPQLIELKNVLNRLLDALQARV 420

******************:** ****************************:***.*****

NCTC11168 GSDMNAIHKIFEEYKSLDFRNKLENASGSVELTTNALGDEIVKMLKQSSDFANALANESG 480

RM3196 GSDMNEIQRVFNSYKSLDFTTEVKDANGAVEVTTNALGQEIIKMLKQSSDFANALANESG 480

***** *:::*:.****** .::::*.*:**:******:**:******************

NCTC11168 KLQTAVQSLTTSSNSQAQSLEETAAALEEITSSMQNVSVKTSDVITQSEEIKNVTGIIGD 540

RM3196 KLQTAVQSLTTSSNSQAQSLEETAAALEEITSSMQNVSVKTSDVITQSEEIKNVTGIIGD 540

************************************************************

NCTC11168 IADQINLLALNAAIEAARAGEHGRGFAVVADEVRKLAERTQKSLSEIEANTNLLVQSIND 600

RM3196 IADQINLLALNAAIEAARAGEHGRGFAVVADEVRKLAERTQKSLSEIEANTNLLVQSIND 600

************************************************************

NCTC11168 MAESIKEQTAGITQINDSVAQIDQTTKDNVEIANESAIISSTVSDIANNILEDVKKKRF 659

RM3196 MAESIKEQTAGITQINDSVAQIDQTTKDNVEIANESAIISSTVSDIANNILEDVKKKRF 659

***********************************************************
