# Supplementary material for: Diversity of transducer-like proteins (Tlps) in Campylobacter
Source: PLoS One. 2019 Mar 25;14(3):e0214228. doi: 10.1371/journal.pone.0214228 (PMC6433261; doi:10.1371/journal.pone.0214228)
Supplement: S2 Archive — (ZIP) [file pone.0214228.s016.zip › Alignment H.docx]

Alignment H. Comparison of Tlp3 and Tlp19 protein variants

CLUSTAL O(1.2.4) multiple sequence alignment 2018/04/13

NCTC11168 is Tlp3; RM3196 is Tlp19

NCTC11168 MLKITKIKRKIMNSIKIKLSLIANLIAIFALIVLGIVSFYFTKTSLYESTLKNQTDLLKV 60

RM3196 -----------MNSIKIKLSLIANLIAIFALIVLGIVSFYFTKTSLYESTLKNQTDLLKV 49

*************************************************

NCTC11168 TQSTVEDFRSTNQSFTRALEKDIANLPYQSLITEENIINNVGPILKYYRHSINALNVYLG 120

RM3196 TQSTVEDFRSTNQSFTRALEKDIANLPYQSLITEENIINNVGPILKYYRHSINALNVYLG 109

************************************************************

NCTC11168 LNNGKVLLSQKSNDAKMPELRDDLDIKTKDWYQEALKTNDIFVTPAYLDTVLKQYVITYS 180

RM3196 LNNGKVLLSQKSNDAKMPELRDDLDIKTKDWYQEALKTNDIFVTPAYLDTVLKQYVITYS 169

************************************************************

NCTC11168 KAIYKDGKIIGVLGVDIPSEDLQNLVAKTPGNTFLFDQKNKIFAATNKELLNPSIDHSPV 240

RM3196 KAIYKDGKIIGVLGVDIPSEDLQNLVAKTPGNTFLFDQKNKIFAATNKELLNPSIDHSPV 229

************************************************************

NCTC11168 LNAYKLNGDNNFFSYKLNNEERLGACTKVFAYTACITESADIINKPIYKAAFIQAIVVII 300

RM3196 SNAYKAHGDNNFFSYKLNNEERLGACTKVFAYTACITESADIINKPIYKAAFIQAIVVII 289

**** :*****************************************************

NCTC11168 VVVFSVILLYFIVSKYLSPLAAIQTGLTSFFDFINYKTKNVSTIEVKSNDEFGQISNAIN 360

RM3196 VVVFSVILLYFIVSKYLSPLAAIQTGLTSFFDFINHKTKNVSTIEVKSNDEFGQISNAIN 349

***********************************:************************

NCTC11168 ENILATKRGLEQDNQAVKESVQTVSVVEGGNLTARITANPRNPQLIELKNVLNKLLDVLQ 420

RM3196 ENILATKRGLEQDNQAVKESVETVHVVEGGNLTARITANPRNPQLIELKNVLNRLLDALQ 409

*********************:** ****************************:***.**

NCTC11168 ARVGSDMNAIHKIFEEYKSLDFRNKLENASGSVELTTNALGDEIVKMLKQSSDFANALAN 480

RM3196 ARVGSDMNEIQRVFNSYKSLDFTTEVKDANGAVEVTTNALGQEIIKMLKQSSDFANALAN 469

******** *:::*:.****** .::::*.*:**:******:**:***************

NCTC11168 ESGKLQTAVQSLTTSSNSQAQSLEETAAALEEITSSMQNVSVKTSDVITQSEEIKNVTGI 540

RM3196 ESGKLQTAVQSLTTSSNSQAQSLEETAAALEEITSSMQNVSVKTSDVITQSEEIKNVTGI 529

************************************************************

NCTC11168 IGDIADQINLLALNAAIEAARAGEHGRGFAVVADEVRKLAERTQKSLSEIEANTNLLVQS 600

RM3196 IGDIADQINLLALNAAIEAARAGEHGRGFAVVADEVRKLAERTQKSLSEIEANTNLLVQS 589

************************************************************

NCTC11168 INDMAESIKEQTAGITQINDSVAQIDQTTKDNVEIANESAIISSTVSDIANNILEDVKKK 660

RM3196 INDMAESIKEQTAGITQINDSVAQIDQTTKDNVEIANESAIISSTVSDIANNILEDVKKK 649

************************************************************

NCTC11168 RF 662

RM3196 RF 651

**
