# Supplementary material for: Diversity of transducer-like proteins (Tlps) in Campylobacter
Source: PLoS One. 2019 Mar 25;14(3):e0214228. doi: 10.1371/journal.pone.0214228 (PMC6433261; doi:10.1371/journal.pone.0214228)
Supplement: S2 Archive — (ZIP) [file pone.0214228.s016.zip › Alignment I.docx]

Alignment I. Comparison of closely related Tlp4 and Tlp12

CLUSTAL O(1.2.4) multiple sequence alignment

FORC_046 MQSINSGKSVGISAKLTLWVGILVVLILAITSAISYFDSRNNTYELLKDTQLKTMQDVDA 60 Tlp4

FDAARGOS_422 MQSINSGKSVGISAKLTLWVGILVVLILAITSAISYFDSRNNTYELLKDTQLKTMQDVDA 60 Tlp4

ICDCCJ07001 MQSINSGKSVGISAKLTLWVGILVVLILAITSAISYFDSRNNTYELLKDTQLKTMQDVGA 60 Tlp4

RM3196 MQSINSGKSVGISAKLTLWVGILVVLILAITSAISYFDSRNNTYELLKDTQLKTMQDVGA 60 Tlp4

T1-21 MQSINSGKSVGISAKLTLWVGILVVLILAITSAISYFDSRNNTYELLKDTQLKTMQDVGA 60 Tlp4

F38011 MQSINSGKSAGISAKLTLWVGILVVLILAITSAVSYFDSRNNTYELLKDTQLKTMQDVGA 60 Tlp4

00-0949 MQSINSGKSVGISAKLTLWVGILVVLILAITSAISYFDSRNNTYELLKDTQLKTMQDVDA 60 Tlp4

01-1512 MQSINSGKSVGISAKLTLWVGILVVLILAITSAISYFDSRNNTYELLKDTQLKTMQDVDA 60 Tlp4

81-176 MQSINSGKSVGISAKLTLWVGILVVLILAITSAISYFDSRNNTYELLKDTQLKTMQDVDA 60 Tlp4

32488 MQSINSGKSVGISAKLTLWVGILVVLILAITSAISYFDSRNNTYELLKDTQLKTMQDVDA 60 Tlp4

NCTC11168 MQSINSGKSVGISAKLTLWVGILVVLILAITSAISYFDSRNNTYELLKDTQLKTMQDVDA 60 Tlp4

CFSAN032806 MQSINSGKSVGISAKLTLWVGILVVLILAITSAISYFDSRNNTYELLKDTQLKTMQDVDA 60 Tlp4

81116 MQSINSGKSVGISAKLTLWVGILVVLILAITSAISYFDSRNNTYELLKDTQLKTMQDVDA 60 Tlp4

RM1285 MQSINSGKSVGISAKLTLWVGILVVLILAITSTVSYFDAKNHTYELLKENQLKTMDDVKV 60 Tlp12

PT14 MQSINSGKSVGISAKLTLWVGILVVLILAITSTVSYFDAKNHTYELLKENQLKTMDDVKV 60 Tlp12

MTVJDCj07 MQSINSGKSVGISAKLTLWVGILVVLILAITSTVSYFDAKNHTYELLKENQLKTMDDVKV 60 Tlp12

RM1221 MQKMDSGKSVGVSVKLTLWVGILVVLILAITSTVSYFDAKNHTYELLKENQLKTMDDVKV 60 Tlp12

FDAARGOS_421 MQKMDSGKSVGVSVKLTLWVGILVVLILAITSTVSYFDAKNHTYELLKENQLKTMDDVKV 60 Tlp12

35925B2 MQKMNSGKSVGISAKLTLWVGILVVLILAITSAVSYFDAKNHTYELLKENQLKTMNDVKV 60 Tlp12

CJM1cam MQKMNSGKSVGISAKLTLWVGILVVLILAITSAVSYFDAKNHTYELLKENQLKTMDDVKV 60 Tlp12

M1 MQKMNSGKSVGISAKLTLWVGILVVLILAITSAVSYFDAKNHTYELLKENQLKTMDDVKV 60 Tlp12

S3 MQKMDSGKSVGVSVKLTLWVGILVVLILAITSTVSYFDAKNHTYELLKENQLKTMDDVKV 60 Tlp12

00-1597 MQSINSGKSVGVSVKLTLWVGILVVLILAITSTVSYFDAKNHTYELLKENQLKTMDDVKV 60 Tlp12

R14 MQSINSGKSVGISAKLTLWVGILVVLILAITSTVSYFDAKNHTYELLKENQLKTMDDVKV 60 Tlp12

**.::****.*:*.******************::****::*:******:.*****:** .

FORC_046 FFKSYAMSKRNGIQILANELTNRPDMSDEELINLIKVIKKVNDYDLVYVGFDNTGKNYQS 120 Tlp4

FDAARGOS_422 FFKSYAMSKRNGIQILANELTNRPDMSDEELINLIKVIKKVNDYDLVYVGFDNTGKNYQS 120 Tlp4

ICDCCJ07001 FFESYGMSKRNGIQILANELNKRPDMSDEELINLIKAFKEVNGYDLVYVGFDNTGKNYQS 120 Tlp4

RM3196 FFESYGMSKRNGIQILANELNKRPDMSDEELINLIKAFKEVNGYDLVYVGFDNTGKNYQS 120 Tlp4

T1-21 FFESYGMSKRNGIQILANELNKRPDMSDEELINLIKAFKEVNGYDLVYVGFDNTGKNYQS 120 Tlp4

F38011 FFESYGMSKRHGIQILANELNKRPDMSDEELINLIKAFKEVNDYDLVYVGFDNTGKNYQS 120 Tlp4

00-0949 FFKSYAMSKRNGIQILANELTNRPDMSDEELINLIKVIKKVNDYDLVYVGFDNTGKNYQS 120 Tlp4

01-1512 FFKSYAMSKRNGIQILANELTNRPDMSDEELINLIKVIKKVNDYDLVYVGFDNTGKNYQS 120 Tlp4

81-176 FFKSYAMSKRNGIQILANELTNRPDMSDEELINLIKVIKKVNDYDLVYVGFDNTGKNYQS 120 Tlp4

32488 FFKSYAMSKRNGIQILANELTNRPDMSDEELINLIKVIKKVNDYDLVYVGFDNTGKNYQS 120 Tlp4

NCTC11168 FFKSYAMSKRNGIQILANELTNRPDMSDEELINLIKVIKKVNDYDLVYVGFDNTGKNYQS 120 Tlp4

CFSAN032806 FFKSYAMSKRNGIQILANELTNRPDMSDEELINLIKVIKKVNDYDLVYVGFDNTGKNYQS 120 Tlp4

81116 FFKSYAMSKRNGIQILANELTNRPDMSDEELINLIKVIKKVNDYDLVYVGFDNTGKNYQS 120 Tlp4

RM1285 TFENYSKSKQKAIEVLAYESAK--KLEDENISLLLDSFKKAFDFDIVFIAFDKNNKMLLS 118 Tlp12

PT14 TFENYSKSKQKAIEVLAYESAK--KLEDENISLLLDSFKKAFDFDIVFIAFDKNNKMLLS 118 Tlp12

MTVJDCj07 TFENYSKSKQKAIEVLAYESAK--KLEDENISLLLDSFKKAFDFDIVFIAFDKNNKMLLS 118 Tlp12

RM1221 TFENYSKSKQKAIEVLAYESAK--KLEDENISLLLDSFKKAFDFDIVFIAFDKNNKMLLS 118 Tlp12

FDAARGOS_421 TFENYSKSKQKAIEVLAYESAK--KLEDENISLLLDSFKKAFDFDIVFIAFDKNNKMLLS 118 Tlp12

35925B2 TFENYSKSKQKAIEVLAYESAK--KLEDENISLLLDSFKKAFDFDIVFIAFEKNNKMLLS 118 Tlp12

CJM1cam TFENYSKSKQKAIEVLAYESAK--KLEDENISLLLDSFKKAFDFDIVFIAFDKNNKMLLS 118 Tlp12

M1 TFENYSKSKQKAIEVLAYESAK--KLEDENISLLLDSFKKAFDFDIVFIAFDKNNKMLLS 118 Tlp12

S3 TFENYSKSKQKAIEVLAYESAK--KLEDENISLLLDSFKKAFDFDIVFIAFDKNNKMLLS 118 Tlp12

00-1597 TFENYSKSKQKAIEVLAYESAK--KLEDENISLLLDSFKKAFDFDIVFIAFDKNNKMLLS 118 Tlp12

R14 TFENYSKSKQKAIEVLAYESAK--KLEDENISLLLDSFKKAFDFDIVFIAFDKNNKMLLS 118 Tlp12

*:.*. **::.*::** * : .:.**:: *:. :*:. .:*:*::.*::..* *

FORC_046 DDQILDLSKGYDTKNRPWYKAAKEAKKLIVTEPYKSAASGEVGLTYAAPFYDRNGNFRGV 180 Tlp4

FDAARGOS_422 DDQILDLSKGYDTKNRPWYKAAKEAKKLIVTEPYKSAASGEVGLTYAAPFYDRNGNFRGV 180

ICDCCJ07001 DDQILDLSKGYDTKNRPWYKAAKEAKKLIVTEPYKSANSGEVGLTYAAPFYDRNGNFRGV 180

RM3196 DDQILDLSKGYDTKNRPWYKAAKEAKKLIVTEPYKSANSGEVGLTYAAPFYDRNGNFRGV 180

T1-21 DDQILDLSKGYDTKNRPWYKAAKEAKKLIVTEPYKSANSGEVGLTYAAPFYDRNGNFRGV 180

F38011 DDQILDLSKGYDTKNRPWYKAAKEAKKLIVTEPYKSAASGEVGLTYAAPFYDRNGNFRGV 180

00-0949 DDQILDLSKGYDTKNRPWYKAAKEAKKLIVTEPYKSAASGEVGLTYAAPFYDRNGNFRGV 180

01-1512 DDQILDLSKGYDTKNRPWYKAAKEAKKLIVTEPYKSAASGEVGLTYAAPFYDRNGNFRGV 180

81-176 DDQILDLSKGYDTKNRPWYKAAKEAKKLIVTEPYKSAASGEVGLTYAAPFYDRNGNFRGV 180

32488 DDQILDLSKGYDTKNRPWYKAAKEAKKLIVTEPYKSAASGEVGLTYAAPFYDRNGNFRGV 180

NCTC11168 DDQILDLSKGYDTKNRPWYKAAKEAKKLIVTEPYKSAASGEVGLTYAAPFYDRNGNFRGV 180

CFSAN032806 DDQILDLSKGYDTKNRPWYKAAKEAKKLIVTEPYKSAASGEVGLTYAAPFYDRNGNFRGV 180

81116 DDQILDLSKGYDTKNRPWYKAAKEAKKLIVTEPYKSAASGEVGLTYAAPFYDRNGNFRGV 180

RM1285 NGTILDKKSNFDITKQIWYQEAKNNKGITITQPYKSPIDQEIGITYVFPIYKNN-QLIAF 177 Tlp12

PT14 NGTILDKKSNFDITKQIWYQEAKNNKGITITQPYKSPIDQEIGITYVFPIYKNN-QLIAF 177

MTVJDCj07 NGTILDKKSNFDITKQIWYQEAKNNKGITITQPYKSPIDQEIGITYVFPIYKNN-QLIAF 177

RM1221 NGTILDKKSNFDITKQIWYQEAKNNKGITITQPYKSPIDQEIGITYVFPIYKNN-QLIAF 177

FDAARGOS_421 NGTILDKKSNFDITKQIWYQEAKNNKGITITQPYKSPIDQEIGITYVFPIYKNN-QLIAF 177

35925B2 NGTILDKKSNFDITKQIWYQEAKNNKGITITQPYKSPIDQEIGITYVFPIYKNN-QLIAF 177

CJM1cam NGTILDKKSNFDITKQIWYQEAKNNKGITITQPYKSPIDQEIGITYVFPIYKNN-QLIAF 177

M1 NGTILDKKSNFDITKQIWYQEAKNNKGITITQPYKSPIDQEIGITYVFPIYKNN-QLIAF 177

S3 NGTILDKKSNFDITKQIWYQEAKNNKGITITQPYKSPIDQEIGITYVFPIYKNN-QLIAF 177

00-1597 NGTILDKKSNFDITKQIWYQEAKNNKGITITQPYKSPIDQEIGITYVFPIYKNN-QLIAF 177

R14 NGTILDKKSNFDITKQIWYQEAKNNKGITITQPYKSPIDQEIGITYVFPIYKNN-QLIAF 177

:. *** ...:* .:: **: **: * : :*:**** . *:*:**. *:*..* :: ..

FORC_046 VGGDYDLANFSTNVLTVGKSDNTFTEVLDSEGTILFNDEVAKILTKTELSINIANAIKAN 240 Tlp4

FDAARGOS_422 VGGDYDLANFSTNVLTVGKSDNTFTEVLDSEGTILFNDEVAKILTKTELSINIANAIKAN 240

ICDCCJ07001 VGGDYDLAKFSTDVLAVGKSQNTYTVVLDPEGTILFRDDITKILTKTELSINIANAIKAN 240

RM3196 VGGDYDLAKFSTDVLAVGKSQNTYTVVLDPEGTILFRDDITKILTKTELSINIANAIKAN 240

T1-21 VGGDYDLAKFSTDVLAVGKSQNTYTVVLDPEGTILFRDDITKILTKTELSINIANAIKAN 240

F38011 VGGDYDLANFSTNVLTVGKSDNTFTEVLDSEGTILFNDEVAKILTKTELSINIANAIKAN 240

00-0949 VGGDYDLANFSTNVLTVGKSDNTFTEVLDSEGTILFNDEVAKILTKTELSINIANAIKAN 240

01-1512 VGGDYDLANFSTNVLTVGKSDNTFTEVLDSEGTILFNDEVAKILTKTELSINIANAIKAN 240

81-176 VGGDYDLANFSTNVLTVGKSDNTFTEVLDSEGTILFNDEVAKILTKTELSINIANAIKAN 240

32488 VGGDYDLANFSTNVLTVGKSDNTFTEVLDSEGTILFNDEVAKILTKTELSINIANAIKAN 240

NCTC11168 VGGDYDLANFSTNVLTVGKSDNTFTEVLDSEGTILFNDEVAKILTKTELSINIANAIKAN 240

CFSAN032806 VGGDYDLANFSTNVLTVGKSDNTFTEVLDSEGTILFNDEVAKILTKTELSINIANAIKAN 240

81116 VGGDYDLANFSTNVLTVGKSDNTFTEVLDSEGTILFNDEVAKILTKTELSINIANAIKAN 240

RM1285 VGGDYNLDKFSKDVLSLGHSSTTYAAVYDSEGRIIFHEVLDRILTKNTLSVNIANAIKEN 237 Tlp12

PT14 VGGDYNLDKFSKDVLSLGHSSTTYAAVYDSEGRIIFHEVLDRILTKNTLSVNIANAIKEN 237

MTVJDCj07 VGGDYNLDKFSKDVLSLGHSSTTYAAVYDSEGRIIFHEVLDRILTKNTLSVNIANAIKEN 237

RM1221 VGGDYNLDKFSKDVLSLGHSSTTYAAVYDSEGRIIFHEVLDRILTKNTLSVNIANAIKEN 237

FDAARGOS_421 VGGDYNLDKFSKDVLSLGHSSTTYAAVYDSEGRIIFHEVLDRILTKNTLSVNIANAIKEN 237

35925B2 VGGDYNLDKFSKDVLSLGHSSTTYAAVYDSEGRIIFHEVLDRILTKNTLSINIANAIKEN 237

CJM1cam VGGDYNLDKFSKDVLSLGHSSTTYAAVYDSEGRIIFHEVLDRILTKNTLSVNIANTIKEN 237

M1 VGGDYNLDKFSKDVLSLGHSSTTYAAVYDSEGRIIFHEVLDRILTKNTLSVNIANTIKEN 237

S3 VGGDYNLDKFSKDVLSLGHSSTTYAAVYDSEGRIIFHEVLDRILTKNTLSVNIANAIKEN 237

00-1597 VGGDYNLDKFSKDVLSLGHSSTTYAAVYDSEGRIIFHEVLDRILTKNTLSVNIANAIKEN 237

R14 VGGDYNLDKFSKDVLSLGHSSTTYAAVYDSEGRIIFHEVLDRILTKNTLSVNIANAIKEN 237

*****:* :**.:**::*:*..*:: * * ** *:*.: : :****. **:****:** *

FORC_046 PALIDPRNQDTLFTAKDHQGVDYAIMCNSAFNPLFRICTITENKVYTEAVNSILMKQVIV 300 Tlp4

FDAARGOS_422 PALIDPRNQDTLFTAKDHQGVDYAIMCNSAFNPLFRICTITENKVYTEAVNSILMKQVIV 300

ICDCCJ07001 PALIDPRNQDTLFTAKDHQGVDYAIMCNSAFNPLFRICTITENKVYTEAVNSILMKQVIV 300

RM3196 PALIDPRNQDTLFTAKDHQGVDYAIMCNSAFNPLFRICTITENKVYTEAVNSILMKQVIV 300

T1-21 PALIDPRNQDTLFTAKDHQGVDYAIMCNSAFNPLFRICTITENKVYTEAVNSILMKQVIV 300

F38011 PALIDPRNQDTLFTAKDHQGVDYAIMCNSAFNPLFRICTITENKVYTEAVNSILMKQVIV 300

00-0949 PALIDPRNQDTLFTAKDHQGVDYAIMCNSAFNPLFRICTITENKVYTEAVNSILMKQVIV 300

01-1512 PALIDPRNQDTLFTAKDHQGVDYAIMCNSAFNPLFRICTITENKVYTEAVNSILMKQVIV 300

81-176 PALIDPRNQDTLFTAKDHQGVDYAIMCNSAFNPLFRICTITENKVYTEAVNSILMKQVIV 300

32488 PALIDPRNQDTLFTAKDHQGVDYAIMCNSAFNPLFRICTITENKVYTEAVNSILMKQVIV 300

NCTC11168 PALIDPRNQDTLFTAKDHQGVDYAIMCNSAFNPLFRICTITENKVYTEAVNSILMKQVIV 300

CFSAN032806 PALIDPRNQDTLFTAKDHQGVDYAIMCNSAFNPLFRICTITENKVYTEAVNSILMKQVIV 300

81116 PALIDPRNQDTLFTAKDHQGVDYAIMCNSAFNPLFRICTITENKVYTEAVNSILMKQVIV 300

RM1285 PEYIDLNKRDILFPVFDDKGIKYEAMCDTSSNGLYRICAVTLDSNYTSAVNSILMKQVIV 297 Tlp12

PT14 PEYIDLNKRDILFPVFDDKGIKYEAMCDTSSNGLYRICAVTLDSNYTSAVNSILMKQVIV 297

MTVJDCj07 PEYIDLNKRDILFPVFDDKGIKYEAMCDTSSNGLYRICAVTLDSNYTSAVNSILMKQVIV 297

RM1221 PEYIDPNKRDILFPVFDDKGIKYETMCDTSSNGLYRICAVTLDSNYTSAVNSILMKQVIV 297

FDAARGOS_421 PEYIDPNKRDILFPVFDDKGIKYETMCDTSSNGLYRICAVTLDSNYTSAVNSILMKQVIV 297

35925B2 PKYIDLNKRDILFPVFDDKGIKYETMCDTSSNGLYRICAVTLDSNYTSAVNSILMKQVIV 297

CJM1cam PEYIDLNKRDILFPVFDDKGIKYEAMCDTSSNGLYRICAVTLDSNYTSAVNSILMKQVIV 297

M1 PEYIDLNKRDILFPVFDDKGIKYEAMCDTSSNGLYRICAVTLDSNYTSAVNSILMKQVIV 297

S3 PEYIDPNKRDILFPVFDDKGIKYETMCDTSSNGLYRICAVTLDSNYTSAVNSILMKQAIV 297

00-1597 PEYIDPNKRDILFPVFDDKGIKYETMCDTSSNGLYRICAVTLDSNYTSAVNSILMKQAIV 297

R14 PEYIDPNKRDILFPVFDDKGIKYETMCDTSSNGLYRICAVTLDSNYTSAVNSILMKQVIV 297

* ** .::* ** . *.:*:.* **::: * *:***::* :. **.*********.**

FORC_046 GIIAIIIALILIRFLISRSLSPLAAIQTGLTSFFDFINYKTKNVSTIEVKSNDEFGQISN 360 Tlp4

FDAARGOS_422 GIIAIIIALILIRFLISRSLSPLAAIQTGLTSFFDFINYKTKNVSTIEVKSNDEFGQISN 360

ICDCCJ07001 GIIAIIIALILIRFLISRSLSPLAAIQTGLTSFFDFINHKTKNVSTIEVKSNDEFGQISN 360

RM3196 GIIAIIIALILIRFLISRSLSPLAAIQTGLTSFFDFINHKTKNVSTIEVKSNDEFGQISN 360

T1-21 GIIAIIIALILIRFLISRSLSPLAAIQTGLTSFFDFINYKTKNVSTIEVKSNDEFGQISN 360

F38011 GIIAIIIALILIRFLISRSLSPLAAIQTGLTSFFDFINYKTKNVSTIEVKSNDEFGQISN 360

00-0949 GIIAIIIALILI-ILISRSLSPLAAIQTGLTSFFDFINYKTKNVSTIEVKSNDEFGQISN 359

01-1512 GIIAIIIALILIRFLISRSLSPLAAIQTGLTSFFDFINYKTKNVSTIEVKSNDEFGQISN 360

81-176 GIIAIIIALILIRFLISRSLSPLAAIQTGLTSFFDFINYKTKNVSTIEVKSNDEFGQISN 360

32488 GIIAIIIALILIRFLISRSLSPLAAIQTGLTSFFDFINYKTKNVSTIEVKSNDEFGQISN 360

NCTC11168 GIIAIIIALILIRFLISRSLSPLAAIQTGLTSFFDFINYKTKNVSTIEVKSNDEFGQISN 360

CFSAN032806 GIIAIIIALILIRFLISRSLSPLAAIQTGLTSFFDFINYKTKNVSTIEVKSNDEFGQISN 360

81116 GIIAIIIALILIRFLISRSLSPLAAIQTGLTSFFDFINYKTKNVSTIEVKSNDEFGQISN 360

RM1285 GIIAIIIALILIRFLISRSLSPLAAIQTGLTSFFDFINYKTKNVSTIEVKSNDEFGQISN 357 Tlp12

PT14 GIIAIIIALILIRFLISRSLSPLAAIQTGLTSFFDFINYKTKNVSTIEVKSNDEFGQISN 357

MTVJDCj07 GIIAIIIALILIRFLISRSLSPLAAIQTGLTSFFDFINYKTKNVSTIEVKSNDEFGQISN 357

RM1221 GIIAIIIALILIRFLISRSLSPLAAIQTGLTSFFDFINYKTKNVSTIEVKSNDEFGQISN 357

FDAARGOS_421 GIIAIIIALILIRFLISRSLSPLAAIQTGLTSFFDFINYKTKNVSTIEVKSNDEFGQISN 357

35925B2 GIIAIIIALILIRFLISRSLSPLAAIQTGLTSFFDFINHKTKNVSTIEVKSNDEFGQISS 357

CJM1cam GIIAIIIALILIRFLISRSLSPLAAIQTGLTSFFDFINYKTKNVSTIEVKSNDEFGQISN 357

M1 GIIAIIIALILIRFLISRSLSPLAAIQTGLTSFFDFINYKTKNVSTIEVKSNDEFGQISN 357

S3 GIIAIIIALILIRFLISRSLSPLAAIQTGLTSFFDFINYKTKNVSTIEVKSNDEFGQISN 357

00-1597 GIIAIIIALILIRFLISRSLSPLAAIQTGLTSFFDFINYKTKNVSTIEVKSNDEFGQISN 357

R14 GIIAIIIALILIRFLISRSLSPLAAIQTGLTSFFDFINYKTKNVSTIEVKSNDEFGQISN 357

************ :************************:********************.

FORC_046 AINENILATKRGLEQDNQAVKESVQTVSVVEGGNLTARITANPRNPQLIELKNVLNRLLD 420 Tlp4

FDAARGOS_422 AINENILATKRGLEQDNQAVKESVQTVSVVEGGNLTARITANPRNPQLIELKNVLNRLLD 420

ICDCCJ07001 AINENILATKQGLEQDAKAVKESVETVGVVESGNLTARITANPRNPQLIELKNVLNRLLD 420

RM3196 AINENILATKQGLEQDAKAVKESVETVGVVESGNLTARITANPRNPQLIELKNVLNRLLD 420

T1-21 AINENILATKRGLEQDNQAVKESVQTVSVVEGGNLTARITANPRNPQLIELKNVLNKLLD 420

F38011 AINENILATKRGLEQDNQAVKESVQTVSVVEGGNLTARITANPRNPQLIELKNVLNKLLD 420

00-0949 AINENILATKRGLEQDNQAVKESVQTVSVVEGGNLTARITANPRNPQLIELKNVLNKLLD 419

01-1512 AINENILATKRGLEQDNQAVKESVQTVSVVEGGNLTARITANPRNPQLIELKNVLNKLLD 420

81-176 AINENILATKRGLEQDNQAVKESVQTVSVVEGGNLTARITANPRNPQLIELKNVLNKLLD 420

32488 AINENILATKRGLEQDNQAVKESVQTVSVVEGGNLTARITANPRNPQLIELKNVLNKLLD 420

NCTC11168 AINENILATKRGLEQDNQAVKESVQTVSVVEGGNLTARITANPRNPQLIELKNVLNKLLD 420

CFSAN032806 AINENILATKRGLEQDNQAVKESVQTVSVVEGGNLTARITANPRNPQLIELKNVLNKLLD 420

81116 AINENILATKRGLEQDNQAVKESVQTVSVVEGGNLTARITANPRNPQLIELKNVLNKLLD 420

RM1285 AINKTFLLLK-EAEQDNQAVKESVQTVSVVEGGNLTARITANPRNPQLIELKNVLNKLLD 416 Tlp12

PT14 AINENILATKRGLEQDNQAVKESVQTVSVVEGGNLTARITANPRNPQLIELKNVLNKLLD 417

MTVJDCj07 AINENILATKRGLEQDNQAVKESVQTVSVVEGGNLTARITANPRNPQLIELKNVLNKLLD 417

RM1221 AINENILATKRGLEQDNQAVKESVQTVSVVEGGNLTARITANPRNPQLIELKNVLNKLLD 417

FDAARGOS_421 AINENILATKRGLEQDNQAVKESVQTVSVVEGGNLTARITANPRNPQLIELKNVLNKLLD 417

35925B2 AINENILQTKKGLEQDNQAVKESVETVSVVESGNLTARITANPRNPQLIELKNVLNRLLD 417

CJM1cam AINENILATKRGLEQDNQAVKESVQTVSVVEGGNLTARITANPRNPQLIELKNVLNRLLD 417

M1 AINENILATKRGLEQDNQAVKESVQTVSVVEGGNLTARITANPRNPQLIELKNVLNRLLD 417

S3 AINENILATKRGLEQDNQAVKESVQTVSVVEGGNLTARITANPRNPQLIELKNVLNRLLD 417

00-1597 AINENILATKRGLEQDNQAVKESVQTVSVVEGGNLTARITANPRNPQLIELKNVLNRLLD 417

R14 AINENILATKRGLEQDNQAVKESVQTVSVVEGGNLTARITANPRNPQLIELKNVLNRLLD 417

***:.:* * *** :******:**.***.************************:***

FORC_046 ALQARVGSDMNEIQRVFNSYKSLDFTTEVKDANGAVEVTTNALGQEIIKMLKQSSDFANA 480 Tlp4

FDAARGOS_422 ALQARVGSDMNEIQRVFNSYKSLDFTTEVKDANGAVEVTTNALGQEIIKMLKQSSDFANA 480

ICDCCJ07001 VLQTRVGSDMNAIHKIFEEYKSLDFRNKLDNANGSVEVTTNALGDEIVKMLKQSSDFANH 480

RM3196 VLQTRVGSDMNAIHKIFEEYKSLDFRNKLDNANGSVEVTTNALGDEIVKMLKQSSDFANH 480

T1-21 VLQARVGSDMNAIHKIFEEYKSLDFRNKLENASGSVELTTNALGDEIVKMLKQSSDFANA 480

F38011 VLQARVGSDMNAIHKIFEEYKSLDFRNKLENASGSVELTTNALGDEIVKMLKQSSDFANA 480

00-0949 VLQARVGSDMNAIHKIFEEYKSLDFRNKLENASGSVELTTNALGDEIVKMLKQSSDFANA 479

01-1512 VLQARVGSDMNAIHKIFEEYKSLDFRNKLENASGSVELTTNALGDEIVKMLKQSSDFANA 480

81-176 VLQARVGSDMNAIHKIFEEYKSLDFRNKLENASGSVELTTNALGDEIVKMLKQSSDFANA 480

32488 VLQARVGSDMNAIHKIFEEYKSLDFRNKLENASGSVELTTNALGDEIVKMLKQSSDFANA 480

NCTC11168 VLQARVGSDMNAIHKIFEEYKSLDFRNKLENASGSVELTTNALGDEIVKMLKQSSDFANA 480

CFSAN032806 VLQARVGSDMNAIHKIFEEYKSLDFRNKLENASGSVELTTNALGDEIVKMLKQSSDFANA 480

81116 VLQARVGSDMNAIHKIFEEYKSLDFRNKLENAGGSVELTTNALGDEIVKMLKQSSDFANA 480

RM1285 VLQARVGSDMNAIHKIFEEYKSLDFRNKLENASGSVELTTNALGDEIVKMLKQSSDFANA 476 Tlp12

PT14 VLQARVGSDMNAIHKIFEEYKSLDFRNKLENASGSVELTTNALGDEIVKMLKQSSDFANA 477

MTVJDCj07 VLQARVGSDMNAIHKIFEEYKSLDFRNKLENASGSVELTTNALGDEIVKMLKQSSDFANA 477

RM1221 VLQARVGSDMNAIHKIFEEYKSLDFRNKLENASGSVELTTNALGDEIVKMLKQSSDFANA 477

FDAARGOS_421 VLQARVGSDMNAIHKIFEEYKSLDFRNKLENASGSVELTTNALGDEIVKMLKQSSDFANA 477

35925B2 ALQTRVGSDMNEIQRVFNSYKSLDFTTEVKDANGAVEVTTNALGQEIIKMLKQSSDFANA 477

CJM1cam ALQARVGSDMNEIQRVFNSYKSLDFTTEVKDANGAVEVTTNALGQEIIKMLKQSSDFANA 477

M1 ALQARVGSDMNEIQRVFNSYKSLDFTTEVKDANGAVEVTTNALGQEIIKMLKQSSDFANA 477

S3 ALQARVGSDMNEIQRVFNSYKSLDFTTEVKDANGAVEVTTNALGQEIIKMLKQSSDFANA 477

00-1597 ALQARVGSDMNEIQRVFNSYKSLDFTTEVKDANGAVEVTTNALGQEIIKMLKQSSDFANA 477

R14 ALQARVGSDMNEIQRVFNSYKSLDFTTEVKDANGAVEVTTNALGQEIIKMLKQSSDFANA 477

.**:******* *:::*:.****** .::.:*.*:**:******:**:***********

FORC_046 LANESGKLQTAVQSLTTSSNSQAQSLEETAAALEEITSSMQNVSVKTSDVITQSEEIKNV 540 Tlp4

FDAARGOS_422 LANESGKLQTAVQSLTTSSNSQAQSLEETAAALEEITSSMQNVSVKTSDVITQSEEIKNV 540

ICDCCJ07001 LASESSKLQSAVQNLTSSSNSQAASLEETAAALEEITSSMQNVSVKTSDVITQSEEIKNV 540

RM3196 LASESSKLQSAVQNLTSSSNSQAASLEETAAALEEITSSMQNVSVKTSDVITQSEEIKNV 540

T1-21 LANESGKLQTAVQSLTTSSNSQAQSLEETAAALEEITSSMQNVSVKTSDVITQSEEIKNV 540

F38011 LANESGKLQTAVQSLTTSSNSQAQSLEETAAALEEITSSMQNVSVKTSDVITQSEEIKNV 540

00-0949 LANESGKLQTAVQSLTTSSNSQAQSLEETAAALEEITSSMQNVSVKTSDVITQSEEIKNV 539

01-1512 LANESGKLQTAVQSLTTSSNSQAQSLEETAAALEEITSSMQNVSVKTSDVITQSEEIKNV 540

81-176 LANESGKLQTAVQSLTTSSNSQAQSLEETAAALEEITSSMQNVSVKTSDVITQSEEIKNV 540

32488 LANESGKLQTAVQSLTTSSNSQAQSLEETAAALEEITSSMQNVSVKTSDVITQSEEIKNV 540

NCTC11168 LANESGKLQTAVQSLTTSSNSQAQSLEETAAALEEITSSMQNVSVKTSDVITQSEEIKNV 540

CFSAN032806 LANESGKLQTAVQSLTTSSNSQAQSLEETAAALEEITSSMQNVSVKTSDVITQSEEIKNV 540

81116 LANESGKLQTAVQSLTTSSNSQAQSLEETAAALEEITSSMQNVSVKTSDVITQSEEIKNV 540

RM1285 LANESGKLQTAVQSLTTSSNSQAQSLEETAAALEEITSSMQNVSVKTSDVITQSEEIKNV 536 Tlp12

PT14 LANESGKLQTAVQSLTTSSNSQAQSLEETAAALEEITSSMQNVSVKTSDVITQSEEIKNV 537

MTVJDCj07 LANESGKLQTAVQSLTTSSNSQAQSLEETAAALEEITSSMQNVSVKTSDVITQSEEIKNV 537

RM1221 LANESGKLQTAVQSLTTSSNSQAQSLEETAAALEEITSSMQNVSVKTSDVITQSEEIKNV 537

FDAARGOS_421 LANESGKLQTAVQSLTTSSNSQAQSLEETAAALEEITSSMQNVSVKTSDVITQSEEIKNV 537

35925B2 LANESGKLQTAVQSLTTSSNSQAQSLEETAAALEEITSSMQNVSVKTSDVITQSEEIKNV 537

CJM1cam LANESGKLQTAVQSLTTSSNSQAQSLEETAAALEEITSSMQNVSVKTSDVITQSEEIKNV 537

M1 LANESGKLQTAVQSLTTSSNSQAQSLEETAAALEEITSSMQNVSVKTSDVITQSEEIKNV 537

S3 LANESGKLQTAVQSLTTSSNSQAQSLEETAAALEEITSSMQNVSVKTSDVITQSEEIKNV 537

00-1597 LANESGKLQTAVQSLTTSSNSQAQSLEETAAALEEITSSMQNVSVKTSDVITQSEEIKNV 537

R14 LANESGKLQTAVQSLTTSSNSQAQSLEETAAALEEITSSMQNVSVKTSDVITQSEEIKNV 537

**.**.***:***.**:****** ************************************

FORC_046 TGIIGDIADQINLLALNAAIEAARAGEHGRGFAVVADEVRKLAERTQKSLSEIEANTNLL 600 Tlp4

FDAARGOS_422 TGIIGDIADQINLLALNAAIEAARAGEHGRGFAVVADEVRKLAERTQKSLSEIEANTNLL 600

ICDCCJ07001 TGIIGDIADQINLLALNAAIEAARAGEHGRGFAVVADEVRKLAERTQKSLSEIEANTNLL 600

RM3196 TGIIGDIADQINLLALNAAIEAARAGEHGRGFAVVADEVRKLAERTQKSLSEIEANTNLL 600

T1-21 TGIIGDIADQINLLALNAAIEAARAGEHGRGFAVVADEVRKLAERTQKSLSEIEANTNLL 600

F38011 TGIIGDIADQINLLALNAAIEAARAGEHGRGFAVVADEVRKLAERTQKSLSEIEANTNLL 600

00-0949 TGIIGDIADQINLLALNAAIEAARAGEHGRGFAVVADEVRKLAERTQKSLSEIEANTNLL 599

01-1512 TGIIGDIADQINLLALNAAIEAARAGEHGRGFAVVADEVRKLAERTQKSLSEIEANTNLL 600

81-176 TGIIGDIADQINLLALNAAIEAARAGEHGRGFAVVADEVRKLAERTQKSLSEIEANTNLL 600

32488 TGIIGDIADQINLLALNAAIEAARAGEHGRGFAVVADEVRKLAERTQKSLSEIEANTNLL 600

NCTC11168 TGIIGDIADQINLLALNAAIEAARAGEHGRGFAVVADEVRKLAERTQKSLSEIEANTNLL 600

CFSAN032806 TGIIGDIADQINLLALNAAIEAARAGEHGRGFAVVADEVRKLAERTQKSLSEIEANTNLL 600

81116 TGIIGDIADQINLLALNAAIEAARAGEHGRGFAVVADEVRKLAERTQKSLSEIEANTNLL 600

RM1285 TGIIGDIADQINLLALNAAIEAARAGEHGRGFAVVADEVRKLAERTQKSLSEIEANTNLL 596 Tlp12

PT14 TGIIGDIADQINLLALNAAIEAARAGEHGRGFAVVADEVRKLAERTQKSLSEIEANTNLL 597

MTVJDCj07 TGIIGDIADQINLLALNAAIEAARAGEHGRGFAVVADEVRKLAERTQKSLSEIEANTNLL 597

RM1221 TGIIGDIADQINLLALNAAIEAARAGEHGRGFAVVADEVRKLAERTQKSLSEIEANTNLL 597

FDAARGOS_421 TGIIGDIADQINLLALNAAIEAARAGEHGRGFAVVADEVRKLAERTQKSLSEIEANTNLL 597

35925B2 TGIIGDIADQINLLALNAAIEAARAGEHGRGFAVVADEVRKLAERTQKSLSEIEANTNLL 597

CJM1cam TGIIGDIADQINLLALNAAIEAARAGEHGRGFAVVADEVRKLAERTQKSLSEIEANTNLL 597

M1 TGIIGDIADQINLLALNAAIEAARAGEHGRGFAVVADEVRKLAERTQKSLSEIEANTNLL 597

S3 TGIIGDIADQINLLALNAAIEAARAGEHGRGFAVVADEVRKLAERTQKSLSEIEANTNLL 597

00-1597 TGIIGDIADQINLLALNAAIEAARAGEHGRGFAVVADEVRKLAERTQKSLSEIEANTNLL 597

R14 TGIIGDIADQINLLALNAAIEAARAGEHGRGFAVVADEVRKLAERTQKSLSEIEANTNLL 597

************************************************************

FORC_046 VQSINDMAESIKEQTAGITQINDSVAQIDQTTKDNVEIANESAIISSTVSDIANNILEDV 660 Tlp4

FDAARGOS_422 VQSINDMAESIKEQTAGITQINDSVAQIDQTTKDNVEIANESAIISSTVSDIANNILEDV 660

ICDCCJ07001 VQSINDMAESIKEQTAGITQINDSVAQIDQTTKDNVEIANESAIISSTVSDIANNILEDV 660

RM3196 VQSINDMAESIKEQTAGITQINDSVAQIDQTTKDNVEIANESAIISSTVSDIANNILEDV 660

T1-21 VQSINDMAESIKEQTAGITQINDSVAQIDQTTKDNVEIANESAIISSTVSDIANNILEDV 660

F38011 VQSINDMAESIKEQTAGITQINDSVAQIDQTTKDNVEIANESAIISSTVSDIANNILEDV 660

00-0949 VQSINDMAESIKEQTAGITQINDSVAQIDQTTKDNVEIANESAIISSTVSDIANNILEDV 659

01-1512 VQSINDMAESIKEQTAGITQINDSVAQIDQTTKDNVEIANESAIISSTVSDIANNILEDV 660

81-176 VQSINDMAESIKEQTAGITQINDSVAQIDQTTKDNVEIANESAIISSTVSDIANNILEDV 660

32488 VQSINDMAESIKEQTAGITQINDSVAQIDQTTKDNVEIANESAIISSTVSDIANNILEDV 660

NCTC11168 VQSINDMAESIKEQTAGITQINDSVAQIDQTTKDNVEIANESAIISSTVSDIANNILEDV 660

CFSAN032806 VQSINDMAESIKEQTAGITQINDSVAQIDQTTKDNVEIANESAIISSTVSDIANNILEDV 660

81116 VQSINDMAESIKEQTAGITQINDSVAQIDQTTKDNVEIANESAIISSTVSDIANNILEDV 660

RM1285 VQSINDMAESIKEQTAGITQINDSVAQIDQTTKDNVEIANESAIISSTVSDIANNILEDV 656 Tlp12

PT14 VQSINDMAESIKEQTAGITQINDSVAQIDQTTKDNVEIANESAIISSTVSDIANNILEDV 657

MTVJDCj07 VQSINDMAESIKEQTAGITQINDSVAQIDQTTKDNVEIANESAIISSTVSDIANNILEDV 657

RM1221 VQSINDMAESIKEQTAGITQINDSVAQIDQTTKDNVEIANESAIISSTVSDIANNILEDV 657

FDAARGOS_421 VQSINDMAESIKEQTAGITQINDSVAQIDQTTKDNVEIANESAIISSTVSDIANNILEDV 657

35925B2 VQSINDMAESIKEQTAGITQINESVAQIDQTTKDNVEIANESAIISSTVSDIANNILEDV 657

CJM1cam VQSINDMAESIKEQTAGITQINDSVAQIDQTTKDNVEIANESAIISSTVSDIANNILEDV 657

M1 VQSINDMAESIKEQTAGITQINDSVAQIDQTTKDNVEIANESAIISSTVSDIANNILEDV 657

S3 VQSINDMAESIKEQTAGITQINDSVAQIDQTTKDNVEIANESAIISSTVSDIANNILEDV 657

00-1597 VQSINDMAESIKEQTAGITQINDSVAQIDQTTKDNVEIANESAIISSTVSDIANNILEDV 657

R14 VQSINDMAESIKEQTAGITQINDSVAQIDQTTKDNVEIANESAIISSTVSDIANNILEDV 657

**********************:*************************************

FORC_046 KKKRF 665

FDAARGOS_422 KKKRF 665

ICDCCJ07001 KKKRF 665

RM3196 KKKRF 665

T1-21 KKKRF 665

F38011 KKKRF 665

00-0949 KKKRF 664

01-1512 KKKRF 665

81-176 KKKRF 665

32488 KKKRF 665

NCTC11168 KKKRF 665

CFSAN032806 KKKRF 665

81116 KKKRF 665

RM1285 KKKRF 661 Tlp12

PT14 KKKRF 662

MTVJDCj07 KKKRF 662

RM1221 KKKRF 662

FDAARGOS_421 KKKRF 662

35925B2 KKKRF 662

CJM1cam KKKRF 662

M1 KKKRF 662

S3 KKKRF 662

00-1597 KKKRF 662

R14 KKKRF 662

*****
