# Supplementary material for: Diversity of transducer-like proteins (Tlps) in Campylobacter
Source: PLoS One. 2019 Mar 25;14(3):e0214228. doi: 10.1371/journal.pone.0214228 (PMC6433261; doi:10.1371/journal.pone.0214228)
Supplement: S2 Archive — (ZIP) [file pone.0214228.s016.zip › Alignment J.docx]

Alignment J. Selected Tlp *C*-termini aligned to show diversity in protein sequence

CLUSTAL O(1.2.4) multiple sequence alignment 2018/05/22

IA3902_Tlp11 KTKNVSTIEVKSNDELGQMGKIINENILATKRGLEQDNQAVKESVETVHVVEGGNLTARI 440

00-2425_Tlp11 KTKNVSTIEVKSNDELGQMGKIINENILATKRGLEQDNQAVKESVQTVSVVEGGNLTARI 440

4031_Tlp17 KKHEVDLISIKADDELGKMGKMINENILATKKGLEQDNQAVKESVQTVSVVESGNLTARI 440

76339_Tlp18 EKIEVHTIKISSNDELGKMAKAINENILATKQGLEQDAKAVKESVETVEVVERGNLTARI 439

MTVDSCj13_Tlp13 EKIEIQTIEIKANDELGKMGKIINENILATKQGLEQDAKAVKESVETVGVVESGNLTARI 439

00-1597_Tlp13 EKIEVQTIEIKANDELGKMGKIINENILATKRGLEQDNQAVKESVQTVSVVEGGNLTARI 439

FB1_Tlp13 EKIEVQTIEIKANDELGKMGKIINENILATKRGLEQDNQAVKESVQTVSVVEGGNLTARI 439

15-537360_Tlp20 KTKNVSTIDVKTNDEFGQISKAINENILATKQGLEQDAKAVKESVETVGVVESGNLTARI 393

14983A_Tlp20 KTKNVSTIEIKSNDEFGQISKAINENILATKQGLEQDAKAVKESVETVGVVESGNLTARI 395

RM3196_Tlp23 KTKNVSTIEVKSNDEFGQISNAINENILATKRGLEQDNQAVKESVETVHVVEGGNLTARI 393

NCTC11168_Tlp2 KTKNVSTIEVKSNDEFGQISNAINENILATKRGLEQDNQAVKESVQTVSVVEGGNLTARI 393

M1_Tlp24 KTKNVSTIEVKSNDEFGQISSAINENILATKRGLEQDNQAVKESVETVSVVESGNLTARI 393

NCTC11168_Tlp3 KTKNVSTIEVKSNDEFGQISNAINENILATKRGLEQDNQAVKESVQTVSVVEGGNLTARI 396

T1-21_Tlp19a KTKNVSTIEVKSNDEFGQISNAINENILATKRGLEQDNQAVKESVQTVSVVEGGNLTARI 340

00-1597_Tlp14 KTKNVSTIEVKSNDEFGQISNAINENILATKRGLEQDNQAVKESVQTVSVVEGGNLTARI 390

BP3181_Tlp14 KTKNVSTIEIKSNDEFGQISKTINENILATKQGLEQDAKAVKESVETVGVVESGNLTARI 390

RM1875_Tlp15 KTKNVSTIEVKSNDEFGQISKAINENILATKQGLEQDAKAVKESVETVGVVESGNLTARI 313

FB1_Tlp16 KTKNVSTIDVKTNDEFGLISKAINENILATKQGLEQDAKAVKESVETVGVVERGNLTARI 313

FDAARGOS_295_Tlp21 KTKNITAINIKSKDEFGQMANAINENILATKKGLEQDNQAVKESVQTVHVVESGNLTARI 387

CG8421_Tlp25 KTKNVSTIEVKSNDEFGQISNAINENILATKRGLEQDNQAVKESVQTVSVVEGGNLTARI 181

00-1597_Tlp12 KTKNVSTIEVKSNDEFGQISNAINENILATKRGLEQDNQAVKESVQTVSVVEGGNLTARI 396

RM1221_Tlp12 KTKNVSTIEVKSNDEFGQISNAINENILATKRGLEQDNQAVKESVQTVSVVEGGNLTARI 396

ICDCCJ07001_Tlp4 KTKNVSTIEVKSNDEFGQISNAINENILATKQGLEQDAKAVKESVETVGVVESGNLTARI 399

NCTC11168_Tlp4 KTKNVSTIEVKSNDEFGQISNAINENILATKRGLEQDNQAVKESVQTVSVVEGGNLTARI 399

HF5-4A-4_Tlp22 KTKNVSTIEVKSNDEFGQISNAINENILATKRGLEQDNQAVKESVQTVSVVEGGNLTARI 345

:. :: *.:.:.**:* :.. *********:***** :******:** *** *******

IA3902_Tlp11 TANPRNPQLIELKNVLNRLLDALQARVGSDMNEIQRVFNSYKSLDFTTEVKDANGAVELT 500

00-2425_Tlp11 TANPRNPQLIELKNVLNKLLDVLQARVGSDMNAIHKIFEEYKSLDFRNKLENASGSVELT 500

4031_Tlp17 TANPRNPQLIELKNVLNKLLDVLQARVGSDMNAIHKIFEEYKSLDFRNKLENASGSVELT 500

76339_Tlp18 TANPRNPQLIELKNVLNKLLDVLQTKVGSDMNAIHKIFEEYKSLDFRNKLDNANGSVEVT 499

MTVDSCj13_Tlp13 TANPRNPQLIELKNVLNKLLDVLQARVGSDMNAIHKIFEEYKSLDFRNKLENASGSVELT 499

00-1597_Tlp13 TANPRNPQLIELKNVLNRLLDALQARVGSDMNEIQRVFNSYKSLDFTTEVKDANGAVEVT 499

FB1_Tlp13 TANPRNPQLIELKNVLNRLLDALQARVGSDMNAIHKIFEEYKSLDFRNKLDNANGSVEVT 499

15-537360_Tlp20 TANPRNPQLIELKNVLNRLLDALQARVGSDMNEIQRVFNSYKSLDFTTEVKDANGAVEVT 453

14983A_Tlp20 TANPRNPQLIELKNVLNRLLDVLQTKVGSDMNAIHKIFEEYKSLDFRNKLDNANGSVEVT 455

RM3196_Tlp23 TANPRNPQLIELKNVLNRLLDALQARVGSDMNEIQRVFNSYKSLDFTTEVKDANGAVEVT 453

NCTC11168_Tlp2 TANPRNPQLIELKNVLNKLLDVLQARVGSDMNAIHKIFEEYKSLDFRNKLENASGSVELT 453

M1_Tlp24 TANPRNPQLIELKNVLNKLLDVLQARVVLI--CYS-NF-RIQSLDFRNKLENASGSVELT 449

NCTC11168_Tlp3 TANPRNPQLIELKNVLNKLLDVLQARVGSDMNAIHKIFEEYKSLDFRNKLENASGSVELT 456

T1-21_Tlp19a TANPRNPQLIELKNVLNKLLDVLQARVGSDMNAIHKIFEEYKSLDFRNKLENASGSVELT 400

00-1597_Tlp14 TANPRNPQLIELKNVLNRLLDALQARVGSDMNEIQRVFNSYKSLDFTTEVKDANGAVEVT 450

BP3181_Tlp14 TANPRNPQLIELKNVLNRLLDVLQTKVGSDMNAIHKIFEEYKSLDFRNKLDNANGSVEVT 450

RM1875_Tlp15 TANPRNPQLIELKNVLNRLLDVLQTRVGSDMNAIHKIFEEYKSLDFRNKLDNANGSVEVT 373

FB1_Tlp16 TANPRNPQLIELKNVLNRLLDVLQTKVGSDMNAIHKIFEEYKSLDFRNKLDNANGSVEVT 373

FDAARGOS_295_Tlp21 TANPRNPQLIELKNVLNKLLDVLQARVGSDMNEIQRVFNSYKSLDFTTEVKDANGAVELT 447

CG8421_Tlp25 TANPRNPQLIELKNVLNKLLDVLQARVGSDMNAIHKIFEEYKSLDFRNKLENASGSVELT 241

00-1597_Tlp12 TANPRNPQLIELKNVLNRLLDALQARVGSDMNEIQRVFNSYKSLDFTTEVKDANGAVEVT 456

RM1221_Tlp12 TANPRNPQLIELKNVLNKLLDVLQARVGSDMNAIHKIFEEYKSLDFRNKLENASGSVELT 456

ICDCCJ07001_Tlp4 TANPRNPQLIELKNVLNRLLDVLQTRVGSDMNAIHKIFEEYKSLDFRNKLDNANGSVEVT 459

NCTC11168_Tlp4 TANPRNPQLIELKNVLNKLLDVLQARVGSDMNAIHKIFEEYKSLDFRNKLENASGSVELT 459

HF5-4A-4_Tlp22 TANPRNPQLIELKNVLNKLLDVLQARVGSDMNAIHKIFEEYKSLDFRNKLENASGSVELT 405

*****************:***.**::* * :**** .::.:*.*:**:*

IA3902_Tlp11 TNALGDEIVKMLKQSSDFANALANESGKLQTAVQSLTTSSNSQAQSLEETAAALEEITSS 560

00-2425_Tlp11 TNALGDEIVKMLKQSSDFANALANESGKLQTAVQSLTTSSNSQAQSLEETAAALEEITSS 560

4031_Tlp17 TNALGDEIVKMLKQSSDFANALANESGKLQTAVQSLTTSSNSQAQSLEETAAALEEITSS 560

76339_Tlp18 TNALGDEIVKMLKQSSDFANHLASESSKLQSAVQNLTSSSNSQAASLEETAAALEEITSS 559

MTVDSCj13_Tlp13 TNALGDEIVKMLKQSSDFANALANESGKLQTAVQSLTTSSNSQAQSLEETAAALEEITSS 559

00-1597_Tlp13 TNALGQEIIKMLKQSSDFANALANESGKLQTAVQSLTTSSNSQAQSLEETAAALEEITSS 559

FB1_Tlp13 TNALGDEIVKMLKQSSDFANHLASESSKLQSAVQNLTSSSNSQAASLEETAAALEEITSS 559

15-537360_Tlp20 TNALGQEIIKMLKQSSDFANALANESGKLQTAVQSLTTSSNSQAASLEETAAALEEITSS 513

14983A_Tlp20 TNALGDEIVKMLKQSSDFANHLASESSKLQSAVQNLTSSSNSQAASLEETAAALEEITSS 515

RM3196_Tlp23 TNALGQEIIKMLKQSSDFANALANESGKLQTAVQSLTTSSNSQAQSLEETAAALEEITSS 513

NCTC11168_Tlp2 TNALGDEIVKMLKQSSDFANALANESGKLQTAVQSLTTSSNSQAQSLEETAAALEEITSS 513

M1_Tlp24 TNALGDEIVKMLKQSSDFANALANESGKLQTAVQSLTTSSNSQAQSLEETAAALEEITSS 509

NCTC11168_Tlp3 TNALGDEIVKMLKQSSDFANALANESGKLQTAVQSLTTSSNSQAQSLEETAAALEEITSS 516

T1-21_Tlp19a TNALGDEIVKMLKQSSDFANALANESGKLQTAVQSLTTSSNSQAQSLEETAAALEEITSS 460

00-1597_Tlp14 TNALGQEIIKMLKQSSDFANALANESGKLQTAVQSLTTSSNSQAQSLEETAAALEEITSS 510

BP3181_Tlp14 TNALGDEIVKMLKQSSDFANHLASESSKLQSAVQNLTSSSNSQAASLEETAAALEEITSS 510

RM1875_Tlp15 TNALGDEIVKMLKQSSDFANHLASESSKLQSAVQNLTSSSNSQAASLEETAAALEEITSS 433

FB1_Tlp16 TNALGDEIVKMLKQSSDFANHLASESSKLQSAVQNLTSSSNSQAASLEETAAALEEITSS 433

FDAARGOS_295_Tlp21 TNALGDEIIKMLKQSSDFANALANESGKLQTAVQSLTTSSNSQAQSLEETAAALEEITSS 507

CG8421_Tlp25 TNALGDEIVKMLKQSSDFANALANESGKLQTAVQSLTTSSNSQAQSLEETAAALEEITSS 301

00-1597_Tlp12 TNALGQEIIKMLKQSSDFANALANESGKLQTAVQSLTTSSNSQAQSLEETAAALEEITSS 516

RM1221_Tlp12 TNALGDEIVKMLKQSSDFANALANESGKLQTAVQSLTTSSNSQAQSLEETAAALEEITSS 516

ICDCCJ07001_Tlp4 TNALGDEIVKMLKQSSDFANHLASESSKLQSAVQNLTSSSNSQAASLEETAAALEEITSS 519

NCTC11168_Tlp4 TNALGDEIVKMLKQSSDFANALANESGKLQTAVQSLTTSSNSQAQSLEETAAALEEITSS 519

HF5-4A-4_Tlp22 TNALGDEIVKMLKQSSDFANALANESGKLQTAVQSLTTSSNSQAQSLEETAAALEEITSS 465

*****:**:*********** **.**.***:***.**:****** ***************

IA3902_Tlp11 MQNVSVKTSDVITQSEEIKNVTGIIGDIADQINLLALNAAIEAARAGEHGRGFAVVADEV 620

00-2425_Tlp11 MQNVSVKTSDVITQSEEIKNVTGIIGDIADQINLLALNAAIEAARAGEHGRGFAVVADEV 620

4031_Tlp17 MQNVSVKTSDVITQSEEIKNVTGIIGDIADQINLLALNAAIEAARAGEHGRGFAVVADEV 620

76339_Tlp18 MQNVSVKLSDVITQSEEIKNVTGIIGDIADQINLLALNAAIEAARAGEHGRGFAVVADEV 619

MTVDSCj13_Tlp13 MQNVSVKTSDVITQSEEIKNVTGIIGDIADQINLLALNAAIEAARAGEHGRGFAVVADEV 619

00-1597_Tlp13 MQNVSVKTSDVITQSEEIKNVTGIIGDIADQINLLALNAAIEAARAGEHGRGFAVVADEV 619

FB1_Tlp13 MQNVSVKTSDVITQSEEIKNVTGIIGDIADQINLLALNAAIEAARAGEHGRGFAVVADEV 619

15-537360_Tlp20 MQNVSVKTSDVITQSEEIKNVTGIIGDIADQINLLALNAAIEAARAGEHGRGFAVVADEV 573

14983A_Tlp20 MQNVSVKTSDVITQSEEIKNVTGIIGDIADQINLLALNAAIEAARAGEHGRGFAVVADEV 575

RM3196_Tlp23 MQNVSVKTSDVITQSEEIKNVTGIIGDIADQINLLALNAAIEAARAGEHGRGFAVVADEV 573

NCTC11168_Tlp2 MQNVSVKTSDVITQSEEIKNVTGIIGDIADQINLLALNAAIEAARAGEHGRGFAVVADEV 573

M1_Tlp24 MQNVSVKTSDVITQSEEIKNVTGIIGDIADQINLLALNAAIEAARAGEHGRGFAVVADEV 569

NCTC11168_Tlp3 MQNVSVKTSDVITQSEEIKNVTGIIGDIADQINLLALNAAIEAARAGEHGRGFAVVADEV 576

T1-21_Tlp19a MQNVSVKTSDVITQSEEIKNVTGIIGDIADQINLLALNAAIEAARAGEHGRGFAVVADEV 520

00-1597_Tlp14 MQNVSVKTSDVITQSEEIKNVTGIIGDIADQINLLALNAAIEAARAGEHGRGFAVVADEV 570

BP3181_Tlp14 MQNVSVKTSDVITQSEEIKNVTGIIGDIADQINLLALNAAIEAARAGEHGRGFAVVADEV 570

RM1875_Tlp15 MQNVSVKTSDVITQSEEIKNVTGIIGDIADQINLLALNAAIEAARAGEHGRGFAVVADEV 493

FB1_Tlp16 MQNVSVKTSDVITQSEEIKNVTGIIGDIADQINLLALNAAIEAARAGEHGRGFAVVADEV 493

FDAARGOS_295_Tlp21 MQNVSVKTSDVITQSEEIKNVTGIIGDIADQINLLALNAAIEAARAGEHGRGFAVVADEV 567

CG8421_Tlp25 MQNVSVKTSDVITQSEEIKNVTGIIGDIADQINLLALNAAIEAARAGEHGRGFAVVADEV 361

00-1597_Tlp12 MQNVSVKTSDVITQSEEIKNVTGIIGDIADQINLLALNAAIEAARAGEHGRGFAVVADEV 576

RM1221_Tlp12 MQNVSVKTSDVITQSEEIKNVTGIIGDIADQINLLALNAAIEAARAGEHGRGFAVVADEV 576

ICDCCJ07001_Tlp4 MQNVSVKTSDVITQSEEIKNVTGIIGDIADQINLLALNAAIEAARAGEHGRGFAVVADEV 579

NCTC11168_Tlp4 MQNVSVKTSDVITQSEEIKNVTGIIGDIADQINLLALNAAIEAARAGEHGRGFAVVADEV 579

HF5-4A-4_Tlp22 MQNVSVKTSDVITQSEEIKNVTGIIGDIADQINLLALNAAIEAARAGEHGRGFAVVADEV 525

******* ****************************************************

IA3902_Tlp11 RKLAERTQKSLSEIEANTNLLVQSINDMAESIKEQTAGITQINDSVAQIDQTTKDNVEIA 680

00-2425_Tlp11 RKLAERTQKSLSEIEANTNLLVQSINDMAESIKEQTAGITQINDSVAQIDQTTKDNVEIA 680

4031_Tlp17 RKLAERTQKSLSEIEANTNLLVQSINDMAESIKEQTAGITQINESVAQIDQTTKDNVEIA 680

76339_Tlp18 RKLAERTQKSLSEIEANTNLLVQSINDMAESIKEQTAGITQINESVAQIDQTTKDNVEIA 679

MTVDSCj13_Tlp13 RKLAERTQKSLSEIEANTNLLVQSINDMAESIKEQTAGITQINDSVAQIDQTTKDNVEIA 679

00-1597_Tlp13 RKLAERTQKSLSEIEANTNLLVQSINDMAESIKEQTAGITQINDSVAQIDQTTKDNVEIA 679

FB1_Tlp13 RKLAERTQKSLSEIEANTNLLVQSINDMAESIKEQTAGITQINESVAQIDQTTKDNVEIA 679

15-537360_Tlp20 RKLAERTQKSLSEIEANTNLLVQSINDMAESIKEQTAGITQINESVAQIDQTTKDNVEIA 633

14983A_Tlp20 RKLAERTQKSLSEIEANTNLLVQSINDMAESIKEQTAGITQINESVAQIDQTTKDNVEIA 635

RM3196_Tlp23 RKLAERTQKSLSEIEANTNLLVQSINDMAESIKEQTAGITQINDSVAQIDQTTKDNVEIA 633

NCTC11168_Tlp2 RKLAERTQKSLSEIEANTNLLVQSINDMAESIKEQTAGITQINDSVAQIDQTTKDNVEIA 633

M1_Tlp24 RKLAERTQKSLSEIEANTNLLVQSINDMAESIKEQTAGITQINESVAQIDQTTKDNVEIA 629

NCTC11168_Tlp3 RKLAERTQKSLSEIEANTNLLVQSINDMAESIKEQTAGITQINDSVAQIDQTTKDNVEIA 636

T1-21_Tlp19a RKLAERTQKSLSEIEANTNLLVQSINDMAESIKEQTAGITQINDSVAQIDQTTKDNVEIA 580

00-1597_Tlp14 RKLAERTQKSLSEIEANTNLLVQSINDMAESIKEQTAGITQINDSVAQIDQTTKDNVEIA 630

BP3181_Tlp14 RKLAERTQKSLSEIEANTNLLVQSINDMAESIKEQTAGITQINESVAQIDQTTKDNVEIA 630

RM1875_Tlp15 RKLAERTQKSLSEIEANTNLLVQSINDMAESIKEQTAGITQINESVAQIDQTTKDNVEIA 553

FB1_Tlp16 RKLAERTQKSLSEIEANTNLLVQSINDMAESIKEQTAGITQINESVAQIDQTTKDNVEIA 553

FDAARGOS_295_Tlp21 RKLAERTQKSLSEIEANTNLLVQSINDMAESIKEQTAGITQINDSVAQIDQTTKDNVEIA 627

CG8421_Tlp25 RKLAERTQKSLSEIEANTNLLVQSINDMAESIKEQTAGITQINDSVAQIDQTTKDNVEIA 421

00-1597_Tlp12 RKLAERTQKSLSEIEANTNLLVQSINDMAESIKEQTAGITQINDSVAQIDQTTKDNVEIA 636

RM1221_Tlp12 RKLAERTQKSLSEIEANTNLLVQSINDMAESIKEQTAGITQINDSVAQIDQTTKDNVEIA 636

ICDCCJ07001_Tlp4 RKLAERTQKSLSEIEANTNLLVQSINDMAESIKEQTAGITQINDSVAQIDQTTKDNVEIA 639

NCTC11168_Tlp4 RKLAERTQKSLSEIEANTNLLVQSINDMAESIKEQTAGITQINDSVAQIDQTTKDNVEIA 639

HF5-4A-4_Tlp22 RKLAERTQKSLSEIEANTNLLVQSINDMAESIKEQTAGITQINDSVAQIDQTTKDNVEIA 585

*******************************************:****************

IA3902_Tlp11 NESAIISSTVSDIANNILEDVKKKRF 706

00-2425_Tlp11 NESAIISSTVSDIANNILEDVKKKRF 706

4031_Tlp17 NESAIISSTVSDIANNILEDVKKKRF 706

76339_Tlp18 NESAIISNTVSDIANNILEDVRKKRF 705

MTVDSCj13_Tlp13 NESAIISSTVSDIANNILEDVKKKRF 705

00-1597_Tlp13 NESAIISSTVSDIANNILEDVKKKRF 705

FB1_Tlp13 NESAIISSTVSDIANNILEDVKKKRF 705

15-537360_Tlp20 NESAIISSTVSDIANNILEDVKKKRF 659

14983A_Tlp20 NESAIISSTVSDIANNILEDVKKKRF 661

RM3196_Tlp23 NESAIISSTVSDIANNILEDVKKKRF 659

NCTC11168_Tlp2 NESAIISSTVSDIANNILEDVKKKRF 659

M1_Tlp24 NESAIISSTVSDIANNILEDVKKKRF 655

NCTC11168_Tlp3 NESAIISSTVSDIANNILEDVKKKRF 662

T1-21_Tlp19a NESAIISSTVSDIANNILEDVKKKRF 606

00-1597_Tlp14 NESAIISSTVSDIANNILEDVKKKRF 655

BP3181_Tlp14 NESAIISSTVSDIANNILEDVKKKRF 656

RM1875_Tlp15 NESAIISSTVSDIANNILEDVKKKRF 579

FB1_Tlp16 NESAIISSTVSDIANNILEDVKKKRF 579

FDAARGOS_295_Tlp21 NESAIISSTVSDIANNILEDVKKKRF 653

CG8421_Tlp25 NESAIISSTVSDIANNILEDVKKKRF 447

00-1597_Tlp12 NESAIISSTVSDIANNILEDVKKKRF 662

RM1221_Tlp12 NESAIISSTVSDIANNILEDVKKKRF 662

ICDCCJ07001_Tlp4 NESAIISSTVSDIANNILEDVKKKRF 665

NCTC11168_Tlp4 NESAIISSTVSDIANNILEDVKKKRF 665

HF5-4A-4_Tlp22 NESAIISSTVSDIANNILEDVKKKRF 611

*******.*************:***
