# Supplementary material for: Diversity of transducer-like proteins (Tlps) in Campylobacter
Source: PLoS One. 2019 Mar 25;14(3):e0214228. doi: 10.1371/journal.pone.0214228 (PMC6433261; doi:10.1371/journal.pone.0214228)
Supplement: S2 Archive — (ZIP) [file pone.0214228.s016.zip › Alignment K.docx]

Alignment K. Deletions in the C-terminal half of Tlp proteins

CLUSTAL O(1.2.4) multiple sequence alignment 2018/05/22

NCTC11168_Tlp1 LGASFIFIFVVLGVVYYCVRKIVASRLPVILSSLESFFRFLNHEKIEPKAIEIRANDELG 391

14983A_Tlp16 ---SIIFILIIIPFIFIFYRNL-IVGVQGIDANITSFFNFINHKTKNVSTIEIKSNDEFG 270

HC2-48_Tlp16 ---SIIFILIIIPFIFIFYRNL-IVGVQGIDANITSFFDFINHN-KNVSTIDVKTNDEFG 269

NCTC11168_Tlp4 VIVGIIAIIIALILIRFLISRS-LSPLAAIQTGLTSFFDFINYKTKNVSTIEVKSNDEFG 356

NCTC11168_Tlp2 TAIVIFTSIISVILLYFIVSKY-LSPLAAIQTGLTSFFDFINYKTKNVSTIEVKSNDEFG 350

HC2-48_Tlp20 IAIVIITSIISVLLLYFIVSRY-LSPLASIQVGLNSFFDFINHN-KNVSTIDVKTNDEFG 349

CO2-160_Tlp20 IAIVIITSIISVLLLYFIVSRY-LSPLASIQVGLNSFFDFINHKTKNVSTIDVKTNDEFG 350

CF2-75_Tlp20 IAIVIITSIISVLLLYFIVSRY-LSPLASIQVGLNSFFDFINHKTKNVSTIDVKTNDEFG 353

CG8421_Tlp14 VIALIVMISISI------VSKY-LSPLAAIQTGLTSFFDFINYKTKNVSTIEVKSNDEFG 341

BG2108_Tlp14 VIALIIMISISIILLYFIVSKY-LSPLAAIQT---------------------------- 319

YF2105_Tlp14 VIALIIMISISIILLYFIVSKY-LSPLAAIQTGLTSFFDFINHKTKNVSLLKQ------- 340

15-537360_Tlp14 VIALIVMISISIILLYFIVSKY-LSPLAAIQTGLTSFFDFINHKTKNVSTIEIKSNDEFG 347

WA333_Tlp14 VIALIVMISISIILLYFIVSKY-LSPLAA-----------INHKTKNVSTIEIKSNDEFG 336

HC2-48_Tlp3 AIVVIIVVVFSVILLYFIVSKY-LSPLAAIQTGLTSFFDFINYKTKNVSTIEVKTNDEFG 342

CJ677CC012_Tlp3 AIVVIIVVVFSVILLYFIVSKY-LSPLAAIQTGLTSFFDFINYKTKNVSIIEVKSNDEFG 342

NCTC11168_Tlp3 AIVVIIVVVFSVILLYFIVSKY-LSPLAAIQTGLTSFFDFINYKTKNVSTIEVKSNDEFG 353

CF2-75_Tlp3 AIVVIIVVVFSVILLYFIVSKY-LSPLAAIQTGLTSFFDFINYKTKNVSTIEVKSNDEFG 353

:. . : . :

NCTC11168_Tlp1 AMGRIINENIEKIQISLEQDQNAVDESVQTAREIEKGNLTARITKNPINPQLVELKDVLN 451

14983A_Tlp16 QISKAINENILATKQGLEQDAKAVKESVETVGVVESGNLTARITANPRNPQLIELKNVLN 330

HC2-48_Tlp16 QISKAINENILATKQGLEQDAKAVKESVETVGVVESGNLTARITANPRNPQLIELKNVLN 329

NCTC11168_Tlp4 QISNAINENILATKRGLEQDNQAVKESVQTVSVVEGGNLTARITANPRNPQLIELKNVLN 416

NCTC11168_Tlp2 QISNAINENILATKRGLEQDNQAVKESVQTVSVVEGGNLTARITANPRNPQLIELKNVLN 410

HC2-48_Tlp20 QISKAINENILATKQGLEQDAKAVKESVETVGVVESGNLTARITANPRNPQLIELKNVLN 409

CO2-160_Tlp20 QISKAINENILATKQGLEQDAKAVKESVETVGVVESGNLTARITANPRNPQLIELKNVLN 410

CF2-75_Tlp20 QISKAINENILATKQGLEQDAKAVKESVETVGVVESGNLTARITANPRNPQLIELKNVLN 413

CG8421_Tlp14 QISNAINENILATKRGLEQDNQAVKESVQTVSVVEGGNLTARITANPRNPQLIELKNVLN 401

BG2108_Tlp14 ----------------------------------------ARITANPRNPQLIELKNVLN 339

YF2105_Tlp14 -------------------------------IWF-RSCLRARITANPRNPQLIELKNVLN 368

15-537360_Tlp14 QISKAINENILATKQGLEQDAKAVKESVETVGVVESGNLTARITANPRNPQLIELKNVLN 407

WA333_Tlp14 QISKTINENILATKQGLEQDAKAVKESVETVGVVESGNLTARITANPRNPQLIELKNVLN 396

HC2-48_Tlp3 QISKAINENILATKQGLEQDAKAVKESVETVGVVESGNLTARITANPRNPQLIELKNVLN 402

CJ677CC012_Tlp3 QISSAINENILATKKGLEQDNQAVKESVQTVSVVEGGNLTARITANPRNPQLIELKNVLN 402

NCTC11168_Tlp3 QISNAINENILATKRGLEQDNQAVKESVQTVSVVEGGNLTARITANPRNPQLIELKNVLN 413

CF2-75_Tlp3 QISNAINENILATKQGLEQDAKAVKESVETVGVVESGNLTARITANPRNPQLIELKNVLN 413

**** ** ****:***:***

NCTC11168_Tlp1 RMLDVLQSKIGSNMNEINRVFDSYKALDFSTEVFNAKGEVEITTNILGKEIKKMLLASSN 511

14983A_Tlp16 RLLDVLQTKVGSDMNAIHKIFEEYKSLDFRNKLDNANGSVEVTTNALGDEIVKMLKQSSD 390

HC2-48_Tlp16 RLLDVLQTKVGSDMNAIHKIFEEYKSLDFRNKLDNANGSVEVTTNALGDEIVKMLKQSSD 389

NCTC11168_Tlp4 KLLDVLQARVGSDMNAIHKIFEEYKSLDFRNKLENASGSVELTTNALGDEIVKMLKQSSD 476

NCTC11168_Tlp2 KLLDVLQARVGSDMNAIHKIFEEYKSLDFRNKLENASGSVELTTNALGDEIVKMLKQSSD 470

HC2-48_Tlp20 RLLDVLQTKVGSDMNAIHKIFEEYKSLDFRNKLDNANGSVEVTTNALGDEIVKMLKQSSD 469

CO2-160_Tlp20 RLLDVLQTKVGSDMNAIHKIFEEYKSLDFRNKLDNANGSVEVTTNALGDEIVKMLKQSSD 470

CF2-75_Tlp20 RLLDVLQTKVGSDMNAIHKIFEEYKSLDFRNKLDNANGSVEVTTNALGDEIVKMLKQSSD 473

CG8421_Tlp14 KLLDVLQARVGSDMNAIHKIFEEYKSLDFRNKLENASGSVELTTNALGDEIVKMLKQSSD 461

BG2108_Tlp14 RLLDVLQTKVGSDMNAIHKIFEEYKSLDFRNKLDNANGSVEVTTNALGDEIVKMLKQSSD 399

YF2105_Tlp14 RLLDVLQTKVGSDMNAIHKIFEEYKSLDFRNKLDNANGSVEVTTNALGDEIVKMLKQSSD 428

15-537360_Tlp14 RLLDVLQTKVGSDMNAIHKIFEEYKSLDFRNKLDNANGSVEVTTNALGDEIVKMLKQSSD 467

WA333_Tlp14 RLLDVLQTKVGSDMNAIHKIFEEYKSLDFRNKLDNANGSVEVTTNALGDEIVKMLKQSSD 456

HC2-48_Tlp3 RLLDVLQTKVGSDMNAIHKIFEEYKSLDFRNKLDNANGSVEVTTNALGDEIVKMLKQSSD 462

CJ677CC012_Tlp3 KLLDVLQARVGSDMNVIH------------------------------------------ 420

NCTC11168_Tlp3 KLLDVLQARVGSDMNAIHKIFEEYKSLDFRNKLENASGSVELTTNALGDEIVKMLKQSSD 473

CF2-75_Tlp3 RLLDVLQTKVGSDMNAIHKIFEEYKSLDFRNKLDNANGSVEVTTNALGDEIVKMLKQSSD 473

::*****:::**:** *:

NCTC11168_Tlp1 FAKDLANQSEELKNSMQKLADGSNAQASSLEQSAAAVEEINSSMQNVSGKTVEVASQADD 571

14983A_Tlp16 FANHLASESSKLQSAVQNLTSSSNSQAASLEETAAALEEITSSMQNVSVKTSDVITQSEE 450

HC2-48_Tlp16 FANHLASESSKLQSAVQNLTSSSNSQAASLEETAAALEEITSSMQNVSVKTSDVITQS-- 447

NCTC11168_Tlp4 FANALANESGKLQTAVQSLTTSSNSQAQSLEETAAALEEITSSMQNVSVKTSDVITQSEE 536

NCTC11168_Tlp2 FANALANESGKLQTAVQSLTTSSNSQAQSLEETAAALEEITSSMQNVSVKTSDVITQSEE 530

HC2-48_Tlp20 FANHLASESSKLQSAVQNLTSSSNSQAASLEETAAALEEITSSMQNVSVKTRCY------ 523

CO2-160_Tlp20 FANHLASESSKLQSAVQNLTSSSNSQAASLEETAAALEEITSSMQNVSVKTSDVITQSEE 530

CF2-75_Tlp20 FANHLASESSKLQSAVQNLTSSSNSQAASLEETAAALEEITSSMQNVSVKTSDVITQSEE 533

CG8421_Tlp14 FANALANESGKLQTAVQSLTTSSNSQAQSLEETAAALEEITSSMQNVSVKTSDVITQSEE 521

BG2108_Tlp14 FANHLASESSKLQSAVQNLTSSSNSQAASLEETAAALEEITSSMQNVSVKTSDVITQSEE 459

YF2105_Tlp14 FANHLASESSKLQSAVQNLTSSSNSQAASLEETAAALEEITSSMQNVSVKTSDVITQSEE 488

15-537360_Tlp14 FANHLASESSKLQSAVQNLTSSSNSQAASLEETAAALEEITSSMQNVSVKTSDVITQSEE 527

WA333_Tlp14 FANHLASESSKLQSAVQNLTSSSNSQAASLEETAAALEEITSSMQNVSVKTSDVITQV-- 514

HC2-48_Tlp3 FANHLARKFKTSKCSSKPYFIF-------------------------------------- 484

CJ677CC012_Tlp3 ------------------------------------------------------------ 420

NCTC11168_Tlp3 FANALANESGKLQTAVQSLTTSSNSQAQSLEETAAALEEITSSMQNVSVKTSDVITQSEE 533

CF2-75_Tlp3 FANHLASESSKLQSAVQNLTSSSNSQAASLEETAAALEEITSSMQNVSVKTSDVITQSEE 533

NCTC11168_Tlp1 IKNIVNVIKDIAEQTNLLALNAAIEAARAGEHGRGFAVVADEVRQLAERTGKSLSEIEAN 631

14983A_Tlp16 IKNVTGIIGDIADQINLLALNAAIEAARAGEHGRGFAVVADEVRKLAERTQKSLSEIEAN 510

HC2-48_Tlp16 -------------------------------------------------------EIEAN 452

NCTC11168_Tlp4 IKNVTGIIGDIADQINLLALNAAIEAARAGEHGRGFAVVADEVRKLAERTQKSLSEIEAN 596

NCTC11168_Tlp2 IKNVTGIIGDIADQINLLALNAAIEAARAGEHGRGFAVVADEVRKLAERTQKSLSEIEAN 590

HC2-48_Tlp20 ----------------------------------------------------HSIEIEAN 531

CO2-160_Tlp20 IKNVTGIIGDIADQINLLALNAAIEAARAGEHGRGFAVVADEVRKLAERTQKSLSEIEAN 590

CF2-75_Tlp20 IKNVTGIIGDIADQINL----------------------------LAEITQKSLSEIEAN 565

CG8421_Tlp14 IKNVTGIIGDIADQINLLALNAAIEAARAGEHGRGFAVVADEVRKLAERTQKSLSEIEAN 581

BG2108_Tlp14 IKNVTGIIGDIADQINLLALNAAIEAARAGEHGRGFAVVADEVRKLAERTQKSLSEIEAN 519

YF2105_Tlp14 IKNVTGIIGDIADQINLLALNAAIEAARAGEHGRGFAVVADEVRKLAERTQKSLSEIEAN 548

15-537360_Tlp14 IKNVTGIIGDIADQINLLALNAAIEAARAGEHGRGFAVVADEVRKLAERTQKSLSEIEAN 587

WA333_Tlp14 -------------------------------------------------------EIEAN 519

HC2-48_Tlp3 -------------------FSSSFFRRNCSCFRRDYFFYAKCF--CKNQ-CYHSIEIEAN 522

CJ677CC012_Tlp3 ----------------------------------------DEVRKLAERTQKSLSEIEAN 440

NCTC11168_Tlp3 IKNVTGIIGDIADQINLLALNAAIEAARAGEHGRGFAVVADEVRKLAERTQKSLSEIEAN 593

CF2-75_Tlp3 IKNVTGIIGDIADQINLLA----------------------------EITQKSLSEIEAN 565

*****

NCTC11168_Tlp1 INILVQSVNEVAESVKEQTAGITQINDAIAQLETVTKENVEVANVTNNITNEVNQIAAAI 691

14983A_Tlp16 TNLLVQSINDMAESIKEQTAGITQINESVAQIDQTTKDNVEIANESAIISSTVSDIANNI 570

HC2-48_Tlp16 TNLLVQSINDMAESIKEQTAGITQINESVAQIDQTTKDNVEIANESAIISSTVSDIANNI 512

NCTC11168_Tlp4 TNLLVQSINDMAESIKEQTAGITQINDSVAQIDQTTKDNVEIANESAIISSTVSDIANNI 656

NCTC11168_Tlp2 TNLLVQSINDMAESIKEQTAGITQINDSVAQIDQTTKDNVEIANESAIISSTVSDIANNI 650

HC2-48_Tlp20 TNLLVQSINDMAESIKEQTAGITQINESVAQIDQTTKDNVEIANESAIISSTVSDIANNI 591

CO2-160_Tlp20 TNLLVQSINDMAESIKEQTAGITQINESVAQIDQTTKDNVEIANESAIISSTVSDIANNI 650

CF2-75_Tlp20 TNLLVQSINDMAESIKEQTAGITQINESVAQIDQTTKDNVEIANESAIISSTVSDIANNI 625

CG8421_Tlp14 TNLLVQSINDMAESIKEQTAGITQINDSVAQIDQTTKDNVEIANESAIISSTVSDIANNI 641

BG2108_Tlp14 TNLLVQSINDMAESIKEQTAGITQINESVAQIDQTTKDNVEIANESAIISSTVSDIANNI 579

YF2105_Tlp14 TNLLVQSINDMAESIKEQTAGITQINESVAQIDQTTKDNVEIANESAIISSTVSDIANNI 608

15-537360_Tlp14 TNLLVQSINDMAESIKEQTAGITQINESVAQIDQTTKDNVEIANESAIISNTVSDIANNI 647

WA333_Tlp14 TNLLVQSINDMAESIKEQTAGITQINESVAQIDQTTKDNVEIANESAIISSTVSDIANNI 579

HC2-48_Tlp3 TNLLVQSINDMAESIKEQTAGITQINESVAQIDQTTKDNVEIANESAIISSTVSDIANNI 582

CJ677CC012_Tlp3 TNLLVQSINDMAESIKEQTAGITQINDSVAQIDQTTKDNVEIANESAIISSTVSDIANNI 500

NCTC11168_Tlp3 TNLLVQSINDMAESIKEQTAGITQINDSVAQIDQTTKDNVEIANESAIISSTVSDIANNI 653

CF2-75_Tlp3 TNLLVQSINDMAESIKEQTAGITQINESVAQIDQTTKDNVEIANESAIISSTVSDIANNI 625

*:****:*::***:***********:::**:: .**:***:** : *:. *.:** *

NCTC11168_Tlp1 LEDVNKKRF 700

14983A_Tlp16 LEDVKKKRF 579

HC2-48_Tlp16 LEDVKKKRF 521

NCTC11168_Tlp4 LEDVKKKRF 665

NCTC11168_Tlp2 LEDVKKKRF 659

HC2-48_Tlp20 LEDVKKKRF 600

CO2-160_Tlp20 LEDVKKKRF 659

CF2-75_Tlp20 LEDVKKKRF 634

CG8421_Tlp14 LEDVKKKRF 650

BG2108_Tlp14 LEDVKKKRF 588

YF2105_Tlp14 LEDVKKKRF 617

15-537360_Tlp14 LEDVKKKRF 656

WA333_Tlp14 LEDVKKKRF 588

HC2-48_Tlp3 LEDVKKKRF 591

CJ677CC012_Tlp3 LEDVKKKRF 509

NCTC11168_Tlp3 LEDVKKKRF 662

CF2-75_Tlp3 LEDVKKKRF
