# Supplementary material for: Diversity of transducer-like proteins (Tlps) in Campylobacter
Source: PLoS One. 2019 Mar 25;14(3):e0214228. doi: 10.1371/journal.pone.0214228 (PMC6433261; doi:10.1371/journal.pone.0214228)
Supplement: S2 Archive — (ZIP) [file pone.0214228.s016.zip › Alignment L.docx]

Alignment L. All *C. jejuni* and *C. coli* full-length Tlps

CLUSTAL O(1.2.4) multiple sequence alignment

FDAARGOS_295_Tlp1 ----------MFKSLNV-GLKLVFSVAIVVVIGLVILISLVTKQVSQSITENAEDIIASV 49

14980A_Tlp1 ----------MFKSLNI-GLKLIFSVAAVVVIGLVILISLITKQVSQNITKNTEDILASI 49

CJ677CC527_Tlp1 ----------MFKSLNI-GLKLIFSVATVVVIGLIILISLITKQVSQNITKNTEDILASI 49

CJ677CC012_Tlp1 ----------MFKSLNI-GLKLIFSVATVVVIGLIILISLITKQVSQNITKNTEDILASI 49

4031_Tlp1 ----------MFKSLNI-GLKLIFSVATVVVIGLVILISLITKQVSQNITKNTEDILASI 49

81116_Tlp1 ----------MFKSLNI-GLKLIFSVATVVVIGLVILISLITKQVSQNITKNTEDILASI 49

35925B2_Tlp1 ----------MFKSLNI-GLKLIFSVATVVVIGLVILISLITKQVSQNITKNTEDILASI 49

M1_Tlp1 ----------MFKSLNI-GLKLIFSVATVVVIGLVILISLITKQVSQNITKNTEDILASI 49

PT14_Tlp1 ----------MFKSLNI-GLKLIFSVATVVVIGLVILISLITKQVSQNITKNTEDILASI 49

81-176_Tlp1 ----------MFKSLNI-GLKLIFSVAAVVVIGLVILISLITKQVSQNITKNTEDILASI 49

CVMN29710_Tlp1 ----------MFKSLNI-GLKLIFSVAAVVVIGLVILISLITKQVSQNITKNTEDILASI 49

FB1_Tlp1 ----------MFKSLNI-GLKLIFSVAAVVVIGLVILISLITKQVSQNITKNTEDILASI 49

BG2108_Tlp1 ----------MFKSLNI-GLKLIFSVAAVVVIGLVILISLITKQVSQNITKNTEDILASI 49

YF2108_Tlp1 ----------MFKSLNI-GLKLIFSVAAVVVIGLVILISLITKQVSQNITKNTEDILASI 49

YH501_Tlp1 ----------MFKSLNI-GLKLIFSVAAVVVIGLVILISLITKQVSQNITKNTEDILASI 49

RM4661_Tlp1 ----------MFKSLNI-GLKLIFSVAAVVVIGLVILISLITKQVSQNITKNTEDILASI 49

F38011_Tlp1 ----------MFKSLNI-GLKLIFSVAAVVVIGLVILISLITKQVSQNITKNTEDILASI 49

T1-21_Tlp1 ----------MFKSLNI-GLKLIFSVAAVVVIGLVILISLITKQVSQNITKNTEDILASI 49

CG8421_Tlp1 ----------MFKSLNI-GLKLIFSVAAVVVIGLVILISLITKQVSQNITKNTEDILASI 49

CJM1cam_Tlp1 ----------MFKSLNI-GLKLIFSVAAVVVIGLVILISLITKQVSQNITKNTEDILASI 49

R14_Tlp1 ----------MFKSLNI-GLKLIFSVAAVVVIGLVILISLITKQVSQNITKNTEDILASI 49

ICDCCJ07001_Tlp1 ----------MFKSLNI-GLKLIFSVAAVVVIGLVILISLITKQVSQNITKNTEDILASI 49

RM3196_Tlp1 ----------MFKSLNI-GLKLIFSVAAVVVIGLVILISLITKQVSQNITKNTEDILASI 49

NCTC11168_Tlp1 ----------MFKSLNI-GLKLIFSVAAVVVIGLVILISLITKQVSQNITKNTEDILASI 49

00-2425_Tlp1 ----------MFKSLNI-GLKLIFSVAAVVVIGLVILISLITKQVSQNITKNTEDILASI 49

IA3902_Tlp1 ----------MFKSLNI-GLKLIFSVAAVVVIGLVILISLITKQVSQNITKNTEDILASI 49

RM1285_Tlp1 ----------MFKSLNI-GLKLIFSVAAVVVIGLVILISLITKQVSQNITKNTEDILASI 49

00-0949_Tlp1 ----------MFKSLNI-GLKLIFSVAAVVVIGLVILISLITKQVSQNITKNTEDILASI 49

01-1512_Tlp1 ----------MFKSLNI-GLKLIFSVAAVVVIGLVILISLITKQVSQNITKNTEDILASI 49

FDAARGOS_422_Tlp1 ----------MFKSLNI-GLKLIFSVAAVVVIGLVILISLITKQVSQNITKNTEDILASI 49

FORC_056_Tlp1 ----------MFKSLNI-GLKLIFSVAAVVVIGLVILISLITKQVSQNITKNTEDILASI 49

32488_Tlp1 ----------MFKSLNI-GLKLIFSVAAVVVIGLVILISLITKQVSQNITKNTEDILASI 49

CFSAN032806_Tlp1 ----------MFKSLNI-GLKLIFSVAAVVVIGLVILISLITKQVSQNITKNTEDILASI 49

YH001_Tlp1 ----------MFKSLNI-GLKLIFSVAAVVVIGLVILISLITKQVSQNITKNTEDILASI 49

00-6200_Tlp1 ----------MFKSLNI-GLKLIFSVAAVVVIGLVILISLITKQVSQNITKNTEDILASI 49

RM1221_Tlp1 ----------MFKSLNI-GLKLIFSVAAVVVIGLVILISLITKQVSQNITKNTEDILASI 49

S3_Tlp1 ----------MFKSLNI-GLKLIFSVAAVVVIGLVILISLITKQVSQNITKNTEDILASI 49

FDAARGOS_421_Tlp1 ----------MFKSLNI-GLKLIFSVAAVVVIGLVILISLITKQVSQNITKNTEDILASI 49

FJ3124_Tlp1 ----------MFKSLNI-GLKLIFSVAAVVVIGLVILISLITKQVSQNITKNTEDILASI 49

00-1597_Tlp1 ----------MFKSLNI-GLKLIFSVAAVVVIGLVILISLITKQVSQNITKNTEDILASI 49

15-537360_Tlp20 ----------------MKSVKIKVSLIANLIAIVCLIF--LGIITFIFVKDEVFNQVVKS 42

76339_Tlp20 ----------------MKSVKIKVSLIANLIAIVCLIF--LGIITFIFVKDEVFNQVVKA 42

HC2-48_Tlp20 ----------------VKSVKIKVSLIANLIAIVCLIF--LGIITFIFVKDEVFNQVVKS 42

CFSAN032805_Tlp20 ----------------VKSVKIKVSLIANLIAIVCLIF--LGIITFIFVKDEVFNQVVKS 42

CVMN29710_Tlp20 ----------------VKSVKIKVSLIANLIAIVCLI---LGIITFIFVKDEVFNQVVKS 41

YH501_Tlp20 -------------VKSVKSVKIKVSLIANLIAIVCLIL--LGIITFIFVKDEVFNQVVKS 45

CF2-75_Tlp20 -------------VKSVKSVKIKVSLIANLIAIVCLIF--LGIITFIFVKDEVFNQVVKS 45

CO2-160_Tlp20 ----------------MKSVKIKVSLIANLIAIVCLIF--LGIITFIFVKDEVFNQVVKS 42

CO2-160_Tlp20b ----------------MKSVKIKVSLIANLIAIVCLIF--LGIITFIFVKDEVFNQVVKS 42

RM5611_Tlp20 ----------------MKSVKIKVSLIANLIAIVCLIF--LGIITFIFVKDEVFNQVVKS 42

14903A_Tlp20 -------------VKSVKSVKIKVSLIANLIAIVCLIF--LGIITFIFVKDEVFNQVVKS 45

YH502_Tlp20 ----------------VKSVKIKVSLIANLIAIVCLIF--LGIITFIFVKDEVFNQVVKS 42

RM3196_Tlp23 ----------------MKSVKLKVTLIANLITVVCLVI--LGVITFMFVKQAIFHEVVNA 42

ICDCCJ07001_Tlp23 ----------------MKSVKLKVTLIANLITVVCLVI--LGVITFMFVKQAIFHEVVNA 42

RM1285_Tlp2 ----------------MKSVKLKVSLIANLIAVVCLII--LGVVTFIFVKQAIFHEVVNA 42

CFSAN032806_Tlp2 ----------------MKSVKLKVSLIANLIAVVCLII--LGVVTFIFVKQAIFHEVVNA 42

RM1221_Tlp2 ----------------MKSVKLKVSLIANLIAVVCLII--LGVVTFIFVKQAIFHEVVNA 42

S3_Tlp2 ----------------MKSVKLKVSLIANLIAVVCLII--LGVVTFIFVKQAIFHEVVNA 42

FDAARGOS_422_Tlp2 ----------------MKSVKLKVSLIANLIAVVCLII--LGVVTFMFVKQAIFHEVVKA 42

81-176_Tlp2 ----------------MKSVKLKVSLIANLIAVVCLII--LGVVTFIFVKQAIFHEVVNA 42

F38011_Tlp2 ----------------MKSVKLKVSLIANLIAVVCLII--LGVVTFIFVKQAIFHEVVNA 42

NCTC11168_Tlp2 ----------------MKSVKLKVSLIANLIAVVCLII--LGVVTFIFVKQAIFHEVVNA 42

MTVDSCj07_Tlp2 ----------------MKSVKLKVSLIANLIAVVCLII--LGVVTFIFVKQAIFHEVVNA 42

CJM1cam_Tlp24 ----------------MKSVKLKVALIANLIAVVCLVI--LGVITFMFVKQAIFHEVVKA 42

M1_Tlp24 ----------------MKSVKLKVALIANLIAVVCLVI--LGVITFMFVKQAIFHEVVKA 42

81116_Tlp2 ----------------MKSVKLKVALIANLIAVVCLVI--LGVITFMFVKQAIFHEVVKA 42

4031_Tlp23 ----------------MKSVKLKVALIANLIAVVCLVI--LGVITFMFVKQAIFHEVVKA 42

CVMN29710_Tlp14 ----------------MNNIKIKLSVIANSIAIFALSI--LSIISFYFTKDSLYQSTLYA 42

RM4661_Tlp14 ----------------MNNIKIKLSVIANSIAIFALSI--LSIISFYFTKDSLYKSTLYA 42

MG1116_Tlp14 ----------------MNNIKIKLSVIANSIAIFALSI--LSIISFYFTKDSLYQSTLHA 42

BG2108_Tlp14 ----------------MNNIKIKLSVIANSIAIFALSI--LSIISFYFTKDSLYQSTLHA 42

YF2105_Tlp14 ----------------MNNIKIKLSVIANSIAIFALSI--LSIISFYFTKDSLYQSTLHA 42

YH502_Tlp14 -----MLKITKIKRKNMNNIKIKLSVIANSIAIFALSI--LSIISFYFTKDSLYQSTLHA 53

WA333_Tlp14 ----------------MNNIKIKLSVIANSIAIFALSI--LSIISFYFTKDSLYQSTLHA 42

BP3181_Tlp14 ----------------MNNIKIKLSVIANSIAIFALSI--LSIISFYFTKDSLYQSTLYT 42

ZV1224_Tlp14a ----------------MNNIKIKLSVIANSIAIFALSI--LSIISFYFTKDSLYQSTLHT 42

ZV1224_Tlp14b ----------------MNNIKIKLSVIANSIAIFALSI--LSIISFYFTKDSLYQSTLHT 42

YH503_Tlp14 -----MLKITKIKRKNMNNIKIKLSVIANSIAIFALSI--LSIISFYFTKDSLYQSTLHA 53

14903A_Tlp14 ----------------MNNIKIKLSVIANSIAIFALSI--LSIISFYFTKDSLYQSTLHA 42

OR12_Tlp14 ----------------MNNIKIKLSVIANSIAIFALSI--LSIISFYFTKDSLYQSTLHA 42

CFSAN032805_Tlp14 -----MLKITKIKRKNMNNIKIKLSVIANSIAIFALSI--LSIISFYFTKDSLYQSTLHA 53

BFR-CA-9557_Tlp14 ----------------MNNIKIKLSVIANSIAIFALSI--LSIISFYFTKDSLYQSTLHA 42

15-537360_Tlp14 ----------------MNNIKIKLSVIANSIAIFALSI--LSIISFYFTKDSLYQSTLHA 42

YH501_Tlp14 ----------------MNNIKIKLSVIANSIAIFALSI--LSIISFYFTKDSLYQSTLHA 42

T1-21_Tlp19b ----------------MNNIKIKLSLIANSITIFALSI--LSIISFYFTKDSLYQSTLYT 42

CG8421_Tlp14 ----------------MNNIKIKLSVIANSIAIFALSI--LSIISFYFTKDSLYQSTLYT 42

MTVDSCj16_Tlp14 ----------------MNNIKIKLSVIANSIAIFALSI--LSIISFYFTKDSLYQSTLYT 42

01-1512_Tlp14 ----------------MNNIKIKLSVIANSIAIFALSI--LSIISFYFTKDSLYQSTLYT 42

00-0949_Tlp14 ----------------MNNIKIKLSVIANSIAIFALSI--LSIISFYFTKDSLYQSTLYT 42

MTVDSCj13_Tlp14 ----------------MNNIKIKLSVIANSIAIFALSI--LSIISFYFTKDSLYQSTLYT 42

S3_Tlp14 ----------------MNNIKIKLSVIANSIAIFALSI--LSIISFYFTKDSLYQSTLYT 42

PT14_Tlp14 ----------------MNNIKIKLSVIANSIAIFALSI--LSIISFYFTKDSLYQSTLYT 42

14980A_Tlp14 MLKVLLQKLIKFKRKNMNNIKIKLSVIANSIAIFALSI--LSIISFYFTKDSLYQSTLYT 58

FJ3124_Tlp14 ----------------MNNIKIKLSVIANSIAIFALSI--LSIISFYFTKDSLYQSTLYT 42

00-1597_Tlp14 ----------------MNNIKIKLSVIANSIAIFALSI--LSIISFYFTKDSLYQSTLYT 42

R14_Tlp14 ----------------MNNIKIKLSVIANSIAIFALSI--LSIISFYFTKDSLYQSTLYT 42

CG8421_Tlp25 ------------------------------------------------------------ 0

HC2-48_Tlp3 ----------------MNSIKIKLSLIANLIAIFALIV--LGIVSFYFTKTSLYESTLKN 42

35925B2_Tlp3 -----MLKITKIKRKIMNSIKIKLSLIANLIAIFALIV--LGIVSFYFTKTSLHESALKN 53

14980A_Tlp3 -----MLKITKIKRKIMNSIKIKLSLIANLIAIFALIV--LGIVSFYFTKTSLYESTLKN 53

00-1597_Tlp3b ----------------MNSIKIKLSLIANLIAIFALIV--LGIVSFYFTKTSLYESTLKN 42

ICDCCJ07001_Tlp3 ----------------MNSIKIKLSLIANLIAIFALIV--LGIVSFYFTKTSLYESTLKN 42

RM3196_Tlp3 ----------------MNSIKIKLSLIANLIAIFALIV--LGIVSFYFTKTSLYESTLKN 42

RM1875_Tlp3 ----------------MNSIKIKLSLIANLIAIFALIV--LGIVSFYFTKTSLYESTLKN 42

CF2-75_Tlp3 -----MLKITKIKRKIMNSIKIKLSLIANLIAIFALIV--LGIVSFYFTKTSLYESTLKN 53

RM5611_Tlp3 ----------------MNSIKIKLSLIANLIAIFALIV--LGIVSFYFTKTSLYESTLKN 42

MTVDSCj16_Tlp3 ----------------MNSIKIKLSLIANLIAIFALIV--LGIVSFYFTKTSLYESTLKN 42

01-1512_Tlp3 ----------------MNSIKIKLSLIANLIAIFALIV--LGIVSFYFTKTSLYESTLKN 42

MTVDSCj13_Tlp3 ----------------MNSIKIKLSLIANLIAIFALIV--LGIVSFYFTKTSLYESTLKN 42

32488_Tlp3a ----------------MNSIKIKLSLIANLIAIFALIV--LGIVSFYFTKTSLYESTLKN 42

81116_Tlp3 ----------------MNSIKIKLSLIANLIAIFALIV--LGIVSFYFTKTSLYESTLKN 42

32488_Tlp3b ----------------MNNIKIKLSVIANSIAIFALIV--LGIVSFYFTKTSLYESTLKN 42

FB1_Tlp3 ----------------MNSIKIKLSLIANLIAIFALIV--LGIVSFYFTKTSLYESTLKN 42

PT14_Tlp3 ----------------MNSIKIKLSLIANLIAIFALIV--LGIVSFYFTKTSLYESTLKN 42

00-6200_Tlp3a ----------------MNSIKIKLSLIANLIAIFALIV--LGIVSFYFTKTSLYESTLKN 42

RM1221_Tlp3 ----------------MNSIKIKLSLIANLIAIFALIV--LGIVSFYFTKTSLYESTLKN 42

S3_Tlp3 ----------------MNSIKIKLSLIANLIAIFALIV--LGIVSFYFTKTSLYESTLKN 42

FDAARGOS_421_Tlp3 -----MLKITKIKRKIMNSIKIKLSLIANLIAIFALIV--LGIVSFYFTKTSLYESTLKN 53

CFSAN032806_Tlp3 -----MLKITKIKRKIMNSIKIKLSLIANLIAIFALIV--LGIVSFYFTKTSLYESTLKN 53

IA3901_Tlp3b ----------------MNSIKIKLSLIANLIAIFALIV--LGIVSFYFTKTSLYESTLKN 42

00-6200_Tlp3b ----------------MNSIKIKLSLIANLIAIFALIV--LGIVSFYFTKTSLYESTLKN 42

BCW_6290_Tlp3b ----------------MNSIKIKLSLIANLIAIFALIV--LGIVSFYFTKTSLYESTLKN 42

00-2425_Tlp3a ----------------MNSIKIKLSLIANLIAIFALIV--LGIVSFYFTKTSLYESTLKN 42

00-2425_Tlp3b ----------------MNSIKIKLSLIANLIAIFALIV--LGIVSFYFTKTSLYESTLKN 42

YH001_Tlp3a ----------------MNSIKIKLSLIANLIAIFALIV--LGIVSFYFTKTSLYESTLKN 42

YH001_Tlp3b ----------------MNSIKIKLSLIANLIAIFALIV--LGIVSFYFTKTSLYESTLKN 42

00-0949_Tlp3 ----------------MNSIKIKLSLIANLIAIFALIV--LGIVSFYFTKTSLYESTLKN 42

NCTC11168_Tlp3 -----MLKITKIKRKIMNSIKIKLSLIANLIAIFALIV--LGIVSFYFTKTSLYESTLKN 53

F38011_Tlp3 ----------------MNSIKIKLSLIANLIAIFALIV--LGIVSFYFTKTSLYESTLKN 42

RM1285_Tlp3 ----------------MNSIKIKLSLIANLIAIFALIV--LGIVSFYFTKTSLYESTLKN 42

FDAARGOS_422_Tlp3 -----MLKITKIKRKIMNSIKIKLSLIANLIAIFALIV--LGIVSFYFTKTSLYESTLKN 53

MTVDSCj07_Tlp3 ----------------MNSIKIKLSLIANLIAIFALIV--LGIVSFYFTKTSLYESTLKN 42

IA3901_Tlp3a ----------------MNSIKIKLSLIANLIAIFALIV--LGIVSFYFTKTSLYESTLKN 42

BCW_6290_Tlp3a ----------------MNSIKIKLSLIANLIAIFALIV--LGIVSFYFTKTSLYESTLKN 42

T1-21_Tlp19a ----------------MNSIKIKLSLIANLIAIFALIV--LGIVSFYFTKTSLYESTLKN 42

CJ677CC012_Tlp3 ----------------MNSIKIKLSLIANLIAIFALIV--LGIVSFYFTKTSLHESALKN 42

CJM1cam_Tlp3 ----------------MNSIKIKLSLIANLIAIFALIV--LGIVSFYFTKTSLHESALKN 42

M1_Tlp3 ----------------MNSIKIKLSLIANLIAIFALIV--LGIVSFYFTKTSLHESALKN 42

4031_Tlp3 ----------------MNSIKIKLSLIANLIAIFALIV--LGIVSFYFTKTSLHESALKN 42

R14_Tlp3 ----------------MNSIKIKLSLIANLIAIFALIV--LGIVSFYFTKTSLYESTLKN 42

FDAARGOS_295_Tlp21 ------------MKSSI-STKLTILIGILIVLAFGISSM-----ISYLSSLNNSRSLLQN 42

FORC_046_Tlp4 -----MQSINSGKSVGI-SAKLTLWVGILVVLILAITSA-----ISYFDSRNNTYELLKD 49

FDAARGOS_422_Tlp4 -----MQSINSGKSVGI-SAKLTLWVGILVVLILAITSA-----ISYFDSRNNTYELLKD 49

ICDCCJ07001_Tlp4 -----MQSINSGKSVGI-SAKLTLWVGILVVLILAITSA-----ISYFDSRNNTYELLKD 49

RM3196_Tlp4 -----MQSINSGKSVGI-SAKLTLWVGILVVLILAITSA-----ISYFDSRNNTYELLKD 49

T1-21_Tlp4 -----MQSINSGKSVGI-SAKLTLWVGILVVLILAITSA-----ISYFDSRNNTYELLKD 49

F38011_Tlp4 -----MQSINSGKSAGI-SAKLTLWVGILVVLILAITSA-----VSYFDSRNNTYELLKD 49

HF5-4A-4_Tlp22 ------------------------------------------------------------ 0

00-0949_Tlp4 -----MQSINSGKSVGI-SAKLTLWVGILVVLILAITSA-----ISYFDSRNNTYELLKD 49

01-1512_Tlp4 -----MQSINSGKSVGI-SAKLTLWVGILVVLILAITSA-----ISYFDSRNNTYELLKD 49

81-176_Tlp4 -----MQSINSGKSVGI-SAKLTLWVGILVVLILAITSA-----ISYFDSRNNTYELLKD 49

32488_Tlp4 -----MQSINSGKSVGI-SAKLTLWVGILVVLILAITSA-----ISYFDSRNNTYELLKD 49

NCTC11168_Tlp4 -----MQSINSGKSVGI-SAKLTLWVGILVVLILAITSA-----ISYFDSRNNTYELLKD 49

CFSAN032806_Tlp4 -----MQSINSGKSVGI-SAKLTLWVGILVVLILAITSA-----ISYFDSRNNTYELLKD 49

81116_Tlp4 -----MQSINSGKSVGI-SAKLTLWVGILVVLILAITSA-----ISYFDSRNNTYELLKD 49

RM1285_Tlp12 -----MQSINSGKSVGI-SAKLTLWVGILVVLILAITST-----VSYFDAKNHTYELLKE 49

PT14_Tlp12 -----MQSINSGKSVGI-SAKLTLWVGILVVLILAITST-----VSYFDAKNHTYELLKE 49

MTVJDCj07_Tlp12 -----MQSINSGKSVGI-SAKLTLWVGILVVLILAITST-----VSYFDAKNHTYELLKE 49

RM1221_Tlp12 -----MQKMDSGKSVGV-SVKLTLWVGILVVLILAITST-----VSYFDAKNHTYELLKE 49

FDAARGOS_421_Tlp12 -----MQKMDSGKSVGV-SVKLTLWVGILVVLILAITST-----VSYFDAKNHTYELLKE 49

35925B2_Tlp12 -----MQKMNSGKSVGI-SAKLTLWVGILVVLILAITSA-----VSYFDAKNHTYELLKE 49

CJM1cam_Tlp12 -----MQKMNSGKSVGI-SAKLTLWVGILVVLILAITSA-----VSYFDAKNHTYELLKE 49

M1_Tlp12 -----MQKMNSGKSVGI-SAKLTLWVGILVVLILAITSA-----VSYFDAKNHTYELLKE 49

S3_Tlp12 -----MQKMDSGKSVGV-SVKLTLWVGILVVLILAITST-----VSYFDAKNHTYELLKE 49

00-1597_Tlp12 -----MQSINSGKSVGV-SVKLTLWVGILVVLILAITST-----VSYFDAKNHTYELLKE 49

R14_Tlp12 -----MQSINSGKSVGI-SAKLTLWVGILVVLILAITST-----VSYFDAKNHTYELLKE 49

RM1875_Tlp15 ------------MKLSI-RKKMLMLG------GICLVSMLITFGIFYYNNLQGSEKIAQI 41

YH503_Tlp16 ------------MQLSI-RKKMLMLG------AICFISMLATFAIFYYNNLKGSQKIAQT 41

FB1_Tlp16 ------------MQLSI-RKKMLMLG------AICFISMLATFAIFYYNNLKGSQKIAQT 41

BFR-CA-9557_Tlp16 ------------MQLSI-RKKMLMLG------AICFISMLATFAIFYYNNLKGSQKIAQT 41

15-537360_Tlp16 ------------MQLSI-RKKMLMLG------AICFISMLATFAIFYYNNLKGSQKIAQT 41

OR12_Tlp16 ------------MQLSI-RKKMLMLG------AICFISMLATFAIFYYNNLKGSQKIAQT 41

YH502_Tlp16 ------------MQLSI-RKKMLMLG------AICFISMLATFAIFYYNNLKGSQKIAQT 41

14903A_Tlp16 ------------MQLSI-RKKMLMLG------AICFISMLATFAIFYYNNLQGSQKIAQT 41

HC2-48_Tlp16 ------------MQLSI-RKKMLMLG------AICFISMLATFAIFYYNNLQGSQKIAQT 41

RM5611_Tlp16 ------------MQLSI-RKKMLMLG------AICFISMLATFAIFYYNNLQGSQKIAQT 41

00-2425_Tlp11 ---------MNFRSLNL-STKLILSVAIGIVLGIVVIVLTVSIYTSKSMEKEAKDSIFLS 50

00-6200_Tlp11 ---------MNFRSLNL-STKLILSVAIGIVLGIVVIVLTVSIYTSKSMEKEAKDSIFLS 50

YH001_Tlp11 ---------MNFRSLNL-STKLILSVAIGIVLGIVVIVLTVSIYTSKSMEKEAKDSIFLS 50

IA3902_Tlp11 ---------MNFRSLNL-STKLILSVAIGIVLGIVVIVLTVSIYTSKSMEKEAKDSIFLS 50

BCW_6290_Tlp11 ---------MNFRSLNL-STKLILSVAIGIVLGIVVIVLTVSIYTSKSMEKEAKDSIFLS 50

76339_Tlp18 ----------MFKSLNI-GSKLVLSVAVSVIAAIAILITILSFEVASYAEKEAKDTIFLS 49

4031_Tlp17 ---------MNFRSLNI-STKLILSVAIGVILGIIVLVSTVSIYISENMEKEAKDSIFLA 50

MTVDSCj13_Tlp13 ----------MFRLSSV-SSKLLLSVAISIIVAIALIIAIVSFQVASYSEKEARNTILLS 49

RM1875_Tlp13 ----------MFRLSSV-SSKLLLSVAISIIVAIALIIAIVSFQVASYSEKEAKNAILLS 49

CF2-75_Tlp13 ----------MFRLSSV-SSKLLLSVAISIIVAIALIIAIVSFQVASYSEKEAKNAILLS 49

15-537360_Tlp13 ----------MFRLSSV-SSKLLLSVAISVILATALMIAIVSFQVASYSEKEARNTILLS 49

CVMN29710_Tlp13 ----------MFRLSSV-SSKLLLSVAISVILATALMIAIVSFQVASYSEKEARNTILLS 49

FB1_Tlp13 ----------MFRLSSV-SSKLLLSVAISVILATALMIAIVSFQVASYSEKEARNTILLS 49

CFCAN032805_Tlp13 ----------MFRLSSV-SSKLLLSVAISVILATALMIAIVSFQVASYSEKEARNTILLS 49

BG2108_Tlp13 ----------MFRLSSV-SSKLLLSVAISVILATALMIAIVSFQVASYSEKEARNTILLS 49

YF2105_Tlp13 ----------MFRLSSV-SSKLLLSVAISVILATALMIAIVSFQVASYSEKEARNTILLS 49

YH503_Tlp13 ----------MFRLSSV-SSKLLLSVAISVILATALMIAIVSFQVASYSEKEARNTILLS 49

BFRCA9557_Tlp13 ----------MFRLSSV-SSKLLLSVAISVILATALMIAIVSFQVASYSEKEARNTILLS 49

YH502_Tlp13 ----------MFRLSSV-SSKLLLSVAISVILATALMIAIVSFQVASYSEKEARNTILLS 49

OR12_Tlp13 ----------MFRLSSV-SSKLLLSVAISVILAIALMIAIVSFQVASYSEKEAKDTILLS 49

00-1597_Tlp13 ----------MFRLSSV-SSKLLLSVAISVILATALMIAIVSFQVASYSEKEAKDTIFLS 49

14903A_Tlp13 ----------MFRLSSV-SSKLLLSVAISVILATALMIAIVSFQVASYSEKEAKDTIFLS 49

FJ3124_Tlp13 ----------MFRLSSV-SSKLLLSVAISVILATALMIAIVSFQVASYSEKEAKDTIFLS 49

R14_Tlp13 ----------MFRLSSV-SSKLLLSVAISVIVAIALMIAIVSFQVASYSEKEAKDTIFLS 49

MTVDSCj16_Tlp13 ----------MFRLSSV-SSKLLLSVAISVIVAIALMIAIVSFQVASYSEKEAKDTIFLS 49

14980A_Tlp13 ----------MFRLSSV-SSKLLLSVAISVIVATALMIAIVSFQVASYSEKEAKDTIFLS 49

FDAARGOS_295_Tlp1 SKEHAVQVQGIF----NEIIA----LSKTVSNTLTEMFRVASKENLDMDSITNIVTNTFD 101

14980A_Tlp1 TKEYATQTQGIF----GEMIA----LNKSISGTLTEMFRSTSKEDLDIDNITNIITNTFD 101

CJ677CC527_Tlp1 TKEYATQTQGIF----GEMIA----LNKSISGTLTEMFRSSSKENLDIDSITNIITNTFD 101

CJ677CC012_Tlp1 TKEYATQTQGIF----GEMIA----LNKSISGTLTEMFRSSSKENLDIDSITNIITNTFD 101

4031_Tlp1 TKEYATQTQGIF----GEMIA----LNKSISGTLTEMFRSTSKEDLDIDNITNIITNTFD 101

81116_Tlp1 TKEYATQTQGIF----GEMIA----LNKSISGTLTEMFRSTSKEDLDIDNITNIITNTFD 101

35925B2_Tlp1 TKEYATQTQGIF----GEMIA----LNKSISGTLTEMFRSTSKEDLDIDNITNIITNTFD 101

M1_Tlp1 TKEYATQTQGIF----GEMIA----LNKSISGTLTEMFRSTSKEDLDIDNITNIITNTFD 101

PT14_Tlp1 TKEYATQTQGIF----GEMIA----LNKSISGTLTEMFRSTSKEDLDIDNITNIITNTFD 101

81-176_Tlp1 TKEYATQTQGIF----GEMIA----LNKSISGTLTEMFRSTSKEDLDIDNITNIITNTFD 101

CVMN29710_Tlp1 TKEYATQTQGIF----GEMIA----LNKSISGTLTEMFRSTSKEDLDIDNITNIITNTFD 101

FB1_Tlp1 TKEYATQTQGIF----GEMIA----LNKSISGTLTEMFRSTSKEDLDIDNITNIITNTFD 101

BG2108_Tlp1 TKEYATQTQGIF----GEMIA----LNKSISGTLTEMFRSTSKEDLDIDNITNIITNTFD 101

YF2108_Tlp1 TKEYATQTQGIF----GEMIA----LNKSISGTLTEMFRSTSKEDLDIDNITNIITNTFD 101

YH501_Tlp1 TKEYATQTQGIF----GEMIA----LNKSISGTLTEMFRSTSKEDLDIDNITNIITNTFD 101

RM4661_Tlp1 TKEYATQTQGIF----GEMIA----LNKSISGTLTEMFRSTSKEDLDIDNITNIITNTFD 101

F38011_Tlp1 TKEYATQTQGIF----GEMIA----LNKSISGTLTEMFRSTSKEDLDIDNITNIITNTFD 101

T1-21_Tlp1 TKEYATQTQGIF----GEMIA----LNKSISGTLTEMFRSTSKEDLDIDNITNIITNTFD 101

CG8421_Tlp1 TKEYATQTQGIF----GEMIA----LNKSISGTLTEMFRSTSKEDLDIDNITNIITNTFD 101

CJM1cam_Tlp1 TKEYATQTQGIF----GEMIA----LNKSISGTLTEMFRSTSKEDLDIDNITNIITNTFD 101

R14_Tlp1 TKEYATQTQGIF----GEMIA----LNKSISGTLTEMFRSTSKEDLDIDNITNIITNTFD 101

ICDCCJ07001_Tlp1 TKEYATQTQGIF----GEMIA----LNKSISGTLTEMFRSTSKEDLDIDNITNIITNTFD 101

RM3196_Tlp1 TKEYATQTQGIF----GEMIA----LNKSISGTLTEMFRSTSKEDLDIDNITNIITNTFD 101

NCTC11168_Tlp1 TKEYATQTQGIF----GEMIA----LNKSISGTLTEMFRSTSKEDLDIDNITNIITNTFD 101

00-2425_Tlp1 TKEYATQTQGIF----GEMIA----LNKSISGTLTEMFRSTSKEDLDIDNITNIITNTFD 101

IA3902_Tlp1 TKEYATQTQGIF----GEMIA----LNKSISGTLTEMFRSTSKEDLDIDNITNIITNTFD 101

RM1285_Tlp1 TKEYATQTQGIF----GEMIA----LNKSISGTLTEMFRSTSKEDLDIDNITNIITNTFD 101

00-0949_Tlp1 TKEYATQTQGIF----GEMIA----LNKSISGTLTEMFRSTSKEDLDIDNITNIITNTFD 101

01-1512_Tlp1 TKEYATQTQGIF----GEMIA----LNKSISGTLTEMFRSTSKEDLDIDNITNIITNTFD 101

FDAARGOS_422_Tlp1 TKEYATQTQGIF----GEMIA----LNKSISGTLTEMFRSTSKEDLDIDNITNIITNTFD 101

FORC_056_Tlp1 TKEYATQTQGIF----GEMIA----LNKSISGTLTEMFRSTSKEDLDIDNITNIITNTFD 101

32488_Tlp1 TKEYATQTQGIF----GEMIA----LNKSISGTLTEMFRSTSKEDLDIDNITNIITNTFD 101

CFSAN032806_Tlp1 TKEYATQTQGIF----GEMIA----LNKSISGTLTEMFRSTSKEDLDIDNITNIITNTFD 101

YH001_Tlp1 TKEYATQTQGIF----GEMIA----LNKSISGTLTEMFRSTSKEDLDIDNITNIITNTFD 101

00-6200_Tlp1 TKEYATQTQGIF----GEMIA----LNKSISGTLTEMFRSTSKEDLDIDNITNIITNTFD 101

RM1221_Tlp1 TKEYATQTQGIF----GEMIA----LNKSISGTLTEMFRSTSKEDLDIDNITNIITNTFD 101

S3_Tlp1 TKEYATQTQGIF----GEMIA----LNKSISGTLTEMFRSTSKEDLDIDNITNIITNTFD 101

FDAARGOS_421_Tlp1 TKEYATQTQGIF----GEMIA----LNKSISGTLTEMFRSTSKEDLDIDNITNIITNTFD 101

FJ3124_Tlp1 TKEYATQTQGIF----GEMIA----LNKSISGTLTEMFRSTSKEDLDIDNITNIITNTFD 101

00-1597_Tlp1 TKEYATQTQGIF----GEMIA----LNKSISGTLTEMFRSTSKEDLDIDNITNIITNTFD 101

15-537360_Tlp20 ESNYVRTAKNSMEAFKARNTAALESLAKNILKLPYEQISNQEAL---MRYVGKDLKVFRD 99

76339_Tlp20 ESNYVRTAKNSMEAFKARNTAALESLAKNILKLPYEQISNQEAL---MRYVGKDLKVFRD 99

HC2-48_Tlp20 ESNYVRTAKNSMEAFKARNTAALESLAKNILKLPYEQISNQEAL---MRYVGKDLKVFRD 99

CFSAN032805_Tlp20 ESNYVRTTKNSMEAFKARNTAALESLAKNILKLPYEQISNQEAL---MRYVGKDLKVFRD 99

CVMN29710_Tlp20 ESNYVRTTKNSMEAFKARNTAALESLAKNILKLPYEQISNQEAL---MRYVGKDLKVFRD 98

YH501_Tlp20 ESNYVRTTKNSMEAFKARNTAALESLAKNILKLPYEQISNQEAL---MRYVGKDLKVFRD 102

CF2-75_Tlp20 ESNYVRTAKNSMEAFKARNTAALESLAKNILKLPYEQISNQEAL---MRYVGKDLKVFRD 102

CO2-160_Tlp20 ESNYVRTAKNSMEAFKARNTAALESLAKNILKLPYEQISNQEAL---MRYVGKDLKVFRD 99

CO2-160_Tlp20b ESNYVRTAKNSMEAFKARNTAALESLAKNILKLPYEQISNQEAL---MRYVGKDLKVFRD 99

RM5611_Tlp20 ESNYVRTAKNSMEAFKARNTAALESLAKNILKLPYEQISNQEAL---MRYVGKDLKVFRD 99

14903A_Tlp20 ESNYVRTAKNSMEAFKARNTAALESLAKNILKLPYEQISNQEAL---MRYVGKDLKVFRD 102

YH502_Tlp20 ESNYVRTAKNSMEAFKARNTAALESLAKNILKLPYEQISNQEAL---MRYVGKDLKVFRD 99

RM3196_Tlp23 EINYVKTAKNSIESFKARNSLALESLAKSILKHPVEQLDNQDAL---MHYVGKDLKNFRD 99

ICDCCJ07001_Tlp23 EINYVKTAKNSIESFKARNSLALESLAKSILKHPVEQLDNQDAL---MHYVGKDLKNFRD 99

RM1285_Tlp2 EINYVKTAKNSIESFKARNSLALESLAKSILKHPIEQLDSQDAL---MHYVGKDLKNFRD 99

CFSAN032806_Tlp2 EINYVKTAKNSIESFKARNSLALESLAKSILKHPIEQLDSQDAL---MHYVGKDLKNFRD 99

RM1221_Tlp2 EINYVKTAKNSIESFKARNSLALESLAKSILKHPIEQLDSQDAL---MHYVGKDLKNFRD 99

S3_Tlp2 EINYVKTAKNSIESFKARNSLALESLAKSILKHPIEQLDSQDAL---MHYVGKDLKNFRD 99

FDAARGOS_422_Tlp2 ETNYVKTAKNSMESFKARNSLALESLAKSILKHPVEQLDNQDAL---MHYVGKDLKNFRD 99

81-176_Tlp2 EINYVKTAKNSIESFKARNSLALESLAKSILKHPIEQLDSQDAL---MHYVGKDLKNFRD 99

F38011_Tlp2 EINYVKTAKNSIESFKARNSLALESLAKSILKHPIEQLDSQDAL---MHYVGKDLKNFRD 99

NCTC11168_Tlp2 EINYVKTAKNSIESFKARNSLALESLAKSILKHPIEQLDSQDAL---MHYVGKDLKNFRD 99

MTVDSCj07_Tlp2 EINYVKTAKNSIESFKARNSLALESLAKSILKHPIEQLDSQDAL---MHYVGKDLKNFRD 99

CJM1cam_Tlp24 ETNYVKTAKNSMESFKARNSLALESLAKSILKHPVEQLDSQDAL---MRYVGKDLKNFRD 99

M1_Tlp24 ETNYVKTAKNSMESFKARNSLALESLAKSILKHPVEQLDSQDAL---MRYVGKDLKNFRD 99

81116_Tlp2 ETNYVKTAKNSMESFKARNSLALESLAKSILKHPVEQLDSQDAL---MRYVGKDLKNFRD 99

4031_Tlp23 ETNYVKTAKNSMESFKARNSLALESLAKSILKHPVEQLDSQDAL---MRYVGKDLKNFRD 99

CVMN29710_Tlp14 ETEFLKATQVSIEDFRSRNISLLNALEKDILNLPYEALNSQDNI---VNNAGVILKYYRD 99

RM4661_Tlp14 ETEFLKATQVSIENFRSRNISLLNALEKDILNLPYEALNSQDNI---VNNAGAILKYYRN 99

MG1116_Tlp14 ETDLLKATQISIEDFRSRNISLLNTLEKDILNLPYEALNSQDNI---INNAGAILKYYRN 99

BG2108_Tlp14 ETDLLKATQISIEDFRSRNISLLNTLEKDILNLPYEALNSQDNI---INNAGAILKYYRN 99

YF2105_Tlp14 ETDLLKATQISIEDFRSRNISLLNTLEKDILNLPYEALNSQDNI---INNAGAILKYYRN 99

YH502_Tlp14 KTDLLKATQISIENFRSRNISLLNALEKDILNLPYEALNSQDNI---VNNVGAILKYYRN 110

WA333_Tlp14 ETDLLKATQISIEDFRSRNISLLNALEKDILNLPYEALNSQDNI---VNNVGAILKYYRN 99

BP3181_Tlp14 ETELLKATQISIEDFRSRNISLLNTLEKDILNLPYEALNSQDNI---INNAGAILKYYRN 99

ZV1224_Tlp14a ETELLKAAQISIEDFRSRNISLLNALEKDILNLPYEALNSQDNI---INNVGAILKYYRN 99

ZV1224_Tlp14b ETELLKAAQISIEDFRSRNISLLNALEKDILNLPYEALNSQDNI---INNVGAILKYYRN 99

YH503_Tlp14 KTELLKATQISIEDFRSRNISLLNTLEKDILNLPYEALNSQDNI---INNAGAILKYYRN 110

14903A_Tlp14 ETDLLKATQISIEDFRSRNISLLNTLEKDILNLPYEALNSQDNI---VNNVGAILKYYRN 99

OR12_Tlp14 ETDLLKATQISIEDFRSRNISLLNTLEKDILNLPYEALNSQDNI---VNNVGAILKYYRN 99

CFSAN032805_Tlp14 ETDLLKATQISIENFRSRNISLLNALEKDILNLPYEALNSQDNI---VNNVGAILKYYRN 110

BFR-CA-9557_Tlp14 ETDLLKATQISIENFRSRNISLLNALEKDILNLPYEALNSQDNI---VNNVGAILKYYRN 99

15-537360_Tlp14 ETDLLKATQISIENFRSRNISLLNALEKDILNLPYEALNSQDNI---VNNVGAILKYYRN 99

YH501_Tlp14 ETDLLKATQISIENFRSRNISLLNALEKDILNLPYEALNSQDNI---VNNVGAILKYYRN 99

T1-21_Tlp19b QTELLKATQISIEDFRSRNISLLNTLEKDILNLPYEALNSQDNI---INNAGAILKYYRN 99

CG8421_Tlp14 ETELLKATQISIEDFRSRNISLLNTLEKDILKLPYEALNSQDNI---VNNVGAILKYYRN 99

MTVDSCj16_Tlp14 ETELLKATQISIEDFRSRNISLLNTLEKDILKLPYEALNSQDNI---VNNVGAILKYYRN 99

01-1512_Tlp14 ETELLKATQISIEDFRSRNISLLNTLEKDILKLPYEALNSQDNI---VNNVGAILKYYRN 99

00-0949_Tlp14 ETELLKATQISIEDFRSRNISLLNTLEKDILKLPYEALNSQDNI---VNNVGAILKYYRN 99

MTVDSCj13_Tlp14 ETELLKATQISIEDFRSRNISLLNTLEKDILKLPYEALNSQDNI---VNNAGAILKYYRN 99

S3_Tlp14 ETELLKATQISIEDFRSRNISLLNTLEKDILKLPYEALNSQDNI---VNNVGAILKYYRN 99

PT14_Tlp14 ETELLKATQISIEDFRSRNISLLNTLEKDILKLPYEALNSQDNI---VNNVGAILKYYRN 99

14980A_Tlp14 ETELLKATQISIEDFRSRNISLLNTLEKDILKLPYEALNSQDNI---VNNVGAILKYYRN 115

FJ3124_Tlp14 ETELLKATQISIEDFRSRNISLLNTLEKDILKLPYEALNSQDNI---VNNVGAILKYYRN 99

00-1597_Tlp14 ETELLKATQISIEDFRSRNISLLNTLEKDILKLPYEALNSQDNI---VNNVGAILKYYRN 99

R14_Tlp14 ETELLKATQISIEDFRSRNISLLNTLEKDILKLPYEALNSQDNI---VNNVGAILKYYRN 99

CG8421_Tlp25 ------------------------------------------------------------ 0

HC2-48_Tlp3 QTDLLKVTQSTVEDFRSTNQSFTRALEKDIANLPYQSLITEENI---INNVGPILKYYHH 99

35925B2_Tlp3 QTDLLKVTQSTVEDFRSTNQSFTRALEKDITNLPYQSLITEENI---INNVGPILKYYRH 110

14980A_Tlp3 QTDLLKVTQSTVEDFRSTNQSFTRALEKDIANLPYQSLITEENI---INNVGPILEYYRH 110

00-1597_Tlp3b QTDLLKVTQSTVEDFRSTNQSFTRALEKDIANLPYQSLITEENI---INNVGPILKYYRH 99

ICDCCJ07001_Tlp3 QTDLLKVTQSTVEDFRSTNQSFTRALEKDIANLPYQSLITEENI---INNVGPILKYYRH 99

RM3196_Tlp3 QTDLLKVTQSTVEDFRSTNQSFTRALEKDIANLPYQSLITEENI---INNVGPILKYYRH 99

RM1875_Tlp3 QTDLLKVTQSTVEDFRSTNQSFIRALEKDIANLPYQSLITEENI---INNVGPILKYYRH 99

CF2-75_Tlp3 QTDLLKVTQSTVEDFRSTNQSFTRALEKDIANLPYQSLITEENI---INNVGPILKYYHH 110

RM5611_Tlp3 QTDLLKVTQSTVEDFRSTNQSFTRALEKDIANLPYQSLITEENI---INNVGPILKYYHH 99

MTVDSCj16_Tlp3 QTDLLKVTQSTVEDFRSTNQSFTRALEKDIANLPYQSLITEENI---INNVGPILKYYRH 99

01-1512_Tlp3 QTDLLKVTQSTVEDFRSTNQSFTRALEKDIANLPYQSLITEENI---INNVGPILKYYRH 99

MTVDSCj13_Tlp3 QTDLLKVTQSTVEDFRSTNQSFTRALEKDIANLPYQSLITEENI---INNVGPILKYYRH 99

32488_Tlp3a QTDLLKVTQSTVEDFRSTNQSFTRALEKDIANLPYQSLITEENI---INNVGPILKYYRH 99

81116_Tlp3 QTDLLKVTQSTVEDFRSTNQSFTRALEKDIANLPYQSLITEENI---INNVGPILKYYRH 99

32488_Tlp3b QTDLLKVTQSTVEDFRSTNQSFTRALEKDIANLPYQSLITEENI---INNVGPILKYYRH 99

FB1_Tlp3 QTDLLKVTQSTVEDFRSTNQSFTRALEKDIANLPYQSLITEENI---INNVGPILKYYRH 99

PT14_Tlp3 QTDLLKVTQSTVEDFRSTNQSFTRALEKDIANLPYQSLITEENI---INNVGPILKYYRH 99

00-6200_Tlp3a QTDLLKVTQSTVEDFRSTNQSFTRALEKDIANLPYQSLITEENI---INNVGPILKYYHH 99

RM1221_Tlp3 QTDLLKVTQSTVEDFRSTNQSFTRALEKDIANLPYQSLITEENI---INNVGPILKYYRH 99

S3_Tlp3 QTDLLKVTQSTVEDFRSTNQSFTRALEKDIANLPYQSLITEENI---INNVGPILKYYRH 99

FDAARGOS_421_Tlp3 QTDLLKVTQSTVEDFRSTNQSFTRALEKDIANLPYQSLITEENI---INNVGPILKYYRH 110

CFSAN032806_Tlp3 QTDLLKVTQSTVEDFRSTNQSFTRALEKDIANLPYQSLITEENI---INNVGPILKYYRH 110

IA3901_Tlp3b QTDLLKVTQSTVEDFRSTNQSFTRALEKDIANLPYQSLITEENI---INNVGPILKYYHH 99

00-6200_Tlp3b QTDLLKVTQSTVEDFRSTNQSFTRALEKDIANLPYQSLITEENI---INNVGPILKYYHH 99

BCW_6290_Tlp3b QTDLLKVTQSTVEDFRSTNQSFTRALEKDIANLPYQSLITEENI---INNVGPILKYYHH 99

00-2425_Tlp3a QTDLLKVTQSTVEDFRSTNQSFTRALEKDIANLPYQSLITEENI---INNVGPILKYYHH 99

00-2425_Tlp3b QTDLLKVTQSTVEDFRSTNQSFTRALEKDIANLPYQSLITEENI---INNVGPILKYYHH 99

YH001_Tlp3a QTDLLKVTQSTVEDFRSTNQSFTRALEKDIANLPYQSLITEENI---INNVGPILKYYHH 99

YH001_Tlp3b QTDLLKVTQSTVEDFRSTNQSFTRALEKDIANLPYQSLITEENI---INNVGPILKYYHH 99

00-0949_Tlp3 QTDLLKVTQSTVEDFRSTNQSFTRALEKDIANLPYQSLITEENI---INNVGPILKYYRH 99

NCTC11168_Tlp3 QTDLLKVTQSTVEDFRSTNQSFTRALEKDIANLPYQSLITEENI---INNVGPILKYYRH 110

F38011_Tlp3 QTDLLKVTQSTVEDFRSTNQSFTRALEKDIANLPYQSLITEENI---INNVGPILKYYRH 99

RM1285_Tlp3 QTDLLKVTQSTVEDFRSTNQSFTRALEKDIANLPYQSLITEENI---INNVGPILKYYRH 99

FDAARGOS_422_Tlp3 QTDLLKVTQSTVEDFRSTNQSFTRALEKDIANLPYQSLITEENI---INNVGPILKYYRH 110

MTVDSCj07_Tlp3 QTDLLKVTQSTVEDFRSTNQSFTRALEKDIANLPYQSLITEENI---INNVGPILKYYRH 99

IA3901_Tlp3a QTDLLKVTQSTVEDFRSTNQSFTRALEKDIANLPYQSLITEENI---INNVGPILKYYHH 99

BCW_6290_Tlp3a QTDLLKVTQSTVEDFRSTNQSFTRALEKDIANLPYQSLITEENI---INNVGPILKYYHH 99

T1-21_Tlp19a QTDLLKVTQSTVEDFRSTNQSFTRALEKDIANLPYQSLITEENI---INNVGPILKYYRH 99

CJ677CC012_Tlp3 QTDLLKVTQSTVEDFRSTNQSFTRALEKDIANLPYQSLITEENI---INNVGPILKYYRH 99

CJM1cam_Tlp3 QTDLLKVTQSTVEDFRSTNQSFTRALEKDITNLPYQSLITEENI---INNVGPILKYYRH 99

M1_Tlp3 QTDLLKVTQSTVEDFRSTNQSFTRALEKDITNLPYQSLITEENI---INNVGPILKYYRH 99

4031_Tlp3 QTDLLKVTQSTVEDFRSTNQSFTRALEKDITNLPYQSLITEENI---INNVGPILKYYRH 99

R14_Tlp3 QTDLLKVTQSTVEDFRSTNQSFTRALEKDIANLPYQSLITEENI---INNVGPILKYYRH 99

FDAARGOS_295_Tlp21 N--QMTVLKNTATAFENANANKELT----MQALAKDLAKNLN-NEKDIYTILADFKNLT- 94

FORC_046_Tlp4 T--QLKTMQDVDAFFKSYAMSKRNG----IQILANELTNRPDMSDEELINLIKVIKKVN- 102

FDAARGOS_422_Tlp4 T--QLKTMQDVDAFFKSYAMSKRNG----IQILANELTNRPDMSDEELINLIKVIKKVN- 102

ICDCCJ07001_Tlp4 T--QLKTMQDVGAFFESYGMSKRNG----IQILANELNKRPDMSDEELINLIKAFKEVN- 102

RM3196_Tlp4 T--QLKTMQDVGAFFESYGMSKRNG----IQILANELNKRPDMSDEELINLIKAFKEVN- 102

T1-21_Tlp4 T--QLKTMQDVGAFFESYGMSKRNG----IQILANELNKRPDMSDEELINLIKAFKEVN- 102

F38011_Tlp4 T--QLKTMQDVGAFFESYGMSKRHG----IQILANELNKRPDMSDEELINLIKAFKEVN- 102

HF5-4A-4_Tlp22 -------MTLICKAVLYYAMSKRNG----IQILANELTNRPDMSDEELINLIKVIKKVN- 48

00-0949_Tlp4 T--QLKTMQDVDAFFKSYAMSKRNG----IQILANELTNRPDMSDEELINLIKVIKKVN- 102

01-1512_Tlp4 T--QLKTMQDVDAFFKSYAMSKRNG----IQILANELTNRPDMSDEELINLIKVIKKVN- 102

81-176_Tlp4 T--QLKTMQDVDAFFKSYAMSKRNG----IQILANELTNRPDMSDEELINLIKVIKKVN- 102

32488_Tlp4 T--QLKTMQDVDAFFKSYAMSKRNG----IQILANELTNRPDMSDEELINLIKVIKKVN- 102

NCTC11168_Tlp4 T--QLKTMQDVDAFFKSYAMSKRNG----IQILANELTNRPDMSDEELINLIKVIKKVN- 102

CFSAN032806_Tlp4 T--QLKTMQDVDAFFKSYAMSKRNG----IQILANELTNRPDMSDEELINLIKVIKKVN- 102

81116_Tlp4 T--QLKTMQDVDAFFKSYAMSKRNG----IQILANELTNRPDMSDEELINLIKVIKKVN- 102

RM1285_Tlp12 N--QLKTMDDVKVTFENYSKSKQKA----IEVLAYESAK--KLEDENISLLLDSFKKAF- 100

PT14_Tlp12 N--QLKTMDDVKVTFENYSKSKQKA----IEVLAYESAK--KLEDENISLLLDSFKKAF- 100

MTVJDCj07_Tlp12 N--QLKTMDDVKVTFENYSKSKQKA----IEVLAYESAK--KLEDENISLLLDSFKKAF- 100

RM1221_Tlp12 N--QLKTMDDVKVTFENYSKSKQKA----IEVLAYESAK--KLEDENISLLLDSFKKAF- 100

FDAARGOS_421_Tlp12 N--QLKTMDDVKVTFENYSKSKQKA----IEVLAYESAK--KLEDENISLLLDSFKKAF- 100

35925B2_Tlp12 N--QLKTMNDVKVTFENYSKSKQKA----IEVLAYESAK--KLEDENISLLLDSFKKAF- 100

CJM1cam_Tlp12 N--QLKTMDDVKVTFENYSKSKQKA----IEVLAYESAK--KLEDENISLLLDSFKKAF- 100

M1_Tlp12 N--QLKTMDDVKVTFENYSKSKQKA----IEVLAYESAK--KLEDENISLLLDSFKKAF- 100

S3_Tlp12 N--QLKTMDDVKVTFENYSKSKQKA----IEVLAYESAK--KLEDENISLLLDSFKKAF- 100

00-1597_Tlp12 N--QLKTMDDVKVTFENYSKSKQKA----IEVLAYESAK--KLEDENISLLLDSFKKAF- 100

R14_Tlp12 N--QLKTMDDVKVTFENYSKSKQKA----IEVLAYESAK--KLEDENISLLLDSFKKAF- 100

RM1875_Tlp15 TKNLINKE--IN----VKVELLTKS----MAIALGDLIKNVHSEEEKVKIIATAIENFRF 91

YH503_Tlp16 TKNLINKE--ID----IKVELLTKS----MAIALGDLIKDVDDEKEKIKISLPQLKILDL 91

FB1_Tlp16 TKNLINKE--ID----IKVELLTKS----MAIALGDLIKDVDDEKEKIKISLPQLKILDL 91

BFR-CA-9557_Tlp16 TKNLINKE--ID----IKVELLTKS----MAIALGDLIKDVDDEKEKIKISLPQLKILDL 91

15-537360_Tlp16 TKNLINKE--ID----IKVELLTKS----MAIALGDLIKDVDDEKEKIKISLPQLKILDL 91

OR12_Tlp16 TKNLINKE--ID----IKVELLTKS----MAIALGDLIKDVDDEKEKIKISLPQLKILDL 91

YH502_Tlp16 TKNLINKE--ID----IKVELLTKS----MAIALGDLIKDVDDEKEKIKISLPQLKILDL 91

14903A_Tlp16 TKNLINKE--ID----IKVELLTKS----MAIALGDLIKDVDDEKEKIKIIATAIENFRF 91

HC2-48_Tlp16 TKNLINKE--IN----IKVELLTKS----MAIALGDLIKNVHSEEEKVKIIATAIENFRF 91

RM5611_Tlp16 TKNLINKE--IN----IKVELLTKS----MAIALGDLIKNVHSEEEKVKIIATAIENFRF 91

00-2425_Tlp11 SKRYVNYMEGIL----NEEVVLTKA----MATSLNEIFSKNDQVN--AGIIESLLRNTFD 100

00-6200_Tlp11 SKRYVNYMEGIL----NEEVVLTKA----MATSLNEIFSKNDQVN--AGIIESLLRNTFD 100

YH001_Tlp11 SKRYVNYMEGIL----NEEVVLTKA----MATSLNEIFSKNDQVN--AGIIESLLRNTFD 100

IA3902_Tlp11 SKRYVNYMEGIL----NEEVVLTKA----MATSLNEIFSKNDQVN--AGIIESLLRNTFD 100

BCW_6290_Tlp11 SKRYVNYMEGIL----NEEVVLTKA----MATSLNEIFSKNDQVN--AGIIESLLRNTFD 100

76339_Tlp18 SKRYANYMEGVL----NESVVLTKG----ISASINEMFSKHDQVG--ADLIESLLKNTFD 99

4031_Tlp17 SKRYTNYMEGIL----NETVALTKG----TATSLNDMFEHNNQVD--ADLIESLMKNLFD 100

MTVDSCj13_Tlp13 SKRYVNYIQGIL----NEEVTLTKV----VATSLNEMFQNNDHVD--INLIESLIKNAFD 99

RM1875_Tlp13 SKRYVNYIQGIL----NEEVTLTKV----VATSLNEMFQNNDHVD--IDLIESLIKNAFD 99

CF2-75_Tlp13 SKRYVNYIQGIL----NEEVTLTKV----VATSLNEMFQNNDHVD--INLIESLIKNAFD 99

15-537360_Tlp13 SKRYVNYIQGML----NEEVTLTKG----VATSLNEMFQNNDHID--IDLIESLIKNTFD 99

CVMN29710_Tlp13 SKRYVNYIQGML----NEEVTLTKG----VATSLNEMFQNNDHID--IDLIESLIKNTFD 99

FB1_Tlp13 SKRYVNYIQGML----NEEVTLTKG----VATSLNEMFQNNDHID--IDLIESLIKNTFD 99

CFCAN032805_Tlp13 SKRYVNYIQGML----NEEVTLTKG----VATSLNEMFQNNDHID--IDLIESLIKNTFD 99

BG2108_Tlp13 SKRYVNYIQGML----NEEVTLTKG----VATSLNEMFQNNDHID--IDLIESLIKNTFD 99

YF2105_Tlp13 SKRYVNYIQGML----NEEVTLTKG----VATSLNEMFQNNDHID--IDLIESLIKNTFD 99

YH503_Tlp13 SKRYVNYIQGML----NEEVTLTKG----VATSLNEMFQNNDHID--IDLIESLIKNTFD 99

BFRCA9557_Tlp13 SKRYVNYIQGML----NEEVTLTKG----VATSLNEMFQNNDHID--IDLIESLIKNTFD 99

YH502_Tlp13 SKRYVNYIQGML----NEEVTLTKG----VATSLNEMFQNNDHID--IDLIESLIKNTFD 99

OR12_Tlp13 SKRYVNYIQGML----NEEVTLTKG----VATSLNEMFQNNDHID--IDLIESLIKNTFD 99

00-1597_Tlp13 SKRYVNYIQGML----NEEVTLTKG----VATSLNEMFQNNDHID--IDLIESLIKNTFD 99

14903A_Tlp13 SKRYVNYIQGML----NEEVTLTKG----VATSLNEMFQNNDHID--IDLIESLIKNTFD 99

FJ3124_Tlp13 SKRYVNYIQGML----NEEVTLTKG----VATSLNEMFQNNDHID--IDLIESLIKNTFD 99

R14_Tlp13 SKRYVNYIQGIL----NEEVTLTKG----VATSLNEMFQNNDHVD--IDLIESLIKNTFD 99

MTVDSCj16_Tlp13 SKRYVNYIQGIL----NEEVTLTKG----VATSLNEMFQNNDHVD--IDLIESLIKNTFD 99

14980A_Tlp13 SKRYVNYIQGIL----NEEVTLTKG----VATSLNEMFQNNDHVD--IDLIESLIKNTFD 99

FDAARGOS_295_Tlp1 NSVYSNFTYLYLIDPPEYFK---EKSKFFNTQNGKFVMLYVDEETDNKGGIKAIQASDEI 158

14980A_Tlp1 NSAYSNFTYLYLIDPPEYFK---EESKFFNTQSGKFVMLYADEEKDNKGGIKAIQASDEI 158

CJ677CC527_Tlp1 NSAYSNFTYLYLIDPPEYFK---EESKFFNTQSGKFVMLYVDEEKDSKGGIKAIQASDEI 158

CJ677CC012_Tlp1 NSAYSNFTYLYLIDPPEYFK---EESKFFNTQSGKFVMLYVDEEKDGKGGIKAIQASDEI 158

4031_Tlp1 NSAYSNFTYLYLIDPPEYFK---EESKFFNTQSGKFVMLYADEEKDNKGGIKAIQASDEI 158

81116_Tlp1 NSAYSNFTYLYLIDPPEYFK---EESKFFNTQSGKFVMLYADEEKDNKGGIKAIQASDEI 158

35925B2_Tlp1 NSAYSNFTYLYLIDPPEYFK---EESKFFNTQSGKFVMLYADEEKDNKGGIKAIQASDEI 158

M1_Tlp1 NSAYSNFTYLYLIDPPEYFK---EESKFFNTQSGKFVMLYADEEKDNKGGIKAIQASDEI 158

PT14_Tlp1 NSAYSNFTYLYLIDPPEYFK---EESKFFNTQSGKFVMLYADEEKDNKGGIKAIQASDEI 158

81-176_Tlp1 NSAYSNFTYLYLIDPPEYFK---EESKFFNTQSGKFVMLYADEEKDNKGGIKAIQASDEI 158

CVMN29710_Tlp1 NSAYSNFTYLYLIDPPEYFK---EESKFFNTQSGKFVMLYADEEKDNKGGIKAIQASDEI 158

FB1_Tlp1 NSAYSNFTYLYLIDPPEYFK---EESKFFNTQSGKFVMLYADEEKDNKGGIKAIQASDEI 158

BG2108_Tlp1 NSAYSNFTYLYLIDPPEYFK---EESKFFNTQSGKFVMLYADEEKDNKGGIKAIQASDEI 158

YF2108_Tlp1 NSAYSNFTYLYLIDPPEYFK---EESKFFNTQSGKFVMLYADEEKDNKGGIKAIQASDEI 158

YH501_Tlp1 NSAYSNFTYLYLIDPPEYFK---EESKFFNTQSGKFVMLYADEEKDNKGGIKAIQASDEI 158

RM4661_Tlp1 NSAYSNFTYLYLIDPPEYFK---EESKFFNTQSGKFVMLYADEEKDNKGGIKAIQASDEI 158

F38011_Tlp1 NSVYSNFTYLYLIDPPEYFK---EESKFFNTQSGKFVMLYADEEKDNKGGIKAIQASDEI 158

T1-21_Tlp1 NSVYSNFTYLYLIDPPEYFK---EESKFFNTQSGKFVMLYADEEKDNKGGIKAIQASDEI 158

CG8421_Tlp1 NSAYSNFTYLYLIDPPEYFK---EESKFFNTQSGKFVMLYADEEKDNKGGIKAIQASDEI 158

CJM1cam_Tlp1 NSAYSNFTYLYLIDPPEYFK---EESKFFNTQSGKFVMLYADEEKDNKGGIKAIQASDEI 158

R14_Tlp1 NSAYSNFTYLYLIDPPEYFK---EESKFFNTQSGKFVMLYADEEKDNKGGIKAIQASDEI 158

ICDCCJ07001_Tlp1 NSAYSNFTYLYLIDPPEYFK---EESKFFNTQSGKFVMLYADEEKDNKGGIKAIQASDEI 158

RM3196_Tlp1 NSAYSNFTYLYLIDPPEYFK---EESKFFNTQSGKFVMLYADEEKDNKGGIKAIQASDEI 158

NCTC11168_Tlp1 NSAYSNFTYLYLIDPPEYFK---EESKFFNTQSGKFVMLYADEEKDNKGGIKAIQASDEI 158

00-2425_Tlp1 NSAYSNFTYLYLIDPPEYFK---EESKFFNTQSGKFVMLYADEEKDNKGGIKAIQASDEI 158

IA3902_Tlp1 NSAYSNFTYLYLIDPPEYFK---EESKFFNTQSGKFVMLYADEEKDNKGGIKAIQASDEI 158

RM1285_Tlp1 NSAYSNFTYLYLIDPPEYFK---EESKFFNTQSGKFVMLYADEEKDNKGGIKAIQASDEI 158

00-0949_Tlp1 NSAYSNFTYLYLIDPPEYFK---EESKFFNTQSGKFVMLYADEEKDNKGGIKAIQASDEI 158

01-1512_Tlp1 NSAYSNFTYLYLIDPPEYFK---EESKFFNTQSGKFVMLYADEEKDNKGGIKAIQASDEI 158

FDAARGOS_422_Tlp1 NSAYSNFTYLYLIDPPEYFK---EESKFFNTQSGKFVMLYADEEKDNKGGIKAIQASDEI 158

FORC_056_Tlp1 NSAYSNFTYLYLIDPPEYFK---EESKFFNTQSGKFVMLYADEEKDNKGGIKAIQASDEI 158

32488_Tlp1 NSAYSNFTYLYLIDPPEYFK---EESKFFNTQSGKFVMLYADEEKDNKGGIKAIQASDEI 158

CFSAN032806_Tlp1 NSAYSNFTYLYLIDPPEYFK---EESKFFNTQSGKFVMLYADEEKDNKGGIKAIQASDEI 158

YH001_Tlp1 NSAYSNFTYLYLIDPPEYFK---EESKFFNTQSGKFVMLYADEEKDNKGGIKAIQASDEI 158

00-6200_Tlp1 NSAYSNFTYLYLIDPPEYFK---EESKFFNTQSGKFVMLYADEEKDNKGGIKAIQASDEI 158

RM1221_Tlp1 NSAYSNFTYLYLIDPPEYFK---EESKFFNTQSGKFVMLYADEEKDNKGGIKAIQASDEI 158

S3_Tlp1 NSAYSNFTYLYLIDPPEYFK---EESKFFNTQSGKFVMLYADEEKDNKGGIKAIQASDEI 158

FDAARGOS_421_Tlp1 NSAYSNFTYLYLIDPPEYFK---EESKFFNTQSGKFVMLYADEEKDNKGGIKAIQASDEI 158

FJ3124_Tlp1 NSAYSNFTYLYLIDPPEYFK---EESKFFNTQSGKFVMLYADEEKDNKGGIKAIQASDEI 158

00-1597_Tlp1 NSAYSNFTYLYLIDPPEYFK---EESKFFNTQSGKFVMLYADEEKDNKGGIKAIQASDEI 158

15-537360_Tlp20 AGGFLAVYIAQPDGELVVTDPDSDEKG----------------------------LNFGI 131

76339_Tlp20 AGGFLAVYIAQPDGELVVTDPDSDEKG----------------------------LNFGI 131

HC2-48_Tlp20 AGGFLAVYIAQPDGELVVTNPDSDEKG----------------------------LNFGI 131

CFSAN032805_Tlp20 AGGFLAVYIAQPDGELVVTDPDSDEKG----------------------------LNF-I 130

CVMN29710_Tlp20 AGGFLAVYIAQPDGELVVTDPDSDEKG----------------------------LNF-I 129

YH501_Tlp20 AGGFLAVYIAQPDGELVVTDPDSDEKG----------------------------LNF-I 133

CF2-75_Tlp20 AGGFLAVYIAQPDGELVVTNPDSDEKG----------------------------LNFGI 134

CO2-160_Tlp20 AGGFLAVYIAQPDGELVVTNPDSDEKG----------------------------LNFGI 131

CO2-160_Tlp20b AGGFLAVYIAQPDGELVVTNPDSDEKG----------------------------LNFGI 131

RM5611_Tlp20 AGGFLAVYIAQPDGELVVTNPDSDEKG----------------------------LNFGI 131

14903A_Tlp20 AGGFLAVYIAQSDGELVVTDPDSDEKG----------------------------LNF-I 133

YH502_Tlp20 AGGFLAVYIAQSDGELVVTDPDSDEKG----------------------------LNF-I 130

RM3196_Tlp23 AGRFLAVYIAQPNGELVVSDPDSDAKN----------------------------LDFGT 131

ICDCCJ07001_Tlp23 AGRFLAVYIAQPNGELVVSDPDSDAKN----------------------------LDFGT 131

RM1285_Tlp2 AGRFLAVYIAQPNGELVVSDPDSDAKN----------------------------LDFGT 131

CFSAN032806_Tlp2 AGRFLAVYIAQPNGELVVSDPDSDAKN----------------------------LDFGT 131

RM1221_Tlp2 AGRFLAVYIAQPNGELVVSDPDSDAKN----------------------------LDFGT 131

S3_Tlp2 AGRFLAVYIAQPNGELVVSDPDSDAKN----------------------------LDFGT 131

FDAARGOS_422_Tlp2 AGRFLAVYIAQPNGELVVSDPDSDAKN----------------------------LDFGT 131

81-176_Tlp2 AGRFLAVYIAQPNGELVVSDPDSDAKN----------------------------LDFGT 131

F38011_Tlp2 AGRFLAVYIAQPNGELVVSDPDSDAKN----------------------------LDFGT 131

NCTC11168_Tlp2 AGRFLAVYIAQPNGELVVSDPDSDAKN----------------------------LDFGT 131

MTVDSCj07_Tlp2 AGRFLAVYIAQPNGELVVSDPDSDAKI----------------------------LDFGT 131

CJM1cam_Tlp24 AGRFLAVYIAQPNGELVVSDPDSDAKK----------------------------VDFGT 131

M1_Tlp24 AGRFLAVYIAQPNGELVVSDPDSDAKK----------------------------VDFGT 131

81116_Tlp2 AGRFLAVYIAQPNGELVVSDPDSDAKK----------------------------VDFGT 131

4031_Tlp23 AGRFLAVYIA-PNGELVVSDPDSDAKK----------------------------VDFGT 130

CVMN29710_Tlp14 SGNFLAVYIGLDNGENIVSNDLSEKKN----------------------------TNITI 131

RM4661_Tlp14 SGNFLAVYIGLDNGENIVSDDLSEKKN----------------------------TNITI 131

MG1116_Tlp14 SGNLLAVYIGLDNGENIVSDDLSEKKN----------------------------TNITI 131

BG2108_Tlp14 SGNLLAVYIGLDNGENIVSDDLSEKKN----------------------------TNITI 131

YF2105_Tlp14 SGNLLAVYIGLDNGENIVSDDLSEKKN----------------------------TNITI 131

YH502_Tlp14 SGNLLAVYIGLDNGENIVSDDLSEKKN----------------------------TNITI 142

WA333_Tlp14 SGNLLAVYIGLDNGENIVSDDLSEKKN----------------------------TNITI 131

BP3181_Tlp14 SGNLLAVYIGLDNGENIVSDDLSEKKN----------------------------TNITI 131

ZV1224_Tlp14a SGNLLAVYIGLDNGENIVSDDLSEKKN----------------------------TNITI 131

ZV1224_Tlp14b SGNLLAVYIGLDNGENIVSDDLSEKKN----------------------------TNITI 131

YH503_Tlp14 SGNLLAVYIGLDNGENIVSDDLSEKKN----------------------------TNITI 142

14903A_Tlp14 SGNLLAVYIGLDNGENIVSDDLSEKKN----------------------------TNITI 131

OR12_Tlp14 SGNLLAVYIGLDNGENIVSDDLSEKKN----------------------------TNITI 131

CFSAN032805_Tlp14 SGNLLAVYIGLDNGENIVSDDLSEKKN----------------------------TNITI 142

BFR-CA-9557_Tlp14 SGNVLAVYIGLDNGENIVSDDLSEKKN----------------------------TNITI 131

15-537360_Tlp14 SGNVLAVYIGLDNGENIVSDDLSEKKN----------------------------TNITI 131

YH501_Tlp14 SGNLLAVYIGLDNGENIVSDDLSEKKN----------------------------TNITI 131

T1-21_Tlp19b SGNLLAVYIGLDNGENIVSDDLSEKKN----------------------------TNITI 131

CG8421_Tlp14 SGNLLAVYIGLDNGENIMSSDLSEKKN----------------------------TNITI 131

MTVDSCj16_Tlp14 SGNLLAVYIGLDNGENIMSSDLSEKKN----------------------------TNITI 131

01-1512_Tlp14 SGNLLAVYIGLDNGENIMSSDLSEKKN----------------------------TNITI 131

00-0949_Tlp14 SGNLLAVYIGLDNGENIMSSDLSEKKN----------------------------TNITI 131

MTVDSCj13_Tlp14 SGNLLAVYIGLDNGENIMSSDLSEKKN----------------------------TNITI 131

S3_Tlp14 SGNLLAVYIGLDNGENIMSSDLSEKKN----------------------------TNITI 131

PT14_Tlp14 SGNLLAVYIGLDNGENIMSSDLSEKKN----------------------------TNITI 131

14980A_Tlp14 SGNLLAVYIGLDNGENIMSSDLSEKKN----------------------------TNITI 147

FJ3124_Tlp14 SGNLLAVYIGLDNGENIMSSDLSEKKN----------------------------TNITI 131

00-1597_Tlp14 SGNLLAVYIGLDNGENIMSSDLSEKKN----------------------------TNITI 131

R14_Tlp14 SGNLLAVYIGLDNGENIMSSDLSEKKN----------------------------TNITI 131

CG8421_Tlp25 ------------------------------------------------------------ 0

HC2-48_Tlp3 SINALNVYLGLNNGKVLLSQKSNDAKM--------------------------------- 126

35925B2_Tlp3 SINALNVYLGLNNGKVLLSQESNDAKM--------------------------------- 137

14980A_Tlp3 SINALNVYLGLNNGKVLLSQKSNDAKM--------------------------------- 137

00-1597_Tlp3b SINALNVYLGLNNGKVLLSQKSNDAKM--------------------------------- 126

ICDCCJ07001_Tlp3 SINALNVYLGLNNGKVLLSQKSNDAKM--------------------------------- 126

RM3196_Tlp3 SINALNVYLGLNNGKVLLSQKSNDAKM--------------------------------- 126

RM1875_Tlp3 SINALNVYLGLNNGKVLLSQKSNDAKM--------------------------------- 126

CF2-75_Tlp3 SINALNVYLGLNNGKVLLSQKSNDAKM--------------------------------- 137

RM5611_Tlp3 SINALNVYLGLNNGKVLLSQKSNDAKM--------------------------------- 126

MTVDSCj16_Tlp3 SINALNVYLGLNNGKVLLSQKSNDAKM--------------------------------- 126

01-1512_Tlp3 SINALNVYLGLNNGKVLLSQKSNDAKM--------------------------------- 126

MTVDSCj13_Tlp3 SINALNVYLGLNNGKVLLSQKSNDAKM--------------------------------- 126

32488_Tlp3a SINALNVYLGLNNGKVLLSQKSNDAKM--------------------------------- 126

81116_Tlp3 SINALNVYLGLNNGKVLLSQKSNDAKM--------------------------------- 126

32488_Tlp3b SINALNVYLGLNNGKVLLSQKSNDAKM--------------------------------- 126

FB1_Tlp3 SINALNVYLGLNNGKVLLSQKSNDAKM--------------------------------- 126

PT14_Tlp3 SINALNVYLGLNNGKVLLSQKSNDAKM--------------------------------- 126

00-6200_Tlp3a SINALNVYLGLNNGKVLLSQKSNDAKM--------------------------------- 126

RM1221_Tlp3 SINALNVYLGLNNGKVLLSQKSNDAKM--------------------------------- 126

S3_Tlp3 SINALNVYLGLNNGKVLLSQKSNDAKM--------------------------------- 126

FDAARGOS_421_Tlp3 SINALNVYLGLNNGKVLLSQKSNDAKM--------------------------------- 137

CFSAN032806_Tlp3 SINALNVYLGLNNGKVLLSQKSNDAKM--------------------------------- 137

IA3901_Tlp3b SINALNVYLGLNNGKVLLSQKSNDAKM--------------------------------- 126

00-6200_Tlp3b SINALNVYLGLNNGKVLLSQKSNDAKM--------------------------------- 126

BCW_6290_Tlp3b SINALNVYLGLNNGKVLLSQKSNDAKM--------------------------------- 126

00-2425_Tlp3a SINALNVYLGLNNGKVLLSQKSNDAKM--------------------------------- 126

00-2425_Tlp3b SINALNVYLGLNNGKVLLSQKSNDAKM--------------------------------- 126

YH001_Tlp3a SINALNVYLGLNNGKVLLSQKSNDAKM--------------------------------- 126

YH001_Tlp3b SINALNVYLGLNNGKVLLSQKSNDAKM--------------------------------- 126

00-0949_Tlp3 SINALNVYLGLNNGKVLLSQKSNDAKM--------------------------------- 126

NCTC11168_Tlp3 SINALNVYLGLNNGKVLLSQKSNDAKM--------------------------------- 137

F38011_Tlp3 SINALNVYLGLNNGKVLLSQKSNDAKM--------------------------------- 126

RM1285_Tlp3 SINALNVYLGLNNGKVLLSQKSNDAKM--------------------------------- 126

FDAARGOS_422_Tlp3 SINALNVYLGLNNGKVLLSQKSNDAKM--------------------------------- 137

MTVDSCj07_Tlp3 SINALNVYLGLNNGKVLLSQKSNDAKM--------------------------------- 126

IA3901_Tlp3a SINALNVYLGLNNGKVLLSQKSNDAKM--------------------------------- 126

BCW_6290_Tlp3a SINALNVYLGLNNGKVLLSQKSNDAKM--------------------------------- 126

T1-21_Tlp19a SINALNVYLGLNNGKVLLSQKSNDAKM--------------------------------- 126

CJ677CC012_Tlp3 SINALNVYLGLNNGKVLLSQKSNDAKM--------------------------------- 126

CJM1cam_Tlp3 SINALNVYLGLNNGKVLLSQKSNDAKM--------------------------------- 126

M1_Tlp3 SINALNVYLGLNNGKVLLSQKSNDAKM--------------------------------- 126

4031_Tlp3 SINALNVYLGLNNGKVLLSQKSNDAKM--------------------------------- 126

R14_Tlp3 SINALNVYLGLNNGKVLLSQKSNDAKM--------------------------------- 126

FDAARGOS_295_Tlp21 --LFDSAFFGYDK----------MGKTYLSSG--------------------------DY 116

FORC_046_Tlp4 --DYDLVYVGFDN----------TGKNYQSDD--------------------------QI 124

FDAARGOS_422_Tlp4 --DYDLVYVGFDN----------TGKNYQSDD--------------------------QI 124

ICDCCJ07001_Tlp4 --GYDLVYVGFDN----------TGKNYQSDD--------------------------QI 124

RM3196_Tlp4 --GYDLVYVGFDN----------TGKNYQSDD--------------------------QI 124

T1-21_Tlp4 --GYDLVYVGFDN----------TGKNYQSDD--------------------------QI 124

F38011_Tlp4 --DYDLVYVGFDN----------TGKNYQSDD--------------------------QI 124

HF5-4A-4_Tlp22 --DYDLVYVGFDN----------TGKNYQSDD--------------------------QI 70

00-0949_Tlp4 --DYDLVYVGFDN----------TGKNYQSDD--------------------------QI 124

01-1512_Tlp4 --DYDLVYVGFDN----------TGKNYQSDD--------------------------QI 124

81-176_Tlp4 --DYDLVYVGFDN----------TGKNYQSDD--------------------------QI 124

32488_Tlp4 --DYDLVYVGFDN----------TGKNYQSDD--------------------------QI 124

NCTC11168_Tlp4 --DYDLVYVGFDN----------TGKNYQSDD--------------------------QI 124

CFSAN032806_Tlp4 --DYDLVYVGFDN----------TGKNYQSDD--------------------------QI 124

81116_Tlp4 --DYDLVYVGFDN----------TGKNYQSDD--------------------------QI 124

RM1285_Tlp12 --DFDIVFIAFDK----------NNKMLLSNG--------------------------TI 122

PT14_Tlp12 --DFDIVFIAFDK----------NNKMLLSNG--------------------------TI 122

MTVJDCj07_Tlp12 --DFDIVFIAFDK----------NNKMLLSNG--------------------------TI 122

RM1221_Tlp12 --DFDIVFIAFDK----------NNKMLLSNG--------------------------TI 122

FDAARGOS_421_Tlp12 --DFDIVFIAFDK----------NNKMLLSNG--------------------------TI 122

35925B2_Tlp12 --DFDIVFIAFEK----------NNKMLLSNG--------------------------TI 122

CJM1cam_Tlp12 --DFDIVFIAFDK----------NNKMLLSNG--------------------------TI 122

M1_Tlp12 --DFDIVFIAFDK----------NNKMLLSNG--------------------------TI 122

S3_Tlp12 --DFDIVFIAFDK----------NNKMLLSNG--------------------------TI 122

00-1597_Tlp12 --DFDIVFIAFDK----------NNKMLLSNG--------------------------TI 122

R14_Tlp12 --DFDIVFIAFDK----------NNKMLLSNG--------------------------TI 122

RM1875_Tlp15 EEDKSGYFFVYQKTTVKAHP---VRKD--------------------------------- 115

YH503_Tlp16 KRIKSGYFFVYQKTTVKAHP---VRKD--------------------------------- 115

FB1_Tlp16 KRIKSGYFFVYQKTTVKAHP---VRKD--------------------------------- 115

BFR-CA-9557_Tlp16 KR-KSGYFFVYQKTTVKAHP---VRKD--------------------------------- 114

15-537360_Tlp16 KRIKSGYFFVYQKTTVKAHP---VRKD--------------------------------- 115

OR12_Tlp16 KRIKSGYFFVYQKTTVKAHP---VRKD--------------------------------- 115

YH502_Tlp16 KRIKSGYFFVYQKTTVKAHP---VRKD--------------------------------- 115

14903A_Tlp16 EEDQSGYFFVYQKTTVKAHP---VRKD--------------------------------- 115

HC2-48_Tlp16 EEDKSGYFFVYQKTTVKAHP---VRKD--------------------------------- 115

RM5611_Tlp16 EEDKSGYFFVYQKTTVKAHP---VRKD--------------------------------- 115

00-2425_Tlp11 SSGYAAYAFLYLQDSSILTHVESLDKNFKNSDGKSVTMIFFDETTGKAGGIKSIHAPSNF 160

00-6200_Tlp11 SSGYAAYAFLYLQDSSILTHVESLDKNFKNSDGKSVTMIFFDETTGKAGGIKSIHAPSNF 160

YH001_Tlp11 SSGYAAYAFLYLQDSSILTHVESLDKNFKNSDGKSVTMIFFDETTGKAGGIKSIHAPSNF 160

IA3902_Tlp11 SSGYAAYAFLYLQDSSILTHVESLDKNFKNSDGKSVTMIFFDETTGKAGGIKSIHAPSNF 160

BCW_6290_Tlp11 SSGYAAYAFLYLQDSSILTHVESLDKNFKNSDGKSVTMIFFDETTGKAGGIKSIHAPSNF 160

76339_Tlp18 SSGYAAYAFLYLKDPSVLSDTYNMDKKYKSQNGNTFAMIFFDETTGKSGGIKAIQTPDNF 159

4031_Tlp17 SSLYSAYTFLYLKDTSVLGDAQGIDKRYTSSDGKTFAMIYFDQTTGKSGGIETIQTPNNF 160

MTVDSCj13_Tlp13 SSHYAAYTFLYLKDTTVLSDMQNVDKKYISPDGKTFSMIFFDQIAEKSGGITTISTPNNF 159

RM1875_Tlp13 SSHYAAYTFLYLKDTTVLSDMQNVDKKYISPDGKTFSMIFFDQIAEKSGGITTISTPNNF 159

CF2-75_Tlp13 SSHYAAYTFLYLKDTTVLSDMQNVDKKYISPDGKTFSMIFFDQIAEKSGGITTISTPNNF 159

15-537360_Tlp13 SSHYAAYTFLYLKDTTVLSDMQNVDKKYISPDGKTFSMIFFDQIVEKSGGITTISTPNNF 159

CVMN29710_Tlp13 SSHYAAYTFLYLKDTTVLSDMQNVDKKYISPDGKTFSMIFFDQIVEKSGGITTISTPNNF 159

FB1_Tlp13 SSHYAAYTFLYLKDTTVLSDMQNVDKKYISPDGKTFSMIFFDQIVEKSGGITTISTPNNF 159

CFCAN032805_Tlp13 SSHYAAYTFLYLKDTTVLSDMQNVDKKYISPDGKTFSMIFFDQIVEKSGGITTISTPNNF 159

BG2108_Tlp13 SSHYAAYTFLYLKDTTVLSDMQNVDKKYISPDGKTFSMIFFDQIVEKSGGITTISTPNNF 159

YF2105_Tlp13 SSHYAAYTFLYLKDTTVLSDMQNVDKKYISPDGKTFSMIFFDQIVEKSGGITTISTPNNF 159

YH503_Tlp13 SSHYAAYTFLYLKDTTVLSDMQNVDKKYISPDGKTFSMIFFDQIVEKSGGITTISTPNNF 159

BFRCA9557_Tlp13 SSHYAAYTFLYLKDTTVLSDMQNVDKKYISPDGKTFSMIFFDQIVEKSGGITTISTPNNF 159

YH502_Tlp13 SSHYAAYTFLYLKDTTVLSDMQNVDKKYISPDGKTFSMIFFDQIVEKSGGITTISTPNNF 159

OR12_Tlp13 SSHYAAYTFLYLKDTTVLSDMQNVDKKYISPDGKTFSMIFFDQIAEKSGGITTISTPNNF 159

00-1597_Tlp13 SSHYAAYTFLYLKDTTVLSDMQNVDKKYISPDGKTFSMIFFDQIVEKSGGITTISTPNNF 159

14903A_Tlp13 SSHYAAYTFLYLKDTTVLSDMQNVDKKYISPDGKTFSMIFFDQIVEKSGGITTISTPNNF 159

FJ3124_Tlp13 SSHYAAYTFLYLKDTTVLSDMQNVDKKYISPDGKTFSMIFFDQIVEKSGGITTISTPNNF 159

R14_Tlp13 SSHYAAYTFLYLKDTTVLSDMQNVDKKYISPDGKTFSMIFFDQIAEKSGGITTISTPNNF 159

MTVDSCj16_Tlp13 SSHYAAYTFLYLKDTTVLSDMQNVDKKYISPDGKTFSMIFFDQIAEKSGGITTISTPNNF 159

14980A_Tlp13 SSHYAAYTFLYLKDTTVLSDMQNVDKKYISPDGKTFSMIFFDQIAEKSGGITTISTPNNF 159

FDAARGOS_295_Tlp1 VNLQVVQDILKKAKYGE----NKVYIGRPIRMNLEDQDFDAVNIAMPIFNRKNQVVGVVG 214

14980A_Tlp1 ANLQVVQDILKKAKYGE----NKVYIGRPIKMNLEGQDFDAVNLAMPIFDRKNQVVGVIG 214

CJ677CC527_Tlp1 ANLQVVQDILKKAKYGE----NKVYIGRPIRMNLEGQDFDAVNIAMPIFDRKNQVVGVIG 214

CJ677CC012_Tlp1 ANLQVVQDILKKAKYGE----NKVYIGRPIRMNLEGQDFDAVNIAMPIFDRKNQVVGVIG 214

4031_Tlp1 ANLQVVQDILKKAKYGE----NKVYIGRPIKMNLEGQDFDAVNVAMPIFDRKNQVVGVIG 214

81116_Tlp1 ANLQVVQDILKKAKYGE----NKVYIGRPIKMNLEGQDFDAVNVAMPIFDRKNQVVGVIG 214

35925B2_Tlp1 ANLQVVQDILKKAKYGE----NKVYIGRPIKMNLEGQDFDAVNVAMPIFDRKNQVVGVIG 214

M1_Tlp1 ANLQVVQDILKKAKYGE----NKVYIGRPIKMNLEGQDFDAVNVAMPIFDRKNQVVGVIG 214

PT14_Tlp1 ANLQVVQDILKKAKYGE----NKVYIGRPIKMNLEGQDFDAVNVAMPIFDRKNQVVGVIG 214

81-176_Tlp1 ANLQVVQDILKKAKYGE----NKVYIGRPIKMNLEGQDFDAVNVAMPIFDRKNQVVGVIG 214

CVMN29710_Tlp1 ANLQVVQDILKKAKYGE----NKVYIGRPIKMNLEGQDFNAVNVAMPIFDRKNQVVGVIG 214

FB1_Tlp1 ANLQVVQDILKKAKYGE----NKVYIGRPIKMNLEGQDFNAVNVAMPIFDRKNQVVGVIG 214

BG2108_Tlp1 ANLQVVQDILKKAKYGE----NKVYIGRPIKMNLEGQDFNAVNVAMPIFDRKNQVVGVIG 214

YF2108_Tlp1 ANLQVVQDILKKAKYGE----NKVYIGRPIKMNLEGQDFNAVNVAMPIFDRKNQVVGVIG 214

YH501_Tlp1 ANLQVVQDILKKAKYGE----NKVYIGRPIKMNLEGQDFNAVNVAMPIFDRKNQVVGVIG 214

RM4661_Tlp1 ANLQVVQDILKKAKYGE----NKVYIGRPIKMNLEGQDFNAVNVAMPIFDRKNQVVGVIG 214

F38011_Tlp1 ANLQVVQDILKKAKYGE----NKVYIGRPIKMNLEGQDFDAVNVAMPIFDRKNQVVGVIG 214

T1-21_Tlp1 ANLQVVQDILKKAKYGE----NKVYIGRPIKMNLEGQDFDAVNVAMPIFDRKNQVVGVIG 214

CG8421_Tlp1 ANLQVVQDILKKAKYGE----NKVYIGRPIKMNLEGQDFDAVNVAMPIFDRKNQVVGVIG 214

CJM1cam_Tlp1 ANLQVVQDILKKAKYGE----NKVYIGRPIKMNLEGQDFDAVNVAMPIFDRKNQVVGVIG 214

R14_Tlp1 ANLQVVQDILKKAKYGE----NKVYIGRPIKMNLEGQDFDAVNVAMPIFDRKNQVVGVIG 214

ICDCCJ07001_Tlp1 ANLQVVQDILKKAKYGE----NKVYIGRPIKMNLEGQDFDAVNVAMPIFDRKNQVVGVIG 214

RM3196_Tlp1 ANLQVVQDILKKAKYGE----NKVYIGRPIKMNLEGQDFDAVNVAMPIFDRKNQVVGVIG 214

NCTC11168_Tlp1 ANLQVVQDILKKAKYGE----NKVYIGRPIKMNLEGQDFDAVNVAIPIFDRKNQVVGVIG 214

00-2425_Tlp1 ANLQVVQDILKKAKYGE----NKVYIGRPIKMNLEGQDFDAVNVAIPIFDRKNQVVGVIG 214

IA3902_Tlp1 ANLQVVQDILKKAKYGE----NKVYIGRPIKMNLEGQDFDAVNVAIPIFDRKNQVVGVIG 214

RM1285_Tlp1 ANLQVVQDILKKAKYGE----NKVYIGRPIKMNLEGQDFDAVNVAIPIFDRKNQVVGVIG 214

00-0949_Tlp1 ANLQVVQDILKKAKYGE----NKVYIGRPIKMNLEGQDFDAVNVAIPIFDRKNQVVGVIG 214

01-1512_Tlp1 ANLQVVQDILKKAKYGE----NKVYIGRPIKMNLEGQDFDAVNVAIPIFDRKNQVVGVIG 214

FDAARGOS_422_Tlp1 ANLQVVQDILKKAKYGE----NKVYIGRPIKMNLEGQDFDAVNVAIPIFDRKNQVVGVIG 214

FORC_056_Tlp1 ANLQVVQDILKKAKYGE----NKVYIGRPIKMNLEGQDFDAVNVAMPIFDRKNQVVGVIG 214

32488_Tlp1 ANLQVVQDILKKAKYGE----NKVYIGHPIKMNLEGQDFDAVNVAMPIFDRKNQVVGVIG 214

CFSAN032806_Tlp1 ANLQVVQDILKKAKYGE----NKVYIGRPIKMNLEGQDFDAVNVAMPIFDRKNQVVGVIG 214

YH001_Tlp1 ANLQVVQDILKKAKYGE----NKVYIGRPIKMNLEGQDFDAVNVAMPIFDRKNQVVGVIG 214

00-6200_Tlp1 ANLQVVQDILKKAKYGE----NKVYIGRPIKMNLEGQDFDAVNVAMPIFDRKNQVVGVIG 214

RM1221_Tlp1 ANLQVVQDILKKAKYGE----NKVYIGRPIKMNLEGQDFDAVNVAMPIFDRKNQVVGVIG 214

S3_Tlp1 ANLQVVQDILKKAKYGE----NKVYIGRPIKMNLEGQDFDAVNVAMPIFDRKNQVVGVIG 214

FDAARGOS_421_Tlp1 ANLQVVQDILKKAKYGE----NKVYIGRPIKMNLEGQDFDAVNVAMPIFDRKNQVVGVIG 214

FJ3124_Tlp1 ANLQVVQDILKKAKYGE----NKVYIGRPIKMNLEGQDFDAVNVAMPIFDRKNQVVGVIG 214

00-1597_Tlp1 ANLQVVQDILKKAKYGE----NKVYIGRPIKMNLEGQDFDAVNVAMPIFDRKNQVVGVIG 214

15-537360_Tlp20 YGKADNYDARTRDYFKGAVKANGLYVTPSYLDLTT--NLPCFTYATPLYK-EGKFIGVLA 188

76339_Tlp20 YGKADNYDARTRDYFKGAVKANGLYVTPSYLDLTT--NLPCFTYAIPLYK-EGKFIGVLA 188

HC2-48_Tlp20 YGKADNYDARTRDYFKGAVKANGLYVTPSYLDLTT--NLPCFTYATPLYK-EGKFIGVLA 188

CFSAN032805_Tlp20 YGKADNYDARTRDYFKGAVKANGLYVTPSYLDLTT--NLPCFTYATPLYK-EGKFIGVLA 187

CVMN29710_Tlp20 YGKADNYDARTRDYFKGAVKANGLYVTPSYLDLTT--NLPCFTYATPLYK-EGKFIGVLA 186

YH501_Tlp20 YGKADNYDARTRDYFKGAVKANGLYVTPSYLDLTT--NLPCFTYATPLYK-EGKFIGVLA 190

CF2-75_Tlp20 YGKADNYDARTRDYFKGAVKANGLYVTPSYLDLTT--NLPCFTYATPLYK-EGKFIGVLA 191

CO2-160_Tlp20 YGKADNYDARTRDYFKGAVKANGLYVTPSYLDLTT--NLPCFTYATPLYK-EGKFIGVLA 188

CO2-160_Tlp20b YGKADNYDARTRDYFKGAVKANGLYVTPSYLDLTT--NLPCFTYATPLYK-EGKFIGVLA 188

RM5611_Tlp20 YGKADNYDARTRDYFKGAVKANGLYVTPSYLDLTT--NLPCFTYATPLYK-EGKFIGVLA 188

14903A_Tlp20 YGKADNYDARTRDYFKGAVKANGLYVTPSYLDLTT--NLPCFTYATPLYK-EGKFIGVLA 190

YH502_Tlp20 YGKADNYDARTRDYFKGAVKANGLYVTPSYLDLTT--NLPCFTYATPLYK-EGKFIGVLA 187

RM3196_Tlp23 YGKADNYDARTREYYIEAVKTNKLYITPSYIDVTT--NLPCFTYSIPLYK-DGKFIGVLA 188

ICDCCJ07001_Tlp23 YGKADNYDARTREYYIEAVKTNKLYITPSYIDVTT--NLPCFTYSIPLYK-DGKFIGVLA 188

RM1285_Tlp2 YGKADNYDARTREYYIEAVKTNKLYITPSYIDVTT--NLPCFTYSIPLYK-DGKFIGVLA 188

CFSAN032806_Tlp2 YGKADNYDARTREYYIEAVKTNKLYITPSYIDVTT--NLPCFTYSIPLYK-DGKFIGVLA 188

RM1221_Tlp2 YGKADNYNARTREYYIEAVKTNKLYITPSYIDVTT--NLPCFTYSIPLYK-DGKFIGVLA 188

S3_Tlp2 YGKADNYDARTREYYIEAVKTNKLYITPSYIDVTT--NLPCFTYSIPLYK-DGKFIGVLA 188

FDAARGOS_422_Tlp2 YGKADNYDARTREYYIEAVKTNKLYITPSYIDVTT--NLPCFTYSIPLYK-DGKFIGVLA 188

81-176_Tlp2 YGKADNYDARTREYYIEAVKTNKLYITPSYIDVTT--NLPCFTYSIPLYK-DGKFIGVLA 188

F38011_Tlp2 YGKADNYDARTREYYIEAVKTNKLYITPSYIDVTT--NLPCFTYSIPLYK-DGKFIGVLA 188

NCTC11168_Tlp2 YGKADNYDARTREYYIEAVKTNKLYITPSYIDVTT--NLPCFTYSIPLYK-DGKFIGVLA 188

MTVDSCj07_Tlp2 YGKADNYDARTREYYIEAVKTNKLYITPSYIDVTT--NLPCFTYSIPLYK-DGKFIGVLA 188

CJM1cam_Tlp24 YGKADNYDARTREYYIEAVKTNKLYVTPSYIDATT--NLPCFTYSTPLYK-DGKFIGVLA 188

M1_Tlp24 YGKADNYDARTREYYIEAVKTNKLYVTPSYIDATT--NLPCFTYSTPLYK-DGKFIGVLA 188

81116_Tlp2 YGKADNYDARTREYYIEAVKTNKLYVTPSYIDATT--NLPCFTYSTPLYK-DGKFIGVLA 188

4031_Tlp23 YGKADNYDARTREYYIEAVKTNKLYVTPSYIDATT--NLPCFTYSTPLFK-DGKFIGVLA 187

CVMN29710_Tlp14 NGKANNYNATTREWYKEARNSNQIYIAPAYIDAVS--NEYTITYSKALYK-DGKFIGVLG 188

RM4661_Tlp14 NGKANNYNATTREWYKEARNSNQIYIVPAYIDTVS--NEYTITYSKALYK-DGKFIGVLG 188

MG1116_Tlp14 NGKANNYNATTREWYKEARNSNQIYITPAYIDVVS--NEYAITYSKALYK-DGKFIGVLG 188

BG2108_Tlp14 NGKANNYNATTREWYKEARNSNQIYITPAYIDVVS--NEYAITYSKALYK-DGKFIGVLG 188

YF2105_Tlp14 NGKANNYNATTREWYKEARNSNQIYITPAYIDVVS--NEYAITYSKALYK-DGKFIGVLG 188

YH502_Tlp14 NGKANNYNATTREWYKEARNSNQIYITPAYIDVVS--NEYAITYSKALYK-DGKFIGVLG 199

WA333_Tlp14 NGKANNYNATTREWYKEARNSNQTYITPAYIDVVS--NEYAITYSKALYK-DGKFIGVLG 188

BP3181_Tlp14 NGKANNYNATTREWYKEARNSNQMYITPAYIDVVS--NEYAITYSKALYK-DGKFIGVLG 188

ZV1224_Tlp14a NGKANNYNATTREWYKEARNSNQTYITPAYIDVVS--NEYAITYSKALYK-DGKFIGVLG 188

ZV1224_Tlp14b NGKANNYNATTREWYKEARNSNQTYITPAYIDVVS--NEYAITYSKALYK-DGKFIGVLG 188

YH503_Tlp14 NGKANNYNATTREWYKEARNSNQIYITPAYIDVVS--NEYAITYSKALYK-DGKFIGVLG 199

14903A_Tlp14 NGKANNYNATTREWYKEARNSNQTYITPAYIDVVS--NEYAITYSKALYK-DGKFIGVLG 188

OR12_Tlp14 NGKANNYNATTREWYKEARNSNQTYITPAYIDVVS--NEYAITYSKALYK-DGKFIGVLG 188

CFSAN032805_Tlp14 NGKANNYNATTREWYKEARNSNQTYITPAYIDVVS--NEYAITYSKALYK-DGKFIGVLG 199

BFR-CA-9557_Tlp14 NEKANNYNATTREWYKEARNSNQTYITPAYIDVVS--NEYAITYSKALYK-DGKFIGVLG 188

15-537360_Tlp14 NGKANNYNATTREWYKEARNSNQTYITPAYIDVVS--NEYAITYSKALYK-DGKFIGVLG 188

YH501_Tlp14 NGKANNYNATTREWYKEARNSNQTYITPAYIDVVS--NEYAITYSKALYK-DGKFIGVLG 188

T1-21_Tlp19b NGKANNYNATTREWYKEARNSNQMYITPAYIDVVS--NEYAITYSKALYK-DGKFIGVLG 188

CG8421_Tlp14 NGKANNYNATTREWYKEARNSNQIYITPAYIDAIS--NEYCITYSKALYK-DGKFIGVLG 188

MTVDSCj16_Tlp14 NGKANNYNATTREWYKEARNSNQINITPAYIDAIS--NEYCITYSKALYK-DGKFIGVLG 188

01-1512_Tlp14 NGKANNYNATTREWYKEARNSNQIYITPAYIDAIS--NEYCITYSKALYK-DGKFIGVLG 188

00-0949_Tlp14 NGKANNYNATTREWYKEARNSNQIYITPAYIDAIS--NEYCITYSKALYK-DGKFIGVLG 188

MTVDSCj13_Tlp14 NGKANNYNATTREWYKEARNSNQIYITPAYIDAIS--NEYCITYSKALYK-DGKFIGVLG 188

S3_Tlp14 NGKANNYNATTREWYKEARNSNQIYITPAYIDAIS--NEYCITYSKALYK-DGKFIGVLG 188

PT14_Tlp14 NGKANNYNATTREWYKEARNSNQIYITPAYIDAVS--NEYCITYSKALYK-DGKFIGVLG 188

14980A_Tlp14 NGKANNYNATTREWYKEARNSNQIYITPAYIDAIS--NEYCITYSKALYK-DGKFIGVLG 204

FJ3124_Tlp14 NGKANNYNATTREWYKEARNSNQIYITPAYIDAIS--NEYCITYSKALYK-DGKFIGVLG 188

00-1597_Tlp14 NGKANNYNATTREWYKEARNSNQIYITPAYIDVVS--NEYCITYSKALYK-DGKFIGVLG 188

R14_Tlp14 NGKANNYNATTREWYKEARNSNQIYITPAYIDAIS--NEYCITYSKALYK-DGKFIGVLG 188

CG8421_Tlp25 ---------------------------------------------MQSIN-SGKSVGVSV 14

HC2-48_Tlp3 PELRDDLDIKTKDWYQEALKTNDIFVTPAYLDTVL--KQYVITYSKAIYK-DGKIIGVLG 183

35925B2_Tlp3 PELRDDLDIKTKDWYQEALKTNDIFVTPAYLDTIL--KQYVITYSKAIYK-DGKIIGVLG 194

14980A_Tlp3 PELRDDLDIKTKDWYQEALKTNDIFVTPAYLDTIL--KQYVITYSKAIYK-DGKIIGVLG 194

00-1597_Tlp3b PELRDDLDIKTKDWYQEALKTNDIFVTPAYLDTVL--KQYVITYSKAIYK-DGKIIGVLG 183

ICDCCJ07001_Tlp3 PELRDDLDIKTKDWYQEALKTNDIFVTPAYLDTVL--KQYVITYSKAIYK-DGKIIGVLG 183

RM3196_Tlp3 PELRDDLDIKTKDWYQEALKTNDIFVTPAYLDTVL--KQYVITYSKAIYK-DGKIIGVLG 183

RM1875_Tlp3 PELRDDLDIKTKDWYQEALKTNDIFDTPAYLDTNL--KQYVITYSKAIYK-DGKIIGVLG 183

CF2-75_Tlp3 PELRDDLDIKTKDWYQEALKTNDIFVTPAYLDTVL--KQYVITYSKAIYK-DGKIIGVLG 194

RM5611_Tlp3 PELRDDLDIKTKDWYQEALKTNDIFVTPAYLDTVL--KQYVITYSKAIYK-DGKIIGVLG 183

MTVDSCj16_Tlp3 PELRDDLDIKTKDWYQEALKTNDIFVTPAYLDTIL--KQYVITYSKAIYK-DGKIIGVLG 183

01-1512_Tlp3 PELRDDLDIKTKDWYQEALKTNDIFVTPAYLDTVL--KQYVITYSKAIYK-DGKIIGVLG 183

MTVDSCj13_Tlp3 PELRDDLDIKTKDWYQEALKTNDIFVTPAYLDTIL--KQYVITYSKAIYK-DGKIIGVLG 183

32488_Tlp3a PELRDDLDIKTKDWYQEALKTNDIFVTPAYLDTIL--KQYVITYSKAIYK-DGKIIGVLG 183

81116_Tlp3 PELRDDLDIKTKDWYQEALKTNDIFVTPAYLDTIL--KQYVITYSKAIYK-DGKIIGVLG 183

32488_Tlp3b PELRDDLDIKTKDWYQEALKTNDIFVTPAYLDTIL--KQYVITYSKAIYK-DGKIIGVLG 183

FB1_Tlp3 PELRDDLDIKTKDWYQEALKTNDIFVTPAYLDTVL--KQYVITYSKAIYK-DGKIIGVLG 183

PT14_Tlp3 PELRDDLDIKTKDWYQEALKTNDIFVTPAYLDTVL--KQYVITYSKAIYK-DGKIIGVLG 183

00-6200_Tlp3a PELRDDLDIKTKDWYQEALKTNDIFVTPAYLDTVL--KQYVITYSKAIYK-DGKIIGVLG 183

RM1221_Tlp3 PELRDDLDIKTKDWYQEALKTNDIFVTPAYLDTIL--KQYVITYSKAIYK-DGKIIGVLG 183

S3_Tlp3 PELRDDLDIKTKDWYQEALKTNDIFVTPAYLDTIL--KQYVITYSKAIYK-DGKIIGVLG 183

FDAARGOS_421_Tlp3 PELRDDLDIKTKDWYQEALKTNDIFVTPAYLDTIL--KQYVITYSKAIYK-DGKIIGVLG 194

CFSAN032806_Tlp3 PELRDDLDIKTKDWYQEALKTNDIFVTPAYLDTVL--KQYVITYSKAIYK-DGKIIGVLG 194

IA3901_Tlp3b PELRDDLDIKTKDWYQEALKTNDIFVTPAYLDTVL--KQYVITYSKAIYK-DGKIIGVLG 183

00-6200_Tlp3b PELRDDLDIKTKDWYQEALKTNDIFVTPAYLDTVL--KQYVITYSKAIYK-DGKIIGVLG 183

BCW_6290_Tlp3b PELRDDLDIKTKDWYQEALKTNDIFVTPAYLDTVL--KQYVITYSKAIYK-DGKIIGVLG 183

00-2425_Tlp3a PELRDDLDIKTKDWYQEALKTNDIFVTPAYLDTVL--KQYVITYSKAIYK-DGKIIGVLG 183

00-2425_Tlp3b PELRDDLDIKTKDWYQEALKTNDIFVTPAYLDTVL--KQYVITYSKAIYK-DGKIIGVLG 183

YH001_Tlp3a PELRDDLDIKTKDWYQEALKTNDIFVTPAYLDTVL--KQYVITYSKAIYK-DGKIIGVLG 183

YH001_Tlp3b PELRDDLDIKTKDWYQEALKTNDIFVTPAYLDTVL--KQYVITYSKAIYK-DGKIIGVLG 183

00-0949_Tlp3 PELRDDLDIKTKDWYQEALKTNDIFVTPAYLDTVL--KQYVITYSKAIYK-DGKIIGVLG 183

NCTC11168_Tlp3 PELRDDLDIKTKDWYQEALKTNDIFVTPAYLDTVL--KQYVITYSKAIYK-DGKIIGVLG 194

F38011_Tlp3 PELRDDLDIKTKDWYQEALKTNDIFVTPAYLDTVL--KQYVITYSKAIYK-DGKIIGVLG 183

RM1285_Tlp3 PELRDDLDIKTKDWYQEALKTNDIFVTPAYLDTVL--KQYVITYSKAIYK-DGKIIGVLG 183

FDAARGOS_422_Tlp3 PELRDDLDIKTKDWYQEALKTNDIFVTPAYLDTVL--KQYVITYSKAIYK-DGKIIGVLG 194

MTVDSCj07_Tlp3 PELRDDLDIKTKDWYQEALKTNDIFVTPAYLDTVL--KQYVITYSKAIYK-DGKIIGVLG 183

IA3901_Tlp3a PELRDDLDIKTKDWYQEALKTNDIFVTPAYLDTVL--KQYVITYSKAIYK-DGKIIGVLG 183

BCW_6290_Tlp3a PELRDDLDIKTKDWYQEALKTNDIFVTPAYLDTVL--KQYVITYSKAIYK-DGKIIGVLG 183

T1-21_Tlp19a PELRDDLDIKTKDWYQEALKTNDIFVTPAYLDTIL--KQYVITYSKAIYK-DGKIIGVLG 183

CJ677CC012_Tlp3 PELRDDLDIKTKDWYQEALKTNDIFVTPAYLDTNL--KQYVITYSKAIYK-DGKMIGVLG 183

CJM1cam_Tlp3 PELRDDLDIKTKDWYQEALKTNDIFVTPAYLDTNL--KQYVITYSKAIYK-DGKIIGVLG 183

M1_Tlp3 PELRDDLDIKTKDWYQEALKTNDIFVTPAYLDTNL--KQYVITYSKAIYK-DGKIIGVLG 183

4031_Tlp3 PELRDDLDIKTKDWYQEALKTNDIFVTPAYLDTNL--KQYVITYSKAIYK-DGKIIGVLG 183

R14_Tlp3 PELRDDLDIKTKDWYQEALKTNDIFVTPAYLDTIL--KQYVITYSKAIYK-DGKIIGVLG 183

FDAARGOS_295_Tlp21 LDLSKNYDVTTRAWYKGAKENNGIVITPPYLSRST--GNIAIGYGIPVVV-EGKIVGVVG 173

FORC_046_Tlp4 LDLSKGYDTKNRPWYKAAKEAKKLIVTEPYKSAAS--GEVGLTYAAPFYDRNGNFRGVVG 182

FDAARGOS_422_Tlp4 LDLSKGYDTKNRPWYKAAKEAKKLIVTEPYKSAAS--GEVGLTYAAPFYDRNGNFRGVVG 182

ICDCCJ07001_Tlp4 LDLSKGYDTKNRPWYKAAKEAKKLIVTEPYKSANS--GEVGLTYAAPFYDRNGNFRGVVG 182

RM3196_Tlp4 LDLSKGYDTKNRPWYKAAKEAKKLIVTEPYKSANS--GEVGLTYAAPFYDRNGNFRGVVG 182

T1-21_Tlp4 LDLSKGYDTKNRPWYKAAKEAKKLIVTEPYKSANS--GEVGLTYAAPFYDRNGNFRGVVG 182

F38011_Tlp4 LDLSKGYDTKNRPWYKAAKEAKKLIVTEPYKSAAS--GEVGLTYAAPFYDRNGNFRGVVG 182

HF5-4A-4_Tlp22 LDLSKGYDTKNRPWYKAAKEAKKLIVTEPYKSAAS--GEVGLTYAAPFYDRNGNFRGVVG 128

00-0949_Tlp4 LDLSKGYDTKNRPWYKAAKEAKKLIVTEPYKSAAS--GEVGLTYAAPFYDRNGNFRGVVG 182

01-1512_Tlp4 LDLSKGYDTKNRPWYKAAKEAKKLIVTEPYKSAAS--GEVGLTYAAPFYDRNGNFRGVVG 182

81-176_Tlp4 LDLSKGYDTKNRPWYKAAKEAKKLIVTEPYKSAAS--GEVGLTYAAPFYDRNGNFRGVVG 182

32488_Tlp4 LDLSKGYDTKNRPWYKAAKEAKKLIVTEPYKSAAS--GEVGLTYAAPFYDRNGNFRGVVG 182

NCTC11168_Tlp4 LDLSKGYDTKNRPWYKAAKEAKKLIVTEPYKSAAS--GEVGLTYAAPFYDRNGNFRGVVG 182

CFSAN032806_Tlp4 LDLSKGYDTKNRPWYKAAKEAKKLIVTEPYKSAAS--GEVGLTYAAPFYDRNGNFRGVVG 182

81116_Tlp4 LDLSKGYDTKNRPWYKAAKEAKKLIVTEPYKSAAS--GEVGLTYAAPFYDRNGNFRGVVG 182

RM1285_Tlp12 LDKKSNFDITKQIWYQEAKNNKGITITQPYKSPID--QEIGITYVFPIYKNN-QLIAFVG 179

PT14_Tlp12 LDKKSNFDITKQIWYQEAKNNKGITITQPYKSPID--QEIGITYVFPIYKNN-QLIAFVG 179

MTVJDCj07_Tlp12 LDKKSNFDITKQIWYQEAKNNKGITITQPYKSPID--QEIGITYVFPIYKNN-QLIAFVG 179

RM1221_Tlp12 LDKKSNFDITKQIWYQEAKNNKGITITQPYKSPID--QEIGITYVFPIYKNN-QLIAFVG 179

FDAARGOS_421_Tlp12 LDKKSNFDITKQIWYQEAKNNKGITITQPYKSPID--QEIGITYVFPIYKNN-QLIAFVG 179

35925B2_Tlp12 LDKKSNFDITKQIWYQEAKNNKGITITQPYKSPID--QEIGITYVFPIYKNN-QLIAFVG 179

CJM1cam_Tlp12 LDKKSNFDITKQIWYQEAKNNKGITITQPYKSPID--QEIGITYVFPIYKNN-QLIAFVG 179

M1_Tlp12 LDKKSNFDITKQIWYQEAKNNKGITITQPYKSPID--QEIGITYVFPIYKNN-QLIAFVG 179

S3_Tlp12 LDKKSNFDITKQIWYQEAKNNKGITITQPYKSPID--QEIGITYVFPIYKNN-QLIAFVG 179

00-1597_Tlp12 LDKKSNFDITKQIWYQEAKNNKGITITQPYKSPID--QEIGITYVFPIYKNN-QLIAFVG 179

R14_Tlp12 LDKKSNFDITKQIWYQEAKNNKGITITQPYKSPID--QEIGITYVFPIYKNN-QLIAFVG 179

RM1875_Tlp15 --------LIGTDLYNAKDENGVFYVRELYQRALEKGGFVTFHFTKPQPNGENTIAEKTA 167

YH503_Tlp16 --------LIGTDLHNAKDENGIFYVRELYQRALDKGGFVTFHFTKPQPNGENTIAEKTA 167

FB1_Tlp16 --------LIGTDLHNAKDENGIFYVRELYQRALDKGGFVTFHFTKPQPNGENTIAEKTA 167

BFR-CA-9557_Tlp16 --------LIGTDLHNAKDENGIFYVRELYQRALDKGGFVTFHFTKPQPNGENTIAEKTA 166

15-537360_Tlp16 --------LIGTDLHNAKDENGIFYVRELYQRALDKGGFVTFHFTKPQPNGENTIAEKTA 167

OR12_Tlp16 --------LIGTDLHNAKDENGIFYVRELYQRALDKGGFVTFHFTKPQPNGENTIAEKTA 167

YH502_Tlp16 --------LIGTDLHNAKDENGIFYVRELYQRALDKGGFVTFHFTKPQPNGENTIAEKTA 167

14903A_Tlp16 --------LIGTDLYNAKDENGIFYVRELYQRALDKGGFVTFHFTKPQPNGENTIAEKTA 167

HC2-48_Tlp16 --------LIGSDLYNAKDENGIFYVRELYQRALDKGGFVTFHFTKPQPNGENTIAEKTA 167

RM5611_Tlp16 --------LIGSDLYNAKDENGIFYVRELYQRALDKGGFVTFHFTKPQPNGENTIAEKTA 167

00-2425_Tlp11 SQLPIIEKIKKNARYGD---LDTIFLGSPSRLNYDGTEFLGINLGMPLFNKEGKFIGIVG 217

00-6200_Tlp11 SQLPIIEKIKKNARYGD---LDTIFLGSPSRLNYDGTEFLGINLGMPLFNKEGKFIGIVG 217

YH001_Tlp11 SQLPIIEKIKKNARYGD---LDTIFLGSPSRLNYDGTEFLGINLGMPLFNKEGKFIGIVG 217

IA3902_Tlp11 SQLPIIEKIKKNARYGD---LDTIFLGSPSRLNYDGTEFLGINLGMPLFNKEGKFIGIVG 217

BCW_6290_Tlp11 SQLPIIEKIKKNARYGD---LDTIFLGSPSRLNYDGTEFLGINLGMPLFNKEGKFIGIVG 217

76339_Tlp18 SQLRIIQDIEKNARYGS---RDTLFIGSPTKLNYDGTEFLGINFGMPIFNSKGNFIGVVG 216

4031_Tlp17 GNLKIIEQVEKNAKYGD---KDSLFVGPPTKLNYDGKDFLGINFGMPIFNNKGKLIGVAG 217

MTVDSCj13_Tlp13 SQLNLIQNIEQNAKYGD---KDSVFVGSPRKLNYDNNEFLGINFGMPIFNNKGKFIGVIG 216

RM1875_Tlp13 SQLNLIQNIEQNAKYGD---KDSVFVGSPRKLNYDNNEFLGINFGMPIFNNKGKFIGVIG 216

CF2-75_Tlp13 SQLNLIQNIEQNAKYGD---KDSVFVGSPRKLNYDNNEFLGINFGMPIFNNKGKFIGVIG 216

15-537360_Tlp13 SQLNLIQNIEQNAKYGD---KDSVFVDSPRKLNYDNNEFLGINFGMPIFNNKGKFIGVIG 216

CVMN29710_Tlp13 SQLNLIQNIEQNAKYGD---KDSVFVDSPRKLNYDNNEFLGINFGMPIFNNKGKFIGVIG 216

FB1_Tlp13 SQLNLIQNIEQNAKYGD---KDSVFVDSPRKLNYDNNEFLGINFGMPIFNNKGKFIGVIG 216

CFCAN032805_Tlp13 SQLNLIQNIEQNAKYGD---KDSVFVDSPRKLNYDNNEFLGINFGMPIFNNKGKFIGVIG 216

BG2108_Tlp13 SQLNLIQNIEQNAKYGD---KDSVFVDSPRKLNYDNNEFLGINFGMPIFNNKGKFIGVIG 216

YF2105_Tlp13 SQLNLIQNIEQNAKYGD---KDSVFVDSPRKLNYDNNEFLGINFGMPIFNNKGKFIGVIG 216

YH503_Tlp13 SQLNLIQNIEQNAKYGD---KDSVFVDSPRKLNYDNNEFLGINFGMPIFNNKGKFIGVIG 216

BFRCA9557_Tlp13 SQLNLIQNIEQNAKYGD---KDSVFVDSPRKLNYDNNEFLGINFGMPIFNNKGKFIGVIG 216

YH502_Tlp13 SQLNLIQNIEQNAKYGD---KDSVFVDSPRKLNYDNNEFLGINFGMPIFNNKGKFIGVIG 216

OR12_Tlp13 SQLNLIQNIEQNAKYGD---KDSVFVDSPRKLNYDNNEFLGINFGMPIFNNKGKFIGVIG 216

00-1597_Tlp13 SQLNLIQNIEQNAKYGD---KDSVFVDSPRKLNYDNNEFLGINFGMPIFNNKGKFIGVIG 216

14903A_Tlp13 SQLNLIQNIEQNAKYGD---KDSVFVDSPRKLNYDNNEFLGINFGMPIFNNKGKFIGVIG 216

FJ3124_Tlp13 SQLNLIQNIEQNAKYGD---KDSVFVDSPRKLNYDNNEFLGINFGMPIFNNKGKFIGVIG 216

R14_Tlp13 SQLNLIQNIEQNAKYGD---KDSVFVGSPRKLNYDNNEFLGINFGMPIFNNKGKFIGVIG 216

MTVDSCj16_Tlp13 SQLNLIQNIEQNAKYGD---KDSVFVGSPRKLNYDNNEFLGINFGMPIFNNKGKFIGVIG 216

14980A_Tlp13 SQLNLIQNIEQNAKYGD---KDSVFVGSPRKLNYDNNEFLGINFGMPIFNNKGKFIGVIG 216

.

FDAARGOS_295_Tlp1 MTLDFSAIAAY--LLDPKSQKYDGELRVLLNSDGFVAIHPNKNLVLKNL-KDVNPNKGAQ 271

14980A_Tlp1 MTLDFSDIATY--LLDPKGQKYDGELRVLLNSDGLMAIHPNKNLVLKNL-KDVNPNKGAQ 271

CJ677CC527_Tlp1 MTLDFSAIATY--LLDPKSQKYDGELRVLLNSDGFVAIHPNKNLVLKNL-KDINPNKGAR 271

CJ677CC012_Tlp1 MTLDFSAIATY--LLDPKSQKYDGELRVLLNSDGFVAIHPNKNLVLKNL-KDINPNKGAR 271

4031_Tlp1 MTLDFSAIATY--LLDPKSQKYNGELRILLNSDGLVAIHPNKNLVLKNL-KDVNPNKGAQ 271

81116_Tlp1 MTLDFSAIATY--LLDPKSQKYNGELRILLNSDGLVAIHPNKNLVLKNL-KDVNPNKGAQ 271

35925B2_Tlp1 MTLDFSAIATY--LLDPKSQKYNGELRILLNSDGLVAIHPNKNLVLKNL-KDVNPNKGAQ 271

M1_Tlp1 MTLDFSAIATY--LLDPKSQKYNGELRILLNSDGLVAIHPNKNLVLKNL-KDVNPNKGAQ 271

PT14_Tlp1 MTLDFSAIATY--LLDPKSQKYNGELRILLNSDGLVAIHPNKNLVLKNL-KDVNPNKGAQ 271

81-176_Tlp1 MTLDFSDIATY--LLDPKGQKYDGELRVLLNSDGLMAIHPNKNLVLKNL-KDVNPNKGAQ 271

CVMN29710_Tlp1 MTLDFSDIATY--LLDPKGQKYDGELRVLLNSDGFMAIHPNKNLVLKNL-KDVNPNKGAQ 271

FB1_Tlp1 MTLDFSDIATY--LLDPKGQKYDGELRVLLNSDGFMAIHPNKNLVLKNL-KDVNPNKGAQ 271

BG2108_Tlp1 MTLDFSDIATY--LLDPKGQKYDGELRVLLNSDGFMAIHPNKNLVLKNL-KDVNPNKGAQ 271

YF2108_Tlp1 MTLDFSDIATY--LLDPKGQKYDGELRVLLNSDGFMAIHPNKNLVLKNL-KDVNPNKGAQ 271

YH501_Tlp1 MTLDFSDIATY--LLDPKGQKYDGELRVLLNSDGFMAIHPNKNLVLKNL-KDVNPNKGAQ 271

RM4661_Tlp1 MTLDFSDIATY--LLDPKGQKYDGELRVLLNSDGFMAIHPNKNLVLKNL-KDVNPNKGAQ 271

F38011_Tlp1 MTLDFSDIATY--LLDPKGQKYDGELRVLLNSDGFMAIHPNKNLVLKNL-KDINPNKGAQ 271

T1-21_Tlp1 MTLDFSDIATY--LLDPKGQKYDGELRVLLNSDGFMAIHPNKNLVLKNL-KDINPNKGAQ 271

CG8421_Tlp1 MTLDFSDIATY--LLDPKGQKYDGELRVLLNSDGFMAIHPNKNLVLKNL-KDINPNKGAQ 271

CJM1cam_Tlp1 MTLDFSDIATY--LLDPKGQKYDGELRVLLNSDGFMAIHPNKNLVLKNL-KDINPNKGAQ 271

R14_Tlp1 MTLDFSDIATY--LLDPKGQKYDGELRVLLNSDGFMAIHPNKNLVLKNL-KDINPNKGAQ 271

ICDCCJ07001_Tlp1 MTLDFSDIATY--LLDPKGQKYDGELRVLLNSDGFMAIHPNKNLVLKNL-KDINPNKGAQ 271

RM3196_Tlp1 MTLDFSDIATY--LLDPKGQKYDGELRVLLNSDGFMAIHPNKNLVLKNL-KDINPNKGAQ 271

NCTC11168_Tlp1 MTLDFSDIATY--LLDPKGQKYDGELRVLLNSDGFMAIHPNKNLVLKNL-KDINPNKGAQ 271

00-2425_Tlp1 MTLDFSDIATY--LLDPKGQKYDGELRVLLNSDGFMAIHPNKNLVLKNL-KDINPNKGAQ 271

IA3902_Tlp1 MTLDFSDIATY--LLDPKGQKYDGELRVLLNSDGFMAIHPNKNLVLKNL-KDINPNKGAQ 271

RM1285_Tlp1 MTLDFSDIATY--LLDPKGQKYDGELRVLLNSDGFMAIHPNKNLVLKNL-KDINPNKGAQ 271

00-0949_Tlp1 MTLDFSDIATY--LLDPKGQKYDGELRVLLNSDGFMAIHPNKNLVLKNL-KDINPNKGAQ 271

01-1512_Tlp1 MTLDFSDIATY--LLDPKGQKYDGELRVLLNSDGFMAIHPNKNLVLKNL-KDINPNKGAQ 271

FDAARGOS_422_Tlp1 MTLDFSDIATY--LLDPKGQKYDGELRVLLNSDGFMAIHPNKNLVLKNL-KDINPNKGAQ 271

FORC_056_Tlp1 MTLDFSDIATY--LLDPKGQKYDGELRVLLNSDGFMAIHPNKNLVLKNL-KDINPNKGAQ 271

32488_Tlp1 MTLDFSDIATY--LLDPKGQKYDGELRVLLNSDGFMAIHPNKNLVLKNL-KDINPNKGAQ 271

CFSAN032806_Tlp1 MTLDFSDIATY--LLDPKGQKYDGELRVLLNSDGFMAIHPNKNLVLKNL-KDINPNKGAQ 271

YH001_Tlp1 MTLDFSDIATY--LLDPKGQKYDGELRVLLNSDGFMAIHPNKNLVLKNL-KDINPNKGAQ 271

00-6200_Tlp1 MTLDFSDIATY--LLDPKGQKYDGELRVLLNSDGFMAIHPNKNLVLKNL-KDINPNKGAQ 271

RM1221_Tlp1 MTLDFSDIATY--LLDPKGQKYDGELRVLLNSDGFMAIHPNKNLVLKNL-KDINPNKGAQ 271

S3_Tlp1 MTLDFSDIATY--LLDPKGQKYDGELRVLLNSDGFMAIHPNKNLVLKNL-KDINPNKGAQ 271

FDAARGOS_421_Tlp1 MTLDFSDIATY--LLDPKGQKYDGELRVLLNSDGFMAIHPNKNLVLKNL-KDINPNKGAQ 271

FJ3124_Tlp1 MTLDFSDIATY--LLDPKGQKYDGELRVLLNSDGFMAIHPNKNLVLKNL-KDINPNKGAQ 271

00-1597_Tlp1 MTLDFSDIATY--LLDPKGQKYDGELRVLLNSDGFMAIHPNKNLVLKNL-KDINPNKGAQ 271

15-537360_Tlp20 I-DILVKDLQREFENLPGR-------TFVFDSENSVFVSTDKEL--------LKPGYDVS 232

76339_Tlp20 I-DILVKDLQREFENLPGR-------TFVFDSKNSIFASTDKEL--------LKPGYDVS 232

HC2-48_Tlp20 I-DILVKDLQREFENLPGR-------TFVFDSENSIFVSTDKEL--------LKPGYDVS 232

CFSAN032805_Tlp20 I-DILVKDLQREFENLPGR-------TFVFDSENSIFVSTNKEL--------LKPGYDVS 231

CVMN29710_Tlp20 I-DILVKDLQREFENLPGR-------TFVFDSENSIFVSTDKEL--------LKPGYDVS 230

YH501_Tlp20 I-DILVKDLQREFENLPGR-------TFVFDSENSIFVSTDKEL--------LKPGYDVS 234

CF2-75_Tlp20 I-DILVKDLQREFENLPGR-------TFVFDSENSIFVSTDKEL--------LKPGYDVS 235

CO2-160_Tlp20 I-DILVKDLQREFENLPGR-------TFVFDSENSIFVSTDKEL--------LKPGYDVS 232

CO2-160_Tlp20b I-DILVKDLQREFENLPGR-------TFVFDSENSIFVSTDKEL--------LKPGYDVS 232

RM5611_Tlp20 I-DILVKDLQREFENLPGR-------TFVFDSENSIFVSTDKEL--------LKPGYDVS 232

14903A_Tlp20 I-DILVKDLQREFENLPGR-------TFVFDSENSIFVSTNKEL--------LKPGYDVS 234

YH502_Tlp20 I-DILVKDLQREFENLPGR-------TFVFDSENSIFVSTNKEL--------LKPGYDVS 231

RM3196_Tlp23 V-DVLAADLQAEFENLPGR-------IFVFDEENKVFVSTDKTL--------LQQGYDIS 232

ICDCCJ07001_Tlp23 V-DILAADLQAEFENLPGR-------IFVFDEENKVFVSTDKTL--------LQQGYDIS 232

RM1285_Tlp2 V-DILAADLQAEFENLPGR-------TFVFDEENKVFVSTDKAL--------LQKGYDIS 232

CFSAN032806_Tlp2 V-DILAADLQAEFENLPGR-------TFVFDEENKVFVSTDKTL--------LQQGYDIS 232

RM1221_Tlp2 V-DILAADLQAEFENLPGR-------TFVFDEENKVFVSTDKTL--------LQQGYDIS 232

S3_Tlp2 V-DILAADLQAEFENLPGR-------TFVFDEENKVFVSTDKTL--------LQQGYDIS 232

FDAARGOS_422_Tlp2 V-DILAADLQAEFENLPGR-------TFVFDEENKVFVSTDKAL--------LQKGYDIS 232

81-176_Tlp2 V-DILAADLQAEFENLPGR-------TFVFDEENKVFVSTDKAL--------LQKGYDIS 232

F38011_Tlp2 V-DILAADLQAEFENLPGR-------TFVFDEENKVFVSTDKAL--------LQKGYDIS 232

NCTC11168_Tlp2 V-DILAADLQAEFENLPGR-------TFVFDEENKVFVSTDKAL--------LQKGYDIS 232

MTVDSCj07_Tlp2 V-DILAADLQAEFENLPGR-------TFVFDEENKVFVSTDKAL--------LQKGYDIS 232

CJM1cam_Tlp24 V-DVLVTDLQAEFENLPGR-------TFVFDEENKVFASTDKTL--------LQQGYDIS 232

M1_Tlp24 V-DVLVTDLQAEFENLPGR-------TFVFDEENKVFASTDKTL--------LQQGYDIS 232

81116_Tlp2 V-DVLVTDLQAEFENLPGR-------TFVFDEENKVFASTDKTL--------LQQGYDIS 232

4031_Tlp23 V-DVLVTDLQAEFENLPGR-------TFVFDEENKVFASTDKTL--------LQQGYDIS 231

CVMN29710_Tlp14 I-DTLLTGLQDAIARTPGN-------AFVFNSKDEIFAAPNKAL--------LDPSVDYS 232

RM4661_Tlp14 I-DALLTGLQDAIARTPGN-------AFVFNSKDEIFAAPNKAL--------LDPSVDYS 232

MG1116_Tlp14 F-DVLLISLQDEIARTPGN-------TFVFDHQDRIFAATNKAL--------LDPSVDHS 232

BG2108_Tlp14 F-DVLLISLQDEITRTPGN-------TFVFDHKDRIFAATNKAL--------LDPSVDHS 232

YF2105_Tlp14 F-DVLLISLQDEITRTPGN-------TFVFDHKDRIFAATNKAL--------LDPSVDHS 232

YH502_Tlp14 I-DVLLTSLQDRIARTPGN-------TFVFDHKDRVFAATNKAL--------LDPSVDHS 243

WA333_Tlp14 F-DVLLISLQDLIARTPGN-------TFVFDHKDRVFAATNKAL--------LDPSVDHS 232

BP3181_Tlp14 F-DVLLTSLQDRIARTPGN-------SFVFDHKDRIFAATNKAL--------LDPSVDHS 232

ZV1224_Tlp14a F-DVLLIDLQDKIARTPGN-------TFVFDHQDRIFAATNKAL--------LDPSVDHS 232

ZV1224_Tlp14b F-DVLLIDLQDKIARTPGN-------TFVFDHQDRIFAATNKAL--------LDPSVDHS 232

YH503_Tlp14 I-DVLLTSLQDRIARTPGN-------TFVFDHKDRVFAATNEAL--------LDPSVDHS 243

14903A_Tlp14 F-DVLLINLQDEIARTPGN-------TFVFDHQDRIFAATNKAL--------LDPSVDHS 232

OR12_Tlp14 I-DVLLTNLQDEIARTPGN-------TFVFDHKDRVFAATNKAL--------LDPSVDHS 232

CFSAN032805_Tlp14 F-DVLLINLQDEIARTPGN-------TFVFDHKDRVFAAANKAL--------LDPSVDHS 243

BFR-CA-9557_Tlp14 F-DVLLISLQDEIARTPGN-------TFVFDHKDRVFAATNKAL--------LDPSVDHS 232

15-537360_Tlp14 L-DVLLISLQDEIARTPGN-------TFVFDHKDRVFAATNKAL--------LDPSVDHS 232

YH501_Tlp14 F-DVLLISLQDEIARTPGN-------TFVFDHKDRVFAATNKAL--------LDPSVDHS 232

T1-21_Tlp19b F-DVLLTSLQDRIARTPGN-------SFVFDHKDRIFAAANKAL--------LDPSVDHS 232

CG8421_Tlp14 I-DILLTSLQDQIARTPGN-------TFVFDNKDKIFAATNEAL--------LDPSVDHS 232

MTVDSCj16_Tlp14 I-DILLTSLQDQIARTPGN-------TFVFDNKDKIFAATNEAL--------LDPSVDHS 232

01-1512_Tlp14 I-DILLTSLQDQIARTPGN-------TFVFDNKDKIFAATNEAL--------LDPSVDHS 232

00-0949_Tlp14 I-DILLTSLQDQIARTPGN-------TFVFDNKDKIFAATNEAL--------LDPSVDHS 232

MTVDSCj13_Tlp14 I-DILLTSLQDQIARTPGN-------TFVFDNKDKIFAATNEAL--------LDPSVDHS 232

S3_Tlp14 I-DILLTSLQDQIARTPGN-------TFVFDNKDKIFAATNEAL--------LDPSVDHS 232

PT14_Tlp14 I-DILLTSLQDQIARTPGN-------TFVFDNKDKIFAATNEAL--------LDPSVDHS 232

14980A_Tlp14 I-DILLTSLQDQIARTPGN-------TFVFDNKDKIFAATNEAL--------LDPSVDHS 248

FJ3124_Tlp14 I-DILLTSLQDQIARTPGN-------TFVFDNKDKIFAATNEAL--------LDPSVDHS 232

00-1597_Tlp14 F-DVLLTSLQDRIARTPGN-------TFVFDHKDKVFAATNKAL--------LDPSVDHS 232

R14_Tlp14 I-DILLTSLQDQIARTPGN-------TFVFDNKDKIFAATNEAL--------LDPSVDHS 232

CG8421_Tlp25 KLTLWVGILVVLILAITST-------VSYFDAKNHT------------------------ 43

HC2-48_Tlp3 V-DIPSEDLQNLVAKTPGN-------TFLFDQKNKIFAATNKEL--------LNPSIDHS 227

35925B2_Tlp3 I-DIPSEDLQNLVAKTPGN-------TFLFDQKNKIFAATNKEL--------LNPSIDHS 238

14980A_Tlp3 V-DIPLEDLQNSVANTPGN-------IFLFDQKNKIFAATNKEL--------LNPSIDHS 238

00-1597_Tlp3b V-DIPSGDLQNLVAKTPGN-------TFLFDQKNKIFAATNKEL--------LNPSIDHS 227

ICDCCJ07001_Tlp3 V-DIPSEDLQNLVAKTPGN-------TFLFDQKNKIFAATNKEL--------LNPSIDHS 227

RM3196_Tlp3 V-DIPSEDLQNLVAKTPGN-------TFLFDQKNKIFAATNKEL--------LNPSIDHS 227

RM1875_Tlp3 V-DIPSEDLQNLVANTPGN-------TFLFDQKNKIFAATNKEL--------LNPSIDHS 227

CF2-75_Tlp3 V-DIPSEDLQNLVAKTPGN-------TFLFDQKNKIFAATNKEL--------LNPSIDHS 238

RM5611_Tlp3 V-DIPSEDLQNLVAKTPGN-------TFLFDQKNKIFAATNKEL--------LNPSIDHS 227

MTVDSCj16_Tlp3 V-DIPSEDLQNLVAKTPGN-------TFLFDQKNKIFAATNEAL--------LDPSVDHS 227

01-1512_Tlp3 V-DIPSEDLQNLVAKTPGN-------TFLFDQKNKIFAATNKEL--------LNPSIDHS 227

MTVDSCj13_Tlp3 V-DIPSEDLQNLVANTPGN-------TFLFDQKNKIFAATNKEL--------LNPSIDHS 227

32488_Tlp3a V-DIPLEDLQNSVAKTPGN-------TFLFDQKNKIFAATNKEL--------LNPSIDHS 227

81116_Tlp3 V-DIPLEDLQNSVAKTPGN-------TFLFDQKNKIFAATNKEL--------LNPSIDHS 227

32488_Tlp3b V-DIPLEDLQNSVAKTPGN-------TFLFDQKNKIFAATNKEL--------LNPSIDHS 227

FB1_Tlp3 V-DIPSEDLQNLVAKTPGN-------TFLFDQKNKIFAATNKEL--------LNPSIDHS 227

PT14_Tlp3 V-DIPSEDLQNLVAKTPGN-------TFLFDQKNKIFAATNEAL--------LDPSVDHS 227

00-6200_Tlp3a V-DIPSEDLQNLVAKTPGN-------TFLFDQKNKIFAATNKEL--------LNPSIDHS 227

RM1221_Tlp3 V-DIPSEDLQNLVANTPGN-------TFLFDQKNKIFAATNKEL--------LNPSIDHS 227

S3_Tlp3 V-DIPSEDLQNLVANTPGN-------TFLFDQKNKIFAATNKEL--------LNPSIDHS 227

FDAARGOS_421_Tlp3 V-DIPSEDLQNLVANTPGN-------TFLFDQKNKIFAATNKEL--------LNPSIDHS 238

CFSAN032806_Tlp3 V-DIPSEDLQNLVAKTPGN-------TFLFDQKNKIFAATNKEL--------LNPSIDHS 238

IA3901_Tlp3b V-DIPSEDLQNLVAKTPGN-------TFLFDQKNKIFAATNKEL--------LNPSIDHS 227

00-6200_Tlp3b V-DIPSEDLQNLVAKTPGN-------TFLFDQKNKIFAATNKEL--------LNPSIDHS 227

BCW_6290_Tlp3b V-DIPSEDLQNLVAKTPGN-------TFLFDQKNKIFAATNKEL--------LNPSIDHS 227

00-2425_Tlp3a V-DIPSEDLQNLVAKTPGN-------TFLFDQKNKIFAATNKEL--------LNPSIDHS 227

00-2425_Tlp3b V-DIPSEDLQNLVAKTPGN-------TFLFDQKNKIFAATNKEL--------LNPSIDHS 227

YH001_Tlp3a V-DIPSEDLQNLVAKTPGN-------TFLFDQKNKIFAATNKEL--------LNPSIDHS 227

YH001_Tlp3b V-DIPSEDLQNLVAKTPGN-------TFLFDQKNKIFAATNKEL--------LNPSIDHS 227

00-0949_Tlp3 V-DIPSEDLQNLVAKTPGN-------TFLFDQKNKIFAATNKEL--------LNPSIDHS 227

NCTC11168_Tlp3 V-DIPSEDLQNLVAKTPGN-------TFLFDQKNKIFAATNKEL--------LNPSIDHS 238

F38011_Tlp3 V-DIPSEDLQNLVAKTPGN-------TFLFDQKNKIFAATNKEL--------LNPSIDHS 227

RM1285_Tlp3 V-DIPSEDLQNLVAKTPGN-------TFLFDQKNKIFAATNKEL--------LNPSIDHS 227

FDAARGOS_422_Tlp3 V-DIPSEDLQNLVAKTPGN-------TFLFDQKNKIFAATNKEL--------LNPSIDHS 238

MTVDSCj07_Tlp3 V-DIPSEDLQNLVAKTPGN-------TFLFDQKNKIFAATNKEL--------LNPSIDHS 227

IA3901_Tlp3a V-DIPSEDLQNLVAKTPGN-------TFLFDQKNKIFAATNKEL--------LNPSIDHS 227

BCW_6290_Tlp3a V-DIPSEDLQNLVAKTPGN-------TFLFDQKNKIFAATNKEL--------LNPSIDHS 227

T1-21_Tlp19a V-DIPLEDLQNSVANTPGN-------TFLFDQKNKIFAATNKEL--------LNPSIDHS 227

CJ677CC012_Tlp3 V-DIPSEDLQNLVAKTPGN-------TFLFDQKNKIFAATNKEL--------LNPSIDHS 227

CJM1cam_Tlp3 V-DIPSEDLQNLVAKTPGN-------TFLFDQKNKIFAATNKEL--------LNPSIDHS 227

M1_Tlp3 V-DIPSEDLQNLVAKTPGN-------TFLFDQKNKIFAATNKEL--------LNPSIDHS 227

4031_Tlp3 V-DIPSEHLQNLVAKTPGN-------TFLFDQKNKIFAATNKEL--------LNPSIDHS 227

R14_Tlp3 V-DIPLEDLQNSVANTPGN-------TFLFDQKNKIFAATNKEL--------LNPSIDHS 227

FDAARGOS_295_Tlp21 SEYNLANYAKD-VL-SVGR--SQNTYTAIYDPQGTILFHEKTELMLQKNTLSTN------ 223

FORC_046_Tlp4 GDYDLANFSTN-VL-TVGK--SDNTFTEVLDSEGTILFNDEVAKILTKTELSIN------ 232

FDAARGOS_422_Tlp4 GDYDLANFSTN-VL-TVGK--SDNTFTEVLDSEGTILFNDEVAKILTKTELSIN------ 232

ICDCCJ07001_Tlp4 GDYDLAKFSTD-VL-AVGK--SQNTYTVVLDPEGTILFRDDITKILTKTELSIN------ 232

RM3196_Tlp4 GDYDLAKFSTD-VL-AVGK--SQNTYTVVLDPEGTILFRDDITKILTKTELSIN------ 232

T1-21_Tlp4 GDYDLAKFSTD-VL-AVGK--SQNTYTVVLDPEGTILFRDDITKILTKTELSIN------ 232

F38011_Tlp4 GDYDLANFSTN-VL-TVGK--SDNTFTEVLDSEGTILFNDEVAKILTKTELSIN------ 232

HF5-4A-4_Tlp22 GDYDLANFSTN-VL-TVGK--SDNTFTEVLDSEGTILFNDEVAKILTKTELSIN------ 178

00-0949_Tlp4 GDYDLANFSTN-VL-TVGK--SDNTFTEVLDSEGTILFNDEVAKILTKTELSIN------ 232

01-1512_Tlp4 GDYDLANFSTN-VL-TVGK--SDNTFTEVLDSEGTILFNDEVAKILTKTELSIN------ 232

81-176_Tlp4 GDYDLANFSTN-VL-TVGK--SDNTFTEVLDSEGTILFNDEVAKILTKTELSIN------ 232

32488_Tlp4 GDYDLANFSTN-VL-TVGK--SDNTFTEVLDSEGTILFNDEVAKILTKTELSIN------ 232

NCTC11168_Tlp4 GDYDLANFSTN-VL-TVGK--SDNTFTEVLDSEGTILFNDEVAKILTKTELSIN------ 232

CFSAN032806_Tlp4 GDYDLANFSTN-VL-TVGK--SDNTFTEVLDSEGTILFNDEVAKILTKTELSIN------ 232

81116_Tlp4 GDYDLANFSTN-VL-TVGK--SDNTFTEVLDSEGTILFNDEVAKILTKTELSIN------ 232

RM1285_Tlp12 GDYNLDKFSKD-VL-SLGH--SSTTYAAVYDSEGRIIFHEVLDRILTKNTLSVN------ 229

PT14_Tlp12 GDYNLDKFSKD-VL-SLGH--SSTTYAAVYDSEGRIIFHEVLDRILTKNTLSVN------ 229

MTVJDCj07_Tlp12 GDYNLDKFSKD-VL-SLGH--SSTTYAAVYDSEGRIIFHEVLDRILTKNTLSVN------ 229

RM1221_Tlp12 GDYNLDKFSKD-VL-SLGH--SSTTYAAVYDSEGRIIFHEVLDRILTKNTLSVN------ 229

FDAARGOS_421_Tlp12 GDYNLDKFSKD-VL-SLGH--SSTTYAAVYDSEGRIIFHEVLDRILTKNTLSVN------ 229

35925B2_Tlp12 GDYNLDKFSKD-VL-SLGH--SSTTYAAVYDSEGRIIFHEVLDRILTKNTLSIN------ 229

CJM1cam_Tlp12 GDYNLDKFSKD-VL-SLGH--SSTTYAAVYDSEGRIIFHEVLDRILTKNTLSVN------ 229

M1_Tlp12 GDYNLDKFSKD-VL-SLGH--SSTTYAAVYDSEGRIIFHEVLDRILTKNTLSVN------ 229

S3_Tlp12 GDYNLDKFSKD-VL-SLGH--SSTTYAAVYDSEGRIIFHEVLDRILTKNTLSVN------ 229

00-1597_Tlp12 GDYNLDKFSKD-VL-SLGH--SSTTYAAVYDSEGRIIFHEVLDRILTKNTLSVN------ 229

R14_Tlp12 GDYNLDKFSKD-VL-SLGH--SSTTYAAVYDSEGRIIFHEVLDRILTKNTLSVN------ 229

RM1875_Tlp15 Y----------------------------------------------------------- 168

YH503_Tlp16 Y----------------------------------------------------------- 168

FB1_Tlp16 Y----------------------------------------------------------- 168

BFR-CA-9557_Tlp16 Y----------------------------------------------------------- 167

15-537360_Tlp16 Y----------------------------------------------------------- 168

OR12_Tlp16 Y----------------------------------------------------------- 168

YH502_Tlp16 Y----------------------------------------------------------- 168

14903A_Tlp16 Y----------------------------------------------------------- 168

HC2-48_Tlp16 Y----------------------------------------------------------- 168

RM5611_Tlp16 Y----------------------------------------------------------- 168

00-2425_Tlp11 FTFDFLEISET-IL-DPKLDFYKDDLRFLITDQGVIVIHKNKDAILKTL-PEINQDASVQ 274

00-6200_Tlp11 FTFDFLEISET-IL-DPKLDFYKDDLRFLITDQGVIVIHKNKDAILKTL-PEINQDASVQ 274

YH001_Tlp11 FTFDFLEISET-IL-DPKLDFYKDDLRFLITDQGVIVIHKNKDAILKTL-PEINQDASVQ 274

IA3902_Tlp11 FTFDFLEISET-IL-DPKLDFYKDDLRFLITDQGVIVIHKNKDAILKTL-PEINQDASVQ 274

BCW_6290_Tlp11 FTFDFLEISET-IL-DPKLDFYKDDLRFLITDQGVIVIHKNKDAILKTL-PEINQDASVQ 274

76339_Tlp18 YSLDFLEISQA-ML-DPKLDFFEGDLRALTTDQGVITIHKDKNAILKTL-TDINKDPSVK 273

4031_Tlp17 YTLDFSEVSET-IL-DPKLDFFEGDLRFLMTDKGVITIHKNHNAILKTL-GDINKDPSVE 274

MTVDSCj13_Tlp13 YTLDLLEISEI-IL-DPKFDFFEGDLRILMNDQGIIAVHKNKNGILKTL-FDINKDQSAQ 273

RM1875_Tlp13 YTLDLLEISEI-IL-DPKFDFFEGDLRILMNDQGIIAVHKIKNGILKTL-FDINKDQSAQ 273

CF2-75_Tlp13 YTLDLLEISEI-IL-DPKFDFFEGDLRILMNDQGIIAVHKIKNGILKTL-FDINKDQSAQ 273

15-537360_Tlp13 YTIDLLEISET-IL-DPKFDFFEGDLRFLMNDQGIIAIHKNKNAILKTL-FDINKDQSAQ 273

CVMN29710_Tlp13 YTIDLLEISET-IL-DPKFDFFEGDLRFLMNDQGIIAIHKNKNAILKTL-FDINKDQSAQ 273

FB1_Tlp13 YTIDLLEISET-IL-DPKFDFFEGDLRFLMNDQGIIAIHKNKNAILKTL-FDINKDQSAQ 273

CFCAN032805_Tlp13 YTIDLLEISET-IL-DPKFDFFEGDLRFLMNDQGIIAIHKNKNAILKTL-FDINKDQSAQ 273

BG2108_Tlp13 YTIDLLEISET-IL-DPKFDFFEGDLRFLMNDQGIIAIHKNKNAILKTL-FDINKDQSAQ 273

YF2105_Tlp13 YTIDLLEISET-IL-DPKFDFFEGDLRFLMNDQGIIAIHKNKNAILKTL-FDINKDQSAQ 273

YH503_Tlp13 YTIDLLEISET-IL-DPKFDFFEGDLRFLMNDQGIIAIHKNKNAILKTL-FDINKDQSAQ 273

BFRCA9557_Tlp13 YTIDLLEISET-IL-DPKFDFFEGDLRFLMNDQGIIAIHKNKNAILKTL-FDINKDQSAQ 273

YH502_Tlp13 YTIDLLEISET-IL-DPKFDFFEGDLRFLMNDQGIIAIHKNKNAILKTL-FDINKDQSAQ 273

OR12_Tlp13 YTIDLLEISET-IL-DPKFDFFEGDLRFLMNDQGIIAIHKNKNAILKTL-FDINKDQSAQ 273

00-1597_Tlp13 YTIDLLEISET-IL-DPKFDFFEGDLRFLMNDQGIIAIHKNKNAILKTL-FDINKDQSAQ 273

14903A_Tlp13 YTIDLLEISET-IL-DPKFDFFEGDLRFLMNDQGIIAIHKNKNAILKTL-FDINKDQSAQ 273

FJ3124_Tlp13 YTIDLLEISET-IL-DPKFDFFEGDLRFLMNDQGIIAIHKNKNAILKTL-FDINKDQSAQ 273

R14_Tlp13 YTIDLLEISET-IL-DPKFDFFEGDLRFLMNDQGIIAIHKNKNAILKTL-FDINKDQSAQ 273

MTVDSCj16_Tlp13 YTIDLLEISET-IL-DPKFDFFEGDLRFLMNDQGIIAIHKNKNAILKTL-FDINKDQSAQ 273

14980A_Tlp13 YTIDLLEISET-IL-DPKFDFFEGDLRFLMNDQGIIAIHKNKNAILKTL-FDINKDQSAQ 273

FDAARGOS_295_Tlp1 ETYKAMSEGK-NGVFNY------IASDGDDSYAAINSFKV--QDSSWTVLVTAPKYSVFE 322

14980A_Tlp1 ETYKAMSEGK-NGVFDY------IASDGDDSYAAINTFKV--QDSSWTVLVTAPKYSVFE 322

CJ677CC527_Tlp1 ETYKAMSEGK-NGVFNY------IAFDGDDSYAAINSFKV--QDSSWTVLVTAPKYSVFE 322

CJ677CC012_Tlp1 ETYKAMSEGK-NGVFNY------IAFDGDDSYAAINSFKV--QDSSWTVLVTAPKYSVFE 322

4031_Tlp1 ETYKAMSEGK-NGVFNY------IAFDGDDSYAAINSFKV--QDSSWTVLVTAPKYSVFE 322

81116_Tlp1 ETYKAMSEGK-NGVFNY------IAFDGDDSYAAINSFKV--QDSSWTVLVTAPKYSVFE 322

35925B2_Tlp1 ETYKAMSEGK-NGVFNY------IAFDGDDSYAAINSFKV--QDSSWTVLVTAPKYSVFE 322

M1_Tlp1 ETYKAMSEGK-NGVFNY------IAFDGDDSYAAINSFKV--QDSSWTVLVTAPKYSVFE 322

PT14_Tlp1 ETYKAMSEGK-NGVFNY------IAFDGDDSYAAINSFKV--QDSSWTVLVTAPKYSVFE 322

81-176_Tlp1 ETYKAISEGK-NGVFDY------IASDGDDSYAAINSFKV--QDSSWAVLVTAPKYSVFK 322

CVMN29710_Tlp1 ETYKAISEGK-NGVFDY------IASDGDDSYAAINSFKV--QDSSWAVLVTAPKYSVFK 322

FB1_Tlp1 ETYKAISEGK-NGVFDY------IASDGDDSYAAINSFKV--QDSSWAVLVTAPKYSVFK 322

BG2108_Tlp1 ETYKAISEGK-NGVFDY------IASDGDDSYAAINSFKV--QDSSWAVLVTAPKYSVFK 322

YF2108_Tlp1 ETYKAISEGK-NGVFDY------IASDGDDSYAAINSFKV--QDSSWAVLVTAPKYSVFK 322

YH501_Tlp1 ETYKAISEGK-NGVFDY------IASDGDDSYAAINSFKV--QDSSWAVLVTAPKYSVFK 322

RM4661_Tlp1 ETYKAISEGK-NGVFDY------IASDGDDSYAAINSFKV--QDSSWAVLVTAPKYSVFK 322

F38011_Tlp1 ETYKAISEGK-NGVFNY------IASDGDDSYAAINSFKV--QDSSWAVLVTAPKYSVFK 322

T1-21_Tlp1 ETYKAISEGK-NGVFNY------IASDGDDSYAAINSFKV--QDSSWAVLVTAPKYSVFK 322

CG8421_Tlp1 ETYKAISEGK-NGVFNY------IASDGDDSYAAINSFKV--QDSSWAVLVTAPKYSVFK 322

CJM1cam_Tlp1 ETYKAISEGK-NGVFNY------IASDGDDSYAAINSFKV--QDSSWAVLVTAPKYSVFK 322

R14_Tlp1 ETYKAISEGK-NGVFNY------IASDGDDSYAAINSFKV--QDSSWAVLVTAPKYSVFK 322

ICDCCJ07001_Tlp1 ETYKAISEGK-NGVFNY------IASDGDDSYAAINSFKV--QDSSWAVLVTTPKYSVFK 322

RM3196_Tlp1 ETYKAISEGK-NGVFNY------IASDGDDSYAAINSFKV--QDSSWAVLVTTPKYSVFK 322

NCTC11168_Tlp1 ETYKAISEGK-NGVFNY------IASDGDDSYAAINSFKV--QDSSWAVLVTAPKYSVFK 322

00-2425_Tlp1 ETYKAISEGK-NGVFNY------IASDGDDSYAAINSFKV--QDSSWAVLVTAPKYSVFK 322

IA3902_Tlp1 ETYKAISEGK-NGVFNY------IASDGDDSYAAINSFKV--QDSSWAVLVTAPKYSVFK 322

RM1285_Tlp1 ETYKAISEGK-NGVFNY------IASDGDDSYAAINSFKV--QDSSWAVLVTAPKYSVFK 322

00-0949_Tlp1 ETYKAISEGK-NGVFNY------IASDGDDSYAAINSFKV--QDSSWAVLVTAPKYSVFK 322

01-1512_Tlp1 ETYKAISEGK-NGVFNY------IASDGDDSYAAINSFKV--QDSSWAVLVTAPKYSVFK 322

FDAARGOS_422_Tlp1 ETYKAISEGK-NGVFNY------IASDGDDSYAAINSFKV--QDSSWAVLVTAPKYSVFK 322

FORC_056_Tlp1 ETYKAISEGK-NGVFNY------IASDGDDSYAAINSFKV--QDSSWAVLVTAPKYSVFK 322

32488_Tlp1 ETYKAISEGK-NGVFNY------IASDGDDSYAAINSFKV--QDSSWAVLVTAPKYSVFK 322

CFSAN032806_Tlp1 ETYKAISEGK-NGVFNY------IASDGDDSYAAINSFKV--QDSSWAVLVTAPKYSVFK 322

YH001_Tlp1 ETYKAISEGK-NGVFNY------IASDGDDSYAAINSFKV--QDSSWAVLVTAPKYSVFK 322

00-6200_Tlp1 ETYKAISEGK-NGVFNY------IASDGDDSYAAINSFKV--QDSSWAVLVTAPKYSVFK 322

RM1221_Tlp1 ETYKAISEGK-NGVFNY------IASDGDDSYAAINSFKV--QDSSWAVLVTAPKYSVFK 322

S3_Tlp1 ETYKAISEGK-NGVFNY------IASDGDDSYAAINSFKV--QDSSWAVLVTAPKYSVFK 322

FDAARGOS_421_Tlp1 ETYKAISEGK-NGVFNY------IASDGDDSYAAINSFKV--QDSSWAVLVTAPKYSVFK 322

FJ3124_Tlp1 ETYKAISEGK-NGVFNY------IASDGDDSYAAINSFKV--QDSSWAVLVTAPKYSVFK 322

00-1597_Tlp1 ETYKAISEGK-NGVFNY------IASDGDDSYAAINSFKV--QDSSWAVLVTAPKYSVFK 322

15-537360_Tlp20 PVANIAKDKKDYEPFRYARP--------------------LDGTQR-----FGVCAKVLG 267

76339_Tlp20 PVADIAKDKKDYEPFHYIRP--------------------LDGTER-----FGVCAKVLG 267

HC2-48_Tlp20 PVANIAKDKKDYEPFRYVRP--------------------LDGTQR-----FGVCAKVLG 267

CFSAN032805_Tlp20 PVANIAKDKKDYEPFRYVRP--------------------LDGTQR-----FGVCAKVLG 266

CVMN29710_Tlp20 PVANIAKDKKDYEPFRYVRP--------------------LDGTQR-----FGVCAKVLG 265

YH501_Tlp20 PVANIAKDKKDYEPFRYVRP--------------------LDGTQR-----FGVCAKVLG 269

CF2-75_Tlp20 PVANIAKDKKDYEPFRYVRP--------------------LDGTQR-----FGVCAKVLG 270

CO2-160_Tlp20 PVANIAKDKKDYEPFRYVRP--------------------LDGTQR-----FGVCAKVLG 267

CO2-160_Tlp20b PVANIAKDKKDYEPFRYVRP--------------------LDGTQR-----FGVCAKVLG 267

RM5611_Tlp20 PVANIAKDKKDYEPFRYVRP--------------------LDGTQR-----FGVCAKVLG 267

14903A_Tlp20 LVANIAKDKKDYEPFRYVRP--------------------LDGTQR-----FGVCAKVLG 269

YH502_Tlp20 LVANIAKDKKDYEPFRYVRP--------------------LDGTQR-----FGVCAKVLG 266

RM3196_Tlp23 TIANLAKTKKDFEPFEYTRP--------------------KDGSER-----FAVCVKVSG 267

ICDCCJ07001_Tlp23 TIANLAKTKKDFEPFEYTRP--------------------KDGSER-----FAVCVKVSG 267

RM1285_Tlp2 AIANLAKTKEDLEPFEYTRP--------------------KDGNER-----FAVCTKVSG 267

CFSAN032806_Tlp2 TIANLAKTKEDLEPFEYTRP--------------------KDGNER-----FAVCTKVSG 267

RM1221_Tlp2 TIANLAKTKEDLEPFEYTRP--------------------KDGNER-----FAVCTKVSG 267

S3_Tlp2 TIANLAKTKEDLEPFEYTRP--------------------KDGNER-----FAVCTKVSG 267

FDAARGOS_422_Tlp2 AIANLAKTKEDLEPFEYTRP--------------------KDGNER-----FAVCTKVSG 267

81-176_Tlp2 AIANLAKTKEDLEPFEYTRP--------------------KDGNER-----FAVCTKVSG 267

F38011_Tlp2 AIANLAKTKEDLEPFEYTRP--------------------KDGNER-----FAVCTKVSG 267

NCTC11168_Tlp2 AIANLAKTKEDLEPFEYTRP--------------------KDGNER-----FAVCTKVSG 267

MTVDSCj07_Tlp2 AIANLAKTKEDLEPFEYTRP--------------------KDGNER-----FAVCTKVSG 267

CJM1cam_Tlp24 AIANLAKIKENFEPFEYTRP--------------------KDGSER-----FAVCTKVSG 267

M1_Tlp24 AIANLAKIKENFEPFEYTRP--------------------KDGSER-----FAVCTKVSG 267

81116_Tlp2 AIANLAKIKENFEPFEYTRP--------------------KDGSER-----FAVCTKVSG 267

4031_Tlp23 AIANLAKIKENFEPFEYTRP--------------------KDGSER-----FAVCTKVSG 266

CVMN29710_Tlp14 PILNAYKLNGDNNFFSYE----------------------LNNEEG-----LGVCKKIF- 264

RM4661_Tlp14 PILNAYKLNGDNNFFSYE----------------------LNNEEG-----LGVCKKIF- 264

MG1116_Tlp14 PVLNAYKAHGDNNFFSYK----------------------LNNEER-----LGVCTKVF- 264

BG2108_Tlp14 PVLNAYKAHGDNNFFSYK----------------------LNNEER-----LGVCTKVF- 264

YF2105_Tlp14 PVLNAYKAHGDNNFFSYK----------------------LNNEER-----LGVCTKVF- 264

YH502_Tlp14 PVLNAYKTHGDYNFFTYG----------------------LDGKER-----LGACTKVF- 275

WA333_Tlp14 PVLNAYKAHGDNNFFSYK----------------------LNNEER-----LGTCTKVF- 264

BP3181_Tlp14 PVLNAYKAHGDNNFFSYK----------------------LNNEER-----LGVCTKVF- 264

ZV1224_Tlp14a PVLNAYKAHGDNNFFSYK----------------------LNNEER-----LGVCTKVF- 264

ZV1224_Tlp14b PVLNAYKAHGDNNFFSYK----------------------LNNEER-----LGVCTKVF- 264

YH503_Tlp14 PVLNAYKAHGDNNFFSYK----------------------LNNEER-----LGACTKAF- 275

14903A_Tlp14 PVLNAYKAHGDNNFFSYK----------------------LNNEER-----LGVCTKVF- 264

OR12_Tlp14 PVLNAYKAHGDNNFFSYK----------------------LNNEER-----LGTCTKVF- 264

CFSAN032805_Tlp14 PVLNAYKAHGDNNFFSYK----------------------LNNEER-----LGTCTKVF- 275

BFR-CA-9557_Tlp14 PVLNAYKAHGDNNFFSYK----------------------LNNEER-----LGTCTKVF- 264

15-537360_Tlp14 PVLNAYKAHGDNNFFSYK----------------------LNNEER-----LGTCTKVF- 264

YH501_Tlp14 PVLNAYKAHGDNNFFSYK----------------------LNNEER-----LGTCTKVF- 264

T1-21_Tlp19b PVLNAYKAHGDNNFFSYK----------------------LNNEER-----LGACTKVF- 264

CG8421_Tlp14 PVLNAYKAHGDNNFFSYK----------------------LNNEER-----LGACTKVF- 264

MTVDSCj16_Tlp14 PVLNAYKAHGDNNFFSYK----------------------LNNEER-----LGACTKVF- 264

01-1512_Tlp14 PVLNAYKAHGDNNFFSYK----------------------LNNEER-----LGACTKVF- 264

00-0949_Tlp14 PVLNAYKAHGDNNFFSYK----------------------LNNEER-----LGACTKVF- 264

MTVDSCj13_Tlp14 PVLNAYKAHGDNNFFSYK----------------------LNNEER-----LGACTKVF- 264

S3_Tlp14 PVLNAYKAHGDNNFFSYK----------------------LNNEER-----LGACTKVF- 264

PT14_Tlp14 PVLNAYKAHGDNNFFSYK----------------------LNNEER-----LGACTKVF- 264

14980A_Tlp14 PVLNAYKAHGDNNFFSYK----------------------LNNEER-----LGACTKVF- 280

FJ3124_Tlp14 PVLNAYKAHGDNNFFSYK----------------------LNNEER-----LGACTKVF- 264

00-1597_Tlp14 PVLNAYKAHGDNNFFSYK----------------------LNNEER-----LGACTKVF- 264

R14_Tlp14 PVLNAYKTHGDYNFFTYG----------------------LDGKER-----LGACTKVF- 264

CG8421_Tlp25 ----------------YE----------------------LLKENQ-----LKTMDDVL- 59

HC2-48_Tlp3 PVLNAYKLNGDNNFFSYK----------------------LNNEER-----LGACTKVF- 259

35925B2_Tlp3 PVLNAYKTHGDYNFFTYG----------------------LDGKER-----LGTCAKVF- 270

14980A_Tlp3 PVLNAYKTHGDYNFFTYG----------------------LDGKER-----LGTCTKVF- 270

00-1597_Tlp3b PVLNAYKAHGDNNFFSYK----------------------LNNEER-----LGACTKVF- 259

ICDCCJ07001_Tlp3 PVSNAYKAHGDNNFFSYK----------------------LNNEER-----LGACTKVF- 259

RM3196_Tlp3 PVSNAYKAHGDNNFFSYK----------------------LNNEER-----LGACTKVF- 259

RM1875_Tlp3 PVLNAYKTHGDHNFFNYG----------------------LDGKER-----LGACTKVF- 259

CF2-75_Tlp3 PVLNAYKLNGDNNFFSYK----------------------LNNEER-----LGACTKVF- 270

RM5611_Tlp3 PVLNAYKLNGDNNFFSYK----------------------LNNEER-----LGACTKVF- 259

MTVDSCj16_Tlp3 PVLNAYKAHGDNNFFSYK----------------------LNNEER-----LGACTKVF- 259

01-1512_Tlp3 PVLNAYKLNGDNNFFSYK----------------------LNNEER-----LGACTKVF- 259

MTVDSCj13_Tlp3 PVLNAYKLNGDNNFFSYK----------------------LNNEER-----LGACTKVF- 259

32488_Tlp3a PVLNAYKLNGDNNFFSYK----------------------LNNEER-----LGACTKVF- 259

81116_Tlp3 PVLNAYKLNGDNNFFSYK----------------------LNNEER-----LGACTKVF- 259

32488_Tlp3b PVLNAYKLNGDNNFFSYK----------------------LNNEER-----LGACTKVF- 259

FB1_Tlp3 PVLNAYKTHGDNNFFSYK----------------------LNNEER-----LGACTKVF- 259

PT14_Tlp3 PVLNAYKAHGDNNFFSYK----------------------LNNEER-----LGACTKVF- 259

00-6200_Tlp3a PVLNAYKLNGDNNFFSYK----------------------LNNEER-----LGACTKVF- 259

RM1221_Tlp3 PVLNAYKLNGDNNFFSYK----------------------LNNEER-----LGACTKVF- 259

S3_Tlp3 PVLNAYKLNGDNNFFSYK----------------------LNNEER-----LGACTKVF- 259

FDAARGOS_421_Tlp3 PVLNAYKLNGDNNFFSYK----------------------LNNEER-----LGACTKVF- 270

CFSAN032806_Tlp3 PVLNAYKLNGDNNFFSYK----------------------LNNEER-----LGACTKVF- 270

IA3901_Tlp3b PVLNAYKLNGDNNFFSYK----------------------LNNEER-----LGACTKVF- 259

00-6200_Tlp3b PVLNAYKLNGDNNFFSYK----------------------LNNEER-----LGACTKVF- 259

BCW_6290_Tlp3b PVLNAYKLNGDNNFFSYK----------------------LNNEER-----LGACTKVF- 259

00-2425_Tlp3a PVLNAYKLNGDNNFFSYK----------------------LNNEER-----LGACTKVF- 259

00-2425_Tlp3b PVLNAYKLNGDNNFFSYK----------------------LNNEER-----LGACTKVF- 259

YH001_Tlp3a PVLNAYKLNGDNNFFSYK----------------------LNNEER-----LGACTKVF- 259

YH001_Tlp3b PVLNAYKLNGDNNFFSYK----------------------LNNEER-----LGACTKVF- 259

00-0949_Tlp3 PVLNAYKLNGDNNFFSYK----------------------LNNEER-----LGACTKVF- 259

NCTC11168_Tlp3 PVLNAYKLNGDNNFFSYK----------------------LNNEER-----LGACTKVF- 270

F38011_Tlp3 PVLNAYKLNGDNNFFSYK----------------------LNNEER-----LGACTKVF- 259

RM1285_Tlp3 PVLNAYKLNGDNNFFSYK----------------------LNNEER-----LGACTKVF- 259

FDAARGOS_422_Tlp3 PVLNAYKLNGDNNFFSYK----------------------LNNEER-----LGACTKVF- 270

MTVDSCj07_Tlp3 PVLNAYKLNGDNNFFSYK----------------------LNNEER-----LGACTKVF- 259

IA3901_Tlp3a PVLNAYKLNGDNNFFSYK----------------------LNNEER-----LGACTKVF- 259

BCW_6290_Tlp3a PVLNAYKLNGDNNFFSYK----------------------LNNEER-----LGACTKVF- 259

T1-21_Tlp19a PVLNAYKTHGDYNFFTYG----------------------LDGKER-----LGTCTKVF- 259

CJ677CC012_Tlp3 PVLNAYKTHGDYNFFTYG----------------------LDGKER-----LGTCTKVF- 259

CJM1cam_Tlp3 PVLNAYKTHGDYNFFTYG----------------------LDGKER-----LGTCTKVF- 259

M1_Tlp3 PVLNAYKTHGDYNFFTYG----------------------LDGKER-----LGTCTKVF- 259

4031_Tlp3 PVLNAYKTHGDYNFFTYG----------------------LDGKER-----LGTCTKVF- 259

R14_Tlp3 PVLNAYKTHGDYNFFTYG----------------------LDGKER-----LGTCTKVF- 259

FDAARGOS_295_Tlp21 -ITKIINQNTALLNAK---TPFVVDNGEGEQYEAF-------------------CRNVVS 260

FORC_046_Tlp4 -IANAIKANPALIDPRNQDTLFTAKDHQGVDYAIM-------------------CNSAFN 272

FDAARGOS_422_Tlp4 -IANAIKANPALIDPRNQDTLFTAKDHQGVDYAIM-------------------CNSAFN 272

ICDCCJ07001_Tlp4 -IANAIKANPALIDPRNQDTLFTAKDHQGVDYAIM-------------------CNSAFN 272

RM3196_Tlp4 -IANAIKANPALIDPRNQDTLFTAKDHQGVDYAIM-------------------CNSAFN 272

T1-21_Tlp4 -IANAIKANPALIDPRNQDTLFTAKDHQGVDYAIM-------------------CNSAFN 272

F38011_Tlp4 -IANAIKANPALIDPRNQDTLFTAKDHQGVDYAIM-------------------CNSAFN 272

HF5-4A-4_Tlp22 -IANAIKANPALIDPRNQDTLFTAKDHQGVDYAIM-------------------CNSAFN 218

00-0949_Tlp4 -IANAIKANPALIDPRNQDTLFTAKDHQGVDYAIM-------------------CNSAFN 272

01-1512_Tlp4 -IANAIKANPALIDPRNQDTLFTAKDHQGVDYAIM-------------------CNSAFN 272

81-176_Tlp4 -IANAIKANPALIDPRNQDTLFTAKDHQGVDYAIM-------------------CNSAFN 272

32488_Tlp4 -IANAIKANPALIDPRNQDTLFTAKDHQGVDYAIM-------------------CNSAFN 272

NCTC11168_Tlp4 -IANAIKANPALIDPRNQDTLFTAKDHQGVDYAIM-------------------CNSAFN 272

CFSAN032806_Tlp4 -IANAIKANPALIDPRNQDTLFTAKDHQGVDYAIM-------------------CNSAFN 272

81116_Tlp4 -IANAIKANPALIDPRNQDTLFTAKDHQGVDYAIM-------------------CNSAFN 272

RM1285_Tlp12 -IANAIKENPEYIDLNKRDILFPVFDDKGIKYEAM-------------------CDTSSN 269

PT14_Tlp12 -IANAIKENPEYIDLNKRDILFPVFDDKGIKYEAM-------------------CDTSSN 269

MTVJDCj07_Tlp12 -IANAIKENPEYIDLNKRDILFPVFDDKGIKYEAM-------------------CDTSSN 269

RM1221_Tlp12 -IANAIKENPEYIDPNKRDILFPVFDDKGIKYETM-------------------CDTSSN 269

FDAARGOS_421_Tlp12 -IANAIKENPEYIDPNKRDILFPVFDDKGIKYETM-------------------CDTSSN 269

35925B2_Tlp12 -IANAIKENPKYIDLNKRDILFPVFDDKGIKYETM-------------------CDTSSN 269

CJM1cam_Tlp12 -IANTIKENPEYIDLNKRDILFPVFDDKGIKYEAM-------------------CDTSSN 269

M1_Tlp12 -IANTIKENPEYIDLNKRDILFPVFDDKGIKYEAM-------------------CDTSSN 269

S3_Tlp12 -IANAIKENPEYIDPNKRDILFPVFDDKGIKYETM-------------------CDTSSN 269

00-1597_Tlp12 -IANAIKENPEYIDPNKRDILFPVFDDKGIKYETM-------------------CDTSSN 269

R14_Tlp12 -IANAIKENPEYIDPNKRDILFPVFDDKGIKYETM-------------------CDTSSN 269

RM1875_Tlp15 ------------------------------------SYLIPNADDLWIS--TGVYKDTLE 190

YH503_Tlp16 ------------------------------------SYLIPNADDLWIS--TGVYKDTLE 190

FB1_Tlp16 ------------------------------------SYLIPNADDLWIS--TGVYKDTLE 190

BFR-CA-9557_Tlp16 ------------------------------------SYLIPNADDLWIS--TGVYKDTLE 189

15-537360_Tlp16 ------------------------------------SYLIPNADDLWIS--TGVYKDTLE 190

OR12_Tlp16 ------------------------------------SYLIPNADDLWIS--TGVYKDTLE 190

YH502_Tlp16 ------------------------------------SYLIPNADDLWIS--TGVYKDTLE 190

14903A_Tlp16 ------------------------------------SYLIPNTDDLWIS--TGVYKDTLE 190

HC2-48_Tlp16 ------------------------------------SYLIPNADDLWIS--TGVYKDTLE 190

RM5611_Tlp16 ------------------------------------SYLIPNADDLWIS--TGVYKDTLE 190

00-2425_Tlp11 LIIDAVKNHKDLIIDNY------VDLSGNLSYAGVASFSTLGDSSHWSMVVTAPKKSIFA 328

00-6200_Tlp11 LIIDAVKNHKDLIIDNY------VDLSGNLSYAGVASFSTLGDSSHWSMVVTAPKKSIFA 328

YH001_Tlp11 LIIDAVKNHKDLIIDNY------VDLSGNLSYAGVASFSTLGDSSHWSMVVTAPKKSIFA 328

IA3902_Tlp11 LIIDAVKNHKDLIIDNY------VDLSGNLSYAGVASFSTLGDSSHWSMVVTAPKKSIFA 328

BCW_6290_Tlp11 LIIDAVKNHKDLIIDNY------VDLSGNLSYAGVASFSTLGDSSHWSMVVTAPKKSIFA 328

76339_Tlp18 LITDLIKEHKDALIDNY------VASTGDLSYASVVSFNTLGDSSRWSMIVTAPKKSALE 327

4031_Tlp17 LVNNAVKEHKTVIIDDY------VASTGDLSYASVSSFSTANNSSHWSMVVTAPKNSVLA 328

MTVDSCj13_Tlp13 LIVEAVKNHKDEILDNY------IASTGDLSYASISSFSTLGNSSHWSVIVTAPKKSVLA 327

RM1875_Tlp13 LIVEAVKNHKDEILDNY------IASTGDPSYASISSFSTLGNSSHWSVIVTTPKKSVLA 327

CF2-75_Tlp13 LIVEAVKNHKDEILDNY------IASTGDPSYASISSFSTLGNSSHWSVIVTAPKKSVLA 327

15-537360_Tlp13 LIVEAVKNHKDEILDNY------IASTGDLSYASISSFSTLGNSSHWSVIVTAPKKSVLA 327

CVMN29710_Tlp13 LIVEAVKNHKDEILDNY------IASTGDLSYASISSFSTLGNSSHWSVIVTAPKKSVLA 327

FB1_Tlp13 LIVEAVKNHKDEILDNY------IASTGDLSYASISSFSTLGNSSHWSVIVTAPKKSVLA 327

CFCAN032805_Tlp13 LIVEAVKNHKDEILDNY------IASTGDLSYASISSFSTLGNSSHWSVIVTAPKKSVLA 327

BG2108_Tlp13 LIVEAVKNHKDEILDNY------IASTGDLSYASISSFSTLGNSSHWSVIVTAPKKSVLA 327

YF2105_Tlp13 LIVEAVKNHKDEILDNY------IASTGDLSYASISSFSTLGNSSHWSVIVTAPKKSVLA 327

YH503_Tlp13 LIVEAVKNHKDEILDNY------IASTGDLSYASISSFSTLGNSSHWSVIVTAPKKSVLA 327

BFRCA9557_Tlp13 LIVEAVKNHKDEILDNY------IASTGDLSYASISSFSTLGNSSHWSVIVTAPKKSVLA 327

YH502_Tlp13 LIVEAVKNHKDEILDNY------IASTGDLSYASISSFSTLGNSSHWSVIVTAPKKSVLA 327

OR12_Tlp13 LIVEAVKNHKDEILDNY------IASTGDLSYASISSFSTLGNSSHWSVIVTAPKKSVLA 327

00-1597_Tlp13 LIVEAVKNHKDEILDNY------IASTGDLSYASISSFSTLGNSSHWSVIVTAPKKSVLA 327

14903A_Tlp13 LIVEAVKNHKDEILDNY------IASTGDLSYASISSFSTLGNSSHWSVIVTAPKKSVLA 327

FJ3124_Tlp13 LIVEAVKNHKDEILDNY------IASTGDLSYASISSFSTLGNSSHWSVIVTAPKKSVLA 327

R14_Tlp13 LIVEAVKNHKDEILDNY------IASTGDLSYASISSFSTLGNSSHWSVIVTAPKKSVLA 327

MTVDSCj16_Tlp13 LIVEAVKNHKDEILDNY------IASTGDLSYASISSFSTLGNSSHWSVIVTAPKKSVLA 327

14980A_Tlp13 LIVEAVKNHKDEILDNY------IASTGDLSYASISSFSTLGNSSHWSVIVTAPKKSVLA 327

FDAARGOS_295_Tlp1 PLKKLQ----------------LIIIGASFIFIFVVLGVVYYCVRKIVATRLPIILNSLE 366

14980A_Tlp1 PLKKLQ----------------MIIISASLIFIIVVLGVVYYCVRKIVAARLPIILNSLE 366

CJ677CC527_Tlp1 PLKKLQ----------------LIIISASLIFIFVVLGVVYYCVRKIVATRLPIILNSLE 366

CJ677CC012_Tlp1 PLKKLQ----------------LIIISASLIFIFVVLGVVYYCVRKIVATRLPIILNSLE 366

4031_Tlp1 PLKKLQ----------------LIIIGASFIFIFVVLGVVYYCVRKIVASRLPVILSSLE 366

81116_Tlp1 PLKKLQ----------------LIIIGASFIFIFVVLGVVYYCVRKIVASRLPVILSSLE 366

35925B2_Tlp1 PLKKLQ----------------LIIIGASFIFIFVVLGVVYYCVRKIVASRLPVILSSLE 366

M1_Tlp1 PLKKLQ----------------LIIIGASFIFIFVVLGVVYYCVRKIVASRLPVILSSLE 366

PT14_Tlp1 PLKKLQ----------------LIIIGASFIFIFVVLGVVYYCVRKIVASRLPVILSSLE 366

81-176_Tlp1 PLKKLQ----------------LIILGASFIFIFVVLGVVYYCVRKIVASRLPVILSSLE 366

CVMN29710_Tlp1 PLKKLQ----------------LIILGASFIFIFVVLGVVYYCVRKIVASRLPVILSSLE 366

FB1_Tlp1 PLKKLQ----------------LIILGASFIFIFVVLGVVYYCVRKIVASRLPVILSSLE 366

BG2108_Tlp1 PLKKLQ----------------LIILGASFIFIFVVLGVVYYCVRKIVASRLPVILSSLE 366

YF2108_Tlp1 PLKKLQ----------------LIILGASFIFIFVVLGVVYYCVRKIVASRLPVILSSLE 366

YH501_Tlp1 PLKKLQ----------------LIILGASFIFIFVVLGVVYYCVRKIVASRLPVILSSLE 366

RM4661_Tlp1 PLKKLQ----------------LIILGASFIFIFVVLGVVYYCVRKIVASRLPVILSSLE 366

F38011_Tlp1 PLKKLQ----------------LIILGASFIFIFVVLGVVYYCVRKIVASRLPVILSSLE 366

T1-21_Tlp1 PLKKLQ----------------LIILGASFIFIFVVLGVVYYCVRKIVASRLPVILSSLE 366

CG8421_Tlp1 PLKKLQ----------------LIILGASFIFIFVVLGVVYYCVRKIVASRLPVILSSLE 366

CJM1cam_Tlp1 PLKKLQ----------------LIILGASFIFIFVVLGVVYYCVRKIVASRLPVILSSLE 366

R14_Tlp1 PLKKLQ----------------LIILGASFIFIFVVLGVVYYCVRKIVASRLPVILSSLE 366

ICDCCJ07001_Tlp1 PLKKLQ----------------LIILGASFIFIFVVLGVVYYCVRKIVASRLPVILSSLE 366

RM3196_Tlp1 PLKKLQ----------------LIILGASFIFIFVVLGVVYYCVRKIVASRLPVILSSLE 366

NCTC11168_Tlp1 PLKKLQ----------------LIILGASFIFIFVVLGVVYYCVRKIVASRLPVILSSLE 366

00-2425_Tlp1 PLKKLQ----------------LIILGASFIFIFVVLGVVYYCVRKIVASRLPVILSSLE 366

IA3902_Tlp1 PLKKLQ----------------LIILGASFIFIFVVLGVVYYCVRKIVASRLPVILSSLE 366

RM1285_Tlp1 PLKKLQ----------------LIILGASFIFIFVVLGVVYYCVRKIVASRLPVILSSLE 366

00-0949_Tlp1 PLKKLQ----------------LIILGASFIFIFVVLGVVYYCVRKIVASRLPVILSSLE 366

01-1512_Tlp1 PLKKLQ----------------LIILGASFIFIFVVLGVVYYCVRKIVASRLPVILSSLE 366

FDAARGOS_422_Tlp1 PLKKLQ----------------LIILGASFIFIFVVLGVVYYCVRKIVASRLPVILSSLE 366

FORC_056_Tlp1 PLKKLQ----------------LIILGASFIFIFVVLGVVYYCVRKIVASRLPVILSSLE 366

32488_Tlp1 PLKKLQ----------------LIILGASFIFIFVVLGVVYYCVRKIVASRLPVILSSLE 366

CFSAN032806_Tlp1 PLKKLQ----------------LIILGASFIFIFVVLGVVYYCVRKIVASRLPVILSSLE 366

YH001_Tlp1 PLKKLQ----------------LIILGASFIFIFVVLGVVYYCVRKIVASRLPVILSSLE 366

00-6200_Tlp1 PLKKLQ----------------LIILGASFIFIFVVLGVVYYCVRKIVASRLPVILSSLE 366

RM1221_Tlp1 PLKKLQ----------------LIILGASFIFIFVVLGVVYYCVRKIVASRLPVILSSLE 366

S3_Tlp1 PLKKLQ----------------LIILGASFIFIFVVLGVVYYCVRKIVASRLPVILSSLE 366

FDAARGOS_421_Tlp1 PLKKLQ----------------LIILGASFIFIFVVLGVVYYCVRKIVASRLPVILSSLE 366

FJ3124_Tlp1 PLKKLQ----------------LIILGASFIFIFVVLGVVYYCVRKIVASRLPVILSSLE 366

00-1597_Tlp1 PLKKLQ----------------LIILGASFIFIFVVLGVVYYCVRKIVASRLPVILSSLE 366

15-537360_Tlp20 -EYTACVGEPIDYIEEPVFKIAYIQIAIVIITSIISVLLLYFIVSR-YLSPLAAIQTGLT 325

76339_Tlp20 -EYTACVGEPIDYIEEPVFKIAYIQIAIVIITSIISVLLLYFIVSR-YLSPLASIQAGLN 325

HC2-48_Tlp20 -EYTACVGESIDYIEEPVFKIAYIQIAIVIITSIISVLLLYFIVSR-YLSPLASIQVGLN 325

CFSAN032805_Tlp20 -EYTACVGEPIDYIEEPVFKIAYIQIAIVIITSIISVLLLYFIVSR-YLSPLAAIQTGLT 324

CVMN29710_Tlp20 -EYTACVGEPIDYIEEPVFKIAYIQIAIVIITSIISVLLLYFIVSR-YLSPLAAIQTGLT 323

YH501_Tlp20 -EYTACVGEPIDYIEEPVFKIAYIQIAIVIITSIISVLLLYFIVSR-YLSPLAAIQTGLT 327

CF2-75_Tlp20 -EYTACVGESIDYIEEPVFKIAYIQIAIVIITSIISVLLLYFIVSR-YLSPLASIQVGLN 328

CO2-160_Tlp20 -EYTACVGESIDYIEEPVFKIAYIQIAIVIITSIISVLLLYFIVSR-YLSPLASIQVGLN 325

CO2-160_Tlp20b -EYTACVGESIDYIEEPVFKIAYIQIAIVIITSIISVLLLYFIVSR-YLSPLASIQVGLN 325

RM5611_Tlp20 -EYTACVGESIDYIEEPVFKIAYIQIAIVIITSIISVLLLYFIVSR-YLSPLASIQVGLN 325

14903A_Tlp20 -EYTACVGEPIDYIEEPVFKIAYIQIAIVIITSIISVLLLYFIVSR-YLSPLASIQVGLN 327

YH502_Tlp20 -EYTACVGEPIDYIEEPVFKIAYIQIAIVIITSIISVLLLYFIVSR-YLSPLASIQVGLN 324

RM3196_Tlp23 -IYTACVAKPIEQIEAPVYKAAFIQAIVVIIVVVFSVILLYFIVSK-YLSPLAAIQTGLT 325

ICDCCJ07001_Tlp23 -IYTACVAKPIEQIEAPVYKAAFIQAIVVIIVVVFSVILLYFIVIK-YLSPLAAIQTGLT 325

RM1285_Tlp2 -IYTACVGEPIEQIEAPVYKIAFIQTAIVIFTSIISVILLYFIVSK-YLSPLAAIQTGLT 325

CFSAN032806_Tlp2 -IYTACVGEPIEQIEAPVYKIAFIQTVIVVFASILSVILLYFIVSK-YLSPLAAIQTGLT 325

RM1221_Tlp2 -IYTACVGEPIEQIEAPVYKIAFIQTVIVVFASILSVILLYFIVSK-YLSPLAAIQTGLT 325

S3_Tlp2 -IYTACVGEPIEQIEAPVYKIAFIQTVIVVFASILSVILLYFIVSK-YLSPLAAIQTGLT 325

FDAARGOS_422_Tlp2 -IYTACVGEPIEQIEAPVYKIAFIQTAIVIFTSIISVILLYFIVSK-YLSPLAAIQTGLT 325

81-176_Tlp2 -IYTACVGEPIEQIEAPVYKIAFIQTAIVIFTSIISVILLYFIVSK-YLSPLAAIQTGLT 325

F38011_Tlp2 -IYTACVGEPIEQIEAPVYKIAFIQTAIVIFTSIISVILLYFIVSK-YLSPLAAIQTGLT 325

NCTC11168_Tlp2 -IYTACVGEPIEQIEAPVYKIAFIQTAIVIFTSIISVILLYFIVSK-YLSPLAAIQTGLT 325

MTVDSCj07_Tlp2 -IYTACVGEPIEQIEAPVYKIAFIQTAIVIFTSIISVILLYFIVSK-YLSPLAAIQTGLT 325

CJM1cam_Tlp24 -VYTACVGEPIEQIEAPVYKIAFIQTAIVIFTSIISVILLYFIVSK-YLSPLAAIQTGLT 325

M1_Tlp24 -VYTACVGEPIEQIEAPVYKIAFIQTAIVIFTSIISVILLYFIVSK-YLSPLAAIQTGLT 325

81116_Tlp2 -VYTACVGEPIEQIEAPVYKIAFIQTAIVIFTSIISVILLYFIVSK-YLSPLAAIQTGLT 325

4031_Tlp23 -AYTACVGEPIEQIEAPVYKIAFIQAIVVIIVVVFSVILLYFIVSK-YLSPLAAIQTGLT 324

CVMN29710_Tlp14 -TYTACITESVDVINEPIFKAVYIQVIALIIMISISIILLYFIVSK-YLSPLAAIQTGLT 322

RM4661_Tlp14 -TYTACITESVDVINEPIFKAVYIQVIALIIMISISIILLYFIVSK-YLSPLAAIQTGLT 322

MG1116_Tlp14 -AYTACITESTDVINKPIFKAAYIQVIALIVMISISIILLYFIVSK-YLSPLATI----- 317

BG2108_Tlp14 -AYTACITESTDVINKPIFKAAYIQVIALIIMISISIILLYFIVSK-YLSPLAAI----- 317

YF2105_Tlp14 -AYTACITESTDVINKPIFKAAYIQVIALIIMISISIILLYFIVSK-YLSPLAAI----- 317

YH502_Tlp14 -AYTACITESADIINKPIFKAAYIQVIALIVMISISIILLYFIVSK-YLSPLAAIQTGLT 333

WA333_Tlp14 -AYTACITESTDVINKPIFKAAYIQVIALIVMISISIILLYFIVSK-YLSPLAA------ 316

BP3181_Tlp14 -AYTACITESADIINKPIFKAAYIQVIALIVMISISIILLYFIVSK-YLSPLAAIQTGLT 322

ZV1224_Tlp14a -AYTACITESTDVINKPIFKAAYIQVIALIIMISISIILLYFIVSK-YLSPLAAIQTGLT 322

ZV1224_Tlp14b -AYTACITESTDVINKPIFKAAYIQVIALIIMISISIILLYFIVSK-YLSPLAAIQTGLT 322

YH503_Tlp14 -AYTACITESTDVINKPIFKAAYIQVIALIIMISISIILLYFIVSK-YLSPLAAIQTGLT 333

14903A_Tlp14 -AYTACITESTDVINKPIFKAAYIQVIALIVMISISIILLYFIVSK-YLSPLAAIQTGLT 322

OR12_Tlp14 -AYTACITESTDVINKPIFKAAYIQVIALIIMISISIILLYFIVSK-YLSPLAAIQTGLT 322

CFSAN032805_Tlp14 -AYTACITESTDVINKPIFKAAYIQVIALIVMISISIILLYFIVSK-YLSPLAAIQTGLT 333

BFR-CA-9557_Tlp14 -AYTACITESTDVINKPIFKAAYIQVIALIIMISISIILLYFIVSK-YLSPLAAIQTGLT 322

15-537360_Tlp14 -AYTACITESTDVINKPIFKAAYIQVIALIVMISISIILLYFIVSK-YLSPLAAIQTGLT 322

YH501_Tlp14 -AYTACITESTDVINKPIFKAAYIQVIALIIMISISIILLYFIVSK-YLSPLAAIQTGLT 322

T1-21_Tlp19b -APLPTCS----------------------------------------------YPNRFN 277

CG8421_Tlp14 -AYTACITESADIINKPIFKAAYIQVIALIVMISISI------VSK-YLSPLAAIQTGLT 316

MTVDSCj16_Tlp14 -AYTACITESADIINKPIYKAAFIQVIALIVMISISIILLYFIVSK-YLSPLAAIQTGLT 322

01-1512_Tlp14 -AYTACITESADIINKPIYKAAFIQVIALIVMISISIILLYFIVSK-YLSPLAAIQTGLT 322

00-0949_Tlp14 -AYTACITESADIINKPIYKAAFIQVIALIVMISISIILLYFIVSK-YLSPLAAIQTGLT 322

MTVDSCj13_Tlp14 -AYTACITESADIINKPIFKAAYIQVIALIVMISISIILLYFIVSK-YLSPLAAIQTGLT 322

S3_Tlp14 -AYTACITESADIINKPIFKAAYIQVIALIVMISISIILLYFIVSK-YLSPLAAIQTGLT 322

PT14_Tlp14 -AYTACITESADIINKPIFKAAYIQVIALIVMISISIILLYFIVSK-YLSPLAAIQTGLT 322

14980A_Tlp14 -AYTACITESADIINKPIFKAAYIQVIALIVMISISIILLYFIVSK-YLSPLAAIQTGLT 338

FJ3124_Tlp14 -AYTACITESADIINKPIFKAAYIQVIALIVMISISIILLYFIVSK-YLSPLAAIQTGLT 322

00-1597_Tlp14 -AYTACITESTDVINKPIFKAAYIQVIALIIMISISIILLYFIVSK-YLSPLAAIQTGLT 322

R14_Tlp14 -AYTACITESADIINKPIFKAAYIQVIALIVMISISIILLYFIVSK-YLSPLAAIQTGLT 322

CG8421_Tlp25 -MI----NIKRFRYKKSCYSILMKQVIVGIIAIIIALILIRFLISR-SLSPLAAIQTGLT 113

HC2-48_Tlp3 -AYTACITESADIINKPIYKAAFIQAIVVIIVVVFSVILLYFIVSK-YLSPLAAIQTGLT 317

35925B2_Tlp3 -AYTACITESADIINKPIHKAAFIQAIVVIIVVVFSVILLYFIISK-YLSPLAAIQTGLT 328

14980A_Tlp3 -AYTACITESADIINKPIHKAAFIQAIVVIIVVVFSVILLYFIVSK-YLSPLAAIQTGLT 328

00-1597_Tlp3b -AYTACITESADIINKPIFKAAYIQVIALIVMISISIILLYFIVSK-YLSPLAAIQTGLT 317

ICDCCJ07001_Tlp3 -AYTACITESADIINKPIYKAAFIQAIVVIIVVVFSVILLYFIVSK-YLSPLAAIQTGLT 317

RM3196_Tlp3 -AYTACITESADIINKPIYKAAFIQAIVVIIVVVFSVILLYFIVSK-YLSPLAAIQTGLT 317

RM1875_Tlp3 -AYTACITESADIINKPIYKAAFIQAIVVIIVVVFSVILLYFIVSK-YLSPLAAIQTGLT 317

CF2-75_Tlp3 -AYTACITESADIINKPIYKAAFIQAIVVIIVVVFSVILLYFIVSK-YLSPLAAIQTGLT 328

RM5611_Tlp3 -AYTACITESADIINKPIYKAAFIQAIVVIIVVVFSVILLYFIVSK-YLSPLAAIQTGLT 317

MTVDSCj16_Tlp3 -AYTACITESADIINKPIYKAAFIQVIALIVMISISIILLYFIVSK-YLSPLAAIQTGLT 317

01-1512_Tlp3 -AYTACITESADIINKPIYKAAFIQVIALIVMISISIILLYFIVSK-YLSPLAAIQTGLT 317

MTVDSCj13_Tlp3 -AYTACITESADIINKPIFKAAYIQVIALIVMISISIILLYFIVSK-YLSPLAAIQTGLT 317

32488_Tlp3a -AYTACITESADIINKPIFKAAYIQVIALIVMISISIILLYFIVSK-YLSPLAAIQTGLT 317

81116_Tlp3 -AYTACITESADIINKPIFKAAYIQVIALIVMISISIILLYFIVSK-YLSPLAAIQTGLT 317

32488_Tlp3b -AYTACITESADIINKPIFKAAYIQVIALIVMISISIILLYFIVSK-YLSPLAAIQTGLT 317

FB1_Tlp3 -AYTACITESADIINKPIYKAAFIQAIVVIIVVVFSVILLYFIVSK-YLSPLAAIQTGLT 317

PT14_Tlp3 -AYTACITESADIINKPIYKAAFIQAIVVIIVVVFSVILLYFIVSK-YLSPLAAIQTGLT 317

00-6200_Tlp3a -AYTACITESADIINKPIYKAAFIQAIVVIIVVVFSVILLYFIVSK-YLSPLAAIQTGLT 317

RM1221_Tlp3 -AYTACITESADIINKPIFKAAFIQAIVVIIVVVFSVILLYFIVSK-YLSPLAAIQTGLT 317

S3_Tlp3 -AYTACITESADIINKPIFKAAFIQAIVVIIVVVFSVILLYFIVSK-YLSPLAAIQTGLT 317

FDAARGOS_421_Tlp3 -AYTACITESADIINKPIFKAAFIQAIVVIIVVVFSVILLYFIVSK-YLSPLAAIQTGLT 328

CFSAN032806_Tlp3 -AYTACITESADIINKPIYKAAFIQAIVVIIVVVFSVILLYFIVSK-YLSPLAAIQTGLT 328

IA3901_Tlp3b -AYTACITESADIINKPIYKAAFIQAIVVIIVVVFSVILLYFIVSK-YLSPLAAIQTGLT 317

00-6200_Tlp3b -AYTACITESADIINKPIYKAAFIQAIVVIIVVVFSVILLYFIVSK-YLSPLAAIQTGLT 317

BCW_6290_Tlp3b -AYTACITESADIINKPIYKAAFIQAIVVIIVVVFSVILLYFIVSK-YLSPLAAIQTGLT 317

00-2425_Tlp3a -AYTACITESADIINKPIYKAAFIQAIVVIIVVVFSVILLYFIVSK-YLSPLAAIQTGLT 317

00-2425_Tlp3b -AYTACITESADIINKPIYKAAFIQAIVVIIVVVFSVILLYFIVSK-YLSPLAAIQTGLT 317

YH001_Tlp3a -AYTACITESADIINKPIYKAAFIQAIVVIIVVVFSVILLYFIVSK-YLSPLAAIQTGLT 317

YH001_Tlp3b -AYTACITESADIINKPIYKAAFIQAIVVIIVVVFSVILLYFIVSK-YLSPLAAIQTGLT 317

00-0949_Tlp3 -AYTACITESADIINKPIYKAAFIQAIVVIIVVVFSIILLYFIVSK-YLSPLAAIQTGLT 317

NCTC11168_Tlp3 -AYTACITESADIINKPIYKAAFIQAIVVIIVVVFSVILLYFIVSK-YLSPLAAIQTGLT 328

F38011_Tlp3 -AYTACITESADIINKPIYKAAFIQAIVVIIVVVFSVILLYFIVSK-YLSPLAAIQTGLT 317

RM1285_Tlp3 -AYTACITESADIINKPIYKAAFIQAIVVIIVVVFSVILLYFIVSK-YLSPLAAIQTGLT 317

FDAARGOS_422_Tlp3 -AYTACITESADIINKPIYKAAFIQAIVVIIVVVFSVILLYFIVSK-YLSPLAAIQTGLT 328

MTVDSCj07_Tlp3 -AYTACITESADIINKPIYKAAFIQAIVVIIVVVFSVILLYFIVSK-YLSPLAAIQTGLT 317

IA3901_Tlp3a -AYTACITESADIINKPIYKAAFIQAIVVIIVVVFSVILLYFIVSK-YLSPLAAIQTGLT 317

BCW_6290_Tlp3a -AYTACITESADIINKPIYKAAFIQAIVVIIVVVFSVILLYFIVSK-YLSPLAAIQTGLT 317

T1-21_Tlp19a -APLPTCS----------------------------------------------YPNRFN 272

CJ677CC012_Tlp3 -AYTACITESADIINKPIHKAAFIQAIVVIIVVVFSVILLYFIVSK-YLSPLAAIQTGLT 317

CJM1cam_Tlp3 -AYTACITESADIINKPIHKAAFIQAIVVIIVVVFSVILLYFIVSK-YLSPLAAIQTGLT 317

M1_Tlp3 -AYTACITESADIINKPIHKAAFIQAIVVIIVVVFSVILLYFIVSK-YLSPLAAIQTGLT 317

4031_Tlp3 -AYTACITESADIINKPIHKAAFIQAIVVIIVVVFSVILLYFIVSK-YLSPLAAIQTGLT 317

R14_Tlp3 -AYTACITESADIINKPIHKAAFIQAIVVIIVVVFSVILLYFIVSK-YLSPLAAIQTGLT 317

FDAARGOS_295_Tlp21 DFYRMCTLTQSKIYSDMANEILFKQILIGIIAISVILLFIQLIIKK-YLSPLAAIQTGLT 319

FORC_046_Tlp4 PLFRICTITENKVYTEAVNSILMKQVIVGIIAIIIALILIRFLISR-SLSPLAAIQTGLT 331

FDAARGOS_422_Tlp4 PLFRICTITENKVYTEAVNSILMKQVIVGIIAIIIALILIRFLISR-SLSPLAAIQTGLT 331

ICDCCJ07001_Tlp4 PLFRICTITENKVYTEAVNSILMKQVIVGIIAIIIALILIRFLISR-SLSPLAAIQTGLT 331

RM3196_Tlp4 PLFRICTITENKVYTEAVNSILMKQVIVGIIAIIIALILIRFLISR-SLSPLAAIQTGLT 331

T1-21_Tlp4 PLFRICTITENKVYTEAVNSILMKQVIVGIIAIIIALILIRFLISR-SLSPLAAIQTGLT 331

F38011_Tlp4 PLFRICTITENKVYTEAVNSILMKQVIVGIIAIIIALILIRFLISR-SLSPLAAIQTGLT 331

HF5-4A-4_Tlp22 PLFRICTITENKVYTEAVNSILMKQVIVGIIAIIIALILIRFLISR-SLSPLAAIQTGLT 277

00-0949_Tlp4 PLFRICTITENKVYTEAVNSILMKQVIVGIIAIIIALILI-ILISR-SLSPLAAIQTGLT 330

01-1512_Tlp4 PLFRICTITENKVYTEAVNSILMKQVIVGIIAIIIALILIRFLISR-SLSPLAAIQTGLT 331

81-176_Tlp4 PLFRICTITENKVYTEAVNSILMKQVIVGIIAIIIALILIRFLISR-SLSPLAAIQTGLT 331

32488_Tlp4 PLFRICTITENKVYTEAVNSILMKQVIVGIIAIIIALILIRFLISR-SLSPLAAIQTGLT 331

NCTC11168_Tlp4 PLFRICTITENKVYTEAVNSILMKQVIVGIIAIIIALILIRFLISR-SLSPLAAIQTGLT 331

CFSAN032806_Tlp4 PLFRICTITENKVYTEAVNSILMKQVIVGIIAIIIALILIRFLISR-SLSPLAAIQTGLT 331

81116_Tlp4 PLFRICTITENKVYTEAVNSILMKQVIVGIIAIIIALILIRFLISR-SLSPLAAIQTGLT 331

RM1285_Tlp12 GLYRICAVTLDSNYTSAVNSILMKQVIVGIIAIIIALILIRFLISR-SLSPLAAIQTGLT 328

PT14_Tlp12 GLYRICAVTLDSNYTSAVNSILMKQVIVGIIAIIIALILIRFLISR-SLSPLAAIQTGLT 328

MTVJDCj07_Tlp12 GLYRICAVTLDSNYTSAVNSILMKQVIVGIIAIIIALILIRFLISR-SLSPLAAIQTGLT 328

RM1221_Tlp12 GLYRICAVTLDSNYTSAVNSILMKQVIVGIIAIIIALILIRFLISR-SLSPLAAIQTGLT 328

FDAARGOS_421_Tlp12 GLYRICAVTLDSNYTSAVNSILMKQVIVGIIAIIIALILIRFLISR-SLSPLAAIQTGLT 328

35925B2_Tlp12 GLYRICAVTLDSNYTSAVNSILMKQVIVGIIAIIIALILIRFLISR-SLSPLAAIQTGLT 328

CJM1cam_Tlp12 GLYRICAVTLDSNYTSAVNSILMKQVIVGIIAIIIALILIRFLISR-SLSPLAAIQTGLT 328

M1_Tlp12 GLYRICAVTLDSNYTSAVNSILMKQVIVGIIAIIIALILIRFLISR-SLSPLAAIQTGLT 328

S3_Tlp12 GLYRICAVTLDSNYTSAVNSILMKQAIVGIIAIIIALILIRFLISR-SLSPLAAIQTGLT 328

00-1597_Tlp12 GLYRICAVTLDSNYTSAVNSILMKQAIVGIIAIIIALILIRFLISR-SLSPLAAIQTGLT 328

R14_Tlp12 GLYRICAVTLDSNYTSAVNSILMKQVIVGIIAIIIALILIRFLISR-SLSPLAAIQTGLT 328

RM1875_Tlp15 PYIDGNL---KNCYHFFSKNFFKTIIFFTLFILIIIPFIFIFYRNLITG--VQGIKTNIT 245

YH503_Tlp16 PYIDRSL---EELLSFFSKSFFKTVLFSIIFILIIIPFIFIFYRNLIVG--VQGIDANIT 245

FB1_Tlp16 PYIDRSL---EELLSFFSKSFFKTVLFSIIFILIIIPFIFIFYRNLIVG--VQGIDANIT 245

BFR-CA-9557_Tlp16 PYIDRSL---EELLSFFSKSFFKTVLFSIIFILIIIPFIFIFYRNLIVG--VQGIDANIT 244

15-537360_Tlp16 PYIDRSL---EELLSFFSKSFFKTVLFSIIFILIIIPFIFIFYRNLIVG--VQGIDANIT 245

OR12_Tlp16 PYIDRSL---EELLSFFSKSFFKTVLFSIIFILIIIPFIFIFYRNLIVG--VQGIDANIT 245

YH502_Tlp16 PYIDRSL---EELLSFFSKSFFKTVLFSIIFILIIIPFIFIFYRNLIVG--VQGIDANIT 245

14903A_Tlp16 PYIDRSL---EELLSFFSKSFFKTVLFSIIFILIIIPFIFIFYRNLIVG--VQGIDANIT 245

HC2-48_Tlp16 PYIDRSL---EELLSFFSKSFFKTVLFSIIFILIIIPFIFIFYRNLIVG--VQGIDANIT 245

RM5611_Tlp16 PYIDRSL---EELLSFFSKSFFKTVLFSIIFILIIIPFIFIFYRNLIVG--VQGIDANIT 245

00-2425_Tlp11 PL--------------YELNFI--LISIAIIVLIAILIILYFCVKNIVGSKLPIIVNSLQ 372

00-6200_Tlp11 PL--------------YELNFI--LISIAIIVLIAILIILYFCVKNIVGSKLPIIVNSLQ 372

YH001_Tlp11 PL--------------YELNFI--LISIAIIVLIAILIILYFCVKNIVGSKLPIIVNSLQ 372

IA3902_Tlp11 PL--------------YELNFI--LISIAIIVLIAILIILYFCVKNIVGSKLPIIVNSLQ 372

BCW_6290_Tlp11 PL--------------YELNFI--LISIAIIVLIAILIILYFCVKNIVGSKLPIIVNSLQ 372

76339_Tlp18 PL--------------FRLQFA--IITTAIIALIVILFIVYFCVRKIVGIRIPVILKSLE 371

4031_Tlp17 PL--------------KKLEII--FIIISFFILLVILIIVYVCVKKIVGSRIPVILKSLE 372

MTVDSCj13_Tlp13 PL--------------YKLQYT--IISVAIIALIAILTVVYFFIRKIIGSRIPLILKSLE 371

RM1875_Tlp13 PL--------------YKLQYT--IISVAIIALIAILTVVYFFIRKIIGSRIPLILKSLE 371

CF2-75_Tlp13 PL--------------YKLQYT--IISVAIIALIAILTVVYFFIRKIIGSRIPLILKSLE 371

15-537360_Tlp13 PL--------------YKLQYI--IISVAIIALIAILAVVYFFIRKIIGSRIPLILKSLE 371

CVMN29710_Tlp13 PL--------------YKLQYI--IISVAIIALIAILAVVYFFIRKIIGSRIPLILKSLE 371

FB1_Tlp13 PL--------------YKLQYI--IISVAIIALIAILAVVYFFIRKIIGSRIPLILKSLE 371

CFCAN032805_Tlp13 PL--------------YKLQYI--IISVAIIALIAILAVVYFFIRKIIGSRIPLILKSLE 371

BG2108_Tlp13 PL--------------YKLQYI--IISVAIIALIAILAVVYFFIRKIIGSRIPLILKSLE 371

YF2105_Tlp13 PL--------------YKLQYI--IISVAIIALIAILAVVYFFIRKIIGSRIPLILKSLE 371

YH503_Tlp13 PL--------------YKLQYI--IISVAIIALIAILAVVYFFIRKIIGSRIPLILKSLE 371

BFRCA9557_Tlp13 PL--------------YKLQYI--IISVAIIALIAILAVVYFFIRKIIGSRIPLILKSLE 371

YH502_Tlp13 PL--------------YKLQYI--IISVAIIALIAILAVVYFFIRKIIGSRIPLILKSLE 371

OR12_Tlp13 PL--------------YKLQYI--IISVAIIALIAILAVVYFFIRKIIGSRIPLILKSLE 371

00-1597_Tlp13 PL--------------YKLQYI--IISVAIIALIAILAVVYFFIRKIIGSRIPLILKSLE 371

14903A_Tlp13 PL--------------YKLQYI--IISVAIIALIAILAVVYFFIRKIIGSRIPLILKSLE 371

FJ3124_Tlp13 PL--------------YKLQYI--IISVAIIALIAILAVVYFFIRKIIGSRIPLILKSLE 371

R14_Tlp13 PL--------------YKLQYI--IISVAIIALIAILAVVYFFIRKIIGSRIPLILKSLE 371

MTVDSCj16_Tlp13 PL--------------YKLQYI--IISVAIIALIAILAVVYFFIRKIIGSRIPLILKSLE 371

14980A_Tlp13 PL--------------YKLQYI--IISVAIIALIAILAVVYFFIRKIIGSRIPLILKSLE 371

FDAARGOS_295_Tlp1 SFFRFLNHEKIELKLIKIRANDELGAMGRIINENIEKIQMSLEQDQNAVDESVQTAREIE 426

14980A_Tlp1 SFFRFLNHEKIELKPIKIRANDELGAMGNIINENIKKIQLSLEQDQSAVDESVQTAKEIE 426

CJ677CC527_Tlp1 SFFRFLNHEKIELKLIKIRANDELGAMGRIINENIEKIQISLEQDQNAVDESVQTAREIE 426

CJ677CC012_Tlp1 SFFRFLNHEKIELKLIKIRANDELGAMGRIINENIEKIQISLEQDQNAVDESVQTAREIE 426

4031_Tlp1 SFFRFLNHEKIEPKAIEIRANDELGAMGRIINENIEKIQISLEQDQNAVDESVQTAREIE 426

81116_Tlp1 SFFRFLNHEKIEPKAIEIRANDELGAMGRIINENIEKIQISLEQDQNAVDESVQTAREIE 426

35925B2_Tlp1 SFFRFLNHEKIEPKAIEIRANDELGAMGRIINENIEKIQISLEQDQNAVDESVQTAREIE 426

M1_Tlp1 SFFRFLNHEKIEPKAIEIRANDELGAMGRIINENIEKIQISLEQDQNAVDESVQTAREIE 426

PT14_Tlp1 SFFRFLNHEKIEPKAIEIRANDELGAMGRIINENIEKIQISLEQDQNAVDESVQTAREIE 426

81-176_Tlp1 SFFRFLNHEKIEPKAIEIRANDELGAMGRIINENIEKIQISLEQDQNAVDESVQTAREIE 426

CVMN29710_Tlp1 SFFRFLNHEKIEPKAIEIRANDELGAMGRIINENIEKIQISLEQDQNAVDESVQTAREIE 426

FB1_Tlp1 SFFRFLNHEKIEPKAIEIRANDELGAMGRIINENIEKIQISLEQDQNAVDESVQTAREIE 426

BG2108_Tlp1 SFFRFLNHEKIEPKAIEIRANDELGAMGRIINENIEKIQISLEQDQNAVDESVQTAREIE 426

YF2108_Tlp1 SFFRFLNHEKIEPKAIEIRANDELGAMGRIINENIEKIQISLEQDQNAVDESVQTAREIE 426

YH501_Tlp1 SFFRFLNHEKIEPKAIEIRANDELGAMGRIINENIEKIQISLEQDQNAVDESVQTAREIE 426

RM4661_Tlp1 SFFRFLNHEKIEPKAIEIRANDELGAMGRIINENIEKIQISLEQDQNAVDESVQTAREIE 426

F38011_Tlp1 SFFRFLNHEKIEPKAIEIRANDELGAMGRIINENIEKIQISLEQDQNAVDESVQTAREIE 426

T1-21_Tlp1 SFFRFLNHEKIEPKAIEIRANDELGAMGRIINENIEKIQISLEQDQNAVDESVQTAREIE 426

CG8421_Tlp1 SFFRFLNHEKIEPKAIEIRANDELGAMGRIINENIEKIQISLEQDQNAVDESVQTAREIE 426

CJM1cam_Tlp1 SFFRFLNHEKIEPKAIEIRANDELGAMGRIINENIEKIQISLEQDQNAVDESVQTAREIE 426

R14_Tlp1 SFFRFLNHEKIEPKAIEIRANDELGAMGRIINENIEKIQISLEQDQNAVDESVQTAREIE 426

ICDCCJ07001_Tlp1 SFFRFLNHEKIEPKAIEIRANDELGAMGRIINENIEKIQISLEQDQNAVDESVQTAREIE 426

RM3196_Tlp1 SFFRFLNHEKIEPKAIEIRANDELGAMGRIINENIEKIQISLEQDQNAVDESVQTAREIE 426

NCTC11168_Tlp1 SFFRFLNHEKIEPKAIEIRANDELGAMGRIINENIEKIQISLEQDQNAVDESVQTAREIE 426

00-2425_Tlp1 SFFRFLNHEKIEPKAIEIRANDELGAMGRIINENIEKIQISLEQDQNAVDESVQTAREIE 426

IA3902_Tlp1 SFFRFLNHEKIEPKAIEIRANDELGAMGRIINENIEKIQISLEQDQNAVDESVQTAREIE 426

RM1285_Tlp1 SFFRFLNHEKIEPKAIEIRANDELGAMGRIINENIEKIQISLEQDQNAVDESVQTAREIE 426

00-0949_Tlp1 SFFRFLNHEKIEPKAIEIRANDELGAMGRIINENIEKIQISLEQDQNAVDESVQTAREIE 426

01-1512_Tlp1 SFFRFLNHEKIEPKAIEIRANDELGAMGRIINENIEKIQISLEQDQNAVDESVQTAREIE 426

FDAARGOS_422_Tlp1 SFFRFLNHEKIEPKAIEIRANDELGAMGRIINENIEKIQISLEQDQNAVDESVQTAREIE 426

FORC_056_Tlp1 SFFRFLNHEKIEPKAIEIRANDELGAMGRIINENIEKIQISLEQDQNAVDESVQTAREIE 426

32488_Tlp1 SFFRFLNHEKIEPKAIEIRANDELGAMGRIINENIEKIQISLEQDQNAVDESVQTAREIE 426

CFSAN032806_Tlp1 SFFRFLNHEKIEPKAIEIRANDELGAMGRIINENIEKIQISLEQDQNAVDESVQTAREIE 426

YH001_Tlp1 SFFRFLNHEKIEPKAIEIRANDELGAMGRIINENIEKIQISLEQDQNAVDESVQTVREIE 426

00-6200_Tlp1 SFFRFLNHEKIEPKAIEIRANDELGAMGRIINENIEKIQISLEQDQNAVDESVQTVREIE 426

RM1221_Tlp1 SFFRFLNHEKIEPKAIEIRANDELGAMGRIINENIEKIQISLEQDQNAVDESVQTAREIE 426

S3_Tlp1 SFFRFLNHEKIEPKAIEIRANDELGAMGRIINENIEKIQISLEQDQNAVDESVQTAREIE 426

FDAARGOS_421_Tlp1 SFFRFLNHEKIEPKAIEIRANDELGAMGRIINENIEKIQISLEQDQNAVDESVQTAREIE 426

FJ3124_Tlp1 SFFRFLNHEKIEPKAIEIRANDELGAMGRIINENIEKIQISLEQDQNAVDESVQTAREIE 426

00-1597_Tlp1 SFFRFLNHEKIEPKAIEIRANDELGAMGRIINENIEKIQISLEQDQNAVDESVQTAREIE 426

15-537360_Tlp20 SFFDFINHKTKNVSTIDVKTNDEFGQISKAINENILATKQGLEQDAKAVKESVETVGVVE 385

76339_Tlp20 SFFDFINHKTKDVSTIDVKTNDEFGQISKAINENILATKQGLEQDAKAVKESVETVGVVE 385

HC2-48_Tlp20 SFFDFINHN-KNVSTIDVKTNDEFGQISKAINENILATKQGLEQDAKAVKESVETVGVVE 384

CFSAN032805_Tlp20 SFFDFINHKTKNVSTIEIKSNDEFGQISKAINENILATKQGLEQDAKAVKESVETVGVVE 384

CVMN29710_Tlp20 SFFDFINHKTKNVSTIEIKTNDEFGQISKTINENILATKQGLEQDAKAVKESVETVGVVE 383

YH501_Tlp20 SFFDFINHKTKNVSTIEIKTNDEFGQISKTINENILATKQGLEQDAKAVKESVETVGVVE 387

CF2-75_Tlp20 SFFDFINHKTKNVSTIDVKTNDEFGQISKAINENILATKQGLEQDAKAVKESVETVGVVE 388

CO2-160_Tlp20 SFFDFINHKTKNVSTIDVKTNDEFGQISKAINENILATKQGLEQDAKAVKESVETVGVVE 385

CO2-160_Tlp20b SFFDFINHKTKNVSTIDVKTNDEFGQISKAINENILATKQGLEQDAKAVKESVETVGVVE 385

RM5611_Tlp20 SFFDFINHKTKNVSTIDVKTNDEFGQISKAINENILATKQGLEQDAKAVKESVETVGVVE 385

14903A_Tlp20 SFFDFINHKTKNVSTIEIKSNDEFGQISKAINENILATKQGLEQDAKAVKESVETVGVVE 387

YH502_Tlp20 SFFDFINHKTKNVSTIDVKTNDEFGQISKAINENILATKQGLEQDAKAVKESVETVGVVE 384

RM3196_Tlp23 SFFDFINHKTKNVSTIEVKSNDEFGQISNAINENILATKRGLEQDNQAVKESVETVHVVE 385

ICDCCJ07001_Tlp23 SFFDFINHKTKNVSTIEVKSNDEFGQISNAINENILATKRGLEQDNQAVKESVETVHVVE 385

RM1285_Tlp2 SFFDFINYKTKNVSTIEVKSNDEFGQISNAINKTFLLLK-EAEQDNQAVKESVQTVSVVE 384

CFSAN032806_Tlp2 SFFDFINYKTKNVSTIEVKSNDEFGQISNAINENILATKRGLEQDNQAVKESVQTVSVVE 385

RM1221_Tlp2 SFFDFINYKTKNVSTIEVKSNDEFGQISNAINENILATKRGLEQDNQAVKESVQTVSVVE 385

S3_Tlp2 SFFDFINYKTKNVSTIEVKSNDEFGQISNAINENILATKRGLEQDNQAVKESVQTVSVVE 385

FDAARGOS_422_Tlp2 SFFDFINYKTKNVSTIEVKSNDEFGQISNAINENILATKRGLEQDNQAVKESVQTVSVVE 385

81-176_Tlp2 SFFDFINYKTKNVSTIEVKSNDEFGQISNAINENILATKRGLEQDNQAVKESVQTVSVVE 385

F38011_Tlp2 SFFDFINYKTKNVSTIEVKSNDEFGQISNAINENILATKRGLEQDNQAVKESVQTVSVVE 385

NCTC11168_Tlp2 SFFDFINYKTKNVSTIEVKSNDEFGQISNAINENILATKRGLEQDNQAVKESVQTVSVVE 385

MTVDSCj07_Tlp2 SFFDFINYKTKNVSTIEVKSNDEFGQISNAINENILATKRGLEQDNQAVKESVQTVSVVE 385

CJM1cam_Tlp24 SFFDFINHKTKNVSTIEVKSNDEFGQISSAINENILATKRGLEQDNQAVKESVETVSVVE 385

M1_Tlp24 SFFDFINHKTKNVSTIEVKSNDEFGQISSAINENILATKRGLEQDNQAVKESVETVSVVE 385

81116_Tlp2 SFFDFINHKTKNVSTIEVKSNDEFGQISNAINENILATKRGLEQDNQAVKESVETVSVVE 385

4031_Tlp23 SFFDFINHKTKNVSTIEVKSNDEFGQISNAINENILATKRGLEQDNQAVKESVETVSVVE 384

CVMN29710_Tlp14 SFFDFINHKTKNVSTIEIKSNDEFGQISKTINENILATKQGLEQDAKAVKESVETVGVVE 382

RM4661_Tlp14 SFFDFINHKTKNVSTIDVKTNDEFGQISKAINENILATKQGLEQDAKAVKESVETVGVVE 382

MG1116_Tlp14 ---------TKNVSTIDIKTNDEFGQISKAINENILATKQGLEQDAKAVKESVETVGVVE 368

BG2108_Tlp14 ---------QT------------------------------------------------- 319

YF2105_Tlp14 ---------QTGLTSFFDFINH-----------------------------KTKNVSLLK 339

YH502_Tlp14 SFFDFINYKTKNVSTIEIKSNDEFGQISKTINENILATKQGLEQDAKAVKESVETVGVVE 393

WA333_Tlp14 -----INHKTKNVSTIEIKSNDEFGQISKTINENILATKQGLEQDAKAVKESVETVGVVE 371

BP3181_Tlp14 SFFDFINHKTKNVSTIEIKSNDEFGQISKTINENILATKQGLEQDAKAVKESVETVGVVE 382

ZV1224_Tlp14a SFFDFINHKTKNVSTIDVKTNDEFGQISKAINENILATKQGLEQDAKAVKESVETVGVVE 382

ZV1224_Tlp14b SFFDFINHKTKNVSTIDVKTNDEFGQISKAINENILATKQGLEQDAKAVKESVETVGVVE 382

YH503_Tlp14 SFFDFINHKTKNVSTIEIKTNDEFGQISKTINENILATKQGLEQDAKAVKESVETVGVVE 393

14903A_Tlp14 SFFDFINHKTKNVSTIEIKSNDEFGQISKTINENILATKQGLEQDAKAVKESVETVGVVE 382

OR12_Tlp14 SFFDFINHKTKNVSTIEIKTNDEFGQISKTINENILATKQGLEQDAKAVKESVETVGVVE 382

CFSAN032805_Tlp14 SFFDFINHKTKNVSTIDVKTNDEFGQISKTINENILATKQGLEQDAKAVKESVETVGVVE 393

BFR-CA-9557_Tlp14 SFFDFINHKTKNVSTIDVKTNDEFGQISKAINENILATKQGLEQDAKAVKESVETVGVVE 382

15-537360_Tlp14 SFFDFINHKTKNVSTIEIKSNDEFGQISKAINENILATKQGLEQDAKAVKESVETVGVVE 382

YH501_Tlp14 SFFDFINHKTKNVSTIEIKSNDEFGQISKAINENILATKQGLEQDAKAVKESVETVGVVE 382

T1-21_Tlp19b FILYFINYKTKNVSTIEVKSNDEFGQISNAINENILATKRGLEQDNQAVKESVQTVSVVE 337

CG8421_Tlp14 SFFDFINYKTKNVSTIEVKSNDEFGQISNAINENILATKRGLEQDNQAVKESVQTVSVVE 376

MTVDSCj16_Tlp14 SFFDFINYKTKNVSTIEVKSNDEFGQISNAINENILATKRGLEQDNQAVKESVQTVSVVE 382

01-1512_Tlp14 SFFDFINYKTKNVSTIEVKSNDEFGQISNAINENILATKRGLEQDNQAVKESVQTVSVVE 382

00-0949_Tlp14 SFFDFINYKTKNVSTIEVKSNDEFGQISNAINENILATKRGLEQDNQAVKESVQTVSVVE 382

MTVDSCj13_Tlp14 SFFDFINYKTKNVSTIEVKSNDEFGQISNAINENILATKRGLEQDNQAVKESVQTVSVVE 382

S3_Tlp14 SFFDFINYKTKNVSTIEVKSNDEFGQISNAINENILATKRGLEQDNQAVKESVQTVSVVE 382

PT14_Tlp14 SFFDFINYKTKNVSTIEVKSNDEFGQISNAINENILATKRGLEQDNQAVKESVQTVSVVE 382

14980A_Tlp14 SFFDFINYKTKNVSTIEVKSNDEFGQISNAINENILATKRGLEQDNQAVKESVQTVSVVE 398

FJ3124_Tlp14 SFFDFINYKTKNVSTIEVKSNDEFGQISNAINENILATKRGLEQDNQAVKESVQTVSVVE 382

00-1597_Tlp14 SFFDFINYKTKNVSTIEVKSNDEFGQISNAINENILATKRGLEQDNQAVKESVQTVSVVE 382

R14_Tlp14 SFFDFINYKTKNVSTIEVKSNDEFGQISNAINENILATKRGLEQDNQAVKESVQTVSVVE 382

CG8421_Tlp25 SFFDFINYKTKNVSTIEVKSNDEFGQISNAINENILATKRGLEQDNQAVKESVQTVSVVE 173

HC2-48_Tlp3 SFFDFINYKTKNVSTIEVKTNDEFGQISKAINENILATKQGLEQDAKAVKESVETVGVVE 377

35925B2_Tlp3 SFFDFINHKTKNVSTIEVKSNDEFGQISSAINENILQTKKGLEQDNQAVKESVETVSVVE 388

14980A_Tlp3 SFFDFINYKTKNVSTIEVKSNDEFGQISNAINENILATKRGLEQDNQAVKESVQTVSVVE 388

00-1597_Tlp3b SFFDFINYKTKNVSTIEVKSNDEFGQISNAINENILATKRGLEQDNQAVKESVQTVSVVE 377

ICDCCJ07001_Tlp3 SFFDFINHKTKNVSTIEVKSNDEFGQISNAINENILATKRGLEQDNQAVKESVETVHVVE 377

RM3196_Tlp3 SFFDFINHKTKNVSTIEVKSNDEFGQISNAINENILATKRGLEQDNQAVKESVETVHVVE 377

RM1875_Tlp3 SFFDFINHKTKNVSTIDVKSNDEFGQISKAINENILATKQGLEQDAKAVKESVETVGVVE 377

CF2-75_Tlp3 SFFDFINYKTKNVSTIEVKSNDEFGQISNAINENILATKQGLEQDAKAVKESVETVGVVE 388

RM5611_Tlp3 SFFDFINYKTKNVSTIEVKSNDEFGQISNAINENILATKQGLEQDAKAVKESVETVGVVE 377

MTVDSCj16_Tlp3 SFFDFINYKTKNVSTIEVKSNDEFGQISNAINENILATKRGLEQDNQAVKESVQTVSVVE 377

01-1512_Tlp3 SFFDFINYKTKNVSTIEVKSNDEFGQISNAINENILATKRGLEQDNQAVKESVQTVSVVE 377

MTVDSCj13_Tlp3 SFFDFINYKTKNVSTIEVKSNDEFGQISNAINENILATKRGLEQDNQAVKESVQTVSVVE 377

32488_Tlp3a SFFDFINYKTKNVSTIEVKSNDEFGQISNAINENILATKRGLEQDNQAVKESVQTVSVVE 377

81116_Tlp3 SFFDFINYKTKNVSTIEVKSNDEFGQISNAINENILATKRGLEQDNQAVKESVQTVSVVE 377

32488_Tlp3b SFFDFINYKTKNVSTIEVKSNDEFGQISNAINENILATKRGLEQDNQAVKESVQTVSVVE 377

FB1_Tlp3 SFFDFINYKTKNVSTIEVKSNDEFGQISNAINENILATKQGLEQDAKAVKESVETVGVVE 377

PT14_Tlp3 SFFDFINYKTKNVSTIEVKSNDEFGQISNAINENILATKRGLEQDNQAVKESVQTVSVVE 377

00-6200_Tlp3a SFFDFINHKTKNVSTIEVKSNDELGQMGKIINENILATKRGLEQDNQAVKESVQTVSVVE 377

RM1221_Tlp3 SFFDFINYKTKNVSTIEVKSNDEFGQISNAINENILATKRGLEQDNQAVKESVQTVSVVE 377

S3_Tlp3 SFFDFINYKTKNVSTIEVKSNDEFGQISNAINENILATKRGLEQDNQAVKESVQTVSVVE 377

FDAARGOS_421_Tlp3 SFFDFINYKTKNVSTIEVKSNDEFGQISNAINENILATKRGLEQDNQAVKESVQTVSVVE 388

CFSAN032806_Tlp3 SFFDFINYKTKNVSTIEVKSNDEFGQISNAINENILATKRGLEQDNQAVKESVQTVSVVE 388

IA3901_Tlp3b SFFDFINYKTKNVSTIEVKSNDEFGQISNAINENILATKRGLEQDNQAVKESVQTVSVVE 377

00-6200_Tlp3b SFFDFINYKTKNVSTIEVKSNDEFGQISNAINENILATKRGLEQDNQAVKESVQTVSVVE 377

BCW_6290_Tlp3b SFFDFINYKTKNVSTIEVKSNDEFGQISNAINENILATKRGLEQDNQAVKESVQTVSVVE 377

00-2425_Tlp3a SFFDFINHKTKNVSTIEVKSNDEFGQISNAINENILATKRGLEQDNQAVKESVQTVSVVE 377

00-2425_Tlp3b SFFDFINHKTKNVSTIEVKSNDEFGQISNAINENILATKRGLEQDNQAVKESVQTVSVVE 377

YH001_Tlp3a SFFDFINHKTKNVSTIEVKSNDEFGQISNAINENILATKRGLEQDNQAVKESVQTVSVVE 377

YH001_Tlp3b SFFDFINHKTKNVSTIEVKSNDEFGQISNAINENILATKRGLEQDNQAVKESVQTVSVVE 377

00-0949_Tlp3 SFFDFINYKTKNVSTIEVKSNDEFGQISNAINENILATKRGLEQDNQAVKESVQTVSVVE 377

NCTC11168_Tlp3 SFFDFINYKTKNVSTIEVKSNDEFGQISNAINENILATKRGLEQDNQAVKESVQTVSVVE 388

F38011_Tlp3 SFFDFINYKTKNVSTIEVKSNDEFGQISNAINENILATKRGLEQDNQAVKESVQTVSVVE 377

RM1285_Tlp3 SFFDFINYKTKNVSTIEVKSNDEFGQISNAINENILATKRGLEQDNQAVKESVQTVSVVE 377

FDAARGOS_422_Tlp3 SFFDFINYKTKNVSTIEVKSNDEFGQISNAINENILATKRGLEQDNQAVKESVQTVSVVE 388

MTVDSCj07_Tlp3 SFFDFINYKTKNVSTIEVKSNDEFGQISNAINENILATKRGLEQDNQAVKESVQTVSVVE 377

IA3901_Tlp3a SFFDFINYKTKNVSTIEVKSNDEFGQISNAINENILATKRGLEQDNQAVKESVQTVSVVE 377

BCW_6290_Tlp3a SFFDFINYKTKNVSTIEVKSNDEFGQISNAINENILATKRGLEQDNQAVKESVQTVSVVE 377

T1-21_Tlp19a FILYFINYKTKNVSTIEVKSNDEFGQISNAINENILATKRGLEQDNQAVKESVQTVSVVE 332

CJ677CC012_Tlp3 SFFDFINYKTKNVSIIEVKSNDEFGQISSAINENILATKKGLEQDNQAVKESVQTVSVVE 377

CJM1cam_Tlp3 SFFDFINHKTKNVSTIEVKSNDEFGQISSAINENILATKRGLEQDNQAVKESVETVSVVE 377

M1_Tlp3 SFFDFINHKTKNVSTIEVKSNDEFGQISSAINENILATKRGLEQDNQAVKESVETVSVVE 377

4031_Tlp3 SFFDFINHKTKNVSTIEVKSNDEFGQISNAINENILATKRGLEQDNQAVKESVETVSVVE 377

R14_Tlp3 SFFDFINYKTKNVSTIEVKSNDEFGQISNAINENILATKRGLEQDNQAVKESVQTVSVVE 377

FDAARGOS_295_Tlp21 SFFDFINHKTKNITAINIKSKDEFGQMANAINENILATKKGLEQDNQAVKESVQTVHVVE 379

FORC_046_Tlp4 SFFDFINYKTKNVSTIEVKSNDEFGQISNAINENILATKRGLEQDNQAVKESVQTVSVVE 391

FDAARGOS_422_Tlp4 SFFDFINYKTKNVSTIEVKSNDEFGQISNAINENILATKRGLEQDNQAVKESVQTVSVVE 391

ICDCCJ07001_Tlp4 SFFDFINHKTKNVSTIEVKSNDEFGQISNAINENILATKQGLEQDAKAVKESVETVGVVE 391

RM3196_Tlp4 SFFDFINHKTKNVSTIEVKSNDEFGQISNAINENILATKQGLEQDAKAVKESVETVGVVE 391

T1-21_Tlp4 SFFDFINYKTKNVSTIEVKSNDEFGQISNAINENILATKRGLEQDNQAVKESVQTVSVVE 391

F38011_Tlp4 SFFDFINYKTKNVSTIEVKSNDEFGQISNAINENILATKRGLEQDNQAVKESVQTVSVVE 391

HF5-4A-4_Tlp22 SFFDFINYKTKNVSTIEVKSNDEFGQISNAINENILATKRGLEQDNQAVKESVQTVSVVE 337

00-0949_Tlp4 SFFDFINYKTKNVSTIEVKSNDEFGQISNAINENILATKRGLEQDNQAVKESVQTVSVVE 390

01-1512_Tlp4 SFFDFINYKTKNVSTIEVKSNDEFGQISNAINENILATKRGLEQDNQAVKESVQTVSVVE 391

81-176_Tlp4 SFFDFINYKTKNVSTIEVKSNDEFGQISNAINENILATKRGLEQDNQAVKESVQTVSVVE 391

32488_Tlp4 SFFDFINYKTKNVSTIEVKSNDEFGQISNAINENILATKRGLEQDNQAVKESVQTVSVVE 391

NCTC11168_Tlp4 SFFDFINYKTKNVSTIEVKSNDEFGQISNAINENILATKRGLEQDNQAVKESVQTVSVVE 391

CFSAN032806_Tlp4 SFFDFINYKTKNVSTIEVKSNDEFGQISNAINENILATKRGLEQDNQAVKESVQTVSVVE 391

81116_Tlp4 SFFDFINYKTKNVSTIEVKSNDEFGQISNAINENILATKRGLEQDNQAVKESVQTVSVVE 391

RM1285_Tlp12 SFFDFINYKTKNVSTIEVKSNDEFGQISNAINKTFLLLK-EAEQDNQAVKESVQTVSVVE 387

PT14_Tlp12 SFFDFINYKTKNVSTIEVKSNDEFGQISNAINENILATKRGLEQDNQAVKESVQTVSVVE 388

MTVJDCj07_Tlp12 SFFDFINYKTKNVSTIEVKSNDEFGQISNAINENILATKRGLEQDNQAVKESVQTVSVVE 388

RM1221_Tlp12 SFFDFINYKTKNVSTIEVKSNDEFGQISNAINENILATKRGLEQDNQAVKESVQTVSVVE 388

FDAARGOS_421_Tlp12 SFFDFINYKTKNVSTIEVKSNDEFGQISNAINENILATKRGLEQDNQAVKESVQTVSVVE 388

35925B2_Tlp12 SFFDFINHKTKNVSTIEVKSNDEFGQISSAINENILQTKKGLEQDNQAVKESVETVSVVE 388

CJM1cam_Tlp12 SFFDFINYKTKNVSTIEVKSNDEFGQISNAINENILATKRGLEQDNQAVKESVQTVSVVE 388

M1_Tlp12 SFFDFINYKTKNVSTIEVKSNDEFGQISNAINENILATKRGLEQDNQAVKESVQTVSVVE 388

S3_Tlp12 SFFDFINYKTKNVSTIEVKSNDEFGQISNAINENILATKRGLEQDNQAVKESVQTVSVVE 388

00-1597_Tlp12 SFFDFINYKTKNVSTIEVKSNDEFGQISNAINENILATKRGLEQDNQAVKESVQTVSVVE 388

R14_Tlp12 SFFDFINYKTKNVSTIEVKSNDEFGQISNAINENILATKRGLEQDNQAVKESVQTVSVVE 388

RM1875_Tlp15 SFFDFINHKTKNVSTIEVKSNDEFGQISKAINENILATKQGLEQDAKAVKESVETVGVVE 305

YH503_Tlp16 SFFNFINHKTKNVSTIEIKSNDEFGQISKTINENILATKRGLEQDNQAVKESVQTVSVVE 305

FB1_Tlp16 SFFNFINHKTKNVSTIDVKTNDEFGLISKAINENILATKQGLEQDAKAVKESVETVGVVE 305

BFR-CA-9557_Tlp16 SFFNFINHKTKNVSTIEIKSNDEFGQISKTINENILATKQGLEQDAKAVKESVETVGVVE 304

15-537360_Tlp16 SFFNFINHKTKNVSTIEIKSNDEFGQISKAINENILATKQGLEQDAKAVKESVETVGVVE 305

OR12_Tlp16 SFFNFINHKTKNVSTIEIKSNDEFGQISKTINENILATKQGLEQDAKAVKESVETVGVVK 305

YH502_Tlp16 SFFNFINHKTKNVSTIEIKSNDEFGQISKTINENILATKQGLEQDAKAVKESVETVGVVE 305

14903A_Tlp16 SFFNFINHKTKNVSTIEIKSNDEFGQISKAINENILATKQGLEQDAKAVKESVETVGVVE 305

HC2-48_Tlp16 SFFDFINHN-KNVSTIDVKTNDEFGQISKAINENILATKQGLEQDAKAVKESVETVGVVE 304

RM5611_Tlp16 SFFDFINHKTKNVSTIDVKTNDEFGQISKAINENILATKQGLEQDAKAVKESVETVGVVE 305

00-2425_Tlp11 NFFDFINHKTKNVSTIEVKSNDELGQMGKIINENILATKRGLEQDNQAVKESVQTVSVVE 432

00-6200_Tlp11 NFFDFINHKTKNVSTIEVKSNDELGQMGKIINENILATKRGLEQDNQAVKESVQTVSVVE 432

YH001_Tlp11 NFFDFINHKTKNVSTIEVKSNDELGQMGKIINENILATKRGLEQDNQAVKESVQTVSVVE 432

IA3902_Tlp11 NFFDFINHKTKNVSTIEVKSNDELGQMGKIINENILATKRGLEQDNQAVKESVETVHVVE 432

BCW_6290_Tlp11 NFFDFINHKTKNVSTIEVKSNDELGQMGKIINENILATKRGLEQDNQAVKESVETVHVVE 432

76339_Tlp18 DFFRFLNHEKIEVHTIKISSNDELGKMAKAINENILATKQGLEQDAKAVKESVETVEVVE 431

4031_Tlp17 NFFHFLNHKKHEVDLISIKADDELGKMGKMINENILATKKGLEQDNQAVKESVQTVSVVE 432

MTVDSCj13_Tlp13 NFFRFLNHEKIEIQTIEIKANDELGKMGKIINENILATKQGLEQDAKAVKESVETVGVVE 431

RM1875_Tlp13 NFFRFLNHEKIEIQTIEIKANDELGKMGKIINENILATKQGLEQDAKAVKESVETVGVVE 431

CF2-75_Tlp13 NFFRFLNHEKIEIQTIEIKANDELGKMGKIINENILATKQGLEQDAKAVKESVETVGVVE 431

15-537360_Tlp13 NFFRFLNHEKIEVQTIEIKANDELGKMGKIINENILATKQGLEQDAKAVKESVETVSVVE 431

CVMN29710_Tlp13 NFFRFLNHEKIEVQTIEIKANDELGKMGKIINENILATKRGLEQDNQAVKESVQTVSVVE 431

FB1_Tlp13 NFFRFLNHEKIEVQTIEIKANDELGKMGKIINENILATKRGLEQDNQAVKESVQTVSVVE 431

CFCAN032805_Tlp13 NFFRFLNHEKIEVQTIEIKANDELGKMGKIINENILATKRGLEQDNQAVKESVQTVSVVE 431

BG2108_Tlp13 NFFRFLNHEKIEVQTIEIKANDELGKMGKIINENILATKRGLEQDNQAVKESVQTVSVVE 431

YF2105_Tlp13 NFFRFLNHEKIEVQTIEIKANDELGKMGKIINENILATKRGLEQDNQAVKESVQTVSVVE 431

YH503_Tlp13 NFFRFLNHEKIEVQTIEIKANDELGKMGKTINENILATKRGLEQDNQAVKESVQTVSVVE 431

BFRCA9557_Tlp13 NFFRFLNHEKIEIQTIEIKANDELGKMGKIINENILATKQGLEQDAKAVKESVETVGVVE 431

YH502_Tlp13 NFFRFLNHEKIEVQTIEIKANDELGKMGKTINENILATKQGLEQDAKAVKESVETVGVVE 431

OR12_Tlp13 NFFRFLNHEKIEVQTIEIKANDELGKMGKIINENILATKRGLEQDNQAVKESVQTVSVVE 431

00-1597_Tlp13 NFFRFLNHEKIEVQTIEIKANDELGKMGKIINENILATKRGLEQDNQAVKESVQTVSVVE 431

14903A_Tlp13 NFFRFLNHEKIEVQTIEIKANDELGKMGKIINENILATKRGLEQDNQAVKESVQTVSVVE 431

FJ3124_Tlp13 NFFRFLNHEKIEVQTIEIKANDELGKMGKIINENILATKRGLEQDNQAVKESVQTVSVVE 431

R14_Tlp13 NFFRFLNHEKIEVQTIEIKANDELGKMGKIINENILATKRGLEQDNQAVKESVQTVSVVE 431

MTVDSCj16_Tlp13 NFFRFLNHEKIEVQTIEIKANDELGKMGKIINENILATKRGLEQDNQAVKESVQTVSVVE 431

14980A_Tlp13 NFFRFLNHEKIEVQTIEIKANDELGKMGKIINENILATKRGLEQDNQAVKESVQTVSVVE 431

FDAARGOS_295_Tlp1 KGNLTARI-------TKNPINPQLVELKNVLNRMLDALQSKIGS---NMNEINRVFDSYK 476

14980A_Tlp1 KGNLTARI-------TKNPINPQLVELKNVLNKMLDVLQNKIGS---NMNEINRVFDSYK 476

CJ677CC527_Tlp1 KGNLTARI-------TKNPINPQLVELKNVLNRMLDVLQSKIGS---NMNEINRVFDSYK 476

CJ677CC012_Tlp1 KGNLTARI-------TKNPINPQLVELKNVLNRMLDVLQSKIGS---NMNEINRVFDSYK 476

4031_Tlp1 KGNLTARI-------TKNPINPQLVELKNVLNRMLDVLQSKIGS---NMNEINRVFDSYK 476

81116_Tlp1 KGNLTARI-------TKNPINPQLVELKNVLNRMLDVLQSKIGS---NMNEINRVFDSYK 476

35925B2_Tlp1 KGNLTARI-------TKNPINPQLVELKNVLNRMLDVLQSKIGS---NMNEINRVFDSYK 476

M1_Tlp1 KGNLTARI-------TKNPINPQLVELKNVLNRMLDVLQSKIGS---NMNEINRVFDSYK 476

PT14_Tlp1 KGNLTARI-------TKNPINPQLVELKNVLNRMLDVLQSKIGS---NMNEINRVFDSYK 476

81-176_Tlp1 KGNLTARI-------TKNPINPQLVELKNVLNRMLDVLQSKIGS---NMNEINRVFDSYK 476

CVMN29710_Tlp1 KGNLTARI-------TKNPINPQLVELKNVLNRMLDVLQSKIGS---NMNEINRVFDSYK 476

FB1_Tlp1 KGNLTARI-------TKNPINPQLVELKNVLNRMLDVLQSKIGS---NMNEINRVFDSYK 476

BG2108_Tlp1 KGNLTARI-------TKNPINPQLVELKNVLNRMLDVLQSKIGS---NMNEINRVFDSYK 476

YF2108_Tlp1 KGNLTARI-------TKNPINPQLVELKNVLNRMLDVLQSKIGS---NMNEINRVFDSYK 476

YH501_Tlp1 KGNLTARI-------TKNPINPQLVELKNVLNRMLDVLQSKIGS---NMNEINRVFDSYK 476

RM4661_Tlp1 KGNLTARI-------TKNPINPQLVELKNVLNRMLDVLQSKIGS---NMNEINRVFDSYK 476

F38011_Tlp1 KGNLTARI-------TKNPINPQLVELKNVLNRMLDVLQSKIGS---NMNEINRVFDSYK 476

T1-21_Tlp1 KGNLTARI-------TKNPINPQLVELKNVLNRMLDVLQSKIGS---NMNEINRVFDSYK 476

CG8421_Tlp1 KGNLTARI-------TKNPINPQLVELKNVLNRMLDVLQSKIGS---NMNEINRVFDSYK 476

CJM1cam_Tlp1 KGNLTARI-------TKNPINPQLVELKNVLNRMLDVLQSKIGS---NMNEINRVFDSYK 476

R14_Tlp1 KGNLTARI-------TKNPINPQLVELKNVLNRMLDVLQSKIGS---NMNEINRVFDSYK 476

ICDCCJ07001_Tlp1 KGNLTARI-------TKNPINPQLVELKDVLNRMLDVLQSKIGS---NMNEINRVFDSYK 476

RM3196_Tlp1 KGNLTARI-------TKNPINPQLVELKDVLNRMLDVLQSKIGS---NMNEINRVFDSYK 476

NCTC11168_Tlp1 KGNLTARI-------TKNPINPQLVELKDVLNRMLDVLQSKIGS---NMNEINRVFDSYK 476

00-2425_Tlp1 KGNLTARI-------TKNPINPQLVELKDVLNRMLDVLQSKIGS---NMNEINRVFDSYK 476

IA3902_Tlp1 KGNLTARI-------TKNPINPQLVELKDVLNRMLDVLQSKIGS---NMNEINRVFDSYK 476

RM1285_Tlp1 KGNLTARI-------TKNPINPQLVELKDVLNRMLDVLQSKIGS---NMNEINRVFDSYK 476

00-0949_Tlp1 KGNLTARI-------TKNPINPQLVELKDVLNRMLDVLQSKIGS---NMNEINRVFDSYK 476

01-1512_Tlp1 KGNLTARI-------TKNPINPQLVELKDVLNRMLDVLQSKIGS---NMNEINRVFDSYK 476

FDAARGOS_422_Tlp1 KGNLTARI-------TKNPINPQLVELKDVLNRMLDVLQSKIGS---NMNEINRVFDSYK 476

FORC_056_Tlp1 KGNLTARI-------TKNPINPQLVELKDVLNRMLDVLQSKIGS---NMNEINRVFDSYK 476

32488_Tlp1 KGDLTARI-------TKNPINPQLVELKNVLNRMLDVLQSKIGS---NMNEINRVFDSYK 476

CFSAN032806_Tlp1 KGDLTARI-------TKNPINPQLVELKNVLNRMLDVLQSKIGS---NMNEINRVFDSYK 476

YH001_Tlp1 KGNLTARI-------TKNPINPQLVELKNVLNRMLDVLQSKIGS---NMNEINRVFDSYK 476

00-6200_Tlp1 KGNLTARI-------TKNPINPQLVELKNVLNRMLDVLQSKIGS---NMNEINRVFDSYK 476

RM1221_Tlp1 KGNLTARI-------TKNPINPQLVELKNVLNRMLDVLQSKIGS---NMNEINRVFDSYK 476

S3_Tlp1 KGNLTARI-------TKNPINPQLVELKNVLNRMLDVLQSKIGS---NMNEINRVFDSYK 476

FDAARGOS_421_Tlp1 KGNLTARI-------TKNPINPQLVELKNVLNRMLDVLQSKIGS---NMNEINRVFDSYK 476

FJ3124_Tlp1 KGNLTARI-------TKNPINPQLVELKNVLNRMLDVLQSKIGS---NMNEINRVFDSYK 476

00-1597_Tlp1 KGNLTARI-------TKNPINPQLVELKNVLNRMLDVLQSKIGS---NMNEINRVFDSYK 476

15-537360_Tlp20 SGNLTARI-------TANPRNPQLIELKNVLNRLLDALQARVGS---DMNEIQRVFNSYK 435

76339_Tlp20 RGNLTARI-------TANPRNPQLIELKNVLNKLLDVLQTKVGS---DMNAIHKIFEEYK 435

HC2-48_Tlp20 SGNLTARI-------TANPRNPQLIELKNVLNRLLDVLQTKVGS---DMNAIHKIFEEYK 434

CFSAN032805_Tlp20 RGNLTARI-------TANPRNPQLIELKNVLNKLLDVLQTKVGS---DMNAIHKIFEEYK 434

CVMN29710_Tlp20 RGNLTARI-------TANPRNPQLIELKNVLNKLLDVLQTKVGS---DMNAIHKIFEEYK 433

YH501_Tlp20 RGNLTARI-------TANPRNPQLIELKNVLNKLLDVLQTKVGS---DMNAIHKIFEEYK 437

CF2-75_Tlp20 SGNLTARI-------TANPRNPQLIELKNVLNRLLDVLQTKVGS---DMNAIHKIFEEYK 438

CO2-160_Tlp20 SGNLTARI-------TANPRNPQLIELKNVLNRLLDVLQTKVGS---DMNAIHKIFEEYK 435

CO2-160_Tlp20b SGNLTARI-------TANPRNPQLIELKNVLNRLLDVLQTKVGS---DMNAIHKIFEEYK 435

RM5611_Tlp20 SGNLTARI-------TANPRNPQLIELKNVLNRLLDVLQTRVGS---DMNAIHKIFEEYK 435

14903A_Tlp20 SGNLTARI-------TANPRNPQLIELKNVLNRLLDVLQTKVGS---DMNAIHKIFEEYK 437

YH502_Tlp20 SGNLTARI-------TANPRNPQLIELKNVLNRLLDVLQTKVGS---DMNAIHKIFEEYK 434

RM3196_Tlp23 GGNLTARI-------TANPRNPQLIELKNVLNRLLDALQARVGS---DMNEIQRVFNSYK 435

ICDCCJ07001_Tlp23 GGNLTARI-------TANPRNPQLIELKNVLNRLLDALQARVGS---DMNEIQRVFNSYK 435

RM1285_Tlp2 GGNLTARI-------TANPRNPQLIELKNVLNKLLDVLQARVGS---DMNAIHKIFEEYK 434

CFSAN032806_Tlp2 GGNLTARI-------TANPRNPQLIELKNVLNKLLDVLQARVGS---DMNAIHKIFEEYK 435

RM1221_Tlp2 GGNLTARI-------TANPRNPQLIELKNVLNKLLDVLQARVGS---DMNAIHKIFEEYK 435

S3_Tlp2 GGNLTARI-------TANPRNPQLIELKNVLNKLLDVLQARVGS---DMNAIHKIFEEYK 435

FDAARGOS_422_Tlp2 GGNLTARI-------TANPRNPQLIELKNVLNKLLDVLQARVGS---DMNAIHKIFEEYK 435

81-176_Tlp2 GGNLTARI-------TANPRNPQLIELKNVLNKLLDVLQARVGS---DMNAIHKIFEEYK 435

F38011_Tlp2 GGNLTARI-------TANPRNPQLIELKNVLNKLLDVLQARVGS---DMNAIHKIFEEYK 435

NCTC11168_Tlp2 GGNLTARI-------TANPRNPQLIELKNVLNKLLDVLQARVGS---DMNAIHKIFEEYK 435

MTVDSCj07_Tlp2 GGNLTARI-------TANPRNPQLIELKNVLNKLLDVLQARVGS---DMNAIHKIFEEYK 435

CJM1cam_Tlp24 SGNLTARI-------TANPRNPQLIELKNVLNKLLDVLQARVVL---Y-ECYS-NF-RIQ 432

M1_Tlp24 SGNLTARI-------TANPRNPQLIELKNVLNKLLDVLQARVVL---I--CYS-NF-RIQ 431

81116_Tlp2 SGNLTARI-------TANPRNPQLIELKNVLNKLLDVLQARVGS---DMNAIHKIFEEYK 435

4031_Tlp23 SGNLTARI-------TANPRNPQLIELKNVLNKLLDVLQARVGS---DMNAIHKIFEEYK 434

CVMN29710_Tlp14 SGNLTARI-------TANPRNPQLIELKNVLNKLLDVLQAKVGS---DMNEIQRVFNSYK 432

RM4661_Tlp14 SGNLTARI-------TANPRNPQLIELKNVLNRLLDVLQTKVGS---DMNAIHKIFEEYK 432

MG1116_Tlp14 SGNLTARITANHARITANPRNPQLIELKNVLNRLLDVLQTKVGS---DMNAIHKIFEEYK 425

BG2108_Tlp14 ------------ARITANPRNPQLIELKNVLNRLLDVLQTKVGS---DMNAIHKIFEEYK 364

YF2105_Tlp14 Q--IWF-RSCLRARITANPRNPQLIELKNVLNRLLDVLQTKVGS---DMNAIHKIFEEYK 393

YH502_Tlp14 SGNLTARI-------TANPRNPQLIELKNVLNRLLDVLQTKVGS---DMNAIHKIFEEYK 443

WA333_Tlp14 SGNLTARI-------TANPRNPQLIELKNVLNRLLDVLQTKVGS---DMNAIHKIFEEYK 421

BP3181_Tlp14 SGNLTARI-------TANPRNPQLIELKNVLNRLLDVLQTKVGS---DMNAIHKIFEEYK 432

ZV1224_Tlp14a SGNLTARI-------TANPRNPQLIELKNVLNRLLDVLQTKVGS---DMNAIHKIFEEYK 432

ZV1224_Tlp14b SGNLTARI-------TANPRNPQLIELKNVLNRLLDVLQTKVGS---DMNAIHKIFEEYK 432

YH503_Tlp14 RGNLTARI-------TANPRNPQLIELKNVLNRLLDVLQTKVGS---DMNAIHKIFEEYK 443

14903A_Tlp14 SGNLTARI-------TANPRNPQLIELKNVLNRLLDVLQTKVGS---DMNAIHKIFEEYK 432

OR12_Tlp14 RGNLTARI-------TANPRNPQLIELKNVLNKLLDVLQTKVGS---DMNAIHKIFEEYK 432

CFSAN032805_Tlp14 RGNLTARI-------TANPRNPQLIELKNVLNKLLDVLQTKVGS---DMNAIHKIFEEYK 443

BFR-CA-9557_Tlp14 RGNLTARI-------TANPRNPQLIELKNVLNRLLDVLQTKVGS---DMNAIHKIFEEYK 432

15-537360_Tlp14 SGNLTARI-------TANPRNPQLIELKNVLNRLLDVLQTKVGS---DMNAIHKIFEEYK 432

YH501_Tlp14 RGNLTARI-------TANPRNPQLIELKNVLNKLLDVLQTKVGS---DMNAIHKIFEEYK 432

T1-21_Tlp19b GGNLTARI-------TANPRNPQLIELKNVLNKLLDVLQARVGS---DMNAIHKIFEEYK 387

CG8421_Tlp14 GGNLTARI-------TANPRNPQLIELKNVLNKLLDVLQARVGS---DMNAIHKIFEEYK 426

MTVDSCj16_Tlp14 GGNLTARI-------TANPRNPQLIELKNVLNKLLDVLQARVGS---DMNAIHKIFEEYK 432

01-1512_Tlp14 GGNLTARI-------TANPRNPQLIELKNVLNKLLDVLQARVGS---DMNAIHKIFEEYK 432

00-0949_Tlp14 GGNLTARI-------TANPRNPQLIELKNVLNKLLDVLQARVGS---DMNAIHKIFEEYK 432

MTVDSCj13_Tlp14 SGNLTARI-------TANPRNPQLIELKNVLNKLLDVLQARVGS---DMNAIHKIFEEYK 432

S3_Tlp14 GGNLTARI-------TANPRNPQLIELKNVLNKLLDVLQARVGS---DMNAIHKIFEEYK 432

PT14_Tlp14 GGNLTARI-------TANPRNPQLIELKNVLNKLLDVLQARVGS---DMNAIHKIFEEYK 432

14980A_Tlp14 GGNLTARI-------TANPRNPQLIELKNVLNKLLDVLQARVGS---DMNAIHKIFEEYK 448

FJ3124_Tlp14 GGNLTARI-------TANPRNPQLIELKNVLNKLLDVLQARVGS---DMNAIHKIFEEYK 432

00-1597_Tlp14 GGNLTARI-------TANPRNPQLIELKNVLNRLLDALQARVGS---DMNEIQRVFNSYK 432

R14_Tlp14 GGNLTARI-------TANPRNPQLIELKNVLNRLLDALQARVGS---DMNEIQRVFNSYK 432

CG8421_Tlp25 GGNLTARI-------TANPRNPQLIELKNVLNKLLDVLQARVGS---DMNAIHKIFEEYK 223

HC2-48_Tlp3 SGNLTARI-------TANPRNPQLIELKNVLNRLLDVLQTKVGS---DMNAIHKIFEEYK 427

35925B2_Tlp3 SGNLTARI-------TANPRNPQLIELKNVLNRLLDALQTRVGSDGSDMNEIQRVFNSYK 441

14980A_Tlp3 GGNLTARI-------TANPRNPQLIELKNVLNRLLDALQARVG---SDMNEIQRVFNSYK 438

00-1597_Tlp3b GGNLTARI-------TANPRNPQLIELKNVLNRLLDALQARVG---SDMNEIQRVFNSYK 427

ICDCCJ07001_Tlp3 GGNLTARI-------TANPRNPQLIELKNVLNRLLDALQARVG---SDMNEIQRVFNSYK 427

RM3196_Tlp3 GGNLTARI-------TANPRNPQLIELKNVLNRLLDALQARVG---SDMNEIQRVFNSYK 427

RM1875_Tlp3 SGNLTARI-------TANPRNPQLIELKNVLNRLLDVLQTRVGS---DMNAIHKIFEEYK 427

CF2-75_Tlp3 SGNLTARI-------TANPRNPQLIELKNVLNRLLDVLQTKVGS---DMNAIHKIFEEYK 438

RM5611_Tlp3 SGNLTARI-------TANPRNPQLIELKNVLNRLLDVLQTKVGS---DMNAIHKIFEEYK 427

MTVDSCj16_Tlp3 GGNLTARI-------TANPRNPQLIELKNVLNKLLDVLQARVGS---DMNAIHKIFEEYK 427

01-1512_Tlp3 GGNLTARI-------TANPRNPQLIELKNVLNKLLDVLQARVGS---DMNAIHKIFEEYK 427

MTVDSCj13_Tlp3 SGNLTARI-------TANPRNPQLIELKNVLNKLLDVLQARVGS---DMNAIHKIFEEYK 427

32488_Tlp3a GGNLTARI-------TANPRNPQLIELKNVLNKLLDVLQARVGS---DMNAIHKIFEEYK 427

81116_Tlp3 GGNLTARI-------TANPRNPQLIELKNVLNKLLDVLQARVGS---DMNAIHKIFEEYK 427

32488_Tlp3b GGNLTARI-------TANPRNPQLIELKNVLNKLLDVLQARVGS---DMNAIHKIFEEYK 427

FB1_Tlp3 RGNLTARI-------TANPRNPQLIELKNVLNKLLDVLQARVGS---DMNAIHKIFEEYK 427

PT14_Tlp3 GGNLTARI-------TANPRNPQLIELKNVLNKLLDVLQARVGS---DMNAIHKIFEEYK 427

00-6200_Tlp3a GGNLTARI-------TANPRNPQLIELKNVLNKLLDVLQARVGS---DMNAIHKIFEEYK 427

RM1221_Tlp3 GGNLTARI-------TANPRNPQLIELKNVLNKLLDVLQARVGS---DMNAIHKIFEEYK 427

S3_Tlp3 GGNLTARI-------TANPRNPQLIELKNVLNKLLDVLQARVGS---DMNAIHKIFEEYK 427

FDAARGOS_421_Tlp3 GGNLTARI-------TANPRNPQLIELKNVLNKLLDVLQARVGS---DMNAIHKIFEEYK 438

CFSAN032806_Tlp3 GGNLTARI-------TANPRNPQLIELKNVLNKLLDVLQARVGS---DMNAIHKIFEEYK 438

IA3901_Tlp3b GGNLTARI-------TANPRNPQLIELKNVLNKLLDVLQARVGS---DMNAIHKIFEEYK 427

00-6200_Tlp3b GGNLTARI-------TANPRNPQLIELKNVLNKLLDVLQARVGS---DMNAIHKIFEEYK 427

BCW_6290_Tlp3b GGNLTARI-------TANPRNPQLIELKNVLNKLLDVLQARVGS---DMNAIHKIFEEYK 427

00-2425_Tlp3a GGNLTARI-------TANPRNPQLIELKNVLNKLLDVLQARVGS---DMNAIHKIFEEYK 427

00-2425_Tlp3b GGNLTARI-------TANPRNPQLIELKNVLNKLLDVLQARVGS---DMNAIHKIFEEYK 427

YH001_Tlp3a GGNLTARI-------TANPRNPQLIELKNVLNKLLDVLQARVGS---DMNAIHKIFEEYK 427

YH001_Tlp3b GGNLTARI-------TANPRNPQLIELKNVLNKLLDVLQARVGS---DMNAIHKIFEEYK 427

00-0949_Tlp3 GGNLTARI-------TANPRNPQLIELKNVLNKLLDVLQARVGS---DMNAIHKIFEEYK 427

NCTC11168_Tlp3 GGNLTARI-------TANPRNPQLIELKNVLNKLLDVLQARVGS---DMNAIHKIFEEYK 438

F38011_Tlp3 GGNLTARI-------TANPRNPQLIELKNVLNKLLDVLQARVGS---DMNAIHKIFEEYK 427

RM1285_Tlp3 GGNLTARI-------TANPRNPQLIELKNVLNKLLDVLQARVGS---DMNAIHKIFEEYK 427

FDAARGOS_422_Tlp3 GGNLTARI-------TANPRNPQLIELKNVLNKLLDVLQARVGS---DMNAIHKIFEEYK 438

MTVDSCj07_Tlp3 GGNLTARI-------TANPRNPQLIELKNVLNKLLDVLQARVGS---DMNAIHKIFEEYK 427

IA3901_Tlp3a GGNLTARI-------TANPRNPQLIELKNVLNKLLDVLQARVGS---DMNAIHKIFEEYK 427

BCW_6290_Tlp3a GGNLTARI-------TANPRNPQLIELKNVLNKLLDVLQARVGS---DMNAIHKIFEEYK 427

T1-21_Tlp19a GGNLTARI-------TANPRNPQLIELKNVLNKLLDVLQARVGS---DMNAIHKIFEEYK 382

CJ677CC012_Tlp3 GGNLTARI-------TANPRNPQLIELKNVLNKLLDVLQARVGS---DMNVIH------- 420

CJM1cam_Tlp3 SGNLTARI-------TANPRNPQLIELKNVLNKLLDVLQARVGS---DMNAIHKIFEEYK 427

M1_Tlp3 SGNLTARI-------TANPRNPQLIELKNVLNKLLDVLQARVGS---DMNAIHKIFEEYK 427

4031_Tlp3 SGNLTARI-------TANPRNPQLIELKNVLNKLLDVLQARVGS---DMNAIHKIFEEYK 427

R14_Tlp3 GGNLTARI-------TANPRNPQLIELKNVLNKLLDVLQARVGS---DMNAIHKIFEEYK 427

FDAARGOS_295_Tlp21 SGNLTARI-------TANPRNPQLIELKNVLNKLLDVLQARVGS---DMNEIQRVFNSYK 429

FORC_046_Tlp4 GGNLTARI-------TANPRNPQLIELKNVLNRLLDALQARVGS---DMNEIQRVFNSYK 441

FDAARGOS_422_Tlp4 GGNLTARI-------TANPRNPQLIELKNVLNRLLDALQARVGS---DMNEIQRVFNSYK 441

ICDCCJ07001_Tlp4 SGNLTARI-------TANPRNPQLIELKNVLNRLLDVLQTRVGS---DMNAIHKIFEEYK 441

RM3196_Tlp4 SGNLTARI-------TANPRNPQLIELKNVLNRLLDVLQTRVGS---DMNAIHKIFEEYK 441

T1-21_Tlp4 GGNLTARI-------TANPRNPQLIELKNVLNKLLDVLQARVGS---DMNAIHKIFEEYK 441

F38011_Tlp4 GGNLTARI-------TANPRNPQLIELKNVLNKLLDVLQARVGS---DMNAIHKIFEEYK 441

HF5-4A-4_Tlp22 GGNLTARI-------TANPRNPQLIELKNVLNKLLDVLQARVGS---DMNAIHKIFEEYK 387

00-0949_Tlp4 GGNLTARI-------TANPRNPQLIELKNVLNKLLDVLQARVGS---DMNAIHKIFEEYK 440

01-1512_Tlp4 GGNLTARI-------TANPRNPQLIELKNVLNKLLDVLQARVGS---DMNAIHKIFEEYK 441

81-176_Tlp4 GGNLTARI-------TANPRNPQLIELKNVLNKLLDVLQARVGS---DMNAIHKIFEEYK 441

32488_Tlp4 GGNLTARI-------TANPRNPQLIELKNVLNKLLDVLQARVGS---DMNAIHKIFEEYK 441

NCTC11168_Tlp4 GGNLTARI-------TANPRNPQLIELKNVLNKLLDVLQARVGS---DMNAIHKIFEEYK 441

CFSAN032806_Tlp4 GGNLTARI-------TANPRNPQLIELKNVLNKLLDVLQARVGS---DMNAIHKIFEEYK 441

81116_Tlp4 GGNLTARI-------TANPRNPQLIELKNVLNKLLDVLQARVGS---DMNAIHKIFEEYK 441

RM1285_Tlp12 GGNLTARI-------TANPRNPQLIELKNVLNKLLDVLQARVGS---DMNAIHKIFEEYK 437

PT14_Tlp12 GGNLTARI-------TANPRNPQLIELKNVLNKLLDVLQARVGS---DMNAIHKIFEEYK 438

MTVJDCj07_Tlp12 GGNLTARI-------TANPRNPQLIELKNVLNKLLDVLQARVGS---DMNAIHKIFEEYK 438

RM1221_Tlp12 GGNLTARI-------TANPRNPQLIELKNVLNKLLDVLQARVGS---DMNAIHKIFEEYK 438

FDAARGOS_421_Tlp12 GGNLTARI-------TANPRNPQLIELKNVLNKLLDVLQARVGS---DMNAIHKIFEEYK 438

35925B2_Tlp12 SGNLTARI-------TANPRNPQLIELKNVLNRLLDALQTRVGS---DMNEIQRVFNSYK 438

CJM1cam_Tlp12 GGNLTARI-------TANPRNPQLIELKNVLNRLLDALQARVGS---DMNEIQRVFNSYK 438

M1_Tlp12 GGNLTARI-------TANPRNPQLIELKNVLNRLLDALQARVGS---DMNEIQRVFNSYK 438

S3_Tlp12 GGNLTARI-------TANPRNPQLIELKNVLNRLLDALQARVGS---DMNEIQRVFNSYK 438

00-1597_Tlp12 GGNLTARI-------TANPRNPQLIELKNVLNRLLDALQARVGS---DMNEIQRVFNSYK 438

R14_Tlp12 GGNLTARI-------TANPRNPQLIELKNVLNRLLDALQARVGS---DMNEIQRVFNSYK 438

RM1875_Tlp15 SGNLTARI-------TANPRNPQLIELKNVLNRLLDVLQTRVGS---DMNAIHKIFEEYK 355

YH503_Tlp16 GGNLTARI-------TANPRNPQLIELKNVLNRLLDVLQTKVGS---DMNAIHKIFEEYK 355

FB1_Tlp16 RGNLTARI-------TANPRNPQLIELKNVLNRLLDVLQTKVGS---DMNAIHKIFEEYK 355

BFR-CA-9557_Tlp16 RGNLTARI-------TANPRNPQLIELKNVLNKLLDVLQTKVGS---DMNAIHKIFEEYK 354

15-537360_Tlp16 SGNLTARI-------TANPRNPQLIELKNVLNRLLDALQARVGS---DMNAIHKIFEEYK 355

OR12_Tlp16 RGNLTARI-------TANPRNPQLIELKNVLNKLLDVLQTKVGS---DMNAIHKIFEEYK 355

YH502_Tlp16 SGNLTARI-------TANPRNPQLIELKNVLNRLLDVLQTKVGS---DMNAIHKIFEEYK 355

14903A_Tlp16 SGNLTARI-------TANPRNPQLIELKNVLNRLLDVLQTKVGS---DMNAIHKIFEEYK 355

HC2-48_Tlp16 SGNLTARI-------TANPRNPQLIELKNVLNRLLDVLQTKVGS---DMNAIHKIFEEYK 354

RM5611_Tlp16 SGNLTARI-------TANPRNPQLIELKNVLNRLLDVLQTKVGS---DMNAIHKIFEEYK 355

00-2425_Tlp11 GGNLTARI-------TANPRNPQLIELKNVLNKLLDVLQARVGS---DMNAIHKIFEEYK 482

00-6200_Tlp11 GGNLTARI-------TANPRNPQLIELKNVLNKLLDVLQARVGS---DMNAIHKIFEEYK 482

YH001_Tlp11 GGNLTARI-------TANPRNPQLIELKNVLNKLLDVLQARVGS---DMNAIHKIFEEYK 482

IA3902_Tlp11 GGNLTARI-------TANPRNPQLIELKNVLNRLLDALQARVGS---DMNEIQRVFNSYK 482

BCW_6290_Tlp11 GGNLTARI-------TANPRNPQLIELKNVLNRLLDALQARVGS---DMNEIQRVFNSYK 482

76339_Tlp18 RGNLTARI-------TANPRNPQLIELKNVLNKLLDVLQTKVGS---DMNAIHKIFEEYK 481

4031_Tlp17 SGNLTARI-------TANPRNPQLIELKNVLNKLLDVLQARVGS---DMNAIHKIFEEYK 482

MTVDSCj13_Tlp13 SGNLTARI-------TANPRNPQLIELKNVLNKLLDVLQARVGS---DMNAIHKIFEEYK 481

RM1875_Tlp13 SGNLTARI-------TANPRNPQLIELKNVLNRLLDVLQTRVGS---DMNAIHKIFEEYK 481

CF2-75_Tlp13 SGNLTARI-------TANPRNPQLIELKNVLNRLLDVLQTKVGS---DMNAIHKIFEEYK 481

15-537360_Tlp13 GGNLTARI-------TANPRNPQLIELKNVLNRLLDALQARVGS---DMNAIHKIFEEYK 481

CVMN29710_Tlp13 GGNLTARI-------TANPRNPQLIELKNVLNRLLDALQARVGS---DMNAIHKIFEEYK 481

FB1_Tlp13 GGNLTARI-------TANPRNPQLIELKNVLNRLLDALQARVGS---DMNAIHKIFEEYK 481

CFCAN032805_Tlp13 GGNLTARI-------TANPRNPQLIELKNVLNRLLDALQARVGS---DMNAIHKIFEEYK 481

BG2108_Tlp13 GGNLTARI-------TANPRNPQLIELKNVLNRLLDVLQTKVGS---DMNAIHKIFEEYK 481

YF2105_Tlp13 GGNLTARI-------TANPRNPQLIELKNVLNRLLDVLQTKVGS---DMNAIHKIFEEYK 481

YH503_Tlp13 GGNLTARI-------TANPRNPQLIELKNVLNRLLDVLQTKVGS---DMNAIHKIFEEYK 481

BFRCA9557_Tlp13 RGNLTARI-------TANPRNPQLIELKNVLNKLLDVLQTKVGS---DMNAIHKIFEEYK 481

YH502_Tlp13 SGNLTARI-------TANPRNPQLIELKNVLNRLLDVLQTKVGS---DMNAIHKIFEEYK 481

OR12_Tlp13 GGNLTARI-------TANPRNPQLIELKNVLNRLLDALQARVGS---DMNEIQRVFNSYK 481

00-1597_Tlp13 GGNLTARI-------TANPRNPQLIELKNVLNRLLDALQARVGS---DMNEIQRVFNSYK 481

14903A_Tlp13 GGNLTARI-------TANPRNPQLIELKNVLNRLLDALQARVGS---DMNEIQRVFNSYK 481

FJ3124_Tlp13 GGNLTARI-------TANPRNPQLIELKNVLNKLLDVLQARVGS---DMNAIHKIFEEYK 481

R14_Tlp13 GGNLTARI-------TANPRNPQLIELKNVLNKLLDVLQARVGS---DMNAIHKIFEEYK 481

MTVDSCj16_Tlp13 GGNLTARI-------TANPRNPQLIELKNVLNKLLDVLQARVGS---DMNAIHKIFEEYK 481

14980A_Tlp13 GGNLTARI-------TANPRNPQLIELKNVLNKLLDVLQARVGS---DMNAIHKIFEEYK 481

* ** ****:***:***::**.** ::

FDAARGOS_295_Tlp1 ALDFSTEVFDAKGEVEITTNILGKEIKKMLVASSNFAKDLANQSEELKNSMQKLADGSNA 536

14980A_Tlp1 ALDFSTEVFDAKGEVEITTNILGKEIKKMLVASSNFAKDLANQSEELKNSMRKLADGSNA 536

CJ677CC527_Tlp1 ALDFSTEVFDAKGEVEITTNILGKEIKKMLVASSNFAKDLANQSEELKNSMQKLADGSNA 536

CJ677CC012_Tlp1 ALDFSTEVFDAKGEVEITTNILGKEIKKMLVASSNFAKDLANQSEELKNSMQKLADGSNA 536

4031_Tlp1 ALDFSTEVLDAKGEVEITTNILGKEIKKMLVASSNFAKDLANQSEELKNSMQKLADGSNA 536

81116_Tlp1 ALDFSTEVLDAKGEVEITTNILGKEIKKMLVASSNFAKDLANQSEELKNSMQKLADGSNA 536

35925B2_Tlp1 ALDFSTEVLDAKGEVEITTNILGKEIKKMLVASSNFAKDLANQSEELKNSMQKLADGSNA 536

M1_Tlp1 ALDFSTEVLDAKGEVEITTNILGKEIKKMLVASSNFAKDLANQSEELKNSMQKLADGSNA 536

PT14_Tlp1 ALDFSTEVLDAKGEVEITTNILGKEIKKMLVASSNFAKDLANQSEELKNSMQKLADGSNA 536

81-176_Tlp1 ALDFSTEVFNAKGEVEITTNILGKEIKKMLLASSNFAKDLANQSEELKNSMQKLADGSNA 536

CVMN29710_Tlp1 ALDFSTEVFNAKGEVEITTNILGKEIKKMLVASSNFAKDLANQSEELKNSMQKLADGSNA 536

FB1_Tlp1 ALDFSTEVFNAKGEVEITTNILGKEIKKMLVASSNFAKDLANQSEELKNSMQKLADGSNA 536

BG2108_Tlp1 ALDFSTEVFNAKGEVEITTNILGKEIKKMLVASSNFAKDLANQSEELKNSMQKLADGSNA 536

YF2108_Tlp1 ALDFSTEVFNAKGEVEITTNILGKEIKKMLVASSNFAKDLANQSEELKNSMQKLADGSNA 536

YH501_Tlp1 ALDFSTEVFNAKGEVEITTNILGKEIKKMLVASSNFAKDLANQSEELKNSMQKLADGSNA 536

RM4661_Tlp1 ALDFSTEVFNAKGEVEITTNILGKEIKKMLLASSNFAKDLANQSEELKNSMQKLADGSNA 536

F38011_Tlp1 ALDFSTEVFNAKGEVEITTNILGKEIKKMLLASSNFAKDLANQSEELKNSMQKLADGSNA 536

T1-21_Tlp1 ALDFSTEVFNAKGEVEITTNILGKEIKKMLLASSNFAKDLANQSEELKNSMQKLADGSNA 536

CG8421_Tlp1 ALDFSTEVFNAKGEVEITTNILGKEIKKMLLASSNFAKDLANQSKELKNSMQKLADGSNA 536

CJM1cam_Tlp1 ALDFSTEVFNAKGEVEITTNILGKEIKKMLLASSNFAKDLANQSKELKNSMQKLADGSNA 536

R14_Tlp1 ALDFSTEVFNAKGEVEITTNILGKEIKKMLLASSNFAKDLANQSKELKNSMQKLADGSNA 536

ICDCCJ07001_Tlp1 ALDFSTEVFNAKGEVEITTNILGKEIKKMLLASSNFAKDLANQSEELKNSMQKLADGSNA 536

RM3196_Tlp1 ALDFSTEVFNAKGEVEITTNILGKEIKKMLLASSNFAKDLANQSEELKNSMQKLADGSNA 536

NCTC11168_Tlp1 ALDFSTEVFNAKGEVEITTNILGKEIKKMLLASSNFAKDLANQSEELKNSMQKLADGSNA 536

00-2425_Tlp1 ALDFSTEVFNAKGEVEITTNILGKEIKKMLLASSNFAKDLANQSEELKNSMQKLADGSNA 536

IA3902_Tlp1 ALDFSTEVFNAKGEVEITTNILGKEIKKMLLASSNFAKDLANQSEELKNSMQKLADGSNA 536

RM1285_Tlp1 ALDFSTEVFNAKGEVEITTNILGKEIKKMLLASSNFAKDLANQSEELKNSMQKLADGSNA 536

00-0949_Tlp1 ALDFSTEVFNAKGEVEITTNILGKEIKKMLLASSNFAKDLANQSEELKNSMQKLADGSNA 536

01-1512_Tlp1 ALDFSTEVFNAKGEVEITTNILGKEIKKMLLASSNFAKDLANQSEELKNSMQKLADGSNA 536

FDAARGOS_422_Tlp1 ALDFSTEVFNAKGEVEITTNILGKEIKKMLLASSNFAKDLANQSEELKNSMQKLADGSNA 536

FORC_056_Tlp1 ALDFSTEVFNAKGEVEITTNILGKEIKKMLLASSNFAKDLANQSEELKNSMQKLADGSNA 536

32488_Tlp1 ALDFSTEVFNAKGEVEITTNILGKEIKKMLLASSNFAKDLANQSEELKNSMQKLADGSNA 536

CFSAN032806_Tlp1 ALDFSTEVFNAKGEVEITTNILGKEIKKMLLASSNFAKDLANQSEELKNSMQKLADGSNA 536

YH001_Tlp1 ALDFSTEVFNAKGEVEITTNILGKEIKKMLLASSNFAKDLANQSEELKNSMQKLADGSNA 536

00-6200_Tlp1 ALDFSTEVFNAKGEVEITTNILGKEIKKMLLASSNFAKDLANQSEELKNSMQKLADGSNA 536

RM1221_Tlp1 ALDFSTEVFNAKGEVEITTNILGKEIKKMLVASSNFAKDLANQSEELKNSMQKLADGSNA 536

S3_Tlp1 ALDFSTEVFNAKGEVEITTNILGKEIKKMLVASSNFAKDLANQSEELKNSMQKLADGSNA 536

FDAARGOS_421_Tlp1 ALDFSTEVFNAKGEVEITTNILGKEIKKMLVASSNFAKDLANQSEELKNSMQKLADGSNA 536

FJ3124_Tlp1 ALDFSTEVFNAKGEVEITTNILGKEIKKMLVASSNFAKDLANQSEELKNSMQKLADGSNA 536

00-1597_Tlp1 ALDFSTEVFNAKGEVEITTNILGKEIKKMLLASSNFAKDLANQSEELKNSMQKLADGSNA 536

15-537360_Tlp20 SLDFTTEVKDANGAVEVTTNALGQEIIKMLKQSSDFANALANESGKLQTAVQSLTTSSNS 495

76339_Tlp20 SLDFRNKLDNANGSVEVTTNALGDEIVKMLKQSSDFANHLASESSKLQSAVQNLTSSSNS 495

HC2-48_Tlp20 SLDFRNKLDNANGSVEVTTNALGDEIVKMLKQSSDFANHLASESSKLQSAVQNLTSSSNS 494

CFSAN032805_Tlp20 SLDFRNKLDNANGSVEVTTNALGDEIVKMLKQSSDFANHLASESSKLQSAVQNLTSSSNS 494

CVMN29710_Tlp20 SLDFRNKLDNANGSVEVTTNALGDEIVKMLKQSSDFANHLASESSKLQSAVQNLTSSSNS 493

YH501_Tlp20 SLDFRNKLDNANGSVEVTTNALGDEIVKMLKQSSDFANHLASESSKLQSAVQNLTSSSNS 497

CF2-75_Tlp20 SLDFRNKLDNANGSVEVTTNALGDEIVKMLKQSSDFANHLASESSKLQSAVQNLTSSSNS 498

CO2-160_Tlp20 SLDFRNKLDNANGSVEVTTNALGDEIVKMLKQSSDFANHLASESSKLQSAVQNLTSSSNS 495

CO2-160_Tlp20b SLDFRNKLDNANGSVEVTTNALGDEIVKMLKQSSDFANHLASESSKLQSAVQNLTSSSNS 495

RM5611_Tlp20 SLDFRNKLDNANGSVEVTTNALGDEIVKMLKQSSDFANHLASESSKLQSAVQNLTSSSNS 495

14903A_Tlp20 SLDFRNKLDNANGSVEVTTNALGDEIVKMLKQSSDFANHLASESSKLQSAVQNLTSSSNS 497

YH502_Tlp20 SLDFRNKLDNANGSVEVTTNALGDEIVKMLKQSSDFANHLASESSKLQSAVQNLTSSSNS 494

RM3196_Tlp23 SLDFTTEVKDANGAVEVTTNALGQEIIKMLKQSSDFANALANESGKLQTAVQSLTTSSNS 495

ICDCCJ07001_Tlp23 SLDFTTEVKDANGAVEVTTNALGQEIIKMLKQSSDFANALANESGKLQTAVQSLTTSSNS 495

RM1285_Tlp2 SLDFRNKLENASGSVELTTNALGDEIVKMLKQSSDFANALANESGKLQTAVQSLTTSSNS 494

CFSAN032806_Tlp2 SLDFRNKLENASGSVELTTNALGDEIVKMLKQSSDFANALANESGKLQTAVQSLTTSSNS 495

RM1221_Tlp2 SLDFRNKLENASGSVELTTNALGDEIVKMLKQSSDFANALANESGKLQTAVQSLTTSSNS 495

S3_Tlp2 SLDFRNKLENASGSVELTTNALGDEIVKMLKQSSDFANALANESGKLQTAVQSLTTSSNS 495

FDAARGOS_422_Tlp2 SLDFRNKLENASGSVELTTNALGDEIVKMLKQSSDFANALANESGKLQTAVQSLTTSSNS 495

81-176_Tlp2 SLDFRNKLENASGSVELTTNALGDEIVKMLKQSSDFANALANESGKLQTAVQSLTTSSNS 495

F38011_Tlp2 SLDFRNKLENASGSVELTTNALGDEIVKMLKQSSDFANALANESGKLQTAVQSLTTSSNS 495

NCTC11168_Tlp2 SLDFRNKLENASGSVELTTNALGDEIVKMLKQSSDFANALANESGKLQTAVQSLTTSSNS 495

MTVDSCj07_Tlp2 SLDFRNKLENASGSVELTTNALGDEIVKMLKQSSDFANALANESGKLQTAVQSLTTSSNS 495

CJM1cam_Tlp24 SLDFRNKLENASGSVELTTNALGDEIVKMLKQSSDFANALANESGKLQTAVQSLTTSSNS 492

M1_Tlp24 SLDFRNKLENASGSVELTTNALGDEIVKMLKQSSDFANALANESGKLQTAVQSLTTSSNS 491

81116_Tlp2 SLDFRNKLENASGSVELTTNALGDEIVKMLKQSSDFANALANESGKLQTAVQSLTTSSNS 495

4031_Tlp23 SLDFRNKLENASGSVELTTNALGDEIVKMLKQSSDFANALANESGKLQTAVQSLTTSSNS 494

CVMN29710_Tlp14 SLDFTTEVKDANGAVEVTTNALGQEIIKMLKQSSDFANHLASESSKLQSAVQNLTSSSNS 492

RM4661_Tlp14 SLDFRNKLDNASGNVEVTTNALGDEIVKMLKQSSDFANHLASESSKLQSAVQNLTSSSNS 492

MG1116_Tlp14 SLDFRNKLDNANGSVEVTTNALGDEIVKMLKQSSDFANHLASESSKLQSAVQNLTSSSNS 485

BG2108_Tlp14 SLDFRNKLDNANGSVEVTTNALGDEIVKMLKQSSDFANHLASESSKLQSAVQNLTSSSNS 424

YF2105_Tlp14 SLDFRNKLDNANGSVEVTTNALGDEIVKMLKQSSDFANHLASESSKLQSAVQNLTSSSNS 453

YH502_Tlp14 SLDFRNKLDNANGSVEVTTNALGDEIVKMLKQSSDFANHLASESSKLQSAVQNLTSSSNS 503

WA333_Tlp14 SLDFRNKLDNANGSVEVTTNALGDEIVKMLKQSSDFANHLASESSKLQSAVQNLTSSSNS 481

BP3181_Tlp14 SLDFRNKLDNANGSVEVTTNALGDEIVKMLKQSSDFANHLASESSKLQSAVQNLTSSSNS 492

ZV1224_Tlp14a SLDFRNKLDNANGSVEVTTNALGDEIVKMLKQSSDFANHLASESSKLQSAVQNLTSSSNS 492

ZV1224_Tlp14b SLDFRNKLDNANGSVEVTTNALGDEIVKMLKQSSDFANHLASESSKLQSAVQNLTSSSNS 492

YH503_Tlp14 SLDFRNKLDNANGSVEVTTNALGDEIVKMLKQSSDFANHLASESSKLQSAVQNLTSSSNS 503

14903A_Tlp14 SLDFRNKLDNANGSVEVTTNALGDEIVKMLKQSSDFANHLASESSKLQSAVQNLTSSSNS 492

OR12_Tlp14 SLDFRNKLDNANGSVEVTTNALGDEIVKMLKQSSDFANHLASESSKLQSAVQNLTSSSNS 492

CFSAN032805_Tlp14 SLDFRNKLDNANGSVEVTTNALGDEIVKMLKQSSDFANHLASESSKLQSAVQNLTSSSNS 503

BFR-CA-9557_Tlp14 SLDFRNKLDNANGSVEVTTNALGDEIVKMLKQSSDFANHLASESSKLQSAVQNLTSSSNS 492

15-537360_Tlp14 SLDFRNKLDNANGSVEVTTNALGDEIVKMLKQSSDFANHLASESSKLQSAVQNLTSSSNS 492

YH501_Tlp14 SLDFRNKLDNANGSVEVTTNALGDEIVKMLKQSSDFANHLASESSKLQSAVQNLTSSSNS 492

T1-21_Tlp19b SLDFRNKLENASGSVELTTNALGDEIVKMLKQSSDFANALANESGKLQTAVQSLTTSSNS 447

CG8421_Tlp14 SLDFRNKLENASGSVELTTNALGDEIVKMLKQSSDFANALANESGKLQTAVQSLTTSSNS 486

MTVDSCj16_Tlp14 SLDFRNKLENASGSVELTTNALGDEIVKMLKQSSDFANALANESGKLQTAVQSLTTSSNS 492

01-1512_Tlp14 SLDFRNKLENASGSVELTTNALGDEIVKMLKQSSDFANALANESGKLQTAVQSLTTSSNS 492

00-0949_Tlp14 SLDFRNKLENASGSVELTTNALGDEIVKMLKQSSDFANALANESGKLQTAVQSLTTSSNS 492

MTVDSCj13_Tlp14 SLDFRNKLENASGSVELTTNALGDEIVKMLKQSSDFANALANESGKLQTAVQSLTTSSNS 492

S3_Tlp14 SLDFRNKLENASGSVELTTNALGDEIVKMLKQSSDFANALANESGKLQTAVQSLTTSSNS 492

PT14_Tlp14 SLDFRNKLENASGSVELTTNALGDEIVKMLKQSSDFANALANESGKLQTAVQSLTTSSNS 492

14980A_Tlp14 SLDFRNKLENASGSVELTTNALGDEIVKMLKQSSDFANALANESGKLQTAVQSLTTSSNS 508

FJ3124_Tlp14 SLDFRNKLENASGSVELTTNALGDEIVKMLKQSSDFANALANESGKLQTAVQSLTTSSNS 492

00-1597_Tlp14 SLDFTTEVKDANGAVEVTTNALGQEIIKMLKQSSDFANALANESGKLQTAVQSLTTSSNS 492

R14_Tlp14 SLDFTTEVKDANGAVEVTTNALGQEIIKMLKQSSDFANALANESGKLQTAVQSLTTSSNS 492

CG8421_Tlp25 SLDFRNKLENASGSVELTTNALGDEIVKMLKQSSDFANALANESGKLQTAVQSLTTSSNS 283

HC2-48_Tlp3 SLDFRNKLDNANGSVEVTTNALGDEIVKMLKQSSDFANHLARKFKTSKCSSKPYFI---- 483

35925B2_Tlp3 SLDFTTEVKDANGAVEVTTNALGQEIIKMLKQSSDFANALANESGKLQTAVQSLTTSSNS 501

14980A_Tlp3 SLDFTTEVKDANGAVEVTTNALGQEIIKMLKQSSDFANALANESGKLQTAVQSLTTSSNS 498

00-1597_Tlp3b SLDFTTEVKDANGAVEVTTNALGQEIIKMLKQSSDFANALANESGKLQTAVQSLTTSSNS 487

ICDCCJ07001_Tlp3 SLDFTTEVKDANGAVEVTTNALGQEIIKMLKQSSDFANALANESGKLQTAVQSLTTSSNS 487

RM3196_Tlp3 SLDFTTEVKDANGAVEVTTNALGQEIIKMLKQSSDFANALANESGKLQTAVQSLTTSSNS 487

RM1875_Tlp3 SLDFRNKLDNANGSVEVTTNALGMKLVKMLKQSSDFANHLASESSKLQSAVQNLTSSSNS 487

CF2-75_Tlp3 SLDFRNKLDNANGSVEVTTNALGDEIVKMLKQSSDFANHLASESSKLQSAVQNLTSSSNS 498

RM5611_Tlp3 SLDFRNKLDNANGSVEVTTNALGDEIVKMLKQSSDFANHLASESSKLQSAVQNLTSSSNS 487

MTVDSCj16_Tlp3 SLDFRNKLENASGSVELTTNALGDEIVKMLKQSSDFANALANESGKLQTAVQSLTTSSNS 487

01-1512_Tlp3 SLDFRNKLENASGSVELTTNALGDEIVKMLKQSSDFANALANESGKLQTAVQSLTTSSNS 487

MTVDSCj13_Tlp3 SLDFRNKLENASGSVELTTNALGDEIVKMLKQSSDFANALANESGKLQTAVQSLTTSSNS 487

32488_Tlp3a SLDFRNKLENASGSVELTTNALGDEIVKMLKQSSDFANALANESGKLQTAVQSLTTSSNS 487

81116_Tlp3 SLDFRNKLENASGSVELTTNALGDEIVKMLKQSSDFANALANESGKLQTAVQSLTTSSNS 487

32488_Tlp3b SLDFRNKLENASGSVELTTNALGDEIVKMLKQSSDFANALANESGKLQTAVQSLTTSSNS 487

FB1_Tlp3 SLDFRNKLENASGSVELTTNALGDEIVKMLKQSSDFANALANESGKLQTAVQSLTTSSNS 487

PT14_Tlp3 SLDFRNKLENASGSVELTTNALGDEIVKMLKQSSDFANALANESGKLQTAVQSLTTSSNS 487

00-6200_Tlp3a SLDFRNKLENASGSVELTTNALGDEIVKMLKQSSDFANALANESGKLQTAVQSLTTSSNS 487

RM1221_Tlp3 SLDFRNKLENASGSVELTTNALGDEIVKMLKQSSDFANALANESGKLQTAVQSLTTSSNS 487

S3_Tlp3 SLDFRNKLENASGSVELTTNALGDEIVKMLKQSSDFANALANESGKLQTAVQSLTTSSNS 487

FDAARGOS_421_Tlp3 SLDFRNKLENASGSVELTTNALGDEIVKMLKQSSDFANALANESGKLQTAVQSLTTSSNS 498

CFSAN032806_Tlp3 SLDFRNKLENASGSVELTTNALGDEIVKMLKQSSDFANALANESGKLQTAVQSLTTSSNS 498

IA3901_Tlp3b SLDFRNKLENASGSVELTTNALGDEIVKMLKQSSDFANALANESGKLQTAVQSLTTSSNS 487

00-6200_Tlp3b SLDFRNKLENASGSVELTTNALGDEIVKMLKQSSDFANALANESGKLQTAVQSLTTSSNS 487

BCW_6290_Tlp3b SLDFRNKLENASGSVELTTNALGDEIVKMLKQSSDFANALANESGKLQTAVQSLTTSSNS 487

00-2425_Tlp3a SLDFRNKLENASGSVELTTNALGDEIVKMLKQSSDFANALANESGKLQTAVQSLTTSSNS 487

00-2425_Tlp3b SLDFRNKLENASGSVELTTNALGDEIVKMLKQSSDFANALANESGKLQTAVQSLTTSSNS 487

YH001_Tlp3a SLDFRNKLENASGSVELTTNALGDEIVKMLKQSSDFANALANESGKLQTAVQSLTTSSNS 487

YH001_Tlp3b SLDFRNKLENASGSVELTTNALGDEIVKMLKQSSDFANALANESGKLQTAVQSLTTSSNS 487

00-0949_Tlp3 SLDFRNKLENASGSVELTTNALGDEIVKMLKQSSDFANALANESGKLQTAVQSLTTSSNS 487

NCTC11168_Tlp3 SLDFRNKLENASGSVELTTNALGDEIVKMLKQSSDFANALANESGKLQTAVQSLTTSSNS 498

F38011_Tlp3 SLDFRNKLENASGSVELTTNALGDEIVKMLKQSSDFANALANESGKLQTAVQSLTTSSNS 487

RM1285_Tlp3 SLDFRNKLENASGSVELTTNALGDEIVKMLKQSSDFANALANESGKLQTAVQSLTTSSNS 487

FDAARGOS_422_Tlp3 SLDFRNKLENASGSVELTTNALGDEIVKMLKQSSDFANALANESGKLQTAVQSLTTSSNS 498

MTVDSCj07_Tlp3 SLDFRNKLENASGSVELTTNALGDEIVKMLKQSSDFANALANESGKLQTAVQSLTTSSNS 487

IA3901_Tlp3a SLDFRNKLENASGSVELTTNALGDEIVKMLKQSSDFANALANESGKLQTAVQSLTTSSNS 487

BCW_6290_Tlp3a SLDFRNKLENASGSVELTTNALGDEIVKMLKQSSDFANALANESGKLQTAVQSLTTSSNS 487

T1-21_Tlp19a SLDFRNKLENASGSVELTTNALGDEIVKMLKQSSDFANALANESGKLQTAVQSLTTSSNS 442

CJ677CC012_Tlp3 ------------------------------------------------------------ 420

CJM1cam_Tlp3 SLDFRNKLENASGSVELTTNALGDEIVKMLKQSSDFANALANESGKLQTAVQSLTTSSNS 487

M1_Tlp3 SLDFRNKLENASGSVELTTNALGDEIVKMLKQSSDFANALANESGKLQTAVQSLTTSSNS 487

4031_Tlp3 SLDFRNKLENASGSVELTTNALGDEIVKMLKQSSDFANALANESGKLQTAVQSLTTSSNS 487

R14_Tlp3 SLDFRNKLENASGSVELTTNALGNEIVKMLKQSSDFANALANESGKLQTAVQSLTTSSNS 487

FDAARGOS_295_Tlp21 SLDFTTEVKDANGAVELTTNALGDEIIKMLKQSSDFANALANESGKLQTAVQSLTTSSNS 489

FORC_046_Tlp4 SLDFTTEVKDANGAVEVTTNALGQEIIKMLKQSSDFANALANESGKLQTAVQSLTTSSNS 501

FDAARGOS_422_Tlp4 SLDFTTEVKDANGAVEVTTNALGQEIIKMLKQSSDFANALANESGKLQTAVQSLTTSSNS 501

ICDCCJ07001_Tlp4 SLDFRNKLDNANGSVEVTTNALGDEIVKMLKQSSDFANHLASESSKLQSAVQNLTSSSNS 501

RM3196_Tlp4 SLDFRNKLDNANGSVEVTTNALGDEIVKMLKQSSDFANHLASESSKLQSAVQNLTSSSNS 501

T1-21_Tlp4 SLDFRNKLENASGSVELTTNALGDEIVKMLKQSSDFANALANESGKLQTAVQSLTTSSNS 501

F38011_Tlp4 SLDFRNKLENASGSVELTTNALGDEIVKMLKQSSDFANALANESGKLQTAVQSLTTSSNS 501

HF5-4A-4_Tlp22 SLDFRNKLENASGSVELTTNALGDEIVKMLKQSSDFANALANESGKLQTAVQSLTTSSNS 447

00-0949_Tlp4 SLDFRNKLENASGSVELTTNALGDEIVKMLKQSSDFANALANESGKLQTAVQSLTTSSNS 500

01-1512_Tlp4 SLDFRNKLENASGSVELTTNALGDEIVKMLKQSSDFANALANESGKLQTAVQSLTTSSNS 501

81-176_Tlp4 SLDFRNKLENASGSVELTTNALGDEIVKMLKQSSDFANALANESGKLQTAVQSLTTSSNS 501

32488_Tlp4 SLDFRNKLENASGSVELTTNALGDEIVKMLKQSSDFANALANESGKLQTAVQSLTTSSNS 501

NCTC11168_Tlp4 SLDFRNKLENASGSVELTTNALGDEIVKMLKQSSDFANALANESGKLQTAVQSLTTSSNS 501

CFSAN032806_Tlp4 SLDFRNKLENASGSVELTTNALGDEIVKMLKQSSDFANALANESGKLQTAVQSLTTSSNS 501

81116_Tlp4 SLDFRNKLENAGGSVELTTNALGDEIVKMLKQSSDFANALANESGKLQTAVQSLTTSSNS 501

RM1285_Tlp12 SLDFRNKLENASGSVELTTNALGDEIVKMLKQSSDFANALANESGKLQTAVQSLTTSSNS 497

PT14_Tlp12 SLDFRNKLENASGSVELTTNALGDEIVKMLKQSSDFANALANESGKLQTAVQSLTTSSNS 498

MTVJDCj07_Tlp12 SLDFRNKLENASGSVELTTNALGDEIVKMLKQSSDFANALANESGKLQTAVQSLTTSSNS 498

RM1221_Tlp12 SLDFRNKLENASGSVELTTNALGDEIVKMLKQSSDFANALANESGKLQTAVQSLTTSSNS 498

FDAARGOS_421_Tlp12 SLDFRNKLENASGSVELTTNALGDEIVKMLKQSSDFANALANESGKLQTAVQSLTTSSNS 498

35925B2_Tlp12 SLDFTTEVKDANGAVEVTTNALGQEIIKMLKQSSDFANALANESGKLQTAVQSLTTSSNS 498

CJM1cam_Tlp12 SLDFTTEVKDANGAVEVTTNALGQEIIKMLKQSSDFANALANESGKLQTAVQSLTTSSNS 498

M1_Tlp12 SLDFTTEVKDANGAVEVTTNALGQEIIKMLKQSSDFANALANESGKLQTAVQSLTTSSNS 498

S3_Tlp12 SLDFTTEVKDANGAVEVTTNALGQEIIKMLKQSSDFANALANESGKLQTAVQSLTTSSNS 498

00-1597_Tlp12 SLDFTTEVKDANGAVEVTTNALGQEIIKMLKQSSDFANALANESGKLQTAVQSLTTSSNS 498

R14_Tlp12 SLDFTTEVKDANGAVEVTTNALGQEIIKMLKQSSDFANALANESGKLQTAVQSLTTSSNS 498

RM1875_Tlp15 SLDFRNKLDNANGSVEVTTNALGDEIVKMLKQSSDFANHLASESSKLQSAVQNLTSSSNS 415

YH503_Tlp16 SLDFRNKLDNANGSVEVTTNALGDEIVKMLKQSSDFANHLASESSKLQSAVQNLTSSSNS 415

FB1_Tlp16 SLDFRNKLDNANGSVEVTTNALGDEIVKMLKQSSDFANHLASESSKLQSAVQNLTSSSNS 415

BFR-CA-9557_Tlp16 SLDFRNKLDNANGSVEVTTNALGDEIVKMLKQSSDFANHLASESSKLQSAVQNLTSSSNS 414

15-537360_Tlp16 SLDFRNKLDNANGSVEVTTNALGDEIVKMLKQSSDFANHLASESSKLQSAVQNLTSSSNS 415

OR12_Tlp16 SLDFRNKLDNANGSVEVTTNALGDEIVKMLKQSSDFANHLASESSKLQSAVQNLTSSSNS 415

YH502_Tlp16 SLDFRNKLDNANGSVEVTTNALGDEIVKMLKQSSDFANHLASESSKLQSAVQNLTSSSNS 415

14903A_Tlp16 SLDFRNKLDNANGSVEVTTNALGDEIVKMLKQSSDFANHLASESSKLQSAVQNLTSSSNS 415

HC2-48_Tlp16 SLDFRNKLDNANGSVEVTTNALGDEIVKMLKQSSDFANHLASESSKLQSAVQNLTSSSNS 414

RM5611_Tlp16 SLDFRNKLDNANGSVEVTTNALGDEIVKMLKQSSDFANHLASESSKLQSAVQNLTSSSNS 415

00-2425_Tlp11 SLDFRNKLENASGSVELTTNALGDEIVKMLKQSSDFANALANESGKLQTAVQSLTTSSNS 542

00-6200_Tlp11 SLDFRNKLENASGSVELTTNALGDEIVKMLKQSSDFANALANESGKLQTAVQSLTTSSNS 542

YH001_Tlp11 SLDFRNKLENASGSVELTTNALGDEIVKMLKQSSDFANALANESGKLQTAVQSLTTSSNS 542

IA3902_Tlp11 SLDFTTEVKDANGAVELTTNALGDEIVKMLKQSSDFANALANESGKLQTAVQSLTTSSNS 542

BCW_6290_Tlp11 SLDFTTEVKDANGAVELTTNALGDEIVKMLKQSSDFANALANESGKLQTAVQSLTTSSNS 542

76339_Tlp18 SLDFRNKLDNANGSVEVTTNALGDEIVKMLKQSSDFANHLASESSKLQSAVQNLTSSSNS 541

4031_Tlp17 SLDFRNKLENASGSVELTTNALGDEIVKMLKQSSDFANALANESGKLQTAVQSLTTSSNS 542

MTVDSCj13_Tlp13 SLDFRNKLENASGSVELTTNALGDEIVKMLKQSSDFANALANESGKLQTAVQSLTTSSNS 541

RM1875_Tlp13 SLDFRNKLDNANGSVEVTTNALGDEIVKMLKQSSDFANHLASESSKLQSAVQNLTSSSNS 541

CF2-75_Tlp13 SLDFRNKLDNANGSVEVTTNALGDEIVKMLKQSSDFANHLASESSKLQSAVQNLTSSSNS 541

15-537360_Tlp13 SLDFRNKLDNANGSVEVTTNALGDEIVKMLKQSSDFANHLASESSKLQSAVQNLTSSSNS 541

CVMN29710_Tlp13 SLDFRNKLDNANGSVEVTTNALGDEIVKMLKQSSDFANHLASESSKLQSAVQNLTSSSNS 541

FB1_Tlp13 SLDFRNKLDNANGSVEVTTNALGDEIVKMLKQSSDFANHLASESSKLQSAVQNLTSSSNS 541

CFCAN032805_Tlp13 SLDFRNKLDNANGSVEVTTNALGDEIVKMLKQSSDFANHLASESSKLQSAVQNLTSSSNS 541

BG2108_Tlp13 SLDFRNKLDNANGSVEVTTNALGDEIVKMLKQSSDFANHLASESSKLQSAVQNLTSSSNS 541

YF2105_Tlp13 SLDFRNKLDNANGSVEVTTNALGDEIVKMLKQSSDFANHLASESSKLQSAVQNLTSSSNS 541

YH503_Tlp13 SLDFRNKLDNANGSVEVTTNALGDEIVKMLKQSSDFANHLASESSKLQSAVQNLTSSSNS 541

BFRCA9557_Tlp13 SLDFRNKLDNANGSVEVTTNALGDEIVKMLKQSSDFANHLASESSKLQSAVQNLTSSSNS 541

YH502_Tlp13 SLDFRNKLDNANGSVEVTTNALGDEIVKMLKQSSDFANHLASESSKLQSAVQNLTSSSNS 541

OR12_Tlp13 SLDFTTEVKDANGAVEVTTNALGQEIIKMLKQSSDFANALANESSKLQTAVQSLTTSSNS 541

00-1597_Tlp13 SLDFTTEVKDANGAVEVTTNALGQEIIKMLKQSSDFANALANESGKLQTAVQSLTTSSNS 541

14903A_Tlp13 SLDFTTEVKDANGAVEVTTNALGQEIIKMLKQSSDFANALANESGKLQTAVQSLTTSSNS 541

FJ3124_Tlp13 SLDFRNKLENASGSVELTTNALGDEIVKMLKQSSDFANALANESGKLQTAVQSLTTSSNS 541

R14_Tlp13 SLDFRNKLENASGSVELTTNALGNEIVKMLKQSSDFANALANESGKLQTAVQSLTTSSNS 541

MTVDSCj16_Tlp13 SLDFRNKLENASGSVELTTNALGDEIVKMLKQSSDFANALANESGKLQTAVQSLTTSSNS 541

14980A_Tlp13 SLDFRNKLENASGSVELTTNALGDEIVKMLKQSSDFANALANESGKLQTAVQSLTTSSNS 541

FDAARGOS_295_Tlp1 QASSLEQSAAAVEEINSSMQNVSGKTVEVASQADDIKNIVNVIKDIAEQTNLLALNAAIE 596

14980A_Tlp1 QASSLEQSAAAVEEINSSMQNVSGKTVEVASQADDIKNIVNVIKDIAEQTNLLALNAAIE 596

CJ677CC527_Tlp1 QASSLEQSAAAVEEINSSMQNVSGKTVEVASQADDIKNIVNVIKDIAEQTNLLALNAAIE 596

CJ677CC012_Tlp1 QASSLEQSAAAVEEINSSMQNVSGKTVEVASQADDIKNIVNVIKDIAEQTNLLALNAAIE 596

4031_Tlp1 QASSLEQSAAAVEEINSSMQNVSGKTVEVASQADDIKNIVNVIKDIAEQTNLLALNAAIE 596

81116_Tlp1 QASSLEQSAAAVEEINSSMQNVSGKTVEVASQADDIKNIVNVIKDIAEQTNLLALNAAIE 596

35925B2_Tlp1 QASSLEQSAAAVEEINSSMQNVSGKTVEVASQADDIKNIVNVIKDIAEQTNLLALNAAIE 596

M1_Tlp1 QASSLEQSAAAVEEINSSMQNVSGKTVEVASQADDIKNIVNVIKDIAEQTNLLALNAAIE 596

PT14_Tlp1 QASSLEQSAAAVEEINSSMQNVSGKTVEVASQADDIKNIVNVIKDIAEQTNLLALNAAIE 596

81-176_Tlp1 QASSLEQSAAAVEEINSSMQNVSGKTVEVASQADDIKNIVNVIKDIAEQTNLLALNAAIE 596

CVMN29710_Tlp1 QASSLEQSAAAVEEINSSMQNVSGKTVEVASQADDIKNIVNVIKDIAEQTNLLALNAAIE 596

FB1_Tlp1 QASSLEQSAAAVEEINSSMQNVSGKTVEVASQADDIKNIVNVIKDIAEQTNLLALNAAIE 596

BG2108_Tlp1 QASSLEQSAAAVEEINSSMQNVSGKTVEVASQADDIKNIVNVIKDIAEQTNLLALNAAIE 596

YF2108_Tlp1 QASSLEQSAAAVEEINSSMQNVSGKTVEVASQADDIKNIVNVIKDIAEQTNLLALNAAIE 596

YH501_Tlp1 QASSLEQSAAAVEEINSSMQNVSGKTVEVASQADDIKNIVNVIKDIAEQTNLLALNAAIE 596

RM4661_Tlp1 QASSLEQSAAAVEEINSSMQNVSGKTVEVASQADDIKNIVNVIKDIAEQTNLLALNAAIE 596

F38011_Tlp1 QASSLEQSAAAVEEINSSMQNVSGKTVEVASQADDIKNIVNVIKDIAEQTNLLALNAAIE 596

T1-21_Tlp1 QASSLEQSAAAVEEINSSMQNVSGKTVEVASQADDIKNIVNVIKDIAEQTNLLALNAAIE 596

CG8421_Tlp1 QASSLEQSAAAVEEINSSMQNVSGKTVEVASQADDIKNIVNVIKDIAEQTNLLALNAAIE 596

CJM1cam_Tlp1 QASSLEQSAAAVEEINSSMQNVSGKTVEVASQADDIKNIVNVIKDIAEQTNLLALNAAIE 596

R14_Tlp1 QASSLEQSAAAVEEINSSMQNVSGKTVEVASQADDIKNIVNVIKDIAEQTNLLALNAAIE 596

ICDCCJ07001_Tlp1 QASSLEQSAAAVEEINSSMQNVSGKTVEVASQADDIKNIVNVIKDIAEQTNLLALNAAIE 596

RM3196_Tlp1 QASSLEQSAAAVEEINSSMQNVSGKTVEVASQADDIKNIVNVIKDIAEQTNLLALNAAIE 596

NCTC11168_Tlp1 QASSLEQSAAAVEEINSSMQNVSGKTVEVASQADDIKNIVNVIKDIAEQTNLLALNAAIE 596

00-2425_Tlp1 QASSLEQSAAAVEEINSSMQNVSGKTVEVASQADDIKNIVNVIKDIAEQTNLLALNAAIE 596

IA3902_Tlp1 QASSLEQSAAAVEEINSSMQNVSGKTVEVASQADDIKNIVNVIKDIAEQTNLLALNAAIE 596

RM1285_Tlp1 QASSLEQSAAAVEEINSSMQNVSGKTVEVASQADDIKNIVNVIKDIAEQTNLLALNAAIE 596

00-0949_Tlp1 QASSLEQSAAAVEEINSSMQNVSGKTVEVASQADDIKNIVNVIKDIAEQTNLLALNAAIE 596

01-1512_Tlp1 QASSLEQSAAAVEEINSSMQNVSGKTVEVASQADDIKNIVNVIKDIAEQTNLLALNAAIE 596

FDAARGOS_422_Tlp1 QASSLEQSAAAVEEINSSMQNVSGKTVEVASQADDIKNIVNVIKDIAEQTNLLALNAAIE 596

FORC_056_Tlp1 QASSLEQSAAAVEEINSSMQNVSGKTVEVASQADDIKNIVNVIKDIAEQTNLLALNAAIE 596

32488_Tlp1 QASSLEQSAAAVEEINSSMQNVSGKTVEVASQADDIKNIVNVIKDIAEQTNLLALNAAIE 596

CFSAN032806_Tlp1 QASSLEQSAAAVEEINSSMQNVSGKTVEVASQADDIKNIVNVIKDIAEQTNLLALNAAIE 596

YH001_Tlp1 QASSLEQSAAAVEEINSSMQNVSGKTVEVASQADDIKNIVNVIKDIAEQTNLLALNAAIE 596

00-6200_Tlp1 QASSLEQSAAAVEEINSSMQNVSGKTVEVASQADDIKNIVNVIKDIAEQTNLLALNAAIE 596

RM1221_Tlp1 QASSLEQSAAAVEEINSSMQNVSGKTVEVASQADDIKNIVNVIKDIAEQTNLLALNAAIE 596

S3_Tlp1 QASSLEQSAAAVEEINSSMQNVSGKTVEVASQADDIKNIVNVIKDIAEQTNLLALNAAIE 596

FDAARGOS_421_Tlp1 QASSLEQSAAAVEEINSSMQNVSGKTVEVASQADDIKNIVNVIKDIAEQTNLLALNAAIE 596

FJ3124_Tlp1 QASSLEQSAAAVEEINSSMQNVSGKTVEVASQADDIKNIVNVIKDIAEQTNLLALNAAIE 596

00-1597_Tlp1 QASSLEQSAAAVEEINSSMQNVSGKTVEVASQADDIKNIVNVIKDIAEQTNLLALNAAIE 596

15-537360_Tlp20 QAASLEETAAALEEITSSMQNVSVKTSDVITQSEEIKNVTGIIGDIADQINLLALNAAIE 555

76339_Tlp20 QAASLEETAAALEEITSSMQNVSVKTSDVITQSEEIKNVTGIIGDIADQINLLALNAAIE 555

HC2-48_Tlp20 QAASLEETAAALEEITSSMQNVSVKTRCY------------------------------- 523

CFSAN032805_Tlp20 QAASLEETAAALEEITSSMQNVSVKTSDVITQSEEIKNVTGIIGDIADQINLLALNAAIE 554

CVMN29710_Tlp20 QAASLEETAAALEEITSSMQNVSVKTSDVITQSEEIKNVTGIIGDIADQINLLALNAAIE 553

YH501_Tlp20 QAASLEETAAALEEITSSMQNVSVKTSDVITQSEEIKNVTGIIGDIADQINLLALNAAIE 557

CF2-75_Tlp20 QAASLEETAAALEEITSSMQNVSVKTSDVITQSEEIKNVTGIIGDIADQINL-------- 550

CO2-160_Tlp20 QAASLEETAAALEEITSSMQNVSVKTSDVITQSEEIKNVTGIIGDIADQINLLALNAAIE 555

CO2-160_Tlp20b QAASLEETAAALEEITSSMQNVSVKTSDVITQSEEIKNVTGIIGDIADQINLLALNAAIE 555

RM5611_Tlp20 QAASLEETAAALEEITSSMQNVSVKTSDVITQSEEIKNVTGIIGDIADQINLLALNAAIE 555

14903A_Tlp20 QAASLEETAAALEEITSSMQNVSVKTSDVITQSEEIKNVTGIIGDIADQINLLALNAAIE 557

YH502_Tlp20 QAASLEETAAALEEITSSMQNVSVKTSDVITQSEEIKNVTGIIGDIADQINLLALNAAIE 554

RM3196_Tlp23 QAQSLEETAAALEEITSSMQNVSVKTSDVITQSEEIKNVTGIIGDIADQINLLALNAAIE 555

ICDCCJ07001_Tlp23 QAQSLEETAAALEEITSSMQNVSVKTSDVITQSEEIKNVTGIIGDIADQINLLALNAAIE 555

RM1285_Tlp2 QAQSLEETAAALEEITSSMQNVSVKTSDVITQSEEIKNVTGIIGDIADQINLLALNAAIE 554

CFSAN032806_Tlp2 QAQSLEETAAALEEITSSMQNVSVKTSDVITQSEEIKNVTGIIGDIADQINLLALNAAIE 555

RM1221_Tlp2 QAQSLEETAAALEEITSSMQNVSVKTSDVITQSEEIKNVTGIIGDIADQINLLALNAAIE 555

S3_Tlp2 QAQSLEETAAALEEITSSMQNVSVKTSDVITQSEEIKNVTGIIGDIADQINLLALNAAIE 555

FDAARGOS_422_Tlp2 QAQSLEETAAALEEITSSMQNVSVKTSDVITQSEEIKNVTGIIGDIADQINLLALNAAIE 555

81-176_Tlp2 QAQSLEETAAALEEITSSMQNVSVKTSDVITQSEEIKNVTGIIGDIADQINLLALNAAIE 555

F38011_Tlp2 QAQSLEETAAALEEITSSMQNVSVKTSDVITQSEEIKNVTGIIGDIADQINLLALNAAIE 555

NCTC11168_Tlp2 QAQSLEETAAALEEITSSMQNVSVKTSDVITQSEEIKNVTGIIGDIADQINLLALNAAIE 555

MTVDSCj07_Tlp2 QAQSLEETAAALEEITSSMQNVSVKTSDVITQSEEIKNVTGIIGDIADQINLLALNAAIE 555

CJM1cam_Tlp24 QAQSLEETAAALEEITSSMQNVSVKTSDVITQSEEIKNVTGIIGDIADQINLLALNAAIE 552

M1_Tlp24 QAQSLEETAAALEEITSSMQNVSVKTSDVITQSEEIKNVTGIIGDIADQINLLALNAAIE 551

81116_Tlp2 QAQSLEETAAALEEITSSMQNVSVKTSDVITQSEEIKNVTGIIGDIADQINLLALNAAIE 555

4031_Tlp23 QAQSLEETAAALEEITSSMQNVSVKTSDVITQSEEIKNVTGIIGDIADQINLLALNAAIE 554

CVMN29710_Tlp14 QAASLEETAAALEEITSSMQNVSVKTSDVITQSEEIKNVTGIIGDIADQINLLALNAAIE 552

RM4661_Tlp14 QAASLEETAAALEEITSSMQNVSVKTSDVITQSEEIKNVTGIIGDIADQINLLALNAAIE 552

MG1116_Tlp14 QAASLEETAAALEEITSSMQNVSVKTSDVITQSEEIKNVTGIIGDIADQINLLALNAAIE 545

BG2108_Tlp14 QAASLEETAAALEEITSSMQNVSVKTSDVITQSEEIKNVTGIIGDIADQINLLALNAAIE 484

YF2105_Tlp14 QAASLEETAAALEEITSSMQNVSVKTSDVITQSEEIKNVTGIIGDIADQINLLALNAAIE 513

YH502_Tlp14 QAASLEETAAALEEITSSMQNVSVKTSDVITQSEEIKNVTGIIGDIADQINLLALNAAIE 563

WA333_Tlp14 QAASLEETAAALEEITSSMQNVSVKTSDVITQV--------------------------- 514

BP3181_Tlp14 QAASLEETAAALEEITSSMQNVSVKTSDVITQSEEIKNVTGIIGDIADQINLLALNAAIE 552

ZV1224_Tlp14a QAASLEETAAALEEITSSMQNVSVKTSDVITQSEEIKNVTGIIGDIADQINLLALNAAIE 552

ZV1224_Tlp14b QAASLEETAAALEEITSSMQNVSVKTSDVITQSEEIKNVTGIIGDIADQINLLALNAAIE 552

YH503_Tlp14 QAASLEETAAALEEITSSMQNVSVKTSDVITQSEEIKNVTGIIGDIADQINLLALNAAIE 563

14903A_Tlp14 QAASLEETAAALEEITSSMQNVSVKTSDVITQSEEIKNVTGIIGDIADQINLLALNAAIE 552

OR12_Tlp14 QAASLEETAAALEEITSSMQNVSVKTSDVITQSEEIKNVTGIIGDIADQINLLALNAAIE 552

CFSAN032805_Tlp14 QAASLEETAAALEEITSSMQNVSVKTSDVITQSEEIKNVTGIIGDIADQINLLALNAAIE 563

BFR-CA-9557_Tlp14 QAASLEETAAALEEITSSMQNVSVKTSDVITQSEEIKNVTGIIGDIADQINLLALNAAIE 552

15-537360_Tlp14 QAASLEETAAALEEITSSMQNVSVKTSDVITQSEEIKNVTGIIGDIADQINLLALNAAIE 552

YH501_Tlp14 QAASLEETAAALEEITSSMQNVSVKTSDVITQSEEIKNVTGIIGDIADQINLLALNAAIE 552

T1-21_Tlp19b QAQSLEETAAALEEITSSMQNVSVKTSDVITQSEEIKNVTGIIGDIADQINLLALNAAIE 507

CG8421_Tlp14 QAQSLEETAAALEEITSSMQNVSVKTSDVITQSEEIKNVTGIIGDIADQINLLALNAAIE 546

MTVDSCj16_Tlp14 QAQSLEETAAALEEITSSMQNVSVKTSDVITQSEEIKNVTGIIGDIADQINLLALNAAIE 552

01-1512_Tlp14 QAQSLEETAAALEEITSSMQNVSVKTSDVITQSEEIKNVTGIIGDIADQINLLALNAAIE 552

00-0949_Tlp14 QAQSLEETAAALEEITSSMQNVSVKTSDVITQSEEIKNVTGIIGDIADQINLLALNAAIE 552

MTVDSCj13_Tlp14 QAQSLEETAAALEEITSSMQNVSVKTSDVITQSEEIKNVTGIIGDIADQINLLALNAAIE 552

S3_Tlp14 QAQSLEETAAALEEITSSMQNVSVKTSDVITQSEEIKNVTGIIGDIADQINLLALNAAIE 552

PT14_Tlp14 QAQSLEETAAALEEITSSMQNVSVKTSDVITQSEEIKNVTGIIGDIADQINLLALNAAIE 552

14980A_Tlp14 QAQSLEETAAALEEITSSMQNVSVKTSDVITQSEEIKNVTGIIGDIADQINLLALNAAIE 568

FJ3124_Tlp14 QAQSLEETAAALEEITSSMQNVSVKTSDVITQSEEIKNVTGIIGDIADQINLLALNAAIE 552

00-1597_Tlp14 QAQSLEETAAALEEITSSMQNVSVKTSDVITQSEEIKNVTGIIGDIADQINLLALNAAIE 552

R14_Tlp14 QAQSLEETAAALEEITSSMQNVSVKTSDVITQSEEIKNVTGIIGDIADQINLLALNAAIE 552

CG8421_Tlp25 QAQSLEETAAALEEITSSMQNVSVKTSDVITQSEEIKNVTGIIGDIADQINLLALNAAIE 343

HC2-48_Tlp3 ------------F-FSSSF----------------------------------------F 490

35925B2_Tlp3 QAQSLEETAAALEEITSSMQNVSVKTSDVITQSEEIKNVTGIIGDIADQINLLALNAAIE 561

14980A_Tlp3 QAQSLEETAAALEEITSSMQNVSVKTSDVITQSEEIKNVTGIIGDIADQINLLALNAAIE 558

00-1597_Tlp3b QAQSLEETAAALEEITSSMQNVSVKTSDVITQSEEIKNVTGIIGDIADQINLLALNAAIE 547

ICDCCJ07001_Tlp3 QAQSLEETAAALEEITSSMQNVSVKTSDVITQSEEIKNVTGIIGDIADQINLLALNAAIE 547

RM3196_Tlp3 QAQSLEETAAALEEITSSMQNVSVKTSDVITQSEEIKNVTGIIGDIADQINLLALNAAIE 547

RM1875_Tlp3 QAASLEETAAALEEITSSMQNVSVKTSDVITQSEEIKNVTGIIGDIADQINLLALNAAIE 547

CF2-75_Tlp3 QAASLEETAAALEEITSSMQNVSVKTSDVITQSEEIKNVTGIIGDIADQINLLA------ 552

RM5611_Tlp3 QAASLEETAAALEEITSSMQNVSVKTSDVITQSEEIKNVTGIIGDIADQINLLALNAAIE 547

MTVDSCj16_Tlp3 QAQSLEETAAALEEITSSMQNVSVKTSDVITQSEEIKNVTGIIGDIADQINLLALNAAIE 547

01-1512_Tlp3 QAQSLEETAAALEEITSSMQNVSVKTSDVITQSEEIKNVTGIIGDIADQINLLALNAAIE 547

MTVDSCj13_Tlp3 QAQSLEETAAALEEITSSMQNVSVKTSDVITQSEEIKNVTGIIGDIADQINLLALNAAIE 547

32488_Tlp3a QAQSLEETAAALEEITSSMQNVSVKTSDVITQSEEIKNVTGIIGDIADQINLLALNAAIE 547

81116_Tlp3 QAQSLEETAAALEEITSSMQNVSVKTSDVITQSEEIKNVTGIIGDIADQINLLALNAAIE 547

32488_Tlp3b QAQSLEETAAALEEITSSMQNVSVKTSDVITQSEEIKNVTGIIGDIADQINLLALNAAIE 547

FB1_Tlp3 QAQSLEETAAALEEITSSMQNVSVKTSDVITQSEEIKNVTGIIGDIADQINLLALNAAIE 547

PT14_Tlp3 QAQSLEETAAALEEITSSMQNVSVKTSDVITQSEEIKNVTGIIGDIADQINLLALNAAIE 547

00-6200_Tlp3a QAQSLEETAAALEEITSSMQNVSVKTSDVITQSEEIKNVTGIIGDIADQINLLALNAAIE 547

RM1221_Tlp3 QAQSLEETAAALEEITSSMQNVSVKTSDVITQSEEIKNVTGIIGDIADQINLLALNAAIE 547

S3_Tlp3 QAQSLEETAAALEEITSSMQNVSVKTSDVITQSEEIKNVTGIIGDIADQINLLALNAAIE 547

FDAARGOS_421_Tlp3 QAQSLEETAAALEEITSSMQNVSVKTSDVITQSEEIKNVTGIIGDIADQINLLALNAAIE 558

CFSAN032806_Tlp3 QAQSLEETAAALEEITSSMQNVSVKTSDVITQSEEIKNVTGIIGDIADQINLLALNAAIE 558

IA3901_Tlp3b QAQSLEETAAALEEITSSMQNVSVKTSDVITQSEEIKNVTGIIGDIADQINLLALNAAIE 547

00-6200_Tlp3b QAQSLEETAAALEEITSSMQNVSVKTSDVITQSEEIKNVTGIIGDIADQINLLALNAAIE 547

BCW_6290_Tlp3b QAQSLEETAAALEEITSSMQNVSVKTSDVITQSEEIKNVTGIIGDIADQINLLALNAAIE 547

00-2425_Tlp3a QAQSLEETAAALEEITSSMQNVSVKTSDVITQSEEIKNVTGIIGDIADQINLLALNAAIE 547

00-2425_Tlp3b QAQSLEETAAALEEITSSMQNVSVKTSDVITQSEEIKNVTGIIGDIADQINLLALNAAIE 547

YH001_Tlp3a QAQSLEETAAALEEITSSMQNVSVKTSDVITQSEEIKNVTGIIGDIADQINLLALNAAIE 547

YH001_Tlp3b QAQSLEETAAALEEITSSMQNVSVKTSDVITQSEEIKNVTGIIGDIADQINLLALNAAIE 547

00-0949_Tlp3 QAQSLEETAAALEEITSSMQNVSVKTSDVITQSEEIKNVTGIIGDIADQINLLALNAAIE 547

NCTC11168_Tlp3 QAQSLEETAAALEEITSSMQNVSVKTSDVITQSEEIKNVTGIIGDIADQINLLALNAAIE 558

F38011_Tlp3 QAQSLEETAAALEEITSSMQNVSVKTSDVITQSEEIKNVTGIIGDIADQINLLALNAAIE 547

RM1285_Tlp3 QAQSLEETAAALEEITSSMQNVSVKTSDVITQSEEIKNVTGIIGDIADQINLLALNAAIE 547

FDAARGOS_422_Tlp3 QAQSLEETAAALEEITSSMQNVSVKTSDVITQSEEIKNVTGIIGDIADQINLLALNAAIE 558

MTVDSCj07_Tlp3 QAQSLEETAAALEEITSSMQNVSVKTSDVITQSEEIKNVTGIIGDIADQINLLALNAAIE 547

IA3901_Tlp3a QAQSLEETAAALEEITSSMQNVSVKTSDVITQSEEIKNVTGIIGDIADQINLLALNAAIE 547

BCW_6290_Tlp3a QAQSLEETAAALEEITSSMQNVSVKTSDVITQSEEIKNVTGIIGDIADQINLLALNAAIE 547

T1-21_Tlp19a QAQSLEETAAALEEITSSMQNVSVKTSDVITQSEEIKNVTGIIGDIADQINLLALNAAIE 502

CJ677CC012_Tlp3 ------------------------------------------------------------ 420

CJM1cam_Tlp3 QAQSLEETAAALEEITSSMQNVSVKTSDVITQSEEIKNVTGIIGDIADQINLLALNAAIE 547

M1_Tlp3 QAQSLEETAAALEEITSSMQNVSVKTSDVITQSEEIKNVTGIIGDIADQINLLALNAAIE 547

4031_Tlp3 QAQSLEETAAALEEITSSMQNVSVKTSDVITQSEEIKNVTGIIGDIADQINLLALNAAIE 547

R14_Tlp3 QAQSLEETAAALEEITSSMQNVSVKTSDVITQSEEIKNVTGIIGDIADQINLLALNAAIE 547

FDAARGOS_295_Tlp21 QAQSLEETAAALEEITSSMQNVSVKTSDVITQSEEIKNVTGIIGDIADQINLLALNAAIE 549

FORC_046_Tlp4 QAQSLEETAAALEEITSSMQNVSVKTSDVITQSEEIKNVTGIIGDIADQINLLALNAAIE 561

FDAARGOS_422_Tlp4 QAQSLEETAAALEEITSSMQNVSVKTSDVITQSEEIKNVTGIIGDIADQINLLALNAAIE 561

ICDCCJ07001_Tlp4 QAASLEETAAALEEITSSMQNVSVKTSDVITQSEEIKNVTGIIGDIADQINLLALNAAIE 561

RM3196_Tlp4 QAASLEETAAALEEITSSMQNVSVKTSDVITQSEEIKNVTGIIGDIADQINLLALNAAIE 561

T1-21_Tlp4 QAQSLEETAAALEEITSSMQNVSVKTSDVITQSEEIKNVTGIIGDIADQINLLALNAAIE 561

F38011_Tlp4 QAQSLEETAAALEEITSSMQNVSVKTSDVITQSEEIKNVTGIIGDIADQINLLALNAAIE 561

HF5-4A-4_Tlp22 QAQSLEETAAALEEITSSMQNVSVKTSDVITQSEEIKNVTGIIGDIADQINLLALNAAIE 507

00-0949_Tlp4 QAQSLEETAAALEEITSSMQNVSVKTSDVITQSEEIKNVTGIIGDIADQINLLALNAAIE 560

01-1512_Tlp4 QAQSLEETAAALEEITSSMQNVSVKTSDVITQSEEIKNVTGIIGDIADQINLLALNAAIE 561

81-176_Tlp4 QAQSLEETAAALEEITSSMQNVSVKTSDVITQSEEIKNVTGIIGDIADQINLLALNAAIE 561

32488_Tlp4 QAQSLEETAAALEEITSSMQNVSVKTSDVITQSEEIKNVTGIIGDIADQINLLALNAAIE 561

NCTC11168_Tlp4 QAQSLEETAAALEEITSSMQNVSVKTSDVITQSEEIKNVTGIIGDIADQINLLALNAAIE 561

CFSAN032806_Tlp4 QAQSLEETAAALEEITSSMQNVSVKTSDVITQSEEIKNVTGIIGDIADQINLLALNAAIE 561

81116_Tlp4 QAQSLEETAAALEEITSSMQNVSVKTSDVITQSEEIKNVTGIIGDIADQINLLALNAAIE 561

RM1285_Tlp12 QAQSLEETAAALEEITSSMQNVSVKTSDVITQSEEIKNVTGIIGDIADQINLLALNAAIE 557

PT14_Tlp12 QAQSLEETAAALEEITSSMQNVSVKTSDVITQSEEIKNVTGIIGDIADQINLLALNAAIE 558

MTVJDCj07_Tlp12 QAQSLEETAAALEEITSSMQNVSVKTSDVITQSEEIKNVTGIIGDIADQINLLALNAAIE 558

RM1221_Tlp12 QAQSLEETAAALEEITSSMQNVSVKTSDVITQSEEIKNVTGIIGDIADQINLLALNAAIE 558

FDAARGOS_421_Tlp12 QAQSLEETAAALEEITSSMQNVSVKTSDVITQSEEIKNVTGIIGDIADQINLLALNAAIE 558

35925B2_Tlp12 QAQSLEETAAALEEITSSMQNVSVKTSDVITQSEEIKNVTGIIGDIADQINLLALNAAIE 558

CJM1cam_Tlp12 QAQSLEETAAALEEITSSMQNVSVKTSDVITQSEEIKNVTGIIGDIADQINLLALNAAIE 558

M1_Tlp12 QAQSLEETAAALEEITSSMQNVSVKTSDVITQSEEIKNVTGIIGDIADQINLLALNAAIE 558

S3_Tlp12 QAQSLEETAAALEEITSSMQNVSVKTSDVITQSEEIKNVTGIIGDIADQINLLALNAAIE 558

00-1597_Tlp12 QAQSLEETAAALEEITSSMQNVSVKTSDVITQSEEIKNVTGIIGDIADQINLLALNAAIE 558

R14_Tlp12 QAQSLEETAAALEEITSSMQNVSVKTSDVITQSEEIKNVTGIIGDIADQINLLALNAAIE 558

RM1875_Tlp15 QAASLEETAAALEEITSSMQNVSVKTSDVITQSEEIKNVTGIIGDIADQINLLALNAAIE 475

YH503_Tlp16 QAASLEETAAALEEITSSMQNVSVKTSDVITQSEEIKNVTGIIGDIADQINLLALNAAIE 475

FB1_Tlp16 QAASLEETAAALEEITSSMQNVSVKTSDVITQSEEIKNVTGIIGDIADQINLLALNAAIE 475

BFR-CA-9557_Tlp16 QAASLEETAAALEEITSSMQNVSVKTSDVITQSEEIKNVTGIIGDIADQINLLALNAAIE 474

15-537360_Tlp16 QAASLEETAAALEEITSSMQNVSVKTSDVITQSEEIKNVTGIIGDIADQINLLALNAAIE 475

OR12_Tlp16 QAASLEETAAALEEITSSMQNVSVKTSDVITQSEEIKNVTGIIGDIADQINLLALNAAIE 475

YH502_Tlp16 QAASLEETAAALEEITSSMQNVSVKTSDVITQSEEIKNVTGIIGDIADQINLLALNAAIE 475

14903A_Tlp16 QAASLEETAAALEEITSSMQNVSVKTSDVITQSEEIKNVTGIIGDIADQINLLALNAAIE 475

HC2-48_Tlp16 QAASLEETAAALEEITSSMQNVSVKTSDVITQS--------------------------- 447

RM5611_Tlp16 QAASLEETAAALEEITSSMQNVSVKTSDVITQSEEIKNVTGIIGDIADQINLLALNAAIE 475

00-2425_Tlp11 QAQSLEETAAALEEITSSMQNVSVKTSDVITQSEEIKNVTGIIGDIADQINLLALNAAIE 602

00-6200_Tlp11 QAQSLEETAAALEEITSSMQNVSVKTSDVITQSEEIKNVTGIIGDIADQINLLALNAAIE 602

YH001_Tlp11 QAQSLEETAAALEEITSSMQNVSVKTSDVITQSEEIKNVTGIIGDIADQINLLALNAAIE 602

IA3902_Tlp11 QAQSLEETAAALEEITSSMQNVSVKTSDVITQSEEIKNVTGIIGDIADQINLLALNAAIE 602

BCW_6290_Tlp11 QAQSLEETAAALEEITSSMQNVSVKTSDVITQSEEIKNVTGIIGDIADQINLLALNAAIE 602

76339_Tlp18 QAASLEETAAALEEITSSMQNVSVKLSDVITQSEEIKNVTGIIGDIADQINLLALNAAIE 601

4031_Tlp17 QAQSLEETAAALEEITSSMQNVSVKTSDVITQSEEIKNVTGIIGDIADQINLLALNAAIE 602

MTVDSCj13_Tlp13 QAQSLEETAAALEEITSSMQNVSVKTSDVITQSEEIKNVTGIIGDIADQINLLALNAAIE 601

RM1875_Tlp13 QAASLEETAAALEEITSSMQNVSVKTSDVITQSEEIKNVTGIIGDIADQINLLALNAAIE 601

CF2-75_Tlp13 QAASLEETAAALEEITSSMQNVSVKTSDVITQSEEIKNVTGIIGDIADQINLLA------ 595

15-537360_Tlp13 QAASLEETAAALEEITSSMQNVSVKTSDVITQSEEIKNVTGIIGDIADQINLLALNAAIE 601

CVMN29710_Tlp13 QAASLEETAAALEEITSSMQNVSVKTSDVITQSEEIKNVTGIIGDIADQINLLALNAAIE 601

FB1_Tlp13 QAASLEETAAALEEITSSMQNVSVKTSDVITQSEEIKNVTGIIGDIADQINLLALNAAIE 601

CFCAN032805_Tlp13 QAASLEETAAALEEITSSMQNVSVKTSDVITQSEEIKNVTGIIGDIADQINLLALNAAIE 601

BG2108_Tlp13 QAASLEETAAALEEITSSMQNVSVKTSDVITQSEEIKNVTGIIGDIADQINLLALNAAIE 601

YF2105_Tlp13 QAASLEETAAALEEITSSMQNVSVKTSDVITQSEEIKNVTGIIGDIADQINLLALNAAIE 601

YH503_Tlp13 QAASLEETAAALEEITSSMQNVSVKTSDVITQSEEIKNVTGIIGDIADQINLLALNAAIE 601

BFRCA9557_Tlp13 QAASLEETAAALEEITSSMQNVSVKTSDVITQSEEIKNVTGIIGDIADQINLLALNAAIE 601

YH502_Tlp13 QAASLEETAAALEEITSSMQNVSVKTSDVITQSEEIKNVTGIIGDIADQINLLALNAAIE 601

OR12_Tlp13 QAQSLEETAAALEEITSSMQNVSVKTSDVITQSEEIKNVTGIIGDIADQINLLALNAAIE 601

00-1597_Tlp13 QAQSLEETAAALEEITSSMQNVSVKTSDVITQSEEIKNVTGIIGDIADQINLLALNAAIE 601

14903A_Tlp13 QAQSLEETAAALEEITSSMQNVSVKTSDVITQSEEIKNVTGIIGDIADQINLLALNAAIE 601

FJ3124_Tlp13 QAQSLEETAAALEEITSSMQNVSVKTSDVITQSEEIKNVTGIIGDIADQINLLALNAAIE 601

R14_Tlp13 QAQSLEETAAALEEITSSMQNVSVKTSDVITQSEEIKNVTGIIGDIADQINLLALNAAIE 601

MTVDSCj16_Tlp13 QAQSLEETAAALEEITSSMQNVSVKTSDVITQSEEIKNVTGIIGDIADQINLLALNAAIE 601

14980A_Tlp13 QAQSLEETAAALEEITSSMQNVSVKTSDVITQSEEIKNVTGIIGDIADQINLLALNAAIE 601

FDAARGOS_295_Tlp1 AARAGEHGRGFAVVADEVRQLAERTGKSLSEIEANINILVQSVNEVAESVKEQTAGITQI 656

14980A_Tlp1 AARAGEHGRGFAVVADEVRQLAERTGKSLSEIEANINILVQSVNEVAESVKEQTTGITQI 656

CJ677CC527_Tlp1 AARAGEHGRGFAVVADEVRQLAERTGKSLSEIEANINILVQSVNEVAESVKEQTAGITQI 656

CJ677CC012_Tlp1 AARAGEHGRGFAVVADEVRQLAERTGKSLSEIEANINILVQSVNEVAESVKEQTAGITQI 656

4031_Tlp1 AARAGEHGRGFAVVADEVRQLAERTGKSLSEIEANINILVQSVNEVAESVKEQTAGITQI 656

81116_Tlp1 AARAGEHGRGFAVVADEVRQLAERTGKSLSEIEANINILVQSVNEVAESVKEQTAGITQI 656

35925B2_Tlp1 AARAGEHGRGFAVVADEVRQLAERTGKSLSEIEANINILVQSVNEVAESVKEQTAGITQI 656

M1_Tlp1 AARAGEHGRGFAVVADEVRQLAERTGKSLSEIEANINILVQSVNEVAESVKEQTAGITQI 656

PT14_Tlp1 AARAGEHGRGFAVVADEVRQLAERTGKSLSEIEANINILVQSVNEVAESVKEQTAGITQI 656

81-176_Tlp1 AARAGEHGRGFAVVADEVRQLAERTGKSLSEIEANINILVQSVNEVAESVKEQTAGITQI 656

CVMN29710_Tlp1 AARAGEHGRGFAVVADEVRQLAERTGKSLSEIEANINILVQSVNEVAESVKEQTAGITQI 656

FB1_Tlp1 AARAGEHGRGFAVVADEVRQLAERTGKSLSEIEANINILVQSVNEVAESVKEQTAGITQI 656

BG2108_Tlp1 AARAGEHGRGFAVVADEVRQLAERTGKSLSEIEANINILVQSVNEVAESVKEQTAGITQI 656

YF2108_Tlp1 AARAGEHGRGFAVVADEVRQLAERTGKSLSEIEANINILVQSVNEVAESVKEQTAGITQI 656

YH501_Tlp1 AARAGEHGRGFAVVADEVRQLAERTGKSLSEIEANINILVQSVNEVAESVKEQTAGITQI 656

RM4661_Tlp1 AARAGEHGRGFAVVADEVRQLAERTGKSLSEIEANINILVQSVNEVAESVKEQTAGITQI 656

F38011_Tlp1 AARAGEHGRGFVVVADEVRQLAERTGKSLSEIEANINILVQSVNEVAESVKEQTAGITQI 656

T1-21_Tlp1 AARAGEHGRGFAVVADEVRQLAERTGKSLSEIEANINILVQSVNEVAESVKEQTAGITQI 656

CG8421_Tlp1 AARAGEHGRGFAVVADEVRQLAERTGKSLSEIEANINILVQSVNEVAESVKEQTAGITQI 656

CJM1cam_Tlp1 AARAGEHGRGFAVVADEVRQLAERTGKSLSEIEANINILVQSVNEVAESVKEQTAGITQI 656

R14_Tlp1 AARAGEHGRGFAVVADEVRQLAERTGKSLSEIEANINILVQSVNEVAESVKEQTAGITQI 656

ICDCCJ07001_Tlp1 AARAGEHGRGFAVVADEVRQLAERTGKSLSEIEANINILVQSVNEVAESVKEQTAGITQI 656

RM3196_Tlp1 AARAGEHGRGFAVVADEVRQLAERTGKSLSEIEANINILVQSVNEVAESVKEQTAGITQI 656

NCTC11168_Tlp1 AARAGEHGRGFAVVADEVRQLAERTGKSLSEIEANINILVQSVNEVAESVKEQTAGITQI 656

00-2425_Tlp1 AARAGEHGRGFAVVADEVRQLAERTGKSLSEIEANINILVQSVNEVAESVKEQTAGITQI 656

IA3902_Tlp1 AARAGEHGRGFAVVADEVRQLAERTGKSLSEIEANINILVQSVNEVAESVKEQTAGITQI 656

RM1285_Tlp1 AARAGEHGRGFAVVADEVRQLAERTGKSLSEIEANINILVQSVNEVAESVKEQTAGITQI 656

00-0949_Tlp1 AARAGEHGRGFAVVADEVRQLAERTGKSLSEIEANINILVQSVNEVAESVKEQTAGITQI 656

01-1512_Tlp1 AARAGEHGRGFAVVADEVRQLAERTGKSLSEIEANINILVQSVNEVAESVKEQTAGITQI 656

FDAARGOS_422_Tlp1 AARAGEHGRGFAVVADEVRQLAERTGKSLSEIEANINILVQSVNEVAESVKEQTAGITQI 656

FORC_056_Tlp1 AARAGEHGRGFAVVADEVRQLAERTGKSLSEIEANINILVQSVNEVAESVKEQTAGITQI 656

32488_Tlp1 AARAGEHGRGFAVVADEVRQLAERTGKSLSEIEANINILVQSVNEVAESVKEQTAGITQI 656

CFSAN032806_Tlp1 AARAGEHGRGFAVVADEVRQLAERTGKSLSEIEANINILVQSVNEVAESVKEQTAGITQI 656

YH001_Tlp1 AARAGEHGRGFAVVADEVRQLAERTGKSLSEIEANINILVQSVNEVAESVKEQTAGITQI 656

00-6200_Tlp1 AARAGEHGRGFAVVADEVRQLAERTGKSLSEIEANINILVQSVNEVAESVKEQTAGITQI 656

RM1221_Tlp1 AARAGEHGRGFAVVADEVRQLAERTGKSLSEIEANINILVQSVNEVAESVKEQTAGITQI 656

S3_Tlp1 AARAGEHGRGFAVVADEVRQLAERTGKSLSEIEANINILVQSVNEVAESVKEQTAGITQI 656

FDAARGOS_421_Tlp1 AARAGEHGRGFAVVADEVRQLAERTGKSLSEIEANINILVQSVNEVAESVKEQTAGITQI 656

FJ3124_Tlp1 AARAGEHGRGFAVVADEVRQLAERTGKSLSEIEANINILVQSVNEVAESVKEQTAGITQI 656

00-1597_Tlp1 AARAGEHGRGFAVVADEVRQLAERTGKSLSEIEANINILVQSVNEVAESVKEQTAGITQI 656

15-537360_Tlp20 AARAGEHGRGFAVVADEVRKLAERTQKSLSEIEANTNLLVQSINDMAESIKEQTAGITQI 615

76339_Tlp20 AARAGEHGRGFAVVADEVRKLAERTQKSLSEIEANTNLLVQSINDMAESIKEQTAGITQI 615

HC2-48_Tlp20 ---------------------------HSIEIEANTNLLVQSINDMAESIKEQTAGITQI 556

CFSAN032805_Tlp20 AARAGEHGRGFAVVADEVRKLAERTQKSLSEIEANTNLLVQSINDMAESIKEQTAGITQI 614

CVMN29710_Tlp20 AARAGEHGRGFAVVADEVRKLAERTQKSLSEIEANTNLLVQSINDMAESIKEQTAGITQI 613

YH501_Tlp20 AARAGEHGRGFAVVADEVRKLAERTQKSLSEIEANTNLLVQSINDMAESIKEQTAGITQI 617

CF2-75_Tlp20 --------------------LAEITQKSLSEIEANTNLLVQSINDMAESIKEQTAGITQI 590

CO2-160_Tlp20 AARAGEHGRGFAVVADEVRKLAERTQKSLSEIEANTNLLVQSINDMAESIKEQTAGITQI 615

CO2-160_Tlp20b AARAGEHGRGFAVVADEVRKLAERTQKSLSEIEANTNLLVQSINDMAESIKEQTAGITQI 615

RM5611_Tlp20 AARAGEHGRGFAVVADEVRKLAERTQKSLSEIEANTNLLVQSINDMAESIKEQTAGITQI 615

14903A_Tlp20 AARAGEHGRGFAVVADEVRKLAERTQKSLSEIEANTNLLVQSINDMAESIKEQTAGITQI 617

YH502_Tlp20 AARAGEHGRGFAVVADEVRKLAERTQKSLSEIEANTNLLVQSINDMAESIKEQTAGITQI 614

RM3196_Tlp23 AARAGEHGRGFAVVADEVRKLAERTQKSLSEIEANTNLLVQSINDMAESIKEQTAGITQI 615

ICDCCJ07001_Tlp23 AARAGEHGRGFAVVADEVRKLAERTQKSLSEIEANTNLLVQSINDMAESIKEQTAGITQI 615

RM1285_Tlp2 AARAGEHGRGFAVVADEVRKLAERTQKSLSEIEANTNLLVQSINDMAESIKEQTAGITQI 614

CFSAN032806_Tlp2 AARAGEHGRGFAVVADEVRKLAERTQKSLSEIEANTNLLVQSINDMAESIKEQTAGITQI 615

RM1221_Tlp2 AARAGEHGRGFAVVADEVRKLAERTQKSLSEIEANTNLLVQSINDMAESIKEQTAGITQI 615

S3_Tlp2 AARAGEHGRGFAVVADEVRKLAERTQKSLSEIEANTNLLVQSINDMAESIKEQTAGITQI 615

FDAARGOS_422_Tlp2 AARAGEHGRGFAVVADEVRKLAERTQKSLSEIEANTNLLVQSINDMAESIKEQTAGITQI 615

81-176_Tlp2 AARAGEHGRGFAVVADEVRKLAERTQKSLSEIEANTNLLVQSINDMAESIKEQTAGITQI 615

F38011_Tlp2 AARAGEHGRGFAVVADEVRKLAERTQKSLSEIEANTNLLVQSINDMAESIKEQTAGITQI 615

NCTC11168_Tlp2 AARAGEHGRGFAVVADEVRKLAERTQKSLSEIEANTNLLVQSINDMAESIKEQTAGITQI 615

MTVDSCj07_Tlp2 AARAGEHGRGFAVVADEVRKLAERTQKSLSEIEANTNLLVQSINDMAESIKEQTAGITQI 615

CJM1cam_Tlp24 AARAGEHGRGFAVVADEVRKLAERTQKSLSEIEANTNLLVQSINDMAESIKEQTAGITQI 612

M1_Tlp24 AARAGEHGRGFAVVADEVRKLAERTQKSLSEIEANTNLLVQSINDMAESIKEQTAGITQI 611

81116_Tlp2 AARAGEHGRGFAVVADEVRKLAERTQKSLSEIEANTNLLVQSINDMAESIKEQTAGITQI 615

4031_Tlp23 AARAGEHGRGFAVVADEVRKLAERTQKSLSEIEANTNLLVQSINDMAESIKEQTAGITQI 614

CVMN29710_Tlp14 AARAGEHGRGFAVVADEVRKLAERTQKSLSEIEANTNLLVQSINDMAESIKEQTAGITQI 612

RM4661_Tlp14 AARAGEHGRGFAVVADEVRKLAERTQKSLSEIEANTNLLVQSINDMAESIKEQTAGITQI 612

MG1116_Tlp14 AARAGEHGRGFAVVADEVRKLAERTQKSLSEIEANTNLLVQSINDMAESIKEQTAGITQI 605

BG2108_Tlp14 AARAGEHGRGFAVVADEVRKLAERTQKSLSEIEANTNLLVQSINDMAESIKEQTAGITQI 544

YF2105_Tlp14 AARAGEHGRGFAVVADEVRKLAERTQKSLSEIEANTNLLVQSINDMAESIKEQTAGITQI 573

YH502_Tlp14 AARAGEHGRGFAVVADEVRKLAERTQKSLSEIEANTNLLVQSINDMAESIKEQTAGITQI 623

WA333_Tlp14 ------------------------------EIEANTNLLVQSINDMAESIKEQTAGITQI 544

BP3181_Tlp14 AARAGEHGRGFAVVADEVRKLAERTQKSLSEIEANTNLLVQSINDMAESIKEQTAGITQI 612

ZV1224_Tlp14a AARAGEHGRGFAVVADEVRKLAERTQKSLSEIEANTNLLVQSINDMAESIKEQTAGITQI 612

ZV1224_Tlp14b AARAGEHGRGFAVVADEVRKLAERTQKSLSEIEANTNLLVQSINDMAESIKEQTAGITQI 612

YH503_Tlp14 AARAGEHGRGFAVVADEVRKLAERTQKSLSEIEANTNLLVQSINDMAESIKEQTAGITQI 623

14903A_Tlp14 AARAGEHGRGFAVVADEVRKLAERTQKSLSEIEANTNLLVQSINDMAESIKEQTAGITQI 612

OR12_Tlp14 AARAGEHGRGFAVVADEVRKLAERTQKSLSEIEANTNLLVQSINDMAESIKEQTAGITQI 612

CFSAN032805_Tlp14 AARAGEHGRGFAVVADEVRKLAERTQKSLSEIEANTNLLVQSINDMAESIKEQTAGITQI 623

BFR-CA-9557_Tlp14 AARAGEHGRGFAVVADEVRKLAERTQKSLSEIEANTNLLVQSINDMAESIKEQTAGITQI 612

15-537360_Tlp14 AARAGEHGRGFAVVADEVRKLAERTQKSLSEIEANTNLLVQSINDMAESIKEQTAGITQI 612

YH501_Tlp14 AARAGEHGRGFAVVADEVRKLAERTQKSLSEIEANTNLLVQSINDMAESIKEQTAGITQI 612

T1-21_Tlp19b AARAGEHGRGFAVVADEVRKLAERTQKSLSEIEANTNLLVQSINDMAESIKEQTAGITQI 567

CG8421_Tlp14 AARAGEHGRGFAVVADEVRKLAERTQKSLSEIEANTNLLVQSINDMAESIKEQTAGITQI 606

MTVDSCj16_Tlp14 AARAGEHGRGFAVVADEVRKLAERTQKSLSEIEANTNLLVQSINDMAESIKEQTAGITQI 612

01-1512_Tlp14 AARAGEHGRGFAVVADEVRKLAERTQKSLSEIEANTNLLVQSINDMAESIKEQTAGITQI 612

00-0949_Tlp14 AARAGEHGRGFAVVADEVRKLAERTQKSLSEIEANTNLLVQSINDMAESIKEQTAGITQI 612

MTVDSCj13_Tlp14 AARAGEHGRGFAVVADEVRKLAERTQKSLSEIEANTNLLVQSINDMAESIKEQTAGITQI 612

S3_Tlp14 AARAGEHGRGFAVVADEVRKLAERTQKSLSEIEANTNLLVQSINDMAESIKEQTAGITQI 612

PT14_Tlp14 AARAGEHGRGFAVVADEVRKLAERTQKSLSEIEANTNLLVQSINDMAESIKEQTAGITQI 612

14980A_Tlp14 AARAGEHGRGFAVVADEVRKLAERTQKSLSEIEANTNLLVQSINDMAESIKEQTAGITQI 628

FJ3124_Tlp14 AARAGEHGRGFAVVADEVRKLAERTQKSLSEIEANTNLLVQSINDMAESIKEQTAGITQI 612

00-1597_Tlp14 AARAGEHGRGFAVVADEVRKLAERTQKSLSEIEANTNLLVQSINDMAESIKEQTAGITQI 612

R14_Tlp14 AARAGEHGRGFAVVADEVRKLAERTQKSLSEIEANTNLLVQSINDMAESIKEQTAGITQI 612

CG8421_Tlp25 AARAGEHGRGFAVVADEVRKLAERTQKSLSEIEANTNLLVQSINDMAESIKEQTAGITQI 403

HC2-48_Tlp3 RRNCSCFRRDYFFYAKCF--CKNQ-CYHSIEIEANTNLLVQSINDMAESIKEQTAGITQI 547

35925B2_Tlp3 AARAGEHGRGFAVVADEVRKLAERTQKSLSEIEANTNLLVQSINDMAESIKEQTAGITQI 621

14980A_Tlp3 AARAGEHGRGFAVVADEVRKLAERTQKSLSEIEANTNLLVQSINDMAESIKEQTAGITQI 618

00-1597_Tlp3b AARAGEHGRGFAVVADEVRKLAERTQKSLSEIEANTNLLVQSINDMAESIKEQTAGITQI 607

ICDCCJ07001_Tlp3 AARAGEHGRGFAVVADEVRKLAERTQKSLSEIEANTNLLVQSINDMAESIKEQTAGITQI 607

RM3196_Tlp3 AARAGEHGRGFAVVADEVRKLAERTQKSLSEIEANTNLLVQSINDMAESIKEQTAGITQI 607

RM1875_Tlp3 AARAGEHGRGFAVVADEVRKLAERTQKSLSEIEANTNLLVQSINDMAESIKEQTAGITQI 607

CF2-75_Tlp3 ----------------------EITQKSLSEIEANTNLLVQSINDMAESIKEQTAGITQI 590

RM5611_Tlp3 AARAGEHGRGFAVVADEVRKLAERTQKSLSEIEANTNLLVQSINDMAESIKEQTAGITQI 607

MTVDSCj16_Tlp3 AARAGEHGRGFAVVADEVRKLAERTQKSLSEIEANTNLLVQSINDMAESIKEQTAGITQI 607

01-1512_Tlp3 AARAGEHGRGFAVVADEVRKLAERTQKSLSEIEANTNLLVQSINDMAESIKEQTAGITQI 607

MTVDSCj13_Tlp3 AARAGEHGRGFAVVADEVRKLAERTQKSLSEIEANTNLLVQSINDMAESIKEQTAGITQI 607

32488_Tlp3a AARAGEHGRGFAVVADEVRKLAERTQKSLSEIEANTNLLVQSINDMAESIKEQTAGITQI 607

81116_Tlp3 AARAGEHGRGFAVVADEVRKLAERTQKSLSEIEANTNLLVQSINDMAESIKEQTAGITQI 607

32488_Tlp3b AARAGEHGRGFAVVADEVRKLAERTQKSLSEIEANTNLLVQSINDMAESIKEQTAGITQI 607

FB1_Tlp3 AARAGEHGRGFAVVADEVRKLAERTQKSLSEIEANTNLLVQSINDMAESIKEQTAGITQI 607

PT14_Tlp3 AARAGEHGRGFAVVADEVRKLAERTQKSLSEIEANTNLLVQSINDMAESIKEQTAGITQI 607

00-6200_Tlp3a AARAGEHGRGFAVVADEVRKLAERTQKSLSEIEANTNLLVQSINDMAESIKEQTAGITQI 607

RM1221_Tlp3 AARAGEHGRGFAVVADEVRKLAERTQKSLSEIEANTNLLVQSINDMAESIKEQTAGITQI 607

S3_Tlp3 AARAGEHGRGFAVVADEVRKLAERTQKSLSEIEANTNLLVQSINDMAESIKEQTAGITQI 607

FDAARGOS_421_Tlp3 AARAGEHGRGFAVVADEVRKLAERTQKSLSEIEANTNLLVQSINDMAESIKEQTAGITQI 618

CFSAN032806_Tlp3 AARAGEHGRGFAVVADEVRKLAERTQKSLSEIEANTNLLVQSINDMAESIKEQTAGITQI 618

IA3901_Tlp3b AARAGEHGRGFAVVADEVRKLAERTQKSLSEIEANTNLLVQSINDMAESIKEQTAGITQI 607

00-6200_Tlp3b AARAGEHGRGFAVVADEVRKLAERTQKSLSEIEANTNLLVQSINDMAESIKEQTAGITQI 607

BCW_6290_Tlp3b AARAGEHGRGFAVVADEVRKLAERTQKSLSEIEANTNLLVQSINDMAESIKEQTAGITQI 607

00-2425_Tlp3a AARAGEHGRGFAVVADEVRKLAERTQKSLSEIEANTNLLVQSINDMAESIKEQTAGITQI 607

00-2425_Tlp3b AARAGEHGRGFAVVADEVRKLAERTQKSLSEIEANTNLLVQSINDMAESIKEQTAGITQI 607

YH001_Tlp3a AARAGEHGRGFAVVADEVRKLAERTQKSLSEIEANTNLLVQSINDMAESIKEQTAGITQI 607

YH001_Tlp3b AARAGEHGRGFAVVADEVRKLAERTQKSLSEIEANTNLLVQSINDMAESIKEQTAGITQI 607

00-0949_Tlp3 AARAGEHGRGFAVVADEVRKLAERTQKSLSEIEANTNLLVQSINDMAESIKEQTAGITQI 607

NCTC11168_Tlp3 AARAGEHGRGFAVVADEVRKLAERTQKSLSEIEANTNLLVQSINDMAESIKEQTAGITQI 618

F38011_Tlp3 AARAGEHGRGFAVVADEVRKLAERTQKSLSEIEANTNLLVQSINDMAESIKEQTAGITQI 607

RM1285_Tlp3 AARAGEHGRGFAVVADEVRKLAERTQKSLSEIEANTNLLVQSINDMAESIKEQTAGITQI 607

FDAARGOS_422_Tlp3 AARAGEHGRGFAVVADEVRKLAERTQKSLSEIEANTNLLVQSINDMAESIKEQTAGITQI 618

MTVDSCj07_Tlp3 AARAGEHGRGFAVVADEVRKLAERTQKSLSEIEANTNLLVQSINDMAESIKEQTAGITQI 607

IA3901_Tlp3a AARAGEHGRGFAVVADEVRKLAERTQKSLSEIEANTNLLVQSINDMAESIKEQTAGITQI 607

BCW_6290_Tlp3a AARAGEHGRGFAVVADEVRKLAERTQKSLSEIEANTNLLVQSINDMAESIKEQTAGITQI 607

T1-21_Tlp19a AARAGEHGRGFAVVADEVRKLAERTQKSLSEIEANTNLLVQSINDMAESIKEQTAGITQI 562

CJ677CC012_Tlp3 ---------------DEVRKLAERTQKSLSEIEANTNLLVQSINDMAESIKEQTAGITQI 465

CJM1cam_Tlp3 AARAGEHGRGFAVVADEVRKLAERTQKSLSEIEANTNLLVQSINDMAESIKEQTAGITQI 607

M1_Tlp3 AARAGEHGRGFAVVADEVRKLAERTQKSLSEIEANTNLLVQSINDMAESIKEQTAGITQI 607

4031_Tlp3 AARAGEHGRGFAVVADEVRKLAERTQKSLSEIEANTNLLVQSINDMAESIKEQTAGITQI 607

R14_Tlp3 AARAGEHGRGFAVVADEVRKLAERTQKSLSEIEANTNLLVQSINDMAESIKEQTAGITQI 607

FDAARGOS_295_Tlp21 AARAGEHGRGFAVVADEVRKLAERTQKSLSEIEANTNLLVQSINDMAESIKEQTAGITQI 609

FORC_046_Tlp4 AARAGEHGRGFAVVADEVRKLAERTQKSLSEIEANTNLLVQSINDMAESIKEQTAGITQI 621

FDAARGOS_422_Tlp4 AARAGEHGRGFAVVADEVRKLAERTQKSLSEIEANTNLLVQSINDMAESIKEQTAGITQI 621

ICDCCJ07001_Tlp4 AARAGEHGRGFAVVADEVRKLAERTQKSLSEIEANTNLLVQSINDMAESIKEQTAGITQI 621

RM3196_Tlp4 AARAGEHGRGFAVVADEVRKLAERTQKSLSEIEANTNLLVQSINDMAESIKEQTAGITQI 621

T1-21_Tlp4 AARAGEHGRGFAVVADEVRKLAERTQKSLSEIEANTNLLVQSINDMAESIKEQTAGITQI 621

F38011_Tlp4 AARAGEHGRGFAVVADEVRKLAERTQKSLSEIEANTNLLVQSINDMAESIKEQTAGITQI 621

HF5-4A-4_Tlp22 AARAGEHGRGFAVVADEVRKLAERTQKSLSEIEANTNLLVQSINDMAESIKEQTAGITQI 567

00-0949_Tlp4 AARAGEHGRGFAVVADEVRKLAERTQKSLSEIEANTNLLVQSINDMAESIKEQTAGITQI 620

01-1512_Tlp4 AARAGEHGRGFAVVADEVRKLAERTQKSLSEIEANTNLLVQSINDMAESIKEQTAGITQI 621

81-176_Tlp4 AARAGEHGRGFAVVADEVRKLAERTQKSLSEIEANTNLLVQSINDMAESIKEQTAGITQI 621

32488_Tlp4 AARAGEHGRGFAVVADEVRKLAERTQKSLSEIEANTNLLVQSINDMAESIKEQTAGITQI 621

NCTC11168_Tlp4 AARAGEHGRGFAVVADEVRKLAERTQKSLSEIEANTNLLVQSINDMAESIKEQTAGITQI 621

CFSAN032806_Tlp4 AARAGEHGRGFAVVADEVRKLAERTQKSLSEIEANTNLLVQSINDMAESIKEQTAGITQI 621

81116_Tlp4 AARAGEHGRGFAVVADEVRKLAERTQKSLSEIEANTNLLVQSINDMAESIKEQTAGITQI 621

RM1285_Tlp12 AARAGEHGRGFAVVADEVRKLAERTQKSLSEIEANTNLLVQSINDMAESIKEQTAGITQI 617

PT14_Tlp12 AARAGEHGRGFAVVADEVRKLAERTQKSLSEIEANTNLLVQSINDMAESIKEQTAGITQI 618

MTVJDCj07_Tlp12 AARAGEHGRGFAVVADEVRKLAERTQKSLSEIEANTNLLVQSINDMAESIKEQTAGITQI 618

RM1221_Tlp12 AARAGEHGRGFAVVADEVRKLAERTQKSLSEIEANTNLLVQSINDMAESIKEQTAGITQI 618

FDAARGOS_421_Tlp12 AARAGEHGRGFAVVADEVRKLAERTQKSLSEIEANTNLLVQSINDMAESIKEQTAGITQI 618

35925B2_Tlp12 AARAGEHGRGFAVVADEVRKLAERTQKSLSEIEANTNLLVQSINDMAESIKEQTAGITQI 618

CJM1cam_Tlp12 AARAGEHGRGFAVVADEVRKLAERTQKSLSEIEANTNLLVQSINDMAESIKEQTAGITQI 618

M1_Tlp12 AARAGEHGRGFAVVADEVRKLAERTQKSLSEIEANTNLLVQSINDMAESIKEQTAGITQI 618

S3_Tlp12 AARAGEHGRGFAVVADEVRKLAERTQKSLSEIEANTNLLVQSINDMAESIKEQTAGITQI 618

00-1597_Tlp12 AARAGEHGRGFAVVADEVRKLAERTQKSLSEIEANTNLLVQSINDMAESIKEQTAGITQI 618

R14_Tlp12 AARAGEHGRGFAVVADEVRKLAERTQKSLSEIEANTNLLVQSINDMAESIKEQTAGITQI 618

RM1875_Tlp15 AARAGEHGRGFAVVADEVRKLAERTQKSLSEIEANTNLLVQSINDMAESIKEQTAGITQI 535

YH503_Tlp16 AARAGEHGRGFAVVADEVRKLAERTQKSLSEIEANTNLLVQSINDMAESIKEQTAGITQI 535

FB1_Tlp16 AARAGEHGRGFAVVADEVRKLAERTQKSLSEIEANTNLLVQSINDMAESIKEQTAGITQI 535

BFR-CA-9557_Tlp16 AARAGEHGRGFAVVADEVRKLAERTQKSLSEIEANTNLLVQSINDMAESIKEQTAGITQI 534

15-537360_Tlp16 AARAGEHGRGFAVVADEVRKLAERTQKSLSEIEANTNLLVQSINDMAESIKEQTAGITQI 535

OR12_Tlp16 AARAGEHGRGFAVVADEVRKLAERTQKSLSEIEANTNLLVQSINDMAESIKEQTAGITQI 535

YH502_Tlp16 AARAGEHGRGFAVVADEVRKLAERTQKSLSEIEANTNLLVQSINDMAESIKEQTAGITQI 535

14903A_Tlp16 AARAGEHGRGFAVVADEVRKLAERTQKSLSEIEANTNLLVQSINDMAESIKEQTAGITQI 535

HC2-48_Tlp16 ------------------------------EIEANTNLLVQSINDMAESIKEQTAGITQI 477

RM5611_Tlp16 AARAGEHGRGFAVVADEVRKLAERTQKSLSEIEANTNLLVQSINDMAESIKEQTAGITQI 535

00-2425_Tlp11 AARAGEHGRGFAVVADEVRKLAERTQKSLSEIEANTNLLVQSINDMAESIKEQTAGITQI 662

00-6200_Tlp11 AARAGEHGRGFAVVADEVRKLAERTQKSLSEIEANTNLLVQSINDMAESIKEQTAGITQI 662

YH001_Tlp11 AARAGEHGRGFAVVADEVRKLAERTQKSLSEIEANTNLLVQSINDMAESIKEQTAGITQI 662

IA3902_Tlp11 AARAGEHGRGFAVVADEVRKLAERTQKSLSEIEANTNLLVQSINDMAESIKEQTAGITQI 662

BCW_6290_Tlp11 AARAGEHGRGFAVVADEVRKLAERTQKSLSEIEANTNLLVQSINDMAESIKEQTAGITQI 662

76339_Tlp18 AARAGEHGRGFAVVADEVRKLAERTQKSLSEIEANTNLLVQSINDMAESIKEQTAGITQI 661

4031_Tlp17 AARAGEHGRGFAVVADEVRKLAERTQKSLSEIEANTNLLVQSINDMAESIKEQTAGITQI 662

MTVDSCj13_Tlp13 AARAGEHGRGFAVVADEVRKLAERTQKSLSEIEANTNLLVQSINDMAESIKEQTAGITQI 661

RM1875_Tlp13 AARAGEHGRGFAVVADEVRKLAERTQKSLSEIEANTNLLVQSINDMAESIKEQTAGITQI 661

CF2-75_Tlp13 ----------------------EITQKSLSEIEANTNLLVQSINDMAESIKEQTAGITQI 633

15-537360_Tlp13 AARAGEHGRGFAVVADEVRKLAERTQKSLSEIEANTNLLVQSINDMAESIKEQTAGITQI 661

CVMN29710_Tlp13 AARAGEHGRGFAVVADEVRKLAERTQKSLSEIEANTNLLVQSINDMAESIKEQTAGITQI 661

FB1_Tlp13 AARAGEHGRGFAVVADEVRKLAERTQKSLSEIEANTNLLVQSINDMAESIKEQTAGITQI 661

CFCAN032805_Tlp13 AARAGEHGRGFAVVADEVRKLAERTQKSLSEIEANTNLLVQSINDMAESIKEQTAGITQI 661

BG2108_Tlp13 AARAGEHGRGFAVVADEVRKLAERTQKSLSEIEANTNLLVQSINDMAESIKEQTAGITQI 661

YF2105_Tlp13 AARAGEHGRGFAVVADEVRKLAERTQKSLSEIEANTNLLVQSINDMAESIKEQTAGITQI 661

YH503_Tlp13 AARAGEHGRGFAVVADEVRKLAERTQKSLSEIEANTNLLVQSINDMAESIKEQTAGITQI 661

BFRCA9557_Tlp13 AARAGEHGRGFAVVADEVRKLAERTQKSLSEIEANTNLLVQSINDMAESIKEQTAGITQI 661

YH502_Tlp13 AARAGEHGRGFAVVADEVRKLAERTQKSLSEIEANTNLLVQSINDMAESIKEQTAGITQI 661

OR12_Tlp13 AARAGEHGRGFAVVADEVRKLAERTQKSLSEIEANTNLLVQSINDMAESIKEQTAGITQI 661

00-1597_Tlp13 AARAGEHGRGFAVVADEVRKLAERTQKSLSEIEANTNLLVQSINDMAESIKEQTAGITQI 661

14903A_Tlp13 AARAGEHGRGFAVVADEVRKLAERTQKSLSEIEANTNLLVQSINDMAESIKEQTAGITQI 661

FJ3124_Tlp13 AARAGEHGRGFAVVADEVRKLAERTQKSLSEIEANTNLLVQSINDMAESIKEQTAGITQI 661

R14_Tlp13 AARAGEHGRGFAVVADEVRKLAERTQKSLSEIEANTNLLVQSINDMAESIKEQTAGITQI 661

MTVDSCj16_Tlp13 AARAGEHGRGFAVVADEVRKLAERTQKSLSEIEANTNLLVQSINDMAESIKEQTAGITQI 661

14980A_Tlp13 AARAGEHGRGFAVVADEVRKLAERTQKSLSEIEANTNLLVQSINDMAESIKEQTAGITQI 661

***** *:****:*::***:****:*****

FDAARGOS_295_Tlp1 NDAIAQLEMVTKENVEVANVTNNITNEVNQIAVAILEDVNKKRF----------- 700

14980A_Tlp1 NDAIAQLESVTKENVEVANATNSITNEVNQIAAAILEDVNKKRF----------- 700

CJ677CC527_Tlp1 NDAIAQLETVTKENVEVANVTNNITNEVNQIAAAILEDVNKKRF----------- 700

CJ677CC012_Tlp1 NDAIAQLETVTKENVEVANVTNNITNEVNQIAAAILEDVNKKRF----------- 700

4031_Tlp1 NDAIAQLETVTKENVEVANVTNNITNEVNQIAAAILEDVDKKRF----------- 700

81116_Tlp1 NDAIAQLETVTKENVEVANVTNNITNEVNQIAAAILEDVDKKRF----------- 700

35925B2_Tlp1 NDAIAQLETVTKENVEVANVTNNITNEVNQIAAAILEDVDKKRF----------- 700

M1_Tlp1 NDAIAQLETVTKENVEVANVTNNITNEVNQIAAAILEDVDKKRF----------- 700

PT14_Tlp1 NDAIAQLETVTKENVEVANVTNNITNEVNQIAAAILEDVDKKRF----------- 700

81-176_Tlp1 NDAIAQLETVTKENVEVANVTNNITNEVNQIAAAILEDVSKKRF----------- 700

CVMN29710_Tlp1 NDAIAQLETVTKENVEVANVTNNITNEVNQIAAAILEDVNKKRF----------- 700

FB1_Tlp1 NDAIAQLETVTKENVEVANVTNNITNEVNQIAAAILEDVNKKRF----------- 700

BG2108_Tlp1 NDAIAQLETVTKENVEVANVTNNITNEVNQIAAAILEDVNKKRF----------- 700

YF2108_Tlp1 NDAIAQLETVTKENVEVANVTNNITNEVNQIAAAILEDVNKKRF----------- 700

YH501_Tlp1 NDAIAQLETVTKENVEVANVTNNITNEVNQIAAAILEDVNKKRF----------- 700

RM4661_Tlp1 NDAIAQLETVTKENVEVANVTNNITNEVNQIAAAILEDVNKKRF----------- 700

F38011_Tlp1 NDAIAQLETVTKENVEVANVTNNITNEVNQIAAAILEDVDKKRF----------- 700

T1-21_Tlp1 NDAIAQLETVTKENVEVANVTNNITNEVNQIAAAILEDVDKKRF----------- 700

CG8421_Tlp1 NDAIAQLETVTKENVEVANVTNNITNEVNQIAAAILEDVNKKRF----------- 700

CJM1cam_Tlp1 NDAIAQLETVTKENVEVANVTNNITNEVNQIAAAILEDVNKKRF----------- 700

R14_Tlp1 NDAIAQLETVTKENVEVANVTNNITNEVNQIAAAILEDVNKKRF----------- 700

ICDCCJ07001_Tlp1 NDAIAQLETVTKENVEVANVTNNITNEVNQIAAAILEDVNKKRF----------- 700

RM3196_Tlp1 NDAIAQLETVTKENVEVANVTNNITNEVNQIAAAILEDVNKKRF----------- 700

NCTC11168_Tlp1 NDAIAQLETVTKENVEVANVTNNITNEVNQIAAAILEDVNKKRF----------- 700

00-2425_Tlp1 NDAIAQLETVTKENVEVANVTNNITNEVNQIAAAILEDVNKKRF----------- 700

IA3902_Tlp1 NDAIAQLETVTKENVEVANVTNNITNEVNQIAAAILEDVNKKRF----------- 700

RM1285_Tlp1 NDAIAQLETVTKENVEVANVTNNITNEVNQIAAAILEDVNKKRF----------- 700

00-0949_Tlp1 NDAIAQLETVTKENVEVANVTNNITNEVNQIAAAILEDVNKKRF----------- 700

01-1512_Tlp1 NDAIAQLETVTKENVEVANVTNNITNEVNQIAAAILEDVNKKRF----------- 700

FDAARGOS_422_Tlp1 NDAIAQLETVTKENVEVANVTNNITNEVNQIAAAILEDVNKKRF----------- 700

FORC_056_Tlp1 NDAIAQLETVTKENVEVANVTNNITNEVNQIAAAILEDVNKKRF----------- 700

32488_Tlp1 NDAIAQLETVTKENVEVANVTNNITNEVNQIAAAILEDVNKKRF----------- 700

CFSAN032806_Tlp1 NDAIAQLETVTKENVEVANVTNNITNEVNQIAAAILEDVNKKRF----------- 700

YH001_Tlp1 NDAIAQLETVTKENVEVANVTNNITNEVNQIAAAILEDVNKKRF----------- 700

00-6200_Tlp1 NDAIAQLETVTKENVEVANVTNNITNEVNQIAAAILEDVNKKRF----------- 700

RM1221_Tlp1 NDAIAQLETVTKENVEVANVTNNITNEVNQIAAAILEDVNKKRF----------- 700

S3_Tlp1 NDAIAQLETVTKENVEVANVTNNITNEVNQIAAAILEDVNKKRF----------- 700

FDAARGOS_421_Tlp1 NDAIAQLETVTKENVEVANVTNNITNEVNQIAAAILEDVNKKRF----------- 700

FJ3124_Tlp1 NDAIAQLETVTKENVEVANVTNNITNEVNQIAAAILEDVNKKRF----------- 700

00-1597_Tlp1 NDAIAQLETVTKENVEVANVTNNITNEVNQIAAAILEDVNKKRF----------- 700

15-537360_Tlp20 NESVAQIDQTTKDNVEIANESAIISSTVSDIANNILEDVKKKRF----------- 659

76339_Tlp20 NESVAQIDQTTKDNVEIANESAIISNTVSDIANNILEDVRKKRF----------- 659

HC2-48_Tlp20 NESVAQIDQTTKDNVEIANESAIISSTVSDIANNILEDVKKKRF----------- 600

CFSAN032805_Tlp20 NESVAQIDQTTKDNVEIANESAIISSTVSDIANNILEDVKKKRF----------- 658

CVMN29710_Tlp20 NESVAQIDQTTKDNVEIANESAIISSTVSDIANNILEDVKKKRF----------- 657

YH501_Tlp20 NESVAQIDQTTKDNVEIANESAIISSTVSDIANNILEDVKKKRF----------- 661

CF2-75_Tlp20 NESVAQIDQTTKDNVEIANESAIISSTVSDIANNILEDVKKKRF----------- 634

CO2-160_Tlp20 NESVAQIDQTTKDNVEIANESAIISSTVSDIANNILEDVKKKRF----------- 659

CO2-160_Tlp20b NESVAQIDQTTKDNVEIANESAIISSTVSDIANNILEDVKKKRF----------- 659

RM5611_Tlp20 NESVAQIDQTTKDNVEIANESAIISSTVSDIANNILEDVKKKRF----------- 659

14903A_Tlp20 NESVAQIDQTTKDNVEIANESAIISSTVSDIANNILEDVKKKRF----------- 661

YH502_Tlp20 NESVAQIDQTTKDNVEIANESAIISSTVSDIANNILEDVKKKRF----------- 658

RM3196_Tlp23 NDSVAQIDQTTKDNVEIANESAIISSTVSDIANNILEDVKKKRF----------- 659

ICDCCJ07001_Tlp23 NDSVAQIDQTTKDNVEIANESAIISSTVSDIANNILEDVKKKRF----------- 659

RM1285_Tlp2 NDSVAQIDQTTKDNVEIANESAIISSTVSDIANNILEDVKKKRF----------- 658

CFSAN032806_Tlp2 NDSVAQIDQTTKDNVEIANESAIISNTVSDIANNILEDVKKKRF----------- 659

RM1221_Tlp2 NDSVAQIDQTTKDNVEIANESAIISSTVSDIANNILEDVKKKRF----------- 659

S3_Tlp2 NDSVAQIDQTTKDNVEIANESAIISSTVSDIANNILEDVKKKRF----------- 659

FDAARGOS_422_Tlp2 NDSVAQIDQTTKDNVEIANESAIISSTVSDIANNILEDVKKKRF----------- 659

81-176_Tlp2 NDSVAQIDQTTKDNVEIANESAIISSTVSDIANNILEDVKKKRF----------- 659

F38011_Tlp2 NDSVAQIDQTTKDNVEIANESAIISSTVSDIANNILEDVKKKRF----------- 659

NCTC11168_Tlp2 NDSVAQIDQTTKDNVEIANESAIISSTVSDIANNILEDVKKKRF----------- 659

MTVDSCj07_Tlp2 NDSVAQIDQTTKDNVEIANESAIISSTVSDIANNILEDVKKKRF----------- 659

CJM1cam_Tlp24 NESVAQIDQTTKDNVEIANESAIISSTVSDIANNILEDVKKKRF----------- 656

M1_Tlp24 NESVAQIDQTTKDNVEIANESAIISSTVSDIANNILEDVKKKRF----------- 655

81116_Tlp2 NESVAQIDQTTKDNVEIANESAIISSTVSDIANNILEDVKKKRF----------- 659

4031_Tlp23 NESVAQIDQTTKDNVEIANESAIISSTVSDIANNILEDVKKKRF----------- 658

CVMN29710_Tlp14 NESVAQIDQTTKDNVEIANESAIISSTVSDIANNILEDVKKKRF----------- 656

RM4661_Tlp14 NESVAQIDQTTKDNVEIANESAIISNTVSDIANSILEDVEKEKVLIDCSLTPSLN 667

MG1116_Tlp14 NESVAQIDQTTKDNVEIANESAIISSTVSDIANNILEDVKKKRF----------- 649

BG2108_Tlp14 NESVAQIDQTTKDNVEIANESAIISSTVSDIANNILEDVKKKRF----------- 588

YF2105_Tlp14 NESVAQIDQTTKDNVEIANESAIISSTVSDIANNILEDVKKKRF----------- 617

YH502_Tlp14 NESVAQIDQTTKDNVEIANESAIISSTVSDIANNILEDVKKKRF----------- 667

WA333_Tlp14 NESVAQIDQTTKDNVEIANESAIISSTVSDIANNILEDVKKKRF----------- 588

BP3181_Tlp14 NESVAQIDQTTKDNVEIANESAIISSTVSDIANNILEDVKKKRF----------- 656

ZV1224_Tlp14a NESVAQIDQTTKDNVEIANESAIISSTVSDIANNILEDVKKKRF----------- 656

ZV1224_Tlp14b NESVAQIDQTTKDNVEIANESAIISSTVSDIANNILEDVKKKRF----------- 656

YH503_Tlp14 NESVAQIDQTTKDNVEIANESAIISSTVSDIANNILEDVKKKRF----------- 667

14903A_Tlp14 NESVAQIDQTTKDNVEIANESAIISSTVSDIANNILEDVKKKRF----------- 656

OR12_Tlp14 NESVAQIDQTTKDNVEIANESAIISSTVSDIANNILEDVKKKRF----------- 656

CFSAN032805_Tlp14 NESVAQIDQTTKDNVEIANESAIISSTVSDIANNILEDVKKKRF----------- 667

BFR-CA-9557_Tlp14 NESVAQIDQTTKDNVEIANESAIISSTVSDIANNILEDVKKKRF----------- 656

15-537360_Tlp14 NESVAQIDQTTKDNVEIANESAIISNTVSDIANNILEDVKKKRF----------- 656

YH501_Tlp14 NESVAQIDQTTKDNVEIANESAIISSTVSDIANNILEDVKKKRF----------- 656

T1-21_Tlp19b NDSVAQIDQTTKDNVEIANESAIISSTVSDIANNILEDVKKKRF----------- 611

CG8421_Tlp14 NDSVAQIDQTTKDNVEIANESAIISSTVSDIANNILEDVKKKRF----------- 650

MTVDSCj16_Tlp14 NDSVAQIDQTTKDNVEIANESAIISSTVSDIANNILEDVKKKRF----------- 656

01-1512_Tlp14 NDSVAQIDQTTKDNVEIANESAIISSTVSDIANNILEDVKKKRF----------- 656

00-0949_Tlp14 NDSVAQIDQTTKDNVEIANESAIISSTVSDIANNILEDVKKKRF----------- 656

MTVDSCj13_Tlp14 NDSVAQIDQTTKDNVEIANESAIISSTVSDIANNILEDVKKKRF----------- 656

S3_Tlp14 NDSVAQIDQTTKDNVEIANESAIISSTVSDIANNILEDVKKRGFN---------- 657

PT14_Tlp14 NDSVAQIDQTTKDNVEIANESAIISSTVSDIANNILEDVKKKRF----------- 656

14980A_Tlp14 NDSVAQIDQTTKDNVEIANESAIISSTVSDIANNILEDVKKKRF----------- 672

FJ3124_Tlp14 NDSVAQIDQTTKDNVEIANESAIISSTVSDIANNILEDVKKKRF----------- 656

00-1597_Tlp14 NDSVAQIDQTTKDNVEIANESAIISSTVSDIANNILEDVKKKR------------ 655

R14_Tlp14 NDSVAQIDQTTKDNVEIANESAIISSTVSDIANNILEDVKKKRFF---------- 657

CG8421_Tlp25 NDSVAQIDQTTKDNVEIANESAIISSTVSDIANNILEDVKKKRF----------- 447

HC2-48_Tlp3 NESVAQIDQTTKDNVEIANESAIISSTVSDIANNILEDVKKKRF----------- 591

35925B2_Tlp3 NESVAQIDQTTKDNVEIANESAIISSTVSDIANNILEDVKKKRF----------- 665

14980A_Tlp3 NDSVAQIDQTTKDNVEIANESAIISSTVSDIANNILEDVKKKRF----------- 662

00-1597_Tlp3b NDSVAQIDQTTKDNVEIANESAIISSTVSDIANNILEDVKKKRF----------- 651

ICDCCJ07001_Tlp3 NDSVAQIDQTTKDNVEIANESAIISSTVSDIANNILEDVKKKR------------ 650

RM3196_Tlp3 NDSVAQIDQTTKDNVEIANESAIISSTVSDIAN---------------------- 640

RM1875_Tlp3 NESVAQIDQTTKDNVEIANESAIISNTVSDIANNILEDVKKKRF----------- 651

CF2-75_Tlp3 NESVAQIDQTTKDNVEIANESAIISSTVSDIANNILEDVKKKRF----------- 634

RM5611_Tlp3 NESVAQIDQTTKDNVEIANESAIISSTVSDIANNILEDVKKKRF----------- 651

MTVDSCj16_Tlp3 NDSVAQIDQTTKDNVEIANESAIISSTVSDIANNILEDVKKKRF----------- 651

01-1512_Tlp3 NDSVAQIDQTTKDNVEIANESAIISSTVSDIANNILEDVKKKRF----------- 651

MTVDSCj13_Tlp3 NDSVAQIDQTTKDNVEIANESAIISSTVSDIANNILEDVKKKRF----------- 651

32488_Tlp3a NDSVAQIDQTTKDNVEIANESAIISSTVSDIANNILEDVKKKRF----------- 651

81116_Tlp3 NDSVAQIDQTTKDNVEIANESAIISSTVSDIANNILEDVKKKRF----------- 651

32488_Tlp3b NDSVAQIDQTTKDNVEIANESAIISSTVSDIANNILEDVKKKRF----------- 651

FB1_Tlp3 NESVAQIDQTTKDNVEIANESAIISSTVSDIANNILEDVKKKRF----------- 651

PT14_Tlp3 NDSVAQIDQTTKDNVEIANESAIISSTVSDIANNILEDVKKKRF----------- 651

00-6200_Tlp3a NDSVAQIDQTTKDNVEIANESAIISSTVSDIANNILEDIKKKRF----------- 651

RM1221_Tlp3 NDSVAQIDQTTKDNVEIANESAIISSTVSDIANNILEDVKKKRF----------- 651

S3_Tlp3 NDSVAQIDQTTKDNVEIANESAIISSTVSDIANNILEDVKKKRF----------- 651

FDAARGOS_421_Tlp3 NDSVAQIDQTTKDNVEIANESAIISSTVSDIANNILEDVKKKRF----------- 662

CFSAN032806_Tlp3 NDSVAQIDQTAKDNVEIANESAIISNTVSDIANNILEDVKKKRF----------- 662

IA3901_Tlp3b NDSVAQIDQTTKDNVEIANESAIISSTVSDIANNILEDIKKKRF----------- 651

00-6200_Tlp3b NDSVAQIDQTTKDNVEIANESAIISSTVSDIANNILEDIKKKRF----------- 651

BCW_6290_Tlp3b NDSVAQIDQTTKDNVEIANESAIISSTVSDIANNILEDIKKKRF----------- 651

00-2425_Tlp3a NDSVAQIDQTTKDNVEIANESAIISSTVSDIANNILEDVKKKRF----------- 651

00-2425_Tlp3b NDSVAQIDQTTKDNVEIANESAIISSTVSDIANNILEDIKKKRF----------- 651

YH001_Tlp3a NDSVAQIDQTTKDNVEIANESAIISSTVSDIANNILEDIKKKRF----------- 651

YH001_Tlp3b NDSVAQIDQTTKDNVEIANESAIISSTVSDIANNILEDIKKKRF----------- 651

00-0949_Tlp3 NDSVAQIDQTTKDNVEIANESAIISSTVSDIANNILEDVKKKRF----------- 651

NCTC11168_Tlp3 NDSVAQIDQTTKDNVEIANESAIISSTVSDIANNILEDVKKKRF----------- 662

F38011_Tlp3 NDSVAQIDQTTKDNVEIANESAIISSTVSDIANNILEDVKKKRF----------- 651

RM1285_Tlp3 NDSVAQIDQTTKDNVEIANESAIISSTVSDIANNILEDVKKKRF----------- 651

FDAARGOS_422_Tlp3 NDSVAQIDQTTKDNVEIANESAIISSTVSDIANNILEDVKKKRF----------- 662

MTVDSCj07_Tlp3 NDSVAQIDQTTKDNVEIANESAIISSTVSDIANNILEDVKKKRF----------- 651

IA3901_Tlp3a NDSVAQIDQTTKDNVEIANESAIISSTVSDIANNILEDVKKKRF----------- 651

BCW_6290_Tlp3a NDSVAQIDQTTKDNVEIANESAIISSTVSDIANNILEDVKKKRF----------- 651

T1-21_Tlp19a NDSVAQIDQTTKDNVEIANESAIISSTVSDIANNILEDVKKKRF----------- 606

CJ677CC012_Tlp3 NDSVAQIDQTTKDNVEIANESAIISSTVSDIANNILEDVKKKRF----------- 509

CJM1cam_Tlp3 NESVAQIDQTTKDNVEIANESAIISSTVSDIANNILEDVKKKRF----------- 651

M1_Tlp3 NESVAQIDQTTKDNVEIANESAIISSTVSDIANNILEDVKKKRF----------- 651

4031_Tlp3 NESVAQIDQTTKDNVEIANESAIISSTVSDIANNILEDVKKKRF----------- 651

R14_Tlp3 NDSVAQIDQTTKDNVEIANESAIISSTVSDIANNILEDVKKKRF----------- 651

FDAARGOS_295_Tlp21 NDSVAQIDQTTKDNVEIANESAIISSTVSDIANNILEDVKKKRF----------- 653

FORC_046_Tlp4 NDSVAQIDQTTKDNVEIANESAIISSTVSDIANNILEDVKKKRF----------- 665

FDAARGOS_422_Tlp4 NDSVAQIDQTTKDNVEIANESAIISSTVSDIANNILEDVKKKRF----------- 665

ICDCCJ07001_Tlp4 NDSVAQIDQTTKDNVEIANESAIISSTVSDIANNILEDVKKKRF----------- 665

RM3196_Tlp4 NDSVAQIDQTTKDNVEIANESAIISSTVSDIANNILEDVKKKRF----------- 665

T1-21_Tlp4 NDSVAQIDQTTKDNVEIANESAIISSTVSDIANNILEDVKKKRF----------- 665

F38011_Tlp4 NDSVAQIDQTTKDNVEIANESAIISSTVSDIANNILEDVKKKRF----------- 665

HF5-4A-4_Tlp22 NDSVAQIDQTTKDNVEIANESAIISSTVSDIANNILEDVKKKRF----------- 611

00-0949_Tlp4 NDSVAQIDQTTKDNVEIANESAIISSTVSDIANNILEDVKKKRF----------- 664

01-1512_Tlp4 NDSVAQIDQTTKDNVEIANESAIISSTVSDIANNILEDVKKKRF----------- 665

81-176_Tlp4 NDSVAQIDQTTKDNVEIANESAIISSTVSDIANNILEDVKKKRF----------- 665

32488_Tlp4 NDSVAQIDQTTKDNVEIANESAIISSTVSDIANNILEDVKKKRF----------- 665

NCTC11168_Tlp4 NDSVAQIDQTTKDNVEIANESAIISSTVSDIANNILEDVKKKRF----------- 665

CFSAN032806_Tlp4 NDSVAQIDQTTKDNVEIANESAIISSTVSDIANNILEDVKKKRF----------- 665

81116_Tlp4 NDSVAQIDQTTKDNVEIANESAIISSTVSDIANNILEDVKKKRF----------- 665

RM1285_Tlp12 NDSVAQIDQTTKDNVEIANESAIISSTVSDIANNILEDVKKKRF----------- 661

PT14_Tlp12 NDSVAQIDQTTKDNVEIANESAIISSTVSDIANNILEDVKKKRF----------- 662

MTVJDCj07_Tlp12 NDSVAQIDQTTKDNVEIANESAIISSTVSDIANNILEDVKKKRF----------- 662

RM1221_Tlp12 NDSVAQIDQTTKDNVEIANESAIISSTVSDIANNILEDVKKKRF----------- 662

FDAARGOS_421_Tlp12 NDSVAQIDQTTKDNVEIANESAIISSTVSDIANNILEDVKKKRF----------- 662

35925B2_Tlp12 NESVAQIDQTTKDNVEIANESAIISSTVSDIANNILEDVKKKRF----------- 662

CJM1cam_Tlp12 NDSVAQIDQTTKDNVEIANESAIISSTVSDIANNILEDVKKKRF----------- 662

M1_Tlp12 NDSVAQIDQTTKDNVEIANESAIISSTVSDIANNILEDVKKKRF----------- 662

S3_Tlp12 NDSVAQIDQTTKDNVEIANESAIISSTVSDIANNILEDVKKKRF----------- 662

00-1597_Tlp12 NDSVAQIDQTTKDNVEIANESAIISSTVSDIANNILEDVKKKRF----------- 662

R14_Tlp12 NDSVAQIDQTTKDNVEIANESAIISSTVSDIANNILEDVKKKRF----------- 662

RM1875_Tlp15 NESVAQIDQTTKDNVEIANESAIISSTVSDIANNILEDVKKKRF----------- 579

YH503_Tlp16 NESVAQIDQTTKDNVEIANESAIISSTVSDIANNILEDVKKKRF----------- 579

FB1_Tlp16 NESVAQIDQTTKDNVEIANESAIISSTVSDIANNILEDVKKKRF----------- 579

BFR-CA-9557_Tlp16 NESVAQIDQTTKDNVEIANESAIISSTVSDIANSILEDVKKKRF----------- 578

15-537360_Tlp16 NESVAQIDQTTKDNVEIANESAIISSTVSDIANNILEDVKKKRF----------- 579

OR12_Tlp16 NESVAQIDQTTKDNVEIANESAIISSTVSDIANNILEDVKKKRF----------- 579

YH502_Tlp16 NESVAQIDQTTKDNVEIANESAIISSTVSDIANNILEDVKKKRF----------- 579

14903A_Tlp16 NESVAQIDQTTKDNVEIANESAIISSTVSDIANNILEDVKKKRF----------- 579

HC2-48_Tlp16 NESVAQIDQTTKDNVEIANESAIISSTVSDIANNILEDVKKKRF----------- 521

RM5611_Tlp16 NESVAQIDQTTKDNVEIANESAIISSTVSDIANNILEDVKKKRF----------- 579

00-2425_Tlp11 NDSVAQIDQTTKDNVEIANESAIISSTVSDIANNILEDVKKKRF----------- 706

00-6200_Tlp11 NDSVAQIDQTTKDNVEIANESAIISSTVSDIANNILEDVKKKRF----------- 706

YH001_Tlp11 NDSVAQIDQTTKDNVEIANESAIISSTVSDIANNILEDVKKKRF----------- 706

IA3902_Tlp11 NDSVAQIDQTTKDNVEIANESAIISSTVSDIANNILEDVKKKRF----------- 706

BCW_6290_Tlp11 NDSVAQIDQTTKDNVEIANESAIISSTVSDIANNILEDVKKKRF----------- 706

76339_Tlp18 NESVAQIDQTTKDNVEIANESAIISNTVSDIANNILEDVRKKRF----------- 705

4031_Tlp17 NESVAQIDQTTKDNVEIANESAIISSTVSDIANNILEDVKKKRF----------- 706

MTVDSCj13_Tlp13 NDSVAQIDQTTKDNVEIANESAIISSTVSDIANNILEDVKKKRF----------- 705

RM1875_Tlp13 NESVAQIDQTTKDNVEIANESAIISNTVSDIANNIL------------------- 697

CF2-75_Tlp13 NESVAQIDQTTKDNVEIANESAIISSTVSDIANNILEDVKKKRF----------- 677

15-537360_Tlp13 NESVAQIDQTTKDNVEIANESAIISSTVSDIANNILEDVKKKRF----------- 705

CVMN29710_Tlp13 NESVAQIDQTTKDNVEIANESAIISSTVSDIANNILEDVKKKRF----------- 705

FB1_Tlp13 NESVAQIDQTTKDNVEIANESAIISSTVSDIANNILEDVKKKRF----------- 705

CFCAN032805_Tlp13 NESVAQIDQTTKDNVEIANESAIISSTVSDIANNILEDVKKKRF----------- 705

BG2108_Tlp13 NESVAQIDQTTKDNVEIANESAIISSTVSDIANNILEDVKKKRF----------- 705

YF2105_Tlp13 NESVAQIDQTTKDNVEIANESAIISSTVSDIANNILEDVKKKRF----------- 705

YH503_Tlp13 NESVAQIDQTTKDNVEIANESAIISSTVSDIANNILEDVKKKRF----------- 705

BFRCA9557_Tlp13 NESVAQIDQTTKDNVEIANESAIISSTVSDIANSILEDVKKKRF----------- 705

YH502_Tlp13 NESVAQIDQTTKDNVEIANESAIISSTVSDIANNILEDVKKKRF----------- 705

OR12_Tlp13 NDSVAQIDQTTKDNVEIANESAIISSTVSDIANNILEDVKKKRF----------- 705

00-1597_Tlp13 NDSVAQIDQTTKDNVEIANESAIISSTVSDIANNILEDVKKKRF----------- 705

14903A_Tlp13 NESVAQIDQTTKDNVEIANESAIISSTVSDIANNILEDVKKKRF----------- 705

FJ3124_Tlp13 NDSVAQIDQTTKDNVEIANESAIISSTVSDIANNILEDVKKKRF----------- 705

R14_Tlp13 NDSVAQIDQTTKDNVEIANESAIISSTVSDIANNILEDVKKKRF----------- 705

MTVDSCj16_Tlp13 NDSVAQIDQTTKDNVEIANESAIISSTVSDIANNILEDVKKKRF----------- 705

14980A_Tlp13 NDSVAQIDQTTKDNVEIANESAIISSTVSDIANNILEDVKKKRF----------- 705

*:::**:: .:*:***:** : *:. *.:**
