# Supplementary material for: Diversity of transducer-like proteins (Tlps) in Campylobacter
Source: PLoS One. 2019 Mar 25;14(3):e0214228. doi: 10.1371/journal.pone.0214228 (PMC6433261; doi:10.1371/journal.pone.0214228)
Supplement: S2 Archive — (ZIP) [file pone.0214228.s016.zip › Alignment M.docx]

Alignment M.Tlp1 protein sequence comparisons

CLUSTAL O(1.2.4) multiple sequence alignment 2018/04/17

FDAARGOS_295 MFKSLNVGLKLVFSVAIVVVIGLVILISLVTKQVSQSITENAEDIIASVSKEHAVQVQGI 60

14980A MFKSLNIGLKLIFSVAAVVVIGLVILISLITKQVSQNITKNTEDILASITKEYATQTQGI 60

CJ677CC527 MFKSLNIGLKLIFSVATVVVIGLIILISLITKQVSQNITKNTEDILASITKEYATQTQGI 60

CJ677CC012 MFKSLNIGLKLIFSVATVVVIGLIILISLITKQVSQNITKNTEDILASITKEYATQTQGI 60

4031 MFKSLNIGLKLIFSVATVVVIGLVILISLITKQVSQNITKNTEDILASITKEYATQTQGI 60

81116 MFKSLNIGLKLIFSVATVVVIGLVILISLITKQVSQNITKNTEDILASITKEYATQTQGI 60

35925B2 MFKSLNIGLKLIFSVATVVVIGLVILISLITKQVSQNITKNTEDILASITKEYATQTQGI 60

M1 MFKSLNIGLKLIFSVATVVVIGLVILISLITKQVSQNITKNTEDILASITKEYATQTQGI 60

PT14 MFKSLNIGLKLIFSVATVVVIGLVILISLITKQVSQNITKNTEDILASITKEYATQTQGI 60

81-176 MFKSLNIGLKLIFSVAAVVVIGLVILISLITKQVSQNITKNTEDILASITKEYATQTQGI 60

CVMN29710 MFKSLNIGLKLIFSVAAVVVIGLVILISLITKQVSQNITKNTEDILASITKEYATQTQGI 60

FB1 MFKSLNIGLKLIFSVAAVVVIGLVILISLITKQVSQNITKNTEDILASITKEYATQTQGI 60

BG2108 MFKSLNIGLKLIFSVAAVVVIGLVILISLITKQVSQNITKNTEDILASITKEYATQTQGI 60

YF2108 MFKSLNIGLKLIFSVAAVVVIGLVILISLITKQVSQNITKNTEDILASITKEYATQTQGI 60

YH501 MFKSLNIGLKLIFSVAAVVVIGLVILISLITKQVSQNITKNTEDILASITKEYATQTQGI 60

RM4661 MFKSLNIGLKLIFSVAAVVVIGLVILISLITKQVSQNITKNTEDILASITKEYATQTQGI 60

F38011 MFKSLNIGLKLIFSVAAVVVIGLVILISLITKQVSQNITKNTEDILASITKEYATQTQGI 60

T1-21 MFKSLNIGLKLIFSVAAVVVIGLVILISLITKQVSQNITKNTEDILASITKEYATQTQGI 60

CG8421 MFKSLNIGLKLIFSVAAVVVIGLVILISLITKQVSQNITKNTEDILASITKEYATQTQGI 60

CJM1cam MFKSLNIGLKLIFSVAAVVVIGLVILISLITKQVSQNITKNTEDILASITKEYATQTQGI 60

R14 MFKSLNIGLKLIFSVAAVVVIGLVILISLITKQVSQNITKNTEDILASITKEYATQTQGI 60

ICDCCJ07001 MFKSLNIGLKLIFSVAAVVVIGLVILISLITKQVSQNITKNTEDILASITKEYATQTQGI 60

RM3196 MFKSLNIGLKLIFSVAAVVVIGLVILISLITKQVSQNITKNTEDILASITKEYATQTQGI 60

NCTC11168 MFKSLNIGLKLIFSVAAVVVIGLVILISLITKQVSQNITKNTEDILASITKEYATQTQGI 60

00-2425 MFKSLNIGLKLIFSVAAVVVIGLVILISLITKQVSQNITKNTEDILASITKEYATQTQGI 60

IA3902 MFKSLNIGLKLIFSVAAVVVIGLVILISLITKQVSQNITKNTEDILASITKEYATQTQGI 60

RM1285 MFKSLNIGLKLIFSVAAVVVIGLVILISLITKQVSQNITKNTEDILASITKEYATQTQGI 60

00-0949 MFKSLNIGLKLIFSVAAVVVIGLVILISLITKQVSQNITKNTEDILASITKEYATQTQGI 60

01-1512 MFKSLNIGLKLIFSVAAVVVIGLVILISLITKQVSQNITKNTEDILASITKEYATQTQGI 60

FDAARGOS_422 MFKSLNIGLKLIFSVAAVVVIGLVILISLITKQVSQNITKNTEDILASITKEYATQTQGI 60

FORC_056 MFKSLNIGLKLIFSVAAVVVIGLVILISLITKQVSQNITKNTEDILASITKEYATQTQGI 60

32488 MFKSLNIGLKLIFSVAAVVVIGLVILISLITKQVSQNITKNTEDILASITKEYATQTQGI 60

CFSAN032806 MFKSLNIGLKLIFSVAAVVVIGLVILISLITKQVSQNITKNTEDILASITKEYATQTQGI 60

YH001 MFKSLNIGLKLIFSVAAVVVIGLVILISLITKQVSQNITKNTEDILASITKEYATQTQGI 60

00-6200 MFKSLNIGLKLIFSVAAVVVIGLVILISLITKQVSQNITKNTEDILASITKEYATQTQGI 60

RM1221 MFKSLNIGLKLIFSVAAVVVIGLVILISLITKQVSQNITKNTEDILASITKEYATQTQGI 60

S3 MFKSLNIGLKLIFSVAAVVVIGLVILISLITKQVSQNITKNTEDILASITKEYATQTQGI 60

FDAARGOS_421 MFKSLNIGLKLIFSVAAVVVIGLVILISLITKQVSQNITKNTEDILASITKEYATQTQGI 60

FJ3124 MFKSLNIGLKLIFSVAAVVVIGLVILISLITKQVSQNITKNTEDILASITKEYATQTQGI 60

00-1597 MFKSLNIGLKLIFSVAAVVVIGLVILISLITKQVSQNITKNTEDILASITKEYATQTQGI 60

******:****:**** ******:*****:******.**:*:***:**::**:*.*.***

FDAARGOS_295 FNEIIALSKTVSNTLTEMFRVASKENLDMDSITNIVTNTFDNSVYSNFTYLYLIDPPEYF 120

14980A FGEMIALNKSISGTLTEMFRSTSKEDLDIDNITNIITNTFDNSAYSNFTYLYLIDPPEYF 120

CJ677CC527 FGEMIALNKSISGTLTEMFRSSSKENLDIDSITNIITNTFDNSAYSNFTYLYLIDPPEYF 120

CJ677CC012 FGEMIALNKSISGTLTEMFRSSSKENLDIDSITNIITNTFDNSAYSNFTYLYLIDPPEYF 120

4031 FGEMIALNKSISGTLTEMFRSTSKEDLDIDNITNIITNTFDNSAYSNFTYLYLIDPPEYF 120

81116 FGEMIALNKSISGTLTEMFRSTSKEDLDIDNITNIITNTFDNSAYSNFTYLYLIDPPEYF 120

35925B2 FGEMIALNKSISGTLTEMFRSTSKEDLDIDNITNIITNTFDNSAYSNFTYLYLIDPPEYF 120

M1 FGEMIALNKSISGTLTEMFRSTSKEDLDIDNITNIITNTFDNSAYSNFTYLYLIDPPEYF 120

PT14 FGEMIALNKSISGTLTEMFRSTSKEDLDIDNITNIITNTFDNSAYSNFTYLYLIDPPEYF 120

81-176 FGEMIALNKSISGTLTEMFRSTSKEDLDIDNITNIITNTFDNSAYSNFTYLYLIDPPEYF 120

CVMN29710 FGEMIALNKSISGTLTEMFRSTSKEDLDIDNITNIITNTFDNSAYSNFTYLYLIDPPEYF 120

FB1 FGEMIALNKSISGTLTEMFRSTSKEDLDIDNITNIITNTFDNSAYSNFTYLYLIDPPEYF 120

BG2108 FGEMIALNKSISGTLTEMFRSTSKEDLDIDNITNIITNTFDNSAYSNFTYLYLIDPPEYF 120

YF2108 FGEMIALNKSISGTLTEMFRSTSKEDLDIDNITNIITNTFDNSAYSNFTYLYLIDPPEYF 120

YH501 FGEMIALNKSISGTLTEMFRSTSKEDLDIDNITNIITNTFDNSAYSNFTYLYLIDPPEYF 120

RM4661 FGEMIALNKSISGTLTEMFRSTSKEDLDIDNITNIITNTFDNSAYSNFTYLYLIDPPEYF 120

F38011 FGEMIALNKSISGTLTEMFRSTSKEDLDIDNITNIITNTFDNSVYSNFTYLYLIDPPEYF 120

T1-21 FGEMIALNKSISGTLTEMFRSTSKEDLDIDNITNIITNTFDNSVYSNFTYLYLIDPPEYF 120

CG8421 FGEMIALNKSISGTLTEMFRSTSKEDLDIDNITNIITNTFDNSAYSNFTYLYLIDPPEYF 120

CJM1cam FGEMIALNKSISGTLTEMFRSTSKEDLDIDNITNIITNTFDNSAYSNFTYLYLIDPPEYF 120

R14 FGEMIALNKSISGTLTEMFRSTSKEDLDIDNITNIITNTFDNSAYSNFTYLYLIDPPEYF 120

ICDCCJ07001 FGEMIALNKSISGTLTEMFRSTSKEDLDIDNITNIITNTFDNSAYSNFTYLYLIDPPEYF 120

RM3196 FGEMIALNKSISGTLTEMFRSTSKEDLDIDNITNIITNTFDNSAYSNFTYLYLIDPPEYF 120

NCTC11168 FGEMIALNKSISGTLTEMFRSTSKEDLDIDNITNIITNTFDNSAYSNFTYLYLIDPPEYF 120

00-2425 FGEMIALNKSISGTLTEMFRSTSKEDLDIDNITNIITNTFDNSAYSNFTYLYLIDPPEYF 120

IA3902 FGEMIALNKSISGTLTEMFRSTSKEDLDIDNITNIITNTFDNSAYSNFTYLYLIDPPEYF 120

RM1285 FGEMIALNKSISGTLTEMFRSTSKEDLDIDNITNIITNTFDNSAYSNFTYLYLIDPPEYF 120

00-0949 FGEMIALNKSISGTLTEMFRSTSKEDLDIDNITNIITNTFDNSAYSNFTYLYLIDPPEYF 120

01-1512 FGEMIALNKSISGTLTEMFRSTSKEDLDIDNITNIITNTFDNSAYSNFTYLYLIDPPEYF 120

FDAARGOS_422 FGEMIALNKSISGTLTEMFRSTSKEDLDIDNITNIITNTFDNSAYSNFTYLYLIDPPEYF 120

FORC_056 FGEMIALNKSISGTLTEMFRSTSKEDLDIDNITNIITNTFDNSAYSNFTYLYLIDPPEYF 120

32488 FGEMIALNKSISGTLTEMFRSTSKEDLDIDNITNIITNTFDNSAYSNFTYLYLIDPPEYF 120

CFSAN032806 FGEMIALNKSISGTLTEMFRSTSKEDLDIDNITNIITNTFDNSAYSNFTYLYLIDPPEYF 120

YH001 FGEMIALNKSISGTLTEMFRSTSKEDLDIDNITNIITNTFDNSAYSNFTYLYLIDPPEYF 120

00-6200 FGEMIALNKSISGTLTEMFRSTSKEDLDIDNITNIITNTFDNSAYSNFTYLYLIDPPEYF 120

RM1221 FGEMIALNKSISGTLTEMFRSTSKEDLDIDNITNIITNTFDNSAYSNFTYLYLIDPPEYF 120

S3 FGEMIALNKSISGTLTEMFRSTSKEDLDIDNITNIITNTFDNSAYSNFTYLYLIDPPEYF 120

FDAARGOS_421 FGEMIALNKSISGTLTEMFRSTSKEDLDIDNITNIITNTFDNSAYSNFTYLYLIDPPEYF 120

FJ3124 FGEMIALNKSISGTLTEMFRSTSKEDLDIDNITNIITNTFDNSAYSNFTYLYLIDPPEYF 120

00-1597 FGEMIALNKSISGTLTEMFRSTSKEDLDIDNITNIITNTFDNSAYSNFTYLYLIDPPEYF 120

*.*:***.*::*.******* :***:**:*.****:*******.****************

FDAARGOS_295 KEKSKFFNTQNGKFVMLYVDEETDNKGGIKAIQASDEIVNLQVVQDILKKAKYGENKVYI 180

14980A KEESKFFNTQSGKFVMLYADEEKDNKGGIKAIQASDEIANLQVVQDILKKAKYGENKVYI 180

CJ677CC527 KEESKFFNTQSGKFVMLYVDEEKDSKGGIKAIQASDEIANLQVVQDILKKAKYGENKVYI 180

CJ677CC012 KEESKFFNTQSGKFVMLYVDEEKDGKGGIKAIQASDEIANLQVVQDILKKAKYGENKVYI 180

4031 KEESKFFNTQSGKFVMLYADEEKDNKGGIKAIQASDEIANLQVVQDILKKAKYGENKVYI 180

81116 KEESKFFNTQSGKFVMLYADEEKDNKGGIKAIQASDEIANLQVVQDILKKAKYGENKVYI 180

35925B2 KEESKFFNTQSGKFVMLYADEEKDNKGGIKAIQASDEIANLQVVQDILKKAKYGENKVYI 180

M1 KEESKFFNTQSGKFVMLYADEEKDNKGGIKAIQASDEIANLQVVQDILKKAKYGENKVYI 180

PT14 KEESKFFNTQSGKFVMLYADEEKDNKGGIKAIQASDEIANLQVVQDILKKAKYGENKVYI 180

81-176 KEESKFFNTQSGKFVMLYADEEKDNKGGIKAIQASDEIANLQVVQDILKKAKYGENKVYI 180

CVMN29710 KEESKFFNTQSGKFVMLYADEEKDNKGGIKAIQASDEIANLQVVQDILKKAKYGENKVYI 180

FB1 KEESKFFNTQSGKFVMLYADEEKDNKGGIKAIQASDEIANLQVVQDILKKAKYGENKVYI 180

BG2108 KEESKFFNTQSGKFVMLYADEEKDNKGGIKAIQASDEIANLQVVQDILKKAKYGENKVYI 180

YF2108 KEESKFFNTQSGKFVMLYADEEKDNKGGIKAIQASDEIANLQVVQDILKKAKYGENKVYI 180

YH501 KEESKFFNTQSGKFVMLYADEEKDNKGGIKAIQASDEIANLQVVQDILKKAKYGENKVYI 180

RM4661 KEESKFFNTQSGKFVMLYADEEKDNKGGIKAIQASDEIANLQVVQDILKKAKYGENKVYI 180

F38011 KEESKFFNTQSGKFVMLYADEEKDNKGGIKAIQASDEIANLQVVQDILKKAKYGENKVYI 180

T1-21 KEESKFFNTQSGKFVMLYADEEKDNKGGIKAIQASDEIANLQVVQDILKKAKYGENKVYI 180

CG8421 KEESKFFNTQSGKFVMLYADEEKDNKGGIKAIQASDEIANLQVVQDILKKAKYGENKVYI 180

CJM1cam KEESKFFNTQSGKFVMLYADEEKDNKGGIKAIQASDEIANLQVVQDILKKAKYGENKVYI 180

R14 KEESKFFNTQSGKFVMLYADEEKDNKGGIKAIQASDEIANLQVVQDILKKAKYGENKVYI 180

ICDCCJ07001 KEESKFFNTQSGKFVMLYADEEKDNKGGIKAIQASDEIANLQVVQDILKKAKYGENKVYI 180

RM3196 KEESKFFNTQSGKFVMLYADEEKDNKGGIKAIQASDEIANLQVVQDILKKAKYGENKVYI 180

NCTC11168 KEESKFFNTQSGKFVMLYADEEKDNKGGIKAIQASDEIANLQVVQDILKKAKYGENKVYI 180

00-2425 KEESKFFNTQSGKFVMLYADEEKDNKGGIKAIQASDEIANLQVVQDILKKAKYGENKVYI 180

IA3902 KEESKFFNTQSGKFVMLYADEEKDNKGGIKAIQASDEIANLQVVQDILKKAKYGENKVYI 180

RM1285 KEESKFFNTQSGKFVMLYADEEKDNKGGIKAIQASDEIANLQVVQDILKKAKYGENKVYI 180

00-0949 KEESKFFNTQSGKFVMLYADEEKDNKGGIKAIQASDEIANLQVVQDILKKAKYGENKVYI 180

01-1512 KEESKFFNTQSGKFVMLYADEEKDNKGGIKAIQASDEIANLQVVQDILKKAKYGENKVYI 180

FDAARGOS_422 KEESKFFNTQSGKFVMLYADEEKDNKGGIKAIQASDEIANLQVVQDILKKAKYGENKVYI 180

FORC_056 KEESKFFNTQSGKFVMLYADEEKDNKGGIKAIQASDEIANLQVVQDILKKAKYGENKVYI 180

32488 KEESKFFNTQSGKFVMLYADEEKDNKGGIKAIQASDEIANLQVVQDILKKAKYGENKVYI 180

CFSAN032806 KEESKFFNTQSGKFVMLYADEEKDNKGGIKAIQASDEIANLQVVQDILKKAKYGENKVYI 180

YH001 KEESKFFNTQSGKFVMLYADEEKDNKGGIKAIQASDEIANLQVVQDILKKAKYGENKVYI 180

00-6200 KEESKFFNTQSGKFVMLYADEEKDNKGGIKAIQASDEIANLQVVQDILKKAKYGENKVYI 180

RM1221 KEESKFFNTQSGKFVMLYADEEKDNKGGIKAIQASDEIANLQVVQDILKKAKYGENKVYI 180

S3 KEESKFFNTQSGKFVMLYADEEKDNKGGIKAIQASDEIANLQVVQDILKKAKYGENKVYI 180

FDAARGOS_421 KEESKFFNTQSGKFVMLYADEEKDNKGGIKAIQASDEIANLQVVQDILKKAKYGENKVYI 180

FJ3124 KEESKFFNTQSGKFVMLYADEEKDNKGGIKAIQASDEIANLQVVQDILKKAKYGENKVYI 180

00-1597 KEESKFFNTQSGKFVMLYADEEKDNKGGIKAIQASDEIANLQVVQDILKKAKYGENKVYI 180

**:*******.*******.***.*.*************.*********************

FDAARGOS_295 GRPIRMNLEDQDFDAVNIAMPIFNRKNQVVGVVGMTLDFSAIAAYLLDPKSQKYDGELRV 240

14980A GRPIKMNLEGQDFDAVNLAMPIFDRKNQVVGVIGMTLDFSDIATYLLDPKGQKYDGELRV 240

CJ677CC527 GRPIRMNLEGQDFDAVNIAMPIFDRKNQVVGVIGMTLDFSAIATYLLDPKSQKYDGELRV 240

CJ677CC012 GRPIRMNLEGQDFDAVNIAMPIFDRKNQVVGVIGMTLDFSAIATYLLDPKSQKYDGELRV 240

4031 GRPIKMNLEGQDFDAVNVAMPIFDRKNQVVGVIGMTLDFSAIATYLLDPKSQKYNGELRI 240

81116 GRPIKMNLEGQDFDAVNVAMPIFDRKNQVVGVIGMTLDFSAIATYLLDPKSQKYNGELRI 240

35925B2 GRPIKMNLEGQDFDAVNVAMPIFDRKNQVVGVIGMTLDFSAIATYLLDPKSQKYNGELRI 240

M1 GRPIKMNLEGQDFDAVNVAMPIFDRKNQVVGVIGMTLDFSAIATYLLDPKSQKYNGELRI 240

PT14 GRPIKMNLEGQDFDAVNVAMPIFDRKNQVVGVIGMTLDFSAIATYLLDPKSQKYNGELRI 240

81-176 GRPIKMNLEGQDFDAVNVAMPIFDRKNQVVGVIGMTLDFSDIATYLLDPKGQKYDGELRV 240

CVMN29710 GRPIKMNLEGQDFNAVNVAMPIFDRKNQVVGVIGMTLDFSDIATYLLDPKGQKYDGELRV 240

FB1 GRPIKMNLEGQDFNAVNVAMPIFDRKNQVVGVIGMTLDFSDIATYLLDPKGQKYDGELRV 240

BG2108 GRPIKMNLEGQDFNAVNVAMPIFDRKNQVVGVIGMTLDFSDIATYLLDPKGQKYDGELRV 240

YF2108 GRPIKMNLEGQDFNAVNVAMPIFDRKNQVVGVIGMTLDFSDIATYLLDPKGQKYDGELRV 240

YH501 GRPIKMNLEGQDFNAVNVAMPIFDRKNQVVGVIGMTLDFSDIATYLLDPKGQKYDGELRV 240

RM4661 GRPIKMNLEGQDFNAVNVAMPIFDRKNQVVGVIGMTLDFSDIATYLLDPKGQKYDGELRV 240

F38011 GRPIKMNLEGQDFDAVNVAMPIFDRKNQVVGVIGMTLDFSDIATYLLDPKGQKYDGELRV 240

T1-21 GRPIKMNLEGQDFDAVNVAMPIFDRKNQVVGVIGMTLDFSDIATYLLDPKGQKYDGELRV 240

CG8421 GRPIKMNLEGQDFDAVNVAMPIFDRKNQVVGVIGMTLDFSDIATYLLDPKGQKYDGELRV 240

CJM1cam GRPIKMNLEGQDFDAVNVAMPIFDRKNQVVGVIGMTLDFSDIATYLLDPKGQKYDGELRV 240

R14 GRPIKMNLEGQDFDAVNVAMPIFDRKNQVVGVIGMTLDFSDIATYLLDPKGQKYDGELRV 240

ICDCCJ07001 GRPIKMNLEGQDFDAVNVAMPIFDRKNQVVGVIGMTLDFSDIATYLLDPKGQKYDGELRV 240

RM3196 GRPIKMNLEGQDFDAVNVAMPIFDRKNQVVGVIGMTLDFSDIATYLLDPKGQKYDGELRV 240

NCTC11168 GRPIKMNLEGQDFDAVNVAIPIFDRKNQVVGVIGMTLDFSDIATYLLDPKGQKYDGELRV 240

00-2425 GRPIKMNLEGQDFDAVNVAIPIFDRKNQVVGVIGMTLDFSDIATYLLDPKGQKYDGELRV 240

IA3902 GRPIKMNLEGQDFDAVNVAIPIFDRKNQVVGVIGMTLDFSDIATYLLDPKGQKYDGELRV 240

RM1285 GRPIKMNLEGQDFDAVNVAIPIFDRKNQVVGVIGMTLDFSDIATYLLDPKGQKYDGELRV 240

00-0949 GRPIKMNLEGQDFDAVNVAIPIFDRKNQVVGVIGMTLDFSDIATYLLDPKGQKYDGELRV 240

01-1512 GRPIKMNLEGQDFDAVNVAIPIFDRKNQVVGVIGMTLDFSDIATYLLDPKGQKYDGELRV 240

FDAARGOS_422 GRPIKMNLEGQDFDAVNVAIPIFDRKNQVVGVIGMTLDFSDIATYLLDPKGQKYDGELRV 240

FORC_056 GRPIKMNLEGQDFDAVNVAMPIFDRKNQVVGVIGMTLDFSDIATYLLDPKGQKYDGELRV 240

32488 GHPIKMNLEGQDFDAVNVAMPIFDRKNQVVGVIGMTLDFSDIATYLLDPKGQKYDGELRV 240

CFSAN032806 GRPIKMNLEGQDFDAVNVAMPIFDRKNQVVGVIGMTLDFSDIATYLLDPKGQKYDGELRV 240

YH001 GRPIKMNLEGQDFDAVNVAMPIFDRKNQVVGVIGMTLDFSDIATYLLDPKGQKYDGELRV 240

00-6200 GRPIKMNLEGQDFDAVNVAMPIFDRKNQVVGVIGMTLDFSDIATYLLDPKGQKYDGELRV 240

RM1221 GRPIKMNLEGQDFDAVNVAMPIFDRKNQVVGVIGMTLDFSDIATYLLDPKGQKYDGELRV 240

S3 GRPIKMNLEGQDFDAVNVAMPIFDRKNQVVGVIGMTLDFSDIATYLLDPKGQKYDGELRV 240

FDAARGOS_421 GRPIKMNLEGQDFDAVNVAMPIFDRKNQVVGVIGMTLDFSDIATYLLDPKGQKYDGELRV 240

FJ3124 GRPIKMNLEGQDFDAVNVAMPIFDRKNQVVGVIGMTLDFSDIATYLLDPKGQKYDGELRV 240

00-1597 GRPIKMNLEGQDFDAVNVAMPIFDRKNQVVGVIGMTLDFSDIATYLLDPKGQKYDGELRV 240

*:**:****.***:***:*:***:********:******* **:******.***:****:

FDAARGOS_295 LLNSDGFVAIHPNKNLVLKNLKDVNPNKGAQETYKAMSEGKNGVFNYIASDGDDSYAAIN 300

14980A LLNSDGLMAIHPNKNLVLKNLKDVNPNKGAQETYKAMSEGKNGVFDYIASDGDDSYAAIN 300

CJ677CC527 LLNSDGFVAIHPNKNLVLKNLKDINPNKGARETYKAMSEGKNGVFNYIAFDGDDSYAAIN 300

CJ677CC012 LLNSDGFVAIHPNKNLVLKNLKDINPNKGARETYKAMSEGKNGVFNYIAFDGDDSYAAIN 300

4031 LLNSDGLVAIHPNKNLVLKNLKDVNPNKGAQETYKAMSEGKNGVFNYIAFDGDDSYAAIN 300

81116 LLNSDGLVAIHPNKNLVLKNLKDVNPNKGAQETYKAMSEGKNGVFNYIAFDGDDSYAAIN 300

35925B2 LLNSDGLVAIHPNKNLVLKNLKDVNPNKGAQETYKAMSEGKNGVFNYIAFDGDDSYAAIN 300

M1 LLNSDGLVAIHPNKNLVLKNLKDVNPNKGAQETYKAMSEGKNGVFNYIAFDGDDSYAAIN 300

PT14 LLNSDGLVAIHPNKNLVLKNLKDVNPNKGAQETYKAMSEGKNGVFNYIAFDGDDSYAAIN 300

81-176 LLNSDGLMAIHPNKNLVLKNLKDVNPNKGAQETYKAISEGKNGVFDYIASDGDDSYAAIN 300

CVMN29710 LLNSDGFMAIHPNKNLVLKNLKDVNPNKGAQETYKAISEGKNGVFDYIASDGDDSYAAIN 300

FB1 LLNSDGFMAIHPNKNLVLKNLKDVNPNKGAQETYKAISEGKNGVFDYIASDGDDSYAAIN 300

BG2108 LLNSDGFMAIHPNKNLVLKNLKDVNPNKGAQETYKAISEGKNGVFDYIASDGDDSYAAIN 300

YF2108 LLNSDGFMAIHPNKNLVLKNLKDVNPNKGAQETYKAISEGKNGVFDYIASDGDDSYAAIN 300

YH501 LLNSDGFMAIHPNKNLVLKNLKDVNPNKGAQETYKAISEGKNGVFDYIASDGDDSYAAIN 300

RM4661 LLNSDGFMAIHPNKNLVLKNLKDVNPNKGAQETYKAISEGKNGVFDYIASDGDDSYAAIN 300

F38011 LLNSDGFMAIHPNKNLVLKNLKDINPNKGAQETYKAISEGKNGVFNYIASDGDDSYAAIN 300

T1-21 LLNSDGFMAIHPNKNLVLKNLKDINPNKGAQETYKAISEGKNGVFNYIASDGDDSYAAIN 300

CG8421 LLNSDGFMAIHPNKNLVLKNLKDINPNKGAQETYKAISEGKNGVFNYIASDGDDSYAAIN 300

CJM1cam LLNSDGFMAIHPNKNLVLKNLKDINPNKGAQETYKAISEGKNGVFNYIASDGDDSYAAIN 300

R14 LLNSDGFMAIHPNKNLVLKNLKDINPNKGAQETYKAISEGKNGVFNYIASDGDDSYAAIN 300

ICDCCJ07001 LLNSDGFMAIHPNKNLVLKNLKDINPNKGAQETYKAISEGKNGVFNYIASDGDDSYAAIN 300

RM3196 LLNSDGFMAIHPNKNLVLKNLKDINPNKGAQETYKAISEGKNGVFNYIASDGDDSYAAIN 300

NCTC11168 LLNSDGFMAIHPNKNLVLKNLKDINPNKGAQETYKAISEGKNGVFNYIASDGDDSYAAIN 300

00-2425 LLNSDGFMAIHPNKNLVLKNLKDINPNKGAQETYKAISEGKNGVFNYIASDGDDSYAAIN 300

IA3902 LLNSDGFMAIHPNKNLVLKNLKDINPNKGAQETYKAISEGKNGVFNYIASDGDDSYAAIN 300

RM1285 LLNSDGFMAIHPNKNLVLKNLKDINPNKGAQETYKAISEGKNGVFNYIASDGDDSYAAIN 300

00-0949 LLNSDGFMAIHPNKNLVLKNLKDINPNKGAQETYKAISEGKNGVFNYIASDGDDSYAAIN 300

01-1512 LLNSDGFMAIHPNKNLVLKNLKDINPNKGAQETYKAISEGKNGVFNYIASDGDDSYAAIN 300

FDAARGOS_422 LLNSDGFMAIHPNKNLVLKNLKDINPNKGAQETYKAISEGKNGVFNYIASDGDDSYAAIN 300

FORC_056 LLNSDGFMAIHPNKNLVLKNLKDINPNKGAQETYKAISEGKNGVFNYIASDGDDSYAAIN 300

32488 LLNSDGFMAIHPNKNLVLKNLKDINPNKGAQETYKAISEGKNGVFNYIASDGDDSYAAIN 300

CFSAN032806 LLNSDGFMAIHPNKNLVLKNLKDINPNKGAQETYKAISEGKNGVFNYIASDGDDSYAAIN 300

YH001 LLNSDGFMAIHPNKNLVLKNLKDINPNKGAQETYKAISEGKNGVFNYIASDGDDSYAAIN 300

00-6200 LLNSDGFMAIHPNKNLVLKNLKDINPNKGAQETYKAISEGKNGVFNYIASDGDDSYAAIN 300

RM1221 LLNSDGFMAIHPNKNLVLKNLKDINPNKGAQETYKAISEGKNGVFNYIASDGDDSYAAIN 300

S3 LLNSDGFMAIHPNKNLVLKNLKDINPNKGAQETYKAISEGKNGVFNYIASDGDDSYAAIN 300

FDAARGOS_421 LLNSDGFMAIHPNKNLVLKNLKDINPNKGAQETYKAISEGKNGVFNYIASDGDDSYAAIN 300

FJ3124 LLNSDGFMAIHPNKNLVLKNLKDINPNKGAQETYKAISEGKNGVFNYIASDGDDSYAAIN 300

00-1597 LLNSDGFMAIHPNKNLVLKNLKDINPNKGAQETYKAISEGKNGVFNYIASDGDDSYAAIN 300

******::***************:******:*****:********:*** **********

FDAARGOS_295 SFKVQDSSWTVLVTAPKYSVFEPLKKLQLIIIGASFIFIFVVLGVVYYCVRKIVATRLPI 360

14980A TFKVQDSSWTVLVTAPKYSVFEPLKKLQMIIISASLIFIIVVLGVVYYCVRKIVAARLPI 360

CJ677CC527 SFKVQDSSWTVLVTAPKYSVFEPLKKLQLIIISASLIFIFVVLGVVYYCVRKIVATRLPI 360

CJ677CC012 SFKVQDSSWTVLVTAPKYSVFEPLKKLQLIIISASLIFIFVVLGVVYYCVRKIVATRLPI 360

4031 SFKVQDSSWTVLVTAPKYSVFEPLKKLQLIIIGASFIFIFVVLGVVYYCVRKIVASRLPV 360

81116 SFKVQDSSWTVLVTAPKYSVFEPLKKLQLIIIGASFIFIFVVLGVVYYCVRKIVASRLPV 360

35925B2 SFKVQDSSWTVLVTAPKYSVFEPLKKLQLIIIGASFIFIFVVLGVVYYCVRKIVASRLPV 360

M1 SFKVQDSSWTVLVTAPKYSVFEPLKKLQLIIIGASFIFIFVVLGVVYYCVRKIVASRLPV 360

PT14 SFKVQDSSWTVLVTAPKYSVFEPLKKLQLIIIGASFIFIFVVLGVVYYCVRKIVASRLPV 360

81-176 SFKVQDSSWAVLVTAPKYSVFKPLKKLQLIILGASFIFIFVVLGVVYYCVRKIVASRLPV 360

CVMN29710 SFKVQDSSWAVLVTAPKYSVFKPLKKLQLIILGASFIFIFVVLGVVYYCVRKIVASRLPV 360

FB1 SFKVQDSSWAVLVTAPKYSVFKPLKKLQLIILGASFIFIFVVLGVVYYCVRKIVASRLPV 360

BG2108 SFKVQDSSWAVLVTAPKYSVFKPLKKLQLIILGASFIFIFVVLGVVYYCVRKIVASRLPV 360

YF2108 SFKVQDSSWAVLVTAPKYSVFKPLKKLQLIILGASFIFIFVVLGVVYYCVRKIVASRLPV 360

YH501 SFKVQDSSWAVLVTAPKYSVFKPLKKLQLIILGASFIFIFVVLGVVYYCVRKIVASRLPV 360

RM4661 SFKVQDSSWAVLVTAPKYSVFKPLKKLQLIILGASFIFIFVVLGVVYYCVRKIVASRLPV 360

F38011 SFKVQDSSWAVLVTAPKYSVFKPLKKLQLIILGASFIFIFVVLGVVYYCVRKIVASRLPV 360

T1-21 SFKVQDSSWAVLVTAPKYSVFKPLKKLQLIILGASFIFIFVVLGVVYYCVRKIVASRLPV 360

CG8421 SFKVQDSSWAVLVTAPKYSVFKPLKKLQLIILGASFIFIFVVLGVVYYCVRKIVASRLPV 360

CJM1cam SFKVQDSSWAVLVTAPKYSVFKPLKKLQLIILGASFIFIFVVLGVVYYCVRKIVASRLPV 360

R14 SFKVQDSSWAVLVTAPKYSVFKPLKKLQLIILGASFIFIFVVLGVVYYCVRKIVASRLPV 360

ICDCCJ07001 SFKVQDSSWAVLVTTPKYSVFKPLKKLQLIILGASFIFIFVVLGVVYYCVRKIVASRLPV 360

RM3196 SFKVQDSSWAVLVTTPKYSVFKPLKKLQLIILGASFIFIFVVLGVVYYCVRKIVASRLPV 360

NCTC11168 SFKVQDSSWAVLVTAPKYSVFKPLKKLQLIILGASFIFIFVVLGVVYYCVRKIVASRLPV 360

00-2425 SFKVQDSSWAVLVTAPKYSVFKPLKKLQLIILGASFIFIFVVLGVVYYCVRKIVASRLPV 360

IA3902 SFKVQDSSWAVLVTAPKYSVFKPLKKLQLIILGASFIFIFVVLGVVYYCVRKIVASRLPV 360

RM1285 SFKVQDSSWAVLVTAPKYSVFKPLKKLQLIILGASFIFIFVVLGVVYYCVRKIVASRLPV 360

00-0949 SFKVQDSSWAVLVTAPKYSVFKPLKKLQLIILGASFIFIFVVLGVVYYCVRKIVASRLPV 360

01-1512 SFKVQDSSWAVLVTAPKYSVFKPLKKLQLIILGASFIFIFVVLGVVYYCVRKIVASRLPV 360

FDAARGOS_422 SFKVQDSSWAVLVTAPKYSVFKPLKKLQLIILGASFIFIFVVLGVVYYCVRKIVASRLPV 360

FORC_056 SFKVQDSSWAVLVTAPKYSVFKPLKKLQLIILGASFIFIFVVLGVVYYCVRKIVASRLPV 360

32488 SFKVQDSSWAVLVTAPKYSVFKPLKKLQLIILGASFIFIFVVLGVVYYCVRKIVASRLPV 360

CFSAN032806 SFKVQDSSWAVLVTAPKYSVFKPLKKLQLIILGASFIFIFVVLGVVYYCVRKIVASRLPV 360

YH001 SFKVQDSSWAVLVTAPKYSVFKPLKKLQLIILGASFIFIFVVLGVVYYCVRKIVASRLPV 360

00-6200 SFKVQDSSWAVLVTAPKYSVFKPLKKLQLIILGASFIFIFVVLGVVYYCVRKIVASRLPV 360

RM1221 SFKVQDSSWAVLVTAPKYSVFKPLKKLQLIILGASFIFIFVVLGVVYYCVRKIVASRLPV 360

S3 SFKVQDSSWAVLVTAPKYSVFKPLKKLQLIILGASFIFIFVVLGVVYYCVRKIVASRLPV 360

FDAARGOS_421 SFKVQDSSWAVLVTAPKYSVFKPLKKLQLIILGASFIFIFVVLGVVYYCVRKIVASRLPV 360

FJ3124 SFKVQDSSWAVLVTAPKYSVFKPLKKLQLIILGASFIFIFVVLGVVYYCVRKIVASRLPV 360

00-1597 SFKVQDSSWAVLVTAPKYSVFKPLKKLQLIILGASFIFIFVVLGVVYYCVRKIVASRLPV 360

:********:****:******:******:**:.**:***:***************:***:

FDAARGOS_295 ILNSLESFFRFLNHEKIELKLIKIRANDELGAMGRIINENIEKIQMSLEQDQNAVDESVQ 420

14980A ILNSLESFFRFLNHEKIELKPIKIRANDELGAMGNIINENIKKIQLSLEQDQSAVDESVQ 420

CJ677CC527 ILNSLESFFRFLNHEKIELKLIKIRANDELGAMGRIINENIEKIQISLEQDQNAVDESVQ 420

CJ677CC012 ILNSLESFFRFLNHEKIELKLIKIRANDELGAMGRIINENIEKIQISLEQDQNAVDESVQ 420

4031 ILSSLESFFRFLNHEKIEPKAIEIRANDELGAMGRIINENIEKIQISLEQDQNAVDESVQ 420

81116 ILSSLESFFRFLNHEKIEPKAIEIRANDELGAMGRIINENIEKIQISLEQDQNAVDESVQ 420

35925B2 ILSSLESFFRFLNHEKIEPKAIEIRANDELGAMGRIINENIEKIQISLEQDQNAVDESVQ 420

M1 ILSSLESFFRFLNHEKIEPKAIEIRANDELGAMGRIINENIEKIQISLEQDQNAVDESVQ 420

PT14 ILSSLESFFRFLNHEKIEPKAIEIRANDELGAMGRIINENIEKIQISLEQDQNAVDESVQ 420

81-176 ILSSLESFFRFLNHEKIEPKAIEIRANDELGAMGRIINENIEKIQISLEQDQNAVDESVQ 420

CVMN29710 ILSSLESFFRFLNHEKIEPKAIEIRANDELGAMGRIINENIEKIQISLEQDQNAVDESVQ 420

FB1 ILSSLESFFRFLNHEKIEPKAIEIRANDELGAMGRIINENIEKIQISLEQDQNAVDESVQ 420

BG2108 ILSSLESFFRFLNHEKIEPKAIEIRANDELGAMGRIINENIEKIQISLEQDQNAVDESVQ 420

YF2108 ILSSLESFFRFLNHEKIEPKAIEIRANDELGAMGRIINENIEKIQISLEQDQNAVDESVQ 420

YH501 ILSSLESFFRFLNHEKIEPKAIEIRANDELGAMGRIINENIEKIQISLEQDQNAVDESVQ 420

RM4661 ILSSLESFFRFLNHEKIEPKAIEIRANDELGAMGRIINENIEKIQISLEQDQNAVDESVQ 420

F38011 ILSSLESFFRFLNHEKIEPKAIEIRANDELGAMGRIINENIEKIQISLEQDQNAVDESVQ 420

T1-21 ILSSLESFFRFLNHEKIEPKAIEIRANDELGAMGRIINENIEKIQISLEQDQNAVDESVQ 420

CG8421 ILSSLESFFRFLNHEKIEPKAIEIRANDELGAMGRIINENIEKIQISLEQDQNAVDESVQ 420

CJM1cam ILSSLESFFRFLNHEKIEPKAIEIRANDELGAMGRIINENIEKIQISLEQDQNAVDESVQ 420

R14 ILSSLESFFRFLNHEKIEPKAIEIRANDELGAMGRIINENIEKIQISLEQDQNAVDESVQ 420

ICDCCJ07001 ILSSLESFFRFLNHEKIEPKAIEIRANDELGAMGRIINENIEKIQISLEQDQNAVDESVQ 420

RM3196 ILSSLESFFRFLNHEKIEPKAIEIRANDELGAMGRIINENIEKIQISLEQDQNAVDESVQ 420

NCTC11168 ILSSLESFFRFLNHEKIEPKAIEIRANDELGAMGRIINENIEKIQISLEQDQNAVDESVQ 420

00-2425 ILSSLESFFRFLNHEKIEPKAIEIRANDELGAMGRIINENIEKIQISLEQDQNAVDESVQ 420

IA3902 ILSSLESFFRFLNHEKIEPKAIEIRANDELGAMGRIINENIEKIQISLEQDQNAVDESVQ 420

RM1285 ILSSLESFFRFLNHEKIEPKAIEIRANDELGAMGRIINENIEKIQISLEQDQNAVDESVQ 420

00-0949 ILSSLESFFRFLNHEKIEPKAIEIRANDELGAMGRIINENIEKIQISLEQDQNAVDESVQ 420

01-1512 ILSSLESFFRFLNHEKIEPKAIEIRANDELGAMGRIINENIEKIQISLEQDQNAVDESVQ 420

FDAARGOS_422 ILSSLESFFRFLNHEKIEPKAIEIRANDELGAMGRIINENIEKIQISLEQDQNAVDESVQ 420

FORC_056 ILSSLESFFRFLNHEKIEPKAIEIRANDELGAMGRIINENIEKIQISLEQDQNAVDESVQ 420

32488 ILSSLESFFRFLNHEKIEPKAIEIRANDELGAMGRIINENIEKIQISLEQDQNAVDESVQ 420

CFSAN032806 ILSSLESFFRFLNHEKIEPKAIEIRANDELGAMGRIINENIEKIQISLEQDQNAVDESVQ 420

YH001 ILSSLESFFRFLNHEKIEPKAIEIRANDELGAMGRIINENIEKIQISLEQDQNAVDESVQ 420

00-6200 ILSSLESFFRFLNHEKIEPKAIEIRANDELGAMGRIINENIEKIQISLEQDQNAVDESVQ 420

RM1221 ILSSLESFFRFLNHEKIEPKAIEIRANDELGAMGRIINENIEKIQISLEQDQNAVDESVQ 420

S3 ILSSLESFFRFLNHEKIEPKAIEIRANDELGAMGRIINENIEKIQISLEQDQNAVDESVQ 420

FDAARGOS_421 ILSSLESFFRFLNHEKIEPKAIEIRANDELGAMGRIINENIEKIQISLEQDQNAVDESVQ 420

FJ3124 ILSSLESFFRFLNHEKIEPKAIEIRANDELGAMGRIINENIEKIQISLEQDQNAVDESVQ 420

00-1597 ILSSLESFFRFLNHEKIEPKAIEIRANDELGAMGRIINENIEKIQISLEQDQNAVDESVQ 420

**.*************** * *:***********.******:***:******.*******

FDAARGOS_295 TAREIEKGNLTARITKNPINPQLVELKNVLNRMLDALQSKIGSNMNEINRVFDSYKALDF 480

14980A TAKEIEKGNLTARITKNPINPQLVELKNVLNKMLDVLQNKIGSNMNEINRVFDSYKALDF 480

CJ677CC527 TAREIEKGNLTARITKNPINPQLVELKNVLNRMLDVLQSKIGSNMNEINRVFDSYKALDF 480

CJ677CC012 TAREIEKGNLTARITKNPINPQLVELKNVLNRMLDVLQSKIGSNMNEINRVFDSYKALDF 480

4031 TAREIEKGNLTARITKNPINPQLVELKNVLNRMLDVLQSKIGSNMNEINRVFDSYKALDF 480

81116 TAREIEKGNLTARITKNPINPQLVELKNVLNRMLDVLQSKIGSNMNEINRVFDSYKALDF 480

35925B2 TAREIEKGNLTARITKNPINPQLVELKNVLNRMLDVLQSKIGSNMNEINRVFDSYKALDF 480

M1 TAREIEKGNLTARITKNPINPQLVELKNVLNRMLDVLQSKIGSNMNEINRVFDSYKALDF 480

PT14 TAREIEKGNLTARITKNPINPQLVELKNVLNRMLDVLQSKIGSNMNEINRVFDSYKALDF 480

81-176 TAREIEKGNLTARITKNPINPQLVELKNVLNRMLDVLQSKIGSNMNEINRVFDSYKALDF 480

CVMN29710 TAREIEKGNLTARITKNPINPQLVELKNVLNRMLDVLQSKIGSNMNEINRVFDSYKALDF 480

FB1 TAREIEKGNLTARITKNPINPQLVELKNVLNRMLDVLQSKIGSNMNEINRVFDSYKALDF 480

BG2108 TAREIEKGNLTARITKNPINPQLVELKNVLNRMLDVLQSKIGSNMNEINRVFDSYKALDF 480

YF2108 TAREIEKGNLTARITKNPINPQLVELKNVLNRMLDVLQSKIGSNMNEINRVFDSYKALDF 480

YH501 TAREIEKGNLTARITKNPINPQLVELKNVLNRMLDVLQSKIGSNMNEINRVFDSYKALDF 480

RM4661 TAREIEKGNLTARITKNPINPQLVELKNVLNRMLDVLQSKIGSNMNEINRVFDSYKALDF 480

F38011 TAREIEKGNLTARITKNPINPQLVELKNVLNRMLDVLQSKIGSNMNEINRVFDSYKALDF 480

T1-21 TAREIEKGNLTARITKNPINPQLVELKNVLNRMLDVLQSKIGSNMNEINRVFDSYKALDF 480

CG8421 TAREIEKGNLTARITKNPINPQLVELKNVLNRMLDVLQSKIGSNMNEINRVFDSYKALDF 480

CJM1cam TAREIEKGNLTARITKNPINPQLVELKNVLNRMLDVLQSKIGSNMNEINRVFDSYKALDF 480

R14 TAREIEKGNLTARITKNPINPQLVELKNVLNRMLDVLQSKIGSNMNEINRVFDSYKALDF 480

ICDCCJ07001 TAREIEKGNLTARITKNPINPQLVELKDVLNRMLDVLQSKIGSNMNEINRVFDSYKALDF 480

RM3196 TAREIEKGNLTARITKNPINPQLVELKDVLNRMLDVLQSKIGSNMNEINRVFDSYKALDF 480

NCTC11168 TAREIEKGNLTARITKNPINPQLVELKDVLNRMLDVLQSKIGSNMNEINRVFDSYKALDF 480

00-2425 TAREIEKGNLTARITKNPINPQLVELKDVLNRMLDVLQSKIGSNMNEINRVFDSYKALDF 480

IA3902 TAREIEKGNLTARITKNPINPQLVELKDVLNRMLDVLQSKIGSNMNEINRVFDSYKALDF 480

RM1285 TAREIEKGNLTARITKNPINPQLVELKDVLNRMLDVLQSKIGSNMNEINRVFDSYKALDF 480

00-0949 TAREIEKGNLTARITKNPINPQLVELKDVLNRMLDVLQSKIGSNMNEINRVFDSYKALDF 480

01-1512 TAREIEKGNLTARITKNPINPQLVELKDVLNRMLDVLQSKIGSNMNEINRVFDSYKALDF 480

FDAARGOS_422 TAREIEKGNLTARITKNPINPQLVELKDVLNRMLDVLQSKIGSNMNEINRVFDSYKALDF 480

FORC_056 TAREIEKGNLTARITKNPINPQLVELKDVLNRMLDVLQSKIGSNMNEINRVFDSYKALDF 480

32488 TAREIEKGDLTARITKNPINPQLVELKNVLNRMLDVLQSKIGSNMNEINRVFDSYKALDF 480

CFSAN032806 TAREIEKGDLTARITKNPINPQLVELKNVLNRMLDVLQSKIGSNMNEINRVFDSYKALDF 480

YH001 TVREIEKGNLTARITKNPINPQLVELKNVLNRMLDVLQSKIGSNMNEINRVFDSYKALDF 480

00-6200 TVREIEKGNLTARITKNPINPQLVELKNVLNRMLDVLQSKIGSNMNEINRVFDSYKALDF 480

RM1221 TAREIEKGNLTARITKNPINPQLVELKNVLNRMLDVLQSKIGSNMNEINRVFDSYKALDF 480

S3 TAREIEKGNLTARITKNPINPQLVELKNVLNRMLDVLQSKIGSNMNEINRVFDSYKALDF 480

FDAARGOS_421 TAREIEKGNLTARITKNPINPQLVELKNVLNRMLDVLQSKIGSNMNEINRVFDSYKALDF 480

FJ3124 TAREIEKGNLTARITKNPINPQLVELKNVLNRMLDVLQSKIGSNMNEINRVFDSYKALDF 480

00-1597 TAREIEKGNLTARITKNPINPQLVELKNVLNRMLDVLQSKIGSNMNEINRVFDSYKALDF 480

*.:*****:******************:***:***.**.*********************

FDAARGOS_295 STEVFDAKGEVEITTNILGKEIKKMLVASSNFAKDLANQSEELKNSMQKLADGSNAQASS 540

14980A STEVFDAKGEVEITTNILGKEIKKMLVASSNFAKDLANQSEELKNSMRKLADGSNAQASS 540

CJ677CC527 STEVFDAKGEVEITTNILGKEIKKMLVASSNFAKDLANQSEELKNSMQKLADGSNAQASS 540

CJ677CC012 STEVFDAKGEVEITTNILGKEIKKMLVASSNFAKDLANQSEELKNSMQKLADGSNAQASS 540

4031 STEVLDAKGEVEITTNILGKEIKKMLVASSNFAKDLANQSEELKNSMQKLADGSNAQASS 540

81116 STEVLDAKGEVEITTNILGKEIKKMLVASSNFAKDLANQSEELKNSMQKLADGSNAQASS 540

35925B2 STEVLDAKGEVEITTNILGKEIKKMLVASSNFAKDLANQSEELKNSMQKLADGSNAQASS 540

M1 STEVLDAKGEVEITTNILGKEIKKMLVASSNFAKDLANQSEELKNSMQKLADGSNAQASS 540

PT14 STEVLDAKGEVEITTNILGKEIKKMLVASSNFAKDLANQSEELKNSMQKLADGSNAQASS 540

81-176 STEVFNAKGEVEITTNILGKEIKKMLLASSNFAKDLANQSEELKNSMQKLADGSNAQASS 540

CVMN29710 STEVFNAKGEVEITTNILGKEIKKMLVASSNFAKDLANQSEELKNSMQKLADGSNAQASS 540

FB1 STEVFNAKGEVEITTNILGKEIKKMLVASSNFAKDLANQSEELKNSMQKLADGSNAQASS 540

BG2108 STEVFNAKGEVEITTNILGKEIKKMLVASSNFAKDLANQSEELKNSMQKLADGSNAQASS 540

YF2108 STEVFNAKGEVEITTNILGKEIKKMLVASSNFAKDLANQSEELKNSMQKLADGSNAQASS 540

YH501 STEVFNAKGEVEITTNILGKEIKKMLVASSNFAKDLANQSEELKNSMQKLADGSNAQASS 540

RM4661 STEVFNAKGEVEITTNILGKEIKKMLLASSNFAKDLANQSEELKNSMQKLADGSNAQASS 540

F38011 STEVFNAKGEVEITTNILGKEIKKMLLASSNFAKDLANQSEELKNSMQKLADGSNAQASS 540

T1-21 STEVFNAKGEVEITTNILGKEIKKMLLASSNFAKDLANQSEELKNSMQKLADGSNAQASS 540

CG8421 STEVFNAKGEVEITTNILGKEIKKMLLASSNFAKDLANQSKELKNSMQKLADGSNAQASS 540

CJM1cam STEVFNAKGEVEITTNILGKEIKKMLLASSNFAKDLANQSKELKNSMQKLADGSNAQASS 540

R14 STEVFNAKGEVEITTNILGKEIKKMLLASSNFAKDLANQSKELKNSMQKLADGSNAQASS 540

ICDCCJ07001 STEVFNAKGEVEITTNILGKEIKKMLLASSNFAKDLANQSEELKNSMQKLADGSNAQASS 540

RM3196 STEVFNAKGEVEITTNILGKEIKKMLLASSNFAKDLANQSEELKNSMQKLADGSNAQASS 540

NCTC11168 STEVFNAKGEVEITTNILGKEIKKMLLASSNFAKDLANQSEELKNSMQKLADGSNAQASS 540

00-2425 STEVFNAKGEVEITTNILGKEIKKMLLASSNFAKDLANQSEELKNSMQKLADGSNAQASS 540

IA3902 STEVFNAKGEVEITTNILGKEIKKMLLASSNFAKDLANQSEELKNSMQKLADGSNAQASS 540

RM1285 STEVFNAKGEVEITTNILGKEIKKMLLASSNFAKDLANQSEELKNSMQKLADGSNAQASS 540

00-0949 STEVFNAKGEVEITTNILGKEIKKMLLASSNFAKDLANQSEELKNSMQKLADGSNAQASS 540

01-1512 STEVFNAKGEVEITTNILGKEIKKMLLASSNFAKDLANQSEELKNSMQKLADGSNAQASS 540

FDAARGOS_422 STEVFNAKGEVEITTNILGKEIKKMLLASSNFAKDLANQSEELKNSMQKLADGSNAQASS 540

FORC_056 STEVFNAKGEVEITTNILGKEIKKMLLASSNFAKDLANQSEELKNSMQKLADGSNAQASS 540

32488 STEVFNAKGEVEITTNILGKEIKKMLLASSNFAKDLANQSEELKNSMQKLADGSNAQASS 540

CFSAN032806 STEVFNAKGEVEITTNILGKEIKKMLLASSNFAKDLANQSEELKNSMQKLADGSNAQASS 540

YH001 STEVFNAKGEVEITTNILGKEIKKMLLASSNFAKDLANQSEELKNSMQKLADGSNAQASS 540

00-6200 STEVFNAKGEVEITTNILGKEIKKMLLASSNFAKDLANQSEELKNSMQKLADGSNAQASS 540

RM1221 STEVFNAKGEVEITTNILGKEIKKMLVASSNFAKDLANQSEELKNSMQKLADGSNAQASS 540

S3 STEVFNAKGEVEITTNILGKEIKKMLVASSNFAKDLANQSEELKNSMQKLADGSNAQASS 540

FDAARGOS_421 STEVFNAKGEVEITTNILGKEIKKMLVASSNFAKDLANQSEELKNSMQKLADGSNAQASS 540

FJ3124 STEVFNAKGEVEITTNILGKEIKKMLVASSNFAKDLANQSEELKNSMQKLADGSNAQASS 540

00-1597 STEVFNAKGEVEITTNILGKEIKKMLLASSNFAKDLANQSEELKNSMQKLADGSNAQASS 540

****::********************:*************:******:************

FDAARGOS_295 LEQSAAAVEEINSSMQNVSGKTVEVASQADDIKNIVNVIKDIAEQTNLLALNAAIEAARA 600

14980A LEQSAAAVEEINSSMQNVSGKTVEVASQADDIKNIVNVIKDIAEQTNLLALNAAIEAARA 600

CJ677CC527 LEQSAAAVEEINSSMQNVSGKTVEVASQADDIKNIVNVIKDIAEQTNLLALNAAIEAARA 600

CJ677CC012 LEQSAAAVEEINSSMQNVSGKTVEVASQADDIKNIVNVIKDIAEQTNLLALNAAIEAARA 600

4031 LEQSAAAVEEINSSMQNVSGKTVEVASQADDIKNIVNVIKDIAEQTNLLALNAAIEAARA 600

81116 LEQSAAAVEEINSSMQNVSGKTVEVASQADDIKNIVNVIKDIAEQTNLLALNAAIEAARA 600

35925B2 LEQSAAAVEEINSSMQNVSGKTVEVASQADDIKNIVNVIKDIAEQTNLLALNAAIEAARA 600

M1 LEQSAAAVEEINSSMQNVSGKTVEVASQADDIKNIVNVIKDIAEQTNLLALNAAIEAARA 600

PT14 LEQSAAAVEEINSSMQNVSGKTVEVASQADDIKNIVNVIKDIAEQTNLLALNAAIEAARA 600

81-176 LEQSAAAVEEINSSMQNVSGKTVEVASQADDIKNIVNVIKDIAEQTNLLALNAAIEAARA 600

CVMN29710 LEQSAAAVEEINSSMQNVSGKTVEVASQADDIKNIVNVIKDIAEQTNLLALNAAIEAARA 600

FB1 LEQSAAAVEEINSSMQNVSGKTVEVASQADDIKNIVNVIKDIAEQTNLLALNAAIEAARA 600

BG2108 LEQSAAAVEEINSSMQNVSGKTVEVASQADDIKNIVNVIKDIAEQTNLLALNAAIEAARA 600

YF2108 LEQSAAAVEEINSSMQNVSGKTVEVASQADDIKNIVNVIKDIAEQTNLLALNAAIEAARA 600

YH501 LEQSAAAVEEINSSMQNVSGKTVEVASQADDIKNIVNVIKDIAEQTNLLALNAAIEAARA 600

RM4661 LEQSAAAVEEINSSMQNVSGKTVEVASQADDIKNIVNVIKDIAEQTNLLALNAAIEAARA 600

F38011 LEQSAAAVEEINSSMQNVSGKTVEVASQADDIKNIVNVIKDIAEQTNLLALNAAIEAARA 600

T1-21 LEQSAAAVEEINSSMQNVSGKTVEVASQADDIKNIVNVIKDIAEQTNLLALNAAIEAARA 600

CG8421 LEQSAAAVEEINSSMQNVSGKTVEVASQADDIKNIVNVIKDIAEQTNLLALNAAIEAARA 600

CJM1cam LEQSAAAVEEINSSMQNVSGKTVEVASQADDIKNIVNVIKDIAEQTNLLALNAAIEAARA 600

R14 LEQSAAAVEEINSSMQNVSGKTVEVASQADDIKNIVNVIKDIAEQTNLLALNAAIEAARA 600

ICDCCJ07001 LEQSAAAVEEINSSMQNVSGKTVEVASQADDIKNIVNVIKDIAEQTNLLALNAAIEAARA 600

RM3196 LEQSAAAVEEINSSMQNVSGKTVEVASQADDIKNIVNVIKDIAEQTNLLALNAAIEAARA 600

NCTC11168 LEQSAAAVEEINSSMQNVSGKTVEVASQADDIKNIVNVIKDIAEQTNLLALNAAIEAARA 600

00-2425 LEQSAAAVEEINSSMQNVSGKTVEVASQADDIKNIVNVIKDIAEQTNLLALNAAIEAARA 600

IA3902 LEQSAAAVEEINSSMQNVSGKTVEVASQADDIKNIVNVIKDIAEQTNLLALNAAIEAARA 600

RM1285 LEQSAAAVEEINSSMQNVSGKTVEVASQADDIKNIVNVIKDIAEQTNLLALNAAIEAARA 600

00-0949 LEQSAAAVEEINSSMQNVSGKTVEVASQADDIKNIVNVIKDIAEQTNLLALNAAIEAARA 600

01-1512 LEQSAAAVEEINSSMQNVSGKTVEVASQADDIKNIVNVIKDIAEQTNLLALNAAIEAARA 600

FDAARGOS_422 LEQSAAAVEEINSSMQNVSGKTVEVASQADDIKNIVNVIKDIAEQTNLLALNAAIEAARA 600

FORC_056 LEQSAAAVEEINSSMQNVSGKTVEVASQADDIKNIVNVIKDIAEQTNLLALNAAIEAARA 600

32488 LEQSAAAVEEINSSMQNVSGKTVEVASQADDIKNIVNVIKDIAEQTNLLALNAAIEAARA 600

CFSAN032806 LEQSAAAVEEINSSMQNVSGKTVEVASQADDIKNIVNVIKDIAEQTNLLALNAAIEAARA 600

YH001 LEQSAAAVEEINSSMQNVSGKTVEVASQADDIKNIVNVIKDIAEQTNLLALNAAIEAARA 600

00-6200 LEQSAAAVEEINSSMQNVSGKTVEVASQADDIKNIVNVIKDIAEQTNLLALNAAIEAARA 600

RM1221 LEQSAAAVEEINSSMQNVSGKTVEVASQADDIKNIVNVIKDIAEQTNLLALNAAIEAARA 600

S3 LEQSAAAVEEINSSMQNVSGKTVEVASQADDIKNIVNVIKDIAEQTNLLALNAAIEAARA 600

FDAARGOS_421 LEQSAAAVEEINSSMQNVSGKTVEVASQADDIKNIVNVIKDIAEQTNLLALNAAIEAARA 600

FJ3124 LEQSAAAVEEINSSMQNVSGKTVEVASQADDIKNIVNVIKDIAEQTNLLALNAAIEAARA 600

00-1597 LEQSAAAVEEINSSMQNVSGKTVEVASQADDIKNIVNVIKDIAEQTNLLALNAAIEAARA 600

************************************************************

FDAARGOS_295 GEHGRGFAVVADEVRQLAERTGKSLSEIEANINILVQSVNEVAESVKEQTAGITQINDAI 660

14980A GEHGRGFAVVADEVRQLAERTGKSLSEIEANINILVQSVNEVAESVKEQTTGITQINDAI 660

CJ677CC527 GEHGRGFAVVADEVRQLAERTGKSLSEIEANINILVQSVNEVAESVKEQTAGITQINDAI 660

CJ677CC012 GEHGRGFAVVADEVRQLAERTGKSLSEIEANINILVQSVNEVAESVKEQTAGITQINDAI 660

4031 GEHGRGFAVVADEVRQLAERTGKSLSEIEANINILVQSVNEVAESVKEQTAGITQINDAI 660

81116 GEHGRGFAVVADEVRQLAERTGKSLSEIEANINILVQSVNEVAESVKEQTAGITQINDAI 660

35925B2 GEHGRGFAVVADEVRQLAERTGKSLSEIEANINILVQSVNEVAESVKEQTAGITQINDAI 660

M1 GEHGRGFAVVADEVRQLAERTGKSLSEIEANINILVQSVNEVAESVKEQTAGITQINDAI 660

PT14 GEHGRGFAVVADEVRQLAERTGKSLSEIEANINILVQSVNEVAESVKEQTAGITQINDAI 660

81-176 GEHGRGFAVVADEVRQLAERTGKSLSEIEANINILVQSVNEVAESVKEQTAGITQINDAI 660

CVMN29710 GEHGRGFAVVADEVRQLAERTGKSLSEIEANINILVQSVNEVAESVKEQTAGITQINDAI 660

FB1 GEHGRGFAVVADEVRQLAERTGKSLSEIEANINILVQSVNEVAESVKEQTAGITQINDAI 660

BG2108 GEHGRGFAVVADEVRQLAERTGKSLSEIEANINILVQSVNEVAESVKEQTAGITQINDAI 660

YF2108 GEHGRGFAVVADEVRQLAERTGKSLSEIEANINILVQSVNEVAESVKEQTAGITQINDAI 660

YH501 GEHGRGFAVVADEVRQLAERTGKSLSEIEANINILVQSVNEVAESVKEQTAGITQINDAI 660

RM4661 GEHGRGFAVVADEVRQLAERTGKSLSEIEANINILVQSVNEVAESVKEQTAGITQINDAI 660

F38011 GEHGRGFVVVADEVRQLAERTGKSLSEIEANINILVQSVNEVAESVKEQTAGITQINDAI 660

T1-21 GEHGRGFAVVADEVRQLAERTGKSLSEIEANINILVQSVNEVAESVKEQTAGITQINDAI 660

CG8421 GEHGRGFAVVADEVRQLAERTGKSLSEIEANINILVQSVNEVAESVKEQTAGITQINDAI 660

CJM1cam GEHGRGFAVVADEVRQLAERTGKSLSEIEANINILVQSVNEVAESVKEQTAGITQINDAI 660

R14 GEHGRGFAVVADEVRQLAERTGKSLSEIEANINILVQSVNEVAESVKEQTAGITQINDAI 660

ICDCCJ07001 GEHGRGFAVVADEVRQLAERTGKSLSEIEANINILVQSVNEVAESVKEQTAGITQINDAI 660

RM3196 GEHGRGFAVVADEVRQLAERTGKSLSEIEANINILVQSVNEVAESVKEQTAGITQINDAI 660

NCTC11168 GEHGRGFAVVADEVRQLAERTGKSLSEIEANINILVQSVNEVAESVKEQTAGITQINDAI 660

00-2425 GEHGRGFAVVADEVRQLAERTGKSLSEIEANINILVQSVNEVAESVKEQTAGITQINDAI 660

IA3902 GEHGRGFAVVADEVRQLAERTGKSLSEIEANINILVQSVNEVAESVKEQTAGITQINDAI 660

RM1285 GEHGRGFAVVADEVRQLAERTGKSLSEIEANINILVQSVNEVAESVKEQTAGITQINDAI 660

00-0949 GEHGRGFAVVADEVRQLAERTGKSLSEIEANINILVQSVNEVAESVKEQTAGITQINDAI 660

01-1512 GEHGRGFAVVADEVRQLAERTGKSLSEIEANINILVQSVNEVAESVKEQTAGITQINDAI 660

FDAARGOS_422 GEHGRGFAVVADEVRQLAERTGKSLSEIEANINILVQSVNEVAESVKEQTAGITQINDAI 660

FORC_056 GEHGRGFAVVADEVRQLAERTGKSLSEIEANINILVQSVNEVAESVKEQTAGITQINDAI 660

32488 GEHGRGFAVVADEVRQLAERTGKSLSEIEANINILVQSVNEVAESVKEQTAGITQINDAI 660

CFSAN032806 GEHGRGFAVVADEVRQLAERTGKSLSEIEANINILVQSVNEVAESVKEQTAGITQINDAI 660

YH001 GEHGRGFAVVADEVRQLAERTGKSLSEIEANINILVQSVNEVAESVKEQTAGITQINDAI 660

00-6200 GEHGRGFAVVADEVRQLAERTGKSLSEIEANINILVQSVNEVAESVKEQTAGITQINDAI 660

RM1221 GEHGRGFAVVADEVRQLAERTGKSLSEIEANINILVQSVNEVAESVKEQTAGITQINDAI 660

S3 GEHGRGFAVVADEVRQLAERTGKSLSEIEANINILVQSVNEVAESVKEQTAGITQINDAI 660

FDAARGOS_421 GEHGRGFAVVADEVRQLAERTGKSLSEIEANINILVQSVNEVAESVKEQTAGITQINDAI 660

FJ3124 GEHGRGFAVVADEVRQLAERTGKSLSEIEANINILVQSVNEVAESVKEQTAGITQINDAI 660

00-1597 GEHGRGFAVVADEVRQLAERTGKSLSEIEANINILVQSVNEVAESVKEQTAGITQINDAI 660

*******.******************************************:*********

FDAARGOS_295 AQLEMVTKENVEVANVTNNITNEVNQIAVAILEDVNKKRF 700

14980A AQLESVTKENVEVANATNSITNEVNQIAAAILEDVNKKRF 700

CJ677CC527 AQLETVTKENVEVANVTNNITNEVNQIAAAILEDVNKKRF 700

CJ677CC012 AQLETVTKENVEVANVTNNITNEVNQIAAAILEDVNKKRF 700

4031 AQLETVTKENVEVANVTNNITNEVNQIAAAILEDVDKKRF 700

81116 AQLETVTKENVEVANVTNNITNEVNQIAAAILEDVDKKRF 700

35925B2 AQLETVTKENVEVANVTNNITNEVNQIAAAILEDVDKKRF 700

M1 AQLETVTKENVEVANVTNNITNEVNQIAAAILEDVDKKRF 700

PT14 AQLETVTKENVEVANVTNNITNEVNQIAAAILEDVDKKRF 700

81-176 AQLETVTKENVEVANVTNNITNEVNQIAAAILEDVSKKRF 700

CVMN29710 AQLETVTKENVEVANVTNNITNEVNQIAAAILEDVNKKRF 700

FB1 AQLETVTKENVEVANVTNNITNEVNQIAAAILEDVNKKRF 700

BG2108 AQLETVTKENVEVANVTNNITNEVNQIAAAILEDVNKKRF 700

YF2108 AQLETVTKENVEVANVTNNITNEVNQIAAAILEDVNKKRF 700

YH501 AQLETVTKENVEVANVTNNITNEVNQIAAAILEDVNKKRF 700

RM4661 AQLETVTKENVEVANVTNNITNEVNQIAAAILEDVNKKRF 700

F38011 AQLETVTKENVEVANVTNNITNEVNQIAAAILEDVDKKRF 700

T1-21 AQLETVTKENVEVANVTNNITNEVNQIAAAILEDVDKKRF 700

CG8421 AQLETVTKENVEVANVTNNITNEVNQIAAAILEDVNKKRF 700

CJM1cam AQLETVTKENVEVANVTNNITNEVNQIAAAILEDVNKKRF 700

R14 AQLETVTKENVEVANVTNNITNEVNQIAAAILEDVNKKRF 700

ICDCCJ07001 AQLETVTKENVEVANVTNNITNEVNQIAAAILEDVNKKRF 700

RM3196 AQLETVTKENVEVANVTNNITNEVNQIAAAILEDVNKKRF 700

NCTC11168 AQLETVTKENVEVANVTNNITNEVNQIAAAILEDVNKKRF 700

00-2425 AQLETVTKENVEVANVTNNITNEVNQIAAAILEDVNKKRF 700

IA3902 AQLETVTKENVEVANVTNNITNEVNQIAAAILEDVNKKRF 700

RM1285 AQLETVTKENVEVANVTNNITNEVNQIAAAILEDVNKKRF 700

00-0949 AQLETVTKENVEVANVTNNITNEVNQIAAAILEDVNKKRF 700

01-1512 AQLETVTKENVEVANVTNNITNEVNQIAAAILEDVNKKRF 700

FDAARGOS_422 AQLETVTKENVEVANVTNNITNEVNQIAAAILEDVNKKRF 700

FORC_056 AQLETVTKENVEVANVTNNITNEVNQIAAAILEDVNKKRF 700

32488 AQLETVTKENVEVANVTNNITNEVNQIAAAILEDVNKKRF 700

CFSAN032806 AQLETVTKENVEVANVTNNITNEVNQIAAAILEDVNKKRF 700

YH001 AQLETVTKENVEVANVTNNITNEVNQIAAAILEDVNKKRF 700

00-6200 AQLETVTKENVEVANVTNNITNEVNQIAAAILEDVNKKRF 700

RM1221 AQLETVTKENVEVANVTNNITNEVNQIAAAILEDVNKKRF 700

S3 AQLETVTKENVEVANVTNNITNEVNQIAAAILEDVNKKRF 700

FDAARGOS_421 AQLETVTKENVEVANVTNNITNEVNQIAAAILEDVNKKRF 700

FJ3124 AQLETVTKENVEVANVTNNITNEVNQIAAAILEDVNKKRF 700

00-1597 AQLETVTKENVEVANVTNNITNEVNQIAAAILEDVNKKRF 700

**** **********.**.*********.******.****
