# Supplementary material for: Diversity of transducer-like proteins (Tlps) in Campylobacter
Source: PLoS One. 2019 Mar 25;14(3):e0214228. doi: 10.1371/journal.pone.0214228 (PMC6433261; doi:10.1371/journal.pone.0214228)
Supplement: S2 Archive — (ZIP) [file pone.0214228.s016.zip › Alignment N.docx]

Alignment N. Tlp2 protein sequence comparisons: individual isolate comparisons

CLUSTAL O(1.2.4) multiple sequence alignment 2018/04/17

81116 MKSVKLKVALIANLIAVVCLVILGVITFMFVKQAIFHEVVKAETNYVKTAKNSMESFKAR 60

RM1285 MKSVKLKVSLIANLIAVVCLIILGVVTFIFVKQAIFHEVVNAEINYVKTAKNSIESFKAR 60

CFSAN032806 MKSVKLKVSLIANLIAVVCLIILGVVTFIFVKQAIFHEVVNAEINYVKTAKNSIESFKAR 60

RM1221 MKSVKLKVSLIANLIAVVCLIILGVVTFIFVKQAIFHEVVNAEINYVKTAKNSIESFKAR 60

S3 MKSVKLKVSLIANLIAVVCLIILGVVTFIFVKQAIFHEVVNAEINYVKTAKNSIESFKAR 60

FDAARGOS_422 MKSVKLKVSLIANLIAVVCLIILGVVTFMFVKQAIFHEVVKAETNYVKTAKNSMESFKAR 60

81-176 MKSVKLKVSLIANLIAVVCLIILGVVTFIFVKQAIFHEVVNAEINYVKTAKNSIESFKAR 60

F38011 MKSVKLKVSLIANLIAVVCLIILGVVTFIFVKQAIFHEVVNAEINYVKTAKNSIESFKAR 60

NCTC11168 MKSVKLKVSLIANLIAVVCLIILGVVTFIFVKQAIFHEVVNAEINYVKTAKNSIESFKAR 60

MTVDSCj07 MKSVKLKVSLIANLIAVVCLIILGVVTFIFVKQAIFHEVVNAEINYVKTAKNSIESFKAR 60

********:***********:****:**:***********:** *********:******

81116 NSLALESLAKSILKHPVEQLDSQDALMRYVGKDLKNFRDAGRFLAVYIAQPNGELVVSDP 120

RM1285 NSLALESLAKSILKHPIEQLDSQDALMHYVGKDLKNFRDAGRFLAVYIAQPNGELVVSDP 120

CFSAN032806 NSLALESLAKSILKHPIEQLDSQDALMHYVGKDLKNFRDAGRFLAVYIAQPNGELVVSDP 120

RM1221 NSLALESLAKSILKHPIEQLDSQDALMHYVGKDLKNFRDAGRFLAVYIAQPNGELVVSDP 120

S3 NSLALESLAKSILKHPIEQLDSQDALMHYVGKDLKNFRDAGRFLAVYIAQPNGELVVSDP 120

FDAARGOS_422 NSLALESLAKSILKHPVEQLDNQDALMHYVGKDLKNFRDAGRFLAVYIAQPNGELVVSDP 120

81-176 NSLALESLAKSILKHPIEQLDSQDALMHYVGKDLKNFRDAGRFLAVYIAQPNGELVVSDP 120

F38011 NSLALESLAKSILKHPIEQLDSQDALMHYVGKDLKNFRDAGRFLAVYIAQPNGELVVSDP 120

NCTC11168 NSLALESLAKSILKHPIEQLDSQDALMHYVGKDLKNFRDAGRFLAVYIAQPNGELVVSDP 120

MTVDSCj07 NSLALESLAKSILKHPIEQLDSQDALMHYVGKDLKNFRDAGRFLAVYIAQPNGELVVSDP 120

****************:****.*****:********************************

81116 DSDAKKVDFGTYGKADNYDARTREYYIEAVKTNKLYVTPSYIDATTNLPCFTYSTPLYKD 180

RM1285 DSDAKNLDFGTYGKADNYDARTREYYIEAVKTNKLYITPSYIDVTTNLPCFTYSIPLYKD 180

CFSAN032806 DSDAKNLDFGTYGKADNYDARTREYYIEAVKTNKLYITPSYIDVTTNLPCFTYSIPLYKD 180

RM1221 DSDAKNLDFGTYGKADNYNARTREYYIEAVKTNKLYITPSYIDVTTNLPCFTYSIPLYKD 180

S3 DSDAKNLDFGTYGKADNYDARTREYYIEAVKTNKLYITPSYIDVTTNLPCFTYSIPLYKD 180

FDAARGOS_422 DSDAKNLDFGTYGKADNYDARTREYYIEAVKTNKLYITPSYIDVTTNLPCFTYSIPLYKD 180

81-176 DSDAKNLDFGTYGKADNYDARTREYYIEAVKTNKLYITPSYIDVTTNLPCFTYSIPLYKD 180

F38011 DSDAKNLDFGTYGKADNYDARTREYYIEAVKTNKLYITPSYIDVTTNLPCFTYSIPLYKD 180

NCTC11168 DSDAKNLDFGTYGKADNYDARTREYYIEAVKTNKLYITPSYIDVTTNLPCFTYSIPLYKD 180

MTVDSCj07 DSDAKILDFGTYGKADNYDARTREYYIEAVKTNKLYITPSYIDVTTNLPCFTYSIPLYKD 180

***** :***********:*****************:******.********** *****

81116 GKFIGVLAVDVLVTDLQAEFENLPGRTFVFDEENKVFASTDKTLLQQGYDISAIANLAKI 240

RM1285 GKFIGVLAVDILAADLQAEFENLPGRTFVFDEENKVFVSTDKALLQKGYDISAIANLAKT 240

CFSAN032806 GKFIGVLAVDILAADLQAEFENLPGRTFVFDEENKVFVSTDKTLLQQGYDISTIANLAKT 240

RM1221 GKFIGVLAVDILAADLQAEFENLPGRTFVFDEENKVFVSTDKTLLQQGYDISTIANLAKT 240

S3 GKFIGVLAVDILAADLQAEFENLPGRTFVFDEENKVFVSTDKTLLQQGYDISTIANLAKT 240

FDAARGOS_422 GKFIGVLAVDILAADLQAEFENLPGRTFVFDEENKVFVSTDKALLQKGYDISAIANLAKT 240

81-176 GKFIGVLAVDILAADLQAEFENLPGRTFVFDEENKVFVSTDKALLQKGYDISAIANLAKT 240

F38011 GKFIGVLAVDILAADLQAEFENLPGRTFVFDEENKVFVSTDKALLQKGYDISAIANLAKT 240

NCTC11168 GKFIGVLAVDILAADLQAEFENLPGRTFVFDEENKVFVSTDKALLQKGYDISAIANLAKT 240

MTVDSCj07 GKFIGVLAVDILAADLQAEFENLPGRTFVFDEENKVFVSTDKALLQKGYDISAIANLAKT 240

**********:*.:***********************.****:***:*****:******

81116 KENFEPFEYTRPKDGSERFAVCTKVSGVYTACVGEPIEQIEAPVYKIAFIQTAIVIFTSI 300

RM1285 KEDLEPFEYTRPKDGNERFAVCTKVSGIYTACVGEPIEQIEAPVYKIAFIQTAIVIFTSI 300

CFSAN032806 KEDLEPFEYTRPKDGNERFAVCTKVSGIYTACVGEPIEQIEAPVYKIAFIQTVIVVFASI 300

RM1221 KEDLEPFEYTRPKDGNERFAVCTKVSGIYTACVGEPIEQIEAPVYKIAFIQTVIVVFASI 300

S3 KEDLEPFEYTRPKDGNERFAVCTKVSGIYTACVGEPIEQIEAPVYKIAFIQTVIVVFASI 300

FDAARGOS_422 KEDLEPFEYTRPKDGNERFAVCTKVSGIYTACVGEPIEQIEAPVYKIAFIQTAIVIFTSI 300

81-176 KEDLEPFEYTRPKDGNERFAVCTKVSGIYTACVGEPIEQIEAPVYKIAFIQTAIVIFTSI 300

F38011 KEDLEPFEYTRPKDGNERFAVCTKVSGIYTACVGEPIEQIEAPVYKIAFIQTAIVIFTSI 300

NCTC11168 KEDLEPFEYTRPKDGNERFAVCTKVSGIYTACVGEPIEQIEAPVYKIAFIQTAIVIFTSI 300

MTVDSCj07 KEDLEPFEYTRPKDGNERFAVCTKVSGIYTACVGEPIEQIEAPVYKIAFIQTAIVIFTSI 300

**::***********.***********:************************.**:*:**

81116 ISVILLYFIVSKYLSPLAAIQTGLTSFFDFINHKTKNVSTIEVKSNDEFGQISNAINENI 360

RM1285 ISVILLYFIVSKYLSPLAAIQTGLTSFFDFINYKTKNVSTIEVKSNDEFGQISNAINKTF 360

CFSAN032806 LSVILLYFIVSKYLSPLAAIQTGLTSFFDFINYKTKNVSTIEVKSNDEFGQISNAINENI 360

RM1221 LSVILLYFIVSKYLSPLAAIQTGLTSFFDFINYKTKNVSTIEVKSNDEFGQISNAINENI 360

S3 LSVILLYFIVSKYLSPLAAIQTGLTSFFDFINYKTKNVSTIEVKSNDEFGQISNAINENI 360

FDAARGOS_422 ISVILLYFIVSKYLSPLAAIQTGLTSFFDFINYKTKNVSTIEVKSNDEFGQISNAINENI 360

81-176 ISVILLYFIVSKYLSPLAAIQTGLTSFFDFINYKTKNVSTIEVKSNDEFGQISNAINENI 360

F38011 ISVILLYFIVSKYLSPLAAIQTGLTSFFDFINYKTKNVSTIEVKSNDEFGQISNAINENI 360

NCTC11168 ISVILLYFIVSKYLSPLAAIQTGLTSFFDFINYKTKNVSTIEVKSNDEFGQISNAINENI 360

MTVDSCj07 ISVILLYFIVSKYLSPLAAIQTGLTSFFDFINYKTKNVSTIEVKSNDEFGQISNAINENI 360

:*******************************:************************:.:

81116 LATKRGLEQDNQAVKESVETVSVVESGNLTARITANPRNPQLIELKNVLNKLLDVLQARV 420

RM1285 LLLK-EAEQDNQAVKESVQTVSVVEGGNLTARITANPRNPQLIELKNVLNKLLDVLQARV 419

CFSAN032806 LATKRGLEQDNQAVKESVQTVSVVEGGNLTARITANPRNPQLIELKNVLNKLLDVLQARV 420

RM1221 LATKRGLEQDNQAVKESVQTVSVVEGGNLTARITANPRNPQLIELKNVLNKLLDVLQARV 420

S3 LATKRGLEQDNQAVKESVQTVSVVEGGNLTARITANPRNPQLIELKNVLNKLLDVLQARV 420

FDAARGOS_422 LATKRGLEQDNQAVKESVQTVSVVEGGNLTARITANPRNPQLIELKNVLNKLLDVLQARV 420

81-176 LATKRGLEQDNQAVKESVQTVSVVEGGNLTARITANPRNPQLIELKNVLNKLLDVLQARV 420

F38011 LATKRGLEQDNQAVKESVQTVSVVEGGNLTARITANPRNPQLIELKNVLNKLLDVLQARV 420

NCTC11168 LATKRGLEQDNQAVKESVQTVSVVEGGNLTARITANPRNPQLIELKNVLNKLLDVLQARV 420

MTVDSCj07 LATKRGLEQDNQAVKESVQTVSVVEGGNLTARITANPRNPQLIELKNVLNKLLDVLQARV 420

* * ***********:******.**********************************

81116 GSDMNAIHKIFEEYKSLDFRNKLENASGSVELTTNALGDEIVKMLKQSSDFANALANESG 480

RM1285 GSDMNAIHKIFEEYKSLDFRNKLENASGSVELTTNALGDEIVKMLKQSSDFANALANESG 479

CFSAN032806 GSDMNAIHKIFEEYKSLDFRNKLENASGSVELTTNALGDEIVKMLKQSSDFANALANESG 480

RM1221 GSDMNAIHKIFEEYKSLDFRNKLENASGSVELTTNALGDEIVKMLKQSSDFANALANESG 480

S3 GSDMNAIHKIFEEYKSLDFRNKLENASGSVELTTNALGDEIVKMLKQSSDFANALANESG 480

FDAARGOS_422 GSDMNAIHKIFEEYKSLDFRNKLENASGSVELTTNALGDEIVKMLKQSSDFANALANESG 480

81-176 GSDMNAIHKIFEEYKSLDFRNKLENASGSVELTTNALGDEIVKMLKQSSDFANALANESG 480

F38011 GSDMNAIHKIFEEYKSLDFRNKLENASGSVELTTNALGDEIVKMLKQSSDFANALANESG 480

NCTC11168 GSDMNAIHKIFEEYKSLDFRNKLENASGSVELTTNALGDEIVKMLKQSSDFANALANESG 480

MTVDSCj07 GSDMNAIHKIFEEYKSLDFRNKLENASGSVELTTNALGDEIVKMLKQSSDFANALANESG 480

************************************************************

81116 KLQTAVQSLTTSSNSQAQSLEETAAALEEITSSMQNVSVKTSDVITQSEEIKNVTGIIGD 540

RM1285 KLQTAVQSLTTSSNSQAQSLEETAAALEEITSSMQNVSVKTSDVITQSEEIKNVTGIIGD 539

CFSAN032806 KLQTAVQSLTTSSNSQAQSLEETAAALEEITSSMQNVSVKTSDVITQSEEIKNVTGIIGD 540

RM1221 KLQTAVQSLTTSSNSQAQSLEETAAALEEITSSMQNVSVKTSDVITQSEEIKNVTGIIGD 540

S3 KLQTAVQSLTTSSNSQAQSLEETAAALEEITSSMQNVSVKTSDVITQSEEIKNVTGIIGD 540

FDAARGOS_422 KLQTAVQSLTTSSNSQAQSLEETAAALEEITSSMQNVSVKTSDVITQSEEIKNVTGIIGD 540

81-176 KLQTAVQSLTTSSNSQAQSLEETAAALEEITSSMQNVSVKTSDVITQSEEIKNVTGIIGD 540

F38011 KLQTAVQSLTTSSNSQAQSLEETAAALEEITSSMQNVSVKTSDVITQSEEIKNVTGIIGD 540

NCTC11168 KLQTAVQSLTTSSNSQAQSLEETAAALEEITSSMQNVSVKTSDVITQSEEIKNVTGIIGD 540

MTVDSCj07 KLQTAVQSLTTSSNSQAQSLEETAAALEEITSSMQNVSVKTSDVITQSEEIKNVTGIIGD 540

************************************************************

81116 IADQINLLALNAAIEAARAGEHGRGFAVVADEVRKLAERTQKSLSEIEANTNLLVQSIND 600

RM1285 IADQINLLALNAAIEAARAGEHGRGFAVVADEVRKLAERTQKSLSEIEANTNLLVQSIND 599

CFSAN032806 IADQINLLALNAAIEAARAGEHGRGFAVVADEVRKLAERTQKSLSEIEANTNLLVQSIND 600

RM1221 IADQINLLALNAAIEAARAGEHGRGFAVVADEVRKLAERTQKSLSEIEANTNLLVQSIND 600

S3 IADQINLLALNAAIEAARAGEHGRGFAVVADEVRKLAERTQKSLSEIEANTNLLVQSIND 600

FDAARGOS_422 IADQINLLALNAAIEAARAGEHGRGFAVVADEVRKLAERTQKSLSEIEANTNLLVQSIND 600

81-176 IADQINLLALNAAIEAARAGEHGRGFAVVADEVRKLAERTQKSLSEIEANTNLLVQSIND 600

F38011 IADQINLLALNAAIEAARAGEHGRGFAVVADEVRKLAERTQKSLSEIEANTNLLVQSIND 600

NCTC11168 IADQINLLALNAAIEAARAGEHGRGFAVVADEVRKLAERTQKSLSEIEANTNLLVQSIND 600

MTVDSCj07 IADQINLLALNAAIEAARAGEHGRGFAVVADEVRKLAERTQKSLSEIEANTNLLVQSIND 600

************************************************************

81116 MAESIKEQTAGITQINESVAQIDQTTKDNVEIANESAIISSTVSDIANNILEDVKKKRF 659

RM1285 MAESIKEQTAGITQINDSVAQIDQTTKDNVEIANESAIISSTVSDIANNILEDVKKKRF 658

CFSAN032806 MAESIKEQTAGITQINDSVAQIDQTTKDNVEIANESAIISNTVSDIANNILEDVKKKRF 659

RM1221 MAESIKEQTAGITQINDSVAQIDQTTKDNVEIANESAIISSTVSDIANNILEDVKKKRF 659

S3 MAESIKEQTAGITQINDSVAQIDQTTKDNVEIANESAIISSTVSDIANNILEDVKKKRF 659

FDAARGOS_422 MAESIKEQTAGITQINDSVAQIDQTTKDNVEIANESAIISSTVSDIANNILEDVKKKRF 659

81-176 MAESIKEQTAGITQINDSVAQIDQTTKDNVEIANESAIISSTVSDIANNILEDVKKKRF 659

F38011 MAESIKEQTAGITQINDSVAQIDQTTKDNVEIANESAIISSTVSDIANNILEDVKKKRF 659

NCTC11168 MAESIKEQTAGITQINDSVAQIDQTTKDNVEIANESAIISSTVSDIANNILEDVKKKRF 659

MTVDSCj07 MAESIKEQTAGITQINDSVAQIDQTTKDNVEIANESAIISSTVSDIANNILEDVKKKRF 659

****************:***********************.******************
