# Supplementary material for: Diversity of transducer-like proteins (Tlps) in Campylobacter
Source: PLoS One. 2019 Mar 25;14(3):e0214228. doi: 10.1371/journal.pone.0214228 (PMC6433261; doi:10.1371/journal.pone.0214228)
Supplement: S2 Archive — (ZIP) [file pone.0214228.s016.zip › Alignment O.docx]

Alignment O. Tlp3 protein sequence comparisons: individual isolate comparisons

CLUSTAL O(1.2.4) multiple sequence alignment 2018/04/17

HC2-48_Tlp3 -----------MNSIKIKLSLIANLIAIFALIVLGIVSFYFTKTSLYESTLKNQTDLLKV 49

RM1875_Tlp3 -----------MNSIKIKLSLIANLIAIFALIVLGIVSFYFTKTSLYESTLKNQTDLLKV 49

CF2-75_Tlp3 MLKITKIKRKIMNSIKIKLSLIANLIAIFALIVLGIVSFYFTKTSLYESTLKNQTDLLKV 60

RM5611_Tlp3 -----------MNSIKIKLSLIANLIAIFALIVLGIVSFYFTKTSLYESTLKNQTDLLKV 49

MTVDSCj16_Tlp3 -----------MNSIKIKLSLIANLIAIFALIVLGIVSFYFTKTSLYESTLKNQTDLLKV 49

01-1512_Tlp3 -----------MNSIKIKLSLIANLIAIFALIVLGIVSFYFTKTSLYESTLKNQTDLLKV 49

MTVDSCj13_Tlp3 -----------MNSIKIKLSLIANLIAIFALIVLGIVSFYFTKTSLYESTLKNQTDLLKV 49

32488_Tlp3a -----------MNSIKIKLSLIANLIAIFALIVLGIVSFYFTKTSLYESTLKNQTDLLKV 49

81116_Tlp3 -----------MNSIKIKLSLIANLIAIFALIVLGIVSFYFTKTSLYESTLKNQTDLLKV 49

32488_Tlp3b -----------MNNIKIKLSVIANSIAIFALIVLGIVSFYFTKTSLYESTLKNQTDLLKV 49

FB1_Tlp3 -----------MNSIKIKLSLIANLIAIFALIVLGIVSFYFTKTSLYESTLKNQTDLLKV 49

PT14_Tlp3 -----------MNSIKIKLSLIANLIAIFALIVLGIVSFYFTKTSLYESTLKNQTDLLKV 49

00-6200_Tlp3a -----------MNSIKIKLSLIANLIAIFALIVLGIVSFYFTKTSLYESTLKNQTDLLKV 49

RM1221_Tlp3 -----------MNSIKIKLSLIANLIAIFALIVLGIVSFYFTKTSLYESTLKNQTDLLKV 49

S3_Tlp3 -----------MNSIKIKLSLIANLIAIFALIVLGIVSFYFTKTSLYESTLKNQTDLLKV 49

FDAARGOS_421_Tlp3 MLKITKIKRKIMNSIKIKLSLIANLIAIFALIVLGIVSFYFTKTSLYESTLKNQTDLLKV 60

CFSAN032806_Tlp3 MLKITKIKRKIMNSIKIKLSLIANLIAIFALIVLGIVSFYFTKTSLYESTLKNQTDLLKV 60

IA3901_Tlp3b -----------MNSIKIKLSLIANLIAIFALIVLGIVSFYFTKTSLYESTLKNQTDLLKV 49

00-6200_Tlp3b -----------MNSIKIKLSLIANLIAIFALIVLGIVSFYFTKTSLYESTLKNQTDLLKV 49

BCW_6290_Tlp3b -----------MNSIKIKLSLIANLIAIFALIVLGIVSFYFTKTSLYESTLKNQTDLLKV 49

00-2425_Tlp3a -----------MNSIKIKLSLIANLIAIFALIVLGIVSFYFTKTSLYESTLKNQTDLLKV 49

00-2425_Tlp3b -----------MNSIKIKLSLIANLIAIFALIVLGIVSFYFTKTSLYESTLKNQTDLLKV 49

YH001_Tlp3a -----------MNSIKIKLSLIANLIAIFALIVLGIVSFYFTKTSLYESTLKNQTDLLKV 49

YH001_Tlp3b -----------MNSIKIKLSLIANLIAIFALIVLGIVSFYFTKTSLYESTLKNQTDLLKV 49

00-0949_Tlp3 -----------MNSIKIKLSLIANLIAIFALIVLGIVSFYFTKTSLYESTLKNQTDLLKV 49

NCTC11168_Tlp3 MLKITKIKRKIMNSIKIKLSLIANLIAIFALIVLGIVSFYFTKTSLYESTLKNQTDLLKV 60

F38011_Tlp3 -----------MNSIKIKLSLIANLIAIFALIVLGIVSFYFTKTSLYESTLKNQTDLLKV 49

RM1285_Tlp3 -----------MNSIKIKLSLIANLIAIFALIVLGIVSFYFTKTSLYESTLKNQTDLLKV 49

FDAARGOS_422_Tlp3 MLKITKIKRKIMNSIKIKLSLIANLIAIFALIVLGIVSFYFTKTSLYESTLKNQTDLLKV 60

MTVDSCj07_Tlp3 -----------MNSIKIKLSLIANLIAIFALIVLGIVSFYFTKTSLYESTLKNQTDLLKV 49

IA3901_Tlp3a -----------MNSIKIKLSLIANLIAIFALIVLGIVSFYFTKTSLYESTLKNQTDLLKV 49

BCW_6290_Tlp3a -----------MNSIKIKLSLIANLIAIFALIVLGIVSFYFTKTSLYESTLKNQTDLLKV 49

CJ677CC012_Tlp3 -----------MNSIKIKLSLIANLIAIFALIVLGIVSFYFTKTSLHESALKNQTDLLKV 49

CJM1cam -----------MNSIKIKLSLIANLIAIFALIVLGIVSFYFTKTSLHESALKNQTDLLKV 49

M1_Tlp3 -----------MNSIKIKLSLIANLIAIFALIVLGIVSFYFTKTSLHESALKNQTDLLKV 49

4031_Tlp3 -----------MNSIKIKLSLIANLIAIFALIVLGIVSFYFTKTSLHESALKNQTDLLKV 49

R14_Tlp3 -----------MNSIKIKLSLIANLIAIFALIVLGIVSFYFTKTSLYESTLKNQTDLLKV 49

35925B2_Tlp3 MLKITKIKRKIMNSIKIKLSLIANLIAIFALIVLGIVSFYFTKTSLHESALKNQTDLLKV 60

14980A MLKITKIKRKIMNSIKIKLSLIANLIAIFALIVLGIVSFYFTKTSLYESTLKNQTDLLKV 60

00-1597_Tlp3b -----------MNSIKIKLSLIANLIAIFALIVLGIVSFYFTKTSLYESTLKNQTDLLKV 49

ICDCCJ07001_Tlp3 -----------MNSIKIKLSLIANLIAIFALIVLGIVSFYFTKTSLYESTLKNQTDLLKV 49

RM3196_Tlp3 -----------MNSIKIKLSLIANLIAIFALIVLGIVSFYFTKTSLYESTLKNQTDLLKV 49

**.******:*** *********************:**:**********

HC2-48_Tlp3 TQSTVEDFRSTNQSFTRALEKDIANLPYQSLITEENIINNVGPILKYYHHSINALNVYLG 109

RM1875_Tlp3 TQSTVEDFRSTNQSFIRALEKDIANLPYQSLITEENIINNVGPILKYYRHSINALNVYLG 109

CF2-75_Tlp3 TQSTVEDFRSTNQSFTRALEKDIANLPYQSLITEENIINNVGPILKYYHHSINALNVYLG 120

RM5611_Tlp3 TQSTVEDFRSTNQSFTRALEKDIANLPYQSLITEENIINNVGPILKYYHHSINALNVYLG 109

MTVDSCj16_Tlp3 TQSTVEDFRSTNQSFTRALEKDIANLPYQSLITEENIINNVGPILKYYRHSINALNVYLG 109

01-1512_Tlp3 TQSTVEDFRSTNQSFTRALEKDIANLPYQSLITEENIINNVGPILKYYRHSINALNVYLG 109

MTVDSCj13_Tlp3 TQSTVEDFRSTNQSFTRALEKDIANLPYQSLITEENIINNVGPILKYYRHSINALNVYLG 109

32488_Tlp3a TQSTVEDFRSTNQSFTRALEKDIANLPYQSLITEENIINNVGPILKYYRHSINALNVYLG 109

81116_Tlp3 TQSTVEDFRSTNQSFTRALEKDIANLPYQSLITEENIINNVGPILKYYRHSINALNVYLG 109

32488_Tlp3b TQSTVEDFRSTNQSFTRALEKDIANLPYQSLITEENIINNVGPILKYYRHSINALNVYLG 109

FB1_Tlp3 TQSTVEDFRSTNQSFTRALEKDIANLPYQSLITEENIINNVGPILKYYRHSINALNVYLG 109

PT14_Tlp3 TQSTVEDFRSTNQSFTRALEKDIANLPYQSLITEENIINNVGPILKYYRHSINALNVYLG 109

00-6200_Tlp3a TQSTVEDFRSTNQSFTRALEKDIANLPYQSLITEENIINNVGPILKYYHHSINALNVYLG 109

RM1221_Tlp3 TQSTVEDFRSTNQSFTRALEKDIANLPYQSLITEENIINNVGPILKYYRHSINALNVYLG 109

S3_Tlp3 TQSTVEDFRSTNQSFTRALEKDIANLPYQSLITEENIINNVGPILKYYRHSINALNVYLG 109

FDAARGOS_421_Tlp3 TQSTVEDFRSTNQSFTRALEKDIANLPYQSLITEENIINNVGPILKYYRHSINALNVYLG 120

CFSAN032806_Tlp3 TQSTVEDFRSTNQSFTRALEKDIANLPYQSLITEENIINNVGPILKYYRHSINALNVYLG 120

IA3901_Tlp3b TQSTVEDFRSTNQSFTRALEKDIANLPYQSLITEENIINNVGPILKYYHHSINALNVYLG 109

00-6200_Tlp3b TQSTVEDFRSTNQSFTRALEKDIANLPYQSLITEENIINNVGPILKYYHHSINALNVYLG 109

BCW_6290_Tlp3b TQSTVEDFRSTNQSFTRALEKDIANLPYQSLITEENIINNVGPILKYYHHSINALNVYLG 109

00-2425_Tlp3a TQSTVEDFRSTNQSFTRALEKDIANLPYQSLITEENIINNVGPILKYYHHSINALNVYLG 109

00-2425_Tlp3b TQSTVEDFRSTNQSFTRALEKDIANLPYQSLITEENIINNVGPILKYYHHSINALNVYLG 109

YH001_Tlp3a TQSTVEDFRSTNQSFTRALEKDIANLPYQSLITEENIINNVGPILKYYHHSINALNVYLG 109

YH001_Tlp3b TQSTVEDFRSTNQSFTRALEKDIANLPYQSLITEENIINNVGPILKYYHHSINALNVYLG 109

00-0949_Tlp3 TQSTVEDFRSTNQSFTRALEKDIANLPYQSLITEENIINNVGPILKYYRHSINALNVYLG 109

NCTC11168_Tlp3 TQSTVEDFRSTNQSFTRALEKDIANLPYQSLITEENIINNVGPILKYYRHSINALNVYLG 120

F38011_Tlp3 TQSTVEDFRSTNQSFTRALEKDIANLPYQSLITEENIINNVGPILKYYRHSINALNVYLG 109

RM1285_Tlp3 TQSTVEDFRSTNQSFTRALEKDIANLPYQSLITEENIINNVGPILKYYRHSINALNVYLG 109

FDAARGOS_422_Tlp3 TQSTVEDFRSTNQSFTRALEKDIANLPYQSLITEENIINNVGPILKYYRHSINALNVYLG 120

MTVDSCj07_Tlp3 TQSTVEDFRSTNQSFTRALEKDIANLPYQSLITEENIINNVGPILKYYRHSINALNVYLG 109

IA3901_Tlp3a TQSTVEDFRSTNQSFTRALEKDIANLPYQSLITEENIINNVGPILKYYHHSINALNVYLG 109

BCW_6290_Tlp3a TQSTVEDFRSTNQSFTRALEKDIANLPYQSLITEENIINNVGPILKYYHHSINALNVYLG 109

CJ677CC012_Tlp3 TQSTVEDFRSTNQSFTRALEKDIANLPYQSLITEENIINNVGPILKYYRHSINALNVYLG 109

CJM1cam TQSTVEDFRSTNQSFTRALEKDITNLPYQSLITEENIINNVGPILKYYRHSINALNVYLG 109

M1_Tlp3 TQSTVEDFRSTNQSFTRALEKDITNLPYQSLITEENIINNVGPILKYYRHSINALNVYLG 109

4031_Tlp3 TQSTVEDFRSTNQSFTRALEKDITNLPYQSLITEENIINNVGPILKYYRHSINALNVYLG 109

R14_Tlp3 TQSTVEDFRSTNQSFTRALEKDIANLPYQSLITEENIINNVGPILKYYRHSINALNVYLG 109

35925B2_Tlp3 TQSTVEDFRSTNQSFTRALEKDITNLPYQSLITEENIINNVGPILKYYRHSINALNVYLG 120

14980A TQSTVEDFRSTNQSFTRALEKDIANLPYQSLITEENIINNVGPILEYYRHSINALNVYLG 120

00-1597_Tlp3b TQSTVEDFRSTNQSFTRALEKDIANLPYQSLITEENIINNVGPILKYYRHSINALNVYLG 109

ICDCCJ07001_Tlp3 TQSTVEDFRSTNQSFTRALEKDIANLPYQSLITEENIINNVGPILKYYRHSINALNVYLG 109

RM3196_Tlp3 TQSTVEDFRSTNQSFTRALEKDIANLPYQSLITEENIINNVGPILKYYRHSINALNVYLG 109

*************** *******:*********************:**:***********

HC2-48_Tlp3 LNNGKVLLSQKSNDAKMPELRDDLDIKTKDWYQEALKTNDIFVTPAYLDTVLKQYVITYS 169

RM1875_Tlp3 LNNGKVLLSQKSNDAKMPELRDDLDIKTKDWYQEALKTNDIFDTPAYLDTNLKQYVITYS 169

CF2-75_Tlp3 LNNGKVLLSQKSNDAKMPELRDDLDIKTKDWYQEALKTNDIFVTPAYLDTVLKQYVITYS 180

RM5611_Tlp3 LNNGKVLLSQKSNDAKMPELRDDLDIKTKDWYQEALKTNDIFVTPAYLDTVLKQYVITYS 169

MTVDSCj16_Tlp3 LNNGKVLLSQKSNDAKMPELRDDLDIKTKDWYQEALKTNDIFVTPAYLDTILKQYVITYS 169

01-1512_Tlp3 LNNGKVLLSQKSNDAKMPELRDDLDIKTKDWYQEALKTNDIFVTPAYLDTVLKQYVITYS 169

MTVDSCj13_Tlp3 LNNGKVLLSQKSNDAKMPELRDDLDIKTKDWYQEALKTNDIFVTPAYLDTILKQYVITYS 169

32488_Tlp3a LNNGKVLLSQKSNDAKMPELRDDLDIKTKDWYQEALKTNDIFVTPAYLDTILKQYVITYS 169

81116_Tlp3 LNNGKVLLSQKSNDAKMPELRDDLDIKTKDWYQEALKTNDIFVTPAYLDTILKQYVITYS 169

32488_Tlp3b LNNGKVLLSQKSNDAKMPELRDDLDIKTKDWYQEALKTNDIFVTPAYLDTILKQYVITYS 169

FB1_Tlp3 LNNGKVLLSQKSNDAKMPELRDDLDIKTKDWYQEALKTNDIFVTPAYLDTVLKQYVITYS 169

PT14_Tlp3 LNNGKVLLSQKSNDAKMPELRDDLDIKTKDWYQEALKTNDIFVTPAYLDTVLKQYVITYS 169

00-6200_Tlp3a LNNGKVLLSQKSNDAKMPELRDDLDIKTKDWYQEALKTNDIFVTPAYLDTVLKQYVITYS 169

RM1221_Tlp3 LNNGKVLLSQKSNDAKMPELRDDLDIKTKDWYQEALKTNDIFVTPAYLDTILKQYVITYS 169

S3_Tlp3 LNNGKVLLSQKSNDAKMPELRDDLDIKTKDWYQEALKTNDIFVTPAYLDTILKQYVITYS 169

FDAARGOS_421_Tlp3 LNNGKVLLSQKSNDAKMPELRDDLDIKTKDWYQEALKTNDIFVTPAYLDTILKQYVITYS 180

CFSAN032806_Tlp3 LNNGKVLLSQKSNDAKMPELRDDLDIKTKDWYQEALKTNDIFVTPAYLDTVLKQYVITYS 180

IA3901_Tlp3b LNNGKVLLSQKSNDAKMPELRDDLDIKTKDWYQEALKTNDIFVTPAYLDTVLKQYVITYS 169

00-6200_Tlp3b LNNGKVLLSQKSNDAKMPELRDDLDIKTKDWYQEALKTNDIFVTPAYLDTVLKQYVITYS 169

BCW_6290_Tlp3b LNNGKVLLSQKSNDAKMPELRDDLDIKTKDWYQEALKTNDIFVTPAYLDTVLKQYVITYS 169

00-2425_Tlp3a LNNGKVLLSQKSNDAKMPELRDDLDIKTKDWYQEALKTNDIFVTPAYLDTVLKQYVITYS 169

00-2425_Tlp3b LNNGKVLLSQKSNDAKMPELRDDLDIKTKDWYQEALKTNDIFVTPAYLDTVLKQYVITYS 169

YH001_Tlp3a LNNGKVLLSQKSNDAKMPELRDDLDIKTKDWYQEALKTNDIFVTPAYLDTVLKQYVITYS 169

YH001_Tlp3b LNNGKVLLSQKSNDAKMPELRDDLDIKTKDWYQEALKTNDIFVTPAYLDTVLKQYVITYS 169

00-0949_Tlp3 LNNGKVLLSQKSNDAKMPELRDDLDIKTKDWYQEALKTNDIFVTPAYLDTVLKQYVITYS 169

NCTC11168_Tlp3 LNNGKVLLSQKSNDAKMPELRDDLDIKTKDWYQEALKTNDIFVTPAYLDTVLKQYVITYS 180

F38011_Tlp3 LNNGKVLLSQKSNDAKMPELRDDLDIKTKDWYQEALKTNDIFVTPAYLDTVLKQYVITYS 169

RM1285_Tlp3 LNNGKVLLSQKSNDAKMPELRDDLDIKTKDWYQEALKTNDIFVTPAYLDTVLKQYVITYS 169

FDAARGOS_422_Tlp3 LNNGKVLLSQKSNDAKMPELRDDLDIKTKDWYQEALKTNDIFVTPAYLDTVLKQYVITYS 180

MTVDSCj07_Tlp3 LNNGKVLLSQKSNDAKMPELRDDLDIKTKDWYQEALKTNDIFVTPAYLDTVLKQYVITYS 169

IA3901_Tlp3a LNNGKVLLSQKSNDAKMPELRDDLDIKTKDWYQEALKTNDIFVTPAYLDTVLKQYVITYS 169

BCW_6290_Tlp3a LNNGKVLLSQKSNDAKMPELRDDLDIKTKDWYQEALKTNDIFVTPAYLDTVLKQYVITYS 169

CJ677CC012_Tlp3 LNNGKVLLSQKSNDAKMPELRDDLDIKTKDWYQEALKTNDIFVTPAYLDTNLKQYVITYS 169

CJM1cam LNNGKVLLSQKSNDAKMPELRDDLDIKTKDWYQEALKTNDIFVTPAYLDTNLKQYVITYS 169

M1_Tlp3 LNNGKVLLSQKSNDAKMPELRDDLDIKTKDWYQEALKTNDIFVTPAYLDTNLKQYVITYS 169

4031_Tlp3 LNNGKVLLSQKSNDAKMPELRDDLDIKTKDWYQEALKTNDIFVTPAYLDTNLKQYVITYS 169

R14_Tlp3 LNNGKVLLSQKSNDAKMPELRDDLDIKTKDWYQEALKTNDIFVTPAYLDTILKQYVITYS 169

35925B2_Tlp3 LNNGKVLLSQESNDAKMPELRDDLDIKTKDWYQEALKTNDIFVTPAYLDTILKQYVITYS 180

14980A LNNGKVLLSQKSNDAKMPELRDDLDIKTKDWYQEALKTNDIFVTPAYLDTILKQYVITYS 180

00-1597_Tlp3b LNNGKVLLSQKSNDAKMPELRDDLDIKTKDWYQEALKTNDIFVTPAYLDTVLKQYVITYS 169

ICDCCJ07001_Tlp3 LNNGKVLLSQKSNDAKMPELRDDLDIKTKDWYQEALKTNDIFVTPAYLDTVLKQYVITYS 169

RM3196_Tlp3 LNNGKVLLSQKSNDAKMPELRDDLDIKTKDWYQEALKTNDIFVTPAYLDTVLKQYVITYS 169

**********:******************************* ******* *********

HC2-48_Tlp3 KAIYKDGKIIGVLGVDIPSEDLQNLVAKTPGNTFLFDQKNKIFAATNKELLNPSIDHSPV 229

RM1875_Tlp3 KAIYKDGKIIGVLGVDIPSEDLQNLVANTPGNTFLFDQKNKIFAATNKELLNPSIDHSPV 229

CF2-75_Tlp3 KAIYKDGKIIGVLGVDIPSEDLQNLVAKTPGNTFLFDQKNKIFAATNKELLNPSIDHSPV 240

RM5611_Tlp3 KAIYKDGKIIGVLGVDIPSEDLQNLVAKTPGNTFLFDQKNKIFAATNKELLNPSIDHSPV 229

MTVDSCj16_Tlp3 KAIYKDGKIIGVLGVDIPSEDLQNLVAKTPGNTFLFDQKNKIFAATNEALLDPSVDHSPV 229

01-1512_Tlp3 KAIYKDGKIIGVLGVDIPSEDLQNLVAKTPGNTFLFDQKNKIFAATNKELLNPSIDHSPV 229

MTVDSCj13_Tlp3 KAIYKDGKIIGVLGVDIPSEDLQNLVANTPGNTFLFDQKNKIFAATNKELLNPSIDHSPV 229

32488_Tlp3a KAIYKDGKIIGVLGVDIPLEDLQNSVAKTPGNTFLFDQKNKIFAATNKELLNPSIDHSPV 229

81116_Tlp3 KAIYKDGKIIGVLGVDIPLEDLQNSVAKTPGNTFLFDQKNKIFAATNKELLNPSIDHSPV 229

32488_Tlp3b KAIYKDGKIIGVLGVDIPLEDLQNSVAKTPGNTFLFDQKNKIFAATNKELLNPSIDHSPV 229

FB1_Tlp3 KAIYKDGKIIGVLGVDIPSEDLQNLVAKTPGNTFLFDQKNKIFAATNKELLNPSIDHSPV 229

PT14_Tlp3 KAIYKDGKIIGVLGVDIPSEDLQNLVAKTPGNTFLFDQKNKIFAATNEALLDPSVDHSPV 229

00-6200_Tlp3a KAIYKDGKIIGVLGVDIPSEDLQNLVAKTPGNTFLFDQKNKIFAATNKELLNPSIDHSPV 229

RM1221_Tlp3 KAIYKDGKIIGVLGVDIPSEDLQNLVANTPGNTFLFDQKNKIFAATNKELLNPSIDHSPV 229

S3_Tlp3 KAIYKDGKIIGVLGVDIPSEDLQNLVANTPGNTFLFDQKNKIFAATNKELLNPSIDHSPV 229

FDAARGOS_421_Tlp3 KAIYKDGKIIGVLGVDIPSEDLQNLVANTPGNTFLFDQKNKIFAATNKELLNPSIDHSPV 240

CFSAN032806_Tlp3 KAIYKDGKIIGVLGVDIPSEDLQNLVAKTPGNTFLFDQKNKIFAATNKELLNPSIDHSPV 240

IA3901_Tlp3b KAIYKDGKIIGVLGVDIPSEDLQNLVAKTPGNTFLFDQKNKIFAATNKELLNPSIDHSPV 229

00-6200_Tlp3b KAIYKDGKIIGVLGVDIPSEDLQNLVAKTPGNTFLFDQKNKIFAATNKELLNPSIDHSPV 229

BCW_6290_Tlp3b KAIYKDGKIIGVLGVDIPSEDLQNLVAKTPGNTFLFDQKNKIFAATNKELLNPSIDHSPV 229

00-2425_Tlp3a KAIYKDGKIIGVLGVDIPSEDLQNLVAKTPGNTFLFDQKNKIFAATNKELLNPSIDHSPV 229

00-2425_Tlp3b KAIYKDGKIIGVLGVDIPSEDLQNLVAKTPGNTFLFDQKNKIFAATNKELLNPSIDHSPV 229

YH001_Tlp3a KAIYKDGKIIGVLGVDIPSEDLQNLVAKTPGNTFLFDQKNKIFAATNKELLNPSIDHSPV 229

YH001_Tlp3b KAIYKDGKIIGVLGVDIPSEDLQNLVAKTPGNTFLFDQKNKIFAATNKELLNPSIDHSPV 229

00-0949_Tlp3 KAIYKDGKIIGVLGVDIPSEDLQNLVAKTPGNTFLFDQKNKIFAATNKELLNPSIDHSPV 229

NCTC11168_Tlp3 KAIYKDGKIIGVLGVDIPSEDLQNLVAKTPGNTFLFDQKNKIFAATNKELLNPSIDHSPV 240

F38011_Tlp3 KAIYKDGKIIGVLGVDIPSEDLQNLVAKTPGNTFLFDQKNKIFAATNKELLNPSIDHSPV 229

RM1285_Tlp3 KAIYKDGKIIGVLGVDIPSEDLQNLVAKTPGNTFLFDQKNKIFAATNKELLNPSIDHSPV 229

FDAARGOS_422_Tlp3 KAIYKDGKIIGVLGVDIPSEDLQNLVAKTPGNTFLFDQKNKIFAATNKELLNPSIDHSPV 240

MTVDSCj07_Tlp3 KAIYKDGKIIGVLGVDIPSEDLQNLVAKTPGNTFLFDQKNKIFAATNKELLNPSIDHSPV 229

IA3901_Tlp3a KAIYKDGKIIGVLGVDIPSEDLQNLVAKTPGNTFLFDQKNKIFAATNKELLNPSIDHSPV 229

BCW_6290_Tlp3a KAIYKDGKIIGVLGVDIPSEDLQNLVAKTPGNTFLFDQKNKIFAATNKELLNPSIDHSPV 229

CJ677CC012_Tlp3 KAIYKDGKMIGVLGVDIPSEDLQNLVAKTPGNTFLFDQKNKIFAATNKELLNPSIDHSPV 229

CJM1cam KAIYKDGKIIGVLGVDIPSEDLQNLVAKTPGNTFLFDQKNKIFAATNKELLNPSIDHSPV 229

M1_Tlp3 KAIYKDGKIIGVLGVDIPSEDLQNLVAKTPGNTFLFDQKNKIFAATNKELLNPSIDHSPV 229

4031_Tlp3 KAIYKDGKIIGVLGVDIPSEHLQNLVAKTPGNTFLFDQKNKIFAATNKELLNPSIDHSPV 229

R14_Tlp3 KAIYKDGKIIGVLGVDIPLEDLQNSVANTPGNTFLFDQKNKIFAATNKELLNPSIDHSPV 229

35925B2_Tlp3 KAIYKDGKIIGVLGIDIPSEDLQNLVAKTPGNTFLFDQKNKIFAATNKELLNPSIDHSPV 240

14980A KAIYKDGKIIGVLGVDIPLEDLQNSVANTPGNIFLFDQKNKIFAATNKELLNPSIDHSPV 240

00-1597_Tlp3b KAIYKDGKIIGVLGVDIPSGDLQNLVAKTPGNTFLFDQKNKIFAATNKELLNPSIDHSPV 229

ICDCCJ07001_Tlp3 KAIYKDGKIIGVLGVDIPSEDLQNLVAKTPGNTFLFDQKNKIFAATNKELLNPSIDHSPV 229

RM3196_Tlp3 KAIYKDGKIIGVLGVDIPSEDLQNLVAKTPGNTFLFDQKNKIFAATNKELLNPSIDHSPV 229

********:*****:*** .*** **:**** **************: **:**:*****

HC2-48_Tlp3 LNAYKLNGDNNFFSYKLNNEERLGACTKVFAYTACITESADIINKPIYKAAFIQAIVVII 289

RM1875_Tlp3 LNAYKTHGDHNFFNYGLDGKERLGACTKVFAYTACITESADIINKPIYKAAFIQAIVVII 289

CF2-75_Tlp3 LNAYKLNGDNNFFSYKLNNEERLGACTKVFAYTACITESADIINKPIYKAAFIQAIVVII 300

RM5611_Tlp3 LNAYKLNGDNNFFSYKLNNEERLGACTKVFAYTACITESADIINKPIYKAAFIQAIVVII 289

MTVDSCj16_Tlp3 LNAYKAHGDNNFFSYKLNNEERLGACTKVFAYTACITESADIINKPIYKAAFIQVIALIV 289

01-1512_Tlp3 LNAYKLNGDNNFFSYKLNNEERLGACTKVFAYTACITESADIINKPIYKAAFIQVIALIV 289

MTVDSCj13_Tlp3 LNAYKLNGDNNFFSYKLNNEERLGACTKVFAYTACITESADIINKPIFKAAYIQVIALIV 289

32488_Tlp3a LNAYKLNGDNNFFSYKLNNEERLGACTKVFAYTACITESADIINKPIFKAAYIQVIALIV 289

81116_Tlp3 LNAYKLNGDNNFFSYKLNNEERLGACTKVFAYTACITESADIINKPIFKAAYIQVIALIV 289

32488_Tlp3b LNAYKLNGDNNFFSYKLNNEERLGACTKVFAYTACITESADIINKPIFKAAYIQVIALIV 289

FB1_Tlp3 LNAYKTHGDNNFFSYKLNNEERLGACTKVFAYTACITESADIINKPIYKAAFIQAIVVII 289

PT14_Tlp3 LNAYKAHGDNNFFSYKLNNEERLGACTKVFAYTACITESADIINKPIYKAAFIQAIVVII 289

00-6200_Tlp3a LNAYKLNGDNNFFSYKLNNEERLGACTKVFAYTACITESADIINKPIYKAAFIQAIVVII 289

RM1221_Tlp3 LNAYKLNGDNNFFSYKLNNEERLGACTKVFAYTACITESADIINKPIFKAAFIQAIVVII 289

S3_Tlp3 LNAYKLNGDNNFFSYKLNNEERLGACTKVFAYTACITESADIINKPIFKAAFIQAIVVII 289

FDAARGOS_421_Tlp3 LNAYKLNGDNNFFSYKLNNEERLGACTKVFAYTACITESADIINKPIFKAAFIQAIVVII 300

CFSAN032806_Tlp3 LNAYKLNGDNNFFSYKLNNEERLGACTKVFAYTACITESADIINKPIYKAAFIQAIVVII 300

IA3901_Tlp3b LNAYKLNGDNNFFSYKLNNEERLGACTKVFAYTACITESADIINKPIYKAAFIQAIVVII 289

00-6200_Tlp3b LNAYKLNGDNNFFSYKLNNEERLGACTKVFAYTACITESADIINKPIYKAAFIQAIVVII 289

BCW_6290_Tlp3b LNAYKLNGDNNFFSYKLNNEERLGACTKVFAYTACITESADIINKPIYKAAFIQAIVVII 289

00-2425_Tlp3a LNAYKLNGDNNFFSYKLNNEERLGACTKVFAYTACITESADIINKPIYKAAFIQAIVVII 289

00-2425_Tlp3b LNAYKLNGDNNFFSYKLNNEERLGACTKVFAYTACITESADIINKPIYKAAFIQAIVVII 289

YH001_Tlp3a LNAYKLNGDNNFFSYKLNNEERLGACTKVFAYTACITESADIINKPIYKAAFIQAIVVII 289

YH001_Tlp3b LNAYKLNGDNNFFSYKLNNEERLGACTKVFAYTACITESADIINKPIYKAAFIQAIVVII 289

00-0949_Tlp3 LNAYKLNGDNNFFSYKLNNEERLGACTKVFAYTACITESADIINKPIYKAAFIQAIVVII 289

NCTC11168_Tlp3 LNAYKLNGDNNFFSYKLNNEERLGACTKVFAYTACITESADIINKPIYKAAFIQAIVVII 300

F38011_Tlp3 LNAYKLNGDNNFFSYKLNNEERLGACTKVFAYTACITESADIINKPIYKAAFIQAIVVII 289

RM1285_Tlp3 LNAYKLNGDNNFFSYKLNNEERLGACTKVFAYTACITESADIINKPIYKAAFIQAIVVII 289

FDAARGOS_422_Tlp3 LNAYKLNGDNNFFSYKLNNEERLGACTKVFAYTACITESADIINKPIYKAAFIQAIVVII 300

MTVDSCj07_Tlp3 LNAYKLNGDNNFFSYKLNNEERLGACTKVFAYTACITESADIINKPIYKAAFIQAIVVII 289

IA3901_Tlp3a LNAYKLNGDNNFFSYKLNNEERLGACTKVFAYTACITESADIINKPIYKAAFIQAIVVII 289

BCW_6290_Tlp3a LNAYKLNGDNNFFSYKLNNEERLGACTKVFAYTACITESADIINKPIYKAAFIQAIVVII 289

CJ677CC012_Tlp3 LNAYKTHGDYNFFTYGLDGKERLGTCTKVFAYTACITESADIINKPIHKAAFIQAIVVII 289

CJM1cam LNAYKTHGDYNFFTYGLDGKERLGTCTKVFAYTACITESADIINKPIHKAAFIQAIVVII 289

M1_Tlp3 LNAYKTHGDYNFFTYGLDGKERLGTCTKVFAYTACITESADIINKPIHKAAFIQAIVVII 289

4031_Tlp3 LNAYKTHGDYNFFTYGLDGKERLGTCTKVFAYTACITESADIINKPIHKAAFIQAIVVII 289

R14_Tlp3 LNAYKTHGDYNFFTYGLDGKERLGTCTKVFAYTACITESADIINKPIHKAAFIQAIVVII 289

35925B2_Tlp3 LNAYKTHGDYNFFTYGLDGKERLGTCAKVFAYTACITESADIINKPIHKAAFIQAIVVII 300

14980A LNAYKTHGDYNFFTYGLDGKERLGTCTKVFAYTACITESADIINKPIHKAAFIQAIVVII 300

00-1597_Tlp3b LNAYKAHGDNNFFSYKLNNEERLGACTKVFAYTACITESADIINKPIFKAAYIQVIALIV 289

ICDCCJ07001_Tlp3 SNAYKAHGDNNFFSYKLNNEERLGACTKVFAYTACITESADIINKPIYKAAFIQAIVVII 289

RM3196_Tlp3 SNAYKAHGDNNFFSYKLNNEERLGACTKVFAYTACITESADIINKPIYKAAFIQAIVVII 289

**** :** ***.* *:.:****:*:********************.***:**.*.:*:

HC2-48_Tlp3 VVVFSVILLYFIVSKYLSPLAAIQTGLTSFFDFINYKTKNVSTIEVKTNDEFGQISKAIN 349

RM1875_Tlp3 VVVFSVILLYFIVSKYLSPLAAIQTGLTSFFDFINHKTKNVSTIDVKSNDEFGQISKAIN 349

CF2-75_Tlp3 VVVFSVILLYFIVSKYLSPLAAIQTGLTSFFDFINYKTKNVSTIEVKSNDEFGQISNAIN 360

RM5611_Tlp3 VVVFSVILLYFIVSKYLSPLAAIQTGLTSFFDFINYKTKNVSTIEVKSNDEFGQISNAIN 349

MTVDSCj16_Tlp3 MISISIILLYFIVSKYLSPLAAIQTGLTSFFDFINYKTKNVSTIEVKSNDEFGQISNAIN 349

01-1512_Tlp3 MISISIILLYFIVSKYLSPLAAIQTGLTSFFDFINYKTKNVSTIEVKSNDEFGQISNAIN 349

MTVDSCj13_Tlp3 MISISIILLYFIVSKYLSPLAAIQTGLTSFFDFINYKTKNVSTIEVKSNDEFGQISNAIN 349

32488_Tlp3a MISISIILLYFIVSKYLSPLAAIQTGLTSFFDFINYKTKNVSTIEVKSNDEFGQISNAIN 349

81116_Tlp3 MISISIILLYFIVSKYLSPLAAIQTGLTSFFDFINYKTKNVSTIEVKSNDEFGQISNAIN 349

32488_Tlp3b MISISIILLYFIVSKYLSPLAAIQTGLTSFFDFINYKTKNVSTIEVKSNDEFGQISNAIN 349

FB1_Tlp3 VVVFSVILLYFIVSKYLSPLAAIQTGLTSFFDFINYKTKNVSTIEVKSNDEFGQISNAIN 349

PT14_Tlp3 VVVFSVILLYFIVSKYLSPLAAIQTGLTSFFDFINYKTKNVSTIEVKSNDEFGQISNAIN 349

00-6200_Tlp3a VVVFSVILLYFIVSKYLSPLAAIQTGLTSFFDFINHKTKNVSTIEVKSNDELGQMGKIIN 349

RM1221_Tlp3 VVVFSVILLYFIVSKYLSPLAAIQTGLTSFFDFINYKTKNVSTIEVKSNDEFGQISNAIN 349

S3_Tlp3 VVVFSVILLYFIVSKYLSPLAAIQTGLTSFFDFINYKTKNVSTIEVKSNDEFGQISNAIN 349

FDAARGOS_421_Tlp3 VVVFSVILLYFIVSKYLSPLAAIQTGLTSFFDFINYKTKNVSTIEVKSNDEFGQISNAIN 360

CFSAN032806_Tlp3 VVVFSVILLYFIVSKYLSPLAAIQTGLTSFFDFINYKTKNVSTIEVKSNDEFGQISNAIN 360

IA3901_Tlp3b VVVFSVILLYFIVSKYLSPLAAIQTGLTSFFDFINYKTKNVSTIEVKSNDEFGQISNAIN 349

00-6200_Tlp3b VVVFSVILLYFIVSKYLSPLAAIQTGLTSFFDFINYKTKNVSTIEVKSNDEFGQISNAIN 349

BCW_6290_Tlp3b VVVFSVILLYFIVSKYLSPLAAIQTGLTSFFDFINYKTKNVSTIEVKSNDEFGQISNAIN 349

00-2425_Tlp3a VVVFSVILLYFIVSKYLSPLAAIQTGLTSFFDFINHKTKNVSTIEVKSNDEFGQISNAIN 349

00-2425_Tlp3b VVVFSVILLYFIVSKYLSPLAAIQTGLTSFFDFINHKTKNVSTIEVKSNDEFGQISNAIN 349

YH001_Tlp3a VVVFSVILLYFIVSKYLSPLAAIQTGLTSFFDFINHKTKNVSTIEVKSNDEFGQISNAIN 349

YH001_Tlp3b VVVFSVILLYFIVSKYLSPLAAIQTGLTSFFDFINHKTKNVSTIEVKSNDEFGQISNAIN 349

00-0949_Tlp3 VVVFSIILLYFIVSKYLSPLAAIQTGLTSFFDFINYKTKNVSTIEVKSNDEFGQISNAIN 349

NCTC11168_Tlp3 VVVFSVILLYFIVSKYLSPLAAIQTGLTSFFDFINYKTKNVSTIEVKSNDEFGQISNAIN 360

F38011_Tlp3 VVVFSVILLYFIVSKYLSPLAAIQTGLTSFFDFINYKTKNVSTIEVKSNDEFGQISNAIN 349

RM1285_Tlp3 VVVFSVILLYFIVSKYLSPLAAIQTGLTSFFDFINYKTKNVSTIEVKSNDEFGQISNAIN 349

FDAARGOS_422_Tlp3 VVVFSVILLYFIVSKYLSPLAAIQTGLTSFFDFINYKTKNVSTIEVKSNDEFGQISNAIN 360

MTVDSCj07_Tlp3 VVVFSVILLYFIVSKYLSPLAAIQTGLTSFFDFINYKTKNVSTIEVKSNDEFGQISNAIN 349

IA3901_Tlp3a VVVFSVILLYFIVSKYLSPLAAIQTGLTSFFDFINYKTKNVSTIEVKSNDEFGQISNAIN 349

BCW_6290_Tlp3a VVVFSVILLYFIVSKYLSPLAAIQTGLTSFFDFINYKTKNVSTIEVKSNDEFGQISNAIN 349

CJ677CC012_Tlp3 VVVFSVILLYFIVSKYLSPLAAIQTGLTSFFDFINYKTKNVSIIEVKSNDEFGQISSAIN 349

CJM1cam VVVFSVILLYFIVSKYLSPLAAIQTGLTSFFDFINHKTKNVSTIEVKSNDEFGQISSAIN 349

M1_Tlp3 VVVFSVILLYFIVSKYLSPLAAIQTGLTSFFDFINHKTKNVSTIEVKSNDEFGQISSAIN 349

4031_Tlp3 VVVFSVILLYFIVSKYLSPLAAIQTGLTSFFDFINHKTKNVSTIEVKSNDEFGQISNAIN 349

R14_Tlp3 VVVFSVILLYFIVSKYLSPLAAIQTGLTSFFDFINYKTKNVSTIEVKSNDEFGQISNAIN 349

35925B2_Tlp3 VVVFSVILLYFIISKYLSPLAAIQTGLTSFFDFINHKTKNVSTIEVKSNDEFGQISSAIN 360

14980A VVVFSVILLYFIVSKYLSPLAAIQTGLTSFFDFINYKTKNVSTIEVKSNDEFGQISNAIN 360

00-1597_Tlp3b MISISIILLYFIVSKYLSPLAAIQTGLTSFFDFINYKTKNVSTIEVKSNDEFGQISNAIN 349

ICDCCJ07001_Tlp3 VVVFSVILLYFIVSKYLSPLAAIQTGLTSFFDFINHKTKNVSTIEVKSNDEFGQISNAIN 349

RM3196_Tlp3 VVVFSVILLYFIVSKYLSPLAAIQTGLTSFFDFINHKTKNVSTIEVKSNDEFGQISNAIN 349

:: :*:******:**********************:****** *:**:***:**:.. **

HC2-48_Tlp3 ENILATKQGLEQDAKAVKESVETVGVVESGNLTARITANPRNPQLIELKNVLNRLLDVLQ 409

RM1875_Tlp3 ENILATKQGLEQDAKAVKESVETVGVVESGNLTARITANPRNPQLIELKNVLNRLLDVLQ 409

CF2-75_Tlp3 ENILATKQGLEQDAKAVKESVETVGVVESGNLTARITANPRNPQLIELKNVLNRLLDVLQ 420

RM5611_Tlp3 ENILATKQGLEQDAKAVKESVETVGVVESGNLTARITANPRNPQLIELKNVLNRLLDVLQ 409

MTVDSCj16_Tlp3 ENILATKRGLEQDNQAVKESVQTVSVVEGGNLTARITANPRNPQLIELKNVLNKLLDVLQ 409

01-1512_Tlp3 ENILATKRGLEQDNQAVKESVQTVSVVEGGNLTARITANPRNPQLIELKNVLNKLLDVLQ 409

MTVDSCj13_Tlp3 ENILATKRGLEQDNQAVKESVQTVSVVESGNLTARITANPRNPQLIELKNVLNKLLDVLQ 409

32488_Tlp3a ENILATKRGLEQDNQAVKESVQTVSVVEGGNLTARITANPRNPQLIELKNVLNKLLDVLQ 409

81116_Tlp3 ENILATKRGLEQDNQAVKESVQTVSVVEGGNLTARITANPRNPQLIELKNVLNKLLDVLQ 409

32488_Tlp3b ENILATKRGLEQDNQAVKESVQTVSVVEGGNLTARITANPRNPQLIELKNVLNKLLDVLQ 409

FB1_Tlp3 ENILATKQGLEQDAKAVKESVETVGVVERGNLTARITANPRNPQLIELKNVLNKLLDVLQ 409

PT14_Tlp3 ENILATKRGLEQDNQAVKESVQTVSVVEGGNLTARITANPRNPQLIELKNVLNKLLDVLQ 409

00-6200_Tlp3a ENILATKRGLEQDNQAVKESVQTVSVVEGGNLTARITANPRNPQLIELKNVLNKLLDVLQ 409

RM1221_Tlp3 ENILATKRGLEQDNQAVKESVQTVSVVEGGNLTARITANPRNPQLIELKNVLNKLLDVLQ 409

S3_Tlp3 ENILATKRGLEQDNQAVKESVQTVSVVEGGNLTARITANPRNPQLIELKNVLNKLLDVLQ 409

FDAARGOS_421_Tlp3 ENILATKRGLEQDNQAVKESVQTVSVVEGGNLTARITANPRNPQLIELKNVLNKLLDVLQ 420

CFSAN032806_Tlp3 ENILATKRGLEQDNQAVKESVQTVSVVEGGNLTARITANPRNPQLIELKNVLNKLLDVLQ 420

IA3901_Tlp3b ENILATKRGLEQDNQAVKESVQTVSVVEGGNLTARITANPRNPQLIELKNVLNKLLDVLQ 409

00-6200_Tlp3b ENILATKRGLEQDNQAVKESVQTVSVVEGGNLTARITANPRNPQLIELKNVLNKLLDVLQ 409

BCW_6290_Tlp3b ENILATKRGLEQDNQAVKESVQTVSVVEGGNLTARITANPRNPQLIELKNVLNKLLDVLQ 409

00-2425_Tlp3a ENILATKRGLEQDNQAVKESVQTVSVVEGGNLTARITANPRNPQLIELKNVLNKLLDVLQ 409

00-2425_Tlp3b ENILATKRGLEQDNQAVKESVQTVSVVEGGNLTARITANPRNPQLIELKNVLNKLLDVLQ 409

YH001_Tlp3a ENILATKRGLEQDNQAVKESVQTVSVVEGGNLTARITANPRNPQLIELKNVLNKLLDVLQ 409

YH001_Tlp3b ENILATKRGLEQDNQAVKESVQTVSVVEGGNLTARITANPRNPQLIELKNVLNKLLDVLQ 409

00-0949_Tlp3 ENILATKRGLEQDNQAVKESVQTVSVVEGGNLTARITANPRNPQLIELKNVLNKLLDVLQ 409

NCTC11168_Tlp3 ENILATKRGLEQDNQAVKESVQTVSVVEGGNLTARITANPRNPQLIELKNVLNKLLDVLQ 420

F38011_Tlp3 ENILATKRGLEQDNQAVKESVQTVSVVEGGNLTARITANPRNPQLIELKNVLNKLLDVLQ 409

RM1285_Tlp3 ENILATKRGLEQDNQAVKESVQTVSVVEGGNLTARITANPRNPQLIELKNVLNKLLDVLQ 409

FDAARGOS_422_Tlp3 ENILATKRGLEQDNQAVKESVQTVSVVEGGNLTARITANPRNPQLIELKNVLNKLLDVLQ 420

MTVDSCj07_Tlp3 ENILATKRGLEQDNQAVKESVQTVSVVEGGNLTARITANPRNPQLIELKNVLNKLLDVLQ 409

IA3901_Tlp3a ENILATKRGLEQDNQAVKESVQTVSVVEGGNLTARITANPRNPQLIELKNVLNKLLDVLQ 409

BCW_6290_Tlp3a ENILATKRGLEQDNQAVKESVQTVSVVEGGNLTARITANPRNPQLIELKNVLNKLLDVLQ 409

CJ677CC012_Tlp3 ENILATKKGLEQDNQAVKESVQTVSVVEGGNLTARITANPRNPQLIELKNVLNKLLDVLQ 409

CJM1cam ENILATKRGLEQDNQAVKESVETVSVVESGNLTARITANPRNPQLIELKNVLNKLLDVLQ 409

M1_Tlp3 ENILATKRGLEQDNQAVKESVETVSVVESGNLTARITANPRNPQLIELKNVLNKLLDVLQ 409

4031_Tlp3 ENILATKRGLEQDNQAVKESVETVSVVESGNLTARITANPRNPQLIELKNVLNKLLDVLQ 409

R14_Tlp3 ENILATKRGLEQDNQAVKESVQTVSVVEGGNLTARITANPRNPQLIELKNVLNKLLDVLQ 409

35925B2_Tlp3 ENILQTKKGLEQDNQAVKESVETVSVVESGNLTARITANPRNPQLIELKNVLNRLLDALQ 420

14980A ENILATKRGLEQDNQAVKESVQTVSVVEGGNLTARITANPRNPQLIELKNVLNRLLDALQ 420

00-1597_Tlp3b ENILATKRGLEQDNQAVKESVQTVSVVEGGNLTARITANPRNPQLIELKNVLNRLLDALQ 409

ICDCCJ07001_Tlp3 ENILATKRGLEQDNQAVKESVETVHVVEGGNLTARITANPRNPQLIELKNVLNRLLDALQ 409

RM3196_Tlp3 ENILATKRGLEQDNQAVKESVETVHVVEGGNLTARITANPRNPQLIELKNVLNRLLDALQ 409

**** **:***** :******:** *** ************************:***.**

HC2-48_Tlp3 TKVGS---DMNAIHKIFEEYKSLDFRNKLDNANGSVEVTTNALGDEIVKMLKQSSDFANH 466

RM1875_Tlp3 TRVGS---DMNAIHKIFEEYKSLDFRNKLDNANGSVEVTTNALGMKLVKMLKQSSDFANH 466

CF2-75_Tlp3 TKVGS---DMNAIHKIFEEYKSLDFRNKLDNANGSVEVTTNALGDEIVKMLKQSSDFANH 477

RM5611_Tlp3 TKVGS---DMNAIHKIFEEYKSLDFRNKLDNANGSVEVTTNALGDEIVKMLKQSSDFANH 466

MTVDSCj16_Tlp3 ARVGS---DMNAIHKIFEEYKSLDFRNKLENASGSVELTTNALGDEIVKMLKQSSDFANA 466

01-1512_Tlp3 ARVGS---DMNAIHKIFEEYKSLDFRNKLENASGSVELTTNALGDEIVKMLKQSSDFANA 466

MTVDSCj13_Tlp3 ARVGS---DMNAIHKIFEEYKSLDFRNKLENASGSVELTTNALGDEIVKMLKQSSDFANA 466

32488_Tlp3a ARVGS---DMNAIHKIFEEYKSLDFRNKLENASGSVELTTNALGDEIVKMLKQSSDFANA 466

81116_Tlp3 ARVGS---DMNAIHKIFEEYKSLDFRNKLENASGSVELTTNALGDEIVKMLKQSSDFANA 466

32488_Tlp3b ARVGS---DMNAIHKIFEEYKSLDFRNKLENASGSVELTTNALGDEIVKMLKQSSDFANA 466

FB1_Tlp3 ARVGS---DMNAIHKIFEEYKSLDFRNKLENASGSVELTTNALGDEIVKMLKQSSDFANA 466

PT14_Tlp3 ARVGS---DMNAIHKIFEEYKSLDFRNKLENASGSVELTTNALGDEIVKMLKQSSDFANA 466

00-6200_Tlp3a ARVGS---DMNAIHKIFEEYKSLDFRNKLENASGSVELTTNALGDEIVKMLKQSSDFANA 466

RM1221_Tlp3 ARVGS---DMNAIHKIFEEYKSLDFRNKLENASGSVELTTNALGDEIVKMLKQSSDFANA 466

S3_Tlp3 ARVGS---DMNAIHKIFEEYKSLDFRNKLENASGSVELTTNALGDEIVKMLKQSSDFANA 466

FDAARGOS_421_Tlp3 ARVGS---DMNAIHKIFEEYKSLDFRNKLENASGSVELTTNALGDEIVKMLKQSSDFANA 477

CFSAN032806_Tlp3 ARVGS---DMNAIHKIFEEYKSLDFRNKLENASGSVELTTNALGDEIVKMLKQSSDFANA 477

IA3901_Tlp3b ARVGS---DMNAIHKIFEEYKSLDFRNKLENASGSVELTTNALGDEIVKMLKQSSDFANA 466

00-6200_Tlp3b ARVGS---DMNAIHKIFEEYKSLDFRNKLENASGSVELTTNALGDEIVKMLKQSSDFANA 466

BCW_6290_Tlp3b ARVGS---DMNAIHKIFEEYKSLDFRNKLENASGSVELTTNALGDEIVKMLKQSSDFANA 466

00-2425_Tlp3a ARVGS---DMNAIHKIFEEYKSLDFRNKLENASGSVELTTNALGDEIVKMLKQSSDFANA 466

00-2425_Tlp3b ARVGS---DMNAIHKIFEEYKSLDFRNKLENASGSVELTTNALGDEIVKMLKQSSDFANA 466

YH001_Tlp3a ARVGS---DMNAIHKIFEEYKSLDFRNKLENASGSVELTTNALGDEIVKMLKQSSDFANA 466

YH001_Tlp3b ARVGS---DMNAIHKIFEEYKSLDFRNKLENASGSVELTTNALGDEIVKMLKQSSDFANA 466

00-0949_Tlp3 ARVGS---DMNAIHKIFEEYKSLDFRNKLENASGSVELTTNALGDEIVKMLKQSSDFANA 466

NCTC11168_Tlp3 ARVGS---DMNAIHKIFEEYKSLDFRNKLENASGSVELTTNALGDEIVKMLKQSSDFANA 477

F38011_Tlp3 ARVGS---DMNAIHKIFEEYKSLDFRNKLENASGSVELTTNALGDEIVKMLKQSSDFANA 466

RM1285_Tlp3 ARVGS---DMNAIHKIFEEYKSLDFRNKLENASGSVELTTNALGDEIVKMLKQSSDFANA 466

FDAARGOS_422_Tlp3 ARVGS---DMNAIHKIFEEYKSLDFRNKLENASGSVELTTNALGDEIVKMLKQSSDFANA 477

MTVDSCj07_Tlp3 ARVGS---DMNAIHKIFEEYKSLDFRNKLENASGSVELTTNALGDEIVKMLKQSSDFANA 466

IA3901_Tlp3a ARVGS---DMNAIHKIFEEYKSLDFRNKLENASGSVELTTNALGDEIVKMLKQSSDFANA 466

BCW_6290_Tlp3a ARVGS---DMNAIHKIFEEYKSLDFRNKLENASGSVELTTNALGDEIVKMLKQSSDFANA 466

CJ677CC012_Tlp3 ARVGS---DMNVIH---------------------------------------------- 420

CJM1cam ARVGS---DMNAIHKIFEEYKSLDFRNKLENASGSVELTTNALGDEIVKMLKQSSDFANA 466

M1_Tlp3 ARVGS---DMNAIHKIFEEYKSLDFRNKLENASGSVELTTNALGDEIVKMLKQSSDFANA 466

4031_Tlp3 ARVGS---DMNAIHKIFEEYKSLDFRNKLENASGSVELTTNALGDEIVKMLKQSSDFANA 466

R14_Tlp3 ARVGS---DMNAIHKIFEEYKSLDFRNKLENASGSVELTTNALGNEIVKMLKQSSDFANA 466

35925B2_Tlp3 TRVGSDGSDMNEIQRVFNSYKSLDFTTEVKDANGAVEVTTNALGQEIIKMLKQSSDFANA 480

14980A ARVG---SDMNEIQRVFNSYKSLDFTTEVKDANGAVEVTTNALGQEIIKMLKQSSDFANA 477

00-1597_Tlp3b ARVG---SDMNEIQRVFNSYKSLDFTTEVKDANGAVEVTTNALGQEIIKMLKQSSDFANA 466

ICDCCJ07001_Tlp3 ARVG---SDMNEIQRVFNSYKSLDFTTEVKDANGAVEVTTNALGQEIIKMLKQSSDFANA 466

RM3196_Tlp3 ARVG---SDMNEIQRVFNSYKSLDFTTEVKDANGAVEVTTNALGQEIIKMLKQSSDFANA 466

::** *** *:

HC2-48_Tlp3 LARKFKTSKCSSKPYFI----------------F-FSSSF-------------------- 489

RM1875_Tlp3 LASESSKLQSAVQNLTSSSNSQAASLEETAAALEEITSSMQNVSVKTSDVITQSEEIKNV 526

CF2-75_Tlp3 LASESSKLQSAVQNLTSSSNSQAASLEETAAALEEITSSMQNVSVKTSDVITQSEEIKNV 537

RM5611_Tlp3 LASESSKLQSAVQNLTSSSNSQAASLEETAAALEEITSSMQNVSVKTSDVITQSEEIKNV 526

MTVDSCj16_Tlp3 LANESGKLQTAVQSLTTSSNSQAQSLEETAAALEEITSSMQNVSVKTSDVITQSEEIKNV 526

01-1512_Tlp3 LANESGKLQTAVQSLTTSSNSQAQSLEETAAALEEITSSMQNVSVKTSDVITQSEEIKNV 526

MTVDSCj13_Tlp3 LANESGKLQTAVQSLTTSSNSQAQSLEETAAALEEITSSMQNVSVKTSDVITQSEEIKNV 526

32488_Tlp3a LANESGKLQTAVQSLTTSSNSQAQSLEETAAALEEITSSMQNVSVKTSDVITQSEEIKNV 526

81116_Tlp3 LANESGKLQTAVQSLTTSSNSQAQSLEETAAALEEITSSMQNVSVKTSDVITQSEEIKNV 526

32488_Tlp3b LANESGKLQTAVQSLTTSSNSQAQSLEETAAALEEITSSMQNVSVKTSDVITQSEEIKNV 526

FB1_Tlp3 LANESGKLQTAVQSLTTSSNSQAQSLEETAAALEEITSSMQNVSVKTSDVITQSEEIKNV 526

PT14_Tlp3 LANESGKLQTAVQSLTTSSNSQAQSLEETAAALEEITSSMQNVSVKTSDVITQSEEIKNV 526

00-6200_Tlp3a LANESGKLQTAVQSLTTSSNSQAQSLEETAAALEEITSSMQNVSVKTSDVITQSEEIKNV 526

RM1221_Tlp3 LANESGKLQTAVQSLTTSSNSQAQSLEETAAALEEITSSMQNVSVKTSDVITQSEEIKNV 526

S3_Tlp3 LANESGKLQTAVQSLTTSSNSQAQSLEETAAALEEITSSMQNVSVKTSDVITQSEEIKNV 526

FDAARGOS_421_Tlp3 LANESGKLQTAVQSLTTSSNSQAQSLEETAAALEEITSSMQNVSVKTSDVITQSEEIKNV 537

CFSAN032806_Tlp3 LANESGKLQTAVQSLTTSSNSQAQSLEETAAALEEITSSMQNVSVKTSDVITQSEEIKNV 537

IA3901_Tlp3b LANESGKLQTAVQSLTTSSNSQAQSLEETAAALEEITSSMQNVSVKTSDVITQSEEIKNV 526

00-6200_Tlp3b LANESGKLQTAVQSLTTSSNSQAQSLEETAAALEEITSSMQNVSVKTSDVITQSEEIKNV 526

BCW_6290_Tlp3b LANESGKLQTAVQSLTTSSNSQAQSLEETAAALEEITSSMQNVSVKTSDVITQSEEIKNV 526

00-2425_Tlp3a LANESGKLQTAVQSLTTSSNSQAQSLEETAAALEEITSSMQNVSVKTSDVITQSEEIKNV 526

00-2425_Tlp3b LANESGKLQTAVQSLTTSSNSQAQSLEETAAALEEITSSMQNVSVKTSDVITQSEEIKNV 526

YH001_Tlp3a LANESGKLQTAVQSLTTSSNSQAQSLEETAAALEEITSSMQNVSVKTSDVITQSEEIKNV 526

YH001_Tlp3b LANESGKLQTAVQSLTTSSNSQAQSLEETAAALEEITSSMQNVSVKTSDVITQSEEIKNV 526

00-0949_Tlp3 LANESGKLQTAVQSLTTSSNSQAQSLEETAAALEEITSSMQNVSVKTSDVITQSEEIKNV 526

NCTC11168_Tlp3 LANESGKLQTAVQSLTTSSNSQAQSLEETAAALEEITSSMQNVSVKTSDVITQSEEIKNV 537

F38011_Tlp3 LANESGKLQTAVQSLTTSSNSQAQSLEETAAALEEITSSMQNVSVKTSDVITQSEEIKNV 526

RM1285_Tlp3 LANESGKLQTAVQSLTTSSNSQAQSLEETAAALEEITSSMQNVSVKTSDVITQSEEIKNV 526

FDAARGOS_422_Tlp3 LANESGKLQTAVQSLTTSSNSQAQSLEETAAALEEITSSMQNVSVKTSDVITQSEEIKNV 537

MTVDSCj07_Tlp3 LANESGKLQTAVQSLTTSSNSQAQSLEETAAALEEITSSMQNVSVKTSDVITQSEEIKNV 526

IA3901_Tlp3a LANESGKLQTAVQSLTTSSNSQAQSLEETAAALEEITSSMQNVSVKTSDVITQSEEIKNV 526

BCW_6290_Tlp3a LANESGKLQTAVQSLTTSSNSQAQSLEETAAALEEITSSMQNVSVKTSDVITQSEEIKNV 526

CJ677CC012_Tlp3 ------------------------------------------------------------ 420

CJM1cam LANESGKLQTAVQSLTTSSNSQAQSLEETAAALEEITSSMQNVSVKTSDVITQSEEIKNV 526

M1_Tlp3 LANESGKLQTAVQSLTTSSNSQAQSLEETAAALEEITSSMQNVSVKTSDVITQSEEIKNV 526

4031_Tlp3 LANESGKLQTAVQSLTTSSNSQAQSLEETAAALEEITSSMQNVSVKTSDVITQSEEIKNV 526

R14_Tlp3 LANESGKLQTAVQSLTTSSNSQAQSLEETAAALEEITSSMQNVSVKTSDVITQSEEIKNV 526

35925B2_Tlp3 LANESGKLQTAVQSLTTSSNSQAQSLEETAAALEEITSSMQNVSVKTSDVITQSEEIKNV 540

14980A LANESGKLQTAVQSLTTSSNSQAQSLEETAAALEEITSSMQNVSVKTSDVITQSEEIKNV 537

00-1597_Tlp3b LANESGKLQTAVQSLTTSSNSQAQSLEETAAALEEITSSMQNVSVKTSDVITQSEEIKNV 526

ICDCCJ07001_Tlp3 LANESGKLQTAVQSLTTSSNSQAQSLEETAAALEEITSSMQNVSVKTSDVITQSEEIKNV 526

RM3196_Tlp3 LANESGKLQTAVQSLTTSSNSQAQSLEETAAALEEITSSMQNVSVKTSDVITQSEEIKNV 526

HC2-48_Tlp3 --------------------FRRNCSCFRRDYFFYAKCF--CKNQ-CYHSIEIEANTNLL 526

RM1875_Tlp3 TGIIGDIADQINLLALNAAIEAARAGEHGRGFAVVADEVRKLAERTQKSLSEIEANTNLL 586

CF2-75_Tlp3 TGIIGDIADQINLLA----------------------------EITQKSLSEIEANTNLL 569

RM5611_Tlp3 TGIIGDIADQINLLALNAAIEAARAGEHGRGFAVVADEVRKLAERTQKSLSEIEANTNLL 586

MTVDSCj16_Tlp3 TGIIGDIADQINLLALNAAIEAARAGEHGRGFAVVADEVRKLAERTQKSLSEIEANTNLL 586

01-1512_Tlp3 TGIIGDIADQINLLALNAAIEAARAGEHGRGFAVVADEVRKLAERTQKSLSEIEANTNLL 586

MTVDSCj13_Tlp3 TGIIGDIADQINLLALNAAIEAARAGEHGRGFAVVADEVRKLAERTQKSLSEIEANTNLL 586

32488_Tlp3a TGIIGDIADQINLLALNAAIEAARAGEHGRGFAVVADEVRKLAERTQKSLSEIEANTNLL 586

81116_Tlp3 TGIIGDIADQINLLALNAAIEAARAGEHGRGFAVVADEVRKLAERTQKSLSEIEANTNLL 586

32488_Tlp3b TGIIGDIADQINLLALNAAIEAARAGEHGRGFAVVADEVRKLAERTQKSLSEIEANTNLL 586

FB1_Tlp3 TGIIGDIADQINLLALNAAIEAARAGEHGRGFAVVADEVRKLAERTQKSLSEIEANTNLL 586

PT14_Tlp3 TGIIGDIADQINLLALNAAIEAARAGEHGRGFAVVADEVRKLAERTQKSLSEIEANTNLL 586

00-6200_Tlp3a TGIIGDIADQINLLALNAAIEAARAGEHGRGFAVVADEVRKLAERTQKSLSEIEANTNLL 586

RM1221_Tlp3 TGIIGDIADQINLLALNAAIEAARAGEHGRGFAVVADEVRKLAERTQKSLSEIEANTNLL 586

S3_Tlp3 TGIIGDIADQINLLALNAAIEAARAGEHGRGFAVVADEVRKLAERTQKSLSEIEANTNLL 586

FDAARGOS_421_Tlp3 TGIIGDIADQINLLALNAAIEAARAGEHGRGFAVVADEVRKLAERTQKSLSEIEANTNLL 597

CFSAN032806_Tlp3 TGIIGDIADQINLLALNAAIEAARAGEHGRGFAVVADEVRKLAERTQKSLSEIEANTNLL 597

IA3901_Tlp3b TGIIGDIADQINLLALNAAIEAARAGEHGRGFAVVADEVRKLAERTQKSLSEIEANTNLL 586

00-6200_Tlp3b TGIIGDIADQINLLALNAAIEAARAGEHGRGFAVVADEVRKLAERTQKSLSEIEANTNLL 586

BCW_6290_Tlp3b TGIIGDIADQINLLALNAAIEAARAGEHGRGFAVVADEVRKLAERTQKSLSEIEANTNLL 586

00-2425_Tlp3a TGIIGDIADQINLLALNAAIEAARAGEHGRGFAVVADEVRKLAERTQKSLSEIEANTNLL 586

00-2425_Tlp3b TGIIGDIADQINLLALNAAIEAARAGEHGRGFAVVADEVRKLAERTQKSLSEIEANTNLL 586

YH001_Tlp3a TGIIGDIADQINLLALNAAIEAARAGEHGRGFAVVADEVRKLAERTQKSLSEIEANTNLL 586

YH001_Tlp3b TGIIGDIADQINLLALNAAIEAARAGEHGRGFAVVADEVRKLAERTQKSLSEIEANTNLL 586

00-0949_Tlp3 TGIIGDIADQINLLALNAAIEAARAGEHGRGFAVVADEVRKLAERTQKSLSEIEANTNLL 586

NCTC11168_Tlp3 TGIIGDIADQINLLALNAAIEAARAGEHGRGFAVVADEVRKLAERTQKSLSEIEANTNLL 597

F38011_Tlp3 TGIIGDIADQINLLALNAAIEAARAGEHGRGFAVVADEVRKLAERTQKSLSEIEANTNLL 586

RM1285_Tlp3 TGIIGDIADQINLLALNAAIEAARAGEHGRGFAVVADEVRKLAERTQKSLSEIEANTNLL 586

FDAARGOS_422_Tlp3 TGIIGDIADQINLLALNAAIEAARAGEHGRGFAVVADEVRKLAERTQKSLSEIEANTNLL 597

MTVDSCj07_Tlp3 TGIIGDIADQINLLALNAAIEAARAGEHGRGFAVVADEVRKLAERTQKSLSEIEANTNLL 586

IA3901_Tlp3a TGIIGDIADQINLLALNAAIEAARAGEHGRGFAVVADEVRKLAERTQKSLSEIEANTNLL 586

BCW_6290_Tlp3a TGIIGDIADQINLLALNAAIEAARAGEHGRGFAVVADEVRKLAERTQKSLSEIEANTNLL 586

CJ677CC012_Tlp3 ------------------------------------DEVRKLAERTQKSLSEIEANTNLL 444

CJM1cam TGIIGDIADQINLLALNAAIEAARAGEHGRGFAVVADEVRKLAERTQKSLSEIEANTNLL 586

M1_Tlp3 TGIIGDIADQINLLALNAAIEAARAGEHGRGFAVVADEVRKLAERTQKSLSEIEANTNLL 586

4031_Tlp3 TGIIGDIADQINLLALNAAIEAARAGEHGRGFAVVADEVRKLAERTQKSLSEIEANTNLL 586

R14_Tlp3 TGIIGDIADQINLLALNAAIEAARAGEHGRGFAVVADEVRKLAERTQKSLSEIEANTNLL 586

35925B2_Tlp3 TGIIGDIADQINLLALNAAIEAARAGEHGRGFAVVADEVRKLAERTQKSLSEIEANTNLL 600

14980A TGIIGDIADQINLLALNAAIEAARAGEHGRGFAVVADEVRKLAERTQKSLSEIEANTNLL 597

00-1597_Tlp3b TGIIGDIADQINLLALNAAIEAARAGEHGRGFAVVADEVRKLAERTQKSLSEIEANTNLL 586

ICDCCJ07001_Tlp3 TGIIGDIADQINLLALNAAIEAARAGEHGRGFAVVADEVRKLAERTQKSLSEIEANTNLL 586

RM3196_Tlp3 TGIIGDIADQINLLALNAAIEAARAGEHGRGFAVVADEVRKLAERTQKSLSEIEANTNLL 586

: *********

HC2-48_Tlp3 VQSINDMAESIKEQTAGITQINESVAQIDQTTKDNVEIANESAIISSTVSDIANNILEDV 586

RM1875_Tlp3 VQSINDMAESIKEQTAGITQINESVAQIDQTTKDNVEIANESAIISNTVSDIANNILEDV 646

CF2-75_Tlp3 VQSINDMAESIKEQTAGITQINESVAQIDQTTKDNVEIANESAIISSTVSDIANNILEDV 629

RM5611_Tlp3 VQSINDMAESIKEQTAGITQINESVAQIDQTTKDNVEIANESAIISSTVSDIANNILEDV 646

MTVDSCj16_Tlp3 VQSINDMAESIKEQTAGITQINDSVAQIDQTTKDNVEIANESAIISSTVSDIANNILEDV 646

01-1512_Tlp3 VQSINDMAESIKEQTAGITQINDSVAQIDQTTKDNVEIANESAIISSTVSDIANNILEDV 646

MTVDSCj13_Tlp3 VQSINDMAESIKEQTAGITQINDSVAQIDQTTKDNVEIANESAIISSTVSDIANNILEDV 646

32488_Tlp3a VQSINDMAESIKEQTAGITQINDSVAQIDQTTKDNVEIANESAIISSTVSDIANNILEDV 646

81116_Tlp3 VQSINDMAESIKEQTAGITQINDSVAQIDQTTKDNVEIANESAIISSTVSDIANNILEDV 646

32488_Tlp3b VQSINDMAESIKEQTAGITQINDSVAQIDQTTKDNVEIANESAIISSTVSDIANNILEDV 646

FB1_Tlp3 VQSINDMAESIKEQTAGITQINESVAQIDQTTKDNVEIANESAIISSTVSDIANNILEDV 646

PT14_Tlp3 VQSINDMAESIKEQTAGITQINDSVAQIDQTTKDNVEIANESAIISSTVSDIANNILEDV 646

00-6200_Tlp3a VQSINDMAESIKEQTAGITQINDSVAQIDQTTKDNVEIANESAIISSTVSDIANNILEDI 646

RM1221_Tlp3 VQSINDMAESIKEQTAGITQINDSVAQIDQTTKDNVEIANESAIISSTVSDIANNILEDV 646

S3_Tlp3 VQSINDMAESIKEQTAGITQINDSVAQIDQTTKDNVEIANESAIISSTVSDIANNILEDV 646

FDAARGOS_421_Tlp3 VQSINDMAESIKEQTAGITQINDSVAQIDQTTKDNVEIANESAIISSTVSDIANNILEDV 657

CFSAN032806_Tlp3 VQSINDMAESIKEQTAGITQINDSVAQIDQTAKDNVEIANESAIISNTVSDIANNILEDV 657

IA3901_Tlp3b VQSINDMAESIKEQTAGITQINDSVAQIDQTTKDNVEIANESAIISSTVSDIANNILEDI 646

00-6200_Tlp3b VQSINDMAESIKEQTAGITQINDSVAQIDQTTKDNVEIANESAIISSTVSDIANNILEDI 646

BCW_6290_Tlp3b VQSINDMAESIKEQTAGITQINDSVAQIDQTTKDNVEIANESAIISSTVSDIANNILEDI 646

00-2425_Tlp3a VQSINDMAESIKEQTAGITQINDSVAQIDQTTKDNVEIANESAIISSTVSDIANNILEDV 646

00-2425_Tlp3b VQSINDMAESIKEQTAGITQINDSVAQIDQTTKDNVEIANESAIISSTVSDIANNILEDI 646

YH001_Tlp3a VQSINDMAESIKEQTAGITQINDSVAQIDQTTKDNVEIANESAIISSTVSDIANNILEDI 646

YH001_Tlp3b VQSINDMAESIKEQTAGITQINDSVAQIDQTTKDNVEIANESAIISSTVSDIANNILEDI 646

00-0949_Tlp3 VQSINDMAESIKEQTAGITQINDSVAQIDQTTKDNVEIANESAIISSTVSDIANNILEDV 646

NCTC11168_Tlp3 VQSINDMAESIKEQTAGITQINDSVAQIDQTTKDNVEIANESAIISSTVSDIANNILEDV 657

F38011_Tlp3 VQSINDMAESIKEQTAGITQINDSVAQIDQTTKDNVEIANESAIISSTVSDIANNILEDV 646

RM1285_Tlp3 VQSINDMAESIKEQTAGITQINDSVAQIDQTTKDNVEIANESAIISSTVSDIANNILEDV 646

FDAARGOS_422_Tlp3 VQSINDMAESIKEQTAGITQINDSVAQIDQTTKDNVEIANESAIISSTVSDIANNILEDV 657

MTVDSCj07_Tlp3 VQSINDMAESIKEQTAGITQINDSVAQIDQTTKDNVEIANESAIISSTVSDIANNILEDV 646

IA3901_Tlp3a VQSINDMAESIKEQTAGITQINDSVAQIDQTTKDNVEIANESAIISSTVSDIANNILEDV 646

BCW_6290_Tlp3a VQSINDMAESIKEQTAGITQINDSVAQIDQTTKDNVEIANESAIISSTVSDIANNILEDV 646

CJ677CC012_Tlp3 VQSINDMAESIKEQTAGITQINDSVAQIDQTTKDNVEIANESAIISSTVSDIANNILEDV 504

CJM1cam VQSINDMAESIKEQTAGITQINESVAQIDQTTKDNVEIANESAIISSTVSDIANNILEDV 646

M1_Tlp3 VQSINDMAESIKEQTAGITQINESVAQIDQTTKDNVEIANESAIISSTVSDIANNILEDV 646

4031_Tlp3 VQSINDMAESIKEQTAGITQINESVAQIDQTTKDNVEIANESAIISSTVSDIANNILEDV 646

R14_Tlp3 VQSINDMAESIKEQTAGITQINDSVAQIDQTTKDNVEIANESAIISSTVSDIANNILEDV 646

35925B2_Tlp3 VQSINDMAESIKEQTAGITQINESVAQIDQTTKDNVEIANESAIISSTVSDIANNILEDV 660

14980A VQSINDMAESIKEQTAGITQINDSVAQIDQTTKDNVEIANESAIISSTVSDIANNILEDV 657

00-1597_Tlp3b VQSINDMAESIKEQTAGITQINDSVAQIDQTTKDNVEIANESAIISSTVSDIANNILEDV 646

ICDCCJ07001_Tlp3 VQSINDMAESIKEQTAGITQINDSVAQIDQTTKDNVEIANESAIISSTVSDIANNILEDV 646

RM3196_Tlp3 VQSINDMAESIKEQTAGITQINDSVAQIDQTTKDNVEIANESAIISSTVSDIAN------ 640

**********************:********:**************.*******

HC2-48_Tlp3 KKKRF 591

RM1875_Tlp3 KKKRF 651

CF2-75_Tlp3 KKKRF 634

RM5611_Tlp3 KKKRF 651

MTVDSCj16_Tlp3 KKKRF 651

01-1512_Tlp3 KKKRF 651

MTVDSCj13_Tlp3 KKKRF 651

32488_Tlp3a KKKRF 651

81116_Tlp3 KKKRF 651

32488_Tlp3b KKKRF 651

FB1_Tlp3 KKKRF 651

PT14_Tlp3 KKKRF 651

00-6200_Tlp3a KKKRF 651

RM1221_Tlp3 KKKRF 651

S3_Tlp3 KKKRF 651

FDAARGOS_421_Tlp3 KKKRF 662

CFSAN032806_Tlp3 KKKRF 662

IA3901_Tlp3b KKKRF 651

00-6200_Tlp3b KKKRF 651

BCW_6290_Tlp3b KKKRF 651

00-2425_Tlp3a KKKRF 651

00-2425_Tlp3b KKKRF 651

YH001_Tlp3a KKKRF 651

YH001_Tlp3b KKKRF 651

00-0949_Tlp3 KKKRF 651

NCTC11168_Tlp3 KKKRF 662

F38011_Tlp3 KKKRF 651

RM1285_Tlp3 KKKRF 651

FDAARGOS_422_Tlp3 KKKRF 662

MTVDSCj07_Tlp3 KKKRF 651

IA3901_Tlp3a KKKRF 651

BCW_6290_Tlp3a KKKRF 651

CJ677CC012_Tlp3 KKKRF 509

CJM1cam KKKRF 651

M1_Tlp3 KKKRF 651

4031_Tlp3 KKKRF 651

R14_Tlp3 KKKRF 651

35925B2_Tlp3 KKKRF 665

14980A KKKRF 662

00-1597_Tlp3b KKKRF 651

ICDCCJ07001_Tlp3 KKKR- 650

RM3196_Tlp3 ----- 640
