# Supplementary material for: Diversity of transducer-like proteins (Tlps) in Campylobacter
Source: PLoS One. 2019 Mar 25;14(3):e0214228. doi: 10.1371/journal.pone.0214228 (PMC6433261; doi:10.1371/journal.pone.0214228)
Supplement: S2 Archive — (ZIP) [file pone.0214228.s016.zip › Alignment P.docx]

Alignment P. Tlp4 protein sequence comparisons: individual isolate comparisons

CLUSTAL O(1.2.4) multiple sequence alignment 2018/04/18

ICDCCJ07001_Tlp4 MQSINSGKSVGISAKLTLWVGILVVLILAITSAISYFDSRNNTYELLKDTQLKTMQDVGA 60

RM3196_Tlp4 MQSINSGKSVGISAKLTLWVGILVVLILAITSAISYFDSRNNTYELLKDTQLKTMQDVGA 60

T1-21_Tlp4 MQSINSGKSVGISAKLTLWVGILVVLILAITSAISYFDSRNNTYELLKDTQLKTMQDVGA 60

F38011_Tlp4 MQSINSGKSAGISAKLTLWVGILVVLILAITSAVSYFDSRNNTYELLKDTQLKTMQDVGA 60

81-176_Tlp4 MQSINSGKSVGISAKLTLWVGILVVLILAITSAISYFDSRNNTYELLKDTQLKTMQDVDA 60

32488_Tlp4 MQSINSGKSVGISAKLTLWVGILVVLILAITSAISYFDSRNNTYELLKDTQLKTMQDVDA 60

NCTC11168_Tlp4 MQSINSGKSVGISAKLTLWVGILVVLILAITSAISYFDSRNNTYELLKDTQLKTMQDVDA 60

81116_Tlp4 MQSINSGKSVGISAKLTLWVGILVVLILAITSAISYFDSRNNTYELLKDTQLKTMQDVDA 60

*********.***********************:************************.*

ICDCCJ07001_Tlp4 FFESYGMSKRNGIQILANELNKRPDMSDEELINLIKAFKEVNGYDLVYVGFDNTGKNYQS 120

RM3196_Tlp4 FFESYGMSKRNGIQILANELNKRPDMSDEELINLIKAFKEVNGYDLVYVGFDNTGKNYQS 120

T1-21_Tlp4 FFESYGMSKRNGIQILANELNKRPDMSDEELINLIKAFKEVNGYDLVYVGFDNTGKNYQS 120

F38011_Tlp4 FFESYGMSKRHGIQILANELNKRPDMSDEELINLIKAFKEVNDYDLVYVGFDNTGKNYQS 120

81-176_Tlp4 FFKSYAMSKRNGIQILANELTNRPDMSDEELINLIKVIKKVNDYDLVYVGFDNTGKNYQS 120

32488_Tlp4 FFKSYAMSKRNGIQILANELTNRPDMSDEELINLIKVIKKVNDYDLVYVGFDNTGKNYQS 120

NCTC11168_Tlp4 FFKSYAMSKRNGIQILANELTNRPDMSDEELINLIKVIKKVNDYDLVYVGFDNTGKNYQS 120

81116_Tlp4 FFKSYAMSKRNGIQILANELTNRPDMSDEELINLIKVIKKVNDYDLVYVGFDNTGKNYQS 120

**:**.****:*********.:**************.:*:**.*****************

ICDCCJ07001_Tlp4 DDQILDLSKGYDTKNRPWYKAAKEAKKLIVTEPYKSANSGEVGLTYAAPFYDRNGNFRGV 180

RM3196_Tlp4 DDQILDLSKGYDTKNRPWYKAAKEAKKLIVTEPYKSANSGEVGLTYAAPFYDRNGNFRGV 180

T1-21_Tlp4 DDQILDLSKGYDTKNRPWYKAAKEAKKLIVTEPYKSANSGEVGLTYAAPFYDRNGNFRGV 180

F38011_Tlp4 DDQILDLSKGYDTKNRPWYKAAKEAKKLIVTEPYKSAASGEVGLTYAAPFYDRNGNFRGV 180

81-176_Tlp4 DDQILDLSKGYDTKNRPWYKAAKEAKKLIVTEPYKSAASGEVGLTYAAPFYDRNGNFRGV 180

32488_Tlp4 DDQILDLSKGYDTKNRPWYKAAKEAKKLIVTEPYKSAASGEVGLTYAAPFYDRNGNFRGV 180

NCTC11168_Tlp4 DDQILDLSKGYDTKNRPWYKAAKEAKKLIVTEPYKSAASGEVGLTYAAPFYDRNGNFRGV 180

81116_Tlp4 DDQILDLSKGYDTKNRPWYKAAKEAKKLIVTEPYKSAASGEVGLTYAAPFYDRNGNFRGV 180

************************************* **********************

ICDCCJ07001_Tlp4 VGGDYDLAKFSTDVLAVGKSQNTYTVVLDPEGTILFRDDITKILTKTELSINIANAIKAN 240

RM3196_Tlp4 VGGDYDLAKFSTDVLAVGKSQNTYTVVLDPEGTILFRDDITKILTKTELSINIANAIKAN 240

T1-21_Tlp4 VGGDYDLAKFSTDVLAVGKSQNTYTVVLDPEGTILFRDDITKILTKTELSINIANAIKAN 240

F38011_Tlp4 VGGDYDLANFSTNVLTVGKSDNTFTEVLDSEGTILFNDEVAKILTKTELSINIANAIKAN 240

81-176_Tlp4 VGGDYDLANFSTNVLTVGKSDNTFTEVLDSEGTILFNDEVAKILTKTELSINIANAIKAN 240

32488_Tlp4 VGGDYDLANFSTNVLTVGKSDNTFTEVLDSEGTILFNDEVAKILTKTELSINIANAIKAN 240

NCTC11168_Tlp4 VGGDYDLANFSTNVLTVGKSDNTFTEVLDSEGTILFNDEVAKILTKTELSINIANAIKAN 240

81116_Tlp4 VGGDYDLANFSTNVLTVGKSDNTFTEVLDSEGTILFNDEVAKILTKTELSINIANAIKAN 240

********:***:**:****:**:* *** ******.*:::*******************

ICDCCJ07001_Tlp4 PALIDPRNQDTLFTAKDHQGVDYAIMCNSAFNPLFRICTITENKVYTEAVNSILMKQVIV 300

RM3196_Tlp4 PALIDPRNQDTLFTAKDHQGVDYAIMCNSAFNPLFRICTITENKVYTEAVNSILMKQVIV 300

T1-21_Tlp4 PALIDPRNQDTLFTAKDHQGVDYAIMCNSAFNPLFRICTITENKVYTEAVNSILMKQVIV 300

F38011_Tlp4 PALIDPRNQDTLFTAKDHQGVDYAIMCNSAFNPLFRICTITENKVYTEAVNSILMKQVIV 300

81-176_Tlp4 PALIDPRNQDTLFTAKDHQGVDYAIMCNSAFNPLFRICTITENKVYTEAVNSILMKQVIV 300

32488_Tlp4 PALIDPRNQDTLFTAKDHQGVDYAIMCNSAFNPLFRICTITENKVYTEAVNSILMKQVIV 300

NCTC11168_Tlp4 PALIDPRNQDTLFTAKDHQGVDYAIMCNSAFNPLFRICTITENKVYTEAVNSILMKQVIV 300

81116_Tlp4 PALIDPRNQDTLFTAKDHQGVDYAIMCNSAFNPLFRICTITENKVYTEAVNSILMKQVIV 300

************************************************************

ICDCCJ07001_Tlp4 GIIAIIIALILIRFLISRSLSPLAAIQTGLTSFFDFINHKTKNVSTIEVKSNDEFGQISN 360

RM3196_Tlp4 GIIAIIIALILIRFLISRSLSPLAAIQTGLTSFFDFINHKTKNVSTIEVKSNDEFGQISN 360

T1-21_Tlp4 GIIAIIIALILIRFLISRSLSPLAAIQTGLTSFFDFINYKTKNVSTIEVKSNDEFGQISN 360

F38011_Tlp4 GIIAIIIALILIRFLISRSLSPLAAIQTGLTSFFDFINYKTKNVSTIEVKSNDEFGQISN 360

81-176_Tlp4 GIIAIIIALILIRFLISRSLSPLAAIQTGLTSFFDFINYKTKNVSTIEVKSNDEFGQISN 360

32488_Tlp4 GIIAIIIALILIRFLISRSLSPLAAIQTGLTSFFDFINYKTKNVSTIEVKSNDEFGQISN 360

NCTC11168_Tlp4 GIIAIIIALILIRFLISRSLSPLAAIQTGLTSFFDFINYKTKNVSTIEVKSNDEFGQISN 360

81116_Tlp4 GIIAIIIALILIRFLISRSLSPLAAIQTGLTSFFDFINYKTKNVSTIEVKSNDEFGQISN 360

**************************************:*********************

ICDCCJ07001_Tlp4 AINENILATKQGLEQDAKAVKESVETVGVVESGNLTARITANPRNPQLIELKNVLNRLLD 420

RM3196_Tlp4 AINENILATKQGLEQDAKAVKESVETVGVVESGNLTARITANPRNPQLIELKNVLNRLLD 420

T1-21_Tlp4 AINENILATKRGLEQDNQAVKESVQTVSVVEGGNLTARITANPRNPQLIELKNVLNKLLD 420

F38011_Tlp4 AINENILATKRGLEQDNQAVKESVQTVSVVEGGNLTARITANPRNPQLIELKNVLNKLLD 420

81-176_Tlp4 AINENILATKRGLEQDNQAVKESVQTVSVVEGGNLTARITANPRNPQLIELKNVLNKLLD 420

32488_Tlp4 AINENILATKRGLEQDNQAVKESVQTVSVVEGGNLTARITANPRNPQLIELKNVLNKLLD 420

NCTC11168_Tlp4 AINENILATKRGLEQDNQAVKESVQTVSVVEGGNLTARITANPRNPQLIELKNVLNKLLD 420

81116_Tlp4 AINENILATKRGLEQDNQAVKESVQTVSVVEGGNLTARITANPRNPQLIELKNVLNKLLD 420

**********:***** :******:**.***.************************:***

ICDCCJ07001_Tlp4 VLQTRVGSDMNAIHKIFEEYKSLDFRNKLDNANGSVEVTTNALGDEIVKMLKQSSDFANH 480

RM3196_Tlp4 VLQTRVGSDMNAIHKIFEEYKSLDFRNKLDNANGSVEVTTNALGDEIVKMLKQSSDFANH 480

T1-21_Tlp4 VLQARVGSDMNAIHKIFEEYKSLDFRNKLENASGSVELTTNALGDEIVKMLKQSSDFANA 480

F38011_Tlp4 VLQARVGSDMNAIHKIFEEYKSLDFRNKLENASGSVELTTNALGDEIVKMLKQSSDFANA 480

81-176_Tlp4 VLQARVGSDMNAIHKIFEEYKSLDFRNKLENASGSVELTTNALGDEIVKMLKQSSDFANA 480

32488_Tlp4 VLQARVGSDMNAIHKIFEEYKSLDFRNKLENASGSVELTTNALGDEIVKMLKQSSDFANA 480

NCTC11168_Tlp4 VLQARVGSDMNAIHKIFEEYKSLDFRNKLENASGSVELTTNALGDEIVKMLKQSSDFANA 480

81116_Tlp4 VLQARVGSDMNAIHKIFEEYKSLDFRNKLENAGGSVELTTNALGDEIVKMLKQSSDFANA 480

***:*************************:**.****:*********************

ICDCCJ07001_Tlp4 LASESSKLQSAVQNLTSSSNSQAASLEETAAALEEITSSMQNVSVKTSDVITQSEEIKNV 540

RM3196_Tlp4 LASESSKLQSAVQNLTSSSNSQAASLEETAAALEEITSSMQNVSVKTSDVITQSEEIKNV 540

T1-21_Tlp4 LANESGKLQTAVQSLTTSSNSQAQSLEETAAALEEITSSMQNVSVKTSDVITQSEEIKNV 540

F38011_Tlp4 LANESGKLQTAVQSLTTSSNSQAQSLEETAAALEEITSSMQNVSVKTSDVITQSEEIKNV 540

81-176_Tlp4 LANESGKLQTAVQSLTTSSNSQAQSLEETAAALEEITSSMQNVSVKTSDVITQSEEIKNV 540

32488_Tlp4 LANESGKLQTAVQSLTTSSNSQAQSLEETAAALEEITSSMQNVSVKTSDVITQSEEIKNV 540

NCTC11168_Tlp4 LANESGKLQTAVQSLTTSSNSQAQSLEETAAALEEITSSMQNVSVKTSDVITQSEEIKNV 540

81116_Tlp4 LANESGKLQTAVQSLTTSSNSQAQSLEETAAALEEITSSMQNVSVKTSDVITQSEEIKNV 540

**.**.***:***.**:****** ************************************

ICDCCJ07001_Tlp4 TGIIGDIADQINLLALNAAIEAARAGEHGRGFAVVADEVRKLAERTQKSLSEIEANTNLL 600

RM3196_Tlp4 TGIIGDIADQINLLALNAAIEAARAGEHGRGFAVVADEVRKLAERTQKSLSEIEANTNLL 600

T1-21_Tlp4 TGIIGDIADQINLLALNAAIEAARAGEHGRGFAVVADEVRKLAERTQKSLSEIEANTNLL 600

F38011_Tlp4 TGIIGDIADQINLLALNAAIEAARAGEHGRGFAVVADEVRKLAERTQKSLSEIEANTNLL 600

81-176_Tlp4 TGIIGDIADQINLLALNAAIEAARAGEHGRGFAVVADEVRKLAERTQKSLSEIEANTNLL 600

32488_Tlp4 TGIIGDIADQINLLALNAAIEAARAGEHGRGFAVVADEVRKLAERTQKSLSEIEANTNLL 600

NCTC11168_Tlp4 TGIIGDIADQINLLALNAAIEAARAGEHGRGFAVVADEVRKLAERTQKSLSEIEANTNLL 600

81116_Tlp4 TGIIGDIADQINLLALNAAIEAARAGEHGRGFAVVADEVRKLAERTQKSLSEIEANTNLL 600

************************************************************

ICDCCJ07001_Tlp4 VQSINDMAESIKEQTAGITQINDSVAQIDQTTKDNVEIANESAIISSTVSDIANNILEDV 660

RM3196_Tlp4 VQSINDMAESIKEQTAGITQINDSVAQIDQTTKDNVEIANESAIISSTVSDIANNILEDV 660

T1-21_Tlp4 VQSINDMAESIKEQTAGITQINDSVAQIDQTTKDNVEIANESAIISSTVSDIANNILEDV 660

F38011_Tlp4 VQSINDMAESIKEQTAGITQINDSVAQIDQTTKDNVEIANESAIISSTVSDIANNILEDV 660

81-176_Tlp4 VQSINDMAESIKEQTAGITQINDSVAQIDQTTKDNVEIANESAIISSTVSDIANNILEDV 660

32488_Tlp4 VQSINDMAESIKEQTAGITQINDSVAQIDQTTKDNVEIANESAIISSTVSDIANNILEDV 660

NCTC11168_Tlp4 VQSINDMAESIKEQTAGITQINDSVAQIDQTTKDNVEIANESAIISSTVSDIANNILEDV 660

81116_Tlp4 VQSINDMAESIKEQTAGITQINDSVAQIDQTTKDNVEIANESAIISSTVSDIANNILEDV 660

************************************************************

ICDCCJ07001_Tlp4 KKKRF 665

RM3196_Tlp4 KKKRF 665

T1-21_Tlp4 KKKRF 665

F38011_Tlp4 KKKRF 665

81-176_Tlp4 KKKRF 665

32488_Tlp4 KKKRF 665

NCTC11168_Tlp4 KKKRF 665

81116_Tlp4 KKKRF 665

*****
