# Supplementary material for: Diversity of transducer-like proteins (Tlps) in Campylobacter
Source: PLoS One. 2019 Mar 25;14(3):e0214228. doi: 10.1371/journal.pone.0214228 (PMC6433261; doi:10.1371/journal.pone.0214228)
Supplement: S2 Archive — (ZIP) [file pone.0214228.s016.zip › Alignment Q.docx]

Alignment Q. Tlp11 protein sequence comparisons: individual isolate comparisons

CLUSTAL O(1.2.4) multiple sequence alignment 2018/04/18

00-2425_Tlp11 MNFRSLNLSTKLILSVAIGIVLGIVVIVLTVSIYTSKSMEKEAKDSIFLSSKRYVNYMEG 60

00-6200_Tlp11 MNFRSLNLSTKLILSVAIGIVLGIVVIVLTVSIYTSKSMEKEAKDSIFLSSKRYVNYMEG 60

YH001_Tlp11 MNFRSLNLSTKLILSVAIGIVLGIVVIVLTVSIYTSKSMEKEAKDSIFLSSKRYVNYMEG 60

IA3902_Tlp11 MNFRSLNLSTKLILSVAIGIVLGIVVIVLTVSIYTSKSMEKEAKDSIFLSSKRYVNYMEG 60

BCW_6290_Tlp11 MNFRSLNLSTKLILSVAIGIVLGIVVIVLTVSIYTSKSMEKEAKDSIFLSSKRYVNYMEG 60

************************************************************

00-2425_Tlp11 ILNEEVVLTKAMATSLNEIFSKNDQVNAGIIESLLRNTFDSSGYAAYAFLYLQDSSILTH 120

00-6200_Tlp11 ILNEEVVLTKAMATSLNEIFSKNDQVNAGIIESLLRNTFDSSGYAAYAFLYLQDSSILTH 120

YH001_Tlp11 ILNEEVVLTKAMATSLNEIFSKNDQVNAGIIESLLRNTFDSSGYAAYAFLYLQDSSILTH 120

IA3902_Tlp11 ILNEEVVLTKAMATSLNEIFSKNDQVNAGIIESLLRNTFDSSGYAAYAFLYLQDSSILTH 120

BCW_6290_Tlp11 ILNEEVVLTKAMATSLNEIFSKNDQVNAGIIESLLRNTFDSSGYAAYAFLYLQDSSILTH 120

************************************************************

00-2425_Tlp11 VESLDKNFKNSDGKSVTMIFFDETTGKAGGIKSIHAPSNFSQLPIIEKIKKNARYGDLDT 180

00-6200_Tlp11 VESLDKNFKNSDGKSVTMIFFDETTGKAGGIKSIHAPSNFSQLPIIEKIKKNARYGDLDT 180

YH001_Tlp11 VESLDKNFKNSDGKSVTMIFFDETTGKAGGIKSIHAPSNFSQLPIIEKIKKNARYGDLDT 180

IA3902_Tlp11 VESLDKNFKNSDGKSVTMIFFDETTGKAGGIKSIHAPSNFSQLPIIEKIKKNARYGDLDT 180

BCW_6290_Tlp11 VESLDKNFKNSDGKSVTMIFFDETTGKAGGIKSIHAPSNFSQLPIIEKIKKNARYGDLDT 180

************************************************************

00-2425_Tlp11 IFLGSPSRLNYDGTEFLGINLGMPLFNKEGKFIGIVGFTFDFLEISETILDPKLDFYKDD 240

00-6200_Tlp11 IFLGSPSRLNYDGTEFLGINLGMPLFNKEGKFIGIVGFTFDFLEISETILDPKLDFYKDD 240

YH001_Tlp11 IFLGSPSRLNYDGTEFLGINLGMPLFNKEGKFIGIVGFTFDFLEISETILDPKLDFYKDD 240

IA3902_Tlp11 IFLGSPSRLNYDGTEFLGINLGMPLFNKEGKFIGIVGFTFDFLEISETILDPKLDFYKDD 240

BCW_6290_Tlp11 IFLGSPSRLNYDGTEFLGINLGMPLFNKEGKFIGIVGFTFDFLEISETILDPKLDFYKDD 240

************************************************************

00-2425_Tlp11 LRFLITDQGVIVIHKNKDAILKTLPEINQDASVQLIIDAVKNHKDLIIDNYVDLSGNLSY 300

00-6200_Tlp11 LRFLITDQGVIVIHKNKDAILKTLPEINQDASVQLIIDAVKNHKDLIIDNYVDLSGNLSY 300

YH001_Tlp11 LRFLITDQGVIVIHKNKDAILKTLPEINQDASVQLIIDAVKNHKDLIIDNYVDLSGNLSY 300

IA3902_Tlp11 LRFLITDQGVIVIHKNKDAILKTLPEINQDASVQLIIDAVKNHKDLIIDNYVDLSGNLSY 300

BCW_6290_Tlp11 LRFLITDQGVIVIHKNKDAILKTLPEINQDASVQLIIDAVKNHKDLIIDNYVDLSGNLSY 300

************************************************************

00-2425_Tlp11 AGVASFSTLGDSSHWSMVVTAPKKSIFAPLYELNFILISIAIIVLIAILIILYFCVKNIV 360

00-6200_Tlp11 AGVASFSTLGDSSHWSMVVTAPKKSIFAPLYELNFILISIAIIVLIAILIILYFCVKNIV 360

YH001_Tlp11 AGVASFSTLGDSSHWSMVVTAPKKSIFAPLYELNFILISIAIIVLIAILIILYFCVKNIV 360

IA3902_Tlp11 AGVASFSTLGDSSHWSMVVTAPKKSIFAPLYELNFILISIAIIVLIAILIILYFCVKNIV 360

BCW_6290_Tlp11 AGVASFSTLGDSSHWSMVVTAPKKSIFAPLYELNFILISIAIIVLIAILIILYFCVKNIV 360

************************************************************

00-2425_Tlp11 GSKLPIIVNSLQNFFDFINHKTKNVSTIEVKSNDELGQMGKIINENILATKRGLEQDNQA 420

00-6200_Tlp11 GSKLPIIVNSLQNFFDFINHKTKNVSTIEVKSNDELGQMGKIINENILATKRGLEQDNQA 420

YH001_Tlp11 GSKLPIIVNSLQNFFDFINHKTKNVSTIEVKSNDELGQMGKIINENILATKRGLEQDNQA 420

IA3902_Tlp11 GSKLPIIVNSLQNFFDFINHKTKNVSTIEVKSNDELGQMGKIINENILATKRGLEQDNQA 420

BCW_6290_Tlp11 GSKLPIIVNSLQNFFDFINHKTKNVSTIEVKSNDELGQMGKIINENILATKRGLEQDNQA 420

************************************************************

00-2425_Tlp11 VKESVQTVSVVEGGNLTARITANPRNPQLIELKNVLNKLLDVLQARVGSDMNAIHKIFEE 480

00-6200_Tlp11 VKESVQTVSVVEGGNLTARITANPRNPQLIELKNVLNKLLDVLQARVGSDMNAIHKIFEE 480

YH001_Tlp11 VKESVQTVSVVEGGNLTARITANPRNPQLIELKNVLNKLLDVLQARVGSDMNAIHKIFEE 480

IA3902_Tlp11 VKESVETVHVVEGGNLTARITANPRNPQLIELKNVLNRLLDALQARVGSDMNEIQRVFNS 480

BCW_6290_Tlp11 VKESVETVHVVEGGNLTARITANPRNPQLIELKNVLNRLLDALQARVGSDMNEIQRVFNS 480

*****:** ****************************:***.********** *:::*:.

00-2425_Tlp11 YKSLDFRNKLENASGSVELTTNALGDEIVKMLKQSSDFANALANESGKLQTAVQSLTTSS 540

00-6200_Tlp11 YKSLDFRNKLENASGSVELTTNALGDEIVKMLKQSSDFANALANESGKLQTAVQSLTTSS 540

YH001_Tlp11 YKSLDFRNKLENASGSVELTTNALGDEIVKMLKQSSDFANALANESGKLQTAVQSLTTSS 540

IA3902_Tlp11 YKSLDFTTEVKDANGAVELTTNALGDEIVKMLKQSSDFANALANESGKLQTAVQSLTTSS 540

BCW_6290_Tlp11 YKSLDFTTEVKDANGAVELTTNALGDEIVKMLKQSSDFANALANESGKLQTAVQSLTTSS 540

****** .::::*.*:********************************************

00-2425_Tlp11 NSQAQSLEETAAALEEITSSMQNVSVKTSDVITQSEEIKNVTGIIGDIADQINLLALNAA 600

00-6200_Tlp11 NSQAQSLEETAAALEEITSSMQNVSVKTSDVITQSEEIKNVTGIIGDIADQINLLALNAA 600

YH001_Tlp11 NSQAQSLEETAAALEEITSSMQNVSVKTSDVITQSEEIKNVTGIIGDIADQINLLALNAA 600

IA3902_Tlp11 NSQAQSLEETAAALEEITSSMQNVSVKTSDVITQSEEIKNVTGIIGDIADQINLLALNAA 600

BCW_6290_Tlp11 NSQAQSLEETAAALEEITSSMQNVSVKTSDVITQSEEIKNVTGIIGDIADQINLLALNAA 600

************************************************************

00-2425_Tlp11 IEAARAGEHGRGFAVVADEVRKLAERTQKSLSEIEANTNLLVQSINDMAESIKEQTAGIT 660

00-6200_Tlp11 IEAARAGEHGRGFAVVADEVRKLAERTQKSLSEIEANTNLLVQSINDMAESIKEQTAGIT 660

YH001_Tlp11 IEAARAGEHGRGFAVVADEVRKLAERTQKSLSEIEANTNLLVQSINDMAESIKEQTAGIT 660

IA3902_Tlp11 IEAARAGEHGRGFAVVADEVRKLAERTQKSLSEIEANTNLLVQSINDMAESIKEQTAGIT 660

BCW_6290_Tlp11 IEAARAGEHGRGFAVVADEVRKLAERTQKSLSEIEANTNLLVQSINDMAESIKEQTAGIT 660

************************************************************

00-2425_Tlp11 QINDSVAQIDQTTKDNVEIANESAIISSTVSDIANNILEDVKKKRF 706

00-6200_Tlp11 QINDSVAQIDQTTKDNVEIANESAIISSTVSDIANNILEDVKKKRF 706

YH001_Tlp11 QINDSVAQIDQTTKDNVEIANESAIISSTVSDIANNILEDVKKKRF 706

IA3902_Tlp11 QINDSVAQIDQTTKDNVEIANESAIISSTVSDIANNILEDVKKKRF 706

BCW_6290_Tlp11 QINDSVAQIDQTTKDNVEIANESAIISSTVSDIANNILEDVKKKRF 706

**********************************************
