# Supplementary material for: Diversity of transducer-like proteins (Tlps) in Campylobacter
Source: PLoS One. 2019 Mar 25;14(3):e0214228. doi: 10.1371/journal.pone.0214228 (PMC6433261; doi:10.1371/journal.pone.0214228)
Supplement: S2 Archive — (ZIP) [file pone.0214228.s016.zip › Alignment R.docx]

Alignment R. Tlp12 protein sequence comparisons: individual isolate comparisons

CLUSTAL O(1.2.4) multiple sequence alignment Clustal Omega 2019/01/30

RM1285_Tlp12 MQSINSGKSVGISAKLTLWVGILVVLILAITSTVSYFDAKNHTYELLKENQLKTMDDVKV 60

PT14_Tlp12 MQSINSGKSVGISAKLTLWVGILVVLILAITSTVSYFDAKNHTYELLKENQLKTMDDVKV 60

MTVJDCj07_Tlp12 MQSINSGKSVGISAKLTLWVGILVVLILAITSTVSYFDAKNHTYELLKENQLKTMDDVKV 60

RM1221_Tlp12 MQKMDSGKSVGVSVKLTLWVGILVVLILAITSTVSYFDAKNHTYELLKENQLKTMDDVKV 60

FDAARGOS_421_Tlp12 MQKMDSGKSVGVSVKLTLWVGILVVLILAITSTVSYFDAKNHTYELLKENQLKTMDDVKV 60

A17_Tlp12 MQKMNSGKSVGVSVKLTLWVGILVVLILAITSTVSYFDAKNHTYELLKENQLKTMDDVKV 60

35925B2_Tlp12 MQKMNSGKSVGISAKLTLWVGILVVLILAITSAVSYFDAKNHTYELLKENQLKTMNDVKV 60

CJM1cam_Tlp12 MQKMNSGKSVGISAKLTLWVGILVVLILAITSAVSYFDAKNHTYELLKENQLKTMDDVKV 60

M1_Tlp12 MQKMNSGKSVGISAKLTLWVGILVVLILAITSAVSYFDAKNHTYELLKENQLKTMDDVKV 60

S3_Tlp12 MQKMDSGKSVGVSVKLTLWVGILVVLILAITSTVSYFDAKNHTYELLKENQLKTMDDVKV 60

00-1597_Tlp12 MQSINSGKSVGVSVKLTLWVGILVVLILAITSTVSYFDAKNHTYELLKENQLKTMDDVKV 60

R14_Tlp12 MQSINSGKSVGISAKLTLWVGILVVLILAITSTVSYFDAKNHTYELLKENQLKTMDDVKV 60

**.::******:*.******************:**********************:****

RM1285_Tlp12 TFENYSKSKQKAIEVLAYESAKKLEDENISLLLDSFKKAFDFDIVFIAFDKNNKMLLSNG 120

PT14_Tlp12 TFENYSKSKQKAIEVLAYESAKKLEDENISLLLDSFKKAFDFDIVFIAFDKNNKMLLSNG 120

MTVJDCj07_Tlp12 TFENYSKSKQKAIEVLAYESAKKLEDENISLLLDSFKKAFDFDIVFIAFDKNNKMLLSNG 120

RM1221_Tlp12 TFENYSKSKQKAIEVLAYESAKKLEDENISLLLDSFKKAFDFDIVFIAFDKNNKMLLSNG 120

FDAARGOS_421_Tlp12 TFENYSKSKQKAIEVLAYESAKKLEDENISLLLDSFKKAFDFDIVFIAFDKNNKMLLSNG 120

A17_Tlp12 TFENYSKSKQKAIEVLAYESAKKLEDENISLLLDSFKKAFDFDIVFIAFDKNNKMLLSNG 120

35925B2_Tlp12 TFENYSKSKQKAIEVLAYESAKKLEDENISLLLDSFKKAFDFDIVFIAFEKNNKMLLSNG 120

CJM1cam_Tlp12 TFENYSKSKQKAIEVLAYESAKKLEDENISLLLDSFKKAFDFDIVFIAFDKNNKMLLSNG 120

M1_Tlp12 TFENYSKSKQKAIEVLAYESAKKLEDENISLLLDSFKKAFDFDIVFIAFDKNNKMLLSNG 120

S3_Tlp12 TFENYSKSKQKAIEVLAYESAKKLEDENISLLLDSFKKAFDFDIVFIAFDKNNKMLLSNG 120

00-1597_Tlp12 TFENYSKSKQKAIEVLAYESAKKLEDENISLLLDSFKKAFDFDIVFIAFDKNNKMLLSNG 120

R14_Tlp12 TFENYSKSKQKAIEVLAYESAKKLEDENISLLLDSFKKAFDFDIVFIAFDKNNKMLLSNG 120

*************************************************:**********

RM1285_Tlp12 TILDKKSNFDITKQIWYQEAKNNKGITITQPYKSPIDQEIGITYVFPIYKNNQLIAFVGG 180

PT14_Tlp12 TILDKKSNFDITKQIWYQEAKNNKGITITQPYKSPIDQEIGITYVFPIYKNNQLIAFVGG 180

MTVJDCj07_Tlp12 TILDKKSNFDITKQIWYQEAKNNKGITITQPYKSPIDQEIGITYVFPIYKNNQLIAFVGG 180

RM1221_Tlp12 TILDKKSNFDITKQIWYQEAKNNKGITITQPYKSPIDQEIGITYVFPIYKNNQLIAFVGG 180

FDAARGOS_421_Tlp12 TILDKKSNFDITKQIWYQEAKNNKGITITQPYKSPIDQEIGITYVFPIYKNNQLIAFVGG 180

A17_Tlp12 TILDKKSNFDITKQIWYQEAKNNKGITITQPYKSPIDQEIGITYVFPIYKNNQLIAFVGG 180

35925B2_Tlp12 TILDKKSNFDITKQIWYQEAKNNKGITITQPYKSPIDQEIGITYVFPIYKNNQLIAFVGG 180

CJM1cam_Tlp12 TILDKKSNFDITKQIWYQEAKNNKGITITQPYKSPIDQEIGITYVFPIYKNNQLIAFVGG 180

M1_Tlp12 TILDKKSNFDITKQIWYQEAKNNKGITITQPYKSPIDQEIGITYVFPIYKNNQLIAFVGG 180

S3_Tlp12 TILDKKSNFDITKQIWYQEAKNNKGITITQPYKSPIDQEIGITYVFPIYKNNQLIAFVGG 180

00-1597_Tlp12 TILDKKSNFDITKQIWYQEAKNNKGITITQPYKSPIDQEIGITYVFPIYKNNQLIAFVGG 180

R14_Tlp12 TILDKKSNFDITKQIWYQEAKNNKGITITQPYKSPIDQEIGITYVFPIYKNNQLIAFVGG 180

************************************************************

RM1285_Tlp12 DYNLDKFSKDVLSLGHSSTTYAAVYDSEGRIIFHEVLDRILTKNTLSVNIANAIKENPEY 240

PT14_Tlp12 DYNLDKFSKDVLSLGHSSTTYAAVYDSEGRIIFHEVLDRILTKNTLSVNIANAIKENPEY 240

MTVJDCj07_Tlp12 DYNLDKFSKDVLSLGHSSTTYAAVYDSEGRIIFHEVLDRILTKNTLSVNIANAIKENPEY 240

RM1221_Tlp12 DYNLDKFSKDVLSLGHSSTTYAAVYDSEGRIIFHEVLDRILTKNTLSVNIANAIKENPEY 240

FDAARGOS_421_Tlp12 DYNLDKFSKDVLSLGHSSTTYAAVYDSEGRIIFHEVLDRILTKNTLSVNIANAIKENPEY 240

A17_Tlp12 DYNLDKFSKDVLSLGHSSTTYAAVYDSEGRIIFHEVLDRILTKNTLSVNIANAIKENPEY 240

35925B2_Tlp12 DYNLDKFSKDVLSLGHSSTTYAAVYDSEGRIIFHEVLDRILTKNTLSINIANAIKENPKY 240

CJM1cam_Tlp12 DYNLDKFSKDVLSLGHSSTTYAAVYDSEGRIIFHEVLDRILTKNTLSVNIANTIKENPEY 240

M1_Tlp12 DYNLDKFSKDVLSLGHSSTTYAAVYDSEGRIIFHEVLDRILTKNTLSVNIANTIKENPEY 240

S3_Tlp12 DYNLDKFSKDVLSLGHSSTTYAAVYDSEGRIIFHEVLDRILTKNTLSVNIANAIKENPEY 240

00-1597_Tlp12 DYNLDKFSKDVLSLGHSSTTYAAVYDSEGRIIFHEVLDRILTKNTLSVNIANAIKENPEY 240

R14_Tlp12 DYNLDKFSKDVLSLGHSSTTYAAVYDSEGRIIFHEVLDRILTKNTLSVNIANAIKENPEY 240

***********************************************:****:*****:*

RM1285_Tlp12 IDLNKRDILFPVFDDKGIKYEAMCDTSSNGLYRICAVTLDSNYTSAVNSILMKQVIVGII 300

PT14_Tlp12 IDLNKRDILFPVFDDKGIKYEAMCDTSSNGLYRICAVTLDSNYTSAVNSILMKQVIVGII 300

MTVJDCj07_Tlp12 IDLNKRDILFPVFDDKGIKYEAMCDTSSNGLYRICAVTLDSNYTSAVNSILMKQVIVGII 300

RM1221_Tlp12 IDPNKRDILFPVFDDKGIKYETMCDTSSNGLYRICAVTLDSNYTSAVNSILMKQVIVGII 300

FDAARGOS_421_Tlp12 IDPNKRDILFPVFDDKGIKYETMCDTSSNGLYRICAVTLDSNYTSAVNSILMKQVIVGII 300

A17_Tlp12 IDPNKRDILFPVFDDKGIKYETMCDTSSNGLYRICAVTLDSNYTSAVNSILMKQVIVGII 300

35925B2_Tlp12 IDLNKRDILFPVFDDKGIKYETMCDTSSNGLYRICAVTLDSNYTSAVNSILMKQVIVGII 300

CJM1cam_Tlp12 IDLNKRDILFPVFDDKGIKYEAMCDTSSNGLYRICAVTLDSNYTSAVNSILMKQVIVGII 300

M1_Tlp12 IDLNKRDILFPVFDDKGIKYEAMCDTSSNGLYRICAVTLDSNYTSAVNSILMKQVIVGII 300

S3_Tlp12 IDPNKRDILFPVFDDKGIKYETMCDTSSNGLYRICAVTLDSNYTSAVNSILMKQAIVGII 300

00-1597_Tlp12 IDPNKRDILFPVFDDKGIKYETMCDTSSNGLYRICAVTLDSNYTSAVNSILMKQAIVGII 300

R14_Tlp12 IDPNKRDILFPVFDDKGIKYETMCDTSSNGLYRICAVTLDSNYTSAVNSILMKQVIVGII 300

** ******************:********************************.*****

RM1285_Tlp12 AIIIALILIRFLISRSLSPLAAIQTGLTSFFDFINYKTKNVSTIEVKSNDEFGQISNAIN 360

PT14_Tlp12 AIIIALILIRFLISRSLSPLAAIQTGLTSFFDFINYKTKNVSTIEVKSNDEFGQISNAIN 360

MTVJDCj07_Tlp12 AIIIALILIRFLISRSLSPLAAIQTGLTSFFDFINYKTKNVSTIEVKSNDEFGQISNAIN 360

RM1221_Tlp12 AIIIALILIRFLISRSLSPLAAIQTGLTSFFDFINYKTKNVSTIEVKSNDEFGQISNAIN 360

FDAARGOS_421_Tlp12 AIIIALILIRFLISRSLSPLAAIQTGLTSFFDFINYKTKNVSTIEVKSNDEFGQISNAIN 360

A17_Tlp12 AIIIALILIRFLISRSLSPLAAIQTGLTSFFDFINYKTKNVSTIEVKSNDEFGQISNAIN 360

35925B2_Tlp12 AIIIALILIRFLISRSLSPLAAIQTGLTSFFDFINHKTKNVSTIEVKSNDEFGQISSAIN 360

CJM1cam_Tlp12 AIIIALILIRFLISRSLSPLAAIQTGLTSFFDFINYKTKNVSTIEVKSNDEFGQISNAIN 360

M1_Tlp12 AIIIALILIRFLISRSLSPLAAIQTGLTSFFDFINYKTKNVSTIEVKSNDEFGQISNAIN 360

S3_Tlp12 AIIIALILIRFLISRSLSPLAAIQTGLTSFFDFINYKTKNVSTIEVKSNDEFGQISNAIN 360

00-1597_Tlp12 AIIIALILIRFLISRSLSPLAAIQTGLTSFFDFINYKTKNVSTIEVKSNDEFGQISNAIN 360

R14_Tlp12 AIIIALILIRFLISRSLSPLAAIQTGLTSFFDFINYKTKNVSTIEVKSNDEFGQISNAIN 360

***********************************:********************.***

RM1285_Tlp12 KTFLLLK-EAEQDNQAVKESVQTVSVVEGGNLTARITANPRNPQLIELKNVLNKLLDVLQ 419

PT14_Tlp12 ENILATKRGLEQDNQAVKESVQTVSVVEGGNLTARITANPRNPQLIELKNVLNKLLDVLQ 420

MTVJDCj07_Tlp12 ENILATKRGLEQDNQAVKESVQTVSVVEGGNLTARITANPRNPQLIELKNVLNKLLDVLQ 420

RM1221_Tlp12 ENILATKRGLEQDNQAVKESVQTVSVVEGGNLTARITANPRNPQLIELKNVLNKLLDVLQ 420

FDAARGOS_421_Tlp12 ENILATKRGLEQDNQAVKESVQTVSVVEGGNLTARITANPRNPQLIELKNVLNKLLDVLQ 420

A17_Tlp12 ENILATKRGLEQDNQAVKESVQTVSVVEGGNLTARITANPRNPQLIELKNVLNKLLDVLQ 420

35925B2_Tlp12 ENILQTKKGLEQDNQAVKESVETVSVVESGNLTARITANPRNPQLIELKNVLNRLLDALQ 420

CJM1cam_Tlp12 ENILATKRGLEQDNQAVKESVQTVSVVEGGNLTARITANPRNPQLIELKNVLNRLLDALQ 420

M1_Tlp12 ENILATKRGLEQDNQAVKESVQTVSVVEGGNLTARITANPRNPQLIELKNVLNRLLDALQ 420

S3_Tlp12 ENILATKRGLEQDNQAVKESVQTVSVVEGGNLTARITANPRNPQLIELKNVLNRLLDALQ 420

00-1597_Tlp12 ENILATKRGLEQDNQAVKESVQTVSVVEGGNLTARITANPRNPQLIELKNVLNRLLDALQ 420

R14_Tlp12 ENILATKRGLEQDNQAVKESVQTVSVVEGGNLTARITANPRNPQLIELKNVLNRLLDALQ 420

:.:* * ***********:******.************************:***.**

RM1285_Tlp12 ARVGSDMNAIHKIFEEYKSLDFRNKLENASGSVELTTNALGDEIVKMLKQSSDFANALAN 479

PT14_Tlp12 ARVGSDMNAIHKIFEEYKSLDFRNKLENASGSVELTTNALGDEIVKMLKQSSDFANALAN 480

MTVJDCj07_Tlp12 ARVGSDMNAIHKIFEEYKSLDFRNKLENASGSVELTTNALGDEIVKMLKQSSDFANALAN 480

RM1221_Tlp12 ARVGSDMNAIHKIFEEYKSLDFRNKLENASGSVELTTNALGDEIVKMLKQSSDFANALAN 480

FDAARGOS_421_Tlp12 ARVGSDMNAIHKIFEEYKSLDFRNKLENASGSVELTTNALGDEIVKMLKQSSDFANALAN 480

A17_Tlp12 ARVGSDMNAIHKIFEEYKSLDFRNKLENASGSVELTTNALGDEIVKMLKQSSDFANALAN 480

35925B2_Tlp12 TRVGSDMNEIQRVFNSYKSLDFTTEVKDANGAVEVTTNALGQEIIKMLKQSSDFANALAN 480

CJM1cam_Tlp12 ARVGSDMNEIQRVFNSYKSLDFTTEVKDANGAVEVTTNALGQEIIKMLKQSSDFANALAN 480

M1_Tlp12 ARVGSDMNEIQRVFNSYKSLDFTTEVKDANGAVEVTTNALGQEIIKMLKQSSDFANALAN 480

S3_Tlp12 ARVGSDMNEIQRVFNSYKSLDFTTEVKDANGAVEVTTNALGQEIIKMLKQSSDFANALAN 480

00-1597_Tlp12 ARVGSDMNEIQRVFNSYKSLDFTTEVKDANGAVEVTTNALGQEIIKMLKQSSDFANALAN 480

R14_Tlp12 ARVGSDMNEIQRVFNSYKSLDFTTEVKDANGAVEVTTNALGQEIIKMLKQSSDFANALAN 480

:******* *:::*:.****** .::::*.*:**:******:**:***************

RM1285_Tlp12 ESGKLQTAVQSLTTSSNSQAQSLEETAAALEEITSSMQNVSVKTSDVITQSEEIKNVTGI 539

PT14_Tlp12 ESGKLQTAVQSLTTSSNSQAQSLEETAAALEEITSSMQNVSVKTSDVITQSEEIKNVTGI 540

MTVJDCj07_Tlp12 ESGKLQTAVQSLTTSSNSQAQSLEETAAALEEITSSMQNVSVKTSDVITQSEEIKNVTGI 540

RM1221_Tlp12 ESGKLQTAVQSLTTSSNSQAQSLEETAAALEEITSSMQNVSVKTSDVITQSEEIKNVTGI 540

FDAARGOS_421_Tlp12 ESGKLQTAVQSLTTSSNSQAQSLEETAAALEEITSSMQNVSVKTSDVITQSEEIKNVTGI 540

A17_Tlp12 ESGKLQTAVQSLTTSSNSQAQSLEETAAALEEITSSMQNVSVKTSDVITQSEEIKNVTGI 540

35925B2_Tlp12 ESGKLQTAVQSLTTSSNSQAQSLEETAAALEEITSSMQNVSVKTSDVITQSEEIKNVTGI 540

CJM1cam_Tlp12 ESGKLQTAVQSLTTSSNSQAQSLEETAAALEEITSSMQNVSVKTSDVITQSEEIKNVTGI 540

M1_Tlp12 ESGKLQTAVQSLTTSSNSQAQSLEETAAALEEITSSMQNVSVKTSDVITQSEEIKNVTGI 540

S3_Tlp12 ESGKLQTAVQSLTTSSNSQAQSLEETAAALEEITSSMQNVSVKTSDVITQSEEIKNVTGI 540

00-1597_Tlp12 ESGKLQTAVQSLTTSSNSQAQSLEETAAALEEITSSMQNVSVKTSDVITQSEEIKNVTGI 540

R14_Tlp12 ESGKLQTAVQSLTTSSNSQAQSLEETAAALEEITSSMQNVSVKTSDVITQSEEIKNVTGI 540

************************************************************

RM1285_Tlp12 IGDIADQINLLALNAAIEAARAGEHGRGFAVVADEVRKLAERTQKSLSEIEANTNLLVQS 599

PT14_Tlp12 IGDIADQINLLALNAAIEAARAGEHGRGFAVVADEVRKLAERTQKSLSEIEANTNLLVQS 600

MTVJDCj07_Tlp12 IGDIADQINLLALNAAIEAARAGEHGRGFAVVADEVRKLAERTQKSLSEIEANTNLLVQS 600

RM1221_Tlp12 IGDIADQINLLALNAAIEAARAGEHGRGFAVVADEVRKLAERTQKSLSEIEANTNLLVQS 600

FDAARGOS_421_Tlp12 IGDIADQINLLALNAAIEAARAGEHGRGFAVVADEVRKLAERTQKSLSEIEANTNLLVQS 600

A17_Tlp12 IGDIADQINLLALNAAIEAARAGEHGRGFAVVADEVRKLAERTQKSLSEIEANTNLLVQS 600

35925B2_Tlp12 IGDIADQINLLALNAAIEAARAGEHGRGFAVVADEVRKLAERTQKSLSEIEANTNLLVQS 600

CJM1cam_Tlp12 IGDIADQINLLALNAAIEAARAGEHGRGFAVVADEVRKLAERTQKSLSEIEANTNLLVQS 600

M1_Tlp12 IGDIADQINLLALNAAIEAARAGEHGRGFAVVADEVRKLAERTQKSLSEIEANTNLLVQS 600

S3_Tlp12 IGDIADQINLLALNAAIEAARAGEHGRGFAVVADEVRKLAERTQKSLSEIEANTNLLVQS 600

00-1597_Tlp12 IGDIADQINLLALNAAIEAARAGEHGRGFAVVADEVRKLAERTQKSLSEIEANTNLLVQS 600

R14_Tlp12 IGDIADQINLLALNAAIEAARAGEHGRGFAVVADEVRKLAERTQKSLSEIEANTNLLVQS 600

************************************************************

RM1285_Tlp12 INDMAESIKEQTAGITQINDSVAQIDQTTKDNVEIANESAIISSTVSDIANNILEDVKKK 659

PT14_Tlp12 INDMAESIKEQTAGITQINDSVAQIDQTTKDNVEIANESAIISSTVSDIANNILEDVKKK 660

MTVJDCj07_Tlp12 INDMAESIKEQTAGITQINDSVAQIDQTTKDNVEIANESAIISSTVSDIANNILEDVKKK 660

RM1221_Tlp12 INDMAESIKEQTAGITQINDSVAQIDQTTKDNVEIANESAIISSTVSDIANNILEDVKKK 660

FDAARGOS_421_Tlp12 INDMAESIKEQTAGITQINDSVAQIDQTTKDNVEIANESAIISSTVSDIANNILEDVKKK 660

A17_Tlp12 INDMAESIKEQTAGITQINDSVAQIDQTTKDNVEIANESAIISNTVSDIANNILEDVKKK 660

35925B2_Tlp12 INDMAESIKEQTAGITQINESVAQIDQTTKDNVEIANESAIISSTVSDIANNILEDVKKK 660

CJM1cam_Tlp12 INDMAESIKEQTAGITQINDSVAQIDQTTKDNVEIANESAIISSTVSDIANNILEDVKKK 660

M1_Tlp12 INDMAESIKEQTAGITQINDSVAQIDQTTKDNVEIANESAIISSTVSDIANNILEDVKKK 660

S3_Tlp12 INDMAESIKEQTAGITQINDSVAQIDQTTKDNVEIANESAIISSTVSDIANNILEDVKKK 660

00-1597_Tlp12 INDMAESIKEQTAGITQINDSVAQIDQTTKDNVEIANESAIISSTVSDIANNILEDVKKK 660

R14_Tlp12 INDMAESIKEQTAGITQINDSVAQIDQTTKDNVEIANESAIISSTVSDIANNILEDVKKK 660

*******************:***********************.****************

RM1285_Tlp12 RF 661

PT14_Tlp12 RF 662

MTVJDCj07_Tlp12 RF 662

RM1221_Tlp12 RF 662

FDAARGOS_421_Tlp12 RF 662

A17_Tlp12 RF 662

35925B2_Tlp12 RF 662

CJM1cam_Tlp12 RF 662

M1_Tlp12 RF 662

S3_Tlp12 RF 662

00-1597_Tlp12 RF 662

R14_Tlp12 RF 662

**
