# Supplementary material for: Diversity of transducer-like proteins (Tlps) in Campylobacter
Source: PLoS One. 2019 Mar 25;14(3):e0214228. doi: 10.1371/journal.pone.0214228 (PMC6433261; doi:10.1371/journal.pone.0214228)
Supplement: S2 Archive — (ZIP) [file pone.0214228.s016.zip › Alignment S.docx]

Alignment S. Tlp13 protein sequence comparisons: individual isolate comparisons

CLUSTAL O(1.2.4) multiple sequence alignment

MTVDSCj13_Tlp13 MFRLSSVSSKLLLSVAISIIVAIALIIAIVSFQVASYSEKEARNTILLSSKRYVNYIQGI 60

RM1875_Tlp13 MFRLSSVSSKLLLSVAISIIVAIALIIAIVSFQVASYSEKEAKNAILLSSKRYVNYIQGI 60

CF2-75_Tlp13 MFRLSSVSSKLLLSVAISIIVAIALIIAIVSFQVASYSEKEAKNAILLSSKRYVNYIQGI 60

15-537360_Tlp13 MFRLSSVSSKLLLSVAISVILATALMIAIVSFQVASYSEKEARNTILLSSKRYVNYIQGM 60

CVM_N29710_Tlp13 MFRLSSVSSKLLLSVAISVILATALMIAIVSFQVASYSEKEARNTILLSSKRYVNYIQGM 60

FB1_Tlp13 MFRLSSVSSKLLLSVAISVILATALMIAIVSFQVASYSEKEARNTILLSSKRYVNYIQGM 60

CFCAN032805_Tlp13 MFRLSSVSSKLLLSVAISVILATALMIAIVSFQVASYSEKEARNTILLSSKRYVNYIQGM 60

BG2108_Tlp13 MFRLSSVSSKLLLSVAISVILATALMIAIVSFQVASYSEKEARNTILLSSKRYVNYIQGM 60

YF2105_Tlp13 MFRLSSVSSKLLLSVAISVILATALMIAIVSFQVASYSEKEARNTILLSSKRYVNYIQGM 60

YH503_Tlp13 MFRLSSVSSKLLLSVAISVILATALMIAIVSFQVASYSEKEARNTILLSSKRYVNYIQGM 60

BFRCA9557_Tlp13 MFRLSSVSSKLLLSVAISVILATALMIAIVSFQVASYSEKEARNTILLSSKRYVNYIQGM 60

YH502_Tlp13 MFRLSSVSSKLLLSVAISVILATALMIAIVSFQVASYSEKEARNTILLSSKRYVNYIQGM 60

OR12_Tlp13 MFRLSSVSSKLLLSVAISVILAIALMIAIVSFQVASYSEKEAKDTILLSSKRYVNYIQGM 60

00-1597_Tlp13 MFRLSSVSSKLLLSVAISVILATALMIAIVSFQVASYSEKEAKDTIFLSSKRYVNYIQGM 60

14903A MFRLSSVSSKLLLSVAISVILATALMIAIVSFQVASYSEKEAKDTIFLSSKRYVNYIQGM 60

FJ3124_Tlp13 MFRLSSVSSKLLLSVAISVILATALMIAIVSFQVASYSEKEAKDTIFLSSKRYVNYIQGM 60

R14_Tlp13 MFRLSSVSSKLLLSVAISVIVAIALMIAIVSFQVASYSEKEAKDTIFLSSKRYVNYIQGI 60

MTVDSCj16_Tlp13 MFRLSSVSSKLLLSVAISVIVAIALMIAIVSFQVASYSEKEAKDTIFLSSKRYVNYIQGI 60

14980A MFRLSSVSSKLLLSVAISVIVATALMIAIVSFQVASYSEKEAKDTIFLSSKRYVNYIQGI 60

******************:*:* **:****************:::*:************:

MTVDSCj13_Tlp13 LNEEVTLTKVVATSLNEMFQNNDHVDINLIESLIKNAFDSSHYAAYTFLYLKDTTVLSDM 120

RM1875_Tlp13 LNEEVTLTKVVATSLNEMFQNNDHVDIDLIESLIKNAFDSSHYAAYTFLYLKDTTVLSDM 120

CF2-75_Tlp13 LNEEVTLTKVVATSLNEMFQNNDHVDINLIESLIKNAFDSSHYAAYTFLYLKDTTVLSDM 120

15-537360_Tlp13 LNEEVTLTKGVATSLNEMFQNNDHIDIDLIESLIKNTFDSSHYAAYTFLYLKDTTVLSDM 120

CVM_N29710_Tlp13 LNEEVTLTKGVATSLNEMFQNNDHIDIDLIESLIKNTFDSSHYAAYTFLYLKDTTVLSDM 120

FB1_Tlp13 LNEEVTLTKGVATSLNEMFQNNDHIDIDLIESLIKNTFDSSHYAAYTFLYLKDTTVLSDM 120

CFCAN032805_Tlp13 LNEEVTLTKGVATSLNEMFQNNDHIDIDLIESLIKNTFDSSHYAAYTFLYLKDTTVLSDM 120

BG2108_Tlp13 LNEEVTLTKGVATSLNEMFQNNDHIDIDLIESLIKNTFDSSHYAAYTFLYLKDTTVLSDM 120

YF2105_Tlp13 LNEEVTLTKGVATSLNEMFQNNDHIDIDLIESLIKNTFDSSHYAAYTFLYLKDTTVLSDM 120

YH503_Tlp13 LNEEVTLTKGVATSLNEMFQNNDHIDIDLIESLIKNTFDSSHYAAYTFLYLKDTTVLSDM 120

BFRCA9557_Tlp13 LNEEVTLTKGVATSLNEMFQNNDHIDIDLIESLIKNTFDSSHYAAYTFLYLKDTTVLSDM 120

YH502_Tlp13 LNEEVTLTKGVATSLNEMFQNNDHIDIDLIESLIKNTFDSSHYAAYTFLYLKDTTVLSDM 120

OR12_Tlp13 LNEEVTLTKGVATSLNEMFQNNDHIDIDLIESLIKNTFDSSHYAAYTFLYLKDTTVLSDM 120

00-1597_Tlp13 LNEEVTLTKGVATSLNEMFQNNDHIDIDLIESLIKNTFDSSHYAAYTFLYLKDTTVLSDM 120

14903A LNEEVTLTKGVATSLNEMFQNNDHIDIDLIESLIKNTFDSSHYAAYTFLYLKDTTVLSDM 120

FJ3124_Tlp13 LNEEVTLTKGVATSLNEMFQNNDHIDIDLIESLIKNTFDSSHYAAYTFLYLKDTTVLSDM 120

R14_Tlp13 LNEEVTLTKGVATSLNEMFQNNDHVDIDLIESLIKNTFDSSHYAAYTFLYLKDTTVLSDM 120

MTVDSCj16_Tlp13 LNEEVTLTKGVATSLNEMFQNNDHVDIDLIESLIKNTFDSSHYAAYTFLYLKDTTVLSDM 120

14980A LNEEVTLTKGVATSLNEMFQNNDHVDIDLIESLIKNTFDSSHYAAYTFLYLKDTTVLSDM 120

********* **************:**:********:***********************

MTVDSCj13_Tlp13 QNVDKKYISPDGKTFSMIFFDQIAEKSGGITTISTPNNFSQLNLIQNIEQNAKYGDKDSV 180

RM1875_Tlp13 QNVDKKYISPDGKTFSMIFFDQIAEKSGGITTISTPNNFSQLNLIQNIEQNAKYGDKDSV 180

CF2-75_Tlp13 QNVDKKYISPDGKTFSMIFFDQIAEKSGGITTISTPNNFSQLNLIQNIEQNAKYGDKDSV 180

15-537360_Tlp13 QNVDKKYISPDGKTFSMIFFDQIVEKSGGITTISTPNNFSQLNLIQNIEQNAKYGDKDSV 180

CVM_N29710_Tlp13 QNVDKKYISPDGKTFSMIFFDQIVEKSGGITTISTPNNFSQLNLIQNIEQNAKYGDKDSV 180

FB1_Tlp13 QNVDKKYISPDGKTFSMIFFDQIVEKSGGITTISTPNNFSQLNLIQNIEQNAKYGDKDSV 180

CFCAN032805_Tlp13 QNVDKKYISPDGKTFSMIFFDQIVEKSGGITTISTPNNFSQLNLIQNIEQNAKYGDKDSV 180

BG2108_Tlp13 QNVDKKYISPDGKTFSMIFFDQIVEKSGGITTISTPNNFSQLNLIQNIEQNAKYGDKDSV 180

YF2105_Tlp13 QNVDKKYISPDGKTFSMIFFDQIVEKSGGITTISTPNNFSQLNLIQNIEQNAKYGDKDSV 180

YH503_Tlp13 QNVDKKYISPDGKTFSMIFFDQIVEKSGGITTISTPNNFSQLNLIQNIEQNAKYGDKDSV 180

BFRCA9557_Tlp13 QNVDKKYISPDGKTFSMIFFDQIVEKSGGITTISTPNNFSQLNLIQNIEQNAKYGDKDSV 180

YH502_Tlp13 QNVDKKYISPDGKTFSMIFFDQIVEKSGGITTISTPNNFSQLNLIQNIEQNAKYGDKDSV 180

OR12_Tlp13 QNVDKKYISPDGKTFSMIFFDQIAEKSGGITTISTPNNFSQLNLIQNIEQNAKYGDKDSV 180

00-1597_Tlp13 QNVDKKYISPDGKTFSMIFFDQIVEKSGGITTISTPNNFSQLNLIQNIEQNAKYGDKDSV 180

14903A QNVDKKYISPDGKTFSMIFFDQIVEKSGGITTISTPNNFSQLNLIQNIEQNAKYGDKDSV 180

FJ3124_Tlp13 QNVDKKYISPDGKTFSMIFFDQIVEKSGGITTISTPNNFSQLNLIQNIEQNAKYGDKDSV 180

R14_Tlp13 QNVDKKYISPDGKTFSMIFFDQIAEKSGGITTISTPNNFSQLNLIQNIEQNAKYGDKDSV 180

MTVDSCj16_Tlp13 QNVDKKYISPDGKTFSMIFFDQIAEKSGGITTISTPNNFSQLNLIQNIEQNAKYGDKDSV 180

14980A QNVDKKYISPDGKTFSMIFFDQIAEKSGGITTISTPNNFSQLNLIQNIEQNAKYGDKDSV 180

***********************.************************************

MTVDSCj13_Tlp13 FVGSPRKLNYDNNEFLGINFGMPIFNNKGKFIGVIGYTLDLLEISEIILDPKFDFFEGDL 240

RM1875_Tlp13 FVGSPRKLNYDNNEFLGINFGMPIFNNKGKFIGVIGYTLDLLEISEIILDPKFDFFEGDL 240

CF2-75_Tlp13 FVGSPRKLNYDNNEFLGINFGMPIFNNKGKFIGVIGYTLDLLEISEIILDPKFDFFEGDL 240

15-537360_Tlp13 FVDSPRKLNYDNNEFLGINFGMPIFNNKGKFIGVIGYTIDLLEISETILDPKFDFFEGDL 240

CVM_N29710_Tlp13 FVDSPRKLNYDNNEFLGINFGMPIFNNKGKFIGVIGYTIDLLEISETILDPKFDFFEGDL 240

FB1_Tlp13 FVDSPRKLNYDNNEFLGINFGMPIFNNKGKFIGVIGYTIDLLEISETILDPKFDFFEGDL 240

CFCAN032805_Tlp13 FVDSPRKLNYDNNEFLGINFGMPIFNNKGKFIGVIGYTIDLLEISETILDPKFDFFEGDL 240

BG2108_Tlp13 FVDSPRKLNYDNNEFLGINFGMPIFNNKGKFIGVIGYTIDLLEISETILDPKFDFFEGDL 240

YF2105_Tlp13 FVDSPRKLNYDNNEFLGINFGMPIFNNKGKFIGVIGYTIDLLEISETILDPKFDFFEGDL 240

YH503_Tlp13 FVDSPRKLNYDNNEFLGINFGMPIFNNKGKFIGVIGYTIDLLEISETILDPKFDFFEGDL 240

BFRCA9557_Tlp13 FVDSPRKLNYDNNEFLGINFGMPIFNNKGKFIGVIGYTIDLLEISETILDPKFDFFEGDL 240

YH502_Tlp13 FVDSPRKLNYDNNEFLGINFGMPIFNNKGKFIGVIGYTIDLLEISETILDPKFDFFEGDL 240

OR12_Tlp13 FVDSPRKLNYDNNEFLGINFGMPIFNNKGKFIGVIGYTIDLLEISETILDPKFDFFEGDL 240

00-1597_Tlp13 FVDSPRKLNYDNNEFLGINFGMPIFNNKGKFIGVIGYTIDLLEISETILDPKFDFFEGDL 240

14903A FVDSPRKLNYDNNEFLGINFGMPIFNNKGKFIGVIGYTIDLLEISETILDPKFDFFEGDL 240

FJ3124_Tlp13 FVDSPRKLNYDNNEFLGINFGMPIFNNKGKFIGVIGYTIDLLEISETILDPKFDFFEGDL 240

R14_Tlp13 FVGSPRKLNYDNNEFLGINFGMPIFNNKGKFIGVIGYTIDLLEISETILDPKFDFFEGDL 240

MTVDSCj16_Tlp13 FVGSPRKLNYDNNEFLGINFGMPIFNNKGKFIGVIGYTIDLLEISETILDPKFDFFEGDL 240

14980A FVGSPRKLNYDNNEFLGINFGMPIFNNKGKFIGVIGYTIDLLEISETILDPKFDFFEGDL 240

**.***********************************:******* *************

MTVDSCj13_Tlp13 RILMNDQGIIAVHKNKNGILKTLFDINKDQSAQLIVEAVKNHKDEILDNYIASTGDLSYA 300

RM1875_Tlp13 RILMNDQGIIAVHKIKNGILKTLFDINKDQSAQLIVEAVKNHKDEILDNYIASTGDPSYA 300

CF2-75_Tlp13 RILMNDQGIIAVHKIKNGILKTLFDINKDQSAQLIVEAVKNHKDEILDNYIASTGDPSYA 300

15-537360_Tlp13 RFLMNDQGIIAIHKNKNAILKTLFDINKDQSAQLIVEAVKNHKDEILDNYIASTGDLSYA 300

CVM_N29710_Tlp13 RFLMNDQGIIAIHKNKNAILKTLFDINKDQSAQLIVEAVKNHKDEILDNYIASTGDLSYA 300

FB1_Tlp13 RFLMNDQGIIAIHKNKNAILKTLFDINKDQSAQLIVEAVKNHKDEILDNYIASTGDLSYA 300

CFCAN032805_Tlp13 RFLMNDQGIIAIHKNKNAILKTLFDINKDQSAQLIVEAVKNHKDEILDNYIASTGDLSYA 300

BG2108_Tlp13 RFLMNDQGIIAIHKNKNAILKTLFDINKDQSAQLIVEAVKNHKDEILDNYIASTGDLSYA 300

YF2105_Tlp13 RFLMNDQGIIAIHKNKNAILKTLFDINKDQSAQLIVEAVKNHKDEILDNYIASTGDLSYA 300

YH503_Tlp13 RFLMNDQGIIAIHKNKNAILKTLFDINKDQSAQLIVEAVKNHKDEILDNYIASTGDLSYA 300

BFRCA9557_Tlp13 RFLMNDQGIIAIHKNKNAILKTLFDINKDQSAQLIVEAVKNHKDEILDNYIASTGDLSYA 300

YH502_Tlp13 RFLMNDQGIIAIHKNKNAILKTLFDINKDQSAQLIVEAVKNHKDEILDNYIASTGDLSYA 300

OR12_Tlp13 RFLMNDQGIIAIHKNKNAILKTLFDINKDQSAQLIVEAVKNHKDEILDNYIASTGDLSYA 300

00-1597_Tlp13 RFLMNDQGIIAIHKNKNAILKTLFDINKDQSAQLIVEAVKNHKDEILDNYIASTGDLSYA 300

14903A RFLMNDQGIIAIHKNKNAILKTLFDINKDQSAQLIVEAVKNHKDEILDNYIASTGDLSYA 300

FJ3124_Tlp13 RFLMNDQGIIAIHKNKNAILKTLFDINKDQSAQLIVEAVKNHKDEILDNYIASTGDLSYA 300

R14_Tlp13 RFLMNDQGIIAIHKNKNAILKTLFDINKDQSAQLIVEAVKNHKDEILDNYIASTGDLSYA 300

MTVDSCj16_Tlp13 RFLMNDQGIIAIHKNKNAILKTLFDINKDQSAQLIVEAVKNHKDEILDNYIASTGDLSYA 300

14980A RFLMNDQGIIAIHKNKNAILKTLFDINKDQSAQLIVEAVKNHKDEILDNYIASTGDLSYA 300

*:*********:** **.************************************** ***

MTVDSCj13_Tlp13 SISSFSTLGNSSHWSVIVTAPKKSVLAPLYKLQYTIISVAIIALIAILTVVYFFIRKIIG 360

RM1875_Tlp13 SISSFSTLGNSSHWSVIVTTPKKSVLAPLYKLQYTIISVAIIALIAILTVVYFFIRKIIG 360

CF2-75_Tlp13 SISSFSTLGNSSHWSVIVTAPKKSVLAPLYKLQYTIISVAIIALIAILTVVYFFIRKIIG 360

15-537360_Tlp13 SISSFSTLGNSSHWSVIVTAPKKSVLAPLYKLQYIIISVAIIALIAILAVVYFFIRKIIG 360

CVM_N29710_Tlp13 SISSFSTLGNSSHWSVIVTAPKKSVLAPLYKLQYIIISVAIIALIAILAVVYFFIRKIIG 360

FB1_Tlp13 SISSFSTLGNSSHWSVIVTAPKKSVLAPLYKLQYIIISVAIIALIAILAVVYFFIRKIIG 360

CFCAN032805_Tlp13 SISSFSTLGNSSHWSVIVTAPKKSVLAPLYKLQYIIISVAIIALIAILAVVYFFIRKIIG 360

BG2108_Tlp13 SISSFSTLGNSSHWSVIVTAPKKSVLAPLYKLQYIIISVAIIALIAILAVVYFFIRKIIG 360

YF2105_Tlp13 SISSFSTLGNSSHWSVIVTAPKKSVLAPLYKLQYIIISVAIIALIAILAVVYFFIRKIIG 360

YH503_Tlp13 SISSFSTLGNSSHWSVIVTAPKKSVLAPLYKLQYIIISVAIIALIAILAVVYFFIRKIIG 360

BFRCA9557_Tlp13 SISSFSTLGNSSHWSVIVTAPKKSVLAPLYKLQYIIISVAIIALIAILAVVYFFIRKIIG 360

YH502_Tlp13 SISSFSTLGNSSHWSVIVTAPKKSVLAPLYKLQYIIISVAIIALIAILAVVYFFIRKIIG 360

OR12_Tlp13 SISSFSTLGNSSHWSVIVTAPKKSVLAPLYKLQYIIISVAIIALIAILAVVYFFIRKIIG 360

00-1597_Tlp13 SISSFSTLGNSSHWSVIVTAPKKSVLAPLYKLQYIIISVAIIALIAILAVVYFFIRKIIG 360

14903A SISSFSTLGNSSHWSVIVTAPKKSVLAPLYKLQYIIISVAIIALIAILAVVYFFIRKIIG 360

FJ3124_Tlp13 SISSFSTLGNSSHWSVIVTAPKKSVLAPLYKLQYIIISVAIIALIAILAVVYFFIRKIIG 360

R14_Tlp13 SISSFSTLGNSSHWSVIVTAPKKSVLAPLYKLQYIIISVAIIALIAILAVVYFFIRKIIG 360

MTVDSCj16_Tlp13 SISSFSTLGNSSHWSVIVTAPKKSVLAPLYKLQYIIISVAIIALIAILAVVYFFIRKIIG 360

14980A SISSFSTLGNSSHWSVIVTAPKKSVLAPLYKLQYIIISVAIIALIAILAVVYFFIRKIIG 360

*******************:************** *************:***********

MTVDSCj13_Tlp13 SRIPLILKSLENFFRFLNHEKIEIQTIEIKANDELGKMGKIINENILATKQGLEQDAKAV 420

RM1875_Tlp13 SRIPLILKSLENFFRFLNHEKIEIQTIEIKANDELGKMGKIINENILATKQGLEQDAKAV 420

CF2-75_Tlp13 SRIPLILKSLENFFRFLNHEKIEIQTIEIKANDELGKMGKIINENILATKQGLEQDAKAV 420

15-537360_Tlp13 SRIPLILKSLENFFRFLNHEKIEVQTIEIKANDELGKMGKIINENILATKQGLEQDAKAV 420

CVM_N29710_Tlp13 SRIPLILKSLENFFRFLNHEKIEVQTIEIKANDELGKMGKIINENILATKRGLEQDNQAV 420

FB1_Tlp13 SRIPLILKSLENFFRFLNHEKIEVQTIEIKANDELGKMGKIINENILATKRGLEQDNQAV 420

CFCAN032805_Tlp13 SRIPLILKSLENFFRFLNHEKIEVQTIEIKANDELGKMGKIINENILATKRGLEQDNQAV 420

BG2108_Tlp13 SRIPLILKSLENFFRFLNHEKIEVQTIEIKANDELGKMGKIINENILATKRGLEQDNQAV 420

YF2105_Tlp13 SRIPLILKSLENFFRFLNHEKIEVQTIEIKANDELGKMGKIINENILATKRGLEQDNQAV 420

YH503_Tlp13 SRIPLILKSLENFFRFLNHEKIEVQTIEIKANDELGKMGKTINENILATKRGLEQDNQAV 420

BFRCA9557_Tlp13 SRIPLILKSLENFFRFLNHEKIEIQTIEIKANDELGKMGKIINENILATKQGLEQDAKAV 420

YH502_Tlp13 SRIPLILKSLENFFRFLNHEKIEVQTIEIKANDELGKMGKTINENILATKQGLEQDAKAV 420

OR12_Tlp13 SRIPLILKSLENFFRFLNHEKIEVQTIEIKANDELGKMGKIINENILATKRGLEQDNQAV 420

00-1597_Tlp13 SRIPLILKSLENFFRFLNHEKIEVQTIEIKANDELGKMGKIINENILATKRGLEQDNQAV 420

14903A SRIPLILKSLENFFRFLNHEKIEVQTIEIKANDELGKMGKIINENILATKRGLEQDNQAV 420

FJ3124_Tlp13 SRIPLILKSLENFFRFLNHEKIEVQTIEIKANDELGKMGKIINENILATKRGLEQDNQAV 420

R14_Tlp13 SRIPLILKSLENFFRFLNHEKIEVQTIEIKANDELGKMGKIINENILATKRGLEQDNQAV 420

MTVDSCj16_Tlp13 SRIPLILKSLENFFRFLNHEKIEVQTIEIKANDELGKMGKIINENILATKRGLEQDNQAV 420

14980A SRIPLILKSLENFFRFLNHEKIEVQTIEIKANDELGKMGKIINENILATKRGLEQDNQAV 420

***********************:**************** *********:***** :**

MTVDSCj13_Tlp13 KESVETVGVVESGNLTARITANPRNPQLIELKNVLNKLLDVLQARVGSDMNAIHKIFEEY 480

RM1875_Tlp13 KESVETVGVVESGNLTARITANPRNPQLIELKNVLNRLLDVLQTRVGSDMNAIHKIFEEY 480

CF2-75_Tlp13 KESVETVGVVESGNLTARITANPRNPQLIELKNVLNRLLDVLQTKVGSDMNAIHKIFEEY 480

15-537360_Tlp13 KESVETVSVVEGGNLTARITANPRNPQLIELKNVLNRLLDALQARVGSDMNAIHKIFEEY 480

CVM_N29710_Tlp13 KESVQTVSVVEGGNLTARITANPRNPQLIELKNVLNRLLDALQARVGSDMNAIHKIFEEY 480

FB1_Tlp13 KESVQTVSVVEGGNLTARITANPRNPQLIELKNVLNRLLDALQARVGSDMNAIHKIFEEY 480

CFCAN032805_Tlp13 KESVQTVSVVEGGNLTARITANPRNPQLIELKNVLNRLLDALQARVGSDMNAIHKIFEEY 480

BG2108_Tlp13 KESVQTVSVVEGGNLTARITANPRNPQLIELKNVLNRLLDVLQTKVGSDMNAIHKIFEEY 480

YF2105_Tlp13 KESVQTVSVVEGGNLTARITANPRNPQLIELKNVLNRLLDVLQTKVGSDMNAIHKIFEEY 480

YH503_Tlp13 KESVQTVSVVEGGNLTARITANPRNPQLIELKNVLNRLLDVLQTKVGSDMNAIHKIFEEY 480

BFRCA9557_Tlp13 KESVETVGVVERGNLTARITANPRNPQLIELKNVLNKLLDVLQTKVGSDMNAIHKIFEEY 480

YH502_Tlp13 KESVETVGVVESGNLTARITANPRNPQLIELKNVLNRLLDVLQTKVGSDMNAIHKIFEEY 480

OR12_Tlp13 KESVQTVSVVEGGNLTARITANPRNPQLIELKNVLNRLLDALQARVGSDMNEIQRVFNSY 480

00-1597_Tlp13 KESVQTVSVVEGGNLTARITANPRNPQLIELKNVLNRLLDALQARVGSDMNEIQRVFNSY 480

14903A KESVQTVSVVEGGNLTARITANPRNPQLIELKNVLNRLLDALQARVGSDMNEIQRVFNSY 480

FJ3124_Tlp13 KESVQTVSVVEGGNLTARITANPRNPQLIELKNVLNKLLDVLQARVGSDMNAIHKIFEEY 480

R14_Tlp13 KESVQTVSVVEGGNLTARITANPRNPQLIELKNVLNKLLDVLQARVGSDMNAIHKIFEEY 480

MTVDSCj16_Tlp13 KESVQTVSVVEGGNLTARITANPRNPQLIELKNVLNKLLDVLQARVGSDMNAIHKIFEEY 480

14980A KESVQTVSVVEGGNLTARITANPRNPQLIELKNVLNKLLDVLQARVGSDMNAIHKIFEEY 480

****:**.*** ************************:***.**::****** *:::*:.*

MTVDSCj13_Tlp13 KSLDFRNKLENASGSVELTTNALGDEIVKMLKQSSDFANALANESGKLQTAVQSLTTSSN 540

RM1875_Tlp13 KSLDFRNKLDNANGSVEVTTNALGDEIVKMLKQSSDFANHLASESSKLQSAVQNLTSSSN 540

CF2-75_Tlp13 KSLDFRNKLDNANGSVEVTTNALGDEIVKMLKQSSDFANHLASESSKLQSAVQNLTSSSN 540

15-537360_Tlp13 KSLDFRNKLDNANGSVEVTTNALGDEIVKMLKQSSDFANHLASESSKLQSAVQNLTSSSN 540

CVM_N29710_Tlp13 KSLDFRNKLDNANGSVEVTTNALGDEIVKMLKQSSDFANHLASESSKLQSAVQNLTSSSN 540

FB1_Tlp13 KSLDFRNKLDNANGSVEVTTNALGDEIVKMLKQSSDFANHLASESSKLQSAVQNLTSSSN 540

CFCAN032805_Tlp13 KSLDFRNKLDNANGSVEVTTNALGDEIVKMLKQSSDFANHLASESSKLQSAVQNLTSSSN 540

BG2108_Tlp13 KSLDFRNKLDNANGSVEVTTNALGDEIVKMLKQSSDFANHLASESSKLQSAVQNLTSSSN 540

YF2105_Tlp13 KSLDFRNKLDNANGSVEVTTNALGDEIVKMLKQSSDFANHLASESSKLQSAVQNLTSSSN 540

YH503_Tlp13 KSLDFRNKLDNANGSVEVTTNALGDEIVKMLKQSSDFANHLASESSKLQSAVQNLTSSSN 540

BFRCA9557_Tlp13 KSLDFRNKLDNANGSVEVTTNALGDEIVKMLKQSSDFANHLASESSKLQSAVQNLTSSSN 540

YH502_Tlp13 KSLDFRNKLDNANGSVEVTTNALGDEIVKMLKQSSDFANHLASESSKLQSAVQNLTSSSN 540

OR12_Tlp13 KSLDFTTEVKDANGAVEVTTNALGQEIIKMLKQSSDFANALANESSKLQTAVQSLTTSSN 540

00-1597_Tlp13 KSLDFTTEVKDANGAVEVTTNALGQEIIKMLKQSSDFANALANESGKLQTAVQSLTTSSN 540

14903A KSLDFTTEVKDANGAVEVTTNALGQEIIKMLKQSSDFANALANESGKLQTAVQSLTTSSN 540

FJ3124_Tlp13 KSLDFRNKLENASGSVELTTNALGDEIVKMLKQSSDFANALANESGKLQTAVQSLTTSSN 540

R14_Tlp13 KSLDFRNKLENASGSVELTTNALGNEIVKMLKQSSDFANALANESGKLQTAVQSLTTSSN 540

MTVDSCj16_Tlp13 KSLDFRNKLENASGSVELTTNALGDEIVKMLKQSSDFANALANESGKLQTAVQSLTTSSN 540

14980A KSLDFRNKLENASGSVELTTNALGDEIVKMLKQSSDFANALANESGKLQTAVQSLTTSSN 540

***** .::.:*.*:**:******:**:*********** **.**.***:***.**:***

MTVDSCj13_Tlp13 SQAQSLEETAAALEEITSSMQNVSVKTSDVITQSEEIKNVTGIIGDIADQINLLALNAAI 600

RM1875_Tlp13 SQAASLEETAAALEEITSSMQNVSVKTSDVITQSEEIKNVTGIIGDIADQINLLALNAAI 600

CF2-75_Tlp13 SQAASLEETAAALEEITSSMQNVSVKTSDVITQSEEIKNVTGIIGDIADQINLLA----- 595

15-537360_Tlp13 SQAASLEETAAALEEITSSMQNVSVKTSDVITQSEEIKNVTGIIGDIADQINLLALNAAI 600

CVM_N29710_Tlp13 SQAASLEETAAALEEITSSMQNVSVKTSDVITQSEEIKNVTGIIGDIADQINLLALNAAI 600

FB1_Tlp13 SQAASLEETAAALEEITSSMQNVSVKTSDVITQSEEIKNVTGIIGDIADQINLLALNAAI 600

CFCAN032805_Tlp13 SQAASLEETAAALEEITSSMQNVSVKTSDVITQSEEIKNVTGIIGDIADQINLLALNAAI 600

BG2108_Tlp13 SQAASLEETAAALEEITSSMQNVSVKTSDVITQSEEIKNVTGIIGDIADQINLLALNAAI 600

YF2105_Tlp13 SQAASLEETAAALEEITSSMQNVSVKTSDVITQSEEIKNVTGIIGDIADQINLLALNAAI 600

YH503_Tlp13 SQAASLEETAAALEEITSSMQNVSVKTSDVITQSEEIKNVTGIIGDIADQINLLALNAAI 600

BFRCA9557_Tlp13 SQAASLEETAAALEEITSSMQNVSVKTSDVITQSEEIKNVTGIIGDIADQINLLALNAAI 600

YH502_Tlp13 SQAASLEETAAALEEITSSMQNVSVKTSDVITQSEEIKNVTGIIGDIADQINLLALNAAI 600

OR12_Tlp13 SQAQSLEETAAALEEITSSMQNVSVKTSDVITQSEEIKNVTGIIGDIADQINLLALNAAI 600

00-1597_Tlp13 SQAQSLEETAAALEEITSSMQNVSVKTSDVITQSEEIKNVTGIIGDIADQINLLALNAAI 600

14903A SQAQSLEETAAALEEITSSMQNVSVKTSDVITQSEEIKNVTGIIGDIADQINLLALNAAI 600

FJ3124_Tlp13 SQAQSLEETAAALEEITSSMQNVSVKTSDVITQSEEIKNVTGIIGDIADQINLLALNAAI 600

R14_Tlp13 SQAQSLEETAAALEEITSSMQNVSVKTSDVITQSEEIKNVTGIIGDIADQINLLALNAAI 600

MTVDSCj16_Tlp13 SQAQSLEETAAALEEITSSMQNVSVKTSDVITQSEEIKNVTGIIGDIADQINLLALNAAI 600

14980A SQAQSLEETAAALEEITSSMQNVSVKTSDVITQSEEIKNVTGIIGDIADQINLLALNAAI 600

*** ***************************************************

MTVDSCj13_Tlp13 EAARAGEHGRGFAVVADEVRKLAERTQKSLSEIEANTNLLVQSINDMAESIKEQTAGITQ 660

RM1875_Tlp13 EAARAGEHGRGFAVVADEVRKLAERTQKSLSEIEANTNLLVQSINDMAESIKEQTAGITQ 660

CF2-75_Tlp13 -----------------------EITQKSLSEIEANTNLLVQSINDMAESIKEQTAGITQ 632

15-537360_Tlp13 EAARAGEHGRGFAVVADEVRKLAERTQKSLSEIEANTNLLVQSINDMAESIKEQTAGITQ 660

CVM_N29710_Tlp13 EAARAGEHGRGFAVVADEVRKLAERTQKSLSEIEANTNLLVQSINDMAESIKEQTAGITQ 660

FB1_Tlp13 EAARAGEHGRGFAVVADEVRKLAERTQKSLSEIEANTNLLVQSINDMAESIKEQTAGITQ 660

CFCAN032805_Tlp13 EAARAGEHGRGFAVVADEVRKLAERTQKSLSEIEANTNLLVQSINDMAESIKEQTAGITQ 660

BG2108_Tlp13 EAARAGEHGRGFAVVADEVRKLAERTQKSLSEIEANTNLLVQSINDMAESIKEQTAGITQ 660

YF2105_Tlp13 EAARAGEHGRGFAVVADEVRKLAERTQKSLSEIEANTNLLVQSINDMAESIKEQTAGITQ 660

YH503_Tlp13 EAARAGEHGRGFAVVADEVRKLAERTQKSLSEIEANTNLLVQSINDMAESIKEQTAGITQ 660

BFRCA9557_Tlp13 EAARAGEHGRGFAVVADEVRKLAERTQKSLSEIEANTNLLVQSINDMAESIKEQTAGITQ 660

YH502_Tlp13 EAARAGEHGRGFAVVADEVRKLAERTQKSLSEIEANTNLLVQSINDMAESIKEQTAGITQ 660

OR12_Tlp13 EAARAGEHGRGFAVVADEVRKLAERTQKSLSEIEANTNLLVQSINDMAESIKEQTAGITQ 660

00-1597_Tlp13 EAARAGEHGRGFAVVADEVRKLAERTQKSLSEIEANTNLLVQSINDMAESIKEQTAGITQ 660

14903A EAARAGEHGRGFAVVADEVRKLAERTQKSLSEIEANTNLLVQSINDMAESIKEQTAGITQ 660

FJ3124_Tlp13 EAARAGEHGRGFAVVADEVRKLAERTQKSLSEIEANTNLLVQSINDMAESIKEQTAGITQ 660

R14_Tlp13 EAARAGEHGRGFAVVADEVRKLAERTQKSLSEIEANTNLLVQSINDMAESIKEQTAGITQ 660

MTVDSCj16_Tlp13 EAARAGEHGRGFAVVADEVRKLAERTQKSLSEIEANTNLLVQSINDMAESIKEQTAGITQ 660

14980A EAARAGEHGRGFAVVADEVRKLAERTQKSLSEIEANTNLLVQSINDMAESIKEQTAGITQ 660

************************ ***********************************

MTVDSCj13_Tlp13 INDSVAQIDQTTKDNVEIANESAIISSTVSDIANNILEDVKKKRF 705

RM1875_Tlp13 INESVAQIDQTTKDNVEIANESAIISNTVSDIANNIL-------- 697

CF2-75_Tlp13 INESVAQIDQTTKDNVEIANESAIISSTVSDIANNILEDVKKKRF 677

15-537360_Tlp13 INESVAQIDQTTKDNVEIANESAIISSTVSDIANNILEDVKKKRF 705

CVM_N29710_Tlp13 INESVAQIDQTTKDNVEIANESAIISSTVSDIANNILEDVKKKRF 705

FB1_Tlp13 INESVAQIDQTTKDNVEIANESAIISSTVSDIANNILEDVKKKRF 705

CFCAN032805_Tlp13 INESVAQIDQTTKDNVEIANESAIISSTVSDIANNILEDVKKKRF 705

BG2108_Tlp13 INESVAQIDQTTKDNVEIANESAIISSTVSDIANNILEDVKKKRF 705

YF2105_Tlp13 INESVAQIDQTTKDNVEIANESAIISSTVSDIANNILEDVKKKRF 705

YH503_Tlp13 INESVAQIDQTTKDNVEIANESAIISSTVSDIANNILEDVKKKRF 705

BFRCA9557_Tlp13 INESVAQIDQTTKDNVEIANESAIISSTVSDIANSILEDVKKKRF 705

YH502_Tlp13 INESVAQIDQTTKDNVEIANESAIISSTVSDIANNILEDVKKKRF 705

OR12_Tlp13 INDSVAQIDQTTKDNVEIANESAIISSTVSDIANNILEDVKKKRF 705

00-1597_Tlp13 INDSVAQIDQTTKDNVEIANESAIISSTVSDIANNILEDVKKKRF 705

14903A INESVAQIDQTTKDNVEIANESAIISSTVSDIANNILEDVKKKRF 705

FJ3124_Tlp13 INDSVAQIDQTTKDNVEIANESAIISSTVSDIANNILEDVKKKRF 705

R14_Tlp13 INDSVAQIDQTTKDNVEIANESAIISSTVSDIANNILEDVKKKRF 705

MTVDSCj16_Tlp13 INDSVAQIDQTTKDNVEIANESAIISSTVSDIANNILEDVKKKRF 705

14980A INDSVAQIDQTTKDNVEIANESAIISSTVSDIANNILEDVKKKRF 705

**:***********************.*******.**********
