# Supplementary material for: Diversity of transducer-like proteins (Tlps) in Campylobacter
Source: PLoS One. 2019 Mar 25;14(3):e0214228. doi: 10.1371/journal.pone.0214228 (PMC6433261; doi:10.1371/journal.pone.0214228)
Supplement: S2 Archive — (ZIP) [file pone.0214228.s016.zip › Alignment T.docx]

Alignment T. Tlp14 protein sequence comparisons: individual isolate comparisons

CLUSTAL O(1.2.4) multiple sequence alignment 2018/04/18

CVM_N29710_Tlp14 ----------------MNNIKIKLSVIANSIAIFALSILSIISFYFTKDSLYQSTLYAET 44

RM4661_Tlp14 ----------------MNNIKIKLSVIANSIAIFALSILSIISFYFTKDSLYKSTLYAET 44

MG1116_Tlp14 ----------------MNNIKIKLSVIANSIAIFALSILSIISFYFTKDSLYQSTLHAET 44

BG2108_Tlp14 ----------------MNNIKIKLSVIANSIAIFALSILSIISFYFTKDSLYQSTLHAET 44

YF2105_Tlp14 ----------------MNNIKIKLSVIANSIAIFALSILSIISFYFTKDSLYQSTLHAET 44

YH502_Tlp14 -----MLKITKIKRKNMNNIKIKLSVIANSIAIFALSILSIISFYFTKDSLYQSTLHAKT 55

WA333_Tlp14 ----------------MNNIKIKLSVIANSIAIFALSILSIISFYFTKDSLYQSTLHAET 44

BP3181_Tlp14 ----------------MNNIKIKLSVIANSIAIFALSILSIISFYFTKDSLYQSTLYTET 44

ZV1224_Tlp14a ----------------MNNIKIKLSVIANSIAIFALSILSIISFYFTKDSLYQSTLHTET 44

ZV1224_Tlp14b ----------------MNNIKIKLSVIANSIAIFALSILSIISFYFTKDSLYQSTLHTET 44

YH503_Tlp14 -----MLKITKIKRKNMNNIKIKLSVIANSIAIFALSILSIISFYFTKDSLYQSTLHAKT 55

14983A ----------------MNNIKIKLSVIANSIAIFALSILSIISFYFTKDSLYQSTLHAET 44

OR12_Tlp14 ----------------MNNIKIKLSVIANSIAIFALSILSIISFYFTKDSLYQSTLHAET 44

CFSAN032805_Tlp14 -----MLKITKIKRKNMNNIKIKLSVIANSIAIFALSILSIISFYFTKDSLYQSTLHAET 55

BFR-CA-9557_Tlp14 ----------------MNNIKIKLSVIANSIAIFALSILSIISFYFTKDSLYQSTLHAET 44

15-537360_Tlp14 ----------------MNNIKIKLSVIANSIAIFALSILSIISFYFTKDSLYQSTLHAET 44

YH501_Tlp14 ----------------MNNIKIKLSVIANSIAIFALSILSIISFYFTKDSLYQSTLHAET 44

CG8421_Tlp14 ----------------MNNIKIKLSVIANSIAIFALSILSIISFYFTKDSLYQSTLYTET 44

MTVDSCj16_Tlp14 ----------------MNNIKIKLSVIANSIAIFALSILSIISFYFTKDSLYQSTLYTET 44

01-1512_Tlp14 ----------------MNNIKIKLSVIANSIAIFALSILSIISFYFTKDSLYQSTLYTET 44

00-0949_Tlp14 ----------------MNNIKIKLSVIANSIAIFALSILSIISFYFTKDSLYQSTLYTET 44

MTVDSCj13_Tlp14 ----------------MNNIKIKLSVIANSIAIFALSILSIISFYFTKDSLYQSTLYTET 44

S3_Tlp14 ----------------MNNIKIKLSVIANSIAIFALSILSIISFYFTKDSLYQSTLYTET 44

PT14_Tlp14 ----------------MNNIKIKLSVIANSIAIFALSILSIISFYFTKDSLYQSTLYTET 44

14980A MLKVLLQKLIKFKRKNMNNIKIKLSVIANSIAIFALSILSIISFYFTKDSLYQSTLYTET 60

FJ3124_Tlp14 ----------------MNNIKIKLSVIANSIAIFALSILSIISFYFTKDSLYQSTLYTET 44

00-1597_Tlp14 ----------------MNNIKIKLSVIANSIAIFALSILSIISFYFTKDSLYQSTLYTET 44

R14_Tlp14 ----------------MNNIKIKLSVIANSIAIFALSILSIISFYFTKDSLYQSTLYTET 44

************************************:***:::*

CVM_N29710_Tlp14 EFLKATQVSIEDFRSRNISLLNALEKDILNLPYEALNSQDNIVNNAGVILKYYRDSGNFL 104

RM4661_Tlp14 EFLKATQVSIENFRSRNISLLNALEKDILNLPYEALNSQDNIVNNAGAILKYYRNSGNFL 104

MG1116_Tlp14 DLLKATQISIEDFRSRNISLLNTLEKDILNLPYEALNSQDNIINNAGAILKYYRNSGNLL 104

BG2108_Tlp14 DLLKATQISIEDFRSRNISLLNTLEKDILNLPYEALNSQDNIINNAGAILKYYRNSGNLL 104

YF2105_Tlp14 DLLKATQISIEDFRSRNISLLNTLEKDILNLPYEALNSQDNIINNAGAILKYYRNSGNLL 104

YH502_Tlp14 DLLKATQISIENFRSRNISLLNALEKDILNLPYEALNSQDNIVNNVGAILKYYRNSGNLL 115

WA333_Tlp14 DLLKATQISIEDFRSRNISLLNALEKDILNLPYEALNSQDNIVNNVGAILKYYRNSGNLL 104

BP3181_Tlp14 ELLKATQISIEDFRSRNISLLNTLEKDILNLPYEALNSQDNIINNAGAILKYYRNSGNLL 104

ZV1224_Tlp14a ELLKAAQISIEDFRSRNISLLNALEKDILNLPYEALNSQDNIINNVGAILKYYRNSGNLL 104

ZV1224_Tlp14b ELLKAAQISIEDFRSRNISLLNALEKDILNLPYEALNSQDNIINNVGAILKYYRNSGNLL 104

YH503_Tlp14 ELLKATQISIEDFRSRNISLLNTLEKDILNLPYEALNSQDNIINNAGAILKYYRNSGNLL 115

14983A DLLKATQISIEDFRSRNISLLNTLEKDILNLPYEALNSQDNIVNNVGAILKYYRNSGNLL 104

OR12_Tlp14 DLLKATQISIEDFRSRNISLLNTLEKDILNLPYEALNSQDNIVNNVGAILKYYRNSGNLL 104

CFSAN032805_Tlp14 DLLKATQISIENFRSRNISLLNALEKDILNLPYEALNSQDNIVNNVGAILKYYRNSGNLL 115

BFR-CA-9557_Tlp14 DLLKATQISIENFRSRNISLLNALEKDILNLPYEALNSQDNIVNNVGAILKYYRNSGNVL 104

15-537360_Tlp14 DLLKATQISIENFRSRNISLLNALEKDILNLPYEALNSQDNIVNNVGAILKYYRNSGNVL 104

YH501_Tlp14 DLLKATQISIENFRSRNISLLNALEKDILNLPYEALNSQDNIVNNVGAILKYYRNSGNLL 104

CG8421_Tlp14 ELLKATQISIEDFRSRNISLLNTLEKDILKLPYEALNSQDNIVNNVGAILKYYRNSGNLL 104

MTVDSCj16_Tlp14 ELLKATQISIEDFRSRNISLLNTLEKDILKLPYEALNSQDNIVNNVGAILKYYRNSGNLL 104

01-1512_Tlp14 ELLKATQISIEDFRSRNISLLNTLEKDILKLPYEALNSQDNIVNNVGAILKYYRNSGNLL 104

00-0949_Tlp14 ELLKATQISIEDFRSRNISLLNTLEKDILKLPYEALNSQDNIVNNVGAILKYYRNSGNLL 104

MTVDSCj13_Tlp14 ELLKATQISIEDFRSRNISLLNTLEKDILKLPYEALNSQDNIVNNAGAILKYYRNSGNLL 104

S3_Tlp14 ELLKATQISIEDFRSRNISLLNTLEKDILKLPYEALNSQDNIVNNVGAILKYYRNSGNLL 104

PT14_Tlp14 ELLKATQISIEDFRSRNISLLNTLEKDILKLPYEALNSQDNIVNNVGAILKYYRNSGNLL 104

14980A ELLKATQISIEDFRSRNISLLNTLEKDILKLPYEALNSQDNIVNNVGAILKYYRNSGNLL 120

FJ3124_Tlp14 ELLKATQISIEDFRSRNISLLNTLEKDILKLPYEALNSQDNIVNNVGAILKYYRNSGNLL 104

00-1597_Tlp14 ELLKATQISIEDFRSRNISLLNTLEKDILKLPYEALNSQDNIVNNVGAILKYYRNSGNLL 104

R14_Tlp14 ELLKATQISIEDFRSRNISLLNTLEKDILKLPYEALNSQDNIVNNVGAILKYYRNSGNLL 104

::***:*:***:**********:******:************:**.*.******:***.*

CVM_N29710_Tlp14 AVYIGLDNGENIVSNDLSEKKNTNITINGKANNYNATTREWYKEARNSNQIYIAPAYIDA 164

RM4661_Tlp14 AVYIGLDNGENIVSDDLSEKKNTNITINGKANNYNATTREWYKEARNSNQIYIVPAYIDT 164

MG1116_Tlp14 AVYIGLDNGENIVSDDLSEKKNTNITINGKANNYNATTREWYKEARNSNQIYITPAYIDV 164

BG2108_Tlp14 AVYIGLDNGENIVSDDLSEKKNTNITINGKANNYNATTREWYKEARNSNQIYITPAYIDV 164

YF2105_Tlp14 AVYIGLDNGENIVSDDLSEKKNTNITINGKANNYNATTREWYKEARNSNQIYITPAYIDV 164

YH502_Tlp14 AVYIGLDNGENIVSDDLSEKKNTNITINGKANNYNATTREWYKEARNSNQIYITPAYIDV 175

WA333_Tlp14 AVYIGLDNGENIVSDDLSEKKNTNITINGKANNYNATTREWYKEARNSNQTYITPAYIDV 164

BP3181_Tlp14 AVYIGLDNGENIVSDDLSEKKNTNITINGKANNYNATTREWYKEARNSNQMYITPAYIDV 164

ZV1224_Tlp14a AVYIGLDNGENIVSDDLSEKKNTNITINGKANNYNATTREWYKEARNSNQTYITPAYIDV 164

ZV1224_Tlp14b AVYIGLDNGENIVSDDLSEKKNTNITINGKANNYNATTREWYKEARNSNQTYITPAYIDV 164

YH503_Tlp14 AVYIGLDNGENIVSDDLSEKKNTNITINGKANNYNATTREWYKEARNSNQIYITPAYIDV 175

14983A AVYIGLDNGENIVSDDLSEKKNTNITINGKANNYNATTREWYKEARNSNQTYITPAYIDV 164

OR12_Tlp14 AVYIGLDNGENIVSDDLSEKKNTNITINGKANNYNATTREWYKEARNSNQTYITPAYIDV 164

CFSAN032805_Tlp14 AVYIGLDNGENIVSDDLSEKKNTNITINGKANNYNATTREWYKEARNSNQTYITPAYIDV 175

BFR-CA-9557_Tlp14 AVYIGLDNGENIVSDDLSEKKNTNITINEKANNYNATTREWYKEARNSNQTYITPAYIDV 164

15-537360_Tlp14 AVYIGLDNGENIVSDDLSEKKNTNITINGKANNYNATTREWYKEARNSNQTYITPAYIDV 164

YH501_Tlp14 AVYIGLDNGENIVSDDLSEKKNTNITINGKANNYNATTREWYKEARNSNQTYITPAYIDV 164

CG8421_Tlp14 AVYIGLDNGENIMSSDLSEKKNTNITINGKANNYNATTREWYKEARNSNQIYITPAYIDA 164

MTVDSCj16_Tlp14 AVYIGLDNGENIMSSDLSEKKNTNITINGKANNYNATTREWYKEARNSNQINITPAYIDA 164

01-1512_Tlp14 AVYIGLDNGENIMSSDLSEKKNTNITINGKANNYNATTREWYKEARNSNQIYITPAYIDA 164

00-0949_Tlp14 AVYIGLDNGENIMSSDLSEKKNTNITINGKANNYNATTREWYKEARNSNQIYITPAYIDA 164

MTVDSCj13_Tlp14 AVYIGLDNGENIMSSDLSEKKNTNITINGKANNYNATTREWYKEARNSNQIYITPAYIDA 164

S3_Tlp14 AVYIGLDNGENIMSSDLSEKKNTNITINGKANNYNATTREWYKEARNSNQIYITPAYIDA 164

PT14_Tlp14 AVYIGLDNGENIMSSDLSEKKNTNITINGKANNYNATTREWYKEARNSNQIYITPAYIDA 164

14980A AVYIGLDNGENIMSSDLSEKKNTNITINGKANNYNATTREWYKEARNSNQIYITPAYIDA 180

FJ3124_Tlp14 AVYIGLDNGENIMSSDLSEKKNTNITINGKANNYNATTREWYKEARNSNQIYITPAYIDA 164

00-1597_Tlp14 AVYIGLDNGENIMSSDLSEKKNTNITINGKANNYNATTREWYKEARNSNQIYITPAYIDV 164

R14_Tlp14 AVYIGLDNGENIMSSDLSEKKNTNITINGKANNYNATTREWYKEARNSNQIYITPAYIDA 164

************:*.************* ********************* *.*****.

CVM_N29710_Tlp14 VSNEYTITYSKALYKDGKFIGVLGIDTLLTGLQDAIARTPGNAFVFNSKDEIFAAPNKAL 224

RM4661_Tlp14 VSNEYTITYSKALYKDGKFIGVLGIDALLTGLQDAIARTPGNAFVFNSKDEIFAAPNKAL 224

MG1116_Tlp14 VSNEYAITYSKALYKDGKFIGVLGFDVLLISLQDEIARTPGNTFVFDHQDRIFAATNKAL 224

BG2108_Tlp14 VSNEYAITYSKALYKDGKFIGVLGFDVLLISLQDEITRTPGNTFVFDHKDRIFAATNKAL 224

YF2105_Tlp14 VSNEYAITYSKALYKDGKFIGVLGFDVLLISLQDEITRTPGNTFVFDHKDRIFAATNKAL 224

YH502_Tlp14 VSNEYAITYSKALYKDGKFIGVLGIDVLLTSLQDRIARTPGNTFVFDHKDRVFAATNKAL 235

WA333_Tlp14 VSNEYAITYSKALYKDGKFIGVLGFDVLLISLQDLIARTPGNTFVFDHKDRVFAATNKAL 224

BP3181_Tlp14 VSNEYAITYSKALYKDGKFIGVLGFDVLLTSLQDRIARTPGNSFVFDHKDRIFAATNKAL 224

ZV1224_Tlp14a VSNEYAITYSKALYKDGKFIGVLGFDVLLIDLQDKIARTPGNTFVFDHQDRIFAATNKAL 224

ZV1224_Tlp14b VSNEYAITYSKALYKDGKFIGVLGFDVLLIDLQDKIARTPGNTFVFDHQDRIFAATNKAL 224

YH503_Tlp14 VSNEYAITYSKALYKDGKFIGVLGIDVLLTSLQDRIARTPGNTFVFDHKDRVFAATNEAL 235

14983A VSNEYAITYSKALYKDGKFIGVLGFDVLLINLQDEIARTPGNTFVFDHQDRIFAATNKAL 224

OR12_Tlp14 VSNEYAITYSKALYKDGKFIGVLGIDVLLTNLQDEIARTPGNTFVFDHKDRVFAATNKAL 224

CFSAN032805_Tlp14 VSNEYAITYSKALYKDGKFIGVLGFDVLLINLQDEIARTPGNTFVFDHKDRVFAAANKAL 235

BFR-CA-9557_Tlp14 VSNEYAITYSKALYKDGKFIGVLGFDVLLISLQDEIARTPGNTFVFDHKDRVFAATNKAL 224

15-537360_Tlp14 VSNEYAITYSKALYKDGKFIGVLGLDVLLISLQDEIARTPGNTFVFDHKDRVFAATNKAL 224

YH501_Tlp14 VSNEYAITYSKALYKDGKFIGVLGFDVLLISLQDEIARTPGNTFVFDHKDRVFAATNKAL 224

CG8421_Tlp14 ISNEYCITYSKALYKDGKFIGVLGIDILLTSLQDQIARTPGNTFVFDNKDKIFAATNEAL 224

MTVDSCj16_Tlp14 ISNEYCITYSKALYKDGKFIGVLGIDILLTSLQDQIARTPGNTFVFDNKDKIFAATNEAL 224

01-1512_Tlp14 ISNEYCITYSKALYKDGKFIGVLGIDILLTSLQDQIARTPGNTFVFDNKDKIFAATNEAL 224

00-0949_Tlp14 ISNEYCITYSKALYKDGKFIGVLGIDILLTSLQDQIARTPGNTFVFDNKDKIFAATNEAL 224

MTVDSCj13_Tlp14 ISNEYCITYSKALYKDGKFIGVLGIDILLTSLQDQIARTPGNTFVFDNKDKIFAATNEAL 224

S3_Tlp14 ISNEYCITYSKALYKDGKFIGVLGIDILLTSLQDQIARTPGNTFVFDNKDKIFAATNEAL 224

PT14_Tlp14 VSNEYCITYSKALYKDGKFIGVLGIDILLTSLQDQIARTPGNTFVFDNKDKIFAATNEAL 224

14980A ISNEYCITYSKALYKDGKFIGVLGIDILLTSLQDQIARTPGNTFVFDNKDKIFAATNEAL 240

FJ3124_Tlp14 ISNEYCITYSKALYKDGKFIGVLGIDILLTSLQDQIARTPGNTFVFDNKDKIFAATNEAL 224

00-1597_Tlp14 VSNEYCITYSKALYKDGKFIGVLGFDVLLTSLQDRIARTPGNTFVFDHKDKVFAATNKAL 224

R14_Tlp14 ISNEYCITYSKALYKDGKFIGVLGIDILLTSLQDQIARTPGNTFVFDNKDKIFAATNEAL 224

:**** ******************:* ** .*** *:*****:***: :*.:*** *:**

CVM_N29710_Tlp14 LDPSVDYSPILNAYKLNGDNNFFSYELNNEEGLGVCKKIFTYTACITESVDVINEPIFKA 284

RM4661_Tlp14 LDPSVDYSPILNAYKLNGDNNFFSYELNNEEGLGVCKKIFTYTACITESVDVINEPIFKA 284

MG1116_Tlp14 LDPSVDHSPVLNAYKAHGDNNFFSYKLNNEERLGVCTKVFAYTACITESTDVINKPIFKA 284

BG2108_Tlp14 LDPSVDHSPVLNAYKAHGDNNFFSYKLNNEERLGVCTKVFAYTACITESTDVINKPIFKA 284

YF2105_Tlp14 LDPSVDHSPVLNAYKAHGDNNFFSYKLNNEERLGVCTKVFAYTACITESTDVINKPIFKA 284

YH502_Tlp14 LDPSVDHSPVLNAYKTHGDYNFFTYGLDGKERLGACTKVFAYTACITESADIINKPIFKA 295

WA333_Tlp14 LDPSVDHSPVLNAYKAHGDNNFFSYKLNNEERLGTCTKVFAYTACITESTDVINKPIFKA 284

BP3181_Tlp14 LDPSVDHSPVLNAYKAHGDNNFFSYKLNNEERLGVCTKVFAYTACITESADIINKPIFKA 284

ZV1224_Tlp14a LDPSVDHSPVLNAYKAHGDNNFFSYKLNNEERLGVCTKVFAYTACITESTDVINKPIFKA 284

ZV1224_Tlp14b LDPSVDHSPVLNAYKAHGDNNFFSYKLNNEERLGVCTKVFAYTACITESTDVINKPIFKA 284

YH503_Tlp14 LDPSVDHSPVLNAYKAHGDNNFFSYKLNNEERLGACTKAFAYTACITESTDVINKPIFKA 295

14983A LDPSVDHSPVLNAYKAHGDNNFFSYKLNNEERLGVCTKVFAYTACITESTDVINKPIFKA 284

OR12_Tlp14 LDPSVDHSPVLNAYKAHGDNNFFSYKLNNEERLGTCTKVFAYTACITESTDVINKPIFKA 284

CFSAN032805_Tlp14 LDPSVDHSPVLNAYKAHGDNNFFSYKLNNEERLGTCTKVFAYTACITESTDVINKPIFKA 295

BFR-CA-9557_Tlp14 LDPSVDHSPVLNAYKAHGDNNFFSYKLNNEERLGTCTKVFAYTACITESTDVINKPIFKA 284

15-537360_Tlp14 LDPSVDHSPVLNAYKAHGDNNFFSYKLNNEERLGTCTKVFAYTACITESTDVINKPIFKA 284

YH501_Tlp14 LDPSVDHSPVLNAYKAHGDNNFFSYKLNNEERLGTCTKVFAYTACITESTDVINKPIFKA 284

CG8421_Tlp14 LDPSVDHSPVLNAYKAHGDNNFFSYKLNNEERLGACTKVFAYTACITESADIINKPIFKA 284

MTVDSCj16_Tlp14 LDPSVDHSPVLNAYKAHGDNNFFSYKLNNEERLGACTKVFAYTACITESADIINKPIYKA 284

01-1512_Tlp14 LDPSVDHSPVLNAYKAHGDNNFFSYKLNNEERLGACTKVFAYTACITESADIINKPIYKA 284

00-0949_Tlp14 LDPSVDHSPVLNAYKAHGDNNFFSYKLNNEERLGACTKVFAYTACITESADIINKPIYKA 284

MTVDSCj13_Tlp14 LDPSVDHSPVLNAYKAHGDNNFFSYKLNNEERLGACTKVFAYTACITESADIINKPIFKA 284

S3_Tlp14 LDPSVDHSPVLNAYKAHGDNNFFSYKLNNEERLGACTKVFAYTACITESADIINKPIFKA 284

PT14_Tlp14 LDPSVDHSPVLNAYKAHGDNNFFSYKLNNEERLGACTKVFAYTACITESADIINKPIFKA 284

14980A LDPSVDHSPVLNAYKAHGDNNFFSYKLNNEERLGACTKVFAYTACITESADIINKPIFKA 300

FJ3124_Tlp14 LDPSVDHSPVLNAYKAHGDNNFFSYKLNNEERLGACTKVFAYTACITESADIINKPIFKA 284

00-1597_Tlp14 LDPSVDHSPVLNAYKAHGDNNFFSYKLNNEERLGACTKVFAYTACITESTDVINKPIFKA 284

R14_Tlp14 LDPSVDHSPVLNAYKTHGDYNFFTYGLDGKERLGACTKVFAYTACITESADIINKPIFKA 284

******:**:***** :** ***:* *:.:* **.*.* *:********.*:**:**:**

CVM_N29710_Tlp14 VYIQVIALIIMISISIILLYFIVSKYLSPLAAIQTGLTSFFDFINHKTKNVSTIEIKSND 344

RM4661_Tlp14 VYIQVIALIIMISISIILLYFIVSKYLSPLAAIQTGLTSFFDFINHKTKNVSTIDVKTND 344

MG1116_Tlp14 AYIQVIALIVMISISIILLYFIVSKYLSPLATI--------------TKNVSTIDIKTND 330

BG2108_Tlp14 AYIQVIALIIMISISIILLYFIVSKYLSPLAAI--------------QT----------- 319

YF2105_Tlp14 AYIQVIALIIMISISIILLYFIVSKYLSPLAAI--------------QTGLTSFFDFINH 330

YH502_Tlp14 AYIQVIALIVMISISIILLYFIVSKYLSPLAAIQTGLTSFFDFINYKTKNVSTIEIKSND 355

WA333_Tlp14 AYIQVIALIVMISISIILLYFIVSKYLSPLAA-----------INHKTKNVSTIEIKSND 333

BP3181_Tlp14 AYIQVIALIVMISISIILLYFIVSKYLSPLAAIQTGLTSFFDFINHKTKNVSTIEIKSND 344

ZV1224_Tlp14a AYIQVIALIIMISISIILLYFIVSKYLSPLAAIQTGLTSFFDFINHKTKNVSTIDVKTND 344

ZV1224_Tlp14b AYIQVIALIIMISISIILLYFIVSKYLSPLAAIQTGLTSFFDFINHKTKNVSTIDVKTND 344

YH503_Tlp14 AYIQVIALIIMISISIILLYFIVSKYLSPLAAIQTGLTSFFDFINHKTKNVSTIEIKTND 355

14983A AYIQVIALIVMISISIILLYFIVSKYLSPLAAIQTGLTSFFDFINHKTKNVSTIEIKSND 344

OR12_Tlp14 AYIQVIALIIMISISIILLYFIVSKYLSPLAAIQTGLTSFFDFINHKTKNVSTIEIKTND 344

CFSAN032805_Tlp14 AYIQVIALIVMISISIILLYFIVSKYLSPLAAIQTGLTSFFDFINHKTKNVSTIDVKTND 355

BFR-CA-9557_Tlp14 AYIQVIALIIMISISIILLYFIVSKYLSPLAAIQTGLTSFFDFINHKTKNVSTIDVKTND 344

15-537360_Tlp14 AYIQVIALIVMISISIILLYFIVSKYLSPLAAIQTGLTSFFDFINHKTKNVSTIEIKSND 344

YH501_Tlp14 AYIQVIALIIMISISIILLYFIVSKYLSPLAAIQTGLTSFFDFINHKTKNVSTIEIKSND 344

CG8421_Tlp14 AYIQVIALIVMISISI------VSKYLSPLAAIQTGLTSFFDFINYKTKNVSTIEVKSND 338

MTVDSCj16_Tlp14 AFIQVIALIVMISISIILLYFIVSKYLSPLAAIQTGLTSFFDFINYKTKNVSTIEVKSND 344

01-1512_Tlp14 AFIQVIALIVMISISIILLYFIVSKYLSPLAAIQTGLTSFFDFINYKTKNVSTIEVKSND 344

00-0949_Tlp14 AFIQVIALIVMISISIILLYFIVSKYLSPLAAIQTGLTSFFDFINYKTKNVSTIEVKSND 344

MTVDSCj13_Tlp14 AYIQVIALIVMISISIILLYFIVSKYLSPLAAIQTGLTSFFDFINYKTKNVSTIEVKSND 344

S3_Tlp14 AYIQVIALIVMISISIILLYFIVSKYLSPLAAIQTGLTSFFDFINYKTKNVSTIEVKSND 344

PT14_Tlp14 AYIQVIALIVMISISIILLYFIVSKYLSPLAAIQTGLTSFFDFINYKTKNVSTIEVKSND 344

14980A AYIQVIALIVMISISIILLYFIVSKYLSPLAAIQTGLTSFFDFINYKTKNVSTIEVKSND 360

FJ3124_Tlp14 AYIQVIALIVMISISIILLYFIVSKYLSPLAAIQTGLTSFFDFINYKTKNVSTIEVKSND 344

00-1597_Tlp14 AYIQVIALIIMISISIILLYFIVSKYLSPLAAIQTGLTSFFDFINYKTKNVSTIEVKSND 344

R14_Tlp14 AYIQVIALIVMISISIILLYFIVSKYLSPLAAIQTGLTSFFDFINYKTKNVSTIEVKSND 344

.:*******:**********************:************ ********

CVM_N29710_Tlp14 EFGQISKTINENILATKQGLEQDAKAVKESVETVGVVESGNLTARI-------TANPRNP 397

RM4661_Tlp14 EFGQISKAINENILATKQGLEQDAKAVKESVETVGVVESGNLTARI-------TANPRNP 397

MG1116_Tlp14 EFGQISKAINENILATKQGLEQDAKAVKESVETVGVVESGNLTARITANHARITANPRNP 390

BG2108_Tlp14 --------------------------------------------------ARITANPRNP 329

YF2105_Tlp14 -----------------------------KTKNVSLLKQ--IWF-RSCLRARITANPRNP 358

YH502_Tlp14 EFGQISKTINENILATKQGLEQDAKAVKESVETVGVVESGNLTARI-------TANPRNP 408

WA333_Tlp14 EFGQISKTINENILATKQGLEQDAKAVKESVETVGVVESGNLTARI-------TANPRNP 386

BP3181_Tlp14 EFGQISKTINENILATKQGLEQDAKAVKESVETVGVVESGNLTARI-------TANPRNP 397

ZV1224_Tlp14a EFGQISKAINENILATKQGLEQDAKAVKESVETVGVVESGNLTARI-------TANPRNP 397

ZV1224_Tlp14b EFGQISKAINENILATKQGLEQDAKAVKESVETVGVVESGNLTARI-------TANPRNP 397

YH503_Tlp14 EFGQISKTINENILATKQGLEQDAKAVKESVETVGVVERGNLTARI-------TANPRNP 408

14983A EFGQISKTINENILATKQGLEQDAKAVKESVETVGVVESGNLTARI-------TANPRNP 397

OR12_Tlp14 EFGQISKTINENILATKQGLEQDAKAVKESVETVGVVERGNLTARI-------TANPRNP 397

CFSAN032805_Tlp14 EFGQISKTINENILATKQGLEQDAKAVKESVETVGVVERGNLTARI-------TANPRNP 408

BFR-CA-9557_Tlp14 EFGQISKAINENILATKQGLEQDAKAVKESVETVGVVERGNLTARI-------TANPRNP 397

15-537360_Tlp14 EFGQISKAINENILATKQGLEQDAKAVKESVETVGVVESGNLTARI-------TANPRNP 397

YH501_Tlp14 EFGQISKAINENILATKQGLEQDAKAVKESVETVGVVERGNLTARI-------TANPRNP 397

CG8421_Tlp14 EFGQISNAINENILATKRGLEQDNQAVKESVQTVSVVEGGNLTARI-------TANPRNP 391

MTVDSCj16_Tlp14 EFGQISNAINENILATKRGLEQDNQAVKESVQTVSVVEGGNLTARI-------TANPRNP 397

01-1512_Tlp14 EFGQISNAINENILATKRGLEQDNQAVKESVQTVSVVEGGNLTARI-------TANPRNP 397

00-0949_Tlp14 EFGQISNAINENILATKRGLEQDNQAVKESVQTVSVVEGGNLTARI-------TANPRNP 397

MTVDSCj13_Tlp14 EFGQISNAINENILATKRGLEQDNQAVKESVQTVSVVESGNLTARI-------TANPRNP 397

S3_Tlp14 EFGQISNAINENILATKRGLEQDNQAVKESVQTVSVVEGGNLTARI-------TANPRNP 397

PT14_Tlp14 EFGQISNAINENILATKRGLEQDNQAVKESVQTVSVVEGGNLTARI-------TANPRNP 397

14980A EFGQISNAINENILATKRGLEQDNQAVKESVQTVSVVEGGNLTARI-------TANPRNP 413

FJ3124_Tlp14 EFGQISNAINENILATKRGLEQDNQAVKESVQTVSVVEGGNLTARI-------TANPRNP 397

00-1597_Tlp14 EFGQISNAINENILATKRGLEQDNQAVKESVQTVSVVEGGNLTARI-------TANPRNP 397

R14_Tlp14 EFGQISNAINENILATKRGLEQDNQAVKESVQTVSVVEGGNLTARI-------TANPRNP 397

****** ********* ***** ****** ** *** ******* *******

CVM_N29710_Tlp14 QLIELKNVLNKLLDVLQAKVGSDMNEIQRVFNSYKSLDFTTEVKDANGAVEVTTNALGQE 457

RM4661_Tlp14 QLIELKNVLNRLLDVLQTKVGSDMNAIHKIFEEYKSLDFRNKLDNASGNVEVTTNALGDE 457

MG1116_Tlp14 QLIELKNVLNRLLDVLQTKVGSDMNAIHKIFEEYKSLDFRNKLDNANGSVEVTTNALGDE 450

BG2108_Tlp14 QLIELKNVLNRLLDVLQTKVGSDMNAIHKIFEEYKSLDFRNKLDNANGSVEVTTNALGDE 389

YF2105_Tlp14 QLIELKNVLNRLLDVLQTKVGSDMNAIHKIFEEYKSLDFRNKLDNANGSVEVTTNALGDE 418

YH502_Tlp14 QLIELKNVLNRLLDVLQTKVGSDMNAIHKIFEEYKSLDFRNKLDNANGSVEVTTNALGDE 468

WA333_Tlp14 QLIELKNVLNRLLDVLQTKVGSDMNAIHKIFEEYKSLDFRNKLDNANGSVEVTTNALGDE 446

BP3181_Tlp14 QLIELKNVLNRLLDVLQTKVGSDMNAIHKIFEEYKSLDFRNKLDNANGSVEVTTNALGDE 457

ZV1224_Tlp14a QLIELKNVLNRLLDVLQTKVGSDMNAIHKIFEEYKSLDFRNKLDNANGSVEVTTNALGDE 457

ZV1224_Tlp14b QLIELKNVLNRLLDVLQTKVGSDMNAIHKIFEEYKSLDFRNKLDNANGSVEVTTNALGDE 457

YH503_Tlp14 QLIELKNVLNRLLDVLQTKVGSDMNAIHKIFEEYKSLDFRNKLDNANGSVEVTTNALGDE 468

14983A QLIELKNVLNRLLDVLQTKVGSDMNAIHKIFEEYKSLDFRNKLDNANGSVEVTTNALGDE 457

OR12_Tlp14 QLIELKNVLNKLLDVLQTKVGSDMNAIHKIFEEYKSLDFRNKLDNANGSVEVTTNALGDE 457

CFSAN032805_Tlp14 QLIELKNVLNKLLDVLQTKVGSDMNAIHKIFEEYKSLDFRNKLDNANGSVEVTTNALGDE 468

BFR-CA-9557_Tlp14 QLIELKNVLNRLLDVLQTKVGSDMNAIHKIFEEYKSLDFRNKLDNANGSVEVTTNALGDE 457

15-537360_Tlp14 QLIELKNVLNRLLDVLQTKVGSDMNAIHKIFEEYKSLDFRNKLDNANGSVEVTTNALGDE 457

YH501_Tlp14 QLIELKNVLNKLLDVLQTKVGSDMNAIHKIFEEYKSLDFRNKLDNANGSVEVTTNALGDE 457

CG8421_Tlp14 QLIELKNVLNKLLDVLQARVGSDMNAIHKIFEEYKSLDFRNKLENASGSVELTTNALGDE 451

MTVDSCj16_Tlp14 QLIELKNVLNKLLDVLQARVGSDMNAIHKIFEEYKSLDFRNKLENASGSVELTTNALGDE 457

01-1512_Tlp14 QLIELKNVLNKLLDVLQARVGSDMNAIHKIFEEYKSLDFRNKLENASGSVELTTNALGDE 457

00-0949_Tlp14 QLIELKNVLNKLLDVLQARVGSDMNAIHKIFEEYKSLDFRNKLENASGSVELTTNALGDE 457

MTVDSCj13_Tlp14 QLIELKNVLNKLLDVLQARVGSDMNAIHKIFEEYKSLDFRNKLENASGSVELTTNALGDE 457

S3_Tlp14 QLIELKNVLNKLLDVLQARVGSDMNAIHKIFEEYKSLDFRNKLENASGSVELTTNALGDE 457

PT14_Tlp14 QLIELKNVLNKLLDVLQARVGSDMNAIHKIFEEYKSLDFRNKLENASGSVELTTNALGDE 457

14980A QLIELKNVLNKLLDVLQARVGSDMNAIHKIFEEYKSLDFRNKLENASGSVELTTNALGDE 473

FJ3124_Tlp14 QLIELKNVLNKLLDVLQARVGSDMNAIHKIFEEYKSLDFRNKLENASGSVELTTNALGDE 457

00-1597_Tlp14 QLIELKNVLNRLLDALQARVGSDMNEIQRVFNSYKSLDFTTEVKDANGAVEVTTNALGQE 457

R14_Tlp14 QLIELKNVLNRLLDALQARVGSDMNEIQRVFNSYKSLDFTTEVKDANGAVEVTTNALGQE 457

**********:***.**::****** *:::*:.****** .::.:*.* **:******:*

CVM_N29710_Tlp14 IIKMLKQSSDFANHLASESSKLQSAVQNLTSSSNSQAASLEETAAALEEITSSMQNVSVK 517

RM4661_Tlp14 IVKMLKQSSDFANHLASESSKLQSAVQNLTSSSNSQAASLEETAAALEEITSSMQNVSVK 517

MG1116_Tlp14 IVKMLKQSSDFANHLASESSKLQSAVQNLTSSSNSQAASLEETAAALEEITSSMQNVSVK 510

BG2108_Tlp14 IVKMLKQSSDFANHLASESSKLQSAVQNLTSSSNSQAASLEETAAALEEITSSMQNVSVK 449

YF2105_Tlp14 IVKMLKQSSDFANHLASESSKLQSAVQNLTSSSNSQAASLEETAAALEEITSSMQNVSVK 478

YH502_Tlp14 IVKMLKQSSDFANHLASESSKLQSAVQNLTSSSNSQAASLEETAAALEEITSSMQNVSVK 528

WA333_Tlp14 IVKMLKQSSDFANHLASESSKLQSAVQNLTSSSNSQAASLEETAAALEEITSSMQNVSVK 506

BP3181_Tlp14 IVKMLKQSSDFANHLASESSKLQSAVQNLTSSSNSQAASLEETAAALEEITSSMQNVSVK 517

ZV1224_Tlp14a IVKMLKQSSDFANHLASESSKLQSAVQNLTSSSNSQAASLEETAAALEEITSSMQNVSVK 517

ZV1224_Tlp14b IVKMLKQSSDFANHLASESSKLQSAVQNLTSSSNSQAASLEETAAALEEITSSMQNVSVK 517

YH503_Tlp14 IVKMLKQSSDFANHLASESSKLQSAVQNLTSSSNSQAASLEETAAALEEITSSMQNVSVK 528

14983A IVKMLKQSSDFANHLASESSKLQSAVQNLTSSSNSQAASLEETAAALEEITSSMQNVSVK 517

OR12_Tlp14 IVKMLKQSSDFANHLASESSKLQSAVQNLTSSSNSQAASLEETAAALEEITSSMQNVSVK 517

CFSAN032805_Tlp14 IVKMLKQSSDFANHLASESSKLQSAVQNLTSSSNSQAASLEETAAALEEITSSMQNVSVK 528

BFR-CA-9557_Tlp14 IVKMLKQSSDFANHLASESSKLQSAVQNLTSSSNSQAASLEETAAALEEITSSMQNVSVK 517

15-537360_Tlp14 IVKMLKQSSDFANHLASESSKLQSAVQNLTSSSNSQAASLEETAAALEEITSSMQNVSVK 517

YH501_Tlp14 IVKMLKQSSDFANHLASESSKLQSAVQNLTSSSNSQAASLEETAAALEEITSSMQNVSVK 517

CG8421_Tlp14 IVKMLKQSSDFANALANESGKLQTAVQSLTTSSNSQAQSLEETAAALEEITSSMQNVSVK 511

MTVDSCj16_Tlp14 IVKMLKQSSDFANALANESGKLQTAVQSLTTSSNSQAQSLEETAAALEEITSSMQNVSVK 517

01-1512_Tlp14 IVKMLKQSSDFANALANESGKLQTAVQSLTTSSNSQAQSLEETAAALEEITSSMQNVSVK 517

00-0949_Tlp14 IVKMLKQSSDFANALANESGKLQTAVQSLTTSSNSQAQSLEETAAALEEITSSMQNVSVK 517

MTVDSCj13_Tlp14 IVKMLKQSSDFANALANESGKLQTAVQSLTTSSNSQAQSLEETAAALEEITSSMQNVSVK 517

S3_Tlp14 IVKMLKQSSDFANALANESGKLQTAVQSLTTSSNSQAQSLEETAAALEEITSSMQNVSVK 517

PT14_Tlp14 IVKMLKQSSDFANALANESGKLQTAVQSLTTSSNSQAQSLEETAAALEEITSSMQNVSVK 517

14980A IVKMLKQSSDFANALANESGKLQTAVQSLTTSSNSQAQSLEETAAALEEITSSMQNVSVK 533

FJ3124_Tlp14 IVKMLKQSSDFANALANESGKLQTAVQSLTTSSNSQAQSLEETAAALEEITSSMQNVSVK 517

00-1597_Tlp14 IIKMLKQSSDFANALANESGKLQTAVQSLTTSSNSQAQSLEETAAALEEITSSMQNVSVK 517

R14_Tlp14 IIKMLKQSSDFANALANESGKLQTAVQSLTTSSNSQAQSLEETAAALEEITSSMQNVSVK 517

*:*********** **.**.***:***.**:****** **********************

CVM_N29710_Tlp14 TSDVITQSEEIKNVTGIIGDIADQINLLALNAAIEAARAGEHGRGFAVVADEVRKLAERT 577

RM4661_Tlp14 TSDVITQSEEIKNVTGIIGDIADQINLLALNAAIEAARAGEHGRGFAVVADEVRKLAERT 577

MG1116_Tlp14 TSDVITQSEEIKNVTGIIGDIADQINLLALNAAIEAARAGEHGRGFAVVADEVRKLAERT 570

BG2108_Tlp14 TSDVITQSEEIKNVTGIIGDIADQINLLALNAAIEAARAGEHGRGFAVVADEVRKLAERT 509

YF2105_Tlp14 TSDVITQSEEIKNVTGIIGDIADQINLLALNAAIEAARAGEHGRGFAVVADEVRKLAERT 538

YH502_Tlp14 TSDVITQSEEIKNVTGIIGDIADQINLLALNAAIEAARAGEHGRGFAVVADEVRKLAERT 588

WA333_Tlp14 TSDVITQV---------------------------------------------------- 514

BP3181_Tlp14 TSDVITQSEEIKNVTGIIGDIADQINLLALNAAIEAARAGEHGRGFAVVADEVRKLAERT 577

ZV1224_Tlp14a TSDVITQSEEIKNVTGIIGDIADQINLLALNAAIEAARAGEHGRGFAVVADEVRKLAERT 577

ZV1224_Tlp14b TSDVITQSEEIKNVTGIIGDIADQINLLALNAAIEAARAGEHGRGFAVVADEVRKLAERT 577

YH503_Tlp14 TSDVITQSEEIKNVTGIIGDIADQINLLALNAAIEAARAGEHGRGFAVVADEVRKLAERT 588

14983A TSDVITQSEEIKNVTGIIGDIADQINLLALNAAIEAARAGEHGRGFAVVADEVRKLAERT 577

OR12_Tlp14 TSDVITQSEEIKNVTGIIGDIADQINLLALNAAIEAARAGEHGRGFAVVADEVRKLAERT 577

CFSAN032805_Tlp14 TSDVITQSEEIKNVTGIIGDIADQINLLALNAAIEAARAGEHGRGFAVVADEVRKLAERT 588

BFR-CA-9557_Tlp14 TSDVITQSEEIKNVTGIIGDIADQINLLALNAAIEAARAGEHGRGFAVVADEVRKLAERT 577

15-537360_Tlp14 TSDVITQSEEIKNVTGIIGDIADQINLLALNAAIEAARAGEHGRGFAVVADEVRKLAERT 577

YH501_Tlp14 TSDVITQSEEIKNVTGIIGDIADQINLLALNAAIEAARAGEHGRGFAVVADEVRKLAERT 577

CG8421_Tlp14 TSDVITQSEEIKNVTGIIGDIADQINLLALNAAIEAARAGEHGRGFAVVADEVRKLAERT 571

MTVDSCj16_Tlp14 TSDVITQSEEIKNVTGIIGDIADQINLLALNAAIEAARAGEHGRGFAVVADEVRKLAERT 577

01-1512_Tlp14 TSDVITQSEEIKNVTGIIGDIADQINLLALNAAIEAARAGEHGRGFAVVADEVRKLAERT 577

00-0949_Tlp14 TSDVITQSEEIKNVTGIIGDIADQINLLALNAAIEAARAGEHGRGFAVVADEVRKLAERT 577

MTVDSCj13_Tlp14 TSDVITQSEEIKNVTGIIGDIADQINLLALNAAIEAARAGEHGRGFAVVADEVRKLAERT 577

S3_Tlp14 TSDVITQSEEIKNVTGIIGDIADQINLLALNAAIEAARAGEHGRGFAVVADEVRKLAERT 577

PT14_Tlp14 TSDVITQSEEIKNVTGIIGDIADQINLLALNAAIEAARAGEHGRGFAVVADEVRKLAERT 577

14980A TSDVITQSEEIKNVTGIIGDIADQINLLALNAAIEAARAGEHGRGFAVVADEVRKLAERT 593

FJ3124_Tlp14 TSDVITQSEEIKNVTGIIGDIADQINLLALNAAIEAARAGEHGRGFAVVADEVRKLAERT 577

00-1597_Tlp14 TSDVITQSEEIKNVTGIIGDIADQINLLALNAAIEAARAGEHGRGFAVVADEVRKLAERT 577

R14_Tlp14 TSDVITQSEEIKNVTGIIGDIADQINLLALNAAIEAARAGEHGRGFAVVADEVRKLAERT 577

******* ****************************************************

CVM_N29710_Tlp14 QKSLSEIEANTNLLVQSINDMAESIKEQTAGITQINESVAQIDQTTKDNVEIANESAIIS 637

RM4661_Tlp14 QKSLSEIEANTNLLVQSINDMAESIKEQTAGITQINESVAQIDQTTKDNVEIANESAIIS 637

MG1116_Tlp14 QKSLSEIEANTNLLVQSINDMAESIKEQTAGITQINESVAQIDQTTKDNVEIANESAIIS 630

BG2108_Tlp14 QKSLSEIEANTNLLVQSINDMAESIKEQTAGITQINESVAQIDQTTKDNVEIANESAIIS 569

YF2105_Tlp14 QKSLSEIEANTNLLVQSINDMAESIKEQTAGITQINESVAQIDQTTKDNVEIANESAIIS 598

YH502_Tlp14 QKSLSEIEANTNLLVQSINDMAESIKEQTAGITQINESVAQIDQTTKDNVEIANESAIIS 648

WA333_Tlp14 -----EIEANTNLLVQSINDMAESIKEQTAGITQINESVAQIDQTTKDNVEIANESAIIS 569

BP3181_Tlp14 QKSLSEIEANTNLLVQSINDMAESIKEQTAGITQINESVAQIDQTTKDNVEIANESAIIS 637

ZV1224_Tlp14a QKSLSEIEANTNLLVQSINDMAESIKEQTAGITQINESVAQIDQTTKDNVEIANESAIIS 637

ZV1224_Tlp14b QKSLSEIEANTNLLVQSINDMAESIKEQTAGITQINESVAQIDQTTKDNVEIANESAIIS 637

YH503_Tlp14 QKSLSEIEANTNLLVQSINDMAESIKEQTAGITQINESVAQIDQTTKDNVEIANESAIIS 648

14983A QKSLSEIEANTNLLVQSINDMAESIKEQTAGITQINESVAQIDQTTKDNVEIANESAIIS 637

OR12_Tlp14 QKSLSEIEANTNLLVQSINDMAESIKEQTAGITQINESVAQIDQTTKDNVEIANESAIIS 637

CFSAN032805_Tlp14 QKSLSEIEANTNLLVQSINDMAESIKEQTAGITQINESVAQIDQTTKDNVEIANESAIIS 648

BFR-CA-9557_Tlp14 QKSLSEIEANTNLLVQSINDMAESIKEQTAGITQINESVAQIDQTTKDNVEIANESAIIS 637

15-537360_Tlp14 QKSLSEIEANTNLLVQSINDMAESIKEQTAGITQINESVAQIDQTTKDNVEIANESAIIS 637

YH501_Tlp14 QKSLSEIEANTNLLVQSINDMAESIKEQTAGITQINESVAQIDQTTKDNVEIANESAIIS 637

CG8421_Tlp14 QKSLSEIEANTNLLVQSINDMAESIKEQTAGITQINDSVAQIDQTTKDNVEIANESAIIS 631

MTVDSCj16_Tlp14 QKSLSEIEANTNLLVQSINDMAESIKEQTAGITQINDSVAQIDQTTKDNVEIANESAIIS 637

01-1512_Tlp14 QKSLSEIEANTNLLVQSINDMAESIKEQTAGITQINDSVAQIDQTTKDNVEIANESAIIS 637

00-0949_Tlp14 QKSLSEIEANTNLLVQSINDMAESIKEQTAGITQINDSVAQIDQTTKDNVEIANESAIIS 637

MTVDSCj13_Tlp14 QKSLSEIEANTNLLVQSINDMAESIKEQTAGITQINDSVAQIDQTTKDNVEIANESAIIS 637

S3_Tlp14 QKSLSEIEANTNLLVQSINDMAESIKEQTAGITQINDSVAQIDQTTKDNVEIANESAIIS 637

PT14_Tlp14 QKSLSEIEANTNLLVQSINDMAESIKEQTAGITQINDSVAQIDQTTKDNVEIANESAIIS 637

14980A QKSLSEIEANTNLLVQSINDMAESIKEQTAGITQINDSVAQIDQTTKDNVEIANESAIIS 653

FJ3124_Tlp14 QKSLSEIEANTNLLVQSINDMAESIKEQTAGITQINDSVAQIDQTTKDNVEIANESAIIS 637

00-1597_Tlp14 QKSLSEIEANTNLLVQSINDMAESIKEQTAGITQINDSVAQIDQTTKDNVEIANESAIIS 637

R14_Tlp14 QKSLSEIEANTNLLVQSINDMAESIKEQTAGITQINDSVAQIDQTTKDNVEIANESAIIS 637

************************************:***********************

CVM_N29710_Tlp14 STVSDIANNILEDVKKKRF----------- 656

RM4661_Tlp14 NTVSDIANSILEDVEKEKVLIDCSLTPSLN 667

MG1116_Tlp14 STVSDIANNILEDVKKKRF----------- 649

BG2108_Tlp14 STVSDIANNILEDVKKKRF----------- 588

YF2105_Tlp14 STVSDIANNILEDVKKKRF----------- 617

YH502_Tlp14 STVSDIANNILEDVKKKRF----------- 667

WA333_Tlp14 STVSDIANNILEDVKKKRF----------- 588

BP3181_Tlp14 STVSDIANNILEDVKKKRF----------- 656

ZV1224_Tlp14a STVSDIANNILEDVKKKRF----------- 656

ZV1224_Tlp14b STVSDIANNILEDVKKKRF----------- 656

YH503_Tlp14 STVSDIANNILEDVKKKRF----------- 667

14983A STVSDIANNILEDVKKKRF----------- 656

OR12_Tlp14 STVSDIANNILEDVKKKRF----------- 656

CFSAN032805_Tlp14 STVSDIANNILEDVKKKRF----------- 667

BFR-CA-9557_Tlp14 STVSDIANNILEDVKKKRF----------- 656

15-537360_Tlp14 NTVSDIANNILEDVKKKRF----------- 656

YH501_Tlp14 STVSDIANNILEDVKKKRF----------- 656

CG8421_Tlp14 STVSDIANNILEDVKKKRF----------- 650

MTVDSCj16_Tlp14 STVSDIANNILEDVKKKRF----------- 656

01-1512_Tlp14 STVSDIANNILEDVKKKRF----------- 656

00-0949_Tlp14 STVSDIANNILEDVKKKRF----------- 656

MTVDSCj13_Tlp14 STVSDIANNILEDVKKKRF----------- 656

S3_Tlp14 STVSDIANNILEDVKKRGFN---------- 657

PT14_Tlp14 STVSDIANNILEDVKKKRF----------- 656

14980A STVSDIANNILEDVKKKRF----------- 672

FJ3124_Tlp14 STVSDIANNILEDVKKKRF----------- 656

00-1597_Tlp14 STVSDIANNILEDVKKKR------------ 655

R14_Tlp14 STVSDIANNILEDVKKKRFF---------- 657

.*******.*****:*.
