# Supplementary material for: Diversity of transducer-like proteins (Tlps) in Campylobacter
Source: PLoS One. 2019 Mar 25;14(3):e0214228. doi: 10.1371/journal.pone.0214228 (PMC6433261; doi:10.1371/journal.pone.0214228)
Supplement: S2 Archive — (ZIP) [file pone.0214228.s016.zip › Alignment U.docx]

Alignment U. Tlp16 protein sequence comparisons: individual isolate comparisons

CLUSTAL O(1.2.4) multiple sequence alignment 2018/04/18

YH503_Tlp16 MQLSIRKKMLMLGAICFISMLATFAIFYYNNLKGSQKIAQTTKNLINKEIDIKVELLTKS 60

FB1_Tlp16 MQLSIRKKMLMLGAICFISMLATFAIFYYNNLKGSQKIAQTTKNLINKEIDIKVELLTKS 60

BFR-CA-9557_Tlp16 MQLSIRKKMLMLGAICFISMLATFAIFYYNNLKGSQKIAQTTKNLINKEIDIKVELLTKS 60

15-537360_Tlp16 MQLSIRKKMLMLGAICFISMLATFAIFYYNNLKGSQKIAQTTKNLINKEIDIKVELLTKS 60

OR12_Tlp16 MQLSIRKKMLMLGAICFISMLATFAIFYYNNLKGSQKIAQTTKNLINKEIDIKVELLTKS 60

YH502_Tlp16 MQLSIRKKMLMLGAICFISMLATFAIFYYNNLKGSQKIAQTTKNLINKEIDIKVELLTKS 60

14983A MQLSIRKKMLMLGAICFISMLATFAIFYYNNLQGSQKIAQTTKNLINKEIDIKVELLTKS 60

HC2-48_Tlp16 MQLSIRKKMLMLGAICFISMLATFAIFYYNNLQGSQKIAQTTKNLINKEINIKVELLTKS 60

RM5611_Tlp16 MQLSIRKKMLMLGAICFISMLATFAIFYYNNLQGSQKIAQTTKNLINKEINIKVELLTKS 60

********************************:*****************:*********

YH503_Tlp16 MAIALGDLIKDVDDEKEKIKISLPQLKILDLKRIKSGYFFVYQKTTVKAHPVRKDLIGTD 120

FB1_Tlp16 MAIALGDLIKDVDDEKEKIKISLPQLKILDLKRIKSGYFFVYQKTTVKAHPVRKDLIGTD 120

BFR-CA-9557_Tlp16 MAIALGDLIKDVDDEKEKIKISLPQLKILDLKR-KSGYFFVYQKTTVKAHPVRKDLIGTD 119

15-537360_Tlp16 MAIALGDLIKDVDDEKEKIKISLPQLKILDLKRIKSGYFFVYQKTTVKAHPVRKDLIGTD 120

OR12_Tlp16 MAIALGDLIKDVDDEKEKIKISLPQLKILDLKRIKSGYFFVYQKTTVKAHPVRKDLIGTD 120

YH502_Tlp16 MAIALGDLIKDVDDEKEKIKISLPQLKILDLKRIKSGYFFVYQKTTVKAHPVRKDLIGTD 120

14983A MAIALGDLIKDVDDEKEKIKIIATAIENFRFEEDQSGYFFVYQKTTVKAHPVRKDLIGTD 120

HC2-48_Tlp16 MAIALGDLIKNVHSEEEKVKIIATAIENFRFEEDKSGYFFVYQKTTVKAHPVRKDLIGSD 120

RM5611_Tlp16 MAIALGDLIKNVHSEEEKVKIIATAIENFRFEEDKSGYFFVYQKTTVKAHPVRKDLIGSD 120

**********:*..*:**:** :: : ::. :***********************:*

YH503_Tlp16 LHNAKDENGIFYVRELYQRALDKGGFVTFHFTKPQPNGENTIAEKTAYSYLIPNADDLWI 180

FB1_Tlp16 LHNAKDENGIFYVRELYQRALDKGGFVTFHFTKPQPNGENTIAEKTAYSYLIPNADDLWI 180

BFR-CA-9557_Tlp16 LHNAKDENGIFYVRELYQRALDKGGFVTFHFTKPQPNGENTIAEKTAYSYLIPNADDLWI 179

15-537360_Tlp16 LHNAKDENGIFYVRELYQRALDKGGFVTFHFTKPQPNGENTIAEKTAYSYLIPNADDLWI 180

OR12_Tlp16 LHNAKDENGIFYVRELYQRALDKGGFVTFHFTKPQPNGENTIAEKTAYSYLIPNADDLWI 180

YH502_Tlp16 LHNAKDENGIFYVRELYQRALDKGGFVTFHFTKPQPNGENTIAEKTAYSYLIPNADDLWI 180

14983A LYNAKDENGIFYVRELYQRALDKGGFVTFHFTKPQPNGENTIAEKTAYSYLIPNTDDLWI 180

HC2-48_Tlp16 LYNAKDENGIFYVRELYQRALDKGGFVTFHFTKPQPNGENTIAEKTAYSYLIPNADDLWI 180

RM5611_Tlp16 LYNAKDENGIFYVRELYQRALDKGGFVTFHFTKPQPNGENTIAEKTAYSYLIPNADDLWI 180

*:****************************************************:*****

YH503_Tlp16 STGVYKDTLEPYIDRSLEELLSFFSKSFFKTVLFSIIFILIIIPFIFIFYRNLIVGVQGI 240

FB1_Tlp16 STGVYKDTLEPYIDRSLEELLSFFSKSFFKTVLFSIIFILIIIPFIFIFYRNLIVGVQGI 240

BFR-CA-9557_Tlp16 STGVYKDTLEPYIDRSLEELLSFFSKSFFKTVLFSIIFILIIIPFIFIFYRNLIVGVQGI 239

15-537360_Tlp16 STGVYKDTLEPYIDRSLEELLSFFSKSFFKTVLFSIIFILIIIPFIFIFYRNLIVGVQGI 240

OR12_Tlp16 STGVYKDTLEPYIDRSLEELLSFFSKSFFKTVLFSIIFILIIIPFIFIFYRNLIVGVQGI 240

YH502_Tlp16 STGVYKDTLEPYIDRSLEELLSFFSKSFFKTVLFSIIFILIIIPFIFIFYRNLIVGVQGI 240

14983A STGVYKDTLEPYIDRSLEELLSFFSKSFFKTVLFSIIFILIIIPFIFIFYRNLIVGVQGI 240

HC2-48_Tlp16 STGVYKDTLEPYIDRSLEELLSFFSKSFFKTVLFSIIFILIIIPFIFIFYRNLIVGVQGI 240

RM5611_Tlp16 STGVYKDTLEPYIDRSLEELLSFFSKSFFKTVLFSIIFILIIIPFIFIFYRNLIVGVQGI 240

************************************************************

YH503_Tlp16 DANITSFFNFINHKTKNVSTIEIKSNDEFGQISKTINENILATKRGLEQDNQAVKESVQT 300

FB1_Tlp16 DANITSFFNFINHKTKNVSTIDVKTNDEFGLISKAINENILATKQGLEQDAKAVKESVET 300

BFR-CA-9557_Tlp16 DANITSFFNFINHKTKNVSTIEIKSNDEFGQISKTINENILATKQGLEQDAKAVKESVET 299

15-537360_Tlp16 DANITSFFNFINHKTKNVSTIEIKSNDEFGQISKAINENILATKQGLEQDAKAVKESVET 300

OR12_Tlp16 DANITSFFNFINHKTKNVSTIEIKSNDEFGQISKTINENILATKQGLEQDAKAVKESVET 300

YH502_Tlp16 DANITSFFNFINHKTKNVSTIEIKSNDEFGQISKTINENILATKQGLEQDAKAVKESVET 300

14983A DANITSFFNFINHKTKNVSTIEIKSNDEFGQISKAINENILATKQGLEQDAKAVKESVET 300

HC2-48_Tlp16 DANITSFFDFINHN-KNVSTIDVKTNDEFGQISKAINENILATKQGLEQDAKAVKESVET 299

RM5611_Tlp16 DANITSFFDFINHKTKNVSTIDVKTNDEFGQISKAINENILATKQGLEQDAKAVKESVET 300

********:****: ******::*:***** ***:*********:***** :******:*

YH503_Tlp16 VSVVEGGNLTARITANPRNPQLIELKNVLNRLLDVLQTKVGSDMNAIHKIFEEYKSLDFR 360

FB1_Tlp16 VGVVERGNLTARITANPRNPQLIELKNVLNRLLDVLQTKVGSDMNAIHKIFEEYKSLDFR 360

BFR-CA-9557_Tlp16 VGVVERGNLTARITANPRNPQLIELKNVLNKLLDVLQTKVGSDMNAIHKIFEEYKSLDFR 359

15-537360_Tlp16 VGVVESGNLTARITANPRNPQLIELKNVLNRLLDALQARVGSDMNAIHKIFEEYKSLDFR 360

OR12_Tlp16 VGVVKRGNLTARITANPRNPQLIELKNVLNKLLDVLQTKVGSDMNAIHKIFEEYKSLDFR 360

YH502_Tlp16 VGVVESGNLTARITANPRNPQLIELKNVLNRLLDVLQTKVGSDMNAIHKIFEEYKSLDFR 360

14983A VGVVESGNLTARITANPRNPQLIELKNVLNRLLDVLQTKVGSDMNAIHKIFEEYKSLDFR 360

HC2-48_Tlp16 VGVVESGNLTARITANPRNPQLIELKNVLNRLLDVLQTKVGSDMNAIHKIFEEYKSLDFR 359

RM5611_Tlp16 VGVVESGNLTARITANPRNPQLIELKNVLNRLLDVLQTKVGSDMNAIHKIFEEYKSLDFR 360

*.**: ************************:***.**::*********************

YH503_Tlp16 NKLDNANGSVEVTTNALGDEIVKMLKQSSDFANHLASESSKLQSAVQNLTSSSNSQAASL 420

FB1_Tlp16 NKLDNANGSVEVTTNALGDEIVKMLKQSSDFANHLASESSKLQSAVQNLTSSSNSQAASL 420

BFR-CA-9557_Tlp16 NKLDNANGSVEVTTNALGDEIVKMLKQSSDFANHLASESSKLQSAVQNLTSSSNSQAASL 419

15-537360_Tlp16 NKLDNANGSVEVTTNALGDEIVKMLKQSSDFANHLASESSKLQSAVQNLTSSSNSQAASL 420

OR12_Tlp16 NKLDNANGSVEVTTNALGDEIVKMLKQSSDFANHLASESSKLQSAVQNLTSSSNSQAASL 420

YH502_Tlp16 NKLDNANGSVEVTTNALGDEIVKMLKQSSDFANHLASESSKLQSAVQNLTSSSNSQAASL 420

14983A NKLDNANGSVEVTTNALGDEIVKMLKQSSDFANHLASESSKLQSAVQNLTSSSNSQAASL 420

HC2-48_Tlp16 NKLDNANGSVEVTTNALGDEIVKMLKQSSDFANHLASESSKLQSAVQNLTSSSNSQAASL 419

RM5611_Tlp16 NKLDNANGSVEVTTNALGDEIVKMLKQSSDFANHLASESSKLQSAVQNLTSSSNSQAASL 420

************************************************************

YH503_Tlp16 EETAAALEEITSSMQNVSVKTSDVITQSEEIKNVTGIIGDIADQINLLALNAAIEAARAG 480

FB1_Tlp16 EETAAALEEITSSMQNVSVKTSDVITQSEEIKNVTGIIGDIADQINLLALNAAIEAARAG 480

BFR-CA-9557_Tlp16 EETAAALEEITSSMQNVSVKTSDVITQSEEIKNVTGIIGDIADQINLLALNAAIEAARAG 479

15-537360_Tlp16 EETAAALEEITSSMQNVSVKTSDVITQSEEIKNVTGIIGDIADQINLLALNAAIEAARAG 480

OR12_Tlp16 EETAAALEEITSSMQNVSVKTSDVITQSEEIKNVTGIIGDIADQINLLALNAAIEAARAG 480

YH502_Tlp16 EETAAALEEITSSMQNVSVKTSDVITQSEEIKNVTGIIGDIADQINLLALNAAIEAARAG 480

14983A EETAAALEEITSSMQNVSVKTSDVITQSEEIKNVTGIIGDIADQINLLALNAAIEAARAG 480

HC2-48_Tlp16 EETAAALEEITSSMQNVSVKTSDVITQS-------------------------------- 447

RM5611_Tlp16 EETAAALEEITSSMQNVSVKTSDVITQSEEIKNVTGIIGDIADQINLLALNAAIEAARAG 480

************************************************************

YH503_Tlp16 EHGRGFAVVADEVRKLAERTQKSLSEIEANTNLLVQSINDMAESIKEQTAGITQINESVA 540

FB1_Tlp16 EHGRGFAVVADEVRKLAERTQKSLSEIEANTNLLVQSINDMAESIKEQTAGITQINESVA 540

BFR-CA-9557_Tlp16 EHGRGFAVVADEVRKLAERTQKSLSEIEANTNLLVQSINDMAESIKEQTAGITQINESVA 539

15-537360_Tlp16 EHGRGFAVVADEVRKLAERTQKSLSEIEANTNLLVQSINDMAESIKEQTAGITQINESVA 540

OR12_Tlp16 EHGRGFAVVADEVRKLAERTQKSLSEIEANTNLLVQSINDMAESIKEQTAGITQINESVA 540

YH502_Tlp16 EHGRGFAVVADEVRKLAERTQKSLSEIEANTNLLVQSINDMAESIKEQTAGITQINESVA 540

14983A EHGRGFAVVADEVRKLAERTQKSLSEIEANTNLLVQSINDMAESIKEQTAGITQINESVA 540

HC2-48_Tlp16 -------------------------EIEANTNLLVQSINDMAESIKEQTAGITQINESVA 482

RM5611_Tlp16 EHGRGFAVVADEVRKLAERTQKSLSEIEANTNLLVQSINDMAESIKEQTAGITQINESVA 540

************************************************************

YH503_Tlp16 QIDQTTKDNVEIANESAIISSTVSDIANNILEDVKKKRF 579

FB1_Tlp16 QIDQTTKDNVEIANESAIISSTVSDIANNILEDVKKKRF 579

BFR-CA-9557_Tlp16 QIDQTTKDNVEIANESAIISSTVSDIANSILEDVKKKRF 578

15-537360_Tlp16 QIDQTTKDNVEIANESAIISSTVSDIANNILEDVKKKRF 579

OR12_Tlp16 QIDQTTKDNVEIANESAIISSTVSDIANNILEDVKKKRF 579

YH502_Tlp16 QIDQTTKDNVEIANESAIISSTVSDIANNILEDVKKKRF 579

14983A QIDQTTKDNVEIANESAIISSTVSDIANNILEDVKKKRF 579

HC2-48_Tlp16 QIDQTTKDNVEIANESAIISSTVSDIANNILEDVKKKRF 521

RM5611_Tlp16 QIDQTTKDNVEIANESAIISSTVSDIANNILEDVKKKRF 579

****************************.**********
