# Supplementary material for: Diversity of transducer-like proteins (Tlps) in Campylobacter
Source: PLoS One. 2019 Mar 25;14(3):e0214228. doi: 10.1371/journal.pone.0214228 (PMC6433261; doi:10.1371/journal.pone.0214228)
Supplement: S2 Archive — (ZIP) [file pone.0214228.s016.zip › Alignment V.docx]

Alignment V. Tlp20 protein sequence comparisons: individual isolate comparisons

CLUSTAL O(1.2.4) multiple sequence alignment 2018/04/18

15-537360_Tlp20 ---MKSVKIKVSLIANLIAIVCLIFLGIITFIFVKDEVFNQVVKSESNYVRTAKNSMEAF 57

76339_Tlp20 ---MKSVKIKVSLIANLIAIVCLIFLGIITFIFVKDEVFNQVVKAESNYVRTAKNSMEAF 57

HC2-48_Tlp20 ---VKSVKIKVSLIANLIAIVCLIFLGIITFIFVKDEVFNQVVKSESNYVRTAKNSMEAF 57

CFSAN032805_Tlp20 ---VKSVKIKVSLIANLIAIVCLIFLGIITFIFVKDEVFNQVVKSESNYVRTTKNSMEAF 57

CVM_N29710_Tlp20 ---VKSVKIKVSLIANLIAIVCLI-LGIITFIFVKDEVFNQVVKSESNYVRTTKNSMEAF 56

YH501_Tlp20 VKSVKSVKIKVSLIANLIAIVCLILLGIITFIFVKDEVFNQVVKSESNYVRTTKNSMEAF 60

CF2-75_Tlp20 VKSVKSVKIKVSLIANLIAIVCLIFLGIITFIFVKDEVFNQVVKSESNYVRTAKNSMEAF 60

CO2-160_Tlp20 ---MKSVKIKVSLIANLIAIVCLIFLGIITFIFVKDEVFNQVVKSESNYVRTAKNSMEAF 57

CO2-160_Tlp20b ---MKSVKIKVSLIANLIAIVCLIFLGIITFIFVKDEVFNQVVKSESNYVRTAKNSMEAF 57

RM5611_Tlp20 ---MKSVKIKVSLIANLIAIVCLIFLGIITFIFVKDEVFNQVVKSESNYVRTAKNSMEAF 57

14983A VKSVKSVKIKVSLIANLIAIVCLIFLGIITFIFVKDEVFNQVVKSESNYVRTAKNSMEAF 60

YH502_Tlp20 ---VKSVKIKVSLIANLIAIVCLIFLGIITFIFVKDEVFNQVVKSESNYVRTAKNSMEAF 57

:******************** *******************:*******:*******

15-537360_Tlp20 KARNTAALESLAKNILKLPYEQISNQEALMRYVGKDLKVFRDAGGFLAVYIAQPDGELVV 117

76339_Tlp20 KARNTAALESLAKNILKLPYEQISNQEALMRYVGKDLKVFRDAGGFLAVYIAQPDGELVV 117

HC2-48_Tlp20 KARNTAALESLAKNILKLPYEQISNQEALMRYVGKDLKVFRDAGGFLAVYIAQPDGELVV 117

CFSAN032805_Tlp20 KARNTAALESLAKNILKLPYEQISNQEALMRYVGKDLKVFRDAGGFLAVYIAQPDGELVV 117

CVM_N29710_Tlp20 KARNTAALESLAKNILKLPYEQISNQEALMRYVGKDLKVFRDAGGFLAVYIAQPDGELVV 116

YH501_Tlp20 KARNTAALESLAKNILKLPYEQISNQEALMRYVGKDLKVFRDAGGFLAVYIAQPDGELVV 120

CF2-75_Tlp20 KARNTAALESLAKNILKLPYEQISNQEALMRYVGKDLKVFRDAGGFLAVYIAQPDGELVV 120

CO2-160_Tlp20 KARNTAALESLAKNILKLPYEQISNQEALMRYVGKDLKVFRDAGGFLAVYIAQPDGELVV 117

CO2-160_Tlp20b KARNTAALESLAKNILKLPYEQISNQEALMRYVGKDLKVFRDAGGFLAVYIAQPDGELVV 117

RM5611_Tlp20 KARNTAALESLAKNILKLPYEQISNQEALMRYVGKDLKVFRDAGGFLAVYIAQPDGELVV 117

14983A KARNTAALESLAKNILKLPYEQISNQEALMRYVGKDLKVFRDAGGFLAVYIAQSDGELVV 120

YH502_Tlp20 KARNTAALESLAKNILKLPYEQISNQEALMRYVGKDLKVFRDAGGFLAVYIAQSDGELVV 117

***************************************************** ******

15-537360_Tlp20 TDPDSDEKGLNFGIYGKADNYDARTRDYFKGAVKANGLYVTPSYLDLTTNLPCFTYATPL 177

76339_Tlp20 TDPDSDEKGLNFGIYGKADNYDARTRDYFKGAVKANGLYVTPSYLDLTTNLPCFTYAIPL 177

HC2-48_Tlp20 TNPDSDEKGLNFGIYGKADNYDARTRDYFKGAVKANGLYVTPSYLDLTTNLPCFTYATPL 177

CFSAN032805_Tlp20 TDPDSDEKGLNF-IYGKADNYDARTRDYFKGAVKANGLYVTPSYLDLTTNLPCFTYATPL 176

CVM_N29710_Tlp20 TDPDSDEKGLNF-IYGKADNYDARTRDYFKGAVKANGLYVTPSYLDLTTNLPCFTYATPL 175

YH501_Tlp20 TDPDSDEKGLNF-IYGKADNYDARTRDYFKGAVKANGLYVTPSYLDLTTNLPCFTYATPL 179

CF2-75_Tlp20 TNPDSDEKGLNFGIYGKADNYDARTRDYFKGAVKANGLYVTPSYLDLTTNLPCFTYATPL 180

CO2-160_Tlp20 TNPDSDEKGLNFGIYGKADNYDARTRDYFKGAVKANGLYVTPSYLDLTTNLPCFTYATPL 177

CO2-160_Tlp20b TNPDSDEKGLNFGIYGKADNYDARTRDYFKGAVKANGLYVTPSYLDLTTNLPCFTYATPL 177

RM5611_Tlp20 TNPDSDEKGLNFGIYGKADNYDARTRDYFKGAVKANGLYVTPSYLDLTTNLPCFTYATPL 177

14983A TDPDSDEKGLNF-IYGKADNYDARTRDYFKGAVKANGLYVTPSYLDLTTNLPCFTYATPL 179

YH502_Tlp20 TDPDSDEKGLNF-IYGKADNYDARTRDYFKGAVKANGLYVTPSYLDLTTNLPCFTYATPL 176

*:********** ******************************************** **

15-537360_Tlp20 YKEGKFIGVLAIDILVKDLQREFENLPGRTFVFDSENSVFVSTDKELLKPGYDVSPVANI 237

76339_Tlp20 YKEGKFIGVLAIDILVKDLQREFENLPGRTFVFDSKNSIFASTDKELLKPGYDVSPVADI 237

HC2-48_Tlp20 YKEGKFIGVLAIDILVKDLQREFENLPGRTFVFDSENSIFVSTDKELLKPGYDVSPVANI 237

CFSAN032805_Tlp20 YKEGKFIGVLAIDILVKDLQREFENLPGRTFVFDSENSIFVSTNKELLKPGYDVSPVANI 236

CVM_N29710_Tlp20 YKEGKFIGVLAIDILVKDLQREFENLPGRTFVFDSENSIFVSTDKELLKPGYDVSPVANI 235

YH501_Tlp20 YKEGKFIGVLAIDILVKDLQREFENLPGRTFVFDSENSIFVSTDKELLKPGYDVSPVANI 239

CF2-75_Tlp20 YKEGKFIGVLAIDILVKDLQREFENLPGRTFVFDSENSIFVSTDKELLKPGYDVSPVANI 240

CO2-160_Tlp20 YKEGKFIGVLAIDILVKDLQREFENLPGRTFVFDSENSIFVSTDKELLKPGYDVSPVANI 237

CO2-160_Tlp20b YKEGKFIGVLAIDILVKDLQREFENLPGRTFVFDSENSIFVSTDKELLKPGYDVSPVANI 237

RM5611_Tlp20 YKEGKFIGVLAIDILVKDLQREFENLPGRTFVFDSENSIFVSTDKELLKPGYDVSPVANI 237

14983A YKEGKFIGVLAIDILVKDLQREFENLPGRTFVFDSENSIFVSTNKELLKPGYDVSLVANI 239

YH502_Tlp20 YKEGKFIGVLAIDILVKDLQREFENLPGRTFVFDSENSIFVSTNKELLKPGYDVSLVANI 236

***********************************:**:*.**:*********** **:*

15-537360_Tlp20 AKDKKDYEPFRYARPLDGTQRFGVCAKVLGEYTACVGEPIDYIEEPVFKIAYIQIAIVII 297

76339_Tlp20 AKDKKDYEPFHYIRPLDGTERFGVCAKVLGEYTACVGEPIDYIEEPVFKIAYIQIAIVII 297

HC2-48_Tlp20 AKDKKDYEPFRYVRPLDGTQRFGVCAKVLGEYTACVGESIDYIEEPVFKIAYIQIAIVII 297

CFSAN032805_Tlp20 AKDKKDYEPFRYVRPLDGTQRFGVCAKVLGEYTACVGEPIDYIEEPVFKIAYIQIAIVII 296

CVM_N29710_Tlp20 AKDKKDYEPFRYVRPLDGTQRFGVCAKVLGEYTACVGEPIDYIEEPVFKIAYIQIAIVII 295

YH501_Tlp20 AKDKKDYEPFRYVRPLDGTQRFGVCAKVLGEYTACVGEPIDYIEEPVFKIAYIQIAIVII 299

CF2-75_Tlp20 AKDKKDYEPFRYVRPLDGTQRFGVCAKVLGEYTACVGESIDYIEEPVFKIAYIQIAIVII 300

CO2-160_Tlp20 AKDKKDYEPFRYVRPLDGTQRFGVCAKVLGEYTACVGESIDYIEEPVFKIAYIQIAIVII 297

CO2-160_Tlp20b AKDKKDYEPFRYVRPLDGTQRFGVCAKVLGEYTACVGESIDYIEEPVFKIAYIQIAIVII 297

RM5611_Tlp20 AKDKKDYEPFRYVRPLDGTQRFGVCAKVLGEYTACVGESIDYIEEPVFKIAYIQIAIVII 297

14983A AKDKKDYEPFRYVRPLDGTQRFGVCAKVLGEYTACVGEPIDYIEEPVFKIAYIQIAIVII 299

YH502_Tlp20 AKDKKDYEPFRYVRPLDGTQRFGVCAKVLGEYTACVGEPIDYIEEPVFKIAYIQIAIVII 296

**********:* ******:****************** *********************

15-537360_Tlp20 TSIISVLLLYFIVSRYLSPLAAIQTGLTSFFDFINHKTKNVSTIDVKTNDEFGQISKAIN 357

76339_Tlp20 TSIISVLLLYFIVSRYLSPLASIQAGLNSFFDFINHKTKDVSTIDVKTNDEFGQISKAIN 357

HC2-48_Tlp20 TSIISVLLLYFIVSRYLSPLASIQVGLNSFFDFINHN-KNVSTIDVKTNDEFGQISKAIN 356

CFSAN032805_Tlp20 TSIISVLLLYFIVSRYLSPLAAIQTGLTSFFDFINHKTKNVSTIEIKSNDEFGQISKAIN 356

CVM_N29710_Tlp20 TSIISVLLLYFIVSRYLSPLAAIQTGLTSFFDFINHKTKNVSTIEIKTNDEFGQISKTIN 355

YH501_Tlp20 TSIISVLLLYFIVSRYLSPLAAIQTGLTSFFDFINHKTKNVSTIEIKTNDEFGQISKTIN 359

CF2-75_Tlp20 TSIISVLLLYFIVSRYLSPLASIQVGLNSFFDFINHKTKNVSTIDVKTNDEFGQISKAIN 360

CO2-160_Tlp20 TSIISVLLLYFIVSRYLSPLASIQVGLNSFFDFINHKTKNVSTIDVKTNDEFGQISKAIN 357

CO2-160_Tlp20b TSIISVLLLYFIVSRYLSPLASIQVGLNSFFDFINHKTKNVSTIDVKTNDEFGQISKAIN 357

RM5611_Tlp20 TSIISVLLLYFIVSRYLSPLASIQVGLNSFFDFINHKTKNVSTIDVKTNDEFGQISKAIN 357

14983A TSIISVLLLYFIVSRYLSPLASIQVGLNSFFDFINHKTKNVSTIEIKSNDEFGQISKAIN 359

YH502_Tlp20 TSIISVLLLYFIVSRYLSPLASIQVGLNSFFDFINHKTKNVSTIDVKTNDEFGQISKAIN 356

*********************:**.**.********: *:****::*:*********:**

15-537360_Tlp20 ENILATKQGLEQDAKAVKESVETVGVVESGNLTARITANPRNPQLIELKNVLNRLLDALQ 417

76339_Tlp20 ENILATKQGLEQDAKAVKESVETVGVVERGNLTARITANPRNPQLIELKNVLNKLLDVLQ 417

HC2-48_Tlp20 ENILATKQGLEQDAKAVKESVETVGVVESGNLTARITANPRNPQLIELKNVLNRLLDVLQ 416

CFSAN032805_Tlp20 ENILATKQGLEQDAKAVKESVETVGVVERGNLTARITANPRNPQLIELKNVLNKLLDVLQ 416

CVM_N29710_Tlp20 ENILATKQGLEQDAKAVKESVETVGVVERGNLTARITANPRNPQLIELKNVLNKLLDVLQ 415

YH501_Tlp20 ENILATKQGLEQDAKAVKESVETVGVVERGNLTARITANPRNPQLIELKNVLNKLLDVLQ 419

CF2-75_Tlp20 ENILATKQGLEQDAKAVKESVETVGVVESGNLTARITANPRNPQLIELKNVLNRLLDVLQ 420

CO2-160_Tlp20 ENILATKQGLEQDAKAVKESVETVGVVESGNLTARITANPRNPQLIELKNVLNRLLDVLQ 417

CO2-160_Tlp20b ENILATKQGLEQDAKAVKESVETVGVVESGNLTARITANPRNPQLIELKNVLNRLLDVLQ 417

RM5611_Tlp20 ENILATKQGLEQDAKAVKESVETVGVVESGNLTARITANPRNPQLIELKNVLNRLLDVLQ 417

14983A ENILATKQGLEQDAKAVKESVETVGVVESGNLTARITANPRNPQLIELKNVLNRLLDVLQ 419

YH502_Tlp20 ENILATKQGLEQDAKAVKESVETVGVVESGNLTARITANPRNPQLIELKNVLNRLLDVLQ 416

**************************** ************************:***.**

15-537360_Tlp20 ARVGSDMNEIQRVFNSYKSLDFTTEVKDANGAVEVTTNALGQEIIKMLKQSSDFANALAN 477

76339_Tlp20 TKVGSDMNAIHKIFEEYKSLDFRNKLDNANGSVEVTTNALGDEIVKMLKQSSDFANHLAS 477

HC2-48_Tlp20 TKVGSDMNAIHKIFEEYKSLDFRNKLDNANGSVEVTTNALGDEIVKMLKQSSDFANHLAS 476

CFSAN032805_Tlp20 TKVGSDMNAIHKIFEEYKSLDFRNKLDNANGSVEVTTNALGDEIVKMLKQSSDFANHLAS 476

CVM_N29710_Tlp20 TKVGSDMNAIHKIFEEYKSLDFRNKLDNANGSVEVTTNALGDEIVKMLKQSSDFANHLAS 475

YH501_Tlp20 TKVGSDMNAIHKIFEEYKSLDFRNKLDNANGSVEVTTNALGDEIVKMLKQSSDFANHLAS 479

CF2-75_Tlp20 TKVGSDMNAIHKIFEEYKSLDFRNKLDNANGSVEVTTNALGDEIVKMLKQSSDFANHLAS 480

CO2-160_Tlp20 TKVGSDMNAIHKIFEEYKSLDFRNKLDNANGSVEVTTNALGDEIVKMLKQSSDFANHLAS 477

CO2-160_Tlp20b TKVGSDMNAIHKIFEEYKSLDFRNKLDNANGSVEVTTNALGDEIVKMLKQSSDFANHLAS 477

RM5611_Tlp20 TRVGSDMNAIHKIFEEYKSLDFRNKLDNANGSVEVTTNALGDEIVKMLKQSSDFANHLAS 477

14983A TKVGSDMNAIHKIFEEYKSLDFRNKLDNANGSVEVTTNALGDEIVKMLKQSSDFANHLAS 479

YH502_Tlp20 TKVGSDMNAIHKIFEEYKSLDFRNKLDNANGSVEVTTNALGDEIVKMLKQSSDFANHLAS 476

::****** *:::*:.****** .::.:***:*********:**:*********** **.

15-537360_Tlp20 ESGKLQTAVQSLTTSSNSQAASLEETAAALEEITSSMQNVSVKTSDVITQSEEIKNVTGI 537

76339_Tlp20 ESSKLQSAVQNLTSSSNSQAASLEETAAALEEITSSMQNVSVKTSDVITQSEEIKNVTGI 537

HC2-48_Tlp20 ESSKLQSAVQNLTSSSNSQAASLEETAAALEEITSSMQNVSVKTRCY------------- 523

CFSAN032805_Tlp20 ESSKLQSAVQNLTSSSNSQAASLEETAAALEEITSSMQNVSVKTSDVITQSEEIKNVTGI 536

CVM_N29710_Tlp20 ESSKLQSAVQNLTSSSNSQAASLEETAAALEEITSSMQNVSVKTSDVITQSEEIKNVTGI 535

YH501_Tlp20 ESSKLQSAVQNLTSSSNSQAASLEETAAALEEITSSMQNVSVKTSDVITQSEEIKNVTGI 539

CF2-75_Tlp20 ESSKLQSAVQNLTSSSNSQAASLEETAAALEEITSSMQNVSVKTSDVITQSEEIKNVTGI 540

CO2-160_Tlp20 ESSKLQSAVQNLTSSSNSQAASLEETAAALEEITSSMQNVSVKTSDVITQSEEIKNVTGI 537

CO2-160_Tlp20b ESSKLQSAVQNLTSSSNSQAASLEETAAALEEITSSMQNVSVKTSDVITQSEEIKNVTGI 537

RM5611_Tlp20 ESSKLQSAVQNLTSSSNSQAASLEETAAALEEITSSMQNVSVKTSDVITQSEEIKNVTGI 537

14983A ESSKLQSAVQNLTSSSNSQAASLEETAAALEEITSSMQNVSVKTSDVITQSEEIKNVTGI 539

YH502_Tlp20 ESSKLQSAVQNLTSSSNSQAASLEETAAALEEITSSMQNVSVKTSDVITQSEEIKNVTGI 536

**.***:***.**:**********************************************

15-537360_Tlp20 IGDIADQINLLALNAAIEAARAGEHGRGFAVVADEVRKLAERTQKSLSEIEANTNLLVQS 597

76339_Tlp20 IGDIADQINLLALNAAIEAARAGEHGRGFAVVADEVRKLAERTQKSLSEIEANTNLLVQS 597

HC2-48_Tlp20 ---------------------------------------------HSIEIEANTNLLVQS 538

CFSAN032805_Tlp20 IGDIADQINLLALNAAIEAARAGEHGRGFAVVADEVRKLAERTQKSLSEIEANTNLLVQS 596

CVM_N29710_Tlp20 IGDIADQINLLALNAAIEAARAGEHGRGFAVVADEVRKLAERTQKSLSEIEANTNLLVQS 595

YH501_Tlp20 IGDIADQINLLALNAAIEAARAGEHGRGFAVVADEVRKLAERTQKSLSEIEANTNLLVQS 599

CF2-75_Tlp20 IGDIADQINL----------------------------LAEITQKSLSEIEANTNLLVQS 572

CO2-160_Tlp20 IGDIADQINLLALNAAIEAARAGEHGRGFAVVADEVRKLAERTQKSLSEIEANTNLLVQS 597

CO2-160_Tlp20b IGDIADQINLLALNAAIEAARAGEHGRGFAVVADEVRKLAERTQKSLSEIEANTNLLVQS 597

RM5611_Tlp20 IGDIADQINLLALNAAIEAARAGEHGRGFAVVADEVRKLAERTQKSLSEIEANTNLLVQS 597

14983A IGDIADQINLLALNAAIEAARAGEHGRGFAVVADEVRKLAERTQKSLSEIEANTNLLVQS 599

YH502_Tlp20 IGDIADQINLLALNAAIEAARAGEHGRGFAVVADEVRKLAERTQKSLSEIEANTNLLVQS 596

***************************************** ***** ************

15-537360_Tlp20 INDMAESIKEQTAGITQINESVAQIDQTTKDNVEIANESAIISSTVSDIANNILEDVKKK 657

76339_Tlp20 INDMAESIKEQTAGITQINESVAQIDQTTKDNVEIANESAIISNTVSDIANNILEDVRKK 657

HC2-48_Tlp20 INDMAESIKEQTAGITQINESVAQIDQTTKDNVEIANESAIISSTVSDIANNILEDVKKK 598

CFSAN032805_Tlp20 INDMAESIKEQTAGITQINESVAQIDQTTKDNVEIANESAIISSTVSDIANNILEDVKKK 656

CVM_N29710_Tlp20 INDMAESIKEQTAGITQINESVAQIDQTTKDNVEIANESAIISSTVSDIANNILEDVKKK 655

YH501_Tlp20 INDMAESIKEQTAGITQINESVAQIDQTTKDNVEIANESAIISSTVSDIANNILEDVKKK 659

CF2-75_Tlp20 INDMAESIKEQTAGITQINESVAQIDQTTKDNVEIANESAIISSTVSDIANNILEDVKKK 632

CO2-160_Tlp20 INDMAESIKEQTAGITQINESVAQIDQTTKDNVEIANESAIISSTVSDIANNILEDVKKK 657

CO2-160_Tlp20b INDMAESIKEQTAGITQINESVAQIDQTTKDNVEIANESAIISSTVSDIANNILEDVKKK 657

RM5611_Tlp20 INDMAESIKEQTAGITQINESVAQIDQTTKDNVEIANESAIISSTVSDIANNILEDVKKK 657

14983A INDMAESIKEQTAGITQINESVAQIDQTTKDNVEIANESAIISSTVSDIANNILEDVKKK 659

YH502_Tlp20 INDMAESIKEQTAGITQINESVAQIDQTTKDNVEIANESAIISSTVSDIANNILEDVKKK 656

*******************************************.*************:**

15-537360_Tlp20 RF 659

76339_Tlp20 RF 659

HC2-48_Tlp20 RF 600

CFSAN032805_Tlp20 RF 658

CVM_N29710_Tlp20 RF 657

YH501_Tlp20 RF 661

CF2-75_Tlp20 RF 634

CO2-160_Tlp20 RF 659

CO2-160_Tlp20b RF 659

RM5611_Tlp20 RF 659

14983A RF 661

YH502_Tlp20 RF 658

**
