# Supplementary material for: Diversity of transducer-like proteins (Tlps) in Campylobacter
Source: PLoS One. 2019 Mar 25;14(3):e0214228. doi: 10.1371/journal.pone.0214228 (PMC6433261; doi:10.1371/journal.pone.0214228)
Supplement: S2 Archive — (ZIP) [file pone.0214228.s016.zip › Alignment W.docx]

Alignment W. Tlp23 protein sequence comparisons: individual isolate comparisons

4031_Tlp23 MKSVKLKVALIANLIAVVCLVILGVITFMFVKQAIFHEVVKAETNYVKTAKNSMESFKAR 60

RM3196_Tlp23 MKSVKLKVTLIANLITVVCLVILGVITFMFVKQAIFHEVVNAEINYVKTAKNSIESFKAR 60

ICDCCJ07001_Tlp23 MKSVKLKVTLIANLITVVCLVILGVITFMFVKQAIFHEVVNAEINYVKTAKNSIESFKAR 60

********:******:************************:** *********:******

4031_Tlp23 NSLALESLAKSILKHPVEQLDSQDALMRYVGKDLKNFRDAGRFLAVYIA-PNGELVVSDP 119

RM3196_Tlp23 NSLALESLAKSILKHPVEQLDNQDALMHYVGKDLKNFRDAGRFLAVYIAQPNGELVVSDP 120

ICDCCJ07001_Tlp23 NSLALESLAKSILKHPVEQLDNQDALMHYVGKDLKNFRDAGRFLAVYIAQPNGELVVSDP 120

*********************.*****:********************* **********

4031_Tlp23 DSDAKKVDFGTYGKADNYDARTREYYIEAVKTNKLYVTPSYIDATTNLPCFTYSTPLFKD 179

RM3196_Tlp23 DSDAKNLDFGTYGKADNYDARTREYYIEAVKTNKLYITPSYIDVTTNLPCFTYSIPLYKD 180

ICDCCJ07001_Tlp23 DSDAKNLDFGTYGKADNYDARTREYYIEAVKTNKLYITPSYIDVTTNLPCFTYSIPLYKD 180

*****::*****************************:******.********** **:**

4031_Tlp23 GKFIGVLAVDVLVTDLQAEFENLPGRTFVFDEENKVFASTDKTLLQQGYDISAIANLAKI 239

RM3196_Tlp23 GKFIGVLAVDVLAADLQAEFENLPGRIFVFDEENKVFVSTDKTLLQQGYDISTIANLAKT 240

ICDCCJ07001_Tlp23 GKFIGVLAVDILAADLQAEFENLPGRIFVFDEENKVFVSTDKTLLQQGYDISTIANLAKT 240

**********:*.:************ **********.**************:******

4031_Tlp23 KENFEPFEYTRPKDGSERFAVCTKVSGAYTACVGEPIEQIEAPVYKIAFIQAIVVIIVVV 299

RM3196_Tlp23 KKDFEPFEYTRPKDGSERFAVCVKVSGIYTACVAKPIEQIEAPVYKAAFIQAIVVIIVVV 300

ICDCCJ07001_Tlp23 KKDFEPFEYTRPKDGSERFAVCVKVSGIYTACVAKPIEQIEAPVYKAAFIQAIVVIIVVV 300

*::*******************.**** *****.:*********** *************

4031_Tlp23 FSVILLYFIVSKYLSPLAAIQTGLTSFFDFINHKTKNVSTIEVKSNDEFGQISNAINENI 359

RM3196_Tlp23 FSVILLYFIVSKYLSPLAAIQTGLTSFFDFINHKTKNVSTIEVKSNDEFGQISNAINENI 360

ICDCCJ07001_Tlp23 FSVILLYFIVIKYLSPLAAIQTGLTSFFDFINHKTKNVSTIEVKSNDEFGQISNAINENI 360

********** *************************************************

4031_Tlp23 LATKRGLEQDNQAVKESVETVSVVESGNLTARITANPRNPQLIELKNVLNKLLDVLQARV 419

RM3196_Tlp23 LATKRGLEQDNQAVKESVETVHVVEGGNLTARITANPRNPQLIELKNVLNRLLDALQARV 420

ICDCCJ07001_Tlp23 LATKRGLEQDNQAVKESVETVHVVEGGNLTARITANPRNPQLIELKNVLNRLLDALQARV 420

********************* ***.************************:***.*****

4031_Tlp23 GSDMNAIHKIFEEYKSLDFRNKLENASGSVELTTNALGDEIVKMLKQSSDFANALANESG 479

RM3196_Tlp23 GSDMNEIQRVFNSYKSLDFTTEVKDANGAVEVTTNALGQEIIKMLKQSSDFANALANESG 480

ICDCCJ07001_Tlp23 GSDMNEIQRVFNSYKSLDFTTEVKDANGAVEVTTNALGQEIIKMLKQSSDFANALANESG 480

***** *:::*:.****** .::::*.*:**:******:**:******************

4031_Tlp23 KLQTAVQSLTTSSNSQAQSLEETAAALEEITSSMQNVSVKTSDVITQSEEIKNVTGIIGD 539

RM3196_Tlp23 KLQTAVQSLTTSSNSQAQSLEETAAALEEITSSMQNVSVKTSDVITQSEEIKNVTGIIGD 540

ICDCCJ07001_Tlp23 KLQTAVQSLTTSSNSQAQSLEETAAALEEITSSMQNVSVKTSDVITQSEEIKNVTGIIGD 540

************************************************************

4031_Tlp23 IADQINLLALNAAIEAARAGEHGRGFAVVADEVRKLAERTQKSLSEIEANTNLLVQSIND 599

RM3196_Tlp23 IADQINLLALNAAIEAARAGEHGRGFAVVADEVRKLAERTQKSLSEIEANTNLLVQSIND 600

ICDCCJ07001_Tlp23 IADQINLLALNAAIEAARAGEHGRGFAVVADEVRKLAERTQKSLSEIEANTNLLVQSIND 600

************************************************************

4031_Tlp23 MAESIKEQTAGITQINESVAQIDQTTKDNVEIANESAIISSTVSDIANNILEDVKKKRF 658

RM3196_Tlp23 MAESIKEQTAGITQINDSVAQIDQTTKDNVEIANESAIISSTVSDIANNILEDVKKKRF 659

ICDCCJ07001_Tlp23 MAESIKEQTAGITQINDSVAQIDQTTKDNVEIANESAIISSTVSDIANNILEDVKKKRF 659

****************:******************************************
