# Supplementary material for: Diversity of transducer-like proteins (Tlps) in Campylobacter
Source: PLoS One. 2019 Mar 25;14(3):e0214228. doi: 10.1371/journal.pone.0214228 (PMC6433261; doi:10.1371/journal.pone.0214228)
Supplement: S2 Archive — (ZIP) [file pone.0214228.s016.zip › Alignment X.docx]

Alignment X. Tlp2 and Tlp24 sequence comparisons

CLUSTAL O(1.2.4) multiple sequence alignment 18/05/28

NCTC11168_Tlp2 MKSVKLKVSLIANLIAVVCLIILGVVTFIFVKQAIFHEVVNAEINYVKTAKNSIESFKAR 60

CJM1cam_Tlp24 MKSVKLKVALIANLIAVVCLVILGVITFMFVKQAIFHEVVKAETNYVKTAKNSMESFKAR 60

M1_Tlp24 MKSVKLKVALIANLIAVVCLVILGVITFMFVKQAIFHEVVKAETNYVKTAKNSMESFKAR 60

********:***********:****:**:***********:** *********:******

NCTC11168_Tlp2 NSLALESLAKSILKHPIEQLDSQDALMHYVGKDLKNFRDAGRFLAVYIAQPNGELVVSDP 120

CJM1cam_Tlp24 NSLALESLAKSILKHPVEQLDSQDALMRYVGKDLKNFRDAGRFLAVYIAQPNGELVVSDP 120

M1_Tlp24 NSLALESLAKSILKHPVEQLDSQDALMRYVGKDLKNFRDAGRFLAVYIAQPNGELVVSDP 120

****************:**********:********************************

NCTC11168_Tlp2 DSDAKNLDFGTYGKADNYDARTREYYIEAVKTNKLYITPSYIDVTTNLPCFTYSIPLYKD 180

CJM1cam_Tlp24 DSDAKKVDFGTYGKADNYDARTREYYIEAVKTNKLYVTPSYIDATTNLPCFTYSTPLYKD 180

M1_Tlp24 DSDAKKVDFGTYGKADNYDARTREYYIEAVKTNKLYVTPSYIDATTNLPCFTYSTPLYKD 180

*****::*****************************:******.********** *****

NCTC11168_Tlp2 GKFIGVLAVDILAADLQAEFENLPGRTFVFDEENKVFVSTDKALLQKGYDISAIANLAKT 240

CJM1cam_Tlp24 GKFIGVLAVDVLVTDLQAEFENLPGRTFVFDEENKVFASTDKTLLQQGYDISAIANLAKI 240

M1_Tlp24 GKFIGVLAVDVLVTDLQAEFENLPGRTFVFDEENKVFASTDKTLLQQGYDISAIANLAKI 240

**********:*.:***********************.****:***:************

NCTC11168_Tlp2 KEDLEPFEYTRPKDGNERFAVCTKVSGIYTACVGEPIEQIEAPVYKIAFIQTAIVIFTSI 300

CJM1cam_Tlp24 KENFEPFEYTRPKDGSERFAVCTKVSGVYTACVGEPIEQIEAPVYKIAFIQTAIVIFTSI 300

M1_Tlp24 KENFEPFEYTRPKDGSERFAVCTKVSGVYTACVGEPIEQIEAPVYKIAFIQTAIVIFTSI 300

**::***********.***********:********************************

NCTC11168_Tlp2 ISVILLYFIVSKYLSPLAAIQTGLTSFFDFINYKTKNVSTIEVKSNDEFGQISNAINENI 360

CJM1cam_Tlp24 ISVILLYFIVSKYLSPLAAIQTGLTSFFDFINHKTKNVSTIEVKSNDEFGQISSAINENI 360

M1_Tlp24 ISVILLYFIVSKYLSPLAAIQTGLTSFFDFINHKTKNVSTIEVKSNDEFGQISSAINENI 360

********************************:********************.******

NCTC11168_Tlp2 LATKRGLEQDNQAVKESVQTVSVVEGGNLTARITANPRNPQLIELKNVLNKLLDVLQARV 420

CJM1cam_Tlp24 LATKRGLEQDNQAVKESVETVSVVESGNLTARITANPRNPQLIELKNVLNKLLDVLQARV 420

M1_Tlp24 LATKRGLEQDNQAVKESVETVSVVESGNLTARITANPRNPQLIELKNVLNKLLDVLQARV 420

******************:******.**********************************

NCTC11168_Tlp2 GSDMNAIHKIFEEYKSLDFRNKLENASGSVELTTNALGDEIVKMLKQSSDFANALANESG 480

CJM1cam_Tlp24 VLY-ECYS-NF-RIQSLDFRNKLENASGSVELTTNALGDEIVKMLKQSSDFANALANESG 477

M1_Tlp24 VLI--CYS-NF-RIQSLDFRNKLENASGSVELTTNALGDEIVKMLKQSSDFANALANESG 476

. * . :*********************************************

NCTC11168_Tlp2 KLQTAVQSLTTSSNSQAQSLEETAAALEEITSSMQNVSVKTSDVITQSEEIKNVTGIIGD 540

CJM1cam_Tlp24 KLQTAVQSLTTSSNSQAQSLEETAAALEEITSSMQNVSVKTSDVITQSEEIKNVTGIIGD 537

M1_Tlp24 KLQTAVQSLTTSSNSQAQSLEETAAALEEITSSMQNVSVKTSDVITQSEEIKNVTGIIGD 536

************************************************************

NCTC11168_Tlp2 IADQINLLALNAAIEAARAGEHGRGFAVVADEVRKLAERTQKSLSEIEANTNLLVQSIND 600

CJM1cam_Tlp24 IADQINLLALNAAIEAARAGEHGRGFAVVADEVRKLAERTQKSLSEIEANTNLLVQSIND 597

M1_Tlp24 IADQINLLALNAAIEAARAGEHGRGFAVVADEVRKLAERTQKSLSEIEANTNLLVQSIND 596

************************************************************

NCTC11168_Tlp2 MAESIKEQTAGITQINDSVAQIDQTTKDNVEIANESAIISSTVSDIANNILEDVKKKRF 659

CJM1cam_Tlp24 MAESIKEQTAGITQINESVAQIDQTTKDNVEIANESAIISSTVSDIANNILEDVKKKRF 656

M1_Tlp24 MAESIKEQTAGITQINESVAQIDQTTKDNVEIANESAIISSTVSDIANNILEDVKKKRF 655

****************:******************************************
