# Supplementary material for: Diversity of transducer-like proteins (Tlps) in Campylobacter
Source: PLoS One. 2019 Mar 25;14(3):e0214228. doi: 10.1371/journal.pone.0214228 (PMC6433261; doi:10.1371/journal.pone.0214228)
Supplement: S2 Archive — (ZIP) [file pone.0214228.s016.zip › Alignment Y.docx]

Alignment Y. Comparison of *tlp2* and *tlp24* genes

CLUSTAL O(1.2.4) multiple sequence alignment 18/05/28

NCTC11168_tlp2gene atgaaaagcgtaaaattgaaggtttcgctgattgcaaatttaatcgcagtagtgtgtttg 60

CJM1cam_tlp24gene atgaaaagcgtaaaattgaaggttgcactgattgcaaatttaatcgcagtagtgtgtttg 60

M1_tlp24gene atgaaaagcgtaaaattgaaggttgcactgattgcaaatttaatcgcagtagtgtgtttg 60

************************ * *********************************

NCTC11168_tlp2gene ataattttaggtgttgtaacatttatatttgtaaagcaagcaatttttcatgaagttgtg 120

CJM1cam_tlp24gene gtaattttaggtgttataacatttatgtttgtaaagcaagcaatttttcatgaagttgta 120

M1_tlp24gene gtaattttaggtgttataacatttatgtttgtaaagcaagcaatttttcatgaagttgta 120

************** ********** ********************************

NCTC11168_tlp2gene aatgctgaaataaattatgttaaaacggctaaaaattctatagagtcttttaaggcaaga 180

CJM1cam_tlp24gene aaagctgaaacaaactatgttaaaacagctaaaaattctatggagtcttttaaggcaaga 180

M1_tlp24gene aaagctgaaacaaactatgttaaaacagctaaaaattctatggagtcttttaaggcaaga 180

** ******* *** *********** ************** ******************

NCTC11168_tlp2gene aattctttagctcttgaaagtttagctaaaagtattttaaagcatcctatagaacagtta 240

CJM1cam_tlp24gene aattctttagctcttgaaagtttggctaaaagtattttaaagcatcctgtagaacagtta 240

M1_tlp24gene aattctttagctcttgaaagtttggctaaaagtattttaaagcatcctgtagaacagtta 240

*********************** ************************ ***********

NCTC11168_tlp2gene gatagtcaagatgctttaatgcattatgttggaaaagatttaaagaattttagagatgct 300

CJM1cam_tlp24gene gatagtcaagatgctttaatgcgttatgttggaaaagatttaaagaattttagagatgct 300

M1_tlp24gene gatagtcaagatgctttaatgcgttatgttggaaaagatttaaagaattttagagatgct 300

********************** *************************************

NCTC11168_tlp2gene ggaagattcttagcagtttatattgctcaaccaaatggcgaacttgttgtaagcgatcca 360

CJM1cam_tlp24gene ggaagatttttagcagtttatatcgctcaaccaaacggagaacttgttgtaagtgatcca 360

M1_tlp24gene ggaagatttttagcagtttatatcgctcaaccaaacggagaacttgttgtaagtgatcca 360

******** ************** *********** ** ************** ******

NCTC11168_tlp2gene gactctgatgctaaaaatttagattttggaacttatggaaaagctgataattatgatgct 420

CJM1cam_tlp24gene gattctgatgctaaaaaagtagattttggaacttatggaaaagctgataattatgatgct 420

M1_tlp24gene gattctgatgctaaaaaagtagattttggaacttatggaaaagctgataattatgatgct 420

** ************** *****************************************

NCTC11168_tlp2gene agaacaagagagtattatatagaagcagttaaaacaaataaactttatattaccccatct 480

CJM1cam_tlp24gene agaacaagagagtattatatagaagcagtaaaaacaaataaactttatgttaccccatct 480

M1_tlp24gene agaacaagagagtattatatagaagcagtaaaaacaaataaactttatgttaccccatct 480

***************************** ****************** ***********

NCTC11168_tlp2gene tatattgatgtaactacaaatttaccttgctttacatattctattccgctttataaagat 540

CJM1cam_tlp24gene tatatagatgcaactacaaatttaccttgttttacatattctacccctctttataaagat 540

M1_tlp24gene tatatagatgcaactacaaatttaccttgttttacatattctacccctctttataaagat 540

***** **** ****************** ************* ** ************

NCTC11168_tlp2gene ggtaaatttataggggttttggctgtagatattcttgcggcagatttgcaagctgaattt 600

CJM1cam_tlp24gene ggtaaatttataggggttttagctgtggatgttcttgtaacagatttgcaagcagaattt 600

M1_tlp24gene ggtaaatttataggggttttagctgtggatgttcttgtaacagatttgcaagcagaattt 600

******************** ***** *** ****** ************* ******

NCTC11168_tlp2gene gaaaatttaccaggtagaacttttgtatttgatgaagaaaataaagtatttgtttctaca 660

CJM1cam_tlp24gene gagaatttaccaggtagaacttttgtatttgatgaagaaaataaagtatttgcttctaca 660

M1_tlp24gene gagaatttaccaggtagaacttttgtatttgatgaagaaaataaagtatttgcttctaca 660

** ************************************************* *******

NCTC11168_tlp2gene gacaaagctcttttacaaaaaggttatgatattagtgcaattgcaaatcttgctaaaact 720

CJM1cam_tlp24gene gacaaaactcttttgcaacaaggttatgatattagtgcaattgcaaatcttgcaaaaatt 720

M1_tlp24gene gacaaaactcttttgcaacaaggttatgatattagtgcaattgcaaatcttgcaaaaatt 720

****** ******* *** ********************************** **** *

NCTC11168_tlp2gene aaagaggatcttgaaccttttgagtatactagaccaaaagatggtaatgaaagatttgct 780

CJM1cam_tlp24gene aaagaaaattttgaaccttttgaatatactagaccaaaagatggtagtgaaagatttgct 780

M1_tlp24gene aaagaaaattttgaaccttttgaatatactagaccaaaagatggtagtgaaagatttgct 780

***** ** ************* ********************** *************

NCTC11168_tlp2gene gtatgcacaaaggtttctggaatttatactgcttgcgttggagagccaatagaacaaata 840

CJM1cam_tlp24gene gtgtgtacaaaagtttctggggtttatactgcttgcgttggagagccaatagaacaaata 840

M1_tlp24gene gtgtgtacaaaagtttctggggtttatactgcttgcgttggagagccaatagaacaaata 840

** ** ***** ******** **************************************

NCTC11168_tlp2gene gaagctccagtttataaaattgcatttatacaaactgcgattgttatttttacaagtatt 900

CJM1cam_tlp24gene gaagctccagtttataaaattgcatttatacaaactgcgattgttatttttacaagtatt 900

M1_tlp24gene gaagctccagtttataaaattgcatttatacaaactgcgattgttatttttacaagtatt 900

************************************************************

NCTC11168_tlp2gene attagcgtcatcctcctttatttcatcgtatcaaaatacctctccccacttgcagctatc 960

CJM1cam_tlp24gene attagcgtaatcctactttatttcatcgtatcaaaatacctctccccacttgcagctatc 960

M1_tlp24gene attagcgtaatcctactttatttcatcgtatcaaaatacctctccccacttgcagctatc 960

******** ***** *********************************************

NCTC11168_tlp2gene caaacaggtttaacttcattctttgattttatcaactataaaacaaaaaatgtttccact 1020

CJM1cam_tlp24gene caaacaggtttaacttcattctttgattttatcaatcataaaacaaaaaatgtttctact 1020

M1_tlp24gene caaacaggtttaacttcattctttgattttatcaatcataaaacaaaaaatgtttctact 1020

*********************************** ******************* ***

NCTC11168_tlp2gene atagaagtaaaaagcaatgatgaatttggacaaatctcaaatgctatcaatgaaaacatt 1080

CJM1cam_tlp24gene atagaagtaaaaagcaatgatgaatttggacaaatctcaagtgctatcaatgaaaacatt 1080

M1_tlp24gene atagaagtaaaaagcaatgatgaatttggacaaatctcaagtgctatcaatgaaaacatt 1080

**************************************** *******************

NCTC11168_tlp2gene cttgctactaaaagaggcttagaacaagacaatcaagccgttaaagaatcagttcaaacc 1140

CJM1cam_tlp24gene cttgctactaaaagaggtttagaacaagacaatcaagccgttaaagaatcggttgaaaca 1140

M1_tlp24gene cttgctactaaaagaggtttagaacaagacaatcaagccgttaaagaatcggttgaaaca 1140

***************** ******************************** *** ****

NCTC11168_tlp2gene gtatcagttgtagaaggtggtaatttaacagcaagaattactgctaatccaagaaaccca 1200

CJM1cam_tlp24gene gtatcagttgtagaaagtggtaatttaacagcaagaattactgctaatccaagaaaccca 1200

M1_tlp24gene gtatcagttgtagaaagtggtaatttaacagcaagaattactgctaatccaagaaaccca 1200

*************** ********************************************

NCTC11168_tlp2gene cagcttattgaacttaaaaatgttctaaataaacttcttgatgttttacaagctagagta 1260

CJM1cam_tlp24gene caacttattgaacttaaaaatgttctaaataaacttcttgatgttttacaagctagagta 1260

M1_tlp24gene caacttattgaacttaaaaatgttctaaataaacttcttgatgttttacaagctagagta 1260

** *********************************************************

NCTC11168_tlp2gene g**g**ttctgatatgaatgctattcataaaatttttgaagaatac**a**aaagcttagactttaga 1320

CJM1cam_tlp24gene g-ttctgatatgaatgctattcataaaatttttgaagaatac-aaagcttagactttaga 1318

M1_tlp24gene g-ttctgatatgaatgctattcataaaatttttgaagaatac-aaagcttagactttaga 1318

* ************************************** *****************

NCTC11168_tlp2gene aataaattagaaaatgctagcggtagtgtagaattaactactaatgctttaggtgatgaa 1380

CJM1cam_tlp24gene aataaattagaaaatgctagcggtagtgtagaattaactactaatgctttaggtgatgaa 1378

M1_tlp24gene aataaattagaaaatgctagcggtagtgtagaattaactactaatgctttaggtgatgaa 1378

************************************************************

NCTC11168_tlp2gene atagttaaaatgctaaaacaaagttcagactttgctaatgctttagctaatgaaagtgga 1440

CJM1cam_tlp24gene atagttaaaatgctaaaacaaagttcagactttgctaatgctttagctaatgaaagtgga 1438

M1_tlp24gene atagttaaaatgctaaaacaaagttcagactttgctaatgctttagctaatgaaagtgga 1438

************************************************************

NCTC11168_tlp2gene aaattacaaactgctgttcaaagcttaaccacttcttcaaattctcaagctcaatcttta 1500

CJM1cam_tlp24gene aaattacaaactgctgttcaaagcttaaccacttcttcaaattctcaagctcaatcttta 1498

M1_tlp24gene aaattacaaactgctgttcaaagcttaaccacttcttcaaattctcaagctcaatcttta 1498

************************************************************

NCTC11168_tlp2gene gaagaaactgcagcagctttagaagagatcacttcttctatgcaaaatgtttcagttaaa 1560

CJM1cam_tlp24gene gaagaaactgcagcagctttagaagagatcacttcttctatgcaaaatgtttcagttaaa 1558

M1_tlp24gene gaagaaactgcagcagctttagaagagatcacttcttctatgcaaaatgtttcagttaaa 1558

************************************************************

NCTC11168_tlp2gene actagtgatgttatcactcaatctgaagagattaaaaatgttacaggtattataggtgat 1620

CJM1cam_tlp24gene actagtgatgttatcactcaatctgaagagattaaaaatgttacaggtattataggtgat 1618

M1_tlp24gene actagtgatgttatcactcaatctgaagagattaaaaatgttacaggtattataggtgat 1618

************************************************************

NCTC11168_tlp2gene attgcagatcaaatcaatcttttagctttaaatgcagctattgaagcagctcgtgctgga 1680

CJM1cam_tlp24gene attgcagatcaaatcaaccttctagctttaaatgcagctattgaagcagctcgtgctgga 1678

M1_tlp24gene attgcagatcaaatcaaccttctagctttaaatgcagctattgaagcagctcgtgctgga 1678

***************** *** **************************************

NCTC11168_tlp2gene gaacatggtagaggctttgcagtggtagctgatgaagttagaaagttagctgaaagaact 1740

CJM1cam_tlp24gene gaacatggtagaggctttgcagtggtagctgatgaagttagaaagttagctgaaagaact 1738

M1_tlp24gene gaacatggtagaggctttgcagtggtagctgatgaagttagaaagttagctgaaagaact 1738

************************************************************

NCTC11168_tlp2gene caaaagtctttatcagaaattgaagctaatactaatttacttgttcaatctatcaatgat 1800

CJM1cam_tlp24gene caaaagtctttatctgaaatagaagctaatactaatttacttgttcaatctatcaatgat 1798

M1_tlp24gene caaaagtctttatctgaaatagaagctaatactaatttacttgttcaatctatcaatgat 1798

************** ***** ***************************************

NCTC11168_tlp2gene atggcagaaagtattaaagaacaaactgcaggtatcactcaaatcaatgatagcgtagct 1860

CJM1cam_tlp24gene atggcagaaagtattaaagaacaaactgcaggtatcactcaaatcaatgaaagtgtagct 1858

M1_tlp24gene atggcagaaagtattaaagaacaaactgcaggtatcactcaaatcaatgaaagtgtagct 1858

************************************************** ** ******

NCTC11168_tlp2gene caaattgatcaaactactaaagataatgttgaaattgctaatgaatcagctattatttct 1920

CJM1cam_tlp24gene caaattgatcaaactactaaagataatgttgaaattgctaatgaatcagctattatttct 1918

M1_tlp24gene caaattgatcaaactactaaagataatgttgaaattgctaatgaatcagctattatttct 1918

************************************************************

NCTC11168_tlp2gene agtacagtaagtgatatagctaataatatcttagaagatgttaagaagaagaggttttaa 1980

CJM1cam_tlp24gene agtacagtaagtgatatagctaataatatcttagaagatgttaagaaaaaaagattttaa 1978

M1_tlp24gene agtacagtaagtgatatagctaataatatcttagaagatgttaagaaaaaaagattttaa 1978

*********************************************** ** ** ******
