# Supplementary material for: Diversity of transducer-like proteins (Tlps) in Campylobacter
Source: PLoS One. 2019 Mar 25;14(3):e0214228. doi: 10.1371/journal.pone.0214228 (PMC6433261; doi:10.1371/journal.pone.0214228)
Supplement: S2 Archive — (ZIP) [file pone.0214228.s016.zip › Alignment Z.docx]

Alignment Z. Comparison of *tlp25* DNA sequence with *tlp4* and *tlp12* DNA sequences

EMBOSS Needle 18/05/28 https://www.ebi.ac.uk/Tools/psa/emboss_needle/nucleotide.html

***tlp4* gene vs *tlp25* gene**

NCTC11168_tlp4 1 atgcaatcaataaattcaggcaaatccgttggaatttcagctaagcttac 50

|||||||||||||||||||||||||||||||||.||||||..||.|||||

CG8421_tlp25 1 atgcaatcaataaattcaggcaaatccgttggagtttcagtcaaacttac 50

NCTC11168_tlp 51 gctatgggttggaattttagttgtattaattttagcaatcacaagtgcta 100

|||.||||||||||||||||||||||||||||||||||||||||||.|..

CG8421_tlp25 51 gctgtgggttggaattttagttgtattaattttagcaatcacaagtaccg 100

NCTC11168_tlp 101 ttagttactttgattcgagaaacaatacatatgaattgctaaaagacact 150

||||||||||||||.|.|.|||..|.||||||||||||||||||||.|.|

CG8421_tlp25 101 ttagttactttgatgccaaaaatcacacatatgaattgctaaaagaaaat 150

NCTC11168_tlp 151 cagttaaaaactatgcaagatgtggatgctttctttaaaagctatgctat 200

||.||||||||||||.|.|||||

CG8421_tlp25 151 caattaaaaactatggatgatgt--------------------------- 173

NCTC11168_tlp 201 gtcaaaaagaaatggtattcaaatactagccaatgagctaacaaatcgtc 250

CG8421_tlp25 174 -------------------------------------------------- 173

NCTC11168_tlp 251 ctgatatgagcgatgaagagctaatcaatcttatcaaagtaattaaaaaa 300

CG8421_tlp25 174 -------------------------------------------------- 173

NCTC11168_tlp 301 gttaatgactacgatctagtttatgtaggatttgataatacaggaaaaaa 350

CG8421_tlp25 174 -------------------------------------------------- 173

NCTC11168_tlp 351 ttatcaatctgatgatcaaattttagatctatcaaaaggttatgatacta 400

|||||||||||| ||||||||||||..|||||.|

CG8421_tlp25 174 -------tctgatgatcaa----------tatcaaaaggtttagatacaa 206

NCTC11168_tlp 401 aaaatcgtccttggtataaagctgccaaagaagcaaaaaagcttatagta 450

|||

CG8421_tlp25 207 aaa----------------------------------------------- 209

NCTC11168_tlp 451 acagaaccttataaatccgccgctagcggagaggttggtttaacttacgc 500

CG8421_tlp25 210 -------------------------------------------------- 209

NCTC11168_tlp 501 tgctccattttatgatagaaatggaaattttagaggtgttgtaggtggag 550

CG8421_tlp25 210 -------------------------------------------------- 209

NCTC11168_tlp 551 attatgatctagcaaatttttcaaccaatgttttaactgtaggaaaatca 600

CG8421_tlp25 210 -------------------------------------------------- 209

NCTC11168_tlp 601 gacaatacctttactgaagtacttgattcagaaggaacaatactttttaa 650

CG8421_tlp25 210 -------------------------------------------------- 209

NCTC11168_tlp 651 tgatgaagttgctaaaatactaacaaaaacagaattaagtatcaatatcg 700

CG8421_tlp25 210 -------------------------------------------------- 209

NCTC11168_tlp 701 ccaatgcaatcaaagcaaatcctgctcttattgatccaagaaaccaagat 750

CG8421_tlp25 210 -------------------------------------------------- 209

NCTC11168_tlp 751 actttatttaccgctaaagatcaccaaggcgtagattatgcgattatgtg 800

CG8421_tlp25 210 -------------------------------------------------- 209

NCTC11168_tlp 801 taattctgcttttaatcctttatttagaatttgtacaataacagaaaaca 850

CG8421_tlp25 210 -------------------------------------------------- 209

NCTC11168_tlp 851 aagtttataccgaagctgttaattctattttaatgaaacaagttatagtt 900

|||||||||||||.|||||||||||||||||||||||.

CG8421_tlp25 210 ------------aagctgttaattccattttaatgaaacaagttatagtc 247

NCTC11168_tlp 901 ggtattatagctataatcatagctttaatcttgattagatttttaatcag 950

||||||||||||||||||||||||||||||||||||||||||||||||||

CG8421_tlp25 248 ggtattatagctataatcatagctttaatcttgattagatttttaatcag 297

NCTC11168_tlp 951 cagaagtctctccccacttgcagctatccaaacaggtttaacttcattct 1000

||||||||||||||||||||||||||||||||||||||||||||||||||

CG8421_tlp25 298 cagaagtctctccccacttgcagctatccaaacaggtttaacttcattct 347

NCTC11168_tlp 1001 ttgattttatcaactataaaacaaaaaatgtttccactatagaagtaaaa 1050

||||||||||||||||||||||||||||||||||.|||||||||||||||

CG8421_tlp25 348 ttgattttatcaactataaaacaaaaaatgtttctactatagaagtaaaa 397

NCTC11168_tlp 1051 agcaatgatgaatttggacaaatctcaaatgctatcaatgaaaacattct 1100

||||||||||||||||||||||||||||||||||||||||||||||||||

CG8421_tlp25 398 agcaatgatgaatttggacaaatctcaaatgctatcaatgaaaacattct 447

NCTC11168_tlp 1101 tgctactaaaagaggcttagaacaagacaatcaagccgttaaagaatcag 1150

||||||||||||||||||||||||||||||||||||||||||||||||||

CG8421_tlp25 448 tgctactaaaagaggcttagaacaagacaatcaagccgttaaagaatcag 497

NCTC11168_tlp 1151 ttcaaaccgtatcagttgtagaaggtggtaatttaacagcaagaattact 1200

||||||||||||||||||||||||||||||||||||||||||||||||||

CG8421_tlp25 498 ttcaaaccgtatcagttgtagaaggtggtaatttaacagcaagaattact 547

NCTC11168_tlp 1201 gctaatccaagaaacccacagcttattgaacttaaaaatgttctaaataa 1250

||||||||||||||||||||.|||||||||||||||||||||||||||||

CG8421_tlp25 548 gctaatccaagaaacccacaacttattgaacttaaaaatgttctaaataa 597

NCTC11168_tlp 1251 acttcttgatgttttacaagctagagtaggttctgatatgaatgctattc 1300

||||||||||||||||||||||||||||||||||||||||||||||||||

CG8421_tlp25 598 acttcttgatgttttacaagctagagtaggttctgatatgaatgctattc 647

NCTC11168_tlp 1301 ataaaatttttgaagaatacaaaagcttagactttagaaataaattagaa 1350

||||||||||||||||||||||||||||||||||||||||||||||||||

CG8421_tlp25 648 ataaaatttttgaagaatacaaaagcttagactttagaaataaattagaa 697

NCTC11168_tlp 1351 aatgctagcggtagtgtagaattaactactaatgctttaggtgatgaaat 1400

||||||||||||||||||||||||||||||||||||||||||||||||||

CG8421_tlp25 698 aatgctagcggtagtgtagaattaactactaatgctttaggtgatgaaat 747

NCTC11168_tlp 1401 agttaaaatgctaaaacaaagttcagactttgctaatgctttagctaatg 1450

|||||||||||||||||||||||||||||||||||||||.||||||||||

CG8421_tlp25 748 agttaaaatgctaaaacaaagttcagactttgctaatgccttagctaatg 797

NCTC11168_tlp 1451 aaagtggaaaattacaaactgctgttcaaagcttaaccacttcttcaaat 1500

||||||||||||||||||||||||||||||||||||||||||||||||||

CG8421_tlp25 798 aaagtggaaaattacaaactgctgttcaaagcttaaccacttcttcaaat 847

NCTC11168_tlp 1501 tctcaagctcaatctttagaagaaactgcagcagctttagaagagatcac 1550

||||||||||||||||||||||||||||||||||||||||||||||||||

CG8421_tlp25 848 tctcaagctcaatctttagaagaaactgcagcagctttagaagagatcac 897

NCTC11168_tlp 1551 ttcttctatgcaaaatgtttcagttaaaactagtgatgttatcactcaat 1600

||||||||||||||||||||||||||||||||||||||||||||||||||

CG8421_tlp25 898 ttcttctatgcaaaatgtttcagttaaaactagtgatgttatcactcaat 947

NCTC11168_tlp 1601 ctgaagagattaaaaatgttacaggtattataggtgatattgcagatcaa 1650

||||||||||||||||||||||||||||||||||||||||||||||||||

CG8421_tlp25 948 ctgaagagattaaaaatgttacaggtattataggtgatattgcagatcaa 997

NCTC11168_tlp 1651 atcaatcttttagctttaaatgcagctattgaagcagctcgtgctggaga 1700

||||||||||||||||||||||||||||||||||||||||||||||||||

CG8421_tlp25 998 atcaatcttttagctttaaatgcagctattgaagcagctcgtgctggaga 1047

NCTC11168_tlp 1701 acatggtagaggctttgcagtggtagctgatgaagttagaaagttagctg 1750

||||||||||||||||||||||||||||||||||||||||||||||||||

CG8421_tlp25 1048 acatggtagaggctttgcagtggtagctgatgaagttagaaagttagctg 1097

NCTC11168_tlp 1751 aaagaactcaaaagtctttatcagaaattgaagctaatactaatttactt 1800

||||||||||||||||||||||||||||||||||||||||||||||||||

CG8421_tlp25 1098 aaagaactcaaaagtctttatcagaaattgaagctaatactaatttactt 1147

NCTC11168_tlp 1801 gttcaatctatcaatgatatggcagaaagtattaaagaacaaactgcagg 1850

||||||||||||||||||||||||||||||||||||||||||||||||||

CG8421_tlp25 1148 gttcaatctatcaatgatatggcagaaagtattaaagaacaaactgcagg 1197

NCTC11168_tlp 1851 tatcactcaaatcaatgatagcgtagctcaaattgatcaaactactaaag 1900

||||||||||||||||||||||||||||||||||||||||||||||||||

CG8421_tlp25 1198 tatcactcaaatcaatgatagcgtagctcaaattgatcaaactactaaag 1247

NCTC11168_tlp 1901 ataatgttgaaattgctaatgaatcagctattatttctagtacagtaagt 1950

||||||||||||||||||||||||||||||||||||||||||||||||||

CG8421_tlp25 1248 ataatgttgaaattgctaatgaatcagctattatttctagtacagtaagt 1297

NCTC11168_tlp 1951 gatatagctaataatatcttagaagatgttaagaagaagaggttttaa 1998

||||||||||||||||||||||||||||||||||||||.|||||||||

CG8421_tlp25 1298 gatatagctaataatatcttagaagatgttaagaagaaaaggttttaa 1345

***tlp12* gene vs *tlp25* gene**

S3_tlp12 1 atgcaaaaaatggattcaggcaaatccgttggagtttcagtcaaacttac 50

||||||..|||..|||||||||||||||||||||||||||||||||||||

CG8421_tlp25 1 atgcaatcaataaattcaggcaaatccgttggagtttcagtcaaacttac 50

S3_tlp12 51 gctgtgggttggaattttagttgtattaattttagcaatcacaagtaccg 100

||||||||||||||||||||||||||||||||||||||||||||||||||

CG8421_tlp25 51 gctgtgggttggaattttagttgtattaattttagcaatcacaagtaccg 100

S3_tlp12 101 ttagttactttgatgccaaaaatcacacatatgaattgctaaaagaaaat 150

||||||||||||||||||||||||||||||||||||||||||||||||||

CG8421_tlp25 101 ttagttactttgatgccaaaaatcacacatatgaattgctaaaagaaaat 150

S3_tlp12 151 caattaaaaactatggatgatgttaaagtaacttttgaaaactattccaa 200

|||||||||||||||||||||||| ||

CG8421_tlp25 151 caattaaaaactatggatgatgtt-------ct----------------- 176

S3_tlp12 201 aagcaagcaaaaagctatagaggttctagcatatgaaagtgctaaaaaat 250

CG8421_tlp25 177 -------------------------------------------------- 176

S3_tlp12 251 tagaagatgaaaatatttcactgcttctagattcttttaagaaggcgttt 300

||||| |||

CG8421_tlp25 177 -----gatga-------tca------------------------------ 184

S3_tlp12 301 gattttgatattgtttttattgcgtttgataaaaacaacaaaatgctttt 350

|||| |||||.|

CG8421_tlp25 185 -------atat---------------------------caaaagg----- 195

S3_tlp12 351 gtcaaatggaacaattttagataaaaaatcaaattttgacattacaaaac 400

||||||||

CG8421_tlp25 196 ---------------tttagata--------------------------- 203

S3_tlp12 401 aaatttggtatcaagaagcaaaaaataacaaaggcatcacaataactcaa 450

|||||||

CG8421_tlp25 204 ------------------caaaaaa------------------------- 210

S3_tlp12 451 ccatataaatcccctatagatcaagagattggtataacttatgttttccc 500

CG8421_tlp25 211 -------------------------------------------------- 210

S3_tlp12 501 tatttataaaaataatcaacttatagctttcgttggaggcgattacaatc 550

CG8421_tlp25 211 -------------------------------------------------- 210

S3_tlp12 551 tagataaattttccaaagatgtattatctttaggacactcatcaactact 600

CG8421_tlp25 211 -------------------------------------------------- 210

S3_tlp12 601 tatgctgctgtttatgactctgaaggaagaattatttttcacgaagtgct 650

CG8421_tlp25 211 -------------------------------------------------- 210

S3_tlp12 651 tgatagaattttaacaaaaaacactttaagcgttaatattgctaacgcca 700

CG8421_tlp25 211 -------------------------------------------------- 210

S3_tlp12 701 taaaagaaaaccctgaatatatagatccaaataagagagatattcttttt 750

CG8421_tlp25 211 -------------------------------------------------- 210

S3_tlp12 751 cctgtatttgatgataaaggtatcaaatatgaaacaatgtgcgatacaag 800

CG8421_tlp25 211 -------------------------------------------------- 210

S3_tlp12 801 ttccaatggattatatcgtatttgtgctgtaactttagatagtaactata 850

CG8421_tlp25 211 -------------------------------------------------- 210

S3_tlp12 851 cttccgctgttaattccattttaatgaaacaagctatagtcggtattata 900

.||||||||||||||||||||||||||||.||||||||||||||||

CG8421_tlp25 211 ----agctgttaattccattttaatgaaacaagttatagtcggtattata 256

S3_tlp12 901 gctataatcatagctttaatcttgattagatttttaatcagcagaagcct 950

|||||||||||||||||||||||||||||||||||||||||||||||.||

CG8421_tlp25 257 gctataatcatagctttaatcttgattagatttttaatcagcagaagtct 306

S3_tlp12 951 ctccccacttgcagctatccaaacaggtttaacttcattctttgatttta 1000

||||||||||||||||||||||||||||||||||||||||||||||||||

CG8421_tlp25 307 ctccccacttgcagctatccaaacaggtttaacttcattctttgatttta 356

S3_tlp12 1001 tcaactataaaacaaaaaatgtttctactatagaagtaaaaagcaatgat 1050

||||||||||||||||||||||||||||||||||||||||||||||||||

CG8421_tlp25 357 tcaactataaaacaaaaaatgtttctactatagaagtaaaaagcaatgat 406

S3_tlp12 1051 gaatttggacaaatctcaaatgctatcaatgaaaacattcttgctactaa 1100

||||||||||||||||||||||||||||||||||||||||||||||||||

CG8421_tlp25 407 gaatttggacaaatctcaaatgctatcaatgaaaacattcttgctactaa 456

S3_tlp12 1101 aagaggcttagaacaagacaatcaagccgttaaagaatcagttcaaaccg 1150

||||||||||||||||||||||||||||||||||||||||||||||||||

CG8421_tlp25 457 aagaggcttagaacaagacaatcaagccgttaaagaatcagttcaaaccg 506

S3_tlp12 1151 tatcagttgtagaaggtggtaatttaacagcaagaattactgctaatcca 1200

||||||||||||||||||||||||||||||||||||||||||||||||||

CG8421_tlp25 507 tatcagttgtagaaggtggtaatttaacagcaagaattactgctaatcca 556

S3_tlp12 1201 agaaacccacaattaatagaattaaaaaatgttctaaatagacttcttga 1250

||||||||||||.|.||.|||.|.||||||||||||||||.|||||||||

CG8421_tlp25 557 agaaacccacaacttattgaacttaaaaatgttctaaataaacttcttga 606

S3_tlp12 1251 tgctttacaggctagagtaggttctgatatgaatgaaattca-aagagta 1299

||.||||||.|||||||||||||||||||||||||..||||| ||.|.|.

CG8421_tlp25 607 tgttttacaagctagagtaggttctgatatgaatgctattcataaaattt 656

S3_tlp12 1300 tttaatagttataaatctcttgactttactactgaagtaaaagatgccaa 1349

||.||.|.|...|||.|| |.|||||||..|.|.||.||.||.||||.|.

CG8421_tlp25 657 ttgaagaatacaaaagct-tagactttagaaataaattagaaaatgctag 705

S3_tlp12 1350 tgg-agctgtagaggtaactactaatgcactaggacaagaaatcattaaa 1398

.|| || ||||||..|||||||||||||..||||..|.|||||..|||||

CG8421_tlp25 706 cggtag-tgtagaattaactactaatgctttaggtgatgaaatagttaaa 754

S3_tlp12 1399 atgctaaaacaaagttcagactttgctaatgctttagctaatgaaagtgg 1448

||||||||||||||||||||||||||||||||.|||||||||||||||||

CG8421_tlp25 755 atgctaaaacaaagttcagactttgctaatgccttagctaatgaaagtgg 804

S3_tlp12 1449 aaaattacaaactgctgttcaaagcttaaccacttcttcaaattctcaag 1498

||||||||||||||||||||||||||||||||||||||||||||||||||

CG8421_tlp25 805 aaaattacaaactgctgttcaaagcttaaccacttcttcaaattctcaag 854

S3_tlp12 1499 ctcaatctttagaagaaactgcagcagctttagaagagatcacttcttct 1548

||||||||||||||||||||||||||||||||||||||||||||||||||

CG8421_tlp25 855 ctcaatctttagaagaaactgcagcagctttagaagagatcacttcttct 904

S3_tlp12 1549 atgcaaaatgtttcagttaaaactagtgatgttatcactcaatctgaaga 1598

||||||||||||||||||||||||||||||||||||||||||||||||||

CG8421_tlp25 905 atgcaaaatgtttcagttaaaactagtgatgttatcactcaatctgaaga 954

S3_tlp12 1599 gattaaaaatgttacaggtattataggtgatattgcagatcaaatcaatc 1648

||||||||||||||||||||||||||||||||||||||||||||||||||

CG8421_tlp25 955 gattaaaaatgttacaggtattataggtgatattgcagatcaaatcaatc 1004

S3_tlp12 1649 ttttagctttaaatgcagctattgaagcagctcgtgctggagaacatggt 1698

||||||||||||||||||||||||||||||||||||||||||||||||||

CG8421_tlp25 1005 ttttagctttaaatgcagctattgaagcagctcgtgctggagaacatggt 1054

S3_tlp12 1699 agaggctttgcagtggtagctgatgaagttagaaagttagctgaaagaac 1748

||||||||||||||||||||||||||||||||||||||||||||||||||

CG8421_tlp25 1055 agaggctttgcagtggtagctgatgaagttagaaagttagctgaaagaac 1104

S3_tlp12 1749 tcaaaagtctttatcagaaattgaagctaatactaatttacttgttcaat 1798

||||||||||||||||||||||||||||||||||||||||||||||||||

CG8421_tlp25 1105 tcaaaagtctttatcagaaattgaagctaatactaatttacttgttcaat 1154

S3_tlp12 1799 ctatcaatgatatggcagaaagtattaaagaacaaactgcaggtatcact 1848

||||||||||||||||||||||||||||||||||||||||||||||||||

CG8421_tlp25 1155 ctatcaatgatatggcagaaagtattaaagaacaaactgcaggtatcact 1204

S3_tlp12 1849 caaatcaatgatagcgtagctcaaattgatcaaactactaaagataatgt 1898

||||||||||||||||||||||||||||||||||||||||||||||||||

CG8421_tlp25 1205 caaatcaatgatagcgtagctcaaattgatcaaactactaaagataatgt 1254

S3_tlp12 1899 tgaaattgctaatgaatcagctattatttctagtacagtaagtgatatag 1948

||||||||||||||||||||||||||||||||||||||||||||||||||

CG8421_tlp25 1255 tgaaattgctaatgaatcagctattatttctagtacagtaagtgatatag 1304

S3_tlp12 1949 ctaataatatcttagaagatgttaagaagaaaaggttttaa 1989

|||||||||||||||||||||||||||||||||||||||||

CG8421_tlp25 1305 ctaataatatcttagaagatgttaagaagaaaaggttttaa 1345
