# Supplementary material for: Diversity of transducer-like proteins (Tlps) in Campylobacter
Source: PLoS One. 2019 Mar 25;14(3):e0214228. doi: 10.1371/journal.pone.0214228 (PMC6433261; doi:10.1371/journal.pone.0214228)
Supplement: S2 Archive — (ZIP) [file pone.0214228.s016.zip › Alignment ZZ.docx]

Alignment ZZ. Comparison of Tlp22 with Tlp4 and Tlp12

CLUSTAL O(1.2.4) multiple sequence alignment 18/05/28

HF5-4A-4_Tlp22 ------------------------------------------------------MTLICK 6

NCTC11168_Tlp4 MQSINSGKSVGISAKLTLWVGILVVLILAITSAISYFDSRNNTYELLKDTQLKTMQDVDA 60

* :

HF5-4A-4_Tlp22 AVLYYAMSKRNGIQILANELTNRPDMSDEELINLIKVIKKVNDYDLVYVGFDNTGKNYQS 66

NCTC11168_Tlp4 FFKSYAMSKRNGIQILANELTNRPDMSDEELINLIKVIKKVNDYDLVYVGFDNTGKNYQS 120

. ********************************************************

HF5-4A-4_Tlp22 DDQILDLSKGYDTKNRPWYKAAKEAKKLIVTEPYKSAASGEVGLTYAAPFYDRNGNFRGV 126

NCTC11168_Tlp4 DDQILDLSKGYDTKNRPWYKAAKEAKKLIVTEPYKSAASGEVGLTYAAPFYDRNGNFRGV 180

************************************************************

HF5-4A-4_Tlp22 VGGDYDLANFSTNVLTVGKSDNTFTEVLDSEGTILFNDEVAKILTKTELSINIANAIKAN 186

NCTC11168_Tlp4 VGGDYDLANFSTNVLTVGKSDNTFTEVLDSEGTILFNDEVAKILTKTELSINIANAIKAN 240

************************************************************

HF5-4A-4_Tlp22 PALIDPRNQDTLFTAKDHQGVDYAIMCNSAFNPLFRICTITENKVYTEAVNSILMKQVIV 246

NCTC11168_Tlp4 PALIDPRNQDTLFTAKDHQGVDYAIMCNSAFNPLFRICTITENKVYTEAVNSILMKQVIV 300

************************************************************

HF5-4A-4_Tlp22 GIIAIIIALILIRFLISRSLSPLAAIQTGLTSFFDFINYKTKNVSTIEVKSNDEFGQISN 306

NCTC11168_Tlp4 GIIAIIIALILIRFLISRSLSPLAAIQTGLTSFFDFINYKTKNVSTIEVKSNDEFGQISN 360

************************************************************

HF5-4A-4_Tlp22 AINENILATKRGLEQDNQAVKESVQTVSVVEGGNLTARITANPRNPQLIELKNVLNKLLD 366

NCTC11168_Tlp4 AINENILATKRGLEQDNQAVKESVQTVSVVEGGNLTARITANPRNPQLIELKNVLNKLLD 420

************************************************************

HF5-4A-4_Tlp22 VLQARVGSDMNAIHKIFEEYKSLDFRNKLENASGSVELTTNALGDEIVKMLKQSSDFANA 426

NCTC11168_Tlp4 VLQARVGSDMNAIHKIFEEYKSLDFRNKLENASGSVELTTNALGDEIVKMLKQSSDFANA 480

************************************************************

HF5-4A-4_Tlp22 LANESGKLQTAVQSLTTSSNSQAQSLEETAAALEEITSSMQNVSVKTSDVITQSEEIKNV 486

NCTC11168_Tlp4 LANESGKLQTAVQSLTTSSNSQAQSLEETAAALEEITSSMQNVSVKTSDVITQSEEIKNV 540

************************************************************

HF5-4A-4_Tlp22 TGIIGDIADQINLLALNAAIEAARAGEHGRGFAVVADEVRKLAERTQKSLSEIEANTNLL 546

NCTC11168_Tlp4 TGIIGDIADQINLLALNAAIEAARAGEHGRGFAVVADEVRKLAERTQKSLSEIEANTNLL 600

************************************************************

HF5-4A-4_Tlp22 VQSINDMAESIKEQTAGITQINDSVAQIDQTTKDNVEIANESAIISSTVSDIANNILEDV 606

NCTC11168_Tlp4 VQSINDMAESIKEQTAGITQINDSVAQIDQTTKDNVEIANESAIISSTVSDIANNILEDV 660

************************************************************

HF5-4A-4_Tlp22 KKKRF 611

NCTC11168_Tlp4 KKKRF 665

*****

CLUSTAL O(1.2.4) multiple sequence alignment

HF5-4A-4_Tlp22 ------------------------------------------------------MTLICK 6

PT14_Tlp12 MQSINSGKSVGISAKLTLWVGILVVLILAITSTVSYFDAKNHTYELLKENQLKTMDDVKV 60

* :

HF5-4A-4_Tlp22 AVLYYAMSKRNGIQILANELTNRPDMSDEELINLIKVIKKVNDYDLVYVGFDNTGKNYQS 66

PT14_Tlp12 TFENYSKSKQKAIEVLAYE--SAKKLEDENISLLLDSFKKAFDFDIVFIAFDKNNKMLLS 118

:. *: **::.*::** * . .:.**:: *:. :**. *:*:*::.**:..* *

HF5-4A-4_Tlp22 DDQILDLSKGYDTKNRPWYKAAKEAKKLIVTEPYKSAASGEVGLTYAAPFYDRNGNFRGV 126

PT14_Tlp12 NGTILDKKSNFDITKQIWYQEAKNNKGITITQPYKSPIDQEIGITYVFPIYKNN-QLIAF 177

:. *** ...:* .:: **: **: * : :*:**** . *:*:**. *:*..* :: ..

HF5-4A-4_Tlp22 VGGDYDLANFSTNVLTVGKSDNTFTEVLDSEGTILFNDEVAKILTKTELSINIANAIKAN 186

PT14_Tlp12 VGGDYNLDKFSKDVLSLGHSSTTYAAVYDSEGRIIFHEVLDRILTKNTLSVNIANAIKEN 237

*****:* :**.:**::*:*..*:: * **** *:*:: : :****. **:******* *

HF5-4A-4_Tlp22 PALIDPRNQDTLFTAKDHQGVDYAIMCNSAFNPLFRICTITENKVYTEAVNSILMKQVIV 246

PT14_Tlp12 PEYIDLNKRDILFPVFDDKGIKYEAMCDTSSNGLYRICAVTLDSNYTSAVNSILMKQVIV 297

* ** .::* ** . *.:*:.* **::: * *:***::* :. **.************

HF5-4A-4_Tlp22 GIIAIIIALILIRFLISRSLSPLAAIQTGLTSFFDFINYKTKNVSTIEVKSNDEFGQISN 306

PT14_Tlp12 GIIAIIIALILIRFLISRSLSPLAAIQTGLTSFFDFINYKTKNVSTIEVKSNDEFGQISN 357

************************************************************

HF5-4A-4_Tlp22 AINENILATKRGLEQDNQAVKESVQTVSVVEGGNLTARITANPRNPQLIELKNVLNKLLD 366

PT14_Tlp12 AINENILATKRGLEQDNQAVKESVQTVSVVEGGNLTARITANPRNPQLIELKNVLNKLLD 417

************************************************************

HF5-4A-4_Tlp22 VLQARVGSDMNAIHKIFEEYKSLDFRNKLENASGSVELTTNALGDEIVKMLKQSSDFANA 426

PT14_Tlp12 VLQARVGSDMNAIHKIFEEYKSLDFRNKLENASGSVELTTNALGDEIVKMLKQSSDFANA 477

************************************************************

HF5-4A-4_Tlp22 LANESGKLQTAVQSLTTSSNSQAQSLEETAAALEEITSSMQNVSVKTSDVITQSEEIKNV 486

PT14_Tlp12 LANESGKLQTAVQSLTTSSNSQAQSLEETAAALEEITSSMQNVSVKTSDVITQSEEIKNV 537

************************************************************

HF5-4A-4_Tlp22 TGIIGDIADQINLLALNAAIEAARAGEHGRGFAVVADEVRKLAERTQKSLSEIEANTNLL 546

PT14_Tlp12 TGIIGDIADQINLLALNAAIEAARAGEHGRGFAVVADEVRKLAERTQKSLSEIEANTNLL 597

************************************************************

HF5-4A-4_Tlp22 VQSINDMAESIKEQTAGITQINDSVAQIDQTTKDNVEIANESAIISSTVSDIANNILEDV 606

PT14_Tlp12 VQSINDMAESIKEQTAGITQINDSVAQIDQTTKDNVEIANESAIISSTVSDIANNILEDV 657

************************************************************

HF5-4A-4_Tlp22 KKKRF 611

PT14_Tlp12 KKKRF 662

*****
